# JUN-induced super-enhancer RNA forms R-loop to promote nasopharyngeal carcinoma metastasis

**DOI:** 10.1038/s41419-023-05985-9

**Published:** 2023-07-21

**Authors:** Qunying Jia, Yuan Tan, Yuejin Li, Yao Wu, Jing Wang, Faqin Tang

**Affiliations:** 1 Hunan Key Laboratory of Oncotarget Gene and Clinical Laboratory, The Affiliated Cancer Hospital of Xiangya School of Medicine, Central South University/Hunan Cancer Hospital, 410013 Changsha, China; 2 Department of Ophthalmology and Otolaryngology, The First Hospital of Hunan University of Chinese Medicine, 410208 Changsha, China

**Keywords:** Translational research, Oncogenes

## Abstract

Oncogenic super-enhancers (SEs) generate noncoding enhancer/SE RNAs (eRNAs/seRNAs) that exert a critical function in malignancy through powerful regulation of target gene expression. Herein, we show that a JUN-mediated seRNA can form R-loop to regulate target genes to promote metastasis of nasopharyngeal carcinoma (NPC). A combination of global run-on sequencing, chromatin-immunoprecipitation sequencing, and RNA sequencing was used to screen seRNAs. A specific seRNA associated with NPC metastasis (seRNA-NPCM) was identified as a transcriptional regulator for N-myc downstream-regulated gene 1 (NDRG1). JUN was found to regulate seRNA-NPCM through motif binding. seRNA-NPCM was elevated in NPC cancer tissues and highly metastatic cell lines, and promoted the metastasis of NPC cells in vitro and in vivo. Mechanistically, the 3’ end of seRNA-NPCM hybridizes with the SE region to form an R-loop, and the middle segment of seRNA-NPCM binds to heterogeneous nuclear ribonucleoprotein R (hnRNPR) at the promoter of distal gene NDRG1 and neighboring gene tribbles pseudokinase 1 (TRIB1). These structures promote chromatin looping and long-distance chromatin interactions between SEs and promoters, thus facilitating NDRG1 and TRIB1 transcription. Furthermore, the clinical analyses showed that seRNA-NPCM and NDRG1 were independent prognostic factors for NPC patients. seRNA-NPCM plays a critical role in orchestrating target gene transcription to promote NPC metastasis.

## Introduction

Enhancers are distal DNA elements that activate or increase the transcription of linked genes in cell-type-specific biological processes [1]. Transcription factors (TFs) bind to these enhancers via special recognition sequences, cooperate with cofactors, and form chromatin loops between enhancers and promoters [2]. Super-enhancers (SEs), a cluster of enhancers, also termed stretch enhancers, are reported to be present near genes crucial for cell survival and tumorigenesis that contribute to oncogenic processes by activating neighboring genes [3]. Enhancers/SEs connect promoters and are often located hundreds of kilobases from their target genes [4]. Compared to typical enhancers (TEs), SEs contain larger open-chromatin domains characterized by high level of H3K27 acetylation (H3K27ac) and are enriched for master TF binding [5, 6]. SEs often bind to RNA polymerase II (RNAPII), producing bi-directional noncoding RNAs termed seRNAs [7]. Depletion of seRNAs or seRNA-like noncoding RNAs led to downregulation of their linked protein-coding genes, suggesting that seRNAs play important roles in tumorigenesis [8, 9].

eRNAs promote the recruitment of TFs, which in turn bind to promoters or enhancers to facilitate enhancer–promoter looping [10]. eRNAs capture free TFs and increase their binding to a nearby locus, which promotes local transcription [11]. eRNAs stabilize enhancer–promoter looping by interacting with mediators or cohesin complexes [12]. Downregulation of specific eRNAs reduces enhancer–promoter connection and limits the interaction between transcription components located within the loop [9, 13]. eRNAs also bind with the transcriptional co-activator and modulate histone acetyltransferase activity, thus promoting target gene expression [14]. eRNAs contribute to conducting chromatin accessibility and promoting target gene expression. Knockdown of these eRNAs reduces chromatin accessibility and RNAPII level at the myogenin loci [15]. eRNAs have been shown to act different roles in the interaction with RNAPII. eRNAs promote a paused RNAPII transition into the elongation stage through acting as a decoy for the negative elongation factor (NELF) [16].

Nasopharyngeal carcinoma (NPC) is a malignant tumor with high metastatic potential originating from the nasopharynx. Although NPC is sensitive to radiotherapy, patients with distant metastatic disease still have very poor prognosis [17]. Epidemiological studies indicate that genetic predisposition, Epstein–Barr virus (EBV) infection, exposure to chemical carcinogens, and certain dietary habits might be etiological factors in NPC oncogenesis and metastasis. Nitro compounds and their metabolites are important carcinogens in NPC. N,N’-dinitrosopiperazine (DNP) is the predominant volatile nitrosamine in pickled foods. Some studies have revealed that DNP can promote NPC carcinogenesis and metastasis in vivo and in vitro [18], DNP may increase metastasis of NPC cells by inducing phosphorylation of ezrin [19], abnormal activation of AGR2 [20], and significantly high expression of clusterin [21].

In this study, we demonstrate a novel molecular mechanism for NPC metastasis. We identified a specific SE for NPC metastasis, which generated a novel seRNA named seRNA-NPCM. The master transcription factor JUN has been verified to be crucial for activating seRNA-NPCM production. The seRNA-NPCM transcribed from the intergenic region promotes NPC metastasis both in vitro and in vivo. In NPC patients, the expression of seRNA-NPCM predicts a poor progress. The seRNA-NPCM forms RNA-DNA hybrids (R-loops) in the SE regions and binds to hnRNPR at the promoter of NDRG1 and TRIB1, which facilitates enhancer–promoter looping and gene transcription. The seRNA-NPCM also directly interacts with ACTA1. These findings have important implications for elucidating molecular mechanisms by which the seRNA-NPCM modulates promoters during NPC metastasis.

## Results

### Global enhancement of SE and seRNA activity in NPC metastasis

Human NPC cell lines S26 and S18, with low transferability and high migration ability, respectively, were used to screen for NPC metastasis enhancers. Their active enhancers were identified by high enrichment of H3K27ac. As a carcinogen for NPC, DNP was used to treat S26 cells to increase their metastasis. First, the ChIP-seq libraries of S18 and S26 were constructed. H3K27ac enrichment using ChIP-seq profiling in the intronic and intergenic regions found 1331 distal active SEs in S18 cells and 819 distal active SEs in S26 cells (Supplementary Fig. S1A, B). SEs increased to 1109 in S26 cells treated with carcinogen DNP (S26-DNP) (Supplementary Fig. S1B). Moreover, the density of average ChIP-seq signals within a ± 10 kb window around the enhancer center for S18 and S26-DNP cells was higher than S26 (Supplementary Fig. S1E). These results show that the number and activity of SEs in S18 and DNP-treated S26 cells were higher than those in S26 cells. Further analysis revealed that several special SEs were related to genes that are essential for oncogenesis and metastasis, including *EGFR*, *FOSL1*, *KLF5*, *ETS1*, etc. (Supplementary Fig. S1A). These results collectively suggest that NPC cells with high migration ability exhibit global enhancement of enhancer activity, as well as enhancement of specific SEs.

GRO-seq was performed on S18 and S26 cells to obtain a panorama of enhancer transcription during NPC metastasis. The GRO-Seq data were studied to clarify transcription units, which were then mapped to the enhancers previously categorized in S18 and S26 cells. SEs exhibited elevated enhancer activities compared to TEs in NPC cells (Supplementary Fig. S1F). Consistently, a higher percentage of SEs generated seRNAs than TE-producing teRNAs (Supplementary Fig. S1C). 825 enhancers were significantly upregulated and 1227 were downregulated (fold change ≥2) in S18 compared to S26 (Supplementary Fig. S1G), following the same trend of H3K27ac level (Supplementary Fig. S1G). This finding suggests that the generation of eRNA is a key marker of active enhancers. According to the gene ontology analysis, the SEs expressing seRNAs in S18 cells were mainly involved in positive regulation of cell migration, while SEs non-expressing seRNAs tended to modulate cell junction assembly and cell-substrate adhesion, suggesting that seRNAs might be important roles in regulation of NPC migration and metastasis (Supplementary Fig. S1I).

To study the correlation between nascent and stable RNA transcripts, the RNA-seq data of NPC cells were reanalyzed and compared with GRO-seq data. When aligned to the NPC genome, GRO-seq signal peaks were highly related with the RNAPII binding sites (Supplementary Fig. S1J). GRO-seq results displayed similar transcription patterns in NPC cells with different metastatic abilities; the only differences were primarily high transcription profiles in NPC cells with high transferability (Supplementary Fig. S1J). Furthermore, the biological relevance of gene alterations at the transcriptional level was evaluated using gene set enrichment analysis (GSEA) of GRO-seq data. The genes were ranked according to fold changes and P value, then the normalized scores of each biological pathway were calculated. The results showed that, in line with H3K27ac ChIP-seq, tumor pathways or markers, including epithelial-to-mesenchymal transition (EMT) and TNFA signaling via NFKB, were significantly upregulated in NPC metastasis (Supplementary Fig. S1D). In addition, regulation of the actin cytoskeleton in S18 was also activated (Supplementary Fig. S1H). eRNAs/seRNAs may be spatiotemporally transcribed in NPC metastasis and act as integral components of SEs.

### Identification of potential seRNAs in NPC metastasis

**Fig. 1:**
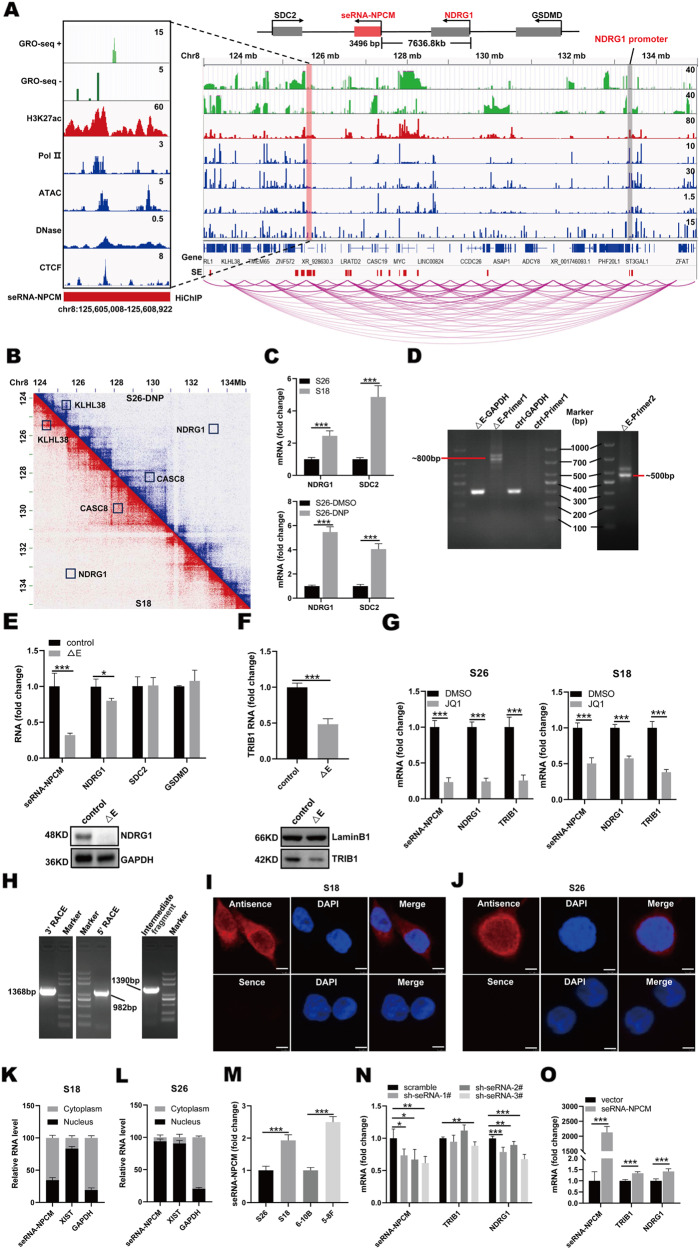
Identification of potential seRNAs in NPC metastasis. **A** Top shows schematic illustration of the genomic structure of seRNA-NPCM relative to NDRG1, SDC2, and GSDMD. Bottom shows enhancer–promoter contacts, ATAC-seq (HepG2, GSE170012), DNase-seq (HepG2, GSE90300), GRO-seq (S18 cells), ChIP-seq for H3K27ac (S18 cells), CTCF (HepG2, GSE170879), and RNAPII (HepG2, GSM935603) at NDRG1 locus. Gray bar highlights the NDRG1 promoter, pink bar stands for SE. **B** HiChIP contact matrix depicting normalized contact frequencies for S18 (red) and S26-DNP (blue) at NDRG1 locus TAD. Gene promoters are highlighted. **C** NDRG1 and SDC2 mRNAs in S18 vs. S26, S26-DNP vs. S26-DMSO (*n* = 3–5 independent experiments). **D** Verified the deletion of seRNA-NPCM locus through CRISPR cas9 by nested PCR. **E** NDRG1 mRNA and protein expression in S18 cells with seRNA-NPCM locus deletion (*n* = 3–5 independent experiments). △E, S18 cells with seRNA-NPCM locus deletion. **F** TRIB1 mRNA and protein level in S18 cells deleting seRNA-NPCM locus region (*n* = 4 independent experiments). △E stands for deletion of seRNA-NPCM locus region. **G** seRNA-NPCM, NDRG1 and TRIB1 mRNA in S18 and S26 cells, treated with JQ1 or DMSO (*n* = 3–5 independent experiments). **H** 5′-RACE and 3′-RACE assays for identifying full length of seRNA-NPCM transcript. **I**–**J** Confocal fluorescence in situ hybridization images showing the localization of seRNA-NPCM in S18 and S26 cells respectively using an antisense probe (red), with nuclei staining with DAPI (blue). Sense probe was used as a negative control. Scale bars: 10 μm. **K**, **L** qRT–PCR analysis of RNAs purified from nuclear and cytosolic fractions of S18 and S26 cells (*n* = 3–5 independent experiments). XIST and GAPDH are nuclear and cytoplasmic markers, respectively. **M** SeRNA-NPCM levels in S26, S18, 6–10B, 5–8F cell lines (*n* = 3–5 independent experiments). **N** Real-time qRT–PCR showing TRIB1 and NDRG1 transcript in S18 cells transfected with shscramble, or sh-seRNA-NPCM (*n* = 4 independent experiments). **O** Real-time qRT–PCR showing the TRIB1 and NDRG1 transcript levels in S26 cells transfected with vector or seRNA-NPCM (*n* = 3–5 independent experiments). Data are expressed as mean ± SEM; *P < 0.05, **P < 0.01, ***P < 0.001.

To further verify the nuclear architecture of SEs in NPC cells, a contact map of active enhancers was generated using the H3K27ac-HiChIP assay. The resulting interaction matrices defined the locations of TADs and sub-TADs in the whole genome (Fig. 1B). Using H3K27ac-HiChIP maps, we examined whether NPC cells had a cell-type-specific nuclear architecture. HiChIP-seq showed that H3K27ac^+^ regions were connected to distal promoters of NPC genes such as *CASC8*, *KLHL38*, and *NDRG1* (Fig. 1A, B). The comparison of interaction frequencies annotated putative SE-promoter contacts, 20,823 interactions were enriched in S18 cells (log2FC > 1 or log2FC < -1; *P* value < 0.05), and 17,669 interactions were detected in S26-DNP cells, suggesting that a large number of SEs can act on distant promoters (Supplementary Data 1). In detail, 7524 genes in S18 were connected to distal regions, 219 of which were significantly upregulated and 219 were downregulated (Supplementary Data 1 and 2). In total, 7320 genes in S26-DNP were connected to distal regions, 178 of which were significantly upregulated and 60 were downregulated (Supplementary Data 1 and 2). The S18 distal SE elements identified in the above studies were highly linked to promoters by gene assignment using HiChIP. The average length of the tumorigenic putative SE-promoter loop was longer than that of the ubiquitous enhancer–promoter loop (Fig. 1A).

To screen for potential eRNAs/seRNAs associated with NPC metastasis and invasion, a specific SE region and its derived seRNA were identified. H3K27ac ChIP-seq and GRO-seq data showed that the seRNA-NPCM locus was a specific SE region (Fig. 1A). To further search for the target genes of this seRNA-expressing SE region, H3K27ac-HiChIP was performed on S18 and S26-DNP cells to test the genomic interaction within these regions. The results showed specific interactions between the SE region and the promoters of NDRG1, SDC2 (syndecan 2), and GSDMD (gasdermin D) (Fig. 1A and Supplementary Data 1). RNA-seq and qRT–PCR analyses further showed that NDRG1 and SDC2 were significantly upregulated at the mRNA level in S18 and S26-DNP cells (Fig. 1C and Supplementary Data 2). An in-depth analysis of H3K27ac ChIP-seq, H3K27ac-HiChIP, and GRO-seq showed extensive overlap at the seRNA-NPCM locus (Fig. 1A). The results further showed that the SE expressing seRNA-NPCM was closely connected to the distal promoter of NDRG1 (Fig. 1A). The reanalysis of publicly available ChIP-seq data showed co-localization of RNAPII and CTCF clusters with the enhancer elements (Fig. 1A). Active enhancers were characterized by H3K27ac enrichment and bi-directional GRO-seq signal (Fig. 1A). These regions need to be accessible so that RNAPII, BRD4 and CTCF can be recruited [22]. To illustrate the way chromatin is integrated in the epigenome of NPC cells, data on chromatin accessibility (ATAC-seq) and occupancy of H3K27ac, RNAP, and the DNA loop regulators CTCF were further analyzed (Fig. 1A). These results revealed that the SE generating seRNA-NPCM was connected with promoters and loop anchors were related to CTCF (Fig. 1A).

### Disruption of SE leads to target genes reduction

To assess the functionality of SEs, the CRISPR Cas9 approach was used to knockout SE. S18 cells were transduced with lentivirus expressing Cas9, together with two pairs of sgRNAs targeting the seRNA-NPCM locus (Fig. 1D). Deletion of the seRNA-NPCM locus resulted in the downregulation of seRNA-NPCM and NDRG1 protein and a slight decrease in NDRG1 mRNA levels (Fig. 1E). The promoter of NDRG1 was identified to have contact with the SE producing seRNA-NPCM by a previous HiChIP assay (Fig. 1A). Although the promoters of SDC2 and GSDMD were connected to SE generating seRNA-NPCM (Fig. 1A), knockout of seRNA-NPCM locus did not change the mRNA levels of SDC2 and GSDMD (Fig. 1E). These results indicate that the seRNA-NPCM locus distal to NDRG1 functions as its SE. In addition, knockout of the seRNA-NPCM locus also led to a decrease in the nearest gene expression of TRIB1 (Fig. 1F), suggesting the SE may play an important role in the regulation of TRIB1 expression. Depletion of SE led to repression of target gene expression.

JQ1 is a special repressor of SE that prevents BRD4 from contacting H3K27ac at SEs, thereby inhibiting seRNAs transcription [23–25]. To detect the effects of JQ1 on seRNA-NPCM expression, S18 and S26 were treated with 0.5 μM JQ1 or dimethyl sulfoxide (DMSO) control for 48 h, and then the transcription of seRNA-NPCM was evaluated by qRT–PCR. Consequently, JQ1 markedly repressed seRNA-NPCM expression and reduced NDRG1 and TRIB1 expression (Fig. 1G). These results confirmed that seRNA-NPCM originated from SE and that NDRG1 and TRIB1 were regulated by SE.

### SeRNA-NPCM promotes target genes expression in NPC cells

To characterize the function of seRNA-NPCM, it was cloned using RACE, and the results revealed that seRNA-NPCM was a 3496 nt transcript (Fig. 1H), the transcripts possessed a polyadenylation site, located at chromosome 8q24.13. The sequence of seRNA-NPCM was listed in Supplementary Data 3. The Coding Potential Assessment Tool revealed a low value for seRNA-NPCM (coding probability = 0.0036). RNA FISH was applied to determine this lncRNA location. The results showed that seRNA-NPCM was distributed in both the nucleus and cytoplasm at S18 (Fig. 1I); however, it appeared to be mainly in the nucleus at S26 (Fig. 1J). These findings were further confirmed using a cellular fractionation assay. As a control, GAPDH RNA was mainly found in the cellular cytoplasm, whereas XIST was found in the nucleus of both S18 and S26. SeRNA-NPCM was slightly enriched in the S18 cytoplasmic fractions (Fig. 1K), whereas it was mainly distributed in the S26 cellular nucleus (Fig. 1L). To gain insight into the functions of seRNAs in NPC cells, seRNA-NPCM expression was detected in several NPC cell lines. The results showed that seRNA-NPCM was highly transcribed in highly metastatic S18 and 5–8F cells, while its transcriptional level was low in low transferability S26 and 6–10B cells (Fig. 1M).

To probe whether seRNA-NPCM activates its neighboring genes, short hairpin RNAs (shRNAs) were used to deplete seRNA-NPCM, and the effect of seRNA-NPCM depletion on the neighboring genes was investigated within a ± 150 kb window. Total seRNA-NPCM was effectively knocked down (Fig. 1N), and loss of seRNA-NPCM resulted in decreased mRNA level of the nearest gene, TRIB1 (Fig. 1N). When seRNA-NPCM was overexpressed in S26, TRIB1 expression was upregulated (Fig. 1O). The above results implied that seRNA-NPCM probably exerted a regulatory effect on the nearest gene through the proximity of its transcription site. In addition, the HiChIP assay showed that the seRNA-NPCM locus was closely linked to the promoter of distal NDRG1 (Fig. 1A), and qRT–PCR showed that the reduction and overexpression of seRNA-NPCM reduced or increased NDRG1 mRNA levels (Fig. 1N, O). Taken together, seRNA-NPCM may regulate the expression of its neighboring target gene, TRIB1, and the distant target gene, NDRG1.

### JUN induces seRNA-NPCM production

**Fig. 2:**
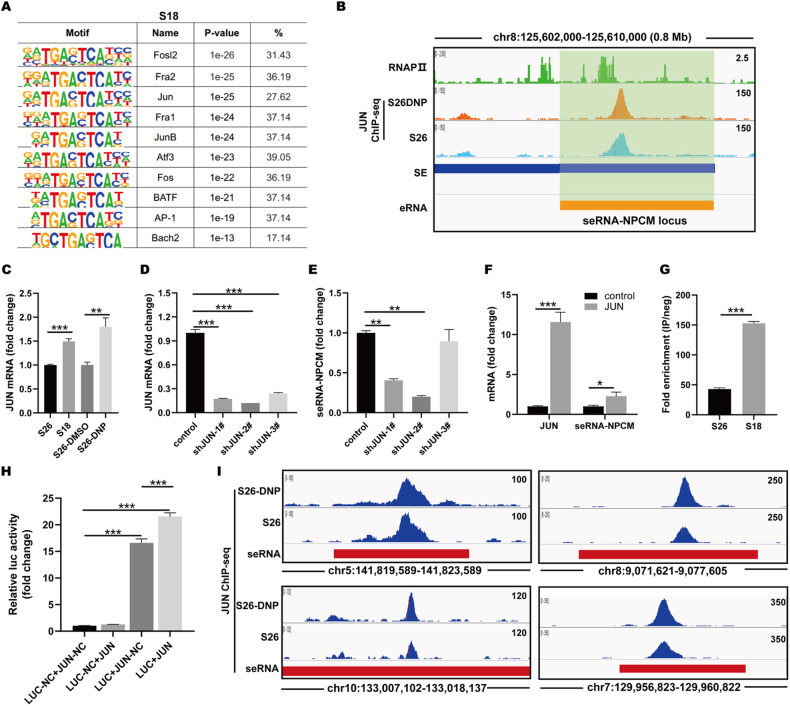
JUN induces seRNA-NPCM production. **A** DNA-binding motifs enriched within the ±2 kb window of TSSs of seRNAs. **B** ChIP-seq of JUN binding to seRNA-NPCM TSS region in line with RNAPII. H3K27ac ChIP-seq and GRO-seq were performed in S26-DNP cells. RNAPII ChIP-seq data were downloaded from ENCODE (HepG2, GSM935603). **C** JUN mRNA level in S26, S18, and S26-DNP cells (*n* = 3–5 independent experiments). **D**, **E** qRT–PCR detection of seRNA-NPCM from S18 transfected with either shJUN or scramble (*n* = 4 independent experiments). **F** qRT–PCR detection of seRNA-NPCM from S26 transfected with empty vector or JUN plasmid (*n* = 4 independent experiments). **G** JUN ChIP-PCR at TSS of seRNA-NPCM in S18 and S26 cells. **H** Luciferase reporter activity of the seRNA-NPCM promoter in 293T cells transfected with JUN plasmid or control (*n* = 2 independent experiments). **I** TF JUN binding to other seRNAs TSS regions in S26 and S26-DNP. Data are expressed as mean ± SEM; *P < 0.05, **P < 0.01, ***P < 0.001.

The next step was to confirm the key TFs that regulate seRNA-NPCM expression in NPC metastasis. HOMER was applied to predict potential TF binding sites located in the ±2 kb region of TSSs of eRNAs that were generated by specific SEs, and the distal target genes of SEs were significantly regulated (log2FC > 2 or <−2, *P* value < 0.05). Interestingly, the same top-ranked motifs were bound by key TFs essential for tumor metastasis and invasion, including Fosl2 and JUN (Fig. 2A and Supplementary Fig. S2A). To further observe the map of eRNAs under the regulation of master TF JUN, JUN ChIP-seq was intersected with the GRO-seq dataset. In the enhancer transcription units identified by GRO-seq, 27.62% of specific SEs expressing seRNAs in S18 and 26.32% in S26-DNP were bound to TF JUN at their TSS, indicating that JUN binding to seRNA TSS is prevalent in both S18 and S26-DNP. In addition, JUN mRNA levels were elevated in both S18 and S26-DNP using RNA-seq (Supplementary Data 2) and RT-qPCR (Fig. 2C). These results suggest that TF JUN may play a crucial role in eRNA induction during NPC metastasis and invasion. Further, ChIP-seq showed that JUN bound to the seRNA-NPCM TSS region in line with RNAPII (Fig. 2B), suggesting that JUN may regulate the production of seRNA-NPCM.

To confirm these findings, shRNAs were designed to knockdown JUN (Fig. 2D). JUN knockdown decreased seRNA-NPCM expression in S18 cells (Fig. 2E), supporting the activating role of JUN in seRNA-NPCM induction. Furthermore, when JUN was overexpressed in S26 cells, seRNA-NPCM expression increased (Fig. 2F). To further elaborate on the direct regulatory effect of JUN, JUN binding was confirmed by ChIP-PCR on seRNA-NPCM loci. ChIP-PCR results suggested that JUN was associated with seRNA-NPCM TSS, and its binding was increased in S18 compared to that in S26 (Fig. 2G). In addition, the TSS regions containing JUN binding sites were cloned into a reporter construct, and their activity was detected. The resulting reporter activity was significantly increased (Fig. 2H). Promoter activity also displayed a concomitant increase with JUN overexpression (Fig. 2H). Moreover, direct binding of JUN to the promoters of seRNAs was detected (Fig. 2I), indicating that JUN induction of seRNA was a crucial phenomenon during JUN-directed NPC metastasis.

### SeRNA-NPCM interacts with hnRNPR and ACTA1

**Fig. 3:**
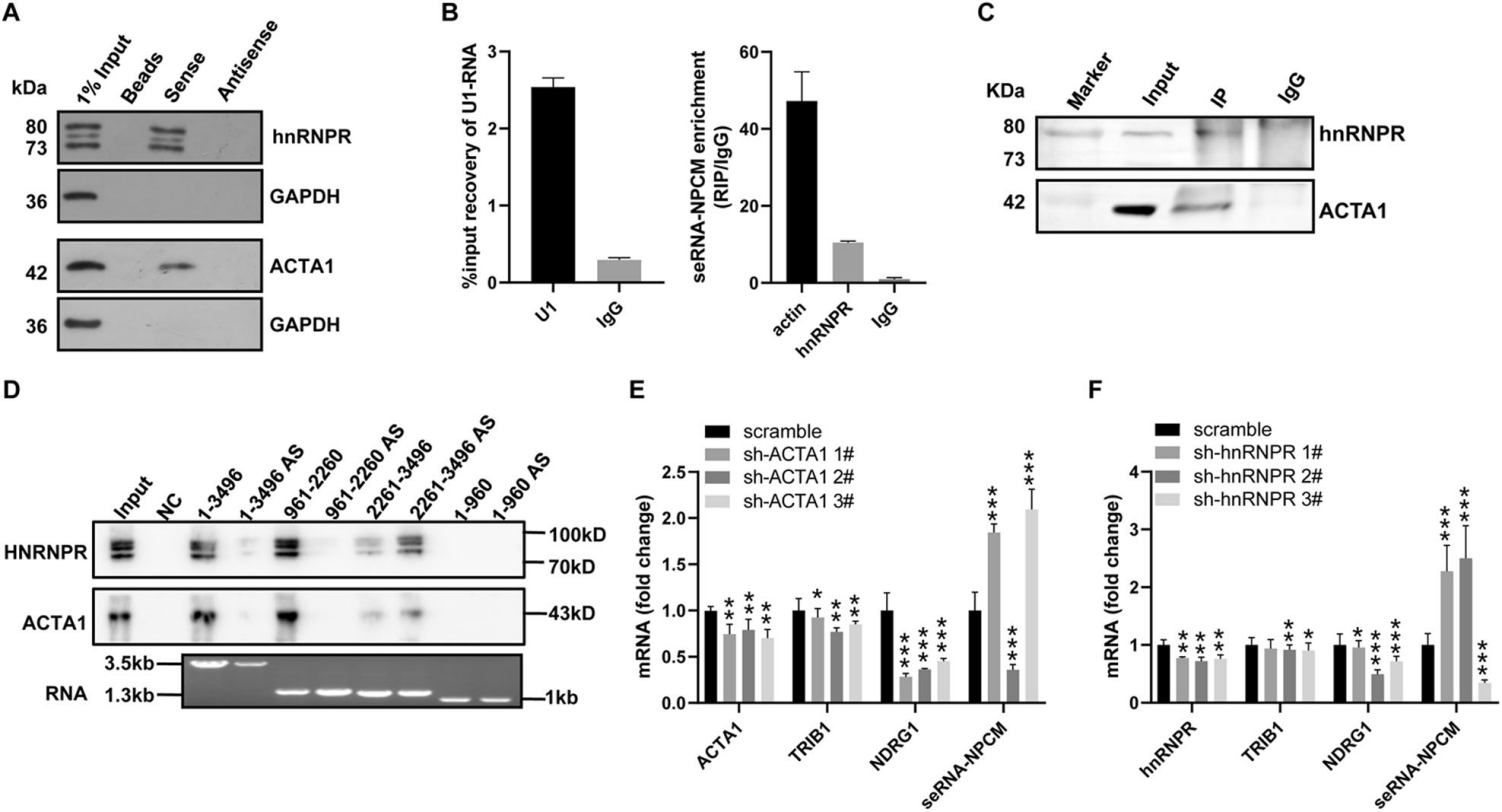
SeRNA-NPCM interacts with hnRNPR and ACTA1. **A** Western blotting analysis on the association of seRNA-NPCM with hnRNPR and ACTA1 in S18 cells. GAPDH served negative control. **B** RNA immunoprecipitation (RIP) with antibody against ACTA1 or hnRNPR in non-crosslinked S18 cells transfected with seRNA-NPCM and qRT–PCR analysis of retrieved RNAs. IgG-bound RNA served as a negative control, U1 as a positive control. **C** Western blotting assay confirming hnRNPR and ACTA1 enrich target protein in RIP. IgG was a negative control. **D** The indicated deletion fragments of seRNA-NPCM and its interacting region with ACTA1 or hnRNPR. AS stands for antisense. **E**, **F** S18 cells were transfected with shRNAs targeting ACTA1, hnRNPR or scrambled negative control. The expression of target genes was measured by qRT–PCR (*n* = 4 independent experiments). Data are expressed as mean ± SEM; *P < 0.05, **P < 0.01, ***P < 0.001.

To investigate the molecular mechanisms underlying the seRNA-NPCM-mediated activation of target genes, their protein partners were identified using an RNA pull-down test. Biotinylated seRNA-NPCM synthesized in vitro was used to capture protein partners. Overlapping analysis of subsequent mass spectrometry (MS) results revealed hnRNPR and ACTA1 as seRNA-NPCM interacting proteins (with 171 detected peptides; Supplementary Data 4), which was verified by western blotting in the seRNA-NPCM pull-down complex (Fig. 3A). G3BP1, EIF2AK2, TRIM25, and ACTA1 were among the top-ranked proteins, and these were closely related to tumor proliferation, metastasis, and invasion, suggesting that seRNA-NPCM may play a crucial role in NPC tumorigenesis and development. Pull-down ACTA1 by seRNA-NPCM revealed that seRNA-NPCM might act in trans to directly interact with ACTA1 to participate in NPC migration. seRNA-NPCM also strongly retrieved heterogeneous nuclear ribonucleoprotein family members, hnRNPR (Fig. 3A). HNRNP family proteins have been found to interact with eRNAs [9, 26]. Therefore, the seRNA-NPCM/ACTA1 and seRNA-NPCM/hnRNPR associations were further explored to gain mechanistic insight. Common eRNA/lncRNA partners, including CREB-binding protein (CBP), Mediator Complex Subunit 1 (MED1), and YY1, were not found to bind with seRNA-NPCM (Supplementary Data 4), implying that seRNA-NPCM had specific partners. Furthermore, a native RIP assay was used to analyze the extracts of S18 cells, and the results showed that the seRNA-NPCM was retrieved by ACTA1 and hnRNPR antibodies (Fig. 3B, C). These results revealed that ACTA1 and hnRNPR are real protein partners of seRNA-NPCM. For a deeper understanding of their binding way, several truncated fragments of seRNA-NPCM were used to map the ACTA1- and hnRNPR-interacting regions. The results showed a 1300 nt region in the middle of seRNA-NPCM (961–2260 nt) and a 1236 nt region at the 3’ end (2261–3496 nt) were essential in mediating the interaction between hnRNPR and ACTA1 (Fig. 3D). In order to examine the functional relevance of seRNA-NPCM/ACTA1 and seRNA-NPCM/hnRNPR, hnRNPR, and ACTA1 were knocked down (Fig. 3E, F), seRNA-NPCM target genes, TRIB1 and NDRG1, were significantly decreased, while seRNA-NPCM itself was upregulated (Fig. 3E, F), suggesting a synergistic effect of hnRNPR or ACTA1 with seRNA-NPCM in facilitating target gene transcription. Moreover, the seRNA-NPCM transcription may be regulated by the hnRNPR/seRNA-NPCM or ACTA1/seRNA-NPCM interaction.

### SeRNA-NPCM modulates chromatin looping via R-loop formation and connection with hnRNPR to promote target gene transcription

**Fig. 4:**
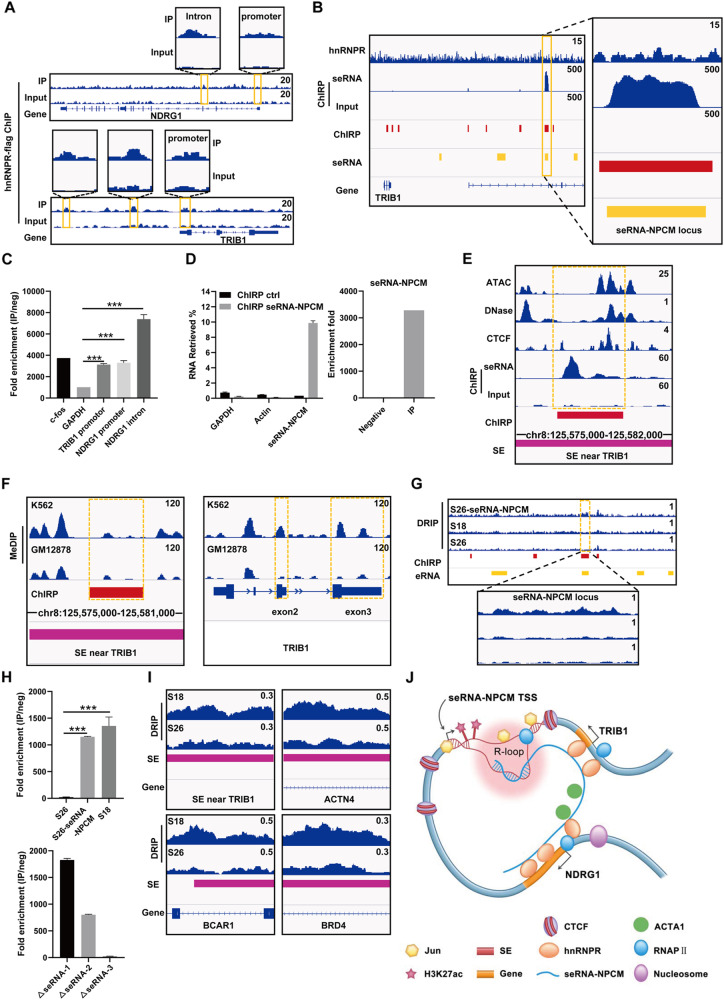
SeRNA-NPCM modulates chromatin looping via R-loop formation and connection with hnRNPR to promote target gene transcription. **A** hnRNPR-flag ChIP-seq signals at TRIB1 and NDRG1 genes. hnRNPR-flag ChIP-seq was performed in S18 cells transfected with hnRNPR-flag. **B** SeRNA-NPCM ChIRP-seq and hnRNPR-flag ChIP-seq signals at seRNA-NPCM locus. ChIRP-seq was performed in S26 cells transfected with seRNA-NPCM. **C** qRT–PCR verified hnRNPR enrichment in TRIB1 and NDRG1 promoters. GAPDH and c-fos were negative and positive control, respectively. **D** qPCR verified seRNA-NPCM enrichment at seRNA-NPCM locus. GAPDH and actin were negative control. **E** Signals of seRNA-NPCM ChIRP-seq, ATAC-seq (HepG2, GSE170012), DNase-seq (HepG2, GSE90300), and CTCF ChIP-seq (HepG2, GSE170879) at a SE near TRIB1. ChIRP-seq was performed in S26 cells transfected with seRNA-NPCM. **F** MeDIP-seq signals in exons of TRIB1, SE near TRIB1. MeDIP-seq data were downloaded from ENCODE database, K562 (GSM1368906) and GM12878 (GSM1368907). ChIRP-seq was performed in S26 cells transfected with seRNA-NPCM. **G** The R-loop signals in the seRNA-NPCM locus detected by DRIP-seq. **H** Top, qPCR detected the R-loop enrichment of seRNA-NPCM locus in S26, S18, and S26 cells overexpressing seRNA-NPCM; bottom, qPCR detected the R-loop enrichment at the seRNA-NPCM locus in S26 cells expressing different fragments of seRNA-NPCm. △seRNA-1 represents the deletion of 1–960 nt of seRNA-NPCM, △seRNA-2 represents the deletion of 961–2260 nt of seRNA-NPCM, △seRNA-3 represents the deletion of 2261–3496 nt of seRNA-NPCM. **I** The R-loop signals of some SEs in S26 vs. S18. **J** The model of seRNA-NPCM regulating NDRG1 and TRIB1 expression through R-loop formation. Data are expressed as mean ± SEM; *P < 0.05, **P < 0.01, ***P < 0.001.

hnRNPR is known to modulate RNA splicing and is directly involved in transcription. hnRNPR stabilizes CCNB1 and CENPF mRNA to promote tumor metastasis [27]. SeRNA-NPCM may bind and tether hnRNPR to the gene promoter to upregulate transcription. To test this hypothesis, the seRNA-hnRNPR connection was verified at NDRG1 and TRIB1 gene regions. The above results showed knockdown of hnRNPR led to a decrease of NDRG1 and TRIB1 (Fig. 3F), suggesting hnRNPR may play a crucial role in regulating the transcription of these two genes. hnRNPR ChIP-seq results suggested that hnRNPR was associated with the NDRG1 and TRIB1 (introns, promoters, and intergenic regions) (Fig. 4A); these results were verified by ChIP-PCR (Fig. 4C). Together, these results support the notion that seRNA-NPCM/hnRNPR can bind to the NDRG1 or TRIB1 locus and regulate transcription.

Then, the seRNA-NPCM binding sites in the genome were confirmed. The expression of seRNA-NPCM in NPC cells was very low, it had to be overexpressed in order to meet the ChIRP detection needs. For ChIRP-seq, 38 specific RNA probes were synthesized against the seRNA-NPCM transcript and captured with magnetic beads. There were powerful specific signals in the seRNA-NPCM locus regions (Fig. 4B). qPCR confirmed that the seRNA-NPCM was located in the seRNA-NPCM locus region (Fig. 4D). In addition, seRNA-NPCM directly bound to the hnRNPR promoter (Supplementary Fig. S2B), suggesting that it may play an important role in hnRNPR expression. To determine how seRNA-NPCM binds to function in SE regions, the public data from ATAC-seq, DNase-seq, and CTCF ChIP-seq were reanalyzed. The co-localization of seRNA-NPCM targeted regions with CTCF clusters and chromatin accessibility (Figs. 1A and 4E), suggesting that seRNA-NPCM might act in chromatin looping. Interestingly, in contrast to hypermethylation in TRIB1 variable exons, the SE near TRIB1 were hypomethylated in both K562 and GM12878 cells (Fig. 4F). The R-loop structure is typically constructed by local RNAs in the hypomethylated and DNase hypersensitive region [28, 29]. Local seRNA-NPCM may form R-loop structures in SE regions. To this end, DRIP assay with the S9.6 antibody was used to recognize DNA-RNA hybrids (R-loop). The DRIP-seq results showed that the signals were significantly enriched in SEs of S18 compared to S26 (Fig. 4G, I). qPCR verified that the enrichment of R-loop at seRNA-NPCM locus in S18 cells was much higher than that in S26 cells, and the overexpression of seRNA-NPCM would increased R-loop enrichment, suggesting that seRNA-NPCM formed R-loop in site (Fig. 4H). Furthermore, qPCR analysis of S26 cells transfected with truncated fragments of seRNA-NPCM showed that R-loop enrichment at seRNA-NPCM locus significantly decreased after the deletion of the 3’ end (2261–3496 nt) (Fig. 4H), suggesting that the 3’ end of seRNA-NPCM may be essential for interaction with DNA.

Above results revealed that seRNA-NPCM was located in seRNA-NPCM locus and formed R-loop in site. hnRNPR, the protein partner of seRNA-NPCM, bound seRNA-NPCM to the promoter of TRIB1 and NDRG1. This structure promotes chromatin looping and long-distance chromatin interactions between SE and promoters, thus facilitating NDRG1 and TRIB1 transcription (Fig. 4J). Knocking down seRNA-NPCM or hnRNPR will reduce the expression of NDRG1 and TRIB1 (Fig. 1N). In addition, R-loop structures were detected in 88.2% SEs (1133/1284) in S18 and 60.3% SEs (494/819) in S26 (Supplementary Data 5), suggesting it was a general phenomenon for SE/seRNA to regulate target genes through R-loop formation.

### SeRNA-NPCM promotes NPC metastasis in vitro and in vitro

**Fig. 5:**
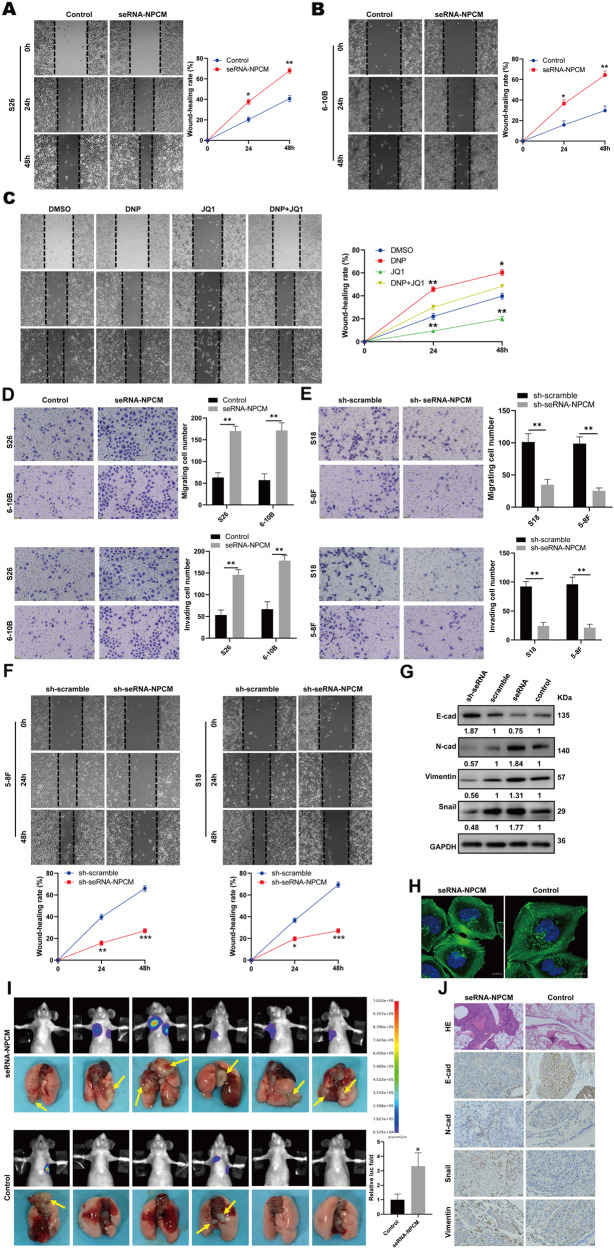
SeRNA-NPCM promoting NPC metastasis in vitro and in vivo. **A**, **B** Migration of S26 and 6–10B cells respectively transfected with seRNA-NPCM and vector control (*n* = 3 independent experiments). **C** Migration of S26 cells treated with DNP, JQ1, DNP + JQ1, and DMSO (*n* = 3 independent experiments). **D** Metastasis and invasion of S26 and 6–10B cells transfected with seRNA-NPCM or vector (*n* = 4 independent experiments). **E** Metastasis and invasion of S18 and 5–8F cells transfected with sh-seRNA-NPCM or shscramble (*n* = 4 independent experiments). **F** Migration of S18 and 5–8F cells transfected with sh-seRNA-NPCM or shscramble (*n* = 4 independent experiments). **G** E-cadherin, N-cadherin, vimentin, and Snail expression in S18 cells transfected with sh-seRNA-NPCM or shscramble, and S26 cells respectively transfected with seRNA-NPCM or control. **H** F-actin and stress fiber expressions in the cells with seRNA-NPCM overexpression. Scale bars, 10 μm. **I** In vivo imaging of nude mice metastatic status by luciferase-based bioluminescence imaging. Yellow arrow, metastatic nodules. **J** IHC staining of E-cadherin, vimentin, and Snail in xenograft tumor model. Scale bars, 100 μm. Data are expressed as mean ± SEM; *P < 0.05, **P < 0.01, ***P < 0.001.

To explore the function of seRNA-NPCM in NPC metastasis, the low metastatic cell lines S26 and 6–10B with low expression of seRNA-NPCM were used to increase ectopic expression of seRNA-NPCM (Fig. 1O). The wound-healing assay showed that overexpression of seRNA-NPCM enhanced the migratory capacity of both S26 and 6–10B cells (Fig. 5A, B). In addition, elevated levels of seRNA-NPCM enhanced the migratory capacity of S26 cells induced with DNP, whereas this cell migration was blocked by JQ1 treatment (Fig. 5C). In Transwell assays, overexpression of seRNA-NPCM facilitated in vitro metastasis and invasion of S26 and 6–10B cells (Fig. 5D). Knockdown of seRNA-NPCM significantly decreased the motility of the highly metastatic cancer cells S18 and 5–8F (Fig. 5E, F). In keeping with this, the upregulation of seRNA-NPCM promoted the expression of EMT proteins such as N-cadherin, Snail, and vimentin, but the expression of E-cadherin was reduced (Fig. 5G). In contrast, the downregulation of seRNA-NPCM reversed the EMT phenotype (Fig. 5G). In addition, seRNA-NPCM also promoted the formation of F-actin and stress fibers (Fig. 5H). These results suggest that seRNA-NPCM might promote NPC metastasis via EMT and cytoskeletal reconstruction.

To further evaluate the metastatic effect of seRNA-NPCM on NPC cells in vivo, initially, 2 × 10^6^ S26 cells transfected with vector-luciferase or seRNA-NPCM-luciferase were injected into the tail veins of nude mice. After 30 days of injection, the fluorescence intensity was detected in the mice, and their lungs were excised for examination. The results showed that S26 cells stably overexpressing seRNA-NPCM presented larger metastatic lesions than the control (Fig. 5I). Immunohistochemical (IHC) staining of pulmonary metastases showed that protein N-cadherin, Snail, and vimentin were elevated, and E-cadherin was decreased in the seRNA-NPCM group (Fig. 5J). Altogether, these findings revealed that seRNA-NPCM markedly contributed to NPC metastasis both in vitro and in vivo.

### SeRNA-NPCM harbors oncogenic properties through hnRNPR-mediated NDRG1 expression

**Fig. 6:**
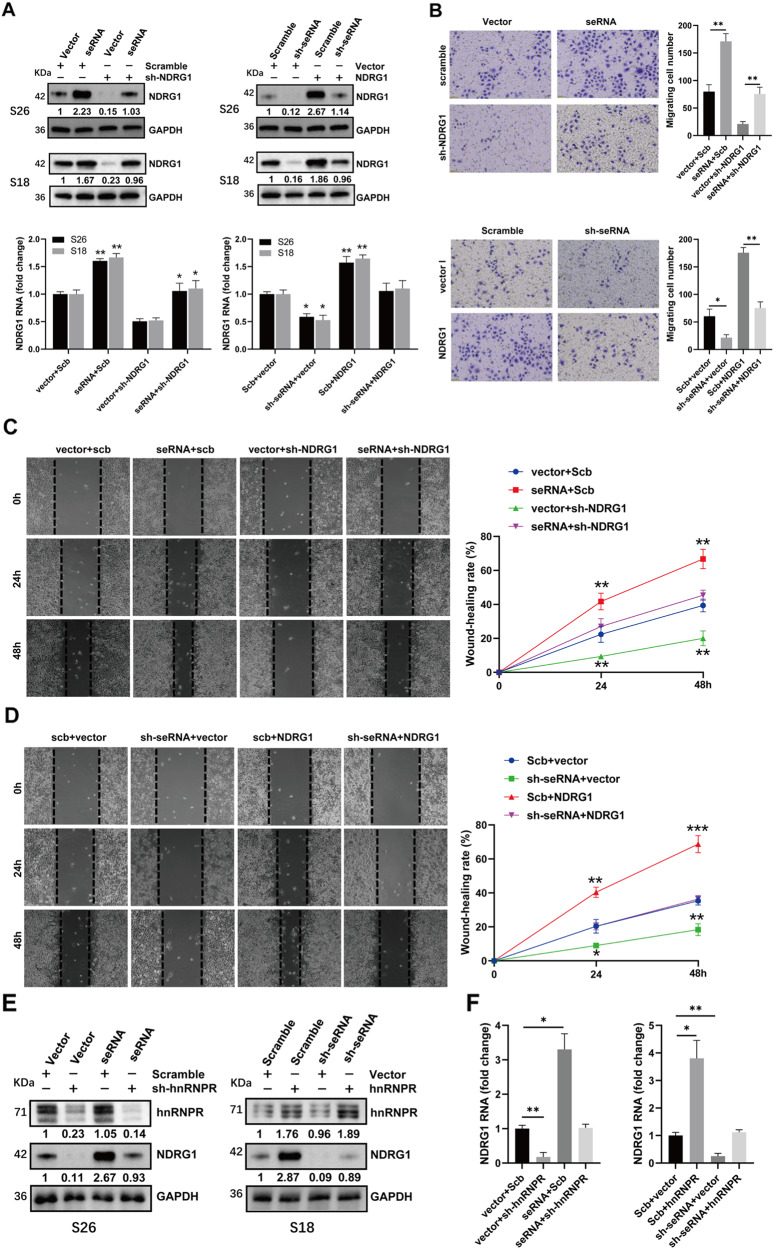
SeRNA-NPCM harbors oncogenic properties through hnRNPR-mediated NDRG1 expression. **A** NDRG1 expression in the cells transfected with vector, seRNA-NPCM, scramble shRNA or sh-seRNA-NPCM, and those co-transfected with shNDRG1 or NDRG1 (top). NDRG1 transcription in the cells transfected with vector, seRNA-NPCM, shscramble or sh-seRNA-NPCM, and those co-transfected with shNDRG1 or NDRG1 (bottom). **B**–**D** Migration and invasion capability of the cells transfected with vector, seRNA-NPCM, shscramble or sh-seRNA-NPCM, and those co-transfected with shNDRG1 or NDRG1 (*n* = 4 independent experiments). **E** hnRNPR expression in the cells transfected with vector, seRNA-NPCM, scramble shRNA or sh-seRNA-NPCM, and those co-transfected with sh-hnRNPR or hnRNPR. **F** NDRG1 transcription in S26 cells transfected with vector, seRNA-NPCM, shscramble or sh-seRNA-NPCM, and those co-transfected with sh-hnRNPR or hnRNPR. Data are expressed as mean ± SEM; *P < 0.05, **P < 0.01, ***P < 0.001.

**Fig. 7:**
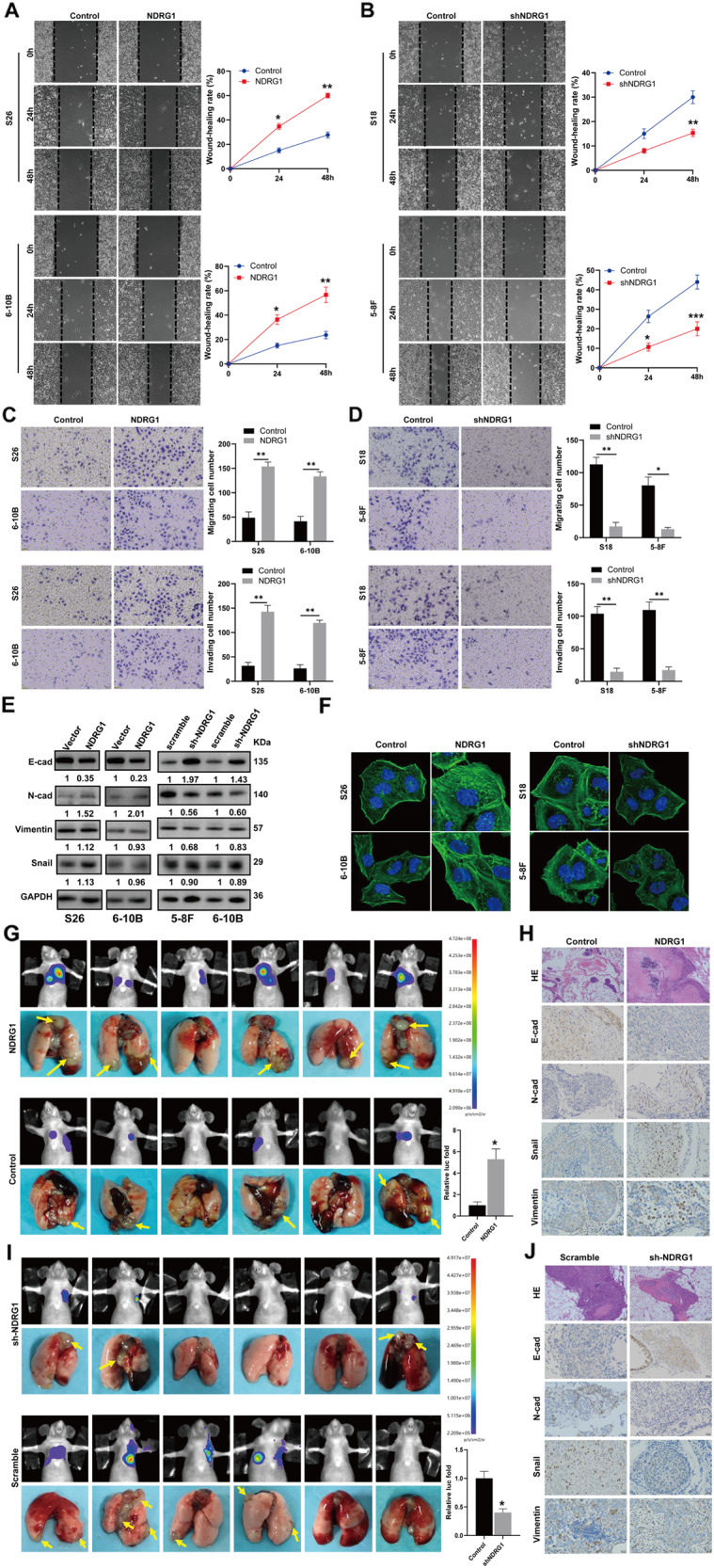
NDRG1 enhancing the motility of NPC cells in vitro and in vivo through EMT and cytoskeleton reconstruction. **A** Migration in S26 and 6–10B cells transfected with NDRG1 or vector control (*n* = 4 independent experiments). **B** Migration in S18 and 5–8F cells transfected with shNDRG1 or shscramble (*n* = 4 independent experiments). **C** Metastasis and invasion in S26 and 6–10B cells transfected with NDRG1 or control (*n* = 4 independent experiments). **D** Metastasis and invasion in S18, and 5–8F cells transfected with shNDRG1 or shscramble (*n* = 4 independent experiments). **E** N-cadherin, E-cadherin, Snail, and vimentin expressions in the cells transfected with vector, NDRG1, shscramble or shNDRG1. **F** F-actin formation of the cells transfected with vector, NDRG1, shscramble or shNDRG1. Scale bars, 10 μm. **G**, **I** The metastases of nude mice injected with the cells upregulated or downregulated NDRG1 by luciferase-based bioluminescence imaging. **H**, **J** IHC staining of EMT markers in the xenograft tumors. Scale bars, 100 μm. Data are expressed as mean ± SEM; *P < 0.05, **P < 0.01, ***P < 0.001.

Rescue experiments were performed to confirm the function of seRNA-NPCM in the NPC cells. The results showed that NDRG1 overexpression prevented NPC cells from reducing the expression of NDRG1 following seRNA-NPCM knockdown (Fig. 6A). In contrast, NDRG1 knockdown also rescued NPC cells overexpressing seRNA-NPCM, followed by increased expression of NDRG1 (Fig. 6A). We further probed whether the restoration of NDRG1 could rescue the phenotype generated by seRNA-NPCM. In Transwell and wound-healing assays, NPC cells stably transfected with NDRG1 or shNDRG1 displayed an increase or decrease in metastasis and migration (Fig. 7A–D). Recovery of NDRG1 levels through transfection with seRNA-NPCM or sh-seRNA-NPCM prevented NPC cells from altered phenotypes caused by downregulation or upregulation of NDRG1 (Fig. 6B–D). These results revealed that seRNA-NPCM harbors oncogenic capabilities by modulating NDRG1 expression. Furthermore, the above ChIP and qPCR results showed that seRNA-NPCM might regulate NDRG1 through binding to protein partner hnRNPR. Knockdown or overexpression of hnRNPR downregulated or upregulated the expression of NDRG1 in NPC cells, while overexpression or knockdown of seRNA-NPCM could restore NDRG1 expression (Fig. 6E, F). These results revealed that seRNA-NPCM regulated NDRG1 expression by interacting with hnRNPR.

### NDRG1 enhances the motility of NPC cells in vitro and in vivo

The effects of NDRG1 on cell motility were assessed in vitro using wound-healing and Transwell assays. The results showed that the migratory ability of S26 and 6–10B cells overexpressing NDRG1 was observably higher than control cells (Fig. 7A). The Transwell assay showed that NDRG1 overexpression significantly increased the metastatic ability of S26 and 6–10B cells (Fig. 7C). In addition, the Transwell invasion assay showed that the invasiveness of both S26 and 6–10B cells overexpressing NDRG1 was significantly higher than that of the corresponding control cells (Fig. 7C). These findings were further validated in S18 and 5–8F cells, which exhibited higher viability than S26 and 6–10B cells. The results showed that NDRG1 knockdown inhibited S18 and 5–8F cells in vitro migration and invasion (Fig. 7B, D). NDRG1 overexpression promotes EMT proteins, as evidenced by decreased E-cadherin and increased N-cadherin expression. However, the Snail and vimentin levels changed little (Fig. 7E). In contrast, NDRG1 knockdown reversed the EMT phenotype (Fig. 7E). In addition, NDRG1 overexpression increased F-actin and stress fibers, whereas NDRG1 knockdown reduced F-actin and stress fibers (Fig. 7F). NDRG1 may promote cytoskeletal reconstruction. These results suggest that NDRG1 significantly promotes cell metastasis and invasion in vitro.

To further explore whether ectopic expression of NDRG1 promotes tumor metastasis in vivo, S26 cells overexpressing NDRG1 or transfected with an empty vector were injected into the tail vein of nude mice. The results showed that overexpression of NDRG1 significantly increased the invasive activity of S26 cells and their ability to form metastases in the lungs (Fig. 7G). In contrast, NDRG1 knockdown reduced the invasive activity of S18 cells and formation of metastases (Fig. 7I). Furthermore, IHC analysis revealed that overexpression of NDRG1 increased N-cadherin, Snail, and vimentin but decreased E-cadherin expression in pulmonary metastases (Fig. 7H), knockdown of NDRG1 decreased N-cadherin, Snail, and vimentin expression but increased E-cadherin expression in pulmonary metastases (Fig. 7J). Together, these results reveal that NDRG1 significantly promotes NPC cell metastasis in vivo.

### seRNA-NPCM and NDRG1 predict a poor clinical outcome in NPC patients

**Fig. 8:**
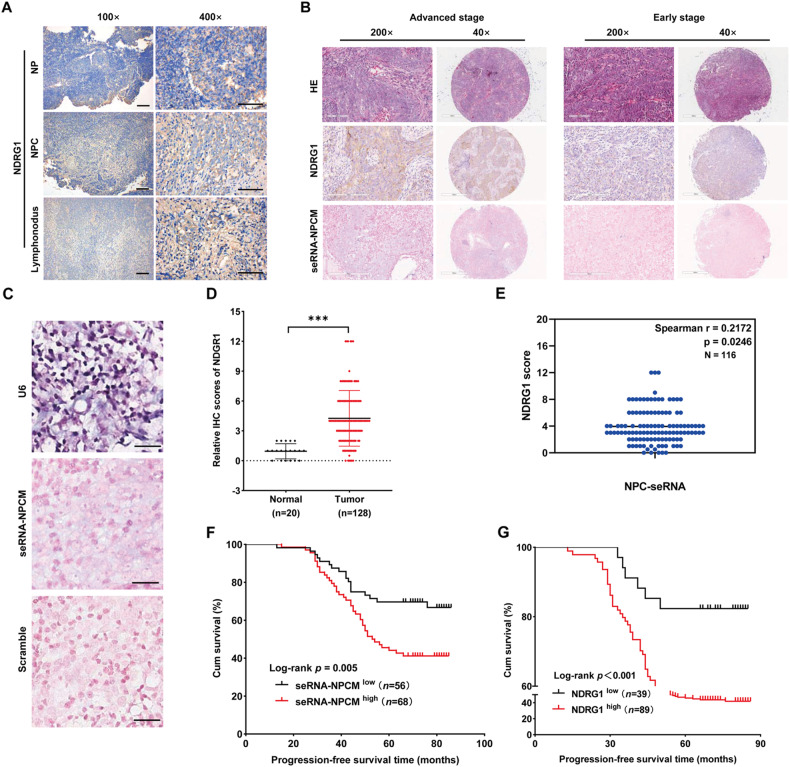
seRNA-NPCM and NDRG1 predict a poor prognosis of NPC patients. **A** The expression and localization of NDRG1 in NPC and normal nasopharynx tissue samples by IHC staining. **B**, **C** Expression of NDRG1 and seRNA-NPCM in NPC patients with different stages, scramble and U6 were used to be negative and positive control, respectively. HE staining results were downloaded from Shanghai Outdo Biotech Corporation. **D** NDRG1 expression in 128 NPC patients. One sample was disqualification. In total, 20 normal cases were used to be control. **E** Spearman’s correlation analysis of seRNA-NPCM and NDRG. **F**, **G** Kaplan–Meier survival curve of NPC patients with high (red) or low (black) expression of seRNA-NPCM (left) and NDRG1 (right).

To investigate the clinical value of NDRG1 in NPC progression, 20 NPC and NPC metastatic tissues and 20 normal cases were used for IHC staining with the NDRG1 antibody. The results revealed that NDRG1 was mostly present in the cytoplasm (Fig. 8A), its expression of NDRG1 was higher in NPC and metastatic tissues than in the normal nasal mucosa. ISH/IHC was used to examine seRNA-NPCM and NDRG1 expression in a tissue microarray containing 129 NPC samples. Compared to normal nasal mucosa, higher NDRG1 and seRNA-NPCM levels were observed in NPC tissues, especially in samples with advanced stages (Fig. 8B–D). In addition, Spearman’s correlation analysis of seRNA-NPCM and NDRG was conducted, eight samples were disqualification, and eight pairs of outliers were excluded. seRNA-NPCM was positively correlated with NDRG1 in the NPC tissues, (*R*, 0.2172, *P* value, 0.0246) (Fig. 8E). Patients with high expression of seRNA-NPCM (*P* value, 0.005) and NDRG1 (*P* value, <0.001) tended to have shorter progression-free survival times (Fig. 8F, G). Multivariate Cox regression analysis showed that TNM stage [hazard ratio (HR), 2.151; *P* value, 0.001], seRNA-NPCM levels (HR, 1.866; *P* value, 0.034), and NDRG1 expression (HR, 3.314; *P* value, 0.015) were independent prognostic factors for NPC patients (Supplementary Data 7). These findings suggest that seRNA-NPCM and NDRG1 expression predict poor clinical outcomes in cancer patients.

## Discussion

eRNAs/seRNAs are ncRNAs that function in enhancer–promoter looping and mRNA transcription [9, 12, 30]. eRNAs/seRNAs interact with macromolecules, including DNA, RNA, and proteins, and promote tumorigenesis in various malignancies [31]. This study introduces a novel seRNA-NPCM that participates in NPC metastasis and proposes a potential molecular mechanism for NPC metastasis. We analyzed ChIP-seq, GRO-seq, HiChIP, and publicly available datasets to gain an in-depth analysis of SEs and seRNAs on NPC metastasis. We showed that seRNA-NPCM may be an eRNA related to NPC metastasis. Many studies have revealed that eRNAs generated from active enhancers play a critical role in oncogene transcriptional regulation by driving chromatin remodeling and looping [32–34]. A JUN-regulated seRNA-NPCM was identified to drive chromatin looping between the SE and distal NDRG1 promoter via R-loop formation in NPC metastasis. SeRNA-NPCM bound to hnRNPR transports a localized activity profile to the target chromatin, in accordance with several previous findings [14, 35]. In addition, rescue experiments showed that the oncogenic roles of seRNA-NPCM are mediated, at least in part, by regulation of NDRG1 expression; this suggests that SE and its derived seRNA-NPCM play a crucial role in NPC invasion and metastasis. We next found that seRNA-NPCM served as a prognostic factor for poor outcome in NPC and promoted in vitro and in vivo metastasis of NPC cells.

HnRNPR is an hnRNP family member that is required for RNA alternative splicing [36], subcellular transport [37] and RNA stabilization [38]. The present findings demonstrate the involvement of hnRNPR in transcriptional control by binding to seRNA and anchoring target genes. HnRNPR has multifunctional properties and may play a role in transcriptional regulation. Recombinant hnRNPR enhanced the transcriptional activity of the promoter of the proto-oncogene c-fos [39]. In tumor migration, hnRNPR directly interacts with CCNB1 and CENPF mRNA to contribute to tumor malignancy and poor outcomes in cancer patients. Ectopic hnRNPR expression increases CCNB1 and CENPF expression and mediates cell invasion and migration [27]. In this study, hnRNPR was identified as a protein that interacted with seRNA-NPCM. HnRNPs are essential for eRNA-mediated transcriptional repression [9, 26] and are closely associated with higher-order organization of chromatin [40]. HnRNPR is essential for seRNA-NPCM-facilitated NDRG1 transcription in NPC cells. HnRNPR contains two RNA recognition motifs (RRM1 and RRM2) that allow specific interactions with mRNAs [41]. HnRNPR interacts directly with the 3’-UTR of β-actin mRNA, which is necessary for the subcellular translocation of β-actin mRNA [37]. In the cell nucleus, actin is also involved in transcriptional regulation as part of chromatin remodeling complexes [42, 43] and as a component of RNP particles [44]. Nuclear actin facilitates RNAPII cluster production via actin polymerization regulated by N-WASP/Arp2/3 [45]. Actin has been found to interact with specific hnRNPs in mammalian pre-mRNP particles [46], and actin in complex with hnRNP U may play a regulatory role during the early stages of transcriptional activation [47]. In our study, seRNA-NPCM was shown to interact with ACTA1 protein, and ACTA1 may cooperate with hnRNPR for productive transcription by RNAPII. ACTA1 is an important protein in cytoskeletal regulation. The dysregulated expression of ACTA1 subunit is prone to form stress fibers and F-actin, which are associated with cytoskeletal stabilization, cell survival, proliferation, and migration [48]. SeRNA-NPCM may facilitate hnRNPR binding to ACTA1; thus, the seRNA-bound RGG domain may cause a structural change in hnRNPR that facilitates its interaction with ACTA1.

JUN is one of the most essential proto-oncogenes involved in cell growth and neoplasia [49]. JUN can form a complex with other transcriptional regulators such as YAP/TAZ/TEAD [50], SWI/SNF (BAF) [51], and ARID1A [52] to regulate chromatin accessibility between distal enhancers and promoters via chromatin looping, thereby synergistically activating target genes [52]. Our analyses revealed that JUN was responsible for induction of seRNA-NPCM. TF JUN binds to several activated SEs and may play a crucial role in NPC metastasis and invasion. In addition, JUN ChIP-seq results of S26 and S26-DNP cells showed a strong signal at the promoter of hnRPNR (Fig. S2F), suggesting that JUN might bind to regulate hnRNPR. Our work suggests that JUN-induced seRNA-NPCM interacts with hnRNPR and ACTA1, resulting in the transcription of NDRG1.

The R-loops were enriched at loci with decreased DNA methylation and increased DNase hypersensitivity [29]. In this study, seRNA-NPCM binding regions were hypomethylated and DNase hypersensitive from publicly available MeDIP-seq and DNase-seq data. Functionally, R-loops have central roles in cancer-associated processes, including DNA damage, hyper-recombination, and genome instability. Changes in R-loop frequency, stability, or genomic position are associated with cancerous states, including oncogene activation or tumor-suppressor gene loss [53]. This study showed that R-loops play an important role in NPC oncogene transcription, and seRNA-NPC was found to form R-loops in the SE regions of NDRG1 and TRIB1. NDRG1 is a member of the NDRG protein family and has been previously identified as a protein involved in epithelial cell differentiation [54]. NDRG1 has also been verified to promote tumorigenesis [55], and its overexpression is associated with invasion and poor prognosis [55–57]. Furthermore, NDRG1 is crucial in facilitating tumorigenesis and metastasis and is an independent predictor of poor survival outcomes in patients [58, 59]. Several studies, however, contradict each other: overexpression of NDRG1 in the mouse breast cancer model was shown to inhibit metastasis by regulating WNT pathway signaling [60], while downregulation of NDRG1 in MCF-7 cells can result in increased proliferation and invasiveness [61]. Our results suggested that NDRG1 might play a role as an oncogene or prognostic biomarker in NPC. The reasons for these contradictory results may be different cell origins and pathological classifications, as well as the different molecular mechanisms that activate NDRG1.

In summary, we found a novel seRNA, seRNA-NPCM, which may be a prognostic biomarker and potential therapeutic target for NPC. SeRNA-NPCM promotes chromatin looping by binding to the hnRNPR and ACTA1 protein, and the hnRNPR protein is anchored near the NDRG1 and TRIB1 promoter. In addition, part of seRNA-NPCM hybridizes with the SE region via R-loop, which pulling SE close to the promoter of NDRG1 and TRIB1 to regulate their transcription. The seRNA-NPCM/hnRNPR/ACTA1/NDRG1 axis may be a novel potential target for NPC therapy.

## Materials and methods

### Cell culture

Human NPC cell lines S18, S26, 6–10B, and 5–8F were purchased from the Institute of Biochemistry and Cell Biology of the Chinese Academy of Sciences (Shanghai, China). S26 and S18 cells were isolated from CNE-2 cells. Unlike S26, which has low transferability, S18 has high migration and invasion abilities. The 6–10B and 5–8F cell lines were derived from the CNE1 cell line. The 6–10B cell line has low transferability and 5–8F cells have high migration and invasion abilities. S26 cells were treated with 40 μmol/L DNP for 48 h. The cells were cultured in RPMI 1640 medium supplemented with 10% FBS and 100 U/ml penicillin/streptomycin at 37 °C in a humidified incubator containing 5% CO_2_. All cell lines were authenticated by short tandem repeat (STR) analysis prior to experiments.

### Antibodies

The following antibodies were used for western blotting or chromatin-immunoprecipitation (ChIP). Anti-NDRG1 (ab124689, Abcam), anti-ACTA1 (ab179467, Abcam), anti-c-Jun (ab32137, Abcam), phalloidin-iFluor 488 (ab176753, Abcam), and anti-hnRNPR (ab30930, Abcam) were purchased from Abcam. The anti-hnRNPR antibody (GTX106526, GeneTex) was purchased from GeneTex. Anti-N-cadherin (13116, Cell Signaling Technology), anti-E-cadherin (3195, Cell Signaling Technology), anti-vimentin (5741, Cell Signaling Technology), and anti-Snail (3879, Cell Signaling Technology) antibodies were purchased from Cell Signaling Technology. Anti-GAPDH (60004-1-Ig, Proteintech) and anti-lamin B1 (66095-1-Ig, Proteintech) antibodies, HRP-conjugated goat anti-mouse IgG (SA00001-1, Proteintech), and HRP-conjugated goat anti-rabbit IgG (SA00001-2, Proteintech) were purchased from Proteintech.

### RT-PCR and qRT–PCR

Nuclear and cytoplasmic RNA were extracted with the Cytoplasmic and Nuclear RNA purification Kit (Norgen Biotek Corp., CA, USA) according to the manufacturer’s instructions. Total RNA was extracted using the Total RNA Kit II reagent (Omega). cDNA was reverse transcribed with SuperScript First-Strand Synthesis System Kit (Thermo Fisher Scientific). SYBR Green Master Mix (4309155, Thermo Fisher Scientific) was used to determine mRNA expression. GAPDH was used for normalization. The primers used are listed in Supplementary Data 6. The transcript levels were analyzed using the 2^−ΔΔCt^ method.

### Animal studies

Animal experiments were performed following the guidelines for experimentation with laboratory animals set by The Affiliated Cancer Hospital of Xiangya School of Medicine, Central South University, and approved by the Animal Experimentation Ethics Committee of The Affiliated Cancer Hospital of Xiangya School of Medicine, Central South University. All in vivo experiments were performed on female BALB/c nude mice aged 4–6 weeks. Mice were obtained from Hunan SJA Laboratory Animal Co. (Changsha, China) and housed in a room at a controlled temperature. For the metastasis model, the nude mice were randomly divided into groups (*n* = 6 for each group); 2 × 10^6^ S26 or S18 cells that stably overexpressed or knocked down seRNA-NPCM or NDRG1, a negative control, and an empty lentivirus were suspended in 100 μL PBS, and then injected into the tail veins of mice. After 30 days, metastasis was measured and quantified by ex vivo bioluminescent imaging using an AniView100 Multi-mode In vivo Animal Imaging System (Guangzhou Biolight Biotechnology Co., China). The excised tissues were fixed in 10% neutral buffered formalin or snap-frozen.

### Luciferase reporter assay

Luciferase reporter assay was performed as previously reported [26]. Briefly, the indicated luciferase reporter plasmids and Renilla plasmid were applied to transfect cells. Luciferase activity was measured using the Dual-luciferase Reporter Assay System (Promega, E1910), according to the instructions. The luciferase/Renilla ratio was calculated for all samples.

### Transient transfections

Transient transfection was performed as previously reported [62]. Briefly, shRNAs for seRNA-NPCM, ACTA1, and hnRNPR were designed, synthesized, and cloned into GV102 vectors (Shanghai Genechem Co., China). Transient transfection of cells with shRNA or DNA constructs was performed using Lipofectamine 3000 reagent (Invitrogen) according to the instructions. The sequences of shRNA oligonucleotides are listed in Supplementary Data 6.

### Plasmid construct

The plasmid construct was constructed as previously reported [30]. Briefly, the sequence of seRNA-NPCM was PCR-amplified and cloned into the BamHI and AgeI sites of CV146 (Shanghai Genechem Co., China). NDRG1 was PCR-amplified and cloned into the BamHI and AgeI sites of GV260 (Shanghai Genechem Co., China). JUN was PCR-amplified and cloned into the BamHI and AgeI sites of GV492 (Shanghai Genechem Co., China). Full-length seRNA-NPCM was PCR-amplified and cloned into the Pstl and Kpnl sites of the pSPT18 vector. SeRNA-NPCM TSS regions that encompass the JUN binding site were amplified from S26 genomic DNA and cloned into the KpnI and XhoI sites of GV238 (Shanghai Genechem Co., China). The primers used are listed in Supplementary Data 6.

### Generation of stable cell lines

Stably transfected cells were constructed as previously reported [8]. Briefly, shRNAs for JUN and NDRG1 were designed, synthesized, and cloned into GV493 vectors (Shanghai Genechem Co., China). GV493/ JUN or GV493/ NDRG1 along with the packaging plasmid, were co-transfected into HEK293T cells. After transfection, lentiviruses were collected from the culture supernatants. NPC cells were infected with lentiviruses and selected using puromycin (5 μg/mL). Treated cells were harvested after selection. The shRNA sequences used are listed in Supplementary Data 6.

### Genetic deletion using CRISPR DNA-fragment editing

CRISPR DNA fragment editing was performed as previously reported [63]. Briefly, four small guide RNAs (sgRNAs) were designed to delete an ~3000-bp region around the seRNA-NPCM locus. Lentiviral vectors expressing sgRNAs together with Cas9 mRNA were constructed by inserting annealed oligoDNAs (Supplementary Data 6) into the CV279 plasmid (Shanghai Genechem Co., China). S26 and S18 cells were infected with the lentiviral particles. The infected cells were selected using puromycin. The cells were then diluted, and single-cell CRISPR clones were acquired. Single-cell CRISPR clones were amplified using primers that spanned deleted sequences. The primers used for CRISPR DNA fragment editing are listed in Supplementary Data 6.

### Rapid amplification of cDNA ends assay

Rapid amplification of cDNA ends (RACE) assay was performed as previously reported [9]. To characterize the 5′ and 3′ ends of seRNA, total RNA extracted from S18 was used to generate RACE-ready cDNA using the GeneRacer^TM^ Kit (Invitrogen, L1500-01) according to the manufacturer’s instructions. cDNA ends were amplified using the primer sets indicated in Supplementary Data 6.

### RNA pull-down and western blotting (WB) analysis

RNA pull-down and western blotting were performed as previously reported [30]. Briefly, the linearized DNA constructs were applied to prepare biotinylated RNAs using the RiboMAX™ Large Scale RNA Production System-T7/SP6 in vitro transcription kit (Promega) and RNA 3’ End Desthiobiotinylation kit (Thermo Fisher). 10^7^ of S18 cells were harvested and lysed using IP lysis buffer (Thermo Fisher). Cell lysates were incubated with biotin-labeled RNAs and streptavidin beads. The samples were analyzed using a Q-Exactive LC-MS (Thermo Fisher), then the obtained top-rank proteins were verified by WB analysis.

### RNA fluorescence in situ hybridization (FISH) assay

RNA-FISH assay was performed as previously reported [26]. Briefly, antisense and sense seRNA-NPCM probes labeled with Biotin-TEG at the 5’ end were designed and synthesized by Shanghai Sangon Biotech Co. (Shanghai, China). The cells were seeded on coverslips and fixed with paraformaldehyde. After fixation, cells were permeabilized. The Ribo^TM^ FISH kit (RiboBio, China) was used to perform the FISH assay according to the manufacturer’s protocol. The cells were blocked with pre-hybridization buffer and then hybridized in hybridization buffer containing the probe. After the cells were washed with wash buffer, they were incubated with Cy3-Streptavidin. After washing, the cells were stained with DAPI, and the slides were mounted with an antifade reagent. Images were obtained using a Leica microscope.

### Wound-healing and Transwell assays

Wound-healing and Transwell assays were performed as previously reported [64]. Briefly, the wounds made with pipette tip were photographed under a phase-contrast microscope (Olympus). The percentage of healed area was measured by ImageJ software (NIH Image). Transwell and Transwell invasion assays were performed using chambers, according to the manual (Corning Costar, 3422). The assays were conducted in triplicates in three independent experiments.

### RNA-seq and data analysis

RNA-seq and data analysis were performed as previously reported [26]. Briefly, RNA extracted from the indicated cells was used to sequence the libraries on the Illumina HiSeq 2000 platform. Pair-end raw reads were aligned to the human reference genome (GRch38) using Hisat2 [65] (http://ccb.jhu.edu/software/hisat2/index.shtml). Result signals were visualized using the IGV software. HTseq was used to calculate the number of reads mapped to the genome. The expression data were normalized by reads per kilobase million reads (RPKM). The DESeq2 algorithm was applied to filter the differentially expressed genes. Significance analysis and FDR analysis were performed using the following criteria: log2FC > 1 and FDR < 0.05. Correlation analyses between the eRNA and mRNA transcription were performed using R scripts and Python. The different genes were identified by EdgeR.

### ChIP-seq and ChIP-PCR

ChIP-seq and ChIP-PCR were performed as previously reported [26]. Briefly, the adapters were ligated to the A-tailed chromatin fragments. Fragments of 200–1500 bp in length were selected using AMPure XP beads. Sequencing was performed on Illumina NovaSeq 6000 according to the manufacturer’s guidelines. qPCR was performed using Power SYBR (Applied Biosystems) and primers for the target genomic locus. The ChIP-PCR primers used are listed in Supplementary Data 6. The percentage of the input recovery was then calculated.

### Chromatin isolation by RNA purification

Chromatin isolation by RNA purification sequencing (ChIRP-seq) was performed as previously reported [62]. Briefly, 38 antisense oligonucleotides were designed to target the seRNA-NPCM. Adaptor-ligated fragments of 200–1500 bp in length were selected using AMPure XP beads. Sequencing was performed on an Illumina NovaSeq 6000 according to the manufacturer’s instructions. qPCR was used to check the enrichment of seRNA-NPCM at certain genomic locus. Sequences of all ChIRP probes and ChIRP-PCR primers are listed in Supplementary Data 6.

### Global run-on sequencing (GRO-seq)

GRO-seq experiments were performed as previously reported [66]. Briefly, prepared nuclei were incubated with nuclear run-on buffer (NRO) to extend the nascent RNAs. 5-bromouridine-5′-triphosphate (BrUTP) (Biotium, San Francisco, USA) was added to BrU-tagged nascent RNA. BrU-tagged NRO RNA (∼100 bases) was selected using anti-BrU agarose beads (Santa Cruz Biotechnology, California, USA). The separated RNAs were reverse transcribed to generate cDNAs. The final cDNA libraries were sequenced on Illumina HiSeq 2500.

### DNA-RNA immunoprecipitation (DRIP) assay

DRIP was performed as previously described with modifications [28]. Briefly, the cells were collected and incubated with lysis buffer containing proteinase K. The fragmented DNA was incubated in immunoprecipitation (IP) buffer containing S9.6 antibody (Millipore) to capture the R-loop in vivo. Protein A-agarose beads (Millipore) were used to enrich DNA-RNA hybrids. Purified DNA was used to generate a library using the KAPA HiFi HotStart ReadyMix (Roche). qPCR was used to check the enrichment of S9.6 antibody at certain genomic locus. Sequences of all DRIP-PCR primers are listed in Supplementary Data 6.

### In situ Hi-C followed by chromatin-immunoprecipitation (HiChIP) assay

HiChIP was performed as previously described with modifications [67]. Briefly, the biotinylated DNA fragments were processed using the ChIP protocol. After sonication, the DNA fragments were incubated with H3K27ac antibody (Abcam, 177178). The binding DNA was treated with proteinase K and purified using Epi^TM^ DNA Clean Beads (Epibiotek, R1809). The selected DNA fragments were applied to generate cDNA libraries using the QIAseq Ultralow Input Library Kit (QIAGEN), according to the manufacturer’s protocol. The libraries were quantified and sequenced on an Illumina NovaSeq 6000 platform.

### Native RNA immunoprecipitation (RIP) assay

Native RNA RIP assay was performed as previously described [68]. Briefly, RIP assay was performed using EZ-Magna RIP RNA-Binding Protein Immunoprecipitation Kit (Millipore, 17-701) according to the manufacturer’s protocol. TRIzol reagent was used to extract RNA. cDNAs were prepared using RevertAid First-Strand cDNA Synthesis Kit (Thermo Fisher Scientific, K1622), qPCR was applied to check the enrichment of RNA using GoTaq qPCR Master Mix (Promega, A6002). Isotype IgG and U1 were used as negative and positive controls, respectively. The specific primers are indicated in Supplementary Data 6.

### Immunohistochemistry (IHC)

NPC tissue microarrays were purchased from Shanghai Outdo Biotech Corporation. The NPC and normal nasopharynx tissue slices were gifts from Xiangya School of Medicine. The study was approved by the ethics committee of Xiangya School of Medicine, Central South University. The Informed Consent was obtained from the used patient. The tissue slices were dewaxed and rehydrated. After antigen retrieval, the slides were incubated with the antibodies. Next, the sections were stained with DAB staining solution and counterstained with hematoxylin. Images were captured using a microscope (Olympus, Valley, PA, USA). The tissues were scored by counting the number of positively stained cells.

### RNA in situ hybridization (ISH)

NPC tissue microarrays were purchased from Shanghai Outdo Biotech Corporation. For the in situ detection of seRNA-NPCM in NPC tissue microarray, a seRNA-NPCM probe was designed and produced by the Boster Corporation. Hybridization and signal detection were performed according to the manufacturer’s guidelines. Images were captured using a microscope (Olympus, Valley, PA, USA). The tissues were scored by counting the number of positively stained cells.

### ChIP-seq analysis

The sequencing reads were aligned to the human reference genome (hg38). The aligned bam files were analyzed with hypergeometric optimization of motif enrichment (HOMER) and visualized with the IGV. The peaks were annotated using the hg38 genome in HOMER. In H3K27ac ChIP-seq analysis, potential active enhancers were identified after the removal of any peaks localized to the TSS. The motif analysis was performed in HOMER, and the heat maps were generated using NGSPlot.

### GRO-seq analysis

The sequencing reads were mapped onto the hg38 genome. NRSA (v2, http://bioinfo.vanderbilt.edu/NRSA/) was applied to analyze the nascent transcription [69]. DESeq2 was used to identify the differentially expressed transcripts. The false discovery rate (FDR) was used to calculate the adjusted *P* value. Genes with adjusted *P* value <0.05 were used for subsequent analysis.

### HiChIP data analysis

Each HiChIP replicate was initially processed using the Hi-C-Pro pipeline [70]. Raw fastq (paired-end reads) were aligned to hg38. The Diffloop framework was used to identify differential intrachromosomal chromatin interactions. After mango algorithm correction with an FDR of 0.05, differential loops (S18 vs. S26) were called and annotated to the promoters and enhancers. The promoter-contacting regions were filtered using a minimum loop width of 1.5 kb. Non-prevalent loops were excluded. Loops log fold change (logFC) and *P* value < 0.05 were used to define loops. Juicer Tools were used to generate contact matrices. The visualization of matrices was performed on Juicebox platform. The WashU Epigenome Browser was used for the visualization of loop plots and DNA-binding profiles.

### Data visualization

IGV software was used to visualize ChIP-seq, ChIRP-seq, and DRIP-seq data. Visualization of GRO-seq was performed on the UCSC genome browser. The mappable reads were normalized to reads per million (RPM). All heat maps and read density plots were generated using NGSPlot and R packages.

### Statistical analysis

Statistical analyses were performed using GraphPad Prism version 8 (GraphPad Software, San Diego, CA, USA). Data are presented as mean ± standard deviation (SD). All tests were two-sided, and *P* < 0.05 was considered statistically significant. Asterisks represent statistically significant differences as measured by the *t* test. Spearman’s correlation coefficient was used to analyze the correlation between the two genes. The log-rank test and Cox regression models were used to analyze the differences in survival and hazard ratios.

## Supplementary information

Supplementary Materials

Original Data File

CDDIS-22-4523-checklist

## Data Availability

All data generated or analyzed during this study are included in this article and its supplementary Data.
