# Supplementary figures and images for: JUN-induced super-enhancer RNA forms R-loop to promote nasopharyngeal carcinoma metastasis
Source: Cell Death Dis. 2023 Jul 21;14(7):459. doi: 10.1038/s41419-023-05985-9 (PMC10361959; doi:10.1038/s41419-023-05985-9)

Fig1E

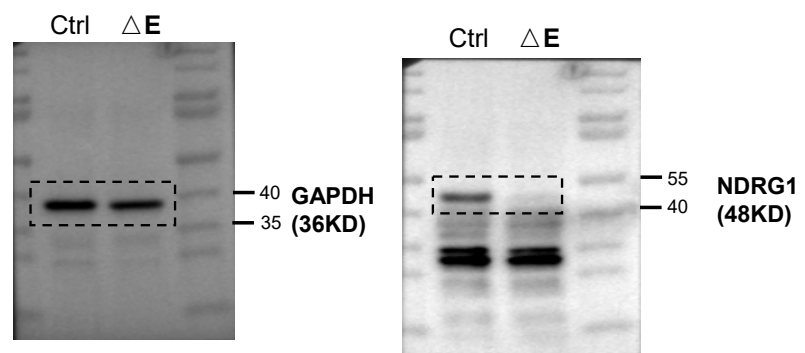

Fig1F

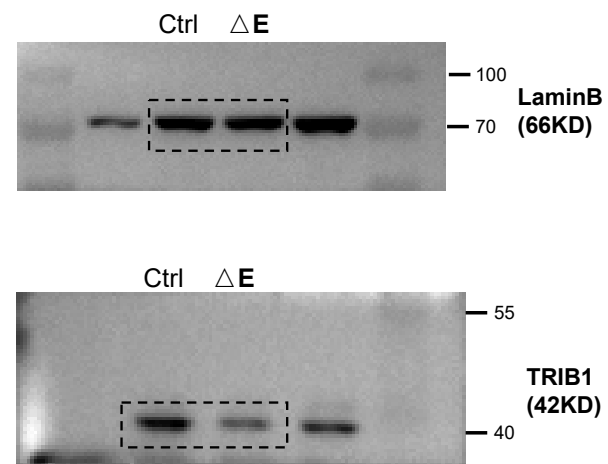

Fig3A

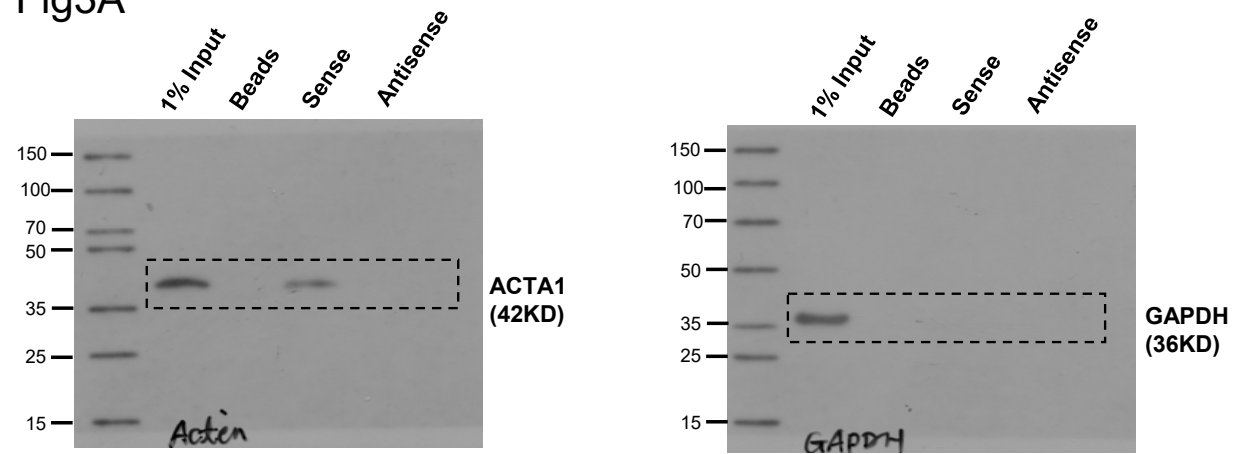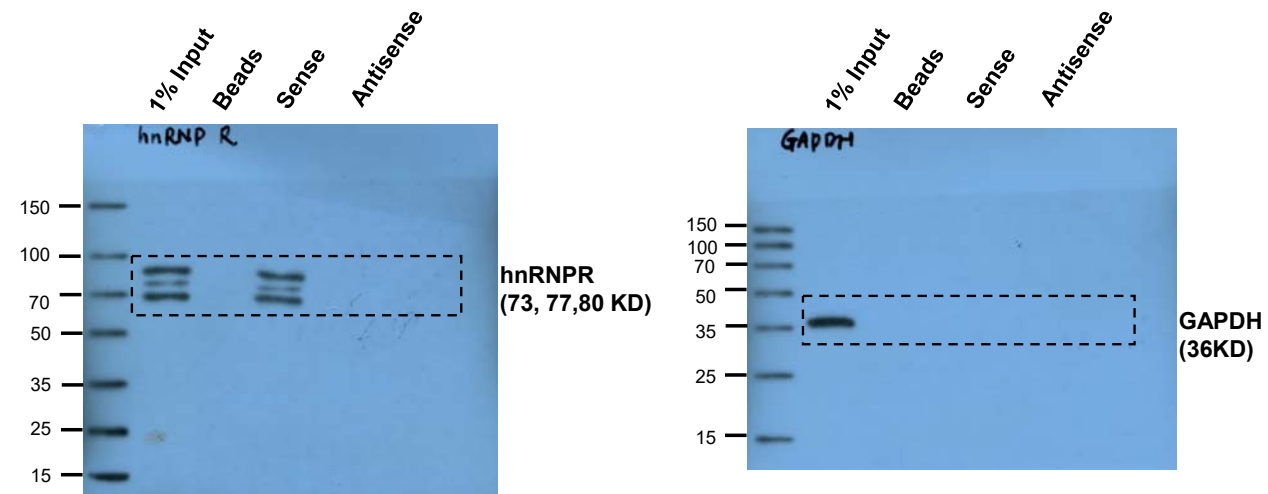

Fig3C

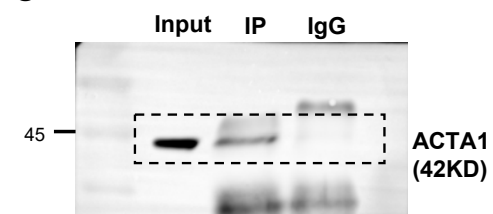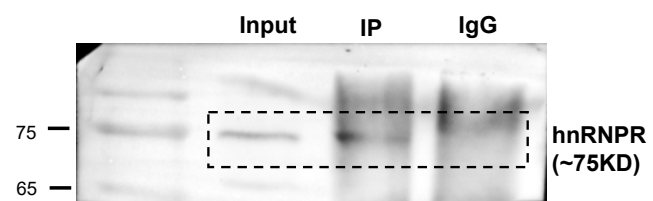

Fig3D

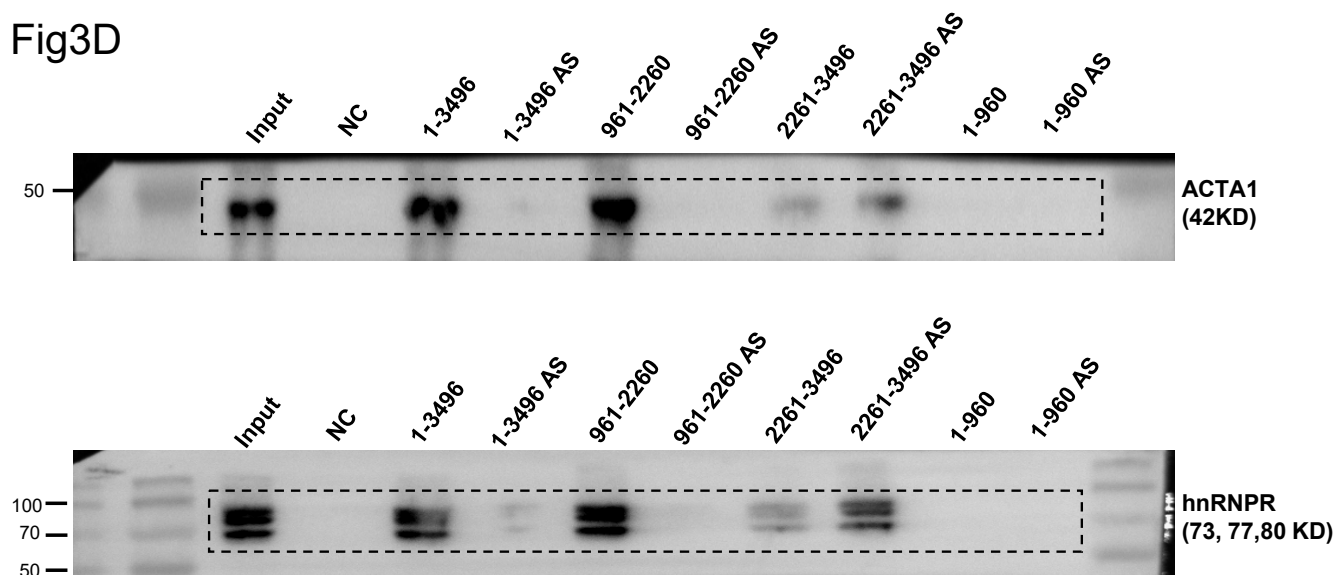

Fig5G

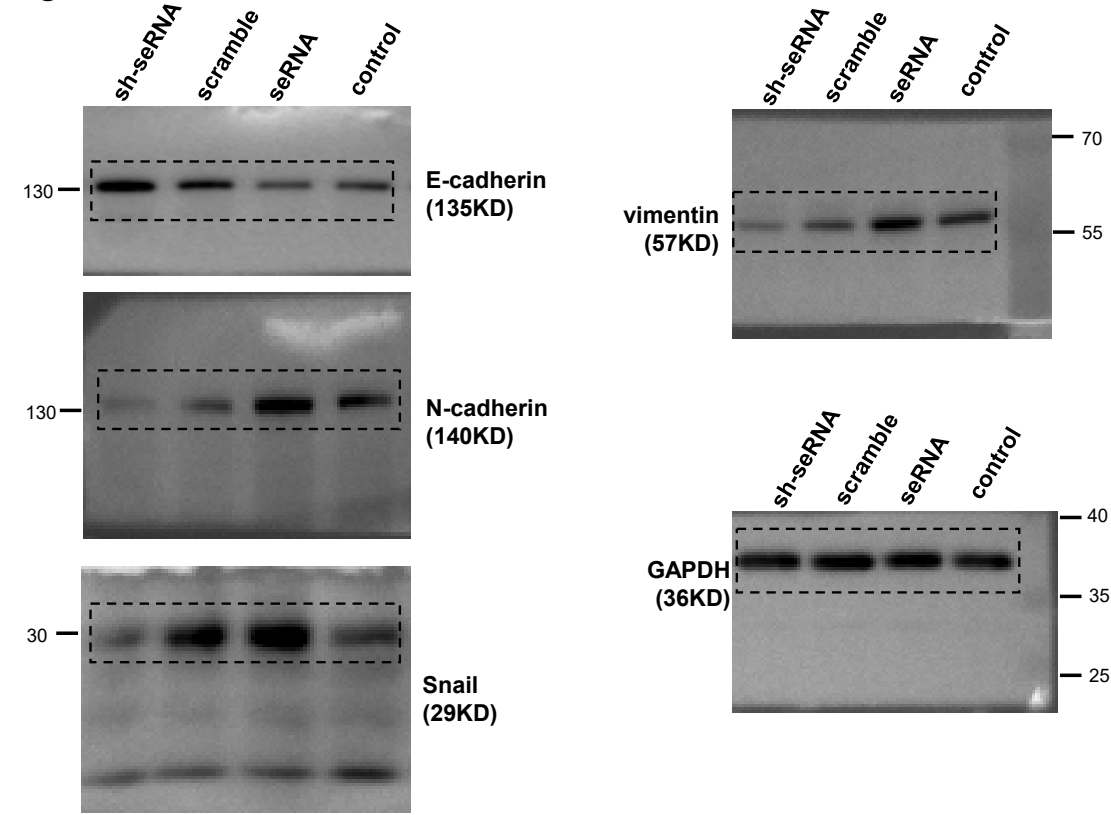

Fig6A

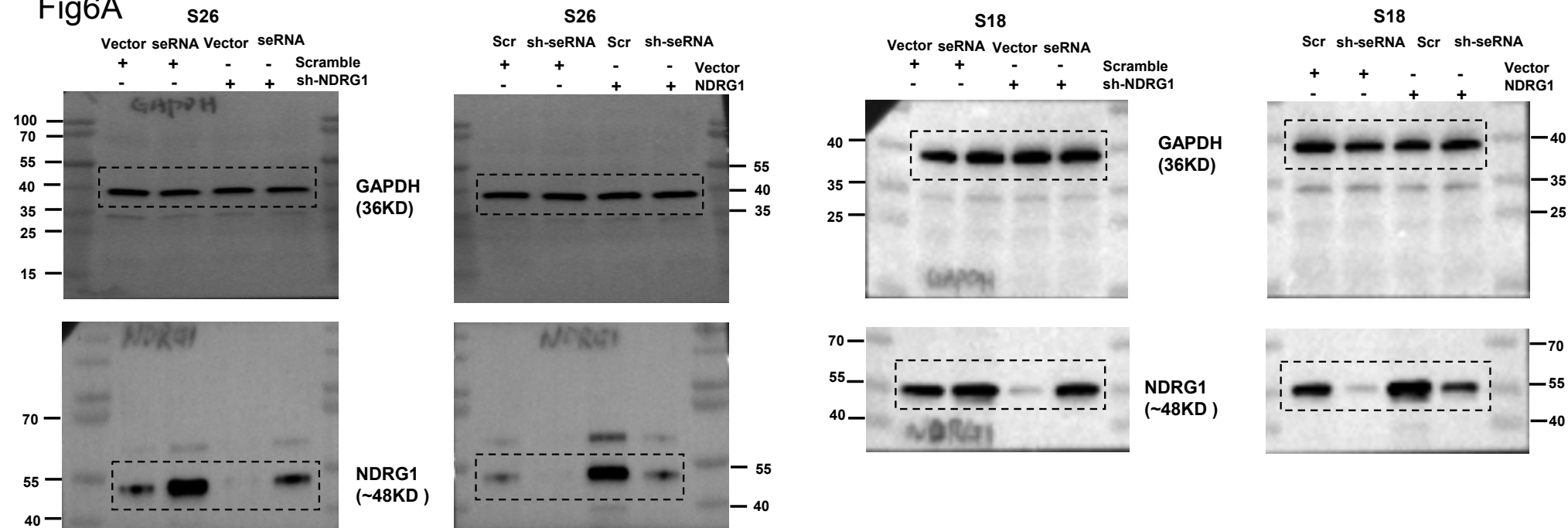

Fig6E

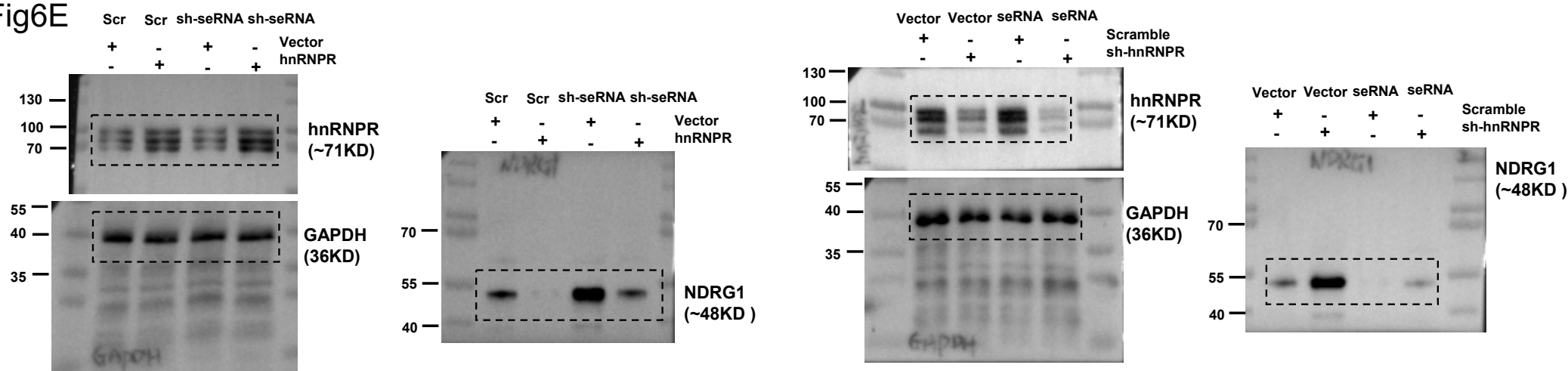

Fig7E

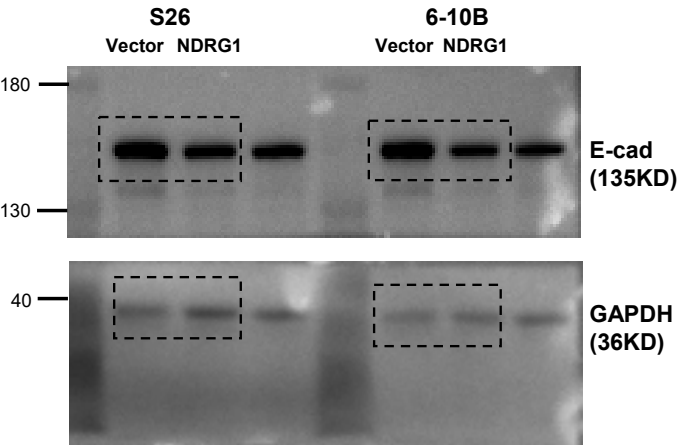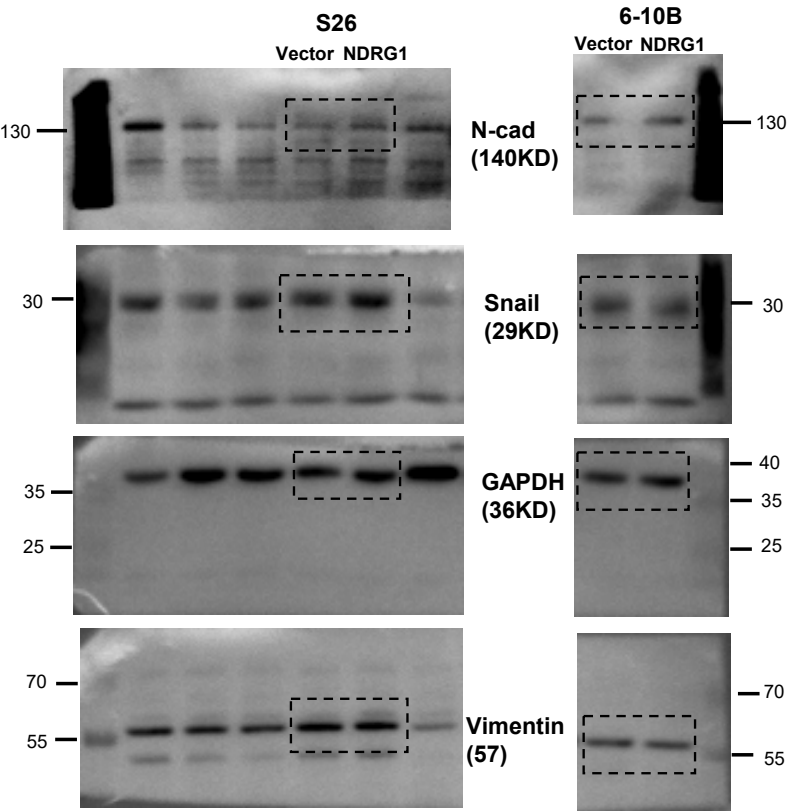

Fig7E

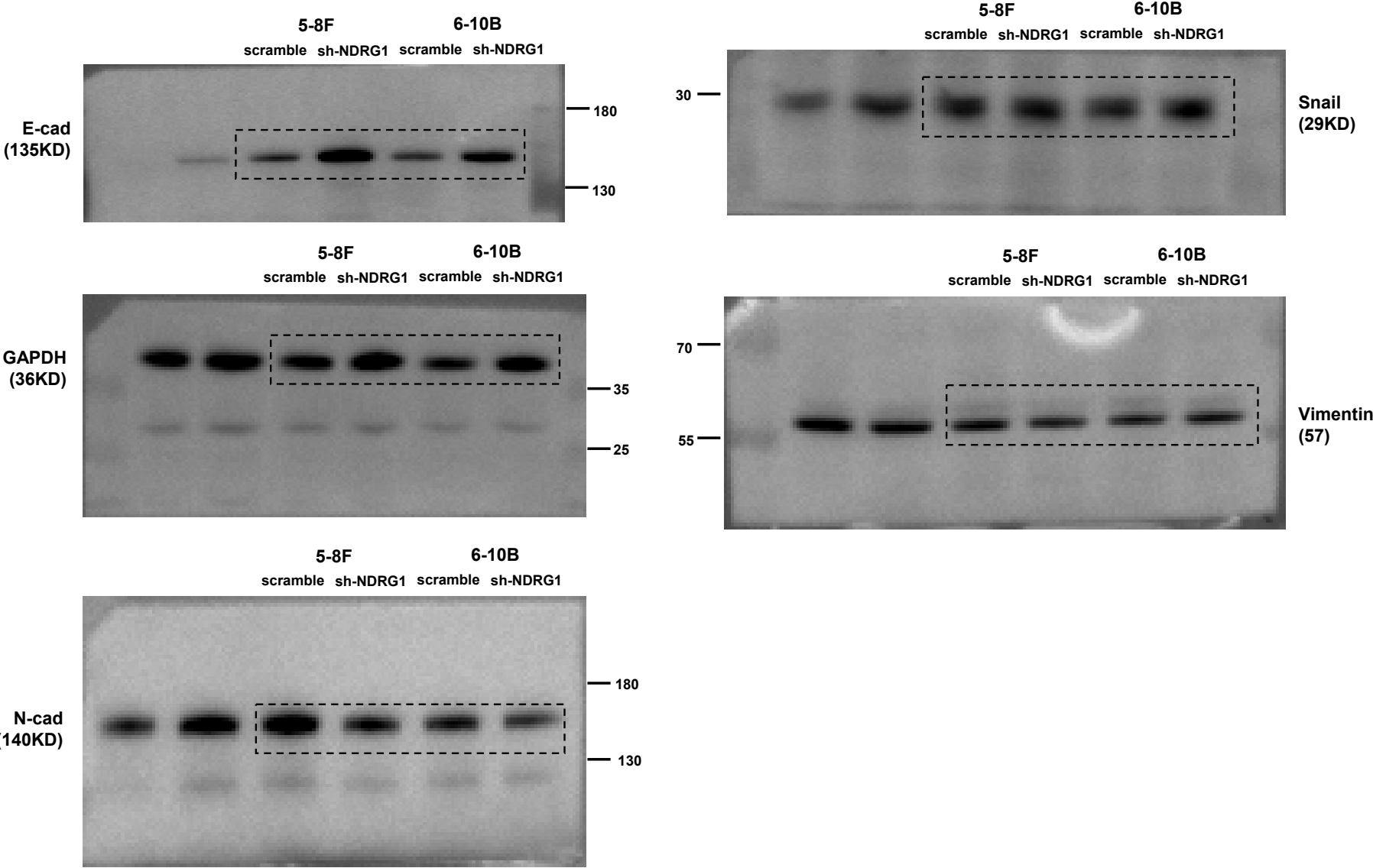

FigS2-E

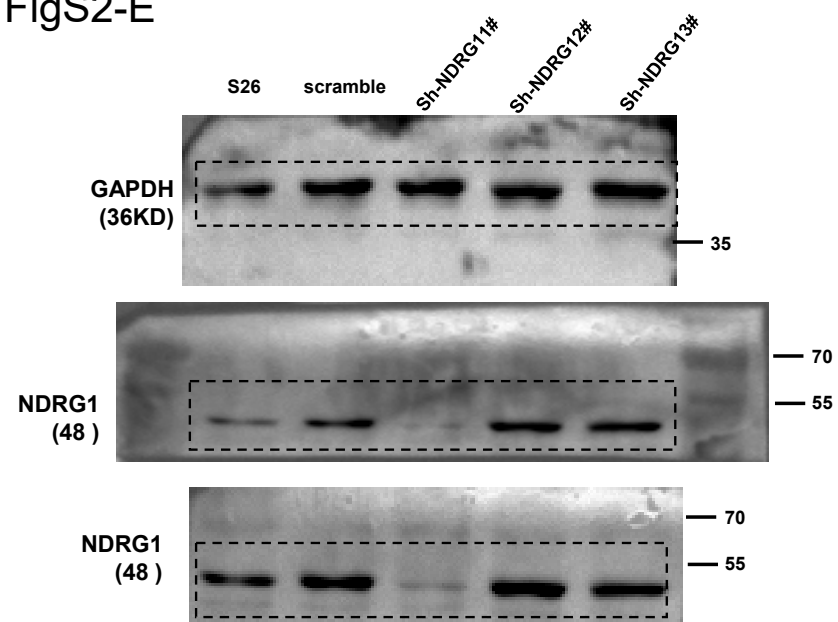

Supplement: Supplementary file 2 — Original Data File [file 41419_2023_5985_MOESM2_ESM.pdf]
