# Supplementary material for: JUN-induced super-enhancer RNA forms R-loop to promote nasopharyngeal carcinoma metastasis
Source: Cell Death Dis. 2023 Jul 21;14(7):459. doi: 10.1038/s41419-023-05985-9 (PMC10361959; doi:10.1038/s41419-023-05985-9)
Supplement: Supplementary file 1 — Supplementary Materials [file 41419_2023_5985_MOESM1_ESM.pdf]

Supplementary figures

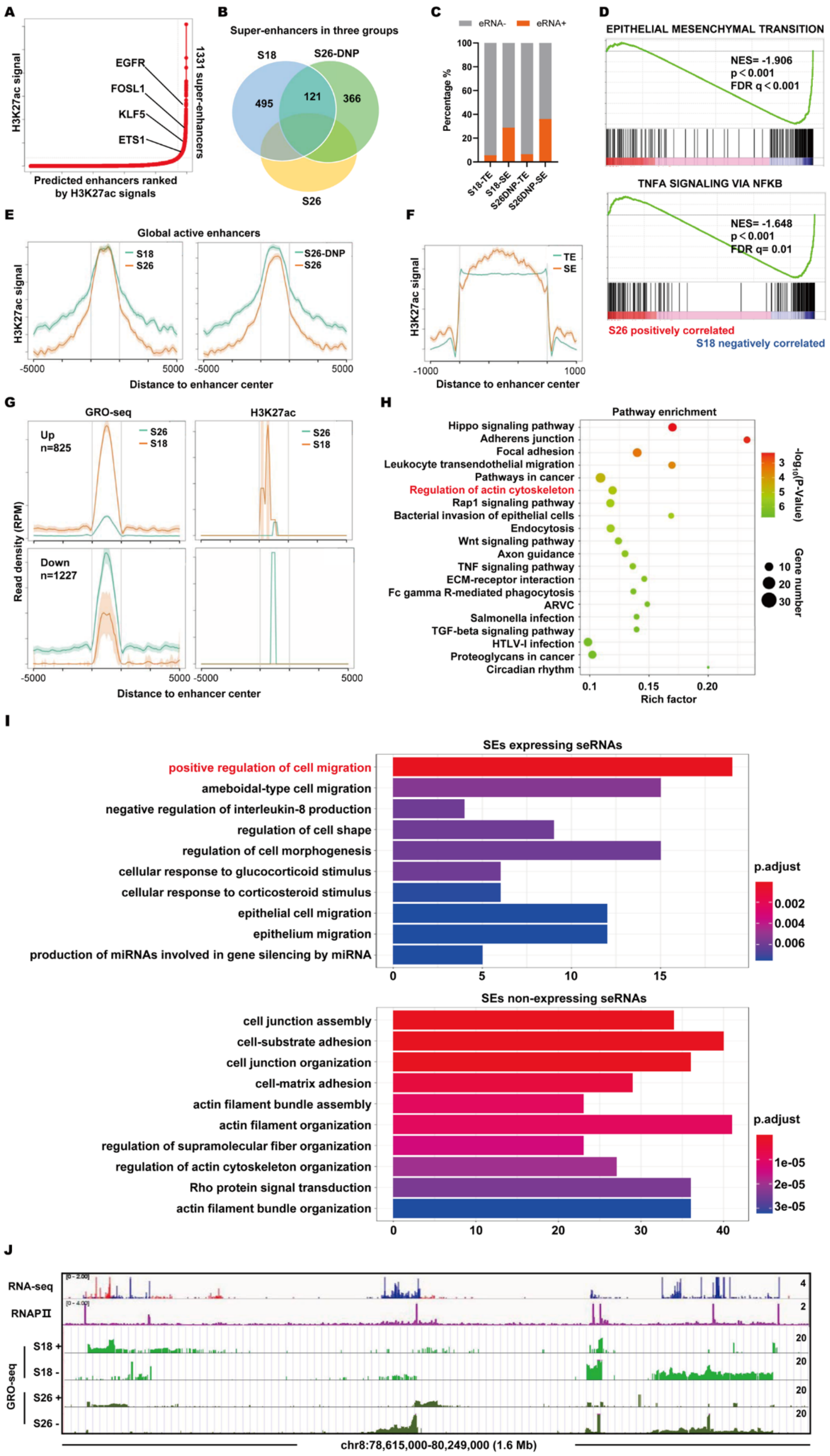

Figure S1. Global enhancement of SE and seRNA activity in NPC metastasis. **A,** Rank order of H3K27ac ChIP-seq signals. SEs are the population on the right side of the dotted line (with slope of more than 1). SEs of S18 were identified and annotated to their nearest genes. Part of proto-oncogenes are indicated. **B,** Venn diagrams of SEs in S18 (blue), S26-DNP (green), and S26 (orange). **C,** Analysis of GRO-seq data on eRNAs ($eRNA^+$) percentages of S18 and S26-DNP SE. **D,** GSEA of GRO-seq data in S18 *vs* S26. Enrichment scores were indicated in each plot along with *P* value and false discovery rate (FDR) q-value. **E,** Density plot of average ChIP-seq signals in 10-kb windows around the center of enhancer for S18 *vs* S26 and S26-DNP *vs* S26 cells. **F,** Comparation of enhancer activities in SEs with TEs. **G,** eRNAs detection of S18 and S26 cells with GRO-seq. **H**, Pathway enrichment of activated SE in S18 cells through KEGG. **I**, Gene ontology analysis of SEs expressing seRNA (top) and not expressing seRNAs (bottom) in S18 cells. **J,** GRO-seq and RNA-seq signals aligned to S18 genome. Red, plus-strand RNA; blue, minus-strand RNA. RNAPII ChIP-seq enrichments were loaded from ENCODE (Hela-S3, GSM822273).

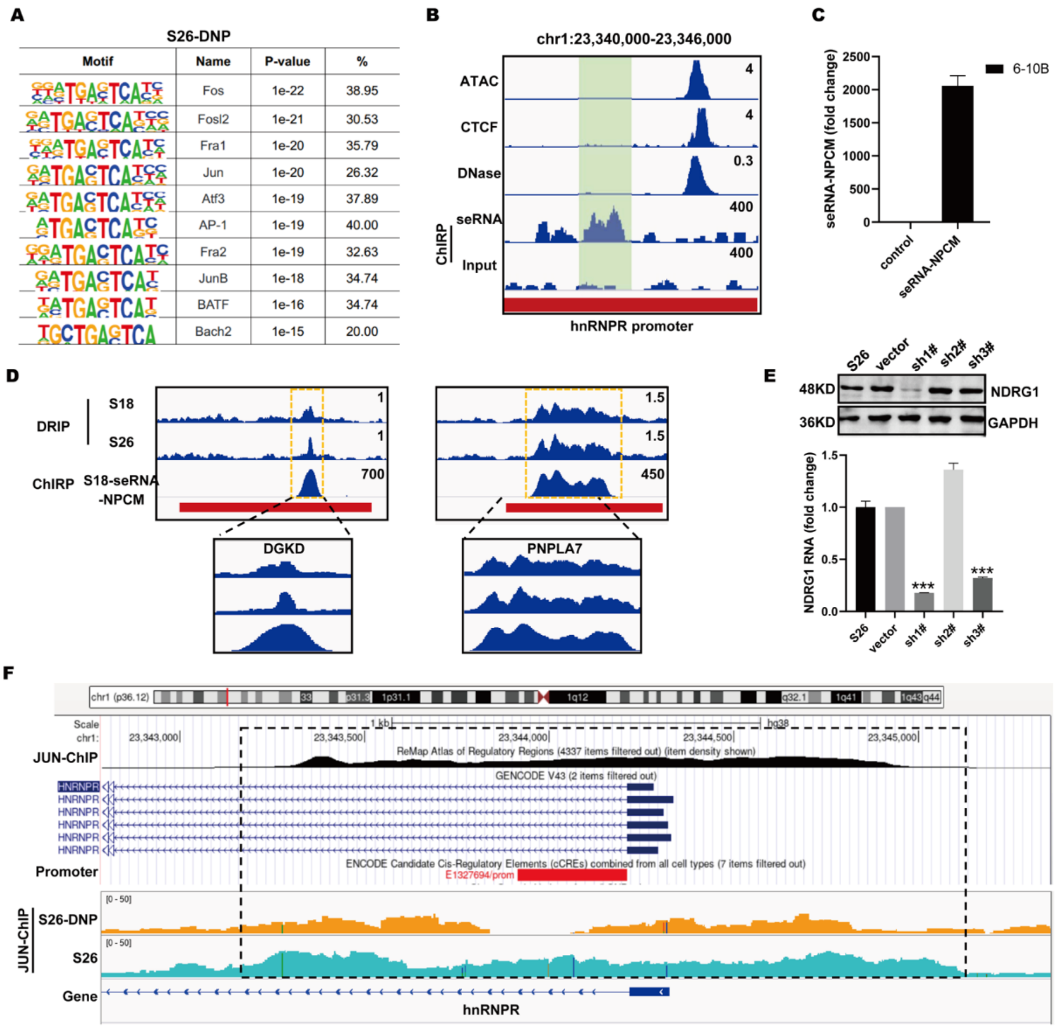

Figure S2. **A**, DNA binding motifs enriched within the ±2 kb window of TSSs of seRNAs in S26-DNP cells. **B**, SeRNA-NPCM enrichment in hnRNPR promotor. Signals of ATAC-seq (HepG2, GSE170012), DNase-seq (HepG2, GSE90300), and CTCF ChIP-seq (HepG2, GSE170879) at hnRNPR promotor. **C**, SeRNA-NPCM overexpression in 6-10B cells. **D**, Signals of ChIRP-seq and DRIP-seq in S18 vs. S26. **E**, Knockdown of NDRG1 in S26 cells. **F**, JUN ChIP signals at the promoter of hnRNPR. Part of the data downloaded from UCSC. Data are expressed as mean ± SEM; *P < 0.05, **P < 0.01, ***P < 0.001.

Supplementary Data 1. Hi-ChIP analysis in S18 and S26-DNP cells

Hi-ChIP analysis in S18 cells

loopChr1	loopMidSite1	loopChr2	loopMidSite2	p-value	loopAnnotType1	loopAnnotType2	targetGene
chr1	715000	chr1	31925000	3.41E-03	promoter	super_enhancer	RP11-206L10.2
chr1	715000	chr1	43475000	3.30E-03	promoter	super_enhancer	RP11-206L10.2
chr1	715000	chr1	224265000	1.45E-03	promoter	super_enhancer	RP11-206L10.2
chr1	895000	chr1	8905000	2.50E-02	promoter	super_enhancer	NOC2L
chr1	895000	chr1	16065000	1.82E-02	promoter	super_enhancer	NOC2L
chr1	895000	chr1	16085000	1.86E-02	promoter	super_enhancer	NOC2L
chr1	895000	chr1	21285000	9.51E-03	promoter	super_enhancer	NOC2L
chr1	895000	chr1	31575000	8.68E-03	promoter	super_enhancer	NOC2L
chr1	945000	chr1	36175000	1.09E-02	promoter	super_enhancer	RP11-54O7.10
chr1	945000	chr1	207005000	3.56E-03	promoter	super_enhancer	RP11-54O7.10
chr1	955000	chr1	16205000	1.43E-02	promoter	super_enhancer	AGRN
chr1	955000	chr1	27515000	9.79E-03	promoter	super_enhancer	AGRN
chr1	955000	chr1	42945000	7.07E-03	promoter	super_enhancer	AGRN
chr1	955000	chr1	202035000	3.72E-03	promoter	super_enhancer	AGRN
chr1	955000	chr1	204075000	2.89E-03	promoter	super_enhancer	AGRN
chr1	955000	chr1	207015000	2.89E-03	promoter	super_enhancer	AGRN
chr1	995000	chr1	6385000	3.28E-02	promoter	super_enhancer	RP11-465B22.3
chr1	995000	chr1	9305000	2.95E-02	promoter	super_enhancer	RP11-465B22.3
chr1	995000	chr1	12145000	1.67E-02	promoter	super_enhancer	RP11-465B22.3
chr1	995000	chr1	16045000	2.02E-02	promoter	super_enhancer	RP11-465B22.3
chr1	995000	chr1	16175000	1.32E-02	promoter	super_enhancer	RP11-465B22.3

chr1	1025000	chr1	1245000	2.39E-02	super_enhancer	promoter	ACAP3
chr1	1025000	chr1	1625000	3.49E-02	super_enhancer	promoter	SLC35E2B
chr1	1025000	chr1	7755000	2.24E-02	super_enhancer	promoter	RP11-338N10.1
chr1	1025000	chr1	151105000	2.10E-03	super_enhancer	promoter	RP11-68I18.10
chr1	1025000	chr1	199845000	2.07E-03	super_enhancer	promoter	RP11-567B20.1
chr1	1065000	chr1	1215000	1.20E-02	super_enhancer	promoter	SCNN1D
chr1	1065000	chr1	1255000	4.48E-02	super_enhancer	promoter	RP5-890O3.9
chr1	1065000	chr1	16565000	1.42E-02	super_enhancer	promoter	RSG1
chr1	1065000	chr1	17515000	1.13E-02	super_enhancer	promoter	RP11-380J14.1
chr1	1065000	chr1	23045000	1.02E-02	super_enhancer	promoter	MIR4684
chr1	1065000	chr1	30885000	7.47E-03	super_enhancer	promoter	RP4-591L5.1
chr1	1075000	chr1	26185000	9.19E-03	super_enhancer	promoter	RP1-125I3.2
chr1	1075000	chr1	95975000	2.44E-03	super_enhancer	promoter	RP11-286B14.1
chr1	1075000	chr1	201435000	2.52E-03	super_enhancer	promoter	RP11-134G8.10
chr1	1075000	chr1	205425000	3.33E-03	super_enhancer	promoter	RP11-576D8.4
chr1	1105000	chr1	6335000	2.72E-02	promoter	super_enhancer	RP11-465B22.8
chr1	1105000	chr1	6355000	3.48E-02	promoter	super_enhancer	RP11-465B22.8
chr1	1105000	chr1	40915000	6.20E-03	promoter	super_enhancer	RP11-465B22.8
chr1	1105000	chr1	151195000	2.93E-03	promoter	super_enhancer	RP11-465B22.8
chr1	1115000	chr1	15945000	1.77E-02	promoter	super_enhancer	TTLL10-AS1
chr1	1115000	chr1	26355000	1.28E-02	promoter	super_enhancer	TTLL10-AS1
chr1	1115000	chr1	43445000	8.32E-03	promoter	super_enhancer	TTLL10-AS1
chr1	1115000	chr1	202055000	3.87E-03	promoter	super_enhancer	TTLL10-AS1
chr1	1115000	chr1	234615000	3.78E-03	promoter	super_enhancer	TTLL10-AS1

chr1	1215000	chr1	23225000	1.03E-02	promoter	super_enhancer	SCNN1D
chr1	1215000	chr1	42925000	6.18E-03	promoter	super_enhancer	SCNN1D
chr1	1245000	chr1	1345000	3.29E-02	promoter	super_enhancer	ACAP3
chr1	1245000	chr1	21605000	1.46E-02	promoter	super_enhancer	ACAP3
chr1	1245000	chr1	25545000	1.15E-02	promoter	super_enhancer	ACAP3
chr1	1245000	chr1	26345000	1.33E-02	promoter	super_enhancer	ACAP3
chr1	1245000	chr1	32995000	9.47E-03	promoter	super_enhancer	ACAP3
chr1	1245000	chr1	109905000	3.03E-03	promoter	super_enhancer	ACAP3
chr1	1255000	chr1	9295000	2.30E-02	promoter	super_enhancer	RP5-890O3.9
chr1	1255000	chr1	9325000	2.65E-02	promoter	super_enhancer	RP5-890O3.9
chr1	1255000	chr1	15955000	1.48E-02	promoter	super_enhancer	RP5-890O3.9
chr1	1255000	chr1	18925000	1.43E-02	promoter	super_enhancer	RP5-890O3.9
chr1	1255000	chr1	26315000	1.04E-02	promoter	super_enhancer	RP5-890O3.9
chr1	1255000	chr1	27495000	1.12E-02	promoter	super_enhancer	RP5-890O3.9
chr1	1255000	chr1	205305000	3.97E-03	promoter	super_enhancer	RP5-890O3.9
chr1	1255000	chr1	225455000	2.11E-03	promoter	super_enhancer	RP5-890O3.9
chr1	1255000	chr1	230135000	2.94E-03	promoter	super_enhancer	RP5-890O3.9
chr1	1275000	chr1	6375000	3.99E-02	promoter	super_enhancer	MIR6808
chr1	1275000	chr1	11785000	2.25E-02	promoter	super_enhancer	MIR6808
chr1	1275000	chr1	16055000	1.93E-02	promoter	super_enhancer	MIR6808
chr1	1275000	chr1	16065000	1.81E-02	promoter	super_enhancer	MIR6808
chr1	1275000	chr1	21265000	1.06E-02	promoter	super_enhancer	MIR6808
chr1	1275000	chr1	25555000	1.06E-02	promoter	super_enhancer	MIR6808
chr1	1275000	chr1	41505000	5.59E-03	promoter	super_enhancer	MIR6808

chr1	1275000	chr1	42975000	6.26E-03	promoter	super_enhancer	MIR6808
chr1	1285000	chr1	6355000	2.39E-02	promoter	super_enhancer	DVL1
chr1	1285000	chr1	9345000	1.46E-02	promoter	super_enhancer	DVL1
chr1	1285000	chr1	25555000	6.39E-03	promoter	super_enhancer	DVL1
chr1	1285000	chr1	43435000	4.39E-03	promoter	super_enhancer	DVL1
chr1	1335000	chr1	1345000	3.30E-02	promoter	super_enhancer	CCNL2
chr1	1335000	chr1	12575000	2.07E-02	promoter	super_enhancer	CCNL2
chr1	1335000	chr1	16195000	1.45E-02	promoter	super_enhancer	CCNL2
chr1	1335000	chr1	43535000	8.04E-03	promoter	super_enhancer	CCNL2
chr1	1335000	chr1	155135000	3.62E-03	promoter	super_enhancer	CCNL2
chr1	1345000	chr1	8485000	1.57E-02	super_enhancer	promoter	RP5-1115A15.1
chr1	1345000	chr1	16245000	1.36E-02	super_enhancer	promoter	RP1-134O19.3
chr1	1345000	chr1	17515000	1.33E-02	super_enhancer	promoter	RP11-380J14.1
chr1	1345000	chr1	17635000	1.48E-02	super_enhancer	promoter	PADI4
chr1	1345000	chr1	32665000	8.70E-03	super_enhancer	promoter	CCDC28B
chr1	1345000	chr1	35735000	6.50E-03	super_enhancer	promoter	ZMYM4
chr1	1345000	chr1	41445000	7.92E-03	super_enhancer	promoter	CTPS1
chr1	1345000	chr1	44115000	5.17E-03	super_enhancer	promoter	KDM4A
chr1	1345000	chr1	172875000	2.50E-03	super_enhancer	promoter	RP3-471M13.2
chr1	1345000	chr1	229645000	2.81E-03	super_enhancer	promoter	NUP133
chr1	1355000	chr1	6345000	2.71E-02	promoter	super_enhancer	RP4-758J18.7
chr1	1355000	chr1	9315000	2.11E-02	promoter	super_enhancer	RP4-758J18.7
chr1	1355000	chr1	15775000	1.68E-02	promoter	super_enhancer	RP4-758J18.7
chr1	1355000	chr1	27565000	9.24E-03	promoter	super_enhancer	RP4-758J18.7

chr1	1355000	chr1	43205000	6.97E-03	promoter	super_enhancer	RP4-758J18.7
chr1	1385000	chr1	6335000	2.84E-02	promoter	super_enhancer	ATAD3C
chr1	1385000	chr1	6375000	3.68E-02	promoter	super_enhancer	ATAD3C
chr1	1385000	chr1	16115000	1.31E-02	promoter	super_enhancer	ATAD3C
chr1	1385000	chr1	42945000	5.99E-03	promoter	super_enhancer	ATAD3C
chr1	1385000	chr1	43475000	6.07E-03	promoter	super_enhancer	ATAD3C
chr1	1515000	chr1	9305000	3.34E-02	promoter	super_enhancer	AL645728.1
chr1	1515000	chr1	11795000	2.46E-02	promoter	super_enhancer	AL645728.1
chr1	1515000	chr1	15425000	1.78E-02	promoter	super_enhancer	AL645728.1
chr1	1515000	chr1	27515000	1.01E-02	promoter	super_enhancer	AL645728.1
chr1	1535000	chr1	27585000	9.12E-03	promoter	super_enhancer	C1orf233
chr1	1535000	chr1	43475000	5.73E-03	promoter	super_enhancer	C1orf233
chr1	1535000	chr1	43545000	5.68E-03	promoter	super_enhancer	C1orf233
chr1	1605000	chr1	12595000	1.33E-02	promoter	super_enhancer	RP11-345P4.6
chr1	1605000	chr1	15775000	1.54E-02	promoter	super_enhancer	RP11-345P4.6
chr1	1605000	chr1	18915000	1.08E-02	promoter	super_enhancer	RP11-345P4.6
chr1	1605000	chr1	21935000	8.76E-03	promoter	super_enhancer	RP11-345P4.6
chr1	1605000	chr1	23205000	1.18E-02	promoter	super_enhancer	RP11-345P4.6
chr1	1605000	chr1	25555000	8.34E-03	promoter	super_enhancer	RP11-345P4.6
chr1	1605000	chr1	150605000	1.91E-03	promoter	super_enhancer	RP11-345P4.6
chr1	1605000	chr1	228145000	2.95E-03	promoter	super_enhancer	RP11-345P4.6
chr1	1625000	chr1	6365000	2.05E-02	promoter	super_enhancer	SLC35E2B
chr1	1625000	chr1	6375000	2.76E-02	promoter	super_enhancer	SLC35E2B
chr1	1625000	chr1	8875000	1.50E-02	promoter	super_enhancer	SLC35E2B

chr1	1625000	chr1	11795000	1.45E-02	promoter	super_enhancer	SLC35E2B
chr1	1625000	chr1	18915000	8.86E-03	promoter	super_enhancer	SLC35E2B
chr1	1625000	chr1	235065000	1.92E-03	promoter	super_enhancer	SLC35E2B
chr1	1655000	chr1	9315000	1.64E-02	promoter	super_enhancer	CDK11B
chr1	1655000	chr1	9335000	1.83E-02	promoter	super_enhancer	CDK11B
chr1	1655000	chr1	15955000	9.65E-03	promoter	super_enhancer	CDK11B
chr1	1655000	chr1	23195000	7.40E-03	promoter	super_enhancer	CDK11B
chr1	1655000	chr1	31695000	6.86E-03	promoter	super_enhancer	CDK11B
chr1	1655000	chr1	32755000	6.28E-03	promoter	super_enhancer	CDK11B
chr1	1845000	chr1	9295000	2.58E-02	promoter	super_enhancer	CALML6
chr1	1845000	chr1	16135000	1.84E-02	promoter	super_enhancer	CALML6
chr1	1845000	chr1	23195000	1.23E-02	promoter	super_enhancer	CALML6
chr1	1845000	chr1	202585000	3.17E-03	promoter	super_enhancer	CALML6
chr1	1845000	chr1	223715000	3.41E-03	promoter	super_enhancer	CALML6
chr1	1935000	chr1	7535000	2.88E-02	promoter	super_enhancer	CFAP74
chr1	1935000	chr1	9305000	3.71E-02	promoter	super_enhancer	CFAP74
chr1	1935000	chr1	23215000	1.77E-02	promoter	super_enhancer	CFAP74
chr1	1935000	chr1	25555000	1.25E-02	promoter	super_enhancer	CFAP74
chr1	1935000	chr1	203515000	3.80E-03	promoter	super_enhancer	CFAP74
chr1	2145000	chr1	9335000	3.35E-02	promoter	super_enhancer	FAAP20
chr1	2145000	chr1	12165000	2.61E-02	promoter	super_enhancer	FAAP20
chr1	2145000	chr1	15325000	1.97E-02	promoter	super_enhancer	FAAP20
chr1	2145000	chr1	15435000	2.22E-02	promoter	super_enhancer	FAAP20
chr1	2145000	chr1	16135000	1.96E-02	promoter	super_enhancer	FAAP20

chr1	2145000	chr1	16155000	2.09E-04	promoter	super_enhancer	FAAP20
chr1	2145000	chr1	40995000	7.90E-03	promoter	super_enhancer	FAAP20
chr1	2145000	chr1	43535000	8.83E-03	promoter	super_enhancer	FAAP20
chr1	2145000	chr1	230125000	2.83E-03	promoter	super_enhancer	FAAP20
chr1	2155000	chr1	7545000	4.21E-02	promoter	super_enhancer	AL590822.1
chr1	2155000	chr1	9325000	2.82E-02	promoter	super_enhancer	AL590822.1
chr1	2155000	chr1	12135000	2.45E-02	promoter	super_enhancer	AL590822.1
chr1	2155000	chr1	31685000	1.07E-02	promoter	super_enhancer	AL590822.1
chr1	2155000	chr1	230155000	2.84E-03	promoter	super_enhancer	AL590822.1
chr1	2285000	chr1	6345000	3.72E-02	promoter	super_enhancer	RP4-740C4.5
chr1	2285000	chr1	7555000	2.88E-02	promoter	super_enhancer	RP4-740C4.5
chr1	2285000	chr1	10905000	2.24E-02	promoter	super_enhancer	RP4-740C4.5
chr1	2285000	chr1	15945000	1.48E-02	promoter	super_enhancer	RP4-740C4.5
chr1	2285000	chr1	43545000	6.35E-03	promoter	super_enhancer	RP4-740C4.5
chr1	2285000	chr1	203525000	3.05E-03	promoter	super_enhancer	RP4-740C4.5
chr1	2295000	chr1	12545000	1.55E-02	promoter	super_enhancer	RP4-740C4.7
chr1	2295000	chr1	16195000	1.33E-02	promoter	super_enhancer	RP4-740C4.7
chr1	2295000	chr1	204435000	2.93E-03	promoter	super_enhancer	RP4-740C4.7
chr1	2345000	chr1	6365000	3.95E-02	promoter	super_enhancer	PEX10
chr1	2345000	chr1	8875000	2.63E-02	promoter	super_enhancer	PEX10
chr1	2345000	chr1	8905000	2.95E-02	promoter	super_enhancer	PEX10
chr1	2345000	chr1	9315000	2.79E-02	promoter	super_enhancer	PEX10
chr1	2345000	chr1	9325000	3.00E-02	promoter	super_enhancer	PEX10
chr1	2345000	chr1	12175000	2.18E-02	promoter	super_enhancer	PEX10

chr1	2345000	chr1	21265000	1.12E-02	promoter	super_enhancer	PEX10
chr1	2345000	chr1	31685000	1.12E-02	promoter	super_enhancer	PEX10
chr1	2345000	chr1	32715000	9.30E-03	promoter	super_enhancer	PEX10
chr1	2345000	chr1	235075000	3.72E-03	promoter	super_enhancer	PEX10
chr1	2425000	chr1	3545000	8.01E-03	promoter	super_enhancer	RP3-395M20.2
chr1	2425000	chr1	6365000	8.10E-04	promoter	super_enhancer	RP3-395M20.2
chr1	2425000	chr1	7555000	3.19E-02	promoter	super_enhancer	RP3-395M20.2
chr1	2425000	chr1	9305000	3.43E-02	promoter	super_enhancer	RP3-395M20.2
chr1	2425000	chr1	21315000	1.24E-02	promoter	super_enhancer	RP3-395M20.2
chr1	2425000	chr1	27525000	1.10E-02	promoter	super_enhancer	RP3-395M20.2
chr1	2425000	chr1	234725000	2.87E-03	promoter	super_enhancer	RP3-395M20.2
chr1	2525000	chr1	18435000	2.09E-02	super_enhancer	promoter	RP11-174G17.3
chr1	2525000	chr1	27995000	1.64E-02	super_enhancer	promoter	RP11-288L9.4
chr1	2525000	chr1	33815000	8.88E-03	super_enhancer	promoter	RP11-415J8.5
chr1	2525000	chr1	116015000	3.64E-03	super_enhancer	promoter	RP4-663N10.2
chr1	2525000	chr1	145515000	3.03E-03	super_enhancer	promoter	PEX11B
chr1	2525000	chr1	159335000	2.64E-03	super_enhancer	promoter	OR10J8P
chr1	2525000	chr1	167165000	2.94E-03	super_enhancer	promoter	LINC01363
chr1	2535000	chr1	37375000	5.61E-03	super_enhancer	promoter	AC117945.1
chr1	2535000	chr1	52645000	3.47E-03	super_enhancer	promoter	PDCL3P6
chr1	2535000	chr1	59485000	3.72E-03	super_enhancer	promoter	LINC01358
chr1	2535000	chr1	179925000	2.31E-03	super_enhancer	promoter	RP11-533E19.5
chr1	2545000	chr1	2565000	3.83E-03	super_enhancer	promoter	MMEL1
chr1	2545000	chr1	7835000	2.83E-02	super_enhancer	promoter	RP3-467L1.6

chr1	2545000	chr1	10115000	2.12E-02	super_enhancer	promoter	RP11-496H15.2
chr1	2545000	chr1	17575000	1.37E-02	super_enhancer	promoter	PADI3
chr1	2545000	chr1	21835000	1.05E-02	super_enhancer	promoter	ALPL
chr1	2545000	chr1	53685000	4.75E-03	super_enhancer	promoter	RP5-1024G6.7
chr1	2545000	chr1	77245000	3.82E-06	super_enhancer	promoter	RP11-415A20.1
chr1	2545000	chr1	85725000	2.91E-03	super_enhancer	promoter	C1orf52
chr1	2545000	chr1	168345000	2.70E-03	super_enhancer	promoter	MIR557
chr1	2565000	chr1	12165000	2.33E-02	promoter	super_enhancer	MMEL1
chr1	2565000	chr1	16085000	1.93E-02	promoter	super_enhancer	MMEL1
chr1	2565000	chr1	26275000	1.15E-02	promoter	super_enhancer	MMEL1
chr1	2565000	chr1	40885000	4.90E-03	promoter	super_enhancer	MMEL1
chr1	2565000	chr1	43235000	6.53E-03	promoter	super_enhancer	MMEL1
chr1	2565000	chr1	67725000	3.24E-03	promoter	super_enhancer	MMEL1
chr1	2565000	chr1	202055000	3.23E-03	promoter	super_enhancer	MMEL1
chr1	2985000	chr1	11795000	2.48E-02	promoter	super_enhancer	LINC00982
chr1	2985000	chr1	205325000	3.71E-03	promoter	super_enhancer	LINC00982
chr1	2985000	chr1	211635000	2.24E-03	promoter	super_enhancer	LINC00982
chr1	3465000	chr1	5955000	1.32E-04	super_enhancer	promoter	AL356261.1
chr1	3465000	chr1	20835000	1.40E-02	super_enhancer	promoter	MUL1
chr1	3465000	chr1	28655000	9.66E-03	super_enhancer	promoter	MED18
chr1	3465000	chr1	40255000	6.76E-03	super_enhancer	promoter	BMP8B
chr1	3465000	chr1	40505000	8.12E-03	super_enhancer	promoter	CAP1
chr1	3465000	chr1	203275000	3.62E-03	super_enhancer	promoter	LINC01136
chr1	3465000	chr1	204135000	3.35E-03	super_enhancer	promoter	REN

chr1	3465000	chr1	209605000	3.27E-03	super_enhancer	promoter	MIR205
chr1	3545000	chr1	3585000	3.40E-02	super_enhancer	promoter	RP5-1092A11.5
chr1	3545000	chr1	8485000	3.01E-02	super_enhancer	promoter	RP5-1115A15.1
chr1	3545000	chr1	16345000	2.43E-02	super_enhancer	promoter	CLCNKA
chr1	3545000	chr1	32645000	1.12E-02	super_enhancer	promoter	TXLNA
chr1	3545000	chr1	47645000	8.41E-03	super_enhancer	promoter	CYP4A22-AS1
chr1	3545000	chr1	51795000	7.23E-03	super_enhancer	promoter	TTC39A-AS1
chr1	3545000	chr1	52175000	5.44E-03	super_enhancer	promoter	GAPDHP51
chr1	3545000	chr1	52325000	6.80E-03	super_enhancer	promoter	TSEN15P2
chr1	3545000	chr1	68945000	3.39E-03	super_enhancer	promoter	RP4-694A7.2
chr1	3585000	chr1	11805000	2.29E-02	promoter	super_enhancer	RP5-1092A11.5
chr1	3585000	chr1	12555000	2.37E-04	promoter	super_enhancer	RP5-1092A11.5
chr1	3585000	chr1	12595000	1.90E-02	promoter	super_enhancer	RP5-1092A11.5
chr1	3585000	chr1	15755000	1.92E-02	promoter	super_enhancer	RP5-1092A11.5
chr1	3585000	chr1	15805000	1.46E-02	promoter	super_enhancer	RP5-1092A11.5
chr1	3585000	chr1	16045000	2.25E-02	promoter	super_enhancer	RP5-1092A11.5
chr1	3585000	chr1	16115000	1.60E-02	promoter	super_enhancer	RP5-1092A11.5
chr1	3585000	chr1	26355000	1.18E-02	promoter	super_enhancer	RP5-1092A11.5
chr1	3585000	chr1	85315000	3.23E-03	promoter	super_enhancer	RP5-1092A11.5
chr1	3775000	chr1	31685000	1.09E-02	promoter	super_enhancer	CEP104
chr1	3775000	chr1	33045000	8.09E-03	promoter	super_enhancer	CEP104
chr1	3775000	chr1	36375000	7.98E-03	promoter	super_enhancer	CEP104
chr1	3775000	chr1	235055000	2.87E-03	promoter	super_enhancer	CEP104
chr1	3775000	chr1	235075000	3.46E-03	promoter	super_enhancer	CEP104

chr1	4715000	chr1	6335000	1.53E-02	promoter	super_enhancer	AJAP1
chr1	4715000	chr1	9305000	1.85E-03	promoter	super_enhancer	AJAP1
chr1	4715000	chr1	11795000	3.42E-02	promoter	super_enhancer	AJAP1
chr1	5955000	chr1	11795000	3.48E-02	promoter	super_enhancer	AL356261.1
chr1	5955000	chr1	12545000	2.31E-02	promoter	super_enhancer	AL356261.1
chr1	5955000	chr1	225465000	2.64E-03	promoter	super_enhancer	AL356261.1
chr1	6265000	chr1	12575000	2.70E-02	promoter	super_enhancer	RNF207
chr1	6265000	chr1	16045000	2.30E-02	promoter	super_enhancer	RNF207
chr1	6265000	chr1	16085000	2.10E-02	promoter	super_enhancer	RNF207
chr1	6265000	chr1	16115000	1.64E-02	promoter	super_enhancer	RNF207
chr1	6265000	chr1	21935000	1.23E-02	promoter	super_enhancer	RNF207
chr1	6265000	chr1	224255000	2.75E-03	promoter	super_enhancer	RNF207
chr1	6295000	chr1	6495000	2.62E-02	promoter	super_enhancer	LINC00337
chr1	6295000	chr1	15765000	3.31E-04	promoter	super_enhancer	LINC00337
chr1	6295000	chr1	16075000	2.63E-02	promoter	super_enhancer	LINC00337
chr1	6295000	chr1	32995000	1.08E-02	promoter	super_enhancer	LINC00337
chr1	6295000	chr1	33095000	1.08E-02	promoter	super_enhancer	LINC00337
chr1	6295000	chr1	36175000	1.17E-02	promoter	super_enhancer	LINC00337
chr1	6335000	chr1	15735000	1.93E-02	super_enhancer	promoter	EFHD2
chr1	6335000	chr1	20835000	1.34E-02	super_enhancer	promoter	MUL1
chr1	6335000	chr1	38555000	6.76E-03	super_enhancer	promoter	MIR3659
chr1	6335000	chr1	119545000	2.39E-03	super_enhancer	promoter	RP4-712E4.1
chr1	6335000	chr1	178695000	2.15E-03	super_enhancer	promoter	RP11-428K3.1
chr1	6335000	chr1	183235000	2.80E-03	super_enhancer	promoter	RP11-548N1.1

chr1	6345000	chr1	16765000	1.76E-02	super_enhancer	promoter	SPATA21
chr1	6345000	chr1	18435000	1.87E-02	super_enhancer	promoter	RP11-174G17.3
chr1	6345000	chr1	28655000	9.10E-03	super_enhancer	promoter	MED18
chr1	6345000	chr1	35315000	7.89E-03	super_enhancer	promoter	RP5-997D16.2
chr1	6345000	chr1	37795000	6.20E-03	super_enhancer	promoter	RPS29P6
chr1	6345000	chr1	43355000	6.69E-03	super_enhancer	promoter	RP11-342M1.7
chr1	6345000	chr1	44585000	6.62E-03	super_enhancer	promoter	KLF17
chr1	6345000	chr1	165645000	2.66E-03	super_enhancer	promoter	RP11-466F5.4
chr1	6355000	chr1	6615000	1.97E-02	super_enhancer	promoter	NOL9
chr1	6355000	chr1	15735000	2.49E-02	super_enhancer	promoter	EFHD2
chr1	6355000	chr1	15935000	2.41E-02	super_enhancer	promoter	RNU7-179P
chr1	6355000	chr1	16295000	2.05E-02	super_enhancer	promoter	RP11-5P18.1
chr1	6355000	chr1	27975000	1.05E-02	super_enhancer	promoter	RP1-159A19.3
chr1	6355000	chr1	42225000	7.62E-03	super_enhancer	promoter	AL451006.1
chr1	6355000	chr1	150265000	3.51E-03	super_enhancer	promoter	MRPS21
chr1	6355000	chr1	215115000	2.84E-03	super_enhancer	promoter	RP11-323K10.1
chr1	6365000	chr1	9885000	4.44E-02	super_enhancer	promoter	CLSTN1
chr1	6365000	chr1	11905000	3.18E-02	super_enhancer	promoter	NPPA-AS1_2
chr1	6365000	chr1	29475000	9.75E-03	super_enhancer	promoter	RP11-242O24.3
chr1	6365000	chr1	36025000	3.79E-05	super_enhancer	promoter	KIAA0319L
chr1	6365000	chr1	45205000	5.31E-03	super_enhancer	promoter	KIF2C
chr1	6365000	chr1	52445000	5.08E-03	super_enhancer	promoter	RP11-91A18.1
chr1	6365000	chr1	149225000	2.49E-03	super_enhancer	promoter	RNVU1-18
chr1	6375000	chr1	110655000	3.75E-03	super_enhancer	promoter	UBL4B

chr1	6375000	chr1	117755000	3.38E-03	super_enhancer	promoter	VTCN1
chr1	6375000	chr1	185265000	2.07E-03	super_enhancer	promoter	RP4-635A23.6
chr1	6385000	chr1	19225000	1.82E-02	super_enhancer	promoter	MIR1290
chr1	6385000	chr1	21115000	1.32E-02	super_enhancer	promoter	HP1BP3
chr1	6385000	chr1	32255000	9.10E-03	super_enhancer	promoter	RP11-84A19.3
chr1	6385000	chr1	44115000	6.13E-03	super_enhancer	promoter	KDM4A
chr1	6385000	chr1	45205000	6.20E-03	super_enhancer	promoter	KIF2C
chr1	6385000	chr1	47435000	6.81E-03	super_enhancer	promoter	CYP4A26P
chr1	6385000	chr1	48695000	6.33E-03	super_enhancer	promoter	RP5-1024N4.4
chr1	6385000	chr1	58525000	4.06E-03	super_enhancer	promoter	RP11-393I23.4
chr1	6385000	chr1	219935000	2.05E-03	super_enhancer	promoter	RNA5SP76
chr1	6455000	chr1	15415000	2.02E-02	promoter	super_enhancer	ACOT7
chr1	6455000	chr1	15785000	1.94E-02	promoter	super_enhancer	ACOT7
chr1	6455000	chr1	15805000	1.61E-02	promoter	super_enhancer	ACOT7
chr1	6455000	chr1	41495000	9.01E-03	promoter	super_enhancer	ACOT7
chr1	6455000	chr1	55425000	4.31E-03	promoter	super_enhancer	ACOT7
chr1	6455000	chr1	109905000	2.70E-03	promoter	super_enhancer	ACOT7
chr1	6485000	chr1	12485000	3.56E-02	promoter	super_enhancer	HES2
chr1	6485000	chr1	15765000	3.11E-02	promoter	super_enhancer	HES2
chr1	6485000	chr1	18935000	1.63E-02	promoter	super_enhancer	HES2
chr1	6485000	chr1	31675000	1.39E-02	promoter	super_enhancer	HES2
chr1	6485000	chr1	31935000	9.64E-03	promoter	super_enhancer	HES2
chr1	6485000	chr1	36165000	1.20E-02	promoter	super_enhancer	HES2
chr1	6485000	chr1	153605000	4.02E-03	promoter	super_enhancer	HES2

chr1	6485000	chr1	181105000	4.09E-03	promoter	super_enhancer	HES2
chr1	6485000	chr1	206905000	4.47E-03	promoter	super_enhancer	HES2
chr1	6495000	chr1	11285000	4.42E-02	super_enhancer	promoter	RNU6-291P
chr1	6495000	chr1	16315000	3.07E-02	super_enhancer	promoter	RP11-5P18.3
chr1	6495000	chr1	22395000	2.11E-02	super_enhancer	promoter	RP1-224A6.8
chr1	6495000	chr1	26535000	1.63E-02	super_enhancer	promoter	RP11-231P20.2
chr1	6495000	chr1	28855000	1.67E-02	super_enhancer	promoter	PRDX3P2
chr1	6495000	chr1	43735000	1.16E-02	super_enhancer	promoter	TMEM125
chr1	6495000	chr1	63545000	5.57E-03	super_enhancer	promoter	RP11-5P4.3
chr1	6495000	chr1	226625000	4.33E-03	super_enhancer	promoter	RP11-118H4.1
chr1	6505000	chr1	16735000	1.71E-02	super_enhancer	promoter	AL358794.1
chr1	6505000	chr1	43835000	6.38E-03	super_enhancer	promoter	ELOVL1
chr1	6505000	chr1	238115000	1.56E-03	super_enhancer	promoter	MTND5P18
chr1	6615000	chr1	9315000	4.09E-03	promoter	super_enhancer	NOL9
chr1	6615000	chr1	16165000	2.05E-02	promoter	super_enhancer	NOL9
chr1	6615000	chr1	21325000	1.29E-02	promoter	super_enhancer	NOL9
chr1	6615000	chr1	36185000	9.77E-03	promoter	super_enhancer	NOL9
chr1	6615000	chr1	43445000	8.61E-03	promoter	super_enhancer	NOL9
chr1	6615000	chr1	155135000	3.59E-03	promoter	super_enhancer	NOL9
chr1	6615000	chr1	234615000	3.36E-03	promoter	super_enhancer	NOL9
chr1	6675000	chr1	16125000	2.08E-02	promoter	super_enhancer	KLHL21
chr1	6675000	chr1	205305000	4.04E-03	promoter	super_enhancer	KLHL21
chr1	6685000	chr1	12145000	2.98E-02	promoter	super_enhancer	THAP3
chr1	6685000	chr1	12175000	3.62E-02	promoter	super_enhancer	THAP3

chr1	6685000	chr1	26355000	1.38E-02	promoter	super_enhancer	THAP3
chr1	6685000	chr1	204305000	3.25E-03	promoter	super_enhancer	THAP3
chr1	6785000	chr1	7535000	9.22E-03	promoter	super_enhancer	RP11-242F24.1
chr1	6785000	chr1	11785000	4.34E-02	promoter	super_enhancer	RP11-242F24.1
chr1	6785000	chr1	15945000	2.06E-02	promoter	super_enhancer	RP11-242F24.1
chr1	6785000	chr1	16045000	2.75E-02	promoter	super_enhancer	RP11-242F24.1
chr1	6785000	chr1	16055000	2.62E-02	promoter	super_enhancer	RP11-242F24.1
chr1	6785000	chr1	16155000	2.35E-02	promoter	super_enhancer	RP11-242F24.1
chr1	6785000	chr1	32735000	1.13E-02	promoter	super_enhancer	RP11-242F24.1
chr1	6845000	chr1	15795000	1.82E-02	promoter	super_enhancer	RP11-312B8.1
chr1	6845000	chr1	21625000	1.20E-02	promoter	super_enhancer	RP11-312B8.1
chr1	6845000	chr1	36135000	7.00E-03	promoter	super_enhancer	RP11-312B8.1
chr1	6845000	chr1	40855000	5.13E-03	promoter	super_enhancer	RP11-312B8.1
chr1	6845000	chr1	109785000	2.19E-03	promoter	super_enhancer	RP11-312B8.1
chr1	6845000	chr1	230145000	1.60E-03	promoter	super_enhancer	RP11-312B8.1
chr1	6895000	chr1	43555000	6.05E-03	promoter	super_enhancer	RP11-312B8.2
chr1	7075000	chr1	12605000	3.98E-02	promoter	super_enhancer	RP11-334N17.1
chr1	7075000	chr1	16065000	2.65E-02	promoter	super_enhancer	RP11-334N17.1
chr1	7075000	chr1	26715000	1.63E-02	promoter	super_enhancer	RP11-334N17.1
chr1	7075000	chr1	109875000	2.94E-03	promoter	super_enhancer	RP11-334N17.1
chr1	7535000	chr1	11905000	3.86E-02	super_enhancer	promoter	NPPA-AS1_2
chr1	7535000	chr1	12835000	2.91E-02	super_enhancer	promoter	PRAMEF12
chr1	7535000	chr1	16485000	2.20E-02	super_enhancer	promoter	RP11-276H7.3
chr1	7535000	chr1	32225000	9.62E-03	super_enhancer	promoter	MIR4254

chr1	7545000	chr1	12805000	3.11E-02	super_enhancer	promoter	C1orf158
chr1	7545000	chr1	18435000	2.95E-02	super_enhancer	promoter	RP11-174G17.3
chr1	7545000	chr1	33005000	1.22E-02	super_enhancer	promoter	ZBTB8A
chr1	7545000	chr1	53175000	9.09E-03	super_enhancer	promoter	NDUFS5P3
chr1	7545000	chr1	58325000	5.07E-03	super_enhancer	promoter	DAB1-AS1
chr1	7545000	chr1	115055000	3.31E-03	super_enhancer	promoter	TRIM33
chr1	7545000	chr1	144875000	5.69E-03	super_enhancer	promoter	RP4-791M13.5
chr1	7545000	chr1	173795000	3.38E-03	super_enhancer	promoter	CENPL
chr1	7545000	chr1	205745000	2.88E-03	super_enhancer	promoter	RAB29
chr1	7545000	chr1	211385000	4.05E-03	super_enhancer	promoter	AC092017.1
chr1	7545000	chr1	245185000	2.61E-03	super_enhancer	promoter	RNU6-1089P
chr1	7555000	chr1	11905000	4.52E-02	super_enhancer	promoter	NPPA-AS1_2
chr1	7555000	chr1	88965000	2.23E-03	super_enhancer	promoter	RP11-76N22.1
chr1	7555000	chr1	147735000	2.27E-03	super_enhancer	promoter	RNU1-120P
chr1	7555000	chr1	157015000	2.49E-03	super_enhancer	promoter	ARHGEF11
chr1	7555000	chr1	246845000	3.05E-03	super_enhancer	promoter	RP11-439E19.9
chr1	7755000	chr1	9295000	2.52E-02	promoter	super_enhancer	RP11-338N10.1
chr1	7755000	chr1	12595000	4.13E-02	promoter	super_enhancer	RP11-338N10.1
chr1	7755000	chr1	15775000	3.39E-02	promoter	super_enhancer	RP11-338N10.1
chr1	7755000	chr1	23225000	1.83E-02	promoter	super_enhancer	RP11-338N10.1
chr1	7755000	chr1	36155000	1.08E-02	promoter	super_enhancer	RP11-338N10.1
chr1	7755000	chr1	42975000	9.17E-03	promoter	super_enhancer	RP11-338N10.1
chr1	7755000	chr1	94635000	3.44E-03	promoter	super_enhancer	RP11-338N10.1
chr1	7755000	chr1	181135000	4.05E-03	promoter	super_enhancer	RP11-338N10.1

chr1	7755000	chr1	203525000	4.00E-03	promoter	super_enhancer	RP11-338N10.1
chr1	7835000	chr1	21635000	1.81E-02	promoter	super_enhancer	RP3-467L1.6
chr1	7835000	chr1	159925000	2.70E-03	promoter	super_enhancer	RP3-467L1.6
chr1	7835000	chr1	202055000	3.09E-03	promoter	super_enhancer	RP3-467L1.6
chr1	7915000	chr1	9315000	2.79E-02	promoter	super_enhancer	UTS2
chr1	7915000	chr1	12585000	4.89E-02	promoter	super_enhancer	UTS2
chr1	7915000	chr1	40975000	8.18E-03	promoter	super_enhancer	UTS2
chr1	7915000	chr1	212595000	3.07E-03	promoter	super_enhancer	UTS2
chr1	8195000	chr1	17635000	2.27E-02	super_enhancer	promoter	PADI4
chr1	8195000	chr1	28835000	1.24E-02	super_enhancer	promoter	SNORA73B
chr1	8195000	chr1	32255000	1.04E-02	super_enhancer	promoter	RP11-84A19.3
chr1	8195000	chr1	34325000	1.07E-02	super_enhancer	promoter	HMGB4
chr1	8195000	chr1	35975000	8.28E-03	super_enhancer	promoter	RP4-728D4.3
chr1	8195000	chr1	86045000	2.90E-03	super_enhancer	promoter	DDAH1
chr1	8195000	chr1	158175000	1.93E-03	super_enhancer	promoter	RP11-404O13.5
chr1	8195000	chr1	224805000	3.29E-03	super_enhancer	promoter	RP11-100E13.1
chr1	8195000	chr1	238055000	2.53E-03	super_enhancer	promoter	ZP4
chr1	8485000	chr1	12135000	4.40E-02	promoter	super_enhancer	RP5-1115A15.1
chr1	8485000	chr1	12145000	3.07E-02	promoter	super_enhancer	RP5-1115A15.1
chr1	8485000	chr1	12175000	3.70E-02	promoter	super_enhancer	RP5-1115A15.1
chr1	8485000	chr1	15955000	1.47E-02	promoter	super_enhancer	RP5-1115A15.1
chr1	8485000	chr1	42935000	5.32E-03	promoter	super_enhancer	RP5-1115A15.1
chr1	8485000	chr1	235045000	1.82E-03	promoter	super_enhancer	RP5-1115A15.1
chr1	8875000	chr1	16345000	2.58E-02	super_enhancer	promoter	CLCNKA

chr1	8875000	chr1	26395000	1.13E-02	super_enhancer	promoter	TRIM63
chr1	8875000	chr1	33285000	8.30E-03	super_enhancer	promoter	YARS
chr1	8875000	chr1	155905000	3.03E-03	super_enhancer	promoter	KIAA0907
chr1	8875000	chr1	170115000	2.06E-03	super_enhancer	promoter	METTL11B
chr1	8875000	chr1	205535000	2.45E-03	super_enhancer	promoter	RNU2-19P
chr1	8875000	chr1	224805000	2.98E-03	super_enhancer	promoter	RP11-100E13.1
chr1	8885000	chr1	17575000	2.55E-02	super_enhancer	promoter	PADI3
chr1	8885000	chr1	26185000	1.62E-02	super_enhancer	promoter	RP1-125I3.2
chr1	8885000	chr1	209175000	2.75E-03	super_enhancer	promoter	RP5-1051D14.2
chr1	8895000	chr1	12705000	4.51E-02	super_enhancer	promoter	AADACL4
chr1	8895000	chr1	16565000	2.92E-02	super_enhancer	promoter	RSG1
chr1	8895000	chr1	35395000	1.14E-02	super_enhancer	promoter	DLGAP3
chr1	8895000	chr1	40025000	7.60E-03	super_enhancer	promoter	PPIEL
chr1	8895000	chr1	43915000	8.76E-03	super_enhancer	promoter	SZT2-AS1
chr1	8895000	chr1	150855000	3.58E-03	super_enhancer	promoter	RP11-235D19.2
chr1	8895000	chr1	211835000	3.11E-03	super_enhancer	promoter	FDPSP8
chr1	8895000	chr1	222185000	2.30E-03	super_enhancer	promoter	AC092765.1
chr1	8895000	chr1	237205000	2.59E-03	super_enhancer	promoter	RYR2
chr1	8895000	chr1	246935000	1.75E-03	super_enhancer	promoter	RP11-439E19.10
chr1	8905000	chr1	16295000	2.43E-02	super_enhancer	promoter	RP11-5P18.1
chr1	8905000	chr1	16565000	2.90E-02	super_enhancer	promoter	RSG1
chr1	8905000	chr1	16865000	2.22E-02	super_enhancer	promoter	FAM231C
chr1	8905000	chr1	52175000	5.30E-03	super_enhancer	promoter	GAPDHP51
chr1	8905000	chr1	110625000	3.95E-03	super_enhancer	promoter	LINC01397

chr1	8905000	chr1	204465000	3.61E-03	super_enhancer	promoter	PIK3C2B
chr1	8905000	chr1	224045000	2.68E-03	super_enhancer	promoter	PHBP11
chr1	8905000	chr1	224955000	3.55E-03	super_enhancer	promoter	RP11-449J1.1
chr1	8945000	chr1	12135000	3.17E-03	promoter	super_enhancer	RNU6-304P
chr1	8945000	chr1	21335000	1.57E-02	promoter	super_enhancer	RNU6-304P
chr1	8945000	chr1	27535000	8.56E-05	promoter	super_enhancer	RNU6-304P
chr1	8945000	chr1	32765000	1.20E-02	promoter	super_enhancer	RNU6-304P
chr1	8945000	chr1	202085000	3.81E-03	promoter	super_enhancer	RNU6-304P
chr1	8945000	chr1	234735000	2.35E-03	promoter	super_enhancer	RNU6-304P
chr1	9005000	chr1	9295000	9.28E-03	promoter	super_enhancer	CA6
chr1	9005000	chr1	9305000	1.44E-03	promoter	super_enhancer	CA6
chr1	9005000	chr1	16045000	3.41E-02	promoter	super_enhancer	CA6
chr1	9005000	chr1	16195000	2.65E-04	promoter	super_enhancer	CA6
chr1	9005000	chr1	27575000	1.48E-02	promoter	super_enhancer	CA6
chr1	9005000	chr1	31715000	1.26E-02	promoter	super_enhancer	CA6
chr1	9005000	chr1	32765000	1.27E-02	promoter	super_enhancer	CA6
chr1	9005000	chr1	36145000	1.12E-02	promoter	super_enhancer	CA6
chr1	9005000	chr1	36155000	9.61E-03	promoter	super_enhancer	CA6
chr1	9005000	chr1	183275000	2.76E-03	promoter	super_enhancer	CA6
chr1	9275000	chr1	20505000	1.28E-02	super_enhancer	promoter	PLA2G2C
chr1	9275000	chr1	110255000	3.07E-03	super_enhancer	promoter	GSTM5
chr1	9285000	chr1	16315000	2.56E-02	super_enhancer	promoter	RP11-5P18.3
chr1	9285000	chr1	21045000	1.43E-02	super_enhancer	promoter	KIF17
chr1	9285000	chr1	53685000	5.69E-03	super_enhancer	promoter	RP5-1024G6.7

chr1	9285000	chr1	159825000	2.67E-03	super_enhancer	promoter	C1orf204
chr1	9285000	chr1	207325000	2.14E-03	super_enhancer	promoter	RP11-164O23.5
chr1	9295000	chr1	22235000	1.14E-02	super_enhancer	promoter	RP11-26H16.1
chr1	9295000	chr1	38275000	8.44E-03	super_enhancer	promoter	YRDC
chr1	9295000	chr1	47435000	7.54E-03	super_enhancer	promoter	CYP4A26P
chr1	9295000	chr1	59545000	4.60E-03	super_enhancer	promoter	RP11-145M4.2
chr1	9295000	chr1	90095000	2.75E-03	super_enhancer	promoter	RP11-413E1.4
chr1	9305000	chr1	9715000	3.71E-02	super_enhancer	promoter	PIK3CD-AS1
chr1	9305000	chr1	16865000	2.85E-02	super_enhancer	promoter	FAM231C
chr1	9305000	chr1	17215000	4.78E-02	super_enhancer	promoter	RP11-108M9.5
chr1	9305000	chr1	26325000	1.82E-02	super_enhancer	promoter	PAFAH2
chr1	9305000	chr1	52175000	6.62E-03	super_enhancer	promoter	GAPDHP51
chr1	9305000	chr1	54305000	8.40E-03	super_enhancer	promoter	NDC1
chr1	9305000	chr1	64255000	5.14E-03	super_enhancer	promoter	RP4-597J3.1
chr1	9305000	chr1	234845000	3.52E-03	super_enhancer	promoter	RP4-781K5.7
chr1	9315000	chr1	9565000	9.49E-03	super_enhancer	promoter	RP13-392I16.1
chr1	9315000	chr1	21115000	1.66E-02	super_enhancer	promoter	HP1BP3
chr1	9315000	chr1	24195000	1.36E-02	super_enhancer	promoter	FUCA1
chr1	9315000	chr1	28835000	1.32E-02	super_enhancer	promoter	SNORA73B
chr1	9315000	chr1	35525000	7.83E-03	super_enhancer	promoter	ZMYM1
chr1	9315000	chr1	101655000	2.65E-03	super_enhancer	promoter	RP5-1033H2.1
chr1	9315000	chr1	151255000	3.66E-03	super_enhancer	promoter	RP11-126K1.2
chr1	9315000	chr1	241815000	2.66E-03	super_enhancer	promoter	WDR64
chr1	9315000	chr1	247375000	2.59E-03	super_enhancer	promoter	RP11-488L18.4

chr1	9325000	chr1	9885000	4.51E-02	super_enhancer	promoter	CLSTN1
chr1	9325000	chr1	25535000	1.87E-02	super_enhancer	promoter	RP4-706G24.1
chr1	9325000	chr1	32395000	9.18E-03	super_enhancer	promoter	AL136115.1
chr1	9325000	chr1	35315000	1.15E-02	super_enhancer	promoter	RP5-997D16.2
chr1	9325000	chr1	46835000	9.34E-03	super_enhancer	promoter	RP4-603I14.3
chr1	9325000	chr1	51245000	7.25E-03	super_enhancer	promoter	PHBP12
chr1	9325000	chr1	150335000	3.16E-03	super_enhancer	promoter	RPRD2
chr1	9325000	chr1	234745000	3.30E-03	super_enhancer	promoter	IRF2BP2
chr1	9335000	chr1	9565000	5.56E-03	super_enhancer	promoter	RP13-392I16.1
chr1	9335000	chr1	9635000	3.42E-03	super_enhancer	promoter	RP13-15M17.1
chr1	9335000	chr1	15935000	3.46E-02	super_enhancer	promoter	RNU7-179P
chr1	9335000	chr1	16735000	3.15E-02	super_enhancer	promoter	AL358794.1
chr1	9335000	chr1	17575000	2.64E-02	super_enhancer	promoter	PADI3
chr1	9335000	chr1	22385000	1.93E-02	super_enhancer	promoter	CDC42-IT1
chr1	9335000	chr1	24515000	1.87E-02	super_enhancer	promoter	IFNLR1
chr1	9335000	chr1	32635000	1.30E-02	super_enhancer	promoter	RP4-622L5.2
chr1	9335000	chr1	43035000	8.22E-03	super_enhancer	promoter	RNU6-536P
chr1	9335000	chr1	44585000	9.90E-03	super_enhancer	promoter	KLF17
chr1	9335000	chr1	52445000	7.83E-03	super_enhancer	promoter	RP11-91A18.1
chr1	9335000	chr1	85725000	4.03E-03	super_enhancer	promoter	C1orf52
chr1	9335000	chr1	166125000	3.74E-03	super_enhancer	promoter	MIR921
chr1	9345000	chr1	19935000	1.92E-02	super_enhancer	promoter	RP5-1056L3.3
chr1	9345000	chr1	24105000	1.55E-02	super_enhancer	promoter	TCEB3-AS1
chr1	9345000	chr1	27975000	1.13E-02	super_enhancer	promoter	RP1-159A19.3

chr1	9345000	chr1	28695000	1.43E-02	super_enhancer	promoter	PHACTR4
chr1	9345000	chr1	40505000	1.01E-02	super_enhancer	promoter	CAP1
chr1	9345000	chr1	45265000	8.40E-03	super_enhancer	promoter	PLK3
chr1	9345000	chr1	156785000	3.80E-03	super_enhancer	promoter	NTRK1
chr1	9565000	chr1	10905000	4.63E-02	promoter	super_enhancer	RP13-392I16.1
chr1	9565000	chr1	15775000	4.31E-02	promoter	super_enhancer	RP13-392I16.1
chr1	9565000	chr1	23205000	2.62E-02	promoter	super_enhancer	RP13-392I16.1
chr1	9565000	chr1	26295000	1.74E-02	promoter	super_enhancer	RP13-392I16.1
chr1	9635000	chr1	31715000	1.25E-02	promoter	super_enhancer	RP13-15M17.1
chr1	9635000	chr1	40895000	8.01E-03	promoter	super_enhancer	RP13-15M17.1
chr1	9715000	chr1	15805000	2.43E-02	promoter	super_enhancer	PIK3CD-AS1
chr1	9715000	chr1	16115000	2.58E-02	promoter	super_enhancer	PIK3CD-AS1
chr1	9715000	chr1	40885000	6.19E-03	promoter	super_enhancer	PIK3CD-AS1
chr1	9715000	chr1	41515000	7.73E-03	promoter	super_enhancer	PIK3CD-AS1
chr1	9715000	chr1	212565000	2.63E-03	promoter	super_enhancer	PIK3CD-AS1
chr1	9885000	chr1	15345000	3.26E-02	promoter	super_enhancer	CLSTN1
chr1	9885000	chr1	18925000	2.04E-02	promoter	super_enhancer	CLSTN1
chr1	9885000	chr1	23195000	1.56E-02	promoter	super_enhancer	CLSTN1
chr1	9885000	chr1	27535000	1.32E-02	promoter	super_enhancer	CLSTN1
chr1	9885000	chr1	206905000	3.44E-03	promoter	super_enhancer	CLSTN1
chr1	10115000	chr1	15945000	2.96E-02	promoter	super_enhancer	RP11-496H15.2
chr1	10115000	chr1	16195000	2.61E-02	promoter	super_enhancer	RP11-496H15.2
chr1	10115000	chr1	39175000	9.35E-03	promoter	super_enhancer	RP11-496H15.2
chr1	10115000	chr1	43475000	8.70E-03	promoter	super_enhancer	RP11-496H15.2

chr1	10895000	chr1	16765000	3.62E-02	super_enhancer	promoter	SPATA21
chr1	10895000	chr1	26485000	1.97E-02	super_enhancer	promoter	FAM110D
chr1	10895000	chr1	28855000	1.63E-02	super_enhancer	promoter	PRDX3P2
chr1	10895000	chr1	46645000	9.92E-03	super_enhancer	promoter	AL672043.1
chr1	10895000	chr1	52615000	7.47E-03	super_enhancer	promoter	RN7SL788P
chr1	10895000	chr1	112015000	3.31E-03	super_enhancer	promoter	C1orf162
chr1	10895000	chr1	150855000	3.79E-03	super_enhancer	promoter	RP11-235D19.2
chr1	10895000	chr1	153515000	4.20E-03	super_enhancer	promoter	S100A5
chr1	10905000	chr1	16315000	4.12E-02	super_enhancer	promoter	RP11-5P18.3
chr1	10905000	chr1	16345000	4.14E-02	super_enhancer	promoter	CLCNKA
chr1	10905000	chr1	23415000	2.21E-02	super_enhancer	promoter	RP1-184J9.2
chr1	10905000	chr1	25595000	1.16E-02	super_enhancer	promoter	RP3-465N24.5
chr1	10905000	chr1	26215000	1.63E-02	super_enhancer	promoter	RP1-125I3.4
chr1	10905000	chr1	28905000	1.44E-02	super_enhancer	promoter	SNORD99
chr1	10905000	chr1	32675000	1.41E-02	super_enhancer	promoter	DCDC2B
chr1	10905000	chr1	35395000	1.27E-02	super_enhancer	promoter	DLGAP3
chr1	10905000	chr1	78245000	3.50E-03	super_enhancer	promoter	FAM73A
chr1	10905000	chr1	111935000	2.88E-03	super_enhancer	promoter	PGCP1
chr1	10955000	chr1	23595000	1.99E-02	promoter	super_enhancer	HSPE1P24
chr1	10955000	chr1	26875000	1.82E-02	promoter	super_enhancer	HSPE1P24
chr1	10955000	chr1	207015000	2.61E-03	promoter	super_enhancer	HSPE1P24
chr1	11285000	chr1	15775000	4.22E-02	promoter	super_enhancer	RNU6-291P
chr1	11285000	chr1	27505000	1.26E-02	promoter	super_enhancer	RNU6-291P
chr1	11285000	chr1	36155000	8.48E-03	promoter	super_enhancer	RNU6-291P

chr1	11785000	chr1	17575000	3.59E-02	super_enhancer	promoter	PADI3
chr1	11785000	chr1	22095000	2.42E-02	super_enhancer	promoter	RP11-132G19.3
chr1	11785000	chr1	22385000	2.13E-02	super_enhancer	promoter	CDC42-IT1
chr1	11785000	chr1	35815000	1.11E-02	super_enhancer	promoter	RPL5P4
chr1	11785000	chr1	42605000	1.05E-02	super_enhancer	promoter	RP11-319C21.1
chr1	11785000	chr1	146645000	2.52E-03	super_enhancer	promoter	PRKAB2
chr1	11785000	chr1	222885000	3.62E-03	super_enhancer	promoter	BROX
chr1	11785000	chr1	248005000	3.29E-03	super_enhancer	promoter	OR11L1
chr1	11795000	chr1	17575000	3.58E-02	super_enhancer	promoter	PADI3
chr1	11795000	chr1	22385000	2.12E-02	super_enhancer	promoter	CDC42-IT1
chr1	11795000	chr1	23075000	2.08E-02	super_enhancer	promoter	AL611946.1
chr1	11795000	chr1	23415000	2.39E-02	super_enhancer	promoter	RP1-184J9.2
chr1	11795000	chr1	27995000	2.35E-02	super_enhancer	promoter	RP11-288L9.4
chr1	11795000	chr1	43915000	1.03E-02	super_enhancer	promoter	SZT2-AS1
chr1	11795000	chr1	47135000	9.67E-03	super_enhancer	promoter	TEX38
chr1	11795000	chr1	53815000	6.80E-03	super_enhancer	promoter	RP11-117D22.1
chr1	11795000	chr1	77815000	4.13E-03	super_enhancer	promoter	RP11-375A5.1
chr1	11795000	chr1	193105000	2.90E-03	super_enhancer	promoter	MIR1278
chr1	11795000	chr1	219935000	2.44E-03	super_enhancer	promoter	RNA5SP76
chr1	11795000	chr1	224445000	3.00E-03	super_enhancer	promoter	MIR320B2
chr1	11805000	chr1	16315000	4.62E-02	super_enhancer	promoter	RP11-5P18.3
chr1	11805000	chr1	16865000	3.31E-02	super_enhancer	promoter	FAM231C
chr1	11805000	chr1	17305000	3.00E-02	super_enhancer	promoter	RP1-37C10.3
chr1	11805000	chr1	21115000	1.81E-02	super_enhancer	promoter	HP1BP3

chr1	11805000	chr1	23045000	2.06E-02	super_enhancer	promoter	MIR4684
chr1	11805000	chr1	228265000	2.88E-03	super_enhancer	promoter	RP5-881P19.7
chr1	11805000	chr1	247365000	2.35E-03	super_enhancer	promoter	MIR3916
chr1	11865000	chr1	15755000	4.98E-02	promoter	super_enhancer	CLCN6
chr1	11865000	chr1	41515000	7.84E-03	promoter	super_enhancer	CLCN6
chr1	11865000	chr1	43425000	9.02E-03	promoter	super_enhancer	CLCN6
chr1	11865000	chr1	159925000	2.72E-03	promoter	super_enhancer	CLCN6
chr1	11865000	chr1	205295000	2.94E-03	promoter	super_enhancer	CLCN6
chr1	11905000	chr1	42955000	8.15E-03	promoter	super_enhancer	NPPA-AS1_2
chr1	12135000	chr1	28995000	2.06E-02	super_enhancer	promoter	GMEB1
chr1	12135000	chr1	42225000	1.04E-02	super_enhancer	promoter	AL451006.1
chr1	12135000	chr1	53385000	9.12E-03	super_enhancer	promoter	RP4-631H13.6
chr1	12135000	chr1	247365000	2.91E-03	super_enhancer	promoter	MIR3916
chr1	12145000	chr1	12835000	1.93E-02	super_enhancer	promoter	PRAMEF12
chr1	12145000	chr1	160175000	2.80E-03	super_enhancer	promoter	PEA15
chr1	12145000	chr1	234795000	2.85E-03	super_enhancer	promoter	RP4-781K5.5
chr1	12155000	chr1	16485000	4.34E-02	super_enhancer	promoter	RP11-276H7.3
chr1	12155000	chr1	17515000	2.43E-02	super_enhancer	promoter	RP11-380J14.1
chr1	12155000	chr1	24285000	1.15E-02	super_enhancer	promoter	PNRC2
chr1	12155000	chr1	32395000	6.78E-03	super_enhancer	promoter	AL136115.1
chr1	12155000	chr1	51245000	5.19E-03	super_enhancer	promoter	PHBP12
chr1	12155000	chr1	203255000	2.32E-03	super_enhancer	promoter	LINC01353
chr1	12155000	chr1	247615000	2.24E-03	super_enhancer	promoter	OR2B11
chr1	12165000	chr1	13185000	3.03E-02	super_enhancer	promoter	HNRNPCL2

chr1	12165000	chr1	16295000	4.92E-02	super_enhancer	promoter	RP11-5P18.1
chr1	12165000	chr1	16555000	4.88E-02	super_enhancer	promoter	ANO7P1
chr1	12165000	chr1	28695000	1.77E-02	super_enhancer	promoter	PHACTR4
chr1	12165000	chr1	29205000	1.88E-02	super_enhancer	promoter	RP1-212P9.3
chr1	12165000	chr1	45455000	1.02E-02	super_enhancer	promoter	RP5-882O7.4
chr1	12165000	chr1	46805000	8.60E-03	super_enhancer	promoter	NSUN4
chr1	12165000	chr1	150265000	3.71E-03	super_enhancer	promoter	MRPS21
chr1	12165000	chr1	229685000	3.36E-03	super_enhancer	promoter	RNA5SP78
chr1	12175000	chr1	17215000	1.88E-03	super_enhancer	promoter	RP11-108M9.5
chr1	12175000	chr1	26795000	1.00E-02	super_enhancer	promoter	RP3-476K8.3
chr1	12175000	chr1	28435000	1.71E-02	super_enhancer	promoter	RP5-1053E7.2
chr1	12175000	chr1	33005000	1.20E-02	super_enhancer	promoter	ZBTB8A
chr1	12175000	chr1	38325000	1.20E-02	super_enhancer	promoter	MTF1
chr1	12175000	chr1	183235000	3.50E-03	super_enhancer	promoter	RP11-548N1.1
chr1	12175000	chr1	184355000	2.93E-03	super_enhancer	promoter	C1orf21
chr1	12175000	chr1	218205000	2.26E-03	super_enhancer	promoter	RP11-152L7.1
chr1	12485000	chr1	22395000	1.95E-02	super_enhancer	promoter	RP1-224A6.8
chr1	12485000	chr1	27995000	2.01E-02	super_enhancer	promoter	RP11-288L9.4
chr1	12485000	chr1	32645000	1.18E-02	super_enhancer	promoter	TXLNA
chr1	12485000	chr1	89295000	1.72E-03	super_enhancer	promoter	TCEB1P19
chr1	12485000	chr1	167905000	2.22E-03	super_enhancer	promoter	DCAF6
chr1	12485000	chr1	185305000	2.76E-03	super_enhancer	promoter	GS1-279B7.1
chr1	12485000	chr1	209605000	3.07E-03	super_enhancer	promoter	MIR205
chr1	12485000	chr1	228585000	1.85E-03	super_enhancer	promoter	MIR6742

chr1	12535000	chr1	15935000	1.86E-03	super_enhancer	promoter	RNU7-179P
chr1	12535000	chr1	16295000	4.60E-02	super_enhancer	promoter	RP11-5P18.1
chr1	12535000	chr1	16555000	4.50E-02	super_enhancer	promoter	ANO7P1
chr1	12535000	chr1	25105000	1.70E-02	super_enhancer	promoter	RNU6-1208P
chr1	12535000	chr1	78445000	3.26E-03	super_enhancer	promoter	FUBP1
chr1	12535000	chr1	161035000	3.16E-03	super_enhancer	promoter	AL591806.1
chr1	12535000	chr1	236305000	2.79E-03	super_enhancer	promoter	GPR137B
chr1	12545000	chr1	16345000	1.21E-03	super_enhancer	promoter	CLCNKA
chr1	12545000	chr1	24195000	1.29E-02	super_enhancer	promoter	FUCA1
chr1	12545000	chr1	65885000	4.17E-03	super_enhancer	promoter	LEPROT
chr1	12545000	chr1	160865000	1.80E-03	super_enhancer	promoter	RP11-312J18.5
chr1	12545000	chr1	248005000	2.44E-03	super_enhancer	promoter	OR11L1
chr1	12555000	chr1	33285000	1.04E-02	super_enhancer	promoter	YARS
chr1	12555000	chr1	59545000	5.20E-03	super_enhancer	promoter	RP11-145M4.2
chr1	12555000	chr1	94345000	3.22E-03	super_enhancer	promoter	DNTTIP2
chr1	12555000	chr1	212865000	2.81E-03	super_enhancer	promoter	RNU6-423P
chr1	12555000	chr1	241295000	1.99E-03	super_enhancer	promoter	MIR3123
chr1	12565000	chr1	12805000	7.10E-03	super_enhancer	promoter	C1orf158
chr1	12565000	chr1	17515000	4.20E-02	super_enhancer	promoter	RP11-380J14.1
chr1	12565000	chr1	18435000	4.44E-02	super_enhancer	promoter	RP11-174G17.3
chr1	12565000	chr1	19935000	2.78E-02	super_enhancer	promoter	RP5-1056L3.3
chr1	12565000	chr1	35395000	1.46E-02	super_enhancer	promoter	DLGAP3
chr1	12565000	chr1	52005000	8.16E-03	super_enhancer	promoter	RNU6-1281P
chr1	12565000	chr1	58325000	5.35E-03	super_enhancer	promoter	DAB1-AS1

chr1	12565000	chr1	65545000	6.53E-03	super_enhancer	promoter	RP4-535B20.2
chr1	12565000	chr1	77885000	3.42E-03	super_enhancer	promoter	RNU7-8P
chr1	12565000	chr1	168185000	3.90E-03	super_enhancer	promoter	RP1-69E11.3
chr1	12565000	chr1	224485000	3.36E-03	super_enhancer	promoter	RP11-365O16.5
chr1	12565000	chr1	228265000	3.41E-03	super_enhancer	promoter	RP5-881P19.7
chr1	12565000	chr1	231115000	3.58E-03	super_enhancer	promoter	TTC13
chr1	12565000	chr1	233035000	3.03E-03	super_enhancer	promoter	AL122003.1
chr1	12575000	chr1	33815000	1.14E-02	super_enhancer	promoter	RP11-415J8.5
chr1	12575000	chr1	44585000	9.77E-03	super_enhancer	promoter	KLF17
chr1	12575000	chr1	57085000	5.53E-03	super_enhancer	promoter	RP11-377K22.2
chr1	12575000	chr1	203275000	3.76E-03	super_enhancer	promoter	LINC01136
chr1	12575000	chr1	234795000	3.44E-03	super_enhancer	promoter	RP4-781K5.5
chr1	12585000	chr1	16245000	1.76E-03	super_enhancer	promoter	RP1-134O19.3
chr1	12585000	chr1	62095000	6.70E-03	super_enhancer	promoter	AC099791.1
chr1	12585000	chr1	165185000	2.75E-03	super_enhancer	promoter	RP11-38C18.3
chr1	12585000	chr1	212225000	3.73E-03	super_enhancer	promoter	RPL21P28
chr1	12585000	chr1	248685000	2.07E-03	super_enhancer	promoter	OR2G6
chr1	12595000	chr1	42805000	9.68E-03	super_enhancer	promoter	RP11-223A3.1
chr1	12595000	chr1	77245000	3.43E-03	super_enhancer	promoter	RP11-415A20.1
chr1	12595000	chr1	84945000	2.70E-03	super_enhancer	promoter	RPF1
chr1	12595000	chr1	101185000	2.13E-03	super_enhancer	promoter	VCAM1
chr1	12595000	chr1	155715000	2.86E-03	super_enhancer	promoter	MSTO2P
chr1	12595000	chr1	158575000	1.52E-03	super_enhancer	promoter	OR10Z1
chr1	12595000	chr1	203765000	2.69E-03	super_enhancer	promoter	ZC3H11A

chr1	12595000	chr1	227915000	2.51E-03	super_enhancer	promoter	SNAP47
chr1	12605000	chr1	21045000	2.17E-02	super_enhancer	promoter	KIF17
chr1	12605000	chr1	24285000	1.73E-02	super_enhancer	promoter	PNRC2
chr1	12605000	chr1	27995000	2.36E-02	super_enhancer	promoter	RP11-288L9.4
chr1	12605000	chr1	35245000	1.24E-02	super_enhancer	promoter	AL121988.1
chr1	12605000	chr1	38555000	1.08E-02	super_enhancer	promoter	MIR3659
chr1	12605000	chr1	43035000	8.60E-03	super_enhancer	promoter	RNU6-536P
chr1	12605000	chr1	236305000	3.23E-03	super_enhancer	promoter	GPR137B
chr1	12705000	chr1	15335000	2.61E-03	promoter	super_enhancer	AADACL4
chr1	12705000	chr1	23215000	2.08E-02	promoter	super_enhancer	AADACL4
chr1	12705000	chr1	26865000	1.47E-02	promoter	super_enhancer	AADACL4
chr1	12775000	chr1	16175000	4.60E-02	promoter	super_enhancer	AADACL3
chr1	12775000	chr1	36175000	1.32E-02	promoter	super_enhancer	AADACL3
chr1	12775000	chr1	37475000	1.13E-02	promoter	super_enhancer	AADACL3
chr1	12805000	chr1	23575000	1.50E-02	promoter	super_enhancer	C1orf158
chr1	12805000	chr1	39195000	4.94E-03	promoter	super_enhancer	C1orf158
chr1	12805000	chr1	230105000	1.64E-03	promoter	super_enhancer	C1orf158
chr1	12835000	chr1	21325000	1.89E-02	promoter	super_enhancer	PRAMEF12
chr1	12985000	chr1	15345000	4.71E-02	promoter	super_enhancer	RP11-450I1.2
chr1	12985000	chr1	16165000	2.86E-02	promoter	super_enhancer	RP11-450I1.2
chr1	13185000	chr1	212595000	1.87E-03	promoter	super_enhancer	HNRNPCL2
chr1	14925000	chr1	15325000	3.68E-02	promoter	super_enhancer	KAZN
chr1	14925000	chr1	15415000	2.20E-02	promoter	super_enhancer	KAZN
chr1	14925000	chr1	15785000	4.21E-02	promoter	super_enhancer	KAZN

chr1	14925000	chr1	16075000	3.38E-02	promoter	super_enhancer	KAZN
chr1	14925000	chr1	26335000	1.35E-02	promoter	super_enhancer	KAZN
chr1	14925000	chr1	26355000	1.42E-02	promoter	super_enhancer	KAZN
chr1	14925000	chr1	43205000	8.90E-03	promoter	super_enhancer	KAZN
chr1	14925000	chr1	212565000	1.92E-03	promoter	super_enhancer	KAZN
chr1	14935000	chr1	36145000	1.28E-02	promoter	super_enhancer	AL034395.1
chr1	14935000	chr1	112705000	2.66E-03	promoter	super_enhancer	AL034395.1
chr1	15325000	chr1	35325000	1.21E-02	super_enhancer	promoter	SMIM12
chr1	15325000	chr1	160085000	3.35E-03	super_enhancer	promoter	ATP1A2
chr1	15325000	chr1	204655000	2.99E-03	super_enhancer	promoter	LRRN2
chr1	15335000	chr1	42845000	1.18E-02	super_enhancer	promoter	RIMKLA
chr1	15335000	chr1	62505000	6.30E-03	super_enhancer	promoter	LAMTOR5P1
chr1	15335000	chr1	100495000	2.14E-03	super_enhancer	promoter	RP4-714D9.5
chr1	15335000	chr1	144945000	3.85E-03	super_enhancer	promoter	RNU2-38P
chr1	15345000	chr1	160765000	3.35E-03	super_enhancer	promoter	LY9
chr1	15345000	chr1	228605000	2.86E-03	super_enhancer	promoter	TRIM17
chr1	15355000	chr1	42605000	1.15E-02	super_enhancer	promoter	RP11-319C21.1
chr1	15355000	chr1	64935000	3.99E-03	super_enhancer	promoter	CACHD1
chr1	15355000	chr1	67995000	6.73E-03	super_enhancer	promoter	RP4-763G1.2
chr1	15355000	chr1	248365000	2.23E-03	super_enhancer	promoter	OR2M3
chr1	15415000	chr1	22385000	2.46E-02	super_enhancer	promoter	CDC42-IT1
chr1	15425000	chr1	15735000	4.01E-02	super_enhancer	promoter	EFHD2
chr1	15425000	chr1	25665000	1.42E-02	super_enhancer	promoter	RSRP1
chr1	15425000	chr1	28435000	1.85E-02	super_enhancer	promoter	RP5-1053E7.2

chr1	15425000	chr1	44115000	8.33E-03	super_enhancer	promoter	KDM4A
chr1	15425000	chr1	92495000	2.81E-03	super_enhancer	promoter	EPHX4
chr1	15425000	chr1	201475000	2.88E-03	super_enhancer	promoter	RP11-134G8.7
chr1	15435000	chr1	26185000	2.15E-02	super_enhancer	promoter	RP1-125I3.2
chr1	15435000	chr1	42845000	1.37E-02	super_enhancer	promoter	RIMKLA
chr1	15435000	chr1	46155000	1.14E-02	super_enhancer	promoter	GPBP1L1
chr1	15435000	chr1	47965000	9.69E-03	super_enhancer	promoter	RPL21P24
chr1	15435000	chr1	52645000	6.70E-03	super_enhancer	promoter	PDCL3P6
chr1	15735000	chr1	33045000	1.46E-02	promoter	super_enhancer	EFHD2
chr1	15735000	chr1	40915000	1.17E-02	promoter	super_enhancer	EFHD2
chr1	15735000	chr1	181125000	3.32E-03	promoter	super_enhancer	EFHD2
chr1	15735000	chr1	206895000	3.05E-03	promoter	super_enhancer	EFHD2
chr1	15735000	chr1	212495000	2.50E-03	promoter	super_enhancer	EFHD2
chr1	15755000	chr1	53795000	7.23E-03	super_enhancer	promoter	LRP8
chr1	15755000	chr1	54305000	8.25E-03	super_enhancer	promoter	NDC1
chr1	15755000	chr1	110655000	3.60E-03	super_enhancer	promoter	UBL4B
chr1	15755000	chr1	191845000	2.13E-03	super_enhancer	promoter	RP11-541F9.2
chr1	15755000	chr1	228465000	3.41E-03	super_enhancer	promoter	RP5-1139B12.3
chr1	15755000	chr1	244515000	3.03E-03	super_enhancer	promoter	C1orf100
chr1	15765000	chr1	23505000	2.53E-02	super_enhancer	promoter	LUZP1
chr1	15765000	chr1	27715000	2.84E-02	super_enhancer	promoter	RP1-144C9.2
chr1	15765000	chr1	46645000	1.23E-02	super_enhancer	promoter	AL672043.1
chr1	15765000	chr1	75165000	3.56E-03	super_enhancer	promoter	RP11-17E13.2
chr1	15765000	chr1	112905000	3.86E-03	super_enhancer	promoter	TXNP3

chr1	15765000	chr1	168745000	2.70E-03	super_enhancer	promoter	RP1-10C16.1
chr1	15765000	chr1	209605000	3.89E-03	super_enhancer	promoter	MIR205
chr1	15775000	chr1	20355000	3.39E-02	super_enhancer	promoter	PLA2G5
chr1	15775000	chr1	20465000	5.00E-02	super_enhancer	promoter	PLA2G2F
chr1	15775000	chr1	28905000	1.96E-02	super_enhancer	promoter	SNORD99
chr1	15775000	chr1	228465000	3.85E-03	super_enhancer	promoter	RP5-1139B12.3
chr1	15785000	chr1	28695000	1.76E-02	super_enhancer	promoter	PHACTR4
chr1	15785000	chr1	32225000	1.69E-02	super_enhancer	promoter	MIR4254
chr1	15785000	chr1	32675000	1.52E-02	super_enhancer	promoter	DCDC2B
chr1	15785000	chr1	35135000	1.08E-02	super_enhancer	promoter	MIR552
chr1	15785000	chr1	43855000	1.11E-02	super_enhancer	promoter	MED8
chr1	15785000	chr1	55505000	6.72E-03	super_enhancer	promoter	PCSK9
chr1	15785000	chr1	75165000	2.89E-03	super_enhancer	promoter	RP11-17E13.2
chr1	15785000	chr1	202765000	3.03E-03	super_enhancer	promoter	SLC25A39P1
chr1	15785000	chr1	240565000	2.25E-03	super_enhancer	promoter	RP11-177F11.1
chr1	15795000	chr1	93435000	3.17E-03	super_enhancer	promoter	RNU6-970P
chr1	15795000	chr1	168195000	2.85E-03	super_enhancer	promoter	SFT2D2
chr1	15805000	chr1	47185000	7.52E-03	super_enhancer	promoter	EFCAB14
chr1	15805000	chr1	55505000	5.60E-03	super_enhancer	promoter	PCSK9
chr1	15805000	chr1	86045000	2.59E-03	super_enhancer	promoter	DDAH1
chr1	15805000	chr1	236305000	2.36E-03	super_enhancer	promoter	GPR137B
chr1	15935000	chr1	16055000	2.22E-03	promoter	super_enhancer	RNU7-179P
chr1	15935000	chr1	16065000	3.94E-02	promoter	super_enhancer	RNU7-179P
chr1	15935000	chr1	26315000	2.05E-02	promoter	super_enhancer	RNU7-179P

chr1	15945000	chr1	15985000	3.83E-03	super_enhancer	promoter	RSC1A1
chr1	15945000	chr1	38325000	1.22E-02	super_enhancer	promoter	MTF1
chr1	15945000	chr1	86495000	2.13E-03	super_enhancer	promoter	RP4-682E18.1
chr1	15945000	chr1	183155000	2.14E-03	super_enhancer	promoter	LAMC2
chr1	15955000	chr1	29475000	1.62E-02	super_enhancer	promoter	RP11-242O24.3
chr1	15955000	chr1	64255000	4.24E-03	super_enhancer	promoter	RP4-597J3.1
chr1	15955000	chr1	120355000	2.38E-03	super_enhancer	promoter	REG4
chr1	15955000	chr1	182915000	2.61E-03	super_enhancer	promoter	RNA5SP71
chr1	15955000	chr1	206715000	3.10E-03	super_enhancer	promoter	CH17-76K2.6
chr1	15955000	chr1	245135000	2.28E-03	super_enhancer	promoter	RP11-156E8.1
chr1	15985000	chr1	26355000	1.88E-02	promoter	super_enhancer	RSC1A1
chr1	15985000	chr1	27575000	1.81E-02	promoter	super_enhancer	RSC1A1
chr1	15985000	chr1	31695000	1.67E-02	promoter	super_enhancer	RSC1A1
chr1	15985000	chr1	36165000	1.25E-02	promoter	super_enhancer	RSC1A1
chr1	16045000	chr1	24335000	2.46E-02	super_enhancer	promoter	RP11-4M23.7
chr1	16045000	chr1	25105000	2.71E-02	super_enhancer	promoter	RNU6-1208P
chr1	16045000	chr1	27995000	3.18E-02	super_enhancer	promoter	RP11-288L9.4
chr1	16045000	chr1	32255000	1.76E-02	super_enhancer	promoter	RP11-84A19.3
chr1	16045000	chr1	32675000	2.01E-02	super_enhancer	promoter	DCDC2B
chr1	16045000	chr1	53925000	1.06E-02	super_enhancer	promoter	DMRTB1
chr1	16045000	chr1	109205000	3.09E-03	super_enhancer	promoter	HENMT1
chr1	16045000	chr1	224485000	3.58E-03	super_enhancer	promoter	RP11-365O16.5
chr1	16045000	chr1	247495000	4.11E-03	super_enhancer	promoter	ZNF496
chr1	16045000	chr1	248605000	2.46E-03	super_enhancer	promoter	OR2T7

chr1	16055000	chr1	28435000	2.34E-02	super_enhancer	promoter	RP5-1053E7.2
chr1	16055000	chr1	28585000	2.40E-02	super_enhancer	promoter	SESN2
chr1	16055000	chr1	34335000	1.58E-02	super_enhancer	promoter	CSMD2-AS1
chr1	16055000	chr1	51625000	5.42E-05	super_enhancer	promoter	CFL1P2
chr1	16055000	chr1	77685000	3.19E-03	super_enhancer	promoter	PIGK
chr1	16055000	chr1	92495000	3.49E-03	super_enhancer	promoter	EPHX4
chr1	16055000	chr1	157015000	3.38E-03	super_enhancer	promoter	ARHGEF11
chr1	16055000	chr1	218475000	2.63E-03	super_enhancer	promoter	RP11-224O19.4
chr1	16055000	chr1	237145000	4.12E-03	super_enhancer	promoter	RPL35P1
chr1	16065000	chr1	24515000	2.57E-02	super_enhancer	promoter	IFNLR1
chr1	16065000	chr1	32635000	1.65E-02	super_enhancer	promoter	RP4-622L5.2
chr1	16065000	chr1	35975000	1.21E-02	super_enhancer	promoter	RP4-728D4.3
chr1	16065000	chr1	54205000	9.10E-03	super_enhancer	promoter	GLIS1
chr1	16065000	chr1	206715000	3.85E-03	super_enhancer	promoter	CH17-76K2.6
chr1	16075000	chr1	16315000	4.92E-02	super_enhancer	promoter	RP11-5P18.3
chr1	16075000	chr1	16345000	3.92E-02	super_enhancer	promoter	CLCNKA
chr1	16075000	chr1	20915000	4.55E-02	super_enhancer	promoter	CDA
chr1	16075000	chr1	32675000	1.97E-04	super_enhancer	promoter	DCDC2B
chr1	16075000	chr1	35245000	1.60E-02	super_enhancer	promoter	AL121988.1
chr1	16085000	chr1	16315000	1.22E-02	super_enhancer	promoter	RP11-5P18.3
chr1	16085000	chr1	16345000	2.16E-03	super_enhancer	promoter	CLCNKA
chr1	16085000	chr1	29475000	2.07E-02	super_enhancer	promoter	RP11-242O24.3
chr1	16085000	chr1	38275000	1.26E-02	super_enhancer	promoter	YRDC
chr1	16085000	chr1	42845000	1.41E-02	super_enhancer	promoter	RIMKLA

chr1	16085000	chr1	45205000	1.01E-02	super_enhancer	promoter	KIF2C
chr1	16085000	chr1	160905000	3.08E-03	super_enhancer	promoter	RP11-312J18.6
chr1	16085000	chr1	214705000	3.38E-03	super_enhancer	promoter	KRT18P12
chr1	16115000	chr1	16295000	1.48E-02	super_enhancer	promoter	RP11-5P18.1
chr1	16115000	chr1	16345000	1.74E-02	super_enhancer	promoter	CLCNKA
chr1	16115000	chr1	23045000	2.58E-02	super_enhancer	promoter	MIR4684
chr1	16115000	chr1	27715000	2.18E-02	super_enhancer	promoter	RP1-144C9.2
chr1	16115000	chr1	48015000	9.17E-03	super_enhancer	promoter	ATP6V0E1P4
chr1	16115000	chr1	248855000	2.10E-03	super_enhancer	promoter	XX-CR54.1
chr1	16125000	chr1	29475000	1.74E-02	super_enhancer	promoter	RP11-242O24.3
chr1	16125000	chr1	33005000	1.29E-02	super_enhancer	promoter	ZBTB8A
chr1	16125000	chr1	78445000	3.58E-03	super_enhancer	promoter	FUBP1
chr1	16125000	chr1	102195000	2.19E-03	super_enhancer	promoter	RNU6-965P
chr1	16125000	chr1	113465000	3.00E-03	super_enhancer	promoter	AKR7A2P1
chr1	16125000	chr1	151805000	3.64E-03	super_enhancer	promoter	RORC
chr1	16125000	chr1	228585000	1.90E-03	super_enhancer	promoter	MIR6742
chr1	16135000	chr1	16245000	8.65E-05	super_enhancer	promoter	RP1-134O19.3
chr1	16135000	chr1	16295000	3.37E-02	super_enhancer	promoter	RP11-5P18.1
chr1	16135000	chr1	16315000	4.14E-03	super_enhancer	promoter	RP11-5P18.3
chr1	16135000	chr1	16345000	1.07E-02	super_enhancer	promoter	CLCNKA
chr1	16135000	chr1	93305000	3.17E-03	super_enhancer	promoter	SNORA66
chr1	16135000	chr1	150855000	3.57E-03	super_enhancer	promoter	RP11-235D19.2
chr1	16135000	chr1	204595000	3.65E-03	super_enhancer	promoter	RP11-430C7.5
chr1	16135000	chr1	234795000	3.46E-03	super_enhancer	promoter	RP4-781K5.5

chr1	16145000	chr1	32675000	1.81E-02	super_enhancer	promoter	DCDC2B
chr1	16145000	chr1	37945000	1.38E-02	super_enhancer	promoter	MIR6732
chr1	16145000	chr1	44855000	9.64E-03	super_enhancer	promoter	RNU6-369P
chr1	16145000	chr1	74215000	3.74E-03	super_enhancer	promoter	RNA5SP50
chr1	16145000	chr1	110935000	2.92E-03	super_enhancer	promoter	SLC16A4
chr1	16145000	chr1	120395000	2.44E-03	super_enhancer	promoter	PFN1P9
chr1	16145000	chr1	232295000	2.47E-03	super_enhancer	promoter	RP5-865N13.2
chr1	16145000	chr1	241815000	2.89E-03	super_enhancer	promoter	WDR64
chr1	16155000	chr1	16295000	4.29E-03	super_enhancer	promoter	RP11-5P18.1
chr1	16155000	chr1	16315000	7.30E-03	super_enhancer	promoter	RP11-5P18.3
chr1	16155000	chr1	24335000	2.14E-02	super_enhancer	promoter	RP11-4M23.7
chr1	16155000	chr1	42605000	1.16E-02	super_enhancer	promoter	RP11-319C21.1
chr1	16155000	chr1	53175000	9.82E-03	super_enhancer	promoter	NDUFS5P3
chr1	16155000	chr1	146475000	2.43E-03	super_enhancer	promoter	RP4-704D21.2
chr1	16155000	chr1	150785000	3.31E-03	super_enhancer	promoter	RNU6-1309P
chr1	16155000	chr1	157895000	3.49E-03	super_enhancer	promoter	RP11-451O13.1
chr1	16155000	chr1	210405000	2.28E-03	super_enhancer	promoter	SERTAD4
chr1	16165000	chr1	16315000	1.62E-02	super_enhancer	promoter	RP11-5P18.3
chr1	16165000	chr1	16345000	3.71E-02	super_enhancer	promoter	CLCNKA
chr1	16165000	chr1	43355000	1.02E-02	super_enhancer	promoter	RP11-342M1.7
chr1	16165000	chr1	113465000	2.95E-03	super_enhancer	promoter	AKR7A2P1
chr1	16165000	chr1	156025000	3.29E-03	super_enhancer	promoter	UBQLN4
chr1	16165000	chr1	160255000	2.35E-03	super_enhancer	promoter	RP11-574F21.3
chr1	16165000	chr1	203765000	2.87E-03	super_enhancer	promoter	ZC3H11A

chr1	16165000	chr1	207145000	3.01E-03	super_enhancer	promoter	FCAMR
chr1	16175000	chr1	20915000	3.11E-02	super_enhancer	promoter	CDA
chr1	16175000	chr1	25105000	1.79E-02	super_enhancer	promoter	RNU6-1208P
chr1	16175000	chr1	32395000	8.88E-03	super_enhancer	promoter	AL136115.1
chr1	16175000	chr1	35245000	1.07E-02	super_enhancer	promoter	AL121988.1
chr1	16175000	chr1	37945000	1.01E-02	super_enhancer	promoter	MIR6732
chr1	16175000	chr1	144945000	3.21E-03	super_enhancer	promoter	RNU2-38P
chr1	16175000	chr1	200285000	2.14E-03	super_enhancer	promoter	RP11-532L16.3
chr1	16175000	chr1	234795000	2.72E-03	super_enhancer	promoter	RP4-781K5.5
chr1	16185000	chr1	16295000	1.50E-02	super_enhancer	promoter	RP11-5P18.1
chr1	16185000	chr1	26645000	2.01E-02	super_enhancer	promoter	UBXN11
chr1	16185000	chr1	39425000	1.15E-02	super_enhancer	promoter	EIF1P2
chr1	16185000	chr1	46805000	7.95E-03	super_enhancer	promoter	NSUN4
chr1	16185000	chr1	53175000	8.47E-03	super_enhancer	promoter	NDUFS5P3
chr1	16185000	chr1	155185000	2.82E-03	super_enhancer	promoter	RP11-263K19.6
chr1	16185000	chr1	228675000	2.94E-03	super_enhancer	promoter	RP5-915N17.11
chr1	16185000	chr1	231475000	2.92E-03	super_enhancer	promoter	EXOC8
chr1	16195000	chr1	16315000	1.88E-02	super_enhancer	promoter	RP11-5P18.3
chr1	16195000	chr1	25665000	1.31E-02	super_enhancer	promoter	RSRP1
chr1	16195000	chr1	28975000	1.41E-02	super_enhancer	promoter	RNU11
chr1	16195000	chr1	44435000	1.06E-02	super_enhancer	promoter	DPH2
chr1	16195000	chr1	51795000	7.18E-03	super_enhancer	promoter	TTC39A-AS1
chr1	16195000	chr1	52325000	6.73E-03	super_enhancer	promoter	TSEN15P2
chr1	16195000	chr1	56675000	4.70E-03	super_enhancer	promoter	RP4-710M16.1

chr1	16195000	chr1	154525000	2.60E-03	super_enhancer	promoter	UBE2Q1-AS1
chr1	16195000	chr1	222015000	2.08E-03	super_enhancer	promoter	RP11-191N8.2
chr1	16195000	chr1	225965000	2.45E-03	super_enhancer	promoter	SRP9
chr1	16205000	chr1	20915000	3.21E-02	super_enhancer	promoter	CDA
chr1	16205000	chr1	28065000	1.51E-02	super_enhancer	promoter	RP3-426I6.2
chr1	16205000	chr1	37795000	8.66E-03	super_enhancer	promoter	RPS29P6
chr1	16205000	chr1	228675000	2.65E-03	super_enhancer	promoter	RP5-915N17.11
chr1	16245000	chr1	21635000	3.60E-02	promoter	super_enhancer	RP1-134O19.3
chr1	16245000	chr1	85305000	2.69E-03	promoter	super_enhancer	RP1-134O19.3
chr1	16245000	chr1	202085000	3.57E-03	promoter	super_enhancer	RP1-134O19.3
chr1	16245000	chr1	230115000	2.74E-03	promoter	super_enhancer	RP1-134O19.3
chr1	16295000	chr1	31585000	1.49E-02	promoter	super_enhancer	RP11-5P18.1
chr1	16295000	chr1	172925000	2.39E-03	promoter	super_enhancer	RP11-5P18.1
chr1	16315000	chr1	33045000	1.52E-02	promoter	super_enhancer	RP11-5P18.3
chr1	16315000	chr1	36175000	1.80E-02	promoter	super_enhancer	RP11-5P18.3
chr1	16315000	chr1	41515000	1.07E-02	promoter	super_enhancer	RP11-5P18.3
chr1	16315000	chr1	42945000	1.17E-02	promoter	super_enhancer	RP11-5P18.3
chr1	16315000	chr1	204295000	3.78E-03	promoter	super_enhancer	RP11-5P18.3
chr1	16485000	chr1	23185000	3.31E-02	promoter	super_enhancer	RP11-276H7.3
chr1	16485000	chr1	23205000	4.59E-02	promoter	super_enhancer	RP11-276H7.3
chr1	16485000	chr1	27495000	2.78E-02	promoter	super_enhancer	RP11-276H7.3
chr1	16485000	chr1	27515000	2.32E-02	promoter	super_enhancer	RP11-276H7.3
chr1	16485000	chr1	43425000	1.48E-02	promoter	super_enhancer	RP11-276H7.3
chr1	16485000	chr1	55425000	7.88E-03	promoter	super_enhancer	RP11-276H7.3

chr1	16485000	chr1	224255000	4.12E-03	promoter	super_enhancer	RP11-276H7.3
chr1	16485000	chr1	234715000	3.12E-03	promoter	super_enhancer	RP11-276H7.3
chr1	16555000	chr1	26865000	2.09E-02	promoter	super_enhancer	ANO7P1
chr1	16555000	chr1	27525000	1.99E-02	promoter	super_enhancer	ANO7P1
chr1	16555000	chr1	33035000	1.43E-02	promoter	super_enhancer	ANO7P1
chr1	16555000	chr1	202025000	3.43E-03	promoter	super_enhancer	ANO7P1
chr1	16565000	chr1	26855000	2.13E-02	promoter	super_enhancer	RSG1
chr1	16565000	chr1	40965000	1.17E-02	promoter	super_enhancer	RSG1
chr1	16565000	chr1	55435000	7.87E-03	promoter	super_enhancer	RSG1
chr1	16565000	chr1	202065000	3.52E-03	promoter	super_enhancer	RSG1
chr1	16735000	chr1	27585000	2.21E-02	promoter	super_enhancer	AL358794.1
chr1	16735000	chr1	181105000	3.58E-03	promoter	super_enhancer	AL358794.1
chr1	16735000	chr1	204305000	3.44E-03	promoter	super_enhancer	AL358794.1
chr1	16735000	chr1	234965000	3.26E-03	promoter	super_enhancer	AL358794.1
chr1	16765000	chr1	32715000	1.64E-02	promoter	super_enhancer	SPATA21
chr1	16765000	chr1	40875000	9.43E-03	promoter	super_enhancer	SPATA21
chr1	16865000	chr1	21625000	3.53E-02	promoter	super_enhancer	FAM231C
chr1	16865000	chr1	31675000	1.54E-02	promoter	super_enhancer	FAM231C
chr1	16865000	chr1	43475000	9.76E-03	promoter	super_enhancer	FAM231C
chr1	16865000	chr1	85295000	3.06E-03	promoter	super_enhancer	FAM231C
chr1	17215000	chr1	26295000	3.27E-02	promoter	super_enhancer	RP11-108M9.5
chr1	17215000	chr1	26335000	3.31E-02	promoter	super_enhancer	RP11-108M9.5
chr1	17215000	chr1	26855000	3.04E-02	promoter	super_enhancer	RP11-108M9.5
chr1	17215000	chr1	27515000	2.84E-02	promoter	super_enhancer	RP11-108M9.5

chr1	17215000	chr1	40895000	1.63E-02	promoter	super_enhancer	RP11-108M9.5
chr1	17215000	chr1	41515000	1.58E-02	promoter	super_enhancer	RP11-108M9.5
chr1	17215000	chr1	43445000	1.83E-02	promoter	super_enhancer	RP11-108M9.5
chr1	17215000	chr1	204275000	4.72E-03	promoter	super_enhancer	RP11-108M9.5
chr1	17215000	chr1	235905000	4.14E-03	promoter	super_enhancer	RP11-108M9.5
chr1	17305000	chr1	21935000	3.23E-02	promoter	super_enhancer	RP1-37C10.3
chr1	17305000	chr1	26305000	2.00E-02	promoter	super_enhancer	RP1-37C10.3
chr1	17305000	chr1	26695000	2.21E-02	promoter	super_enhancer	RP1-37C10.3
chr1	17305000	chr1	40905000	9.52E-03	promoter	super_enhancer	RP1-37C10.3
chr1	17515000	chr1	23195000	2.90E-02	promoter	super_enhancer	RP11-380J14.1
chr1	17515000	chr1	26865000	2.10E-02	promoter	super_enhancer	RP11-380J14.1
chr1	17515000	chr1	27535000	1.87E-02	promoter	super_enhancer	RP11-380J14.1
chr1	17515000	chr1	43235000	1.00E-02	promoter	super_enhancer	RP11-380J14.1
chr1	17515000	chr1	172895000	2.08E-03	promoter	super_enhancer	RP11-380J14.1
chr1	17515000	chr1	235005000	2.96E-03	promoter	super_enhancer	RP11-380J14.1
chr1	17575000	chr1	31945000	1.26E-02	promoter	super_enhancer	PADI3
chr1	17575000	chr1	36165000	1.44E-02	promoter	super_enhancer	PADI3
chr1	17575000	chr1	41895000	1.21E-02	promoter	super_enhancer	PADI3
chr1	17575000	chr1	204115000	3.56E-03	promoter	super_enhancer	PADI3
chr1	17635000	chr1	26705000	2.19E-02	promoter	super_enhancer	PADI4
chr1	17635000	chr1	43455000	1.29E-02	promoter	super_enhancer	PADI4
chr1	17635000	chr1	204105000	4.01E-03	promoter	super_enhancer	PADI4
chr1	17635000	chr1	230105000	2.49E-03	promoter	super_enhancer	PADI4
chr1	17865000	chr1	27515000	1.99E-02	promoter	super_enhancer	ARHGEF10L

chr1	17865000	chr1	31925000	1.41E-02	promoter	super_enhancer	ARHGEF10L
chr1	17865000	chr1	205285000	3.34E-03	promoter	super_enhancer	ARHGEF10L
chr1	17865000	chr1	205305000	4.19E-03	promoter	super_enhancer	ARHGEF10L
chr1	18435000	chr1	43505000	1.51E-02	promoter	super_enhancer	RP11-174G17.3
chr1	18915000	chr1	25535000	2.95E-02	super_enhancer	promoter	RP4-706G24.1
chr1	18915000	chr1	38475000	1.17E-02	super_enhancer	promoter	UTP11L
chr1	18915000	chr1	93545000	2.51E-03	super_enhancer	promoter	MTF2
chr1	18925000	chr1	19935000	4.88E-02	super_enhancer	promoter	RP5-1056L3.3
chr1	18925000	chr1	20355000	1.14E-02	super_enhancer	promoter	PLA2G5
chr1	18925000	chr1	35525000	1.09E-02	super_enhancer	promoter	ZMYM1
chr1	18925000	chr1	36205000	1.34E-02	super_enhancer	promoter	RP11-435D7.3
chr1	18925000	chr1	43355000	1.17E-02	super_enhancer	promoter	RP11-342M1.7
chr1	18925000	chr1	88275000	2.57E-03	super_enhancer	promoter	RP11-60A14.1
chr1	18925000	chr1	150945000	2.71E-03	super_enhancer	promoter	RP11-316M1.3
chr1	18925000	chr1	228755000	1.62E-03	super_enhancer	promoter	RNA5S5
chr1	18935000	chr1	26495000	2.27E-02	super_enhancer	promoter	ZNF593
chr1	18935000	chr1	26645000	1.92E-02	super_enhancer	promoter	UBXN11
chr1	18935000	chr1	28435000	1.65E-02	super_enhancer	promoter	RP5-1053E7.2
chr1	18935000	chr1	35135000	9.38E-03	super_enhancer	promoter	MIR552
chr1	18935000	chr1	47135000	7.83E-03	super_enhancer	promoter	TEX38
chr1	18935000	chr1	155905000	2.40E-03	super_enhancer	promoter	KIAA0907
chr1	18945000	chr1	28855000	1.99E-02	super_enhancer	promoter	PRDX3P2
chr1	18945000	chr1	221725000	2.18E-03	super_enhancer	promoter	RP11-103C3.2
chr1	18945000	chr1	237145000	3.19E-03	super_enhancer	promoter	RPL35P1

chr1	19225000	chr1	212505000	2.96E-03	promoter	super_enhancer	MIR1290
chr1	19225000	chr1	235075000	3.99E-03	promoter	super_enhancer	MIR1290
chr1	19935000	chr1	21335000	1.70E-02	promoter	super_enhancer	RP5-1056L3.3
chr1	19935000	chr1	26875000	3.05E-02	promoter	super_enhancer	RP5-1056L3.3
chr1	19935000	chr1	40985000	1.01E-02	promoter	super_enhancer	RP5-1056L3.3
chr1	19935000	chr1	235905000	2.44E-03	promoter	super_enhancer	RP5-1056L3.3
chr1	20355000	chr1	235905000	1.80E-03	promoter	super_enhancer	PLA2G5
chr1	20465000	chr1	155135000	3.64E-03	promoter	super_enhancer	PLA2G2F
chr1	20465000	chr1	202595000	2.62E-03	promoter	super_enhancer	PLA2G2F
chr1	20505000	chr1	21315000	3.98E-02	promoter	super_enhancer	PLA2G2C
chr1	20505000	chr1	183275000	1.84E-03	promoter	super_enhancer	PLA2G2C
chr1	20835000	chr1	26285000	3.14E-02	promoter	super_enhancer	MUL1
chr1	20835000	chr1	32995000	1.84E-02	promoter	super_enhancer	MUL1
chr1	20915000	chr1	21285000	3.84E-02	promoter	super_enhancer	CDA
chr1	20915000	chr1	67685000	4.49E-03	promoter	super_enhancer	CDA
chr1	21045000	chr1	26285000	2.96E-02	promoter	super_enhancer	KIF17
chr1	21045000	chr1	153565000	2.67E-03	promoter	super_enhancer	KIF17
chr1	21115000	chr1	32735000	1.70E-02	promoter	super_enhancer	HP1BP3
chr1	21115000	chr1	40985000	9.71E-03	promoter	super_enhancer	HP1BP3
chr1	21115000	chr1	152045000	2.70E-03	promoter	super_enhancer	HP1BP3
chr1	21115000	chr1	204285000	2.31E-03	promoter	super_enhancer	HP1BP3
chr1	21265000	chr1	28435000	2.29E-02	super_enhancer	promoter	RP5-1053E7.2
chr1	21265000	chr1	28835000	1.96E-02	super_enhancer	promoter	SNORA73B
chr1	21275000	chr1	36395000	9.36E-03	super_enhancer	promoter	AGO3

chr1	21275000	chr1	39325000	1.03E-02	super_enhancer	promoter	RRAGC
chr1	21275000	chr1	52005000	7.27E-03	super_enhancer	promoter	RNU6-1281P
chr1	21275000	chr1	192765000	1.92E-03	super_enhancer	promoter	RP3-419C19.3
chr1	21275000	chr1	232835000	2.32E-03	super_enhancer	promoter	RNU6-1211P
chr1	21285000	chr1	24515000	4.79E-02	super_enhancer	promoter	IFNLR1
chr1	21295000	chr1	26605000	3.12E-02	super_enhancer	promoter	SH3BGRL3
chr1	21295000	chr1	33445000	1.75E-02	super_enhancer	promoter	RP4-803A2.1
chr1	21295000	chr1	35975000	1.11E-02	super_enhancer	promoter	RP4-728D4.3
chr1	21295000	chr1	150875000	2.56E-03	super_enhancer	promoter	CYCSP51
chr1	21305000	chr1	32665000	1.34E-02	super_enhancer	promoter	CCDC28B
chr1	21305000	chr1	92415000	2.26E-03	super_enhancer	promoter	BRDT
chr1	21305000	chr1	149215000	1.66E-03	super_enhancer	promoter	RNU1-92P
chr1	21315000	chr1	27715000	3.43E-02	super_enhancer	promoter	RP1-144C9.2
chr1	21315000	chr1	28015000	2.42E-02	super_enhancer	promoter	CHMP1AP1
chr1	21315000	chr1	28285000	2.76E-02	super_enhancer	promoter	XKR8
chr1	21315000	chr1	38275000	1.25E-02	super_enhancer	promoter	YRDC
chr1	21315000	chr1	62785000	6.07E-03	super_enhancer	promoter	KANK4
chr1	21315000	chr1	184985000	2.90E-03	super_enhancer	promoter	RNA5SP72
chr1	21315000	chr1	232835000	2.63E-03	super_enhancer	promoter	RNU6-1211P
chr1	21325000	chr1	94345000	2.65E-03	super_enhancer	promoter	DNTTIP2
chr1	21325000	chr1	240935000	1.79E-03	super_enhancer	promoter	PRKRIRP8
chr1	21335000	chr1	26185000	3.28E-02	super_enhancer	promoter	RP1-125I3.2
chr1	21335000	chr1	65765000	4.88E-03	super_enhancer	promoter	COX6CP13
chr1	21335000	chr1	93745000	2.47E-03	super_enhancer	promoter	RP4-717I23.2

chr1	21335000	chr1	166765000	1.91E-03	super_enhancer	promoter	CNN2P10
chr1	21335000	chr1	224955000	3.02E-03	super_enhancer	promoter	RP11-449J1.1
chr1	21335000	chr1	244015000	2.06E-03	super_enhancer	promoter	AKT3
chr1	21605000	chr1	26485000	4.66E-02	super_enhancer	promoter	FAM110D
chr1	21605000	chr1	27995000	4.24E-02	super_enhancer	promoter	RP11-288L9.4
chr1	21605000	chr1	112015000	3.21E-03	super_enhancer	promoter	C1orf162
chr1	21605000	chr1	151735000	2.91E-03	super_enhancer	promoter	OAZ3
chr1	21605000	chr1	155035000	3.42E-03	super_enhancer	promoter	EFNA4
chr1	21615000	chr1	26395000	3.14E-02	super_enhancer	promoter	TRIM63
chr1	21615000	chr1	44585000	1.20E-02	super_enhancer	promoter	KLF17
chr1	21615000	chr1	46805000	9.57E-03	super_enhancer	promoter	NSUN4
chr1	21615000	chr1	47965000	9.75E-03	super_enhancer	promoter	RPL21P24
chr1	21615000	chr1	53165000	8.03E-03	super_enhancer	promoter	COA7
chr1	21625000	chr1	29205000	3.82E-04	super_enhancer	promoter	RP1-212P9.3
chr1	21625000	chr1	87595000	4.20E-03	super_enhancer	promoter	LINC01140
chr1	21625000	chr1	117285000	3.46E-03	super_enhancer	promoter	NEFHP1
chr1	21625000	chr1	150945000	2.72E-03	super_enhancer	promoter	RP11-316M1.3
chr1	21625000	chr1	151105000	3.13E-03	super_enhancer	promoter	RP11-68I18.10
chr1	21625000	chr1	228585000	1.97E-03	super_enhancer	promoter	MIR6742
chr1	21635000	chr1	22265000	3.11E-02	super_enhancer	promoter	HSPG2
chr1	21635000	chr1	23345000	7.92E-03	super_enhancer	promoter	KDM1A
chr1	21635000	chr1	23505000	1.08E-02	super_enhancer	promoter	LUZP1
chr1	21635000	chr1	25595000	3.68E-02	super_enhancer	promoter	RP3-465N24.5
chr1	21635000	chr1	32675000	2.37E-02	super_enhancer	promoter	DCDC2B

chr1	21635000	chr1	33005000	1.98E-02	super_enhancer	promoter	ZBTB8A
chr1	21635000	chr1	46915000	1.70E-02	super_enhancer	promoter	LINC01398
chr1	21635000	chr1	156265000	3.57E-03	super_enhancer	promoter	GLMP
chr1	21635000	chr1	201965000	3.92E-03	super_enhancer	promoter	RP11-465N4.5
chr1	21635000	chr1	226335000	3.35E-03	super_enhancer	promoter	RP11-275I14.4
chr1	21635000	chr1	230185000	3.64E-03	super_enhancer	promoter	BX323860.1
chr1	21835000	chr1	23585000	9.05E-03	promoter	super_enhancer	ALPL
chr1	21835000	chr1	32715000	1.82E-02	promoter	super_enhancer	ALPL
chr1	21835000	chr1	40905000	1.16E-02	promoter	super_enhancer	ALPL
chr1	21835000	chr1	41895000	1.30E-02	promoter	super_enhancer	ALPL
chr1	21925000	chr1	26185000	4.66E-02	super_enhancer	promoter	RP1-125I3.2
chr1	21925000	chr1	28285000	3.55E-02	super_enhancer	promoter	XKR8
chr1	21925000	chr1	32665000	2.30E-02	super_enhancer	promoter	CCDC28B
chr1	21925000	chr1	35815000	1.61E-02	super_enhancer	promoter	RPL5P4
chr1	21925000	chr1	165645000	3.41E-03	super_enhancer	promoter	RP11-466F5.4
chr1	21925000	chr1	192765000	2.57E-03	super_enhancer	promoter	RP3-419C19.3
chr1	21925000	chr1	227895000	2.46E-03	super_enhancer	promoter	ZNF847P
chr1	21935000	chr1	53925000	8.91E-03	super_enhancer	promoter	DMRTB1
chr1	21935000	chr1	150875000	2.77E-03	super_enhancer	promoter	CYCSP51
chr1	22095000	chr1	26285000	4.60E-02	promoter	super_enhancer	RP11-132G19.3
chr1	22095000	chr1	43525000	1.26E-02	promoter	super_enhancer	RP11-132G19.3
chr1	22095000	chr1	207005000	3.33E-03	promoter	super_enhancer	RP11-132G19.3
chr1	22235000	chr1	27565000	2.44E-02	promoter	super_enhancer	RP11-26H16.1
chr1	22235000	chr1	203515000	2.21E-03	promoter	super_enhancer	RP11-26H16.1

chr1	22265000	chr1	26285000	4.12E-02	promoter	super_enhancer	HSPG2
chr1	22265000	chr1	27585000	3.27E-02	promoter	super_enhancer	HSPG2
chr1	22265000	chr1	230115000	2.70E-03	promoter	super_enhancer	HSPG2
chr1	22385000	chr1	43545000	1.24E-02	promoter	super_enhancer	CDC42-IT1
chr1	22385000	chr1	55435000	7.74E-03	promoter	super_enhancer	CDC42-IT1
chr1	22385000	chr1	234615000	3.06E-03	promoter	super_enhancer	CDC42-IT1
chr1	22395000	chr1	26295000	4.88E-02	promoter	super_enhancer	RP1-224A6.8
chr1	22395000	chr1	27595000	3.50E-02	promoter	super_enhancer	RP1-224A6.8
chr1	23045000	chr1	27575000	4.68E-02	promoter	super_enhancer	MIR4684
chr1	23045000	chr1	212575000	3.29E-03	promoter	super_enhancer	MIR4684
chr1	23045000	chr1	223705000	2.61E-03	promoter	super_enhancer	MIR4684
chr1	23075000	chr1	23195000	1.49E-02	promoter	super_enhancer	AL611946.1
chr1	23075000	chr1	27555000	8.70E-04	promoter	super_enhancer	AL611946.1
chr1	23075000	chr1	41495000	1.62E-02	promoter	super_enhancer	AL611946.1
chr1	23075000	chr1	42975000	1.22E-02	promoter	super_enhancer	AL611946.1
chr1	23075000	chr1	155005000	2.95E-03	promoter	super_enhancer	AL611946.1
chr1	23185000	chr1	45265000	1.15E-02	super_enhancer	promoter	PLK3
chr1	23185000	chr1	189745000	1.60E-03	super_enhancer	promoter	RP11-398M15.1
chr1	23185000	chr1	219585000	2.22E-03	super_enhancer	promoter	RP11-392O17.1
chr1	23195000	chr1	42845000	1.50E-02	super_enhancer	promoter	RIMKLA
chr1	23195000	chr1	112905000	3.06E-03	super_enhancer	promoter	TXNP3
chr1	23195000	chr1	120355000	2.49E-03	super_enhancer	promoter	REG4
chr1	23195000	chr1	232295000	2.06E-03	super_enhancer	promoter	RP5-865N13.2
chr1	23205000	chr1	53925000	1.31E-02	super_enhancer	promoter	DMRTB1

chr1	23205000	chr1	153725000	3.73E-03	super_enhancer	promoter	RP11-216N14.8
chr1	23205000	chr1	204595000	4.42E-03	super_enhancer	promoter	RP11-430C7.5
chr1	23205000	chr1	240715000	3.98E-03	super_enhancer	promoter	RP11-467I20.4
chr1	23215000	chr1	29475000	3.84E-02	super_enhancer	promoter	RP11-242O24.3
chr1	23215000	chr1	32225000	3.15E-02	super_enhancer	promoter	MIR4254
chr1	23215000	chr1	109255000	3.42E-03	super_enhancer	promoter	FNDC7
chr1	23215000	chr1	229645000	3.82E-03	super_enhancer	promoter	NUP133
chr1	23225000	chr1	56405000	6.97E-03	super_enhancer	promoter	PIGQP1
chr1	23225000	chr1	100565000	2.66E-03	super_enhancer	promoter	RP4-714D9.4
chr1	23225000	chr1	111415000	2.35E-03	super_enhancer	promoter	CD53
chr1	23225000	chr1	154525000	2.90E-03	super_enhancer	promoter	UBE2Q1-AS1
chr1	23225000	chr1	173985000	2.00E-03	super_enhancer	promoter	RC3H1-IT1
chr1	23225000	chr1	244615000	2.79E-03	super_enhancer	promoter	ADSS
chr1	23345000	chr1	23565000	1.31E-02	promoter	super_enhancer	KDM1A
chr1	23345000	chr1	40855000	8.07E-03	promoter	super_enhancer	KDM1A
chr1	23345000	chr1	202035000	2.28E-03	promoter	super_enhancer	KDM1A
chr1	23345000	chr1	212485000	2.07E-03	promoter	super_enhancer	KDM1A
chr1	23385000	chr1	36145000	1.67E-02	promoter	super_enhancer	MIR4419A
chr1	23385000	chr1	42945000	1.17E-02	promoter	super_enhancer	MIR4419A
chr1	23385000	chr1	43425000	1.23E-02	promoter	super_enhancer	MIR4419A
chr1	23385000	chr1	228145000	3.28E-03	promoter	super_enhancer	MIR4419A
chr1	23415000	chr1	23575000	8.48E-03	promoter	super_enhancer	RP1-184J9.2
chr1	23415000	chr1	23595000	2.53E-02	promoter	super_enhancer	RP1-184J9.2
chr1	23415000	chr1	31675000	2.73E-02	promoter	super_enhancer	RP1-184J9.2

chr1	23415000	chr1	31685000	3.08E-02	promoter	super_enhancer	RP1-184J9.2
chr1	23415000	chr1	42945000	1.54E-02	promoter	super_enhancer	RP1-184J9.2
chr1	23415000	chr1	152045000	3.55E-03	promoter	super_enhancer	RP1-184J9.2
chr1	23505000	chr1	23555000	1.96E-04	promoter	super_enhancer	LUZP1
chr1	23505000	chr1	31695000	2.39E-02	promoter	super_enhancer	LUZP1
chr1	23505000	chr1	235005000	2.76E-03	promoter	super_enhancer	LUZP1
chr1	23555000	chr1	28905000	3.40E-02	super_enhancer	promoter	SNORD99
chr1	23555000	chr1	28995000	4.02E-02	super_enhancer	promoter	GMEB1
chr1	23555000	chr1	32675000	2.41E-02	super_enhancer	promoter	DCDC2B
chr1	23555000	chr1	162795000	2.52E-03	super_enhancer	promoter	RP11-331H2.4
chr1	23555000	chr1	192765000	2.49E-03	super_enhancer	promoter	RP3-419C19.3
chr1	23565000	chr1	32225000	2.35E-02	super_enhancer	promoter	MIR4254
chr1	23565000	chr1	44115000	1.03E-02	super_enhancer	promoter	KDM4A
chr1	23565000	chr1	46015000	1.27E-02	super_enhancer	promoter	AKR1A1
chr1	23565000	chr1	247095000	2.80E-03	super_enhancer	promoter	AHCTF1
chr1	23575000	chr1	26795000	4.16E-02	super_enhancer	promoter	RP3-476K8.3
chr1	23575000	chr1	27975000	3.77E-02	super_enhancer	promoter	RP1-159A19.3
chr1	23575000	chr1	29475000	3.58E-02	super_enhancer	promoter	RP11-242O24.3
chr1	23575000	chr1	54305000	1.08E-02	super_enhancer	promoter	NDC1
chr1	23585000	chr1	41445000	1.65E-02	super_enhancer	promoter	CTPS1
chr1	23585000	chr1	47965000	1.02E-02	super_enhancer	promoter	RPL21P24
chr1	23585000	chr1	104065000	1.75E-03	super_enhancer	promoter	RN7SKP285
chr1	23585000	chr1	229705000	2.67E-03	super_enhancer	promoter	HMGN2P19
chr1	23595000	chr1	24335000	1.77E-02	super_enhancer	promoter	RP11-4M23.7

chr1	23595000	chr1	68945000	5.09E-03	super_enhancer	promoter	RP4-694A7.2
chr1	23595000	chr1	238025000	2.62E-03	super_enhancer	promoter	RP11-193H5.1
chr1	23695000	chr1	27565000	4.25E-02	promoter	super_enhancer	ZNF436-AS1
chr1	23695000	chr1	181085000	2.74E-03	promoter	super_enhancer	ZNF436-AS1
chr1	24105000	chr1	43455000	1.42E-02	promoter	super_enhancer	TCEB3-AS1
chr1	24165000	chr1	31705000	1.99E-02	promoter	super_enhancer	HMGCL
chr1	24195000	chr1	42965000	7.72E-03	promoter	super_enhancer	FUCA1
chr1	24195000	chr1	202065000	2.49E-03	promoter	super_enhancer	FUCA1
chr1	24285000	chr1	32725000	2.06E-02	promoter	super_enhancer	PNRC2
chr1	24285000	chr1	41505000	1.03E-02	promoter	super_enhancer	PNRC2
chr1	24285000	chr1	43215000	1.37E-02	promoter	super_enhancer	PNRC2
chr1	24285000	chr1	43445000	1.27E-02	promoter	super_enhancer	PNRC2
chr1	24335000	chr1	43445000	1.29E-02	promoter	super_enhancer	RP11-4M23.7
chr1	24335000	chr1	151195000	2.75E-03	promoter	super_enhancer	RP11-4M23.7
chr1	24515000	chr1	26295000	6.19E-04	promoter	super_enhancer	IFNLR1
chr1	24515000	chr1	40895000	1.40E-02	promoter	super_enhancer	IFNLR1
chr1	24515000	chr1	85335000	5.00E-03	promoter	super_enhancer	IFNLR1
chr1	24645000	chr1	32725000	3.12E-02	promoter	super_enhancer	GRHL3
chr1	24645000	chr1	42965000	1.20E-02	promoter	super_enhancer	GRHL3
chr1	24645000	chr1	43545000	1.77E-02	promoter	super_enhancer	GRHL3
chr1	24645000	chr1	94635000	4.03E-03	promoter	super_enhancer	GRHL3
chr1	24645000	chr1	109905000	3.51E-03	promoter	super_enhancer	GRHL3
chr1	24645000	chr1	204275000	3.69E-03	promoter	super_enhancer	GRHL3
chr1	24645000	chr1	205325000	4.58E-03	promoter	super_enhancer	GRHL3

chr1	25105000	chr1	39195000	1.16E-02	promoter	super_enhancer	RNU6-1208P
chr1	25105000	chr1	235075000	3.69E-03	promoter	super_enhancer	RNU6-1208P
chr1	25545000	chr1	26145000	3.51E-02	super_enhancer	promoter	MTFR1L
chr1	25545000	chr1	47875000	1.15E-02	super_enhancer	promoter	LINC01389
chr1	25545000	chr1	110285000	2.56E-03	super_enhancer	promoter	GSTM3
chr1	25545000	chr1	188875000	1.81E-03	super_enhancer	promoter	LINC01035
chr1	25555000	chr1	32675000	2.70E-02	super_enhancer	promoter	DCDC2B
chr1	25555000	chr1	44555000	1.53E-02	super_enhancer	promoter	RP5-1198O20.5
chr1	25565000	chr1	53385000	1.08E-02	super_enhancer	promoter	RP4-631H13.6
chr1	25565000	chr1	118185000	2.43E-03	super_enhancer	promoter	AL365331.2
chr1	25565000	chr1	120355000	2.58E-03	super_enhancer	promoter	REG4
chr1	25565000	chr1	153765000	2.84E-03	super_enhancer	promoter	RP11-216N14.5
chr1	25595000	chr1	32755000	1.88E-02	promoter	super_enhancer	RP3-465N24.5
chr1	25595000	chr1	43545000	9.75E-03	promoter	super_enhancer	RP3-465N24.5
chr1	25595000	chr1	235045000	1.82E-03	promoter	super_enhancer	RP3-465N24.5
chr1	25665000	chr1	26715000	3.57E-02	promoter	super_enhancer	RSRP1
chr1	25665000	chr1	32995000	1.90E-02	promoter	super_enhancer	RSRP1
chr1	25665000	chr1	204085000	2.65E-03	promoter	super_enhancer	RSRP1
chr1	25665000	chr1	205325000	2.67E-03	promoter	super_enhancer	RSRP1
chr1	25665000	chr1	207025000	1.98E-03	promoter	super_enhancer	RSRP1
chr1	25725000	chr1	26285000	4.14E-02	promoter	super_enhancer	RP3-469D22.1
chr1	26145000	chr1	41505000	1.39E-02	promoter	super_enhancer	MTFR1L
chr1	26145000	chr1	225465000	2.84E-03	promoter	super_enhancer	MTFR1L
chr1	26185000	chr1	26345000	1.87E-02	promoter	super_enhancer	RP1-125I3.2

chr1	26185000	chr1	202085000	3.76E-03	promoter	super_enhancer	RP1-125I3.2
chr1	26185000	chr1	230135000	2.80E-03	promoter	super_enhancer	RP1-125I3.2
chr1	26215000	chr1	26355000	4.48E-02	promoter	super_enhancer	RP1-125I3.4
chr1	26215000	chr1	33095000	2.64E-02	promoter	super_enhancer	RP1-125I3.4
chr1	26215000	chr1	202055000	3.12E-03	promoter	super_enhancer	RP1-125I3.4
chr1	26275000	chr1	26605000	4.35E-02	super_enhancer	promoter	SH3BGRL3
chr1	26275000	chr1	32395000	2.25E-02	super_enhancer	promoter	AL136115.1
chr1	26275000	chr1	33285000	2.27E-02	super_enhancer	promoter	YARS
chr1	26275000	chr1	41405000	1.89E-02	super_enhancer	promoter	RP11-348A7.1
chr1	26275000	chr1	150265000	3.38E-03	super_enhancer	promoter	MRPS21
chr1	26275000	chr1	205055000	2.98E-03	super_enhancer	promoter	TMEM81
chr1	26275000	chr1	220265000	3.14E-03	super_enhancer	promoter	BPNT1
chr1	26275000	chr1	234745000	3.07E-03	super_enhancer	promoter	IRF2BP2
chr1	26275000	chr1	240755000	2.56E-03	super_enhancer	promoter	RP11-467I20.6
chr1	26275000	chr1	248285000	2.10E-03	super_enhancer	promoter	OR2M1P
chr1	26285000	chr1	35735000	1.78E-02	super_enhancer	promoter	ZMYM4
chr1	26285000	chr1	44175000	1.53E-02	super_enhancer	promoter	KDM4A-AS1
chr1	26285000	chr1	155905000	3.12E-03	super_enhancer	promoter	KIAA0907
chr1	26285000	chr1	161375000	2.33E-03	super_enhancer	promoter	RP11-122G18.7
chr1	26285000	chr1	187685000	1.86E-03	super_enhancer	promoter	RP5-925F19.1
chr1	26285000	chr1	206715000	3.27E-03	super_enhancer	promoter	CH17-76K2.6
chr1	26285000	chr1	220535000	3.05E-03	super_enhancer	promoter	RP11-302I18.3
chr1	26295000	chr1	32665000	3.02E-02	super_enhancer	promoter	CCDC28B
chr1	26295000	chr1	42225000	1.37E-02	super_enhancer	promoter	AL451006.1

chr1	26295000	chr1	145525000	2.56E-03	super_enhancer	promoter	ITGA10
chr1	26295000	chr1	235705000	2.53E-03	super_enhancer	promoter	MTND4P10
chr1	26305000	chr1	32395000	2.24E-02	super_enhancer	promoter	AL136115.1
chr1	26305000	chr1	42225000	1.47E-02	super_enhancer	promoter	AL451006.1
chr1	26305000	chr1	174965000	2.34E-03	super_enhancer	promoter	RNU6-307P
chr1	26305000	chr1	209405000	1.77E-03	super_enhancer	promoter	RP11-385M4.3
chr1	26305000	chr1	215115000	2.71E-03	super_enhancer	promoter	RP11-323K10.1
chr1	26315000	chr1	26495000	1.88E-02	super_enhancer	promoter	ZNF593
chr1	26315000	chr1	28015000	1.19E-02	super_enhancer	promoter	CHMP1AP1
chr1	26315000	chr1	32665000	3.03E-02	super_enhancer	promoter	CCDC28B
chr1	26315000	chr1	35525000	1.50E-02	super_enhancer	promoter	ZMYM1
chr1	26315000	chr1	36025000	2.31E-02	super_enhancer	promoter	KIAA0319L
chr1	26315000	chr1	45265000	1.38E-02	super_enhancer	promoter	PLK3
chr1	26315000	chr1	51985000	6.57E-03	super_enhancer	promoter	EPS15
chr1	26315000	chr1	53795000	9.11E-03	super_enhancer	promoter	LRP8
chr1	26315000	chr1	100715000	2.15E-03	super_enhancer	promoter	DBT
chr1	26315000	chr1	154245000	3.53E-03	super_enhancer	promoter	HAX1
chr1	26315000	chr1	175845000	2.66E-03	super_enhancer	promoter	RP11-222A5.1
chr1	26315000	chr1	202115000	3.85E-03	super_enhancer	promoter	ARL8A
chr1	26315000	chr1	203765000	2.92E-03	super_enhancer	promoter	ZC3H11A
chr1	26315000	chr1	204245000	3.27E-03	super_enhancer	promoter	RP11-203F10.5
chr1	26315000	chr1	228755000	1.57E-03	super_enhancer	promoter	RNA5S5
chr1	26315000	chr1	234745000	2.83E-03	super_enhancer	promoter	IRF2BP2
chr1	26325000	chr1	27495000	4.45E-03	promoter	super_enhancer	PAFAH2

chr1	26325000	chr1	43235000	1.44E-02	promoter	super_enhancer	PAFAH2
chr1	26325000	chr1	67735000	7.39E-03	promoter	super_enhancer	PAFAH2
chr1	26325000	chr1	230135000	2.82E-03	promoter	super_enhancer	PAFAH2
chr1	26335000	chr1	144875000	4.39E-03	super_enhancer	promoter	RP4-791M13.5
chr1	26335000	chr1	153515000	3.71E-03	super_enhancer	promoter	S100A5
chr1	26335000	chr1	155145000	3.49E-03	super_enhancer	promoter	TRIM46
chr1	26335000	chr1	155815000	3.14E-03	super_enhancer	promoter	RP11-101O6.2
chr1	26335000	chr1	167165000	2.54E-03	super_enhancer	promoter	LINC01363
chr1	26335000	chr1	211385000	3.12E-03	super_enhancer	promoter	AC092017.1
chr1	26345000	chr1	26795000	3.54E-03	super_enhancer	promoter	RP3-476K8.3
chr1	26345000	chr1	32255000	3.36E-02	super_enhancer	promoter	RP11-84A19.3
chr1	26345000	chr1	62785000	8.92E-03	super_enhancer	promoter	KANK4
chr1	26345000	chr1	153725000	3.46E-03	super_enhancer	promoter	RP11-216N14.8
chr1	26345000	chr1	182655000	2.93E-03	super_enhancer	promoter	RGS8
chr1	26345000	chr1	201475000	3.45E-03	super_enhancer	promoter	RP11-134G8.7
chr1	26355000	chr1	26485000	8.98E-04	super_enhancer	promoter	FAM110D
chr1	26355000	chr1	44855000	1.27E-02	super_enhancer	promoter	RNU6-369P
chr1	26355000	chr1	47135000	1.43E-02	super_enhancer	promoter	TEX38
chr1	26355000	chr1	161655000	2.14E-03	super_enhancer	promoter	RPL31P11
chr1	26355000	chr1	178695000	2.62E-03	super_enhancer	promoter	RP11-428K3.1
chr1	26355000	chr1	207935000	2.59E-03	super_enhancer	promoter	CDCA4P4
chr1	26395000	chr1	94325000	2.42E-03	promoter	super_enhancer	TRIM63
chr1	26395000	chr1	202045000	2.64E-03	promoter	super_enhancer	TRIM63
chr1	26485000	chr1	32765000	3.68E-02	promoter	super_enhancer	FAM110D

chr1	26485000	chr1	156465000	4.80E-03	promoter	super_enhancer	FAM110D
chr1	26495000	chr1	26715000	2.28E-02	promoter	super_enhancer	ZNF593
chr1	26495000	chr1	31705000	4.30E-02	promoter	super_enhancer	ZNF593
chr1	26495000	chr1	31935000	3.15E-02	promoter	super_enhancer	ZNF593
chr1	26495000	chr1	154965000	4.36E-03	promoter	super_enhancer	ZNF593
chr1	26535000	chr1	31935000	2.37E-02	promoter	super_enhancer	RP11-231P20.2
chr1	26535000	chr1	43425000	1.53E-02	promoter	super_enhancer	RP11-231P20.2
chr1	26605000	chr1	26715000	2.44E-03	promoter	super_enhancer	SH3BGRL3
chr1	26605000	chr1	36155000	2.13E-02	promoter	super_enhancer	SH3BGRL3
chr1	26605000	chr1	41515000	1.59E-02	promoter	super_enhancer	SH3BGRL3
chr1	26605000	chr1	202025000	3.77E-03	promoter	super_enhancer	SH3BGRL3
chr1	26645000	chr1	26715000	1.35E-03	promoter	super_enhancer	UBXN11
chr1	26645000	chr1	41915000	2.27E-02	promoter	super_enhancer	UBXN11
chr1	26645000	chr1	42925000	1.76E-02	promoter	super_enhancer	UBXN11
chr1	26645000	chr1	43425000	1.77E-02	promoter	super_enhancer	UBXN11
chr1	26645000	chr1	204295000	3.69E-03	promoter	super_enhancer	UBXN11
chr1	26695000	chr1	35735000	2.25E-02	super_enhancer	promoter	ZMYM4
chr1	26695000	chr1	43915000	1.83E-02	super_enhancer	promoter	SZT2-AS1
chr1	26695000	chr1	218065000	2.83E-03	super_enhancer	promoter	LINC00210
chr1	26705000	chr1	32255000	2.95E-02	super_enhancer	promoter	RP11-84A19.3
chr1	26705000	chr1	46155000	1.43E-02	super_enhancer	promoter	GPBP1L1
chr1	26705000	chr1	201475000	2.84E-03	super_enhancer	promoter	RP11-134G8.7
chr1	26715000	chr1	26795000	3.61E-02	super_enhancer	promoter	RP3-476K8.3
chr1	26715000	chr1	28905000	7.40E-03	super_enhancer	promoter	SNORD99

chr1	26715000	chr1	32225000	4.43E-02	super_enhancer	promoter	MIR4254
chr1	26715000	chr1	34325000	2.85E-02	super_enhancer	promoter	HMGB4
chr1	26715000	chr1	91965000	3.23E-03	super_enhancer	promoter	CDC7
chr1	26715000	chr1	146645000	3.16E-06	super_enhancer	promoter	PRKAB2
chr1	26715000	chr1	202765000	3.58E-03	super_enhancer	promoter	SLC25A39P1
chr1	26715000	chr1	244515000	3.25E-03	super_enhancer	promoter	C1orf100
chr1	26795000	chr1	32735000	1.92E-02	promoter	super_enhancer	RP3-476K8.3
chr1	26795000	chr1	32765000	1.99E-02	promoter	super_enhancer	RP3-476K8.3
chr1	26795000	chr1	43545000	9.15E-03	promoter	super_enhancer	RP3-476K8.3
chr1	26855000	chr1	28435000	1.72E-02	super_enhancer	promoter	RP5-1053E7.2
chr1	26855000	chr1	32395000	2.21E-02	super_enhancer	promoter	AL136115.1
chr1	26855000	chr1	41405000	1.73E-02	super_enhancer	promoter	RP11-348A7.1
chr1	26855000	chr1	41445000	1.94E-02	super_enhancer	promoter	CTPS1
chr1	26855000	chr1	44445000	1.77E-02	super_enhancer	promoter	B4GALT2
chr1	26855000	chr1	53815000	8.79E-03	super_enhancer	promoter	RP11-117D22.1
chr1	26855000	chr1	93545000	2.78E-03	super_enhancer	promoter	MTF2
chr1	26855000	chr1	173795000	2.47E-03	super_enhancer	promoter	CENPL
chr1	26855000	chr1	212005000	2.73E-03	super_enhancer	promoter	LPGAT1
chr1	26865000	chr1	33005000	2.87E-02	super_enhancer	promoter	ZBTB8A
chr1	26865000	chr1	46835000	1.57E-02	super_enhancer	promoter	RP4-603I14.3
chr1	26865000	chr1	47645000	1.50E-02	super_enhancer	promoter	CYP4A22-AS1
chr1	26865000	chr1	52325000	1.15E-02	super_enhancer	promoter	TSEN15P2
chr1	26865000	chr1	54205000	1.19E-02	super_enhancer	promoter	GLIS1
chr1	26865000	chr1	172875000	2.85E-03	super_enhancer	promoter	RP3-471M13.2

chr1	26865000	chr1	234795000	3.49E-03	super_enhancer	promoter	RP4-781K5.5
chr1	26875000	chr1	28415000	2.80E-02	super_enhancer	promoter	EYA3
chr1	26875000	chr1	41445000	2.56E-02	super_enhancer	promoter	CTPS1
chr1	26875000	chr1	42505000	1.89E-02	super_enhancer	promoter	HNRNPFP1
chr1	26875000	chr1	55505000	1.20E-02	super_enhancer	promoter	PCSK9
chr1	26875000	chr1	63155000	8.37E-03	super_enhancer	promoter	DOCK7
chr1	26875000	chr1	151805000	4.63E-03	super_enhancer	promoter	RORC
chr1	26875000	chr1	201965000	4.20E-03	super_enhancer	promoter	RP11-465N4.5
chr1	26875000	chr1	203275000	4.45E-03	super_enhancer	promoter	LINC01136
chr1	26875000	chr1	244515000	3.51E-03	super_enhancer	promoter	C1orf100
chr1	27495000	chr1	36335000	2.42E-02	super_enhancer	promoter	AGO1
chr1	27495000	chr1	53165000	1.12E-02	super_enhancer	promoter	COA7
chr1	27495000	chr1	155555000	3.23E-03	super_enhancer	promoter	DAP3P1
chr1	27495000	chr1	165645000	3.40E-03	super_enhancer	promoter	RP11-466F5.4
chr1	27495000	chr1	236135000	2.98E-03	super_enhancer	promoter	RP4-580N22.2
chr1	27505000	chr1	46835000	1.51E-02	super_enhancer	promoter	RP4-603I14.3
chr1	27505000	chr1	93305000	3.67E-03	super_enhancer	promoter	SNORA66
chr1	27505000	chr1	151805000	3.72E-03	super_enhancer	promoter	RORC
chr1	27505000	chr1	236715000	2.40E-03	super_enhancer	promoter	RP11-385F5.4
chr1	27525000	chr1	82455000	2.14E-03	super_enhancer	promoter	AC113949.1
chr1	27525000	chr1	94345000	4.02E-03	super_enhancer	promoter	DNTTIP2
chr1	27525000	chr1	109585000	3.24E-03	super_enhancer	promoter	WDR47
chr1	27535000	chr1	27975000	3.50E-02	super_enhancer	promoter	RP1-159A19.3
chr1	27535000	chr1	41405000	1.87E-02	super_enhancer	promoter	RP11-348A7.1

chr1	27535000	chr1	43355000	1.62E-02	super_enhancer	promoter	RP11-342M1.7
chr1	27535000	chr1	159335000	2.26E-03	super_enhancer	promoter	OR10J8P
chr1	27535000	chr1	203275000	3.53E-03	super_enhancer	promoter	LINC01136
chr1	27545000	chr1	32225000	4.56E-02	super_enhancer	promoter	MIR4254
chr1	27545000	chr1	33005000	3.01E-02	super_enhancer	promoter	ZBTB8A
chr1	27545000	chr1	39425000	1.95E-02	super_enhancer	promoter	EIF1P2
chr1	27545000	chr1	42805000	1.87E-02	super_enhancer	promoter	RP11-223A3.1
chr1	27545000	chr1	98515000	3.20E-03	super_enhancer	promoter	MIR137HG
chr1	27545000	chr1	154975000	3.02E-03	super_enhancer	promoter	ZBTB7B
chr1	27545000	chr1	169455000	2.74E-03	super_enhancer	promoter	SLC19A2
chr1	27555000	chr1	28135000	2.64E-03	super_enhancer	promoter	AL020997.1
chr1	27555000	chr1	36205000	2.11E-02	super_enhancer	promoter	RP11-435D7.3
chr1	27555000	chr1	150945000	2.79E-03	super_enhancer	promoter	RP11-316M1.3
chr1	27555000	chr1	151765000	2.96E-03	super_enhancer	promoter	TDRKH
chr1	27555000	chr1	201475000	3.02E-03	super_enhancer	promoter	RP11-134G8.7
chr1	27555000	chr1	203145000	3.89E-03	super_enhancer	promoter	MYBPH
chr1	27555000	chr1	235815000	3.31E-03	super_enhancer	promoter	GNG4
chr1	27555000	chr1	248285000	2.05E-03	super_enhancer	promoter	OR2M1P
chr1	27565000	chr1	151345000	3.88E-03	super_enhancer	promoter	SELENBP1
chr1	27565000	chr1	156585000	3.55E-03	super_enhancer	promoter	RP11-284F21.11
chr1	27575000	chr1	27715000	1.99E-02	super_enhancer	promoter	RP1-144C9.2
chr1	27575000	chr1	51525000	9.63E-03	super_enhancer	promoter	MIR4421
chr1	27575000	chr1	78555000	4.15E-03	super_enhancer	promoter	RNU6-1102P
chr1	27575000	chr1	113215000	2.26E-03	super_enhancer	promoter	MOV10

chr1	27575000	chr1	114805000	2.44E-03	super_enhancer	promoter	RP4-543J13.1
chr1	27575000	chr1	155225000	3.63E-03	super_enhancer	promoter	FAM189B
chr1	27575000	chr1	224445000	2.78E-03	super_enhancer	promoter	MIR320B2
chr1	27575000	chr1	233425000	2.55E-03	super_enhancer	promoter	RPS7P3
chr1	27585000	chr1	35735000	2.16E-02	super_enhancer	promoter	ZMYM4
chr1	27585000	chr1	45965000	1.67E-02	super_enhancer	promoter	MMACHC
chr1	27585000	chr1	156265000	3.26E-03	super_enhancer	promoter	GLMP
chr1	27585000	chr1	209175000	2.47E-03	super_enhancer	promoter	RP5-1051D14.2
chr1	27595000	chr1	27995000	1.11E-02	super_enhancer	promoter	RP11-288L9.4
chr1	27595000	chr1	32635000	3.51E-02	super_enhancer	promoter	RP4-622L5.2
chr1	27595000	chr1	93435000	3.35E-03	super_enhancer	promoter	RNU6-970P
chr1	27595000	chr1	152635000	2.25E-03	super_enhancer	promoter	LCE2D
chr1	27595000	chr1	160905000	2.59E-03	super_enhancer	promoter	RP11-312J18.6
chr1	27595000	chr1	235595000	2.46E-03	super_enhancer	promoter	RPS21P1
chr1	27715000	chr1	40885000	1.61E-02	promoter	super_enhancer	RP1-144C9.2
chr1	27715000	chr1	43425000	2.23E-02	promoter	super_enhancer	RP1-144C9.2
chr1	27715000	chr1	155985000	4.28E-03	promoter	super_enhancer	RP1-144C9.2
chr1	27715000	chr1	204435000	4.17E-03	promoter	super_enhancer	RP1-144C9.2
chr1	27715000	chr1	211615000	4.53E-03	promoter	super_enhancer	RP1-144C9.2
chr1	27975000	chr1	31925000	3.09E-02	promoter	super_enhancer	RP1-159A19.3
chr1	27975000	chr1	153535000	2.46E-03	promoter	super_enhancer	RP1-159A19.3
chr1	27975000	chr1	207005000	2.49E-03	promoter	super_enhancer	RP1-159A19.3
chr1	27995000	chr1	33045000	4.66E-02	promoter	super_enhancer	RP11-288L9.4
chr1	27995000	chr1	204305000	4.36E-03	promoter	super_enhancer	RP11-288L9.4

chr1	27995000	chr1	211655000	4.11E-03	promoter	super_enhancer	RP11-288L9.4
chr1	28015000	chr1	31955000	3.91E-02	promoter	super_enhancer	CHMP1AP1
chr1	28015000	chr1	32995000	3.68E-02	promoter	super_enhancer	CHMP1AP1
chr1	28015000	chr1	40895000	1.55E-02	promoter	super_enhancer	CHMP1AP1
chr1	28015000	chr1	43445000	1.69E-02	promoter	super_enhancer	CHMP1AP1
chr1	28015000	chr1	43465000	1.89E-02	promoter	super_enhancer	CHMP1AP1
chr1	28065000	chr1	32725000	4.17E-02	promoter	super_enhancer	RP3-426I6.2
chr1	28065000	chr1	156505000	3.37E-03	promoter	super_enhancer	RP3-426I6.2
chr1	28065000	chr1	202015000	3.36E-03	promoter	super_enhancer	RP3-426I6.2
chr1	28065000	chr1	209645000	2.83E-03	promoter	super_enhancer	RP3-426I6.2
chr1	28135000	chr1	32745000	3.90E-02	promoter	super_enhancer	AL020997.1
chr1	28135000	chr1	32755000	3.50E-02	promoter	super_enhancer	AL020997.1
chr1	28135000	chr1	32995000	3.08E-02	promoter	super_enhancer	AL020997.1
chr1	28135000	chr1	36135000	1.85E-02	promoter	super_enhancer	AL020997.1
chr1	28135000	chr1	37475000	1.85E-02	promoter	super_enhancer	AL020997.1
chr1	28145000	chr1	204425000	2.68E-03	promoter	super_enhancer	RP3-426I6.6
chr1	28285000	chr1	32985000	4.82E-02	promoter	super_enhancer	XKR8
chr1	28285000	chr1	36185000	2.74E-02	promoter	super_enhancer	XKR8
chr1	28285000	chr1	204425000	3.84E-03	promoter	super_enhancer	XKR8
chr1	28285000	chr1	204435000	3.63E-03	promoter	super_enhancer	XKR8
chr1	28415000	chr1	32735000	4.71E-02	promoter	super_enhancer	EYA3
chr1	28415000	chr1	93705000	3.42E-03	promoter	super_enhancer	EYA3
chr1	28415000	chr1	209655000	2.95E-03	promoter	super_enhancer	EYA3
chr1	28585000	chr1	42945000	1.91E-02	promoter	super_enhancer	SESN2

chr1	28655000	chr1	31945000	4.42E-02	promoter	super_enhancer	MED18
chr1	28655000	chr1	41915000	2.09E-02	promoter	super_enhancer	MED18
chr1	28655000	chr1	43495000	1.79E-02	promoter	super_enhancer	MED18
chr1	28695000	chr1	67685000	5.99E-03	promoter	super_enhancer	PHACTR4
chr1	28835000	chr1	31955000	1.50E-03	promoter	super_enhancer	SNORA73B
chr1	28835000	chr1	41895000	1.87E-02	promoter	super_enhancer	SNORA73B
chr1	28835000	chr1	235905000	2.52E-03	promoter	super_enhancer	SNORA73B
chr1	28855000	chr1	40925000	1.67E-02	promoter	super_enhancer	PRDX3P2
chr1	28855000	chr1	155125000	3.46E-03	promoter	super_enhancer	PRDX3P2
chr1	28855000	chr1	211615000	3.66E-03	promoter	super_enhancer	PRDX3P2
chr1	28905000	chr1	50975000	8.11E-03	promoter	super_enhancer	SNORD99
chr1	28975000	chr1	36175000	2.96E-02	promoter	super_enhancer	RNU11
chr1	28995000	chr1	41905000	2.66E-02	promoter	super_enhancer	GMEB1
chr1	29205000	chr1	43425000	2.06E-02	promoter	super_enhancer	RP1-212P9.3
chr1	29205000	chr1	43435000	2.15E-02	promoter	super_enhancer	RP1-212P9.3
chr1	29205000	chr1	43475000	1.98E-02	promoter	super_enhancer	RP1-212P9.3
chr1	29205000	chr1	234975000	3.54E-03	promoter	super_enhancer	RP1-212P9.3
chr1	29475000	chr1	36135000	2.92E-02	promoter	super_enhancer	RP11-242O24.3
chr1	29475000	chr1	44805000	1.85E-02	promoter	super_enhancer	RP11-242O24.3
chr1	29475000	chr1	94635000	3.74E-03	promoter	super_enhancer	RP11-242O24.3
chr1	29475000	chr1	152045000	3.30E-03	promoter	super_enhancer	RP11-242O24.3
chr1	29475000	chr1	202075000	3.32E-03	promoter	super_enhancer	RP11-242O24.3
chr1	30885000	chr1	41895000	2.21E-02	promoter	super_enhancer	RP4-591L5.1
chr1	30885000	chr1	41915000	2.64E-02	promoter	super_enhancer	RP4-591L5.1

chr1	30885000	chr1	67695000	7.29E-03	promoter	super_enhancer	RP4-591L5.1
chr1	31575000	chr1	42605000	1.93E-02	super_enhancer	promoter	RP11-319C21.1
chr1	31575000	chr1	56675000	8.40E-03	super_enhancer	promoter	RP4-710M16.1
chr1	31575000	chr1	244815000	2.92E-03	super_enhancer	promoter	DESI2
chr1	31575000	chr1	247805000	2.71E-03	super_enhancer	promoter	RP11-978I15.10
chr1	31585000	chr1	35815000	4.02E-02	super_enhancer	promoter	RPL5P4
chr1	31585000	chr1	36205000	3.82E-02	super_enhancer	promoter	RP11-435D7.3
chr1	31585000	chr1	46835000	1.85E-02	super_enhancer	promoter	RP4-603I14.3
chr1	31585000	chr1	93955000	4.33E-03	super_enhancer	promoter	RNA5SP53
chr1	31665000	chr1	36025000	4.66E-02	super_enhancer	promoter	KIAA0319L
chr1	31665000	chr1	157895000	2.97E-03	super_enhancer	promoter	RP11-451O13.1
chr1	31665000	chr1	172855000	2.36E-03	super_enhancer	promoter	AIMP1P2
chr1	31665000	chr1	203445000	2.48E-03	super_enhancer	promoter	PRELP
chr1	31675000	chr1	211385000	3.37E-03	super_enhancer	promoter	AC092017.1
chr1	31685000	chr1	36395000	3.44E-02	super_enhancer	promoter	AGO3
chr1	31685000	chr1	42845000	2.78E-02	super_enhancer	promoter	RIMKLA
chr1	31685000	chr1	44115000	1.94E-02	super_enhancer	promoter	KDM4A
chr1	31685000	chr1	44445000	2.84E-02	super_enhancer	promoter	B4GALT2
chr1	31685000	chr1	56725000	1.22E-02	super_enhancer	promoter	RP11-504A18.1
chr1	31685000	chr1	71515000	8.93E-03	super_enhancer	promoter	ZRANB2-AS1
chr1	31685000	chr1	158765000	2.77E-03	super_enhancer	promoter	OR2AQ1P
chr1	31685000	chr1	159245000	3.18E-03	super_enhancer	promoter	MPTX1
chr1	31685000	chr1	161735000	3.56E-03	super_enhancer	promoter	ATF6
chr1	31685000	chr1	241995000	3.59E-03	super_enhancer	promoter	RP11-553N16.1

chr1	31695000	chr1	41445000	3.08E-02	super_enhancer	promoter	CTPS1
chr1	31695000	chr1	53815000	1.32E-02	super_enhancer	promoter	RP11-117D22.1
chr1	31695000	chr1	153505000	3.71E-03	super_enhancer	promoter	BX470102.3
chr1	31695000	chr1	161035000	3.91E-03	super_enhancer	promoter	AL591806.1
chr1	31695000	chr1	164325000	1.87E-03	super_enhancer	promoter	HMGB3P6
chr1	31695000	chr1	204595000	4.11E-03	super_enhancer	promoter	RP11-430C7.5
chr1	31695000	chr1	205535000	3.12E-03	super_enhancer	promoter	RNU2-19P
chr1	31705000	chr1	35815000	3.98E-02	super_enhancer	promoter	RPL5P4
chr1	31705000	chr1	36395000	2.80E-02	super_enhancer	promoter	AGO3
chr1	31705000	chr1	45805000	1.31E-02	super_enhancer	promoter	TOE1
chr1	31705000	chr1	56405000	9.25E-03	super_enhancer	promoter	PIGQP1
chr1	31705000	chr1	151585000	2.86E-03	super_enhancer	promoter	SNX27
chr1	31715000	chr1	43355000	2.13E-02	super_enhancer	promoter	RP11-342M1.7
chr1	31715000	chr1	93645000	3.78E-03	super_enhancer	promoter	CCDC18
chr1	31715000	chr1	150025000	2.31E-03	super_enhancer	promoter	RP11-458I7.1
chr1	31925000	chr1	37945000	2.46E-02	super_enhancer	promoter	MIR6732
chr1	31925000	chr1	43355000	1.61E-02	super_enhancer	promoter	RP11-342M1.7
chr1	31925000	chr1	53175000	1.20E-02	super_enhancer	promoter	NDUFS5P3
chr1	31925000	chr1	100435000	2.79E-03	super_enhancer	promoter	RP5-884G6.2
chr1	31925000	chr1	168185000	2.59E-03	super_enhancer	promoter	RP1-69E11.3
chr1	31935000	chr1	35315000	4.44E-02	super_enhancer	promoter	RP5-997D16.2
chr1	31935000	chr1	40255000	1.55E-02	super_enhancer	promoter	BMP8B
chr1	31935000	chr1	204465000	2.56E-03	super_enhancer	promoter	PIK3C2B
chr1	31955000	chr1	48015000	1.52E-02	super_enhancer	promoter	ATP6V0E1P4

chr1	31955000	chr1	52645000	8.43E-03	super_enhancer	promoter	PDCL3P6
chr1	31955000	chr1	55505000	9.64E-03	super_enhancer	promoter	PCSK9
chr1	31955000	chr1	85725000	4.55E-03	super_enhancer	promoter	C1orf52
chr1	32225000	chr1	40915000	2.60E-02	promoter	super_enhancer	MIR4254
chr1	32225000	chr1	206905000	4.22E-03	promoter	super_enhancer	MIR4254
chr1	32255000	chr1	43475000	1.88E-02	promoter	super_enhancer	RP11-84A19.3
chr1	32255000	chr1	43505000	2.07E-02	promoter	super_enhancer	RP11-84A19.3
chr1	32255000	chr1	202055000	3.04E-03	promoter	super_enhancer	RP11-84A19.3
chr1	32255000	chr1	234965000	2.77E-03	promoter	super_enhancer	RP11-84A19.3
chr1	32395000	chr1	40965000	1.48E-02	promoter	super_enhancer	AL136115.1
chr1	32635000	chr1	42965000	1.38E-02	promoter	super_enhancer	RP4-622L5.2
chr1	32635000	chr1	43425000	2.14E-02	promoter	super_enhancer	RP4-622L5.2
chr1	32645000	chr1	43425000	2.12E-02	promoter	super_enhancer	TXLNA
chr1	32645000	chr1	67695000	7.26E-03	promoter	super_enhancer	TXLNA
chr1	32645000	chr1	153605000	3.16E-03	promoter	super_enhancer	TXLNA
chr1	32645000	chr1	204325000	3.17E-03	promoter	super_enhancer	TXLNA
chr1	32645000	chr1	205305000	3.83E-03	promoter	super_enhancer	TXLNA
chr1	32665000	chr1	36135000	2.05E-03	promoter	super_enhancer	CCDC28B
chr1	32665000	chr1	42955000	1.72E-02	promoter	super_enhancer	CCDC28B
chr1	32675000	chr1	33055000	3.43E-02	promoter	super_enhancer	DCDC2B
chr1	32675000	chr1	234995000	3.43E-03	promoter	super_enhancer	DCDC2B
chr1	32715000	chr1	36395000	4.01E-02	super_enhancer	promoter	AGO3
chr1	32715000	chr1	40025000	2.17E-02	super_enhancer	promoter	PPIEL
chr1	32715000	chr1	63605000	8.28E-03	super_enhancer	promoter	RP11-335E6.2

chr1	32715000	chr1	204245000	3.49E-03	super_enhancer	promoter	RP11-203F10.5
chr1	32725000	chr1	35315000	4.55E-03	super_enhancer	promoter	RP5-997D16.2
chr1	32725000	chr1	44585000	2.22E-02	super_enhancer	promoter	KLF17
chr1	32725000	chr1	51425000	1.59E-02	super_enhancer	promoter	CDKN2C
chr1	32725000	chr1	53165000	1.36E-02	super_enhancer	promoter	COA7
chr1	32725000	chr1	154955000	3.10E-03	super_enhancer	promoter	FLAD1
chr1	32725000	chr1	179335000	2.86E-03	super_enhancer	promoter	AXDND1
chr1	32725000	chr1	186265000	2.31E-03	super_enhancer	promoter	PRG4
chr1	32725000	chr1	203595000	3.44E-03	super_enhancer	promoter	ATP2B4
chr1	32735000	chr1	36395000	4.16E-02	super_enhancer	promoter	AGO3
chr1	32735000	chr1	42805000	2.59E-02	super_enhancer	promoter	RP11-223A3.1
chr1	32735000	chr1	77245000	6.57E-03	super_enhancer	promoter	RP11-415A20.1
chr1	32735000	chr1	151085000	3.51E-03	super_enhancer	promoter	RP11-68I18.2
chr1	32735000	chr1	172115000	1.92E-03	super_enhancer	promoter	DNM3OS
chr1	32745000	chr1	43735000	2.82E-02	super_enhancer	promoter	TMEM125
chr1	32745000	chr1	48015000	2.33E-02	super_enhancer	promoter	ATP6V0E1P4
chr1	32745000	chr1	52175000	9.21E-05	super_enhancer	promoter	GAPDHP51
chr1	32745000	chr1	205425000	4.62E-03	super_enhancer	promoter	RP11-576D8.4
chr1	32745000	chr1	208085000	2.98E-03	super_enhancer	promoter	CD34
chr1	32745000	chr1	224625000	3.71E-03	super_enhancer	promoter	WDR26
chr1	32745000	chr1	229645000	3.77E-03	super_enhancer	promoter	NUP133
chr1	32755000	chr1	37795000	3.45E-02	super_enhancer	promoter	RPS29P6
chr1	32755000	chr1	38475000	3.30E-02	super_enhancer	promoter	UTP11L
chr1	32755000	chr1	65525000	7.50E-03	super_enhancer	promoter	MIR3671

chr1	32755000	chr1	110715000	3.64E-03	super_enhancer	promoter	RP5-1028L10.1
chr1	32755000	chr1	150335000	3.12E-03	super_enhancer	promoter	RPRD2
chr1	32755000	chr1	153895000	3.35E-03	super_enhancer	promoter	GATAD2B
chr1	32765000	chr1	73365000	3.99E-03	super_enhancer	promoter	RP4-660H19.1
chr1	32765000	chr1	236135000	3.03E-03	super_enhancer	promoter	RP4-580N22.2
chr1	32775000	chr1	45805000	1.51E-02	super_enhancer	promoter	TOE1
chr1	32775000	chr1	151105000	3.32E-03	super_enhancer	promoter	RP11-68I18.10
chr1	32985000	chr1	46805000	1.74E-02	super_enhancer	promoter	NSUN4
chr1	32985000	chr1	54205000	1.51E-02	super_enhancer	promoter	GLIS1
chr1	32985000	chr1	68945000	6.15E-03	super_enhancer	promoter	RP4-694A7.2
chr1	32985000	chr1	87595000	5.82E-03	super_enhancer	promoter	LINC01140
chr1	32985000	chr1	179805000	2.29E-03	super_enhancer	promoter	RP11-12M5.4
chr1	32985000	chr1	182295000	2.81E-03	super_enhancer	promoter	RNU6-152P
chr1	32985000	chr1	244815000	3.28E-03	super_enhancer	promoter	DESI2
chr1	32995000	chr1	33285000	2.34E-02	super_enhancer	promoter	YARS
chr1	32995000	chr1	43915000	2.16E-02	super_enhancer	promoter	SZT2-AS1
chr1	32995000	chr1	84545000	3.57E-03	super_enhancer	promoter	RP11-486G15.2
chr1	32995000	chr1	115055000	2.70E-03	super_enhancer	promoter	TRIM33
chr1	32995000	chr1	182295000	2.70E-03	super_enhancer	promoter	RNU6-152P
chr1	32995000	chr1	206305000	2.86E-03	super_enhancer	promoter	C1orf186
chr1	32995000	chr1	228675000	3.25E-03	super_enhancer	promoter	RP5-915N17.11
chr1	33005000	chr1	181075000	2.51E-03	promoter	super_enhancer	ZBTB8A
chr1	33035000	chr1	43735000	2.23E-02	super_enhancer	promoter	TMEM125
chr1	33035000	chr1	47965000	1.56E-02	super_enhancer	promoter	RPL21P24

chr1	33035000	chr1	52615000	1.33E-02	super_enhancer	promoter	RN7SL788P
chr1	33035000	chr1	205745000	2.18E-03	super_enhancer	promoter	RAB29
chr1	33035000	chr1	231805000	2.35E-03	super_enhancer	promoter	RNU5A-5P
chr1	33045000	chr1	39425000	2.89E-02	super_enhancer	promoter	EIF1P2
chr1	33055000	chr1	52445000	1.58E-02	super_enhancer	promoter	RP11-91A18.1
chr1	33055000	chr1	60255000	9.17E-03	super_enhancer	promoter	RP4-782L23.2
chr1	33055000	chr1	166765000	2.37E-03	super_enhancer	promoter	CNN2P10
chr1	33055000	chr1	183235000	3.64E-03	super_enhancer	promoter	RP11-548N1.1
chr1	33055000	chr1	244015000	2.56E-03	super_enhancer	promoter	AKT3
chr1	33095000	chr1	35395000	6.88E-03	super_enhancer	promoter	DLGAP3
chr1	33095000	chr1	52615000	1.44E-02	super_enhancer	promoter	RN7SL788P
chr1	33095000	chr1	53835000	1.33E-02	super_enhancer	promoter	RP11-117D22.2
chr1	33095000	chr1	54155000	1.57E-02	super_enhancer	promoter	RNU7-95P
chr1	33095000	chr1	145515000	2.77E-03	super_enhancer	promoter	PEX11B
chr1	33095000	chr1	149285000	3.13E-03	super_enhancer	promoter	RP11-403I13.7
chr1	33095000	chr1	155145000	3.71E-03	super_enhancer	promoter	TRIM46
chr1	33285000	chr1	156095000	2.95E-03	promoter	super_enhancer	YARS
chr1	33285000	chr1	202055000	2.72E-03	promoter	super_enhancer	YARS
chr1	33285000	chr1	234975000	2.59E-03	promoter	super_enhancer	YARS
chr1	33815000	chr1	55435000	1.08E-02	promoter	super_enhancer	RP11-415J8.5
chr1	35135000	chr1	39185000	4.01E-02	promoter	super_enhancer	MIR552
chr1	35225000	chr1	36155000	4.67E-02	promoter	super_enhancer	GJB4
chr1	35225000	chr1	66275000	7.61E-03	promoter	super_enhancer	GJB4
chr1	35225000	chr1	94625000	3.46E-03	promoter	super_enhancer	GJB4

chr1	35315000	chr1	67695000	8.01E-03	promoter	super_enhancer	RP5-997D16.2
chr1	35315000	chr1	206895000	2.78E-03	promoter	super_enhancer	RP5-997D16.2
chr1	35325000	chr1	151195000	2.99E-03	promoter	super_enhancer	SMIM12
chr1	35395000	chr1	40865000	2.66E-02	promoter	super_enhancer	DLGAP3
chr1	35525000	chr1	40965000	2.32E-02	promoter	super_enhancer	ZMYM1
chr1	35525000	chr1	41495000	2.76E-02	promoter	super_enhancer	ZMYM1
chr1	35525000	chr1	204125000	2.39E-03	promoter	super_enhancer	ZMYM1
chr1	35525000	chr1	235905000	1.91E-03	promoter	super_enhancer	ZMYM1
chr1	35735000	chr1	39195000	3.64E-02	promoter	super_enhancer	ZMYM4
chr1	35815000	chr1	41515000	2.59E-02	promoter	super_enhancer	RPL5P4
chr1	35815000	chr1	109885000	2.99E-03	promoter	super_enhancer	RPL5P4
chr1	35815000	chr1	172895000	1.94E-03	promoter	super_enhancer	RPL5P4
chr1	35815000	chr1	202055000	2.91E-03	promoter	super_enhancer	RPL5P4
chr1	35815000	chr1	234725000	2.54E-03	promoter	super_enhancer	RPL5P4
chr1	35975000	chr1	40905000	2.98E-02	promoter	super_enhancer	RP4-728D4.3
chr1	35975000	chr1	181135000	2.78E-03	promoter	super_enhancer	RP4-728D4.3
chr1	36025000	chr1	36185000	6.94E-03	promoter	super_enhancer	KIAA0319L
chr1	36025000	chr1	40875000	3.66E-02	promoter	super_enhancer	KIAA0319L
chr1	36025000	chr1	42955000	2.46E-02	promoter	super_enhancer	KIAA0319L
chr1	36025000	chr1	109885000	3.97E-03	promoter	super_enhancer	KIAA0319L
chr1	36025000	chr1	158035000	3.93E-03	promoter	super_enhancer	KIAA0319L
chr1	36025000	chr1	228145000	4.45E-03	promoter	super_enhancer	KIAA0319L
chr1	36025000	chr1	234975000	3.67E-03	promoter	super_enhancer	KIAA0319L
chr1	36135000	chr1	45265000	2.31E-02	super_enhancer	promoter	PLK3

chr1	36135000	chr1	45805000	1.70E-02	super_enhancer	promoter	TOE1
chr1	36135000	chr1	46805000	1.90E-02	super_enhancer	promoter	NSUN4
chr1	36135000	chr1	51985000	1.01E-02	super_enhancer	promoter	EPS15
chr1	36135000	chr1	74215000	5.53E-03	super_enhancer	promoter	RNA5SP50
chr1	36135000	chr1	150785000	3.08E-03	super_enhancer	promoter	RNU6-1309P
chr1	36135000	chr1	189745000	1.79E-03	super_enhancer	promoter	RP11-398M15.1
chr1	36135000	chr1	193075000	2.80E-03	super_enhancer	promoter	GLRX2
chr1	36145000	chr1	48695000	2.07E-02	super_enhancer	promoter	RP5-1024N4.4
chr1	36145000	chr1	52175000	1.39E-02	super_enhancer	promoter	GAPDHP51
chr1	36145000	chr1	53175000	1.98E-02	super_enhancer	promoter	NDUFS5P3
chr1	36145000	chr1	59465000	1.00E-02	super_enhancer	promoter	PHBP3
chr1	36145000	chr1	95285000	4.43E-03	super_enhancer	promoter	SLC44A3
chr1	36145000	chr1	161995000	3.15E-03	super_enhancer	promoter	OLFML2B
chr1	36155000	chr1	46805000	1.76E-02	super_enhancer	promoter	NSUN4
chr1	36155000	chr1	46915000	2.56E-02	super_enhancer	promoter	LINC01398
chr1	36155000	chr1	52645000	1.13E-02	super_enhancer	promoter	PDCL3P6
chr1	36155000	chr1	53815000	1.26E-02	super_enhancer	promoter	RP11-117D22.1
chr1	36155000	chr1	94395000	3.89E-03	super_enhancer	promoter	RP5-837O21.1
chr1	36155000	chr1	187675000	2.31E-03	super_enhancer	promoter	SLC4A1APP2
chr1	36155000	chr1	236645000	2.66E-03	super_enhancer	promoter	ENO1P1
chr1	36155000	chr1	241995000	2.81E-03	super_enhancer	promoter	RP11-553N16.1
chr1	36165000	chr1	47965000	2.00E-02	super_enhancer	promoter	RPL21P24
chr1	36165000	chr1	57285000	1.27E-02	super_enhancer	promoter	C1orf168
chr1	36165000	chr1	65765000	9.01E-03	super_enhancer	promoter	COX6CP13

chr1	36165000	chr1	67995000	1.13E-02	super_enhancer	promoter	RP4-763G1.2
chr1	36165000	chr1	111725000	2.81E-03	super_enhancer	promoter	RP5-1180E21.4
chr1	36165000	chr1	228775000	1.98E-03	super_enhancer	promoter	RNA5S14
chr1	36175000	chr1	52325000	1.97E-02	super_enhancer	promoter	TSEN15P2
chr1	36175000	chr1	95585000	5.13E-03	super_enhancer	promoter	RP11-57H12.2
chr1	36175000	chr1	168755000	3.43E-03	super_enhancer	promoter	LINC00626
chr1	36175000	chr1	226375000	4.39E-03	super_enhancer	promoter	ACBD3
chr1	36185000	chr1	42225000	2.90E-02	super_enhancer	promoter	AL451006.1
chr1	36185000	chr1	43035000	2.42E-02	super_enhancer	promoter	RNU6-536P
chr1	36185000	chr1	46805000	1.93E-02	super_enhancer	promoter	NSUN4
chr1	36185000	chr1	47135000	2.20E-02	super_enhancer	promoter	TEX38
chr1	36185000	chr1	51985000	1.03E-02	super_enhancer	promoter	EPS15
chr1	36185000	chr1	58695000	9.47E-03	super_enhancer	promoter	RP11-414C16.1
chr1	36185000	chr1	63545000	9.51E-03	super_enhancer	promoter	RP11-5P4.3
chr1	36185000	chr1	160255000	2.53E-03	super_enhancer	promoter	RP11-574F21.3
chr1	36185000	chr1	203595000	3.25E-03	super_enhancer	promoter	ATP2B4
chr1	36185000	chr1	245185000	2.10E-03	super_enhancer	promoter	RNU6-1089P
chr1	36205000	chr1	41515000	2.94E-02	promoter	super_enhancer	RP11-435D7.3
chr1	36205000	chr1	43535000	4.13E-04	promoter	super_enhancer	RP11-435D7.3
chr1	36335000	chr1	153545000	2.94E-03	promoter	super_enhancer	AGO1
chr1	36335000	chr1	234965000	3.09E-03	promoter	super_enhancer	AGO1
chr1	36375000	chr1	38475000	6.57E-03	super_enhancer	promoter	UTP11L
chr1	36375000	chr1	41405000	4.26E-02	super_enhancer	promoter	RP11-348A7.1
chr1	36375000	chr1	46645000	2.40E-02	super_enhancer	promoter	AL672043.1

chr1	36375000	chr1	47135000	2.21E-02	super_enhancer	promoter	TEX38
chr1	36375000	chr1	51245000	1.66E-02	super_enhancer	promoter	PHBP12
chr1	36375000	chr1	66045000	6.56E-03	super_enhancer	promoter	RP11-430H12.2
chr1	36395000	chr1	41495000	3.21E-02	promoter	super_enhancer	AGO3
chr1	36395000	chr1	42955000	1.62E-02	promoter	super_enhancer	AGO3
chr1	36395000	chr1	42965000	1.43E-02	promoter	super_enhancer	AGO3
chr1	36395000	chr1	43435000	2.21E-02	promoter	super_enhancer	AGO3
chr1	37095000	chr1	43485000	2.73E-02	promoter	super_enhancer	FTLP18
chr1	37375000	chr1	40995000	1.18E-03	promoter	super_enhancer	AC117945.1
chr1	37475000	chr1	59545000	1.24E-02	super_enhancer	promoter	RP11-145M4.2
chr1	37475000	chr1	197745000	2.44E-03	super_enhancer	promoter	DENND1B
chr1	37795000	chr1	234995000	2.70E-03	promoter	super_enhancer	RPS29P6
chr1	37945000	chr1	225465000	2.75E-03	promoter	super_enhancer	MIR6732
chr1	38325000	chr1	230105000	2.60E-03	promoter	super_enhancer	MTF1
chr1	38555000	chr1	67735000	1.05E-02	promoter	super_enhancer	MIR3659
chr1	38555000	chr1	209665000	3.00E-03	promoter	super_enhancer	MIR3659
chr1	39175000	chr1	45205000	2.88E-02	super_enhancer	promoter	KIF2C
chr1	39175000	chr1	47185000	2.54E-02	super_enhancer	promoter	EFCAB14
chr1	39175000	chr1	48555000	1.80E-02	super_enhancer	promoter	CYP46A4P
chr1	39175000	chr1	204245000	3.40E-03	super_enhancer	promoter	RP11-203F10.5
chr1	39185000	chr1	45805000	2.26E-02	super_enhancer	promoter	TOE1
chr1	39185000	chr1	88275000	4.05E-03	super_enhancer	promoter	RP11-60A14.1
chr1	39185000	chr1	116915000	2.57E-03	super_enhancer	promoter	ATP1A1
chr1	39185000	chr1	161995000	2.87E-03	super_enhancer	promoter	OLFML2B

chr1	39185000	chr1	247205000	2.12E-03	super_enhancer	promoter	RP11-551G24.2
chr1	39195000	chr1	43355000	3.45E-02	super_enhancer	promoter	RP11-342M1.7
chr1	39195000	chr1	48695000	1.54E-02	super_enhancer	promoter	RP5-1024N4.4
chr1	39195000	chr1	64255000	6.46E-03	super_enhancer	promoter	RP4-597J3.1
chr1	39195000	chr1	226625000	2.22E-03	super_enhancer	promoter	RP11-118H4.1
chr1	39325000	chr1	55445000	1.68E-02	promoter	super_enhancer	RRAGC
chr1	40025000	chr1	202065000	2.55E-03	promoter	super_enhancer	PPIEL
chr1	40255000	chr1	44805000	4.26E-02	promoter	super_enhancer	BMP8B
chr1	40725000	chr1	93705000	3.48E-03	promoter	super_enhancer	RP1-39G22.7
chr1	40725000	chr1	156745000	2.57E-03	promoter	super_enhancer	RP1-39G22.7
chr1	40855000	chr1	41445000	7.83E-03	super_enhancer	promoter	CTPS1
chr1	40855000	chr1	93955000	4.54E-03	super_enhancer	promoter	RNA5SP53
chr1	40855000	chr1	112015000	4.15E-06	super_enhancer	promoter	C1orf162
chr1	40855000	chr1	184985000	2.71E-03	super_enhancer	promoter	RNA5SP72
chr1	40855000	chr1	193105000	2.20E-03	super_enhancer	promoter	MIR1278
chr1	40865000	chr1	44555000	4.66E-02	super_enhancer	promoter	RP5-1198O20.5
chr1	40865000	chr1	94375000	1.64E-03	super_enhancer	promoter	GCLM
chr1	40875000	chr1	41405000	3.65E-02	super_enhancer	promoter	RP11-348A7.1
chr1	40875000	chr1	53795000	1.39E-02	super_enhancer	promoter	LRP8
chr1	40875000	chr1	79215000	4.47E-03	super_enhancer	promoter	RP4-641G12.4
chr1	40875000	chr1	150255000	2.60E-03	super_enhancer	promoter	CIART
chr1	40875000	chr1	152995000	2.20E-03	super_enhancer	promoter	RP1-20N18.4
chr1	40875000	chr1	218065000	1.97E-03	super_enhancer	promoter	LINC00210
chr1	40885000	chr1	53405000	1.36E-02	super_enhancer	promoter	MIR1273G

chr1	40885000	chr1	95585000	3.26E-03	super_enhancer	promoter	RP11-57H12.2
chr1	40885000	chr1	174965000	1.61E-03	super_enhancer	promoter	RNU6-307P
chr1	40895000	chr1	53925000	1.90E-02	super_enhancer	promoter	DMRTB1
chr1	40895000	chr1	204465000	3.19E-03	super_enhancer	promoter	PIK3C2B
chr1	40905000	chr1	46915000	4.05E-02	super_enhancer	promoter	LINC01398
chr1	40905000	chr1	174865000	2.15E-03	super_enhancer	promoter	RABGAP1L-IT1
chr1	40915000	chr1	46155000	3.99E-02	super_enhancer	promoter	GPBP1L1
chr1	40915000	chr1	46915000	4.32E-02	super_enhancer	promoter	LINC01398
chr1	40915000	chr1	204245000	3.43E-03	super_enhancer	promoter	RP11-203F10.5
chr1	40915000	chr1	204465000	3.50E-03	super_enhancer	promoter	PIK3C2B
chr1	40925000	chr1	52615000	1.71E-02	super_enhancer	promoter	RN7SL788P
chr1	40925000	chr1	53795000	1.47E-02	super_enhancer	promoter	LRP8
chr1	40925000	chr1	54155000	1.84E-02	super_enhancer	promoter	RNU7-95P
chr1	40925000	chr1	166975000	2.25E-03	super_enhancer	promoter	RNA5SP65
chr1	40925000	chr1	201925000	2.39E-03	super_enhancer	promoter	TIMM17A
chr1	40925000	chr1	218475000	1.90E-03	super_enhancer	promoter	RP11-224O19.4
chr1	40925000	chr1	228285000	3.10E-03	super_enhancer	promoter	MIR3620
chr1	40945000	chr1	158575000	1.30E-03	super_enhancer	promoter	OR10Z1
chr1	40945000	chr1	228265000	2.19E-03	super_enhancer	promoter	RP5-881P19.7
chr1	40955000	chr1	42845000	9.10E-03	super_enhancer	promoter	RIMKLA
chr1	40955000	chr1	46645000	2.96E-02	super_enhancer	promoter	AL672043.1
chr1	40955000	chr1	62505000	1.08E-02	super_enhancer	promoter	LAMTOR5P1
chr1	40955000	chr1	81065000	3.29E-03	super_enhancer	promoter	AL136234.1
chr1	40955000	chr1	84485000	2.59E-03	super_enhancer	promoter	RP4-552O12.1

chr1	40955000	chr1	90405000	3.25E-03	super_enhancer	promoter	RP11-302M6.5
chr1	40955000	chr1	156615000	2.62E-03	super_enhancer	promoter	RP11-284F21.10
chr1	40955000	chr1	193075000	2.14E-03	super_enhancer	promoter	GLRX2
chr1	40955000	chr1	206225000	2.62E-03	super_enhancer	promoter	RP11-38J22.3
chr1	40965000	chr1	53405000	1.79E-02	super_enhancer	promoter	MIR1273G
chr1	40965000	chr1	84945000	5.35E-03	super_enhancer	promoter	RPF1
chr1	40965000	chr1	93435000	4.36E-03	super_enhancer	promoter	RNU6-970P
chr1	40965000	chr1	203275000	3.37E-03	super_enhancer	promoter	LINC01136
chr1	40975000	chr1	41445000	2.29E-02	super_enhancer	promoter	CTPS1
chr1	40975000	chr1	47135000	3.20E-02	super_enhancer	promoter	TEX38
chr1	40975000	chr1	53705000	1.62E-02	super_enhancer	promoter	MAGOH
chr1	40975000	chr1	93545000	3.95E-03	super_enhancer	promoter	MTF2
chr1	40975000	chr1	144875000	4.31E-03	super_enhancer	promoter	RP4-791M13.5
chr1	40975000	chr1	151585000	2.84E-03	super_enhancer	promoter	SNX27
chr1	40985000	chr1	47875000	2.33E-02	super_enhancer	promoter	LINC01389
chr1	40985000	chr1	103575000	2.26E-03	super_enhancer	promoter	COL11A1
chr1	40985000	chr1	153515000	3.30E-03	super_enhancer	promoter	S100A5
chr1	40985000	chr1	225925000	1.98E-03	super_enhancer	promoter	RP11-145A3.2
chr1	40995000	chr1	48015000	3.19E-02	super_enhancer	promoter	ATP6V0E1P4
chr1	40995000	chr1	74215000	6.42E-03	super_enhancer	promoter	RNA5SP50
chr1	40995000	chr1	84865000	5.25E-03	super_enhancer	promoter	UOX
chr1	40995000	chr1	159245000	2.69E-03	super_enhancer	promoter	MPTX1
chr1	40995000	chr1	207925000	2.35E-03	super_enhancer	promoter	CD46
chr1	40995000	chr1	248285000	2.03E-03	super_enhancer	promoter	OR2M1P

chr1	41405000	chr1	94645000	4.92E-03	promoter	super_enhancer	RP11-348A7.1
chr1	41405000	chr1	204445000	3.15E-03	promoter	super_enhancer	RP11-348A7.1
chr1	41445000	chr1	42945000	3.39E-02	promoter	super_enhancer	CTPS1
chr1	41445000	chr1	55435000	2.13E-02	promoter	super_enhancer	CTPS1
chr1	41485000	chr1	48555000	2.35E-02	super_enhancer	promoter	CYP46A4P
chr1	41485000	chr1	113435000	3.11E-03	super_enhancer	promoter	RP3-522D1.2
chr1	41485000	chr1	220375000	2.38E-03	super_enhancer	promoter	MIR664A
chr1	41495000	chr1	48015000	4.11E-02	super_enhancer	promoter	ATP6V0E1P4
chr1	41495000	chr1	48695000	3.31E-02	super_enhancer	promoter	RP5-1024N4.4
chr1	41495000	chr1	62675000	1.42E-02	super_enhancer	promoter	RP5-1155K23.4
chr1	41495000	chr1	100315000	4.87E-03	super_enhancer	promoter	AGL
chr1	41495000	chr1	160255000	3.03E-03	super_enhancer	promoter	RP11-574F21.3
chr1	41495000	chr1	246845000	4.20E-03	super_enhancer	promoter	RP11-439E19.9
chr1	41505000	chr1	43035000	1.07E-02	super_enhancer	promoter	RNU6-536P
chr1	41505000	chr1	46015000	4.17E-02	super_enhancer	promoter	AKR1A1
chr1	41505000	chr1	46835000	3.36E-02	super_enhancer	promoter	RP4-603I14.3
chr1	41505000	chr1	46915000	4.01E-02	super_enhancer	promoter	LINC01398
chr1	41505000	chr1	53815000	1.45E-02	super_enhancer	promoter	RP11-117D22.1
chr1	41505000	chr1	86175000	5.00E-03	super_enhancer	promoter	ZNHIT6
chr1	41505000	chr1	88945000	3.36E-03	super_enhancer	promoter	RN7SL583P
chr1	41505000	chr1	110625000	3.73E-03	super_enhancer	promoter	LINC01397
chr1	41505000	chr1	185125000	1.99E-03	super_enhancer	promoter	SWT1
chr1	41515000	chr1	46155000	4.11E-02	super_enhancer	promoter	GPBP1L1
chr1	41515000	chr1	111175000	3.27E-03	super_enhancer	promoter	KCNA2

chr1	41515000	chr1	112015000	3.08E-03	super_enhancer	promoter	C1orf162
chr1	41515000	chr1	147735000	2.32E-03	super_enhancer	promoter	RNU1-120P
chr1	41515000	chr1	179335000	2.40E-03	super_enhancer	promoter	AXDND1
chr1	41515000	chr1	228195000	3.07E-03	super_enhancer	promoter	WNT3A
chr1	41515000	chr1	228285000	3.25E-03	super_enhancer	promoter	MIR3620
chr1	41515000	chr1	228395000	2.69E-03	super_enhancer	promoter	OBSCN
chr1	41895000	chr1	43355000	3.01E-02	super_enhancer	promoter	RP11-342M1.7
chr1	41895000	chr1	62995000	1.17E-02	super_enhancer	promoter	RP11-293K19.1
chr1	41895000	chr1	117665000	3.47E-03	super_enhancer	promoter	TRIM45
chr1	41895000	chr1	207325000	2.72E-03	super_enhancer	promoter	RP11-164O23.5
chr1	41905000	chr1	54485000	2.35E-02	super_enhancer	promoter	LDLRAD1
chr1	41905000	chr1	88275000	5.83E-03	super_enhancer	promoter	RP11-60A14.1
chr1	41905000	chr1	93745000	5.83E-03	super_enhancer	promoter	RP4-717I23.2
chr1	41905000	chr1	114415000	3.00E-03	super_enhancer	promoter	PTPN22
chr1	41915000	chr1	47435000	4.84E-02	super_enhancer	promoter	CYP4A26P
chr1	41915000	chr1	53925000	2.87E-02	super_enhancer	promoter	DMRTB1
chr1	41915000	chr1	168465000	2.52E-03	super_enhancer	promoter	RP5-968D22.3
chr1	41915000	chr1	209345000	3.42E-03	super_enhancer	promoter	RP11-385M4.2
chr1	41915000	chr1	224805000	4.29E-03	super_enhancer	promoter	RP11-100E13.1
chr1	41915000	chr1	226375000	4.67E-03	super_enhancer	promoter	ACBD3
chr1	41915000	chr1	238655000	2.52E-03	super_enhancer	promoter	KRT18P32
chr1	42225000	chr1	43445000	3.81E-02	promoter	super_enhancer	AL451006.1
chr1	42225000	chr1	43535000	3.64E-02	promoter	super_enhancer	AL451006.1
chr1	42225000	chr1	109895000	2.98E-03	promoter	super_enhancer	AL451006.1

chr1	42605000	chr1	43215000	1.85E-03	promoter	super_enhancer	RP11-319C21.1
chr1	42605000	chr1	55445000	2.27E-02	promoter	super_enhancer	RP11-319C21.1
chr1	42605000	chr1	85315000	7.32E-03	promoter	super_enhancer	RP11-319C21.1
chr1	42805000	chr1	42935000	1.62E-02	promoter	super_enhancer	RP11-223A3.1
chr1	42805000	chr1	42955000	3.74E-02	promoter	super_enhancer	RP11-223A3.1
chr1	42805000	chr1	43525000	3.01E-02	promoter	super_enhancer	RP11-223A3.1
chr1	42805000	chr1	85315000	8.50E-03	promoter	super_enhancer	RP11-223A3.1
chr1	42805000	chr1	109825000	3.80E-03	promoter	super_enhancer	RP11-223A3.1
chr1	42805000	chr1	150605000	2.99E-03	promoter	super_enhancer	RP11-223A3.1
chr1	42805000	chr1	235075000	4.46E-03	promoter	super_enhancer	RP11-223A3.1
chr1	42845000	chr1	43215000	2.06E-02	promoter	super_enhancer	RIMKLA
chr1	42845000	chr1	109795000	4.67E-03	promoter	super_enhancer	RIMKLA
chr1	42845000	chr1	150605000	3.01E-03	promoter	super_enhancer	RIMKLA
chr1	42845000	chr1	156495000	4.53E-03	promoter	super_enhancer	RIMKLA
chr1	42925000	chr1	158725000	2.48E-03	super_enhancer	promoter	OR6K6
chr1	42925000	chr1	203225000	2.41E-03	super_enhancer	promoter	NPM1P40
chr1	42925000	chr1	204245000	3.66E-03	super_enhancer	promoter	RP11-203F10.5
chr1	42935000	chr1	48015000	4.49E-02	super_enhancer	promoter	ATP6V0E1P4
chr1	42935000	chr1	109655000	4.83E-03	super_enhancer	promoter	KIAA1324
chr1	42945000	chr1	53175000	2.58E-02	super_enhancer	promoter	NDUFS5P3
chr1	42945000	chr1	88945000	4.29E-03	super_enhancer	promoter	RN7SL583P
chr1	42945000	chr1	149285000	3.12E-03	super_enhancer	promoter	RP11-403I13.7
chr1	42945000	chr1	209145000	3.20E-03	super_enhancer	promoter	RP5-1051D14.1
chr1	42955000	chr1	52345000	1.64E-02	super_enhancer	promoter	NRD1

chr1	42955000	chr1	100435000	3.55E-03	super_enhancer	promoter	RP5-884G6.2
chr1	42965000	chr1	47185000	3.42E-02	super_enhancer	promoter	EFCAB14
chr1	42965000	chr1	51245000	1.63E-02	super_enhancer	promoter	PHBP12
chr1	42965000	chr1	67995000	9.34E-03	super_enhancer	promoter	RP4-763G1.2
chr1	42965000	chr1	233585000	1.59E-03	super_enhancer	promoter	RNU4-77P
chr1	42975000	chr1	44605000	1.13E-02	super_enhancer	promoter	KLF18
chr1	42975000	chr1	51795000	2.34E-02	super_enhancer	promoter	TTC39A-AS1
chr1	43205000	chr1	53405000	2.73E-02	super_enhancer	promoter	MIR1273G
chr1	43205000	chr1	109255000	4.31E-03	super_enhancer	promoter	FNDC7
chr1	43205000	chr1	110355000	4.17E-03	super_enhancer	promoter	NDUFA5P10
chr1	43205000	chr1	151255000	4.48E-03	super_enhancer	promoter	RP11-126K1.2
chr1	43205000	chr1	157015000	3.59E-03	super_enhancer	promoter	ARHGEF11
chr1	43205000	chr1	162795000	3.06E-03	super_enhancer	promoter	RP11-331H2.4
chr1	43205000	chr1	201925000	3.53E-03	super_enhancer	promoter	TIMM17A
chr1	43205000	chr1	234765000	4.19E-03	super_enhancer	promoter	LINC00184
chr1	43215000	chr1	71515000	1.28E-02	super_enhancer	promoter	ZRANB2-AS1
chr1	43215000	chr1	180925000	3.71E-03	super_enhancer	promoter	KIAA1614-AS1
chr1	43215000	chr1	205425000	4.50E-03	super_enhancer	promoter	RP11-576D8.4
chr1	43215000	chr1	206305000	3.33E-03	super_enhancer	promoter	C1orf186
chr1	43215000	chr1	211385000	3.84E-03	super_enhancer	promoter	AC092017.1
chr1	43215000	chr1	234745000	3.54E-03	super_enhancer	promoter	IRF2BP2
chr1	43225000	chr1	65885000	1.40E-02	super_enhancer	promoter	LEPROT
chr1	43225000	chr1	78695000	5.75E-03	super_enhancer	promoter	MGC27382
chr1	43225000	chr1	95785000	5.71E-03	super_enhancer	promoter	RP4-586O15.1

chr1	43225000	chr1	153015000	2.82E-03	super_enhancer	promoter	SPRR2D
chr1	43235000	chr1	55505000	1.90E-02	super_enhancer	promoter	PCSK9
chr1	43235000	chr1	64255000	1.09E-02	super_enhancer	promoter	RP4-597J3.1
chr1	43235000	chr1	93665000	5.05E-03	super_enhancer	promoter	RP4-612C19.2
chr1	43235000	chr1	94345000	5.61E-03	super_enhancer	promoter	DNTTIP2
chr1	43235000	chr1	102855000	2.97E-03	super_enhancer	promoter	RP11-202K23.1
chr1	43355000	chr1	158035000	3.64E-03	promoter	super_enhancer	RP11-342M1.7
chr1	43425000	chr1	46015000	2.71E-04	super_enhancer	promoter	AKR1A1
chr1	43425000	chr1	53795000	2.13E-02	super_enhancer	promoter	LRP8
chr1	43425000	chr1	54205000	2.48E-02	super_enhancer	promoter	GLIS1
chr1	43425000	chr1	87595000	7.78E-03	super_enhancer	promoter	LINC01140
chr1	43425000	chr1	150025000	2.43E-03	super_enhancer	promoter	RP11-458I7.1
chr1	43425000	chr1	204655000	3.30E-03	super_enhancer	promoter	LRRN2
chr1	43435000	chr1	95585000	5.64E-03	super_enhancer	promoter	RP11-57H12.2
chr1	43435000	chr1	114575000	3.33E-03	super_enhancer	promoter	RP4-590F24.2
chr1	43435000	chr1	150255000	3.69E-03	super_enhancer	promoter	CIART
chr1	43435000	chr1	169075000	3.05E-03	super_enhancer	promoter	ATP1B1
chr1	43435000	chr1	239415000	2.64E-03	super_enhancer	promoter	RP11-544D21.2
chr1	43445000	chr1	47875000	4.76E-02	super_enhancer	promoter	LINC01389
chr1	43445000	chr1	52345000	2.40E-02	super_enhancer	promoter	NRD1
chr1	43445000	chr1	54205000	2.52E-02	super_enhancer	promoter	GLIS1
chr1	43445000	chr1	78555000	6.82E-03	super_enhancer	promoter	RNU6-1102P
chr1	43445000	chr1	113435000	3.45E-03	super_enhancer	promoter	RP3-522D1.2
chr1	43455000	chr1	51795000	2.90E-02	super_enhancer	promoter	TTC39A-AS1

chr1	43455000	chr1	53175000	2.99E-02	super_enhancer	promoter	NDUFS5P3
chr1	43455000	chr1	64495000	1.19E-02	super_enhancer	promoter	RNU6-809P
chr1	43455000	chr1	109235000	3.87E-03	super_enhancer	promoter	PRPF38B
chr1	43455000	chr1	171795000	3.44E-03	super_enhancer	promoter	RP4-612B18.1
chr1	43455000	chr1	204465000	4.06E-03	super_enhancer	promoter	PIK3C2B
chr1	43465000	chr1	51425000	3.19E-02	super_enhancer	promoter	CDKN2C
chr1	43465000	chr1	154975000	3.89E-03	super_enhancer	promoter	ZBTB7B
chr1	43465000	chr1	155555000	3.77E-03	super_enhancer	promoter	DAP3P1
chr1	43465000	chr1	205055000	3.59E-03	super_enhancer	promoter	TMEM81
chr1	43465000	chr1	223045000	2.05E-03	super_enhancer	promoter	DISP1
chr1	43475000	chr1	85515000	6.25E-03	super_enhancer	promoter	MCOLN3
chr1	43475000	chr1	161705000	2.88E-03	super_enhancer	promoter	RN7SL466P
chr1	43485000	chr1	58515000	1.50E-02	super_enhancer	promoter	HNRNPA1P6
chr1	43485000	chr1	161135000	3.00E-03	super_enhancer	promoter	PPOX
chr1	43485000	chr1	171455000	2.83E-03	super_enhancer	promoter	PRRC2C
chr1	43485000	chr1	236645000	3.31E-03	super_enhancer	promoter	ENO1P1
chr1	43495000	chr1	53795000	2.35E-02	super_enhancer	promoter	LRP8
chr1	43495000	chr1	57285000	2.00E-02	super_enhancer	promoter	C1orf168
chr1	43495000	chr1	174065000	3.70E-03	super_enhancer	promoter	RNU7-57P
chr1	43505000	chr1	52615000	2.65E-02	super_enhancer	promoter	RN7SL788P
chr1	43505000	chr1	157895000	3.79E-03	super_enhancer	promoter	RP11-451O13.1
chr1	43525000	chr1	51425000	2.49E-02	super_enhancer	promoter	CDKN2C
chr1	43525000	chr1	54485000	1.85E-02	super_enhancer	promoter	LDLRAD1
chr1	43525000	chr1	59485000	1.52E-02	super_enhancer	promoter	LINC01358

chr1	43525000	chr1	62095000	1.43E-02	super_enhancer	promoter	AC099791.1
chr1	43525000	chr1	74215000	6.67E-03	super_enhancer	promoter	RNA5SP50
chr1	43535000	chr1	54155000	2.92E-02	super_enhancer	promoter	RNU7-95P
chr1	43535000	chr1	85465000	7.68E-03	super_enhancer	promoter	WDR63
chr1	43535000	chr1	97145000	4.27E-03	super_enhancer	promoter	RPL7P9
chr1	43535000	chr1	204915000	3.35E-03	super_enhancer	promoter	RP11-494K3.2
chr1	43535000	chr1	240135000	2.84E-03	super_enhancer	promoter	RP11-438O11.1
chr1	43535000	chr1	244615000	3.53E-03	super_enhancer	promoter	ADSS
chr1	43545000	chr1	44445000	2.51E-02	super_enhancer	promoter	B4GALT2
chr1	43545000	chr1	44605000	4.19E-02	super_enhancer	promoter	KLF18
chr1	43545000	chr1	53705000	2.02E-02	super_enhancer	promoter	MAGOH
chr1	43545000	chr1	199985000	3.57E-03	super_enhancer	promoter	RNU6-609P
chr1	43545000	chr1	221725000	2.49E-03	super_enhancer	promoter	RP11-103C3.2
chr1	43555000	chr1	50865000	2.38E-02	super_enhancer	promoter	HMGB1P45
chr1	43555000	chr1	53815000	1.91E-02	super_enhancer	promoter	RP11-117D22.1
chr1	43555000	chr1	203055000	2.67E-03	super_enhancer	promoter	MYOG
chr1	43555000	chr1	229695000	2.66E-03	super_enhancer	promoter	ABCB10
chr1	43735000	chr1	66275000	1.37E-02	promoter	super_enhancer	TMEM125
chr1	43825000	chr1	66275000	1.41E-02	promoter	super_enhancer	RP1-92O14.3
chr1	43835000	chr1	155005000	3.44E-03	promoter	super_enhancer	ELOVL1
chr1	43835000	chr1	204425000	3.81E-03	promoter	super_enhancer	ELOVL1
chr1	43855000	chr1	172905000	2.97E-03	promoter	super_enhancer	MED8
chr1	43915000	chr1	50975000	2.05E-02	promoter	super_enhancer	SZT2-AS1
chr1	43915000	chr1	109275000	3.13E-03	promoter	super_enhancer	SZT2-AS1

chr1	44115000	chr1	67735000	1.20E-02	promoter	super_enhancer	KDM4A
chr1	44175000	chr1	164745000	2.59E-03	promoter	super_enhancer	KDM4A-AS1
chr1	44175000	chr1	181115000	3.44E-03	promoter	super_enhancer	KDM4A-AS1
chr1	44435000	chr1	94625000	6.30E-03	promoter	super_enhancer	DPH2
chr1	44445000	chr1	181075000	3.70E-03	promoter	super_enhancer	B4GALT2
chr1	44495000	chr1	202015000	4.59E-03	promoter	super_enhancer	RP5-1198O20.4
chr1	44555000	chr1	44805000	3.88E-02	promoter	super_enhancer	RP5-1198O20.5
chr1	44555000	chr1	67725000	1.22E-02	promoter	super_enhancer	RP5-1198O20.5
chr1	44555000	chr1	94645000	5.49E-03	promoter	super_enhancer	RP5-1198O20.5
chr1	44555000	chr1	151195000	3.79E-03	promoter	super_enhancer	RP5-1198O20.5
chr1	44585000	chr1	85335000	8.91E-03	promoter	super_enhancer	KLF17
chr1	44605000	chr1	50975000	1.81E-02	promoter	super_enhancer	KLF18
chr1	44655000	chr1	228145000	3.89E-03	promoter	super_enhancer	RP5-891H21.5
chr1	44805000	chr1	50865000	3.18E-02	super_enhancer	promoter	HMGB1P45
chr1	44805000	chr1	51625000	3.44E-02	super_enhancer	promoter	CFL1P2
chr1	44805000	chr1	53175000	3.15E-02	super_enhancer	promoter	NDUFS5P3
chr1	44805000	chr1	53795000	2.33E-02	super_enhancer	promoter	LRP8
chr1	44805000	chr1	54485000	2.28E-02	super_enhancer	promoter	LDLRAD1
chr1	44805000	chr1	93475000	5.91E-03	super_enhancer	promoter	RNU6-210P
chr1	44805000	chr1	117285000	4.27E-03	super_enhancer	promoter	NEFHP1
chr1	44805000	chr1	154525000	3.39E-03	super_enhancer	promoter	UBE2Q1-AS1
chr1	44805000	chr1	155815000	3.59E-03	super_enhancer	promoter	RP11-101O6.2
chr1	44805000	chr1	213225000	3.45E-03	super_enhancer	promoter	RPS6KC1
chr1	44805000	chr1	219935000	2.42E-03	super_enhancer	promoter	RNA5SP76

chr1	45205000	chr1	85295000	6.23E-03	promoter	super_enhancer	KIF2C
chr1	45455000	chr1	109815000	4.21E-03	promoter	super_enhancer	RP5-882O7.4
chr1	45455000	chr1	155125000	3.59E-03	promoter	super_enhancer	RP5-882O7.4
chr1	45455000	chr1	181075000	3.00E-03	promoter	super_enhancer	RP5-882O7.4
chr1	45455000	chr1	204115000	3.90E-03	promoter	super_enhancer	RP5-882O7.4
chr1	45805000	chr1	212595000	2.41E-03	promoter	super_enhancer	TOE1
chr1	46015000	chr1	94335000	6.28E-03	promoter	super_enhancer	AKR1A1
chr1	46015000	chr1	203515000	3.93E-03	promoter	super_enhancer	AKR1A1
chr1	46645000	chr1	50975000	3.75E-02	promoter	super_enhancer	AL672043.1
chr1	46805000	chr1	153565000	2.91E-03	promoter	super_enhancer	NSUN4
chr1	46805000	chr1	224255000	2.95E-03	promoter	super_enhancer	NSUN4
chr1	46835000	chr1	66265000	1.27E-02	promoter	super_enhancer	RP4-603I14.3
chr1	46915000	chr1	109815000	5.59E-03	promoter	super_enhancer	LINC01398
chr1	46915000	chr1	153605000	4.55E-03	promoter	super_enhancer	LINC01398
chr1	47135000	chr1	55445000	3.10E-02	promoter	super_enhancer	TEX38
chr1	47135000	chr1	156905000	4.83E-03	promoter	super_enhancer	TEX38
chr1	47435000	chr1	156125000	4.57E-03	promoter	super_enhancer	CYP4A26P
chr1	47435000	chr1	164765000	3.33E-03	promoter	super_enhancer	CYP4A26P
chr1	47645000	chr1	205305000	4.48E-03	promoter	super_enhancer	CYP4A22-AS1
chr1	47965000	chr1	204455000	3.21E-03	promoter	super_enhancer	RPL21P24
chr1	48695000	chr1	67705000	1.38E-02	promoter	super_enhancer	RP5-1024N4.4
chr1	50865000	chr1	94335000	5.58E-03	promoter	super_enhancer	HMGB1P45
chr1	50975000	chr1	59755000	1.20E-02	super_enhancer	promoter	RP11-467C18.1
chr1	50975000	chr1	63155000	1.21E-02	super_enhancer	promoter	DOCK7

chr1	50975000	chr1	63605000	1.19E-02	super_enhancer	promoter	RP11-335E6.2
chr1	50975000	chr1	64495000	1.02E-02	super_enhancer	promoter	RNU6-809P
chr1	50975000	chr1	207925000	1.68E-03	super_enhancer	promoter	CD46
chr1	51245000	chr1	67725000	1.38E-02	promoter	super_enhancer	PHBP12
chr1	51245000	chr1	225445000	1.95E-03	promoter	super_enhancer	PHBP12
chr1	51425000	chr1	109805000	5.40E-03	promoter	super_enhancer	CDKN2C
chr1	51625000	chr1	63665000	2.12E-02	promoter	super_enhancer	CFL1P2
chr1	51795000	chr1	156135000	4.41E-03	promoter	super_enhancer	TTC39A-AS1
chr1	51985000	chr1	55425000	3.30E-02	promoter	super_enhancer	EPS15
chr1	51985000	chr1	55445000	4.67E-02	promoter	super_enhancer	EPS15
chr1	52005000	chr1	94625000	6.03E-03	promoter	super_enhancer	RNU6-1281P
chr1	52005000	chr1	234965000	3.04E-03	promoter	super_enhancer	RNU6-1281P
chr1	52175000	chr1	109905000	3.49E-03	promoter	super_enhancer	GAPDHP51
chr1	52325000	chr1	63665000	2.01E-02	promoter	super_enhancer	TSEN15P2
chr1	52325000	chr1	94635000	6.40E-03	promoter	super_enhancer	TSEN15P2
chr1	52345000	chr1	230075000	2.67E-03	promoter	super_enhancer	NRD1
chr1	52445000	chr1	225465000	2.96E-03	promoter	super_enhancer	RP11-91A18.1
chr1	52615000	chr1	109825000	4.09E-03	promoter	super_enhancer	RN7SL788P
chr1	52615000	chr1	204315000	3.14E-03	promoter	super_enhancer	RN7SL788P
chr1	52645000	chr1	67705000	1.26E-02	promoter	super_enhancer	PDCL3P6
chr1	53175000	chr1	153565000	3.78E-03	promoter	super_enhancer	NDUFS5P3
chr1	53175000	chr1	234735000	2.98E-03	promoter	super_enhancer	NDUFS5P3
chr1	53385000	chr1	225445000	2.19E-03	promoter	super_enhancer	RP4-631H13.6
chr1	53685000	chr1	85305000	8.15E-03	promoter	super_enhancer	RP5-1024G6.7

chr1	53685000	chr1	156745000	3.58E-03	promoter	super_enhancer	RP5-1024G6.7
chr1	53685000	chr1	156905000	4.74E-03	promoter	super_enhancer	RP5-1024G6.7
chr1	53685000	chr1	172925000	2.81E-03	promoter	super_enhancer	RP5-1024G6.7
chr1	53685000	chr1	212485000	3.45E-03	promoter	super_enhancer	RP5-1024G6.7
chr1	53795000	chr1	94625000	6.02E-03	promoter	super_enhancer	LRP8
chr1	53795000	chr1	172905000	2.39E-03	promoter	super_enhancer	LRP8
chr1	53835000	chr1	204275000	3.04E-03	promoter	super_enhancer	RP11-117D22.2
chr1	54155000	chr1	152035000	3.98E-03	promoter	super_enhancer	RNU7-95P
chr1	54205000	chr1	67735000	2.31E-02	promoter	super_enhancer	GLIS1
chr1	54205000	chr1	204305000	3.64E-03	promoter	super_enhancer	GLIS1
chr1	54305000	chr1	202045000	3.45E-03	promoter	super_enhancer	NDC1
chr1	54555000	chr1	204105000	2.77E-03	promoter	super_enhancer	RP4-758J24.4
chr1	55425000	chr1	146715000	1.72E-03	super_enhancer	promoter	FMO5
chr1	55425000	chr1	154525000	2.58E-03	super_enhancer	promoter	UBE2Q1-AS1
chr1	55425000	chr1	214205000	2.16E-03	super_enhancer	promoter	PROX1-AS1
chr1	55435000	chr1	69715000	1.24E-02	super_enhancer	promoter	RP11-74K19.1
chr1	55435000	chr1	86495000	6.01E-03	super_enhancer	promoter	RP4-682E18.1
chr1	55435000	chr1	93545000	5.93E-03	super_enhancer	promoter	MTF2
chr1	55435000	chr1	111725000	3.87E-03	super_enhancer	promoter	RP5-1180E21.4
chr1	55435000	chr1	185305000	2.92E-03	super_enhancer	promoter	GS1-279B7.1
chr1	55445000	chr1	111725000	4.78E-03	super_enhancer	promoter	RP5-1180E21.4
chr1	55445000	chr1	240715000	3.87E-03	super_enhancer	promoter	RP11-467I20.4
chr1	55465000	chr1	67735000	2.71E-02	promoter	super_enhancer	BSND
chr1	55465000	chr1	202075000	4.03E-03	promoter	super_enhancer	BSND

chr1	55465000	chr1	228145000	4.84E-03	promoter	super_enhancer	BSND
chr1	55505000	chr1	63665000	2.42E-02	promoter	super_enhancer	PCSK9
chr1	55505000	chr1	66275000	2.00E-02	promoter	super_enhancer	PCSK9
chr1	55505000	chr1	204435000	3.31E-03	promoter	super_enhancer	PCSK9
chr1	56675000	chr1	85305000	6.76E-03	promoter	super_enhancer	RP4-710M16.1
chr1	56675000	chr1	172935000	1.68E-03	promoter	super_enhancer	RP4-710M16.1
chr1	56725000	chr1	112705000	4.11E-03	promoter	super_enhancer	RP11-504A18.1
chr1	56725000	chr1	224255000	2.93E-03	promoter	super_enhancer	RP11-504A18.1
chr1	57085000	chr1	115185000	3.40E-03	promoter	super_enhancer	RP11-377K22.2
chr1	57285000	chr1	158015000	2.88E-03	promoter	super_enhancer	C1orf168
chr1	58325000	chr1	63665000	2.77E-02	promoter	super_enhancer	DAB1-AS1
chr1	58325000	chr1	67695000	1.59E-02	promoter	super_enhancer	DAB1-AS1
chr1	58325000	chr1	150615000	2.60E-03	promoter	super_enhancer	DAB1-AS1
chr1	59465000	chr1	66265000	1.83E-02	promoter	super_enhancer	PHBP3
chr1	59485000	chr1	94335000	7.79E-03	promoter	super_enhancer	LINC01358
chr1	59545000	chr1	230075000	2.51E-03	promoter	super_enhancer	RP11-145M4.2
chr1	59755000	chr1	66285000	1.68E-02	promoter	super_enhancer	RP11-467C18.1
chr1	62095000	chr1	66285000	3.88E-02	promoter	super_enhancer	AC099791.1
chr1	62505000	chr1	155135000	3.97E-03	promoter	super_enhancer	LAMTOR5P1
chr1	62655000	chr1	66285000	4.87E-02	promoter	super_enhancer	PIGPP2
chr1	62655000	chr1	202085000	4.39E-03	promoter	super_enhancer	PIGPP2
chr1	62675000	chr1	66295000	4.17E-02	promoter	super_enhancer	RP5-1155K23.4
chr1	62675000	chr1	67685000	2.93E-02	promoter	super_enhancer	RP5-1155K23.4
chr1	62765000	chr1	224265000	2.67E-03	promoter	super_enhancer	RNU6-371P

chr1	63605000	chr1	67695000	4.12E-02	promoter	super_enhancer	RP11-335E6.2
chr1	63605000	chr1	67715000	3.08E-02	promoter	super_enhancer	RP11-335E6.2
chr1	63605000	chr1	67725000	3.86E-02	promoter	super_enhancer	RP11-335E6.2
chr1	63605000	chr1	85295000	1.09E-02	promoter	super_enhancer	RP11-335E6.2
chr1	63605000	chr1	156465000	3.84E-03	promoter	super_enhancer	RP11-335E6.2
chr1	63605000	chr1	235055000	2.79E-03	promoter	super_enhancer	RP11-335E6.2
chr1	63665000	chr1	79215000	1.24E-02	super_enhancer	promoter	RP4-641G12.4
chr1	63665000	chr1	85465000	1.21E-02	super_enhancer	promoter	WDR63
chr1	63665000	chr1	161195000	3.70E-03	super_enhancer	promoter	TOMM40L
chr1	63665000	chr1	220745000	2.11E-03	super_enhancer	promoter	RN7SL464P
chr1	63665000	chr1	240175000	2.84E-03	super_enhancer	promoter	RPS7P5
chr1	63665000	chr1	240715000	3.21E-03	super_enhancer	promoter	RP11-467I20.4
chr1	63715000	chr1	67705000	4.23E-02	promoter	super_enhancer	RP11-335E6.3
chr1	63715000	chr1	109275000	4.13E-03	promoter	super_enhancer	RP11-335E6.3
chr1	64935000	chr1	181135000	2.29E-03	promoter	super_enhancer	CACHD1
chr1	65045000	chr1	181085000	2.69E-03	promoter	super_enhancer	MIR4794
chr1	65045000	chr1	202015000	2.76E-03	promoter	super_enhancer	MIR4794
chr1	65525000	chr1	85315000	1.19E-02	promoter	super_enhancer	MIR3671
chr1	65545000	chr1	66285000	1.41E-02	promoter	super_enhancer	RP4-535B20.2
chr1	65765000	chr1	85295000	1.21E-02	promoter	super_enhancer	COX6CP13
chr1	65885000	chr1	85305000	1.18E-02	promoter	super_enhancer	LEPROT
chr1	65885000	chr1	109875000	5.78E-03	promoter	super_enhancer	LEPROT
chr1	65885000	chr1	204095000	3.98E-03	promoter	super_enhancer	LEPROT
chr1	66255000	chr1	73365000	8.19E-03	super_enhancer	promoter	RP4-660H19.1

chr1	66265000	chr1	70385000	2.63E-02	super_enhancer	promoter	PIN1P1
chr1	66265000	chr1	81545000	9.08E-03	super_enhancer	promoter	MTND2P30
chr1	66275000	chr1	78355000	1.80E-02	super_enhancer	promoter	NEXN-AS1
chr1	66275000	chr1	101185000	6.38E-03	super_enhancer	promoter	VCAM1
chr1	66275000	chr1	110025000	6.26E-03	super_enhancer	promoter	ATXN7L2
chr1	66275000	chr1	151105000	3.11E-03	super_enhancer	promoter	RP11-68I18.10
chr1	66275000	chr1	159945000	2.72E-03	super_enhancer	promoter	RP11-48O20.5
chr1	66275000	chr1	172855000	2.53E-03	super_enhancer	promoter	AIMP1P2
chr1	66285000	chr1	74665000	1.25E-02	super_enhancer	promoter	LRRIQ3
chr1	66285000	chr1	197725000	2.12E-03	super_enhancer	promoter	RP11-448G4.4
chr1	66295000	chr1	86065000	9.88E-03	super_enhancer	promoter	RP11-290M5.2
chr1	67685000	chr1	93435000	8.38E-03	super_enhancer	promoter	RNU6-970P
chr1	67685000	chr1	106625000	2.89E-03	super_enhancer	promoter	RP5-947P14.2
chr1	67695000	chr1	109585000	6.58E-03	super_enhancer	promoter	WDR47
chr1	67705000	chr1	95745000	7.95E-03	super_enhancer	promoter	RP11-57H12.5
chr1	67715000	chr1	76255000	1.40E-02	super_enhancer	promoter	SNORD45B
chr1	67715000	chr1	155715000	2.31E-03	super_enhancer	promoter	MSTO2P
chr1	67715000	chr1	161645000	1.21E-03	super_enhancer	promoter	RP11-25K21.1
chr1	67725000	chr1	85515000	1.18E-02	super_enhancer	promoter	MCOLN3
chr1	67725000	chr1	86045000	1.22E-02	super_enhancer	promoter	DDAH1
chr1	67725000	chr1	95585000	8.72E-03	super_enhancer	promoter	RP11-57H12.2
chr1	67725000	chr1	154245000	3.32E-03	super_enhancer	promoter	HAX1
chr1	67725000	chr1	203145000	3.44E-03	super_enhancer	promoter	MYBPH
chr1	67735000	chr1	75165000	2.14E-02	super_enhancer	promoter	RP11-17E13.2

chr1	67735000	chr1	119545000	5.74E-03	super_enhancer	promoter	RP4-712E4.1
chr1	68515000	chr1	204275000	2.44E-03	promoter	super_enhancer	ARL5AP3
chr1	68945000	chr1	156745000	2.49E-03	promoter	super_enhancer	RP4-694A7.2
chr1	70075000	chr1	109785000	4.67E-03	promoter	super_enhancer	SGOL1P1
chr1	73445000	chr1	94625000	6.92E-03	promoter	super_enhancer	RP11-25L19.1
chr1	74665000	chr1	207025000	1.91E-03	promoter	super_enhancer	LRRIQ3
chr1	75165000	chr1	202025000	2.50E-03	promoter	super_enhancer	RP11-17E13.2
chr1	77245000	chr1	94635000	1.47E-02	promoter	super_enhancer	RP11-415A20.1
chr1	77595000	chr1	155985000	1.64E-03	promoter	super_enhancer	RP4-706A16.3
chr1	77815000	chr1	109805000	1.02E-02	promoter	super_enhancer	RP11-375A5.1
chr1	77815000	chr1	202045000	3.14E-03	promoter	super_enhancer	RP11-375A5.1
chr1	78245000	chr1	85315000	2.69E-02	promoter	super_enhancer	FAM73A
chr1	78245000	chr1	94325000	1.14E-02	promoter	super_enhancer	FAM73A
chr1	78245000	chr1	94625000	1.32E-02	promoter	super_enhancer	FAM73A
chr1	78245000	chr1	212485000	2.89E-03	promoter	super_enhancer	FAM73A
chr1	78445000	chr1	115185000	6.25E-03	promoter	super_enhancer	FUBP1
chr1	78445000	chr1	230085000	2.46E-03	promoter	super_enhancer	FUBP1
chr1	78695000	chr1	109815000	5.86E-03	promoter	super_enhancer	MGC27382
chr1	79115000	chr1	85285000	2.13E-02	promoter	super_enhancer	IFI44
chr1	79115000	chr1	94335000	1.16E-02	promoter	super_enhancer	IFI44
chr1	79115000	chr1	206905000	2.70E-03	promoter	super_enhancer	IFI44
chr1	79215000	chr1	85325000	1.96E-02	promoter	super_enhancer	RP4-641G12.4
chr1	81065000	chr1	158015000	1.93E-03	promoter	super_enhancer	AL136234.1
chr1	81565000	chr1	85305000	3.58E-02	promoter	super_enhancer	RPL7P10

chr1	81565000	chr1	202025000	2.70E-03	promoter	super_enhancer	RPL7P10
chr1	82025000	chr1	94325000	1.08E-02	promoter	super_enhancer	MED28P8
chr1	82455000	chr1	93705000	7.93E-03	promoter	super_enhancer	AC113949.1
chr1	83125000	chr1	234975000	1.77E-03	promoter	super_enhancer	AL357973.1
chr1	84465000	chr1	156125000	3.46E-03	promoter	super_enhancer	TTLL7
chr1	84865000	chr1	205315000	2.72E-03	promoter	super_enhancer	UOX
chr1	84865000	chr1	206895000	2.40E-03	promoter	super_enhancer	UOX
chr1	84945000	chr1	94635000	1.92E-02	promoter	super_enhancer	RPF1
chr1	84945000	chr1	204315000	2.84E-03	promoter	super_enhancer	RPF1
chr1	85285000	chr1	88965000	4.43E-02	super_enhancer	promoter	RP11-76N22.1
chr1	85285000	chr1	98435000	1.14E-02	super_enhancer	promoter	RP11-490G2.2
chr1	85285000	chr1	173605000	2.39E-03	super_enhancer	promoter	RP11-360D2.1
chr1	85285000	chr1	240715000	3.00E-03	super_enhancer	promoter	RP11-467I20.4
chr1	85295000	chr1	92655000	2.80E-02	super_enhancer	promoter	RP4-621B10.8
chr1	85295000	chr1	164525000	2.46E-03	super_enhancer	promoter	PBX1
chr1	85295000	chr1	182405000	2.71E-03	super_enhancer	promoter	RP1-223H12.5
chr1	85305000	chr1	88945000	3.44E-02	super_enhancer	promoter	RN7SL583P
chr1	85305000	chr1	89295000	2.60E-02	super_enhancer	promoter	TCEB1P19
chr1	85305000	chr1	205595000	2.36E-03	super_enhancer	promoter	RP11-6B6.3
chr1	85305000	chr1	237145000	2.97E-03	super_enhancer	promoter	RPL35P1
chr1	85315000	chr1	93665000	2.48E-02	super_enhancer	promoter	RP4-612C19.2
chr1	85315000	chr1	103575000	1.04E-02	super_enhancer	promoter	COL11A1
chr1	85315000	chr1	106435000	1.01E-02	super_enhancer	promoter	RP11-90H3.2
chr1	85325000	chr1	101185000	1.06E-02	super_enhancer	promoter	VCAM1

chr1	85325000	chr1	154955000	2.75E-03	super_enhancer	promoter	FLAD1
chr1	85325000	chr1	242935000	1.94E-03	super_enhancer	promoter	RSL24D1P4
chr1	85335000	chr1	91965000	2.65E-02	super_enhancer	promoter	CDC7
chr1	85335000	chr1	100495000	1.45E-02	super_enhancer	promoter	RP4-714D9.5
chr1	85335000	chr1	150255000	4.95E-03	super_enhancer	promoter	CIART
chr1	85335000	chr1	151765000	4.36E-03	super_enhancer	promoter	TDRKH
chr1	85335000	chr1	155815000	4.46E-03	super_enhancer	promoter	RP11-101O6.2
chr1	85515000	chr1	204285000	2.49E-03	promoter	super_enhancer	MCOLN3
chr1	86065000	chr1	153565000	3.46E-03	promoter	super_enhancer	RP11-290M5.2
chr1	86065000	chr1	156465000	4.51E-03	promoter	super_enhancer	RP11-290M5.2
chr1	86175000	chr1	156465000	4.81E-03	promoter	super_enhancer	ZNHIT6
chr1	86495000	chr1	109825000	7.00E-03	promoter	super_enhancer	RP4-682E18.1
chr1	87595000	chr1	94335000	3.13E-02	promoter	super_enhancer	LINC01140
chr1	88965000	chr1	93705000	3.16E-02	promoter	super_enhancer	RP11-76N22.1
chr1	89295000	chr1	156495000	2.78E-03	promoter	super_enhancer	TCEB1P19
chr1	89295000	chr1	172905000	1.53E-03	promoter	super_enhancer	TCEB1P19
chr1	90095000	chr1	94605000	3.03E-02	promoter	super_enhancer	RP11-413E1.4
chr1	90095000	chr1	109825000	1.14E-02	promoter	super_enhancer	RP11-413E1.4
chr1	90095000	chr1	205275000	3.29E-03	promoter	super_enhancer	RP11-413E1.4
chr1	90405000	chr1	94645000	3.25E-02	promoter	super_enhancer	RP11-302M6.5
chr1	90405000	chr1	109885000	1.15E-02	promoter	super_enhancer	RP11-302M6.5
chr1	90405000	chr1	159925000	2.67E-03	promoter	super_enhancer	RP11-302M6.5
chr1	90455000	chr1	156125000	5.55E-03	promoter	super_enhancer	RN7SKP272
chr1	91965000	chr1	94605000	4.99E-02	promoter	super_enhancer	CDC7

chr1	92065000	chr1	109805000	1.42E-02	promoter	super_enhancer	RP11-91I11.1
chr1	92415000	chr1	112705000	1.31E-02	promoter	super_enhancer	BRDT
chr1	92545000	chr1	156135000	4.83E-03	promoter	super_enhancer	BTBD8
chr1	92655000	chr1	115185000	1.17E-02	promoter	super_enhancer	RP4-621B10.8
chr1	92655000	chr1	204115000	4.02E-03	promoter	super_enhancer	RP4-621B10.8
chr1	92655000	chr1	209655000	3.26E-03	promoter	super_enhancer	RP4-621B10.8
chr1	92695000	chr1	94325000	6.02E-03	promoter	super_enhancer	ACTBP12
chr1	92695000	chr1	109895000	9.88E-03	promoter	super_enhancer	ACTBP12
chr1	93215000	chr1	93705000	3.10E-02	promoter	super_enhancer	RP11-330C7.4
chr1	93305000	chr1	109275000	1.25E-02	promoter	super_enhancer	SNORA66
chr1	93395000	chr1	109895000	1.20E-02	promoter	super_enhancer	RP11-386I23.1
chr1	93395000	chr1	164775000	2.45E-03	promoter	super_enhancer	RP11-386I23.1
chr1	93435000	chr1	234725000	2.76E-03	promoter	super_enhancer	RNU6-970P
chr1	93475000	chr1	94645000	3.13E-02	promoter	super_enhancer	RNU6-210P
chr1	93475000	chr1	205275000	3.31E-03	promoter	super_enhancer	RNU6-210P
chr1	93545000	chr1	150605000	3.15E-03	promoter	super_enhancer	MTF2
chr1	93645000	chr1	207015000	2.46E-03	promoter	super_enhancer	CCDC18
chr1	93665000	chr1	151195000	4.67E-03	promoter	super_enhancer	RP4-612C19.2
chr1	93665000	chr1	209655000	2.82E-03	promoter	super_enhancer	RP4-612C19.2
chr1	93695000	chr1	98515000	3.33E-02	super_enhancer	promoter	MIR137HG
chr1	93695000	chr1	100715000	1.65E-02	super_enhancer	promoter	DBT
chr1	93695000	chr1	221725000	2.02E-03	super_enhancer	promoter	RP11-103C3.2
chr1	93705000	chr1	101655000	1.91E-02	super_enhancer	promoter	RP5-1033H2.1
chr1	93705000	chr1	110355000	1.28E-02	super_enhancer	promoter	NDUFA5P10

chr1	93705000	chr1	146555000	2.81E-03	super_enhancer	promoter	RP11-325P15.2
chr1	93705000	chr1	147245000	3.99E-03	super_enhancer	promoter	GJA5
chr1	93705000	chr1	167905000	2.33E-03	super_enhancer	promoter	DCAF6
chr1	93705000	chr1	211555000	2.00E-03	super_enhancer	promoter	LINC00467
chr1	93745000	chr1	94325000	1.50E-02	promoter	super_enhancer	RP4-717I23.2
chr1	93955000	chr1	94325000	1.59E-02	promoter	super_enhancer	RNA5SP53
chr1	93955000	chr1	109875000	1.57E-02	promoter	super_enhancer	RNA5SP53
chr1	94325000	chr1	152635000	2.79E-03	super_enhancer	promoter	LCE2D
chr1	94335000	chr1	158725000	2.89E-03	super_enhancer	promoter	OR6K6
chr1	94345000	chr1	109805000	2.02E-02	promoter	super_enhancer	DNTTIP2
chr1	94345000	chr1	156115000	5.33E-03	promoter	super_enhancer	DNTTIP2
chr1	94375000	chr1	94625000	2.51E-02	promoter	super_enhancer	GCLM
chr1	94605000	chr1	170085000	1.77E-03	super_enhancer	promoter	SIGLEC30P
chr1	94605000	chr1	202115000	2.96E-03	super_enhancer	promoter	ARL8A
chr1	94605000	chr1	242155000	2.30E-03	super_enhancer	promoter	CFL1P4
chr1	94615000	chr1	110285000	1.10E-02	super_enhancer	promoter	GSTM3
chr1	94615000	chr1	151765000	3.43E-03	super_enhancer	promoter	TDRKH
chr1	94615000	chr1	212225000	2.47E-03	super_enhancer	promoter	RPL21P28
chr1	94615000	chr1	245185000	1.62E-03	super_enhancer	promoter	RNU6-1089P
chr1	94635000	chr1	101655000	2.26E-02	super_enhancer	promoter	RP5-1033H2.1
chr1	94635000	chr1	109235000	1.46E-02	super_enhancer	promoter	PRPF38B
chr1	94635000	chr1	146715000	3.34E-03	super_enhancer	promoter	FMO5
chr1	94645000	chr1	154245000	4.70E-03	super_enhancer	promoter	HAX1
chr1	94645000	chr1	166135000	2.10E-03	super_enhancer	promoter	RP11-9L18.3

chr1	94655000	chr1	245135000	2.13E-03	super_enhancer	promoter	RP11-156E8.1
chr1	95585000	chr1	109815000	1.70E-02	promoter	super_enhancer	RP11-57H12.2
chr1	95585000	chr1	212495000	2.27E-03	promoter	super_enhancer	RP11-57H12.2
chr1	95785000	chr1	172925000	2.64E-03	promoter	super_enhancer	RP4-586O15.1
chr1	96855000	chr1	152045000	3.62E-03	promoter	super_enhancer	RP4-736I12.1
chr1	96855000	chr1	234985000	2.39E-03	promoter	super_enhancer	RP4-736I12.1
chr1	98515000	chr1	109875000	1.76E-02	promoter	super_enhancer	MIR137HG
chr1	98675000	chr1	205295000	2.00E-03	promoter	super_enhancer	RP5-1070A16.1
chr1	100315000	chr1	109275000	1.64E-02	promoter	super_enhancer	AGL
chr1	100435000	chr1	109885000	2.37E-02	promoter	super_enhancer	RP5-884G6.2
chr1	100435000	chr1	112705000	1.81E-02	promoter	super_enhancer	RP5-884G6.2
chr1	100435000	chr1	156905000	6.86E-03	promoter	super_enhancer	RP5-884G6.2
chr1	100565000	chr1	109275000	1.65E-02	promoter	super_enhancer	RP4-714D9.4
chr1	100565000	chr1	109805000	2.35E-02	promoter	super_enhancer	RP4-714D9.4
chr1	102195000	chr1	202065000	2.40E-03	promoter	super_enhancer	RNU6-965P
chr1	102325000	chr1	224275000	2.57E-03	promoter	super_enhancer	RNU6-352P
chr1	103575000	chr1	235005000	2.09E-03	promoter	super_enhancer	COL11A1
chr1	104065000	chr1	150615000	4.22E-03	promoter	super_enhancer	RN7SKP285
chr1	109275000	chr1	109585000	2.03E-02	super_enhancer	promoter	WDR47
chr1	109275000	chr1	110135000	4.38E-02	super_enhancer	promoter	RNU6V
chr1	109275000	chr1	110655000	2.23E-02	super_enhancer	promoter	UBL4B
chr1	109275000	chr1	118035000	1.20E-02	super_enhancer	promoter	RP4-675C20.4
chr1	109275000	chr1	147735000	4.92E-03	super_enhancer	promoter	RNU1-120P
chr1	109275000	chr1	155905000	5.07E-03	super_enhancer	promoter	KIAA0907

chr1	109655000	chr1	204125000	4.31E-03	promoter	super_enhancer	KIAA1324
chr1	109655000	chr1	207015000	3.55E-03	promoter	super_enhancer	KIAA1324
chr1	109785000	chr1	170085000	3.25E-03	super_enhancer	promoter	SIGLEC30P
chr1	109795000	chr1	110655000	2.19E-02	super_enhancer	promoter	UBL4B
chr1	109795000	chr1	111935000	6.82E-03	super_enhancer	promoter	PGCP1
chr1	109795000	chr1	114415000	3.11E-02	super_enhancer	promoter	PTPN22
chr1	109795000	chr1	115825000	3.89E-02	super_enhancer	promoter	RP4-663N10.1
chr1	109795000	chr1	117505000	2.68E-02	super_enhancer	promoter	RNA5SP55
chr1	109795000	chr1	150335000	6.96E-03	super_enhancer	promoter	RPRD2
chr1	109795000	chr1	151345000	8.89E-03	super_enhancer	promoter	SELENBP1
chr1	109795000	chr1	159825000	5.91E-03	super_enhancer	promoter	C1orf204
chr1	109795000	chr1	229455000	3.21E-03	super_enhancer	promoter	RP4-803J11.2
chr1	109805000	chr1	110025000	3.98E-02	super_enhancer	promoter	ATXN7L2
chr1	109805000	chr1	116915000	2.70E-02	super_enhancer	promoter	ATP1A1
chr1	109805000	chr1	228265000	3.39E-03	super_enhancer	promoter	RP5-881P19.7
chr1	109815000	chr1	117135000	2.17E-02	super_enhancer	promoter	AL355794.1
chr1	109815000	chr1	155555000	6.17E-03	super_enhancer	promoter	DAP3P1
chr1	109815000	chr1	205535000	2.76E-03	super_enhancer	promoter	RNU2-19P
chr1	109825000	chr1	110285000	3.40E-02	super_enhancer	promoter	GSTM3
chr1	109825000	chr1	119875000	2.08E-02	super_enhancer	promoter	RNU1-75P
chr1	109825000	chr1	152995000	5.09E-03	super_enhancer	promoter	RP1-20N18.4
chr1	109875000	chr1	150255000	6.75E-03	super_enhancer	promoter	CIART
chr1	109875000	chr1	152995000	5.31E-03	super_enhancer	promoter	RP1-20N18.4
chr1	109885000	chr1	114355000	3.99E-02	super_enhancer	promoter	RSBN1

chr1	109885000	chr1	114415000	3.21E-02	super_enhancer	promoter	PTPN22
chr1	109885000	chr1	161005000	5.43E-03	super_enhancer	promoter	RP11-544M22.3
chr1	109885000	chr1	162415000	6.20E-03	super_enhancer	promoter	SLAMF6P1
chr1	109885000	chr1	232835000	3.13E-03	super_enhancer	promoter	RNU6-1211P
chr1	109905000	chr1	110355000	4.91E-02	super_enhancer	promoter	NDUFA5P10
chr1	109905000	chr1	113615000	3.34E-02	super_enhancer	promoter	LRIG2
chr1	109905000	chr1	119875000	2.29E-02	super_enhancer	promoter	RNU1-75P
chr1	109905000	chr1	157895000	5.89E-03	super_enhancer	promoter	RP11-451O13.1
chr1	109905000	chr1	184985000	3.24E-03	super_enhancer	promoter	RNA5SP72
chr1	110025000	chr1	164755000	5.46E-03	promoter	super_enhancer	ATXN7L2
chr1	110135000	chr1	159925000	4.66E-03	promoter	super_enhancer	RNU6V
chr1	110285000	chr1	158025000	5.00E-03	promoter	super_enhancer	GSTM3
chr1	110625000	chr1	158005000	6.67E-03	promoter	super_enhancer	LINC01397
chr1	110715000	chr1	150975000	8.76E-03	promoter	super_enhancer	RP5-1028L10.1
chr1	110935000	chr1	204455000	2.81E-03	promoter	super_enhancer	SLC16A4
chr1	111505000	chr1	153535000	5.34E-03	promoter	super_enhancer	RP11-96K19.5
chr1	111505000	chr1	155005000	5.09E-03	promoter	super_enhancer	RP11-96K19.5
chr1	111865000	chr1	230145000	1.91E-03	promoter	super_enhancer	RP5-1125M8.3
chr1	111935000	chr1	235065000	2.74E-03	promoter	super_enhancer	PGCP1
chr1	112015000	chr1	156105000	6.31E-03	promoter	super_enhancer	C1orf162
chr1	112705000	chr1	116915000	3.84E-02	super_enhancer	promoter	ATP1A1
chr1	112705000	chr1	149195000	4.42E-03	super_enhancer	promoter	RNVU1-17
chr1	112705000	chr1	212525000	2.21E-03	super_enhancer	promoter	SNORA16B
chr1	113465000	chr1	156105000	6.84E-03	promoter	super_enhancer	AKR7A2P1

chr1	113465000	chr1	156465000	9.57E-03	promoter	super_enhancer	AKR7A2P1
chr1	113465000	chr1	235005000	3.35E-03	promoter	super_enhancer	AKR7A2P1
chr1	114355000	chr1	156465000	7.91E-03	promoter	super_enhancer	RSBN1
chr1	114355000	chr1	156485000	8.06E-03	promoter	super_enhancer	RSBN1
chr1	114545000	chr1	156895000	9.09E-03	promoter	super_enhancer	RP4-590F24.1
chr1	114545000	chr1	202035000	4.03E-03	promoter	super_enhancer	RP4-590F24.1
chr1	114575000	chr1	234715000	2.06E-03	promoter	super_enhancer	RP4-590F24.2
chr1	114805000	chr1	156095000	6.42E-03	promoter	super_enhancer	RP4-543J13.1
chr1	115055000	chr1	164755000	5.33E-03	promoter	super_enhancer	TRIM33
chr1	115125000	chr1	156095000	7.60E-03	promoter	super_enhancer	BCAS2
chr1	115175000	chr1	147735000	7.25E-03	super_enhancer	promoter	RNU1-120P
chr1	115175000	chr1	152585000	5.99E-03	super_enhancer	promoter	LCE3B
chr1	115175000	chr1	153365000	8.40E-03	super_enhancer	promoter	S100A8
chr1	115175000	chr1	192965000	2.48E-03	super_enhancer	promoter	ZNF101P2
chr1	116185000	chr1	181095000	3.20E-03	promoter	super_enhancer	VANGL1
chr1	116915000	chr1	234965000	2.64E-03	promoter	super_enhancer	ATP1A1
chr1	117115000	chr1	202075000	2.78E-03	promoter	super_enhancer	CD58
chr1	117115000	chr1	234965000	2.63E-03	promoter	super_enhancer	CD58
chr1	117135000	chr1	212515000	2.31E-03	promoter	super_enhancer	AL355794.1
chr1	117285000	chr1	203525000	3.94E-03	promoter	super_enhancer	NEFHP1
chr1	117285000	chr1	235905000	3.12E-03	promoter	super_enhancer	NEFHP1
chr1	117505000	chr1	151195000	9.11E-03	promoter	super_enhancer	RNA5SP55
chr1	117505000	chr1	211615000	3.56E-03	promoter	super_enhancer	RNA5SP55
chr1	117755000	chr1	205285000	3.29E-03	promoter	super_enhancer	VTCN1

chr1	118185000	chr1	202075000	2.72E-03	promoter	super_enhancer	AL365331.2
chr1	119545000	chr1	204425000	3.33E-03	promoter	super_enhancer	RP4-712E4.1
chr1	120395000	chr1	230115000	2.22E-03	promoter	super_enhancer	PFN1P9
chr1	144875000	chr1	202065000	7.12E-03	promoter	super_enhancer	RP4-791M13.5
chr1	144875000	chr1	207015000	5.58E-03	promoter	super_enhancer	RP4-791M13.5
chr1	144945000	chr1	205285000	5.88E-03	promoter	super_enhancer	RNU2-38P
chr1	144945000	chr1	205305000	7.37E-03	promoter	super_enhancer	RNU2-38P
chr1	145095000	chr1	164765000	1.60E-02	promoter	super_enhancer	SEC22B
chr1	145475000	chr1	150615000	4.07E-02	promoter	super_enhancer	CH17-270A2.1
chr1	145475000	chr1	204075000	3.90E-03	promoter	super_enhancer	CH17-270A2.1
chr1	145475000	chr1	211625000	2.79E-03	promoter	super_enhancer	CH17-270A2.1
chr1	145515000	chr1	156465000	2.57E-02	promoter	super_enhancer	PEX11B
chr1	145525000	chr1	156465000	2.55E-02	promoter	super_enhancer	ITGA10
chr1	145575000	chr1	202055000	5.11E-03	promoter	super_enhancer	PIAS3
chr1	146555000	chr1	152035000	2.39E-02	promoter	super_enhancer	RP11-325P15.2
chr1	146555000	chr1	161125000	1.15E-02	promoter	super_enhancer	RP11-325P15.2
chr1	146555000	chr1	212485000	2.47E-03	promoter	super_enhancer	RP11-325P15.2
chr1	146555000	chr1	225455000	1.32E-03	promoter	super_enhancer	RP11-325P15.2
chr1	146645000	chr1	150975000	3.91E-02	promoter	super_enhancer	PRKAB2
chr1	146645000	chr1	153535000	2.18E-02	promoter	super_enhancer	PRKAB2
chr1	146645000	chr1	153615000	2.13E-02	promoter	super_enhancer	PRKAB2
chr1	146695000	chr1	153605000	2.52E-02	promoter	super_enhancer	RPL7AP15
chr1	146695000	chr1	204435000	4.02E-03	promoter	super_enhancer	RPL7AP15
chr1	146715000	chr1	150975000	3.65E-02	promoter	super_enhancer	FMO5

chr1	147245000	chr1	152045000	3.83E-02	promoter	super_enhancer	GJA5
chr1	148855000	chr1	181075000	9.91E-03	promoter	super_enhancer	RP11-763B22.7
chr1	148865000	chr1	164785000	6.97E-03	promoter	super_enhancer	NKAIN1P1
chr1	148865000	chr1	230125000	1.64E-03	promoter	super_enhancer	NKAIN1P1
chr1	149195000	chr1	156895000	1.87E-02	promoter	super_enhancer	RNVU1-17
chr1	149215000	chr1	155975000	2.49E-02	promoter	super_enhancer	RNU1-92P
chr1	149215000	chr1	156745000	2.46E-02	promoter	super_enhancer	RNU1-92P
chr1	149215000	chr1	158015000	1.98E-02	promoter	super_enhancer	RNU1-92P
chr1	149225000	chr1	161125000	2.18E-02	promoter	super_enhancer	RNVU1-18
chr1	149225000	chr1	202035000	6.11E-03	promoter	super_enhancer	RNVU1-18
chr1	150025000	chr1	158005000	1.65E-02	promoter	super_enhancer	RP11-458I7.1
chr1	150255000	chr1	150975000	3.76E-02	promoter	super_enhancer	CIART
chr1	150255000	chr1	151195000	1.46E-02	promoter	super_enhancer	CIART
chr1	150255000	chr1	183275000	8.32E-03	promoter	super_enhancer	CIART
chr1	150255000	chr1	207015000	4.48E-03	promoter	super_enhancer	CIART
chr1	150255000	chr1	235075000	4.04E-03	promoter	super_enhancer	CIART
chr1	150265000	chr1	156475000	4.00E-02	promoter	super_enhancer	MRPS21
chr1	150265000	chr1	156905000	4.59E-02	promoter	super_enhancer	MRPS21
chr1	150335000	chr1	150975000	6.61E-03	promoter	super_enhancer	RPRD2
chr1	150335000	chr1	155125000	4.28E-02	promoter	super_enhancer	RPRD2
chr1	150605000	chr1	150785000	1.97E-02	super_enhancer	promoter	RNU6-1309P
chr1	150605000	chr1	155185000	3.65E-02	super_enhancer	promoter	RP11-263K19.6
chr1	150605000	chr1	201965000	5.03E-03	super_enhancer	promoter	RP11-465N4.5
chr1	150615000	chr1	150785000	2.07E-02	super_enhancer	promoter	RNU6-1309P

chr1	150615000	chr1	150945000	8.37E-03	super_enhancer	promoter	RP11-316M1.3
chr1	150615000	chr1	165645000	1.97E-02	super_enhancer	promoter	RP11-466F5.4
chr1	150615000	chr1	169765000	1.33E-02	super_enhancer	promoter	METTL18
chr1	150615000	chr1	203255000	5.94E-03	super_enhancer	promoter	LINC01353
chr1	150615000	chr1	224805000	3.92E-03	super_enhancer	promoter	RP11-100E13.1
chr1	150615000	chr1	237145000	3.91E-03	super_enhancer	promoter	RPL35P1
chr1	150785000	chr1	205285000	5.26E-03	promoter	super_enhancer	RNU6-1309P
chr1	150855000	chr1	172895000	1.07E-02	promoter	super_enhancer	RP11-235D19.2
chr1	150855000	chr1	235075000	4.34E-03	promoter	super_enhancer	RP11-235D19.2
chr1	150875000	chr1	155985000	4.40E-02	promoter	super_enhancer	CYCSP51
chr1	150875000	chr1	156105000	3.87E-02	promoter	super_enhancer	CYCSP51
chr1	150875000	chr1	156115000	1.09E-03	promoter	super_enhancer	CYCSP51
chr1	150875000	chr1	156505000	4.23E-02	promoter	super_enhancer	CYCSP51
chr1	150875000	chr1	164765000	1.88E-02	promoter	super_enhancer	CYCSP51
chr1	150875000	chr1	181095000	9.09E-03	promoter	super_enhancer	CYCSP51
chr1	150875000	chr1	202015000	6.51E-03	promoter	super_enhancer	CYCSP51
chr1	150945000	chr1	155005000	4.68E-02	promoter	super_enhancer	RP11-316M1.3
chr1	150945000	chr1	158015000	2.52E-02	promoter	super_enhancer	RP11-316M1.3
chr1	150975000	chr1	155815000	4.94E-02	super_enhancer	promoter	RP11-101O6.2
chr1	150975000	chr1	156265000	4.37E-02	super_enhancer	promoter	GLMP
chr1	150975000	chr1	202115000	8.12E-03	super_enhancer	promoter	ARL8A
chr1	150975000	chr1	204465000	6.66E-03	super_enhancer	promoter	PIK3C2B
chr1	150975000	chr1	224135000	2.11E-03	super_enhancer	promoter	CICP5
chr1	150975000	chr1	229645000	3.57E-03	super_enhancer	promoter	NUP133

chr1	150995000	chr1	164755000	2.01E-02	promoter	super_enhancer	RNU6-884P
chr1	151085000	chr1	202045000	6.16E-03	promoter	super_enhancer	RP11-68I18.2
chr1	151085000	chr1	206895000	4.95E-03	promoter	super_enhancer	RP11-68I18.2
chr1	151105000	chr1	155985000	4.66E-02	promoter	super_enhancer	RP11-68I18.10
chr1	151105000	chr1	204285000	4.82E-03	promoter	super_enhancer	RP11-68I18.10
chr1	151195000	chr1	159685000	2.55E-02	super_enhancer	promoter	CRP
chr1	151195000	chr1	161995000	2.14E-02	super_enhancer	promoter	OLFML2B
chr1	151195000	chr1	203765000	5.62E-03	super_enhancer	promoter	ZC3H11A
chr1	151195000	chr1	205425000	6.66E-03	super_enhancer	promoter	RP11-576D8.4
chr1	151255000	chr1	234605000	3.29E-03	promoter	super_enhancer	RP11-126K1.2
chr1	151255000	chr1	235005000	3.78E-03	promoter	super_enhancer	RP11-126K1.2
chr1	151345000	chr1	183275000	9.99E-03	promoter	super_enhancer	SELENBP1
chr1	151585000	chr1	156745000	4.12E-02	promoter	super_enhancer	SNX27
chr1	151585000	chr1	202025000	6.22E-03	promoter	super_enhancer	SNX27
chr1	151765000	chr1	156475000	4.79E-02	promoter	super_enhancer	TDRKH
chr1	151765000	chr1	156505000	4.73E-02	promoter	super_enhancer	TDRKH
chr1	151765000	chr1	158025000	2.99E-02	promoter	super_enhancer	TDRKH
chr1	151765000	chr1	159915000	2.38E-02	promoter	super_enhancer	TDRKH
chr1	151765000	chr1	159925000	2.30E-02	promoter	super_enhancer	TDRKH
chr1	151765000	chr1	230105000	2.35E-03	promoter	super_enhancer	TDRKH
chr1	151805000	chr1	158005000	3.56E-02	promoter	super_enhancer	RORC
chr1	151805000	chr1	205315000	6.75E-03	promoter	super_enhancer	RORC
chr1	152035000	chr1	152095000	4.41E-02	super_enhancer	promoter	AL589986.2
chr1	152035000	chr1	153515000	4.17E-02	super_enhancer	promoter	S100A5

chr1	152035000	chr1	168745000	1.30E-02	super_enhancer	promoter	RP1-10C16.1
chr1	152045000	chr1	153175000	4.68E-02	super_enhancer	promoter	LELP1
chr1	152045000	chr1	153515000	4.09E-03	super_enhancer	promoter	S100A5
chr1	152045000	chr1	159825000	2.74E-02	super_enhancer	promoter	C1orf204
chr1	152045000	chr1	168345000	1.76E-02	super_enhancer	promoter	MIR557
chr1	152045000	chr1	178995000	9.18E-03	super_enhancer	promoter	FAM20B
chr1	152045000	chr1	204595000	2.04E-05	super_enhancer	promoter	RP11-430C7.5
chr1	152045000	chr1	228395000	3.32E-03	super_enhancer	promoter	OBSCN
chr1	152485000	chr1	211655000	3.59E-03	promoter	super_enhancer	AL135842.1
chr1	152585000	chr1	158015000	2.94E-02	promoter	super_enhancer	LCE3B
chr1	152585000	chr1	202065000	4.68E-03	promoter	super_enhancer	LCE3B
chr1	152815000	chr1	158025000	2.58E-02	promoter	super_enhancer	LCE6A
chr1	152995000	chr1	158005000	3.18E-02	promoter	super_enhancer	RP1-20N18.4
chr1	153365000	chr1	164765000	2.39E-02	promoter	super_enhancer	S100A8
chr1	153505000	chr1	153545000	3.84E-02	promoter	super_enhancer	BX470102.3
chr1	153505000	chr1	156115000	6.87E-03	promoter	super_enhancer	BX470102.3
chr1	153505000	chr1	204125000	6.26E-03	promoter	super_enhancer	BX470102.3
chr1	153515000	chr1	153535000	4.63E-03	promoter	super_enhancer	S100A5
chr1	153515000	chr1	204065000	6.09E-03	promoter	super_enhancer	S100A5
chr1	153515000	chr1	204115000	8.21E-03	promoter	super_enhancer	S100A5
chr1	153535000	chr1	153645000	1.44E-02	super_enhancer	promoter	ILF2
chr1	153535000	chr1	153765000	2.15E-02	super_enhancer	promoter	RP11-216N14.5
chr1	153535000	chr1	154975000	2.83E-02	super_enhancer	promoter	ZBTB7B
chr1	153535000	chr1	157995000	4.06E-02	super_enhancer	promoter	KIRREL-IT1

chr1	153545000	chr1	166125000	1.95E-02	super_enhancer	promoter	MIR921
chr1	153545000	chr1	182295000	8.37E-03	super_enhancer	promoter	RNU6-152P
chr1	153545000	chr1	237145000	3.38E-03	super_enhancer	promoter	RPL35P1
chr1	153545000	chr1	247095000	3.03E-03	super_enhancer	promoter	AHCTF1
chr1	153565000	chr1	153725000	4.78E-02	super_enhancer	promoter	RP11-216N14.8
chr1	153565000	chr1	161005000	2.58E-02	super_enhancer	promoter	RP11-544M22.3
chr1	153565000	chr1	161375000	2.12E-02	super_enhancer	promoter	RP11-122G18.7
chr1	153565000	chr1	165415000	1.98E-02	super_enhancer	promoter	RXRG
chr1	153565000	chr1	205425000	6.68E-03	super_enhancer	promoter	RP11-576D8.4
chr1	153605000	chr1	153725000	3.86E-02	super_enhancer	promoter	RP11-216N14.8
chr1	153605000	chr1	161375000	2.27E-02	super_enhancer	promoter	RP11-122G18.7
chr1	153605000	chr1	161645000	1.53E-02	super_enhancer	promoter	RP11-25K21.1
chr1	153605000	chr1	207325000	4.46E-03	super_enhancer	promoter	RP11-164O23.5
chr1	153605000	chr1	225965000	3.49E-03	super_enhancer	promoter	SRP9
chr1	153615000	chr1	158665000	2.92E-02	super_enhancer	promoter	OR6K1P
chr1	153615000	chr1	160175000	3.32E-02	super_enhancer	promoter	PEA15
chr1	153615000	chr1	160255000	2.51E-02	super_enhancer	promoter	RP11-574F21.3
chr1	153615000	chr1	160865000	1.98E-02	super_enhancer	promoter	RP11-312J18.5
chr1	153645000	chr1	204085000	6.77E-03	promoter	super_enhancer	ILF2
chr1	153645000	chr1	212595000	4.48E-03	promoter	super_enhancer	ILF2
chr1	153765000	chr1	159915000	3.09E-02	promoter	super_enhancer	RP11-216N14.5
chr1	153765000	chr1	202085000	7.26E-03	promoter	super_enhancer	RP11-216N14.5
chr1	153895000	chr1	202045000	6.01E-03	promoter	super_enhancer	GATAD2B
chr1	153935000	chr1	235915000	2.18E-03	promoter	super_enhancer	MIR6737

chr1	154245000	chr1	154965000	1.13E-02	promoter	super_enhancer	HAX1
chr1	154285000	chr1	158025000	4.81E-02	promoter	super_enhancer	RP11-137P24.5
chr1	154285000	chr1	234735000	2.15E-03	promoter	super_enhancer	RP11-137P24.5
chr1	154525000	chr1	155005000	1.48E-02	promoter	super_enhancer	UBE2Q1-AS1
chr1	154525000	chr1	155125000	9.52E-03	promoter	super_enhancer	UBE2Q1-AS1
chr1	154955000	chr1	183275000	8.71E-03	promoter	super_enhancer	FLAD1
chr1	154955000	chr1	204105000	6.79E-03	promoter	super_enhancer	FLAD1
chr1	154965000	chr1	161195000	4.40E-02	super_enhancer	promoter	TOMM40L
chr1	154965000	chr1	186345000	7.10E-03	super_enhancer	promoter	TPR
chr1	154965000	chr1	218205000	3.37E-03	super_enhancer	promoter	RP11-152L7.1
chr1	154975000	chr1	156895000	1.66E-02	promoter	super_enhancer	ZBTB7B
chr1	154975000	chr1	181105000	1.25E-02	promoter	super_enhancer	ZBTB7B
chr1	155005000	chr1	161375000	2.58E-02	super_enhancer	promoter	RP11-122G18.7
chr1	155005000	chr1	211385000	5.15E-03	super_enhancer	promoter	AC092017.1
chr1	155005000	chr1	227085000	3.90E-03	super_enhancer	promoter	ADCK3
chr1	155125000	chr1	173605000	1.36E-02	super_enhancer	promoter	RP11-360D2.1
chr1	155125000	chr1	241295000	2.25E-03	super_enhancer	promoter	MIR3123
chr1	155135000	chr1	224485000	3.92E-03	super_enhancer	promoter	RP11-365O16.5
chr1	155145000	chr1	230125000	3.26E-03	promoter	super_enhancer	TRIM46
chr1	155165000	chr1	172895000	1.13E-02	promoter	super_enhancer	RP11-263K19.4
chr1	155165000	chr1	181075000	1.03E-02	promoter	super_enhancer	RP11-263K19.4
chr1	155215000	chr1	205305000	6.82E-03	promoter	super_enhancer	GBA
chr1	155215000	chr1	207005000	5.22E-03	promoter	super_enhancer	GBA
chr1	155295000	chr1	202015000	7.41E-03	promoter	super_enhancer	RUSC1-AS1

chr1	155715000	chr1	156095000	1.47E-02	promoter	super_enhancer	MSTO2P
chr1	155715000	chr1	164785000	1.66E-02	promoter	super_enhancer	MSTO2P
chr1	155715000	chr1	204285000	5.55E-03	promoter	super_enhancer	MSTO2P
chr1	155715000	chr1	209645000	5.30E-03	promoter	super_enhancer	MSTO2P
chr1	155815000	chr1	164755000	2.71E-02	promoter	super_enhancer	RP11-101O6.2
chr1	155815000	chr1	204115000	7.35E-03	promoter	super_enhancer	RP11-101O6.2
chr1	155905000	chr1	155975000	2.45E-02	promoter	super_enhancer	KIAA0907
chr1	155905000	chr1	204435000	6.71E-03	promoter	super_enhancer	KIAA0907
chr1	155905000	chr1	225445000	2.52E-03	promoter	super_enhancer	KIAA0907
chr1	155975000	chr1	164645000	1.94E-02	super_enhancer	promoter	AL391001.2
chr1	155975000	chr1	179545000	1.16E-02	super_enhancer	promoter	NPHS2
chr1	155975000	chr1	211835000	4.75E-03	super_enhancer	promoter	FDPSP8
chr1	155975000	chr1	220745000	2.73E-03	super_enhancer	promoter	RN7SL464P
chr1	155985000	chr1	156025000	2.97E-02	super_enhancer	promoter	UBQLN4
chr1	155985000	chr1	161705000	3.40E-02	super_enhancer	promoter	RN7SL466P
chr1	155985000	chr1	169075000	1.80E-02	super_enhancer	promoter	ATP1B1
chr1	155985000	chr1	211555000	3.85E-03	super_enhancer	promoter	LINC00467
chr1	155985000	chr1	211835000	5.22E-03	super_enhancer	promoter	FDPSP8
chr1	155985000	chr1	231125000	3.05E-03	super_enhancer	promoter	AL844165.1
chr1	156095000	chr1	161195000	1.58E-03	super_enhancer	promoter	TOMM40L
chr1	156095000	chr1	161475000	3.91E-02	super_enhancer	promoter	FCGR2A
chr1	156095000	chr1	165415000	2.66E-02	super_enhancer	promoter	RXRG
chr1	156095000	chr1	168755000	2.08E-02	super_enhancer	promoter	LINC00626
chr1	156095000	chr1	198125000	6.41E-03	super_enhancer	promoter	NEK7

chr1	156105000	chr1	161705000	3.13E-02	super_enhancer	promoter	RN7SL466P
chr1	156115000	chr1	206305000	6.05E-03	super_enhancer	promoter	C1orf186
chr1	156125000	chr1	171315000	2.06E-02	super_enhancer	promoter	TOP1P1
chr1	156125000	chr1	203255000	8.53E-03	super_enhancer	promoter	LINC01353
chr1	156125000	chr1	204245000	9.07E-03	super_enhancer	promoter	RP11-203F10.5
chr1	156125000	chr1	220745000	3.79E-03	super_enhancer	promoter	RN7SL464P
chr1	156135000	chr1	179925000	1.49E-02	super_enhancer	promoter	RP11-533E19.5
chr1	156135000	chr1	192685000	8.01E-03	super_enhancer	promoter	RP11-92K2.2
chr1	156135000	chr1	204465000	8.95E-03	super_enhancer	promoter	PIK3C2B
chr1	156135000	chr1	220265000	5.38E-03	super_enhancer	promoter	BPNT1
chr1	156135000	chr1	245135000	3.52E-03	super_enhancer	promoter	RP11-156E8.1
chr1	156135000	chr1	247375000	3.46E-03	super_enhancer	promoter	RP11-488L18.4
chr1	156265000	chr1	204275000	5.99E-03	promoter	super_enhancer	GLMP
chr1	156465000	chr1	156585000	3.19E-02	super_enhancer	promoter	RP11-284F21.11
chr1	156465000	chr1	161645000	3.02E-02	super_enhancer	promoter	RP11-25K21.1
chr1	156465000	chr1	161655000	3.70E-02	super_enhancer	promoter	RPL31P11
chr1	156465000	chr1	171795000	2.30E-02	super_enhancer	promoter	RP4-612B18.1
chr1	156465000	chr1	183155000	1.15E-02	super_enhancer	promoter	LAMC2
chr1	156465000	chr1	199845000	9.41E-03	super_enhancer	promoter	RP11-567B20.1
chr1	156475000	chr1	161005000	4.94E-02	super_enhancer	promoter	RP11-544M22.3
chr1	156475000	chr1	171415000	1.59E-02	super_enhancer	promoter	CYCSP53
chr1	156475000	chr1	178695000	1.26E-02	super_enhancer	promoter	RP11-428K3.1
chr1	156475000	chr1	179805000	1.02E-02	super_enhancer	promoter	RP11-12M5.4
chr1	156475000	chr1	180925000	1.41E-02	super_enhancer	promoter	KIAA1614-AS1

chr1	156475000	chr1	184355000	1.13E-02	super_enhancer	promoter	C1orf21
chr1	156475000	chr1	185265000	7.41E-03	super_enhancer	promoter	RP4-635A23.6
chr1	156475000	chr1	203275000	8.48E-03	super_enhancer	promoter	LINC01136
chr1	156475000	chr1	214205000	4.55E-03	super_enhancer	promoter	PROX1-AS1
chr1	156485000	chr1	160865000	4.66E-02	super_enhancer	promoter	RP11-312J18.5
chr1	156485000	chr1	173145000	1.98E-02	super_enhancer	promoter	RP3-395P12.2
chr1	156485000	chr1	174965000	1.58E-02	super_enhancer	promoter	RNU6-307P
chr1	156485000	chr1	179925000	1.60E-02	super_enhancer	promoter	RP11-533E19.5
chr1	156485000	chr1	208085000	6.09E-03	super_enhancer	promoter	CD34
chr1	156485000	chr1	213665000	5.64E-03	super_enhancer	promoter	RP11-554K11.2
chr1	156485000	chr1	224445000	4.66E-03	super_enhancer	promoter	MIR320B2
chr1	156495000	chr1	175985000	1.30E-02	super_enhancer	promoter	RP11-492I21.1
chr1	156495000	chr1	183155000	1.02E-02	super_enhancer	promoter	LAMC2
chr1	156505000	chr1	161135000	4.34E-02	super_enhancer	promoter	PPOX
chr1	156505000	chr1	161375000	3.89E-02	super_enhancer	promoter	RP11-122G18.7
chr1	156505000	chr1	170115000	1.59E-02	super_enhancer	promoter	METTL11B
chr1	156505000	chr1	197745000	5.86E-03	super_enhancer	promoter	DENND1B
chr1	156505000	chr1	199585000	5.48E-03	super_enhancer	promoter	AL450244.1
chr1	156505000	chr1	203275000	8.46E-03	super_enhancer	promoter	LINC01136
chr1	156505000	chr1	225965000	3.98E-03	super_enhancer	promoter	SRP9
chr1	156585000	chr1	202065000	7.14E-03	promoter	super_enhancer	RP11-284F21.11
chr1	156615000	chr1	156895000	1.22E-02	promoter	super_enhancer	RP11-284F21.10
chr1	156615000	chr1	202075000	7.39E-03	promoter	super_enhancer	RP11-284F21.10
chr1	156745000	chr1	160865000	3.87E-02	super_enhancer	promoter	RP11-312J18.5

chr1	156745000	chr1	161135000	4.33E-02	super_enhancer	promoter	PPOX
chr1	156745000	chr1	173605000	1.44E-02	super_enhancer	promoter	RP11-360D2.1
chr1	156745000	chr1	174965000	1.22E-02	super_enhancer	promoter	RNU6-307P
chr1	156745000	chr1	177135000	1.49E-02	super_enhancer	promoter	ASTN1
chr1	156745000	chr1	220265000	4.43E-03	super_enhancer	promoter	BPNT1
chr1	156785000	chr1	204415000	8.40E-03	promoter	super_enhancer	NTRK1
chr1	156885000	chr1	172855000	1.21E-02	super_enhancer	promoter	AIMP1P2
chr1	156885000	chr1	230185000	3.08E-03	super_enhancer	promoter	BX323860.1
chr1	156895000	chr1	166125000	2.92E-02	super_enhancer	promoter	MIR921
chr1	156895000	chr1	172745000	1.65E-02	super_enhancer	promoter	RP1-15D23.2
chr1	156895000	chr1	203775000	7.06E-03	super_enhancer	promoter	RP11-397P13.6
chr1	156905000	chr1	174965000	1.63E-02	super_enhancer	promoter	RNU6-307P
chr1	156905000	chr1	182575000	1.36E-02	super_enhancer	promoter	RGS16
chr1	156905000	chr1	247205000	3.18E-03	super_enhancer	promoter	RP11-551G24.2
chr1	157695000	chr1	164775000	1.84E-02	promoter	super_enhancer	VDAC1P9
chr1	157695000	chr1	204455000	4.95E-03	promoter	super_enhancer	VDAC1P9
chr1	157895000	chr1	164765000	3.10E-02	promoter	super_enhancer	RP11-451O13.1
chr1	157895000	chr1	205315000	6.67E-03	promoter	super_enhancer	RP11-451O13.1
chr1	158005000	chr1	159245000	2.47E-02	super_enhancer	promoter	MPTX1
chr1	158005000	chr1	239415000	2.10E-03	super_enhancer	promoter	RP11-544D21.2
chr1	158015000	chr1	161645000	3.30E-02	super_enhancer	promoter	RP11-25K21.1
chr1	158015000	chr1	165645000	2.71E-02	super_enhancer	promoter	RP11-466F5.4
chr1	158015000	chr1	166125000	2.64E-02	super_enhancer	promoter	MIR921
chr1	158015000	chr1	171795000	1.76E-02	super_enhancer	promoter	RP4-612B18.1

chr1	158015000	chr1	173985000	1.13E-02	super_enhancer	promoter	RC3H1-IT1
chr1	158015000	chr1	185305000	1.03E-02	super_enhancer	promoter	GS1-279B7.1
chr1	158015000	chr1	192685000	6.26E-03	super_enhancer	promoter	RP11-92K2.2
chr1	158015000	chr1	203775000	5.98E-03	super_enhancer	promoter	RP11-397P13.6
chr1	158025000	chr1	162795000	3.30E-02	super_enhancer	promoter	RP11-331H2.4
chr1	158035000	chr1	166125000	2.99E-02	super_enhancer	promoter	MIR921
chr1	158035000	chr1	168345000	2.50E-02	super_enhancer	promoter	MIR557
chr1	158035000	chr1	177135000	1.62E-02	super_enhancer	promoter	ASTN1
chr1	158435000	chr1	164755000	2.02E-02	promoter	super_enhancer	OR10K1
chr1	158485000	chr1	181095000	9.41E-03	promoter	super_enhancer	OR10R1P
chr1	158575000	chr1	209665000	3.23E-03	promoter	super_enhancer	OR10Z1
chr1	158575000	chr1	212575000	2.91E-03	promoter	super_enhancer	OR10Z1
chr1	158665000	chr1	235915000	1.49E-03	promoter	super_enhancer	OR6K1P
chr1	158765000	chr1	202065000	5.18E-03	promoter	super_enhancer	OR2AQ1P
chr1	158765000	chr1	209665000	4.39E-03	promoter	super_enhancer	OR2AQ1P
chr1	159245000	chr1	202025000	6.79E-03	promoter	super_enhancer	MPTX1
chr1	159285000	chr1	172905000	1.19E-02	promoter	super_enhancer	OR10J3
chr1	159335000	chr1	202035000	5.96E-03	promoter	super_enhancer	OR10J8P
chr1	159685000	chr1	212485000	5.39E-03	promoter	super_enhancer	CRP
chr1	159915000	chr1	159945000	4.16E-03	super_enhancer	promoter	RP11-48O20.5
chr1	159915000	chr1	161655000	7.30E-03	super_enhancer	promoter	RPL31P11
chr1	159915000	chr1	168465000	1.50E-02	super_enhancer	promoter	RP5-968D22.3
chr1	159925000	chr1	160255000	1.72E-02	super_enhancer	promoter	RP11-574F21.3
chr1	159925000	chr1	166635000	2.14E-02	super_enhancer	promoter	FMO10P

chr1	159925000	chr1	166975000	2.35E-02	super_enhancer	promoter	RNA5SP65
chr1	159925000	chr1	175265000	1.44E-02	super_enhancer	promoter	RP5-1114G22.1
chr1	159925000	chr1	183155000	8.92E-03	super_enhancer	promoter	LAMC2
chr1	159925000	chr1	231665000	3.36E-03	super_enhancer	promoter	RP11-295G20.2
chr1	160175000	chr1	181095000	1.31E-02	promoter	super_enhancer	PEA15
chr1	160175000	chr1	206895000	6.07E-03	promoter	super_enhancer	PEA15
chr1	160765000	chr1	161115000	2.79E-02	promoter	super_enhancer	LY9
chr1	160865000	chr1	204085000	5.71E-03	promoter	super_enhancer	RP11-312J18.5
chr1	160905000	chr1	172925000	1.51E-02	promoter	super_enhancer	RP11-312J18.6
chr1	160905000	chr1	204085000	7.18E-03	promoter	super_enhancer	RP11-312J18.6
chr1	161005000	chr1	172925000	1.61E-02	promoter	super_enhancer	RP11-544M22.3
chr1	161035000	chr1	161125000	4.84E-02	promoter	super_enhancer	AL591806.1
chr1	161115000	chr1	166945000	3.05E-02	super_enhancer	promoter	ILDR2
chr1	161115000	chr1	201435000	5.82E-03	super_enhancer	promoter	RP11-134G8.10
chr1	161115000	chr1	225925000	2.59E-03	super_enhancer	promoter	RP11-145A3.2
chr1	161115000	chr1	227085000	3.74E-03	super_enhancer	promoter	ADCK3
chr1	161115000	chr1	230185000	3.34E-03	super_enhancer	promoter	BX323860.1
chr1	161125000	chr1	165185000	4.23E-02	super_enhancer	promoter	RP11-38C18.3
chr1	161125000	chr1	166945000	3.78E-02	super_enhancer	promoter	ILDR2
chr1	161125000	chr1	182915000	1.34E-02	super_enhancer	promoter	RNA5SP71
chr1	161125000	chr1	183155000	1.05E-02	super_enhancer	promoter	LAMC2
chr1	161125000	chr1	186555000	1.02E-02	super_enhancer	promoter	AL096803.1
chr1	161125000	chr1	201475000	7.53E-03	super_enhancer	promoter	RP11-134G8.7
chr1	161125000	chr1	213225000	5.83E-03	super_enhancer	promoter	RPS6KC1

chr1	161125000	chr1	227085000	4.64E-03	super_enhancer	promoter	ADCK3
chr1	161185000	chr1	181135000	1.16E-04	promoter	super_enhancer	FCER1G
chr1	161195000	chr1	181115000	1.69E-02	promoter	super_enhancer	TOMM40L
chr1	161195000	chr1	206895000	6.97E-03	promoter	super_enhancer	TOMM40L
chr1	161375000	chr1	164775000	4.39E-02	promoter	super_enhancer	RP11-122G18.7
chr1	161375000	chr1	230095000	1.74E-03	promoter	super_enhancer	RP11-122G18.7
chr1	161475000	chr1	202085000	8.19E-03	promoter	super_enhancer	FCGR2A
chr1	161475000	chr1	205315000	6.11E-03	promoter	super_enhancer	FCGR2A
chr1	161575000	chr1	223715000	2.57E-03	promoter	super_enhancer	HSPA7
chr1	161705000	chr1	207005000	5.83E-03	promoter	super_enhancer	RN7SL466P
chr1	161735000	chr1	204435000	7.06E-03	promoter	super_enhancer	ATF6
chr1	161735000	chr1	205295000	6.71E-03	promoter	super_enhancer	ATF6
chr1	161735000	chr1	205325000	7.95E-03	promoter	super_enhancer	ATF6
chr1	162335000	chr1	204095000	8.22E-03	promoter	super_enhancer	RP11-565P22.6
chr1	162335000	chr1	205285000	6.47E-03	promoter	super_enhancer	RP11-565P22.6
chr1	162415000	chr1	202585000	7.96E-03	promoter	super_enhancer	SLAMF6P1
chr1	162795000	chr1	164755000	8.11E-03	promoter	super_enhancer	RP11-331H2.4
chr1	162795000	chr1	204305000	5.81E-03	promoter	super_enhancer	RP11-331H2.4
chr1	163235000	chr1	204105000	8.37E-03	promoter	super_enhancer	NUF2
chr1	164525000	chr1	164745000	3.82E-03	promoter	super_enhancer	PBX1
chr1	164525000	chr1	234975000	3.07E-03	promoter	super_enhancer	PBX1
chr1	164745000	chr1	186265000	7.01E-03	super_enhancer	promoter	PRG4
chr1	164745000	chr1	218205000	2.77E-03	super_enhancer	promoter	RP11-152L7.1
chr1	164745000	chr1	224045000	2.89E-03	super_enhancer	promoter	PHBP11

chr1	164755000	chr1	169075000	4.51E-02	super_enhancer	promoter	ATP1B1
chr1	164755000	chr1	224045000	4.19E-03	super_enhancer	promoter	PHBP11
chr1	164765000	chr1	168755000	4.77E-02	super_enhancer	promoter	LINC00626
chr1	164765000	chr1	184305000	8.48E-03	super_enhancer	promoter	RN7SL654P
chr1	164775000	chr1	172855000	1.78E-02	super_enhancer	promoter	AIMP1P2
chr1	164785000	chr1	171315000	1.79E-02	super_enhancer	promoter	TOP1P1
chr1	164785000	chr1	171455000	1.62E-02	super_enhancer	promoter	PRRC2C
chr1	165645000	chr1	202025000	9.61E-03	promoter	super_enhancer	RP11-466F5.4
chr1	166135000	chr1	234975000	2.66E-03	promoter	super_enhancer	RP11-9L18.3
chr1	166535000	chr1	207005000	3.57E-03	promoter	super_enhancer	FMO8P
chr1	166945000	chr1	212485000	6.74E-03	promoter	super_enhancer	ILDR2
chr1	167165000	chr1	202595000	5.85E-03	promoter	super_enhancer	LINC01363
chr1	167165000	chr1	207005000	6.47E-03	promoter	super_enhancer	LINC01363
chr1	168195000	chr1	204295000	7.68E-03	promoter	super_enhancer	SFT2D2
chr1	168345000	chr1	172895000	3.38E-02	promoter	super_enhancer	MIR557
chr1	168345000	chr1	202065000	9.20E-03	promoter	super_enhancer	MIR557
chr1	168545000	chr1	172925000	2.71E-02	promoter	super_enhancer	XCL1
chr1	168745000	chr1	204445000	5.68E-03	promoter	super_enhancer	RP1-10C16.1
chr1	168755000	chr1	172925000	3.85E-02	promoter	super_enhancer	LINC00626
chr1	168755000	chr1	206905000	8.32E-03	promoter	super_enhancer	LINC00626
chr1	169075000	chr1	202065000	7.91E-03	promoter	super_enhancer	ATP1B1
chr1	169765000	chr1	202585000	7.45E-03	promoter	super_enhancer	METTL18
chr1	170055000	chr1	202085000	7.83E-03	promoter	super_enhancer	KIFAP3
chr1	170055000	chr1	207005000	5.41E-03	promoter	super_enhancer	KIFAP3

chr1	171455000	chr1	181125000	1.82E-02	promoter	super_enhancer	PRRC2C
chr1	171655000	chr1	181075000	1.69E-02	promoter	super_enhancer	RPL4P3
chr1	171795000	chr1	181125000	2.24E-02	promoter	super_enhancer	RP4-612B18.1
chr1	171795000	chr1	202045000	9.70E-03	promoter	super_enhancer	RP4-612B18.1
chr1	172875000	chr1	181105000	2.44E-02	promoter	super_enhancer	RP3-471M13.2
chr1	172895000	chr1	173145000	1.61E-02	super_enhancer	promoter	RP3-395P12.2
chr1	172895000	chr1	179805000	1.50E-02	super_enhancer	promoter	RP11-12M5.4
chr1	172895000	chr1	207975000	4.83E-03	super_enhancer	promoter	MIR29C
chr1	172905000	chr1	180875000	2.26E-02	super_enhancer	promoter	RP11-46A10.2
chr1	172905000	chr1	231665000	3.77E-03	super_enhancer	promoter	RP11-295G20.2
chr1	172915000	chr1	178695000	2.33E-02	super_enhancer	promoter	RP11-428K3.1
chr1	172915000	chr1	179555000	2.70E-02	super_enhancer	promoter	RP11-545A16.3
chr1	172915000	chr1	182925000	1.88E-02	super_enhancer	promoter	RP11-505O17.1
chr1	172915000	chr1	183725000	1.17E-02	super_enhancer	promoter	RP11-444D13.1
chr1	172915000	chr1	245135000	2.30E-03	super_enhancer	promoter	RP11-156E8.1
chr1	172925000	chr1	173145000	1.53E-03	super_enhancer	promoter	RP3-395P12.2
chr1	172925000	chr1	202115000	1.13E-02	super_enhancer	promoter	ARL8A
chr1	172925000	chr1	234795000	3.97E-03	super_enhancer	promoter	RP4-781K5.5
chr1	172935000	chr1	179925000	1.80E-02	super_enhancer	promoter	RP11-533E19.5
chr1	172935000	chr1	180515000	1.99E-02	super_enhancer	promoter	AL358354.1
chr1	172935000	chr1	180875000	1.87E-02	super_enhancer	promoter	RP11-46A10.2
chr1	172935000	chr1	183155000	1.11E-02	super_enhancer	promoter	LAMC2
chr1	173445000	chr1	204295000	8.31E-03	promoter	super_enhancer	PRDX6
chr1	173605000	chr1	181105000	2.45E-02	promoter	super_enhancer	RP11-360D2.1

chr1	173795000	chr1	204455000	8.86E-03	promoter	super_enhancer	CENPL
chr1	174965000	chr1	181135000	2.68E-02	promoter	super_enhancer	RNU6-307P
chr1	174965000	chr1	205325000	8.90E-03	promoter	super_enhancer	RNU6-307P
chr1	175015000	chr1	230105000	3.88E-03	promoter	super_enhancer	SDCCAG3P2
chr1	175265000	chr1	212515000	6.44E-03	promoter	super_enhancer	RP5-1114G22.1
chr1	175845000	chr1	181105000	3.80E-02	promoter	super_enhancer	RP11-222A5.1
chr1	175985000	chr1	181075000	2.66E-02	promoter	super_enhancer	RP11-492I21.1
chr1	175985000	chr1	181105000	3.16E-02	promoter	super_enhancer	RP11-492I21.1
chr1	177135000	chr1	181075000	4.85E-02	promoter	super_enhancer	ASTN1
chr1	178165000	chr1	204105000	1.05E-02	promoter	super_enhancer	RP11-21M7.2
chr1	178695000	chr1	183275000	3.33E-02	promoter	super_enhancer	RP11-428K3.1
chr1	179225000	chr1	204455000	1.10E-02	promoter	super_enhancer	COX5BP8
chr1	179225000	chr1	211655000	8.20E-03	promoter	super_enhancer	COX5BP8
chr1	179335000	chr1	223705000	4.54E-03	promoter	super_enhancer	AXDND1
chr1	179555000	chr1	181125000	2.60E-02	promoter	super_enhancer	RP11-545A16.3
chr1	179805000	chr1	235065000	3.50E-03	promoter	super_enhancer	RP11-12M5.4
chr1	179925000	chr1	234605000	4.16E-03	promoter	super_enhancer	RP11-533E19.5
chr1	180515000	chr1	181075000	4.70E-02	promoter	super_enhancer	AL358354.1
chr1	180515000	chr1	181125000	1.50E-03	promoter	super_enhancer	AL358354.1
chr1	180515000	chr1	181135000	3.65E-05	promoter	super_enhancer	AL358354.1
chr1	180875000	chr1	181125000	2.54E-02	promoter	super_enhancer	RP11-46A10.2
chr1	180875000	chr1	181135000	3.22E-02	promoter	super_enhancer	RP11-46A10.2
chr1	180925000	chr1	181105000	5.20E-03	promoter	super_enhancer	KIAA1614-AS1
chr1	180925000	chr1	230125000	4.87E-03	promoter	super_enhancer	KIAA1614-AS1

chr1	181075000	chr1	186345000	2.30E-02	super_enhancer	promoter	TPR
chr1	181075000	chr1	193075000	1.67E-02	super_enhancer	promoter	GLRX2
chr1	181075000	chr1	200285000	1.14E-02	super_enhancer	promoter	RP11-532L16.3
chr1	181075000	chr1	205595000	9.74E-03	super_enhancer	promoter	RP11-6B6.3
chr1	181075000	chr1	231665000	5.08E-03	super_enhancer	promoter	RP11-295G20.2
chr1	181075000	chr1	236715000	3.60E-03	super_enhancer	promoter	RP11-385F5.4
chr1	181085000	chr1	204135000	1.55E-02	super_enhancer	promoter	REN
chr1	181085000	chr1	205745000	9.94E-03	super_enhancer	promoter	RAB29
chr1	181085000	chr1	223045000	4.19E-03	super_enhancer	promoter	DISP1
chr1	181085000	chr1	247615000	4.33E-03	super_enhancer	promoter	OR2B11
chr1	181095000	chr1	192605000	1.54E-02	super_enhancer	promoter	RGS13
chr1	181095000	chr1	203255000	1.20E-02	super_enhancer	promoter	LINC01353
chr1	181105000	chr1	203145000	1.77E-02	super_enhancer	promoter	MYBPH
chr1	181105000	chr1	230185000	6.59E-03	super_enhancer	promoter	BX323860.1
chr1	181115000	chr1	192765000	1.59E-02	super_enhancer	promoter	RP3-419C19.3
chr1	181115000	chr1	204245000	1.39E-02	super_enhancer	promoter	RP11-203F10.5
chr1	181115000	chr1	219935000	5.36E-03	super_enhancer	promoter	RNA5SP76
chr1	181115000	chr1	224135000	3.68E-03	super_enhancer	promoter	CICP5
chr1	181115000	chr1	228645000	5.70E-03	super_enhancer	promoter	AL139288.1
chr1	181125000	chr1	203225000	9.61E-03	super_enhancer	promoter	NPM1P40
chr1	181125000	chr1	204465000	1.42E-02	super_enhancer	promoter	PIK3C2B
chr1	181125000	chr1	206305000	1.06E-02	super_enhancer	promoter	C1orf186
chr1	181125000	chr1	212965000	8.62E-03	super_enhancer	promoter	NSL1
chr1	181125000	chr1	222015000	5.71E-03	super_enhancer	promoter	RP11-191N8.2

chr1	181135000	chr1	189635000	1.50E-02	super_enhancer	promoter	RNA5SP73
chr1	181135000	chr1	246845000	4.93E-03	super_enhancer	promoter	RP11-439E19.9
chr1	182055000	chr1	202045000	1.52E-02	promoter	super_enhancer	RP11-538D16.3
chr1	182055000	chr1	230145000	9.82E-06	promoter	super_enhancer	RP11-538D16.3
chr1	182295000	chr1	204445000	1.02E-02	promoter	super_enhancer	RNU6-152P
chr1	182655000	chr1	212585000	8.57E-03	promoter	super_enhancer	RGS8
chr1	182925000	chr1	183275000	2.83E-02	promoter	super_enhancer	RP11-505O17.1
chr1	182925000	chr1	212505000	9.69E-03	promoter	super_enhancer	RP11-505O17.1
chr1	183155000	chr1	202055000	1.21E-02	promoter	super_enhancer	LAMC2
chr1	183155000	chr1	211635000	5.75E-03	promoter	super_enhancer	LAMC2
chr1	183235000	chr1	204285000	1.32E-02	promoter	super_enhancer	RP11-548N1.1
chr1	183275000	chr1	183605000	4.94E-02	super_enhancer	promoter	ARPC5
chr1	183275000	chr1	199845000	1.46E-02	super_enhancer	promoter	RP11-567B20.1
chr1	183605000	chr1	211625000	5.86E-03	promoter	super_enhancer	ARPC5
chr1	183605000	chr1	211655000	7.71E-03	promoter	super_enhancer	ARPC5
chr1	184305000	chr1	202595000	7.34E-03	promoter	super_enhancer	RN7SL654P
chr1	184355000	chr1	204105000	1.62E-02	promoter	super_enhancer	C1orf21
chr1	184355000	chr1	212585000	1.01E-02	promoter	super_enhancer	C1orf21
chr1	184535000	chr1	205295000	9.93E-03	promoter	super_enhancer	GS1-115G20.2
chr1	184535000	chr1	235055000	3.97E-03	promoter	super_enhancer	GS1-115G20.2
chr1	184725000	chr1	205295000	1.35E-02	promoter	super_enhancer	EDEM3
chr1	184725000	chr1	207005000	1.27E-02	promoter	super_enhancer	EDEM3
chr1	184945000	chr1	204115000	1.66E-02	promoter	super_enhancer	FAM129A
chr1	184985000	chr1	235905000	4.96E-03	promoter	super_enhancer	RNA5SP72

chr1	185405000	chr1	202015000	1.24E-02	promoter	super_enhancer	FAM195DP
chr1	185405000	chr1	202595000	8.62E-03	promoter	super_enhancer	FAM195DP
chr1	186265000	chr1	211635000	5.96E-03	promoter	super_enhancer	PRG4
chr1	186265000	chr1	225445000	3.34E-03	promoter	super_enhancer	PRG4
chr1	186345000	chr1	202075000	1.16E-02	promoter	super_enhancer	TPR
chr1	186555000	chr1	202085000	1.80E-02	promoter	super_enhancer	AL096803.1
chr1	186555000	chr1	203525000	1.43E-02	promoter	super_enhancer	AL096803.1
chr1	188125000	chr1	202065000	8.40E-03	promoter	super_enhancer	RN7SKP156
chr1	188125000	chr1	212585000	5.63E-03	promoter	super_enhancer	RN7SKP156
chr1	188875000	chr1	235065000	3.77E-03	promoter	super_enhancer	LINC01035
chr1	189635000	chr1	205325000	1.14E-02	promoter	super_enhancer	RNA5SP73
chr1	191845000	chr1	206905000	1.38E-02	promoter	super_enhancer	RP11-541F9.2
chr1	192285000	chr1	202045000	1.28E-02	promoter	super_enhancer	RGS21
chr1	192545000	chr1	212575000	1.18E-02	promoter	super_enhancer	RGS1
chr1	192545000	chr1	224255000	7.93E-03	promoter	super_enhancer	RGS1
chr1	192545000	chr1	234995000	6.01E-03	promoter	super_enhancer	RGS1
chr1	192605000	chr1	212585000	1.22E-02	promoter	super_enhancer	RGS13
chr1	192685000	chr1	202055000	1.87E-02	promoter	super_enhancer	RP11-92K2.2
chr1	192845000	chr1	235075000	7.18E-03	promoter	super_enhancer	RN7SKP126
chr1	193075000	chr1	230125000	6.05E-03	promoter	super_enhancer	GLRX2
chr1	197705000	chr1	202035000	3.60E-02	promoter	super_enhancer	RP11-448G4.2
chr1	197725000	chr1	202595000	3.03E-02	promoter	super_enhancer	RP11-448G4.4
chr1	197725000	chr1	212515000	1.45E-02	promoter	super_enhancer	RP11-448G4.4
chr1	197745000	chr1	202015000	4.01E-02	promoter	super_enhancer	DENND1B

chr1	197745000	chr1	206895000	1.54E-02	promoter	super_enhancer	DENND1B
chr1	197745000	chr1	212515000	1.17E-02	promoter	super_enhancer	DENND1B
chr1	198125000	chr1	235915000	3.91E-03	promoter	super_enhancer	NEK7
chr1	198985000	chr1	204065000	2.08E-02	promoter	super_enhancer	LINC01221
chr1	198985000	chr1	212505000	1.00E-02	promoter	super_enhancer	LINC01221
chr1	198985000	chr1	223705000	5.59E-03	promoter	super_enhancer	LINC01221
chr1	199355000	chr1	204105000	3.98E-02	promoter	super_enhancer	RP11-104D3.2
chr1	199845000	chr1	224275000	1.13E-02	promoter	super_enhancer	RP11-567B20.1
chr1	201435000	chr1	203515000	9.29E-03	promoter	super_enhancer	RP11-134G8.10
chr1	201435000	chr1	207025000	3.03E-02	promoter	super_enhancer	RP11-134G8.10
chr1	201435000	chr1	234975000	8.54E-03	promoter	super_enhancer	RP11-134G8.10
chr1	201475000	chr1	212565000	1.78E-02	promoter	super_enhancer	RP11-134G8.7
chr1	201925000	chr1	207025000	3.27E-02	promoter	super_enhancer	TIMM17A
chr1	201925000	chr1	223705000	9.58E-03	promoter	super_enhancer	TIMM17A
chr1	201965000	chr1	202055000	8.44E-03	promoter	super_enhancer	RP11-465N4.5
chr1	201965000	chr1	202075000	1.01E-02	promoter	super_enhancer	RP11-465N4.5
chr1	201965000	chr1	202085000	2.33E-02	promoter	super_enhancer	RP11-465N4.5
chr1	201965000	chr1	204085000	1.54E-02	promoter	super_enhancer	RP11-465N4.5
chr1	202015000	chr1	203805000	1.82E-02	super_enhancer	promoter	RP11-397P13.7
chr1	202015000	chr1	227895000	9.77E-03	super_enhancer	promoter	ZNF847P
chr1	202015000	chr1	228195000	1.44E-02	super_enhancer	promoter	WNT3A
chr1	202015000	chr1	230185000	1.27E-02	super_enhancer	promoter	BX323860.1
chr1	202025000	chr1	224035000	1.36E-02	super_enhancer	promoter	TP53BP2
chr1	202025000	chr1	224135000	8.12E-03	super_enhancer	promoter	CICP5

chr1	202025000	chr1	228285000	1.50E-02	super_enhancer	promoter	MIR3620
chr1	202035000	chr1	222885000	1.63E-02	super_enhancer	promoter	BROX
chr1	202045000	chr1	203775000	1.53E-02	super_enhancer	promoter	RP11-397P13.6
chr1	202045000	chr1	219935000	1.12E-02	super_enhancer	promoter	RNA5SP76
chr1	202055000	chr1	211835000	2.39E-02	super_enhancer	promoter	FDPSP8
chr1	202055000	chr1	212005000	2.35E-02	super_enhancer	promoter	LPGAT1
chr1	202055000	chr1	212965000	2.12E-02	super_enhancer	promoter	NSL1
chr1	202055000	chr1	234745000	9.59E-03	super_enhancer	promoter	IRF2BP2
chr1	202065000	chr1	207145000	4.20E-02	super_enhancer	promoter	FCAMR
chr1	202065000	chr1	240755000	6.23E-03	super_enhancer	promoter	RP11-467I20.6
chr1	202075000	chr1	222835000	1.15E-02	super_enhancer	promoter	RP11-378J18.8
chr1	202075000	chr1	230185000	1.15E-02	super_enhancer	promoter	BX323860.1
chr1	202075000	chr1	241695000	4.58E-03	super_enhancer	promoter	KMO
chr1	202085000	chr1	228285000	1.70E-02	super_enhancer	promoter	MIR3620
chr1	202085000	chr1	228675000	1.47E-02	super_enhancer	promoter	RP5-915N17.11
chr1	202085000	chr1	230405000	1.13E-02	super_enhancer	promoter	RP5-956O18.2
chr1	202085000	chr1	244615000	8.39E-03	super_enhancer	promoter	ADSS
chr1	202115000	chr1	205275000	1.99E-04	promoter	super_enhancer	ARL8A
chr1	202115000	chr1	212565000	2.49E-02	promoter	super_enhancer	ARL8A
chr1	202115000	chr1	228145000	1.87E-02	promoter	super_enhancer	ARL8A
chr1	202115000	chr1	235045000	1.09E-02	promoter	super_enhancer	ARL8A
chr1	202585000	chr1	212005000	2.18E-02	super_enhancer	promoter	LPGAT1
chr1	202585000	chr1	248365000	4.09E-03	super_enhancer	promoter	OR2M3
chr1	202595000	chr1	207975000	2.43E-02	super_enhancer	promoter	MIR29C

chr1	202595000	chr1	209175000	1.96E-02	super_enhancer	promoter	RP5-1051D14.2
chr1	202595000	chr1	209345000	2.08E-02	super_enhancer	promoter	RP11-385M4.2
chr1	202595000	chr1	243905000	4.46E-03	super_enhancer	promoter	RP11-370K11.1
chr1	202765000	chr1	203535000	2.72E-02	promoter	super_enhancer	SLC25A39P1
chr1	202765000	chr1	204325000	2.05E-03	promoter	super_enhancer	SLC25A39P1
chr1	202765000	chr1	228145000	1.51E-02	promoter	super_enhancer	SLC25A39P1
chr1	202765000	chr1	235055000	9.61E-03	promoter	super_enhancer	SLC25A39P1
chr1	202995000	chr1	211615000	1.98E-02	promoter	super_enhancer	PPFIA4
chr1	203145000	chr1	230145000	9.70E-03	promoter	super_enhancer	MYBPH
chr1	203225000	chr1	207015000	3.70E-02	promoter	super_enhancer	NPM1P40
chr1	203225000	chr1	228145000	1.09E-02	promoter	super_enhancer	NPM1P40
chr1	203255000	chr1	204085000	2.87E-02	promoter	super_enhancer	LINC01353
chr1	203255000	chr1	204435000	6.06E-03	promoter	super_enhancer	LINC01353
chr1	203275000	chr1	204085000	8.42E-03	promoter	super_enhancer	LINC01136
chr1	203275000	chr1	211635000	2.18E-02	promoter	super_enhancer	LINC01136
chr1	203275000	chr1	212565000	2.40E-02	promoter	super_enhancer	LINC01136
chr1	203275000	chr1	224255000	1.64E-02	promoter	super_enhancer	LINC01136
chr1	203275000	chr1	225445000	9.81E-03	promoter	super_enhancer	LINC01136
chr1	203275000	chr1	225465000	1.38E-02	promoter	super_enhancer	LINC01136
chr1	203445000	chr1	224265000	1.16E-02	promoter	super_enhancer	PRELP
chr1	203525000	chr1	228605000	1.18E-02	super_enhancer	promoter	TRIM17
chr1	203525000	chr1	231475000	1.19E-02	super_enhancer	promoter	EXOC8
chr1	203525000	chr1	244615000	7.38E-03	super_enhancer	promoter	ADSS
chr1	203535000	chr1	203595000	4.93E-02	super_enhancer	promoter	ATP2B4

chr1	203535000	chr1	208085000	3.85E-02	super_enhancer	promoter	CD34
chr1	203535000	chr1	229385000	1.21E-02	super_enhancer	promoter	TMEM78
chr1	203535000	chr1	233085000	1.04E-02	super_enhancer	promoter	NTPCR
chr1	203595000	chr1	209655000	3.33E-02	promoter	super_enhancer	ATP2B4
chr1	203765000	chr1	212515000	2.22E-02	promoter	super_enhancer	ZC3H11A
chr1	203775000	chr1	212565000	2.03E-02	promoter	super_enhancer	RP11-397P13.6
chr1	203805000	chr1	204085000	2.90E-02	promoter	super_enhancer	RP11-397P13.7
chr1	203805000	chr1	204105000	2.66E-02	promoter	super_enhancer	RP11-397P13.7
chr1	203805000	chr1	204425000	4.82E-02	promoter	super_enhancer	RP11-397P13.7
chr1	203805000	chr1	235065000	9.42E-03	promoter	super_enhancer	RP11-397P13.7
chr1	204065000	chr1	207325000	4.92E-02	super_enhancer	promoter	RP11-164O23.5
chr1	204065000	chr1	240135000	5.53E-03	super_enhancer	promoter	RP11-438O11.1
chr1	204075000	chr1	222885000	1.38E-02	super_enhancer	promoter	BROX
chr1	204075000	chr1	228195000	1.19E-02	super_enhancer	promoter	WNT3A
chr1	204075000	chr1	236305000	7.97E-03	super_enhancer	promoter	GPR137B
chr1	204085000	chr1	209175000	3.74E-02	super_enhancer	promoter	RP5-1051D14.2
chr1	204095000	chr1	206715000	9.12E-03	super_enhancer	promoter	CH17-76K2.6
chr1	204095000	chr1	212525000	2.36E-02	super_enhancer	promoter	SNORA16B
chr1	204095000	chr1	230955000	1.42E-02	super_enhancer	promoter	RNA5SP79
chr1	204095000	chr1	235705000	1.03E-02	super_enhancer	promoter	MTND4P10
chr1	204095000	chr1	236305000	1.14E-02	super_enhancer	promoter	GPR137B
chr1	204105000	chr1	229685000	1.38E-02	super_enhancer	promoter	RNA5SP78
chr1	204105000	chr1	247375000	6.85E-03	super_enhancer	promoter	RP11-488L18.4
chr1	204115000	chr1	212965000	2.58E-02	super_enhancer	promoter	NSL1

chr1	204115000	chr1	222015000	1.44E-02	super_enhancer	promoter	RP11-191N8.2
chr1	204125000	chr1	218615000	1.43E-02	super_enhancer	promoter	TGFB2-OT1
chr1	204135000	chr1	204285000	4.97E-03	promoter	super_enhancer	REN
chr1	204135000	chr1	204435000	5.38E-03	promoter	super_enhancer	REN
chr1	204135000	chr1	234965000	1.06E-02	promoter	super_enhancer	REN
chr1	204245000	chr1	204325000	8.73E-05	promoter	super_enhancer	RP11-203F10.5
chr1	204245000	chr1	211655000	3.01E-02	promoter	super_enhancer	RP11-203F10.5
chr1	204245000	chr1	212485000	2.91E-02	promoter	super_enhancer	RP11-203F10.5
chr1	204275000	chr1	204915000	3.86E-03	super_enhancer	promoter	RP11-494K3.2
chr1	204275000	chr1	224545000	1.10E-02	super_enhancer	promoter	CNIH4
chr1	204275000	chr1	228785000	9.70E-03	super_enhancer	promoter	RNA5SP18
chr1	204275000	chr1	236135000	8.32E-03	super_enhancer	promoter	RP4-580N22.2
chr1	204285000	chr1	214705000	1.88E-02	super_enhancer	promoter	KRT18P12
chr1	204285000	chr1	231665000	9.63E-03	super_enhancer	promoter	RP11-295G20.2
chr1	204295000	chr1	220375000	1.39E-02	super_enhancer	promoter	MIR664A
chr1	204305000	chr1	230185000	1.24E-02	super_enhancer	promoter	BX323860.1
chr1	204315000	chr1	212605000	2.52E-02	super_enhancer	promoter	NENF
chr1	204315000	chr1	213905000	1.63E-02	super_enhancer	promoter	RP11-323I1.1
chr1	204315000	chr1	229695000	9.91E-03	super_enhancer	promoter	ABCB10
chr1	204325000	chr1	234765000	1.13E-02	super_enhancer	promoter	LINC00184
chr1	204325000	chr1	244615000	7.43E-03	super_enhancer	promoter	ADSS
chr1	204415000	chr1	204465000	4.24E-02	super_enhancer	promoter	PIK3C2B
chr1	204415000	chr1	204485000	3.32E-02	super_enhancer	promoter	MDM4
chr1	204415000	chr1	224035000	1.52E-02	super_enhancer	promoter	TP53BP2

chr1	204415000	chr1	228285000	1.67E-02	super_enhancer	promoter	MIR3620
chr1	204415000	chr1	228605000	1.31E-02	super_enhancer	promoter	TRIM17
chr1	204415000	chr1	235325000	1.13E-02	super_enhancer	promoter	RBM34
chr1	204415000	chr1	244515000	8.03E-03	super_enhancer	promoter	C1orf100
chr1	204415000	chr1	246845000	9.06E-03	super_enhancer	promoter	RP11-439E19.9
chr1	204425000	chr1	228465000	1.43E-02	super_enhancer	promoter	RP5-1139B12.3
chr1	204435000	chr1	208085000	4.98E-02	super_enhancer	promoter	CD34
chr1	204435000	chr1	209145000	4.64E-02	super_enhancer	promoter	RP5-1051D14.1
chr1	204435000	chr1	222185000	1.19E-02	super_enhancer	promoter	AC092765.1
chr1	204435000	chr1	226625000	1.44E-02	super_enhancer	promoter	RP11-118H4.1
chr1	204435000	chr1	235325000	1.02E-02	super_enhancer	promoter	RBM34
chr1	204435000	chr1	236645000	9.16E-03	super_enhancer	promoter	ENO1P1
chr1	204445000	chr1	204465000	3.49E-02	super_enhancer	promoter	PIK3C2B
chr1	204455000	chr1	204915000	1.37E-02	super_enhancer	promoter	RP11-494K3.2
chr1	204455000	chr1	234795000	1.11E-02	super_enhancer	promoter	RP4-781K5.5
chr1	204465000	chr1	234725000	1.07E-02	promoter	super_enhancer	PIK3C2B
chr1	204595000	chr1	207015000	6.00E-03	promoter	super_enhancer	RP11-430C7.5
chr1	204595000	chr1	230135000	1.27E-02	promoter	super_enhancer	RP11-430C7.5
chr1	204655000	chr1	235065000	9.54E-03	promoter	super_enhancer	LRRN2
chr1	205275000	chr1	224485000	1.51E-02	super_enhancer	promoter	RP11-365O16.5
chr1	205275000	chr1	225965000	1.42E-02	super_enhancer	promoter	SRP9
chr1	205275000	chr1	227915000	1.31E-02	super_enhancer	promoter	SNAP47
chr1	205275000	chr1	229455000	1.30E-02	super_enhancer	promoter	RP4-803J11.2
chr1	205285000	chr1	228755000	6.83E-03	super_enhancer	promoter	RNA5S5

chr1	205305000	chr1	205425000	3.74E-02	super_enhancer	promoter	RP11-576D8.4
chr1	205305000	chr1	205535000	3.70E-02	super_enhancer	promoter	RNU2-19P
chr1	205305000	chr1	205745000	1.67E-02	super_enhancer	promoter	RAB29
chr1	205305000	chr1	227895000	1.23E-02	super_enhancer	promoter	ZNF847P
chr1	205315000	chr1	205425000	4.29E-02	super_enhancer	promoter	RP11-576D8.4
chr1	205315000	chr1	212605000	2.98E-02	super_enhancer	promoter	NENF
chr1	205325000	chr1	205535000	1.05E-02	super_enhancer	promoter	RNU2-19P
chr1	205325000	chr1	212605000	3.47E-02	super_enhancer	promoter	NENF
chr1	205325000	chr1	213665000	2.54E-02	super_enhancer	promoter	RP11-554K11.2
chr1	205325000	chr1	224955000	1.96E-02	super_enhancer	promoter	RP11-449J1.1
chr1	205325000	chr1	230955000	1.42E-02	super_enhancer	promoter	RNA5SP79
chr1	205325000	chr1	244515000	8.61E-03	super_enhancer	promoter	C1orf100
chr1	205425000	chr1	224275000	1.69E-02	promoter	super_enhancer	RP11-576D8.4
chr1	205595000	chr1	206905000	3.87E-02	promoter	super_enhancer	RP11-6B6.3
chr1	205595000	chr1	209645000	4.53E-02	promoter	super_enhancer	RP11-6B6.3
chr1	205595000	chr1	212515000	2.44E-02	promoter	super_enhancer	RP11-6B6.3
chr1	206225000	chr1	207015000	1.85E-02	promoter	super_enhancer	RP11-38J22.3
chr1	206225000	chr1	207025000	1.87E-02	promoter	super_enhancer	RP11-38J22.3
chr1	206225000	chr1	230085000	1.15E-02	promoter	super_enhancer	RP11-38J22.3
chr1	206305000	chr1	225465000	1.19E-02	promoter	super_enhancer	C1orf186
chr1	206305000	chr1	234715000	7.33E-03	promoter	super_enhancer	C1orf186
chr1	206895000	chr1	207925000	3.34E-02	super_enhancer	promoter	CD46
chr1	206895000	chr1	207935000	3.78E-02	super_enhancer	promoter	CDCA4P4
chr1	206895000	chr1	225925000	1.01E-02	super_enhancer	promoter	RP11-145A3.2

chr1	206905000	chr1	207145000	1.43E-02	super_enhancer	promoter	FCAMR
chr1	206905000	chr1	212005000	4.55E-02	super_enhancer	promoter	LPGAT1
chr1	206905000	chr1	219585000	1.88E-02	super_enhancer	promoter	RP11-392O17.1
chr1	207005000	chr1	207145000	3.09E-02	super_enhancer	promoter	FCAMR
chr1	207005000	chr1	212225000	4.11E-02	super_enhancer	promoter	RPL21P28
chr1	207005000	chr1	219935000	1.41E-02	super_enhancer	promoter	RNA5SP76
chr1	207015000	chr1	222815000	1.17E-02	super_enhancer	promoter	RP11-378J18.6
chr1	207015000	chr1	230405000	9.24E-03	super_enhancer	promoter	RP5-956O18.2
chr1	207025000	chr1	227085000	1.40E-02	super_enhancer	promoter	ADCK3
chr1	207025000	chr1	244515000	6.71E-03	super_enhancer	promoter	C1orf100
chr1	207025000	chr1	244815000	7.11E-03	super_enhancer	promoter	DESI2
chr1	207325000	chr1	224255000	1.32E-02	promoter	super_enhancer	RP11-164O23.5
chr1	207325000	chr1	235905000	7.22E-03	promoter	super_enhancer	RP11-164O23.5
chr1	207495000	chr1	211635000	1.91E-02	promoter	super_enhancer	CD55
chr1	207495000	chr1	212585000	2.13E-02	promoter	super_enhancer	CD55
chr1	207495000	chr1	234995000	5.78E-03	promoter	super_enhancer	CD55
chr1	207925000	chr1	211655000	4.36E-02	promoter	super_enhancer	CD46
chr1	207935000	chr1	211625000	3.66E-02	promoter	super_enhancer	CDCA4P4
chr1	207975000	chr1	212565000	3.08E-02	promoter	super_enhancer	MIR29C
chr1	208085000	chr1	223715000	1.42E-02	promoter	super_enhancer	CD34
chr1	209175000	chr1	209665000	3.83E-02	promoter	super_enhancer	RP5-1051D14.2
chr1	209175000	chr1	212595000	4.96E-02	promoter	super_enhancer	RP5-1051D14.2
chr1	209345000	chr1	212495000	4.69E-02	promoter	super_enhancer	RP11-385M4.2
chr1	209345000	chr1	234715000	7.56E-03	promoter	super_enhancer	RP11-385M4.2

chr1	209605000	chr1	230105000	1.15E-02	promoter	super_enhancer	MIR205
chr1	209605000	chr1	234985000	1.34E-02	promoter	super_enhancer	MIR205
chr1	209645000	chr1	243245000	3.79E-03	super_enhancer	promoter	RNU6-747P
chr1	209645000	chr1	248685000	4.20E-03	super_enhancer	promoter	OR2G6
chr1	209655000	chr1	228195000	1.59E-02	super_enhancer	promoter	WNT3A
chr1	209655000	chr1	238655000	6.14E-03	super_enhancer	promoter	KRT18P32
chr1	209665000	chr1	224045000	1.59E-02	super_enhancer	promoter	PHBP11
chr1	210405000	chr1	230115000	9.78E-03	promoter	super_enhancer	SERTAD4
chr1	211385000	chr1	224265000	1.95E-02	promoter	super_enhancer	AC092017.1
chr1	211555000	chr1	211635000	2.18E-04	promoter	super_enhancer	LINC00467
chr1	211555000	chr1	212485000	4.64E-02	promoter	super_enhancer	LINC00467
chr1	211555000	chr1	212515000	3.51E-02	promoter	super_enhancer	LINC00467
chr1	211615000	chr1	212965000	3.49E-02	super_enhancer	promoter	NSL1
chr1	211615000	chr1	219935000	2.04E-02	super_enhancer	promoter	RNA5SP76
chr1	211615000	chr1	231115000	1.65E-02	super_enhancer	promoter	TTC13
chr1	211615000	chr1	241295000	7.49E-03	super_enhancer	promoter	MIR3123
chr1	211625000	chr1	224445000	1.27E-02	super_enhancer	promoter	MIR320B2
chr1	211645000	chr1	211835000	2.01E-02	super_enhancer	promoter	FDPSP8
chr1	211645000	chr1	233585000	8.53E-03	super_enhancer	promoter	RNU4-77P
chr1	211645000	chr1	240175000	8.35E-03	super_enhancer	promoter	RPS7P5
chr1	211645000	chr1	244815000	7.88E-03	super_enhancer	promoter	DESI2
chr1	211655000	chr1	211665000	3.46E-06	super_enhancer	promoter	RP11-359E8.3
chr1	211655000	chr1	222885000	2.15E-02	super_enhancer	promoter	BROX
chr1	211655000	chr1	226125000	1.53E-02	super_enhancer	promoter	RP4-559A3.6

chr1	211655000	chr1	229685000	1.46E-02	super_enhancer	promoter	RNA5SP78
chr1	212005000	chr1	212515000	4.52E-02	promoter	super_enhancer	LPGAT1
chr1	212005000	chr1	230155000	1.42E-02	promoter	super_enhancer	LPGAT1
chr1	212225000	chr1	212485000	2.30E-02	promoter	super_enhancer	RPL21P28
chr1	212225000	chr1	212585000	4.03E-02	promoter	super_enhancer	RPL21P28
chr1	212225000	chr1	212595000	3.08E-02	promoter	super_enhancer	RPL21P28
chr1	212225000	chr1	235065000	1.30E-02	promoter	super_enhancer	RPL21P28
chr1	212485000	chr1	212605000	4.32E-02	super_enhancer	promoter	NENF
chr1	212485000	chr1	226125000	1.68E-02	super_enhancer	promoter	RP4-559A3.6
chr1	212485000	chr1	228755000	9.23E-03	super_enhancer	promoter	RNA5S5
chr1	212485000	chr1	246845000	1.02E-02	super_enhancer	promoter	RP11-439E19.9
chr1	212495000	chr1	224625000	1.50E-02	super_enhancer	promoter	WDR26
chr1	212505000	chr1	212525000	2.35E-02	super_enhancer	promoter	SNORA16B
chr1	212505000	chr1	227365000	9.93E-03	super_enhancer	promoter	RP11-1B20.1
chr1	212505000	chr1	248005000	7.16E-03	super_enhancer	promoter	OR11L1
chr1	212505000	chr1	248855000	5.65E-03	super_enhancer	promoter	XX-CR54.1
chr1	212515000	chr1	224545000	1.50E-02	super_enhancer	promoter	CNIH4
chr1	212515000	chr1	247375000	6.48E-03	super_enhancer	promoter	RP11-488L18.4
chr1	212515000	chr1	247405000	5.15E-03	super_enhancer	promoter	RP11-488L18.8
chr1	212565000	chr1	212605000	9.08E-03	super_enhancer	promoter	NENF
chr1	212565000	chr1	218025000	2.66E-02	super_enhancer	promoter	UBBP2
chr1	212565000	chr1	228195000	1.65E-02	super_enhancer	promoter	WNT3A
chr1	212565000	chr1	228595000	1.41E-02	super_enhancer	promoter	TRIM11
chr1	212575000	chr1	214705000	7.99E-03	super_enhancer	promoter	KRT18P12

chr1	212575000	chr1	220535000	2.73E-02	super_enhancer	promoter	RP11-302I18.3
chr1	212575000	chr1	226625000	1.93E-02	super_enhancer	promoter	RP11-118H4.1
chr1	212575000	chr1	228265000	1.61E-02	super_enhancer	promoter	RP5-881P19.7
chr1	212575000	chr1	233085000	1.33E-02	super_enhancer	promoter	NTPCR
chr1	212575000	chr1	248165000	5.14E-03	super_enhancer	promoter	OR2L6P
chr1	212585000	chr1	212865000	4.80E-02	super_enhancer	promoter	RNU6-423P
chr1	212585000	chr1	243905000	7.65E-03	super_enhancer	promoter	RP11-370K11.1
chr1	212595000	chr1	212965000	2.11E-02	super_enhancer	promoter	NSL1
chr1	212595000	chr1	224035000	1.85E-02	super_enhancer	promoter	TP53BP2
chr1	212595000	chr1	231475000	1.49E-02	super_enhancer	promoter	EXOC8
chr1	212595000	chr1	236645000	1.11E-02	super_enhancer	promoter	ENO1P1
chr1	212595000	chr1	244015000	7.15E-03	super_enhancer	promoter	AKT3
chr1	212605000	chr1	234995000	1.39E-02	promoter	super_enhancer	NENF
chr1	212865000	chr1	230155000	1.39E-02	promoter	super_enhancer	RNU6-423P
chr1	214205000	chr1	234975000	1.18E-02	promoter	super_enhancer	PROX1-AS1
chr1	214705000	chr1	234985000	1.46E-02	promoter	super_enhancer	KRT18P12
chr1	215115000	chr1	234715000	9.55E-03	promoter	super_enhancer	RP11-323K10.1
chr1	217805000	chr1	224255000	2.56E-02	promoter	super_enhancer	GPATCH2
chr1	218205000	chr1	225465000	1.77E-02	promoter	super_enhancer	RP11-152L7.1
chr1	220375000	chr1	224275000	3.78E-02	promoter	super_enhancer	MIR664A
chr1	220745000	chr1	230095000	9.17E-03	promoter	super_enhancer	RN7SL464P
chr1	222015000	chr1	228145000	3.28E-02	promoter	super_enhancer	RP11-191N8.2
chr1	222015000	chr1	234985000	1.65E-02	promoter	super_enhancer	RP11-191N8.2
chr1	222815000	chr1	234725000	1.13E-04	promoter	super_enhancer	RP11-378J18.6

chr1	222835000	chr1	235055000	1.62E-02	promoter	super_enhancer	RP11-378J18.8
chr1	222885000	chr1	224275000	2.34E-03	promoter	super_enhancer	BROX
chr1	223705000	chr1	235595000	1.36E-02	super_enhancer	promoter	RPS21P1
chr1	223715000	chr1	230405000	2.67E-02	super_enhancer	promoter	RP5-956O18.2
chr1	223715000	chr1	235325000	2.17E-02	super_enhancer	promoter	RBM34
chr1	223715000	chr1	236445000	1.83E-02	super_enhancer	promoter	ERO1LB
chr1	224255000	chr1	228785000	3.83E-02	super_enhancer	promoter	RNA5SP18
chr1	224255000	chr1	229685000	3.64E-02	super_enhancer	promoter	RNA5SP78
chr1	224255000	chr1	234765000	2.38E-02	super_enhancer	promoter	LINC00184
chr1	224255000	chr1	234845000	1.98E-02	super_enhancer	promoter	RP4-781K5.7
chr1	224265000	chr1	226125000	8.99E-03	super_enhancer	promoter	RP4-559A3.6
chr1	224275000	chr1	235815000	2.04E-02	super_enhancer	promoter	GNG4
chr1	224295000	chr1	225455000	2.73E-02	promoter	super_enhancer	RN7SKP49
chr1	224485000	chr1	234975000	2.10E-02	promoter	super_enhancer	RP11-365O16.5
chr1	224545000	chr1	230085000	2.49E-02	promoter	super_enhancer	CNIH4
chr1	225445000	chr1	226375000	4.52E-02	super_enhancer	promoter	ACBD3
chr1	225445000	chr1	248605000	5.10E-03	super_enhancer	promoter	OR2T7
chr1	225455000	chr1	240715000	1.24E-02	super_enhancer	promoter	RP11-467I20.4
chr1	225465000	chr1	227365000	3.87E-03	super_enhancer	promoter	RP11-1B20.1
chr1	226035000	chr1	234615000	2.48E-02	promoter	super_enhancer	RP11-285F7.2
chr1	226315000	chr1	235075000	3.39E-02	promoter	super_enhancer	RP11-396C23.3
chr1	226335000	chr1	234605000	2.16E-02	promoter	super_enhancer	RP11-275I14.4
chr1	226375000	chr1	234615000	3.08E-02	promoter	super_enhancer	ACBD3
chr1	226625000	chr1	228145000	3.83E-02	promoter	super_enhancer	RP11-118H4.1

chr1	226625000	chr1	235065000	2.59E-02	promoter	super_enhancer	RP11-118H4.1
chr1	227895000	chr1	235075000	2.56E-02	promoter	super_enhancer	ZNF847P
chr1	227915000	chr1	228145000	1.25E-02	promoter	super_enhancer	SNAP47
chr1	227935000	chr1	234985000	4.22E-02	promoter	super_enhancer	SNAP47-AS1
chr1	227935000	chr1	235005000	4.01E-02	promoter	super_enhancer	SNAP47-AS1
chr1	227935000	chr1	235055000	3.89E-02	promoter	super_enhancer	SNAP47-AS1
chr1	228145000	chr1	230185000	1.62E-02	super_enhancer	promoter	BX323860.1
chr1	228195000	chr1	235915000	1.89E-02	promoter	super_enhancer	WNT3A
chr1	228265000	chr1	235065000	2.80E-02	promoter	super_enhancer	RP5-881P19.7
chr1	228395000	chr1	234995000	3.15E-02	promoter	super_enhancer	OBSCN
chr1	228595000	chr1	234985000	3.29E-02	promoter	super_enhancer	TRIM11
chr1	228675000	chr1	235045000	3.01E-02	promoter	super_enhancer	RP5-915N17.11
chr1	228785000	chr1	235905000	2.03E-02	promoter	super_enhancer	RNA5SP18
chr1	229175000	chr1	234975000	2.86E-02	promoter	super_enhancer	RP5-1065P14.3
chr1	229385000	chr1	235065000	3.48E-02	promoter	super_enhancer	TMEM78
chr1	229695000	chr1	235915000	1.82E-02	promoter	super_enhancer	ABCB10
chr1	229705000	chr1	234605000	6.43E-04	promoter	super_enhancer	HMGN2P19
chr1	229705000	chr1	234615000	4.21E-02	promoter	super_enhancer	HMGN2P19
chr1	229705000	chr1	235915000	2.00E-02	promoter	super_enhancer	HMGN2P19
chr1	230075000	chr1	234795000	4.23E-02	super_enhancer	promoter	RP4-781K5.5
chr1	230095000	chr1	234795000	2.76E-02	super_enhancer	promoter	RP4-781K5.5
chr1	230105000	chr1	236305000	2.45E-02	super_enhancer	promoter	GPR137B
chr1	230105000	chr1	238025000	1.51E-02	super_enhancer	promoter	RP11-193H5.1
chr1	230105000	chr1	240175000	1.58E-02	super_enhancer	promoter	RPS7P5

chr1	230105000	chr1	243265000	8.27E-03	super_enhancer	promoter	LINC01347
chr1	230115000	chr1	242935000	1.47E-02	super_enhancer	promoter	RSL24D1P4
chr1	230125000	chr1	247365000	1.02E-02	super_enhancer	promoter	MIR3916
chr1	230135000	chr1	231125000	4.65E-02	super_enhancer	promoter	AL844165.1
chr1	230135000	chr1	235705000	3.00E-02	super_enhancer	promoter	MTND4P10
chr1	230135000	chr1	236715000	2.35E-02	super_enhancer	promoter	RP11-385F5.4
chr1	230155000	chr1	248525000	1.16E-02	super_enhancer	promoter	OR2T4
chr1	230185000	chr1	234725000	4.49E-02	promoter	super_enhancer	BX323860.1
chr1	230405000	chr1	235065000	3.68E-02	promoter	super_enhancer	RP5-956O18.2
chr1	230405000	chr1	235075000	4.45E-02	promoter	super_enhancer	RP5-956O18.2
chr1	231125000	chr1	234725000	4.85E-02	promoter	super_enhancer	AL844165.1
chr1	233425000	chr1	234615000	3.28E-02	promoter	super_enhancer	RPS7P3
chr1	233585000	chr1	234605000	3.10E-02	promoter	super_enhancer	RNU4-77P
chr1	233585000	chr1	235005000	1.70E-02	promoter	super_enhancer	RNU4-77P
chr1	234605000	chr1	240755000	2.38E-02	super_enhancer	promoter	RP11-467I20.6
chr1	234725000	chr1	241995000	2.62E-02	super_enhancer	promoter	RP11-553N16.1
chr1	234725000	chr1	244815000	2.11E-02	super_enhancer	promoter	DESI2
chr1	234725000	chr1	245135000	1.72E-02	super_enhancer	promoter	RP11-156E8.1
chr1	234745000	chr1	235075000	4.02E-03	promoter	super_enhancer	IRF2BP2
chr1	234765000	chr1	234995000	2.78E-03	promoter	super_enhancer	LINC00184
chr1	234765000	chr1	235075000	3.04E-02	promoter	super_enhancer	LINC00184
chr1	234845000	chr1	235075000	3.31E-02	promoter	super_enhancer	RP4-781K5.7
chr1	234965000	chr1	235325000	3.13E-02	super_enhancer	promoter	RBM34
chr1	234965000	chr1	247095000	2.01E-02	super_enhancer	promoter	AHCTF1

chr1	234975000	chr1	240755000	4.31E-04	super_enhancer	promoter	RP11-467I20.6
chr1	234985000	chr1	240565000	3.01E-02	super_enhancer	promoter	RP11-177F11.1
chr1	235045000	chr1	239415000	3.28E-02	super_enhancer	promoter	RP11-544D21.2
chr1	235045000	chr1	240135000	2.81E-02	super_enhancer	promoter	RP11-438O11.1
chr1	235055000	chr1	235705000	2.32E-02	super_enhancer	promoter	MTND4P10
chr1	235055000	chr1	243245000	1.12E-02	super_enhancer	promoter	RNU6-747P
chr1	235075000	chr1	239895000	3.15E-02	super_enhancer	promoter	CHRM3-AS2
chr1	235075000	chr1	240135000	3.73E-02	super_enhancer	promoter	RP11-438O11.1
chr1	235075000	chr1	241995000	3.49E-02	super_enhancer	promoter	RP11-553N16.1
chr1	235075000	chr1	247205000	1.70E-02	super_enhancer	promoter	RP11-551G24.2
chr1	235905000	chr1	241695000	1.78E-02	super_enhancer	promoter	KMO
chr2	285000	chr2	10255000	1.24E-02	promoter	super_enhancer	AC079779.4
chr2	285000	chr2	36555000	3.37E-03	promoter	super_enhancer	AC079779.4
chr2	315000	chr2	9265000	1.44E-02	promoter	super_enhancer	AC079779.5
chr2	315000	chr2	10295000	1.41E-02	promoter	super_enhancer	AC079779.5
chr2	545000	chr2	235015000	1.13E-03	promoter	super_enhancer	AC093326.2
chr2	865000	chr2	20075000	9.94E-03	promoter	super_enhancer	LINC01115
chr2	865000	chr2	24025000	4.51E-03	promoter	super_enhancer	LINC01115
chr2	1585000	chr2	71945000	3.21E-03	promoter	super_enhancer	AC141930.2
chr2	1625000	chr2	20515000	9.16E-03	promoter	super_enhancer	AC144450.1
chr2	1625000	chr2	20595000	1.24E-02	promoter	super_enhancer	AC144450.1
chr2	1625000	chr2	29125000	7.39E-03	promoter	super_enhancer	AC144450.1
chr2	1625000	chr2	46855000	5.00E-03	promoter	super_enhancer	AC144450.1
chr2	1625000	chr2	85445000	2.54E-03	promoter	super_enhancer	AC144450.1

chr2	1625000	chr2	231625000	2.80E-03	promoter	super_enhancer	AC144450.1
chr2	1625000	chr2	235005000	2.51E-03	promoter	super_enhancer	AC144450.1
chr2	2735000	chr2	218285000	1.95E-03	promoter	super_enhancer	AC018685.2
chr2	2735000	chr2	233405000	2.06E-03	promoter	super_enhancer	AC018685.2
chr2	2875000	chr2	10385000	1.81E-02	promoter	super_enhancer	AC011995.3
chr2	2875000	chr2	10405000	2.30E-02	promoter	super_enhancer	AC011995.3
chr2	3305000	chr2	10405000	2.37E-02	promoter	super_enhancer	TSSC1-IT1
chr2	3305000	chr2	46105000	4.54E-03	promoter	super_enhancer	TSSC1-IT1
chr2	3305000	chr2	85465000	2.60E-03	promoter	super_enhancer	TSSC1-IT1
chr2	3525000	chr2	20515000	8.92E-03	promoter	super_enhancer	ADI1
chr2	3525000	chr2	43175000	6.08E-03	promoter	super_enhancer	ADI1
chr2	3585000	chr2	9285000	3.05E-02	promoter	super_enhancer	AC108488.4
chr2	3585000	chr2	9555000	2.84E-02	promoter	super_enhancer	AC108488.4
chr2	3585000	chr2	20535000	1.11E-02	promoter	super_enhancer	AC108488.4
chr2	3585000	chr2	43195000	7.04E-03	promoter	super_enhancer	AC108488.4
chr2	3585000	chr2	46185000	5.59E-03	promoter	super_enhancer	AC108488.4
chr2	3605000	chr2	9255000	3.34E-02	promoter	super_enhancer	RNASEH1-AS1
chr2	3605000	chr2	10285000	3.45E-02	promoter	super_enhancer	RNASEH1-AS1
chr2	3605000	chr2	46145000	5.98E-03	promoter	super_enhancer	RNASEH1-AS1
chr2	3605000	chr2	46305000	5.14E-03	promoter	super_enhancer	RNASEH1-AS1
chr2	3665000	chr2	46975000	3.44E-03	promoter	super_enhancer	TMSB4XP2
chr2	3705000	chr2	9275000	3.79E-02	promoter	super_enhancer	ALLC
chr2	3705000	chr2	10265000	3.42E-02	promoter	super_enhancer	ALLC
chr2	5455000	chr2	9215000	3.36E-02	promoter	super_enhancer	AC073143.1

chr2	5455000	chr2	10265000	3.38E-02	promoter	super_enhancer	AC073143.1
chr2	5455000	chr2	110105000	1.90E-03	promoter	super_enhancer	AC073143.1
chr2	5835000	chr2	10395000	4.40E-02	promoter	super_enhancer	AC010729.1
chr2	6115000	chr2	10405000	4.12E-02	promoter	super_enhancer	MIR7158
chr2	6635000	chr2	9565000	3.79E-03	promoter	super_enhancer	AC021021.2
chr2	6635000	chr2	20055000	1.98E-02	promoter	super_enhancer	AC021021.2
chr2	6635000	chr2	43175000	8.06E-03	promoter	super_enhancer	AC021021.2
chr2	6635000	chr2	113225000	2.94E-03	promoter	super_enhancer	AC021021.2
chr2	7465000	chr2	43155000	6.63E-03	promoter	super_enhancer	AC010904.1
chr2	7465000	chr2	47045000	4.99E-03	promoter	super_enhancer	AC010904.1
chr2	7865000	chr2	9555000	2.85E-04	promoter	super_enhancer	AC092580.4
chr2	7865000	chr2	20615000	1.28E-02	promoter	super_enhancer	AC092580.4
chr2	7865000	chr2	46075000	5.27E-03	promoter	super_enhancer	AC092580.4
chr2	8725000	chr2	36355000	1.10E-02	promoter	super_enhancer	AC011747.4
chr2	8725000	chr2	43195000	8.84E-03	promoter	super_enhancer	AC011747.4
chr2	8725000	chr2	85465000	3.27E-03	promoter	super_enhancer	AC011747.4
chr2	9145000	chr2	9225000	2.32E-03	promoter	super_enhancer	MBOAT2
chr2	9145000	chr2	10405000	1.43E-02	promoter	super_enhancer	MBOAT2
chr2	9145000	chr2	28335000	6.46E-03	promoter	super_enhancer	MBOAT2
chr2	9145000	chr2	38075000	4.97E-03	promoter	super_enhancer	MBOAT2
chr2	9215000	chr2	16405000	1.93E-02	super_enhancer	promoter	AC010745.2
chr2	9215000	chr2	37095000	6.30E-03	super_enhancer	promoter	RNU6-577P
chr2	9215000	chr2	37385000	9.30E-03	super_enhancer	promoter	EIF2AK2
chr2	9215000	chr2	58135000	2.77E-03	super_enhancer	promoter	VRK2

chr2	9215000	chr2	175105000	2.13E-03	super_enhancer	promoter	RN7SL65P
chr2	9225000	chr2	11885000	4.67E-03	super_enhancer	promoter	AC012456.4
chr2	9225000	chr2	25265000	1.99E-02	super_enhancer	promoter	EFR3B
chr2	9225000	chr2	60645000	4.03E-03	super_enhancer	promoter	AC007381.1
chr2	9225000	chr2	74535000	3.42E-03	super_enhancer	promoter	KRT18P26
chr2	9225000	chr2	219135000	3.54E-03	super_enhancer	promoter	AAMP
chr2	9225000	chr2	223165000	3.22E-03	super_enhancer	promoter	PAX3
chr2	9225000	chr2	226625000	2.13E-03	super_enhancer	promoter	RP11-314B1.1
chr2	9235000	chr2	25565000	1.91E-02	super_enhancer	promoter	DNMT3A
chr2	9235000	chr2	26325000	1.64E-02	super_enhancer	promoter	RP11-214J9.1
chr2	9235000	chr2	42175000	8.49E-03	super_enhancer	promoter	AC013480.1
chr2	9235000	chr2	237995000	2.91E-03	super_enhancer	promoter	AC105760.2
chr2	9245000	chr2	26365000	1.34E-02	super_enhancer	promoter	AC011742.3
chr2	9245000	chr2	217075000	2.89E-03	super_enhancer	promoter	RP11-566E18.1
chr2	9245000	chr2	226625000	2.17E-03	super_enhancer	promoter	RP11-314B1.1
chr2	9255000	chr2	23605000	1.51E-02	super_enhancer	promoter	AC012506.4
chr2	9255000	chr2	69615000	3.63E-03	super_enhancer	promoter	GFPT1
chr2	9255000	chr2	208545000	3.16E-03	super_enhancer	promoter	RNU6-664P
chr2	9255000	chr2	219065000	2.97E-03	super_enhancer	promoter	HMGB1P9
chr2	9265000	chr2	28115000	1.54E-02	super_enhancer	promoter	RBKS
chr2	9265000	chr2	45165000	1.18E-02	super_enhancer	promoter	Six3os1_5
chr2	9265000	chr2	53995000	5.55E-03	super_enhancer	promoter	CHAC2
chr2	9265000	chr2	65045000	4.66E-03	super_enhancer	promoter	RN7SL341P
chr2	9275000	chr2	32035000	1.06E-02	super_enhancer	promoter	AL121652.2

chr2	9275000	chr2	52155000	4.45E-03	super_enhancer	promoter	AC097463.1
chr2	9285000	chr2	26915000	1.61E-02	super_enhancer	promoter	KCNK3
chr2	9285000	chr2	219145000	3.17E-03	super_enhancer	promoter	MIR6513
chr2	9285000	chr2	239065000	2.91E-03	super_enhancer	promoter	KLHL30-AS1
chr2	9295000	chr2	26865000	1.69E-02	super_enhancer	promoter	CIB4
chr2	9295000	chr2	82495000	2.30E-03	super_enhancer	promoter	RNU6-685P
chr2	9305000	chr2	10195000	1.09E-03	super_enhancer	promoter	RP11-254F7.3
chr2	9305000	chr2	20025000	1.59E-02	super_enhancer	promoter	CISD1P1
chr2	9305000	chr2	27805000	1.14E-02	super_enhancer	promoter	AC074091.1
chr2	9305000	chr2	39205000	7.92E-03	super_enhancer	promoter	AC019171.1
chr2	9305000	chr2	46485000	7.75E-03	super_enhancer	promoter	AC016696.1
chr2	9305000	chr2	62095000	4.68E-03	super_enhancer	promoter	AC107081.5
chr2	9305000	chr2	130635000	1.57E-03	super_enhancer	promoter	AC079776.1
chr2	9315000	chr2	20425000	1.60E-02	super_enhancer	promoter	SDC1
chr2	9315000	chr2	31875000	9.89E-03	super_enhancer	promoter	AL133247.3
chr2	9315000	chr2	38605000	7.77E-03	super_enhancer	promoter	ATL2
chr2	9315000	chr2	201935000	2.76E-03	super_enhancer	promoter	NDUFB3
chr2	9555000	chr2	20425000	1.41E-02	super_enhancer	promoter	SDC1
chr2	9555000	chr2	26465000	1.46E-02	super_enhancer	promoter	HADHB
chr2	9555000	chr2	27265000	1.13E-02	super_enhancer	promoter	AGBL5
chr2	9555000	chr2	27435000	1.44E-02	super_enhancer	promoter	ATRAID
chr2	9555000	chr2	37195000	8.37E-03	super_enhancer	promoter	STRN
chr2	9555000	chr2	39665000	7.74E-03	super_enhancer	promoter	MAP4K3
chr2	9555000	chr2	241925000	2.30E-03	super_enhancer	promoter	AC104809.2

chr2	9565000	chr2	38995000	9.27E-03	super_enhancer	promoter	AC018693.5
chr2	9565000	chr2	62395000	6.22E-03	super_enhancer	promoter	RP11-270B14.1
chr2	9565000	chr2	146095000	2.72E-03	super_enhancer	promoter	RPL6P5
chr2	9565000	chr2	217075000	3.07E-03	super_enhancer	promoter	RP11-566E18.1
chr2	9565000	chr2	221655000	1.99E-03	super_enhancer	promoter	AC093843.1
chr2	9645000	chr2	26765000	1.30E-02	promoter	super_enhancer	RP11-400L8.2
chr2	9645000	chr2	177255000	2.15E-03	promoter	super_enhancer	RP11-400L8.2
chr2	9695000	chr2	10265000	4.49E-02	promoter	super_enhancer	RP11-214N9.1
chr2	9695000	chr2	20615000	1.78E-02	promoter	super_enhancer	RP11-214N9.1
chr2	9695000	chr2	28405000	1.09E-02	promoter	super_enhancer	RP11-214N9.1
chr2	9695000	chr2	46065000	6.80E-03	promoter	super_enhancer	RP11-214N9.1
chr2	9695000	chr2	46875000	7.00E-03	promoter	super_enhancer	RP11-214N9.1
chr2	9695000	chr2	47065000	8.03E-03	promoter	super_enhancer	RP11-214N9.1
chr2	10075000	chr2	20065000	1.78E-02	promoter	super_enhancer	RP11-95D17.1
chr2	10075000	chr2	46115000	3.43E-03	promoter	super_enhancer	RP11-95D17.1
chr2	10075000	chr2	46175000	5.13E-03	promoter	super_enhancer	RP11-95D17.1
chr2	10195000	chr2	24025000	8.05E-03	promoter	super_enhancer	RP11-254F7.3
chr2	10195000	chr2	26765000	1.45E-02	promoter	super_enhancer	RP11-254F7.3
chr2	10195000	chr2	46985000	6.10E-03	promoter	super_enhancer	RP11-254F7.3
chr2	10255000	chr2	175105000	2.63E-03	super_enhancer	promoter	RN7SL65P
chr2	10255000	chr2	236945000	3.05E-03	super_enhancer	promoter	RNU7-127P
chr2	10265000	chr2	24715000	1.33E-02	super_enhancer	promoter	NCOA1
chr2	10265000	chr2	47185000	8.42E-03	super_enhancer	promoter	RP11-15I20.1
chr2	10265000	chr2	150445000	2.92E-03	super_enhancer	promoter	MMADHC

chr2	10265000	chr2	152225000	2.71E-03	super_enhancer	promoter	MIR4773-1
chr2	10275000	chr2	27295000	1.26E-02	super_enhancer	promoter	OST4
chr2	10275000	chr2	47125000	6.08E-03	super_enhancer	promoter	AC016722.4
chr2	10275000	chr2	65135000	4.32E-03	super_enhancer	promoter	RN7SL211P
chr2	10275000	chr2	170365000	2.32E-03	super_enhancer	promoter	KLHL41
chr2	10275000	chr2	171675000	3.27E-03	super_enhancer	promoter	AC007405.8
chr2	10275000	chr2	201755000	2.93E-03	super_enhancer	promoter	PPIL3
chr2	10275000	chr2	239465000	2.55E-03	super_enhancer	promoter	LINC01107
chr2	10285000	chr2	26265000	1.81E-02	super_enhancer	promoter	RNU6-942P
chr2	10285000	chr2	27505000	2.13E-02	super_enhancer	promoter	TRIM54
chr2	10285000	chr2	46895000	7.40E-03	super_enhancer	promoter	RP11-536C12.1
chr2	10285000	chr2	71255000	4.26E-03	super_enhancer	promoter	OR7E91P
chr2	10285000	chr2	242715000	3.18E-03	super_enhancer	promoter	GAL3ST2
chr2	10295000	chr2	42395000	8.79E-03	super_enhancer	promoter	EML4
chr2	10295000	chr2	69665000	4.55E-03	super_enhancer	promoter	NFU1
chr2	10295000	chr2	71205000	4.09E-03	super_enhancer	promoter	ANKRD53
chr2	10385000	chr2	47295000	6.20E-03	super_enhancer	promoter	AC073283.7
chr2	10385000	chr2	240685000	2.03E-03	super_enhancer	promoter	AC093802.1
chr2	10395000	chr2	25265000	2.15E-02	super_enhancer	promoter	EFR3B
chr2	10395000	chr2	28615000	1.45E-02	super_enhancer	promoter	FOSL2
chr2	10395000	chr2	29035000	1.29E-02	super_enhancer	promoter	RP11-713D19.1
chr2	10395000	chr2	32265000	1.31E-02	super_enhancer	promoter	DPY30
chr2	10405000	chr2	20375000	1.82E-02	super_enhancer	promoter	RN7SL140P
chr2	10405000	chr2	38605000	7.85E-03	super_enhancer	promoter	ATL2

chr2	10405000	chr2	42175000	7.66E-03	super_enhancer	promoter	AC013480.1
chr2	10405000	chr2	62115000	5.01E-03	super_enhancer	promoter	COMMD1
chr2	10405000	chr2	68695000	2.90E-03	super_enhancer	promoter	FBXO48
chr2	10405000	chr2	75145000	4.51E-03	super_enhancer	promoter	LINC01291
chr2	11015000	chr2	20485000	1.70E-02	promoter	super_enhancer	RP11-245G13.1
chr2	11015000	chr2	29115000	1.39E-02	promoter	super_enhancer	RP11-245G13.1
chr2	11015000	chr2	36565000	8.86E-03	promoter	super_enhancer	RP11-245G13.1
chr2	11295000	chr2	234975000	2.81E-03	promoter	super_enhancer	PQLC3
chr2	11725000	chr2	20085000	2.50E-02	promoter	super_enhancer	RN7SL674P
chr2	11725000	chr2	20505000	1.83E-02	promoter	super_enhancer	RN7SL674P
chr2	11725000	chr2	46975000	5.95E-03	promoter	super_enhancer	RN7SL674P
chr2	11865000	chr2	20075000	2.42E-02	promoter	super_enhancer	AC012456.3
chr2	11885000	chr2	20615000	2.29E-02	promoter	super_enhancer	AC012456.4
chr2	11885000	chr2	36545000	1.03E-02	promoter	super_enhancer	AC012456.4
chr2	14415000	chr2	20595000	2.21E-02	promoter	super_enhancer	RNU6-1288P
chr2	14415000	chr2	46855000	5.79E-03	promoter	super_enhancer	RNU6-1288P
chr2	16405000	chr2	36565000	9.61E-03	promoter	super_enhancer	AC010745.2
chr2	16405000	chr2	46105000	6.81E-03	promoter	super_enhancer	AC010745.2
chr2	16535000	chr2	43175000	9.01E-03	promoter	super_enhancer	AC010880.1
chr2	17305000	chr2	26725000	1.51E-02	promoter	super_enhancer	RN7SKP168
chr2	17305000	chr2	36355000	8.77E-03	promoter	super_enhancer	RN7SKP168
chr2	17305000	chr2	55095000	4.30E-03	promoter	super_enhancer	RN7SKP168
chr2	17465000	chr2	20515000	3.76E-02	promoter	super_enhancer	ZFYVE9P2
chr2	17465000	chr2	36385000	8.33E-03	promoter	super_enhancer	ZFYVE9P2

chr2	17565000	chr2	20495000	3.46E-02	promoter	super_enhancer	PSMC1P10
chr2	17935000	chr2	64845000	3.02E-03	promoter	super_enhancer	GEN1
chr2	18765000	chr2	20065000	2.89E-02	promoter	super_enhancer	RNU6-1215P
chr2	18765000	chr2	20085000	1.48E-04	promoter	super_enhancer	RNU6-1215P
chr2	18765000	chr2	20575000	1.05E-02	promoter	super_enhancer	RNU6-1215P
chr2	18965000	chr2	20055000	3.88E-02	promoter	super_enhancer	AC106053.1
chr2	19675000	chr2	36375000	1.33E-02	promoter	super_enhancer	AC010096.2
chr2	19675000	chr2	47045000	9.20E-03	promoter	super_enhancer	AC010096.2
chr2	20025000	chr2	20475000	3.48E-03	promoter	super_enhancer	CISD1P1
chr2	20045000	chr2	25305000	3.70E-02	super_enhancer	promoter	SUCLA2P3
chr2	20045000	chr2	69615000	5.09E-03	super_enhancer	promoter	GFPT1
chr2	20045000	chr2	197015000	2.68E-03	super_enhancer	promoter	RP11-347P5.1
chr2	20045000	chr2	237995000	2.72E-03	super_enhancer	promoter	AC105760.2
chr2	20055000	chr2	27995000	2.06E-02	super_enhancer	promoter	MRPL33
chr2	20055000	chr2	32035000	1.72E-02	super_enhancer	promoter	AL121652.2
chr2	20055000	chr2	110875000	2.84E-03	super_enhancer	promoter	MALL
chr2	20055000	chr2	178095000	2.10E-03	super_enhancer	promoter	DNAJC19P5
chr2	20055000	chr2	219645000	3.34E-03	super_enhancer	promoter	CYP27A1
chr2	20065000	chr2	37385000	1.80E-02	super_enhancer	promoter	EIF2AK2
chr2	20065000	chr2	68385000	6.13E-03	super_enhancer	promoter	WDR92
chr2	20065000	chr2	131035000	1.85E-03	super_enhancer	promoter	AC068137.8
chr2	20065000	chr2	218115000	2.16E-03	super_enhancer	promoter	RN7SKP43
chr2	20075000	chr2	25265000	4.05E-02	super_enhancer	promoter	EFR3B
chr2	20075000	chr2	140425000	2.10E-03	super_enhancer	promoter	MRPS18BP2

chr2	20075000	chr2	148165000	1.52E-03	super_enhancer	promoter	RNU6-692P
chr2	20085000	chr2	26365000	2.77E-02	super_enhancer	promoter	AC011742.3
chr2	20085000	chr2	37195000	1.47E-02	super_enhancer	promoter	STRN
chr2	20085000	chr2	88715000	3.10E-03	super_enhancer	promoter	RNU6-1007P
chr2	20085000	chr2	193495000	1.90E-03	super_enhancer	promoter	AC013401.1
chr2	20095000	chr2	27435000	2.04E-02	super_enhancer	promoter	ATRAID
chr2	20095000	chr2	37425000	9.11E-03	super_enhancer	promoter	SULT6B1
chr2	20095000	chr2	46655000	6.50E-03	super_enhancer	promoter	TMEM247
chr2	20095000	chr2	47295000	7.56E-03	super_enhancer	promoter	AC073283.7
chr2	20095000	chr2	61245000	5.22E-03	super_enhancer	promoter	PUS10
chr2	20095000	chr2	158345000	1.76E-03	super_enhancer	promoter	CYTIP
chr2	20375000	chr2	24015000	2.94E-02	promoter	super_enhancer	RN7SL140P
chr2	20425000	chr2	20485000	1.82E-02	promoter	super_enhancer	SDC1
chr2	20425000	chr2	38085000	1.07E-02	promoter	super_enhancer	SDC1
chr2	20425000	chr2	46095000	7.32E-03	promoter	super_enhancer	SDC1
chr2	20475000	chr2	85295000	3.42E-03	super_enhancer	promoter	RP11-60H5.1
chr2	20475000	chr2	86115000	2.83E-03	super_enhancer	promoter	ST3GAL5-AS1
chr2	20475000	chr2	86565000	3.21E-03	super_enhancer	promoter	REEP1
chr2	20485000	chr2	35035000	9.24E-03	super_enhancer	promoter	RN7SL602P
chr2	20485000	chr2	42175000	8.43E-03	super_enhancer	promoter	AC013480.1
chr2	20485000	chr2	54395000	6.28E-03	super_enhancer	promoter	RNU7-172P
chr2	20485000	chr2	86565000	3.07E-03	super_enhancer	promoter	REEP1
chr2	20485000	chr2	96875000	2.14E-03	super_enhancer	promoter	STARD7
chr2	20495000	chr2	21225000	4.87E-02	super_enhancer	promoter	RP11-116D2.1

chr2	20495000	chr2	36885000	8.59E-03	super_enhancer	promoter	RP11-294L11.1
chr2	20495000	chr2	47405000	6.93E-03	super_enhancer	promoter	RP11-761B3.1
chr2	20505000	chr2	23555000	2.56E-03	super_enhancer	promoter	AC012506.1
chr2	20505000	chr2	28975000	1.43E-02	super_enhancer	promoter	PPP1CB
chr2	20505000	chr2	51255000	4.24E-03	super_enhancer	promoter	AC007682.4
chr2	20505000	chr2	65135000	4.58E-03	super_enhancer	promoter	RN7SL211P
chr2	20505000	chr2	121935000	1.87E-03	super_enhancer	promoter	AC079988.3
chr2	20515000	chr2	39665000	8.79E-03	super_enhancer	promoter	MAP4K3
chr2	20515000	chr2	66585000	3.21E-03	super_enhancer	promoter	MIR4778
chr2	20525000	chr2	20865000	4.56E-02	super_enhancer	promoter	GDF7
chr2	20525000	chr2	25565000	3.74E-02	super_enhancer	promoter	DNMT3A
chr2	20525000	chr2	74195000	4.01E-03	super_enhancer	promoter	RNA5SP97
chr2	20525000	chr2	230935000	3.00E-03	super_enhancer	promoter	SLC16A14
chr2	20535000	chr2	38605000	8.38E-03	super_enhancer	promoter	ATL2
chr2	20575000	chr2	20865000	2.58E-02	super_enhancer	promoter	GDF7
chr2	20575000	chr2	32035000	1.68E-02	super_enhancer	promoter	AL121652.2
chr2	20575000	chr2	75825000	4.09E-03	super_enhancer	promoter	AC005034.2
chr2	20585000	chr2	29305000	1.98E-02	super_enhancer	promoter	AC105398.1
chr2	20585000	chr2	61165000	4.12E-03	super_enhancer	promoter	NONOP2
chr2	20595000	chr2	35445000	8.42E-03	super_enhancer	promoter	AC013442.1
chr2	20595000	chr2	89065000	2.45E-03	super_enhancer	promoter	ANKRD36BP2
chr2	20595000	chr2	219925000	3.39E-03	super_enhancer	promoter	IHH
chr2	20605000	chr2	38605000	1.22E-02	super_enhancer	promoter	ATL2
chr2	20615000	chr2	24585000	3.81E-02	super_enhancer	promoter	ITSN2

chr2	20615000	chr2	25265000	4.82E-02	super_enhancer	promoter	EFR3B
chr2	20615000	chr2	26035000	3.35E-02	super_enhancer	promoter	TPM3P7
chr2	20615000	chr2	35445000	8.44E-03	super_enhancer	promoter	AC013442.1
chr2	20615000	chr2	39165000	1.21E-02	super_enhancer	promoter	RN7SL96P
chr2	20615000	chr2	47295000	1.02E-02	super_enhancer	promoter	AC073283.7
chr2	20615000	chr2	47405000	9.35E-03	super_enhancer	promoter	RP11-761B3.1
chr2	20615000	chr2	74535000	4.06E-03	super_enhancer	promoter	KRT18P26
chr2	20625000	chr2	25015000	3.59E-02	super_enhancer	promoter	CENPO
chr2	20625000	chr2	31875000	1.50E-02	super_enhancer	promoter	AL133247.3
chr2	20625000	chr2	32145000	1.27E-02	super_enhancer	promoter	AL121652.1
chr2	20625000	chr2	71205000	4.37E-03	super_enhancer	promoter	ANKRD53
chr2	20625000	chr2	105375000	2.58E-03	super_enhancer	promoter	LINC01114
chr2	20865000	chr2	29125000	2.23E-02	promoter	super_enhancer	GDF7
chr2	21225000	chr2	27795000	2.10E-02	promoter	super_enhancer	RP11-116D2.1
chr2	21245000	chr2	36435000	1.45E-02	promoter	super_enhancer	AC010872.1
chr2	22145000	chr2	46085000	6.65E-03	promoter	super_enhancer	RN7SL117P
chr2	22755000	chr2	29115000	2.29E-02	promoter	super_enhancer	AC068490.2
chr2	23605000	chr2	28405000	2.92E-02	promoter	super_enhancer	AC012506.4
chr2	23605000	chr2	29125000	2.36E-02	promoter	super_enhancer	AC012506.4
chr2	23605000	chr2	46185000	9.40E-03	promoter	super_enhancer	AC012506.4
chr2	23605000	chr2	64835000	6.12E-03	promoter	super_enhancer	AC012506.4
chr2	23605000	chr2	71935000	6.50E-03	promoter	super_enhancer	AC012506.4
chr2	24025000	chr2	24585000	3.38E-02	super_enhancer	promoter	ITSN2
chr2	24025000	chr2	24715000	1.79E-02	super_enhancer	promoter	NCOA1

chr2	24425000	chr2	27795000	4.71E-02	promoter	super_enhancer	AC008073.9
chr2	24425000	chr2	36355000	1.76E-02	promoter	super_enhancer	AC008073.9
chr2	24425000	chr2	43035000	1.30E-02	promoter	super_enhancer	AC008073.9
chr2	25015000	chr2	28335000	4.07E-02	promoter	super_enhancer	CENPO
chr2	25015000	chr2	46965000	7.68E-03	promoter	super_enhancer	CENPO
chr2	25015000	chr2	71945000	5.44E-03	promoter	super_enhancer	CENPO
chr2	25015000	chr2	231605000	2.00E-03	promoter	super_enhancer	CENPO
chr2	25265000	chr2	46065000	1.50E-02	promoter	super_enhancer	EFR3B
chr2	25265000	chr2	46175000	1.32E-02	promoter	super_enhancer	EFR3B
chr2	25305000	chr2	43075000	1.78E-02	promoter	super_enhancer	SUCLA2P3
chr2	25305000	chr2	46075000	1.19E-02	promoter	super_enhancer	SUCLA2P3
chr2	25565000	chr2	46945000	1.17E-02	promoter	super_enhancer	DNMT3A
chr2	25565000	chr2	85445000	4.01E-03	promoter	super_enhancer	DNMT3A
chr2	25565000	chr2	234955000	2.70E-03	promoter	super_enhancer	DNMT3A
chr2	25945000	chr2	43155000	1.68E-02	promoter	super_enhancer	AC010150.2
chr2	25945000	chr2	46195000	1.15E-02	promoter	super_enhancer	AC010150.2
chr2	25945000	chr2	46945000	1.08E-02	promoter	super_enhancer	AC010150.2
chr2	26035000	chr2	47045000	1.29E-02	promoter	super_enhancer	TPM3P7
chr2	26035000	chr2	64835000	7.50E-03	promoter	super_enhancer	TPM3P7
chr2	26045000	chr2	38075000	1.28E-02	promoter	super_enhancer	PTGES3P2
chr2	26045000	chr2	55105000	5.96E-03	promoter	super_enhancer	PTGES3P2
chr2	26265000	chr2	46315000	1.30E-02	promoter	super_enhancer	RNU6-942P
chr2	26265000	chr2	54565000	6.99E-03	promoter	super_enhancer	RNU6-942P
chr2	26325000	chr2	43085000	1.58E-02	promoter	super_enhancer	RP11-214J9.1

chr2	26365000	chr2	36355000	1.83E-02	promoter	super_enhancer	AC011742.3
chr2	26465000	chr2	26765000	2.21E-03	promoter	super_enhancer	HADHB
chr2	26465000	chr2	36415000	1.54E-02	promoter	super_enhancer	HADHB
chr2	26465000	chr2	43225000	1.52E-02	promoter	super_enhancer	HADHB
chr2	26465000	chr2	46965000	1.04E-02	promoter	super_enhancer	HADHB
chr2	26465000	chr2	47055000	1.37E-02	promoter	super_enhancer	HADHB
chr2	26465000	chr2	231615000	2.97E-03	promoter	super_enhancer	HADHB
chr2	26725000	chr2	233925000	3.26E-03	super_enhancer	promoter	INPP5D
chr2	26765000	chr2	26865000	1.65E-02	super_enhancer	promoter	CIB4
chr2	26765000	chr2	32605000	2.48E-02	super_enhancer	promoter	BIRC6-AS1
chr2	26765000	chr2	46395000	1.01E-02	super_enhancer	promoter	AC017006.3
chr2	26765000	chr2	86795000	3.46E-03	super_enhancer	promoter	AC015971.3
chr2	26765000	chr2	201715000	2.72E-03	super_enhancer	promoter	RNA5SP115
chr2	26785000	chr2	43165000	1.71E-02	promoter	super_enhancer	C2orf70
chr2	26785000	chr2	55105000	8.77E-03	promoter	super_enhancer	C2orf70
chr2	26785000	chr2	84905000	5.63E-03	promoter	super_enhancer	C2orf70
chr2	26785000	chr2	85465000	5.06E-03	promoter	super_enhancer	C2orf70
chr2	26915000	chr2	43065000	2.20E-02	promoter	super_enhancer	KCNK3
chr2	26915000	chr2	46865000	1.61E-02	promoter	super_enhancer	KCNK3
chr2	26915000	chr2	47055000	1.55E-02	promoter	super_enhancer	KCNK3
chr2	26915000	chr2	231625000	3.50E-03	promoter	super_enhancer	KCNK3
chr2	27255000	chr2	38075000	1.61E-02	promoter	super_enhancer	TMEM214
chr2	27265000	chr2	36375000	1.80E-02	promoter	super_enhancer	AGBL5
chr2	27265000	chr2	38085000	1.62E-02	promoter	super_enhancer	AGBL5

chr2	27265000	chr2	43045000	1.15E-02	promoter	super_enhancer	AGBL5
chr2	27265000	chr2	46955000	9.39E-03	promoter	super_enhancer	AGBL5
chr2	27265000	chr2	231605000	2.05E-03	promoter	super_enhancer	AGBL5
chr2	27295000	chr2	46165000	9.98E-03	promoter	super_enhancer	OST4
chr2	27295000	chr2	46945000	1.01E-02	promoter	super_enhancer	OST4
chr2	27505000	chr2	36375000	2.49E-02	promoter	super_enhancer	TRIM54
chr2	27505000	chr2	46845000	1.77E-02	promoter	super_enhancer	TRIM54
chr2	27605000	chr2	64845000	6.66E-03	promoter	super_enhancer	ZNF513
chr2	27605000	chr2	84915000	4.04E-03	promoter	super_enhancer	ZNF513
chr2	27605000	chr2	127635000	2.40E-03	promoter	super_enhancer	ZNF513
chr2	27665000	chr2	36425000	2.59E-02	promoter	super_enhancer	KRTCAP3
chr2	27665000	chr2	36555000	1.94E-02	promoter	super_enhancer	KRTCAP3
chr2	27665000	chr2	43045000	1.56E-02	promoter	super_enhancer	KRTCAP3
chr2	27665000	chr2	43085000	2.02E-02	promoter	super_enhancer	KRTCAP3
chr2	27665000	chr2	43155000	2.15E-02	promoter	super_enhancer	KRTCAP3
chr2	27665000	chr2	218275000	2.94E-03	promoter	super_enhancer	KRTCAP3
chr2	27795000	chr2	35035000	1.52E-02	super_enhancer	promoter	RN7SL602P
chr2	27795000	chr2	46485000	1.22E-02	super_enhancer	promoter	AC016696.1
chr2	27795000	chr2	55335000	7.53E-03	super_enhancer	promoter	AC093165.1
chr2	27795000	chr2	74875000	3.98E-03	super_enhancer	promoter	M1AP
chr2	27795000	chr2	118755000	2.02E-03	super_enhancer	promoter	RP11-98C1.2
chr2	27795000	chr2	202415000	1.69E-03	super_enhancer	promoter	AC007282.6
chr2	27805000	chr2	36425000	1.89E-02	promoter	super_enhancer	AC074091.1
chr2	27805000	chr2	55105000	6.38E-03	promoter	super_enhancer	AC074091.1

chr2	27805000	chr2	233425000	2.07E-03	promoter	super_enhancer	AC074091.1
chr2	27805000	chr2	238445000	2.62E-03	promoter	super_enhancer	AC074091.1
chr2	27885000	chr2	47075000	1.15E-02	promoter	super_enhancer	SLC4A1AP
chr2	27885000	chr2	240425000	2.83E-03	promoter	super_enhancer	SLC4A1AP
chr2	27995000	chr2	29115000	2.79E-02	promoter	super_enhancer	MRPL33
chr2	28115000	chr2	43055000	1.71E-02	promoter	super_enhancer	RBKS
chr2	28335000	chr2	38995000	1.36E-02	super_enhancer	promoter	AC018693.5
chr2	28335000	chr2	46485000	1.14E-02	super_enhancer	promoter	AC016696.1
chr2	28335000	chr2	48845000	9.03E-03	super_enhancer	promoter	GTF2A1L
chr2	28345000	chr2	32265000	3.29E-02	super_enhancer	promoter	DPY30
chr2	28345000	chr2	32605000	2.32E-02	super_enhancer	promoter	BIRC6-AS1
chr2	28345000	chr2	71405000	3.80E-03	super_enhancer	promoter	AC007881.4
chr2	28345000	chr2	156125000	1.51E-03	super_enhancer	promoter	ATP5A1P2
chr2	28355000	chr2	62085000	5.05E-03	super_enhancer	promoter	RP11-681L4.2
chr2	28355000	chr2	96805000	2.12E-03	super_enhancer	promoter	ASTL
chr2	28355000	chr2	112055000	8.59E-04	super_enhancer	promoter	RPS14P4
chr2	28365000	chr2	38605000	1.37E-02	super_enhancer	promoter	ATL2
chr2	28365000	chr2	44065000	1.38E-02	super_enhancer	promoter	ABCG8
chr2	28365000	chr2	61165000	4.43E-03	super_enhancer	promoter	NONOP2
chr2	28395000	chr2	35445000	1.50E-02	super_enhancer	promoter	AC013442.1
chr2	28395000	chr2	37425000	1.96E-02	super_enhancer	promoter	SULT6B1
chr2	28395000	chr2	190525000	2.10E-03	super_enhancer	promoter	ASNSD1
chr2	28405000	chr2	78015000	2.47E-03	super_enhancer	promoter	AC105399.2
chr2	28405000	chr2	152955000	2.35E-03	super_enhancer	promoter	AC079790.2

chr2	28615000	chr2	38075000	1.74E-02	promoter	super_enhancer	FOSL2
chr2	28615000	chr2	43195000	1.66E-02	promoter	super_enhancer	FOSL2
chr2	28615000	chr2	218295000	2.59E-03	promoter	super_enhancer	FOSL2
chr2	28975000	chr2	47065000	1.25E-02	promoter	super_enhancer	PPP1CB
chr2	28975000	chr2	231625000	2.25E-03	promoter	super_enhancer	PPP1CB
chr2	29005000	chr2	47025000	9.22E-03	promoter	super_enhancer	SPDYA
chr2	29035000	chr2	46325000	1.15E-02	promoter	super_enhancer	RP11-713D19.1
chr2	29035000	chr2	70085000	5.31E-03	promoter	super_enhancer	RP11-713D19.1
chr2	29035000	chr2	71945000	6.33E-03	promoter	super_enhancer	RP11-713D19.1
chr2	29115000	chr2	33165000	4.67E-02	super_enhancer	promoter	AL133244.2
chr2	29115000	chr2	37195000	2.14E-02	super_enhancer	promoter	STRN
chr2	29115000	chr2	209335000	1.36E-03	super_enhancer	promoter	AC019185.4
chr2	29125000	chr2	38295000	1.25E-02	super_enhancer	promoter	RMDN2-AS1
chr2	29125000	chr2	207025000	1.88E-03	super_enhancer	promoter	NDUFS1
chr2	29305000	chr2	113245000	2.21E-03	promoter	super_enhancer	AC105398.1
chr2	29905000	chr2	36355000	2.26E-02	promoter	super_enhancer	AC093756.1
chr2	29905000	chr2	43065000	1.60E-02	promoter	super_enhancer	AC093756.1
chr2	30145000	chr2	43075000	1.98E-02	promoter	super_enhancer	ALK
chr2	30145000	chr2	71945000	6.27E-03	promoter	super_enhancer	ALK
chr2	30145000	chr2	84915000	3.47E-03	promoter	super_enhancer	ALK
chr2	30655000	chr2	43035000	1.71E-02	promoter	super_enhancer	AC073255.1
chr2	30655000	chr2	46325000	1.28E-02	promoter	super_enhancer	AC073255.1
chr2	30655000	chr2	47045000	1.39E-02	promoter	super_enhancer	AC073255.1
chr2	31315000	chr2	43165000	1.86E-02	promoter	super_enhancer	AC009305.2

chr2	31315000	chr2	46105000	1.39E-02	promoter	super_enhancer	AC009305.2
chr2	31875000	chr2	36375000	3.25E-02	promoter	super_enhancer	AL133247.3
chr2	31875000	chr2	36565000	2.68E-02	promoter	super_enhancer	AL133247.3
chr2	32035000	chr2	36405000	2.32E-02	promoter	super_enhancer	AL121652.2
chr2	32035000	chr2	46065000	1.36E-02	promoter	super_enhancer	AL121652.2
chr2	32035000	chr2	46955000	1.13E-02	promoter	super_enhancer	AL121652.2
chr2	32035000	chr2	64835000	7.39E-03	promoter	super_enhancer	AL121652.2
chr2	32145000	chr2	36415000	2.14E-02	promoter	super_enhancer	AL121652.1
chr2	32265000	chr2	36385000	3.90E-02	promoter	super_enhancer	DPY30
chr2	32265000	chr2	71935000	9.31E-03	promoter	super_enhancer	DPY30
chr2	32605000	chr2	70135000	4.86E-03	promoter	super_enhancer	BIRC6-AS1
chr2	32605000	chr2	100885000	2.32E-03	promoter	super_enhancer	BIRC6-AS1
chr2	33165000	chr2	36405000	4.02E-02	promoter	super_enhancer	AL133244.2
chr2	35025000	chr2	43045000	9.59E-03	promoter	super_enhancer	AC012593.1
chr2	35035000	chr2	36355000	1.58E-02	promoter	super_enhancer	RN7SL602P
chr2	35035000	chr2	46995000	9.31E-03	promoter	super_enhancer	RN7SL602P
chr2	35445000	chr2	36415000	1.03E-02	promoter	super_enhancer	AC013442.1
chr2	35695000	chr2	36545000	3.87E-02	promoter	super_enhancer	MIR548AD
chr2	35695000	chr2	47085000	1.17E-02	promoter	super_enhancer	MIR548AD
chr2	36365000	chr2	40155000	3.12E-02	super_enhancer	promoter	AC007253.1
chr2	36375000	chr2	68385000	8.64E-03	super_enhancer	promoter	WDR92
chr2	36385000	chr2	42395000	2.22E-02	super_enhancer	promoter	EML4
chr2	36385000	chr2	52155000	8.44E-03	super_enhancer	promoter	AC097463.1
chr2	36385000	chr2	85615000	3.67E-03	super_enhancer	promoter	RP11-717A5.2

chr2	36385000	chr2	121625000	2.39E-03	super_enhancer	promoter	RP11-297J22.1
chr2	36395000	chr2	45395000	8.95E-03	super_enhancer	promoter	AC009236.2
chr2	36395000	chr2	228735000	1.80E-03	super_enhancer	promoter	TDGF1P2
chr2	36405000	chr2	62115000	6.41E-03	super_enhancer	promoter	COMMD1
chr2	36415000	chr2	74195000	4.45E-03	super_enhancer	promoter	RNA5SP97
chr2	36415000	chr2	75945000	4.01E-03	super_enhancer	promoter	RP11-342K6.4
chr2	36415000	chr2	121225000	1.83E-03	super_enhancer	promoter	LINC01101
chr2	36415000	chr2	189155000	1.76E-03	super_enhancer	promoter	GULP1
chr2	36425000	chr2	46655000	1.82E-02	super_enhancer	promoter	TMEM247
chr2	36425000	chr2	102005000	3.25E-03	super_enhancer	promoter	CREG2
chr2	36425000	chr2	152265000	3.45E-03	super_enhancer	promoter	RIF1
chr2	36425000	chr2	216745000	2.84E-03	super_enhancer	promoter	AC122136.1
chr2	36425000	chr2	242665000	2.48E-03	super_enhancer	promoter	AC114730.11
chr2	36435000	chr2	65685000	7.24E-03	super_enhancer	promoter	AC012370.3
chr2	36435000	chr2	96675000	2.87E-03	super_enhancer	promoter	FAHD2CP
chr2	36435000	chr2	110425000	2.43E-03	super_enhancer	promoter	BMS1P19
chr2	36435000	chr2	145275000	2.52E-03	super_enhancer	promoter	ZEB2-AS1
chr2	36545000	chr2	40155000	4.62E-02	super_enhancer	promoter	AC007253.1
chr2	36545000	chr2	51255000	8.26E-03	super_enhancer	promoter	AC007682.4
chr2	36555000	chr2	90465000	4.43E-03	super_enhancer	promoter	CH17-132F21.4
chr2	36565000	chr2	47185000	1.66E-02	super_enhancer	promoter	RP11-15I20.1
chr2	36565000	chr2	62375000	7.38E-03	super_enhancer	promoter	AC018462.2
chr2	36565000	chr2	90465000	4.80E-03	super_enhancer	promoter	CH17-132F21.4
chr2	36565000	chr2	101925000	2.93E-03	super_enhancer	promoter	RNF149

chr2	36565000	chr2	234655000	1.77E-03	super_enhancer	promoter	UGT1A2P
chr2	36885000	chr2	38085000	2.23E-02	promoter	super_enhancer	RP11-294L11.1
chr2	36885000	chr2	46065000	1.63E-02	promoter	super_enhancer	RP11-294L11.1
chr2	36885000	chr2	46855000	1.46E-02	promoter	super_enhancer	RP11-294L11.1
chr2	37095000	chr2	46855000	1.43E-02	promoter	super_enhancer	RNU6-577P
chr2	37195000	chr2	43155000	3.43E-02	promoter	super_enhancer	STRN
chr2	37195000	chr2	47035000	1.85E-02	promoter	super_enhancer	STRN
chr2	37375000	chr2	38105000	1.07E-03	promoter	super_enhancer	AC007899.3
chr2	37375000	chr2	47015000	1.61E-02	promoter	super_enhancer	AC007899.3
chr2	37375000	chr2	55105000	9.86E-03	promoter	super_enhancer	AC007899.3
chr2	37385000	chr2	43025000	3.85E-02	promoter	super_enhancer	EIF2AK2
chr2	37385000	chr2	46125000	2.36E-02	promoter	super_enhancer	EIF2AK2
chr2	37425000	chr2	38095000	2.41E-02	promoter	super_enhancer	SULT6B1
chr2	37425000	chr2	46195000	1.79E-02	promoter	super_enhancer	SULT6B1
chr2	37425000	chr2	46845000	2.07E-02	promoter	super_enhancer	SULT6B1
chr2	38075000	chr2	47755000	1.80E-02	super_enhancer	promoter	HCG2040054
chr2	38075000	chr2	86115000	4.26E-03	super_enhancer	promoter	ST3GAL5-AS1
chr2	38075000	chr2	103035000	1.73E-03	super_enhancer	promoter	IL18RAP
chr2	38075000	chr2	166325000	1.56E-03	super_enhancer	promoter	CSRNP3
chr2	38075000	chr2	206905000	1.85E-03	super_enhancer	promoter	AC007679.4
chr2	38085000	chr2	74195000	6.37E-03	super_enhancer	promoter	RNA5SP97
chr2	38095000	chr2	38605000	3.73E-02	super_enhancer	promoter	ATL2
chr2	38105000	chr2	42275000	4.22E-02	super_enhancer	promoter	PKDCC
chr2	38105000	chr2	61225000	1.25E-02	super_enhancer	promoter	RNA5SP95

chr2	38105000	chr2	233595000	2.08E-03	super_enhancer	promoter	EEF1B2P7
chr2	38295000	chr2	43225000	2.67E-02	promoter	super_enhancer	RMDN2-AS1
chr2	38375000	chr2	46865000	1.91E-02	promoter	super_enhancer	RNU6-951P
chr2	38375000	chr2	58115000	8.20E-03	promoter	super_enhancer	RNU6-951P
chr2	38605000	chr2	43075000	4.39E-02	promoter	super_enhancer	ATL2
chr2	38605000	chr2	231605000	1.87E-03	promoter	super_enhancer	ATL2
chr2	38995000	chr2	46855000	1.90E-02	promoter	super_enhancer	AC018693.5
chr2	38995000	chr2	234995000	2.52E-03	promoter	super_enhancer	AC018693.5
chr2	39165000	chr2	43185000	4.16E-02	promoter	super_enhancer	RN7SL96P
chr2	39165000	chr2	239265000	2.33E-03	promoter	super_enhancer	RN7SL96P
chr2	39205000	chr2	46075000	2.31E-02	promoter	super_enhancer	AC019171.1
chr2	39205000	chr2	46845000	2.40E-02	promoter	super_enhancer	AC019171.1
chr2	39205000	chr2	47025000	1.60E-02	promoter	super_enhancer	AC019171.1
chr2	39205000	chr2	85465000	5.01E-03	promoter	super_enhancer	AC019171.1
chr2	39665000	chr2	47055000	2.80E-04	promoter	super_enhancer	MAP4K3
chr2	40155000	chr2	47075000	2.57E-02	promoter	super_enhancer	AC007253.1
chr2	42175000	chr2	46965000	2.57E-02	promoter	super_enhancer	AC013480.1
chr2	42175000	chr2	71925000	8.81E-03	promoter	super_enhancer	AC013480.1
chr2	42175000	chr2	120915000	1.88E-03	promoter	super_enhancer	AC013480.1
chr2	42275000	chr2	46965000	3.19E-02	promoter	super_enhancer	PKDCC
chr2	42275000	chr2	55095000	1.78E-02	promoter	super_enhancer	PKDCC
chr2	42275000	chr2	74855000	6.17E-03	promoter	super_enhancer	PKDCC
chr2	42275000	chr2	239275000	2.75E-03	promoter	super_enhancer	PKDCC
chr2	42275000	chr2	242075000	3.41E-03	promoter	super_enhancer	PKDCC

chr2	42395000	chr2	46075000	4.71E-02	promoter	super_enhancer	EML4
chr2	42395000	chr2	46945000	3.28E-02	promoter	super_enhancer	EML4
chr2	42395000	chr2	46965000	2.77E-02	promoter	super_enhancer	EML4
chr2	43025000	chr2	46395000	4.87E-02	super_enhancer	promoter	AC017006.3
chr2	43025000	chr2	64805000	9.46E-03	super_enhancer	promoter	RNU6-100P
chr2	43025000	chr2	189705000	2.74E-03	super_enhancer	promoter	AC079613.1
chr2	43035000	chr2	47405000	4.33E-02	super_enhancer	promoter	RP11-761B3.1
chr2	43035000	chr2	52155000	1.48E-02	super_enhancer	promoter	AC097463.1
chr2	43035000	chr2	58135000	1.03E-02	super_enhancer	promoter	VRK2
chr2	43035000	chr2	73965000	8.92E-03	super_enhancer	promoter	TPRKB
chr2	43035000	chr2	74375000	8.91E-03	super_enhancer	promoter	BOLA3
chr2	43035000	chr2	103035000	2.36E-03	super_enhancer	promoter	IL18RAP
chr2	43035000	chr2	202655000	3.35E-03	super_enhancer	promoter	CDK15
chr2	43045000	chr2	43455000	1.27E-02	super_enhancer	promoter	ZFP36L2
chr2	43045000	chr2	44065000	4.68E-03	super_enhancer	promoter	ABCG8
chr2	43055000	chr2	85645000	8.47E-03	super_enhancer	promoter	SH2D6
chr2	43055000	chr2	204955000	2.11E-03	super_enhancer	promoter	AC009965.2
chr2	43065000	chr2	43455000	1.06E-02	super_enhancer	promoter	ZFP36L2
chr2	43065000	chr2	48025000	4.23E-02	super_enhancer	promoter	RPL36AP15
chr2	43065000	chr2	131595000	3.72E-03	super_enhancer	promoter	AC133785.1
chr2	43065000	chr2	242005000	4.04E-03	super_enhancer	promoter	AC005237.4
chr2	43075000	chr2	43455000	1.61E-02	super_enhancer	promoter	ZFP36L2
chr2	43075000	chr2	112945000	4.45E-03	super_enhancer	promoter	RP11-399B17.1
chr2	43085000	chr2	51255000	1.51E-02	super_enhancer	promoter	AC007682.4

chr2	43085000	chr2	65045000	1.23E-02	super_enhancer	promoter	RN7SL341P
chr2	43085000	chr2	101685000	3.65E-03	super_enhancer	promoter	RP11-426K3.1
chr2	43085000	chr2	219135000	3.81E-03	super_enhancer	promoter	AAMP
chr2	43155000	chr2	54805000	2.25E-02	super_enhancer	promoter	AC093110.1
chr2	43155000	chr2	63815000	1.37E-02	super_enhancer	promoter	MDH1
chr2	43155000	chr2	79385000	6.87E-03	super_enhancer	promoter	AC011754.1
chr2	43155000	chr2	85795000	8.81E-03	super_enhancer	promoter	AC016753.1
chr2	43155000	chr2	142895000	2.65E-03	super_enhancer	promoter	AC078882.1
chr2	43165000	chr2	43455000	1.03E-02	super_enhancer	promoter	ZFP36L2
chr2	43165000	chr2	208135000	2.26E-03	super_enhancer	promoter	MIR1302-4
chr2	43165000	chr2	241395000	2.88E-03	super_enhancer	promoter	MIR149
chr2	43175000	chr2	43455000	1.53E-02	super_enhancer	promoter	ZFP36L2
chr2	43175000	chr2	44395000	2.24E-02	super_enhancer	promoter	PPM1B
chr2	43175000	chr2	59875000	1.04E-02	super_enhancer	promoter	RNU6-508P
chr2	43175000	chr2	105655000	2.78E-03	super_enhancer	promoter	AC010884.1
chr2	43175000	chr2	108835000	2.00E-03	super_enhancer	promoter	LINC01594
chr2	43175000	chr2	153245000	2.48E-03	super_enhancer	promoter	AC012066.1
chr2	43175000	chr2	236945000	3.08E-03	super_enhancer	promoter	RNU7-127P
chr2	43185000	chr2	46655000	4.86E-02	super_enhancer	promoter	TMEM247
chr2	43185000	chr2	47295000	4.62E-02	super_enhancer	promoter	AC073283.7
chr2	43195000	chr2	43455000	9.03E-04	super_enhancer	promoter	ZFP36L2
chr2	43195000	chr2	75185000	8.14E-03	super_enhancer	promoter	POLE4
chr2	43195000	chr2	103035000	2.38E-03	super_enhancer	promoter	IL18RAP
chr2	43225000	chr2	54805000	1.79E-02	super_enhancer	promoter	AC093110.1

chr2	43225000	chr2	89215000	3.62E-03	super_enhancer	promoter	IGKV7-3
chr2	43455000	chr2	177245000	1.83E-03	promoter	super_enhancer	ZFP36L2
chr2	43635000	chr2	47035000	4.17E-02	promoter	super_enhancer	RNU6-958P
chr2	43635000	chr2	84945000	4.43E-03	promoter	super_enhancer	RNU6-958P
chr2	44065000	chr2	64835000	1.35E-02	promoter	super_enhancer	ABCG8
chr2	44395000	chr2	55115000	1.12E-02	promoter	super_enhancer	PPM1B
chr2	45165000	chr2	58115000	1.88E-02	promoter	super_enhancer	Six3os1_5
chr2	45165000	chr2	84905000	1.05E-02	promoter	super_enhancer	Six3os1_5
chr2	45165000	chr2	237475000	2.67E-03	promoter	super_enhancer	Six3os1_5
chr2	46065000	chr2	71255000	8.69E-03	super_enhancer	promoter	OR7E91P
chr2	46065000	chr2	162935000	1.91E-03	super_enhancer	promoter	TIMM8AP1
chr2	46085000	chr2	61225000	1.85E-02	super_enhancer	promoter	RNA5SP95
chr2	46085000	chr2	86795000	5.30E-03	super_enhancer	promoter	AC015971.3
chr2	46085000	chr2	112945000	3.34E-03	super_enhancer	promoter	RP11-399B17.1
chr2	46085000	chr2	144535000	1.81E-03	super_enhancer	promoter	CTD-2252P21.1
chr2	46095000	chr2	50105000	2.82E-02	super_enhancer	promoter	RPL7P13
chr2	46095000	chr2	55335000	1.71E-02	super_enhancer	promoter	AC093165.1
chr2	46095000	chr2	70315000	7.78E-03	super_enhancer	promoter	PCBP1
chr2	46095000	chr2	70995000	9.34E-03	super_enhancer	promoter	ADD2
chr2	46105000	chr2	54805000	1.85E-02	super_enhancer	promoter	AC093110.1
chr2	46105000	chr2	59875000	9.52E-03	super_enhancer	promoter	RNU6-508P
chr2	46105000	chr2	74055000	8.12E-03	super_enhancer	promoter	STAMBP
chr2	46105000	chr2	92145000	2.39E-03	super_enhancer	promoter	CHEK2P3
chr2	46105000	chr2	146095000	2.01E-03	super_enhancer	promoter	RPL6P5

chr2	46115000	chr2	60645000	7.15E-03	super_enhancer	promoter	AC007381.1
chr2	46115000	chr2	85295000	4.28E-03	super_enhancer	promoter	RP11-60H5.1
chr2	46115000	chr2	219745000	1.59E-03	super_enhancer	promoter	WNT10A
chr2	46125000	chr2	53995000	1.85E-02	super_enhancer	promoter	CHAC2
chr2	46125000	chr2	60645000	1.18E-02	super_enhancer	promoter	AC007381.1
chr2	46125000	chr2	61245000	1.64E-02	super_enhancer	promoter	PUS10
chr2	46125000	chr2	86565000	6.54E-03	super_enhancer	promoter	REEP1
chr2	46125000	chr2	208135000	2.18E-03	super_enhancer	promoter	MIR1302-4
chr2	46135000	chr2	47125000	4.39E-02	super_enhancer	promoter	AC016722.4
chr2	46135000	chr2	218115000	2.05E-03	super_enhancer	promoter	RN7SKP43
chr2	46135000	chr2	242665000	2.48E-03	super_enhancer	promoter	AC114730.11
chr2	46145000	chr2	47185000	4.80E-02	super_enhancer	promoter	RP11-15I20.1
chr2	46145000	chr2	47605000	2.16E-02	super_enhancer	promoter	MIR559
chr2	46145000	chr2	73965000	9.05E-03	super_enhancer	promoter	TPRKB
chr2	46165000	chr2	65045000	1.06E-02	super_enhancer	promoter	RN7SL341P
chr2	46185000	chr2	46925000	1.23E-02	super_enhancer	promoter	SOCS5
chr2	46185000	chr2	47295000	4.02E-03	super_enhancer	promoter	AC073283.7
chr2	46185000	chr2	71265000	6.54E-03	super_enhancer	promoter	OR7E46P
chr2	46185000	chr2	81895000	4.27E-03	super_enhancer	promoter	AC013262.1
chr2	46195000	chr2	46655000	1.06E-04	super_enhancer	promoter	TMEM247
chr2	46195000	chr2	47295000	4.38E-02	super_enhancer	promoter	AC073283.7
chr2	46195000	chr2	75825000	7.32E-03	super_enhancer	promoter	AC005034.2
chr2	46195000	chr2	80095000	6.55E-03	super_enhancer	promoter	MIR8080
chr2	46195000	chr2	113815000	3.29E-03	super_enhancer	promoter	IL36RN

chr2	46305000	chr2	47125000	4.44E-02	super_enhancer	promoter	AC016722.4
chr2	46305000	chr2	65135000	1.04E-02	super_enhancer	promoter	RN7SL211P
chr2	46305000	chr2	65685000	9.40E-03	super_enhancer	promoter	AC012370.3
chr2	46305000	chr2	71205000	7.67E-03	super_enhancer	promoter	ANKRD53
chr2	46305000	chr2	73385000	1.05E-02	super_enhancer	promoter	RAB11FIP5
chr2	46315000	chr2	47185000	1.32E-02	super_enhancer	promoter	RP11-15I20.1
chr2	46315000	chr2	54755000	2.43E-02	super_enhancer	promoter	RPL23AP32
chr2	46315000	chr2	139035000	2.48E-03	super_enhancer	promoter	AC097523.1
chr2	46395000	chr2	70085000	8.08E-03	promoter	super_enhancer	AC017006.3
chr2	46655000	chr2	46845000	2.61E-02	promoter	super_enhancer	TMEM247
chr2	46845000	chr2	47125000	3.47E-02	super_enhancer	promoter	AC016722.4
chr2	46845000	chr2	62115000	1.75E-02	super_enhancer	promoter	COMMD1
chr2	46845000	chr2	64055000	1.02E-02	super_enhancer	promoter	WDPCP
chr2	46855000	chr2	166325000	1.65E-03	super_enhancer	promoter	CSRNP3
chr2	46855000	chr2	239895000	2.29E-03	super_enhancer	promoter	AC114788.1
chr2	46865000	chr2	47185000	3.60E-02	super_enhancer	promoter	RP11-15I20.1
chr2	46865000	chr2	48445000	2.12E-02	super_enhancer	promoter	RN7SKP224
chr2	46865000	chr2	55335000	2.13E-02	super_enhancer	promoter	AC093165.1
chr2	46865000	chr2	55845000	1.63E-02	super_enhancer	promoter	RP11-554J4.1
chr2	46865000	chr2	131975000	1.47E-03	super_enhancer	promoter	POTEE
chr2	46865000	chr2	213775000	1.54E-03	super_enhancer	promoter	AC108066.1
chr2	46865000	chr2	220175000	3.54E-03	super_enhancer	promoter	PTPRN
chr2	46875000	chr2	47125000	6.74E-03	super_enhancer	promoter	AC016722.4
chr2	46895000	chr2	46965000	3.59E-02	promoter	super_enhancer	RP11-536C12.1

chr2	46925000	chr2	46995000	4.82E-03	promoter	super_enhancer	SOCS5
chr2	46925000	chr2	47055000	1.59E-02	promoter	super_enhancer	SOCS5
chr2	46945000	chr2	53995000	1.97E-02	super_enhancer	promoter	CHAC2
chr2	46945000	chr2	62095000	1.37E-02	super_enhancer	promoter	AC107081.5
chr2	46945000	chr2	202005000	2.48E-03	super_enhancer	promoter	IMPDH1P10
chr2	46955000	chr2	54755000	2.03E-02	super_enhancer	promoter	RPL23AP32
chr2	46955000	chr2	74105000	8.00E-03	super_enhancer	promoter	RP11-51I5.1
chr2	46955000	chr2	75145000	9.42E-03	super_enhancer	promoter	LINC01291
chr2	46955000	chr2	234525000	1.78E-03	super_enhancer	promoter	UGT1A8
chr2	46965000	chr2	62115000	1.18E-02	super_enhancer	promoter	COMMD1
chr2	46965000	chr2	65045000	9.76E-03	super_enhancer	promoter	RN7SL341P
chr2	46965000	chr2	71115000	9.03E-03	super_enhancer	promoter	LINC01143
chr2	46965000	chr2	71405000	7.91E-03	super_enhancer	promoter	AC007881.4
chr2	46965000	chr2	91925000	5.13E-03	super_enhancer	promoter	AC027612.4
chr2	46975000	chr2	55335000	1.58E-02	super_enhancer	promoter	AC093165.1
chr2	46975000	chr2	62735000	8.79E-03	super_enhancer	promoter	AC107083.1
chr2	46975000	chr2	74375000	7.48E-03	super_enhancer	promoter	BOLA3
chr2	46975000	chr2	75145000	8.71E-03	super_enhancer	promoter	LINC01291
chr2	46985000	chr2	71295000	1.00E-02	super_enhancer	promoter	RP11-467P9.1
chr2	46995000	chr2	85795000	5.88E-03	super_enhancer	promoter	AC016753.1
chr2	46995000	chr2	241375000	2.74E-03	super_enhancer	promoter	GPC1
chr2	47005000	chr2	203045000	2.49E-03	super_enhancer	promoter	AC079354.5
chr2	47015000	chr2	71295000	1.04E-02	super_enhancer	promoter	RP11-467P9.1
chr2	47015000	chr2	73525000	7.70E-03	super_enhancer	promoter	RNU6-111P

chr2	47015000	chr2	85795000	7.09E-03	super_enhancer	promoter	AC016753.1
chr2	47025000	chr2	74685000	8.44E-03	super_enhancer	promoter	WBP1
chr2	47025000	chr2	132145000	1.52E-03	super_enhancer	promoter	MTND1P26
chr2	47025000	chr2	159305000	2.18E-03	super_enhancer	promoter	RN7SL393P
chr2	47025000	chr2	175585000	1.85E-03	super_enhancer	promoter	H3F3AP4
chr2	47025000	chr2	240015000	1.69E-03	super_enhancer	promoter	AC017028.12
chr2	47035000	chr2	54755000	2.40E-02	super_enhancer	promoter	RPL23AP32
chr2	47035000	chr2	62395000	1.75E-02	super_enhancer	promoter	RP11-270B14.1
chr2	47035000	chr2	217725000	2.86E-03	super_enhancer	promoter	TNP1
chr2	47045000	chr2	47605000	4.41E-02	super_enhancer	promoter	MIR559
chr2	47045000	chr2	54395000	2.48E-02	super_enhancer	promoter	RNU7-172P
chr2	47045000	chr2	85525000	7.80E-03	super_enhancer	promoter	AC093162.1
chr2	47045000	chr2	110875000	3.38E-03	super_enhancer	promoter	MALL
chr2	47045000	chr2	220265000	3.38E-03	super_enhancer	promoter	DNPEP
chr2	47055000	chr2	80095000	7.25E-03	super_enhancer	promoter	MIR8080
chr2	47055000	chr2	107095000	2.06E-03	super_enhancer	promoter	AC108868.3
chr2	47055000	chr2	130635000	1.57E-03	super_enhancer	promoter	AC079776.1
chr2	47055000	chr2	232645000	3.49E-03	super_enhancer	promoter	COPS7B
chr2	47065000	chr2	61245000	2.13E-02	super_enhancer	promoter	PUS10
chr2	47065000	chr2	64055000	1.05E-02	super_enhancer	promoter	WDPCP
chr2	47065000	chr2	71295000	1.39E-02	super_enhancer	promoter	RP11-467P9.1
chr2	47065000	chr2	71405000	1.21E-02	super_enhancer	promoter	AC007881.4
chr2	47065000	chr2	85615000	6.92E-03	super_enhancer	promoter	RP11-717A5.2
chr2	47075000	chr2	54775000	1.92E-02	super_enhancer	promoter	AC092839.4

chr2	47075000	chr2	56375000	1.47E-02	super_enhancer	promoter	RP11-482H16.1
chr2	47075000	chr2	239645000	2.00E-03	super_enhancer	promoter	AC113618.2
chr2	47185000	chr2	70095000	1.04E-02	promoter	super_enhancer	RP11-15I20.1
chr2	47295000	chr2	71935000	1.52E-02	promoter	super_enhancer	AC073283.7
chr2	47295000	chr2	218395000	2.40E-03	promoter	super_enhancer	AC073283.7
chr2	47295000	chr2	231615000	2.88E-03	promoter	super_enhancer	AC073283.7
chr2	47295000	chr2	233415000	3.00E-03	promoter	super_enhancer	AC073283.7
chr2	47295000	chr2	238445000	3.32E-03	promoter	super_enhancer	AC073283.7
chr2	47405000	chr2	85445000	6.27E-03	promoter	super_enhancer	RP11-761B3.1
chr2	47405000	chr2	112645000	3.07E-03	promoter	super_enhancer	RP11-761B3.1
chr2	47605000	chr2	71935000	1.54E-02	promoter	super_enhancer	MIR559
chr2	48025000	chr2	70095000	1.00E-02	promoter	super_enhancer	RPL36AP15
chr2	48445000	chr2	84965000	7.47E-03	promoter	super_enhancer	RN7SKP224
chr2	48845000	chr2	70085000	1.04E-02	promoter	super_enhancer	GTF2A1L
chr2	50105000	chr2	74845000	7.25E-03	promoter	super_enhancer	RPL7P13
chr2	54395000	chr2	85455000	8.74E-03	promoter	super_enhancer	RNU7-172P
chr2	54395000	chr2	101115000	4.55E-03	promoter	super_enhancer	RNU7-172P
chr2	54565000	chr2	74685000	1.10E-02	super_enhancer	promoter	WBP1
chr2	54565000	chr2	86425000	6.62E-03	super_enhancer	promoter	MRPL35
chr2	54565000	chr2	159275000	1.66E-03	super_enhancer	promoter	HNRNPDLP2
chr2	54575000	chr2	85615000	4.34E-03	super_enhancer	promoter	RP11-717A5.2
chr2	54755000	chr2	113225000	4.19E-03	promoter	super_enhancer	RPL23AP32
chr2	54755000	chr2	235005000	2.67E-03	promoter	super_enhancer	RPL23AP32
chr2	54775000	chr2	74845000	1.03E-02	promoter	super_enhancer	AC092839.4

chr2	54805000	chr2	64845000	1.77E-02	promoter	super_enhancer	AC093110.1
chr2	54805000	chr2	112825000	3.67E-03	promoter	super_enhancer	AC093110.1
chr2	55095000	chr2	239335000	2.27E-03	super_enhancer	promoter	ASB1
chr2	55105000	chr2	60615000	2.72E-02	super_enhancer	promoter	MIR4432
chr2	55105000	chr2	62085000	1.89E-02	super_enhancer	promoter	RP11-681L4.2
chr2	55105000	chr2	65455000	1.32E-02	super_enhancer	promoter	ACTR2
chr2	55115000	chr2	69065000	1.11E-02	super_enhancer	promoter	AC097495.3
chr2	55335000	chr2	235005000	2.37E-03	promoter	super_enhancer	AC093165.1
chr2	55845000	chr2	84905000	7.54E-03	promoter	super_enhancer	RP11-554J4.1
chr2	56275000	chr2	177245000	1.14E-03	promoter	super_enhancer	MIR217HG
chr2	58755000	chr2	70095000	1.38E-02	promoter	super_enhancer	AC007367.1
chr2	60645000	chr2	84945000	8.14E-03	promoter	super_enhancer	AC007381.1
chr2	60645000	chr2	109255000	2.44E-03	promoter	super_enhancer	AC007381.1
chr2	61405000	chr2	85455000	1.22E-02	promoter	super_enhancer	AHSA2
chr2	61645000	chr2	112825000	4.02E-03	promoter	super_enhancer	AC016894.1
chr2	62085000	chr2	84905000	1.19E-02	promoter	super_enhancer	RP11-681L4.2
chr2	62095000	chr2	70095000	2.03E-02	promoter	super_enhancer	AC107081.5
chr2	62115000	chr2	101115000	5.62E-03	promoter	super_enhancer	COMMD1
chr2	62115000	chr2	112795000	4.44E-03	promoter	super_enhancer	COMMD1
chr2	62375000	chr2	112625000	3.33E-03	promoter	super_enhancer	AC018462.2
chr2	64055000	chr2	71935000	1.98E-02	promoter	super_enhancer	WDPCP
chr2	64055000	chr2	84965000	7.24E-03	promoter	super_enhancer	WDPCP
chr2	64055000	chr2	109255000	2.13E-03	promoter	super_enhancer	WDPCP
chr2	64055000	chr2	113205000	2.09E-03	promoter	super_enhancer	WDPCP

chr2	64315000	chr2	85455000	9.60E-03	promoter	super_enhancer	AC012368.1
chr2	64835000	chr2	86115000	1.19E-02	super_enhancer	promoter	ST3GAL5-AS1
chr2	64845000	chr2	65135000	6.81E-04	super_enhancer	promoter	RN7SL211P
chr2	64845000	chr2	78825000	9.12E-03	super_enhancer	promoter	AC092660.1
chr2	64845000	chr2	85525000	1.21E-02	super_enhancer	promoter	AC093162.1
chr2	64845000	chr2	85595000	6.46E-05	super_enhancer	promoter	RN7SL113P
chr2	64845000	chr2	85915000	1.05E-02	super_enhancer	promoter	AC012454.4
chr2	64855000	chr2	71205000	2.70E-02	super_enhancer	promoter	ANKRD53
chr2	64855000	chr2	83885000	7.30E-03	super_enhancer	promoter	RNU6-1312P
chr2	64855000	chr2	96035000	7.83E-03	super_enhancer	promoter	FABP7P2
chr2	65455000	chr2	84945000	1.07E-02	promoter	super_enhancer	ACTR2
chr2	69615000	chr2	113225000	5.19E-03	promoter	super_enhancer	GFPT1
chr2	69665000	chr2	70085000	4.93E-02	promoter	super_enhancer	NFU1
chr2	69665000	chr2	112625000	4.43E-03	promoter	super_enhancer	NFU1
chr2	69665000	chr2	242075000	3.03E-03	promoter	super_enhancer	NFU1
chr2	70085000	chr2	220175000	3.12E-03	super_enhancer	promoter	PTPRN
chr2	70095000	chr2	70315000	2.36E-02	super_enhancer	promoter	PCBP1
chr2	70095000	chr2	85645000	1.79E-02	super_enhancer	promoter	SH2D6
chr2	70095000	chr2	111725000	4.03E-03	super_enhancer	promoter	RPL5P9
chr2	70095000	chr2	233515000	2.47E-03	super_enhancer	promoter	RN7SL359P
chr2	70105000	chr2	71265000	1.73E-02	super_enhancer	promoter	OR7E46P
chr2	70135000	chr2	88665000	1.03E-02	super_enhancer	promoter	MRPL45P1
chr2	70145000	chr2	70485000	1.01E-02	super_enhancer	promoter	PCYOX1
chr2	70145000	chr2	155315000	1.45E-03	super_enhancer	promoter	AC009227.2

chr2	70145000	chr2	242675000	1.89E-03	super_enhancer	promoter	AC114730.8
chr2	70995000	chr2	85445000	1.47E-02	promoter	super_enhancer	ADD2
chr2	70995000	chr2	112625000	4.81E-03	promoter	super_enhancer	ADD2
chr2	71115000	chr2	112645000	6.03E-03	promoter	super_enhancer	LINC01143
chr2	71925000	chr2	72375000	8.96E-03	super_enhancer	promoter	CYP26B1
chr2	71925000	chr2	79775000	1.72E-02	super_enhancer	promoter	RP11-191H23.1
chr2	71925000	chr2	112895000	5.76E-03	super_enhancer	promoter	FBLN7
chr2	71925000	chr2	220175000	3.78E-03	super_enhancer	promoter	PTPRN
chr2	71935000	chr2	85135000	2.35E-02	super_enhancer	promoter	TRABD2A
chr2	71935000	chr2	86565000	2.31E-02	super_enhancer	promoter	REEP1
chr2	71935000	chr2	170935000	3.00E-03	super_enhancer	promoter	AC092641.2
chr2	71935000	chr2	239465000	3.43E-03	super_enhancer	promoter	LINC01107
chr2	71945000	chr2	79315000	2.45E-02	super_enhancer	promoter	REG1B
chr2	71945000	chr2	85645000	2.51E-02	super_enhancer	promoter	SH2D6
chr2	72375000	chr2	84905000	2.51E-02	promoter	super_enhancer	CYP26B1
chr2	72375000	chr2	84945000	2.33E-04	promoter	super_enhancer	CYP26B1
chr2	72515000	chr2	101135000	6.14E-03	promoter	super_enhancer	AC006461.2
chr2	73385000	chr2	101125000	8.96E-03	promoter	super_enhancer	RAB11FIP5
chr2	74055000	chr2	230785000	2.39E-03	promoter	super_enhancer	STAMBP
chr2	74195000	chr2	237465000	2.37E-03	promoter	super_enhancer	RNA5SP97
chr2	74425000	chr2	112815000	5.09E-03	promoter	super_enhancer	MTHFD2
chr2	74685000	chr2	74855000	1.37E-02	promoter	super_enhancer	WBP1
chr2	74685000	chr2	112835000	7.23E-03	promoter	super_enhancer	WBP1
chr2	74775000	chr2	85435000	1.74E-02	promoter	super_enhancer	DOK1

chr2	74775000	chr2	101115000	8.45E-03	promoter	super_enhancer	DOK1	
chr2	74845000	chr2	203045000	2.58E-03	super_enhancer	promoter	AC079354.5	
chr2	74845000	chr2	242255000	2.59E-03	super_enhancer	promoter		2-Sep
chr2	74855000	chr2	241395000	2.10E-03	super_enhancer	promoter	MIR149	
chr2	74865000	chr2	101685000	6.30E-03	super_enhancer	promoter	RP11-426K3.1	
chr2	75145000	chr2	84965000	2.28E-02	promoter	super_enhancer	LINC01291	
chr2	75145000	chr2	85465000	2.41E-02	promoter	super_enhancer	LINC01291	
chr2	75685000	chr2	84915000	1.68E-02	promoter	super_enhancer	GAPDHP57	
chr2	75685000	chr2	84955000	1.57E-02	promoter	super_enhancer	GAPDHP57	
chr2	75925000	chr2	85465000	1.98E-02	promoter	super_enhancer	RP11-342K6.3	
chr2	75945000	chr2	85425000	1.64E-02	promoter	super_enhancer	RP11-342K6.4	
chr2	76335000	chr2	84905000	1.73E-02	promoter	super_enhancer	SUCLA2P2	
chr2	76335000	chr2	84945000	1.48E-02	promoter	super_enhancer	SUCLA2P2	
chr2	78015000	chr2	84955000	1.25E-02	promoter	super_enhancer	AC105399.2	
chr2	78015000	chr2	112805000	3.94E-03	promoter	super_enhancer	AC105399.2	
chr2	78825000	chr2	113225000	4.78E-03	promoter	super_enhancer	AC092660.1	
chr2	79315000	chr2	84915000	2.45E-02	promoter	super_enhancer	REG1B	
chr2	79385000	chr2	235025000	1.66E-03	promoter	super_enhancer	AC011754.1	
chr2	79865000	chr2	231615000	1.62E-03	promoter	super_enhancer	RNU6-561P	
chr2	81895000	chr2	85425000	3.35E-02	promoter	super_enhancer	AC013262.1	
chr2	81895000	chr2	85465000	3.61E-02	promoter	super_enhancer	AC013262.1	
chr2	81895000	chr2	230795000	1.65E-03	promoter	super_enhancer	AC013262.1	
chr2	84905000	chr2	128285000	7.51E-03	super_enhancer	promoter	IWS1	
chr2	84945000	chr2	88665000	4.50E-02	super_enhancer	promoter	MRPL45P1	

chr2	84955000	chr2	113825000	8.08E-03	super_enhancer	promoter	IL1F10
chr2	84965000	chr2	88355000	4.67E-02	super_enhancer	promoter	KRCC1
chr2	84965000	chr2	92145000	1.22E-02	super_enhancer	promoter	CHEK2P3
chr2	85135000	chr2	112815000	8.86E-03	promoter	super_enhancer	TRABD2A
chr2	85135000	chr2	142875000	3.00E-03	promoter	super_enhancer	TRABD2A
chr2	85295000	chr2	85435000	4.67E-02	promoter	super_enhancer	RP11-60H5.1
chr2	85425000	chr2	109745000	5.85E-03	super_enhancer	promoter	SH3RF3-AS1
chr2	85435000	chr2	91875000	2.12E-02	super_enhancer	promoter	DRD5P1
chr2	85445000	chr2	85615000	4.97E-02	super_enhancer	promoter	RP11-717A5.2
chr2	85465000	chr2	136745000	4.86E-03	super_enhancer	promoter	DARS
chr2	85595000	chr2	230775000	2.20E-03	promoter	super_enhancer	RN7SL113P
chr2	85795000	chr2	234975000	4.04E-06	promoter	super_enhancer	AC016753.1
chr2	86425000	chr2	234995000	2.86E-03	promoter	super_enhancer	MRPL35
chr2	86565000	chr2	113205000	6.88E-03	promoter	super_enhancer	REEP1
chr2	86565000	chr2	200785000	2.64E-03	promoter	super_enhancer	REEP1
chr2	88715000	chr2	113215000	8.21E-03	promoter	super_enhancer	RNU6-1007P
chr2	90465000	chr2	238445000	3.86E-03	promoter	super_enhancer	CH17-132F21.4
chr2	91875000	chr2	127095000	4.85E-03	promoter	super_enhancer	DRD5P1
chr2	91925000	chr2	127095000	6.74E-03	promoter	super_enhancer	AC027612.4
chr2	95815000	chr2	110105000	1.08E-02	promoter	super_enhancer	MRPS5
chr2	95815000	chr2	127635000	5.12E-03	promoter	super_enhancer	MRPS5
chr2	96035000	chr2	101135000	2.52E-02	promoter	super_enhancer	FABP7P2
chr2	96035000	chr2	112825000	1.06E-02	promoter	super_enhancer	FABP7P2
chr2	96675000	chr2	177255000	1.86E-03	promoter	super_enhancer	FAHD2CP

chr2	97175000	chr2	101135000	3.93E-02	promoter	super_enhancer	NEURL3
chr2	99225000	chr2	110105000	1.01E-04	promoter	super_enhancer	COA5
chr2	100825000	chr2	112625000	8.53E-03	promoter	super_enhancer	LINC01104
chr2	100885000	chr2	105655000	2.29E-02	super_enhancer	promoter	AC010884.1
chr2	100885000	chr2	136595000	5.51E-03	super_enhancer	promoter	LCT
chr2	100895000	chr2	101595000	3.92E-02	super_enhancer	promoter	AC016738.3
chr2	100895000	chr2	135555000	4.24E-03	super_enhancer	promoter	AC110620.2
chr2	100895000	chr2	171265000	1.75E-03	super_enhancer	promoter	AC012594.1
chr2	101115000	chr2	104765000	2.53E-02	super_enhancer	promoter	AC096554.1
chr2	101115000	chr2	105185000	2.68E-02	super_enhancer	promoter	AC013402.1
chr2	101115000	chr2	107155000	1.90E-02	super_enhancer	promoter	AC108868.5
chr2	101115000	chr2	112055000	7.04E-03	super_enhancer	promoter	RPS14P4
chr2	101125000	chr2	117485000	7.52E-03	super_enhancer	promoter	AC062016.1
chr2	101125000	chr2	136835000	4.20E-03	super_enhancer	promoter	AC068492.1
chr2	101125000	chr2	223785000	1.23E-03	super_enhancer	promoter	AC097461.4
chr2	101135000	chr2	105295000	2.65E-02	super_enhancer	promoter	RPL23AP27
chr2	101595000	chr2	112825000	1.37E-02	promoter	super_enhancer	AC016738.3
chr2	102095000	chr2	240435000	1.37E-03	promoter	super_enhancer	AC092570.2
chr2	103055000	chr2	200745000	1.74E-03	promoter	super_enhancer	AC007278.3
chr2	105275000	chr2	109255000	1.54E-02	promoter	super_enhancer	AC013402.5
chr2	105295000	chr2	110105000	2.31E-02	promoter	super_enhancer	RPL23AP27
chr2	105895000	chr2	160215000	2.90E-03	promoter	super_enhancer	AC012360.1
chr2	105895000	chr2	160225000	3.42E-03	promoter	super_enhancer	AC012360.1
chr2	106795000	chr2	109265000	4.18E-02	promoter	super_enhancer	AC018878.3

chr2	106795000	chr2	200765000	1.93E-03	promoter	super_enhancer	AC018878.3
chr2	107155000	chr2	239265000	1.89E-03	promoter	super_enhancer	AC108868.5
chr2	107315000	chr2	127095000	5.65E-03	promoter	super_enhancer	EEF1A1P12
chr2	108715000	chr2	127635000	6.91E-03	promoter	super_enhancer	ACTP1
chr2	108905000	chr2	113215000	2.76E-02	promoter	super_enhancer	SULT1C2
chr2	108905000	chr2	142875000	3.82E-03	promoter	super_enhancer	SULT1C2
chr2	109245000	chr2	237075000	2.21E-03	super_enhancer	promoter	AC079135.1
chr2	109255000	chr2	128175000	7.82E-03	super_enhancer	promoter	PROC
chr2	109265000	chr2	110495000	3.11E-03	super_enhancer	promoter	RPL22P11
chr2	109745000	chr2	110105000	1.64E-02	promoter	super_enhancer	SH3RF3-AS1
chr2	110495000	chr2	112825000	4.26E-02	promoter	super_enhancer	RPL22P11
chr2	110875000	chr2	200775000	2.37E-03	promoter	super_enhancer	MALL
chr2	111965000	chr2	112795000	4.90E-02	promoter	super_enhancer	AC108463.2
chr2	112625000	chr2	113825000	2.41E-02	super_enhancer	promoter	IL1F10
chr2	112625000	chr2	197125000	1.82E-03	super_enhancer	promoter	AC020571.3
chr2	112645000	chr2	112895000	1.62E-02	super_enhancer	promoter	FBLN7
chr2	112645000	chr2	121225000	1.75E-02	super_enhancer	promoter	LINC01101
chr2	112655000	chr2	112795000	4.74E-02	promoter	super_enhancer	MERTK
chr2	112655000	chr2	113225000	1.70E-02	promoter	super_enhancer	MERTK
chr2	112655000	chr2	113245000	1.13E-02	promoter	super_enhancer	MERTK
chr2	112785000	chr2	113865000	3.57E-02	super_enhancer	promoter	IL1RN
chr2	112785000	chr2	171265000	3.52E-03	super_enhancer	promoter	AC012594.1
chr2	112785000	chr2	232395000	2.30E-03	super_enhancer	promoter	NMUR1
chr2	112795000	chr2	113915000	4.41E-02	super_enhancer	promoter	PSD4

chr2	112795000	chr2	121625000	1.96E-02	super_enhancer	promoter	RP11-297J22.1
chr2	112795000	chr2	169105000	2.94E-03	super_enhancer	promoter	STK39
chr2	112795000	chr2	199565000	1.40E-03	super_enhancer	promoter	AC068121.1
chr2	112805000	chr2	113915000	4.49E-02	super_enhancer	promoter	PSD4
chr2	112805000	chr2	128825000	1.47E-02	super_enhancer	promoter	AC108059.1
chr2	112815000	chr2	121225000	1.83E-02	super_enhancer	promoter	LINC01101
chr2	112815000	chr2	159825000	4.38E-03	super_enhancer	promoter	TANC1
chr2	112815000	chr2	220085000	2.55E-03	super_enhancer	promoter	ABCB6
chr2	112825000	chr2	132145000	7.77E-03	super_enhancer	promoter	MTND1P26
chr2	112825000	chr2	172865000	3.74E-03	super_enhancer	promoter	SLC25A12
chr2	112825000	chr2	182545000	3.08E-03	super_enhancer	promoter	CERKL
chr2	112825000	chr2	219135000	2.90E-03	super_enhancer	promoter	AAMP
chr2	112835000	chr2	136635000	7.91E-03	super_enhancer	promoter	MCM6
chr2	112895000	chr2	142885000	4.68E-03	promoter	super_enhancer	FBLN7
chr2	112945000	chr2	113215000	3.37E-02	promoter	super_enhancer	RP11-399B17.1
chr2	112945000	chr2	113225000	3.91E-02	promoter	super_enhancer	RP11-399B17.1
chr2	112945000	chr2	113235000	2.57E-02	promoter	super_enhancer	RP11-399B17.1
chr2	113045000	chr2	113225000	1.66E-02	promoter	super_enhancer	AC115115.3
chr2	113215000	chr2	120975000	1.81E-02	super_enhancer	promoter	AC012363.4
chr2	113235000	chr2	113815000	3.76E-03	super_enhancer	promoter	IL36RN
chr2	113235000	chr2	121225000	1.84E-02	super_enhancer	promoter	LINC01101
chr2	113245000	chr2	128285000	1.50E-02	super_enhancer	promoter	IWS1
chr2	113245000	chr2	162935000	3.08E-03	super_enhancer	promoter	TIMM8AP1
chr2	113245000	chr2	233515000	2.36E-03	super_enhancer	promoter	RN7SL359P

chr2	113245000	chr2	241505000	2.43E-03	super_enhancer	promoter	RNPEPL1
chr2	113595000	chr2	160215000	5.30E-03	promoter	super_enhancer	IL1B
chr2	113595000	chr2	177245000	2.60E-03	promoter	super_enhancer	IL1B
chr2	113815000	chr2	127085000	1.32E-02	promoter	super_enhancer	IL36RN
chr2	113825000	chr2	127095000	1.37E-02	promoter	super_enhancer	IL1F10
chr2	113865000	chr2	239265000	2.17E-03	promoter	super_enhancer	IL1RN
chr2	113915000	chr2	142885000	6.91E-03	promoter	super_enhancer	PSD4
chr2	113915000	chr2	238445000	3.58E-03	promoter	super_enhancer	PSD4
chr2	114195000	chr2	160225000	2.58E-03	promoter	super_enhancer	CBWD2
chr2	114345000	chr2	127085000	9.12E-03	promoter	super_enhancer	WASH2P
chr2	114415000	chr2	127065000	6.90E-03	promoter	super_enhancer	RP11-395L14.17
chr2	114415000	chr2	127085000	8.17E-03	promoter	super_enhancer	RP11-395L14.17
chr2	114415000	chr2	234245000	2.00E-03	promoter	super_enhancer	RP11-395L14.17
chr2	117575000	chr2	120915000	3.44E-02	promoter	super_enhancer	AC062016.3
chr2	118755000	chr2	238445000	2.62E-03	promoter	super_enhancer	RP11-98C1.2
chr2	119605000	chr2	242075000	3.43E-03	promoter	super_enhancer	EN1
chr2	120455000	chr2	120915000	2.19E-02	promoter	super_enhancer	AC069154.4
chr2	121935000	chr2	238425000	2.16E-03	promoter	super_enhancer	AC079988.3
chr2	122445000	chr2	142885000	8.16E-03	promoter	super_enhancer	RP11-416H1.2
chr2	122495000	chr2	230795000	2.88E-03	promoter	super_enhancer	NIFK
chr2	125885000	chr2	142875000	5.22E-03	promoter	super_enhancer	AC104842.1
chr2	127055000	chr2	131035000	1.92E-02	super_enhancer	promoter	AC068137.8
chr2	127055000	chr2	135805000	1.33E-02	super_enhancer	promoter	MAP3K19
chr2	127065000	chr2	132145000	1.59E-02	super_enhancer	promoter	MTND1P26

chr2	127065000	chr2	140425000	8.72E-03	super_enhancer	promoter	MRPS18BP2
chr2	127065000	chr2	168725000	3.69E-03	super_enhancer	promoter	B3GALT1
chr2	127075000	chr2	132295000	2.14E-02	super_enhancer	promoter	MED15P4
chr2	127075000	chr2	139035000	1.20E-02	super_enhancer	promoter	AC097523.1
chr2	127085000	chr2	136745000	1.48E-02	super_enhancer	promoter	DARS
chr2	127085000	chr2	161265000	5.34E-03	super_enhancer	promoter	MIR4785
chr2	127095000	chr2	176195000	3.29E-03	super_enhancer	promoter	AC096649.4
chr2	127095000	chr2	179315000	2.74E-03	super_enhancer	promoter	DFNB59
chr2	127095000	chr2	186665000	2.24E-03	super_enhancer	promoter	FSIP2-AS1
chr2	127095000	chr2	197125000	2.07E-03	super_enhancer	promoter	AC020571.3
chr2	127105000	chr2	131595000	3.53E-02	super_enhancer	promoter	AC133785.1
chr2	127105000	chr2	136635000	1.29E-02	super_enhancer	promoter	MCM6
chr2	127105000	chr2	142365000	1.15E-02	super_enhancer	promoter	AC093859.1
chr2	127105000	chr2	166325000	3.46E-03	super_enhancer	promoter	CSRNP3
chr2	127635000	chr2	132295000	2.39E-02	super_enhancer	promoter	MED15P4
chr2	128005000	chr2	238455000	2.81E-03	promoter	super_enhancer	AC110926.4
chr2	128175000	chr2	177245000	3.08E-03	promoter	super_enhancer	PROC
chr2	128285000	chr2	234965000	3.41E-03	promoter	super_enhancer	IWS1
chr2	128605000	chr2	231625000	3.28E-03	promoter	super_enhancer	AC006011.4
chr2	128825000	chr2	237455000	2.40E-03	promoter	super_enhancer	AC108059.1
chr2	131595000	chr2	233425000	2.65E-03	promoter	super_enhancer	AC133785.1
chr2	134885000	chr2	230785000	2.12E-03	promoter	super_enhancer	MIR3679
chr2	135675000	chr2	230795000	2.28E-03	promoter	super_enhancer	CCNT2
chr2	136415000	chr2	160205000	8.03E-03	promoter	super_enhancer	RNU6-512P

chr2	136595000	chr2	237465000	2.29E-03	promoter	super_enhancer	LCT
chr2	136635000	chr2	235015000	1.52E-03	promoter	super_enhancer	MCM6
chr2	136745000	chr2	160225000	1.03E-02	promoter	super_enhancer	DARS
chr2	140425000	chr2	238425000	2.19E-03	promoter	super_enhancer	MRPS18BP2
chr2	141655000	chr2	233405000	1.46E-03	promoter	super_enhancer	AC010740.1
chr2	142865000	chr2	158345000	8.84E-03	super_enhancer	promoter	CYTIP
chr2	142865000	chr2	196935000	2.66E-03	super_enhancer	promoter	DNAH7
chr2	142875000	chr2	174675000	6.32E-03	super_enhancer	promoter	RP11-796E10.1
chr2	142885000	chr2	147345000	1.78E-02	super_enhancer	promoter	PABPC1P2
chr2	144535000	chr2	231605000	1.58E-03	promoter	super_enhancer	CTD-2252P21.1
chr2	145275000	chr2	177255000	6.72E-03	promoter	super_enhancer	ZEB2-AS1
chr2	146095000	chr2	234965000	2.59E-03	promoter	super_enhancer	RPL6P5
chr2	148225000	chr2	200765000	4.05E-03	promoter	super_enhancer	AC013406.1
chr2	149895000	chr2	233425000	2.00E-03	promoter	super_enhancer	LYPD6B
chr2	149925000	chr2	177245000	4.14E-03	promoter	super_enhancer	TXNP5
chr2	150295000	chr2	181785000	4.98E-03	promoter	super_enhancer	AC007394.3
chr2	152225000	chr2	181775000	4.63E-03	promoter	super_enhancer	MIR4773-1
chr2	152265000	chr2	218295000	3.80E-03	promoter	super_enhancer	RIF1
chr2	158345000	chr2	218405000	3.21E-03	promoter	super_enhancer	CYTIP
chr2	160225000	chr2	218765000	4.49E-03	super_enhancer	promoter	MIR6809
chr2	160225000	chr2	219525000	4.54E-03	super_enhancer	promoter	ZNF142
chr2	160235000	chr2	166095000	1.45E-02	super_enhancer	promoter	SCN2A
chr2	160235000	chr2	177795000	9.05E-03	super_enhancer	promoter	RNU6-187P
chr2	160235000	chr2	203095000	4.67E-03	super_enhancer	promoter	AC079354.6

chr2	160235000	chr2	225365000	1.96E-03	super_enhancer	promoter	AC073052.1
chr2	162365000	chr2	237465000	2.00E-03	promoter	super_enhancer	AC009487.6
chr2	163015000	chr2	234975000	1.87E-03	promoter	super_enhancer	AC007750.5
chr2	168725000	chr2	177245000	1.21E-02	promoter	super_enhancer	B3GALT1
chr2	170335000	chr2	231605000	2.93E-03	promoter	super_enhancer	BBS5
chr2	171035000	chr2	242075000	3.05E-03	promoter	super_enhancer	MYO3B
chr2	171675000	chr2	231675000	5.13E-03	promoter	super_enhancer	AC007405.8
chr2	171675000	chr2	234255000	4.55E-03	promoter	super_enhancer	AC007405.8
chr2	172375000	chr2	218295000	5.94E-03	promoter	super_enhancer	AC007969.5
chr2	174675000	chr2	231655000	3.82E-03	promoter	super_enhancer	RP11-796E10.1
chr2	175105000	chr2	234985000	2.68E-03	promoter	super_enhancer	RN7SL65P
chr2	177135000	chr2	231665000	1.89E-03	promoter	super_enhancer	AC016739.3
chr2	177235000	chr2	179915000	2.81E-02	super_enhancer	promoter	CCDC141
chr2	177235000	chr2	181665000	1.78E-04	super_enhancer	promoter	RP11-739A13.1
chr2	177235000	chr2	186605000	1.13E-02	super_enhancer	promoter	AC007966.1
chr2	177235000	chr2	215275000	3.22E-03	super_enhancer	promoter	VWC2L
chr2	177245000	chr2	201945000	6.10E-03	super_enhancer	promoter	RNU6-1206P
chr2	177685000	chr2	237465000	3.15E-03	promoter	super_enhancer	AC092162.1
chr2	177715000	chr2	238435000	2.70E-03	promoter	super_enhancer	AC073636.1
chr2	178165000	chr2	234975000	2.87E-03	promoter	super_enhancer	RP11-337N6.2
chr2	179315000	chr2	200785000	7.69E-03	promoter	super_enhancer	DFNB59
chr2	179345000	chr2	233415000	3.29E-03	promoter	super_enhancer	PLEKHA3
chr2	179345000	chr2	242075000	3.07E-03	promoter	super_enhancer	PLEKHA3
chr2	179385000	chr2	218295000	4.60E-03	promoter	super_enhancer	TTN-AS1

chr2	179915000	chr2	181775000	4.66E-02	promoter	super_enhancer	CCDC141
chr2	179915000	chr2	237455000	1.68E-03	promoter	super_enhancer	CCDC141
chr2	181785000	chr2	202655000	1.07E-02	super_enhancer	promoter	CDK15
chr2	181785000	chr2	208735000	7.76E-03	super_enhancer	promoter	AC083900.1
chr2	181785000	chr2	215375000	3.77E-03	super_enhancer	promoter	VWC2L-IT1
chr2	181785000	chr2	240685000	2.80E-03	super_enhancer	promoter	AC093802.1
chr2	182545000	chr2	238435000	4.18E-03	promoter	super_enhancer	CERKL
chr2	183325000	chr2	200755000	7.00E-03	promoter	super_enhancer	AC010888.1
chr2	183325000	chr2	231645000	2.67E-03	promoter	super_enhancer	AC010888.1
chr2	189655000	chr2	200795000	1.00E-02	promoter	super_enhancer	DIRC1
chr2	189655000	chr2	230795000	3.84E-03	promoter	super_enhancer	DIRC1
chr2	189705000	chr2	200785000	1.52E-02	promoter	super_enhancer	AC079613.1
chr2	189705000	chr2	200795000	1.01E-04	promoter	super_enhancer	AC079613.1
chr2	191885000	chr2	231625000	5.84E-03	promoter	super_enhancer	AC067945.4
chr2	191885000	chr2	231675000	6.43E-03	promoter	super_enhancer	AC067945.4
chr2	196535000	chr2	200775000	3.54E-02	promoter	super_enhancer	RNU6-915P
chr2	197125000	chr2	234995000	5.68E-03	promoter	super_enhancer	AC020571.3
chr2	197675000	chr2	200795000	4.73E-02	promoter	super_enhancer	C2orf66
chr2	197895000	chr2	238445000	5.02E-03	promoter	super_enhancer	AC017035.2
chr2	198365000	chr2	231625000	7.94E-03	promoter	super_enhancer	HSPE1
chr2	200745000	chr2	207475000	1.93E-02	super_enhancer	promoter	AC010731.2
chr2	200745000	chr2	208125000	2.15E-02	super_enhancer	promoter	AC007879.6
chr2	200745000	chr2	215375000	9.58E-03	super_enhancer	promoter	VWC2L-IT1
chr2	200745000	chr2	240985000	5.71E-03	super_enhancer	promoter	OR6B3

chr2	200755000	chr2	208125000	1.75E-02	super_enhancer	promoter	AC007879.6
chr2	200755000	chr2	219065000	1.09E-02	super_enhancer	promoter	HMGB1P9
chr2	200755000	chr2	219135000	1.27E-02	super_enhancer	promoter	AAMP
chr2	200755000	chr2	232425000	6.63E-03	super_enhancer	promoter	AC104634.3
chr2	200765000	chr2	215275000	1.05E-02	super_enhancer	promoter	VWC2L
chr2	200765000	chr2	240685000	5.31E-03	super_enhancer	promoter	AC093802.1
chr2	200775000	chr2	219135000	1.48E-02	super_enhancer	promoter	AAMP
chr2	200775000	chr2	232395000	6.27E-03	super_enhancer	promoter	NMUR1
chr2	200775000	chr2	241925000	5.15E-03	super_enhancer	promoter	AC104809.2
chr2	200785000	chr2	204195000	4.27E-02	super_enhancer	promoter	RP11-363J17.1
chr2	200785000	chr2	204955000	2.73E-02	super_enhancer	promoter	AC009965.2
chr2	200785000	chr2	208575000	1.60E-02	super_enhancer	promoter	CCNYL1
chr2	200795000	chr2	202415000	5.14E-03	super_enhancer	promoter	AC007282.6
chr2	200795000	chr2	207085000	1.89E-02	super_enhancer	promoter	RN7SKP200
chr2	200795000	chr2	220175000	1.24E-02	super_enhancer	promoter	PTPRN
chr2	201675000	chr2	238455000	5.84E-03	promoter	super_enhancer	BZW1
chr2	201695000	chr2	218295000	1.20E-02	promoter	super_enhancer	RNU6-31P
chr2	201695000	chr2	230795000	7.48E-03	promoter	super_enhancer	RNU6-31P
chr2	201695000	chr2	235035000	6.30E-03	promoter	super_enhancer	RNU6-31P
chr2	201755000	chr2	234995000	8.06E-03	promoter	super_enhancer	PPIL3
chr2	201935000	chr2	234965000	7.95E-03	promoter	super_enhancer	NDUFB3
chr2	201945000	chr2	231605000	7.12E-03	promoter	super_enhancer	RNU6-1206P
chr2	201965000	chr2	231645000	7.50E-03	promoter	super_enhancer	RP11-13J8.1
chr2	201965000	chr2	238425000	7.20E-03	promoter	super_enhancer	RP11-13J8.1

chr2	202005000	chr2	234995000	7.64E-03	promoter	super_enhancer	IMPDH1P10
chr2	202485000	chr2	237455000	4.22E-03	promoter	super_enhancer	ALS2CR11
chr2	202705000	chr2	242065000	6.14E-03	promoter	super_enhancer	AC007242.1
chr2	203045000	chr2	218275000	1.21E-02	promoter	super_enhancer	AC079354.5
chr2	203045000	chr2	230775000	6.21E-03	promoter	super_enhancer	AC079354.5
chr2	203045000	chr2	231665000	5.17E-03	promoter	super_enhancer	AC079354.5
chr2	203155000	chr2	231625000	7.55E-03	promoter	super_enhancer	SNORD11B
chr2	203385000	chr2	231675000	1.11E-02	promoter	super_enhancer	AC073410.1
chr2	203485000	chr2	233415000	7.35E-03	promoter	super_enhancer	AC009960.3
chr2	204955000	chr2	218395000	9.37E-03	promoter	super_enhancer	AC009965.2
chr2	204955000	chr2	242065000	5.39E-03	promoter	super_enhancer	AC009965.2
chr2	207125000	chr2	234985000	7.42E-03	promoter	super_enhancer	HMGN1P6
chr2	207125000	chr2	234995000	9.67E-03	promoter	super_enhancer	HMGN1P6
chr2	208125000	chr2	231615000	9.43E-03	promoter	super_enhancer	AC007879.6
chr2	208395000	chr2	230795000	9.20E-03	promoter	super_enhancer	AC007879.5
chr2	208395000	chr2	231655000	8.02E-03	promoter	super_enhancer	AC007879.5
chr2	208575000	chr2	231635000	7.22E-03	promoter	super_enhancer	CCNYL1
chr2	208995000	chr2	238455000	3.04E-05	promoter	super_enhancer	CRYGC
chr2	209135000	chr2	218285000	1.63E-02	promoter	super_enhancer	MYL6BP1
chr2	209135000	chr2	235025000	6.43E-03	promoter	super_enhancer	MYL6BP1
chr2	210165000	chr2	218405000	1.32E-02	promoter	super_enhancer	AC016743.1
chr2	212165000	chr2	231615000	8.07E-03	promoter	super_enhancer	AC013404.1
chr2	213775000	chr2	235025000	4.68E-03	promoter	super_enhancer	AC108066.1
chr2	215675000	chr2	237465000	8.70E-03	promoter	super_enhancer	BARD1

chr2	216745000	chr2	230785000	1.31E-02	promoter	super_enhancer	AC122136.1
chr2	217075000	chr2	218275000	2.58E-02	promoter	super_enhancer	RP11-566E18.1
chr2	217075000	chr2	218285000	2.71E-02	promoter	super_enhancer	RP11-566E18.1
chr2	217075000	chr2	240425000	1.21E-02	promoter	super_enhancer	RP11-566E18.1
chr2	218295000	chr2	240965000	7.83E-03	super_enhancer	promoter	NDUFA10
chr2	218395000	chr2	236945000	1.10E-02	super_enhancer	promoter	RNU7-127P
chr2	218405000	chr2	223165000	3.92E-02	super_enhancer	promoter	PAX3
chr2	218405000	chr2	240985000	9.70E-03	super_enhancer	promoter	OR6B3
chr2	218765000	chr2	230795000	1.86E-02	promoter	super_enhancer	MIR6809
chr2	218765000	chr2	234255000	1.81E-02	promoter	super_enhancer	MIR6809
chr2	218765000	chr2	234955000	1.47E-02	promoter	super_enhancer	MIR6809
chr2	219135000	chr2	230785000	1.79E-02	promoter	super_enhancer	AAMP
chr2	219145000	chr2	240425000	1.61E-02	promoter	super_enhancer	MIR6513
chr2	219525000	chr2	233415000	1.90E-02	promoter	super_enhancer	ZNF142
chr2	219525000	chr2	234245000	2.09E-02	promoter	super_enhancer	ZNF142
chr2	219575000	chr2	233405000	1.81E-02	promoter	super_enhancer	TTLL4
chr2	219745000	chr2	231615000	1.71E-02	promoter	super_enhancer	WNT10A
chr2	219745000	chr2	234245000	1.79E-02	promoter	super_enhancer	WNT10A
chr2	219795000	chr2	231615000	1.49E-02	promoter	super_enhancer	LINC01494
chr2	219795000	chr2	233405000	1.40E-02	promoter	super_enhancer	LINC01494
chr2	219795000	chr2	234245000	1.57E-02	promoter	super_enhancer	LINC01494
chr2	219795000	chr2	237465000	1.11E-02	promoter	super_enhancer	LINC01494
chr2	219795000	chr2	242065000	1.07E-02	promoter	super_enhancer	LINC01494
chr2	219935000	chr2	242065000	1.37E-02	promoter	super_enhancer	RP11-33O4.1

chr2	220175000	chr2	231675000	2.45E-02	promoter	super_enhancer	PTPRN
chr2	220175000	chr2	239265000	1.37E-02	promoter	super_enhancer	PTPRN
chr2	220175000	chr2	239275000	1.41E-02	promoter	super_enhancer	PTPRN
chr2	220175000	chr2	240435000	1.01E-02	promoter	super_enhancer	PTPRN
chr2	220225000	chr2	233415000	1.92E-02	promoter	super_enhancer	AC053503.2
chr2	220265000	chr2	234995000	1.82E-02	promoter	super_enhancer	DNPEP
chr2	220415000	chr2	231605000	1.49E-02	promoter	super_enhancer	MIR3132
chr2	221315000	chr2	231605000	1.44E-02	promoter	super_enhancer	AC067956.1
chr2	221985000	chr2	240565000	6.86E-03	promoter	super_enhancer	AC093160.1
chr2	223165000	chr2	233405000	2.07E-02	promoter	super_enhancer	PAX3
chr2	223165000	chr2	233425000	1.84E-02	promoter	super_enhancer	PAX3
chr2	223565000	chr2	231665000	1.05E-02	promoter	super_enhancer	AC016712.1
chr2	223565000	chr2	234245000	1.60E-02	promoter	super_enhancer	AC016712.1
chr2	223725000	chr2	237475000	1.24E-02	promoter	super_enhancer	ACSL3
chr2	223725000	chr2	240425000	1.85E-02	promoter	super_enhancer	ACSL3
chr2	223785000	chr2	240435000	5.94E-03	promoter	super_enhancer	AC097461.4
chr2	224905000	chr2	230785000	1.78E-02	promoter	super_enhancer	SERPINE2
chr2	225365000	chr2	237475000	8.03E-03	promoter	super_enhancer	AC073052.1
chr2	225875000	chr2	231615000	2.10E-02	promoter	super_enhancer	MIR4439
chr2	226265000	chr2	234985000	9.19E-03	promoter	super_enhancer	NYAP2
chr2	226265000	chr2	237465000	9.82E-03	promoter	super_enhancer	NYAP2
chr2	227665000	chr2	234245000	3.43E-02	promoter	super_enhancer	IRS1
chr2	230775000	chr2	237075000	2.22E-02	super_enhancer	promoter	AC079135.1
chr2	230795000	chr2	239645000	1.36E-02	super_enhancer	promoter	AC113618.2

chr2	230795000	chr2	241545000	1.85E-02	super_enhancer	promoter	GPR35
chr2	230935000	chr2	231605000	1.79E-02	promoter	super_enhancer	SLC16A14
chr2	230935000	chr2	233415000	5.15E-03	promoter	super_enhancer	SLC16A14
chr2	230985000	chr2	231615000	1.75E-02	promoter	super_enhancer	AC009950.2
chr2	230985000	chr2	231625000	1.96E-02	promoter	super_enhancer	AC009950.2
chr2	231565000	chr2	231635000	1.08E-02	promoter	super_enhancer	AC098823.3
chr2	231565000	chr2	231655000	5.22E-03	promoter	super_enhancer	AC098823.3
chr2	231565000	chr2	242065000	2.10E-02	promoter	super_enhancer	AC098823.3
chr2	231605000	chr2	242185000	1.78E-02	super_enhancer	promoter	AC104841.1
chr2	231625000	chr2	241505000	2.01E-02	super_enhancer	promoter	RNPEPL1
chr2	231635000	chr2	233285000	1.64E-02	super_enhancer	promoter	ECEL1P1
chr2	231655000	chr2	238475000	2.81E-02	super_enhancer	promoter	PRLH
chr2	231665000	chr2	239335000	1.25E-02	super_enhancer	promoter	ASB1
chr2	231665000	chr2	240015000	1.09E-02	super_enhancer	promoter	AC017028.12
chr2	231665000	chr2	242255000	1.08E-02	super_enhancer	promoter	2-Sep
chr2	231675000	chr2	241505000	2.22E-02	super_enhancer	promoter	RNPEPL1
chr2	231675000	chr2	242715000	1.79E-04	super_enhancer	promoter	GAL3ST2
chr2	232355000	chr2	234965000	2.99E-03	promoter	super_enhancer	AC017104.1
chr2	232395000	chr2	240425000	2.11E-02	promoter	super_enhancer	NMUR1
chr2	232645000	chr2	233415000	2.50E-02	promoter	super_enhancer	COPS7B
chr2	232645000	chr2	238425000	3.61E-02	promoter	super_enhancer	COPS7B
chr2	232675000	chr2	234245000	2.25E-02	promoter	super_enhancer	RP11-690I21.2
chr2	232675000	chr2	239265000	2.48E-02	promoter	super_enhancer	RP11-690I21.2
chr2	232825000	chr2	238445000	3.27E-02	promoter	super_enhancer	DIS3L2

chr2	233405000	chr2	240085000	2.08E-02	super_enhancer	promoter	AC017028.10
chr2	233415000	chr2	234665000	2.48E-02	super_enhancer	promoter	AC114812.5
chr2	233415000	chr2	237925000	3.94E-02	super_enhancer	promoter	RNU6-1051P
chr2	233515000	chr2	238425000	3.41E-02	promoter	super_enhancer	RN7SL359P
chr2	233595000	chr2	234965000	2.04E-02	promoter	super_enhancer	EEF1B2P7
chr2	233595000	chr2	239275000	2.41E-02	promoter	super_enhancer	EEF1B2P7
chr2	233925000	chr2	238445000	4.47E-02	promoter	super_enhancer	INPP5D
chr2	234215000	chr2	239265000	3.68E-02	promoter	super_enhancer	SAG
chr2	234245000	chr2	240085000	2.78E-02	super_enhancer	promoter	AC017028.10
chr2	234255000	chr2	238875000	4.26E-02	super_enhancer	promoter	UBE2F
chr2	234255000	chr2	239065000	9.31E-04	super_enhancer	promoter	KLHL30-AS1
chr2	234255000	chr2	240985000	2.53E-02	super_enhancer	promoter	OR6B3
chr2	234255000	chr2	241625000	1.73E-02	super_enhancer	promoter	AC011298.2
chr2	234475000	chr2	237465000	4.48E-02	promoter	super_enhancer	USP40
chr2	234655000	chr2	238435000	3.27E-02	promoter	super_enhancer	UGT1A2P
chr2	234665000	chr2	238425000	4.32E-02	promoter	super_enhancer	AC114812.5
chr2	234665000	chr2	240435000	1.89E-02	promoter	super_enhancer	AC114812.5
chr2	234865000	chr2	240565000	2.63E-02	promoter	super_enhancer	AC005538.5
chr2	234965000	chr2	241085000	3.27E-02	super_enhancer	promoter	OTOS
chr2	234975000	chr2	238875000	4.18E-02	super_enhancer	promoter	UBE2F
chr2	235005000	chr2	235655000	1.81E-02	super_enhancer	promoter	RP13-977E11.1
chr2	235005000	chr2	242675000	1.92E-02	super_enhancer	promoter	AC114730.8
chr2	235015000	chr2	239335000	2.43E-02	super_enhancer	promoter	ASB1
chr2	235025000	chr2	242695000	1.73E-02	super_enhancer	promoter	AC114730.7

chr2	235045000	chr2	235365000	2.44E-02	super_enhancer	promoter	HSPE1P9
chr2	235045000	chr2	239065000	4.71E-02	super_enhancer	promoter	KLHL30-AS1
chr2	237445000	chr2	237925000	9.35E-04	super_enhancer	promoter	RNU6-1051P
chr2	237445000	chr2	237965000	2.52E-02	super_enhancer	promoter	AC105760.3
chr2	237455000	chr2	237925000	4.94E-04	super_enhancer	promoter	RNU6-1051P
chr2	237455000	chr2	241545000	3.74E-02	super_enhancer	promoter	GPR35
chr2	237465000	chr2	237925000	1.21E-03	super_enhancer	promoter	RNU6-1051P
chr2	237475000	chr2	237925000	2.01E-02	super_enhancer	promoter	RNU6-1051P
chr2	237475000	chr2	237965000	1.54E-03	super_enhancer	promoter	AC105760.3
chr2	238165000	chr2	238435000	5.48E-03	promoter	super_enhancer	AC112715.2
chr2	238435000	chr2	238535000	2.81E-02	super_enhancer	promoter	LRRFIP1
chr2	238445000	chr2	240015000	6.78E-04	super_enhancer	promoter	AC017028.12
chr2	238445000	chr2	242295000	4.67E-02	super_enhancer	promoter	FARP2
chr2	239275000	chr2	239335000	3.46E-02	super_enhancer	promoter	ASB1
chr2	239465000	chr2	240565000	2.31E-02	promoter	super_enhancer	LINC01107
chr2	240085000	chr2	240425000	7.69E-05	promoter	super_enhancer	AC017028.10
chr2	240085000	chr2	240435000	2.08E-02	promoter	super_enhancer	AC017028.10
chr2	240575000	chr2	240685000	1.36E-02	super_enhancer	promoter	AC093802.1
chr2	240575000	chr2	241085000	4.60E-02	super_enhancer	promoter	OTOS
chr2	240965000	chr2	242065000	2.97E-02	promoter	super_enhancer	NDUFA10
chr2	241195000	chr2	242075000	1.65E-02	promoter	super_enhancer	AC124861.1
chr2	242065000	chr2	242295000	1.15E-02	super_enhancer	promoter	FARP2
chr3	65000	chr3	5015000	2.39E-02	promoter	super_enhancer	AY269186.1
chr3	65000	chr3	13855000	1.13E-02	promoter	super_enhancer	AY269186.1

chr3	325000	chr3	16115000	1.23E-02	promoter	super_enhancer	RNU6-1194P
chr3	325000	chr3	126215000	3.61E-03	promoter	super_enhancer	RNU6-1194P
chr3	3195000	chr3	14405000	1.77E-02	promoter	super_enhancer	RP11-97C16.1
chr3	3195000	chr3	49135000	4.33E-03	promoter	super_enhancer	RP11-97C16.1
chr3	3195000	chr3	49475000	5.80E-03	promoter	super_enhancer	RP11-97C16.1
chr3	3195000	chr3	141955000	2.98E-03	promoter	super_enhancer	RP11-97C16.1
chr3	4345000	chr3	14415000	3.17E-02	promoter	super_enhancer	SETMAR
chr3	4345000	chr3	45115000	9.47E-03	promoter	super_enhancer	SETMAR
chr3	4345000	chr3	46935000	1.55E-02	promoter	super_enhancer	SETMAR
chr3	4725000	chr3	93695000	1.90E-03	super_enhancer	promoter	RNU6-511P
chr3	4725000	chr3	196725000	4.45E-03	super_enhancer	promoter	MFI2-AS1
chr3	4735000	chr3	19985000	1.55E-02	super_enhancer	promoter	HSPA8P18
chr3	4745000	chr3	9005000	4.80E-02	super_enhancer	promoter	RAD18
chr3	4745000	chr3	10325000	4.16E-02	super_enhancer	promoter	LINC00852
chr3	4745000	chr3	13095000	2.65E-02	super_enhancer	promoter	RP11-715I4.1
chr3	4755000	chr3	6005000	3.05E-02	super_enhancer	promoter	AC027119.1
chr3	4755000	chr3	25215000	8.94E-03	super_enhancer	promoter	RARB
chr3	4755000	chr3	44625000	7.77E-03	super_enhancer	promoter	ZNF197
chr3	4755000	chr3	48265000	7.77E-03	super_enhancer	promoter	CAMP
chr3	5015000	chr3	8655000	3.89E-02	super_enhancer	promoter	LMCD1-AS1
chr3	5015000	chr3	32545000	1.12E-02	super_enhancer	promoter	CMTM6
chr3	5025000	chr3	13115000	3.70E-02	super_enhancer	promoter	IQSEC1
chr3	5025000	chr3	14345000	3.68E-02	super_enhancer	promoter	RP11-536I6.2
chr3	5025000	chr3	27765000	1.30E-02	super_enhancer	promoter	EOMES

chr3	5025000	chr3	42815000	9.17E-03	super_enhancer	promoter	CCDC13
chr3	5295000	chr3	11285000	3.57E-02	promoter	super_enhancer	RN7SL553P
chr3	5295000	chr3	45135000	9.18E-03	promoter	super_enhancer	RN7SL553P
chr3	6005000	chr3	134375000	3.79E-03	promoter	super_enhancer	AC027119.1
chr3	8635000	chr3	14395000	3.51E-02	promoter	super_enhancer	AC034187.2
chr3	8635000	chr3	15305000	3.06E-02	promoter	super_enhancer	AC034187.2
chr3	8635000	chr3	45135000	7.92E-03	promoter	super_enhancer	AC034187.2
chr3	8655000	chr3	16065000	1.29E-02	promoter	super_enhancer	LMCD1-AS1
chr3	9005000	chr3	14405000	4.96E-02	promoter	super_enhancer	RAD18
chr3	9005000	chr3	129595000	3.16E-03	promoter	super_enhancer	RAD18
chr3	9055000	chr3	46985000	1.32E-02	promoter	super_enhancer	SRGAP3-AS1
chr3	9055000	chr3	50265000	1.22E-02	promoter	super_enhancer	SRGAP3-AS1
chr3	9405000	chr3	15655000	3.93E-02	promoter	super_enhancer	SRGAP3
chr3	9405000	chr3	49495000	8.69E-03	promoter	super_enhancer	SRGAP3
chr3	9405000	chr3	197495000	3.06E-03	promoter	super_enhancer	SRGAP3
chr3	9595000	chr3	14435000	2.97E-03	promoter	super_enhancer	LHFPL4
chr3	9595000	chr3	15275000	3.98E-02	promoter	super_enhancer	LHFPL4
chr3	9595000	chr3	15285000	4.21E-02	promoter	super_enhancer	LHFPL4
chr3	9895000	chr3	23175000	2.50E-02	super_enhancer	promoter	RPL24P7
chr3	9895000	chr3	39685000	1.62E-02	super_enhancer	promoter	NFU1P1
chr3	9895000	chr3	46855000	1.47E-02	super_enhancer	promoter	PRSS50
chr3	9895000	chr3	48725000	1.61E-02	super_enhancer	promoter	NCKIPSD
chr3	9895000	chr3	48885000	1.30E-02	super_enhancer	promoter	PRKAR2A
chr3	9895000	chr3	49505000	1.32E-02	super_enhancer	promoter	RNA5SP130

chr3	9895000	chr3	182705000	5.08E-03	super_enhancer	promoter	DCUN1D1
chr3	9975000	chr3	42075000	1.82E-02	promoter	super_enhancer	CRELD1
chr3	9975000	chr3	45175000	1.24E-02	promoter	super_enhancer	CRELD1
chr3	9975000	chr3	45215000	1.27E-02	promoter	super_enhancer	CRELD1
chr3	9975000	chr3	195885000	4.50E-03	promoter	super_enhancer	CRELD1
chr3	10175000	chr3	15915000	4.08E-02	super_enhancer	promoter	MIR563
chr3	10175000	chr3	42005000	1.22E-02	super_enhancer	promoter	ULK4
chr3	10175000	chr3	51575000	7.33E-03	super_enhancer	promoter	RAD54L2
chr3	10175000	chr3	51905000	7.44E-03	super_enhancer	promoter	RN7SL504P
chr3	10175000	chr3	109215000	2.02E-03	super_enhancer	promoter	AC092905.1
chr3	10185000	chr3	10325000	4.92E-02	super_enhancer	promoter	LINC00852
chr3	10185000	chr3	11995000	2.53E-02	super_enhancer	promoter	RN7SL147P
chr3	10185000	chr3	15475000	4.64E-02	super_enhancer	promoter	RNU6-1024P
chr3	10185000	chr3	50195000	1.14E-02	super_enhancer	promoter	SEMA3F-AS1
chr3	10195000	chr3	33535000	1.23E-02	super_enhancer	promoter	RNA5SP128
chr3	10195000	chr3	37035000	9.83E-03	super_enhancer	promoter	EPM2AIP1
chr3	10195000	chr3	46185000	8.74E-03	super_enhancer	promoter	FLT1P1
chr3	10195000	chr3	48505000	9.58E-03	super_enhancer	promoter	TREX1
chr3	10195000	chr3	103775000	1.89E-03	super_enhancer	promoter	AC016970.1
chr3	10195000	chr3	158055000	2.87E-03	super_enhancer	promoter	RP11-113A11.3
chr3	10195000	chr3	196595000	3.63E-03	super_enhancer	promoter	SENP5
chr3	10205000	chr3	18055000	2.98E-02	super_enhancer	promoter	RP11-320G10.1
chr3	10205000	chr3	196475000	3.52E-03	super_enhancer	promoter	RNU4-89P
chr3	10215000	chr3	15175000	3.76E-02	super_enhancer	promoter	AC090954.5

chr3	10215000	chr3	15475000	4.25E-02	super_enhancer	promoter	RNU6-1024P
chr3	10215000	chr3	22855000	1.68E-02	super_enhancer	promoter	RP11-1037D22.1
chr3	10215000	chr3	127295000	4.88E-03	super_enhancer	promoter	MIR6825
chr3	10225000	chr3	15915000	4.34E-02	super_enhancer	promoter	MIR563
chr3	10225000	chr3	46065000	1.10E-02	super_enhancer	promoter	NRBF2P2
chr3	10225000	chr3	123435000	4.31E-03	super_enhancer	promoter	RP11-9N20.3
chr3	10225000	chr3	148415000	2.95E-03	super_enhancer	promoter	RPL38P1
chr3	10225000	chr3	183165000	3.35E-03	super_enhancer	promoter	LINC00888
chr3	10325000	chr3	13835000	5.75E-03	promoter	super_enhancer	LINC00852
chr3	10325000	chr3	15285000	4.57E-02	promoter	super_enhancer	LINC00852
chr3	10325000	chr3	30605000	1.87E-02	promoter	super_enhancer	LINC00852
chr3	10325000	chr3	45175000	1.06E-02	promoter	super_enhancer	LINC00852
chr3	10325000	chr3	46985000	1.33E-02	promoter	super_enhancer	LINC00852
chr3	10335000	chr3	14465000	1.79E-03	promoter	super_enhancer	GHRL
chr3	10335000	chr3	14475000	4.72E-02	promoter	super_enhancer	GHRL
chr3	10335000	chr3	15275000	3.24E-02	promoter	super_enhancer	GHRL
chr3	11275000	chr3	27555000	1.82E-02	super_enhancer	promoter	RNU1-96P
chr3	11275000	chr3	44915000	9.18E-03	super_enhancer	promoter	TGM4
chr3	11275000	chr3	49405000	9.61E-03	super_enhancer	promoter	RHOA-IT1
chr3	11275000	chr3	65995000	3.12E-03	super_enhancer	promoter	RP11-320P11.1
chr3	11285000	chr3	13095000	2.14E-02	super_enhancer	promoter	RP11-715I4.1
chr3	11285000	chr3	16215000	4.85E-02	super_enhancer	promoter	GALNT15
chr3	11285000	chr3	34585000	1.02E-02	super_enhancer	promoter	AC018359.3
chr3	11285000	chr3	44915000	9.86E-03	super_enhancer	promoter	TGM4

chr3	11285000	chr3	46185000	8.95E-03	super_enhancer	promoter	FLT1P1
chr3	11285000	chr3	47025000	1.18E-02	super_enhancer	promoter	CCDC12
chr3	11285000	chr3	49205000	1.17E-02	super_enhancer	promoter	CCDC71
chr3	11285000	chr3	196125000	3.84E-03	super_enhancer	promoter	RN7SL434P
chr3	11295000	chr3	37015000	1.55E-02	super_enhancer	promoter	RP11-129K12.1
chr3	11295000	chr3	39255000	1.04E-02	super_enhancer	promoter	DSTNP4
chr3	11295000	chr3	41235000	7.39E-03	super_enhancer	promoter	CTNNB1
chr3	11295000	chr3	52005000	9.43E-03	super_enhancer	promoter	ABHD14A
chr3	11295000	chr3	122995000	3.92E-03	super_enhancer	promoter	RP11-797D24.3
chr3	11295000	chr3	143575000	3.62E-03	super_enhancer	promoter	RP11-8J23.1
chr3	11305000	chr3	49315000	1.24E-02	super_enhancer	promoter	C3orf62
chr3	11305000	chr3	49405000	1.20E-02	super_enhancer	promoter	RHOA-IT1
chr3	11305000	chr3	57205000	6.53E-03	super_enhancer	promoter	IL17RD
chr3	11305000	chr3	105085000	2.51E-03	super_enhancer	promoter	ALCAM
chr3	11305000	chr3	109115000	3.58E-03	super_enhancer	promoter	RP11-702L6.1
chr3	11995000	chr3	42065000	1.57E-02	promoter	super_enhancer	RN7SL147P
chr3	11995000	chr3	126915000	4.34E-03	promoter	super_enhancer	RN7SL147P
chr3	11995000	chr3	139315000	3.20E-03	promoter	super_enhancer	RN7SL147P
chr3	12045000	chr3	13845000	1.29E-02	promoter	super_enhancer	SYN2
chr3	12045000	chr3	194135000	2.04E-03	promoter	super_enhancer	SYN2
chr3	12185000	chr3	20215000	2.23E-02	super_enhancer	promoter	SGOL1-AS1
chr3	12195000	chr3	36865000	1.41E-02	super_enhancer	promoter	RP11-640L9.1
chr3	12195000	chr3	50195000	1.00E-02	super_enhancer	promoter	SEMA3F-AS1
chr3	12195000	chr3	138555000	4.24E-03	super_enhancer	promoter	PIK3CB

chr3	12525000	chr3	45185000	7.56E-03	promoter	super_enhancer	TSEN2
chr3	12525000	chr3	128485000	2.86E-03	promoter	super_enhancer	TSEN2
chr3	12715000	chr3	38175000	1.19E-02	promoter	super_enhancer	CRIP1P1
chr3	12715000	chr3	45145000	1.17E-02	promoter	super_enhancer	CRIP1P1
chr3	12715000	chr3	50255000	1.22E-02	promoter	super_enhancer	CRIP1P1
chr3	12755000	chr3	33455000	1.61E-02	super_enhancer	promoter	RNU7-110P
chr3	12755000	chr3	120525000	3.40E-03	super_enhancer	promoter	NAP1L1P3
chr3	12755000	chr3	149695000	3.79E-03	super_enhancer	promoter	RP11-651P23.5
chr3	12755000	chr3	182225000	3.22E-03	super_enhancer	promoter	RP11-139K4.2
chr3	12755000	chr3	186285000	3.68E-03	super_enhancer	promoter	DNAJB11
chr3	12755000	chr3	186435000	5.37E-06	super_enhancer	promoter	KNG1
chr3	12765000	chr3	14585000	2.67E-02	super_enhancer	promoter	GRIP2
chr3	12765000	chr3	47705000	8.59E-03	super_enhancer	promoter	RP11-395P16.1
chr3	12765000	chr3	48955000	1.02E-02	super_enhancer	promoter	ARIH2
chr3	12765000	chr3	195635000	2.52E-03	super_enhancer	promoter	TNK2-AS1
chr3	12875000	chr3	30625000	1.74E-02	promoter	super_enhancer	RP11-767C1.2
chr3	12995000	chr3	30565000	1.37E-02	super_enhancer	promoter	AC116035.1
chr3	13005000	chr3	43355000	1.10E-02	super_enhancer	promoter	RN7SL517P
chr3	13005000	chr3	44625000	1.46E-02	super_enhancer	promoter	ZNF197
chr3	13005000	chr3	47825000	1.44E-02	super_enhancer	promoter	SMARCC1
chr3	13005000	chr3	58365000	6.12E-03	super_enhancer	promoter	RP11-802O23.4
chr3	13005000	chr3	96495000	1.89E-03	super_enhancer	promoter	CDV3P1
chr3	13005000	chr3	125095000	4.76E-03	super_enhancer	promoter	ZNF148
chr3	13005000	chr3	148415000	3.80E-03	super_enhancer	promoter	RPL38P1

chr3	13015000	chr3	24535000	2.37E-02	super_enhancer	promoter	THRB-AS1
chr3	13015000	chr3	35785000	1.45E-02	super_enhancer	promoter	MIR128-2
chr3	13015000	chr3	37225000	1.42E-02	super_enhancer	promoter	LRRFIP2
chr3	13015000	chr3	44915000	1.13E-02	super_enhancer	promoter	TGM4
chr3	13015000	chr3	46735000	1.37E-02	super_enhancer	promoter	ALS2CL
chr3	13015000	chr3	146135000	2.98E-03	super_enhancer	promoter	RP11-758I14.3
chr3	13015000	chr3	149105000	4.02E-03	super_enhancer	promoter	RP11-278L15.2
chr3	13025000	chr3	13095000	4.54E-02	super_enhancer	promoter	RP11-715I4.1
chr3	13025000	chr3	46735000	1.43E-02	super_enhancer	promoter	ALS2CL
chr3	13025000	chr3	47025000	1.40E-02	super_enhancer	promoter	CCDC12
chr3	13025000	chr3	48955000	1.24E-02	super_enhancer	promoter	ARIH2
chr3	13025000	chr3	50385000	1.27E-02	super_enhancer	promoter	ZMYND10
chr3	13025000	chr3	196065000	3.56E-03	super_enhancer	promoter	TM4SF19-TCTEX1D2
chr3	13095000	chr3	13825000	1.29E-02	promoter	super_enhancer	RP11-715I4.1
chr3	13095000	chr3	14485000	4.70E-02	promoter	super_enhancer	RP11-715I4.1
chr3	13095000	chr3	49485000	1.23E-02	promoter	super_enhancer	RP11-715I4.1
chr3	13095000	chr3	50175000	1.19E-02	promoter	super_enhancer	RP11-715I4.1
chr3	13115000	chr3	13415000	1.87E-04	promoter	super_enhancer	IQSEC1
chr3	13115000	chr3	42075000	1.67E-02	promoter	super_enhancer	IQSEC1
chr3	13115000	chr3	128485000	4.80E-03	promoter	super_enhancer	IQSEC1
chr3	13415000	chr3	42005000	1.55E-02	super_enhancer	promoter	ULK4
chr3	13415000	chr3	50125000	9.27E-03	super_enhancer	promoter	RBM5
chr3	13415000	chr3	58315000	5.12E-03	super_enhancer	promoter	RP11-80H18.4
chr3	13415000	chr3	138665000	4.47E-03	super_enhancer	promoter	FOXL2NB

chr3	13415000	chr3	149695000	3.60E-03	super_enhancer	promoter	RP11-651P23.5
chr3	13415000	chr3	182225000	3.05E-03	super_enhancer	promoter	RP11-139K4.2
chr3	13415000	chr3	183815000	4.38E-03	super_enhancer	promoter	HTR3E
chr3	13425000	chr3	33595000	1.74E-02	super_enhancer	promoter	COX6CP10
chr3	13425000	chr3	40495000	1.19E-02	super_enhancer	promoter	ENTPD3-AS1
chr3	13425000	chr3	46185000	1.06E-02	super_enhancer	promoter	FLT1P1
chr3	13425000	chr3	47205000	1.11E-02	super_enhancer	promoter	SETD2
chr3	13425000	chr3	49505000	1.06E-02	super_enhancer	promoter	RNA5SP130
chr3	13505000	chr3	20055000	3.05E-02	super_enhancer	promoter	PP2D1
chr3	13505000	chr3	46735000	1.37E-02	super_enhancer	promoter	ALS2CL
chr3	13505000	chr3	196715000	3.85E-03	super_enhancer	promoter	AC011322.1
chr3	13535000	chr3	37175000	1.06E-02	super_enhancer	promoter	UBE2D3P2
chr3	13545000	chr3	14345000	1.81E-02	super_enhancer	promoter	RP11-536I6.2
chr3	13545000	chr3	34245000	1.19E-02	super_enhancer	promoter	AC123023.1
chr3	13545000	chr3	42005000	1.76E-02	super_enhancer	promoter	ULK4
chr3	13545000	chr3	49235000	1.36E-02	super_enhancer	promoter	CCDC36
chr3	13545000	chr3	109035000	2.70E-03	super_enhancer	promoter	DPPA2
chr3	13825000	chr3	14165000	7.23E-03	super_enhancer	promoter	TMEM43
chr3	13825000	chr3	19985000	4.20E-02	super_enhancer	promoter	HSPA8P18
chr3	13825000	chr3	42575000	1.64E-02	super_enhancer	promoter	VIPR1-AS1
chr3	13825000	chr3	48885000	1.09E-02	super_enhancer	promoter	PRKAR2A
chr3	13825000	chr3	50385000	1.33E-02	super_enhancer	promoter	ZMYND10
chr3	13825000	chr3	108015000	3.01E-03	super_enhancer	promoter	HHLA2
chr3	13825000	chr3	125465000	3.25E-03	super_enhancer	promoter	OR7E97P

chr3	13835000	chr3	25215000	1.95E-02	super_enhancer	promoter	RARB
chr3	13835000	chr3	37615000	1.84E-02	super_enhancer	promoter	RNU7-73P
chr3	13835000	chr3	48265000	1.46E-02	super_enhancer	promoter	CAMP
chr3	13835000	chr3	50375000	1.65E-02	super_enhancer	promoter	RASSF1-AS1
chr3	13845000	chr3	39235000	1.82E-02	super_enhancer	promoter	XIRP1
chr3	13845000	chr3	132105000	3.92E-03	super_enhancer	promoter	RP11-65E22.2
chr3	13845000	chr3	187825000	4.33E-03	super_enhancer	promoter	RP11-430L16.1
chr3	13855000	chr3	31225000	2.02E-02	super_enhancer	promoter	CNN2P6
chr3	13975000	chr3	14415000	4.91E-03	promoter	super_enhancer	FGD5P1
chr3	13975000	chr3	14465000	4.45E-02	promoter	super_enhancer	FGD5P1
chr3	14165000	chr3	52055000	9.46E-03	promoter	super_enhancer	TMEM43
chr3	14185000	chr3	14465000	1.38E-03	promoter	super_enhancer	AC093495.4
chr3	14185000	chr3	45185000	1.27E-02	promoter	super_enhancer	AC093495.4
chr3	14185000	chr3	50165000	1.34E-02	promoter	super_enhancer	AC093495.4
chr3	14185000	chr3	123585000	4.62E-03	promoter	super_enhancer	AC093495.4
chr3	14345000	chr3	30615000	1.88E-02	promoter	super_enhancer	RP11-536I6.2
chr3	14395000	chr3	44155000	1.39E-02	super_enhancer	promoter	MIR138-1
chr3	14395000	chr3	48725000	1.45E-02	super_enhancer	promoter	NCKIPSD
chr3	14395000	chr3	64005000	3.26E-03	super_enhancer	promoter	RP11-245J9.6
chr3	14395000	chr3	122785000	5.35E-03	super_enhancer	promoter	PDIA5
chr3	14405000	chr3	31225000	2.36E-02	super_enhancer	promoter	CNN2P6
chr3	14405000	chr3	38345000	1.44E-02	super_enhancer	promoter	RNU6-235P
chr3	14405000	chr3	42745000	1.76E-02	super_enhancer	promoter	HHATL
chr3	14405000	chr3	52725000	5.34E-03	super_enhancer	promoter	SNORD19B

chr3	14415000	chr3	23655000	1.98E-02	super_enhancer	promoter	RNU6-922P
chr3	14415000	chr3	27745000	1.52E-02	super_enhancer	promoter	AC098614.3
chr3	14415000	chr3	30565000	1.33E-02	super_enhancer	promoter	AC116035.1
chr3	14415000	chr3	52715000	3.63E-03	super_enhancer	promoter	GNL3
chr3	14425000	chr3	23175000	2.55E-02	super_enhancer	promoter	RPL24P7
chr3	14425000	chr3	27405000	2.18E-02	super_enhancer	promoter	AC099535.1
chr3	14425000	chr3	37225000	1.70E-02	super_enhancer	promoter	LRRFIP2
chr3	14425000	chr3	42575000	1.79E-02	super_enhancer	promoter	VIPR1-AS1
chr3	14425000	chr3	130745000	4.94E-03	super_enhancer	promoter	NEK11
chr3	14425000	chr3	146135000	3.38E-03	super_enhancer	promoter	RP11-758I14.3
chr3	14435000	chr3	26665000	3.02E-02	super_enhancer	promoter	AC099754.1
chr3	14435000	chr3	37285000	2.01E-02	super_enhancer	promoter	GOLGA4
chr3	14435000	chr3	39235000	2.14E-02	super_enhancer	promoter	XIRP1
chr3	14435000	chr3	51895000	8.79E-03	super_enhancer	promoter	IQCF2
chr3	14435000	chr3	52805000	7.42E-03	super_enhancer	promoter	NEK4
chr3	14435000	chr3	57605000	6.42E-03	super_enhancer	promoter	RNU6ATAC26P
chr3	14435000	chr3	128635000	5.33E-03	super_enhancer	promoter	RP11-723O4.7
chr3	14445000	chr3	19985000	4.43E-02	super_enhancer	promoter	HSPA8P18
chr3	14445000	chr3	20215000	3.01E-02	super_enhancer	promoter	SGOL1-AS1
chr3	14445000	chr3	119355000	4.58E-03	super_enhancer	promoter	RP11-190C22.1
chr3	14455000	chr3	194255000	3.23E-03	super_enhancer	promoter	RNU6-1101P
chr3	14465000	chr3	23655000	2.66E-02	super_enhancer	promoter	RNU6-922P
chr3	14465000	chr3	33595000	1.87E-02	super_enhancer	promoter	COX6CP10
chr3	14465000	chr3	49845000	1.18E-02	super_enhancer	promoter	MIR5193

chr3	14475000	chr3	27555000	2.07E-02	super_enhancer	promoter	RNU1-96P
chr3	14475000	chr3	27765000	1.64E-02	super_enhancer	promoter	EOMES
chr3	14475000	chr3	35785000	1.29E-02	super_enhancer	promoter	MIR128-2
chr3	14475000	chr3	52445000	5.18E-03	super_enhancer	promoter	BAP1
chr3	14485000	chr3	44835000	1.33E-02	super_enhancer	promoter	AC098649.1
chr3	14485000	chr3	44915000	1.11E-02	super_enhancer	promoter	TGM4
chr3	14485000	chr3	51575000	9.11E-03	super_enhancer	promoter	RAD54L2
chr3	14585000	chr3	16105000	4.68E-02	promoter	super_enhancer	GRIP2
chr3	14585000	chr3	30605000	2.52E-02	promoter	super_enhancer	GRIP2
chr3	14585000	chr3	30625000	2.35E-02	promoter	super_enhancer	GRIP2
chr3	14585000	chr3	45165000	1.28E-02	promoter	super_enhancer	GRIP2
chr3	15275000	chr3	23245000	2.04E-02	super_enhancer	promoter	UBE2E2-AS1
chr3	15275000	chr3	25795000	1.43E-02	super_enhancer	promoter	TAF9BP1
chr3	15275000	chr3	42895000	9.88E-03	super_enhancer	promoter	RP11-70C1.3
chr3	15275000	chr3	52005000	7.94E-03	super_enhancer	promoter	ABHD14A
chr3	15285000	chr3	25395000	1.27E-02	super_enhancer	promoter	RNA5SP126
chr3	15285000	chr3	33595000	1.38E-02	super_enhancer	promoter	COX6CP10
chr3	15285000	chr3	37225000	1.17E-02	super_enhancer	promoter	LRRFIP2
chr3	15285000	chr3	151195000	3.22E-03	super_enhancer	promoter	MRPL42P6
chr3	15295000	chr3	16355000	4.67E-02	super_enhancer	promoter	RP11-415F23.2
chr3	15295000	chr3	34245000	9.80E-03	super_enhancer	promoter	AC123023.1
chr3	15295000	chr3	47025000	1.24E-02	super_enhancer	promoter	CCDC12
chr3	15295000	chr3	148805000	3.51E-03	super_enhancer	promoter	HLTF
chr3	15305000	chr3	20055000	4.84E-02	super_enhancer	promoter	PP2D1

chr3	15305000	chr3	27555000	2.86E-02	super_enhancer	promoter	RNU1-96P
chr3	15305000	chr3	158055000	3.46E-03	super_enhancer	promoter	RP11-113A11.3
chr3	15635000	chr3	27745000	1.77E-02	super_enhancer	promoter	AC098614.3
chr3	15635000	chr3	37185000	1.45E-02	super_enhancer	promoter	UBE2FP1
chr3	15635000	chr3	49315000	1.14E-02	super_enhancer	promoter	C3orf62
chr3	15635000	chr3	147795000	1.98E-03	super_enhancer	promoter	RP11-639B1.1
chr3	15645000	chr3	187665000	2.60E-03	super_enhancer	promoter	RP11-44H4.1
chr3	15655000	chr3	26665000	2.60E-02	super_enhancer	promoter	AC099754.1
chr3	16055000	chr3	33595000	1.79E-02	super_enhancer	promoter	COX6CP10
chr3	16055000	chr3	39425000	1.56E-02	super_enhancer	promoter	SLC25A38
chr3	16055000	chr3	40565000	1.36E-02	super_enhancer	promoter	ZNF621
chr3	16055000	chr3	63845000	3.69E-03	super_enhancer	promoter	THOC7-AS1
chr3	16055000	chr3	125775000	4.73E-03	super_enhancer	promoter	RP11-158I23.1
chr3	16065000	chr3	191955000	1.56E-03	super_enhancer	promoter	FGF12-AS1
chr3	16075000	chr3	33455000	1.71E-02	super_enhancer	promoter	RNU7-110P
chr3	16075000	chr3	41235000	8.83E-03	super_enhancer	promoter	CTNNB1
chr3	16075000	chr3	49205000	1.40E-02	super_enhancer	promoter	CCDC71
chr3	16085000	chr3	16215000	2.14E-03	super_enhancer	promoter	GALNT15
chr3	16085000	chr3	16975000	4.79E-02	super_enhancer	promoter	MIR3714
chr3	16085000	chr3	25215000	1.47E-02	super_enhancer	promoter	RARB
chr3	16085000	chr3	37035000	1.01E-02	super_enhancer	promoter	EPM2AIP1
chr3	16085000	chr3	42305000	1.09E-02	super_enhancer	promoter	RP11-333B11.1
chr3	16085000	chr3	42815000	1.00E-02	super_enhancer	promoter	CCDC13
chr3	16095000	chr3	16215000	2.66E-02	super_enhancer	promoter	GALNT15

chr3	16095000	chr3	19985000	4.91E-02	super_enhancer	promoter	HSPA8P18
chr3	16095000	chr3	33075000	1.34E-02	super_enhancer	promoter	SEC13P1
chr3	16095000	chr3	43355000	7.75E-03	super_enhancer	promoter	RN7SL517P
chr3	16095000	chr3	49845000	8.65E-03	super_enhancer	promoter	MIR5193
chr3	16105000	chr3	16215000	2.44E-03	super_enhancer	promoter	GALNT15
chr3	16105000	chr3	32545000	1.73E-02	super_enhancer	promoter	CMTM6
chr3	16105000	chr3	38345000	1.29E-02	super_enhancer	promoter	RNU6-235P
chr3	16105000	chr3	52715000	4.28E-03	super_enhancer	promoter	GNL3
chr3	16115000	chr3	33075000	1.51E-02	super_enhancer	promoter	SEC13P1
chr3	16115000	chr3	46855000	1.04E-02	super_enhancer	promoter	PRSS50
chr3	16115000	chr3	50375000	1.27E-02	super_enhancer	promoter	RASSF1-AS1
chr3	16125000	chr3	46735000	1.04E-02	super_enhancer	promoter	ALS2CL
chr3	16125000	chr3	155525000	2.99E-03	super_enhancer	promoter	C3orf33
chr3	16135000	chr3	23655000	3.06E-02	super_enhancer	promoter	RNU6-922P
chr3	16135000	chr3	27745000	2.20E-02	super_enhancer	promoter	AC098614.3
chr3	16135000	chr3	28615000	2.43E-02	super_enhancer	promoter	LINC00693
chr3	16135000	chr3	46855000	1.32E-02	super_enhancer	promoter	PRSS50
chr3	16135000	chr3	49825000	1.32E-02	super_enhancer	promoter	IP6K1
chr3	16135000	chr3	52445000	6.76E-03	super_enhancer	promoter	BAP1
chr3	16145000	chr3	22415000	2.54E-02	super_enhancer	promoter	ZNF385D
chr3	16215000	chr3	38175000	1.31E-02	promoter	super_enhancer	GALNT15
chr3	16215000	chr3	50255000	1.32E-02	promoter	super_enhancer	GALNT15
chr3	16355000	chr3	45205000	6.23E-03	promoter	super_enhancer	RP11-415F23.2
chr3	17705000	chr3	42075000	1.36E-02	promoter	super_enhancer	RNU7-10P

chr3	17705000	chr3	45205000	6.43E-03	promoter	super_enhancer	RNU7-10P
chr3	17915000	chr3	52055000	7.94E-03	promoter	super_enhancer	AC104297.1
chr3	18055000	chr3	38175000	1.16E-02	promoter	super_enhancer	RP11-320G10.1
chr3	19985000	chr3	43195000	1.35E-02	promoter	super_enhancer	HSPA8P18
chr3	20055000	chr3	43165000	1.08E-02	promoter	super_enhancer	PP2D1
chr3	22415000	chr3	197515000	2.74E-03	promoter	super_enhancer	ZNF385D
chr3	22945000	chr3	30605000	2.90E-02	promoter	super_enhancer	RANP7
chr3	22945000	chr3	46945000	1.49E-02	promoter	super_enhancer	RANP7
chr3	23175000	chr3	49455000	1.03E-02	promoter	super_enhancer	RPL24P7
chr3	23245000	chr3	30625000	2.58E-02	promoter	super_enhancer	UBE2E2-AS1
chr3	23245000	chr3	194305000	3.42E-03	promoter	super_enhancer	UBE2E2-AS1
chr3	23655000	chr3	38165000	1.88E-02	promoter	super_enhancer	RNU6-922P
chr3	23655000	chr3	141935000	3.04E-03	promoter	super_enhancer	RNU6-922P
chr3	24145000	chr3	30625000	3.50E-02	promoter	super_enhancer	LINC00691
chr3	24535000	chr3	42025000	2.30E-02	promoter	super_enhancer	THRB-AS1
chr3	25215000	chr3	30605000	3.82E-04	promoter	super_enhancer	RARB
chr3	25365000	chr3	171825000	2.77E-03	promoter	super_enhancer	CFL1P7
chr3	25795000	chr3	42055000	1.65E-02	promoter	super_enhancer	TAF9BP1
chr3	25795000	chr3	49495000	1.04E-02	promoter	super_enhancer	TAF9BP1
chr3	25915000	chr3	43195000	1.48E-02	promoter	super_enhancer	LINC00692
chr3	27745000	chr3	52045000	1.31E-02	promoter	super_enhancer	AC098614.3
chr3	29685000	chr3	42055000	2.01E-02	promoter	super_enhancer	RBMS3-AS2
chr3	30565000	chr3	38165000	2.66E-02	promoter	super_enhancer	AC116035.1
chr3	30605000	chr3	39685000	2.44E-02	super_enhancer	promoter	NFU1P1

chr3	30615000	chr3	42005000	2.23E-02	super_enhancer	promoter	ULK4
chr3	30615000	chr3	46925000	1.91E-02	super_enhancer	promoter	MYL3
chr3	30615000	chr3	49795000	1.43E-02	super_enhancer	promoter	COX6CP14
chr3	30615000	chr3	52005000	1.32E-02	super_enhancer	promoter	ABHD14A
chr3	30615000	chr3	117395000	2.44E-03	super_enhancer	promoter	RP11-768G7.1
chr3	30615000	chr3	125635000	2.64E-03	super_enhancer	promoter	RP11-379B18.5
chr3	30625000	chr3	42845000	2.16E-02	super_enhancer	promoter	ACKR2
chr3	30625000	chr3	49205000	2.04E-02	super_enhancer	promoter	CCDC71
chr3	30625000	chr3	171955000	3.19E-03	super_enhancer	promoter	RN7SL141P
chr3	31225000	chr3	43165000	2.39E-02	promoter	super_enhancer	CNN2P6
chr3	31745000	chr3	45165000	1.58E-02	promoter	super_enhancer	OSBPL10-AS1
chr3	31745000	chr3	129595000	2.66E-03	promoter	super_enhancer	OSBPL10-AS1
chr3	32545000	chr3	42075000	3.03E-02	promoter	super_enhancer	CMTM6
chr3	32545000	chr3	49905000	2.13E-02	promoter	super_enhancer	CMTM6
chr3	33075000	chr3	46945000	2.11E-02	promoter	super_enhancer	SEC13P1
chr3	33145000	chr3	42075000	3.28E-02	promoter	super_enhancer	RN7SL296P
chr3	33145000	chr3	49495000	1.87E-02	promoter	super_enhancer	RN7SL296P
chr3	33155000	chr3	50165000	3.21E-04	promoter	super_enhancer	CRTAP
chr3	33455000	chr3	185735000	3.28E-03	promoter	super_enhancer	RNU7-110P
chr3	33535000	chr3	38165000	4.57E-02	promoter	super_enhancer	RNA5SP128
chr3	33535000	chr3	45155000	1.89E-02	promoter	super_enhancer	RNA5SP128
chr3	33535000	chr3	49895000	1.61E-02	promoter	super_enhancer	RNA5SP128
chr3	33595000	chr3	45135000	2.68E-02	promoter	super_enhancer	COX6CP10
chr3	33595000	chr3	57975000	8.28E-03	promoter	super_enhancer	COX6CP10

chr3	33595000	chr3	194325000	4.03E-03	promoter	super_enhancer	COX6CP10
chr3	34245000	chr3	124855000	2.59E-03	promoter	super_enhancer	AC123023.1
chr3	34585000	chr3	45115000	1.39E-02	promoter	super_enhancer	AC018359.3
chr3	36865000	chr3	149575000	3.91E-03	promoter	super_enhancer	RP11-640L9.1
chr3	36865000	chr3	172175000	3.98E-03	promoter	super_enhancer	RP11-640L9.1
chr3	37035000	chr3	42065000	4.48E-02	promoter	super_enhancer	EPM2AIP1
chr3	37175000	chr3	45165000	1.57E-02	promoter	super_enhancer	UBE2D3P2
chr3	37225000	chr3	46975000	2.32E-02	promoter	super_enhancer	LRRFIP2
chr3	37225000	chr3	50255000	2.66E-02	promoter	super_enhancer	LRRFIP2
chr3	37285000	chr3	45135000	3.85E-02	promoter	super_enhancer	GOLGA4
chr3	37285000	chr3	50245000	2.96E-02	promoter	super_enhancer	GOLGA4
chr3	37615000	chr3	46945000	3.03E-02	promoter	super_enhancer	RNU7-73P
chr3	37615000	chr3	123585000	4.32E-03	promoter	super_enhancer	RNU7-73P
chr3	38165000	chr3	49235000	3.01E-02	super_enhancer	promoter	CCDC36
chr3	38165000	chr3	51575000	2.22E-02	super_enhancer	promoter	RAD54L2
chr3	38165000	chr3	52595000	1.36E-02	super_enhancer	promoter	RNU6ATAC16P
chr3	38165000	chr3	64675000	6.21E-03	super_enhancer	promoter	ADAMTS9
chr3	38165000	chr3	186435000	3.06E-03	super_enhancer	promoter	KNG1
chr3	38175000	chr3	38345000	3.77E-04	super_enhancer	promoter	RNU6-235P
chr3	38175000	chr3	38495000	4.44E-02	super_enhancer	promoter	ACVR2B
chr3	38175000	chr3	47825000	2.57E-02	super_enhancer	promoter	SMARCC1
chr3	38175000	chr3	50195000	2.41E-02	super_enhancer	promoter	SEMA3F-AS1
chr3	38175000	chr3	50315000	2.25E-02	super_enhancer	promoter	LSMEM2
chr3	38175000	chr3	50385000	2.41E-02	super_enhancer	promoter	ZMYND10

chr3	38175000	chr3	191275000	2.11E-03	super_enhancer	promoter	RP11-197K6.1
chr3	38345000	chr3	43195000	4.21E-02	promoter	super_enhancer	RNU6-235P
chr3	38345000	chr3	45165000	2.62E-02	promoter	super_enhancer	RNU6-235P
chr3	38495000	chr3	45205000	2.33E-02	promoter	super_enhancer	ACVR2B
chr3	39155000	chr3	49205000	2.88E-02	super_enhancer	promoter	CCDC71
chr3	39155000	chr3	50375000	2.94E-02	super_enhancer	promoter	RASSF1-AS1
chr3	39155000	chr3	52595000	1.18E-02	super_enhancer	promoter	RNU6ATAC16P
chr3	39155000	chr3	120315000	3.16E-03	super_enhancer	promoter	NDUFB4
chr3	39235000	chr3	42045000	1.15E-02	promoter	super_enhancer	XIRP1
chr3	39255000	chr3	45185000	3.36E-02	promoter	super_enhancer	DSTNP4
chr3	39425000	chr3	45155000	4.03E-02	promoter	super_enhancer	SLC25A38
chr3	39425000	chr3	49485000	3.20E-02	promoter	super_enhancer	SLC25A38
chr3	39425000	chr3	49895000	2.64E-02	promoter	super_enhancer	SLC25A38
chr3	39425000	chr3	52055000	2.46E-02	promoter	super_enhancer	SLC25A38
chr3	39685000	chr3	49475000	3.00E-02	promoter	super_enhancer	NFU1P1
chr3	39685000	chr3	52045000	2.43E-02	promoter	super_enhancer	NFU1P1
chr3	40365000	chr3	42045000	2.09E-02	promoter	super_enhancer	PGAM1P3
chr3	40495000	chr3	45155000	4.03E-02	promoter	super_enhancer	ENTPD3-AS1
chr3	40565000	chr3	42065000	4.35E-02	promoter	super_enhancer	ZNF621
chr3	40565000	chr3	49485000	3.11E-02	promoter	super_enhancer	ZNF621
chr3	42005000	chr3	50165000	4.46E-02	promoter	super_enhancer	ULK4
chr3	42005000	chr3	57975000	1.45E-02	promoter	super_enhancer	ULK4
chr3	42025000	chr3	50125000	3.75E-02	super_enhancer	promoter	RBM5
chr3	42025000	chr3	51975000	4.24E-02	super_enhancer	promoter	PARP3

chr3	42025000	chr3	127295000	6.71E-03	super_enhancer	promoter	MIR6825
chr3	42025000	chr3	176845000	3.11E-03	super_enhancer	promoter	RNU6-681P
chr3	42045000	chr3	50315000	4.08E-02	super_enhancer	promoter	LSMEM2
chr3	42045000	chr3	51575000	3.14E-02	super_enhancer	promoter	RAD54L2
chr3	42045000	chr3	186915000	4.82E-03	super_enhancer	promoter	RTP1
chr3	42055000	chr3	47705000	4.49E-02	super_enhancer	promoter	RP11-395P16.1
chr3	42065000	chr3	42895000	4.69E-02	super_enhancer	promoter	RP11-70C1.3
chr3	42065000	chr3	51575000	3.20E-02	super_enhancer	promoter	RAD54L2
chr3	42065000	chr3	123435000	5.53E-03	super_enhancer	promoter	RP11-9N20.3
chr3	42065000	chr3	132035000	3.80E-03	super_enhancer	promoter	ACPP
chr3	42065000	chr3	152785000	4.37E-03	super_enhancer	promoter	HMGN2P13
chr3	42075000	chr3	50195000	4.31E-02	super_enhancer	promoter	SEMA3F-AS1
chr3	42075000	chr3	125985000	5.59E-03	super_enhancer	promoter	RP11-71E19.1
chr3	42075000	chr3	155755000	4.89E-03	super_enhancer	promoter	KCNAB1
chr3	42305000	chr3	134485000	4.16E-03	promoter	super_enhancer	RP11-333B11.1
chr3	42465000	chr3	50175000	3.74E-02	promoter	super_enhancer	AC092048.1
chr3	42745000	chr3	126905000	5.74E-03	promoter	super_enhancer	HHATL
chr3	42815000	chr3	49895000	3.08E-02	promoter	super_enhancer	CCDC13
chr3	42895000	chr3	49495000	6.64E-04	promoter	super_enhancer	RP11-70C1.3
chr3	43165000	chr3	49505000	3.40E-02	super_enhancer	promoter	RNA5SP130
chr3	43165000	chr3	149485000	3.72E-03	super_enhancer	promoter	AC069216.1
chr3	43175000	chr3	138515000	4.45E-03	super_enhancer	promoter	RPL23AP40
chr3	43175000	chr3	151355000	3.44E-03	super_enhancer	promoter	RP11-454C18.1
chr3	43185000	chr3	44465000	4.80E-02	super_enhancer	promoter	LINC00694

chr3	43185000	chr3	66025000	8.29E-03	super_enhancer	promoter	MAGI1
chr3	43185000	chr3	122135000	3.65E-03	super_enhancer	promoter	WDR5B
chr3	43185000	chr3	149955000	3.72E-03	super_enhancer	promoter	LINC01213
chr3	43195000	chr3	44835000	2.50E-03	super_enhancer	promoter	AC098649.1
chr3	43195000	chr3	51535000	2.66E-02	super_enhancer	promoter	VPRBP
chr3	43195000	chr3	62935000	6.47E-03	super_enhancer	promoter	LINC00698
chr3	44465000	chr3	49135000	4.95E-02	promoter	super_enhancer	LINC00694
chr3	44835000	chr3	141365000	4.33E-03	promoter	super_enhancer	AC098649.1
chr3	45105000	chr3	46065000	1.36E-02	super_enhancer	promoter	NRBF2P2
chr3	45105000	chr3	46395000	4.76E-02	super_enhancer	promoter	CCR2
chr3	45105000	chr3	46925000	2.01E-02	super_enhancer	promoter	MYL3
chr3	45105000	chr3	49235000	1.62E-03	super_enhancer	promoter	CCDC36
chr3	45135000	chr3	63995000	9.58E-03	super_enhancer	promoter	RP11-245J9.5
chr3	45135000	chr3	107095000	3.66E-03	super_enhancer	promoter	CCDC54
chr3	45135000	chr3	143575000	4.15E-03	super_enhancer	promoter	RP11-8J23.1
chr3	45135000	chr3	149105000	4.35E-03	super_enhancer	promoter	RP11-278L15.2
chr3	45145000	chr3	108445000	3.62E-03	super_enhancer	promoter	RP11-148O7.2
chr3	45145000	chr3	141595000	3.53E-03	super_enhancer	promoter	ATP1B3
chr3	45155000	chr3	51575000	3.29E-02	super_enhancer	promoter	RAD54L2
chr3	45155000	chr3	52865000	2.15E-02	super_enhancer	promoter	ITIH4
chr3	45155000	chr3	58365000	1.36E-02	super_enhancer	promoter	RP11-802O23.4
chr3	45155000	chr3	63845000	8.42E-03	super_enhancer	promoter	THOC7-AS1
chr3	45155000	chr3	193345000	2.50E-03	super_enhancer	promoter	OPA1-AS1
chr3	45165000	chr3	46065000	1.83E-02	super_enhancer	promoter	NRBF2P2

chr3	45165000	chr3	49505000	4.69E-02	super_enhancer	promoter	RNA5SP130
chr3	45165000	chr3	141585000	4.06E-03	super_enhancer	promoter	HMGN2P25
chr3	45175000	chr3	51535000	3.16E-02	super_enhancer	promoter	VPRBP
chr3	45175000	chr3	66025000	8.43E-03	super_enhancer	promoter	MAGI1
chr3	45175000	chr3	186285000	2.94E-03	super_enhancer	promoter	DNAJB11
chr3	45185000	chr3	51895000	2.74E-02	super_enhancer	promoter	IQCF2
chr3	45185000	chr3	196065000	3.26E-03	super_enhancer	promoter	TM4SF19-TCTEX1D2
chr3	45195000	chr3	47205000	1.49E-02	super_enhancer	promoter	SETD2
chr3	45195000	chr3	51535000	3.82E-02	super_enhancer	promoter	VPRBP
chr3	45195000	chr3	132105000	3.92E-03	super_enhancer	promoter	RP11-65E22.2
chr3	45205000	chr3	52865000	1.47E-02	super_enhancer	promoter	ITIH4
chr3	45205000	chr3	141205000	2.72E-03	super_enhancer	promoter	RASA2
chr3	45215000	chr3	52005000	3.53E-02	super_enhancer	promoter	ABHD14A
chr3	45215000	chr3	64675000	7.10E-03	super_enhancer	promoter	ADAMTS9
chr3	45215000	chr3	66345000	7.00E-03	super_enhancer	promoter	RNU6-787P
chr3	45215000	chr3	169775000	2.94E-03	super_enhancer	promoter	RNU4-38P
chr3	45215000	chr3	184015000	3.16E-03	super_enhancer	promoter	PSMD2
chr3	46395000	chr3	123605000	4.23E-03	promoter	super_enhancer	CCR2
chr3	46855000	chr3	46935000	2.48E-02	promoter	super_enhancer	PRSS50
chr3	46935000	chr3	49205000	2.62E-02	super_enhancer	promoter	CCDC71
chr3	46935000	chr3	156165000	4.98E-03	super_enhancer	promoter	KCNAB1-AS1
chr3	46935000	chr3	197025000	4.11E-03	super_enhancer	promoter	DLG1-AS1
chr3	46945000	chr3	47825000	3.70E-02	super_enhancer	promoter	SMARCC1
chr3	46945000	chr3	52725000	2.34E-02	super_enhancer	promoter	SNORD19B

chr3	46955000	chr3	136665000	3.62E-03	super_enhancer	promoter	IL20RB
chr3	46965000	chr3	47025000	4.91E-02	super_enhancer	promoter	CCDC12
chr3	46965000	chr3	47825000	2.60E-02	super_enhancer	promoter	SMARCC1
chr3	46975000	chr3	47555000	1.84E-02	super_enhancer	promoter	ELP6
chr3	46975000	chr3	139565000	3.11E-03	super_enhancer	promoter	TRMT112P5
chr3	46985000	chr3	52865000	3.54E-02	super_enhancer	promoter	ITIH4
chr3	46985000	chr3	124775000	5.36E-03	super_enhancer	promoter	HEG1
chr3	47025000	chr3	185735000	4.49E-03	promoter	super_enhancer	CCDC12
chr3	47555000	chr3	195915000	4.56E-03	promoter	super_enhancer	ELP6
chr3	47705000	chr3	195905000	3.39E-03	promoter	super_enhancer	RP11-395P16.1
chr3	47705000	chr3	196325000	3.86E-03	promoter	super_enhancer	RP11-395P16.1
chr3	48265000	chr3	48465000	1.96E-04	promoter	super_enhancer	CAMP
chr3	48265000	chr3	48565000	3.00E-03	promoter	super_enhancer	CAMP
chr3	48265000	chr3	184315000	5.65E-03	promoter	super_enhancer	CAMP
chr3	48465000	chr3	48725000	1.38E-02	super_enhancer	promoter	NCKIPSD
chr3	48465000	chr3	195635000	3.28E-03	super_enhancer	promoter	TNK2-AS1
chr3	48475000	chr3	172545000	3.79E-03	super_enhancer	promoter	AC108667.1
chr3	48505000	chr3	48595000	1.88E-03	promoter	super_enhancer	TREX1
chr3	48505000	chr3	159765000	4.29E-03	promoter	super_enhancer	TREX1
chr3	48505000	chr3	195885000	3.42E-03	promoter	super_enhancer	TREX1
chr3	48565000	chr3	52715000	2.87E-02	super_enhancer	promoter	GNL3
chr3	48565000	chr3	98935000	3.02E-03	super_enhancer	promoter	ACTG1P13
chr3	48595000	chr3	113995000	2.71E-03	super_enhancer	promoter	TIGIT
chr3	48595000	chr3	196715000	3.41E-03	super_enhancer	promoter	AC011322.1

chr3	48725000	chr3	197505000	3.88E-03	promoter	super_enhancer	NCKIPSD
chr3	48955000	chr3	124845000	5.61E-03	promoter	super_enhancer	ARIH2
chr3	49135000	chr3	49505000	3.77E-02	super_enhancer	promoter	RNA5SP130
chr3	49135000	chr3	121555000	3.36E-03	super_enhancer	promoter	IQCB1
chr3	49135000	chr3	138665000	3.89E-03	super_enhancer	promoter	FOXL2NB
chr3	49205000	chr3	49455000	3.75E-02	promoter	super_enhancer	CCDC71
chr3	49205000	chr3	49475000	6.98E-03	promoter	super_enhancer	CCDC71
chr3	49315000	chr3	49475000	9.18E-03	promoter	super_enhancer	C3orf62
chr3	49405000	chr3	194295000	4.17E-03	promoter	super_enhancer	RHOA-IT1
chr3	49445000	chr3	171945000	3.36E-03	super_enhancer	promoter	RP11-423E7.1
chr3	49455000	chr3	58365000	1.93E-02	super_enhancer	promoter	RP11-802O23.4
chr3	49455000	chr3	127795000	4.54E-03	super_enhancer	promoter	RNU2-37P
chr3	49465000	chr3	51575000	1.52E-02	super_enhancer	promoter	RAD54L2
chr3	49475000	chr3	142895000	3.63E-03	super_enhancer	promoter	PBX2P1
chr3	49495000	chr3	194475000	4.34E-03	super_enhancer	promoter	AC090505.5
chr3	49845000	chr3	50165000	1.60E-02	promoter	super_enhancer	MIR5193
chr3	49845000	chr3	50255000	2.66E-02	promoter	super_enhancer	MIR5193
chr3	49845000	chr3	141935000	3.74E-03	promoter	super_enhancer	MIR5193
chr3	49895000	chr3	55645000	2.27E-02	super_enhancer	promoter	RP11-58O15.1
chr3	49895000	chr3	179755000	2.57E-03	super_enhancer	promoter	PEX5L
chr3	49905000	chr3	170825000	3.16E-03	super_enhancer	promoter	MIR569
chr3	49905000	chr3	196725000	5.02E-03	super_enhancer	promoter	MFI2-AS1
chr3	50125000	chr3	184325000	3.45E-03	promoter	super_enhancer	RBM5
chr3	50165000	chr3	50195000	1.17E-02	super_enhancer	promoter	SEMA3F-AS1

chr3	50175000	chr3	50305000	9.17E-03	super_enhancer	promoter	SEMA3B-AS1
chr3	50175000	chr3	55385000	2.49E-02	super_enhancer	promoter	RP11-875H7.1
chr3	50195000	chr3	194585000	4.05E-03	promoter	super_enhancer	SEMA3F-AS1
chr3	50245000	chr3	54675000	2.88E-02	super_enhancer	promoter	ESRG
chr3	50245000	chr3	98245000	4.00E-03	super_enhancer	promoter	RPL38P4
chr3	50255000	chr3	50315000	8.58E-03	super_enhancer	promoter	LSMEM2
chr3	50255000	chr3	52005000	3.67E-03	super_enhancer	promoter	ABHD14A
chr3	50255000	chr3	55385000	2.73E-02	super_enhancer	promoter	RP11-875H7.1
chr3	50255000	chr3	58365000	2.47E-02	super_enhancer	promoter	RP11-802O23.4
chr3	50255000	chr3	111315000	5.68E-03	super_enhancer	promoter	ZBED2
chr3	50255000	chr3	125465000	3.78E-03	super_enhancer	promoter	OR7E97P
chr3	50255000	chr3	153075000	3.97E-03	super_enhancer	promoter	RP11-292E2.1
chr3	50265000	chr3	66675000	1.56E-02	super_enhancer	promoter	RP11-814M22.2
chr3	50265000	chr3	125455000	4.15E-03	super_enhancer	promoter	AF186996.1
chr3	50305000	chr3	195915000	4.37E-03	promoter	super_enhancer	SEMA3B-AS1
chr3	50315000	chr3	149385000	3.91E-03	promoter	super_enhancer	LSMEM2
chr3	50315000	chr3	195865000	4.80E-03	promoter	super_enhancer	LSMEM2
chr3	52005000	chr3	134475000	3.27E-03	promoter	super_enhancer	ABHD14A
chr3	52055000	chr3	183415000	4.27E-03	super_enhancer	promoter	YEATS2
chr3	55385000	chr3	57985000	3.82E-02	promoter	super_enhancer	RP11-875H7.1
chr3	57985000	chr3	58365000	2.51E-02	super_enhancer	promoter	RP11-802O23.4
chr3	57985000	chr3	63805000	1.69E-02	super_enhancer	promoter	C3orf49
chr3	57985000	chr3	114035000	2.65E-03	super_enhancer	promoter	RP11-553L6.5
chr3	63995000	chr3	126895000	2.65E-03	promoter	super_enhancer	RP11-245J9.5

chr3	93695000	chr3	123585000	6.20E-03	promoter	super_enhancer	RNU6-511P
chr3	94655000	chr3	124845000	5.99E-03	promoter	super_enhancer	RP11-118N24.2
chr3	96495000	chr3	111925000	3.89E-03	promoter	super_enhancer	CDV3P1
chr3	97755000	chr3	194565000	1.69E-03	promoter	super_enhancer	GABRR3
chr3	97915000	chr3	124835000	6.79E-03	promoter	super_enhancer	OR5H5P
chr3	97915000	chr3	141935000	3.96E-03	promoter	super_enhancer	OR5H5P
chr3	98245000	chr3	124845000	7.80E-03	promoter	super_enhancer	RPL38P4
chr3	98245000	chr3	134365000	5.39E-03	promoter	super_enhancer	RPL38P4
chr3	98935000	chr3	123585000	7.07E-03	promoter	super_enhancer	ACTG1P13
chr3	98935000	chr3	139295000	3.71E-03	promoter	super_enhancer	ACTG1P13
chr3	99525000	chr3	115785000	1.02E-02	promoter	super_enhancer	RP11-383I23.2
chr3	99525000	chr3	126905000	1.36E-02	promoter	super_enhancer	RP11-383I23.2
chr3	99525000	chr3	171795000	3.55E-03	promoter	super_enhancer	RP11-383I23.2
chr3	99905000	chr3	124855000	9.75E-03	promoter	super_enhancer	TMEM30C
chr3	99905000	chr3	127595000	8.57E-03	promoter	super_enhancer	TMEM30C
chr3	102475000	chr3	170745000	2.50E-03	promoter	super_enhancer	RNU6-461P
chr3	105085000	chr3	123595000	1.05E-02	promoter	super_enhancer	ALCAM
chr3	105085000	chr3	141955000	6.93E-03	promoter	super_enhancer	ALCAM
chr3	106615000	chr3	120165000	1.10E-02	promoter	super_enhancer	MTND4P16
chr3	106615000	chr3	127595000	1.16E-02	promoter	super_enhancer	MTND4P16
chr3	106725000	chr3	172165000	3.19E-03	promoter	super_enhancer	RNU6-1308P
chr3	108305000	chr3	111925000	2.19E-02	promoter	super_enhancer	AC069499.1
chr3	108305000	chr3	126895000	1.29E-02	promoter	super_enhancer	AC069499.1
chr3	109035000	chr3	124835000	1.18E-02	promoter	super_enhancer	DPPA2

chr3	109115000	chr3	111905000	3.70E-02	promoter	super_enhancer	RP11-702L6.1
chr3	109115000	chr3	127595000	1.60E-02	promoter	super_enhancer	RP11-702L6.1
chr3	109215000	chr3	129605000	9.03E-03	promoter	super_enhancer	AC092905.1
chr3	111315000	chr3	120145000	2.54E-02	promoter	super_enhancer	ZBED2
chr3	111315000	chr3	120155000	2.63E-02	promoter	super_enhancer	ZBED2
chr3	111315000	chr3	149375000	7.82E-03	promoter	super_enhancer	ZBED2
chr3	111805000	chr3	111915000	4.53E-02	promoter	super_enhancer	C3orf52
chr3	111905000	chr3	112245000	6.16E-03	super_enhancer	promoter	OR7E100P
chr3	111905000	chr3	120555000	9.69E-03	super_enhancer	promoter	RP11-500K19.1
chr3	111905000	chr3	123305000	1.24E-02	super_enhancer	promoter	HACD2
chr3	111915000	chr3	173775000	2.10E-03	super_enhancer	promoter	RN7SKP234
chr3	111925000	chr3	125985000	1.27E-02	super_enhancer	promoter	RP11-71E19.1
chr3	111925000	chr3	169665000	2.65E-03	super_enhancer	promoter	RP11-379K17.5
chr3	111935000	chr3	169755000	2.66E-03	super_enhancer	promoter	GPR160
chr3	111935000	chr3	187895000	1.92E-03	super_enhancer	promoter	FLJ42393
chr3	112245000	chr3	126905000	1.50E-02	promoter	super_enhancer	OR7E100P
chr3	113235000	chr3	129625000	1.40E-02	promoter	super_enhancer	SPICE1
chr3	113415000	chr3	129615000	1.47E-02	promoter	super_enhancer	KIAA2018
chr3	113415000	chr3	172165000	5.98E-03	promoter	super_enhancer	KIAA2018
chr3	113465000	chr3	123615000	1.72E-02	promoter	super_enhancer	NAA50
chr3	113775000	chr3	120155000	2.83E-02	promoter	super_enhancer	KIAA1407
chr3	113775000	chr3	139305000	1.05E-02	promoter	super_enhancer	KIAA1407
chr3	113995000	chr3	141365000	8.02E-03	promoter	super_enhancer	TIGIT
chr3	114035000	chr3	126285000	1.66E-02	promoter	super_enhancer	RP11-553L6.5

chr3	114035000	chr3	170715000	2.69E-03	promoter	super_enhancer	RP11-553L6.5
chr3	115785000	chr3	125465000	1.21E-02	super_enhancer	promoter	OR7E97P
chr3	115785000	chr3	125985000	1.91E-02	super_enhancer	promoter	RP11-71E19.1
chr3	115785000	chr3	129755000	1.09E-02	super_enhancer	promoter	OR7E21P
chr3	115785000	chr3	157825000	4.92E-03	super_enhancer	promoter	SHOX2
chr3	115785000	chr3	175485000	2.22E-03	super_enhancer	promoter	RNU6-1317P
chr3	115795000	chr3	125065000	2.11E-02	super_enhancer	promoter	RP11-775J23.2
chr3	115795000	chr3	125635000	1.70E-02	super_enhancer	promoter	RP11-379B18.5
chr3	115795000	chr3	155705000	5.41E-03	super_enhancer	promoter	SETP14
chr3	115805000	chr3	121265000	4.40E-02	super_enhancer	promoter	POLQ
chr3	115805000	chr3	121375000	4.43E-02	super_enhancer	promoter	RNU4-62P
chr3	115805000	chr3	129815000	2.39E-02	super_enhancer	promoter	AC083906.2
chr3	115805000	chr3	149095000	7.38E-03	super_enhancer	promoter	TM4SF1-AS1
chr3	116745000	chr3	128485000	2.51E-02	promoter	super_enhancer	PTMAP8
chr3	117395000	chr3	134365000	1.90E-02	promoter	super_enhancer	RP11-768G7.1
chr3	118865000	chr3	124865000	3.92E-02	promoter	super_enhancer	IGSF11
chr3	118945000	chr3	126215000	4.31E-02	promoter	super_enhancer	B4GALT4-AS1
chr3	119355000	chr3	120175000	4.60E-02	promoter	super_enhancer	RP11-190C22.1
chr3	119355000	chr3	123595000	5.07E-05	promoter	super_enhancer	RP11-190C22.1
chr3	119355000	chr3	139295000	1.82E-02	promoter	super_enhancer	RP11-190C22.1
chr3	119355000	chr3	197505000	3.70E-03	promoter	super_enhancer	RP11-190C22.1
chr3	120005000	chr3	185735000	5.32E-03	promoter	super_enhancer	GPR156
chr3	120025000	chr3	172135000	6.83E-03	promoter	super_enhancer	RP11-174O3.1
chr3	120145000	chr3	121265000	1.11E-02	super_enhancer	promoter	POLQ

chr3	120145000	chr3	125455000	4.03E-02	super_enhancer	promoter	AF186996.1
chr3	120145000	chr3	155705000	9.19E-03	super_enhancer	promoter	SETP14
chr3	120155000	chr3	121555000	3.52E-02	super_enhancer	promoter	IQCB1
chr3	120155000	chr3	127005000	4.10E-02	super_enhancer	promoter	RP11-88I21.1
chr3	120155000	chr3	148705000	1.14E-02	super_enhancer	promoter	UBQLN4P1
chr3	120165000	chr3	120525000	1.77E-03	super_enhancer	promoter	NAP1L1P3
chr3	120165000	chr3	132365000	1.73E-02	super_enhancer	promoter	HSPA8P19
chr3	120165000	chr3	146135000	7.43E-03	super_enhancer	promoter	RP11-758I14.3
chr3	120175000	chr3	129775000	1.62E-02	super_enhancer	promoter	RP11-77P16.3
chr3	120175000	chr3	134155000	2.29E-02	super_enhancer	promoter	HMGB3P13
chr3	120175000	chr3	135685000	1.76E-02	super_enhancer	promoter	PPP2R3A
chr3	120175000	chr3	138725000	2.34E-02	super_enhancer	promoter	PRR23A
chr3	120175000	chr3	149105000	1.34E-02	super_enhancer	promoter	RP11-278L15.2
chr3	120175000	chr3	196595000	4.13E-03	super_enhancer	promoter	SENP5
chr3	120525000	chr3	149575000	9.15E-03	promoter	super_enhancer	NAP1L1P3
chr3	120555000	chr3	134365000	2.02E-02	promoter	super_enhancer	RP11-500K19.1
chr3	120645000	chr3	141945000	1.84E-02	promoter	super_enhancer	RP11-500K19.2
chr3	121075000	chr3	129625000	2.02E-02	promoter	super_enhancer	RP11-10G15.4
chr3	121265000	chr3	170735000	5.83E-03	promoter	super_enhancer	POLQ
chr3	121265000	chr3	170745000	6.99E-03	promoter	super_enhancer	POLQ
chr3	122035000	chr3	134325000	2.96E-02	promoter	super_enhancer	HNRNPA1P23
chr3	122035000	chr3	149575000	1.10E-02	promoter	super_enhancer	HNRNPA1P23
chr3	122135000	chr3	195895000	4.18E-03	promoter	super_enhancer	WDR5B
chr3	122235000	chr3	134315000	3.24E-02	promoter	super_enhancer	KPNA1

chr3	122235000	chr3	184315000	7.13E-03	promoter	super_enhancer	KPNA1
chr3	122295000	chr3	134365000	3.28E-02	promoter	super_enhancer	PARP15
chr3	123305000	chr3	124855000	4.90E-02	promoter	super_enhancer	HACD2
chr3	123585000	chr3	155755000	1.33E-02	super_enhancer	promoter	KCNAB1
chr3	123585000	chr3	196065000	3.94E-03	super_enhancer	promoter	TM4SF19-TCTEX1D2
chr3	123595000	chr3	138555000	2.63E-02	super_enhancer	promoter	PIK3CB
chr3	123595000	chr3	170075000	7.27E-03	super_enhancer	promoter	SKIL
chr3	123595000	chr3	195415000	3.43E-03	super_enhancer	promoter	AC233280.3
chr3	123605000	chr3	137835000	2.16E-02	super_enhancer	promoter	DZIP1L
chr3	123605000	chr3	142315000	2.10E-02	super_enhancer	promoter	PLS1
chr3	123605000	chr3	179065000	4.32E-03	super_enhancer	promoter	MFN1
chr3	123615000	chr3	135685000	1.74E-02	super_enhancer	promoter	PPP2R3A
chr3	123615000	chr3	180455000	3.93E-03	super_enhancer	promoter	RN7SL229P
chr3	124835000	chr3	150005000	1.38E-02	super_enhancer	promoter	LINC01214
chr3	124845000	chr3	139555000	2.02E-02	super_enhancer	promoter	RP11-166D18.1
chr3	124845000	chr3	161045000	1.10E-02	super_enhancer	promoter	EEF1GP4
chr3	124845000	chr3	168645000	7.31E-03	super_enhancer	promoter	RP11-368I23.3
chr3	124845000	chr3	194255000	4.63E-03	super_enhancer	promoter	RNU6-1101P
chr3	124855000	chr3	125095000	2.07E-02	super_enhancer	promoter	ZNF148
chr3	124855000	chr3	138065000	3.16E-02	super_enhancer	promoter	MRAS
chr3	124855000	chr3	141585000	2.76E-02	super_enhancer	promoter	HMGN2P25
chr3	124855000	chr3	149075000	1.43E-02	super_enhancer	promoter	RP11-2G17.1
chr3	124855000	chr3	157825000	1.22E-02	super_enhancer	promoter	SHOX2
chr3	124855000	chr3	193025000	3.96E-03	super_enhancer	promoter	ATP13A5-AS1

chr3	124865000	chr3	139565000	2.23E-02	super_enhancer	promoter	TRMT112P5
chr3	124865000	chr3	160395000	1.00E-02	super_enhancer	promoter	ARL14
chr3	124865000	chr3	186525000	5.13E-03	super_enhancer	promoter	RFC4
chr3	124875000	chr3	137835000	2.36E-02	super_enhancer	promoter	DZIP1L
chr3	124875000	chr3	138555000	2.56E-02	super_enhancer	promoter	PIK3CB
chr3	124875000	chr3	194255000	3.43E-03	super_enhancer	promoter	RNU6-1101P
chr3	125455000	chr3	129595000	4.36E-02	promoter	super_enhancer	AF186996.1
chr3	125455000	chr3	134325000	3.33E-02	promoter	super_enhancer	AF186996.1
chr3	125475000	chr3	128485000	4.40E-03	promoter	super_enhancer	GS1-388B5.8
chr3	125775000	chr3	194305000	5.69E-03	promoter	super_enhancer	RP11-158I23.1
chr3	125985000	chr3	141955000	3.07E-02	promoter	super_enhancer	RP11-71E19.1
chr3	126215000	chr3	131705000	4.88E-02	super_enhancer	promoter	MIR5704
chr3	126215000	chr3	138665000	3.63E-02	super_enhancer	promoter	FOXL2NB
chr3	126215000	chr3	151515000	1.42E-02	super_enhancer	promoter	RP11-64D22.5
chr3	126215000	chr3	179615000	4.83E-03	super_enhancer	promoter	PEX5L-AS2
chr3	126215000	chr3	184275000	8.53E-03	super_enhancer	promoter	EIF2B5-AS1
chr3	126275000	chr3	194255000	5.73E-03	super_enhancer	promoter	RNU6-1101P
chr3	126285000	chr3	187825000	6.04E-03	super_enhancer	promoter	RP11-430L16.1
chr3	126375000	chr3	170735000	6.33E-03	promoter	super_enhancer	TXNRD3
chr3	126895000	chr3	142315000	3.00E-02	super_enhancer	promoter	PLS1
chr3	126895000	chr3	155095000	1.13E-02	super_enhancer	promoter	RP11-639F1.1
chr3	126895000	chr3	155895000	1.26E-02	super_enhancer	promoter	RP11-118D22.3
chr3	126895000	chr3	156535000	1.06E-02	super_enhancer	promoter	LINC00886
chr3	126895000	chr3	171185000	9.70E-03	super_enhancer	promoter	RP11-185E8.1

chr3	126905000	chr3	178985000	6.44E-03	super_enhancer	promoter	KCNMB3
chr3	126905000	chr3	183815000	7.57E-03	super_enhancer	promoter	HTR3E
chr3	126915000	chr3	154055000	9.83E-03	super_enhancer	promoter	RP11-316N24.2
chr3	126915000	chr3	164925000	7.02E-03	super_enhancer	promoter	LINC01322
chr3	127005000	chr3	184315000	8.09E-03	promoter	super_enhancer	RP11-88I21.1
chr3	127005000	chr3	195875000	5.69E-03	promoter	super_enhancer	RP11-88I21.1
chr3	127595000	chr3	135685000	2.57E-02	super_enhancer	promoter	PPP2R3A
chr3	128445000	chr3	141935000	2.60E-02	promoter	super_enhancer	RAB7A
chr3	128445000	chr3	171815000	5.51E-03	promoter	super_enhancer	RAB7A
chr3	128485000	chr3	138665000	3.22E-02	super_enhancer	promoter	FOXL2NB
chr3	128485000	chr3	179065000	5.91E-03	super_enhancer	promoter	MFN1
chr3	128495000	chr3	147885000	1.34E-02	super_enhancer	promoter	HNRNPA1P20
chr3	128495000	chr3	153075000	1.19E-02	super_enhancer	promoter	RP11-292E2.1
chr3	128495000	chr3	157825000	1.23E-02	super_enhancer	promoter	SHOX2
chr3	128495000	chr3	184015000	5.42E-03	super_enhancer	promoter	PSMD2
chr3	128635000	chr3	149585000	1.80E-02	promoter	super_enhancer	RP11-723O4.7
chr3	129035000	chr3	184315000	9.16E-03	promoter	super_enhancer	H1FX
chr3	129035000	chr3	194305000	5.85E-03	promoter	super_enhancer	H1FX
chr3	129035000	chr3	195885000	4.72E-03	promoter	super_enhancer	H1FX
chr3	129045000	chr3	129595000	1.93E-02	promoter	super_enhancer	NUP210P3
chr3	129045000	chr3	194325000	6.60E-03	promoter	super_enhancer	NUP210P3
chr3	129045000	chr3	197515000	5.30E-03	promoter	super_enhancer	NUP210P3
chr3	129595000	chr3	137725000	1.61E-02	super_enhancer	promoter	RP11-461M2.2
chr3	129595000	chr3	170305000	7.12E-03	super_enhancer	promoter	SLC7A14

chr3	129595000	chr3	172545000	5.41E-03	super_enhancer	promoter	AC108667.1
chr3	129595000	chr3	194815000	2.69E-03	super_enhancer	promoter	XXYLT1-AS1
chr3	129595000	chr3	195425000	4.58E-03	super_enhancer	promoter	MIR570
chr3	129605000	chr3	168645000	6.21E-03	super_enhancer	promoter	RP11-368I23.3
chr3	129605000	chr3	169165000	7.38E-03	super_enhancer	promoter	RP11-641D5.2
chr3	129615000	chr3	134075000	4.91E-02	super_enhancer	promoter	RPL39P5
chr3	129615000	chr3	156535000	8.14E-03	super_enhancer	promoter	LINC00886
chr3	129615000	chr3	169665000	6.91E-03	super_enhancer	promoter	RP11-379K17.5
chr3	129615000	chr3	176915000	3.73E-03	super_enhancer	promoter	TBL1XR1
chr3	129615000	chr3	196295000	3.29E-03	super_enhancer	promoter	FBXO45
chr3	129625000	chr3	134155000	4.23E-02	super_enhancer	promoter	HMGB3P13
chr3	129625000	chr3	141595000	1.79E-02	super_enhancer	promoter	ATP1B3
chr3	129625000	chr3	168005000	5.25E-03	super_enhancer	promoter	RP11-637O11.2
chr3	131245000	chr3	141955000	3.46E-02	promoter	super_enhancer	BCL2L12P1
chr3	131245000	chr3	149395000	2.28E-02	promoter	super_enhancer	BCL2L12P1
chr3	132005000	chr3	141945000	3.23E-02	promoter	super_enhancer	CPNE4
chr3	132315000	chr3	171815000	4.38E-03	promoter	super_enhancer	ACKR4
chr3	132315000	chr3	177355000	5.05E-03	promoter	super_enhancer	ACKR4
chr3	132365000	chr3	149375000	1.81E-02	promoter	super_enhancer	HSPA8P19
chr3	133265000	chr3	170745000	1.08E-02	promoter	super_enhancer	RP11-91K8.2
chr3	133265000	chr3	194295000	5.84E-03	promoter	super_enhancer	RP11-91K8.2
chr3	133615000	chr3	141935000	2.64E-02	promoter	super_enhancer	RAB6B
chr3	133615000	chr3	195885000	4.10E-03	promoter	super_enhancer	RAB6B
chr3	134075000	chr3	141365000	3.77E-02	promoter	super_enhancer	RPL39P5

chr3	134095000	chr3	141955000	4.61E-02	promoter	super_enhancer	AMOTL2
chr3	134095000	chr3	172135000	1.08E-02	promoter	super_enhancer	AMOTL2
chr3	134095000	chr3	172745000	7.19E-03	promoter	super_enhancer	AMOTL2
chr3	134095000	chr3	195895000	6.18E-03	promoter	super_enhancer	AMOTL2
chr3	134155000	chr3	141945000	3.47E-02	promoter	super_enhancer	HMGB3P13
chr3	134205000	chr3	149575000	1.77E-02	promoter	super_enhancer	CEP63
chr3	134315000	chr3	135645000	1.11E-02	super_enhancer	promoter	RP11-237P21.1
chr3	134315000	chr3	135685000	4.25E-03	super_enhancer	promoter	PPP2R3A
chr3	134315000	chr3	149455000	2.35E-02	super_enhancer	promoter	WWTR1
chr3	134315000	chr3	170075000	1.21E-02	super_enhancer	promoter	SKIL
chr3	134325000	chr3	157825000	2.02E-02	super_enhancer	promoter	SHOX2
chr3	134325000	chr3	169755000	1.40E-02	super_enhancer	promoter	GPR160
chr3	134325000	chr3	180585000	5.55E-03	super_enhancer	promoter	FXR1
chr3	134365000	chr3	158345000	1.35E-02	super_enhancer	promoter	RP11-79M21.3
chr3	134365000	chr3	192235000	3.69E-03	super_enhancer	promoter	FGF12-AS3
chr3	134375000	chr3	146225000	2.59E-02	super_enhancer	promoter	RP11-329N15.3
chr3	134375000	chr3	148415000	2.02E-02	super_enhancer	promoter	RPL38P1
chr3	134375000	chr3	149955000	2.43E-02	super_enhancer	promoter	LINC01213
chr3	134475000	chr3	138765000	4.76E-02	super_enhancer	promoter	PRR23C
chr3	134475000	chr3	155525000	1.38E-02	super_enhancer	promoter	C3orf33
chr3	134485000	chr3	141205000	4.54E-02	super_enhancer	promoter	RASA2
chr3	134485000	chr3	148415000	1.97E-02	super_enhancer	promoter	RPL38P1
chr3	135925000	chr3	149375000	2.49E-02	promoter	super_enhancer	RP11-463H24.1
chr3	136665000	chr3	141935000	3.21E-02	promoter	super_enhancer	IL20RB

chr3	137255000	chr3	185735000	5.94E-03	promoter	super_enhancer	RP11-575C1.1
chr3	137835000	chr3	172145000	9.11E-03	promoter	super_enhancer	DZIP1L
chr3	137835000	chr3	196335000	6.66E-03	promoter	super_enhancer	DZIP1L
chr3	138065000	chr3	149375000	3.01E-02	promoter	super_enhancer	MRAS
chr3	138065000	chr3	149395000	3.56E-02	promoter	super_enhancer	MRAS
chr3	138515000	chr3	197515000	6.66E-03	promoter	super_enhancer	RPL23AP40
chr3	138555000	chr3	171805000	1.16E-02	promoter	super_enhancer	PIK3CB
chr3	138555000	chr3	172185000	1.17E-02	promoter	super_enhancer	PIK3CB
chr3	138725000	chr3	197515000	6.29E-03	promoter	super_enhancer	PRR23A
chr3	139185000	chr3	149395000	3.16E-02	promoter	super_enhancer	RP11-319G6.3
chr3	139295000	chr3	168005000	8.22E-03	super_enhancer	promoter	RP11-637O11.2
chr3	139295000	chr3	176845000	5.42E-03	super_enhancer	promoter	RNU6-681P
chr3	139295000	chr3	181225000	6.07E-03	super_enhancer	promoter	RPL7AP25
chr3	139305000	chr3	154055000	1.62E-02	super_enhancer	promoter	RP11-316N24.2
chr3	139305000	chr3	158345000	1.51E-02	super_enhancer	promoter	RP11-79M21.3
chr3	139315000	chr3	139555000	2.68E-02	super_enhancer	promoter	RP11-166D18.1
chr3	139315000	chr3	170825000	6.12E-03	super_enhancer	promoter	MIR569
chr3	139315000	chr3	172515000	7.13E-03	super_enhancer	promoter	RNU4-4P
chr3	139555000	chr3	194575000	4.24E-03	promoter	super_enhancer	RP11-166D18.1
chr3	141205000	chr3	141945000	4.76E-04	promoter	super_enhancer	RASA2
chr3	141205000	chr3	149575000	3.14E-02	promoter	super_enhancer	RASA2
chr3	141365000	chr3	146135000	3.80E-02	super_enhancer	promoter	RP11-758I14.3
chr3	141365000	chr3	155375000	2.01E-02	super_enhancer	promoter	RPL6P7
chr3	141365000	chr3	187465000	5.92E-03	super_enhancer	promoter	BCL6

chr3	141585000	chr3	194305000	8.36E-03	promoter	super_enhancer	HMGN2P25
chr3	141585000	chr3	195885000	6.71E-03	promoter	super_enhancer	HMGN2P25
chr3	141585000	chr3	196325000	8.50E-03	promoter	super_enhancer	HMGN2P25
chr3	141935000	chr3	146215000	4.54E-02	super_enhancer	promoter	PLSCR2
chr3	141935000	chr3	153075000	1.98E-02	super_enhancer	promoter	RP11-292E2.1
chr3	141935000	chr3	156535000	1.58E-02	super_enhancer	promoter	LINC00886
chr3	141945000	chr3	146325000	4.22E-02	super_enhancer	promoter	PLSCR5
chr3	141945000	chr3	158055000	1.77E-02	super_enhancer	promoter	RP11-113A11.3
chr3	141955000	chr3	148705000	3.82E-02	super_enhancer	promoter	UBQLN4P1
chr3	141955000	chr3	196725000	8.22E-03	super_enhancer	promoter	MFI2-AS1
chr3	142315000	chr3	171795000	1.53E-02	promoter	super_enhancer	PLS1
chr3	142645000	chr3	149385000	3.42E-02	promoter	super_enhancer	RP11-372E1.4
chr3	142645000	chr3	149585000	3.57E-02	promoter	super_enhancer	RP11-372E1.4
chr3	142895000	chr3	194565000	4.13E-03	promoter	super_enhancer	PBX2P1
chr3	144165000	chr3	194585000	4.68E-03	promoter	super_enhancer	RP11-42F12.1
chr3	146135000	chr3	170735000	8.67E-03	promoter	super_enhancer	RP11-758I14.3
chr3	146215000	chr3	170745000	1.17E-02	promoter	super_enhancer	PLSCR2
chr3	147885000	chr3	170755000	9.26E-03	promoter	super_enhancer	HNRNPA1P20
chr3	148805000	chr3	149395000	2.32E-02	promoter	super_enhancer	HLTF
chr3	148805000	chr3	160675000	1.82E-02	promoter	super_enhancer	HLTF
chr3	148805000	chr3	170725000	9.17E-03	promoter	super_enhancer	HLTF
chr3	148805000	chr3	172145000	1.19E-02	promoter	super_enhancer	HLTF
chr3	148805000	chr3	172165000	1.73E-02	promoter	super_enhancer	HLTF
chr3	148805000	chr3	172745000	9.68E-03	promoter	super_enhancer	HLTF

chr3	149075000	chr3	172155000	1.31E-02	promoter	super_enhancer	RP11-2G17.1
chr3	149095000	chr3	149375000	6.67E-03	promoter	super_enhancer	TM4SF1-AS1
chr3	149105000	chr3	172145000	1.19E-02	promoter	super_enhancer	RP11-278L15.2
chr3	149375000	chr3	169685000	1.50E-02	super_enhancer	promoter	RP11-379K17.4
chr3	149385000	chr3	149485000	3.77E-02	super_enhancer	promoter	AC069216.1
chr3	149385000	chr3	156695000	2.27E-02	super_enhancer	promoter	KLF3P2
chr3	149395000	chr3	161045000	2.53E-02	super_enhancer	promoter	EEF1GP4
chr3	149395000	chr3	170075000	1.84E-02	super_enhancer	promoter	SKIL
chr3	149395000	chr3	197235000	8.07E-03	super_enhancer	promoter	RP13-616I3.1
chr3	149485000	chr3	185725000	8.09E-03	promoter	super_enhancer	AC069216.1
chr3	149575000	chr3	155755000	3.90E-02	super_enhancer	promoter	KCNAB1
chr3	149575000	chr3	156165000	3.35E-02	super_enhancer	promoter	KCNAB1-AS1
chr3	149575000	chr3	171945000	1.39E-02	super_enhancer	promoter	RP11-423E7.1
chr3	149585000	chr3	155755000	4.12E-02	super_enhancer	promoter	KCNAB1
chr3	149955000	chr3	159765000	2.99E-02	promoter	super_enhancer	LINC01213
chr3	150005000	chr3	170725000	9.70E-03	promoter	super_enhancer	LINC01214
chr3	150005000	chr3	172145000	1.26E-02	promoter	super_enhancer	LINC01214
chr3	150005000	chr3	194575000	7.37E-03	promoter	super_enhancer	LINC01214
chr3	151195000	chr3	197505000	6.77E-03	promoter	super_enhancer	MRPL42P6
chr3	151355000	chr3	197495000	5.81E-03	promoter	super_enhancer	RP11-454C18.1
chr3	152785000	chr3	197515000	6.87E-03	promoter	super_enhancer	HMGN2P13
chr3	154375000	chr3	171805000	1.41E-02	promoter	super_enhancer	DYNLL1P5
chr3	154375000	chr3	172175000	1.51E-02	promoter	super_enhancer	DYNLL1P5
chr3	154375000	chr3	184325000	1.02E-02	promoter	super_enhancer	DYNLL1P5

chr3	155525000	chr3	172175000	1.97E-02	promoter	super_enhancer	C3orf33
chr3	155525000	chr3	184325000	1.32E-02	promoter	super_enhancer	C3orf33
chr3	155525000	chr3	195885000	8.03E-03	promoter	super_enhancer	C3orf33
chr3	155705000	chr3	160665000	2.35E-02	promoter	super_enhancer	SETP14
chr3	155705000	chr3	177355000	1.06E-02	promoter	super_enhancer	SETP14
chr3	155755000	chr3	194325000	1.11E-02	promoter	super_enhancer	KCNAB1
chr3	155895000	chr3	194325000	9.43E-03	promoter	super_enhancer	RP11-118D22.3
chr3	156535000	chr3	192895000	6.36E-03	promoter	super_enhancer	LINC00886
chr3	157155000	chr3	195915000	8.53E-03	promoter	super_enhancer	PTX3
chr3	157825000	chr3	171825000	1.72E-02	promoter	super_enhancer	SHOX2
chr3	157825000	chr3	172135000	2.11E-02	promoter	super_enhancer	SHOX2
chr3	157825000	chr3	184315000	1.73E-02	promoter	super_enhancer	SHOX2
chr3	158055000	chr3	197485000	5.36E-03	promoter	super_enhancer	RP11-113A11.3
chr3	160205000	chr3	196345000	9.01E-03	promoter	super_enhancer	TRIM59
chr3	160665000	chr3	180585000	4.96E-03	super_enhancer	promoter	FXR1
chr3	160665000	chr3	196015000	4.74E-03	super_enhancer	promoter	PCYT1A
chr3	160675000	chr3	183795000	1.26E-02	super_enhancer	promoter	RP11-778D9.9
chr3	164915000	chr3	170745000	2.41E-02	promoter	super_enhancer	SLITRK3
chr3	164915000	chr3	172165000	2.26E-02	promoter	super_enhancer	SLITRK3
chr3	167815000	chr3	194565000	6.70E-03	promoter	super_enhancer	GOLIM4
chr3	168645000	chr3	194325000	1.13E-02	promoter	super_enhancer	RP11-368I23.3
chr3	169685000	chr3	185725000	1.42E-02	promoter	super_enhancer	RP11-379K17.4
chr3	169775000	chr3	197485000	8.51E-03	promoter	super_enhancer	RNU4-38P
chr3	169805000	chr3	195915000	1.35E-02	promoter	super_enhancer	RP11-379K17.12

chr3	170715000	chr3	174095000	4.30E-02	super_enhancer	promoter	RP11-393N4.2
chr3	170715000	chr3	177535000	1.70E-02	super_enhancer	promoter	RP11-91K9.1
chr3	170715000	chr3	194255000	9.08E-03	super_enhancer	promoter	RNU6-1101P
chr3	170735000	chr3	183415000	2.15E-02	super_enhancer	promoter	YEATS2
chr3	170745000	chr3	171185000	4.41E-02	super_enhancer	promoter	RP11-185E8.1
chr3	170745000	chr3	183605000	1.67E-02	super_enhancer	promoter	MIR4448
chr3	170825000	chr3	172155000	2.23E-02	promoter	super_enhancer	MIR569
chr3	171795000	chr3	183605000	1.71E-02	super_enhancer	promoter	MIR4448
chr3	171795000	chr3	194255000	1.25E-02	super_enhancer	promoter	RNU6-1101P
chr3	171805000	chr3	194855000	1.11E-02	super_enhancer	promoter	MIR3137
chr3	171815000	chr3	176845000	1.64E-02	super_enhancer	promoter	RNU6-681P
chr3	171815000	chr3	176915000	1.75E-02	super_enhancer	promoter	TBL1XR1
chr3	171815000	chr3	195425000	9.59E-03	super_enhancer	promoter	MIR570
chr3	171945000	chr3	184325000	2.30E-02	promoter	super_enhancer	RP11-423E7.1
chr3	171955000	chr3	177355000	3.14E-02	promoter	super_enhancer	RN7SL141P
chr3	171955000	chr3	184325000	2.21E-02	promoter	super_enhancer	RN7SL141P
chr3	171955000	chr3	194315000	1.31E-02	promoter	super_enhancer	RN7SL141P
chr3	171955000	chr3	194565000	1.18E-02	promoter	super_enhancer	RN7SL141P
chr3	172135000	chr3	175115000	4.72E-02	super_enhancer	promoter	RNU6-1233P
chr3	172135000	chr3	193345000	1.12E-02	super_enhancer	promoter	OPA1-AS1
chr3	172135000	chr3	195415000	1.16E-02	super_enhancer	promoter	AC233280.3
chr3	172145000	chr3	195425000	1.38E-02	super_enhancer	promoter	MIR570
chr3	172155000	chr3	178985000	2.89E-02	super_enhancer	promoter	KCNMB3
chr3	172155000	chr3	182705000	2.56E-04	super_enhancer	promoter	DCUN1D1

chr3	172165000	chr3	176845000	3.64E-02	super_enhancer	promoter	RNU6-681P
chr3	172165000	chr3	178985000	3.39E-02	super_enhancer	promoter	KCNMB3
chr3	172165000	chr3	182835000	2.78E-02	super_enhancer	promoter	MCCC1
chr3	172165000	chr3	183415000	3.19E-02	super_enhancer	promoter	YEATS2
chr3	172165000	chr3	195425000	1.99E-02	super_enhancer	promoter	MIR570
chr3	172175000	chr3	183795000	2.63E-02	super_enhancer	promoter	RP11-778D9.9
chr3	172175000	chr3	196045000	1.36E-02	super_enhancer	promoter	RP11-447L10.1
chr3	172185000	chr3	196475000	1.03E-02	super_enhancer	promoter	RNU4-89P
chr3	172545000	chr3	195915000	1.23E-02	promoter	super_enhancer	AC108667.1
chr3	172745000	chr3	176845000	2.45E-02	super_enhancer	promoter	RNU6-681P
chr3	172745000	chr3	187955000	1.36E-02	super_enhancer	promoter	AC117507.1
chr3	174155000	chr3	177355000	3.66E-02	promoter	super_enhancer	NAALADL2
chr3	175485000	chr3	184315000	2.59E-02	promoter	super_enhancer	RNU6-1317P
chr3	175485000	chr3	194585000	1.09E-02	promoter	super_enhancer	RNU6-1317P
chr3	176755000	chr3	197495000	6.63E-03	promoter	super_enhancer	TBL1XR1-AS1
chr3	176845000	chr3	195875000	1.23E-02	promoter	super_enhancer	RNU6-681P
chr3	177345000	chr3	182835000	3.13E-02	super_enhancer	promoter	MCCC1
chr3	177345000	chr3	184965000	1.75E-02	super_enhancer	promoter	EIF2S2P2
chr3	177345000	chr3	191275000	1.11E-02	super_enhancer	promoter	RP11-197K6.1
chr3	177355000	chr3	188665000	1.65E-02	super_enhancer	promoter	TPRG1-AS1
chr3	177355000	chr3	196045000	1.32E-02	super_enhancer	promoter	RP11-447L10.1
chr3	178985000	chr3	196335000	1.70E-02	promoter	super_enhancer	KCNMB3
chr3	179065000	chr3	185735000	2.84E-02	promoter	super_enhancer	MFN1
chr3	179065000	chr3	195885000	1.33E-02	promoter	super_enhancer	MFN1

chr3	179615000	chr3	184325000	4.24E-04	promoter	super_enhancer	PEX5L-AS2
chr3	179615000	chr3	194305000	1.22E-02	promoter	super_enhancer	PEX5L-AS2
chr3	179755000	chr3	192895000	1.31E-02	promoter	super_enhancer	PEX5L
chr3	179755000	chr3	195885000	1.18E-02	promoter	super_enhancer	PEX5L
chr3	179755000	chr3	196325000	1.48E-02	promoter	super_enhancer	PEX5L
chr3	180585000	chr3	194315000	1.11E-02	promoter	super_enhancer	FXR1
chr3	181225000	chr3	195915000	1.68E-02	promoter	super_enhancer	RPL7AP25
chr3	181685000	chr3	196325000	1.53E-02	promoter	super_enhancer	RN7SKP265
chr3	182705000	chr3	197505000	1.62E-02	promoter	super_enhancer	DCUN1D1
chr3	183605000	chr3	194325000	2.08E-02	promoter	super_enhancer	MIR4448
chr3	183795000	chr3	194135000	2.13E-02	promoter	super_enhancer	RP11-778D9.9
chr3	183795000	chr3	195895000	2.73E-02	promoter	super_enhancer	RP11-778D9.9
chr3	183795000	chr3	197515000	2.93E-04	promoter	super_enhancer	RP11-778D9.9
chr3	183815000	chr3	195885000	2.25E-02	promoter	super_enhancer	HTR3E
chr3	184275000	chr3	194325000	3.25E-02	promoter	super_enhancer	EIF2B5-AS1
chr3	184275000	chr3	195915000	2.79E-02	promoter	super_enhancer	EIF2B5-AS1
chr3	184275000	chr3	196345000	2.55E-02	promoter	super_enhancer	EIF2B5-AS1
chr3	184325000	chr3	194425000	2.64E-02	super_enhancer	promoter	AC106706.1
chr3	185725000	chr3	186285000	4.20E-03	super_enhancer	promoter	DNAJB11
chr3	185735000	chr3	186415000	2.70E-03	super_enhancer	promoter	RP11-795A2.3
chr3	185735000	chr3	195425000	3.06E-02	super_enhancer	promoter	MIR570
chr3	186195000	chr3	194325000	2.86E-02	promoter	super_enhancer	RP11-48F14.2
chr3	186195000	chr3	195915000	2.37E-02	promoter	super_enhancer	RP11-48F14.2
chr3	186265000	chr3	195865000	2.63E-02	promoter	super_enhancer	CRYGS

chr3	186285000	chr3	194295000	2.59E-02	promoter	super_enhancer	DNAJB11
chr3	186285000	chr3	195895000	2.84E-04	promoter	super_enhancer	DNAJB11
chr3	186525000	chr3	195885000	1.98E-02	promoter	super_enhancer	RFC4
chr3	186575000	chr3	195915000	2.27E-02	promoter	super_enhancer	ADIPOQ-AS1
chr3	187465000	chr3	192895000	2.91E-02	promoter	super_enhancer	BCL6
chr3	187665000	chr3	195915000	2.84E-02	promoter	super_enhancer	RP11-44H4.1
chr3	187695000	chr3	195895000	1.89E-02	promoter	super_enhancer	RP11-132N15.3
chr3	187825000	chr3	192895000	4.22E-02	promoter	super_enhancer	RP11-430L16.1
chr3	187825000	chr3	195865000	3.64E-02	promoter	super_enhancer	RP11-430L16.1
chr3	187955000	chr3	196335000	3.20E-02	promoter	super_enhancer	AC117507.1
chr3	189885000	chr3	197485000	1.48E-02	promoter	super_enhancer	NMNAT1P3
chr3	192875000	chr3	192905000	3.49E-02	promoter	super_enhancer	VEZF1P1
chr3	194135000	chr3	194355000	2.73E-04	super_enhancer	promoter	TMEM44
chr3	194285000	chr3	194305000	2.83E-02	promoter	super_enhancer	RPL23AP93
chr3	194325000	chr3	196045000	2.69E-02	super_enhancer	promoter	RP11-447L10.1
chr3	194575000	chr3	195415000	1.18E-02	super_enhancer	promoter	AC233280.3
chr3	194585000	chr3	194815000	2.91E-02	super_enhancer	promoter	XXYLT1-AS1
chr3	195865000	chr3	197355000	9.82E-03	super_enhancer	promoter	AC024560.3
chr3	195885000	chr3	196125000	2.63E-02	super_enhancer	promoter	RN7SL434P
chr3	195885000	chr3	196725000	2.15E-02	super_enhancer	promoter	MFI2-AS1
chr3	196335000	chr3	196715000	1.51E-02	super_enhancer	promoter	AC011322.1
chr3	196715000	chr3	197495000	1.60E-02	promoter	super_enhancer	AC011322.1
chr4	1245000	chr4	10115000	1.47E-02	promoter	super_enhancer	CTBP1
chr4	1385000	chr4	8195000	2.19E-02	promoter	super_enhancer	CRIPAK

chr4	1715000	chr4	7985000	2.23E-02	promoter	super_enhancer	SLBP
chr4	1715000	chr4	10095000	1.52E-02	promoter	super_enhancer	SLBP
chr4	1715000	chr4	40315000	7.85E-03	promoter	super_enhancer	SLBP
chr4	1795000	chr4	168485000	1.59E-03	promoter	super_enhancer	FGFR3
chr4	2265000	chr4	41515000	6.73E-03	promoter	super_enhancer	MXD4
chr4	2325000	chr4	2805000	2.92E-02	promoter	super_enhancer	RP11-478C1.8
chr4	2465000	chr4	7975000	2.50E-02	promoter	super_enhancer	RP11-503N18.4
chr4	3075000	chr4	10095000	1.25E-02	promoter	super_enhancer	HTT
chr4	3075000	chr4	10105000	1.43E-02	promoter	super_enhancer	HTT
chr4	3245000	chr4	39045000	5.66E-03	promoter	super_enhancer	MSANTD1
chr4	3295000	chr4	41365000	5.62E-03	promoter	super_enhancer	RGS12
chr4	3535000	chr4	7975000	1.78E-02	promoter	super_enhancer	LRPAP1
chr4	3635000	chr4	8195000	2.66E-02	promoter	super_enhancer	RP3-368B9.2
chr4	3765000	chr4	7975000	2.56E-02	promoter	super_enhancer	AC141928.1
chr4	4845000	chr4	8195000	4.09E-02	promoter	super_enhancer	LINC01396
chr4	5525000	chr4	7965000	4.29E-02	promoter	super_enhancer	LINC01587
chr4	6675000	chr4	24325000	8.15E-03	promoter	super_enhancer	AC093323.3
chr4	7115000	chr4	7975000	2.93E-03	promoter	super_enhancer	RN7SKP36
chr4	7115000	chr4	8195000	2.80E-02	promoter	super_enhancer	RN7SKP36
chr4	7755000	chr4	7985000	7.66E-03	promoter	super_enhancer	AFAP1-AS1
chr4	7965000	chr4	24565000	9.12E-03	super_enhancer	promoter	RN7SL16P
chr4	7965000	chr4	54525000	3.21E-03	super_enhancer	promoter	RP11-317M11.1
chr4	7985000	chr4	8875000	3.23E-02	super_enhancer	promoter	HMX1
chr4	7985000	chr4	14165000	1.30E-02	super_enhancer	promoter	RP11-669M16.1

chr4	7985000	chr4	25865000	1.06E-02	super_enhancer	promoter	SEL1L3
chr4	8185000	chr4	15705000	1.65E-02	super_enhancer	promoter	BST1
chr4	8185000	chr4	55095000	7.88E-03	super_enhancer	promoter	PDGFRA
chr4	8185000	chr4	188595000	1.90E-03	super_enhancer	promoter	RP11-565A3.2
chr4	8195000	chr4	20255000	9.61E-03	super_enhancer	promoter	SLIT2
chr4	8195000	chr4	78005000	1.82E-03	super_enhancer	promoter	RPL7P17
chr4	8395000	chr4	41365000	7.39E-03	promoter	super_enhancer	RNA5SP152
chr4	9515000	chr4	10095000	2.34E-02	promoter	super_enhancer	OR7E83P
chr4	9565000	chr4	38665000	6.20E-03	promoter	super_enhancer	RP11-1396O13.2
chr4	9565000	chr4	39045000	5.09E-03	promoter	super_enhancer	RP11-1396O13.2
chr4	10075000	chr4	10095000	7.16E-03	promoter	super_enhancer	RP11-448G15.3
chr4	10105000	chr4	55095000	6.82E-03	super_enhancer	promoter	PDGFRA
chr4	10105000	chr4	89505000	1.91E-03	super_enhancer	promoter	RN7SKP244
chr4	10115000	chr4	57155000	4.12E-03	super_enhancer	promoter	RNU6-197P
chr4	10145000	chr4	39045000	1.06E-02	promoter	super_enhancer	RP11-480G3.1
chr4	14165000	chr4	168675000	9.97E-04	promoter	super_enhancer	RP11-669M16.1
chr4	17515000	chr4	41345000	6.26E-03	promoter	super_enhancer	QDPR
chr4	17615000	chr4	38665000	1.09E-02	promoter	super_enhancer	MED28
chr4	17615000	chr4	39035000	7.87E-03	promoter	super_enhancer	MED28
chr4	17615000	chr4	41535000	8.61E-03	promoter	super_enhancer	MED28
chr4	24325000	chr4	158125000	1.52E-03	super_enhancer	promoter	GRIA2
chr4	25235000	chr4	41355000	1.05E-02	promoter	super_enhancer	PI4K2B
chr4	25865000	chr4	40335000	1.83E-02	promoter	super_enhancer	SEL1L3
chr4	25865000	chr4	41515000	1.41E-02	promoter	super_enhancer	SEL1L3

chr4	26165000	chr4	38675000	1.24E-02	promoter	super_enhancer	RBPJ
chr4	26165000	chr4	41355000	7.87E-03	promoter	super_enhancer	RBPJ
chr4	33435000	chr4	41325000	1.82E-02	promoter	super_enhancer	RP11-802H3.2
chr4	33435000	chr4	54065000	5.62E-03	promoter	super_enhancer	RP11-802H3.2
chr4	37455000	chr4	38675000	3.08E-03	promoter	super_enhancer	C4orf19
chr4	37455000	chr4	41515000	4.26E-02	promoter	super_enhancer	C4orf19
chr4	38665000	chr4	141445000	1.89E-03	super_enhancer	promoter	ELMOD2
chr4	38665000	chr4	171065000	2.21E-03	super_enhancer	promoter	RP11-654F3.1
chr4	39035000	chr4	53655000	1.44E-02	super_enhancer	promoter	RP11-752D24.3
chr4	39035000	chr4	83955000	3.60E-03	super_enhancer	promoter	COPS4
chr4	39045000	chr4	42895000	3.90E-02	super_enhancer	promoter	GRXCR1
chr4	39045000	chr4	63375000	6.27E-03	super_enhancer	promoter	HMGN1P11
chr4	39045000	chr4	175205000	1.79E-03	super_enhancer	promoter	FBXO8
chr4	40145000	chr4	40315000	3.61E-02	promoter	super_enhancer	RP11-395I6.1
chr4	40325000	chr4	77135000	7.76E-03	super_enhancer	promoter	SCARB2
chr4	40325000	chr4	100485000	5.58E-03	super_enhancer	promoter	TRMT10A
chr4	40325000	chr4	184245000	3.44E-03	super_enhancer	promoter	CLDN24
chr4	40335000	chr4	87855000	5.60E-03	super_enhancer	promoter	AFF1
chr4	40335000	chr4	88245000	4.83E-03	super_enhancer	promoter	HSD17B13
chr4	40335000	chr4	120255000	2.12E-03	super_enhancer	promoter	KLHL2P1
chr4	40335000	chr4	184775000	2.19E-03	super_enhancer	promoter	STOX2
chr4	40335000	chr4	190945000	1.97E-03	super_enhancer	promoter	DUX4L9
chr4	41325000	chr4	83485000	6.54E-03	super_enhancer	promoter	TMEM150C
chr4	41335000	chr4	76825000	7.76E-03	super_enhancer	promoter	PPEF2

chr4	41345000	chr4	43985000	4.20E-02	super_enhancer	promoter	RP11-663P9.1
chr4	41345000	chr4	121845000	1.75E-03	super_enhancer	promoter	PRDM5
chr4	41355000	chr4	171665000	1.30E-03	super_enhancer	promoter	RP11-322J23.1
chr4	41365000	chr4	41695000	3.62E-02	super_enhancer	promoter	RP11-227F19.5
chr4	41365000	chr4	56815000	1.94E-02	super_enhancer	promoter	CEP135
chr4	41365000	chr4	57575000	2.28E-02	super_enhancer	promoter	RPL17P20
chr4	41365000	chr4	151345000	1.75E-03	super_enhancer	promoter	RP11-174F8.1
chr4	41495000	chr4	62295000	5.96E-03	super_enhancer	promoter	RP11-798L4.1
chr4	41495000	chr4	83935000	5.12E-03	super_enhancer	promoter	LIN54
chr4	41495000	chr4	161925000	2.63E-03	super_enhancer	promoter	AC106860.1
chr4	41505000	chr4	43985000	4.77E-02	super_enhancer	promoter	RP11-663P9.1
chr4	41505000	chr4	74575000	5.00E-03	super_enhancer	promoter	AC112518.3
chr4	41525000	chr4	44535000	2.32E-02	super_enhancer	promoter	RP11-500G9.1
chr4	41525000	chr4	76945000	3.66E-03	super_enhancer	promoter	CXCL10
chr4	41525000	chr4	83925000	3.77E-03	super_enhancer	promoter	RNU6-615P
chr4	41535000	chr4	47655000	1.99E-02	super_enhancer	promoter	MIR8053
chr4	41535000	chr4	52905000	1.18E-02	super_enhancer	promoter	SGCB
chr4	41535000	chr4	120135000	2.11E-03	super_enhancer	promoter	RP11-455G16.1
chr4	41535000	chr4	144105000	2.12E-03	super_enhancer	promoter	USP38
chr4	41545000	chr4	69085000	5.84E-03	super_enhancer	promoter	TMPRSS11BNL
chr4	41545000	chr4	84445000	4.34E-03	super_enhancer	promoter	FAM175A
chr4	46995000	chr4	83255000	5.30E-03	promoter	super_enhancer	GABRB1
chr4	54065000	chr4	54795000	1.01E-02	super_enhancer	promoter	RP11-89B16.2
chr4	54065000	chr4	54865000	4.31E-02	super_enhancer	promoter	RP11-571I18.4

chr4	54065000	chr4	54925000	3.72E-02	super_enhancer	promoter	RP11-571I18.5
chr4	54065000	chr4	57255000	8.61E-04	super_enhancer	promoter	AASDH
chr4	54075000	chr4	54925000	4.04E-02	super_enhancer	promoter	RP11-571I18.5
chr4	61615000	chr4	168485000	9.22E-04	promoter	super_enhancer	RP11-593F5.2
chr4	74095000	chr4	74905000	1.73E-02	super_enhancer	promoter	CXCL3
chr4	74095000	chr4	82655000	9.08E-03	super_enhancer	promoter	RP11-112L18.1
chr4	74105000	chr4	74575000	3.02E-02	super_enhancer	promoter	AC112518.3
chr4	74105000	chr4	155535000	7.35E-04	super_enhancer	promoter	FGG
chr4	74115000	chr4	74865000	2.58E-02	super_enhancer	promoter	CXCL5
chr4	77135000	chr4	83255000	1.89E-02	promoter	super_enhancer	SCARB2
chr4	78005000	chr4	83265000	1.64E-02	promoter	super_enhancer	RPL7P17
chr4	83255000	chr4	86395000	4.13E-02	super_enhancer	promoter	ARHGAP24
chr4	83255000	chr4	99065000	1.02E-02	super_enhancer	promoter	STPG2
chr4	83255000	chr4	100275000	8.70E-03	super_enhancer	promoter	ADH1C
chr4	83265000	chr4	89085000	1.53E-02	super_enhancer	promoter	RP11-147K6.1
chr4	83265000	chr4	126605000	2.49E-03	super_enhancer	promoter	NUPL1P1
chr4	83265000	chr4	138135000	1.79E-03	super_enhancer	promoter	RP11-138I17.1
chr4	109695000	chr4	168645000	1.94E-03	promoter	super_enhancer	RP11-75N20.2
chr4	111775000	chr4	168585000	1.98E-03	promoter	super_enhancer	AC024198.1
chr4	113485000	chr4	138245000	3.82E-03	promoter	super_enhancer	RP11-402J6.3
chr4	122325000	chr4	168665000	2.05E-03	promoter	super_enhancer	AC093816.1
chr4	131415000	chr4	168555000	3.48E-03	promoter	super_enhancer	PGBD4P4
chr4	131415000	chr4	168585000	3.24E-03	promoter	super_enhancer	PGBD4P4
chr4	131425000	chr4	138245000	7.74E-03	promoter	super_enhancer	GAPDHP56

chr4	131425000	chr4	168535000	2.97E-03	promoter	super_enhancer	GAPDHP56	
chr4	138135000	chr4	168645000	3.47E-03	promoter	super_enhancer	RP11-138I17.1	
chr4	138245000	chr4	144265000	9.09E-03	super_enhancer	promoter	MIR3139	
chr4	138245000	chr4	144345000	1.03E-02	super_enhancer	promoter	RP11-58H15.1	
chr4	138245000	chr4	146425000	7.44E-03	super_enhancer	promoter	SMAD1-AS2	
chr4	138455000	chr4	168615000	3.19E-03	promoter	super_enhancer	PCDH18	
chr4	138455000	chr4	168645000	3.05E-03	promoter	super_enhancer	PCDH18	
chr4	144435000	chr4	168665000	4.03E-03	promoter	super_enhancer	SMARCA5	
chr4	149865000	chr4	168615000	5.68E-03	promoter	super_enhancer	CTB-191D16.1	
chr4	150855000	chr4	168485000	5.96E-03	promoter	super_enhancer	RNU6-1230P	
chr4	155445000	chr4	168635000	6.53E-03	promoter	super_enhancer	RP11-158C21.2	
chr4	155475000	chr4	168525000	7.21E-03	promoter	super_enhancer	RP11-158C21.3	
chr4	156095000	chr4	168545000	1.03E-02	promoter	super_enhancer	AC097467.2	
chr4	156875000	chr4	168665000	8.02E-03	promoter	super_enhancer	CTSO	
chr4	163905000	chr4	168535000	1.78E-02	promoter	super_enhancer	RP11-580J4.1	
chr4	163905000	chr4	168555000	1.94E-02	promoter	super_enhancer	RP11-580J4.1	
chr4	164415000	chr4	168535000	1.68E-02	promoter	super_enhancer	TMA16	
chr4	165305000	chr4	168675000	2.68E-02	promoter	super_enhancer		1-Mar
chr4	165645000	chr4	168615000	3.47E-02	promoter	super_enhancer	RP11-294O2.1	
chr4	165935000	chr4	168485000	3.99E-02	promoter	super_enhancer	RP11-366M4.1	
chr4	166605000	chr4	168635000	4.77E-02	promoter	super_enhancer	LINC01179	
chr4	166975000	chr4	168595000	4.95E-02	promoter	super_enhancer	RNA5SP170	
chr4	168485000	chr4	170955000	9.01E-04	super_enhancer	promoter	MFAP3L	
chr4	168485000	chr4	171065000	3.43E-02	super_enhancer	promoter	RP11-654F3.1	

chr4	168485000	chr4	176495000	1.00E-02	super_enhancer	promoter	ADAM20P2
chr4	168485000	chr4	185655000	6.40E-03	super_enhancer	promoter	CENPU
chr4	168525000	chr4	170955000	8.22E-04	super_enhancer	promoter	MFAP3L
chr4	168525000	chr4	175845000	7.79E-03	super_enhancer	promoter	RP13-577H12.3
chr4	168535000	chr4	184425000	7.88E-03	super_enhancer	promoter	ING2
chr4	168555000	chr4	169755000	1.56E-02	super_enhancer	promoter	RP11-635L1.3
chr4	168555000	chr4	171065000	4.33E-02	super_enhancer	promoter	RP11-654F3.1
chr4	168605000	chr4	170535000	4.56E-02	super_enhancer	promoter	NEK1
chr4	168625000	chr4	184775000	6.27E-03	super_enhancer	promoter	STOX2
chr4	168635000	chr4	190935000	6.11E-03	super_enhancer	promoter	RNA5SP174
chr4	168645000	chr4	186125000	9.77E-03	super_enhancer	promoter	SNX25
chr4	168665000	chr4	170535000	4.35E-02	super_enhancer	promoter	NEK1
chr4	168675000	chr4	171665000	2.77E-02	super_enhancer	promoter	RP11-322J23.1
chr5	385000	chr5	17065000	3.93E-02	super_enhancer	promoter	BASP1
chr5	385000	chr5	55595000	5.62E-03	super_enhancer	promoter	RNU6ATAC2P
chr5	385000	chr5	67455000	5.81E-03	super_enhancer	promoter	RNU6-1232P
chr5	385000	chr5	133735000	6.19E-03	super_enhancer	promoter	RP11-215P8.3
chr5	405000	chr5	12575000	2.93E-02	super_enhancer	promoter	RP11-308B16.2
chr5	405000	chr5	43065000	1.09E-02	super_enhancer	promoter	ZNF131
chr5	405000	chr5	43145000	1.39E-02	super_enhancer	promoter	RP11-447H19.4
chr5	425000	chr5	10355000	3.60E-02	super_enhancer	promoter	CTD-2256P15.2
chr5	425000	chr5	36155000	1.22E-02	super_enhancer	promoter	RNU6-1305P
chr5	815000	chr5	168305000	4.34E-03	promoter	super_enhancer	SPCS2P3
chr5	895000	chr5	12575000	2.70E-02	super_enhancer	promoter	RP11-308B16.2

chr5	895000	chr5	87735000	3.21E-03	super_enhancer	promoter	CTC-498M16.2
chr5	895000	chr5	169455000	3.74E-03	super_enhancer	promoter	MIR378E
chr5	895000	chr5	172085000	3.43E-03	super_enhancer	promoter	CTB-79E8.3
chr5	1295000	chr5	1515000	2.69E-02	promoter	super_enhancer	TERT
chr5	1295000	chr5	14195000	4.28E-02	promoter	super_enhancer	TERT
chr5	1295000	chr5	35875000	2.10E-02	promoter	super_enhancer	TERT
chr5	1345000	chr5	131535000	5.38E-03	super_enhancer	promoter	AC063976.7
chr5	1345000	chr5	139025000	6.69E-03	super_enhancer	promoter	CTD-3224K15.3
chr5	1345000	chr5	180415000	5.20E-03	super_enhancer	promoter	BTNL3
chr5	1515000	chr5	22855000	2.69E-02	super_enhancer	promoter	CDH12
chr5	1515000	chr5	38765000	1.74E-02	super_enhancer	promoter	RP11-122C5.2
chr5	1515000	chr5	133735000	6.06E-03	super_enhancer	promoter	RP11-215P8.3
chr5	1535000	chr5	40835000	9.93E-03	super_enhancer	promoter	RPL37
chr5	1535000	chr5	66675000	5.28E-03	super_enhancer	promoter	RP11-434D9.1
chr5	1535000	chr5	96785000	3.06E-03	super_enhancer	promoter	RP11-155G15.3
chr5	1545000	chr5	1615000	1.60E-02	super_enhancer	promoter	CTD-2012J19.1
chr5	1545000	chr5	12795000	4.02E-02	super_enhancer	promoter	RP11-419C19.3
chr5	1545000	chr5	34125000	1.29E-02	super_enhancer	promoter	C1QTNF3-AMACR
chr5	1545000	chr5	41895000	1.02E-02	super_enhancer	promoter	CTD-2062O1.1
chr5	1545000	chr5	41925000	7.32E-03	super_enhancer	promoter	FBXO4
chr5	1545000	chr5	81575000	4.01E-03	super_enhancer	promoter	RPS23
chr5	1545000	chr5	92025000	4.87E-03	super_enhancer	promoter	CTC-529L17.2
chr5	1595000	chr5	140315000	3.61E-03	promoter	super_enhancer	SDHAP3
chr5	1635000	chr5	14265000	4.13E-02	promoter	super_enhancer	RP11-43F13.1

chr5	2965000	chr5	141855000	4.97E-03	promoter	super_enhancer	RP11-35O7.1
chr5	3505000	chr5	14275000	3.92E-02	promoter	super_enhancer	LINC01017
chr5	3505000	chr5	96095000	4.44E-03	promoter	super_enhancer	LINC01017
chr5	3505000	chr5	115935000	4.98E-03	promoter	super_enhancer	LINC01017
chr5	3505000	chr5	148975000	5.11E-03	promoter	super_enhancer	LINC01017
chr5	4135000	chr5	35875000	2.11E-02	promoter	super_enhancer	CTD-2008N3.1
chr5	5035000	chr5	135225000	6.32E-03	promoter	super_enhancer	CTD-2247C11.1
chr5	5125000	chr5	6705000	4.74E-02	promoter	super_enhancer	RN7SKP73
chr5	6705000	chr5	11905000	1.92E-03	super_enhancer	promoter	CTNND2
chr5	6705000	chr5	43485000	1.38E-02	super_enhancer	promoter	C5orf28
chr5	6705000	chr5	172385000	4.61E-03	super_enhancer	promoter	RPL26L1
chr5	6705000	chr5	176785000	4.48E-03	super_enhancer	promoter	RGS14
chr5	6715000	chr5	31745000	2.88E-02	super_enhancer	promoter	RP11-5N11.5
chr5	6715000	chr5	38405000	2.03E-02	super_enhancer	promoter	EGFLAM-AS2
chr5	6715000	chr5	68775000	8.78E-03	super_enhancer	promoter	RN7SL616P
chr5	6715000	chr5	72795000	7.27E-03	super_enhancer	promoter	RP11-79P5.9
chr5	6715000	chr5	73125000	6.08E-03	super_enhancer	promoter	RNU7-196P
chr5	6715000	chr5	94315000	5.01E-03	super_enhancer	promoter	CTC-558O19.1
chr5	6715000	chr5	136935000	7.46E-03	super_enhancer	promoter	SPOCK1
chr5	6725000	chr5	17065000	4.93E-02	super_enhancer	promoter	BASP1
chr5	6725000	chr5	32445000	2.78E-02	super_enhancer	promoter	ZFR
chr5	6725000	chr5	33935000	1.99E-02	super_enhancer	promoter	RXFP3
chr5	6725000	chr5	52285000	6.74E-03	super_enhancer	promoter	ITGA2
chr5	6725000	chr5	78825000	5.83E-03	super_enhancer	promoter	RP11-342F17.2

chr5	6725000	chr5	79315000	6.12E-03	super_enhancer	promoter	RP11-168A11.4
chr5	6725000	chr5	97015000	3.00E-03	super_enhancer	promoter	RP11-72K17.2
chr5	6795000	chr5	38465000	2.42E-02	promoter	super_enhancer	RP11-332J15.1
chr5	6795000	chr5	136015000	5.21E-03	promoter	super_enhancer	RP11-332J15.1
chr5	7325000	chr5	142325000	5.77E-03	promoter	super_enhancer	RP11-404K5.4
chr5	7345000	chr5	91295000	5.03E-03	promoter	super_enhancer	CTD-2296D1.1
chr5	7365000	chr5	38465000	1.57E-02	promoter	super_enhancer	CTD-2296D1.3
chr5	7365000	chr5	172735000	4.46E-03	promoter	super_enhancer	CTD-2296D1.3
chr5	7395000	chr5	42985000	8.53E-03	promoter	super_enhancer	ADCY2
chr5	8525000	chr5	35875000	2.04E-02	promoter	super_enhancer	RP11-417J1.1
chr5	10205000	chr5	115935000	4.56E-03	promoter	super_enhancer	CTD-2199O4.6
chr5	10355000	chr5	42995000	1.99E-02	promoter	super_enhancer	CTD-2256P15.2
chr5	10355000	chr5	136015000	4.97E-03	promoter	super_enhancer	CTD-2256P15.2
chr5	10545000	chr5	36155000	2.61E-02	super_enhancer	promoter	RNU6-1305P
chr5	10545000	chr5	43515000	1.90E-02	super_enhancer	promoter	C5orf34
chr5	10545000	chr5	57195000	8.06E-03	super_enhancer	promoter	RP11-478P10.1
chr5	10545000	chr5	79785000	5.80E-03	super_enhancer	promoter	CTD-2015H6.3
chr5	10545000	chr5	96425000	4.71E-03	super_enhancer	promoter	CTD-2215E18.1
chr5	10545000	chr5	179285000	5.51E-03	super_enhancer	promoter	CTC-241N9.1
chr5	11905000	chr5	43035000	1.30E-02	promoter	super_enhancer	CTNND2
chr5	11905000	chr5	136035000	4.34E-03	promoter	super_enhancer	CTNND2
chr5	12795000	chr5	132465000	5.54E-03	promoter	super_enhancer	RP11-419C19.3
chr5	12795000	chr5	150645000	5.02E-03	promoter	super_enhancer	RP11-419C19.3
chr5	13175000	chr5	172735000	5.85E-03	promoter	super_enhancer	CTC-305H11.1

chr5	13945000	chr5	38855000	1.90E-02	promoter	super_enhancer	DNAH5
chr5	14035000	chr5	29515000	3.32E-02	super_enhancer	promoter	CTD-2345H1.1
chr5	14035000	chr5	32235000	2.80E-02	super_enhancer	promoter	RNU6-1079P
chr5	14035000	chr5	134585000	5.94E-03	super_enhancer	promoter	C5orf66-AS2
chr5	14175000	chr5	22855000	4.03E-02	super_enhancer	promoter	CDH12
chr5	14175000	chr5	34045000	2.78E-02	super_enhancer	promoter	C1QTNF3
chr5	14175000	chr5	43315000	1.42E-02	super_enhancer	promoter	HMGCS1
chr5	14185000	chr5	43065000	1.64E-02	super_enhancer	promoter	ZNF131
chr5	14185000	chr5	121415000	5.43E-03	super_enhancer	promoter	LOX
chr5	14195000	chr5	32445000	3.42E-02	super_enhancer	promoter	ZFR
chr5	14195000	chr5	41925000	1.10E-02	super_enhancer	promoter	FBXO4
chr5	14195000	chr5	61875000	5.05E-03	super_enhancer	promoter	LRRC70
chr5	14195000	chr5	78805000	6.63E-03	super_enhancer	promoter	RP11-342F17.1
chr5	14195000	chr5	108065000	3.89E-03	super_enhancer	promoter	LINC01023
chr5	14195000	chr5	139025000	6.14E-03	super_enhancer	promoter	CTD-3224K15.3
chr5	14245000	chr5	43145000	2.22E-02	super_enhancer	promoter	RP11-447H19.4
chr5	14245000	chr5	74225000	8.48E-03	super_enhancer	promoter	CTD-2503O16.3
chr5	14245000	chr5	81575000	5.69E-03	super_enhancer	promoter	RPS23
chr5	14265000	chr5	32395000	3.21E-02	super_enhancer	promoter	MIR579
chr5	14265000	chr5	54605000	5.79E-03	super_enhancer	promoter	DHX29
chr5	14265000	chr5	94775000	5.32E-03	super_enhancer	promoter	CTD-2538A21.1
chr5	14265000	chr5	121415000	4.87E-03	super_enhancer	promoter	LOX
chr5	14265000	chr5	128795000	4.33E-03	super_enhancer	promoter	ADAMTS19
chr5	14265000	chr5	129865000	1.03E-05	super_enhancer	promoter	ARL2BPP4

chr5	14265000	chr5	175775000	4.60E-03	super_enhancer	promoter	RP11-843P14.2
chr5	14275000	chr5	20305000	4.74E-02	super_enhancer	promoter	RP11-420O16.1
chr5	14275000	chr5	117115000	3.48E-03	super_enhancer	promoter	RPL7L1P4
chr5	14275000	chr5	135265000	4.16E-03	super_enhancer	promoter	FBXL21
chr5	15935000	chr5	150465000	4.98E-03	promoter	super_enhancer	MIR887
chr5	17065000	chr5	42995000	2.36E-02	promoter	super_enhancer	BASP1
chr5	17065000	chr5	132475000	4.17E-03	promoter	super_enhancer	BASP1
chr5	17065000	chr5	136035000	4.85E-03	promoter	super_enhancer	BASP1
chr5	17345000	chr5	96515000	2.88E-03	promoter	super_enhancer	RN7SKP133
chr5	17345000	chr5	151055000	5.43E-03	promoter	super_enhancer	RN7SKP133
chr5	17355000	chr5	38855000	2.03E-02	promoter	super_enhancer	FTH1P10
chr5	17355000	chr5	168315000	4.06E-03	promoter	super_enhancer	FTH1P10
chr5	17525000	chr5	154785000	2.31E-03	promoter	super_enhancer	RP11-321E2.8
chr5	17605000	chr5	136015000	5.04E-03	promoter	super_enhancer	RP11-432M8.8
chr5	17605000	chr5	142355000	5.75E-03	promoter	super_enhancer	RP11-432M8.8
chr5	17605000	chr5	172415000	5.94E-03	promoter	super_enhancer	RP11-432M8.8
chr5	17605000	chr5	180335000	3.37E-03	promoter	super_enhancer	RP11-432M8.8
chr5	17635000	chr5	38465000	2.71E-02	promoter	super_enhancer	RP11-432M8.13
chr5	17635000	chr5	141845000	5.03E-03	promoter	super_enhancer	RP11-432M8.13
chr5	17635000	chr5	142355000	4.57E-03	promoter	super_enhancer	RP11-432M8.13
chr5	17655000	chr5	91325000	5.24E-03	promoter	super_enhancer	RP11-432M8.18
chr5	17655000	chr5	168325000	5.22E-03	promoter	super_enhancer	RP11-432M8.18
chr5	17655000	chr5	172445000	4.65E-03	promoter	super_enhancer	RP11-432M8.18
chr5	17685000	chr5	173455000	4.99E-03	promoter	super_enhancer	RP11-454P21.1

chr5	18895000	chr5	65875000	7.13E-03	promoter	super_enhancer	CTD-2151L9.2
chr5	18895000	chr5	65885000	8.20E-03	promoter	super_enhancer	CTD-2151L9.2
chr5	21615000	chr5	65875000	7.44E-03	promoter	super_enhancer	RP11-804N13.1
chr5	21615000	chr5	172715000	4.45E-03	promoter	super_enhancer	RP11-804N13.1
chr5	22855000	chr5	53405000	6.02E-03	promoter	super_enhancer	CDH12
chr5	23305000	chr5	180335000	3.00E-03	promoter	super_enhancer	CTD-2272G21.2
chr5	24355000	chr5	150485000	3.43E-03	promoter	super_enhancer	CTD-2074D8.1
chr5	25715000	chr5	42995000	2.23E-02	promoter	super_enhancer	CTD-2026A21.1
chr5	28625000	chr5	42985000	1.93E-02	promoter	super_enhancer	RNU6-909P
chr5	29105000	chr5	91295000	6.74E-03	promoter	super_enhancer	RP11-42L13.2
chr5	29515000	chr5	53355000	1.34E-02	promoter	super_enhancer	CTD-2345H1.1
chr5	31745000	chr5	115925000	5.73E-03	promoter	super_enhancer	RP11-5N11.5
chr5	32235000	chr5	141835000	5.77E-03	promoter	super_enhancer	RNU6-1079P
chr5	32395000	chr5	42995000	1.06E-03	promoter	super_enhancer	MIR579
chr5	32395000	chr5	143405000	5.32E-03	promoter	super_enhancer	MIR579
chr5	32395000	chr5	172445000	4.84E-03	promoter	super_enhancer	MIR579
chr5	32445000	chr5	65875000	1.33E-02	promoter	super_enhancer	ZFR
chr5	32445000	chr5	135995000	4.51E-03	promoter	super_enhancer	ZFR
chr5	32445000	chr5	173465000	5.09E-03	promoter	super_enhancer	ZFR
chr5	33935000	chr5	42945000	3.65E-02	promoter	super_enhancer	RXFP3
chr5	33935000	chr5	142825000	4.39E-03	promoter	super_enhancer	RXFP3
chr5	34045000	chr5	148965000	5.11E-03	promoter	super_enhancer	C1QTNF3
chr5	34655000	chr5	53425000	1.39E-02	promoter	super_enhancer	RAI14
chr5	34915000	chr5	42945000	4.56E-02	promoter	super_enhancer	BRIX1

chr5	35875000	chr5	45575000	2.41E-02	super_enhancer	promoter	RP11-484L7.2
chr5	35875000	chr5	177615000	5.24E-03	super_enhancer	promoter	GMCL1P1
chr5	35885000	chr5	110405000	3.79E-03	super_enhancer	promoter	TSLP
chr5	36155000	chr5	151065000	4.70E-03	promoter	super_enhancer	RNU6-1305P
chr5	36605000	chr5	42985000	4.07E-02	promoter	super_enhancer	SLC1A3
chr5	36725000	chr5	43035000	8.82E-04	promoter	super_enhancer	CTD-2353F22.1
chr5	36725000	chr5	168305000	4.38E-03	promoter	super_enhancer	CTD-2353F22.1
chr5	36885000	chr5	91315000	8.31E-03	promoter	super_enhancer	KRT18P31
chr5	36885000	chr5	96535000	6.15E-03	promoter	super_enhancer	KRT18P31
chr5	37795000	chr5	65875000	1.53E-02	promoter	super_enhancer	AC008869.1
chr5	37795000	chr5	142325000	5.61E-03	promoter	super_enhancer	AC008869.1
chr5	38025000	chr5	172855000	6.58E-03	promoter	super_enhancer	CTD-2116N24.1
chr5	38405000	chr5	53395000	1.14E-02	promoter	super_enhancer	EGFLAM-AS2
chr5	38445000	chr5	50725000	1.43E-02	super_enhancer	promoter	CTD-2335O3.2
chr5	38465000	chr5	44715000	4.68E-02	super_enhancer	promoter	RN7SL383P
chr5	38835000	chr5	63985000	1.72E-02	super_enhancer	promoter	FAM159B
chr5	38835000	chr5	71805000	1.19E-02	super_enhancer	promoter	ZNF366
chr5	38855000	chr5	140305000	4.88E-03	super_enhancer	promoter	PCDHAC1
chr5	39075000	chr5	151095000	2.44E-03	promoter	super_enhancer	RICTOR
chr5	39075000	chr5	168305000	2.84E-03	promoter	super_enhancer	RICTOR
chr5	39085000	chr5	67485000	1.25E-02	super_enhancer	promoter	RP11-404L6.2
chr5	39085000	chr5	95385000	7.74E-03	super_enhancer	promoter	RP11-254I22.1
chr5	39085000	chr5	134915000	5.55E-03	super_enhancer	promoter	CXCL14
chr5	39095000	chr5	45035000	2.07E-02	super_enhancer	promoter	RP11-357F12.1

chr5	39095000	chr5	59925000	1.13E-02	super_enhancer	promoter	CAB39P1
chr5	39425000	chr5	142355000	3.12E-03	promoter	super_enhancer	C9
chr5	41895000	chr5	42945000	1.93E-03	promoter	super_enhancer	CTD-2062O1.1
chr5	41925000	chr5	142345000	2.38E-03	promoter	super_enhancer	FBXO4
chr5	42805000	chr5	115945000	3.21E-03	promoter	super_enhancer	CTD-2325A15.5
chr5	42805000	chr5	132075000	2.52E-03	promoter	super_enhancer	CTD-2325A15.5
chr5	42945000	chr5	80255000	8.78E-03	super_enhancer	promoter	RASGRF2
chr5	42945000	chr5	175845000	4.75E-03	super_enhancer	promoter	CLTB
chr5	42985000	chr5	43065000	3.33E-02	super_enhancer	promoter	ZNF131
chr5	42985000	chr5	175965000	3.37E-03	super_enhancer	promoter	RNF44
chr5	42995000	chr5	43065000	2.12E-02	super_enhancer	promoter	ZNF131
chr5	42995000	chr5	72805000	1.62E-02	super_enhancer	promoter	RP11-79P5.10
chr5	42995000	chr5	94775000	9.17E-03	super_enhancer	promoter	CTD-2538A21.1
chr5	43005000	chr5	139025000	5.40E-03	super_enhancer	promoter	CTD-3224K15.3
chr5	43035000	chr5	54455000	1.49E-02	super_enhancer	promoter	GPX8
chr5	43035000	chr5	68365000	1.11E-02	super_enhancer	promoter	SUMO2P4
chr5	43035000	chr5	71805000	1.02E-02	super_enhancer	promoter	ZNF366
chr5	43315000	chr5	65865000	1.21E-02	promoter	super_enhancer	HMGCS1
chr5	43315000	chr5	91375000	6.97E-03	promoter	super_enhancer	HMGCS1
chr5	43485000	chr5	137565000	6.03E-03	promoter	super_enhancer	C5orf28
chr5	43515000	chr5	53385000	1.52E-02	promoter	super_enhancer	C5orf34
chr5	45035000	chr5	96085000	3.38E-03	promoter	super_enhancer	RP11-357F12.1
chr5	50265000	chr5	53345000	4.12E-02	promoter	super_enhancer	CTD-2089N3.3
chr5	50265000	chr5	53395000	3.02E-02	promoter	super_enhancer	CTD-2089N3.3

chr5	52185000	chr5	134245000	2.25E-03	promoter	super_enhancer	B3GNTL1P1
chr5	52285000	chr5	53355000	1.91E-02	promoter	super_enhancer	ITGA2
chr5	52405000	chr5	53355000	7.81E-04	promoter	super_enhancer	CTD-2366F13.1
chr5	52775000	chr5	53385000	3.05E-02	promoter	super_enhancer	FST
chr5	52855000	chr5	53415000	3.79E-02	promoter	super_enhancer	NDUFS4
chr5	52855000	chr5	136005000	1.97E-03	promoter	super_enhancer	NDUFS4
chr5	52905000	chr5	53345000	4.86E-02	promoter	super_enhancer	AC024569.1
chr5	52905000	chr5	53385000	2.80E-02	promoter	super_enhancer	AC024569.1
chr5	53155000	chr5	53405000	1.45E-04	promoter	super_enhancer	ASS1P9
chr5	53155000	chr5	96085000	4.63E-03	promoter	super_enhancer	ASS1P9
chr5	53345000	chr5	80295000	7.31E-03	super_enhancer	promoter	CTD-2193P3.1
chr5	53355000	chr5	68665000	1.18E-02	super_enhancer	promoter	RAD17
chr5	53355000	chr5	97975000	4.05E-03	super_enhancer	promoter	AC022142.1
chr5	53355000	chr5	134455000	2.86E-03	super_enhancer	promoter	CTC-276P9.2
chr5	53365000	chr5	72795000	9.74E-03	super_enhancer	promoter	RP11-79P5.9
chr5	53365000	chr5	126075000	2.73E-03	super_enhancer	promoter	HSPE1P10
chr5	53375000	chr5	75205000	6.30E-03	super_enhancer	promoter	BIN2P2
chr5	53385000	chr5	55595000	4.48E-02	super_enhancer	promoter	RNU6ATAC2P
chr5	53385000	chr5	120395000	1.67E-03	super_enhancer	promoter	CTD-2613O8.1
chr5	53395000	chr5	137405000	1.88E-03	super_enhancer	promoter	RNU6-1148P
chr5	53405000	chr5	68645000	7.73E-03	super_enhancer	promoter	RP11-1113N2.4
chr5	53405000	chr5	169815000	1.64E-03	super_enhancer	promoter	CTD-2270F17.1
chr5	53425000	chr5	140345000	2.32E-03	super_enhancer	promoter	PCDHAC2
chr5	53425000	chr5	163315000	1.10E-03	super_enhancer	promoter	LSM1P2

chr5	54455000	chr5	96095000	4.57E-03	promoter	super_enhancer	GPX8
chr5	54805000	chr5	148965000	1.94E-03	promoter	super_enhancer	MIR5687
chr5	55355000	chr5	65885000	1.56E-02	promoter	super_enhancer	CTD-2227I18.1
chr5	55835000	chr5	135995000	1.42E-03	promoter	super_enhancer	AC022431.3
chr5	57195000	chr5	135235000	3.32E-03	promoter	super_enhancer	RP11-478P10.1
chr5	58825000	chr5	65885000	2.57E-02	promoter	super_enhancer	NDUFB4P2
chr5	63835000	chr5	154755000	2.07E-03	promoter	super_enhancer	RN7SL169P
chr5	64135000	chr5	135145000	2.20E-03	promoter	super_enhancer	AC109465.1
chr5	65435000	chr5	151075000	3.86E-03	promoter	super_enhancer	SREK1
chr5	65505000	chr5	91325000	1.60E-02	promoter	super_enhancer	CTD-2016O11.1
chr5	65505000	chr5	96085000	9.48E-05	promoter	super_enhancer	CTD-2016O11.1
chr5	65505000	chr5	135235000	6.34E-03	promoter	super_enhancer	CTD-2016O11.1
chr5	65865000	chr5	74095000	1.86E-02	super_enhancer	promoter	CTD-2060C23.1
chr5	65865000	chr5	89825000	1.14E-02	super_enhancer	promoter	LYSMD3
chr5	65885000	chr5	72175000	3.36E-02	super_enhancer	promoter	CTD-2339M3.1
chr5	65885000	chr5	75805000	2.95E-02	super_enhancer	promoter	CTD-2275D10.2
chr5	65885000	chr5	115555000	5.88E-03	super_enhancer	promoter	HMGN2P27
chr5	66675000	chr5	174365000	3.47E-03	promoter	super_enhancer	RP11-434D9.1
chr5	67455000	chr5	91295000	1.15E-02	promoter	super_enhancer	RNU6-1232P
chr5	67455000	chr5	91315000	1.29E-02	promoter	super_enhancer	RNU6-1232P
chr5	68365000	chr5	141835000	4.44E-03	promoter	super_enhancer	SUMO2P4
chr5	68485000	chr5	143245000	3.83E-03	promoter	super_enhancer	CTC-351M12.1
chr5	68665000	chr5	95865000	1.13E-02	promoter	super_enhancer	RAD17
chr5	68665000	chr5	142805000	3.26E-03	promoter	super_enhancer	RAD17

chr5	68765000	chr5	74635000	4.92E-02	promoter	super_enhancer	RPS27P14
chr5	68765000	chr5	141835000	6.88E-03	promoter	super_enhancer	RPS27P14
chr5	68765000	chr5	173325000	4.46E-03	promoter	super_enhancer	RPS27P14
chr5	68775000	chr5	141825000	5.56E-03	promoter	super_enhancer	RN7SL616P
chr5	68775000	chr5	172715000	4.61E-03	promoter	super_enhancer	RN7SL616P
chr5	68855000	chr5	150515000	2.35E-03	promoter	super_enhancer	GTF2H2C
chr5	72175000	chr5	132495000	4.29E-03	promoter	super_enhancer	CTD-2339M3.1
chr5	72175000	chr5	139645000	4.59E-03	promoter	super_enhancer	CTD-2339M3.1
chr5	72415000	chr5	134255000	5.97E-03	promoter	super_enhancer	TMEM171
chr5	72745000	chr5	96085000	1.30E-02	promoter	super_enhancer	FOXD1
chr5	72745000	chr5	96545000	1.19E-02	promoter	super_enhancer	FOXD1
chr5	73605000	chr5	74645000	4.99E-02	promoter	super_enhancer	LINC01335
chr5	73935000	chr5	168325000	3.32E-03	promoter	super_enhancer	HEXB
chr5	74095000	chr5	142345000	3.19E-03	promoter	super_enhancer	CTD-2060C23.1
chr5	74095000	chr5	168335000	2.63E-03	promoter	super_enhancer	CTD-2060C23.1
chr5	74225000	chr5	135155000	6.28E-03	promoter	super_enhancer	CTD-2503O16.3
chr5	74635000	chr5	89825000	1.78E-02	super_enhancer	promoter	LYSMD3
chr5	74635000	chr5	92405000	2.06E-02	super_enhancer	promoter	CTD-2091N23.1
chr5	74635000	chr5	94945000	1.70E-02	super_enhancer	promoter	AC008547.1
chr5	74635000	chr5	116355000	3.92E-03	super_enhancer	promoter	RP11-492L8.1
chr5	74645000	chr5	82375000	1.76E-02	super_enhancer	promoter	TMEM167A
chr5	74835000	chr5	135155000	5.18E-03	promoter	super_enhancer	RNU7-175P
chr5	74835000	chr5	172445000	2.99E-03	promoter	super_enhancer	RNU7-175P
chr5	75005000	chr5	134525000	3.91E-03	promoter	super_enhancer	RNU6-680P

chr5	75205000	chr5	142335000	5.42E-03	promoter	super_enhancer	BIN2P2
chr5	75805000	chr5	91335000	1.79E-02	promoter	super_enhancer	CTD-2275D10.2
chr5	75805000	chr5	96525000	1.64E-04	promoter	super_enhancer	CTD-2275D10.2
chr5	75905000	chr5	96495000	1.48E-02	promoter	super_enhancer	CTD-2236F14.1
chr5	76145000	chr5	91325000	2.45E-02	promoter	super_enhancer	S100Z
chr5	76505000	chr5	96065000	1.41E-02	promoter	super_enhancer	PDE8B
chr5	77165000	chr5	132075000	3.91E-03	promoter	super_enhancer	TBCA
chr5	77655000	chr5	168305000	3.14E-03	promoter	super_enhancer	SCAMP1
chr5	78985000	chr5	135155000	7.08E-03	promoter	super_enhancer	CMYA5
chr5	78985000	chr5	180345000	3.49E-03	promoter	super_enhancer	CMYA5
chr5	79615000	chr5	134245000	6.70E-03	promoter	super_enhancer	SPZ1
chr5	79785000	chr5	95865000	1.95E-02	promoter	super_enhancer	CTD-2015H6.3
chr5	79795000	chr5	177495000	4.07E-03	promoter	super_enhancer	CTD-2015H6.2
chr5	81575000	chr5	91335000	1.73E-02	promoter	super_enhancer	RPS23
chr5	81575000	chr5	142335000	5.23E-03	promoter	super_enhancer	RPS23
chr5	81575000	chr5	142365000	4.47E-03	promoter	super_enhancer	RPS23
chr5	82265000	chr5	150125000	2.96E-03	promoter	super_enhancer	ST13P12
chr5	84825000	chr5	140325000	2.27E-03	promoter	super_enhancer	AC026700.1
chr5	87735000	chr5	91325000	4.65E-02	promoter	super_enhancer	CTC-498M16.2
chr5	87795000	chr5	96525000	2.47E-02	promoter	super_enhancer	CTC-498M16.4
chr5	89195000	chr5	151075000	4.67E-03	promoter	super_enhancer	AC113167.1
chr5	89685000	chr5	96085000	3.02E-02	promoter	super_enhancer	CTD-2373J6.1
chr5	89705000	chr5	96495000	2.97E-02	promoter	super_enhancer	CTD-2373J6.2
chr5	89825000	chr5	134555000	7.69E-03	promoter	super_enhancer	LYSMD3

chr5	90675000	chr5	95865000	3.88E-02	promoter	super_enhancer	ARRDC3-AS1
chr5	90675000	chr5	132095000	7.08E-03	promoter	super_enhancer	ARRDC3-AS1
chr5	91295000	chr5	115415000	1.35E-02	super_enhancer	promoter	CTD-2287O16.4
chr5	91305000	chr5	98225000	1.91E-02	super_enhancer	promoter	RNU6-402P
chr5	91315000	chr5	92025000	4.65E-02	super_enhancer	promoter	CTC-529L17.2
chr5	91315000	chr5	101445000	2.48E-02	super_enhancer	promoter	AC106765.1
chr5	91315000	chr5	104435000	1.52E-02	super_enhancer	promoter	RAB9BP1
chr5	91315000	chr5	176695000	3.16E-03	super_enhancer	promoter	PRMT1P1
chr5	91325000	chr5	112665000	1.49E-02	super_enhancer	promoter	AC126917.1
chr5	91325000	chr5	140305000	8.82E-03	super_enhancer	promoter	PCDHAC1
chr5	91335000	chr5	121415000	9.26E-03	super_enhancer	promoter	LOX
chr5	91375000	chr5	136935000	1.02E-02	super_enhancer	promoter	SPOCK1
chr5	91625000	chr5	132475000	5.34E-03	promoter	super_enhancer	RP11-348J24.1
chr5	92025000	chr5	115935000	1.38E-02	promoter	super_enhancer	CTC-529L17.2
chr5	92275000	chr5	151105000	4.19E-03	promoter	super_enhancer	CTC-458G6.2
chr5	92935000	chr5	141835000	9.68E-03	promoter	super_enhancer	RP11-65F13.3
chr5	93905000	chr5	142335000	7.89E-03	promoter	super_enhancer	MTND6P3
chr5	93905000	chr5	154785000	3.71E-03	promoter	super_enhancer	MTND6P3
chr5	94315000	chr5	96075000	1.79E-02	promoter	super_enhancer	CTC-558O19.1
chr5	94775000	chr5	115925000	1.93E-02	promoter	super_enhancer	CTD-2538A21.1
chr5	94945000	chr5	151055000	7.56E-03	promoter	super_enhancer	AC008547.1
chr5	94955000	chr5	132465000	1.29E-02	promoter	super_enhancer	GPR150
chr5	94955000	chr5	134245000	1.18E-02	promoter	super_enhancer	GPR150
chr5	94955000	chr5	136015000	9.40E-03	promoter	super_enhancer	GPR150

chr5	94955000	chr5	168305000	4.82E-03	promoter	super_enhancer	GPR150
chr5	95185000	chr5	132265000	1.09E-02	promoter	super_enhancer	AC008592.8
chr5	95185000	chr5	154795000	6.04E-03	promoter	super_enhancer	AC008592.8
chr5	95385000	chr5	139475000	8.98E-03	promoter	super_enhancer	RP11-254I22.1
chr5	95385000	chr5	142805000	8.98E-03	promoter	super_enhancer	RP11-254I22.1
chr5	95415000	chr5	135145000	8.91E-03	promoter	super_enhancer	MIR583
chr5	95415000	chr5	136035000	8.66E-03	promoter	super_enhancer	MIR583
chr5	95865000	chr5	129865000	1.09E-02	super_enhancer	promoter	ARL2BPP4
chr5	95865000	chr5	131395000	1.62E-02	super_enhancer	promoter	IL3
chr5	96065000	chr5	99405000	3.80E-02	super_enhancer	promoter	CTD-2012B7.1
chr5	96065000	chr5	115525000	1.08E-02	super_enhancer	promoter	RNU6-644P
chr5	96065000	chr5	141705000	8.06E-03	super_enhancer	promoter	AC005592.2
chr5	96075000	chr5	122805000	1.21E-02	super_enhancer	promoter	HMGB3P17
chr5	96075000	chr5	124145000	1.29E-02	super_enhancer	promoter	RP11-436H11.1
chr5	96075000	chr5	133985000	1.24E-02	super_enhancer	promoter	SAR1B
chr5	96075000	chr5	135365000	9.54E-03	super_enhancer	promoter	TGFBI
chr5	96075000	chr5	169455000	3.33E-03	super_enhancer	promoter	MIR378E
chr5	96085000	chr5	122425000	1.14E-02	super_enhancer	promoter	PRDM6
chr5	96085000	chr5	179655000	2.82E-03	super_enhancer	promoter	RP11-252I14.1
chr5	96095000	chr5	125515000	7.56E-03	super_enhancer	promoter	CTC-339D2.1
chr5	96145000	chr5	180345000	3.39E-03	promoter	super_enhancer	ERAP1
chr5	96505000	chr5	98015000	7.51E-04	super_enhancer	promoter	CTC-463N11.1
chr5	96505000	chr5	98265000	1.39E-02	super_enhancer	promoter	CHD1
chr5	96505000	chr5	132155000	8.83E-03	super_enhancer	promoter	AC004775.5

chr5	96515000	chr5	107145000	1.10E-02	super_enhancer	promoter	RN7SKP122
chr5	96525000	chr5	98225000	5.48E-04	super_enhancer	promoter	RNU6-402P
chr5	96525000	chr5	118475000	1.45E-02	super_enhancer	promoter	RNU6-701P
chr5	96525000	chr5	127445000	9.49E-03	super_enhancer	promoter	KDELC1P1
chr5	96525000	chr5	139575000	7.67E-03	super_enhancer	promoter	AC011379.1
chr5	96525000	chr5	167555000	4.40E-03	super_enhancer	promoter	CTB-105N12.2
chr5	96535000	chr5	98265000	1.16E-02	super_enhancer	promoter	CHD1
chr5	96535000	chr5	120535000	1.14E-02	super_enhancer	promoter	CTC-546K23.1
chr5	96545000	chr5	97015000	1.99E-02	super_enhancer	promoter	RP11-72K17.2
chr5	96545000	chr5	103435000	2.40E-02	super_enhancer	promoter	RNU1-140P
chr5	96545000	chr5	121465000	1.06E-02	super_enhancer	promoter	ZNF474
chr5	96545000	chr5	140685000	8.00E-03	super_enhancer	promoter	SLC25A2
chr5	96545000	chr5	176875000	2.94E-03	super_enhancer	promoter	PRR7-AS1
chr5	98015000	chr5	148985000	3.62E-03	promoter	super_enhancer	CTC-463N11.1
chr5	98225000	chr5	134245000	7.37E-03	promoter	super_enhancer	RNU6-402P
chr5	98265000	chr5	132465000	9.72E-03	promoter	super_enhancer	CHD1
chr5	99285000	chr5	136025000	7.28E-03	promoter	super_enhancer	CTD-2160D9.1
chr5	99995000	chr5	150125000	3.77E-03	promoter	super_enhancer	CTC-293G12.1
chr5	101445000	chr5	132495000	9.32E-03	promoter	super_enhancer	AC106765.1
chr5	101445000	chr5	173465000	3.46E-03	promoter	super_enhancer	AC106765.1
chr5	102455000	chr5	139485000	8.84E-03	promoter	super_enhancer	PPIP5K2
chr5	103415000	chr5	174345000	1.70E-03	promoter	super_enhancer	RP11-138J23.1
chr5	104435000	chr5	177495000	2.83E-03	promoter	super_enhancer	RAB9BP1
chr5	108785000	chr5	142355000	4.91E-05	promoter	super_enhancer	CTD-2128F4.1

chr5	108785000	chr5	150495000	8.39E-03	promoter	super_enhancer	CTD-2128F4.1
chr5	109025000	chr5	137575000	1.54E-02	promoter	super_enhancer	CTC-332L22.1
chr5	109025000	chr5	154795000	7.24E-03	promoter	super_enhancer	CTC-332L22.1
chr5	110075000	chr5	135155000	1.33E-02	promoter	super_enhancer	TMEM232
chr5	110405000	chr5	172425000	4.75E-03	promoter	super_enhancer	TSLP
chr5	111755000	chr5	143405000	8.35E-03	promoter	super_enhancer	EPB41L4A
chr5	112665000	chr5	136015000	1.33E-02	promoter	super_enhancer	AC126917.1
chr5	112665000	chr5	143585000	9.54E-03	promoter	super_enhancer	AC126917.1
chr5	112825000	chr5	142835000	1.15E-02	promoter	super_enhancer	MCC
chr5	114425000	chr5	132485000	2.37E-02	promoter	super_enhancer	RP11-438C19.1
chr5	114425000	chr5	139645000	1.71E-02	promoter	super_enhancer	RP11-438C19.1
chr5	114425000	chr5	150635000	1.01E-02	promoter	super_enhancer	RP11-438C19.1
chr5	114485000	chr5	135145000	1.42E-02	promoter	super_enhancer	TRIM36-IT1
chr5	114485000	chr5	143255000	1.03E-02	promoter	super_enhancer	TRIM36-IT1
chr5	114485000	chr5	151085000	9.51E-03	promoter	super_enhancer	TRIM36-IT1
chr5	114625000	chr5	132265000	1.58E-02	promoter	super_enhancer	HMGN1P15
chr5	114625000	chr5	134555000	1.67E-02	promoter	super_enhancer	HMGN1P15
chr5	114625000	chr5	168325000	5.95E-03	promoter	super_enhancer	HMGN1P15
chr5	114825000	chr5	135225000	2.20E-02	promoter	super_enhancer	CTB-161J7.1
chr5	115105000	chr5	132495000	1.40E-02	promoter	super_enhancer	CTC-505O3.1
chr5	115365000	chr5	141845000	1.27E-02	promoter	super_enhancer	AC034236.2
chr5	115415000	chr5	137565000	1.75E-04	promoter	super_enhancer	CTD-2287O16.4
chr5	115415000	chr5	151085000	1.06E-02	promoter	super_enhancer	CTD-2287O16.4
chr5	115415000	chr5	180345000	4.12E-03	promoter	super_enhancer	CTD-2287O16.4

chr5	115925000	chr5	116355000	3.14E-02	super_enhancer	promoter	RP11-492L8.1
chr5	115925000	chr5	125935000	3.13E-02	super_enhancer	promoter	PHAX
chr5	115925000	chr5	137515000	2.18E-02	super_enhancer	promoter	BRD8
chr5	115925000	chr5	145215000	1.39E-02	super_enhancer	promoter	PRELID2
chr5	115935000	chr5	122805000	2.92E-02	super_enhancer	promoter	HMGB3P17
chr5	115935000	chr5	159685000	7.12E-03	super_enhancer	promoter	RP11-34P1.2
chr5	115945000	chr5	117115000	4.04E-02	super_enhancer	promoter	RPL7L1P4
chr5	115945000	chr5	120375000	4.14E-02	super_enhancer	promoter	AC008565.1
chr5	115945000	chr5	125515000	1.91E-02	super_enhancer	promoter	CTC-339D2.1
chr5	115945000	chr5	133735000	1.90E-02	super_enhancer	promoter	RP11-215P8.3
chr5	115945000	chr5	145215000	1.21E-02	super_enhancer	promoter	PRELID2
chr5	118465000	chr5	132255000	2.30E-02	promoter	super_enhancer	RP11-950K24.2
chr5	118465000	chr5	134525000	1.45E-02	promoter	super_enhancer	RP11-950K24.2
chr5	118745000	chr5	142365000	1.64E-02	promoter	super_enhancer	AC035144.1
chr5	118965000	chr5	150475000	9.86E-03	promoter	super_enhancer	FAM170A
chr5	119015000	chr5	139475000	1.02E-02	promoter	super_enhancer	CTC-507E12.1
chr5	119015000	chr5	142365000	9.99E-03	promoter	super_enhancer	CTC-507E12.1
chr5	119995000	chr5	134555000	1.51E-02	promoter	super_enhancer	RP11-565F4.1
chr5	119995000	chr5	174365000	3.86E-03	promoter	super_enhancer	RP11-565F4.1
chr5	120535000	chr5	139655000	1.50E-02	promoter	super_enhancer	CTC-546K23.1
chr5	120535000	chr5	142815000	1.63E-02	promoter	super_enhancer	CTC-546K23.1
chr5	121415000	chr5	132265000	2.32E-02	promoter	super_enhancer	LOX
chr5	121415000	chr5	150645000	1.27E-02	promoter	super_enhancer	LOX
chr5	121815000	chr5	132075000	1.30E-02	promoter	super_enhancer	MGC32805

chr5	121815000	chr5	143595000	7.09E-03	promoter	super_enhancer	MGC32805
chr5	122425000	chr5	132495000	2.12E-02	promoter	super_enhancer	PRDM6
chr5	122425000	chr5	142805000	1.50E-02	promoter	super_enhancer	PRDM6
chr5	122805000	chr5	135245000	2.70E-02	promoter	super_enhancer	HMGB3P17
chr5	122805000	chr5	136025000	1.98E-02	promoter	super_enhancer	HMGB3P17
chr5	122805000	chr5	172735000	7.38E-03	promoter	super_enhancer	HMGB3P17
chr5	122845000	chr5	132495000	2.11E-02	promoter	super_enhancer	CTD-2169H9.1
chr5	123805000	chr5	134555000	2.68E-02	promoter	super_enhancer	CTD-2308B18.2
chr5	123805000	chr5	135225000	3.20E-02	promoter	super_enhancer	CTD-2308B18.2
chr5	123805000	chr5	154765000	1.02E-02	promoter	super_enhancer	CTD-2308B18.2
chr5	124085000	chr5	132455000	2.91E-02	promoter	super_enhancer	ZNF608
chr5	124145000	chr5	172415000	7.49E-03	promoter	super_enhancer	RP11-436H11.1
chr5	124165000	chr5	134555000	2.61E-02	promoter	super_enhancer	RP11-284A20.3
chr5	124165000	chr5	136025000	2.28E-02	promoter	super_enhancer	RP11-284A20.3
chr5	124255000	chr5	132495000	1.81E-02	promoter	super_enhancer	RP11-284A20.1
chr5	125305000	chr5	180325000	3.65E-03	promoter	super_enhancer	RPSAP37
chr5	125515000	chr5	139665000	1.61E-02	promoter	super_enhancer	CTC-339D2.1
chr5	125515000	chr5	143595000	9.96E-03	promoter	super_enhancer	CTC-339D2.1
chr5	125615000	chr5	150465000	1.40E-02	promoter	super_enhancer	RP11-114J13.1
chr5	125615000	chr5	154785000	8.57E-03	promoter	super_enhancer	RP11-114J13.1
chr5	125615000	chr5	172885000	6.98E-03	promoter	super_enhancer	RP11-114J13.1
chr5	125615000	chr5	173325000	5.90E-03	promoter	super_enhancer	RP11-114J13.1
chr5	125695000	chr5	132255000	4.06E-02	promoter	super_enhancer	GRAMD3
chr5	125695000	chr5	142835000	2.18E-02	promoter	super_enhancer	GRAMD3

chr5	125935000	chr5	143585000	1.73E-02	promoter	super_enhancer	PHAX
chr5	125935000	chr5	151075000	1.46E-02	promoter	super_enhancer	PHAX
chr5	125965000	chr5	135245000	4.09E-02	promoter	super_enhancer	RP11-395C3.1
chr5	125965000	chr5	141825000	2.68E-02	promoter	super_enhancer	RP11-395C3.1
chr5	125965000	chr5	141835000	2.87E-02	promoter	super_enhancer	RP11-395C3.1
chr5	125965000	chr5	150145000	1.20E-02	promoter	super_enhancer	RP11-395C3.1
chr5	126075000	chr5	173305000	7.26E-03	promoter	super_enhancer	HSPE1P10
chr5	127445000	chr5	132085000	4.63E-02	promoter	super_enhancer	KDELC1P1
chr5	127445000	chr5	136045000	2.08E-02	promoter	super_enhancer	KDELC1P1
chr5	127445000	chr5	137565000	2.68E-02	promoter	super_enhancer	KDELC1P1
chr5	127445000	chr5	151075000	1.19E-02	promoter	super_enhancer	KDELC1P1
chr5	127445000	chr5	172415000	7.00E-03	promoter	super_enhancer	KDELC1P1
chr5	127995000	chr5	134525000	2.98E-02	promoter	super_enhancer	FBN2
chr5	127995000	chr5	150635000	1.42E-02	promoter	super_enhancer	FBN2
chr5	127995000	chr5	151055000	1.72E-02	promoter	super_enhancer	FBN2
chr5	127995000	chr5	172185000	7.61E-03	promoter	super_enhancer	FBN2
chr5	127995000	chr5	173295000	6.26E-03	promoter	super_enhancer	FBN2
chr5	128795000	chr5	137565000	3.10E-02	promoter	super_enhancer	ADAMTS19
chr5	129475000	chr5	135145000	2.73E-02	promoter	super_enhancer	HSPA8P4
chr5	129865000	chr5	154785000	9.97E-03	promoter	super_enhancer	ARL2BPP4
chr5	130325000	chr5	142345000	2.47E-02	promoter	super_enhancer	RP11-114H7.1
chr5	130325000	chr5	172175000	8.36E-03	promoter	super_enhancer	RP11-114H7.1
chr5	131395000	chr5	132465000	4.30E-02	promoter	super_enhancer	IL3
chr5	131395000	chr5	139485000	4.91E-02	promoter	super_enhancer	IL3

chr5	131395000	chr5	150485000	2.62E-02	promoter	super_enhancer	IL3
chr5	131395000	chr5	151085000	2.67E-02	promoter	super_enhancer	IL3
chr5	131395000	chr5	154765000	1.93E-02	promoter	super_enhancer	IL3
chr5	131395000	chr5	173335000	1.18E-02	promoter	super_enhancer	IL3
chr5	131395000	chr5	174375000	1.13E-02	promoter	super_enhancer	IL3
chr5	131485000	chr5	142805000	2.12E-02	promoter	super_enhancer	AC063976.1
chr5	131485000	chr5	150505000	1.53E-02	promoter	super_enhancer	AC063976.1
chr5	131535000	chr5	137575000	4.53E-02	promoter	super_enhancer	AC063976.7
chr5	131705000	chr5	139645000	3.27E-02	promoter	super_enhancer	SLC22A5
chr5	131705000	chr5	139655000	2.80E-02	promoter	super_enhancer	SLC22A5
chr5	131705000	chr5	142355000	2.53E-02	promoter	super_enhancer	SLC22A5
chr5	131705000	chr5	151065000	1.56E-02	promoter	super_enhancer	SLC22A5
chr5	131745000	chr5	139645000	2.67E-02	promoter	super_enhancer	C5orf56
chr5	131745000	chr5	142815000	2.28E-02	promoter	super_enhancer	C5orf56
chr5	131745000	chr5	150145000	1.01E-02	promoter	super_enhancer	C5orf56
chr5	132075000	chr5	137515000	4.52E-02	super_enhancer	promoter	BRD8
chr5	132075000	chr5	139575000	3.26E-04	super_enhancer	promoter	AC011379.1
chr5	132075000	chr5	176785000	6.72E-03	super_enhancer	promoter	RGS14
chr5	132085000	chr5	135365000	3.83E-03	super_enhancer	promoter	TGFBI
chr5	132085000	chr5	139155000	4.37E-02	super_enhancer	promoter	CTB-35F21.3
chr5	132085000	chr5	140705000	2.54E-02	super_enhancer	promoter	AC005618.6
chr5	132085000	chr5	145625000	1.97E-02	super_enhancer	promoter	RNU7-180P
chr5	132095000	chr5	140535000	3.34E-02	super_enhancer	promoter	PCDHB17P
chr5	132095000	chr5	141545000	2.93E-02	super_enhancer	promoter	CTC-463A16.1

chr5	132095000	chr5	142755000	2.58E-02	super_enhancer	promoter	RP11-278J6.5
chr5	132095000	chr5	148925000	2.13E-02	super_enhancer	promoter	RPL29P14
chr5	132155000	chr5	139475000	2.79E-02	promoter	super_enhancer	AC004775.5
chr5	132155000	chr5	141825000	2.84E-02	promoter	super_enhancer	AC004775.5
chr5	132155000	chr5	141855000	2.33E-02	promoter	super_enhancer	AC004775.5
chr5	132155000	chr5	148915000	1.60E-02	promoter	super_enhancer	AC004775.5
chr5	132155000	chr5	150465000	1.74E-02	promoter	super_enhancer	AC004775.5
chr5	132255000	chr5	133305000	1.87E-03	super_enhancer	promoter	C5orf15
chr5	132255000	chr5	139575000	3.73E-02	super_enhancer	promoter	AC011379.1
chr5	132255000	chr5	149865000	2.08E-02	super_enhancer	promoter	NDST1
chr5	132255000	chr5	154065000	1.99E-02	super_enhancer	promoter	MIR1303
chr5	132255000	chr5	171615000	1.29E-02	super_enhancer	promoter	STK10
chr5	132265000	chr5	158745000	1.32E-02	super_enhancer	promoter	RNU4ATAC2P
chr5	132265000	chr5	170815000	7.50E-03	super_enhancer	promoter	MIR3912
chr5	132325000	chr5	142805000	2.84E-02	promoter	super_enhancer	AC010240.3
chr5	132325000	chr5	151055000	2.21E-02	promoter	super_enhancer	AC010240.3
chr5	132445000	chr5	132805000	3.59E-03	super_enhancer	promoter	AC010608.1
chr5	132455000	chr5	140165000	3.91E-02	super_enhancer	promoter	PCDHA1
chr5	132455000	chr5	140235000	2.71E-02	super_enhancer	promoter	PCDHA10
chr5	132455000	chr5	142755000	2.82E-04	super_enhancer	promoter	RP11-278J6.5
chr5	132455000	chr5	149535000	2.23E-02	super_enhancer	promoter	PDGFRB
chr5	132455000	chr5	159675000	1.19E-02	super_enhancer	promoter	RP11-34P1.1
chr5	132465000	chr5	138615000	9.46E-04	super_enhancer	promoter	AC011404.1
chr5	132465000	chr5	140305000	4.69E-02	super_enhancer	promoter	PCDHAC1

chr5	132465000	chr5	146465000	1.86E-02	super_enhancer	promoter	PPP2R2B
chr5	132465000	chr5	148885000	1.85E-02	super_enhancer	promoter	CTB-89H12.4
chr5	132475000	chr5	138635000	3.56E-02	super_enhancer	promoter	RNA5SP195
chr5	132475000	chr5	175055000	6.42E-03	super_enhancer	promoter	RN7SKP148
chr5	132485000	chr5	134895000	1.93E-02	super_enhancer	promoter	CTC-321K16.1
chr5	132485000	chr5	141945000	3.56E-02	super_enhancer	promoter	RPS12P10
chr5	132485000	chr5	157405000	1.53E-02	super_enhancer	promoter	RNU2-48P
chr5	132485000	chr5	173485000	9.80E-03	super_enhancer	promoter	RP11-619L12.3
chr5	132485000	chr5	180415000	8.18E-03	super_enhancer	promoter	BTNL3
chr5	132495000	chr5	139025000	4.14E-02	super_enhancer	promoter	CTD-3224K15.3
chr5	132805000	chr5	150465000	1.84E-02	promoter	super_enhancer	AC010608.1
chr5	133305000	chr5	139485000	4.02E-02	promoter	super_enhancer	C5orf15
chr5	133305000	chr5	172425000	4.32E-05	promoter	super_enhancer	C5orf15
chr5	133735000	chr5	173335000	9.35E-03	promoter	super_enhancer	RP11-215P8.3
chr5	133735000	chr5	173455000	8.83E-03	promoter	super_enhancer	RP11-215P8.3
chr5	134235000	chr5	149545000	3.10E-02	super_enhancer	promoter	CDX1
chr5	134235000	chr5	175085000	1.14E-02	super_enhancer	promoter	HRH2
chr5	134235000	chr5	180675000	7.52E-03	super_enhancer	promoter	GNB2L1
chr5	134245000	chr5	145485000	2.21E-02	super_enhancer	promoter	PLAC8L1
chr5	134245000	chr5	173485000	9.49E-03	super_enhancer	promoter	RP11-619L12.3
chr5	134255000	chr5	139175000	4.99E-02	super_enhancer	promoter	PSD2
chr5	134255000	chr5	139505000	3.59E-02	super_enhancer	promoter	IGIP
chr5	134265000	chr5	142835000	3.87E-02	promoter	super_enhancer	MTND4P12
chr5	134265000	chr5	150635000	2.04E-02	promoter	super_enhancer	MTND4P12

chr5	134455000	chr5	140315000	3.26E-02	promoter	super_enhancer	CTC-276P9.2
chr5	134455000	chr5	140325000	3.65E-02	promoter	super_enhancer	CTC-276P9.2
chr5	134455000	chr5	151105000	1.65E-02	promoter	super_enhancer	CTC-276P9.2
chr5	134455000	chr5	174355000	9.12E-03	promoter	super_enhancer	CTC-276P9.2
chr5	134505000	chr5	139505000	3.84E-02	super_enhancer	promoter	IGIP
chr5	134505000	chr5	179335000	8.92E-03	super_enhancer	promoter	TBC1D9B
chr5	134525000	chr5	148725000	1.74E-02	super_enhancer	promoter	GRPEL2
chr5	134525000	chr5	167955000	9.08E-03	super_enhancer	promoter	FBLL1
chr5	134555000	chr5	139505000	3.88E-02	super_enhancer	promoter	IGIP
chr5	134555000	chr5	140575000	2.40E-02	super_enhancer	promoter	CH17-140K24.6
chr5	134555000	chr5	157175000	1.35E-02	super_enhancer	promoter	CTC-370J7.1
chr5	134585000	chr5	136015000	4.60E-02	promoter	super_enhancer	C5orf66-AS2
chr5	134585000	chr5	143255000	3.19E-02	promoter	super_enhancer	C5orf66-AS2
chr5	134585000	chr5	150465000	2.62E-02	promoter	super_enhancer	C5orf66-AS2
chr5	134735000	chr5	142345000	3.09E-02	promoter	super_enhancer	CTC-203F4.2
chr5	134735000	chr5	142835000	3.72E-02	promoter	super_enhancer	CTC-203F4.2
chr5	134735000	chr5	172875000	9.97E-03	promoter	super_enhancer	CTC-203F4.2
chr5	134785000	chr5	135225000	3.01E-02	promoter	super_enhancer	CTB-138E5.1
chr5	134785000	chr5	141845000	4.30E-02	promoter	super_enhancer	CTB-138E5.1
chr5	134785000	chr5	173305000	8.31E-03	promoter	super_enhancer	CTB-138E5.1
chr5	134895000	chr5	135245000	4.17E-03	promoter	super_enhancer	CTC-321K16.1
chr5	134895000	chr5	151085000	2.80E-02	promoter	super_enhancer	CTC-321K16.1
chr5	134895000	chr5	173305000	1.01E-02	promoter	super_enhancer	CTC-321K16.1
chr5	134915000	chr5	135155000	8.66E-03	promoter	super_enhancer	CXCL14

chr5	135145000	chr5	140685000	4.63E-02	super_enhancer	promoter	SLC25A2
chr5	135145000	chr5	145485000	1.99E-02	super_enhancer	promoter	PLAC8L1
chr5	135145000	chr5	150225000	1.55E-02	super_enhancer	promoter	IRGM
chr5	135145000	chr5	165555000	7.84E-03	super_enhancer	promoter	RP11-67M9.1
chr5	135155000	chr5	167395000	1.07E-02	super_enhancer	promoter	CTB-77H17.1
chr5	135155000	chr5	171435000	1.05E-02	super_enhancer	promoter	FBXW11
chr5	135155000	chr5	180675000	6.73E-03	super_enhancer	promoter	GNB2L1
chr5	135225000	chr5	135465000	1.30E-02	super_enhancer	promoter	SMAD5-AS1_1
chr5	135235000	chr5	136305000	1.01E-02	super_enhancer	promoter	RNA5SP193
chr5	135235000	chr5	140235000	4.90E-02	super_enhancer	promoter	PCDHA10
chr5	135235000	chr5	140625000	4.01E-02	super_enhancer	promoter	CH17-140K24.2
chr5	135235000	chr5	149865000	2.40E-02	super_enhancer	promoter	NDST1
chr5	135235000	chr5	172125000	1.44E-02	super_enhancer	promoter	CTB-79E8.2
chr5	135245000	chr5	169815000	1.29E-02	super_enhancer	promoter	CTD-2270F17.1
chr5	135245000	chr5	174045000	1.09E-02	super_enhancer	promoter	RP11-267A15.3
chr5	135255000	chr5	136025000	6.85E-03	promoter	super_enhancer	GS1-39E22.1
chr5	135255000	chr5	141825000	4.63E-02	promoter	super_enhancer	GS1-39E22.1
chr5	135255000	chr5	150125000	1.70E-02	promoter	super_enhancer	GS1-39E22.1
chr5	135255000	chr5	172855000	1.20E-02	promoter	super_enhancer	GS1-39E22.1
chr5	135255000	chr5	180325000	6.43E-03	promoter	super_enhancer	GS1-39E22.1
chr5	135265000	chr5	143405000	2.99E-02	promoter	super_enhancer	FBXL21
chr5	135365000	chr5	141825000	4.42E-02	promoter	super_enhancer	TGFBI
chr5	135465000	chr5	137575000	1.60E-02	promoter	super_enhancer	SMAD5-AS1_1
chr5	135465000	chr5	142835000	8.09E-04	promoter	super_enhancer	SMAD5-AS1_1

chr5	135465000	chr5	173325000	8.35E-03	promoter	super_enhancer	SMAD5-AS1_1
chr5	135765000	chr5	136035000	4.60E-02	promoter	super_enhancer	HNRNPA1P13
chr5	135765000	chr5	136055000	4.42E-02	promoter	super_enhancer	HNRNPA1P13
chr5	135765000	chr5	151065000	1.63E-02	promoter	super_enhancer	HNRNPA1P13
chr5	135765000	chr5	154775000	1.24E-02	promoter	super_enhancer	HNRNPA1P13
chr5	135765000	chr5	168325000	9.25E-03	promoter	super_enhancer	HNRNPA1P13
chr5	135995000	chr5	140515000	3.57E-02	super_enhancer	promoter	PCDHB5
chr5	135995000	chr5	142985000	2.51E-02	super_enhancer	promoter	CTB-57H20.1
chr5	135995000	chr5	151305000	1.44E-02	super_enhancer	promoter	GLRA1
chr5	135995000	chr5	175815000	7.22E-03	super_enhancer	promoter	HIGD2A
chr5	136025000	chr5	149535000	2.59E-02	super_enhancer	promoter	PDGFRB
chr5	136035000	chr5	150605000	2.01E-02	super_enhancer	promoter	CCDC69
chr5	136035000	chr5	179685000	6.46E-03	super_enhancer	promoter	RP11-252I14.2
chr5	136045000	chr5	159675000	1.15E-02	super_enhancer	promoter	RP11-34P1.1
chr5	136045000	chr5	179335000	7.46E-03	super_enhancer	promoter	TBC1D9B
chr5	136055000	chr5	171435000	9.25E-03	super_enhancer	promoter	FBXW11
chr5	136305000	chr5	172185000	7.86E-03	promoter	super_enhancer	RNA5SP193
chr5	136935000	chr5	150505000	3.05E-02	promoter	super_enhancer	SPOCK1
chr5	136935000	chr5	168315000	1.20E-02	promoter	super_enhancer	SPOCK1
chr5	137225000	chr5	143245000	3.83E-02	promoter	super_enhancer	RP11-381K20.2
chr5	137225000	chr5	143395000	2.89E-02	promoter	super_enhancer	RP11-381K20.2
chr5	137375000	chr5	148975000	2.67E-02	promoter	super_enhancer	RP11-325L7.2
chr5	137375000	chr5	150475000	3.29E-04	promoter	super_enhancer	RP11-325L7.2
chr5	137375000	chr5	150495000	2.88E-02	promoter	super_enhancer	RP11-325L7.2

chr5	137405000	chr5	150515000	2.68E-02	promoter	super_enhancer	RNU6-1148P
chr5	137475000	chr5	150495000	2.63E-02	promoter	super_enhancer	NME5
chr5	137475000	chr5	168315000	9.54E-03	promoter	super_enhancer	NME5
chr5	137475000	chr5	173335000	9.80E-03	promoter	super_enhancer	NME5
chr5	137515000	chr5	173295000	1.04E-02	promoter	super_enhancer	BRD8
chr5	137565000	chr5	137775000	3.14E-02	super_enhancer	promoter	REEP2
chr5	137565000	chr5	154065000	2.57E-02	super_enhancer	promoter	MIR1303
chr5	137575000	chr5	150605000	2.63E-02	super_enhancer	promoter	CCDC69
chr5	137685000	chr5	173315000	1.03E-02	promoter	super_enhancer	RP11-256P1.1
chr5	137685000	chr5	173335000	1.17E-02	promoter	super_enhancer	RP11-256P1.1
chr5	137775000	chr5	139485000	3.89E-03	promoter	super_enhancer	REEP2
chr5	137775000	chr5	148965000	3.00E-02	promoter	super_enhancer	REEP2
chr5	137955000	chr5	138575000	2.18E-02	promoter	super_enhancer	CTB-164L20.1
chr5	138575000	chr5	180675000	6.74E-03	super_enhancer	promoter	GNB2L1
chr5	138615000	chr5	148965000	2.52E-02	promoter	super_enhancer	AC011404.1
chr5	138635000	chr5	150515000	2.92E-02	promoter	super_enhancer	RNA5SP195
chr5	138635000	chr5	151055000	3.07E-02	promoter	super_enhancer	RNA5SP195
chr5	138635000	chr5	173455000	1.06E-02	promoter	super_enhancer	RNA5SP195
chr5	138905000	chr5	148985000	2.59E-02	promoter	super_enhancer	UBE2D2
chr5	138905000	chr5	151085000	2.86E-02	promoter	super_enhancer	UBE2D2
chr5	138905000	chr5	172725000	1.21E-02	promoter	super_enhancer	UBE2D2
chr5	139025000	chr5	150465000	3.28E-02	promoter	super_enhancer	CTD-3224K15.3
chr5	139155000	chr5	148975000	3.18E-02	promoter	super_enhancer	CTB-35F21.3
chr5	139155000	chr5	151085000	3.16E-02	promoter	super_enhancer	CTB-35F21.3

chr5	139155000	chr5	177495000	1.22E-02	promoter	super_enhancer	CTB-35F21.3
chr5	139175000	chr5	140325000	4.79E-02	promoter	super_enhancer	PSD2
chr5	139475000	chr5	150225000	1.90E-02	super_enhancer	promoter	IRGM
chr5	139475000	chr5	157235000	1.38E-02	super_enhancer	promoter	RNU6-260P
chr5	139475000	chr5	163285000	9.41E-03	super_enhancer	promoter	CTC-320C6.1
chr5	139475000	chr5	172385000	1.12E-02	super_enhancer	promoter	RPL26L1
chr5	139485000	chr5	140105000	3.35E-03	super_enhancer	promoter	VTRNA1-3
chr5	139485000	chr5	148205000	2.36E-02	super_enhancer	promoter	ADRB2
chr5	139485000	chr5	148725000	3.04E-02	super_enhancer	promoter	GRPEL2
chr5	139485000	chr5	167955000	1.45E-02	super_enhancer	promoter	FBLL1
chr5	139485000	chr5	176695000	8.40E-03	super_enhancer	promoter	PRMT1P1
chr5	139485000	chr5	178955000	9.88E-03	super_enhancer	promoter	RP11-798K23.5
chr5	139505000	chr5	150465000	2.04E-02	promoter	super_enhancer	IGIP
chr5	139575000	chr5	150125000	1.87E-02	promoter	super_enhancer	AC011379.1
chr5	139575000	chr5	150645000	2.53E-02	promoter	super_enhancer	AC011379.1
chr5	139645000	chr5	144385000	4.58E-02	super_enhancer	promoter	NAMPTP2
chr5	139645000	chr5	158655000	1.66E-02	super_enhancer	promoter	CTB-11I22.2
chr5	139655000	chr5	148785000	2.64E-02	super_enhancer	promoter	IL17B
chr5	139655000	chr5	162465000	8.70E-03	super_enhancer	promoter	MRPL57P6
chr5	139655000	chr5	165035000	8.09E-03	super_enhancer	promoter	RN7SKP60
chr5	139655000	chr5	171615000	1.33E-02	super_enhancer	promoter	STK10
chr5	139665000	chr5	154065000	2.33E-02	super_enhancer	promoter	MIR1303
chr5	140105000	chr5	150515000	2.81E-02	promoter	super_enhancer	VTRNA1-3
chr5	140165000	chr5	151085000	3.35E-02	promoter	super_enhancer	PCDHA1

chr5	140165000	chr5	154765000	2.34E-02	promoter	super_enhancer	PCDHA1
chr5	140235000	chr5	150505000	2.36E-02	promoter	super_enhancer	PCDHA10
chr5	140315000	chr5	145485000	2.89E-02	super_enhancer	promoter	PLAC8L1
chr5	140325000	chr5	144385000	4.20E-02	super_enhancer	promoter	NAMPTP2
chr5	140325000	chr5	149995000	2.84E-02	super_enhancer	promoter	CTB-140J7.2
chr5	140325000	chr5	156695000	1.46E-02	super_enhancer	promoter	CTC-248O19.1
chr5	140345000	chr5	148965000	3.33E-02	promoter	super_enhancer	PCDHAC2
chr5	140345000	chr5	150645000	3.12E-02	promoter	super_enhancer	PCDHAC2
chr5	140515000	chr5	150625000	1.94E-02	promoter	super_enhancer	PCDHB5
chr5	140515000	chr5	172175000	8.38E-03	promoter	super_enhancer	PCDHB5
chr5	140535000	chr5	150465000	2.91E-02	promoter	super_enhancer	PCDHB17P
chr5	140625000	chr5	154795000	1.47E-02	promoter	super_enhancer	CH17-140K24.2
chr5	140685000	chr5	148985000	2.95E-02	promoter	super_enhancer	SLC25A2
chr5	140685000	chr5	168325000	1.49E-02	promoter	super_enhancer	SLC25A2
chr5	140705000	chr5	150125000	1.71E-02	promoter	super_enhancer	AC005618.6
chr5	140705000	chr5	173315000	7.80E-03	promoter	super_enhancer	AC005618.6
chr5	140855000	chr5	150625000	2.06E-02	promoter	super_enhancer	PCDHGC3
chr5	141545000	chr5	143245000	2.28E-02	promoter	super_enhancer	CTC-463A16.1
chr5	141705000	chr5	141835000	2.53E-02	promoter	super_enhancer	AC005592.2
chr5	141705000	chr5	141845000	3.14E-02	promoter	super_enhancer	AC005592.2
chr5	141825000	chr5	148205000	3.25E-02	super_enhancer	promoter	ADRB2
chr5	141825000	chr5	154065000	3.03E-02	super_enhancer	promoter	MIR1303
chr5	141825000	chr5	175875000	1.12E-02	super_enhancer	promoter	FAF2
chr5	141825000	chr5	176785000	1.28E-02	super_enhancer	promoter	RGS14

chr5	141825000	chr5	180415000	1.03E-02	super_enhancer	promoter	BTNL3
chr5	141835000	chr5	149535000	4.63E-02	super_enhancer	promoter	PDGFRB
chr5	141835000	chr5	175795000	1.25E-02	super_enhancer	promoter	MIR1271
chr5	141845000	chr5	149395000	2.62E-02	super_enhancer	promoter	AC011406.2
chr5	141845000	chr5	158745000	2.41E-02	super_enhancer	promoter	RNU4ATAC2P
chr5	141845000	chr5	175865000	1.03E-02	super_enhancer	promoter	CTB-162J7.1
chr5	141845000	chr5	175965000	7.10E-05	super_enhancer	promoter	RNF44
chr5	141915000	chr5	172715000	1.21E-02	promoter	super_enhancer	AC005215.1
chr5	141915000	chr5	173465000	1.03E-02	promoter	super_enhancer	AC005215.1
chr5	141945000	chr5	150125000	2.48E-02	promoter	super_enhancer	RPS12P10
chr5	142125000	chr5	148915000	3.16E-02	promoter	super_enhancer	AC005592.1
chr5	142325000	chr5	148725000	3.86E-02	super_enhancer	promoter	GRPEL2
chr5	142325000	chr5	149325000	4.05E-04	super_enhancer	promoter	PDE6A
chr5	142335000	chr5	149325000	3.26E-02	super_enhancer	promoter	PDE6A
chr5	142335000	chr5	149545000	4.67E-02	super_enhancer	promoter	CDX1
chr5	142335000	chr5	157175000	2.12E-02	super_enhancer	promoter	CTC-370J7.1
chr5	142345000	chr5	179515000	9.43E-03	super_enhancer	promoter	RP11-39H3.1
chr5	142355000	chr5	149995000	4.08E-02	super_enhancer	promoter	CTB-140J7.2
chr5	142355000	chr5	150605000	3.16E-02	super_enhancer	promoter	CCDC69
chr5	142355000	chr5	179235000	1.11E-02	super_enhancer	promoter	MGAT4B
chr5	142365000	chr5	157415000	1.93E-02	super_enhancer	promoter	CTB-47B8.5
chr5	142365000	chr5	173045000	1.23E-02	super_enhancer	promoter	BOD1
chr5	142365000	chr5	173115000	8.82E-03	super_enhancer	promoter	CTB-43E15.2
chr5	142365000	chr5	179085000	7.99E-03	super_enhancer	promoter	RP11-1379J22.5

chr5	142755000	chr5	143245000	3.85E-03	promoter	super_enhancer	RP11-278J6.5
chr5	142755000	chr5	148915000	3.25E-02	promoter	super_enhancer	RP11-278J6.5
chr5	142755000	chr5	150475000	2.84E-02	promoter	super_enhancer	RP11-278J6.5
chr5	142755000	chr5	177495000	1.00E-02	promoter	super_enhancer	RP11-278J6.5
chr5	142805000	chr5	175815000	1.11E-02	super_enhancer	promoter	HIGD2A
chr5	142815000	chr5	142985000	3.72E-02	super_enhancer	promoter	CTB-57H20.1
chr5	142815000	chr5	159675000	2.09E-02	super_enhancer	promoter	RP11-34P1.1
chr5	142815000	chr5	159685000	2.16E-02	super_enhancer	promoter	RP11-34P1.2
chr5	142815000	chr5	169725000	1.55E-02	super_enhancer	promoter	LCP2
chr5	142825000	chr5	170785000	1.23E-02	super_enhancer	promoter	RN7SL339P
chr5	142835000	chr5	154065000	3.28E-02	super_enhancer	promoter	MIR1303
chr5	142835000	chr5	158745000	2.64E-02	super_enhancer	promoter	RNU4ATAC2P
chr5	142835000	chr5	176945000	1.39E-02	super_enhancer	promoter	DDX41
chr5	142835000	chr5	178955000	1.18E-02	super_enhancer	promoter	RP11-798K23.5
chr5	143245000	chr5	170815000	1.20E-02	super_enhancer	promoter	MIR3912
chr5	143255000	chr5	145215000	1.41E-02	super_enhancer	promoter	PRELID2
chr5	143255000	chr5	148205000	3.28E-02	super_enhancer	promoter	ADRB2
chr5	143255000	chr5	157235000	1.58E-02	super_enhancer	promoter	RNU6-260P
chr5	143255000	chr5	176785000	1.04E-02	super_enhancer	promoter	RGS14
chr5	143395000	chr5	150225000	2.23E-02	super_enhancer	promoter	IRGM
chr5	143395000	chr5	170785000	9.42E-03	super_enhancer	promoter	RN7SL339P
chr5	143405000	chr5	180395000	7.94E-03	super_enhancer	promoter	CTD-2593A12.2
chr5	143585000	chr5	145215000	1.55E-03	super_enhancer	promoter	PRELID2
chr5	143595000	chr5	145315000	8.09E-03	super_enhancer	promoter	SH3RF2

chr5	143595000	chr5	148885000	2.76E-02	super_enhancer	promoter	CTB-89H12.4
chr5	143595000	chr5	179235000	8.08E-03	super_enhancer	promoter	MGAT4B
chr5	143915000	chr5	148985000	3.67E-02	promoter	super_enhancer	RN7SKP246
chr5	144365000	chr5	150515000	2.81E-02	promoter	super_enhancer	AC005371.1
chr5	144365000	chr5	154755000	1.20E-02	promoter	super_enhancer	AC005371.1
chr5	145215000	chr5	150625000	4.28E-02	promoter	super_enhancer	PRELID2
chr5	145215000	chr5	150655000	4.72E-02	promoter	super_enhancer	PRELID2
chr5	145315000	chr5	150125000	3.03E-02	promoter	super_enhancer	SH3RF2
chr5	145485000	chr5	172735000	1.30E-02	promoter	super_enhancer	PLAC8L1
chr5	146465000	chr5	150515000	4.90E-02	promoter	super_enhancer	PPP2R2B
chr5	146465000	chr5	154785000	1.68E-02	promoter	super_enhancer	PPP2R2B
chr5	148725000	chr5	172185000	1.43E-02	promoter	super_enhancer	GRPEL2
chr5	148785000	chr5	148985000	9.93E-03	promoter	super_enhancer	IL17B
chr5	148915000	chr5	165555000	1.27E-02	super_enhancer	promoter	RP11-67M9.1
chr5	148915000	chr5	166595000	1.36E-02	super_enhancer	promoter	CTC-264O10.1
chr5	148915000	chr5	167955000	1.69E-02	super_enhancer	promoter	FBLL1
chr5	148915000	chr5	175865000	1.03E-02	super_enhancer	promoter	CTB-162J7.1
chr5	148915000	chr5	178245000	1.05E-02	super_enhancer	promoter	AACSP1
chr5	148925000	chr5	172715000	1.53E-02	promoter	super_enhancer	RPL29P14
chr5	148965000	chr5	171705000	1.41E-02	super_enhancer	promoter	AC008671.1
chr5	148965000	chr5	176925000	1.16E-02	super_enhancer	promoter	PDLIM7
chr5	148975000	chr5	171435000	1.41E-02	super_enhancer	promoter	FBXW11
chr5	148975000	chr5	171705000	1.37E-02	super_enhancer	promoter	AC008671.1
chr5	148985000	chr5	158655000	1.98E-02	super_enhancer	promoter	CTB-11I22.2

chr5	149395000	chr5	168295000	1.05E-02	promoter	super_enhancer	AC011406.2
chr5	149395000	chr5	173315000	8.77E-03	promoter	super_enhancer	AC011406.2
chr5	149535000	chr5	151055000	6.02E-04	promoter	super_enhancer	PDGFRB
chr5	149535000	chr5	173325000	1.51E-02	promoter	super_enhancer	PDGFRB
chr5	149545000	chr5	172735000	2.15E-02	promoter	super_enhancer	CDX1
chr5	149865000	chr5	154785000	3.58E-02	promoter	super_enhancer	NDST1
chr5	149865000	chr5	177495000	1.33E-02	promoter	super_enhancer	NDST1
chr5	149995000	chr5	172445000	1.72E-02	promoter	super_enhancer	CTB-140J7.2
chr5	149995000	chr5	172725000	1.91E-02	promoter	super_enhancer	CTB-140J7.2
chr5	150125000	chr5	179105000	1.01E-02	super_enhancer	promoter	CANX
chr5	150125000	chr5	179655000	6.94E-03	super_enhancer	promoter	RP11-252I14.1
chr5	150135000	chr5	150605000	4.52E-02	super_enhancer	promoter	CCDC69
chr5	150135000	chr5	153825000	4.14E-02	super_enhancer	promoter	SAP30L-AS1
chr5	150145000	chr5	151305000	2.32E-03	super_enhancer	promoter	GLRA1
chr5	150145000	chr5	175775000	9.56E-03	super_enhancer	promoter	RP11-843P14.2
chr5	150465000	chr5	161905000	1.50E-02	super_enhancer	promoter	RNU6-164P
chr5	150465000	chr5	172385000	1.85E-02	super_enhancer	promoter	RPL26L1
chr5	150465000	chr5	177615000	1.35E-02	super_enhancer	promoter	GMCL1P1
chr5	150475000	chr5	157405000	3.18E-02	super_enhancer	promoter	RNU2-48P
chr5	150475000	chr5	171615000	1.98E-02	super_enhancer	promoter	STK10
chr5	150475000	chr5	175865000	1.18E-02	super_enhancer	promoter	CTB-162J7.1
chr5	150485000	chr5	156695000	3.45E-02	super_enhancer	promoter	CTC-248O19.1
chr5	150485000	chr5	172385000	1.78E-02	super_enhancer	promoter	RPL26L1
chr5	150485000	chr5	175055000	1.42E-02	super_enhancer	promoter	RN7SKP148

chr5	150485000	chr5	175975000	1.57E-02	super_enhancer	promoter	RN7SL684P
chr5	150495000	chr5	175965000	1.53E-02	super_enhancer	promoter	RNF44
chr5	150505000	chr5	173045000	1.65E-02	super_enhancer	promoter	BOD1
chr5	150505000	chr5	179285000	1.26E-02	super_enhancer	promoter	CTC-241N9.1
chr5	150515000	chr5	151255000	2.43E-02	super_enhancer	promoter	RNA5SP198
chr5	150515000	chr5	159675000	2.73E-02	super_enhancer	promoter	RP11-34P1.1
chr5	150515000	chr5	167955000	2.10E-02	super_enhancer	promoter	FBLL1
chr5	150515000	chr5	179515000	1.36E-02	super_enhancer	promoter	RP11-39H3.1
chr5	150515000	chr5	179655000	1.01E-02	super_enhancer	promoter	RP11-252I14.1
chr5	150605000	chr5	168325000	1.92E-02	promoter	super_enhancer	CCDC69
chr5	150625000	chr5	157405000	2.98E-02	super_enhancer	promoter	RNU2-48P
chr5	150625000	chr5	175735000	7.89E-03	super_enhancer	promoter	BRCC3P1
chr5	150645000	chr5	175795000	1.37E-02	super_enhancer	promoter	MIR1271
chr5	150655000	chr5	173115000	1.16E-02	super_enhancer	promoter	CTB-43E15.2
chr5	150655000	chr5	178805000	1.03E-02	super_enhancer	promoter	RP11-443C10.2
chr5	151055000	chr5	158655000	3.10E-02	super_enhancer	promoter	CTB-11I22.2
chr5	151055000	chr5	165555000	1.76E-02	super_enhancer	promoter	RP11-67M9.1
chr5	151055000	chr5	166595000	1.87E-02	super_enhancer	promoter	CTC-264O10.1
chr5	151055000	chr5	171615000	2.36E-02	super_enhancer	promoter	STK10
chr5	151055000	chr5	178245000	1.41E-02	super_enhancer	promoter	AACSP1
chr5	151065000	chr5	159675000	2.56E-02	super_enhancer	promoter	RP11-34P1.1
chr5	151065000	chr5	167615000	1.53E-02	super_enhancer	promoter	CTB-178M22.1
chr5	151065000	chr5	173485000	1.42E-02	super_enhancer	promoter	RP11-619L12.3
chr5	151065000	chr5	179285000	1.18E-02	super_enhancer	promoter	CTC-241N9.1

chr5	151075000	chr5	159435000	2.69E-02	super_enhancer	promoter	TTC1
chr5	151075000	chr5	162465000	1.43E-02	super_enhancer	promoter	MRPL57P6
chr5	151085000	chr5	153135000	1.54E-02	super_enhancer	promoter	RN7SL177P
chr5	151085000	chr5	166595000	1.74E-02	super_enhancer	promoter	CTC-264O10.1
chr5	151085000	chr5	178955000	1.35E-02	super_enhancer	promoter	RP11-798K23.5
chr5	154065000	chr5	168305000	2.30E-02	promoter	super_enhancer	MIR1303
chr5	154065000	chr5	172855000	2.27E-02	promoter	super_enhancer	MIR1303
chr5	154755000	chr5	175085000	1.17E-02	super_enhancer	promoter	HRH2
chr5	154755000	chr5	180395000	7.72E-03	super_enhancer	promoter	CTD-2593A12.2
chr5	154765000	chr5	180215000	1.38E-02	super_enhancer	promoter	CTC-205M6.5
chr5	154785000	chr5	169455000	1.25E-02	super_enhancer	promoter	MIR378E
chr5	154795000	chr5	175085000	1.51E-02	super_enhancer	promoter	HRH2
chr5	157175000	chr5	172875000	1.80E-02	promoter	super_enhancer	CTC-370J7.1
chr5	157405000	chr5	168305000	2.28E-02	promoter	super_enhancer	RNU2-48P
chr5	157415000	chr5	174345000	1.54E-02	promoter	super_enhancer	CTB-47B8.5
chr5	158655000	chr5	172715000	1.93E-02	promoter	super_enhancer	CTB-11I22.2
chr5	158655000	chr5	173315000	1.52E-02	promoter	super_enhancer	CTB-11I22.2
chr5	158745000	chr5	177495000	2.10E-02	promoter	super_enhancer	RNU4ATAC2P
chr5	159435000	chr5	168295000	2.26E-02	promoter	super_enhancer	TTC1
chr5	159435000	chr5	172425000	2.39E-02	promoter	super_enhancer	TTC1
chr5	159435000	chr5	172855000	2.48E-02	promoter	super_enhancer	TTC1
chr5	159435000	chr5	172875000	2.23E-02	promoter	super_enhancer	TTC1
chr5	159675000	chr5	172445000	1.95E-02	promoter	super_enhancer	RP11-34P1.1
chr5	159675000	chr5	172875000	2.20E-02	promoter	super_enhancer	RP11-34P1.1

chr5	159685000	chr5	173305000	1.76E-02	promoter	super_enhancer	RP11-34P1.2
chr5	159895000	chr5	172425000	2.30E-02	promoter	super_enhancer	CTC-231O11.1
chr5	161905000	chr5	173325000	1.24E-02	promoter	super_enhancer	RNU6-164P
chr5	162465000	chr5	168305000	2.41E-02	promoter	super_enhancer	MRPL57P6
chr5	162865000	chr5	168315000	3.02E-02	promoter	super_enhancer	RP11-541P9.3
chr5	162865000	chr5	174355000	2.07E-02	promoter	super_enhancer	RP11-541P9.3
chr5	163895000	chr5	168305000	3.53E-02	promoter	super_enhancer	CTD-2313P7.1
chr5	164775000	chr5	173455000	1.93E-02	promoter	super_enhancer	CTC-535M15.2
chr5	165555000	chr5	172885000	2.44E-02	promoter	super_enhancer	RP11-67M9.1
chr5	165555000	chr5	173325000	1.99E-02	promoter	super_enhancer	RP11-67M9.1
chr5	167395000	chr5	173335000	3.37E-02	promoter	super_enhancer	CTB-77H17.1
chr5	167395000	chr5	173465000	3.13E-02	promoter	super_enhancer	CTB-77H17.1
chr5	167555000	chr5	174375000	3.54E-02	promoter	super_enhancer	CTB-105N12.2
chr5	167615000	chr5	172415000	4.47E-02	promoter	super_enhancer	CTB-178M22.1
chr5	168295000	chr5	169815000	2.16E-02	super_enhancer	promoter	CTD-2270F17.1
chr5	168295000	chr5	175975000	2.97E-02	super_enhancer	promoter	RN7SL684P
chr5	168295000	chr5	178955000	2.16E-02	super_enhancer	promoter	RP11-798K23.5
chr5	168305000	chr5	169455000	4.02E-02	super_enhancer	promoter	MIR378E
chr5	168305000	chr5	172085000	4.73E-02	super_enhancer	promoter	CTB-79E8.3
chr5	168315000	chr5	178955000	2.07E-02	super_enhancer	promoter	RP11-798K23.5
chr5	168325000	chr5	178805000	2.14E-02	super_enhancer	promoter	RP11-443C10.2
chr5	168335000	chr5	169455000	3.96E-03	super_enhancer	promoter	MIR378E
chr5	169455000	chr5	174385000	3.11E-02	promoter	super_enhancer	MIR378E
chr5	170785000	chr5	172875000	1.19E-02	promoter	super_enhancer	RN7SL339P

chr5	170785000	chr5	180325000	1.94E-02	promoter	super_enhancer	RN7SL339P
chr5	171435000	chr5	173305000	1.11E-02	promoter	super_enhancer	FBXW11
chr5	172085000	chr5	173295000	2.23E-02	promoter	super_enhancer	CTB-79E8.3
chr5	172125000	chr5	172415000	2.85E-02	promoter	super_enhancer	CTB-79E8.2
chr5	172125000	chr5	172715000	8.55E-03	promoter	super_enhancer	CTB-79E8.2
chr5	172125000	chr5	174345000	1.32E-02	promoter	super_enhancer	CTB-79E8.2
chr5	172175000	chr5	172385000	1.94E-02	super_enhancer	promoter	RPL26L1
chr5	172175000	chr5	173045000	2.02E-02	super_enhancer	promoter	BOD1
chr5	172175000	chr5	176925000	4.37E-02	super_enhancer	promoter	PDLIM7
chr5	172175000	chr5	180415000	2.68E-02	super_enhancer	promoter	BTNL3
chr5	172415000	chr5	173045000	3.02E-03	super_enhancer	promoter	BOD1
chr5	172415000	chr5	179685000	3.13E-02	super_enhancer	promoter	RP11-252I14.2
chr5	172425000	chr5	173045000	3.58E-03	super_enhancer	promoter	BOD1
chr5	172425000	chr5	174045000	2.75E-02	super_enhancer	promoter	RP11-267A15.3
chr5	172445000	chr5	175665000	4.56E-02	super_enhancer	promoter	SIMC1
chr5	172445000	chr5	178805000	2.79E-02	super_enhancer	promoter	RP11-443C10.2
chr5	172445000	chr5	179285000	3.16E-02	super_enhancer	promoter	CTC-241N9.1
chr5	172725000	chr5	180675000	2.48E-02	super_enhancer	promoter	GNB2L1
chr5	172855000	chr5	179235000	4.88E-02	super_enhancer	promoter	MGAT4B
chr5	172855000	chr5	179335000	4.75E-02	super_enhancer	promoter	TBC1D9B
chr5	172875000	chr5	179655000	2.91E-02	super_enhancer	promoter	RP11-252I14.1
chr5	172875000	chr5	180395000	2.82E-02	super_enhancer	promoter	CTD-2593A12.2
chr5	172885000	chr5	179235000	4.33E-02	super_enhancer	promoter	MGAT4B
chr5	172885000	chr5	179285000	3.81E-02	super_enhancer	promoter	CTC-241N9.1

chr5	172905000	chr5	176875000	3.33E-02	super_enhancer	promoter	PRR7-AS1
chr5	172905000	chr5	176945000	8.65E-04	super_enhancer	promoter	DDX41
chr5	172905000	chr5	179335000	2.47E-02	super_enhancer	promoter	TBC1D9B
chr5	173045000	chr5	173455000	1.50E-03	promoter	super_enhancer	BOD1
chr5	173295000	chr5	178395000	3.31E-02	super_enhancer	promoter	RP11-281O15.4
chr5	173315000	chr5	179105000	3.92E-02	super_enhancer	promoter	CANX
chr5	173325000	chr5	176925000	1.77E-03	super_enhancer	promoter	PDLIM7
chr5	173335000	chr5	179105000	4.45E-02	super_enhancer	promoter	CANX
chr5	173335000	chr5	180675000	2.51E-02	super_enhancer	promoter	GNB2L1
chr5	173455000	chr5	175085000	2.32E-02	super_enhancer	promoter	HRH2
chr5	173465000	chr5	175965000	6.16E-03	super_enhancer	promoter	RNF44
chr5	173515000	chr5	174345000	3.15E-02	promoter	super_enhancer	RP11-619L12.4
chr5	173515000	chr5	174355000	4.06E-02	promoter	super_enhancer	RP11-619L12.4
chr5	174345000	chr5	179085000	6.89E-04	super_enhancer	promoter	RP11-1379J22.5
chr5	174345000	chr5	180415000	3.40E-02	super_enhancer	promoter	BTNL3
chr5	174355000	chr5	175735000	1.70E-02	super_enhancer	promoter	BRCC3P1
chr5	174365000	chr5	175085000	3.61E-02	super_enhancer	promoter	HRH2
chr5	174375000	chr5	175085000	3.83E-02	super_enhancer	promoter	HRH2
chr5	174375000	chr5	175775000	3.05E-03	super_enhancer	promoter	RP11-843P14.2
chr5	176695000	chr5	180325000	4.41E-02	promoter	super_enhancer	PRMT1P1
chr5	180325000	chr5	180395000	1.31E-02	super_enhancer	promoter	CTD-2593A12.2
chr6	205000	chr6	2625000	1.97E-02	promoter	super_enhancer	RP3-416J7.4
chr6	205000	chr6	73605000	3.87E-03	promoter	super_enhancer	RP3-416J7.4
chr6	205000	chr6	150855000	5.54E-03	promoter	super_enhancer	RP3-416J7.4

chr6	1385000	chr6	2625000	2.34E-02	promoter	super_enhancer	RP4-668J24.2
chr6	1385000	chr6	10745000	4.18E-02	promoter	super_enhancer	RP4-668J24.2
chr6	1385000	chr6	31195000	2.10E-02	promoter	super_enhancer	RP4-668J24.2
chr6	1385000	chr6	63535000	3.88E-03	promoter	super_enhancer	RP4-668J24.2
chr6	1605000	chr6	2635000	9.86E-05	promoter	super_enhancer	FOXCUT
chr6	1605000	chr6	26335000	1.07E-02	promoter	super_enhancer	FOXCUT
chr6	2625000	chr6	19805000	1.58E-04	super_enhancer	promoter	RP4-625H18.2
chr6	2625000	chr6	20725000	2.12E-02	super_enhancer	promoter	RP3-348I23.3
chr6	2625000	chr6	25065000	2.13E-02	super_enhancer	promoter	RP3-425P12.2
chr6	2625000	chr6	28585000	1.92E-02	super_enhancer	promoter	ZBED9
chr6	2625000	chr6	31675000	2.27E-02	super_enhancer	promoter	LY6G6F
chr6	2625000	chr6	50825000	8.93E-03	super_enhancer	promoter	RPS17P5
chr6	2635000	chr6	15995000	3.79E-02	super_enhancer	promoter	RP11-401E14.2
chr6	2635000	chr6	18155000	2.46E-02	super_enhancer	promoter	TPMT
chr6	2635000	chr6	35765000	2.17E-02	super_enhancer	promoter	CLPS
chr6	2635000	chr6	36515000	1.38E-02	super_enhancer	promoter	STK38
chr6	2635000	chr6	44235000	1.06E-02	super_enhancer	promoter	NFKBIE
chr6	2765000	chr6	9515000	3.37E-02	promoter	super_enhancer	WRNIP1
chr6	2765000	chr6	10725000	4.38E-02	promoter	super_enhancer	WRNIP1
chr6	2765000	chr6	16755000	2.30E-02	promoter	super_enhancer	WRNIP1
chr6	2765000	chr6	26355000	1.27E-04	promoter	super_enhancer	WRNIP1
chr6	2855000	chr6	3055000	4.82E-02	super_enhancer	promoter	RP1-40E16.2
chr6	2855000	chr6	14395000	4.04E-02	super_enhancer	promoter	RP3-448I9.2
chr6	2855000	chr6	26195000	2.60E-02	super_enhancer	promoter	HIST1H1PS1

chr6	2855000	chr6	27105000	1.48E-02	super_enhancer	promoter	HIST1H4I
chr6	2855000	chr6	27235000	2.13E-02	super_enhancer	promoter	XXbac-BPGBPG24O18.1
chr6	2855000	chr6	29895000	2.15E-02	super_enhancer	promoter	HCG4B
chr6	2855000	chr6	33175000	1.57E-02	super_enhancer	promoter	MIR219A1
chr6	2855000	chr6	74405000	6.15E-03	super_enhancer	promoter	CD109
chr6	2855000	chr6	137115000	5.45E-03	super_enhancer	promoter	MAP3K5
chr6	2875000	chr6	27105000	1.17E-02	super_enhancer	promoter	HIST1H4I
chr6	2875000	chr6	29695000	1.48E-02	super_enhancer	promoter	RPL23AP1
chr6	2905000	chr6	35485000	1.43E-02	promoter	super_enhancer	SERPINB9
chr6	2905000	chr6	42305000	1.04E-02	promoter	super_enhancer	SERPINB9
chr6	2975000	chr6	10695000	4.23E-02	super_enhancer	promoter	C6orf52
chr6	2975000	chr6	15935000	3.02E-02	super_enhancer	promoter	ARPC3P5
chr6	2975000	chr6	18305000	2.77E-02	super_enhancer	promoter	RP11-14J16.1
chr6	2975000	chr6	20995000	1.97E-02	super_enhancer	promoter	RNU6-150P
chr6	2975000	chr6	24365000	2.00E-02	super_enhancer	promoter	RNU6-391P
chr6	2975000	chr6	26365000	1.70E-02	super_enhancer	promoter	BTN3A2
chr6	2975000	chr6	29465000	1.99E-02	super_enhancer	promoter	XXbac-BPG13B8.10
chr6	2975000	chr6	31775000	1.89E-02	super_enhancer	promoter	LSM2
chr6	2975000	chr6	41755000	9.54E-03	super_enhancer	promoter	FRS3
chr6	2985000	chr6	10575000	4.53E-02	super_enhancer	promoter	RP11-360O19.5
chr6	2985000	chr6	24365000	2.23E-02	super_enhancer	promoter	RNU6-391P
chr6	2985000	chr6	35435000	1.57E-02	super_enhancer	promoter	RPL10A
chr6	3055000	chr6	3825000	1.72E-02	promoter	super_enhancer	RP1-40E16.2
chr6	3055000	chr6	14735000	2.77E-02	promoter	super_enhancer	RP1-40E16.2

chr6	3625000	chr6	11775000	3.45E-02	promoter	super_enhancer	RP1-223B1.1
chr6	3625000	chr6	30785000	1.59E-02	promoter	super_enhancer	RP1-223B1.1
chr6	3625000	chr6	44085000	6.91E-03	promoter	super_enhancer	RP1-223B1.1
chr6	3695000	chr6	73585000	3.35E-03	promoter	super_enhancer	RP1-72E17.2
chr6	3795000	chr6	8065000	4.75E-02	super_enhancer	promoter	BLOC1S5-TXNDC5
chr6	3795000	chr6	13825000	1.77E-02	super_enhancer	promoter	RP3-500L14.2
chr6	3795000	chr6	15105000	2.37E-02	super_enhancer	promoter	RP1-190J20.2
chr6	3795000	chr6	19735000	1.70E-02	super_enhancer	promoter	RP3-471C18.1
chr6	3795000	chr6	20725000	1.45E-02	super_enhancer	promoter	RP3-348I23.3
chr6	3795000	chr6	28555000	1.47E-02	super_enhancer	promoter	RP5-1186N24.3
chr6	3795000	chr6	31495000	1.58E-02	super_enhancer	promoter	RPL15P4
chr6	3795000	chr6	33405000	9.13E-03	super_enhancer	promoter	MIR5004
chr6	3795000	chr6	142505000	2.67E-03	super_enhancer	promoter	AL033522.1
chr6	3805000	chr6	10215000	4.32E-02	super_enhancer	promoter	RP11-339A7.1
chr6	3805000	chr6	11155000	3.01E-02	super_enhancer	promoter	RP4-761I2.5
chr6	3805000	chr6	11505000	3.30E-02	super_enhancer	promoter	RNU1-64P
chr6	3805000	chr6	14285000	3.88E-02	super_enhancer	promoter	LINC01108
chr6	3805000	chr6	15315000	3.67E-02	super_enhancer	promoter	RNU6-522P
chr6	3805000	chr6	16165000	3.17E-02	super_enhancer	promoter	RP1-13D10.3
chr6	3805000	chr6	25325000	2.18E-02	super_enhancer	promoter	AL024509.1
chr6	3805000	chr6	29695000	1.68E-02	super_enhancer	promoter	RPL23AP1
chr6	3805000	chr6	31325000	1.72E-02	super_enhancer	promoter	HLA-C
chr6	3805000	chr6	37535000	1.35E-02	super_enhancer	promoter	RP1-153P14.7
chr6	3805000	chr6	41835000	1.05E-02	super_enhancer	promoter	RP5-973N23.4

chr6	3805000	chr6	44865000	6.74E-03	super_enhancer	promoter	NUDT19P4
chr6	3815000	chr6	13615000	3.93E-02	super_enhancer	promoter	RP1-223E5.4
chr6	3815000	chr6	14285000	4.40E-02	super_enhancer	promoter	LINC01108
chr6	3815000	chr6	15105000	3.70E-02	super_enhancer	promoter	RP1-190J20.2
chr6	3815000	chr6	18155000	2.55E-02	super_enhancer	promoter	TPMT
chr6	3815000	chr6	26045000	2.23E-02	super_enhancer	promoter	HIST1H3C
chr6	3815000	chr6	28155000	2.26E-02	super_enhancer	promoter	ZNF192P2
chr6	3815000	chr6	33865000	1.63E-02	super_enhancer	promoter	LINC01016
chr6	3815000	chr6	36355000	1.53E-02	super_enhancer	promoter	RP1-50J22.4
chr6	3815000	chr6	53015000	7.12E-03	super_enhancer	promoter	GCM1
chr6	3825000	chr6	23125000	1.76E-02	super_enhancer	promoter	RNU6-1060P
chr6	3825000	chr6	30525000	2.22E-02	super_enhancer	promoter	GNL1
chr6	3825000	chr6	33065000	1.23E-02	super_enhancer	promoter	HLA-DPA2
chr6	3825000	chr6	33175000	1.46E-02	super_enhancer	promoter	MIR219A1
chr6	3825000	chr6	150115000	4.20E-03	super_enhancer	promoter	BTBD10P2
chr6	3915000	chr6	30685000	1.80E-02	promoter	super_enhancer	RP1-140K8.2
chr6	3915000	chr6	44025000	8.02E-03	promoter	super_enhancer	RP1-140K8.2
chr6	4315000	chr6	24775000	2.22E-02	super_enhancer	promoter	GMNN
chr6	4315000	chr6	29695000	1.72E-02	super_enhancer	promoter	RPL23AP1
chr6	4315000	chr6	30595000	2.41E-02	super_enhancer	promoter	ATAT1
chr6	4315000	chr6	31775000	2.10E-02	super_enhancer	promoter	LSM2
chr6	4315000	chr6	34545000	1.51E-02	super_enhancer	promoter	RP3-391O22.3
chr6	4315000	chr6	34725000	1.43E-02	super_enhancer	promoter	SNRPC
chr6	4315000	chr6	150205000	5.00E-03	super_enhancer	promoter	RAET1E-AS1

chr6	4325000	chr6	5085000	4.13E-02	super_enhancer	promoter	PPP1R3G
chr6	4325000	chr6	17705000	3.06E-02	super_enhancer	promoter	RP11-500C11.3
chr6	4325000	chr6	22215000	2.02E-02	super_enhancer	promoter	RP11-524C21.1
chr6	4325000	chr6	30525000	1.82E-02	super_enhancer	promoter	GNL1
chr6	4325000	chr6	111135000	3.42E-03	super_enhancer	promoter	AMD1
chr6	4325000	chr6	169365000	2.86E-03	super_enhancer	promoter	RP3-495K2.3
chr6	4335000	chr6	5085000	1.59E-02	super_enhancer	promoter	PPP1R3G
chr6	4335000	chr6	30595000	1.37E-02	super_enhancer	promoter	ATAT1
chr6	4335000	chr6	36955000	6.75E-03	super_enhancer	promoter	MTCH1
chr6	4335000	chr6	71105000	2.58E-03	super_enhancer	promoter	RP11-462G2.1
chr6	4345000	chr6	4705000	2.62E-02	super_enhancer	promoter	CDYL
chr6	4345000	chr6	14005000	2.93E-02	super_enhancer	promoter	RP3-380E11.2
chr6	4345000	chr6	20995000	2.18E-02	super_enhancer	promoter	RNU6-150P
chr6	4345000	chr6	32685000	1.24E-02	super_enhancer	promoter	XXbac-BPG254F23.7
chr6	4345000	chr6	71875000	3.18E-03	super_enhancer	promoter	LYPLA1P3
chr6	4345000	chr6	105405000	2.79E-03	super_enhancer	promoter	LIN28B
chr6	4345000	chr6	108615000	4.13E-03	super_enhancer	promoter	LACE1
chr6	4345000	chr6	160185000	3.84E-03	super_enhancer	promoter	SOD2
chr6	4355000	chr6	5005000	6.42E-03	super_enhancer	promoter	RPP40
chr6	4355000	chr6	5085000	4.05E-03	super_enhancer	promoter	PPP1R3G
chr6	4355000	chr6	16265000	2.84E-02	super_enhancer	promoter	RP3-467D16.3
chr6	4355000	chr6	18265000	2.30E-02	super_enhancer	promoter	DEK
chr6	4355000	chr6	20995000	2.39E-02	super_enhancer	promoter	RNU6-150P
chr6	4355000	chr6	23855000	2.24E-02	super_enhancer	promoter	RP11-440J4.2

chr6	4355000	chr6	26205000	2.15E-02	super_enhancer	promoter	HIST1H4E
chr6	4355000	chr6	27115000	1.94E-02	super_enhancer	promoter	HIST1H2BK
chr6	4355000	chr6	35705000	1.58E-02	super_enhancer	promoter	RP3-510O8.4
chr6	4355000	chr6	36705000	1.37E-02	super_enhancer	promoter	RP3-431A14.4
chr6	4355000	chr6	43485000	1.21E-02	super_enhancer	promoter	YIPF3
chr6	4365000	chr6	15245000	2.85E-02	super_enhancer	promoter	RP11-560J1.2
chr6	4365000	chr6	17055000	3.11E-02	super_enhancer	promoter	AL133405.1
chr6	4365000	chr6	20995000	2.04E-02	super_enhancer	promoter	RNU6-150P
chr6	4365000	chr6	26045000	1.85E-02	super_enhancer	promoter	HIST1H3C
chr6	4365000	chr6	31635000	1.99E-02	super_enhancer	promoter	GPANK1
chr6	4365000	chr6	149565000	3.26E-03	super_enhancer	promoter	RP1-111D6.2
chr6	4375000	chr6	7505000	1.48E-02	super_enhancer	promoter	RP11-288G3.3
chr6	4375000	chr6	13825000	3.03E-02	super_enhancer	promoter	RP3-500L14.2
chr6	4375000	chr6	22085000	2.53E-04	super_enhancer	promoter	RN7SKP240
chr6	4375000	chr6	27835000	3.13E-02	super_enhancer	promoter	Z98744.2
chr6	4705000	chr6	6795000	2.01E-02	promoter	super_enhancer	CDYL
chr6	4705000	chr6	6815000	2.05E-02	promoter	super_enhancer	CDYL
chr6	5005000	chr6	16765000	3.03E-02	promoter	super_enhancer	RPP40
chr6	5005000	chr6	125945000	3.38E-03	promoter	super_enhancer	RPP40
chr6	5055000	chr6	11775000	4.53E-02	promoter	super_enhancer	RP11-428J1.4
chr6	5055000	chr6	44045000	7.67E-03	promoter	super_enhancer	RP11-428J1.4
chr6	5085000	chr6	6825000	4.11E-02	promoter	super_enhancer	PPP1R3G
chr6	5085000	chr6	10735000	4.52E-02	promoter	super_enhancer	PPP1R3G
chr6	5225000	chr6	6805000	4.36E-02	promoter	super_enhancer	AL162381.1

chr6	5225000	chr6	6815000	6.73E-03	promoter	super_enhancer	AL162381.1
chr6	5225000	chr6	14745000	3.24E-02	promoter	super_enhancer	AL162381.1
chr6	6685000	chr6	6805000	1.24E-02	promoter	super_enhancer	RP1-80N2.3
chr6	6685000	chr6	39185000	9.37E-03	promoter	super_enhancer	RP1-80N2.3
chr6	6795000	chr6	15315000	4.70E-02	super_enhancer	promoter	RNU6-522P
chr6	6795000	chr6	28105000	2.38E-02	super_enhancer	promoter	ZSCAN16-AS1
chr6	6795000	chr6	41635000	1.22E-02	super_enhancer	promoter	NPM1P51
chr6	6805000	chr6	7505000	3.64E-02	super_enhancer	promoter	RP11-288G3.3
chr6	6805000	chr6	13815000	3.91E-02	super_enhancer	promoter	MCUR1
chr6	6805000	chr6	18155000	2.49E-02	super_enhancer	promoter	TPMT
chr6	6805000	chr6	22595000	1.87E-02	super_enhancer	promoter	RP1-309H15.2
chr6	6805000	chr6	28425000	1.83E-02	super_enhancer	promoter	OR2E1P
chr6	6805000	chr6	76185000	3.80E-03	super_enhancer	promoter	RNU1-34P
chr6	6815000	chr6	15245000	3.97E-02	super_enhancer	promoter	RP11-560J1.2
chr6	6815000	chr6	16165000	4.09E-02	super_enhancer	promoter	RP1-13D10.3
chr6	6815000	chr6	28285000	1.98E-02	super_enhancer	promoter	RP5-874C20.7
chr6	6815000	chr6	33205000	1.85E-02	super_enhancer	promoter	HTATSF1P1
chr6	6815000	chr6	35265000	1.38E-02	super_enhancer	promoter	DEF6
chr6	6815000	chr6	55805000	4.83E-03	super_enhancer	promoter	NPM1P36
chr6	6815000	chr6	122965000	3.99E-03	super_enhancer	promoter	RP11-95G17.2
chr6	6825000	chr6	14005000	4.07E-02	super_enhancer	promoter	RP3-380E11.2
chr6	6825000	chr6	15325000	4.30E-02	super_enhancer	promoter	RNU6-645P
chr6	6825000	chr6	25025000	2.27E-02	super_enhancer	promoter	ASS1P1
chr6	6825000	chr6	26205000	2.41E-02	super_enhancer	promoter	HIST1H4E

chr6	6825000	chr6	28325000	2.40E-02	super_enhancer	promoter	ZSCAN31
chr6	6825000	chr6	31495000	2.66E-02	super_enhancer	promoter	RPL15P4
chr6	6825000	chr6	43675000	1.20E-02	super_enhancer	promoter	RP1-261G23.4
chr6	7505000	chr6	35485000	1.98E-02	promoter	super_enhancer	RP11-288G3.3
chr6	7505000	chr6	158735000	4.19E-03	promoter	super_enhancer	RP11-288G3.3
chr6	8065000	chr6	43925000	6.37E-03	promoter	super_enhancer	BLOC1S5-TXNDC5
chr6	9505000	chr6	10435000	2.77E-03	super_enhancer	promoter	LINC00518
chr6	9505000	chr6	13925000	4.03E-02	super_enhancer	promoter	RNF182
chr6	9505000	chr6	17705000	3.49E-02	super_enhancer	promoter	RP11-500C11.3
chr6	9505000	chr6	29445000	1.74E-02	super_enhancer	promoter	MAS1LP1
chr6	9505000	chr6	35575000	6.47E-05	super_enhancer	promoter	RP3-340B19.5
chr6	9515000	chr6	10215000	2.86E-02	super_enhancer	promoter	RP11-339A7.1
chr6	9515000	chr6	27925000	1.31E-02	super_enhancer	promoter	OR2B6
chr6	9515000	chr6	32595000	5.54E-03	super_enhancer	promoter	HLA-DQA1
chr6	9525000	chr6	15935000	4.35E-02	super_enhancer	promoter	ARPC3P5
chr6	9525000	chr6	31745000	2.25E-02	super_enhancer	promoter	VWA7
chr6	9525000	chr6	53285000	4.89E-03	super_enhancer	promoter	NANOGP3
chr6	9525000	chr6	123065000	2.97E-03	super_enhancer	promoter	RN7SL564P
chr6	9535000	chr6	10435000	3.20E-02	super_enhancer	promoter	LINC00518
chr6	9535000	chr6	15995000	4.47E-02	super_enhancer	promoter	RP11-401E14.2
chr6	9535000	chr6	29545000	2.08E-02	super_enhancer	promoter	TMEM183AP1
chr6	9535000	chr6	29975000	2.01E-02	super_enhancer	promoter	HCG4P3
chr6	9535000	chr6	42695000	1.10E-02	super_enhancer	promoter	ATP6V0CP3
chr6	10215000	chr6	30685000	2.26E-02	promoter	super_enhancer	RP11-339A7.1

chr6	10515000	chr6	10745000	7.55E-03	promoter	super_enhancer	RP11-360O19.4
chr6	10515000	chr6	10755000	3.40E-03	promoter	super_enhancer	RP11-360O19.4
chr6	10515000	chr6	42305000	1.28E-02	promoter	super_enhancer	RP11-360O19.4
chr6	10575000	chr6	26305000	2.13E-02	promoter	super_enhancer	RP11-360O19.5
chr6	10695000	chr6	30785000	2.29E-02	promoter	super_enhancer	C6orf52
chr6	10725000	chr6	21525000	3.36E-02	super_enhancer	promoter	RP11-204E9.1
chr6	10725000	chr6	29555000	2.51E-02	super_enhancer	promoter	OR2H2
chr6	10725000	chr6	29715000	3.08E-02	super_enhancer	promoter	HCG9P5
chr6	10725000	chr6	40315000	1.31E-02	super_enhancer	promoter	LINC00951
chr6	10735000	chr6	21525000	2.47E-02	super_enhancer	promoter	RP11-204E9.1
chr6	10735000	chr6	26205000	2.13E-02	super_enhancer	promoter	HIST1H4E
chr6	10735000	chr6	26365000	2.03E-02	super_enhancer	promoter	BTN3A2
chr6	10735000	chr6	27775000	1.91E-02	super_enhancer	promoter	HIST1H4PS1
chr6	10745000	chr6	26195000	3.29E-02	super_enhancer	promoter	HIST1H1PS1
chr6	10745000	chr6	29385000	1.70E-02	super_enhancer	promoter	OR12D1
chr6	10745000	chr6	34525000	2.04E-02	super_enhancer	promoter	SPDEF
chr6	10745000	chr6	53015000	7.89E-03	super_enhancer	promoter	GCM1
chr6	10755000	chr6	18265000	2.96E-02	super_enhancer	promoter	DEK
chr6	10755000	chr6	22215000	3.06E-02	super_enhancer	promoter	RP11-524C21.1
chr6	10755000	chr6	31865000	2.29E-02	super_enhancer	promoter	EHMT2
chr6	11775000	chr6	13575000	2.75E-02	super_enhancer	promoter	SIRT5
chr6	11775000	chr6	30665000	2.63E-02	super_enhancer	promoter	RPL7P4
chr6	13815000	chr6	31195000	2.71E-02	promoter	super_enhancer	MCUR1
chr6	13815000	chr6	44055000	1.05E-02	promoter	super_enhancer	MCUR1

chr6	13815000	chr6	85475000	4.73E-03	promoter	super_enhancer	MCUR1
chr6	13815000	chr6	165575000	2.83E-03	promoter	super_enhancer	MCUR1
chr6	13825000	chr6	158725000	2.34E-03	promoter	super_enhancer	RP3-500L14.2
chr6	13925000	chr6	31195000	2.32E-02	promoter	super_enhancer	RNF182
chr6	14005000	chr6	30775000	2.25E-02	promoter	super_enhancer	RP3-380E11.2
chr6	14005000	chr6	41715000	1.22E-02	promoter	super_enhancer	RP3-380E11.2
chr6	14005000	chr6	42285000	1.03E-02	promoter	super_enhancer	RP3-380E11.2
chr6	14285000	chr6	25185000	2.96E-02	promoter	super_enhancer	LINC01108
chr6	14735000	chr6	26225000	2.70E-02	super_enhancer	promoter	HIST1H3E
chr6	14735000	chr6	28155000	2.62E-02	super_enhancer	promoter	ZNF192P2
chr6	14735000	chr6	30845000	2.79E-02	super_enhancer	promoter	DDR1-AS1
chr6	14735000	chr6	31925000	2.79E-02	super_enhancer	promoter	MIR1236
chr6	14735000	chr6	149435000	4.29E-03	super_enhancer	promoter	RP11-365H23.1
chr6	14735000	chr6	150285000	4.44E-03	super_enhancer	promoter	ULBP1
chr6	14745000	chr6	26045000	2.86E-02	super_enhancer	promoter	HIST1H3C
chr6	14745000	chr6	26125000	2.65E-02	super_enhancer	promoter	HIST1H2BB
chr6	14745000	chr6	30615000	2.79E-02	super_enhancer	promoter	C6orf136
chr6	14745000	chr6	30845000	3.02E-02	super_enhancer	promoter	DDR1-AS1
chr6	15245000	chr6	31195000	2.97E-02	promoter	super_enhancer	RP11-560J1.2
chr6	15245000	chr6	42305000	1.33E-02	promoter	super_enhancer	RP11-560J1.2
chr6	15315000	chr6	158065000	4.65E-03	promoter	super_enhancer	RNU6-522P
chr6	15325000	chr6	25175000	2.56E-02	promoter	super_enhancer	RNU6-645P
chr6	15325000	chr6	26335000	3.22E-02	promoter	super_enhancer	RNU6-645P
chr6	15995000	chr6	31195000	3.66E-02	promoter	super_enhancer	RP11-401E14.2

chr6	16165000	chr6	26295000	3.42E-02	promoter	super_enhancer	RP1-13D10.3
chr6	16205000	chr6	26355000	3.08E-02	promoter	super_enhancer	RNU6-1114P
chr6	16205000	chr6	42295000	1.57E-02	promoter	super_enhancer	RNU6-1114P
chr6	16265000	chr6	26305000	2.25E-02	promoter	super_enhancer	RP3-467D16.3
chr6	16265000	chr6	37135000	1.51E-02	promoter	super_enhancer	RP3-467D16.3
chr6	16265000	chr6	73605000	4.07E-03	promoter	super_enhancer	RP3-467D16.3
chr6	16265000	chr6	151025000	3.79E-03	promoter	super_enhancer	RP3-467D16.3
chr6	16755000	chr6	28155000	2.84E-02	super_enhancer	promoter	ZNF192P2
chr6	16755000	chr6	28945000	2.95E-02	super_enhancer	promoter	RN7SL471P
chr6	16765000	chr6	22595000	3.87E-02	super_enhancer	promoter	RP1-309H15.2
chr6	16765000	chr6	24495000	4.27E-02	super_enhancer	promoter	GPLD1
chr6	16765000	chr6	25025000	3.32E-02	super_enhancer	promoter	ASS1P1
chr6	16765000	chr6	29445000	3.25E-02	super_enhancer	promoter	MAS1LP1
chr6	16765000	chr6	29555000	2.86E-02	super_enhancer	promoter	OR2H2
chr6	16765000	chr6	31495000	6.26E-04	super_enhancer	promoter	RPL15P4
chr6	16765000	chr6	31925000	3.55E-02	super_enhancer	promoter	MIR1236
chr6	16765000	chr6	35265000	1.74E-02	super_enhancer	promoter	DEF6
chr6	16765000	chr6	93435000	2.21E-03	super_enhancer	promoter	RP11-127B16.1
chr6	17705000	chr6	25175000	3.76E-02	promoter	super_enhancer	RP11-500C11.3
chr6	17705000	chr6	26295000	4.55E-02	promoter	super_enhancer	RP11-500C11.3
chr6	17705000	chr6	165985000	3.09E-03	promoter	super_enhancer	RP11-500C11.3
chr6	18305000	chr6	30815000	3.26E-02	promoter	super_enhancer	RP11-14J16.1
chr6	18305000	chr6	44055000	1.46E-02	promoter	super_enhancer	RP11-14J16.1
chr6	19735000	chr6	25185000	4.01E-02	promoter	super_enhancer	RP3-471C18.1

chr6	19735000	chr6	30775000	3.12E-02	promoter	super_enhancer	RP3-471C18.1
chr6	19735000	chr6	30785000	2.83E-02	promoter	super_enhancer	RP3-471C18.1
chr6	19735000	chr6	44045000	1.01E-02	promoter	super_enhancer	RP3-471C18.1
chr6	20535000	chr6	30885000	4.11E-02	promoter	super_enhancer	CDKAL1
chr6	20725000	chr6	26325000	3.85E-02	promoter	super_enhancer	RP3-348I23.3
chr6	20725000	chr6	39195000	1.34E-02	promoter	super_enhancer	RP3-348I23.3
chr6	20995000	chr6	26335000	4.88E-02	promoter	super_enhancer	RNU6-150P
chr6	20995000	chr6	26345000	4.76E-02	promoter	super_enhancer	RNU6-150P
chr6	21525000	chr6	39205000	1.67E-02	promoter	super_enhancer	RP11-204E9.1
chr6	21525000	chr6	151025000	4.10E-03	promoter	super_enhancer	RP11-204E9.1
chr6	21665000	chr6	41735000	2.65E-02	promoter	super_enhancer	CASC15
chr6	21665000	chr6	43915000	1.73E-02	promoter	super_enhancer	CASC15
chr6	22085000	chr6	44005000	1.06E-02	promoter	super_enhancer	RN7SKP240
chr6	22085000	chr6	151015000	3.26E-03	promoter	super_enhancer	RN7SKP240
chr6	22215000	chr6	39215000	1.96E-02	promoter	super_enhancer	RP11-524C21.1
chr6	22215000	chr6	158045000	4.02E-03	promoter	super_enhancer	RP11-524C21.1
chr6	22595000	chr6	42315000	1.17E-02	promoter	super_enhancer	RP1-309H15.2
chr6	23125000	chr6	39185000	1.24E-02	promoter	super_enhancer	RNU6-1060P
chr6	23855000	chr6	105595000	3.37E-03	promoter	super_enhancer	RP11-440J4.2
chr6	24125000	chr6	37135000	2.17E-02	promoter	super_enhancer	NRSN1
chr6	24775000	chr6	41705000	2.17E-02	promoter	super_enhancer	GMNN
chr6	25025000	chr6	26305000	4.07E-02	promoter	super_enhancer	ASS1P1
chr6	25025000	chr6	35485000	2.76E-02	promoter	super_enhancer	ASS1P1
chr6	25175000	chr6	25885000	5.31E-04	super_enhancer	promoter	RP1-139G21.4

chr6	25175000	chr6	30205000	4.91E-02	super_enhancer	promoter	TRIM26BP
chr6	25175000	chr6	44355000	1.14E-02	super_enhancer	promoter	CDC5L
chr6	25175000	chr6	115955000	1.83E-03	super_enhancer	promoter	RP11-282C5.1
chr6	25185000	chr6	25885000	8.23E-04	super_enhancer	promoter	RP1-139G21.4
chr6	25185000	chr6	26125000	1.69E-03	super_enhancer	promoter	HIST1H2BB
chr6	25185000	chr6	32375000	3.08E-02	super_enhancer	promoter	BTNL2
chr6	25185000	chr6	36095000	2.45E-02	super_enhancer	promoter	MAPK13
chr6	25185000	chr6	79945000	3.39E-03	super_enhancer	promoter	HMGN3
chr6	25185000	chr6	109705000	2.98E-03	super_enhancer	promoter	CD164
chr6	25755000	chr6	32195000	3.07E-02	promoter	super_enhancer	SLC17A4
chr6	25755000	chr6	44015000	1.38E-02	promoter	super_enhancer	SLC17A4
chr6	25885000	chr6	158855000	3.73E-03	promoter	super_enhancer	RP1-139G21.4
chr6	26035000	chr6	35475000	3.02E-02	promoter	super_enhancer	HIST1H2AB
chr6	26045000	chr6	26155000	2.03E-02	promoter	super_enhancer	HIST1H3C
chr6	26125000	chr6	42285000	1.75E-02	promoter	super_enhancer	HIST1H2BB
chr6	26125000	chr6	43925000	1.25E-02	promoter	super_enhancer	HIST1H2BB
chr6	26125000	chr6	43945000	1.31E-02	promoter	super_enhancer	HIST1H2BB
chr6	26125000	chr6	151005000	3.92E-03	promoter	super_enhancer	HIST1H2BB
chr6	26155000	chr6	27775000	3.00E-02	super_enhancer	promoter	HIST1H4PS1
chr6	26155000	chr6	27805000	3.65E-02	super_enhancer	promoter	HIST1H2BN
chr6	26155000	chr6	28155000	2.12E-02	super_enhancer	promoter	ZNF192P2
chr6	26155000	chr6	30295000	2.99E-03	super_enhancer	promoter	HCG17
chr6	26155000	chr6	33175000	3.54E-02	super_enhancer	promoter	MIR219A1
chr6	26155000	chr6	35705000	3.09E-02	super_enhancer	promoter	RP3-510O8.4

chr6	26155000	chr6	69925000	4.90E-03	super_enhancer	promoter	AL158051.1
chr6	26165000	chr6	27105000	1.25E-02	super_enhancer	promoter	HIST1H4I
chr6	26165000	chr6	27775000	3.68E-02	super_enhancer	promoter	HIST1H4PS1
chr6	26165000	chr6	27805000	4.46E-02	super_enhancer	promoter	HIST1H2BN
chr6	26165000	chr6	41255000	2.22E-02	super_enhancer	promoter	TREM1
chr6	26195000	chr6	26315000	3.62E-03	promoter	super_enhancer	HIST1H1PS1
chr6	26195000	chr6	26335000	5.51E-03	promoter	super_enhancer	HIST1H1PS1
chr6	26195000	chr6	26345000	2.82E-02	promoter	super_enhancer	HIST1H1PS1
chr6	26195000	chr6	41695000	2.63E-02	promoter	super_enhancer	HIST1H1PS1
chr6	26205000	chr6	26295000	3.36E-03	promoter	super_enhancer	HIST1H4E
chr6	26205000	chr6	26325000	3.71E-02	promoter	super_enhancer	HIST1H4E
chr6	26205000	chr6	42295000	2.12E-02	promoter	super_enhancer	HIST1H4E
chr6	26205000	chr6	149885000	5.15E-03	promoter	super_enhancer	HIST1H4E
chr6	26225000	chr6	26305000	4.25E-03	promoter	super_enhancer	HIST1H3E
chr6	26225000	chr6	26325000	1.01E-02	promoter	super_enhancer	HIST1H3E
chr6	26225000	chr6	34235000	3.24E-02	promoter	super_enhancer	HIST1H3E
chr6	26225000	chr6	42315000	1.85E-02	promoter	super_enhancer	HIST1H3E
chr6	26235000	chr6	26315000	3.53E-02	promoter	super_enhancer	HIST1H1D
chr6	26295000	chr6	27775000	4.58E-02	super_enhancer	promoter	HIST1H4PS1
chr6	26295000	chr6	28555000	1.85E-02	super_enhancer	promoter	RP5-1186N24.3
chr6	26295000	chr6	41755000	2.20E-02	super_enhancer	promoter	FRS3
chr6	26295000	chr6	42695000	2.36E-02	super_enhancer	promoter	ATP6V0CP3
chr6	26295000	chr6	96025000	3.51E-03	super_enhancer	promoter	MANEA-AS1
chr6	26295000	chr6	137815000	5.47E-03	super_enhancer	promoter	OLIG3

chr6	26305000	chr6	27865000	2.71E-03	super_enhancer	promoter	RNU7-26P
chr6	26305000	chr6	28555000	1.25E-02	super_enhancer	promoter	RP5-1186N24.3
chr6	26305000	chr6	44235000	1.56E-02	super_enhancer	promoter	NFKBIE
chr6	26305000	chr6	133085000	3.22E-03	super_enhancer	promoter	VNN2
chr6	26315000	chr6	27865000	3.42E-02	super_enhancer	promoter	RNU7-26P
chr6	26315000	chr6	28155000	2.35E-02	super_enhancer	promoter	ZNF192P2
chr6	26315000	chr6	28585000	1.07E-02	super_enhancer	promoter	ZBED9
chr6	26315000	chr6	37475000	2.02E-02	super_enhancer	promoter	RP1-153P14.8
chr6	26315000	chr6	45985000	1.39E-02	super_enhancer	promoter	RNU6-754P
chr6	26315000	chr6	52225000	8.94E-03	super_enhancer	promoter	PAQR8
chr6	26325000	chr6	29795000	3.78E-03	super_enhancer	promoter	HLA-G
chr6	26325000	chr6	158665000	2.88E-03	super_enhancer	promoter	SRP72P2
chr6	26335000	chr6	41755000	2.17E-02	super_enhancer	promoter	FRS3
chr6	26335000	chr6	46655000	1.19E-02	super_enhancer	promoter	TDRD6
chr6	26335000	chr6	163835000	2.91E-03	super_enhancer	promoter	CAHM
chr6	26345000	chr6	35705000	3.29E-02	super_enhancer	promoter	RP3-510O8.4
chr6	26345000	chr6	36515000	2.78E-02	super_enhancer	promoter	STK38
chr6	26345000	chr6	119255000	3.00E-03	super_enhancer	promoter	RP11-351A11.1
chr6	26345000	chr6	134375000	3.94E-03	super_enhancer	promoter	SLC2A12
chr6	26355000	chr6	26365000	1.98E-02	super_enhancer	promoter	BTN3A2
chr6	26355000	chr6	37225000	2.28E-02	super_enhancer	promoter	TBC1D22B
chr6	26355000	chr6	138785000	3.12E-03	super_enhancer	promoter	RP11-43P8.2
chr6	26355000	chr6	168595000	3.04E-03	super_enhancer	promoter	RP11-503C24.1
chr6	26365000	chr6	32195000	3.64E-02	promoter	super_enhancer	BTN3A2

chr6	26865000	chr6	43975000	1.12E-02	promoter	super_enhancer	POM121L6P
chr6	26865000	chr6	43995000	1.33E-02	promoter	super_enhancer	POM121L6P
chr6	27105000	chr6	30775000	5.15E-05	promoter	super_enhancer	HIST1H4I
chr6	27105000	chr6	37125000	2.01E-04	promoter	super_enhancer	HIST1H4I
chr6	27105000	chr6	39195000	1.53E-02	promoter	super_enhancer	HIST1H4I
chr6	27105000	chr6	158065000	2.79E-03	promoter	super_enhancer	HIST1H4I
chr6	27215000	chr6	37125000	3.55E-02	promoter	super_enhancer	PRSS16
chr6	27235000	chr6	32195000	4.59E-02	promoter	super_enhancer	XXbac-BPGBPG24O18.1
chr6	27235000	chr6	73605000	6.33E-03	promoter	super_enhancer	XXbac-BPGBPG24O18.1
chr6	27775000	chr6	44045000	1.44E-02	promoter	super_enhancer	HIST1H4PS1
chr6	27805000	chr6	41695000	2.58E-02	promoter	super_enhancer	HIST1H2BN
chr6	27865000	chr6	43985000	1.81E-02	promoter	super_enhancer	RNU7-26P
chr6	27865000	chr6	151085000	4.99E-03	promoter	super_enhancer	RNU7-26P
chr6	27865000	chr6	158045000	4.01E-03	promoter	super_enhancer	RNU7-26P
chr6	27925000	chr6	34225000	2.73E-02	promoter	super_enhancer	OR2B6
chr6	28105000	chr6	44055000	2.07E-02	promoter	super_enhancer	ZSCAN16-AS1
chr6	28155000	chr6	37135000	3.34E-02	promoter	super_enhancer	ZNF192P2
chr6	28325000	chr6	43915000	1.88E-02	promoter	super_enhancer	ZSCAN31
chr6	28325000	chr6	44055000	2.08E-02	promoter	super_enhancer	ZSCAN31
chr6	28415000	chr6	63535000	6.57E-03	promoter	super_enhancer	COX11P1
chr6	28425000	chr6	73575000	6.69E-03	promoter	super_enhancer	OR2E1P
chr6	28555000	chr6	43635000	2.59E-02	promoter	super_enhancer	RP5-1186N24.3
chr6	28585000	chr6	35485000	4.17E-02	promoter	super_enhancer	ZBED9
chr6	28585000	chr6	37135000	3.16E-02	promoter	super_enhancer	ZBED9

chr6	28945000	chr6	44045000	1.87E-02	promoter	super_enhancer	RN7SL471P
chr6	28955000	chr6	43975000	1.96E-02	promoter	super_enhancer	HCG16
chr6	28995000	chr6	42285000	1.80E-02	promoter	super_enhancer	OR2AD1P
chr6	29045000	chr6	133595000	3.07E-03	promoter	super_enhancer	SAR1P1
chr6	29425000	chr6	41725000	2.95E-02	promoter	super_enhancer	OR11A1
chr6	29425000	chr6	43985000	1.85E-02	promoter	super_enhancer	OR11A1
chr6	29545000	chr6	43625000	2.92E-02	promoter	super_enhancer	TMEM183AP1
chr6	29545000	chr6	151005000	4.98E-03	promoter	super_enhancer	TMEM183AP1
chr6	29555000	chr6	43945000	1.63E-02	promoter	super_enhancer	OR2H2
chr6	29605000	chr6	37125000	4.69E-02	promoter	super_enhancer	SUMO2P1
chr6	29605000	chr6	37135000	4.75E-02	promoter	super_enhancer	SUMO2P1
chr6	29605000	chr6	44035000	2.81E-02	promoter	super_enhancer	SUMO2P1
chr6	29695000	chr6	44075000	2.06E-02	promoter	super_enhancer	RPL23AP1
chr6	29715000	chr6	158855000	4.90E-03	promoter	super_enhancer	HCG9P5
chr6	29795000	chr6	42285000	2.34E-02	promoter	super_enhancer	HLA-G
chr6	29795000	chr6	44045000	1.73E-02	promoter	super_enhancer	HLA-G
chr6	29855000	chr6	43925000	1.67E-02	promoter	super_enhancer	HLA-H
chr6	29895000	chr6	43625000	2.85E-02	promoter	super_enhancer	HCG4B
chr6	29975000	chr6	41705000	3.02E-02	promoter	super_enhancer	HCG4P3
chr6	29975000	chr6	43995000	2.40E-02	promoter	super_enhancer	HCG4P3
chr6	30045000	chr6	43975000	2.03E-02	promoter	super_enhancer	RNF39
chr6	30045000	chr6	43985000	2.24E-02	promoter	super_enhancer	RNF39
chr6	30525000	chr6	44085000	2.27E-02	promoter	super_enhancer	GNL1
chr6	30595000	chr6	43985000	2.81E-02	promoter	super_enhancer	ATAT1

chr6	30615000	chr6	30885000	3.69E-02	promoter	super_enhancer	C6orf136
chr6	30615000	chr6	41735000	3.65E-02	promoter	super_enhancer	C6orf136
chr6	30665000	chr6	129675000	4.15E-03	promoter	super_enhancer	RPL7P4
chr6	30665000	chr6	150895000	6.12E-03	promoter	super_enhancer	RPL7P4
chr6	30685000	chr6	36915000	4.18E-02	super_enhancer	promoter	PI16
chr6	30685000	chr6	64345000	1.21E-02	super_enhancer	promoter	PHF3
chr6	30735000	chr6	30775000	2.10E-02	promoter	super_enhancer	HCG20
chr6	30735000	chr6	44045000	1.93E-02	promoter	super_enhancer	HCG20
chr6	30735000	chr6	44065000	1.54E-02	promoter	super_enhancer	HCG20
chr6	30775000	chr6	31165000	2.52E-03	super_enhancer	promoter	HCG27
chr6	30775000	chr6	31925000	4.41E-02	super_enhancer	promoter	MIR1236
chr6	30775000	chr6	33205000	1.36E-02	super_enhancer	promoter	HTATSF1P1
chr6	30775000	chr6	37515000	4.86E-02	super_enhancer	promoter	RP1-153P14.3
chr6	30775000	chr6	72595000	8.33E-03	super_enhancer	promoter	RIMS1
chr6	30775000	chr6	90585000	5.41E-03	super_enhancer	promoter	RP11-63K6.4
chr6	30785000	chr6	43425000	3.10E-02	super_enhancer	promoter	DLK2
chr6	30785000	chr6	97285000	4.86E-03	super_enhancer	promoter	GPR63
chr6	30785000	chr6	107235000	3.86E-03	super_enhancer	promoter	RP1-60O19.1
chr6	30785000	chr6	170125000	2.56E-03	super_enhancer	promoter	RP1-266L20.4
chr6	30815000	chr6	32005000	2.85E-02	super_enhancer	promoter	C4B-AS1
chr6	30815000	chr6	36665000	4.57E-02	super_enhancer	promoter	RAB44
chr6	30815000	chr6	42715000	2.76E-02	super_enhancer	promoter	TBCC
chr6	30815000	chr6	112195000	2.37E-03	super_enhancer	promoter	FYN
chr6	30815000	chr6	153665000	2.74E-03	super_enhancer	promoter	AL358134.1

chr6	30845000	chr6	43995000	4.10E-04	promoter	super_enhancer	DDR1-AS1
chr6	30885000	chr6	31125000	1.39E-02	super_enhancer	promoter	TCF19
chr6	30885000	chr6	31335000	3.81E-02	super_enhancer	promoter	DHFRP2
chr6	30885000	chr6	43485000	3.34E-02	super_enhancer	promoter	YIPF3
chr6	30885000	chr6	57255000	1.92E-02	super_enhancer	promoter	MIR548U
chr6	31125000	chr6	42315000	3.05E-02	promoter	super_enhancer	TCF19
chr6	31165000	chr6	42305000	3.42E-02	promoter	super_enhancer	HCG27
chr6	31195000	chr6	31325000	1.46E-02	super_enhancer	promoter	HLA-C
chr6	31195000	chr6	43295000	3.88E-02	super_enhancer	promoter	RP11-480N24.4
chr6	31195000	chr6	46405000	1.41E-02	super_enhancer	promoter	RP11-795J1.2
chr6	31195000	chr6	100875000	4.59E-03	super_enhancer	promoter	RP1-121G13.2
chr6	31205000	chr6	31345000	3.77E-02	super_enhancer	promoter	FGFR3P1
chr6	31325000	chr6	44025000	2.47E-02	promoter	super_enhancer	HLA-C
chr6	31325000	chr6	71405000	7.42E-03	promoter	super_enhancer	HLA-C
chr6	31335000	chr6	44075000	2.41E-02	promoter	super_enhancer	DHFRP2
chr6	31345000	chr6	41725000	4.18E-02	promoter	super_enhancer	FGFR3P1
chr6	31345000	chr6	43925000	2.16E-02	promoter	super_enhancer	FGFR3P1
chr6	31495000	chr6	41725000	4.44E-02	promoter	super_enhancer	RPL15P4
chr6	31495000	chr6	43635000	3.53E-02	promoter	super_enhancer	RPL15P4
chr6	31495000	chr6	150865000	4.70E-03	promoter	super_enhancer	RPL15P4
chr6	31515000	chr6	31795000	3.52E-03	promoter	super_enhancer	ATP6V1G2-DDX39B
chr6	31635000	chr6	39215000	4.16E-02	promoter	super_enhancer	GPANK1
chr6	31675000	chr6	31795000	3.66E-02	promoter	super_enhancer	LY6G6F
chr6	31675000	chr6	31945000	1.92E-02	promoter	super_enhancer	LY6G6F

chr6	31675000	chr6	43925000	2.27E-02	promoter	super_enhancer	LY6G6F
chr6	31675000	chr6	44075000	3.03E-02	promoter	super_enhancer	LY6G6F
chr6	31745000	chr6	31945000	2.21E-02	promoter	super_enhancer	VWA7
chr6	31745000	chr6	44025000	3.29E-02	promoter	super_enhancer	VWA7
chr6	31765000	chr6	43985000	2.31E-02	promoter	super_enhancer	VARS
chr6	31775000	chr6	44025000	3.07E-02	promoter	super_enhancer	LSM2
chr6	31895000	chr6	44035000	2.97E-02	promoter	super_enhancer	XXbac-BPG116M5.17
chr6	31925000	chr6	43635000	3.72E-02	promoter	super_enhancer	MIR1236
chr6	31925000	chr6	44005000	2.85E-02	promoter	super_enhancer	MIR1236
chr6	31925000	chr6	85405000	7.17E-03	promoter	super_enhancer	MIR1236
chr6	31945000	chr6	42715000	2.77E-02	super_enhancer	promoter	TBCC
chr6	31945000	chr6	126965000	3.04E-03	super_enhancer	promoter	PRELID1P1
chr6	31975000	chr6	41725000	1.82E-02	promoter	super_enhancer	AL645922.1
chr6	32005000	chr6	39185000	1.46E-02	promoter	super_enhancer	C4B-AS1
chr6	32175000	chr6	32675000	9.12E-03	super_enhancer	promoter	MTCO3P1
chr6	32175000	chr6	38895000	3.25E-02	super_enhancer	promoter	RP1-207H1.2
chr6	32175000	chr6	42695000	3.31E-02	super_enhancer	promoter	ATP6V0CP3
chr6	32175000	chr6	108445000	5.09E-03	super_enhancer	promoter	OSTM1-AS1
chr6	32195000	chr6	32785000	3.20E-02	super_enhancer	promoter	HLA-DOB
chr6	32195000	chr6	35435000	3.41E-03	super_enhancer	promoter	RPL10A
chr6	32195000	chr6	52285000	1.14E-02	super_enhancer	promoter	EFHC1
chr6	32195000	chr6	107055000	2.73E-03	super_enhancer	promoter	RNU6-527P
chr6	32675000	chr6	73575000	4.52E-03	promoter	super_enhancer	MTCO3P1
chr6	32685000	chr6	42305000	2.28E-02	promoter	super_enhancer	XXbac-BPG254F23.7

chr6	32905000	chr6	43915000	2.14E-02	promoter	super_enhancer	AL645941.1
chr6	33065000	chr6	43945000	1.49E-02	promoter	super_enhancer	HLA-DPA2
chr6	33075000	chr6	44025000	2.52E-02	promoter	super_enhancer	COL11A2P1
chr6	33175000	chr6	42305000	2.72E-02	promoter	super_enhancer	MIR219A1
chr6	33245000	chr6	39195000	3.13E-02	promoter	super_enhancer	B3GALT4
chr6	33255000	chr6	165585000	2.74E-03	promoter	super_enhancer	MIR6873
chr6	33335000	chr6	150875000	3.86E-03	promoter	super_enhancer	LYPLA2P1
chr6	33665000	chr6	34235000	3.95E-02	promoter	super_enhancer	MIR3934
chr6	33665000	chr6	43915000	1.73E-02	promoter	super_enhancer	MIR3934
chr6	33665000	chr6	158035000	3.05E-03	promoter	super_enhancer	MIR3934
chr6	33715000	chr6	44015000	2.09E-04	promoter	super_enhancer	IP6K3
chr6	34205000	chr6	43995000	2.03E-02	promoter	super_enhancer	HMGA1
chr6	34215000	chr6	43945000	1.59E-02	promoter	super_enhancer	RP11-513I15.6
chr6	34215000	chr6	158865000	2.83E-03	promoter	super_enhancer	RP11-513I15.6
chr6	34225000	chr6	109755000	2.83E-03	super_enhancer	promoter	AL109947.1
chr6	34225000	chr6	153665000	2.04E-03	super_enhancer	promoter	AL358134.1
chr6	34235000	chr6	40555000	3.30E-02	super_enhancer	promoter	LRFN2
chr6	34235000	chr6	44265000	2.01E-02	super_enhancer	promoter	TCTE1
chr6	34245000	chr6	43445000	2.23E-02	super_enhancer	promoter	TJAP1
chr6	34245000	chr6	52535000	1.26E-04	super_enhancer	promoter	TMEM14A
chr6	34395000	chr6	35475000	9.47E-03	promoter	super_enhancer	RPS10-NUDT3
chr6	34395000	chr6	44035000	2.45E-02	promoter	super_enhancer	RPS10-NUDT3
chr6	34395000	chr6	44065000	1.56E-02	promoter	super_enhancer	RPS10-NUDT3
chr6	34525000	chr6	41715000	3.86E-02	promoter	super_enhancer	SPDEF

chr6	34525000	chr6	158705000	3.19E-03	promoter	super_enhancer	SPDEF
chr6	34545000	chr6	41735000	4.09E-02	promoter	super_enhancer	RP3-391O22.3
chr6	34665000	chr6	41695000	3.47E-02	promoter	super_enhancer	C6orf106
chr6	35465000	chr6	42305000	3.09E-02	promoter	super_enhancer	TEAD3
chr6	35465000	chr6	43945000	1.85E-02	promoter	super_enhancer	TEAD3
chr6	35465000	chr6	150865000	2.87E-03	promoter	super_enhancer	TEAD3
chr6	35475000	chr6	36165000	5.04E-03	super_enhancer	promoter	RP1-179N16.6
chr6	35475000	chr6	70455000	7.57E-03	super_enhancer	promoter	GAPDHP42
chr6	35485000	chr6	35705000	8.05E-04	super_enhancer	promoter	RP3-510O8.4
chr6	35485000	chr6	36165000	4.99E-02	super_enhancer	promoter	RP1-179N16.6
chr6	35485000	chr6	41195000	4.74E-02	super_enhancer	promoter	TREML4
chr6	35485000	chr6	82245000	5.49E-03	super_enhancer	promoter	RP1-300G12.2
chr6	35485000	chr6	150115000	3.79E-03	super_enhancer	promoter	BTBD10P2
chr6	35575000	chr6	42285000	3.16E-02	promoter	super_enhancer	RP3-340B19.5
chr6	35575000	chr6	158035000	3.66E-03	promoter	super_enhancer	RP3-340B19.5
chr6	35705000	chr6	41705000	4.35E-02	promoter	super_enhancer	RP3-510O8.4
chr6	35705000	chr6	44025000	3.05E-02	promoter	super_enhancer	RP3-510O8.4
chr6	35705000	chr6	105605000	3.79E-03	promoter	super_enhancer	RP3-510O8.4
chr6	35765000	chr6	84625000	7.32E-03	promoter	super_enhancer	CLPS
chr6	36095000	chr6	43995000	2.88E-02	promoter	super_enhancer	MAPK13
chr6	36305000	chr6	43985000	2.26E-02	promoter	super_enhancer	C6orf222
chr6	36305000	chr6	63535000	6.97E-03	promoter	super_enhancer	C6orf222
chr6	36355000	chr6	44025000	3.12E-02	promoter	super_enhancer	RP1-50J22.4
chr6	36515000	chr6	44015000	2.47E-02	promoter	super_enhancer	STK38

chr6	36665000	chr6	42285000	3.67E-02	promoter	super_enhancer	RAB44
chr6	36705000	chr6	41695000	4.70E-02	promoter	super_enhancer	RP3-431A14.4
chr6	36705000	chr6	44055000	2.85E-02	promoter	super_enhancer	RP3-431A14.4
chr6	36705000	chr6	44075000	2.92E-02	promoter	super_enhancer	RP3-431A14.4
chr6	36705000	chr6	149885000	4.68E-03	promoter	super_enhancer	RP3-431A14.4
chr6	36915000	chr6	41705000	3.93E-02	promoter	super_enhancer	PI16
chr6	36915000	chr6	42285000	3.08E-02	promoter	super_enhancer	PI16
chr6	36915000	chr6	44035000	2.35E-02	promoter	super_enhancer	PI16
chr6	37135000	chr6	43675000	3.55E-02	super_enhancer	promoter	RP1-261G23.4
chr6	37135000	chr6	44265000	2.71E-02	super_enhancer	promoter	TCTE1
chr6	37135000	chr6	45295000	1.74E-02	super_enhancer	promoter	RUNX2
chr6	37135000	chr6	109705000	3.59E-03	super_enhancer	promoter	CD164
chr6	37225000	chr6	41705000	4.90E-02	promoter	super_enhancer	TBC1D22B
chr6	37265000	chr6	165575000	2.30E-03	promoter	super_enhancer	RP4-744I24.4
chr6	37475000	chr6	39195000	1.13E-02	promoter	super_enhancer	RP1-153P14.8
chr6	37475000	chr6	41715000	4.99E-02	promoter	super_enhancer	RP1-153P14.8
chr6	37475000	chr6	44005000	2.45E-02	promoter	super_enhancer	RP1-153P14.8
chr6	37515000	chr6	39195000	2.03E-02	promoter	super_enhancer	RP1-153P14.3
chr6	37515000	chr6	43945000	2.90E-02	promoter	super_enhancer	RP1-153P14.3
chr6	38555000	chr6	39205000	3.07E-02	promoter	super_enhancer	RP1-319M7.2
chr6	38895000	chr6	39185000	1.43E-02	promoter	super_enhancer	RP1-207H1.2
chr6	39195000	chr6	43675000	4.43E-02	super_enhancer	promoter	RP1-261G23.4
chr6	39195000	chr6	44265000	5.15E-04	super_enhancer	promoter	TCTE1
chr6	39195000	chr6	46715000	1.82E-02	super_enhancer	promoter	ANKRD66

chr6	39195000	chr6	72295000	1.09E-02	super_enhancer	promoter	AL354933.1
chr6	39195000	chr6	167685000	2.18E-03	super_enhancer	promoter	UNC93A
chr6	39215000	chr6	57255000	1.77E-02	super_enhancer	promoter	MIR548U
chr6	39215000	chr6	73765000	8.73E-03	super_enhancer	promoter	PGAM1P10
chr6	39215000	chr6	116575000	2.77E-03	super_enhancer	promoter	TSPYL4
chr6	40245000	chr6	43995000	4.41E-02	promoter	super_enhancer	RP11-552E20.1
chr6	40375000	chr6	158055000	2.48E-03	promoter	super_enhancer	RNU6-250P
chr6	41255000	chr6	41735000	2.93E-02	promoter	super_enhancer	TREM1
chr6	41565000	chr6	41735000	4.63E-02	promoter	super_enhancer	MIR4641
chr6	41565000	chr6	105605000	3.87E-03	promoter	super_enhancer	MIR4641
chr6	41705000	chr6	50825000	2.67E-02	super_enhancer	promoter	RPS17P5
chr6	41715000	chr6	45985000	4.99E-02	super_enhancer	promoter	RNU6-754P
chr6	41725000	chr6	52225000	2.07E-02	super_enhancer	promoter	PAQR8
chr6	41725000	chr6	57255000	2.53E-02	super_enhancer	promoter	MIR548U
chr6	41735000	chr6	53215000	2.19E-02	super_enhancer	promoter	ELOVL5
chr6	41735000	chr6	105645000	5.05E-03	super_enhancer	promoter	AL359709.1
chr6	41755000	chr6	71415000	9.34E-03	promoter	super_enhancer	FRS3
chr6	42285000	chr6	53215000	1.77E-02	super_enhancer	promoter	ELOVL5
chr6	42295000	chr6	43295000	2.68E-03	super_enhancer	promoter	RP11-480N24.4
chr6	42295000	chr6	150225000	4.63E-03	super_enhancer	promoter	RP11-350J20.9
chr6	42295000	chr6	158355000	3.01E-03	super_enhancer	promoter	RNU6-786P
chr6	42305000	chr6	73765000	1.03E-02	super_enhancer	promoter	PGAM1P10
chr6	42305000	chr6	125695000	3.18E-03	super_enhancer	promoter	RP11-735G4.1
chr6	42715000	chr6	151085000	4.48E-03	promoter	super_enhancer	TBCC

chr6	42925000	chr6	158715000	3.13E-03	promoter	super_enhancer	RPL24P4
chr6	43395000	chr6	73565000	8.49E-03	promoter	super_enhancer	ABCC10
chr6	43445000	chr6	43975000	3.16E-02	promoter	super_enhancer	TJAP1
chr6	43625000	chr6	74715000	6.90E-03	super_enhancer	promoter	TXNP7
chr6	43635000	chr6	53795000	1.97E-02	super_enhancer	promoter	MLIP
chr6	43635000	chr6	74105000	9.05E-03	super_enhancer	promoter	OOEP
chr6	43675000	chr6	43935000	1.35E-02	promoter	super_enhancer	RP1-261G23.4
chr6	43675000	chr6	44045000	2.73E-03	promoter	super_enhancer	RP1-261G23.4
chr6	43935000	chr6	55805000	1.05E-02	super_enhancer	promoter	NPM1P36
chr6	43945000	chr6	85865000	4.41E-03	super_enhancer	promoter	RP3-455E7.1
chr6	43985000	chr6	109825000	3.05E-03	super_enhancer	promoter	AL109947.2
chr6	43985000	chr6	151255000	3.09E-03	super_enhancer	promoter	RP1-99E18.2
chr6	43995000	chr6	74235000	1.01E-02	super_enhancer	promoter	EEF1A1
chr6	43995000	chr6	87865000	6.81E-03	super_enhancer	promoter	RP11-393I2.4
chr6	43995000	chr6	169655000	2.90E-03	super_enhancer	promoter	THBS2
chr6	44005000	chr6	71305000	9.66E-03	super_enhancer	promoter	RP11-134K13.4
chr6	44005000	chr6	82245000	4.97E-03	super_enhancer	promoter	RP1-300G12.2
chr6	44025000	chr6	50605000	1.51E-02	super_enhancer	promoter	RP1-28O17.1
chr6	44025000	chr6	52775000	1.93E-02	super_enhancer	promoter	GSTA3
chr6	44025000	chr6	53285000	1.64E-02	super_enhancer	promoter	NANOGP3
chr6	44025000	chr6	91155000	6.25E-03	super_enhancer	promoter	AL391559.1
chr6	44035000	chr6	52225000	1.83E-02	super_enhancer	promoter	PAQR8
chr6	44035000	chr6	53015000	1.93E-02	super_enhancer	promoter	GCM1
chr6	44045000	chr6	53215000	1.56E-02	super_enhancer	promoter	ELOVL5

chr6	44045000	chr6	105745000	3.31E-03	super_enhancer	promoter	RP3-355L5.3
chr6	44045000	chr6	126925000	2.01E-03	super_enhancer	promoter	VIM2P
chr6	44065000	chr6	44185000	6.46E-03	super_enhancer	promoter	RP1-302G2.5
chr6	44065000	chr6	44235000	1.46E-03	super_enhancer	promoter	NFKBIE
chr6	44065000	chr6	44355000	8.96E-04	super_enhancer	promoter	CDC5L
chr6	44075000	chr6	44185000	5.65E-04	super_enhancer	promoter	RP1-302G2.5
chr6	44075000	chr6	44235000	1.63E-02	super_enhancer	promoter	NFKBIE
chr6	44075000	chr6	53215000	2.01E-02	super_enhancer	promoter	ELOVL5
chr6	44075000	chr6	71785000	8.26E-03	super_enhancer	promoter	BECN1P2
chr6	44085000	chr6	44095000	4.12E-03	super_enhancer	promoter	MRPL14
chr6	44085000	chr6	44235000	2.81E-02	super_enhancer	promoter	NFKBIE
chr6	44185000	chr6	158735000	3.02E-03	promoter	super_enhancer	RP1-302G2.5
chr6	45065000	chr6	73585000	5.19E-03	promoter	super_enhancer	RP11-491H9.4
chr6	46715000	chr6	84645000	4.25E-03	promoter	super_enhancer	ANKRD66
chr6	49755000	chr6	71415000	8.34E-03	promoter	super_enhancer	PGK2
chr6	50825000	chr6	73585000	1.07E-02	promoter	super_enhancer	RPS17P5
chr6	50825000	chr6	165575000	2.70E-03	promoter	super_enhancer	RPS17P5
chr6	52225000	chr6	73595000	8.07E-03	promoter	super_enhancer	PAQR8
chr6	52955000	chr6	125915000	2.39E-03	promoter	super_enhancer	RN7SL244P
chr6	54635000	chr6	73575000	6.65E-03	promoter	super_enhancer	KRASP1
chr6	55805000	chr6	133595000	1.46E-03	promoter	super_enhancer	NPM1P36
chr6	56735000	chr6	85475000	9.63E-03	promoter	super_enhancer	RPL17P26
chr6	63525000	chr6	100035000	5.71E-03	super_enhancer	promoter	RP1-199J3.6
chr6	63535000	chr6	76185000	1.10E-02	super_enhancer	promoter	RNU1-34P

chr6	63535000	chr6	81085000	7.54E-03	super_enhancer	promoter	RPL17P25
chr6	63535000	chr6	90585000	6.51E-03	super_enhancer	promoter	RP11-63K6.4
chr6	70455000	chr6	133615000	3.97E-03	promoter	super_enhancer	GAPDHP42
chr6	71125000	chr6	125945000	4.40E-03	promoter	super_enhancer	RP11-462G2.2
chr6	71375000	chr6	125915000	3.86E-03	promoter	super_enhancer	SLC25A6P6
chr6	71375000	chr6	149885000	3.61E-03	promoter	super_enhancer	SLC25A6P6
chr6	71395000	chr6	71875000	1.05E-03	super_enhancer	promoter	LYPLA1P3
chr6	71395000	chr6	79945000	1.18E-02	super_enhancer	promoter	HMGN3
chr6	71395000	chr6	109755000	5.04E-03	super_enhancer	promoter	AL109947.1
chr6	71395000	chr6	113755000	3.16E-03	super_enhancer	promoter	RP1-124C6.1
chr6	71395000	chr6	130335000	3.76E-03	super_enhancer	promoter	L3MBTL3
chr6	71405000	chr6	76175000	2.07E-02	super_enhancer	promoter	RP11-415D17.1
chr6	71405000	chr6	111185000	5.80E-03	super_enhancer	promoter	RP3-418A9.3
chr6	71875000	chr6	125955000	3.84E-03	promoter	super_enhancer	LYPLA1P3
chr6	73565000	chr6	80665000	1.59E-02	super_enhancer	promoter	GAPDHP63
chr6	73575000	chr6	82245000	1.68E-02	super_enhancer	promoter	RP1-300G12.2
chr6	73585000	chr6	82525000	1.44E-02	super_enhancer	promoter	LINC01526
chr6	73585000	chr6	166655000	2.62E-03	super_enhancer	promoter	GNG5P1
chr6	73605000	chr6	137115000	4.08E-03	super_enhancer	promoter	MAP3K5
chr6	74235000	chr6	133615000	5.26E-03	promoter	super_enhancer	EEF1A1
chr6	74405000	chr6	85455000	1.60E-02	promoter	super_enhancer	CD109
chr6	74405000	chr6	125925000	5.37E-03	promoter	super_enhancer	CD109
chr6	75995000	chr6	84635000	1.42E-02	promoter	super_enhancer	TMEM30A
chr6	76295000	chr6	151035000	3.93E-03	promoter	super_enhancer	RP11-474L11.3

chr6	76575000	chr6	158725000	1.96E-03	promoter	super_enhancer	RNA5SP209
chr6	83905000	chr6	150865000	3.19E-03	promoter	super_enhancer	PGM3
chr6	84625000	chr6	97285000	1.53E-02	super_enhancer	promoter	GPR63
chr6	84625000	chr6	105745000	1.04E-02	super_enhancer	promoter	RP3-355L5.3
chr6	84625000	chr6	141245000	3.36E-03	super_enhancer	promoter	RP11-471B18.1
chr6	84625000	chr6	155585000	2.10E-03	super_enhancer	promoter	CLDN20
chr6	84635000	chr6	98165000	6.73E-03	super_enhancer	promoter	RP1-104O17.3
chr6	84635000	chr6	106905000	9.23E-03	super_enhancer	promoter	RP3-335E1.1
chr6	84645000	chr6	91055000	2.38E-02	super_enhancer	promoter	RP1-45N11.1
chr6	85405000	chr6	85995000	1.91E-02	super_enhancer	promoter	RP11-30P6.1
chr6	85405000	chr6	90585000	2.90E-02	super_enhancer	promoter	RP11-63K6.4
chr6	85405000	chr6	137115000	5.30E-03	super_enhancer	promoter	MAP3K5
chr6	85475000	chr6	105215000	1.29E-02	super_enhancer	promoter	RNU6-897P
chr6	85475000	chr6	108615000	1.55E-02	super_enhancer	promoter	LACE1
chr6	85475000	chr6	116785000	1.03E-02	super_enhancer	promoter	RP1-93H18.6
chr6	85475000	chr6	168195000	3.48E-03	super_enhancer	promoter	RP3-431P23.5
chr6	85865000	chr6	151015000	3.61E-03	promoter	super_enhancer	RP3-455E7.1
chr6	86305000	chr6	142015000	6.32E-03	promoter	super_enhancer	SNX14
chr6	86355000	chr6	125905000	6.98E-03	promoter	super_enhancer	SYNCRIP
chr6	87805000	chr6	151055000	3.39E-03	promoter	super_enhancer	CGA
chr6	88155000	chr6	151005000	3.90E-03	promoter	super_enhancer	ST13P16
chr6	89855000	chr6	150875000	4.08E-03	promoter	super_enhancer	PM20D2
chr6	89855000	chr6	158055000	2.56E-03	promoter	super_enhancer	PM20D2
chr6	90025000	chr6	105605000	1.13E-02	promoter	super_enhancer	GABRR2

chr6	93435000	chr6	125935000	4.55E-03	promoter	super_enhancer	RP11-127B16.1
chr6	96025000	chr6	149885000	5.46E-03	promoter	super_enhancer	MANEA-AS1
chr6	97285000	chr6	158705000	3.58E-03	promoter	super_enhancer	GPR63
chr6	100015000	chr6	158065000	4.23E-03	promoter	super_enhancer	RP1-199J3.7
chr6	105585000	chr6	158715000	3.88E-03	promoter	super_enhancer	BVES
chr6	105595000	chr6	113835000	2.02E-02	super_enhancer	promoter	AL513123.1
chr6	105595000	chr6	150945000	8.22E-03	super_enhancer	promoter	RP11-136K14.2
chr6	105605000	chr6	119255000	1.28E-02	super_enhancer	promoter	RP11-351A11.1
chr6	105645000	chr6	129695000	7.20E-03	promoter	super_enhancer	AL359709.1
chr6	107055000	chr6	151045000	5.88E-03	promoter	super_enhancer	RNU6-527P
chr6	108445000	chr6	125935000	1.68E-02	promoter	super_enhancer	OSTM1-AS1
chr6	108445000	chr6	158025000	4.45E-03	promoter	super_enhancer	OSTM1-AS1
chr6	108615000	chr6	125905000	1.38E-02	promoter	super_enhancer	LACE1
chr6	109705000	chr6	125945000	1.40E-02	promoter	super_enhancer	CD164
chr6	109705000	chr6	158065000	5.85E-03	promoter	super_enhancer	CD164
chr6	109805000	chr6	125935000	1.60E-02	promoter	super_enhancer	ZBTB24
chr6	111135000	chr6	151045000	7.52E-03	promoter	super_enhancer	AMD1
chr6	112685000	chr6	158725000	5.27E-03	promoter	super_enhancer	KRT18P65
chr6	113755000	chr6	151085000	6.55E-03	promoter	super_enhancer	RP1-124C6.1
chr6	114225000	chr6	150875000	7.06E-03	promoter	super_enhancer	RP11-544L8__B.4
chr6	115955000	chr6	151005000	6.00E-03	promoter	super_enhancer	RP11-282C5.1
chr6	116575000	chr6	151045000	7.55E-03	promoter	super_enhancer	TSPYL4
chr6	116785000	chr6	165985000	3.30E-03	promoter	super_enhancer	RP1-93H18.6
chr6	117925000	chr6	129685000	1.48E-02	promoter	super_enhancer	GOPC

chr6	119255000	chr6	151085000	9.53E-03	promoter	super_enhancer	RP11-351A11.1
chr6	120335000	chr6	151025000	7.33E-03	promoter	super_enhancer	MIR3144
chr6	121675000	chr6	125915000	3.45E-02	promoter	super_enhancer	RNU6-1286P
chr6	121775000	chr6	125925000	3.54E-02	promoter	super_enhancer	RNU4-35P
chr6	121975000	chr6	133615000	2.02E-02	promoter	super_enhancer	SLC25A5P7
chr6	123065000	chr6	158025000	4.94E-03	promoter	super_enhancer	RN7SL564P
chr6	124125000	chr6	158035000	8.40E-03	promoter	super_enhancer	NKAIN2
chr6	125285000	chr6	133605000	2.09E-02	promoter	super_enhancer	RNF217-AS1
chr6	125695000	chr6	150895000	1.34E-02	promoter	super_enhancer	RP11-735G4.1
chr6	125695000	chr6	151015000	1.18E-02	promoter	super_enhancer	RP11-735G4.1
chr6	125905000	chr6	129875000	3.46E-02	super_enhancer	promoter	RP1-69D17.4
chr6	125915000	chr6	149435000	1.43E-02	super_enhancer	promoter	RP11-365H23.1
chr6	125925000	chr6	131385000	3.28E-02	super_enhancer	promoter	EPB41L2
chr6	125925000	chr6	133085000	2.39E-02	super_enhancer	promoter	VNN2
chr6	125925000	chr6	137815000	2.34E-02	super_enhancer	promoter	OLIG3
chr6	125925000	chr6	138785000	1.46E-02	super_enhancer	promoter	RP11-43P8.2
chr6	125925000	chr6	150245000	1.34E-02	super_enhancer	promoter	RAET1G
chr6	125925000	chr6	150285000	1.30E-02	super_enhancer	promoter	ULBP1
chr6	125925000	chr6	153665000	6.14E-03	super_enhancer	promoter	AL358134.1
chr6	125925000	chr6	158345000	6.02E-03	super_enhancer	promoter	HSPE1P26
chr6	125935000	chr6	149915000	1.15E-02	super_enhancer	promoter	SNORA2
chr6	129495000	chr6	130335000	3.67E-02	super_enhancer	promoter	L3MBTL3
chr6	129495000	chr6	134715000	2.33E-02	super_enhancer	promoter	CHCHD2P4
chr6	129495000	chr6	151255000	7.25E-03	super_enhancer	promoter	RP1-99E18.2

chr6	129675000	chr6	135425000	3.00E-02	super_enhancer	promoter	HBS1L
chr6	129695000	chr6	138725000	1.53E-02	super_enhancer	promoter	MARCKSL1P2
chr6	129875000	chr6	133605000	4.17E-02	promoter	super_enhancer	RP1-69D17.4
chr6	130335000	chr6	150885000	2.07E-02	promoter	super_enhancer	L3MBTL3
chr6	130755000	chr6	150875000	1.61E-02	promoter	super_enhancer	AL137251.1
chr6	131385000	chr6	158845000	9.13E-03	promoter	super_enhancer	EPB41L2
chr6	132405000	chr6	151055000	9.85E-03	promoter	super_enhancer	RP3-523C21.2
chr6	133135000	chr6	139375000	3.42E-02	promoter	super_enhancer	RPS12
chr6	133595000	chr6	149435000	1.65E-02	super_enhancer	promoter	RP11-365H23.1
chr6	133605000	chr6	134375000	1.96E-02	super_enhancer	promoter	SLC2A12
chr6	133605000	chr6	138785000	3.34E-02	super_enhancer	promoter	RP11-43P8.2
chr6	133605000	chr6	150225000	2.07E-02	super_enhancer	promoter	RP11-350J20.9
chr6	133615000	chr6	150255000	1.87E-02	super_enhancer	promoter	RP11-244K5.6
chr6	134215000	chr6	150875000	2.61E-02	promoter	super_enhancer	TARID
chr6	134215000	chr6	158065000	1.81E-02	promoter	super_enhancer	TARID
chr6	135425000	chr6	158835000	1.11E-02	promoter	super_enhancer	HBS1L
chr6	136665000	chr6	150875000	2.03E-02	promoter	super_enhancer	AL023284.1
chr6	137295000	chr6	158705000	1.17E-02	promoter	super_enhancer	RPL35AP3
chr6	137815000	chr6	158055000	1.60E-02	promoter	super_enhancer	OLIG3
chr6	138315000	chr6	151015000	2.16E-02	promoter	super_enhancer	RPSAP42
chr6	138785000	chr6	151085000	2.31E-02	promoter	super_enhancer	RP11-43P8.2
chr6	139015000	chr6	150885000	2.89E-02	promoter	super_enhancer	NHSL1
chr6	139095000	chr6	150895000	2.89E-02	promoter	super_enhancer	RP3-509I19.11
chr6	140175000	chr6	165985000	6.03E-03	promoter	super_enhancer	RP5-899B16.1

chr6	141005000	chr6	142015000	2.44E-02	promoter	super_enhancer	MIR4465
chr6	141175000	chr6	151025000	2.15E-02	promoter	super_enhancer	RP3-460G2.2
chr6	141245000	chr6	151065000	2.16E-02	promoter	super_enhancer	RP11-471B18.1
chr6	142015000	chr6	153305000	1.87E-02	super_enhancer	promoter	FBXO5
chr6	142505000	chr6	149885000	3.40E-02	promoter	super_enhancer	AL033522.1
chr6	144185000	chr6	150895000	3.86E-02	promoter	super_enhancer	ZC2HC1B
chr6	145785000	chr6	151035000	3.61E-02	promoter	super_enhancer	RNU1-33P
chr6	146865000	chr6	151025000	4.98E-02	promoter	super_enhancer	RAB32
chr6	148845000	chr6	158045000	2.21E-02	promoter	super_enhancer	AL033378.1
chr6	148845000	chr6	166005000	8.42E-03	promoter	super_enhancer	AL033378.1
chr6	149885000	chr6	163475000	1.57E-02	super_enhancer	promoter	RP1-257A15.1
chr6	149895000	chr6	150225000	4.49E-02	super_enhancer	promoter	RP11-350J20.9
chr6	149895000	chr6	158355000	2.45E-02	super_enhancer	promoter	RNU6-786P
chr6	149895000	chr6	168085000	1.32E-02	super_enhancer	promoter	RP11-351J23.1
chr6	149915000	chr6	158715000	2.61E-04	promoter	super_enhancer	SNORA2
chr6	149915000	chr6	166005000	9.98E-03	promoter	super_enhancer	SNORA2
chr6	150115000	chr6	165985000	1.07E-02	promoter	super_enhancer	BTBD10P2
chr6	150225000	chr6	158035000	3.71E-02	promoter	super_enhancer	RP11-350J20.9
chr6	150225000	chr6	158735000	2.79E-02	promoter	super_enhancer	RP11-350J20.9
chr6	150245000	chr6	151055000	4.59E-02	promoter	super_enhancer	RAET1G
chr6	150285000	chr6	158025000	2.28E-02	promoter	super_enhancer	ULBP1
chr6	150285000	chr6	158045000	3.53E-02	promoter	super_enhancer	ULBP1
chr6	150285000	chr6	158865000	3.08E-02	promoter	super_enhancer	ULBP1
chr6	150855000	chr6	170125000	1.24E-02	super_enhancer	promoter	RP1-266L20.4

chr6	150875000	chr6	157745000	3.43E-02	super_enhancer	promoter	TMEM242
chr6	150885000	chr6	151735000	4.76E-02	super_enhancer	promoter	HSPA8P15
chr6	150895000	chr6	150945000	4.01E-02	super_enhancer	promoter	RP11-136K14.2
chr6	150895000	chr6	166655000	2.16E-02	super_enhancer	promoter	GNG5P1
chr6	150945000	chr6	158045000	4.00E-02	promoter	super_enhancer	RP11-136K14.2
chr6	150945000	chr6	158705000	2.92E-02	promoter	super_enhancer	RP11-136K14.2
chr6	151015000	chr6	164125000	1.87E-02	super_enhancer	promoter	RP1-230L10.1
chr6	151035000	chr6	167525000	1.73E-02	super_enhancer	promoter	CCR6
chr6	153325000	chr6	158835000	2.24E-02	promoter	super_enhancer	MTRF1L
chr6	153605000	chr6	165595000	1.01E-02	promoter	super_enhancer	RP11-475C16.1
chr6	153665000	chr6	158065000	3.41E-02	promoter	super_enhancer	AL358134.1
chr6	157745000	chr6	158025000	1.66E-02	promoter	super_enhancer	TMEM242
chr6	157745000	chr6	158035000	8.79E-04	promoter	super_enhancer	TMEM242
chr6	157795000	chr6	158025000	2.69E-02	promoter	super_enhancer	RP3-395C13.1
chr6	157795000	chr6	158045000	2.64E-02	promoter	super_enhancer	RP3-395C13.1
chr6	158025000	chr6	163835000	1.73E-02	super_enhancer	promoter	CAHM
chr6	158035000	chr6	158345000	5.05E-03	super_enhancer	promoter	HSPE1P26
chr6	158035000	chr6	170895000	1.34E-02	super_enhancer	promoter	PDCD2
chr6	158045000	chr6	169655000	2.03E-02	super_enhancer	promoter	THBS2
chr6	158065000	chr6	158355000	3.99E-02	super_enhancer	promoter	RNU6-786P
chr6	158065000	chr6	169655000	2.06E-02	super_enhancer	promoter	THBS2
chr6	158735000	chr6	170895000	1.11E-02	super_enhancer	promoter	PDCD2
chr6	158845000	chr6	166655000	2.16E-02	super_enhancer	promoter	GNG5P1
chr6	158855000	chr6	169655000	2.15E-02	super_enhancer	promoter	THBS2

chr6	160585000	chr6	165585000	3.16E-02	promoter	super_enhancer	RP11-317M22.1
chr6	160585000	chr6	166005000	2.08E-02	promoter	super_enhancer	RP11-317M22.1
chr6	161695000	chr6	165595000	2.53E-02	promoter	super_enhancer	AGPAT4
chr7	175000	chr7	825000	2.22E-03	promoter	super_enhancer	AC093627.11
chr7	175000	chr7	159015000	2.99E-03	promoter	super_enhancer	AC093627.11
chr7	195000	chr7	48095000	6.78E-03	promoter	super_enhancer	AC093627.12
chr7	515000	chr7	19185000	8.65E-03	super_enhancer	promoter	FERD3L
chr7	515000	chr7	99065000	2.99E-03	super_enhancer	promoter	PTCD1
chr7	515000	chr7	152455000	2.91E-03	super_enhancer	promoter	RN7SL845P
chr7	525000	chr7	3085000	7.63E-03	super_enhancer	promoter	CARD11
chr7	525000	chr7	29235000	1.42E-02	super_enhancer	promoter	CPVL
chr7	525000	chr7	104885000	3.12E-03	super_enhancer	promoter	RP4-778K6.1
chr7	535000	chr7	765000	2.63E-02	super_enhancer	promoter	DNAAF5
chr7	535000	chr7	995000	3.32E-02	super_enhancer	promoter	ADAP1
chr7	535000	chr7	129415000	4.85E-03	super_enhancer	promoter	MIR96
chr7	535000	chr7	129985000	3.60E-03	super_enhancer	promoter	CPA5
chr7	545000	chr7	765000	3.48E-02	super_enhancer	promoter	DNAAF5
chr7	545000	chr7	135345000	3.50E-03	super_enhancer	promoter	AC093107.7
chr7	795000	chr7	55865000	6.56E-03	super_enhancer	promoter	CICP12
chr7	795000	chr7	55955000	8.06E-03	super_enhancer	promoter	ZNF713
chr7	795000	chr7	96655000	2.95E-03	super_enhancer	promoter	DLX5
chr7	795000	chr7	100495000	3.63E-03	super_enhancer	promoter	ACHE
chr7	805000	chr7	30545000	1.07E-02	super_enhancer	promoter	GGCT
chr7	805000	chr7	44165000	6.88E-03	super_enhancer	promoter	POLD2

chr7	805000	chr7	47835000	5.61E-03	super_enhancer	promoter	C7orf69
chr7	805000	chr7	111595000	3.26E-03	super_enhancer	promoter	RNA5SP237
chr7	815000	chr7	12535000	1.84E-02	super_enhancer	promoter	AC005281.1
chr7	815000	chr7	37415000	8.18E-03	super_enhancer	promoter	RPS10P14
chr7	815000	chr7	99065000	3.49E-03	super_enhancer	promoter	PTCD1
chr7	835000	chr7	66205000	4.05E-03	super_enhancer	promoter	RP11-792A8.4
chr7	835000	chr7	73605000	3.02E-03	super_enhancer	promoter	MIR590
chr7	855000	chr7	6435000	3.53E-02	promoter	super_enhancer	SUN1
chr7	855000	chr7	73845000	3.45E-03	promoter	super_enhancer	SUN1
chr7	915000	chr7	48075000	6.12E-03	promoter	super_enhancer	GET4
chr7	1015000	chr7	2785000	1.58E-02	promoter	super_enhancer	COX19
chr7	1015000	chr7	6295000	4.92E-02	promoter	super_enhancer	COX19
chr7	1015000	chr7	22735000	1.56E-02	promoter	super_enhancer	COX19
chr7	1515000	chr7	16685000	1.46E-02	super_enhancer	promoter	ANKMY2
chr7	1515000	chr7	32935000	1.02E-02	super_enhancer	promoter	KBTBD2
chr7	1515000	chr7	44925000	6.24E-03	super_enhancer	promoter	PURB
chr7	1515000	chr7	73605000	3.38E-03	super_enhancer	promoter	MIR590
chr7	1515000	chr7	98475000	3.61E-03	super_enhancer	promoter	TRRAP
chr7	1535000	chr7	1545000	3.64E-02	super_enhancer	promoter	INTS1
chr7	1535000	chr7	2435000	4.18E-03	super_enhancer	promoter	RP4-607J2.1
chr7	1535000	chr7	30115000	9.29E-03	super_enhancer	promoter	AC007285.7
chr7	1535000	chr7	65305000	2.85E-03	super_enhancer	promoter	RP13-254B10.1
chr7	1535000	chr7	101935000	3.18E-03	super_enhancer	promoter	MIR4285
chr7	1535000	chr7	127235000	4.52E-03	super_enhancer	promoter	GCC1

chr7	1535000	chr7	128865000	3.53E-03	super_enhancer	promoter	AHCYL2
chr7	1535000	chr7	152065000	2.99E-03	super_enhancer	promoter	SEPT7P6
chr7	2395000	chr7	151755000	2.75E-03	promoter	super_enhancer	SNX8
chr7	2525000	chr7	9655000	1.87E-02	super_enhancer	promoter	GAPDHP68
chr7	2525000	chr7	26215000	1.00E-02	super_enhancer	promoter	CTB-119C2.1
chr7	2535000	chr7	6875000	4.28E-02	super_enhancer	promoter	OR7E39P
chr7	2535000	chr7	65875000	3.19E-03	super_enhancer	promoter	GTF2IP9
chr7	2595000	chr7	4695000	2.06E-02	promoter	super_enhancer	BRAT1
chr7	2595000	chr7	54985000	5.18E-03	promoter	super_enhancer	BRAT1
chr7	2765000	chr7	7295000	4.44E-02	super_enhancer	promoter	AC005532.5
chr7	2765000	chr7	22765000	1.21E-02	super_enhancer	promoter	IL6
chr7	2765000	chr7	39855000	6.81E-03	super_enhancer	promoter	AC072061.2
chr7	2765000	chr7	44375000	5.07E-03	super_enhancer	promoter	CAMK2B
chr7	2765000	chr7	66205000	3.89E-03	super_enhancer	promoter	RP11-792A8.4
chr7	2765000	chr7	128215000	2.59E-03	super_enhancer	promoter	RP11-274B21.14
chr7	2765000	chr7	139025000	2.88E-03	super_enhancer	promoter	C7orf55-LUC7L2
chr7	2775000	chr7	16685000	1.35E-02	super_enhancer	promoter	ANKMY2
chr7	2775000	chr7	30565000	9.90E-03	super_enhancer	promoter	AC005154.7
chr7	2775000	chr7	56755000	4.48E-03	super_enhancer	promoter	RP11-10F11.3
chr7	2785000	chr7	6875000	4.37E-02	super_enhancer	promoter	OR7E39P
chr7	2785000	chr7	23055000	9.48E-03	super_enhancer	promoter	FAM126A
chr7	2785000	chr7	25165000	6.81E-03	super_enhancer	promoter	CYCS
chr7	2785000	chr7	33945000	5.98E-03	super_enhancer	promoter	BMPER
chr7	2785000	chr7	64315000	1.80E-03	super_enhancer	promoter	SEPHS1P1

chr7	2785000	chr7	148975000	3.31E-03	super_enhancer	promoter	RP4-800G7.3
chr7	2795000	chr7	3215000	4.01E-02	super_enhancer	promoter	AC091801.1
chr7	2795000	chr7	6875000	4.76E-02	super_enhancer	promoter	OR7E39P
chr7	2795000	chr7	65955000	3.45E-03	super_enhancer	promoter	GS1-124K5.2
chr7	2795000	chr7	116255000	2.34E-03	super_enhancer	promoter	LINC01510
chr7	2795000	chr7	130125000	2.70E-03	super_enhancer	promoter	RP11-2E11.5
chr7	2805000	chr7	2885000	4.00E-03	super_enhancer	promoter	GNA12
chr7	2805000	chr7	5185000	6.29E-03	super_enhancer	promoter	ZNF890P
chr7	2805000	chr7	22815000	9.48E-03	super_enhancer	promoter	AC073072.7
chr7	2805000	chr7	23605000	9.58E-03	super_enhancer	promoter	AC006026.9
chr7	2805000	chr7	44105000	5.35E-03	super_enhancer	promoter	PGAM2
chr7	2805000	chr7	46185000	3.46E-03	super_enhancer	promoter	ZNF619P1
chr7	2865000	chr7	5085000	1.21E-02	super_enhancer	promoter	RBAK
chr7	2865000	chr7	36765000	9.01E-03	super_enhancer	promoter	AOAH
chr7	2865000	chr7	37415000	7.49E-03	super_enhancer	promoter	RPS10P14
chr7	2865000	chr7	38385000	6.21E-03	super_enhancer	promoter	TRGV5P
chr7	2865000	chr7	66345000	2.86E-03	super_enhancer	promoter	GTF2IP23
chr7	2865000	chr7	73185000	3.02E-03	super_enhancer	promoter	CLDN3
chr7	2875000	chr7	56665000	4.63E-03	super_enhancer	promoter	RP11-760D2.10
chr7	2875000	chr7	100255000	3.35E-03	super_enhancer	promoter	ACTL6B
chr7	2885000	chr7	73835000	3.95E-03	promoter	super_enhancer	GNA12
chr7	3215000	chr7	48095000	7.85E-03	promoter	super_enhancer	AC091801.1
chr7	3215000	chr7	155955000	3.23E-03	promoter	super_enhancer	AC091801.1
chr7	3215000	chr7	159015000	3.16E-03	promoter	super_enhancer	AC091801.1

chr7	4685000	chr7	66445000	3.52E-03	super_enhancer	promoter	RN7SL43P
chr7	4685000	chr7	96635000	1.69E-03	super_enhancer	promoter	DLX6
chr7	4695000	chr7	55955000	8.36E-03	super_enhancer	promoter	ZNF713
chr7	4695000	chr7	65325000	3.65E-03	super_enhancer	promoter	RNU6-973P
chr7	4695000	chr7	129895000	3.45E-03	super_enhancer	promoter	RP11-190G13.4
chr7	4705000	chr7	30115000	1.15E-02	super_enhancer	promoter	AC007285.7
chr7	4705000	chr7	39895000	6.60E-03	super_enhancer	promoter	RWDD4P2
chr7	4705000	chr7	129395000	3.80E-03	super_enhancer	promoter	RNA5SP244
chr7	4715000	chr7	12725000	2.71E-02	super_enhancer	promoter	CTD-2320J21.1
chr7	4715000	chr7	50245000	8.11E-03	super_enhancer	promoter	AC020743.3
chr7	4715000	chr7	55865000	7.33E-03	super_enhancer	promoter	CICP12
chr7	4715000	chr7	94955000	2.87E-03	super_enhancer	promoter	PON1
chr7	4715000	chr7	129415000	5.25E-03	super_enhancer	promoter	MIR96
chr7	4725000	chr7	9765000	3.31E-02	super_enhancer	promoter	AC060834.2
chr7	4725000	chr7	35735000	1.13E-02	super_enhancer	promoter	HERPUD2
chr7	4725000	chr7	41745000	6.57E-03	super_enhancer	promoter	AC005027.3
chr7	4735000	chr7	39605000	8.44E-03	super_enhancer	promoter	YAE1D1
chr7	4735000	chr7	64125000	2.98E-03	super_enhancer	promoter	ZNF107
chr7	4735000	chr7	65325000	3.50E-03	super_enhancer	promoter	RNU6-973P
chr7	4745000	chr7	23245000	1.64E-02	super_enhancer	promoter	AC005082.12
chr7	4745000	chr7	38395000	6.87E-03	super_enhancer	promoter	TRGV4
chr7	4745000	chr7	150975000	4.05E-03	super_enhancer	promoter	SMARCD3
chr7	4815000	chr7	5415000	3.24E-02	promoter	super_enhancer	AP5Z1
chr7	4815000	chr7	44735000	7.71E-03	promoter	super_enhancer	AP5Z1

chr7	4815000	chr7	131185000	3.76E-03	promoter	super_enhancer	AP5Z1
chr7	4905000	chr7	47595000	8.70E-03	promoter	super_enhancer	AC092610.12
chr7	4905000	chr7	131165000	3.77E-03	promoter	super_enhancer	AC092610.12
chr7	4905000	chr7	131635000	4.65E-03	promoter	super_enhancer	AC092610.12
chr7	5315000	chr7	73825000	3.83E-03	promoter	super_enhancer	AC093376.1
chr7	5395000	chr7	40155000	1.05E-02	super_enhancer	promoter	RP5-1147A1.1
chr7	5395000	chr7	41745000	7.98E-03	super_enhancer	promoter	AC005027.3
chr7	5395000	chr7	103125000	3.05E-03	super_enhancer	promoter	RN7SKP86
chr7	5395000	chr7	150885000	5.15E-03	super_enhancer	promoter	ASB10
chr7	5415000	chr7	26315000	1.84E-02	super_enhancer	promoter	AC010677.5
chr7	5415000	chr7	29845000	1.66E-02	super_enhancer	promoter	WIPF3
chr7	5415000	chr7	36765000	1.56E-02	super_enhancer	promoter	AOAH
chr7	5415000	chr7	93345000	2.51E-03	super_enhancer	promoter	MIR4652
chr7	5415000	chr7	123565000	3.86E-03	super_enhancer	promoter	SPAM1
chr7	5415000	chr7	141505000	2.69E-03	super_enhancer	promoter	MTND1P3
chr7	5465000	chr7	44645000	1.07E-02	promoter	super_enhancer	TNRC18
chr7	5515000	chr7	17645000	1.78E-02	promoter	super_enhancer	AC092171.4
chr7	5515000	chr7	155965000	3.79E-03	promoter	super_enhancer	AC092171.4
chr7	5535000	chr7	30635000	1.38E-02	super_enhancer	promoter	AC005154.6
chr7	5555000	chr7	12445000	3.37E-02	super_enhancer	promoter	VWDE
chr7	5555000	chr7	26315000	1.98E-02	super_enhancer	promoter	AC010677.5
chr7	5555000	chr7	63045000	3.31E-03	super_enhancer	promoter	TNRC18P2
chr7	5555000	chr7	73135000	5.98E-03	super_enhancer	promoter	STX1A
chr7	5555000	chr7	75705000	4.45E-03	super_enhancer	promoter	AC005077.14

chr7	6145000	chr7	6385000	3.87E-02	promoter	super_enhancer	USP42
chr7	6225000	chr7	155965000	4.03E-03	promoter	super_enhancer	AC004895.1
chr7	6285000	chr7	12445000	2.59E-02	super_enhancer	promoter	VWDE
chr7	6285000	chr7	22705000	1.79E-02	super_enhancer	promoter	AC002480.2
chr7	6285000	chr7	23145000	1.52E-02	super_enhancer	promoter	KLHL7-AS1
chr7	6285000	chr7	44375000	6.33E-03	super_enhancer	promoter	CAMK2B
chr7	6295000	chr7	8195000	1.22E-02	super_enhancer	promoter	AC007009.2
chr7	6295000	chr7	36185000	8.54E-03	super_enhancer	promoter	RP11-377H23.1
chr7	6305000	chr7	127235000	5.23E-03	super_enhancer	promoter	GCC1
chr7	6315000	chr7	12445000	4.86E-04	super_enhancer	promoter	VWDE
chr7	6315000	chr7	19745000	1.94E-02	super_enhancer	promoter	MIR3146
chr7	6315000	chr7	99725000	4.55E-03	super_enhancer	promoter	RP11-506M12.1
chr7	6315000	chr7	139045000	3.66E-03	super_enhancer	promoter	RP11-634H22.1
chr7	6375000	chr7	44375000	8.34E-03	super_enhancer	promoter	CAMK2B
chr7	6385000	chr7	12545000	2.72E-02	super_enhancer	promoter	AC013470.8
chr7	6385000	chr7	20875000	1.68E-02	super_enhancer	promoter	LINC01162
chr7	6385000	chr7	55945000	8.63E-03	super_enhancer	promoter	RP11-419M24.5
chr7	6385000	chr7	102715000	4.99E-03	super_enhancer	promoter	FBXL13
chr7	6395000	chr7	98835000	3.60E-03	super_enhancer	promoter	MYH16
chr7	6395000	chr7	149095000	3.63E-03	super_enhancer	promoter	AC004941.3
chr7	6405000	chr7	45145000	1.14E-02	super_enhancer	promoter	SNORA5A
chr7	6405000	chr7	56175000	9.67E-03	super_enhancer	promoter	CHCHD2
chr7	6405000	chr7	102005000	2.68E-03	super_enhancer	promoter	RP11-163E9.2
chr7	6405000	chr7	102715000	5.01E-03	super_enhancer	promoter	FBXL13

chr7	6405000	chr7	102985000	3.96E-03	super_enhancer	promoter	DNAJC2
chr7	6405000	chr7	150775000	5.26E-03	super_enhancer	promoter	RP11-148K1.12
chr7	6435000	chr7	43965000	7.20E-03	super_enhancer	promoter	UBE2D4
chr7	6435000	chr7	64485000	3.02E-03	super_enhancer	promoter	RNU6-1229P
chr7	6435000	chr7	99155000	2.98E-03	super_enhancer	promoter	ZNF655
chr7	6435000	chr7	140635000	2.42E-03	super_enhancer	promoter	RP4-813F11.3
chr7	6445000	chr7	12445000	3.27E-02	super_enhancer	promoter	VWDE
chr7	6445000	chr7	29665000	1.06E-04	super_enhancer	promoter	TMSB4XP3
chr7	6445000	chr7	35985000	1.01E-02	super_enhancer	promoter	SEPT7P3
chr7	6445000	chr7	84295000	2.29E-03	super_enhancer	promoter	AC092022.1
chr7	6455000	chr7	36765000	1.62E-02	super_enhancer	promoter	AOAH
chr7	6455000	chr7	55955000	1.13E-02	super_enhancer	promoter	ZNF713
chr7	6455000	chr7	65325000	4.94E-03	super_enhancer	promoter	RNU6-973P
chr7	6455000	chr7	66355000	5.11E-03	super_enhancer	promoter	RNU6-1254P
chr7	6455000	chr7	130475000	4.49E-03	super_enhancer	promoter	RP11-36B6.1
chr7	6525000	chr7	17625000	2.80E-02	promoter	super_enhancer	DAGLB
chr7	6525000	chr7	23475000	2.25E-02	promoter	super_enhancer	DAGLB
chr7	6525000	chr7	47575000	1.02E-02	promoter	super_enhancer	DAGLB
chr7	6655000	chr7	17625000	2.77E-02	promoter	super_enhancer	ZNF853
chr7	6655000	chr7	101105000	4.89E-03	promoter	super_enhancer	ZNF853
chr7	6875000	chr7	17615000	1.49E-02	promoter	super_enhancer	OR7E39P
chr7	6965000	chr7	17605000	1.52E-04	promoter	super_enhancer	RP11-740N7.4
chr7	6965000	chr7	100435000	3.44E-03	promoter	super_enhancer	RP11-740N7.4
chr7	7575000	chr7	44735000	6.37E-03	promoter	super_enhancer	COL28A1

chr7	9635000	chr7	17645000	1.34E-02	promoter	super_enhancer	AC079756.1
chr7	9635000	chr7	23475000	1.13E-02	promoter	super_enhancer	AC079756.1
chr7	12445000	chr7	44625000	5.52E-03	promoter	super_enhancer	VWDE
chr7	12915000	chr7	55135000	3.79E-03	promoter	super_enhancer	RN7SKP228
chr7	15015000	chr7	44615000	3.96E-03	promoter	super_enhancer	DGKB
chr7	16635000	chr7	157305000	2.31E-03	promoter	super_enhancer	GS1-166A23.2
chr7	16685000	chr7	47605000	6.31E-03	promoter	super_enhancer	ANKMY2
chr7	16685000	chr7	48085000	7.75E-03	promoter	super_enhancer	ANKMY2
chr7	17605000	chr7	54015000	3.84E-03	super_enhancer	promoter	RP11-806J6.1
chr7	17605000	chr7	127235000	2.71E-03	super_enhancer	promoter	GCC1
chr7	17615000	chr7	32535000	1.61E-02	super_enhancer	promoter	LSM5
chr7	17615000	chr7	54625000	5.15E-03	super_enhancer	promoter	VSTM2A-OT1
chr7	17625000	chr7	37145000	8.49E-03	super_enhancer	promoter	AC009196.1
chr7	17635000	chr7	20135000	4.02E-02	super_enhancer	promoter	RP11-486P11.1
chr7	17635000	chr7	20655000	3.23E-02	super_enhancer	promoter	ABCB5
chr7	17635000	chr7	31725000	1.01E-02	super_enhancer	promoter	PPP1R17
chr7	17635000	chr7	40815000	6.03E-03	super_enhancer	promoter	AC005160.3
chr7	17645000	chr7	22815000	2.25E-02	super_enhancer	promoter	AC073072.7
chr7	17645000	chr7	23695000	1.74E-02	super_enhancer	promoter	AC006026.12
chr7	17645000	chr7	99215000	2.16E-03	super_enhancer	promoter	ZSCAN25
chr7	19745000	chr7	22725000	3.86E-02	promoter	super_enhancer	MIR3146
chr7	20135000	chr7	23475000	3.06E-02	promoter	super_enhancer	RP11-486P11.1
chr7	20335000	chr7	76335000	1.72E-03	promoter	super_enhancer	AC099342.1
chr7	21255000	chr7	22575000	2.12E-03	promoter	super_enhancer	RN7SL542P

chr7	22565000	chr7	29845000	1.63E-02	super_enhancer	promoter	WIPF3
chr7	22565000	chr7	36345000	8.96E-03	super_enhancer	promoter	AC006960.5
chr7	22565000	chr7	65875000	3.44E-03	super_enhancer	promoter	GTF2IP9
chr7	22585000	chr7	22815000	2.39E-02	super_enhancer	promoter	AC073072.7
chr7	22585000	chr7	23145000	2.90E-03	super_enhancer	promoter	KLHL7-AS1
chr7	22585000	chr7	24295000	7.14E-03	super_enhancer	promoter	AC004485.3
chr7	22585000	chr7	35985000	1.17E-02	super_enhancer	promoter	SEPT7P3
chr7	22595000	chr7	22765000	2.78E-03	super_enhancer	promoter	IL6
chr7	22595000	chr7	23275000	1.60E-02	super_enhancer	promoter	GPNMB
chr7	22595000	chr7	29845000	2.09E-02	super_enhancer	promoter	WIPF3
chr7	22595000	chr7	135335000	2.42E-03	super_enhancer	promoter	RP11-751K21.1
chr7	22705000	chr7	48095000	1.11E-02	promoter	super_enhancer	AC002480.2
chr7	22725000	chr7	27245000	2.45E-02	super_enhancer	promoter	HOTTIP_4
chr7	22725000	chr7	36185000	8.96E-03	super_enhancer	promoter	RP11-377H23.1
chr7	22725000	chr7	44925000	6.40E-03	super_enhancer	promoter	PURB
chr7	22725000	chr7	64125000	2.58E-03	super_enhancer	promoter	ZNF107
chr7	22725000	chr7	138485000	2.22E-03	super_enhancer	promoter	ATP6V0A4
chr7	22735000	chr7	23245000	4.88E-02	super_enhancer	promoter	AC005082.12
chr7	22735000	chr7	38065000	1.24E-02	super_enhancer	promoter	SFRP4
chr7	22735000	chr7	38385000	1.37E-02	super_enhancer	promoter	TRGV5P
chr7	22735000	chr7	38395000	1.12E-02	super_enhancer	promoter	TRGV4
chr7	22735000	chr7	38695000	1.64E-02	super_enhancer	promoter	KRT8P20
chr7	22815000	chr7	151715000	2.53E-03	promoter	super_enhancer	AC073072.7
chr7	23145000	chr7	47595000	9.61E-03	promoter	super_enhancer	KLHL7-AS1

chr7	23275000	chr7	44635000	1.16E-02	promoter	super_enhancer	GPNMB
chr7	23475000	chr7	39855000	1.21E-02	super_enhancer	promoter	AC072061.2
chr7	23475000	chr7	65305000	3.86E-03	super_enhancer	promoter	RP13-254B10.1
chr7	23475000	chr7	137775000	2.72E-03	super_enhancer	promoter	RN7SKP223
chr7	23695000	chr7	47595000	8.31E-03	promoter	super_enhancer	AC006026.12
chr7	26315000	chr7	55125000	9.32E-03	promoter	super_enhancer	AC010677.5
chr7	27035000	chr7	54975000	9.31E-03	promoter	super_enhancer	SKAP2
chr7	27215000	chr7	100435000	2.41E-03	promoter	super_enhancer	HOXA9
chr7	29845000	chr7	54965000	1.16E-02	promoter	super_enhancer	WIPF3
chr7	30115000	chr7	44745000	1.42E-02	promoter	super_enhancer	AC007285.7
chr7	30635000	chr7	47565000	1.16E-02	promoter	super_enhancer	AC005154.6
chr7	30635000	chr7	54975000	1.21E-02	promoter	super_enhancer	AC005154.6
chr7	32535000	chr7	74485000	6.91E-03	promoter	super_enhancer	LSM5
chr7	32935000	chr7	55055000	1.16E-02	promoter	super_enhancer	KBTBD2
chr7	32935000	chr7	55085000	1.51E-02	promoter	super_enhancer	KBTBD2
chr7	35295000	chr7	151715000	3.11E-03	promoter	super_enhancer	TBX20
chr7	35755000	chr7	131185000	3.37E-03	promoter	super_enhancer	AC018647.3
chr7	36135000	chr7	55135000	1.26E-02	promoter	super_enhancer	RP11-196O2.1
chr7	37415000	chr7	55075000	1.84E-02	promoter	super_enhancer	RPS10P14
chr7	38385000	chr7	44615000	2.25E-02	promoter	super_enhancer	TRGV5P
chr7	38395000	chr7	47575000	1.29E-02	promoter	super_enhancer	TRGV4
chr7	39855000	chr7	73835000	6.95E-03	promoter	super_enhancer	AC072061.2
chr7	39895000	chr7	55125000	1.17E-02	promoter	super_enhancer	RWDD4P2
chr7	41745000	chr7	48075000	2.22E-02	promoter	super_enhancer	AC005027.3

chr7	43965000	chr7	131545000	2.21E-03	promoter	super_enhancer	UBE2D4
chr7	44105000	chr7	47575000	4.88E-02	promoter	super_enhancer	PGAM2
chr7	44105000	chr7	55135000	1.67E-02	promoter	super_enhancer	PGAM2
chr7	44165000	chr7	44615000	4.39E-02	promoter	super_enhancer	POLD2
chr7	44165000	chr7	48095000	4.73E-02	promoter	super_enhancer	POLD2
chr7	44615000	chr7	44835000	8.37E-03	super_enhancer	promoter	PPIA
chr7	44615000	chr7	80805000	4.05E-03	super_enhancer	promoter	AC005008.2
chr7	44615000	chr7	117645000	2.73E-03	super_enhancer	promoter	AC003084.2
chr7	44625000	chr7	45145000	1.30E-05	super_enhancer	promoter	SNORA5A
chr7	44635000	chr7	44835000	4.33E-02	super_enhancer	promoter	PPIA
chr7	44635000	chr7	45145000	9.10E-03	super_enhancer	promoter	SNORA5A
chr7	44635000	chr7	57295000	1.57E-02	super_enhancer	promoter	RNU7-157P
chr7	44645000	chr7	113055000	2.95E-03	super_enhancer	promoter	RP11-703P11.1
chr7	44745000	chr7	44835000	7.53E-03	super_enhancer	promoter	PPIA
chr7	44745000	chr7	98835000	4.28E-03	super_enhancer	promoter	MYH16
chr7	44745000	chr7	99065000	4.79E-03	super_enhancer	promoter	PTCD1
chr7	44745000	chr7	99755000	4.48E-03	super_enhancer	promoter	MIR4658
chr7	44745000	chr7	155755000	1.91E-03	super_enhancer	promoter	AC021218.2
chr7	45145000	chr7	101115000	4.55E-03	promoter	super_enhancer	SNORA5A
chr7	47565000	chr7	66385000	1.00E-02	super_enhancer	promoter	TMEM248
chr7	47575000	chr7	56595000	2.16E-02	super_enhancer	promoter	RP11-760D2.4
chr7	47575000	chr7	57715000	2.00E-02	super_enhancer	promoter	RP11-368M16.7
chr7	47575000	chr7	75115000	5.55E-03	super_enhancer	promoter	AC006014.7
chr7	47575000	chr7	121375000	2.52E-03	super_enhancer	promoter	RN7SKP277

chr7	47585000	chr7	66205000	1.17E-02	super_enhancer	promoter	RP11-792A8.4
chr7	47595000	chr7	55955000	3.04E-02	super_enhancer	promoter	ZNF713
chr7	47605000	chr7	66355000	1.02E-02	super_enhancer	promoter	RNU6-1254P
chr7	47605000	chr7	75545000	8.81E-03	super_enhancer	promoter	MIR4651
chr7	48085000	chr7	65305000	1.19E-02	super_enhancer	promoter	RP13-254B10.1
chr7	48095000	chr7	63425000	8.40E-03	super_enhancer	promoter	VN1R35P
chr7	48095000	chr7	94955000	4.28E-03	super_enhancer	promoter	PON1
chr7	50315000	chr7	54965000	3.08E-02	promoter	super_enhancer	AC020743.4
chr7	51385000	chr7	116675000	3.50E-03	promoter	super_enhancer	COBL
chr7	51455000	chr7	54975000	3.62E-02	promoter	super_enhancer	CTD-2021A8.2
chr7	53255000	chr7	54975000	1.86E-02	promoter	super_enhancer	HAUS6P1
chr7	54975000	chr7	75625000	1.78E-02	super_enhancer	promoter	TMEM120A
chr7	54975000	chr7	128805000	4.71E-03	super_enhancer	promoter	RNY1P11
chr7	55055000	chr7	79175000	5.36E-03	super_enhancer	promoter	AC091813.2
chr7	55055000	chr7	106505000	5.70E-03	super_enhancer	promoter	PIK3CG
chr7	55065000	chr7	64255000	1.89E-02	super_enhancer	promoter	ZNF138
chr7	55065000	chr7	73135000	1.70E-02	super_enhancer	promoter	STX1A
chr7	55065000	chr7	75465000	1.47E-02	super_enhancer	promoter	AC005102.1
chr7	55075000	chr7	66205000	2.58E-02	super_enhancer	promoter	RP11-792A8.4
chr7	55075000	chr7	87845000	8.15E-03	super_enhancer	promoter	CTB-167B5.1
chr7	55075000	chr7	100035000	8.10E-03	super_enhancer	promoter	PPP1R35
chr7	55085000	chr7	56645000	2.75E-02	super_enhancer	promoter	RP11-760D2.9
chr7	55085000	chr7	75625000	1.88E-02	super_enhancer	promoter	TMEM120A
chr7	55125000	chr7	143105000	3.07E-03	super_enhancer	promoter	EPHA1-AS1

chr7	55865000	chr7	151745000	2.61E-03	promoter	super_enhancer	CICP12
chr7	57295000	chr7	73845000	1.60E-02	promoter	super_enhancer	RNU7-157P
chr7	62695000	chr7	73815000	1.35E-02	promoter	super_enhancer	PHKG1P1
chr7	63505000	chr7	151815000	2.02E-03	promoter	super_enhancer	ZNF727
chr7	63545000	chr7	131185000	2.62E-03	promoter	super_enhancer	TRIM60P17
chr7	64125000	chr7	73845000	1.44E-02	promoter	super_enhancer	ZNF107
chr7	65305000	chr7	151715000	2.08E-03	promoter	super_enhancer	RP13-254B10.1
chr7	65325000	chr7	73705000	1.69E-02	promoter	super_enhancer	RNU6-973P
chr7	65325000	chr7	73715000	1.59E-02	promoter	super_enhancer	RNU6-973P
chr7	65875000	chr7	130355000	2.75E-03	promoter	super_enhancer	GTF2IP9
chr7	65955000	chr7	155955000	2.18E-03	promoter	super_enhancer	GS1-124K5.2
chr7	66205000	chr7	151725000	2.33E-03	promoter	super_enhancer	RP11-792A8.4
chr7	66345000	chr7	73835000	1.82E-02	promoter	super_enhancer	GTF2IP23
chr7	66355000	chr7	73835000	1.86E-02	promoter	super_enhancer	RNU6-1254P
chr7	66385000	chr7	73815000	2.18E-02	promoter	super_enhancer	TMEM248
chr7	66445000	chr7	116685000	4.40E-03	promoter	super_enhancer	RN7SL43P
chr7	66555000	chr7	73815000	1.69E-02	promoter	super_enhancer	RP11-324F21.1
chr7	66555000	chr7	73835000	1.52E-02	promoter	super_enhancer	RP11-324F21.1
chr7	66555000	chr7	159025000	2.47E-03	promoter	super_enhancer	RP11-324F21.1
chr7	66605000	chr7	73835000	1.41E-02	promoter	super_enhancer	AC073089.1
chr7	68105000	chr7	131505000	2.07E-03	promoter	super_enhancer	RP5-859M6.1
chr7	73275000	chr7	73815000	4.92E-02	promoter	super_enhancer	WBSCR28
chr7	73605000	chr7	131245000	4.63E-03	promoter	super_enhancer	MIR590
chr7	73705000	chr7	156665000	2.88E-03	super_enhancer	promoter	RNU4-31P

chr7	73715000	chr7	100255000	1.07E-02	super_enhancer	promoter	ACTL6B
chr7	73715000	chr7	100465000	9.64E-03	super_enhancer	promoter	TRIP6
chr7	73715000	chr7	116255000	4.66E-03	super_enhancer	promoter	LINC01510
chr7	73715000	chr7	128865000	4.79E-03	super_enhancer	promoter	AHCYL2
chr7	73715000	chr7	129895000	4.11E-03	super_enhancer	promoter	RP11-190G13.4
chr7	73715000	chr7	152065000	2.58E-03	super_enhancer	promoter	SEPT7P6
chr7	73815000	chr7	79175000	1.75E-02	super_enhancer	promoter	AC091813.2
chr7	73815000	chr7	93345000	7.60E-03	super_enhancer	promoter	MIR4652
chr7	73815000	chr7	106775000	8.54E-03	super_enhancer	promoter	CTA-360L10.1
chr7	73835000	chr7	129415000	5.92E-03	super_enhancer	promoter	MIR96
chr7	73835000	chr7	148975000	3.52E-03	super_enhancer	promoter	RP4-800G7.3
chr7	73845000	chr7	94325000	7.35E-03	super_enhancer	promoter	RPS3AP25
chr7	73845000	chr7	99065000	1.26E-02	super_enhancer	promoter	PTCD1
chr7	74485000	chr7	75115000	4.69E-02	super_enhancer	promoter	AC006014.7
chr7	74485000	chr7	100425000	9.01E-03	super_enhancer	promoter	EPHB4
chr7	74485000	chr7	104845000	6.21E-03	super_enhancer	promoter	CTB-152G17.4
chr7	75115000	chr7	131225000	3.14E-03	promoter	super_enhancer	AC006014.7
chr7	84295000	chr7	131535000	3.96E-03	promoter	super_enhancer	AC092022.1
chr7	86975000	chr7	131525000	3.41E-03	promoter	super_enhancer	TP53TG1
chr7	91875000	chr7	101135000	1.43E-02	promoter	super_enhancer	KRIT1
chr7	93635000	chr7	98285000	2.36E-02	promoter	super_enhancer	BET1
chr7	94325000	chr7	131235000	4.64E-03	promoter	super_enhancer	RPS3AP25
chr7	95065000	chr7	131645000	4.29E-03	promoter	super_enhancer	PON2
chr7	96655000	chr7	100445000	3.35E-02	promoter	super_enhancer	DLX5

chr7	97595000	chr7	131175000	3.70E-03	promoter	super_enhancer	MIR5692C2
chr7	97735000	chr7	130915000	8.84E-03	promoter	super_enhancer	LMTK2
chr7	98285000	chr7	130125000	6.84E-03	super_enhancer	promoter	RP11-2E11.5
chr7	98285000	chr7	148685000	6.39E-03	super_enhancer	promoter	RNY1
chr7	98285000	chr7	151105000	5.59E-03	super_enhancer	promoter	WDR86-AS1
chr7	98475000	chr7	129955000	1.08E-02	promoter	super_enhancer	TRRAP
chr7	98835000	chr7	129965000	1.02E-02	promoter	super_enhancer	MYH16
chr7	99155000	chr7	116685000	1.17E-02	promoter	super_enhancer	ZNF655
chr7	99425000	chr7	116685000	1.27E-02	promoter	super_enhancer	CYP3A43
chr7	99425000	chr7	151795000	5.46E-03	promoter	super_enhancer	CYP3A43
chr7	99475000	chr7	130365000	7.64E-03	promoter	super_enhancer	OR2AE1
chr7	99475000	chr7	151805000	3.37E-03	promoter	super_enhancer	OR2AE1
chr7	99575000	chr7	131535000	9.20E-03	promoter	super_enhancer	AZGP1
chr7	99775000	chr7	100435000	1.54E-02	promoter	super_enhancer	GPC2
chr7	99775000	chr7	100445000	1.23E-04	promoter	super_enhancer	GPC2
chr7	99905000	chr7	131545000	3.57E-03	promoter	super_enhancer	SPDYE3
chr7	100025000	chr7	159015000	3.07E-03	promoter	super_enhancer	MEPCE
chr7	100425000	chr7	131505000	8.30E-03	promoter	super_enhancer	EPHB4
chr7	100435000	chr7	135345000	6.57E-03	super_enhancer	promoter	AC093107.7
chr7	100445000	chr7	138795000	6.12E-03	super_enhancer	promoter	ZC3HAV1
chr7	100465000	chr7	159025000	5.82E-03	promoter	super_enhancer	TRIP6
chr7	100525000	chr7	101115000	1.23E-02	promoter	super_enhancer	RP13-650G11.1
chr7	100845000	chr7	131535000	1.37E-02	promoter	super_enhancer	MOGAT3
chr7	100965000	chr7	101105000	1.56E-02	promoter	super_enhancer	IFT22

chr7	100965000	chr7	101135000	3.49E-02	promoter	super_enhancer	IFT22
chr7	101005000	chr7	101105000	2.74E-03	promoter	super_enhancer	COL26A1
chr7	101005000	chr7	101125000	2.81E-02	promoter	super_enhancer	COL26A1
chr7	101095000	chr7	102005000	2.16E-02	super_enhancer	promoter	RP11-163E9.2
chr7	101095000	chr7	104655000	2.38E-02	super_enhancer	promoter	KMT2E-AS1
chr7	101095000	chr7	104885000	3.84E-02	super_enhancer	promoter	RP4-778K6.1
chr7	101095000	chr7	105225000	4.08E-02	super_enhancer	promoter	YBX1P2
chr7	101095000	chr7	129425000	1.02E-02	super_enhancer	promoter	RP11-738B7.1
chr7	101105000	chr7	102105000	4.52E-02	super_enhancer	promoter	ALKBH4
chr7	101105000	chr7	104885000	4.40E-02	super_enhancer	promoter	RP4-778K6.1
chr7	101105000	chr7	135415000	7.38E-03	super_enhancer	promoter	SLC13A4
chr7	101115000	chr7	101935000	1.11E-02	super_enhancer	promoter	MIR4285
chr7	101115000	chr7	105925000	3.01E-02	super_enhancer	promoter	RP11-22N19.2
chr7	101125000	chr7	102105000	4.90E-02	super_enhancer	promoter	ALKBH4
chr7	101125000	chr7	104885000	4.52E-02	super_enhancer	promoter	RP4-778K6.1
chr7	101125000	chr7	128555000	1.03E-02	super_enhancer	promoter	RP11-309L24.6
chr7	101135000	chr7	102005000	2.78E-03	super_enhancer	promoter	RP11-163E9.2
chr7	101805000	chr7	131555000	1.03E-02	promoter	super_enhancer	AC005086.3
chr7	102075000	chr7	116715000	1.36E-02	promoter	super_enhancer	RP11-514P8.2
chr7	102075000	chr7	130885000	6.84E-03	promoter	super_enhancer	RP11-514P8.2
chr7	102075000	chr7	155965000	4.67E-03	promoter	super_enhancer	RP11-514P8.2
chr7	102475000	chr7	131225000	9.57E-03	promoter	super_enhancer	RNU6-1136P
chr7	102475000	chr7	131245000	1.07E-02	promoter	super_enhancer	RNU6-1136P
chr7	102655000	chr7	131245000	9.47E-03	promoter	super_enhancer	AC073127.1

chr7	102985000	chr7	131525000	9.13E-03	promoter	super_enhancer	DNAJC2
chr7	104445000	chr7	151725000	4.47E-03	promoter	super_enhancer	LHFPL3-AS1
chr7	105225000	chr7	129955000	1.37E-02	promoter	super_enhancer	YBX1P2
chr7	105225000	chr7	151805000	4.63E-03	promoter	super_enhancer	YBX1P2
chr7	105675000	chr7	131235000	1.25E-02	promoter	super_enhancer	RP4-568B10.1
chr7	106415000	chr7	130375000	6.19E-03	promoter	super_enhancer	RP5-884M6.1
chr7	106415000	chr7	131235000	9.65E-03	promoter	super_enhancer	RP5-884M6.1
chr7	106505000	chr7	131645000	1.38E-02	promoter	super_enhancer	PIK3CG
chr7	106685000	chr7	131175000	1.29E-02	promoter	super_enhancer	PRKAR2B
chr7	107205000	chr7	116715000	1.37E-02	promoter	super_enhancer	COG5
chr7	107205000	chr7	129955000	1.10E-02	promoter	super_enhancer	COG5
chr7	107205000	chr7	130915000	9.93E-03	promoter	super_enhancer	COG5
chr7	107645000	chr7	116675000	2.13E-02	promoter	super_enhancer	LAMB1
chr7	107645000	chr7	116715000	2.14E-02	promoter	super_enhancer	LAMB1
chr7	107645000	chr7	131545000	1.10E-02	promoter	super_enhancer	LAMB1
chr7	107645000	chr7	131635000	1.47E-02	promoter	super_enhancer	LAMB1
chr7	107645000	chr7	151745000	5.70E-03	promoter	super_enhancer	LAMB1
chr7	108545000	chr7	116685000	1.52E-02	promoter	super_enhancer	CTB-22K14.1
chr7	108545000	chr7	116695000	1.72E-02	promoter	super_enhancer	CTB-22K14.1
chr7	109855000	chr7	131555000	1.13E-02	promoter	super_enhancer	RP11-745H7.1
chr7	110175000	chr7	131625000	1.09E-02	promoter	super_enhancer	AC003088.1
chr7	111035000	chr7	130365000	1.02E-02	promoter	super_enhancer	AC003989.3
chr7	112015000	chr7	116675000	2.70E-02	promoter	super_enhancer	AC004112.5
chr7	112015000	chr7	131525000	1.11E-02	promoter	super_enhancer	AC004112.5

chr7	113055000	chr7	116715000	3.70E-02	promoter	super_enhancer	RP11-703P11.1
chr7	113725000	chr7	116675000	9.22E-04	promoter	super_enhancer	FOXP2
chr7	113725000	chr7	116685000	4.41E-02	promoter	super_enhancer	FOXP2
chr7	113725000	chr7	131235000	1.21E-02	promoter	super_enhancer	FOXP2
chr7	113725000	chr7	131515000	1.25E-02	promoter	super_enhancer	FOXP2
chr7	116165000	chr7	116675000	3.45E-02	promoter	super_enhancer	CAV1
chr7	116165000	chr7	131165000	1.53E-02	promoter	super_enhancer	CAV1
chr7	116165000	chr7	131225000	1.84E-02	promoter	super_enhancer	CAV1
chr7	116255000	chr7	130355000	1.15E-02	promoter	super_enhancer	LINC01510
chr7	116595000	chr7	131625000	1.79E-02	promoter	super_enhancer	ST7-AS1
chr7	116675000	chr7	117645000	4.05E-02	super_enhancer	promoter	AC003084.2
chr7	116685000	chr7	124385000	1.31E-02	super_enhancer	promoter	RP5-907C10.3
chr7	116685000	chr7	128215000	1.45E-02	super_enhancer	promoter	RP11-274B21.14
chr7	116695000	chr7	121785000	2.56E-02	super_enhancer	promoter	AASS
chr7	116695000	chr7	128085000	2.13E-02	super_enhancer	promoter	RNU7-54P
chr7	116695000	chr7	138485000	1.40E-02	super_enhancer	promoter	ATP6V0A4
chr7	116695000	chr7	151125000	1.04E-02	super_enhancer	promoter	RP4-555L14.4
chr7	116695000	chr7	156985000	6.69E-03	super_enhancer	promoter	RP4-814D15.2
chr7	116705000	chr7	121785000	2.44E-02	super_enhancer	promoter	AASS
chr7	116705000	chr7	128095000	2.07E-02	super_enhancer	promoter	RP11-212P7.3
chr7	116705000	chr7	129985000	1.67E-02	super_enhancer	promoter	CPA5
chr7	116705000	chr7	138125000	1.12E-02	super_enhancer	promoter	AC008265.2
chr7	116715000	chr7	123295000	1.92E-02	super_enhancer	promoter	LMOD2
chr7	116715000	chr7	151825000	6.85E-03	super_enhancer	promoter	YBX1P4

chr7	117105000	chr7	131645000	1.81E-02	promoter	super_enhancer	CFTR
chr7	117855000	chr7	131625000	1.86E-02	promoter	super_enhancer	ANKRD7
chr7	120835000	chr7	151795000	7.99E-03	promoter	super_enhancer	RNU6-517P
chr7	120965000	chr7	155965000	7.14E-03	promoter	super_enhancer	WNT16
chr7	121375000	chr7	151805000	6.49E-03	promoter	super_enhancer	RN7SKP277
chr7	121485000	chr7	131165000	1.53E-02	promoter	super_enhancer	PNPT1P2
chr7	121785000	chr7	131185000	1.76E-02	promoter	super_enhancer	AASS
chr7	121875000	chr7	131525000	1.39E-02	promoter	super_enhancer	RP11-560I19.2
chr7	123175000	chr7	151795000	9.22E-03	promoter	super_enhancer	IQUB
chr7	123175000	chr7	155965000	7.25E-03	promoter	super_enhancer	IQUB
chr7	123265000	chr7	130885000	1.65E-02	promoter	super_enhancer	RP11-390E23.3
chr7	123295000	chr7	131185000	2.26E-02	promoter	super_enhancer	LMOD2
chr7	123565000	chr7	130375000	1.75E-02	promoter	super_enhancer	SPAM1
chr7	123565000	chr7	131645000	2.26E-02	promoter	super_enhancer	SPAM1
chr7	123635000	chr7	131555000	2.38E-02	promoter	super_enhancer	RP4-630C24.3
chr7	123635000	chr7	151695000	7.86E-03	promoter	super_enhancer	RP4-630C24.3
chr7	124905000	chr7	130375000	1.79E-02	promoter	super_enhancer	RP11-3B12.3
chr7	124985000	chr7	157305000	6.01E-03	promoter	super_enhancer	RP11-420H19.1
chr7	127005000	chr7	131235000	4.33E-02	promoter	super_enhancer	AC000123.2
chr7	127235000	chr7	159015000	1.01E-02	promoter	super_enhancer	GCC1
chr7	127905000	chr7	151735000	1.27E-02	promoter	super_enhancer	RP11-62J1.4
chr7	127905000	chr7	157305000	1.05E-02	promoter	super_enhancer	RP11-62J1.4
chr7	127985000	chr7	131185000	5.52E-03	promoter	super_enhancer	RBM28
chr7	127985000	chr7	151425000	1.68E-02	promoter	super_enhancer	RBM28

chr7	128095000	chr7	151705000	1.60E-02	promoter	super_enhancer	RP11-212P7.3
chr7	128215000	chr7	151425000	1.09E-02	promoter	super_enhancer	RP11-274B21.14
chr7	128295000	chr7	131225000	4.29E-02	promoter	super_enhancer	RP11-274B21.12
chr7	128805000	chr7	159025000	1.48E-02	promoter	super_enhancer	RNY1P11
chr7	128865000	chr7	151725000	1.03E-02	promoter	super_enhancer	AHCYL2
chr7	129415000	chr7	131225000	2.54E-02	promoter	super_enhancer	MIR96
chr7	129425000	chr7	131175000	1.93E-02	promoter	super_enhancer	RP11-738B7.1
chr7	129425000	chr7	151745000	1.19E-02	promoter	super_enhancer	RP11-738B7.1
chr7	129425000	chr7	151805000	1.17E-02	promoter	super_enhancer	RP11-738B7.1
chr7	129425000	chr7	157305000	1.12E-02	promoter	super_enhancer	RP11-738B7.1
chr7	129895000	chr7	130375000	3.95E-03	promoter	super_enhancer	RP11-190G13.4
chr7	129955000	chr7	130135000	2.03E-02	super_enhancer	promoter	MIR335
chr7	129955000	chr7	157075000	1.59E-02	super_enhancer	promoter	RP5-1142J19.1
chr7	129965000	chr7	130475000	3.37E-03	super_enhancer	promoter	RP11-36B6.1
chr7	129975000	chr7	142745000	1.60E-02	super_enhancer	promoter	OR9P1P
chr7	129975000	chr7	150375000	9.13E-03	super_enhancer	promoter	RP11-632K21.6
chr7	130125000	chr7	155955000	8.06E-03	promoter	super_enhancer	RP11-2E11.5
chr7	130125000	chr7	157305000	8.46E-03	promoter	super_enhancer	RP11-2E11.5
chr7	130135000	chr7	157285000	6.71E-03	promoter	super_enhancer	MIR335
chr7	130355000	chr7	135195000	3.47E-02	super_enhancer	promoter	CNOT4
chr7	130355000	chr7	138125000	2.06E-02	super_enhancer	promoter	AC008265.2
chr7	130355000	chr7	149095000	1.12E-02	super_enhancer	promoter	AC004941.3
chr7	130365000	chr7	152065000	1.14E-02	super_enhancer	promoter	SEPT7P6
chr7	130375000	chr7	138485000	2.09E-02	super_enhancer	promoter	ATP6V0A4

chr7	130885000	chr7	138385000	2.06E-02	super_enhancer	promoter	UQCRFS1P2
chr7	130885000	chr7	138885000	1.68E-02	super_enhancer	promoter	RP11-365F18.6
chr7	130885000	chr7	142635000	1.44E-02	super_enhancer	promoter	C7orf34
chr7	130885000	chr7	151125000	7.93E-05	super_enhancer	promoter	RP4-555L14.4
chr7	130915000	chr7	142995000	2.10E-02	super_enhancer	promoter	RN7SL481P
chr7	130915000	chr7	152455000	1.43E-02	super_enhancer	promoter	RN7SL845P
chr7	131165000	chr7	138125000	2.38E-02	super_enhancer	promoter	AC008265.2
chr7	131165000	chr7	138885000	2.33E-04	super_enhancer	promoter	RP11-365F18.6
chr7	131165000	chr7	155665000	8.74E-03	super_enhancer	promoter	AC104133.1
chr7	131175000	chr7	138795000	2.61E-02	super_enhancer	promoter	ZC3HAV1
chr7	131175000	chr7	148395000	1.52E-02	super_enhancer	promoter	CUL1
chr7	131175000	chr7	149535000	1.62E-02	super_enhancer	promoter	ZNF862
chr7	131175000	chr7	150775000	1.58E-02	super_enhancer	promoter	RP11-148K1.12
chr7	131185000	chr7	142225000	1.30E-02	super_enhancer	promoter	TRBV11-1
chr7	131185000	chr7	146795000	1.65E-02	super_enhancer	promoter	AC006004.1
chr7	131185000	chr7	150065000	1.93E-02	super_enhancer	promoter	REPIN1
chr7	131205000	chr7	131505000	3.60E-02	promoter	super_enhancer	AC008264.4
chr7	131225000	chr7	137745000	2.61E-02	super_enhancer	promoter	AC024082.4
chr7	131235000	chr7	142985000	2.64E-02	super_enhancer	promoter	AC073342.12
chr7	131235000	chr7	148395000	1.86E-02	super_enhancer	promoter	CUL1
chr7	131235000	chr7	150675000	1.97E-02	super_enhancer	promoter	KCNH2
chr7	131245000	chr7	142985000	2.68E-02	super_enhancer	promoter	AC073342.12
chr7	131245000	chr7	155605000	1.50E-02	super_enhancer	promoter	SHH
chr7	131245000	chr7	156985000	1.31E-02	super_enhancer	promoter	RP4-814D15.2

chr7	131505000	chr7	134145000	2.94E-03	super_enhancer	promoter	AKR1B1
chr7	131505000	chr7	152135000	1.33E-02	super_enhancer	promoter	AC005631.1
chr7	131515000	chr7	138125000	3.52E-02	super_enhancer	promoter	AC008265.2
chr7	131515000	chr7	142585000	1.80E-02	super_enhancer	promoter	TRPV6
chr7	131515000	chr7	151575000	2.12E-02	super_enhancer	promoter	PRKAG2
chr7	131515000	chr7	156665000	1.37E-02	super_enhancer	promoter	RNU4-31P
chr7	131525000	chr7	137745000	2.63E-02	super_enhancer	promoter	AC024082.4
chr7	131525000	chr7	142585000	1.48E-02	super_enhancer	promoter	TRPV6
chr7	131545000	chr7	135645000	2.77E-02	super_enhancer	promoter	RNU6-223P
chr7	131545000	chr7	140105000	1.91E-02	super_enhancer	promoter	SLC37A3
chr7	131545000	chr7	155175000	1.13E-02	super_enhancer	promoter	AC008060.7
chr7	131555000	chr7	142125000	2.00E-02	super_enhancer	promoter	TRBV6-8
chr7	131555000	chr7	142635000	2.52E-02	super_enhancer	promoter	C7orf34
chr7	131555000	chr7	142995000	2.36E-02	super_enhancer	promoter	RN7SL481P
chr7	131555000	chr7	149095000	1.68E-02	super_enhancer	promoter	AC004941.3
chr7	131625000	chr7	140345000	2.19E-02	super_enhancer	promoter	RN7SL771P
chr7	131625000	chr7	150745000	1.95E-02	super_enhancer	promoter	ASIC3
chr7	131625000	chr7	150935000	2.03E-02	super_enhancer	promoter	MIR671
chr7	131635000	chr7	149095000	1.52E-02	super_enhancer	promoter	AC004941.3
chr7	132855000	chr7	151805000	1.03E-02	promoter	super_enhancer	AC083873.4
chr7	132975000	chr7	151755000	7.93E-03	promoter	super_enhancer	MIR6133
chr7	134145000	chr7	155955000	9.96E-03	promoter	super_enhancer	AKR1B1
chr7	134145000	chr7	159025000	1.42E-02	promoter	super_enhancer	AKR1B1
chr7	135195000	chr7	151725000	1.23E-02	promoter	super_enhancer	CNOT4

chr7	135195000	chr7	159015000	9.56E-03	promoter	super_enhancer	CNOT4
chr7	135335000	chr7	151695000	1.51E-02	promoter	super_enhancer	RP11-751K21.1
chr7	135645000	chr7	151685000	1.16E-02	promoter	super_enhancer	RNU6-223P
chr7	138125000	chr7	151795000	1.86E-02	promoter	super_enhancer	AC008265.2
chr7	138125000	chr7	159015000	1.01E-02	promoter	super_enhancer	AC008265.2
chr7	138485000	chr7	157295000	1.69E-02	promoter	super_enhancer	ATP6V0A4
chr7	138485000	chr7	159025000	2.01E-02	promoter	super_enhancer	ATP6V0A4
chr7	138795000	chr7	155965000	1.55E-02	promoter	super_enhancer	ZC3HAV1
chr7	139025000	chr7	151725000	1.39E-02	promoter	super_enhancer	C7orf55-LUC7L2
chr7	139025000	chr7	159025000	1.70E-02	promoter	super_enhancer	C7orf55-LUC7L2
chr7	139045000	chr7	151695000	1.66E-02	promoter	super_enhancer	RP11-634H22.1
chr7	139765000	chr7	151715000	1.81E-02	promoter	super_enhancer	PARP12
chr7	140375000	chr7	151705000	2.36E-02	promoter	super_enhancer	DENND2A
chr7	140715000	chr7	151705000	1.98E-02	promoter	super_enhancer	MRPS33
chr7	141365000	chr7	159015000	1.10E-02	promoter	super_enhancer	RP5-894A10.6
chr7	141675000	chr7	151695000	1.30E-02	promoter	super_enhancer	TAS2R38
chr7	141985000	chr7	151705000	2.37E-02	promoter	super_enhancer	U66059.58
chr7	141985000	chr7	157295000	1.57E-02	promoter	super_enhancer	U66059.58
chr7	142125000	chr7	151755000	1.31E-02	promoter	super_enhancer	TRBV6-8
chr7	142585000	chr7	151755000	1.20E-02	promoter	super_enhancer	TRPV6
chr7	142635000	chr7	159015000	1.31E-02	promoter	super_enhancer	C7orf34
chr7	148685000	chr7	157285000	2.90E-02	promoter	super_enhancer	RNY1
chr7	149535000	chr7	159025000	3.30E-02	promoter	super_enhancer	ZNF862
chr7	150065000	chr7	151425000	4.35E-02	promoter	super_enhancer	REPIN1

chr7	150445000	chr7	155965000	3.84E-02	promoter	super_enhancer	GIMAP3P
chr7	150725000	chr7	155945000	2.94E-02	promoter	super_enhancer	ABCB8
chr7	150725000	chr7	155955000	3.39E-02	promoter	super_enhancer	ABCB8
chr7	150725000	chr7	155965000	4.00E-02	promoter	super_enhancer	ABCB8
chr7	150725000	chr7	159025000	3.49E-02	promoter	super_enhancer	ABCB8
chr7	150885000	chr7	151755000	1.03E-02	promoter	super_enhancer	ASB10
chr7	150925000	chr7	157305000	3.50E-02	promoter	super_enhancer	ABCF2
chr7	150925000	chr7	159015000	2.51E-02	promoter	super_enhancer	ABCF2
chr7	150935000	chr7	157285000	3.86E-02	promoter	super_enhancer	MIR671
chr7	150945000	chr7	155955000	2.84E-02	promoter	super_enhancer	RP5-1070G24.2
chr7	150975000	chr7	151685000	3.36E-02	promoter	super_enhancer	SMARCD3
chr7	151105000	chr7	151725000	3.40E-02	promoter	super_enhancer	WDR86-AS1
chr7	151105000	chr7	151815000	3.14E-02	promoter	super_enhancer	WDR86-AS1
chr7	151125000	chr7	151425000	3.47E-02	promoter	super_enhancer	RP4-555L14.4
chr7	151125000	chr7	151725000	3.80E-02	promoter	super_enhancer	RP4-555L14.4
chr7	151125000	chr7	157285000	3.65E-02	promoter	super_enhancer	RP4-555L14.4
chr7	151125000	chr7	159015000	2.63E-02	promoter	super_enhancer	RP4-555L14.4
chr7	151425000	chr7	156665000	7.30E-04	super_enhancer	promoter	RNU4-31P
chr7	151695000	chr7	154995000	2.24E-03	super_enhancer	promoter	AC099552.3
chr7	151735000	chr7	155755000	3.05E-02	super_enhancer	promoter	AC021218.2
chr7	151755000	chr7	156425000	3.36E-02	super_enhancer	promoter	AC005534.6
chr7	151795000	chr7	152445000	4.80E-02	super_enhancer	promoter	ATP5F1P3
chr7	151795000	chr7	155665000	4.93E-02	super_enhancer	promoter	AC104133.1
chr7	151815000	chr7	157075000	4.07E-02	super_enhancer	promoter	RP5-1142J19.1

chr7	154795000	chr7	157285000	5.69E-03	promoter	super_enhancer	PAXIP1
chr7	155605000	chr7	155945000	2.43E-03	promoter	super_enhancer	SHH
chr7	155605000	chr7	155955000	2.09E-02	promoter	super_enhancer	SHH
chr7	155605000	chr7	155965000	7.58E-03	promoter	super_enhancer	SHH
chr7	155605000	chr7	157305000	1.47E-02	promoter	super_enhancer	SHH
chr7	155945000	chr7	156425000	2.97E-02	super_enhancer	promoter	AC005534.6
chr8	1055000	chr8	11855000	1.47E-02	promoter	super_enhancer	CTD-2281E23.2
chr8	1055000	chr8	23345000	8.09E-03	promoter	super_enhancer	CTD-2281E23.2
chr8	1325000	chr8	8255000	1.68E-02	promoter	super_enhancer	AF067845.3
chr8	1325000	chr8	11895000	5.79E-03	promoter	super_enhancer	AF067845.3
chr8	1325000	chr8	28365000	4.82E-03	promoter	super_enhancer	AF067845.3
chr8	1765000	chr8	13355000	1.31E-02	promoter	super_enhancer	MIR596
chr8	1765000	chr8	22595000	1.43E-02	promoter	super_enhancer	MIR596
chr8	1765000	chr8	23355000	1.28E-02	promoter	super_enhancer	MIR596
chr8	1765000	chr8	27875000	1.11E-02	promoter	super_enhancer	MIR596
chr8	2025000	chr8	8235000	2.77E-02	promoter	super_enhancer	MIR7160
chr8	2025000	chr8	8315000	2.71E-02	promoter	super_enhancer	MIR7160
chr8	2025000	chr8	8635000	2.94E-02	promoter	super_enhancer	MIR7160
chr8	2025000	chr8	11885000	1.43E-02	promoter	super_enhancer	MIR7160
chr8	2025000	chr8	101025000	2.66E-03	promoter	super_enhancer	MIR7160
chr8	2375000	chr8	13365000	1.22E-02	promoter	super_enhancer	AC133633.1
chr8	2375000	chr8	22595000	1.21E-02	promoter	super_enhancer	AC133633.1
chr8	2585000	chr8	11505000	2.25E-04	promoter	super_enhancer	GS1-57L11.1
chr8	2585000	chr8	11895000	7.96E-03	promoter	super_enhancer	GS1-57L11.1

chr8	2585000	chr8	27865000	6.53E-03	promoter		super_enhancer	GS1-57L11.1
chr8	2585000	chr8	37145000	4.88E-03	promoter		super_enhancer	GS1-57L11.1
chr8	2585000	chr8	143515000	3.14E-03	promoter		super_enhancer	GS1-57L11.1
chr8	4645000	chr8	11875000	2.13E-02	promoter		super_enhancer	PAICSP4
chr8	4645000	chr8	93895000	1.42E-03	promoter		super_enhancer	PAICSP4
chr8	5705000	chr8	11855000	2.96E-02	promoter		super_enhancer	RP11-728L1.1
chr8	5705000	chr8	22185000	1.28E-02	promoter		super_enhancer	RP11-728L1.1
chr8	5705000	chr8	38905000	4.46E-03	promoter		super_enhancer	RP11-728L1.1
chr8	5895000	chr8	11485000	3.70E-02	promoter		super_enhancer	RP11-158A14.1
chr8	5895000	chr8	23345000	1.59E-02	promoter		super_enhancer	RP11-158A14.1
chr8	5895000	chr8	58855000	4.22E-03	promoter		super_enhancer	RP11-158A14.1
chr8	6115000	chr8	29595000	1.09E-02	promoter		super_enhancer	RP11-124B13.1
chr8	6115000	chr8	66515000	4.12E-03	promoter		super_enhancer	RP11-124B13.1
chr8	6115000	chr8	143585000	4.13E-03	promoter		super_enhancer	RP11-124B13.1
chr8	6265000	chr8	11505000	4.96E-02	promoter		super_enhancer	RP11-115C21.2
chr8	6265000	chr8	11885000	3.15E-02	promoter		super_enhancer	RP11-115C21.2
chr8	6265000	chr8	22985000	1.76E-02	promoter		super_enhancer	RP11-115C21.2
chr8	6265000	chr8	102765000	3.95E-03	promoter		super_enhancer	RP11-115C21.2
chr8	6265000	chr8	143535000	4.58E-03	promoter		super_enhancer	RP11-115C21.2
chr8	6475000	chr8	11475000	4.63E-02	promoter		super_enhancer	CTD-2541M15.3
chr8	6565000	chr8	22175000	1.87E-02	promoter		super_enhancer	AGPAT5
chr8	6565000	chr8	23755000	1.26E-02	promoter		super_enhancer	AGPAT5
chr8	6565000	chr8	38895000	6.25E-03	promoter		super_enhancer	AGPAT5
chr8	6565000	chr8	61775000	5.82E-03	promoter		super_enhancer	AGPAT5

chr8	6785000	chr8	11465000	1.38E-03	promoter	super_enhancer	DEFA6
chr8	6785000	chr8	11475000	4.99E-02	promoter	super_enhancer	DEFA6
chr8	6785000	chr8	11515000	9.03E-04	promoter	super_enhancer	DEFA6
chr8	6785000	chr8	13355000	1.91E-02	promoter	super_enhancer	DEFA6
chr8	6915000	chr8	11485000	4.34E-02	promoter	super_enhancer	DEFA5
chr8	6915000	chr8	11515000	4.01E-02	promoter	super_enhancer	DEFA5
chr8	6915000	chr8	11855000	4.29E-02	promoter	super_enhancer	DEFA5
chr8	6915000	chr8	11875000	3.90E-02	promoter	super_enhancer	DEFA5
chr8	6915000	chr8	11885000	3.02E-02	promoter	super_enhancer	DEFA5
chr8	6975000	chr8	11485000	6.07E-04	promoter	super_enhancer	AF228730.1
chr8	6975000	chr8	11505000	3.86E-02	promoter	super_enhancer	AF228730.1
chr8	6975000	chr8	11855000	3.42E-02	promoter	super_enhancer	AF228730.1
chr8	6975000	chr8	11875000	3.10E-02	promoter	super_enhancer	AF228730.1
chr8	6975000	chr8	22595000	7.72E-05	promoter	super_enhancer	AF228730.1
chr8	6975000	chr8	29645000	8.93E-03	promoter	super_enhancer	AF228730.1
chr8	8085000	chr8	11885000	1.35E-03	promoter	super_enhancer	FAM85B
chr8	8085000	chr8	19675000	2.14E-02	promoter	super_enhancer	FAM85B
chr8	8085000	chr8	23525000	1.67E-02	promoter	super_enhancer	FAM85B
chr8	8235000	chr8	12585000	4.37E-02	super_enhancer	promoter	MIR3926-1
chr8	8235000	chr8	12615000	4.70E-02	super_enhancer	promoter	LONRF1
chr8	8235000	chr8	99405000	4.69E-03	super_enhancer	promoter	KB-1517D11.3
chr8	8245000	chr8	23385000	1.93E-02	super_enhancer	promoter	SLC25A37
chr8	8245000	chr8	26695000	1.51E-02	super_enhancer	promoter	COX6B1P4
chr8	8245000	chr8	38855000	8.97E-03	super_enhancer	promoter	TM2D2

chr8	8245000	chr8	144365000	6.29E-03	super_enhancer	promoter	LINC01604
chr8	8255000	chr8	9015000	1.21E-02	super_enhancer	promoter	RP11-10A14.9
chr8	8255000	chr8	12615000	4.31E-02	super_enhancer	promoter	LONRF1
chr8	8255000	chr8	124135000	3.67E-03	super_enhancer	promoter	HMGB1P19
chr8	8265000	chr8	12035000	2.74E-02	super_enhancer	promoter	ALG1L11P
chr8	8265000	chr8	12615000	4.54E-02	super_enhancer	promoter	LONRF1
chr8	8265000	chr8	22015000	2.08E-02	super_enhancer	promoter	LGI3
chr8	8265000	chr8	41155000	8.54E-03	super_enhancer	promoter	RNU6-895P
chr8	8275000	chr8	14345000	2.89E-02	super_enhancer	promoter	RNU6-397P
chr8	8275000	chr8	19135000	1.43E-02	super_enhancer	promoter	RP11-618M23.3
chr8	8275000	chr8	135725000	3.46E-03	super_enhancer	promoter	ZFAT
chr8	8285000	chr8	12035000	2.61E-02	super_enhancer	promoter	ALG1L11P
chr8	8285000	chr8	12535000	3.20E-02	super_enhancer	promoter	RP11-303G3.10
chr8	8285000	chr8	98655000	3.82E-03	super_enhancer	promoter	MTDH
chr8	8295000	chr8	8905000	2.15E-02	super_enhancer	promoter	MIR4660
chr8	8295000	chr8	20055000	1.19E-02	super_enhancer	promoter	ATP6V1B2
chr8	8305000	chr8	13195000	4.47E-02	super_enhancer	promoter	RP11-10C8.2
chr8	8305000	chr8	23565000	2.04E-02	super_enhancer	promoter	NKX2-6
chr8	8305000	chr8	26115000	1.67E-02	super_enhancer	promoter	RP11-98P2.1
chr8	8315000	chr8	12035000	2.89E-02	super_enhancer	promoter	ALG1L11P
chr8	8315000	chr8	17455000	2.22E-02	super_enhancer	promoter	RP11-349F21.1
chr8	8315000	chr8	19135000	1.62E-02	super_enhancer	promoter	RP11-618M23.3
chr8	8315000	chr8	22475000	1.85E-02	super_enhancer	promoter	RP11-582J16.5
chr8	8315000	chr8	22735000	1.76E-02	super_enhancer	promoter	RP11-87E22.2

chr8	8315000	chr8	23565000	1.73E-02	super_enhancer	promoter	NKX2-6
chr8	8315000	chr8	26115000	1.42E-02	super_enhancer	promoter	RP11-98P2.1
chr8	8315000	chr8	33195000	9.13E-03	super_enhancer	promoter	RNU6-528P
chr8	8315000	chr8	38705000	7.78E-03	super_enhancer	promoter	RP11-723D22.3
chr8	8315000	chr8	98155000	3.47E-03	super_enhancer	promoter	KB-1958F4.1
chr8	8325000	chr8	12815000	3.45E-02	super_enhancer	promoter	RP11-369K16.1
chr8	8325000	chr8	15665000	1.21E-02	super_enhancer	promoter	PPM1AP1
chr8	8325000	chr8	37375000	6.95E-03	super_enhancer	promoter	RP11-150O12.6
chr8	8325000	chr8	55065000	4.66E-03	super_enhancer	promoter	RNU6ATAC32P
chr8	8335000	chr8	12035000	1.97E-02	super_enhancer	promoter	ALG1L11P
chr8	8335000	chr8	18245000	1.54E-02	super_enhancer	promoter	RP11-685B14.3
chr8	8335000	chr8	28305000	9.37E-03	super_enhancer	promoter	RP11-181B11.2
chr8	8335000	chr8	42235000	5.51E-03	super_enhancer	promoter	DKK4
chr8	8605000	chr8	22735000	1.87E-02	super_enhancer	promoter	RP11-87E22.2
chr8	8605000	chr8	22925000	2.19E-02	super_enhancer	promoter	RP11-875O11.2
chr8	8605000	chr8	33085000	1.02E-02	super_enhancer	promoter	RP11-451O18.1
chr8	8605000	chr8	41755000	7.66E-03	super_enhancer	promoter	ANK1
chr8	8615000	chr8	17605000	2.49E-02	super_enhancer	promoter	RNA5SP256
chr8	8615000	chr8	20055000	1.85E-02	super_enhancer	promoter	ATP6V1B2
chr8	8615000	chr8	22425000	2.35E-02	super_enhancer	promoter	RP11-582J16.3
chr8	8615000	chr8	23315000	1.57E-02	super_enhancer	promoter	ENTPD4
chr8	8615000	chr8	48045000	8.46E-03	super_enhancer	promoter	RNU6-819P
chr8	8615000	chr8	102505000	5.03E-03	super_enhancer	promoter	KB-1562D12.1
chr8	8615000	chr8	134585000	4.76E-03	super_enhancer	promoter	ST3GAL1

chr8	8625000	chr8	23385000	1.60E-02	super_enhancer	promoter	SLC25A37
chr8	8635000	chr8	12585000	2.95E-05	super_enhancer	promoter	MIR3926-1
chr8	8635000	chr8	18245000	2.64E-02	super_enhancer	promoter	RP11-685B14.3
chr8	8635000	chr8	22475000	2.14E-02	super_enhancer	promoter	RP11-582J16.5
chr8	8635000	chr8	29885000	1.37E-02	super_enhancer	promoter	MAP2K1P1
chr8	8635000	chr8	30585000	1.34E-02	super_enhancer	promoter	GSR
chr8	8635000	chr8	95825000	4.52E-03	super_enhancer	promoter	INTS8
chr8	8635000	chr8	124275000	4.34E-03	super_enhancer	promoter	RNU6-628P
chr8	8645000	chr8	12535000	3.85E-02	super_enhancer	promoter	RP11-303G3.10
chr8	8645000	chr8	12585000	4.81E-02	super_enhancer	promoter	MIR3926-1
chr8	8645000	chr8	23665000	1.17E-02	super_enhancer	promoter	RP11-175E9.2
chr8	8645000	chr8	37595000	1.06E-02	super_enhancer	promoter	RP11-863K10.7
chr8	8645000	chr8	56015000	4.91E-03	super_enhancer	promoter	XKR4
chr8	8645000	chr8	95825000	3.89E-03	super_enhancer	promoter	INTS8
chr8	8645000	chr8	109145000	4.41E-03	super_enhancer	promoter	AP001331.1
chr8	9015000	chr8	27905000	1.02E-02	promoter	super_enhancer	RP11-10A14.9
chr8	9015000	chr8	143535000	3.43E-03	promoter	super_enhancer	RP11-10A14.9
chr8	9075000	chr8	9945000	8.17E-03	super_enhancer	promoter	AC023385.1
chr8	9075000	chr8	124055000	3.50E-03	super_enhancer	promoter	DERL1
chr8	9085000	chr8	17455000	1.87E-02	super_enhancer	promoter	RP11-349F21.1
chr8	9145000	chr8	28375000	1.00E-02	promoter	super_enhancer	RNU6-1151P
chr8	9295000	chr8	26455000	1.10E-02	promoter	super_enhancer	RP11-115J16.2
chr8	9295000	chr8	29635000	1.44E-02	promoter	super_enhancer	RP11-115J16.2
chr8	9415000	chr8	23015000	1.51E-02	promoter	super_enhancer	RP11-375N15.2

chr8	9415000	chr8	26465000	7.42E-03	promoter	super_enhancer	RP11-375N15.2
chr8	9945000	chr8	38905000	5.78E-03	promoter	super_enhancer	AC023385.1
chr8	10195000	chr8	11515000	3.79E-02	promoter	super_enhancer	RP11-212F11.1
chr8	10405000	chr8	10715000	4.46E-05	promoter	super_enhancer	PRSS51
chr8	10405000	chr8	23535000	1.96E-02	promoter	super_enhancer	PRSS51
chr8	10715000	chr8	11225000	3.75E-02	super_enhancer	promoter	FAM167A-AS1
chr8	10715000	chr8	40755000	8.76E-03	super_enhancer	promoter	ZMAT4
chr8	10715000	chr8	41435000	1.05E-02	super_enhancer	promoter	AGPAT6
chr8	10925000	chr8	23005000	2.02E-02	promoter	super_enhancer	AF131215.6
chr8	10925000	chr8	27895000	1.31E-02	promoter	super_enhancer	AF131215.6
chr8	10925000	chr8	66515000	4.41E-03	promoter	super_enhancer	AF131215.6
chr8	10985000	chr8	23345000	2.08E-02	promoter	super_enhancer	AF131215.3
chr8	10985000	chr8	23545000	1.89E-02	promoter	super_enhancer	AF131215.3
chr8	10985000	chr8	58835000	5.41E-03	promoter	super_enhancer	AF131215.3
chr8	11125000	chr8	22975000	1.95E-02	promoter	super_enhancer	LINC00529
chr8	11125000	chr8	27885000	1.82E-02	promoter	super_enhancer	LINC00529
chr8	11125000	chr8	129975000	2.90E-03	promoter	super_enhancer	LINC00529
chr8	11125000	chr8	130425000	3.60E-03	promoter	super_enhancer	LINC00529
chr8	11225000	chr8	11465000	3.18E-02	promoter	super_enhancer	FAM167A-AS1
chr8	11455000	chr8	22015000	3.11E-02	super_enhancer	promoter	LGI3
chr8	11455000	chr8	22755000	2.71E-02	super_enhancer	promoter	RP11-87E22.1
chr8	11455000	chr8	27745000	2.36E-02	super_enhancer	promoter	MIR4287
chr8	11455000	chr8	56685000	7.26E-03	super_enhancer	promoter	TGS1
chr8	11465000	chr8	37375000	1.10E-02	super_enhancer	promoter	RP11-150O12.6

chr8	11485000	chr8	16425000	2.11E-02	super_enhancer	promoter	MSR1
chr8	11485000	chr8	39615000	6.66E-03	super_enhancer	promoter	AC136365.1
chr8	11485000	chr8	98655000	4.15E-03	super_enhancer	promoter	MTDH
chr8	11485000	chr8	104155000	4.06E-03	super_enhancer	promoter	BAALC-AS2
chr8	11505000	chr8	28095000	1.65E-02	super_enhancer	promoter	RP11-380I10.3
chr8	11505000	chr8	29885000	1.50E-02	super_enhancer	promoter	MAP2K1P1
chr8	11505000	chr8	38035000	1.18E-02	super_enhancer	promoter	LSM1
chr8	11505000	chr8	124205000	4.63E-03	super_enhancer	promoter	RP11-539E17.3
chr8	11505000	chr8	127155000	3.23E-03	super_enhancer	promoter	AC090573.1
chr8	11515000	chr8	29485000	1.47E-02	super_enhancer	promoter	AC069181.1
chr8	11515000	chr8	124205000	3.83E-03	super_enhancer	promoter	RP11-539E17.3
chr8	11705000	chr8	28375000	1.46E-02	promoter	super_enhancer	RP11-589N15.2
chr8	11705000	chr8	123175000	4.03E-03	promoter	super_enhancer	RP11-589N15.2
chr8	11705000	chr8	125465000	5.13E-03	promoter	super_enhancer	RP11-589N15.2
chr8	11705000	chr8	126465000	5.53E-03	promoter	super_enhancer	RP11-589N15.2
chr8	11705000	chr8	133575000	4.77E-03	promoter	super_enhancer	RP11-589N15.2
chr8	11755000	chr8	23015000	2.13E-02	promoter	super_enhancer	RP11-589N15.1
chr8	11755000	chr8	25175000	1.57E-02	promoter	super_enhancer	RP11-589N15.1
chr8	11755000	chr8	27875000	1.65E-02	promoter	super_enhancer	RP11-589N15.1
chr8	11855000	chr8	22435000	2.70E-02	super_enhancer	promoter	PDLIM2
chr8	11855000	chr8	54905000	7.35E-03	super_enhancer	promoter	AC100821.2
chr8	11855000	chr8	132445000	2.37E-03	super_enhancer	promoter	SNORA72
chr8	11865000	chr8	18245000	1.41E-02	super_enhancer	promoter	RP11-685B14.3
chr8	11875000	chr8	49835000	5.68E-03	super_enhancer	promoter	SNAI2

chr8	11875000	chr8	126365000	4.42E-03	super_enhancer	promoter	RP11-550A5.2
chr8	11885000	chr8	31035000	9.07E-03	super_enhancer	promoter	RP11-363L24.3
chr8	11885000	chr8	142335000	2.52E-03	super_enhancer	promoter	RP11-10J21.6
chr8	11885000	chr8	145925000	3.35E-03	super_enhancer	promoter	AF186192.1
chr8	12035000	chr8	23345000	1.00E-02	promoter	super_enhancer	ALG1L11P
chr8	12535000	chr8	26455000	9.34E-03	promoter	super_enhancer	RP11-303G3.10
chr8	12535000	chr8	37145000	6.00E-03	promoter	super_enhancer	RP11-303G3.10
chr8	12555000	chr8	22595000	1.89E-02	promoter	super_enhancer	OR7E15P
chr8	12585000	chr8	28375000	1.07E-02	promoter	super_enhancer	MIR3926-1
chr8	12615000	chr8	27905000	1.35E-02	promoter	super_enhancer	LONRF1
chr8	13355000	chr8	17665000	2.11E-02	super_enhancer	promoter	RP11-806O11.1
chr8	13355000	chr8	23665000	8.63E-03	super_enhancer	promoter	RP11-175E9.2
chr8	13355000	chr8	37715000	6.36E-03	super_enhancer	promoter	RP11-205M5.3
chr8	13365000	chr8	23385000	1.30E-02	super_enhancer	promoter	SLC25A37
chr8	13365000	chr8	42995000	4.40E-03	super_enhancer	promoter	HGSNAT
chr8	14345000	chr8	22205000	2.35E-02	promoter	super_enhancer	RNU6-397P
chr8	15275000	chr8	61735000	3.26E-03	promoter	super_enhancer	TUSC3
chr8	17105000	chr8	28405000	1.18E-02	promoter	super_enhancer	CNOT7
chr8	17455000	chr8	23525000	2.53E-02	promoter	super_enhancer	RP11-349F21.1
chr8	17455000	chr8	38915000	8.05E-03	promoter	super_enhancer	RP11-349F21.1
chr8	17455000	chr8	129995000	3.54E-03	promoter	super_enhancer	RP11-349F21.1
chr8	17605000	chr8	26465000	1.21E-02	promoter	super_enhancer	RNA5SP256
chr8	17605000	chr8	101025000	3.04E-03	promoter	super_enhancer	RNA5SP256
chr8	17665000	chr8	22225000	2.89E-02	promoter	super_enhancer	RP11-806O11.1

chr8	17665000	chr8	127855000	2.43E-03	promoter	super_enhancer	RP11-806O11.1
chr8	18245000	chr8	26445000	2.12E-02	promoter	super_enhancer	RP11-685B14.3
chr8	19095000	chr8	27865000	1.02E-02	promoter	super_enhancer	RP11-618M23.1
chr8	19095000	chr8	125465000	2.39E-03	promoter	super_enhancer	RP11-618M23.1
chr8	19115000	chr8	23015000	4.28E-02	promoter	super_enhancer	RP11-618M23.4
chr8	19115000	chr8	26465000	1.25E-02	promoter	super_enhancer	RP11-618M23.4
chr8	19115000	chr8	58855000	4.85E-03	promoter	super_enhancer	RP11-618M23.4
chr8	19535000	chr8	23355000	3.87E-02	promoter	super_enhancer	RP11-1105O14.1
chr8	19535000	chr8	61725000	4.03E-03	promoter	super_enhancer	RP11-1105O14.1
chr8	19665000	chr8	22835000	4.18E-02	super_enhancer	promoter	RN7SL303P
chr8	19665000	chr8	33195000	9.30E-03	super_enhancer	promoter	RNU6-528P
chr8	20055000	chr8	27885000	2.21E-02	promoter	super_enhancer	ATP6V1B2
chr8	20055000	chr8	28385000	1.47E-02	promoter	super_enhancer	ATP6V1B2
chr8	20055000	chr8	29635000	1.97E-02	promoter	super_enhancer	ATP6V1B2
chr8	20055000	chr8	38895000	7.04E-03	promoter	super_enhancer	ATP6V1B2
chr8	20055000	chr8	61705000	4.47E-03	promoter	super_enhancer	ATP6V1B2
chr8	21155000	chr8	25175000	2.86E-02	promoter	super_enhancer	RP11-24P4.1
chr8	21155000	chr8	28355000	1.68E-02	promoter	super_enhancer	RP11-24P4.1
chr8	21155000	chr8	38935000	8.58E-03	promoter	super_enhancer	RP11-24P4.1
chr8	22015000	chr8	22195000	4.37E-02	promoter	super_enhancer	LGI3
chr8	22175000	chr8	57135000	7.94E-03	super_enhancer	promoter	RP11-140I16.3
chr8	22185000	chr8	26405000	4.17E-02	super_enhancer	promoter	RP11-1G11.2
chr8	22195000	chr8	28925000	2.86E-02	super_enhancer	promoter	CTD-2647L4.5
chr8	22205000	chr8	22435000	2.06E-03	super_enhancer	promoter	PDLIM2

chr8	22205000	chr8	29385000	2.46E-02	super_enhancer	promoter	RP11-486M23.1
chr8	22205000	chr8	37595000	1.66E-02	super_enhancer	promoter	RP11-863K10.7
chr8	22205000	chr8	42735000	1.10E-02	super_enhancer	promoter	RN7SL806P
chr8	22205000	chr8	48425000	9.35E-03	super_enhancer	promoter	RP11-697N18.3
chr8	22205000	chr8	86375000	3.52E-03	super_enhancer	promoter	CA2
chr8	22205000	chr8	145135000	4.44E-03	super_enhancer	promoter	CTD-3065J16.9
chr8	22215000	chr8	22425000	5.45E-03	super_enhancer	promoter	RP11-582J16.3
chr8	22215000	chr8	23385000	2.61E-02	super_enhancer	promoter	SLC25A37
chr8	22225000	chr8	41435000	1.26E-02	super_enhancer	promoter	AGPAT6
chr8	22225000	chr8	59415000	5.40E-03	super_enhancer	promoter	RP11-114M5.3
chr8	22225000	chr8	104035000	3.25E-03	super_enhancer	promoter	KB-1639H6.2
chr8	22225000	chr8	124665000	4.04E-03	super_enhancer	promoter	KLHL38
chr8	22425000	chr8	26455000	4.33E-02	promoter	super_enhancer	RP11-582J16.3
chr8	22435000	chr8	29625000	1.85E-02	promoter	super_enhancer	PDLIM2
chr8	22475000	chr8	26445000	4.48E-02	promoter	super_enhancer	RP11-582J16.5
chr8	22475000	chr8	29665000	2.11E-02	promoter	super_enhancer	RP11-582J16.5
chr8	22595000	chr8	29485000	2.90E-02	super_enhancer	promoter	AC069181.1
chr8	22595000	chr8	29785000	2.03E-02	super_enhancer	promoter	AC131254.1
chr8	22595000	chr8	29885000	2.37E-02	super_enhancer	promoter	MAP2K1P1
chr8	22595000	chr8	104495000	3.28E-03	super_enhancer	promoter	RP11-1C8.7
chr8	22595000	chr8	120725000	3.00E-03	super_enhancer	promoter	RN7SKP153
chr8	22735000	chr8	28395000	2.56E-02	promoter	super_enhancer	RP11-87E22.2
chr8	22735000	chr8	38925000	1.22E-02	promoter	super_enhancer	RP11-87E22.2
chr8	22755000	chr8	125465000	4.14E-03	promoter	super_enhancer	RP11-87E22.1

chr8	22835000	chr8	27875000	3.25E-02	promoter	super_enhancer	RN7SL303P
chr8	22835000	chr8	28365000	2.32E-02	promoter	super_enhancer	RN7SL303P
chr8	22835000	chr8	28395000	2.39E-02	promoter	super_enhancer	RN7SL303P
chr8	22845000	chr8	27895000	4.61E-04	promoter	super_enhancer	RHOBTB2
chr8	22845000	chr8	28405000	2.78E-02	promoter	super_enhancer	RHOBTB2
chr8	22925000	chr8	23525000	3.37E-02	promoter	super_enhancer	RP11-875O11.2
chr8	22975000	chr8	50655000	6.46E-03	super_enhancer	promoter	PSAT1P1
chr8	22985000	chr8	27695000	3.06E-02	super_enhancer	promoter	RP11-1149M3.2
chr8	22985000	chr8	38025000	1.72E-02	super_enhancer	promoter	RNU6-323P
chr8	22985000	chr8	145515000	4.40E-03	super_enhancer	promoter	BOP1
chr8	22995000	chr8	30055000	1.68E-02	super_enhancer	promoter	RP11-51J9.6
chr8	22995000	chr8	41155000	1.12E-02	super_enhancer	promoter	RNU6-895P
chr8	22995000	chr8	67875000	5.95E-03	super_enhancer	promoter	TCF24
chr8	23005000	chr8	27695000	3.48E-02	super_enhancer	promoter	RP11-1149M3.2
chr8	23005000	chr8	29815000	2.62E-02	super_enhancer	promoter	MIR3148
chr8	23005000	chr8	144625000	5.47E-03	super_enhancer	promoter	ZC3H3
chr8	23005000	chr8	145135000	5.09E-03	super_enhancer	promoter	CTD-3065J16.9
chr8	23015000	chr8	24035000	2.85E-03	super_enhancer	promoter	RP11-203E8.1
chr8	23315000	chr8	23525000	2.55E-02	promoter	super_enhancer	ENTPD4
chr8	23315000	chr8	23555000	6.11E-03	promoter	super_enhancer	ENTPD4
chr8	23315000	chr8	23765000	3.98E-02	promoter	super_enhancer	ENTPD4
chr8	23335000	chr8	23565000	5.46E-03	super_enhancer	promoter	NKX2-6
chr8	23335000	chr8	37595000	1.86E-02	super_enhancer	promoter	RP11-863K10.7
chr8	23335000	chr8	48045000	1.20E-02	super_enhancer	promoter	RNU6-819P

chr8	23335000	chr8	48115000	1.25E-02	super_enhancer	promoter	IGLV8OR8-1
chr8	23335000	chr8	108995000	1.84E-03	super_enhancer	promoter	NRBF2P4
chr8	23335000	chr8	125185000	3.27E-03	super_enhancer	promoter	FER1L6-AS2
chr8	23345000	chr8	27695000	3.50E-02	super_enhancer	promoter	RP11-1149M3.2
chr8	23365000	chr8	27005000	3.86E-02	super_enhancer	promoter	AC090150.1
chr8	23385000	chr8	29635000	3.16E-02	promoter	super_enhancer	SLC25A37
chr8	23525000	chr8	29885000	2.28E-02	super_enhancer	promoter	MAP2K1P1
chr8	23525000	chr8	84115000	3.12E-03	super_enhancer	promoter	RP11-813B8.1
chr8	23525000	chr8	134585000	3.49E-03	super_enhancer	promoter	ST3GAL1
chr8	23535000	chr8	29385000	2.93E-02	super_enhancer	promoter	RP11-486M23.1
chr8	23535000	chr8	53635000	7.80E-03	super_enhancer	promoter	CTD-3214K23.1
chr8	23545000	chr8	23935000	7.29E-03	super_enhancer	promoter	RNU1-148P
chr8	23545000	chr8	27695000	3.40E-02	super_enhancer	promoter	RP11-1149M3.2
chr8	23545000	chr8	29385000	2.92E-02	super_enhancer	promoter	RP11-486M23.1
chr8	23545000	chr8	37765000	1.18E-02	super_enhancer	promoter	RN7SL709P
chr8	23545000	chr8	41755000	1.10E-02	super_enhancer	promoter	ANK1
chr8	23545000	chr8	102405000	2.64E-03	super_enhancer	promoter	KB-1930G5.3
chr8	23545000	chr8	124625000	3.53E-03	super_enhancer	promoter	CTD-2552K11.2
chr8	23555000	chr8	113655000	2.63E-03	super_enhancer	promoter	MIR2053
chr8	23565000	chr8	126455000	4.45E-03	promoter	super_enhancer	NKX2-6
chr8	23665000	chr8	61695000	3.68E-03	promoter	super_enhancer	RP11-175E9.2
chr8	23755000	chr8	28095000	2.98E-02	super_enhancer	promoter	RP11-380I10.3
chr8	23755000	chr8	28175000	2.23E-02	super_enhancer	promoter	PNOC
chr8	23755000	chr8	67415000	5.30E-03	super_enhancer	promoter	RNU6-1324P

chr8	23755000	chr8	130845000	2.84E-03	super_enhancer	promoter	RP11-473O4.5
chr8	23755000	chr8	144295000	2.60E-03	super_enhancer	promoter	GPIHBP1
chr8	23765000	chr8	26115000	4.72E-03	super_enhancer	promoter	RP11-98P2.1
chr8	23765000	chr8	134405000	3.29E-03	super_enhancer	promoter	CTC-369M3.1
chr8	24035000	chr8	133345000	3.05E-03	promoter	super_enhancer	RP11-203E8.1
chr8	25175000	chr8	37265000	1.46E-02	super_enhancer	promoter	RP11-527N22.2
chr8	25185000	chr8	25285000	4.79E-02	super_enhancer	promoter	RP11-395I14.2
chr8	25185000	chr8	37375000	1.29E-02	super_enhancer	promoter	RP11-150O12.6
chr8	25185000	chr8	62215000	4.93E-03	super_enhancer	promoter	RP11-787D18.1
chr8	25195000	chr8	26115000	4.89E-02	super_enhancer	promoter	RP11-98P2.1
chr8	25195000	chr8	30585000	2.79E-02	super_enhancer	promoter	GSR
chr8	25195000	chr8	42395000	9.00E-03	super_enhancer	promoter	SMIM19
chr8	25195000	chr8	59465000	5.54E-03	super_enhancer	promoter	SDCBP
chr8	25195000	chr8	99645000	2.65E-03	super_enhancer	promoter	KB-1205A7.1
chr8	25285000	chr8	29635000	3.47E-02	promoter	super_enhancer	RP11-395I14.2
chr8	25285000	chr8	29675000	2.71E-02	promoter	super_enhancer	RP11-395I14.2
chr8	26445000	chr8	27555000	3.26E-02	super_enhancer	promoter	RP11-16P20.2
chr8	26455000	chr8	38585000	1.29E-02	super_enhancer	promoter	TACC1
chr8	26455000	chr8	69765000	4.06E-03	super_enhancer	promoter	RP11-865I6.2
chr8	26465000	chr8	38585000	9.62E-03	super_enhancer	promoter	TACC1
chr8	26725000	chr8	101155000	2.41E-03	promoter	super_enhancer	ADRA1A
chr8	26725000	chr8	102735000	2.22E-03	promoter	super_enhancer	ADRA1A
chr8	27005000	chr8	61735000	5.39E-03	promoter	super_enhancer	AC090150.1
chr8	27695000	chr8	27915000	3.82E-02	promoter	super_enhancer	RP11-1149M3.2

chr8	27865000	chr8	29785000	3.40E-03	super_enhancer	promoter	AC131254.1
chr8	27865000	chr8	98255000	3.04E-03	super_enhancer	promoter	KB-1000E4.2
chr8	27875000	chr8	31355000	2.58E-02	super_enhancer	promoter	RP11-566H8.1
chr8	27885000	chr8	28175000	1.81E-02	super_enhancer	promoter	PNOC
chr8	27885000	chr8	36135000	1.76E-02	super_enhancer	promoter	MTND6P19
chr8	27885000	chr8	38585000	2.02E-02	super_enhancer	promoter	TACC1
chr8	27885000	chr8	54905000	1.15E-02	super_enhancer	promoter	AC100821.2
chr8	27895000	chr8	28305000	3.23E-04	super_enhancer	promoter	RP11-181B11.2
chr8	27905000	chr8	53375000	7.19E-03	super_enhancer	promoter	ST18
chr8	27905000	chr8	56435000	9.18E-03	super_enhancer	promoter	RP11-628E19.2
chr8	27905000	chr8	82435000	2.86E-03	super_enhancer	promoter	FTH1P11
chr8	27905000	chr8	125185000	2.64E-03	super_enhancer	promoter	FER1L6-AS2
chr8	27915000	chr8	28175000	4.21E-03	super_enhancer	promoter	PNOC
chr8	27915000	chr8	42235000	1.69E-02	super_enhancer	promoter	DKK4
chr8	27915000	chr8	49825000	1.30E-02	super_enhancer	promoter	RP11-22C8.1
chr8	27915000	chr8	143825000	2.87E-03	super_enhancer	promoter	SLURP1
chr8	28355000	chr8	28925000	3.51E-02	super_enhancer	promoter	CTD-2647L4.5
chr8	28355000	chr8	29815000	1.30E-02	super_enhancer	promoter	MIR3148
chr8	28355000	chr8	33295000	3.15E-02	super_enhancer	promoter	RP11-359B20.1
chr8	28355000	chr8	37765000	1.40E-02	super_enhancer	promoter	RN7SL709P
chr8	28355000	chr8	38965000	1.63E-02	super_enhancer	promoter	ADAM32
chr8	28355000	chr8	95545000	3.59E-03	super_enhancer	promoter	RP11-267M23.3
chr8	28365000	chr8	33195000	2.32E-02	super_enhancer	promoter	RNU6-528P
chr8	28365000	chr8	37575000	1.63E-02	super_enhancer	promoter	RP11-863K10.2

chr8	28365000	chr8	56435000	8.31E-03	super_enhancer	promoter	RP11-628E19.2
chr8	28365000	chr8	86435000	3.58E-03	super_enhancer	promoter	RP11-317J10.4
chr8	28375000	chr8	50425000	5.49E-03	super_enhancer	promoter	RP11-738G5.1
chr8	28375000	chr8	53805000	7.44E-03	super_enhancer	promoter	AC087348.1
chr8	28375000	chr8	62115000	6.64E-03	super_enhancer	promoter	NPM1P6
chr8	28385000	chr8	57015000	6.95E-03	super_enhancer	promoter	NPM1P21
chr8	28395000	chr8	38035000	1.59E-02	super_enhancer	promoter	LSM1
chr8	28405000	chr8	33195000	2.90E-02	super_enhancer	promoter	RNU6-528P
chr8	28405000	chr8	37575000	2.02E-02	super_enhancer	promoter	RP11-863K10.2
chr8	28405000	chr8	41135000	1.56E-02	super_enhancer	promoter	CTD-3080F16.3
chr8	28405000	chr8	49535000	1.34E-02	super_enhancer	promoter	RP11-567J20.1
chr8	28405000	chr8	96145000	4.53E-03	super_enhancer	promoter	PLEKHF2
chr8	28405000	chr8	101505000	3.84E-03	super_enhancer	promoter	KB-1615E4.3
chr8	28405000	chr8	145515000	4.13E-03	super_enhancer	promoter	BOP1
chr8	28655000	chr8	38925000	1.43E-02	promoter	super_enhancer	RP11-662B19.2
chr8	28925000	chr8	127795000	3.51E-03	promoter	super_enhancer	CTD-2647L4.5
chr8	29075000	chr8	38905000	1.09E-02	promoter	super_enhancer	HMGB1P23
chr8	29075000	chr8	38935000	1.42E-02	promoter	super_enhancer	HMGB1P23
chr8	29385000	chr8	29665000	1.75E-02	promoter	super_enhancer	RP11-486M23.1
chr8	29385000	chr8	61735000	7.67E-03	promoter	super_enhancer	RP11-486M23.1
chr8	29385000	chr8	130425000	3.12E-03	promoter	super_enhancer	RP11-486M23.1
chr8	29385000	chr8	144425000	2.91E-03	promoter	super_enhancer	RP11-486M23.1
chr8	29405000	chr8	38905000	1.30E-02	promoter	super_enhancer	RP11-486M23.2
chr8	29445000	chr8	38925000	1.76E-02	promoter	super_enhancer	RP11-486M23.3

chr8	29445000	chr8	66525000	7.77E-03	promoter	super_enhancer	RP11-486M23.3
chr8	29485000	chr8	101465000	3.24E-03	promoter	super_enhancer	AC069181.1
chr8	29595000	chr8	33085000	4.60E-02	super_enhancer	promoter	RP11-451O18.1
chr8	29595000	chr8	37375000	2.01E-02	super_enhancer	promoter	RP11-150O12.6
chr8	29595000	chr8	42395000	1.18E-02	super_enhancer	promoter	SMIM19
chr8	29595000	chr8	54625000	8.31E-03	super_enhancer	promoter	RP11-463C14.1
chr8	29605000	chr8	145515000	3.71E-03	super_enhancer	promoter	BOP1
chr8	29635000	chr8	37265000	2.72E-02	super_enhancer	promoter	RP11-527N22.2
chr8	29635000	chr8	39725000	1.67E-02	super_enhancer	promoter	KB-1582A10.2
chr8	29635000	chr8	51675000	8.20E-03	super_enhancer	promoter	CYCSP22
chr8	29635000	chr8	53635000	1.13E-02	super_enhancer	promoter	CTD-3214K23.1
chr8	29635000	chr8	101845000	4.29E-03	super_enhancer	promoter	RNU4-83P
chr8	29635000	chr8	124015000	3.64E-03	super_enhancer	promoter	RP11-557C18.3
chr8	29645000	chr8	38025000	2.39E-02	super_enhancer	promoter	RNU6-323P
chr8	29645000	chr8	144655000	3.78E-03	super_enhancer	promoter	MROH6
chr8	29655000	chr8	39725000	1.23E-02	super_enhancer	promoter	KB-1582A10.2
chr8	29655000	chr8	55065000	8.61E-03	super_enhancer	promoter	RNU6ATAC32P
chr8	29655000	chr8	74965000	4.68E-03	super_enhancer	promoter	RP11-6I2.3
chr8	29665000	chr8	29785000	1.07E-02	super_enhancer	promoter	AC131254.1
chr8	29665000	chr8	125835000	2.81E-03	super_enhancer	promoter	MIR4662B
chr8	29665000	chr8	142335000	2.45E-03	super_enhancer	promoter	RP11-10J21.6
chr8	29815000	chr8	66535000	7.10E-03	promoter	super_enhancer	MIR3148
chr8	29885000	chr8	47375000	7.76E-03	promoter	super_enhancer	MAP2K1P1
chr8	29885000	chr8	123185000	2.70E-03	promoter	super_enhancer	MAP2K1P1

chr8	30095000	chr8	125435000	3.29E-03	promoter	super_enhancer	HSPA8P11
chr8	30495000	chr8	118005000	2.51E-03	promoter	super_enhancer	SMIM18
chr8	30585000	chr8	125455000	3.92E-03	promoter	super_enhancer	GSR
chr8	30585000	chr8	140635000	3.37E-03	promoter	super_enhancer	GSR
chr8	33085000	chr8	101035000	3.39E-03	promoter	super_enhancer	RP11-451O18.1
chr8	33425000	chr8	123175000	3.44E-03	promoter	super_enhancer	RNF122
chr8	34085000	chr8	37145000	2.59E-02	promoter	super_enhancer	RP1-84O15.2
chr8	34085000	chr8	61705000	3.99E-03	promoter	super_enhancer	RP1-84O15.2
chr8	36745000	chr8	61725000	7.06E-03	promoter	super_enhancer	TPT1P8
chr8	36745000	chr8	101145000	3.56E-03	promoter	super_enhancer	TPT1P8
chr8	36745000	chr8	130445000	2.50E-03	promoter	super_enhancer	TPT1P8
chr8	37145000	chr8	41155000	3.64E-02	super_enhancer	promoter	RNU6-895P
chr8	37375000	chr8	93855000	3.58E-03	promoter	super_enhancer	RP11-150O12.6
chr8	37575000	chr8	101025000	4.09E-03	promoter	super_enhancer	RP11-863K10.2
chr8	37595000	chr8	66505000	9.93E-03	promoter	super_enhancer	RP11-863K10.7
chr8	37765000	chr8	125465000	2.90E-03	promoter	super_enhancer	RN7SL709P
chr8	38025000	chr8	129995000	3.94E-03	promoter	super_enhancer	RNU6-323P
chr8	38025000	chr8	143975000	3.52E-03	promoter	super_enhancer	RNU6-323P
chr8	38035000	chr8	38905000	3.25E-02	promoter	super_enhancer	LSM1
chr8	38585000	chr8	125445000	3.28E-03	promoter	super_enhancer	TACC1
chr8	38585000	chr8	126445000	3.49E-03	promoter	super_enhancer	TACC1
chr8	38705000	chr8	126445000	3.04E-03	promoter	super_enhancer	RP11-723D22.3
chr8	38905000	chr8	42065000	4.34E-02	super_enhancer	promoter	PLAT
chr8	38905000	chr8	82025000	3.27E-03	super_enhancer	promoter	PAG1

chr8	38905000	chr8	124215000	2.71E-03	super_enhancer	promoter	FAM83A-AS1
chr8	38925000	chr8	119775000	1.94E-03	super_enhancer	promoter	RPS26P35
chr8	38925000	chr8	128095000	2.53E-03	super_enhancer	promoter	PCAT2
chr8	38925000	chr8	128215000	1.89E-03	super_enhancer	promoter	CASC19
chr8	38935000	chr8	56685000	1.09E-02	super_enhancer	promoter	TGS1
chr8	38935000	chr8	79675000	3.41E-03	super_enhancer	promoter	PRKRIRP7
chr8	40755000	chr8	47345000	2.04E-02	promoter	super_enhancer	ZMAT4
chr8	41155000	chr8	143975000	3.26E-03	promoter	super_enhancer	RNU6-895P
chr8	42195000	chr8	143585000	2.99E-03	promoter	super_enhancer	POLB
chr8	42395000	chr8	126435000	3.09E-03	promoter	super_enhancer	SMIM19
chr8	47345000	chr8	56055000	1.93E-02	super_enhancer	promoter	RP11-386G21.1
chr8	47345000	chr8	70855000	1.03E-02	super_enhancer	promoter	SDCBPP2
chr8	47345000	chr8	87355000	6.42E-03	super_enhancer	promoter	WWP1
chr8	47375000	chr8	54075000	1.34E-02	super_enhancer	promoter	RP11-162D9.1
chr8	47385000	chr8	56685000	1.10E-02	super_enhancer	promoter	TGS1
chr8	47385000	chr8	80485000	4.63E-03	super_enhancer	promoter	RPL3P9
chr8	47385000	chr8	99405000	4.12E-03	super_enhancer	promoter	KB-1517D11.3
chr8	47395000	chr8	73165000	4.90E-03	super_enhancer	promoter	RP11-656G20.1
chr8	47395000	chr8	82445000	4.87E-03	super_enhancer	promoter	FABP12
chr8	47395000	chr8	95825000	4.05E-03	super_enhancer	promoter	INTS8
chr8	47395000	chr8	97275000	3.52E-03	super_enhancer	promoter	MTERF3
chr8	47785000	chr8	58835000	1.32E-02	promoter	super_enhancer	RP11-101E19.7
chr8	48075000	chr8	101465000	3.50E-03	promoter	super_enhancer	NDUFA5P12
chr8	48115000	chr8	61715000	1.71E-02	promoter	super_enhancer	IGLV8OR8-1

chr8	48425000	chr8	125435000	3.84E-03	promoter	super_enhancer	RP11-697N18.3
chr8	48505000	chr8	61715000	1.35E-02	promoter	super_enhancer	RP11-697N18.2
chr8	49825000	chr8	102765000	5.54E-03	promoter	super_enhancer	RP11-22C8.1
chr8	50265000	chr8	126545000	2.68E-03	promoter	super_enhancer	RFPL4AP7
chr8	50425000	chr8	61705000	9.86E-03	promoter	super_enhancer	RP11-738G5.1
chr8	50655000	chr8	140595000	2.43E-03	promoter	super_enhancer	PSAT1P1
chr8	51675000	chr8	66535000	1.10E-02	promoter	super_enhancer	CYCSP22
chr8	53375000	chr8	61735000	1.73E-02	promoter	super_enhancer	ST18
chr8	53375000	chr8	61755000	1.55E-02	promoter	super_enhancer	ST18
chr8	53805000	chr8	61785000	2.53E-02	promoter	super_enhancer	AC087348.1
chr8	53805000	chr8	123175000	3.35E-03	promoter	super_enhancer	AC087348.1
chr8	54075000	chr8	93825000	5.13E-03	promoter	super_enhancer	RP11-162D9.1
chr8	54625000	chr8	61735000	2.00E-02	promoter	super_enhancer	RP11-463C14.1
chr8	54905000	chr8	58835000	4.59E-02	promoter	super_enhancer	AC100821.2
chr8	54935000	chr8	61755000	2.58E-02	promoter	super_enhancer	TCEA1
chr8	54985000	chr8	80085000	6.58E-03	promoter	super_enhancer	TDGF1P5
chr8	55065000	chr8	61755000	2.30E-02	promoter	super_enhancer	RNU6ATAC32P
chr8	55105000	chr8	61755000	2.05E-02	promoter	super_enhancer	RP11-626A5.2
chr8	56015000	chr8	101465000	4.64E-03	promoter	super_enhancer	XKR4
chr8	56015000	chr8	140595000	2.93E-03	promoter	super_enhancer	XKR4
chr8	56055000	chr8	93905000	7.16E-03	promoter	super_enhancer	RP11-386G21.1
chr8	56435000	chr8	61715000	3.61E-02	promoter	super_enhancer	RP11-628E19.2
chr8	56435000	chr8	61775000	4.20E-02	promoter	super_enhancer	RP11-628E19.2
chr8	56685000	chr8	61755000	2.89E-02	promoter	super_enhancer	TGS1

chr8	56805000	chr8	93905000	8.95E-03	promoter	super_enhancer	RP11-318K15.2
chr8	56805000	chr8	102755000	9.55E-03	promoter	super_enhancer	RP11-318K15.2
chr8	56805000	chr8	143525000	4.70E-03	promoter	super_enhancer	RP11-318K15.2
chr8	56955000	chr8	61785000	4.79E-02	promoter	super_enhancer	AC046176.1
chr8	56965000	chr8	61695000	3.14E-02	promoter	super_enhancer	RP11-446E9.1
chr8	56965000	chr8	125445000	4.42E-03	promoter	super_enhancer	RP11-446E9.1
chr8	57015000	chr8	61695000	2.85E-02	promoter	super_enhancer	NPM1P21
chr8	57015000	chr8	61705000	3.18E-02	promoter	super_enhancer	NPM1P21
chr8	57015000	chr8	140585000	3.15E-03	promoter	super_enhancer	NPM1P21
chr8	57135000	chr8	127735000	3.24E-03	promoter	super_enhancer	RP11-140I16.3
chr8	57135000	chr8	143535000	3.89E-03	promoter	super_enhancer	RP11-140I16.3
chr8	58055000	chr8	61785000	4.50E-02	promoter	super_enhancer	LINC01606
chr8	58055000	chr8	66535000	1.90E-02	promoter	super_enhancer	LINC01606
chr8	58095000	chr8	61725000	3.07E-02	promoter	super_enhancer	AC025674.1
chr8	58305000	chr8	61715000	4.90E-02	promoter	super_enhancer	RPL30P10
chr8	58305000	chr8	140605000	3.42E-03	promoter	super_enhancer	RPL30P10
chr8	58305000	chr8	143515000	3.47E-03	promoter	super_enhancer	RPL30P10
chr8	58405000	chr8	61715000	4.05E-02	promoter	super_enhancer	RP11-246K15.1
chr8	58405000	chr8	93855000	5.03E-03	promoter	super_enhancer	RP11-246K15.1
chr8	58405000	chr8	127735000	2.34E-03	promoter	super_enhancer	RP11-246K15.1
chr8	58845000	chr8	66475000	1.96E-02	super_enhancer	promoter	LINC01299
chr8	58845000	chr8	91015000	7.65E-03	super_enhancer	promoter	NBN
chr8	58845000	chr8	99615000	5.77E-03	super_enhancer	promoter	KB-1205A7.2
chr8	58845000	chr8	119965000	3.46E-03	super_enhancer	promoter	TNFRSF11B

chr8	59185000	chr8	80105000	1.14E-02	promoter	super_enhancer	RP11-716D16.1
chr8	59185000	chr8	93825000	8.03E-03	promoter	super_enhancer	RP11-716D16.1
chr8	59185000	chr8	127225000	3.22E-03	promoter	super_enhancer	RP11-716D16.1
chr8	59415000	chr8	93935000	6.60E-03	promoter	super_enhancer	RP11-114M5.3
chr8	59415000	chr8	102765000	5.70E-03	promoter	super_enhancer	RP11-114M5.3
chr8	59465000	chr8	102755000	6.46E-03	promoter	super_enhancer	SDCBP
chr8	59905000	chr8	133575000	3.70E-03	promoter	super_enhancer	RP11-328K2.1
chr8	61695000	chr8	67975000	2.65E-02	super_enhancer	promoter	CSPP1
chr8	61695000	chr8	145635000	3.20E-03	super_enhancer	promoter	CPSF1
chr8	61705000	chr8	73795000	8.96E-03	super_enhancer	promoter	RP11-1145L24.1
chr8	61705000	chr8	101505000	7.36E-03	super_enhancer	promoter	KB-1615E4.3
chr8	61705000	chr8	125185000	3.41E-03	super_enhancer	promoter	FER1L6-AS2
chr8	61715000	chr8	134585000	4.01E-03	super_enhancer	promoter	ST3GAL1
chr8	61715000	chr8	144925000	4.34E-03	super_enhancer	promoter	AC234917.1
chr8	61725000	chr8	124015000	3.43E-03	super_enhancer	promoter	RP11-557C18.3
chr8	61725000	chr8	142335000	2.49E-03	super_enhancer	promoter	RP11-10J21.6
chr8	61725000	chr8	142365000	3.12E-03	super_enhancer	promoter	CTD-3064M3.3
chr8	61735000	chr8	70985000	2.52E-02	super_enhancer	promoter	PRDM14
chr8	61735000	chr8	75015000	1.54E-02	super_enhancer	promoter	RP11-6I2.4
chr8	61735000	chr8	88985000	1.14E-02	super_enhancer	promoter	CTB-118P15.2
chr8	61755000	chr8	97505000	8.65E-03	super_enhancer	promoter	SDC2
chr8	61755000	chr8	120885000	4.28E-03	super_enhancer	promoter	DEPTOR
chr8	61755000	chr8	126105000	4.40E-03	super_enhancer	promoter	KIAA0196
chr8	61765000	chr8	68255000	3.05E-02	super_enhancer	promoter	RP11-7F18.2

chr8	61765000	chr8	87355000	9.90E-03	super_enhancer	promoter	WWP1
chr8	61765000	chr8	103635000	7.05E-03	super_enhancer	promoter	POU5F1P2
chr8	61765000	chr8	106285000	5.48E-03	super_enhancer	promoter	AC041039.1
chr8	61775000	chr8	67415000	4.16E-02	super_enhancer	promoter	RNU6-1324P
chr8	61775000	chr8	89745000	1.08E-02	super_enhancer	promoter	AC090578.1
chr8	61785000	chr8	67415000	4.42E-02	super_enhancer	promoter	RNU6-1324P
chr8	61785000	chr8	124195000	7.16E-03	super_enhancer	promoter	RP11-539E17.5
chr8	61785000	chr8	128095000	4.42E-03	super_enhancer	promoter	PCAT2
chr8	61785000	chr8	144295000	4.06E-03	super_enhancer	promoter	GPIHBP1
chr8	61785000	chr8	144635000	4.01E-03	super_enhancer	promoter	GSDMD
chr8	62115000	chr8	141215000	4.50E-03	promoter	super_enhancer	NPM1P6
chr8	62115000	chr8	143975000	3.61E-03	promoter	super_enhancer	NPM1P6
chr8	62215000	chr8	140635000	3.07E-03	promoter	super_enhancer	RP11-787D18.1
chr8	62545000	chr8	66535000	1.45E-03	promoter	super_enhancer	RN7SKP97
chr8	62545000	chr8	143585000	4.00E-03	promoter	super_enhancer	RN7SKP97
chr8	65285000	chr8	102765000	8.28E-03	promoter	super_enhancer	MIR124-2HG
chr8	65285000	chr8	127205000	2.97E-03	promoter	super_enhancer	MIR124-2HG
chr8	65285000	chr8	143975000	3.29E-03	promoter	super_enhancer	MIR124-2HG
chr8	65485000	chr8	66505000	3.30E-02	promoter	super_enhancer	RP11-21C4.5
chr8	65485000	chr8	66525000	3.32E-02	promoter	super_enhancer	RP11-21C4.5
chr8	65485000	chr8	80105000	1.05E-02	promoter	super_enhancer	RP11-21C4.5
chr8	65485000	chr8	93845000	8.12E-03	promoter	super_enhancer	RP11-21C4.5
chr8	65495000	chr8	66535000	9.28E-03	promoter	super_enhancer	RP11-21C4.1
chr8	65495000	chr8	80105000	1.41E-02	promoter	super_enhancer	RP11-21C4.1

chr8	65495000	chr8	130445000	3.97E-03	promoter	super_enhancer	RP11-21C4.1
chr8	66505000	chr8	99205000	6.13E-03	super_enhancer	promoter	RNU6-914P
chr8	66515000	chr8	70855000	4.62E-02	super_enhancer	promoter	SDCBPP2
chr8	66515000	chr8	81725000	1.59E-02	super_enhancer	promoter	HMGB1P41
chr8	66525000	chr8	70855000	4.71E-02	super_enhancer	promoter	SDCBPP2
chr8	66525000	chr8	88615000	1.13E-02	super_enhancer	promoter	IARS2P1
chr8	66525000	chr8	120875000	5.98E-03	super_enhancer	promoter	RN7SL396P
chr8	67025000	chr8	140465000	4.18E-03	promoter	super_enhancer	AC084082.3
chr8	67415000	chr8	126545000	6.09E-03	promoter	super_enhancer	RNU6-1324P
chr8	67415000	chr8	141205000	3.96E-03	promoter	super_enhancer	RNU6-1324P
chr8	67625000	chr8	93945000	8.50E-03	promoter	super_enhancer	SGK3
chr8	67975000	chr8	133395000	5.38E-03	promoter	super_enhancer	CSPP1
chr8	68255000	chr8	143505000	5.06E-03	promoter	super_enhancer	RP11-7F18.2
chr8	69245000	chr8	126465000	5.68E-03	promoter	super_enhancer	C8orf34-AS1
chr8	69765000	chr8	101015000	7.92E-03	promoter	super_enhancer	RP11-865I6.2
chr8	69765000	chr8	102745000	6.45E-03	promoter	super_enhancer	RP11-865I6.2
chr8	70835000	chr8	133385000	4.93E-03	promoter	super_enhancer	RN7SL675P
chr8	70855000	chr8	126565000	6.58E-03	promoter	super_enhancer	SDCBPP2
chr8	71185000	chr8	93905000	9.38E-03	promoter	super_enhancer	BTF3P12
chr8	71185000	chr8	143975000	2.93E-03	promoter	super_enhancer	BTF3P12
chr8	72275000	chr8	93855000	7.43E-03	promoter	super_enhancer	EYA1
chr8	72505000	chr8	130435000	2.60E-03	promoter	super_enhancer	RP11-1102P16.1
chr8	72705000	chr8	133375000	3.48E-03	promoter	super_enhancer	RP11-281N10.1
chr8	72745000	chr8	125415000	5.75E-03	promoter	super_enhancer	AC104012.1

chr8	73165000	chr8	93845000	1.10E-02	promoter	super_enhancer	RP11-656G20.1
chr8	73165000	chr8	101465000	7.22E-03	promoter	super_enhancer	RP11-656G20.1
chr8	73165000	chr8	102745000	6.53E-03	promoter	super_enhancer	RP11-656G20.1
chr8	73165000	chr8	133385000	3.99E-03	promoter	super_enhancer	RP11-656G20.1
chr8	73375000	chr8	93835000	1.16E-02	promoter	super_enhancer	RP11-351E7.1
chr8	73375000	chr8	101155000	1.04E-02	promoter	super_enhancer	RP11-351E7.1
chr8	73575000	chr8	117985000	6.05E-03	promoter	super_enhancer	RP11-313C15.2
chr8	73795000	chr8	143945000	2.56E-03	promoter	super_enhancer	RP11-1145L24.1
chr8	74185000	chr8	117985000	6.22E-03	promoter	super_enhancer	AC008598.1
chr8	74185000	chr8	126555000	6.12E-03	promoter	super_enhancer	AC008598.1
chr8	74885000	chr8	93955000	1.31E-02	promoter	super_enhancer	TCEB1
chr8	75015000	chr8	126555000	6.21E-03	promoter	super_enhancer	RP11-6I2.4
chr8	75235000	chr8	93845000	1.44E-02	promoter	super_enhancer	JPH1
chr8	75235000	chr8	101035000	1.16E-02	promoter	super_enhancer	JPH1
chr8	76215000	chr8	93955000	1.11E-02	promoter	super_enhancer	HIGD1AP6
chr8	76215000	chr8	101465000	7.55E-03	promoter	super_enhancer	HIGD1AP6
chr8	79675000	chr8	118025000	4.45E-03	promoter	super_enhancer	PRKRIRP7
chr8	80085000	chr8	84115000	2.35E-02	super_enhancer	promoter	RP11-813B8.1
chr8	80095000	chr8	80485000	4.41E-02	super_enhancer	promoter	RPL3P9
chr8	80095000	chr8	104105000	1.10E-02	super_enhancer	promoter	AP003550.1
chr8	80105000	chr8	101395000	4.72E-05	super_enhancer	promoter	MIR4471
chr8	82155000	chr8	143535000	3.88E-03	promoter	super_enhancer	RP11-363E6.1
chr8	82155000	chr8	143565000	3.61E-03	promoter	super_enhancer	RP11-363E6.1
chr8	82395000	chr8	143535000	3.66E-03	promoter	super_enhancer	FABP4

chr8	82435000	chr8	117985000	6.00E-03	promoter	super_enhancer	FTH1P11
chr8	82445000	chr8	129995000	7.36E-03	promoter	super_enhancer	FABP12
chr8	82645000	chr8	140635000	5.74E-03	promoter	super_enhancer	ZFAND1
chr8	82755000	chr8	93825000	1.77E-02	promoter	super_enhancer	SNX16
chr8	83205000	chr8	101025000	8.76E-03	promoter	super_enhancer	HNRNPA1P4
chr8	83205000	chr8	125415000	4.89E-03	promoter	super_enhancer	HNRNPA1P4
chr8	83205000	chr8	140635000	3.19E-03	promoter	super_enhancer	HNRNPA1P4
chr8	83205000	chr8	144425000	2.40E-03	promoter	super_enhancer	HNRNPA1P4
chr8	84115000	chr8	101155000	1.43E-02	promoter	super_enhancer	RP11-813B8.1
chr8	84115000	chr8	117985000	7.82E-03	promoter	super_enhancer	RP11-813B8.1
chr8	84115000	chr8	140625000	4.99E-03	promoter	super_enhancer	RP11-813B8.1
chr8	84315000	chr8	93845000	1.59E-02	promoter	super_enhancer	LINC01419
chr8	84825000	chr8	102745000	1.05E-02	promoter	super_enhancer	RP11-120I21.2
chr8	85095000	chr8	125425000	7.44E-03	promoter	super_enhancer	RALYL
chr8	86285000	chr8	140465000	5.08E-03	promoter	super_enhancer	CA3
chr8	86375000	chr8	93885000	1.97E-02	promoter	super_enhancer	CA2
chr8	86375000	chr8	101465000	1.45E-02	promoter	super_enhancer	CA2
chr8	86435000	chr8	93835000	2.59E-02	promoter	super_enhancer	RP11-317J10.4
chr8	87495000	chr8	125435000	1.01E-02	promoter	super_enhancer	NTAN1P2
chr8	87515000	chr8	101145000	1.90E-02	promoter	super_enhancer	SLC2A3P4
chr8	87675000	chr8	93855000	2.18E-02	promoter	super_enhancer	UBE2Q2P10
chr8	87675000	chr8	141215000	5.29E-03	promoter	super_enhancer	UBE2Q2P10
chr8	88985000	chr8	126555000	1.16E-02	promoter	super_enhancer	CTB-118P15.2
chr8	93365000	chr8	102745000	1.76E-02	promoter	super_enhancer	RP11-700E23.3

chr8	93365000	chr8	102755000	2.62E-02	promoter	super_enhancer	RP11-700E23.3
chr8	93365000	chr8	118005000	1.20E-02	promoter	super_enhancer	RP11-700E23.3
chr8	93365000	chr8	143565000	5.94E-03	promoter	super_enhancer	RP11-700E23.3
chr8	93365000	chr8	143945000	6.32E-03	promoter	super_enhancer	RP11-700E23.3
chr8	93825000	chr8	104155000	2.45E-02	super_enhancer	promoter	BAALC-AS2
chr8	93825000	chr8	118175000	1.15E-02	super_enhancer	promoter	RN7SL826P
chr8	93835000	chr8	95565000	1.58E-02	super_enhancer	promoter	RP11-267M23.4
chr8	93835000	chr8	99205000	2.38E-02	super_enhancer	promoter	RNU6-914P
chr8	93835000	chr8	105435000	1.95E-02	super_enhancer	promoter	KB-1552D7.2
chr8	93845000	chr8	99475000	4.49E-02	super_enhancer	promoter	RNU6-748P
chr8	93845000	chr8	134405000	8.20E-03	super_enhancer	promoter	CTC-369M3.1
chr8	93855000	chr8	104155000	2.42E-02	super_enhancer	promoter	BAALC-AS2
chr8	93855000	chr8	104515000	1.64E-02	super_enhancer	promoter	RP11-1C8.4
chr8	93855000	chr8	109665000	1.13E-02	super_enhancer	promoter	RP11-790J24.2
chr8	93875000	chr8	99645000	2.96E-02	super_enhancer	promoter	KB-1205A7.1
chr8	93875000	chr8	107995000	1.78E-02	super_enhancer	promoter	KB-1433G4.1
chr8	93875000	chr8	110985000	1.67E-02	super_enhancer	promoter	RP11-696P8.2
chr8	93875000	chr8	134585000	8.76E-03	super_enhancer	promoter	ST3GAL1
chr8	93885000	chr8	95275000	3.60E-02	super_enhancer	promoter	GEM
chr8	93885000	chr8	104495000	1.81E-02	super_enhancer	promoter	RP11-1C8.7
chr8	93885000	chr8	140115000	6.20E-03	super_enhancer	promoter	RP11-324F11.1
chr8	93895000	chr8	97005000	3.92E-02	super_enhancer	promoter	RP11-31K23.2
chr8	93895000	chr8	127595000	8.60E-03	super_enhancer	promoter	RP11-89K10.2
chr8	93905000	chr8	106285000	1.94E-02	super_enhancer	promoter	AC041039.1

chr8	93905000	chr8	126365000	1.20E-02	super_enhancer	promoter	RP11-550A5.2
chr8	93905000	chr8	132915000	7.87E-03	super_enhancer	promoter	EFR3A
chr8	93935000	chr8	101735000	2.65E-02	super_enhancer	promoter	PABPC1
chr8	93945000	chr8	113655000	1.00E-02	super_enhancer	promoter	MIR2053
chr8	93985000	chr8	102795000	1.65E-02	promoter	super_enhancer	IRF5P1
chr8	93985000	chr8	125405000	8.31E-03	promoter	super_enhancer	IRF5P1
chr8	94955000	chr8	102735000	3.51E-02	promoter	super_enhancer	PSMA2P2
chr8	94985000	chr8	143945000	9.59E-03	promoter	super_enhancer	AC105081.1
chr8	95215000	chr8	125465000	1.56E-02	promoter	super_enhancer	KB-1247B1.1
chr8	95215000	chr8	140465000	8.67E-03	promoter	super_enhancer	KB-1247B1.1
chr8	95275000	chr8	101155000	4.99E-02	promoter	super_enhancer	GEM
chr8	95275000	chr8	127905000	1.04E-02	promoter	super_enhancer	GEM
chr8	95365000	chr8	102785000	4.57E-02	promoter	super_enhancer	RPS4XP10
chr8	95365000	chr8	143535000	1.01E-02	promoter	super_enhancer	RPS4XP10
chr8	95545000	chr8	141215000	9.32E-03	promoter	super_enhancer	RP11-267M23.3
chr8	95805000	chr8	118025000	1.25E-02	promoter	super_enhancer	KB-1608C10.2
chr8	96085000	chr8	102805000	3.35E-02	promoter	super_enhancer	RP11-320N21.1
chr8	96145000	chr8	102745000	3.48E-02	promoter	super_enhancer	PLEKHF2
chr8	96145000	chr8	102805000	3.89E-02	promoter	super_enhancer	PLEKHF2
chr8	96145000	chr8	125445000	1.57E-02	promoter	super_enhancer	PLEKHF2
chr8	96415000	chr8	101455000	4.64E-02	promoter	super_enhancer	RP11-328L11.1
chr8	96415000	chr8	102735000	3.50E-02	promoter	super_enhancer	RP11-328L11.1
chr8	97005000	chr8	127735000	8.03E-03	promoter	super_enhancer	RP11-31K23.2
chr8	97005000	chr8	133575000	8.59E-03	promoter	super_enhancer	RP11-31K23.2

chr8	97275000	chr8	143585000	9.11E-03	promoter	super_enhancer	MTERF3
chr8	97505000	chr8	140625000	1.04E-02	promoter	super_enhancer	SDC2
chr8	98255000	chr8	126435000	1.78E-02	promoter	super_enhancer	KB-1000E4.2
chr8	98255000	chr8	129995000	1.32E-02	promoter	super_enhancer	KB-1000E4.2
chr8	98255000	chr8	140605000	1.00E-02	promoter	super_enhancer	KB-1000E4.2
chr8	98655000	chr8	133345000	1.47E-02	promoter	super_enhancer	MTDH
chr8	98655000	chr8	143935000	7.20E-03	promoter	super_enhancer	MTDH
chr8	98865000	chr8	125455000	1.47E-02	promoter	super_enhancer	KB-1589B1.4
chr8	99205000	chr8	101015000	7.11E-03	promoter	super_enhancer	RNU6-914P
chr8	99205000	chr8	101145000	7.11E-03	promoter	super_enhancer	RNU6-914P
chr8	99205000	chr8	130435000	5.25E-03	promoter	super_enhancer	RNU6-914P
chr8	99205000	chr8	133365000	8.75E-03	promoter	super_enhancer	RNU6-914P
chr8	99405000	chr8	101045000	2.66E-03	promoter	super_enhancer	KB-1517D11.3
chr8	99405000	chr8	101145000	3.60E-02	promoter	super_enhancer	KB-1517D11.3
chr8	99405000	chr8	101455000	2.00E-02	promoter	super_enhancer	KB-1517D11.3
chr8	99405000	chr8	102785000	6.76E-03	promoter	super_enhancer	KB-1517D11.3
chr8	99405000	chr8	126435000	2.58E-04	promoter	super_enhancer	KB-1517D11.3
chr8	99405000	chr8	126465000	2.15E-02	promoter	super_enhancer	KB-1517D11.3
chr8	99405000	chr8	127225000	1.23E-02	promoter	super_enhancer	KB-1517D11.3
chr8	99405000	chr8	133345000	1.67E-02	promoter	super_enhancer	KB-1517D11.3
chr8	99475000	chr8	101055000	3.30E-02	promoter	super_enhancer	RNU6-748P
chr8	99475000	chr8	101135000	2.25E-02	promoter	super_enhancer	RNU6-748P
chr8	99475000	chr8	101145000	2.90E-02	promoter	super_enhancer	RNU6-748P
chr8	99475000	chr8	126455000	1.87E-02	promoter	super_enhancer	RNU6-748P

chr8	99615000	chr8	102775000	4.38E-03	promoter	super_enhancer	KB-1205A7.2
chr8	99615000	chr8	126565000	1.58E-02	promoter	super_enhancer	KB-1205A7.2
chr8	99615000	chr8	133365000	1.37E-02	promoter	super_enhancer	KB-1205A7.2
chr8	99645000	chr8	101155000	2.19E-02	promoter	super_enhancer	KB-1205A7.1
chr8	99645000	chr8	101455000	7.12E-04	promoter	super_enhancer	KB-1205A7.1
chr8	99955000	chr8	101455000	3.79E-02	promoter	super_enhancer	STK3
chr8	100025000	chr8	101455000	4.06E-02	promoter	super_enhancer	RP11-410L14.2
chr8	100355000	chr8	101135000	1.46E-02	promoter	super_enhancer	AP004289.2
chr8	100355000	chr8	101145000	2.87E-03	promoter	super_enhancer	AP004289.2
chr8	100355000	chr8	143535000	7.92E-03	promoter	super_enhancer	AP004289.2
chr8	101005000	chr8	125185000	1.13E-02	super_enhancer	promoter	FER1L6-AS2
chr8	101005000	chr8	144365000	9.83E-03	super_enhancer	promoter	LINC01604
chr8	101025000	chr8	120885000	1.67E-02	super_enhancer	promoter	DEPTOR
chr8	101025000	chr8	142525000	7.14E-03	super_enhancer	promoter	AC138647.1
chr8	101025000	chr8	145635000	9.28E-03	super_enhancer	promoter	CPSF1
chr8	101035000	chr8	119635000	1.74E-02	super_enhancer	promoter	SAMD12
chr8	101035000	chr8	124135000	1.72E-02	super_enhancer	promoter	HMGB1P19
chr8	101055000	chr8	120875000	1.87E-02	super_enhancer	promoter	RN7SL396P
chr8	101055000	chr8	139075000	7.62E-03	super_enhancer	promoter	RP11-489O18.1
chr8	101135000	chr8	110985000	2.46E-02	super_enhancer	promoter	RP11-696P8.2
chr8	101145000	chr8	110375000	2.87E-02	super_enhancer	promoter	PKHD1L1
chr8	101145000	chr8	131905000	1.41E-02	super_enhancer	promoter	RP11-737F9.2
chr8	101155000	chr8	101695000	3.06E-02	super_enhancer	promoter	RNU6-1092P
chr8	101155000	chr8	120845000	1.87E-02	super_enhancer	promoter	TAF2

chr8	101155000	chr8	120875000	1.62E-04	super_enhancer	promoter	RN7SL396P
chr8	101155000	chr8	145925000	9.19E-03	super_enhancer	promoter	AF186192.1
chr8	101455000	chr8	124015000	1.40E-02	super_enhancer	promoter	RP11-557C18.3
chr8	101455000	chr8	142445000	9.93E-03	super_enhancer	promoter	CTD-3064M3.7
chr8	101465000	chr8	124205000	1.48E-02	super_enhancer	promoter	RP11-539E17.3
chr8	101465000	chr8	132915000	9.55E-03	super_enhancer	promoter	EFR3A
chr8	101465000	chr8	136245000	8.71E-03	super_enhancer	promoter	LINC01591
chr8	101465000	chr8	145755000	7.24E-03	super_enhancer	promoter	C8orf82
chr8	101505000	chr8	129995000	1.56E-02	promoter	super_enhancer	KB-1615E4.3
chr8	101505000	chr8	143575000	8.79E-03	promoter	super_enhancer	KB-1615E4.3
chr8	101565000	chr8	126545000	1.58E-02	promoter	super_enhancer	GAPDHP62
chr8	101695000	chr8	117985000	2.29E-02	promoter	super_enhancer	RNU6-1092P
chr8	101715000	chr8	102745000	4.64E-02	promoter	super_enhancer	MIR7705
chr8	101735000	chr8	140635000	9.37E-03	promoter	super_enhancer	PABPC1
chr8	101845000	chr8	125465000	1.78E-02	promoter	super_enhancer	RNU4-83P
chr8	102275000	chr8	117985000	2.04E-02	promoter	super_enhancer	KB-1410C5.2
chr8	102465000	chr8	126445000	1.77E-02	promoter	super_enhancer	KB-1930G5.4
chr8	102465000	chr8	143975000	8.98E-03	promoter	super_enhancer	KB-1930G5.4
chr8	102575000	chr8	133365000	1.59E-02	promoter	super_enhancer	KB-1562D12.3
chr8	102575000	chr8	133385000	1.33E-02	promoter	super_enhancer	KB-1562D12.3
chr8	102735000	chr8	143835000	9.74E-03	super_enhancer	promoter	LYPD2
chr8	102745000	chr8	108995000	1.61E-02	super_enhancer	promoter	NRBF2P4
chr8	102745000	chr8	135735000	9.92E-03	super_enhancer	promoter	CTD-2342N23.3
chr8	102745000	chr8	142365000	8.19E-03	super_enhancer	promoter	CTD-3064M3.3

chr8	102755000	chr8	110705000	3.35E-02	super_enhancer	promoter	SYBU
chr8	102755000	chr8	126965000	1.70E-02	super_enhancer	promoter	SOD1P3
chr8	102755000	chr8	132055000	1.79E-02	super_enhancer	promoter	ADCY8
chr8	102775000	chr8	110375000	3.50E-02	super_enhancer	promoter	PKHD1L1
chr8	102775000	chr8	119995000	1.71E-02	super_enhancer	promoter	RP11-278I4.1
chr8	102775000	chr8	134015000	1.31E-02	super_enhancer	promoter	CTD-2182N23.1
chr8	102775000	chr8	144655000	1.04E-02	super_enhancer	promoter	MROH6
chr8	102785000	chr8	108995000	2.35E-02	super_enhancer	promoter	NRBF2P4
chr8	102785000	chr8	124215000	2.34E-02	super_enhancer	promoter	FAM83A-AS1
chr8	102785000	chr8	127155000	1.33E-02	super_enhancer	promoter	AC090573.1
chr8	102795000	chr8	126965000	1.10E-02	super_enhancer	promoter	SOD1P3
chr8	102795000	chr8	135685000	6.07E-03	super_enhancer	promoter	CTD-2342N23.2
chr8	102805000	chr8	118175000	1.79E-02	super_enhancer	promoter	RN7SL826P
chr8	103425000	chr8	141235000	9.77E-03	promoter	super_enhancer	UBR5
chr8	103515000	chr8	141155000	7.94E-03	promoter	super_enhancer	RPS12P15
chr8	103635000	chr8	123175000	1.81E-02	promoter	super_enhancer	POU5F1P2
chr8	103635000	chr8	126435000	2.30E-02	promoter	super_enhancer	POU5F1P2
chr8	104035000	chr8	125405000	1.64E-02	promoter	super_enhancer	KB-1639H6.2
chr8	104155000	chr8	129995000	1.73E-02	promoter	super_enhancer	BAALC-AS2
chr8	104155000	chr8	130435000	1.09E-02	promoter	super_enhancer	BAALC-AS2
chr8	104155000	chr8	133405000	1.24E-02	promoter	super_enhancer	BAALC-AS2
chr8	104155000	chr8	140455000	1.06E-02	promoter	super_enhancer	BAALC-AS2
chr8	104495000	chr8	125405000	1.51E-02	promoter	super_enhancer	RP11-1C8.7
chr8	104495000	chr8	142685000	9.91E-03	promoter	super_enhancer	RP11-1C8.7

chr8	105435000	chr8	125455000	2.12E-02	promoter	super_enhancer	KB-1552D7.2
chr8	105995000	chr8	140585000	7.57E-03	promoter	super_enhancer	RP11-200A13.1
chr8	109145000	chr8	129995000	2.26E-02	promoter	super_enhancer	AP001331.1
chr8	109145000	chr8	133575000	1.96E-02	promoter	super_enhancer	AP001331.1
chr8	109145000	chr8	140635000	1.57E-02	promoter	super_enhancer	AP001331.1
chr8	109455000	chr8	125435000	1.30E-02	promoter	super_enhancer	EMC2
chr8	109585000	chr8	118005000	2.26E-02	promoter	super_enhancer	RP11-25P11.2
chr8	109585000	chr8	127735000	1.15E-02	promoter	super_enhancer	RP11-25P11.2
chr8	109585000	chr8	140575000	8.48E-03	promoter	super_enhancer	RP11-25P11.2
chr8	109665000	chr8	117985000	1.97E-02	promoter	super_enhancer	RP11-790J24.2
chr8	110375000	chr8	126565000	2.43E-02	promoter	super_enhancer	PKHD1L1
chr8	110375000	chr8	130425000	1.67E-02	promoter	super_enhancer	PKHD1L1
chr8	110375000	chr8	133405000	1.36E-02	promoter	super_enhancer	PKHD1L1
chr8	110655000	chr8	143575000	9.24E-03	promoter	super_enhancer	RP11-422N16.3
chr8	110705000	chr8	118005000	3.20E-02	promoter	super_enhancer	SYBU
chr8	110705000	chr8	126565000	2.25E-02	promoter	super_enhancer	SYBU
chr8	110705000	chr8	133395000	1.52E-02	promoter	super_enhancer	SYBU
chr8	110705000	chr8	143505000	1.16E-02	promoter	super_enhancer	SYBU
chr8	110985000	chr8	125425000	1.99E-02	promoter	super_enhancer	RP11-696P8.2
chr8	110985000	chr8	126555000	2.46E-02	promoter	super_enhancer	RP11-696P8.2
chr8	115805000	chr8	117985000	4.31E-03	promoter	super_enhancer	CARSP2
chr8	117035000	chr8	140645000	9.10E-03	promoter	super_enhancer	RNA5SP276
chr8	117985000	chr8	138865000	1.33E-02	super_enhancer	promoter	RP11-238K6.2
chr8	117985000	chr8	144295000	1.36E-02	super_enhancer	promoter	GPIHBP1

chr8	117995000	chr8	142335000	1.30E-02	super_enhancer	promoter	RP11-10J21.6
chr8	118005000	chr8	124015000	3.98E-02	super_enhancer	promoter	RP11-557C18.3
chr8	118005000	chr8	124055000	1.13E-03	super_enhancer	promoter	DERL1
chr8	118005000	chr8	127595000	4.21E-02	super_enhancer	promoter	RP11-89K10.2
chr8	118015000	chr8	124355000	4.44E-02	super_enhancer	promoter	RNU6-875P
chr8	118035000	chr8	134585000	1.81E-02	super_enhancer	promoter	ST3GAL1
chr8	118035000	chr8	142445000	1.33E-02	super_enhancer	promoter	CTD-3064M3.7
chr8	118175000	chr8	123165000	4.74E-02	promoter	super_enhancer	RN7SL826P
chr8	119125000	chr8	125465000	4.92E-02	promoter	super_enhancer	EXT1
chr8	119125000	chr8	126545000	3.89E-02	promoter	super_enhancer	EXT1
chr8	119125000	chr8	130445000	2.53E-02	promoter	super_enhancer	EXT1
chr8	119125000	chr8	143585000	1.95E-02	promoter	super_enhancer	EXT1
chr8	119635000	chr8	126455000	4.38E-02	promoter	super_enhancer	SAMD12
chr8	119635000	chr8	140575000	1.54E-02	promoter	super_enhancer	SAMD12
chr8	119775000	chr8	125425000	2.74E-02	promoter	super_enhancer	RPS26P35
chr8	119775000	chr8	125465000	3.61E-02	promoter	super_enhancer	RPS26P35
chr8	119965000	chr8	125445000	4.64E-02	promoter	super_enhancer	TNFRSF11B
chr8	119965000	chr8	141205000	1.49E-02	promoter	super_enhancer	TNFRSF11B
chr8	119995000	chr8	126555000	3.56E-02	promoter	super_enhancer	RP11-278I4.1
chr8	119995000	chr8	133385000	2.05E-02	promoter	super_enhancer	RP11-278I4.1
chr8	119995000	chr8	144425000	1.09E-02	promoter	super_enhancer	RP11-278I4.1
chr8	120875000	chr8	127855000	3.64E-02	promoter	super_enhancer	RN7SL396P
chr8	120875000	chr8	129995000	3.39E-02	promoter	super_enhancer	RN7SL396P
chr8	120875000	chr8	130435000	2.12E-02	promoter	super_enhancer	RN7SL396P

chr8	120875000	chr8	143505000	1.92E-02	promoter	super_enhancer	RN7SL396P
chr8	120885000	chr8	127225000	2.99E-02	promoter	super_enhancer	DEPTOR
chr8	122655000	chr8	123175000	1.28E-02	promoter	super_enhancer	HAS2
chr8	122655000	chr8	141215000	2.06E-02	promoter	super_enhancer	HAS2
chr8	123155000	chr8	124195000	1.71E-02	super_enhancer	promoter	RP11-539E17.5
chr8	123155000	chr8	140715000	1.40E-02	super_enhancer	promoter	KCNK9
chr8	123155000	chr8	143835000	1.65E-02	super_enhancer	promoter	LYPD2
chr8	123165000	chr8	138865000	1.75E-02	super_enhancer	promoter	RP11-238K6.2
chr8	123165000	chr8	140475000	1.52E-02	super_enhancer	promoter	RP11-398H6.1
chr8	123175000	chr8	132725000	2.10E-02	super_enhancer	promoter	CTD-2008O4.1
chr8	123185000	chr8	128495000	4.01E-02	super_enhancer	promoter	CASC8
chr8	123185000	chr8	132915000	2.44E-02	super_enhancer	promoter	EFR3A
chr8	123185000	chr8	145635000	1.76E-02	super_enhancer	promoter	CPSF1
chr8	124015000	chr8	143935000	1.31E-02	promoter	super_enhancer	RP11-557C18.3
chr8	124055000	chr8	133385000	3.42E-02	promoter	super_enhancer	DERL1
chr8	124135000	chr8	143515000	2.23E-02	promoter	super_enhancer	HMGB1P19
chr8	124195000	chr8	140455000	2.63E-02	promoter	super_enhancer	RP11-539E17.5
chr8	124195000	chr8	143495000	2.32E-02	promoter	super_enhancer	RP11-539E17.5
chr8	124195000	chr8	143525000	2.77E-02	promoter	super_enhancer	RP11-539E17.5
chr8	124205000	chr8	130435000	3.20E-02	promoter	super_enhancer	RP11-539E17.3
chr8	124205000	chr8	141225000	2.29E-02	promoter	super_enhancer	RP11-539E17.3
chr8	124215000	chr8	143575000	2.08E-02	promoter	super_enhancer	FAM83A-AS1
chr8	124255000	chr8	125465000	1.49E-02	promoter	super_enhancer	C8orf76
chr8	124275000	chr8	142685000	2.13E-02	promoter	super_enhancer	RNU6-628P

chr8	124355000	chr8	125455000	4.43E-03	promoter	super_enhancer	RNU6-875P
chr8	124355000	chr8	126545000	1.60E-02	promoter	super_enhancer	RNU6-875P
chr8	124355000	chr8	140595000	2.37E-02	promoter	super_enhancer	RNU6-875P
chr8	124355000	chr8	143565000	2.15E-02	promoter	super_enhancer	RNU6-875P
chr8	124625000	chr8	125465000	9.48E-03	promoter	super_enhancer	CTD-2552K11.2
chr8	124625000	chr8	130435000	3.26E-02	promoter	super_enhancer	CTD-2552K11.2
chr8	124625000	chr8	133365000	4.11E-02	promoter	super_enhancer	CTD-2552K11.2
chr8	124665000	chr8	125415000	2.58E-02	promoter	super_enhancer	KLHL38
chr8	124665000	chr8	133345000	4.67E-02	promoter	super_enhancer	KLHL38
chr8	124665000	chr8	133385000	4.12E-02	promoter	super_enhancer	KLHL38
chr8	124665000	chr8	140615000	3.51E-02	promoter	super_enhancer	KLHL38
chr8	124665000	chr8	141225000	2.66E-02	promoter	super_enhancer	KLHL38
chr8	124665000	chr8	143495000	2.22E-02	promoter	super_enhancer	KLHL38
chr8	124665000	chr8	143575000	2.19E-02	promoter	super_enhancer	KLHL38
chr8	125185000	chr8	125435000	2.02E-02	promoter	super_enhancer	FER1L6-AS2
chr8	125385000	chr8	133345000	4.42E-02	promoter	super_enhancer	TMEM65
chr8	125385000	chr8	141155000	2.11E-02	promoter	super_enhancer	TMEM65
chr8	125385000	chr8	143945000	2.45E-02	promoter	super_enhancer	TMEM65
chr8	125405000	chr8	143915000	1.19E-02	super_enhancer	promoter	GML
chr8	125415000	chr8	135845000	2.61E-02	super_enhancer	promoter	AC083843.2
chr8	125415000	chr8	144365000	3.00E-02	super_enhancer	promoter	LINC01604
chr8	125425000	chr8	134015000	2.75E-02	super_enhancer	promoter	CTD-2182N23.1
chr8	125435000	chr8	136755000	3.29E-02	super_enhancer	promoter	RNU1-35P
chr8	125455000	chr8	134015000	4.00E-02	super_enhancer	promoter	CTD-2182N23.1

chr8	125455000	chr8	135725000	3.66E-02	super_enhancer	promoter	ZFAT
chr8	125455000	chr8	144655000	2.59E-02	super_enhancer	promoter	MROH6
chr8	125465000	chr8	141645000	3.23E-02	super_enhancer	promoter	ERICD
chr8	125465000	chr8	145135000	2.70E-02	super_enhancer	promoter	CTD-3065J16.9
chr8	125835000	chr8	141155000	1.97E-02	promoter	super_enhancer	MIR4662B
chr8	126105000	chr8	127755000	3.38E-03	promoter	super_enhancer	KIAA0196
chr8	126365000	chr8	143585000	2.95E-02	promoter	super_enhancer	RP11-550A5.2
chr8	126435000	chr8	135725000	4.00E-02	super_enhancer	promoter	ZFAT
chr8	126435000	chr8	135735000	4.28E-02	super_enhancer	promoter	CTD-2342N23.3
chr8	126435000	chr8	145515000	3.07E-02	super_enhancer	promoter	BOP1
chr8	126445000	chr8	132445000	2.83E-02	super_enhancer	promoter	SNORA72
chr8	126445000	chr8	142265000	4.11E-04	super_enhancer	promoter	RP11-10J21.3
chr8	126445000	chr8	143845000	2.21E-02	super_enhancer	promoter	RP11-706C16.7
chr8	126465000	chr8	142265000	3.19E-02	super_enhancer	promoter	RP11-10J21.3
chr8	126465000	chr8	143485000	2.31E-02	super_enhancer	promoter	TSNARE1
chr8	126465000	chr8	144295000	2.50E-02	super_enhancer	promoter	GPIHBP1
chr8	126545000	chr8	135685000	2.23E-02	super_enhancer	promoter	CTD-2342N23.2
chr8	126545000	chr8	135725000	3.25E-02	super_enhancer	promoter	ZFAT
chr8	126555000	chr8	128095000	3.62E-02	super_enhancer	promoter	PCAT2
chr8	126555000	chr8	145135000	2.78E-02	super_enhancer	promoter	CTD-3065J16.9
chr8	126565000	chr8	141645000	3.48E-02	super_enhancer	promoter	ERICD
chr8	126565000	chr8	144295000	2.38E-02	super_enhancer	promoter	GPIHBP1
chr8	126965000	chr8	133355000	3.20E-02	promoter	super_enhancer	SOD1P3
chr8	126965000	chr8	143565000	2.13E-02	promoter	super_enhancer	SOD1P3

chr8	127085000	chr8	127735000	5.09E-03	promoter	super_enhancer	KNOP1P5
chr8	127085000	chr8	127795000	7.37E-04	promoter	super_enhancer	KNOP1P5
chr8	127085000	chr8	133345000	4.49E-02	promoter	super_enhancer	KNOP1P5
chr8	127085000	chr8	133375000	3.13E-02	promoter	super_enhancer	KNOP1P5
chr8	127085000	chr8	133385000	3.95E-02	promoter	super_enhancer	KNOP1P5
chr8	127155000	chr8	130435000	4.77E-02	promoter	super_enhancer	AC090573.1
chr8	127215000	chr8	134115000	2.30E-02	super_enhancer	promoter	SLA
chr8	127215000	chr8	134585000	2.56E-02	super_enhancer	promoter	ST3GAL1
chr8	127215000	chr8	140715000	1.35E-02	super_enhancer	promoter	KCNK9
chr8	127215000	chr8	143825000	9.91E-03	super_enhancer	promoter	SLURP1
chr8	127215000	chr8	143845000	1.29E-02	super_enhancer	promoter	RP11-706C16.7
chr8	127215000	chr8	145135000	1.59E-02	super_enhancer	promoter	CTD-3065J16.9
chr8	127225000	chr8	132445000	2.15E-02	super_enhancer	promoter	SNORA72
chr8	127225000	chr8	134585000	2.99E-02	super_enhancer	promoter	ST3GAL1
chr8	127485000	chr8	129995000	1.16E-02	promoter	super_enhancer	RP11-103H7.1
chr8	127485000	chr8	130435000	3.09E-03	promoter	super_enhancer	RP11-103H7.1
chr8	127485000	chr8	133355000	3.95E-02	promoter	super_enhancer	RP11-103H7.1
chr8	127485000	chr8	140585000	3.49E-04	promoter	super_enhancer	RP11-103H7.1
chr8	127595000	chr8	143585000	3.68E-02	promoter	super_enhancer	RP11-89K10.2
chr8	127595000	chr8	143945000	3.43E-02	promoter	super_enhancer	RP11-89K10.2
chr8	127745000	chr8	132915000	3.77E-02	super_enhancer	promoter	EFR3A
chr8	127745000	chr8	138865000	1.73E-02	super_enhancer	promoter	RP11-238K6.2
chr8	127745000	chr8	143485000	1.54E-02	super_enhancer	promoter	TSNARE1
chr8	127745000	chr8	144855000	1.78E-02	super_enhancer	promoter	RP11-429J17.7

chr8	127795000	chr8	138865000	2.08E-02	super_enhancer	promoter	RP11-238K6.2
chr8	127905000	chr8	128215000	9.89E-03	super_enhancer	promoter	CASC19
chr8	127905000	chr8	135735000	2.72E-02	super_enhancer	promoter	CTD-2342N23.3
chr8	128095000	chr8	143505000	2.23E-02	promoter	super_enhancer	PCAT2
chr8	128215000	chr8	133405000	2.94E-02	promoter	super_enhancer	CASC19
chr8	128495000	chr8	129975000	2.15E-02	promoter	super_enhancer	CASC8
chr8	129985000	chr8	143485000	1.78E-02	super_enhancer	promoter	TSNARE1
chr8	129995000	chr8	143485000	2.36E-02	super_enhancer	promoter	TSNARE1
chr8	130425000	chr8	136245000	4.11E-02	super_enhancer	promoter	LINC01591
chr8	130445000	chr8	139075000	2.18E-02	super_enhancer	promoter	RP11-489O18.1
chr8	130445000	chr8	143915000	1.42E-02	super_enhancer	promoter	GML
chr8	130445000	chr8	145755000	2.09E-02	super_enhancer	promoter	C8orf82
chr8	130845000	chr8	141235000	3.32E-02	promoter	super_enhancer	RP11-473O4.5
chr8	130845000	chr8	143505000	3.11E-02	promoter	super_enhancer	RP11-473O4.5
chr8	130845000	chr8	143535000	3.15E-02	promoter	super_enhancer	RP11-473O4.5
chr8	131905000	chr8	140465000	3.51E-02	promoter	super_enhancer	RP11-737F9.2
chr8	132445000	chr8	140635000	2.08E-02	promoter	super_enhancer	SNORA72
chr8	132915000	chr8	143495000	2.47E-02	promoter	super_enhancer	EFR3A
chr8	133345000	chr8	135735000	1.66E-02	super_enhancer	promoter	CTD-2342N23.3
chr8	133355000	chr8	140475000	2.30E-02	super_enhancer	promoter	RP11-398H6.1
chr8	133365000	chr8	142265000	4.57E-02	super_enhancer	promoter	RP11-10J21.3
chr8	133375000	chr8	138865000	3.23E-02	super_enhancer	promoter	RP11-238K6.2
chr8	133375000	chr8	139075000	2.96E-02	super_enhancer	promoter	RP11-489O18.1
chr8	133375000	chr8	140715000	2.65E-02	super_enhancer	promoter	KCNK9

chr8	133395000	chr8	139075000	3.74E-02	super_enhancer	promoter	RP11-489O18.1
chr8	134015000	chr8	141215000	4.41E-02	promoter	super_enhancer	CTD-2182N23.1
chr8	134015000	chr8	143525000	3.31E-02	promoter	super_enhancer	CTD-2182N23.1
chr8	134115000	chr8	140455000	3.68E-02	promoter	super_enhancer	SLA
chr8	134315000	chr8	143495000	2.66E-02	promoter	super_enhancer	NDRG1
chr8	134405000	chr8	140575000	3.81E-02	promoter	super_enhancer	CTC-369M3.1
chr8	134585000	chr8	140455000	4.69E-02	promoter	super_enhancer	ST3GAL1
chr8	135685000	chr8	141205000	3.03E-02	promoter	super_enhancer	CTD-2342N23.2
chr8	135725000	chr8	143935000	2.64E-02	promoter	super_enhancer	ZFAT
chr8	135845000	chr8	140575000	4.45E-02	promoter	super_enhancer	AC083843.2
chr8	136245000	chr8	140615000	2.93E-03	promoter	super_enhancer	LINC01591
chr8	136755000	chr8	143515000	4.43E-02	promoter	super_enhancer	RNU1-35P
chr8	138865000	chr8	140455000	1.90E-02	promoter	super_enhancer	RP11-238K6.2
chr8	140475000	chr8	140615000	7.33E-03	promoter	super_enhancer	RP11-398H6.1
chr8	140475000	chr8	141235000	2.46E-02	promoter	super_enhancer	RP11-398H6.1
chr8	140715000	chr8	141215000	8.73E-03	promoter	super_enhancer	KCNK9
chr8	142365000	chr8	143515000	2.70E-03	promoter	super_enhancer	CTD-3064M3.3
chr8	142365000	chr8	143525000	1.82E-02	promoter	super_enhancer	CTD-3064M3.3
chr8	142445000	chr8	143505000	4.47E-03	promoter	super_enhancer	CTD-3064M3.7
chr8	142445000	chr8	143515000	4.43E-03	promoter	super_enhancer	CTD-3064M3.7
chr8	142445000	chr8	143525000	2.61E-02	promoter	super_enhancer	CTD-3064M3.7
chr8	142525000	chr8	142685000	1.17E-02	promoter	super_enhancer	AC138647.1
chr8	142525000	chr8	143505000	1.71E-02	promoter	super_enhancer	AC138647.1
chr8	142525000	chr8	143515000	2.20E-03	promoter	super_enhancer	AC138647.1

chr8	142525000	chr8	143525000	1.56E-02	promoter	super_enhancer	AC138647.1
chr8	142525000	chr8	143565000	1.37E-03	promoter	super_enhancer	AC138647.1
chr8	143485000	chr8	143585000	4.55E-03	promoter	super_enhancer	TSNARE1
chr8	143975000	chr8	145925000	2.28E-02	super_enhancer	promoter	AF186192.1
chr9	2015000	chr9	33175000	5.90E-03	promoter	super_enhancer	SMARCA2
chr9	2845000	chr9	33125000	7.20E-03	promoter	super_enhancer	KIAA0020
chr9	3875000	chr9	33255000	5.79E-03	promoter	super_enhancer	RP11-252M18.3
chr9	3875000	chr9	128875000	2.05E-03	promoter	super_enhancer	RP11-252M18.3
chr9	4805000	chr9	127735000	2.24E-03	promoter	super_enhancer	KLF4P1
chr9	5305000	chr9	91205000	1.38E-03	promoter	super_enhancer	RLN2
chr9	5305000	chr9	127515000	1.34E-03	promoter	super_enhancer	RLN2
chr9	6195000	chr9	33815000	7.25E-03	promoter	super_enhancer	GTF3AP1
chr9	6195000	chr9	37925000	7.14E-03	promoter	super_enhancer	GTF3AP1
chr9	6645000	chr9	124795000	2.30E-03	promoter	super_enhancer	RP11-390F4.6
chr9	6715000	chr9	91185000	2.20E-03	promoter	super_enhancer	RP11-390F4.3
chr9	12765000	chr9	21555000	6.52E-03	promoter	super_enhancer	RN7SL849P
chr9	12775000	chr9	33255000	9.33E-03	promoter	super_enhancer	LURAP1L
chr9	12775000	chr9	129405000	2.60E-03	promoter	super_enhancer	LURAP1L
chr9	13445000	chr9	127595000	2.05E-03	promoter	super_enhancer	RP11-536O18.1
chr9	13985000	chr9	38025000	5.73E-03	promoter	super_enhancer	PES1P2
chr9	13985000	chr9	129315000	2.14E-03	promoter	super_enhancer	PES1P2
chr9	14025000	chr9	33145000	9.22E-03	promoter	super_enhancer	RP11-284P20.3
chr9	15525000	chr9	38045000	7.02E-03	promoter	super_enhancer	RP11-518K17.4
chr9	15525000	chr9	134595000	2.59E-03	promoter	super_enhancer	RP11-518K17.4

chr9	17135000	chr9	129395000	1.33E-03	promoter	super_enhancer	CNTLN
chr9	19065000	chr9	112045000	2.28E-03	promoter	super_enhancer	SCARNA8
chr9	19375000	chr9	111945000	1.81E-03	promoter	super_enhancer	RP11-513M16.8
chr9	19385000	chr9	33165000	1.45E-02	promoter	super_enhancer	NDUFA5P3
chr9	19465000	chr9	33825000	1.31E-02	promoter	super_enhancer	MAP1LC3BP1
chr9	19705000	chr9	33125000	1.04E-02	promoter	super_enhancer	C11orf98P1
chr9	19705000	chr9	112005000	1.89E-03	promoter	super_enhancer	C11orf98P1
chr9	20685000	chr9	133875000	1.87E-03	promoter	super_enhancer	FOCAD-AS1
chr9	20685000	chr9	133905000	2.25E-03	promoter	super_enhancer	FOCAD-AS1
chr9	21305000	chr9	134665000	2.89E-03	promoter	super_enhancer	IFNA5
chr9	21305000	chr9	136115000	2.87E-03	promoter	super_enhancer	IFNA5
chr9	21385000	chr9	33105000	1.18E-04	promoter	super_enhancer	IFNA2
chr9	21385000	chr9	111965000	2.02E-03	promoter	super_enhancer	IFNA2
chr9	21455000	chr9	128905000	2.18E-03	promoter	super_enhancer	IFNWP19
chr9	21555000	chr9	21995000	3.28E-02	super_enhancer	promoter	CDKN2A
chr9	21555000	chr9	117335000	1.99E-03	super_enhancer	promoter	RP11-402G3.6
chr9	21555000	chr9	135895000	1.91E-03	super_enhancer	promoter	EEF1A1P5
chr9	21555000	chr9	136225000	1.85E-03	super_enhancer	promoter	SURF1
chr9	21575000	chr9	33615000	8.28E-03	super_enhancer	promoter	PRSS3P4
chr9	21575000	chr9	35875000	6.95E-03	super_enhancer	promoter	NDUFA5P4
chr9	21585000	chr9	21995000	4.94E-02	super_enhancer	promoter	CDKN2A
chr9	21585000	chr9	32335000	1.16E-02	super_enhancer	promoter	SLC25A5P8
chr9	21585000	chr9	32385000	1.35E-02	super_enhancer	promoter	ACO1
chr9	21995000	chr9	33105000	1.41E-02	promoter	super_enhancer	CDKN2A

chr9	21995000	chr9	33445000	1.62E-02	promoter	super_enhancer	CDKN2A
chr9	21995000	chr9	35515000	1.02E-02	promoter	super_enhancer	CDKN2A
chr9	22215000	chr9	33235000	1.40E-02	promoter	super_enhancer	RP11-408N14.1
chr9	22215000	chr9	38005000	9.18E-03	promoter	super_enhancer	RP11-408N14.1
chr9	22645000	chr9	33175000	1.79E-02	promoter	super_enhancer	LINC01239
chr9	24545000	chr9	33145000	1.92E-02	promoter	super_enhancer	RP11-20A20.2
chr9	26895000	chr9	33155000	2.53E-02	promoter	super_enhancer	RN7SL100P
chr9	26895000	chr9	134655000	3.71E-03	promoter	super_enhancer	RN7SL100P
chr9	27575000	chr9	33105000	4.54E-02	promoter	super_enhancer	C9orf72
chr9	27575000	chr9	33155000	3.17E-02	promoter	super_enhancer	C9orf72
chr9	27575000	chr9	33825000	3.50E-02	promoter	super_enhancer	C9orf72
chr9	27575000	chr9	37945000	2.05E-02	promoter	super_enhancer	C9orf72
chr9	27575000	chr9	38005000	2.28E-02	promoter	super_enhancer	C9orf72
chr9	27945000	chr9	33155000	3.17E-02	promoter	super_enhancer	RP11-438B23.2
chr9	27945000	chr9	38045000	1.99E-02	promoter	super_enhancer	RP11-438B23.2
chr9	28865000	chr9	33235000	2.74E-02	promoter	super_enhancer	MIR876
chr9	28865000	chr9	94915000	1.78E-03	promoter	super_enhancer	MIR876
chr9	30935000	chr9	137295000	2.42E-03	promoter	super_enhancer	FTLP4
chr9	31505000	chr9	37955000	2.85E-02	promoter	super_enhancer	RP11-291J9.1
chr9	32335000	chr9	38005000	3.23E-02	promoter	super_enhancer	SLC25A5P8
chr9	32335000	chr9	134645000	3.35E-03	promoter	super_enhancer	SLC25A5P8
chr9	32385000	chr9	37965000	2.99E-02	promoter	super_enhancer	ACO1
chr9	32385000	chr9	135135000	4.48E-03	promoter	super_enhancer	ACO1
chr9	32645000	chr9	37955000	4.78E-02	promoter	super_enhancer	RP11-555J4.3

chr9	32645000	chr9	94925000	4.62E-03	promoter	super_enhancer	RP11-555J4.3
chr9	32645000	chr9	129505000	4.37E-03	promoter	super_enhancer	RP11-555J4.3
chr9	32645000	chr9	130925000	4.52E-03	promoter	super_enhancer	RP11-555J4.3
chr9	32945000	chr9	37935000	3.44E-02	promoter	super_enhancer	ASS1P12
chr9	33025000	chr9	107745000	1.92E-03	promoter	super_enhancer	APTX
chr9	33025000	chr9	111855000	3.67E-03	promoter	super_enhancer	APTX
chr9	33025000	chr9	129725000	4.99E-03	promoter	super_enhancer	APTX
chr9	33115000	chr9	35605000	9.94E-03	super_enhancer	promoter	TESK1
chr9	33115000	chr9	123575000	4.70E-03	super_enhancer	promoter	RP11-27I1.6
chr9	33115000	chr9	136185000	4.84E-03	super_enhancer	promoter	LCN1P2
chr9	33125000	chr9	33265000	1.92E-02	super_enhancer	promoter	BAG1
chr9	33125000	chr9	109355000	3.46E-03	super_enhancer	promoter	RP11-540H22.2
chr9	33125000	chr9	111695000	3.39E-03	super_enhancer	promoter	FAM206A
chr9	33135000	chr9	140145000	3.68E-03	super_enhancer	promoter	C9orf173
chr9	33145000	chr9	109625000	2.69E-03	super_enhancer	promoter	ZNF462
chr9	33155000	chr9	35565000	4.62E-03	super_enhancer	promoter	FAM166B
chr9	33155000	chr9	72805000	4.68E-03	super_enhancer	promoter	RNU2-5P
chr9	33155000	chr9	95055000	3.68E-03	super_enhancer	promoter	MIR3651
chr9	33155000	chr9	109485000	2.38E-03	super_enhancer	promoter	RP11-308N19.5
chr9	33165000	chr9	140145000	4.23E-03	super_enhancer	promoter	C9orf173
chr9	33175000	chr9	38545000	4.16E-02	super_enhancer	promoter	FAM95C
chr9	33175000	chr9	79395000	6.57E-03	super_enhancer	promoter	PCA3_1
chr9	33175000	chr9	92805000	5.60E-03	super_enhancer	promoter	RP11-406A20.4
chr9	33175000	chr9	131645000	4.84E-03	super_enhancer	promoter	CCBL1

chr9	33175000	chr9	132565000	4.47E-03	super_enhancer	promoter	TOR1B
chr9	33235000	chr9	33475000	4.05E-02	super_enhancer	promoter	NOL6
chr9	33245000	chr9	100745000	4.15E-03	super_enhancer	promoter	ANP32B
chr9	33245000	chr9	107485000	2.14E-03	super_enhancer	promoter	OR13D3P
chr9	33245000	chr9	115985000	3.27E-03	super_enhancer	promoter	FKBP15
chr9	33245000	chr9	139555000	5.10E-03	super_enhancer	promoter	RP11-251M1.1
chr9	33255000	chr9	33935000	1.70E-03	super_enhancer	promoter	SNORD121B
chr9	33255000	chr9	139695000	5.82E-03	super_enhancer	promoter	RP11-216L13.18
chr9	33265000	chr9	37995000	3.81E-02	promoter	super_enhancer	BAG1
chr9	33265000	chr9	130135000	3.59E-03	promoter	super_enhancer	BAG1
chr9	33445000	chr9	35645000	2.32E-02	super_enhancer	promoter	RP11-331F9.4
chr9	33445000	chr9	66495000	7.67E-03	super_enhancer	promoter	RP11-262H14.5
chr9	33445000	chr9	98275000	5.84E-03	super_enhancer	promoter	RP11-435O5.4
chr9	33445000	chr9	132095000	5.96E-03	super_enhancer	promoter	LINC01503
chr9	33445000	chr9	135755000	7.09E-03	super_enhancer	promoter	AK8
chr9	33475000	chr9	72155000	8.54E-03	promoter	super_enhancer	NOL6
chr9	33475000	chr9	110745000	3.50E-03	promoter	super_enhancer	NOL6
chr9	33475000	chr9	111805000	3.98E-03	promoter	super_enhancer	NOL6
chr9	33475000	chr9	133875000	5.48E-03	promoter	super_enhancer	NOL6
chr9	33475000	chr9	134635000	5.55E-03	promoter	super_enhancer	NOL6
chr9	33515000	chr9	37915000	3.83E-02	promoter	super_enhancer	RP11-255A11.2
chr9	33515000	chr9	37945000	3.96E-02	promoter	super_enhancer	RP11-255A11.2
chr9	33615000	chr9	111815000	2.93E-03	promoter	super_enhancer	PRSS3P4
chr9	33615000	chr9	127535000	4.32E-03	promoter	super_enhancer	PRSS3P4

chr9	33695000	chr9	136085000	3.77E-03	promoter	super_enhancer	TRBV26OR9-2
chr9	33785000	chr9	37955000	4.89E-02	promoter	super_enhancer	TRBV29OR9-2
chr9	33785000	chr9	134675000	3.09E-03	promoter	super_enhancer	TRBV29OR9-2
chr9	33805000	chr9	110475000	3.88E-03	super_enhancer	promoter	AL389915.1
chr9	33805000	chr9	125695000	4.33E-03	super_enhancer	promoter	ZBTB26
chr9	33805000	chr9	139725000	4.35E-03	super_enhancer	promoter	MIR4292
chr9	33815000	chr9	95645000	5.29E-03	super_enhancer	promoter	RP11-526D8.7
chr9	33815000	chr9	114335000	3.41E-03	super_enhancer	promoter	RP11-16L21.7
chr9	33815000	chr9	117085000	3.19E-03	super_enhancer	promoter	ORM1
chr9	33815000	chr9	117445000	3.06E-03	super_enhancer	promoter	RP11-402G3.3
chr9	33815000	chr9	127615000	4.96E-03	super_enhancer	promoter	WDR38
chr9	33825000	chr9	128135000	4.49E-03	super_enhancer	promoter	RP11-184B22.2
chr9	33835000	chr9	78505000	7.71E-03	super_enhancer	promoter	PCSK5
chr9	33835000	chr9	95245000	3.71E-03	super_enhancer	promoter	ASPN
chr9	33835000	chr9	132215000	3.05E-03	super_enhancer	promoter	RP11-65J3.3
chr9	33935000	chr9	111855000	3.33E-03	promoter	super_enhancer	SNORD121B
chr9	33935000	chr9	127535000	4.56E-03	promoter	super_enhancer	SNORD121B
chr9	33935000	chr9	137275000	4.34E-03	promoter	super_enhancer	SNORD121B
chr9	34135000	chr9	124875000	4.05E-03	promoter	super_enhancer	RP11-537H15.4
chr9	34135000	chr9	127545000	4.84E-03	promoter	super_enhancer	RP11-537H15.4
chr9	34135000	chr9	127575000	5.09E-03	promoter	super_enhancer	RP11-537H15.4
chr9	34225000	chr9	111965000	3.83E-03	promoter	super_enhancer	RP11-571F15.3
chr9	34295000	chr9	112005000	4.04E-03	promoter	super_enhancer	AL353662.1
chr9	34295000	chr9	127545000	4.48E-03	promoter	super_enhancer	AL353662.1

chr9	34295000	chr9	129455000	4.41E-03	promoter	super_enhancer	AL353662.1
chr9	34665000	chr9	35905000	1.41E-02	promoter	super_enhancer	CCL27
chr9	34665000	chr9	91065000	4.03E-03	promoter	super_enhancer	CCL27
chr9	34665000	chr9	107735000	2.34E-03	promoter	super_enhancer	CCL27
chr9	35045000	chr9	112015000	4.32E-03	promoter	super_enhancer	RP11-182N22.9
chr9	35095000	chr9	38045000	4.74E-03	promoter	super_enhancer	RP11-182N22.8
chr9	35095000	chr9	72135000	8.28E-03	promoter	super_enhancer	RP11-182N22.8
chr9	35095000	chr9	127175000	4.10E-03	promoter	super_enhancer	RP11-182N22.8
chr9	35095000	chr9	134615000	5.71E-03	promoter	super_enhancer	RP11-182N22.8
chr9	35515000	chr9	127615000	4.62E-03	super_enhancer	promoter	WDR38
chr9	35515000	chr9	140355000	3.79E-03	super_enhancer	promoter	NSMF
chr9	35525000	chr9	88715000	4.00E-03	super_enhancer	promoter	GOLM1
chr9	35525000	chr9	100745000	2.84E-03	super_enhancer	promoter	ANP32B
chr9	35645000	chr9	111805000	4.07E-03	promoter	super_enhancer	RP11-331F9.4
chr9	35645000	chr9	128095000	5.04E-03	promoter	super_enhancer	RP11-331F9.4
chr9	35645000	chr9	130915000	6.22E-03	promoter	super_enhancer	RP11-331F9.4
chr9	35645000	chr9	133905000	6.53E-03	promoter	super_enhancer	RP11-331F9.4
chr9	35815000	chr9	35905000	7.76E-03	promoter	super_enhancer	HINT2
chr9	35815000	chr9	124795000	4.70E-03	promoter	super_enhancer	HINT2
chr9	35875000	chr9	94925000	4.22E-03	promoter	super_enhancer	NDUFA5P4
chr9	35875000	chr9	124865000	3.72E-03	promoter	super_enhancer	NDUFA5P4
chr9	35905000	chr9	36035000	2.01E-02	super_enhancer	promoter	RECK
chr9	35905000	chr9	131395000	6.04E-03	super_enhancer	promoter	RP11-216B9.9
chr9	35905000	chr9	135905000	3.50E-03	super_enhancer	promoter	GTF3C5

chr9	35945000	chr9	38035000	1.76E-02	promoter	super_enhancer	PGAM1P2
chr9	35945000	chr9	91065000	3.83E-03	promoter	super_enhancer	PGAM1P2
chr9	35945000	chr9	129315000	5.61E-03	promoter	super_enhancer	PGAM1P2
chr9	35945000	chr9	135145000	3.70E-03	promoter	super_enhancer	PGAM1P2
chr9	36005000	chr9	37945000	1.10E-02	promoter	super_enhancer	OR13C7
chr9	36005000	chr9	94945000	5.64E-03	promoter	super_enhancer	OR13C7
chr9	36005000	chr9	129755000	3.05E-03	promoter	super_enhancer	OR13C7
chr9	36035000	chr9	111735000	2.88E-03	promoter	super_enhancer	RECK
chr9	36305000	chr9	128875000	4.62E-03	promoter	super_enhancer	HMGB3P24
chr9	36305000	chr9	133895000	4.16E-03	promoter	super_enhancer	HMGB3P24
chr9	36825000	chr9	72165000	1.08E-02	promoter	super_enhancer	MIR4475
chr9	36825000	chr9	111945000	5.55E-03	promoter	super_enhancer	MIR4475
chr9	36825000	chr9	128685000	3.65E-03	promoter	super_enhancer	MIR4475
chr9	36865000	chr9	37915000	8.17E-03	promoter	super_enhancer	MIR4540
chr9	36865000	chr9	111815000	3.80E-03	promoter	super_enhancer	MIR4540
chr9	36865000	chr9	134375000	4.18E-03	promoter	super_enhancer	MIR4540
chr9	36895000	chr9	133865000	6.38E-03	promoter	super_enhancer	MIR4476
chr9	37035000	chr9	134635000	5.30E-03	promoter	super_enhancer	PAX5
chr9	37035000	chr9	137415000	4.40E-03	promoter	super_enhancer	PAX5
chr9	37115000	chr9	110745000	3.00E-03	promoter	super_enhancer	RP11-220I1.2
chr9	37115000	chr9	111805000	3.40E-03	promoter	super_enhancer	RP11-220I1.2
chr9	37205000	chr9	73135000	7.74E-03	promoter	super_enhancer	RP11-220I1.4
chr9	37205000	chr9	127535000	4.66E-03	promoter	super_enhancer	RP11-220I1.4
chr9	37335000	chr9	137315000	3.23E-03	promoter	super_enhancer	RNU6-677P

chr9	37465000	chr9	38025000	2.75E-03	promoter	super_enhancer	ZBTB5
chr9	37465000	chr9	38055000	4.53E-02	promoter	super_enhancer	ZBTB5
chr9	37465000	chr9	130125000	3.98E-03	promoter	super_enhancer	ZBTB5
chr9	37485000	chr9	37925000	9.78E-03	promoter	super_enhancer	POLR1E
chr9	37485000	chr9	37935000	9.34E-03	promoter	super_enhancer	POLR1E
chr9	37485000	chr9	111845000	2.75E-03	promoter	super_enhancer	POLR1E
chr9	37485000	chr9	124305000	5.05E-03	promoter	super_enhancer	POLR1E
chr9	37485000	chr9	127965000	4.28E-03	promoter	super_enhancer	POLR1E
chr9	37835000	chr9	129505000	3.82E-03	promoter	super_enhancer	RP11-3J10.7
chr9	37885000	chr9	129485000	3.56E-03	promoter	super_enhancer	TMX2P1
chr9	37885000	chr9	129735000	3.57E-03	promoter	super_enhancer	TMX2P1
chr9	37915000	chr9	101475000	2.63E-03	super_enhancer	promoter	AL353782.1
chr9	37925000	chr9	95675000	7.63E-03	super_enhancer	promoter	ALOX15P2
chr9	37925000	chr9	99915000	4.78E-03	super_enhancer	promoter	RP11-520B13.8
chr9	37925000	chr9	125165000	5.17E-03	super_enhancer	promoter	RP11-542K23.10
chr9	37925000	chr9	134955000	4.52E-03	super_enhancer	promoter	MED27
chr9	37935000	chr9	79655000	6.18E-03	super_enhancer	promoter	ATP5J2P3
chr9	37935000	chr9	91795000	3.61E-03	super_enhancer	promoter	SHC3
chr9	37955000	chr9	38545000	2.18E-02	super_enhancer	promoter	FAM95C
chr9	37955000	chr9	73425000	5.93E-03	super_enhancer	promoter	MIR204
chr9	37955000	chr9	82005000	5.59E-03	super_enhancer	promoter	CHCHD2P9
chr9	37955000	chr9	88715000	6.65E-03	super_enhancer	promoter	GOLM1
chr9	37955000	chr9	95245000	4.41E-03	super_enhancer	promoter	ASPN
chr9	37955000	chr9	98535000	5.79E-03	super_enhancer	promoter	RP11-180I4.4

chr9	37955000	chr9	132215000	3.30E-03	super_enhancer	promoter	RP11-65J3.3
chr9	37965000	chr9	69825000	7.60E-03	super_enhancer	promoter	BNIP3P4
chr9	37965000	chr9	132935000	3.04E-03	super_enhancer	promoter	NCS1
chr9	37965000	chr9	134955000	3.01E-03	super_enhancer	promoter	MED27
chr9	37975000	chr9	95645000	5.92E-03	super_enhancer	promoter	RP11-526D8.7
chr9	37985000	chr9	71155000	7.20E-03	super_enhancer	promoter	RP11-274B18.4
chr9	37985000	chr9	90355000	3.79E-03	super_enhancer	promoter	RP11-65B23.3
chr9	37995000	chr9	38425000	4.74E-02	super_enhancer	promoter	IGFBPL1
chr9	37995000	chr9	73195000	7.81E-03	super_enhancer	promoter	RN7SL726P
chr9	37995000	chr9	99065000	4.87E-03	super_enhancer	promoter	HSD17B3
chr9	37995000	chr9	128275000	4.40E-03	super_enhancer	promoter	RN7SL30P
chr9	38005000	chr9	100395000	3.77E-03	super_enhancer	promoter	NCBP1
chr9	38005000	chr9	115045000	2.85E-03	super_enhancer	promoter	HSPE1P28
chr9	38015000	chr9	38145000	2.56E-02	super_enhancer	promoter	AL161448.1
chr9	38015000	chr9	72875000	7.48E-03	super_enhancer	promoter	SMC5-AS1
chr9	38015000	chr9	86585000	2.99E-03	super_enhancer	promoter	MIR7-1
chr9	38015000	chr9	91795000	4.06E-03	super_enhancer	promoter	SHC3
chr9	38015000	chr9	100615000	5.22E-03	super_enhancer	promoter	FOXE1
chr9	38015000	chr9	118915000	2.77E-03	super_enhancer	promoter	PAPPA
chr9	38025000	chr9	82005000	4.93E-03	super_enhancer	promoter	CHCHD2P9
chr9	38025000	chr9	115125000	3.69E-03	super_enhancer	promoter	RP11-432F4.2
chr9	38025000	chr9	133275000	5.02E-03	super_enhancer	promoter	RN7SL665P
chr9	38025000	chr9	136325000	3.53E-03	super_enhancer	promoter	CACFD1
chr9	38035000	chr9	116165000	4.57E-03	super_enhancer	promoter	ALAD

chr9	38045000	chr9	66495000	5.90E-03	super_enhancer	promoter	RP11-262H14.5
chr9	38045000	chr9	71735000	7.53E-03	super_enhancer	promoter	TJP2
chr9	38045000	chr9	115125000	3.28E-03	super_enhancer	promoter	RP11-432F4.2
chr9	38045000	chr9	127455000	3.31E-03	super_enhancer	promoter	MIR181A2
chr9	38055000	chr9	67335000	4.85E-03	super_enhancer	promoter	BMS1P10
chr9	38055000	chr9	109355000	3.27E-03	super_enhancer	promoter	RP11-540H22.2
chr9	38065000	chr9	45375000	1.59E-02	super_enhancer	promoter	MYO5BP1
chr9	38065000	chr9	67285000	6.00E-03	super_enhancer	promoter	RP11-236F9.2
chr9	38065000	chr9	72375000	8.41E-03	super_enhancer	promoter	PTAR1
chr9	38065000	chr9	132515000	5.10E-03	super_enhancer	promoter	PTGES
chr9	38145000	chr9	111745000	3.39E-03	promoter	super_enhancer	AL161448.1
chr9	38545000	chr9	127965000	4.55E-03	promoter	super_enhancer	FAM95C
chr9	38545000	chr9	129495000	4.00E-03	promoter	super_enhancer	FAM95C
chr9	38545000	chr9	130925000	4.91E-03	promoter	super_enhancer	FAM95C
chr9	45375000	chr9	124805000	1.27E-03	promoter	super_enhancer	MYO5BP1
chr9	66495000	chr9	111745000	3.34E-03	promoter	super_enhancer	RP11-262H14.5
chr9	66495000	chr9	112035000	3.69E-03	promoter	super_enhancer	RP11-262H14.5
chr9	66495000	chr9	129775000	3.14E-03	promoter	super_enhancer	RP11-262H14.5
chr9	67285000	chr9	134645000	2.32E-03	promoter	super_enhancer	RP11-236F9.2
chr9	67335000	chr9	111805000	3.24E-03	promoter	super_enhancer	BMS1P10
chr9	68325000	chr9	120875000	1.89E-03	promoter	super_enhancer	RP11-149F8.5
chr9	68325000	chr9	135115000	1.87E-03	promoter	super_enhancer	RP11-149F8.5
chr9	69825000	chr9	94915000	1.16E-02	promoter	super_enhancer	BNIP3P4
chr9	71155000	chr9	94935000	9.66E-03	promoter	super_enhancer	RP11-274B18.4

chr9	71155000	chr9	124405000	5.69E-03	promoter	super_enhancer	RP11-274B18.4
chr9	71395000	chr9	94925000	1.17E-02	promoter	super_enhancer	FAM122A
chr9	71395000	chr9	127525000	5.25E-03	promoter	super_enhancer	FAM122A
chr9	71395000	chr9	127735000	4.62E-03	promoter	super_enhancer	FAM122A
chr9	71405000	chr9	111985000	6.93E-03	promoter	super_enhancer	RP11-203L2.3
chr9	71405000	chr9	113605000	5.94E-03	promoter	super_enhancer	RP11-203L2.3
chr9	71735000	chr9	112045000	7.87E-03	promoter	super_enhancer	TJP2
chr9	71735000	chr9	127495000	5.65E-03	promoter	super_enhancer	TJP2
chr9	71735000	chr9	137305000	4.37E-03	promoter	super_enhancer	TJP2
chr9	72045000	chr9	72165000	5.46E-03	promoter	super_enhancer	RP11-548B3.3
chr9	72045000	chr9	137305000	4.69E-03	promoter	super_enhancer	RP11-548B3.3
chr9	72135000	chr9	73195000	3.52E-02	super_enhancer	promoter	RN7SL726P
chr9	72135000	chr9	79635000	2.13E-02	super_enhancer	promoter	FOXB2
chr9	72135000	chr9	93515000	1.01E-02	super_enhancer	promoter	OR7E108P
chr9	72145000	chr9	78195000	2.10E-02	super_enhancer	promoter	RP11-470F18.1
chr9	72145000	chr9	79585000	1.92E-02	super_enhancer	promoter	LYPLA2P3
chr9	72145000	chr9	98055000	7.81E-03	super_enhancer	promoter	RP11-173G21.1
chr9	72145000	chr9	99065000	8.23E-03	super_enhancer	promoter	HSD17B3
chr9	72145000	chr9	126165000	4.43E-03	super_enhancer	promoter	MIR601
chr9	72155000	chr9	79635000	2.32E-02	super_enhancer	promoter	FOXB2
chr9	72155000	chr9	125165000	6.28E-03	super_enhancer	promoter	RP11-542K23.10
chr9	72155000	chr9	136185000	4.90E-03	super_enhancer	promoter	LCN1P2
chr9	72165000	chr9	101475000	6.73E-03	super_enhancer	promoter	AL353782.1
chr9	72165000	chr9	134955000	4.58E-03	super_enhancer	promoter	MED27

chr9	72435000	chr9	111935000	5.62E-03	promoter	super_enhancer	C9orf135
chr9	72805000	chr9	111805000	5.45E-03	promoter	super_enhancer	RNU2-5P
chr9	72875000	chr9	73125000	3.40E-02	promoter	super_enhancer	SMC5-AS1
chr9	72875000	chr9	111955000	9.12E-03	promoter	super_enhancer	SMC5-AS1
chr9	72925000	chr9	73145000	1.57E-02	promoter	super_enhancer	RP11-386J22.3
chr9	72925000	chr9	111865000	6.29E-03	promoter	super_enhancer	RP11-386J22.3
chr9	72925000	chr9	124305000	7.61E-03	promoter	super_enhancer	RP11-386J22.3
chr9	72925000	chr9	136905000	4.18E-03	promoter	super_enhancer	RP11-386J22.3
chr9	73115000	chr9	77415000	3.77E-02	super_enhancer	promoter	RN7SKP47
chr9	73115000	chr9	79585000	3.32E-02	super_enhancer	promoter	LYPLA2P3
chr9	73115000	chr9	97905000	1.50E-02	super_enhancer	promoter	RNA5SP288
chr9	73115000	chr9	99915000	1.04E-02	super_enhancer	promoter	RP11-520B13.8
chr9	73115000	chr9	109485000	7.00E-03	super_enhancer	promoter	RP11-308N19.5
chr9	73115000	chr9	116615000	7.11E-03	super_enhancer	promoter	RP11-534I8.1
chr9	73115000	chr9	121525000	5.57E-03	super_enhancer	promoter	TUBB4BP6
chr9	73125000	chr9	74205000	4.09E-02	super_enhancer	promoter	RPL35AP21
chr9	73125000	chr9	82005000	1.32E-02	super_enhancer	promoter	CHCHD2P9
chr9	73125000	chr9	114175000	6.95E-03	super_enhancer	promoter	RNA5SP294
chr9	73135000	chr9	97905000	1.35E-02	super_enhancer	promoter	RNA5SP288
chr9	73145000	chr9	93345000	1.36E-02	super_enhancer	promoter	LINC01501
chr9	73145000	chr9	110575000	7.94E-03	super_enhancer	promoter	RN7SL659P
chr9	73145000	chr9	123555000	6.24E-03	super_enhancer	promoter	RP11-27I1.4
chr9	73145000	chr9	127615000	6.98E-03	super_enhancer	promoter	WDR38
chr9	73145000	chr9	128135000	6.28E-03	super_enhancer	promoter	RP11-184B22.2

chr9	73155000	chr9	112275000	7.64E-03	super_enhancer	promoter	MIR3927
chr9	74205000	chr9	91065000	1.13E-02	promoter	super_enhancer	RPL35AP21
chr9	74205000	chr9	113625000	7.22E-03	promoter	super_enhancer	RPL35AP21
chr9	74205000	chr9	115675000	7.82E-03	promoter	super_enhancer	RPL35AP21
chr9	74205000	chr9	127535000	7.97E-03	promoter	super_enhancer	RPL35AP21
chr9	74205000	chr9	136685000	6.56E-03	promoter	super_enhancer	RPL35AP21
chr9	74525000	chr9	111955000	9.66E-03	promoter	super_enhancer	C9orf85
chr9	74525000	chr9	112065000	8.26E-03	promoter	super_enhancer	C9orf85
chr9	74525000	chr9	129315000	7.82E-03	promoter	super_enhancer	C9orf85
chr9	74525000	chr9	135115000	4.43E-03	promoter	super_enhancer	C9orf85
chr9	75085000	chr9	111955000	8.78E-03	promoter	super_enhancer	RP11-313E4.1
chr9	75085000	chr9	127555000	7.90E-03	promoter	super_enhancer	RP11-313E4.1
chr9	75695000	chr9	129385000	5.37E-03	promoter	super_enhancer	ALDH1A1
chr9	76985000	chr9	94915000	1.25E-02	promoter	super_enhancer	RP11-171A24.2
chr9	77415000	chr9	94905000	1.27E-02	promoter	super_enhancer	RN7SKP47
chr9	78195000	chr9	127195000	7.70E-03	promoter	super_enhancer	RP11-470F18.1
chr9	78505000	chr9	121285000	5.63E-03	promoter	super_enhancer	PCSK5
chr9	78505000	chr9	124795000	7.49E-03	promoter	super_enhancer	PCSK5
chr9	78505000	chr9	134595000	7.35E-03	promoter	super_enhancer	PCSK5
chr9	79035000	chr9	130925000	5.65E-03	promoter	super_enhancer	GCNT1
chr9	79165000	chr9	124795000	2.52E-05	promoter	super_enhancer	RP11-214N16.2
chr9	79395000	chr9	91065000	1.06E-04	promoter	super_enhancer	PCA3_1
chr9	79585000	chr9	127545000	8.19E-03	promoter	super_enhancer	LYPLA2P3
chr9	79585000	chr9	136675000	6.45E-03	promoter	super_enhancer	LYPLA2P3

chr9	79585000	chr9	136685000	7.30E-03	promoter	super_enhancer	LYPLA2P3
chr9	79655000	chr9	127565000	8.53E-03	promoter	super_enhancer	ATP5J2P3
chr9	80195000	chr9	107735000	6.36E-03	promoter	super_enhancer	NUTF2P3
chr9	80195000	chr9	129425000	8.06E-03	promoter	super_enhancer	NUTF2P3
chr9	82005000	chr9	129475000	5.43E-03	promoter	super_enhancer	CHCHD2P9
chr9	82465000	chr9	129505000	5.27E-03	promoter	super_enhancer	RP11-403N16.5
chr9	82975000	chr9	136115000	6.62E-03	promoter	super_enhancer	NPAP1P4
chr9	83625000	chr9	91185000	1.80E-02	promoter	super_enhancer	AL354682.1
chr9	83625000	chr9	128875000	5.82E-03	promoter	super_enhancer	AL354682.1
chr9	84705000	chr9	111995000	1.06E-02	promoter	super_enhancer	DDX10P2
chr9	86445000	chr9	94925000	1.57E-02	promoter	super_enhancer	GKAP1
chr9	86445000	chr9	128085000	5.35E-03	promoter	super_enhancer	GKAP1
chr9	86595000	chr9	130915000	8.57E-03	promoter	super_enhancer	RMI1
chr9	87775000	chr9	91195000	4.57E-02	promoter	super_enhancer	UBE2V1P10
chr9	87775000	chr9	129735000	6.12E-03	promoter	super_enhancer	UBE2V1P10
chr9	88535000	chr9	98275000	2.44E-02	super_enhancer	promoter	RP11-435O5.4
chr9	88535000	chr9	109885000	1.15E-02	super_enhancer	promoter	RP11-196I18.4
chr9	88555000	chr9	94905000	3.65E-02	promoter	super_enhancer	NAA35
chr9	88555000	chr9	94945000	3.90E-02	promoter	super_enhancer	NAA35
chr9	88555000	chr9	113605000	1.21E-02	promoter	super_enhancer	NAA35
chr9	88555000	chr9	124875000	9.75E-03	promoter	super_enhancer	NAA35
chr9	88555000	chr9	133865000	1.13E-02	promoter	super_enhancer	NAA35
chr9	88555000	chr9	136685000	9.40E-03	promoter	super_enhancer	NAA35
chr9	88715000	chr9	129385000	8.05E-03	promoter	super_enhancer	GOLM1

chr9	88715000	chr9	129735000	7.90E-03	promoter	super_enhancer	GOLM1
chr9	88715000	chr9	133905000	1.02E-02	promoter	super_enhancer	GOLM1
chr9	88875000	chr9	94945000	3.55E-02	promoter	super_enhancer	C9orf153
chr9	88875000	chr9	107735000	7.54E-03	promoter	super_enhancer	C9orf153
chr9	88875000	chr9	112025000	1.22E-02	promoter	super_enhancer	C9orf153
chr9	88875000	chr9	124395000	7.07E-03	promoter	super_enhancer	C9orf153
chr9	88875000	chr9	129145000	9.31E-03	promoter	super_enhancer	C9orf153
chr9	88875000	chr9	129505000	8.97E-03	promoter	super_enhancer	C9orf153
chr9	90355000	chr9	130195000	6.54E-03	promoter	super_enhancer	RP11-65B23.3
chr9	90475000	chr9	111845000	1.03E-02	promoter	super_enhancer	XXyac-YM21GA2.7
chr9	90475000	chr9	112055000	1.23E-02	promoter	super_enhancer	XXyac-YM21GA2.7
chr9	90475000	chr9	121285000	7.58E-03	promoter	super_enhancer	XXyac-YM21GA2.7
chr9	90475000	chr9	127735000	8.31E-03	promoter	super_enhancer	XXyac-YM21GA2.7
chr9	90475000	chr9	128075000	1.02E-02	promoter	super_enhancer	XXyac-YM21GA2.7
chr9	90475000	chr9	128865000	1.05E-02	promoter	super_enhancer	XXyac-YM21GA2.7
chr9	90805000	chr9	91065000	4.50E-02	promoter	super_enhancer	RP11-1E11.2
chr9	90805000	chr9	91185000	1.59E-02	promoter	super_enhancer	RP11-1E11.2
chr9	90805000	chr9	94905000	4.35E-02	promoter	super_enhancer	RP11-1E11.2
chr9	90805000	chr9	112035000	1.06E-02	promoter	super_enhancer	RP11-1E11.2
chr9	90805000	chr9	133855000	6.15E-03	promoter	super_enhancer	RP11-1E11.2
chr9	91065000	chr9	91925000	2.62E-02	super_enhancer	promoter	CKS2
chr9	91065000	chr9	95055000	3.50E-02	super_enhancer	promoter	MIR3651
chr9	91065000	chr9	120545000	4.89E-03	super_enhancer	promoter	RP11-281A20.2
chr9	91075000	chr9	95245000	2.66E-02	super_enhancer	promoter	ASPN

chr9	91075000	chr9	111695000	9.42E-03	super_enhancer	promoter	FAM206A
chr9	91075000	chr9	123965000	5.75E-03	super_enhancer	promoter	RN7SL181P
chr9	91075000	chr9	140345000	4.90E-03	super_enhancer	promoter	MIR7114
chr9	91185000	chr9	132095000	9.84E-03	super_enhancer	promoter	LINC01503
chr9	91195000	chr9	91605000	1.99E-02	super_enhancer	promoter	C9orf47
chr9	91195000	chr9	91795000	4.04E-04	super_enhancer	promoter	SHC3
chr9	91195000	chr9	99915000	2.01E-02	super_enhancer	promoter	RP11-520B13.8
chr9	91195000	chr9	100955000	1.39E-02	super_enhancer	promoter	CORO2A
chr9	91195000	chr9	140065000	7.81E-03	super_enhancer	promoter	LRRC26
chr9	91205000	chr9	95605000	3.95E-02	super_enhancer	promoter	RP11-526D8.11
chr9	91205000	chr9	98055000	2.90E-02	super_enhancer	promoter	RP11-173G21.1
chr9	91205000	chr9	139685000	7.00E-03	super_enhancer	promoter	TMEM141
chr9	91205000	chr9	140345000	6.78E-03	super_enhancer	promoter	MIR7114
chr9	91205000	chr9	140355000	6.62E-03	super_enhancer	promoter	NSMF
chr9	91725000	chr9	94915000	4.92E-02	promoter	super_enhancer	RP11-82L18.2
chr9	91725000	chr9	124305000	9.53E-03	promoter	super_enhancer	RP11-82L18.2
chr9	91795000	chr9	127505000	9.74E-03	promoter	super_enhancer	SHC3
chr9	91795000	chr9	130195000	9.18E-03	promoter	super_enhancer	SHC3
chr9	91795000	chr9	133845000	8.50E-03	promoter	super_enhancer	SHC3
chr9	91925000	chr9	129415000	8.83E-03	promoter	super_enhancer	CKS2
chr9	92505000	chr9	129355000	1.30E-02	promoter	super_enhancer	RP5-1050E16.1
chr9	92845000	chr9	110745000	1.09E-02	promoter	super_enhancer	IL6RP1
chr9	93345000	chr9	112025000	1.60E-02	promoter	super_enhancer	LINC01501
chr9	93345000	chr9	130135000	1.06E-02	promoter	super_enhancer	LINC01501

chr9	93405000	chr9	125245000	6.53E-03	promoter	super_enhancer	DIRAS2
chr9	93515000	chr9	113615000	8.92E-03	promoter	super_enhancer	OR7E108P
chr9	93515000	chr9	128065000	1.15E-02	promoter	super_enhancer	OR7E108P
chr9	93515000	chr9	134615000	9.29E-03	promoter	super_enhancer	OR7E108P
chr9	93925000	chr9	136115000	8.62E-03	promoter	super_enhancer	RP11-305L7.3
chr9	93925000	chr9	136685000	9.13E-03	promoter	super_enhancer	RP11-305L7.3
chr9	94125000	chr9	129475000	1.17E-02	promoter	super_enhancer	AUH
chr9	94125000	chr9	137615000	8.05E-03	promoter	super_enhancer	AUH
chr9	94895000	chr9	111985000	1.73E-02	promoter	super_enhancer	RP11-100G15.7
chr9	94895000	chr9	127175000	4.55E-05	promoter	super_enhancer	RP11-100G15.7
chr9	94905000	chr9	100505000	4.52E-02	super_enhancer	promoter	RP11-546O6.4
chr9	94905000	chr9	116555000	1.80E-02	super_enhancer	promoter	AL157702.1
chr9	94905000	chr9	117445000	1.22E-02	super_enhancer	promoter	RP11-402G3.3
chr9	94905000	chr9	131085000	1.42E-02	super_enhancer	promoter	TRUB2
chr9	94905000	chr9	139935000	7.46E-03	super_enhancer	promoter	RP11-229P13.27
chr9	94915000	chr9	100395000	3.13E-02	super_enhancer	promoter	NCBP1
chr9	94915000	chr9	111465000	1.92E-02	super_enhancer	promoter	RP11-339N8.1
chr9	94915000	chr9	116615000	1.36E-02	super_enhancer	promoter	RP11-534I8.1
chr9	94915000	chr9	136205000	8.51E-03	super_enhancer	promoter	RP11-244N20.7
chr9	94925000	chr9	125485000	1.03E-02	super_enhancer	promoter	OR1L4
chr9	94925000	chr9	132405000	1.10E-02	super_enhancer	promoter	ASB6
chr9	94935000	chr9	107525000	1.07E-02	super_enhancer	promoter	NIPSNAP3B
chr9	94935000	chr9	117335000	1.63E-02	super_enhancer	promoter	RP11-402G3.6
chr9	94935000	chr9	123985000	1.22E-02	super_enhancer	promoter	RAB14

chr9	94935000	chr9	139335000	8.31E-03	super_enhancer	promoter	INPP5E
chr9	94945000	chr9	111625000	2.08E-02	super_enhancer	promoter	ACTL7A
chr9	94945000	chr9	127625000	1.52E-02	super_enhancer	promoter	RPL35
chr9	95105000	chr9	111955000	1.37E-02	promoter	super_enhancer	RP11-62C3.8
chr9	95105000	chr9	127725000	6.87E-03	promoter	super_enhancer	RP11-62C3.8
chr9	95605000	chr9	124805000	7.32E-03	promoter	super_enhancer	RP11-526D8.11
chr9	95645000	chr9	130175000	1.23E-02	promoter	super_enhancer	RP11-526D8.7
chr9	95645000	chr9	130195000	1.32E-02	promoter	super_enhancer	RP11-526D8.7
chr9	95645000	chr9	131315000	1.31E-02	promoter	super_enhancer	RP11-526D8.7
chr9	96195000	chr9	111805000	1.79E-02	promoter	super_enhancer	RP11-165J3.5
chr9	96195000	chr9	131305000	1.31E-02	promoter	super_enhancer	RP11-165J3.5
chr9	97095000	chr9	127165000	5.33E-03	promoter	super_enhancer	RP11-307E17.8
chr9	97095000	chr9	129775000	5.03E-03	promoter	super_enhancer	RP11-307E17.8
chr9	97875000	chr9	111985000	2.00E-02	promoter	super_enhancer	RP11-80I15.4
chr9	97875000	chr9	127575000	1.43E-02	promoter	super_enhancer	RP11-80I15.4
chr9	97875000	chr9	137275000	1.05E-02	promoter	super_enhancer	RP11-80I15.4
chr9	97885000	chr9	111745000	1.83E-02	promoter	super_enhancer	RP11-80I15.1
chr9	97885000	chr9	111805000	2.04E-02	promoter	super_enhancer	RP11-80I15.1
chr9	97885000	chr9	111855000	2.15E-02	promoter	super_enhancer	RP11-80I15.1
chr9	97885000	chr9	127195000	1.61E-02	promoter	super_enhancer	RP11-80I15.1
chr9	97885000	chr9	127545000	1.48E-02	promoter	super_enhancer	RP11-80I15.1
chr9	97885000	chr9	134595000	1.29E-02	promoter	super_enhancer	RP11-80I15.1
chr9	97885000	chr9	137275000	1.15E-02	promoter	super_enhancer	RP11-80I15.1
chr9	97905000	chr9	124865000	1.81E-02	promoter	super_enhancer	RNA5SP288

chr9	97905000	chr9	129425000	1.51E-02	promoter	super_enhancer	RNA5SP288
chr9	98055000	chr9	111965000	1.97E-02	promoter	super_enhancer	RP11-173G21.1
chr9	98055000	chr9	127515000	9.63E-03	promoter	super_enhancer	RP11-173G21.1
chr9	98055000	chr9	127555000	1.69E-02	promoter	super_enhancer	RP11-173G21.1
chr9	98055000	chr9	134615000	1.17E-02	promoter	super_enhancer	RP11-173G21.1
chr9	98275000	chr9	123925000	6.02E-05	promoter	super_enhancer	RP11-435O5.4
chr9	98275000	chr9	127545000	1.35E-02	promoter	super_enhancer	RP11-435O5.4
chr9	98275000	chr9	127725000	1.09E-02	promoter	super_enhancer	RP11-435O5.4
chr9	98415000	chr9	128095000	1.22E-02	promoter	super_enhancer	RP11-180I4.1
chr9	98535000	chr9	111815000	1.98E-02	promoter	super_enhancer	RP11-180I4.4
chr9	98535000	chr9	111855000	2.11E-02	promoter	super_enhancer	RP11-180I4.4
chr9	98535000	chr9	111865000	1.68E-04	promoter	super_enhancer	RP11-180I4.4
chr9	98535000	chr9	136085000	9.79E-03	promoter	super_enhancer	RP11-180I4.4
chr9	99065000	chr9	111745000	1.93E-02	promoter	super_enhancer	HSD17B3
chr9	99065000	chr9	112035000	2.11E-02	promoter	super_enhancer	HSD17B3
chr9	99065000	chr9	113625000	1.83E-02	promoter	super_enhancer	HSD17B3
chr9	99065000	chr9	124815000	1.56E-02	promoter	super_enhancer	HSD17B3
chr9	99845000	chr9	111805000	1.69E-02	promoter	super_enhancer	GAS2L1P2
chr9	99845000	chr9	129505000	1.13E-02	promoter	super_enhancer	GAS2L1P2
chr9	99915000	chr9	112065000	1.92E-02	promoter	super_enhancer	RP11-520B13.8
chr9	99915000	chr9	123935000	1.27E-02	promoter	super_enhancer	RP11-520B13.8
chr9	99955000	chr9	112025000	1.22E-02	promoter	super_enhancer	RNU6-798P
chr9	99995000	chr9	123925000	9.96E-03	promoter	super_enhancer	RP11-498P14.4
chr9	99995000	chr9	127165000	1.17E-02	promoter	super_enhancer	RP11-498P14.4

chr9	99995000	chr9	130135000	1.09E-02	promoter	super_enhancer	RP11-498P14.4
chr9	100395000	chr9	124395000	9.41E-03	promoter	super_enhancer	NCBP1
chr9	100505000	chr9	111955000	2.96E-02	promoter	super_enhancer	RP11-546O6.4
chr9	100505000	chr9	111975000	2.71E-02	promoter	super_enhancer	RP11-546O6.4
chr9	100505000	chr9	137615000	1.06E-02	promoter	super_enhancer	RP11-546O6.4
chr9	100615000	chr9	111835000	3.25E-02	promoter	super_enhancer	FOXE1
chr9	100615000	chr9	111935000	2.86E-02	promoter	super_enhancer	FOXE1
chr9	100615000	chr9	128905000	1.70E-02	promoter	super_enhancer	FOXE1
chr9	100615000	chr9	133905000	2.05E-02	promoter	super_enhancer	FOXE1
chr9	100615000	chr9	134675000	1.49E-02	promoter	super_enhancer	FOXE1
chr9	100685000	chr9	107715000	8.49E-03	promoter	super_enhancer	C9orf156
chr9	100685000	chr9	107735000	8.09E-03	promoter	super_enhancer	C9orf156
chr9	100685000	chr9	111855000	6.41E-05	promoter	super_enhancer	C9orf156
chr9	100685000	chr9	127525000	6.71E-03	promoter	super_enhancer	C9orf156
chr9	100685000	chr9	131305000	7.32E-03	promoter	super_enhancer	C9orf156
chr9	100745000	chr9	111745000	1.71E-02	promoter	super_enhancer	ANP32B
chr9	100745000	chr9	111855000	2.00E-02	promoter	super_enhancer	ANP32B
chr9	100745000	chr9	128905000	1.11E-02	promoter	super_enhancer	ANP32B
chr9	100745000	chr9	130125000	1.06E-02	promoter	super_enhancer	ANP32B
chr9	100745000	chr9	137275000	1.01E-02	promoter	super_enhancer	ANP32B
chr9	100795000	chr9	111815000	2.67E-02	promoter	super_enhancer	RNU6-918P
chr9	100795000	chr9	127595000	1.85E-02	promoter	super_enhancer	RNU6-918P
chr9	100955000	chr9	107715000	1.17E-02	promoter	super_enhancer	CORO2A
chr9	100955000	chr9	129145000	9.79E-03	promoter	super_enhancer	CORO2A

chr9	101475000	chr9	107755000	1.14E-02	promoter	super_enhancer	AL353782.1
chr9	101475000	chr9	115675000	1.37E-02	promoter	super_enhancer	AL353782.1
chr9	101475000	chr9	127505000	1.06E-02	promoter	super_enhancer	AL353782.1
chr9	101635000	chr9	111805000	1.60E-02	promoter	super_enhancer	RP11-92C4.3
chr9	101635000	chr9	129325000	8.31E-03	promoter	super_enhancer	RP11-92C4.3
chr9	101635000	chr9	134645000	7.92E-03	promoter	super_enhancer	RP11-92C4.3
chr9	101705000	chr9	111945000	1.73E-02	promoter	super_enhancer	COL15A1
chr9	101705000	chr9	133865000	9.93E-03	promoter	super_enhancer	COL15A1
chr9	101705000	chr9	136095000	6.36E-03	promoter	super_enhancer	COL15A1
chr9	101865000	chr9	111805000	1.16E-02	promoter	super_enhancer	TGFBR1
chr9	101985000	chr9	111805000	1.47E-02	promoter	super_enhancer	ALG2
chr9	101985000	chr9	111815000	1.45E-02	promoter	super_enhancer	ALG2
chr9	101985000	chr9	111845000	1.14E-02	promoter	super_enhancer	ALG2
chr9	102045000	chr9	111745000	1.12E-02	promoter	super_enhancer	RN7SKP225
chr9	102045000	chr9	124815000	8.66E-03	promoter	super_enhancer	RN7SKP225
chr9	102865000	chr9	111805000	1.34E-02	promoter	super_enhancer	RN7SL75P
chr9	103205000	chr9	111945000	1.80E-02	promoter	super_enhancer	MSANTD3-TMEFF1
chr9	103205000	chr9	124825000	9.46E-03	promoter	super_enhancer	MSANTD3-TMEFF1
chr9	103205000	chr9	133865000	9.87E-03	promoter	super_enhancer	MSANTD3-TMEFF1
chr9	103235000	chr9	124865000	1.11E-02	promoter	super_enhancer	TMEFF1
chr9	103235000	chr9	127535000	1.09E-02	promoter	super_enhancer	TMEFF1
chr9	103345000	chr9	134655000	6.60E-03	promoter	super_enhancer	RN7SKP87
chr9	104265000	chr9	111995000	1.70E-02	promoter	super_enhancer	RP11-490D19.8
chr9	104265000	chr9	127535000	1.13E-02	promoter	super_enhancer	RP11-490D19.8

chr9	104265000	chr9	130125000	8.27E-03	promoter	super_enhancer	RP11-490D19.8
chr9	104295000	chr9	107755000	2.07E-04	promoter	super_enhancer	RNF20
chr9	104295000	chr9	129405000	1.14E-02	promoter	super_enhancer	RNF20
chr9	105375000	chr9	127195000	1.03E-02	promoter	super_enhancer	ZYG11AP1
chr9	106855000	chr9	111815000	1.98E-02	promoter	super_enhancer	SMC2-AS1
chr9	107485000	chr9	111815000	2.50E-02	promoter	super_enhancer	OR13D3P
chr9	107525000	chr9	107755000	8.36E-03	promoter	super_enhancer	NIPSNAP3B
chr9	107525000	chr9	111865000	2.25E-02	promoter	super_enhancer	NIPSNAP3B
chr9	107525000	chr9	130195000	8.85E-03	promoter	super_enhancer	NIPSNAP3B
chr9	107715000	chr9	133275000	9.64E-03	super_enhancer	promoter	RN7SL665P
chr9	107745000	chr9	109885000	1.48E-03	super_enhancer	promoter	RP11-196I18.4
chr9	107755000	chr9	117425000	1.27E-02	super_enhancer	promoter	RP11-402G3.5
chr9	107755000	chr9	126605000	6.17E-03	super_enhancer	promoter	PIGFP2
chr9	108425000	chr9	113615000	2.61E-02	promoter	super_enhancer	TAL2
chr9	109355000	chr9	128885000	1.82E-02	promoter	super_enhancer	RP11-540H22.2
chr9	109485000	chr9	129405000	1.84E-02	promoter	super_enhancer	RP11-308N19.5
chr9	109485000	chr9	137275000	1.23E-02	promoter	super_enhancer	RP11-308N19.5
chr9	109625000	chr9	124805000	1.29E-02	promoter	super_enhancer	ZNF462
chr9	109625000	chr9	127545000	1.83E-02	promoter	super_enhancer	ZNF462
chr9	109885000	chr9	110745000	4.69E-02	promoter	super_enhancer	RP11-196I18.4
chr9	109885000	chr9	127185000	1.85E-02	promoter	super_enhancer	RP11-196I18.4
chr9	109885000	chr9	133845000	1.32E-02	promoter	super_enhancer	RP11-196I18.4
chr9	110045000	chr9	124395000	1.41E-02	promoter	super_enhancer	RAD23B
chr9	110045000	chr9	129395000	1.04E-02	promoter	super_enhancer	RAD23B

chr9	110055000	chr9	111835000	1.13E-02	promoter	super_enhancer	AL137852.1
chr9	110475000	chr9	124875000	2.17E-02	promoter	super_enhancer	AL389915.1
chr9	110475000	chr9	129405000	2.48E-02	promoter	super_enhancer	AL389915.1
chr9	110575000	chr9	111815000	4.24E-02	promoter	super_enhancer	RN7SL659P
chr9	110575000	chr9	127165000	2.15E-02	promoter	super_enhancer	RN7SL659P
chr9	110575000	chr9	127595000	2.20E-02	promoter	super_enhancer	RN7SL659P
chr9	110575000	chr9	129725000	2.20E-02	promoter	super_enhancer	RN7SL659P
chr9	110575000	chr9	130195000	2.03E-02	promoter	super_enhancer	RN7SL659P
chr9	110745000	chr9	127615000	2.00E-02	super_enhancer	promoter	WDR38
chr9	110745000	chr9	139555000	1.19E-02	super_enhancer	promoter	RP11-251M1.1
chr9	111465000	chr9	127565000	2.50E-02	promoter	super_enhancer	RP11-339N8.1
chr9	111465000	chr9	128905000	2.16E-02	promoter	super_enhancer	RP11-339N8.1
chr9	111465000	chr9	129505000	2.41E-02	promoter	super_enhancer	RP11-339N8.1
chr9	111465000	chr9	130185000	1.97E-02	promoter	super_enhancer	RP11-339N8.1
chr9	111625000	chr9	111955000	2.94E-02	promoter	super_enhancer	ACTL7A
chr9	111625000	chr9	127735000	1.94E-02	promoter	super_enhancer	ACTL7A
chr9	111625000	chr9	129745000	1.97E-02	promoter	super_enhancer	ACTL7A
chr9	111625000	chr9	136905000	1.43E-02	promoter	super_enhancer	ACTL7A
chr9	111735000	chr9	112275000	4.27E-02	super_enhancer	promoter	MIR3927
chr9	111735000	chr9	117085000	3.28E-02	super_enhancer	promoter	ORM1
chr9	111755000	chr9	124405000	3.12E-02	promoter	super_enhancer	RNA5-8SP3
chr9	111805000	chr9	117335000	4.33E-02	super_enhancer	promoter	RP11-402G3.6
chr9	111815000	chr9	124045000	2.67E-02	super_enhancer	promoter	RP11-477J21.6
chr9	111815000	chr9	131085000	2.32E-02	super_enhancer	promoter	TRUB2

chr9	111815000	chr9	131645000	2.14E-02	super_enhancer	promoter	CCBL1
chr9	111825000	chr9	122735000	5.13E-04	super_enhancer	promoter	RP11-360A18.1
chr9	111825000	chr9	132815000	1.98E-02	super_enhancer	promoter	GPR107
chr9	111835000	chr9	125875000	3.11E-02	super_enhancer	promoter	MIR600
chr9	111845000	chr9	112275000	4.43E-02	super_enhancer	promoter	MIR3927
chr9	111845000	chr9	116555000	4.27E-02	super_enhancer	promoter	AL157702.1
chr9	111845000	chr9	139875000	1.21E-02	super_enhancer	promoter	LCNL1
chr9	111855000	chr9	112275000	2.39E-02	super_enhancer	promoter	MIR3927
chr9	111855000	chr9	127615000	2.55E-02	super_enhancer	promoter	WDR38
chr9	111865000	chr9	116615000	4.03E-02	super_enhancer	promoter	RP11-534I8.1
chr9	111865000	chr9	141045000	1.03E-02	super_enhancer	promoter	TUBBP5
chr9	111935000	chr9	116615000	4.46E-02	super_enhancer	promoter	RP11-534I8.1
chr9	111935000	chr9	117335000	4.40E-02	super_enhancer	promoter	RP11-402G3.6
chr9	111965000	chr9	123575000	2.80E-02	super_enhancer	promoter	RP11-27I1.6
chr9	111975000	chr9	132805000	2.29E-02	super_enhancer	promoter	FNBP1
chr9	111985000	chr9	128005000	2.16E-02	super_enhancer	promoter	HSPA5
chr9	111985000	chr9	132045000	2.19E-02	super_enhancer	promoter	RP11-344B5.2
chr9	111985000	chr9	134955000	1.58E-02	super_enhancer	promoter	MED27
chr9	111985000	chr9	139965000	1.52E-02	super_enhancer	promoter	SAPCD2
chr9	111995000	chr9	136605000	1.82E-02	super_enhancer	promoter	SARDH
chr9	111995000	chr9	140485000	1.37E-02	super_enhancer	promoter	ZMYND19
chr9	112005000	chr9	125875000	3.32E-02	super_enhancer	promoter	MIR600
chr9	112005000	chr9	140485000	1.59E-02	super_enhancer	promoter	ZMYND19
chr9	112015000	chr9	117335000	4.69E-02	super_enhancer	promoter	RP11-402G3.6

chr9	112015000	chr9	125695000	2.39E-02	super_enhancer	promoter	ZBTB26
chr9	112015000	chr9	133815000	1.83E-02	super_enhancer	promoter	FIBCD1
chr9	112015000	chr9	134955000	1.53E-02	super_enhancer	promoter	MED27
chr9	112015000	chr9	139335000	1.35E-02	super_enhancer	promoter	INPP5E
chr9	112025000	chr9	127615000	2.57E-02	super_enhancer	promoter	WDR38
chr9	112045000	chr9	117445000	4.05E-02	super_enhancer	promoter	RP11-402G3.3
chr9	112045000	chr9	123575000	3.26E-02	super_enhancer	promoter	RP11-27I1.6
chr9	112045000	chr9	124045000	3.34E-02	super_enhancer	promoter	RP11-477J21.6
chr9	112045000	chr9	131085000	2.90E-02	super_enhancer	promoter	TRUB2
chr9	112045000	chr9	139695000	1.87E-02	super_enhancer	promoter	RP11-216L13.18
chr9	112055000	chr9	126605000	1.40E-02	super_enhancer	promoter	PIGFP2
chr9	112055000	chr9	129885000	2.02E-02	super_enhancer	promoter	ANGPTL2
chr9	112075000	chr9	132695000	2.07E-02	super_enhancer	promoter	RP11-409K20.6
chr9	112075000	chr9	140445000	1.54E-02	super_enhancer	promoter	PNPLA7
chr9	112275000	chr9	128065000	2.42E-02	promoter	super_enhancer	MIR3927
chr9	112275000	chr9	128095000	2.12E-02	promoter	super_enhancer	MIR3927
chr9	112275000	chr9	129505000	2.09E-02	promoter	super_enhancer	MIR3927
chr9	113605000	chr9	136325000	1.26E-02	super_enhancer	promoter	CACFD1
chr9	113615000	chr9	125875000	2.09E-02	super_enhancer	promoter	MIR600
chr9	113625000	chr9	125135000	3.05E-02	super_enhancer	promoter	RP11-498E2.9
chr9	113625000	chr9	132095000	1.79E-02	super_enhancer	promoter	LINC01503
chr9	113665000	chr9	130945000	1.68E-02	promoter	super_enhancer	RNU6-432P
chr9	113665000	chr9	136905000	1.31E-02	promoter	super_enhancer	RNU6-432P
chr9	114175000	chr9	127515000	2.15E-02	promoter	super_enhancer	RNA5SP294

chr9	114175000	chr9	129475000	2.73E-02	promoter	super_enhancer	RNA5SP294
chr9	114335000	chr9	124825000	2.48E-02	promoter	super_enhancer	RP11-16L21.7
chr9	114335000	chr9	130185000	1.88E-02	promoter	super_enhancer	RP11-16L21.7
chr9	114335000	chr9	130945000	1.81E-02	promoter	super_enhancer	RP11-16L21.7
chr9	114335000	chr9	136085000	1.53E-02	promoter	super_enhancer	RP11-16L21.7
chr9	114395000	chr9	124405000	3.33E-02	promoter	super_enhancer	LRRC37A5P
chr9	114395000	chr9	124865000	2.99E-02	promoter	super_enhancer	LRRC37A5P
chr9	114395000	chr9	130135000	2.24E-02	promoter	super_enhancer	LRRC37A5P
chr9	114565000	chr9	124835000	2.51E-02	promoter	super_enhancer	RP11-430K21.2
chr9	115005000	chr9	127185000	3.37E-02	promoter	super_enhancer	RN7SL57P
chr9	115005000	chr9	128095000	2.77E-02	promoter	super_enhancer	RN7SL57P
chr9	115005000	chr9	129415000	3.10E-02	promoter	super_enhancer	RN7SL57P
chr9	115005000	chr9	130115000	2.59E-02	promoter	super_enhancer	RN7SL57P
chr9	115015000	chr9	130195000	1.29E-02	promoter	super_enhancer	RNA5SP295
chr9	115035000	chr9	129145000	2.45E-02	promoter	super_enhancer	RN7SL430P
chr9	115045000	chr9	127565000	2.12E-02	promoter	super_enhancer	HSPE1P28
chr9	115045000	chr9	129395000	1.27E-02	promoter	super_enhancer	HSPE1P28
chr9	115125000	chr9	130945000	2.00E-02	promoter	super_enhancer	RP11-432F4.2
chr9	115125000	chr9	135155000	1.46E-02	promoter	super_enhancer	RP11-432F4.2
chr9	115345000	chr9	124795000	2.81E-02	promoter	super_enhancer	RP11-576C12.1
chr9	115345000	chr9	124805000	2.02E-02	promoter	super_enhancer	RP11-576C12.1
chr9	115345000	chr9	127725000	2.28E-02	promoter	super_enhancer	RP11-576C12.1
chr9	115345000	chr9	133845000	2.20E-02	promoter	super_enhancer	RP11-576C12.1
chr9	115675000	chr9	115725000	1.82E-02	super_enhancer	promoter	MUPP

chr9	115675000	chr9	132935000	1.97E-02	super_enhancer	promoter	NCS1
chr9	115675000	chr9	134165000	2.06E-02	super_enhancer	promoter	PPAPDC3
chr9	115675000	chr9	137545000	1.13E-02	super_enhancer	promoter	COL5A1-AS1
chr9	115725000	chr9	124835000	3.49E-02	promoter	super_enhancer	MUPP
chr9	115725000	chr9	127545000	2.93E-02	promoter	super_enhancer	MUPP
chr9	115725000	chr9	129145000	2.81E-02	promoter	super_enhancer	MUPP
chr9	115985000	chr9	124395000	1.93E-02	promoter	super_enhancer	FKBP15
chr9	115985000	chr9	127535000	2.55E-02	promoter	super_enhancer	FKBP15
chr9	116165000	chr9	127505000	3.13E-02	promoter	super_enhancer	ALAD
chr9	116165000	chr9	127575000	3.30E-02	promoter	super_enhancer	ALAD
chr9	116165000	chr9	127585000	3.51E-02	promoter	super_enhancer	ALAD
chr9	116165000	chr9	129405000	3.36E-02	promoter	super_enhancer	ALAD
chr9	116165000	chr9	136675000	2.06E-02	promoter	super_enhancer	ALAD
chr9	116165000	chr9	136695000	1.96E-02	promoter	super_enhancer	ALAD
chr9	116165000	chr9	137315000	2.31E-02	promoter	super_enhancer	ALAD
chr9	116555000	chr9	129415000	6.94E-04	promoter	super_enhancer	AL157702.1
chr9	116615000	chr9	134595000	2.27E-02	promoter	super_enhancer	RP11-534I8.1
chr9	117085000	chr9	127185000	3.04E-02	promoter	super_enhancer	ORM1
chr9	117335000	chr9	124815000	4.00E-02	promoter	super_enhancer	RP11-402G3.6
chr9	117335000	chr9	136905000	1.91E-02	promoter	super_enhancer	RP11-402G3.6
chr9	117425000	chr9	134645000	2.15E-02	promoter	super_enhancer	RP11-402G3.5
chr9	117425000	chr9	136695000	2.02E-02	promoter	super_enhancer	RP11-402G3.5
chr9	117445000	chr9	124835000	3.36E-02	promoter	super_enhancer	RP11-402G3.3
chr9	117445000	chr9	137615000	1.41E-02	promoter	super_enhancer	RP11-402G3.3

chr9	118775000	chr9	123935000	4.04E-02	promoter	super_enhancer	RP11-787B4.2
chr9	118775000	chr9	127165000	2.80E-02	promoter	super_enhancer	RP11-787B4.2
chr9	118775000	chr9	127515000	2.04E-02	promoter	super_enhancer	RP11-787B4.2
chr9	118915000	chr9	130195000	2.76E-02	promoter	super_enhancer	PAPPA
chr9	120545000	chr9	124865000	4.64E-02	promoter	super_enhancer	RP11-281A20.2
chr9	120545000	chr9	134615000	1.88E-02	promoter	super_enhancer	RP11-281A20.2
chr9	121285000	chr9	134955000	1.72E-02	super_enhancer	promoter	MED27
chr9	121525000	chr9	129415000	3.61E-02	promoter	super_enhancer	TUBB4BP6
chr9	123555000	chr9	123935000	1.87E-02	promoter	super_enhancer	RP11-27I1.4
chr9	123555000	chr9	130945000	3.60E-02	promoter	super_enhancer	RP11-27I1.4
chr9	123555000	chr9	134635000	3.40E-02	promoter	super_enhancer	RP11-27I1.4
chr9	123575000	chr9	123935000	3.28E-02	promoter	super_enhancer	RP11-27I1.6
chr9	123575000	chr9	128635000	4.21E-02	promoter	super_enhancer	RP11-27I1.6
chr9	123575000	chr9	129765000	3.61E-02	promoter	super_enhancer	RP11-27I1.6
chr9	123575000	chr9	135115000	2.68E-02	promoter	super_enhancer	RP11-27I1.6
chr9	123605000	chr9	130125000	4.15E-02	promoter	super_enhancer	PSMD5
chr9	123925000	chr9	134955000	2.36E-02	super_enhancer	promoter	MED27
chr9	123925000	chr9	135285000	2.03E-02	super_enhancer	promoter	C9orf171
chr9	123925000	chr9	139415000	2.19E-02	super_enhancer	promoter	MIR4673
chr9	123965000	chr9	129455000	4.34E-02	promoter	super_enhancer	RN7SL181P
chr9	124045000	chr9	134595000	4.19E-02	promoter	super_enhancer	RP11-477J21.6
chr9	124395000	chr9	125485000	4.59E-02	super_enhancer	promoter	OR1L4
chr9	124395000	chr9	130215000	4.21E-02	super_enhancer	promoter	RPL12
chr9	124395000	chr9	131085000	4.43E-02	super_enhancer	promoter	TRUB2

chr9	124405000	chr9	139695000	3.58E-02	super_enhancer	promoter	RP11-216L13.18
chr9	124795000	chr9	128275000	5.00E-03	super_enhancer	promoter	RN7SL30P
chr9	124795000	chr9	133275000	4.11E-02	super_enhancer	promoter	RN7SL665P
chr9	124795000	chr9	135955000	2.02E-02	super_enhancer	promoter	CELP
chr9	124805000	chr9	132045000	3.35E-02	super_enhancer	promoter	RP11-344B5.2
chr9	124815000	chr9	132515000	4.43E-02	super_enhancer	promoter	PTGES
chr9	124825000	chr9	125165000	4.39E-02	super_enhancer	promoter	RP11-542K23.10
chr9	124825000	chr9	138245000	2.35E-02	super_enhancer	promoter	RP11-555H7.2
chr9	124835000	chr9	140485000	3.00E-02	super_enhancer	promoter	ZMYND19
chr9	124865000	chr9	125135000	1.01E-04	super_enhancer	promoter	RP11-498E2.9
chr9	124865000	chr9	139625000	3.64E-02	super_enhancer	promoter	RP11-216L13.21
chr9	124875000	chr9	125165000	1.82E-02	super_enhancer	promoter	RP11-542K23.10
chr9	125165000	chr9	135135000	4.09E-02	promoter	super_enhancer	RP11-542K23.10
chr9	125875000	chr9	127195000	2.54E-02	promoter	super_enhancer	MIR600
chr9	126165000	chr9	137615000	2.97E-02	promoter	super_enhancer	MIR601
chr9	126605000	chr9	134385000	2.69E-02	promoter	super_enhancer	PIGFP2
chr9	127025000	chr9	128085000	3.46E-02	promoter	super_enhancer	RP11-121A14.3
chr9	127025000	chr9	134385000	4.78E-02	promoter	super_enhancer	RP11-121A14.3
chr9	127025000	chr9	135125000	4.89E-02	promoter	super_enhancer	RP11-121A14.3
chr9	127025000	chr9	136115000	4.55E-02	promoter	super_enhancer	RP11-121A14.3
chr9	127175000	chr9	135905000	2.11E-02	super_enhancer	promoter	GTF3C5
chr9	127175000	chr9	139725000	2.50E-02	super_enhancer	promoter	MIR4292
chr9	127185000	chr9	140145000	3.91E-02	super_enhancer	promoter	C9orf173
chr9	127455000	chr9	133885000	4.43E-02	promoter	super_enhancer	MIR181A2

chr9	127455000	chr9	136085000	3.07E-02	promoter	super_enhancer	MIR181A2
chr9	127495000	chr9	136205000	3.73E-02	super_enhancer	promoter	RP11-244N20.7
chr9	127515000	chr9	136185000	3.03E-02	super_enhancer	promoter	LCN1P2
chr9	127525000	chr9	136935000	3.47E-02	super_enhancer	promoter	BRD3
chr9	127535000	chr9	127625000	3.04E-03	super_enhancer	promoter	RPL35
chr9	127535000	chr9	128135000	7.04E-04	super_enhancer	promoter	RP11-184B22.2
chr9	127535000	chr9	136225000	3.87E-02	super_enhancer	promoter	SURF1
chr9	127535000	chr9	140065000	4.18E-02	super_enhancer	promoter	LRRC26
chr9	127535000	chr9	140355000	3.31E-02	super_enhancer	promoter	NSMF
chr9	127545000	chr9	127615000	1.84E-03	super_enhancer	promoter	WDR38
chr9	127555000	chr9	127615000	6.12E-03	super_enhancer	promoter	WDR38
chr9	127555000	chr9	128135000	3.58E-04	super_enhancer	promoter	RP11-184B22.2
chr9	127555000	chr9	135285000	4.42E-02	super_enhancer	promoter	C9orf171
chr9	127565000	chr9	139565000	3.17E-02	super_enhancer	promoter	MIR126
chr9	127575000	chr9	128135000	1.72E-02	super_enhancer	promoter	RP11-184B22.2
chr9	127575000	chr9	134955000	4.46E-02	super_enhancer	promoter	MED27
chr9	127575000	chr9	139625000	4.31E-02	super_enhancer	promoter	RP11-216L13.21
chr9	127585000	chr9	127615000	8.91E-03	super_enhancer	promoter	WDR38
chr9	127585000	chr9	128135000	2.33E-02	super_enhancer	promoter	RP11-184B22.2
chr9	127585000	chr9	135285000	3.99E-02	super_enhancer	promoter	C9orf171
chr9	127595000	chr9	140135000	3.59E-02	super_enhancer	promoter	TUBB4B
chr9	127615000	chr9	129335000	6.44E-03	promoter	super_enhancer	WDR38
chr9	127615000	chr9	129425000	4.32E-02	promoter	super_enhancer	WDR38
chr9	127725000	chr9	139335000	2.67E-02	super_enhancer	promoter	INPP5E

chr9	127965000	chr9	129985000	3.54E-02	super_enhancer	promoter	RP13-225O21.2
chr9	127965000	chr9	136205000	3.87E-02	super_enhancer	promoter	RP11-244N20.7
chr9	127965000	chr9	138145000	3.57E-02	super_enhancer	promoter	RP11-399H11.2
chr9	128005000	chr9	137615000	3.08E-02	promoter	super_enhancer	HSPA5
chr9	128065000	chr9	139415000	4.02E-02	super_enhancer	promoter	MIR4673
chr9	128065000	chr9	139555000	3.98E-02	super_enhancer	promoter	RP11-251M1.1
chr9	128065000	chr9	139745000	3.61E-02	super_enhancer	promoter	MAMDC4
chr9	128075000	chr9	140355000	3.06E-02	super_enhancer	promoter	NSMF
chr9	128075000	chr9	140485000	3.30E-02	super_enhancer	promoter	ZMYND19
chr9	128085000	chr9	130965000	1.22E-02	super_enhancer	promoter	DNM1
chr9	128085000	chr9	138395000	3.72E-02	super_enhancer	promoter	C9orf116
chr9	128095000	chr9	139875000	3.79E-02	super_enhancer	promoter	LCNL1
chr9	128135000	chr9	129315000	3.17E-02	promoter	super_enhancer	RP11-184B22.2
chr9	128275000	chr9	128895000	2.65E-03	promoter	super_enhancer	RN7SL30P
chr9	128635000	chr9	138555000	2.13E-02	super_enhancer	promoter	LCN9
chr9	128685000	chr9	134365000	3.80E-02	super_enhancer	promoter	SNORD62B
chr9	128685000	chr9	139695000	2.52E-02	super_enhancer	promoter	RP11-216L13.18
chr9	128885000	chr9	136325000	3.68E-02	super_enhancer	promoter	CACFD1
chr9	128905000	chr9	135545000	3.61E-02	super_enhancer	promoter	GTF3C4
chr9	129145000	chr9	140125000	3.43E-02	super_enhancer	promoter	SLC34A3
chr9	129315000	chr9	139415000	4.54E-02	super_enhancer	promoter	MIR4673
chr9	129315000	chr9	139965000	4.41E-02	super_enhancer	promoter	SAPCD2
chr9	129315000	chr9	140065000	4.84E-02	super_enhancer	promoter	LRRC26
chr9	129335000	chr9	135285000	4.63E-02	super_enhancer	promoter	C9orf171

chr9	129345000	chr9	139305000	4.33E-02	super_enhancer	promoter	SDCCAG3
chr9	129355000	chr9	139305000	4.32E-02	super_enhancer	promoter	SDCCAG3
chr9	129385000	chr9	136935000	3.92E-02	super_enhancer	promoter	BRD3
chr9	129385000	chr9	138395000	3.64E-02	super_enhancer	promoter	C9orf116
chr9	129385000	chr9	139565000	3.13E-02	super_enhancer	promoter	MIR126
chr9	129385000	chr9	139775000	2.91E-02	super_enhancer	promoter	TRAF2
chr9	129405000	chr9	131445000	3.62E-02	super_enhancer	promoter	SET
chr9	129405000	chr9	140345000	3.97E-02	super_enhancer	promoter	MIR7114
chr9	129425000	chr9	130505000	3.20E-02	super_enhancer	promoter	AL162426.1
chr9	129475000	chr9	136395000	4.87E-02	super_enhancer	promoter	TMEM8C
chr9	129475000	chr9	139875000	4.14E-02	super_enhancer	promoter	LCNL1
chr9	129485000	chr9	137545000	2.36E-02	super_enhancer	promoter	COL5A1-AS1
chr9	129485000	chr9	138395000	3.62E-02	super_enhancer	promoter	C9orf116
chr9	129505000	chr9	140345000	3.44E-02	super_enhancer	promoter	MIR7114
chr9	129505000	chr9	140445000	3.68E-02	super_enhancer	promoter	PNPLA7
chr9	129725000	chr9	129885000	9.18E-04	super_enhancer	promoter	ANGPTL2
chr9	129725000	chr9	130505000	2.80E-02	super_enhancer	promoter	AL162426.1
chr9	129725000	chr9	136325000	4.46E-02	super_enhancer	promoter	CACFD1
chr9	129735000	chr9	136205000	4.26E-02	super_enhancer	promoter	RP11-244N20.7
chr9	129745000	chr9	129885000	1.04E-02	super_enhancer	promoter	ANGPTL2
chr9	129755000	chr9	139685000	2.48E-02	super_enhancer	promoter	TMEM141
chr9	129775000	chr9	138395000	4.17E-02	super_enhancer	promoter	C9orf116
chr9	129885000	chr9	130185000	2.12E-02	promoter	super_enhancer	ANGPTL2
chr9	129885000	chr9	137415000	4.35E-02	promoter	super_enhancer	ANGPTL2

chr9	129985000	chr9	130135000	1.13E-03	promoter	super_enhancer	RP13-225O21.2
chr9	130125000	chr9	136215000	4.23E-02	super_enhancer	promoter	MED22
chr9	130135000	chr9	138245000	3.52E-02	super_enhancer	promoter	RP11-555H7.2
chr9	130135000	chr9	138555000	3.73E-02	super_enhancer	promoter	LCN9
chr9	130135000	chr9	139775000	3.38E-02	super_enhancer	promoter	TRAF2
chr9	130175000	chr9	136215000	4.63E-02	super_enhancer	promoter	MED22
chr9	130175000	chr9	137545000	2.81E-02	super_enhancer	promoter	COL5A1-AS1
chr9	130195000	chr9	137445000	4.91E-02	super_enhancer	promoter	RP11-473E2.3
chr9	130915000	chr9	132095000	5.29E-03	super_enhancer	promoter	LINC01503
chr9	130915000	chr9	139745000	4.55E-02	super_enhancer	promoter	MAMDC4
chr9	130925000	chr9	139335000	4.09E-02	super_enhancer	promoter	INPP5E
chr9	130925000	chr9	139695000	4.71E-02	super_enhancer	promoter	RP11-216L13.18
chr9	130935000	chr9	139555000	4.53E-02	super_enhancer	promoter	RP11-251M1.1
chr9	130945000	chr9	135905000	3.95E-02	super_enhancer	promoter	GTF3C5
chr9	130955000	chr9	131155000	2.85E-02	super_enhancer	promoter	MIR219B
chr9	130955000	chr9	137545000	3.27E-02	super_enhancer	promoter	COL5A1-AS1
chr9	130955000	chr9	139305000	4.46E-02	super_enhancer	promoter	SDCCAG3
chr9	131305000	chr9	139745000	4.40E-02	super_enhancer	promoter	MAMDC4
chr9	131305000	chr9	140355000	4.07E-02	super_enhancer	promoter	NSMF
chr9	131315000	chr9	140135000	4.63E-02	super_enhancer	promoter	TUBB4B
chr9	131445000	chr9	134385000	8.07E-03	promoter	super_enhancer	SET
chr9	132815000	chr9	134375000	4.48E-02	promoter	super_enhancer	GPR107
chr9	133275000	chr9	133905000	2.06E-03	promoter	super_enhancer	RN7SL665P
chr9	133815000	chr9	133885000	1.39E-02	promoter	super_enhancer	FIBCD1

chr9	133815000	chr9	133895000	1.84E-02	promoter	super_enhancer	FIBCD1
chr9	134365000	chr9	134375000	4.79E-02	promoter	super_enhancer	SNORD62B
chr9	134365000	chr9	134595000	3.09E-02	promoter	super_enhancer	SNORD62B
chr9	134365000	chr9	134635000	4.71E-02	promoter	super_enhancer	SNORD62B
chr9	134365000	chr9	135115000	7.67E-03	promoter	super_enhancer	SNORD62B
chr9	134375000	chr9	136215000	2.03E-02	super_enhancer	promoter	MED22
chr9	134615000	chr9	135545000	4.79E-03	super_enhancer	promoter	GTF3C4
chr9	134635000	chr9	138145000	5.98E-03	super_enhancer	promoter	RP11-399H11.2
chr9	134645000	chr9	136205000	4.23E-02	super_enhancer	promoter	RP11-244N20.7
chr9	134675000	chr9	136215000	3.84E-02	super_enhancer	promoter	MED22
chr9	135115000	chr9	140125000	4.77E-02	super_enhancer	promoter	SLC34A3
chr9	135115000	chr9	140355000	4.56E-02	super_enhancer	promoter	NSMF
chr9	135145000	chr9	139725000	1.64E-03	super_enhancer	promoter	MIR4292
chr9	135895000	chr9	136085000	3.66E-02	promoter	super_enhancer	EEF1A1P5
chr9	135955000	chr9	136105000	2.50E-02	promoter	super_enhancer	CELP
chr9	136025000	chr9	137275000	2.67E-02	promoter	super_enhancer	RALGDS
chr9	136085000	chr9	136185000	2.45E-02	super_enhancer	promoter	LCN1P2
chr9	136085000	chr9	136605000	1.93E-02	super_enhancer	promoter	SARDH
chr9	136115000	chr9	136185000	1.84E-02	super_enhancer	promoter	LCN1P2
chr9	136115000	chr9	136395000	4.19E-02	super_enhancer	promoter	TMEM8C
chr9	136395000	chr9	136675000	3.40E-02	promoter	super_enhancer	TMEM8C
chr9	136695000	chr9	139415000	1.09E-02	super_enhancer	promoter	MIR4673
chr9	137275000	chr9	139335000	2.43E-02	super_enhancer	promoter	INPP5E
chr9	137285000	chr9	141075000	4.11E-02	super_enhancer	promoter	RP11-424E7.6

chr9	137315000	chr9	137445000	3.75E-02	super_enhancer	promoter	RP11-473E2.3
chr10	195000	chr10	1955000	4.44E-02	promoter	super_enhancer	RP11-631M21.7
chr10	195000	chr10	11885000	6.86E-03	promoter	super_enhancer	RP11-631M21.7
chr10	675000	chr10	79085000	2.00E-03	promoter	super_enhancer	RP11-809C18.3
chr10	675000	chr10	91605000	2.26E-03	promoter	super_enhancer	RP11-809C18.3
chr10	695000	chr10	71255000	2.68E-03	promoter	super_enhancer	PRR26
chr10	695000	chr10	125135000	1.47E-03	promoter	super_enhancer	PRR26
chr10	1035000	chr10	74055000	3.27E-03	promoter	super_enhancer	RP11-363N22.3
chr10	1095000	chr10	1955000	9.81E-03	promoter	super_enhancer	IDI1
chr10	1335000	chr10	11885000	8.47E-03	promoter	super_enhancer	RP11-29A19.2
chr10	1405000	chr10	3085000	3.26E-03	promoter	super_enhancer	RP11-398B16.2
chr10	1955000	chr10	5885000	1.75E-02	super_enhancer	promoter	GDI2
chr10	1955000	chr10	13635000	5.63E-03	super_enhancer	promoter	RP11-295P9.2
chr10	1955000	chr10	121885000	1.32E-03	super_enhancer	promoter	AL353664.1
chr10	1965000	chr10	2495000	2.61E-02	super_enhancer	promoter	AC024908.1
chr10	1965000	chr10	19025000	6.88E-03	super_enhancer	promoter	AIFM1P1
chr10	1965000	chr10	23075000	4.55E-03	super_enhancer	promoter	RNU6-413P
chr10	1975000	chr10	5275000	2.66E-02	super_enhancer	promoter	RP11-445P17.3
chr10	1975000	chr10	119805000	1.58E-03	super_enhancer	promoter	CASC2
chr10	3075000	chr10	15065000	6.69E-03	super_enhancer	promoter	DCLRE1CP1
chr10	3075000	chr10	34965000	3.18E-03	super_enhancer	promoter	RP11-8L18.2
chr10	3085000	chr10	3215000	1.32E-02	super_enhancer	promoter	PITRM1
chr10	3095000	chr10	105005000	1.94E-03	super_enhancer	promoter	RPEL1
chr10	3215000	chr10	3825000	3.46E-02	promoter	super_enhancer	PITRM1

chr10	3215000	chr10	88895000	1.38E-03	promoter	super_enhancer	PITRM1
chr10	3215000	chr10	133275000	1.66E-03	promoter	super_enhancer	PITRM1
chr10	3265000	chr10	3805000	3.13E-02	promoter	super_enhancer	RP11-298E9.5
chr10	3265000	chr10	3815000	2.82E-02	promoter	super_enhancer	RP11-298E9.5
chr10	3825000	chr10	104195000	2.35E-03	super_enhancer	promoter	MIR146B
chr10	3875000	chr10	79165000	2.66E-03	promoter	super_enhancer	RP11-464C19.2
chr10	3875000	chr10	122115000	1.23E-03	promoter	super_enhancer	RP11-464C19.2
chr10	4425000	chr10	80455000	2.36E-03	promoter	super_enhancer	LINC00703
chr10	5005000	chr10	89275000	1.37E-03	promoter	super_enhancer	AKR1C1
chr10	5855000	chr10	11905000	1.58E-02	promoter	super_enhancer	RP11-318E3.9
chr10	5855000	chr10	52485000	2.37E-03	promoter	super_enhancer	RP11-318E3.9
chr10	5905000	chr10	52455000	2.82E-03	promoter	super_enhancer	RP11-318E3.4
chr10	6105000	chr10	71965000	2.72E-03	promoter	super_enhancer	IL2RA
chr10	6115000	chr10	11875000	2.02E-02	promoter	super_enhancer	RP11-414H17.2
chr10	6115000	chr10	11915000	2.16E-02	promoter	super_enhancer	RP11-414H17.2
chr10	6245000	chr10	11915000	2.05E-02	promoter	super_enhancer	RP11-414H17.5
chr10	6245000	chr10	101045000	2.41E-03	promoter	super_enhancer	RP11-414H17.5
chr10	7515000	chr10	11875000	2.31E-02	promoter	super_enhancer	RP5-1031D4.2
chr10	7515000	chr10	11895000	2.62E-02	promoter	super_enhancer	RP5-1031D4.2
chr10	7515000	chr10	119255000	1.82E-03	promoter	super_enhancer	RP5-1031D4.2
chr10	7575000	chr10	11895000	2.43E-02	promoter	super_enhancer	RP11-385N23.1
chr10	7745000	chr10	93425000	2.26E-03	promoter	super_enhancer	ITIH2
chr10	8095000	chr10	11915000	3.25E-02	promoter	super_enhancer	GATA3-AS1
chr10	10995000	chr10	52355000	5.36E-03	promoter	super_enhancer	LINC00710

chr10	10995000	chr10	110505000	1.49E-03	promoter	super_enhancer	LINC00710
chr10	11655000	chr10	101055000	2.46E-03	promoter	super_enhancer	USP6NL
chr10	11885000	chr10	14425000	4.83E-02	super_enhancer	promoter	MIR4293
chr10	11885000	chr10	17685000	1.22E-02	super_enhancer	promoter	STAM
chr10	11885000	chr10	24565000	6.73E-03	super_enhancer	promoter	MIR603
chr10	11885000	chr10	27445000	5.95E-03	super_enhancer	promoter	YME1L1
chr10	11885000	chr10	81205000	2.68E-03	super_enhancer	promoter	ZCCHC24
chr10	11895000	chr10	29085000	9.19E-03	super_enhancer	promoter	LINC00837
chr10	11905000	chr10	11965000	2.84E-02	super_enhancer	promoter	RNU6-1095P
chr10	11915000	chr10	11965000	4.39E-02	super_enhancer	promoter	RNU6-1095P
chr10	11915000	chr10	15065000	3.30E-02	super_enhancer	promoter	DCLRE1CP1
chr10	11965000	chr10	71865000	4.61E-03	promoter	super_enhancer	RNU6-1095P
chr10	11965000	chr10	72255000	4.25E-03	promoter	super_enhancer	RNU6-1095P
chr10	12085000	chr10	71975000	3.66E-03	promoter	super_enhancer	UPF2
chr10	13185000	chr10	72315000	3.22E-03	promoter	super_enhancer	RP11-730A19.5
chr10	13185000	chr10	79295000	2.99E-03	promoter	super_enhancer	RP11-730A19.5
chr10	13725000	chr10	33005000	5.52E-03	promoter	super_enhancer	RP11-295P9.8
chr10	14425000	chr10	80455000	3.52E-03	promoter	super_enhancer	MIR4293
chr10	14425000	chr10	93435000	2.40E-03	promoter	super_enhancer	MIR4293
chr10	15065000	chr10	69225000	2.86E-03	promoter	super_enhancer	DCLRE1CP1
chr10	16115000	chr10	71975000	2.99E-03	promoter	super_enhancer	FTLP19
chr10	16115000	chr10	74005000	3.66E-03	promoter	super_enhancer	FTLP19
chr10	16565000	chr10	69225000	3.50E-03	promoter	super_enhancer	C1QL3
chr10	17245000	chr10	73995000	3.30E-03	promoter	super_enhancer	TRDMT1

chr10	17645000	chr10	99785000	2.04E-03	promoter	super_enhancer	AC069542.1
chr10	17685000	chr10	101035000	1.45E-03	promoter	super_enhancer	STAM
chr10	20105000	chr10	80205000	3.47E-03	promoter	super_enhancer	PLXDC2
chr10	21815000	chr10	49555000	4.76E-03	promoter	super_enhancer	SKIDA1
chr10	21815000	chr10	71835000	3.71E-03	promoter	super_enhancer	SKIDA1
chr10	21815000	chr10	72245000	3.05E-03	promoter	super_enhancer	SKIDA1
chr10	21915000	chr10	125035000	2.27E-03	promoter	super_enhancer	HNRNPRP1
chr10	22155000	chr10	79335000	3.74E-03	promoter	super_enhancer	RP11-399C16.3
chr10	22515000	chr10	69235000	2.92E-03	promoter	super_enhancer	RP11-108B14.4
chr10	22515000	chr10	99965000	1.32E-03	promoter	super_enhancer	RP11-108B14.4
chr10	22555000	chr10	52355000	7.74E-03	promoter	super_enhancer	RP11-573G6.2
chr10	22605000	chr10	71765000	3.99E-03	promoter	super_enhancer	COMMD3
chr10	22605000	chr10	122585000	1.94E-03	promoter	super_enhancer	COMMD3
chr10	26505000	chr10	100005000	2.49E-03	promoter	super_enhancer	GAD2
chr10	27445000	chr10	52355000	6.89E-03	promoter	super_enhancer	YME1L1
chr10	27625000	chr10	80205000	2.52E-03	promoter	super_enhancer	RNU6-452P
chr10	28725000	chr10	33005000	2.04E-02	promoter	super_enhancer	RP11-351M16.1
chr10	28725000	chr10	110375000	1.48E-03	promoter	super_enhancer	RP11-351M16.1
chr10	28785000	chr10	101045000	2.49E-03	promoter	super_enhancer	RP11-351M16.3
chr10	28965000	chr10	71845000	6.24E-03	promoter	super_enhancer	BAMBI
chr10	29095000	chr10	74035000	4.87E-03	promoter	super_enhancer	RP11-478H13.4
chr10	29135000	chr10	71845000	5.83E-03	promoter	super_enhancer	C10orf126
chr10	29135000	chr10	80465000	4.33E-03	promoter	super_enhancer	C10orf126
chr10	29835000	chr10	79315000	3.10E-03	promoter	super_enhancer	MIR604

chr10	30405000	chr10	122595000	1.68E-03	promoter	super_enhancer	KIAA1462
chr10	30595000	chr10	33005000	3.09E-02	promoter	super_enhancer	RP11-305E6.4
chr10	30845000	chr10	72295000	6.39E-03	promoter	super_enhancer	RN7SL63P
chr10	30865000	chr10	33005000	4.98E-02	promoter	super_enhancer	RP11-253D19.2
chr10	32345000	chr10	102595000	2.59E-03	promoter	super_enhancer	KIF5B
chr10	32555000	chr10	75215000	3.78E-03	promoter	super_enhancer	RP11-166N17.1
chr10	32635000	chr10	74015000	5.84E-03	promoter	super_enhancer	RP11-135A24.4
chr10	32685000	chr10	72295000	5.58E-03	promoter	super_enhancer	RNU6-1244P
chr10	32685000	chr10	79135000	2.74E-03	promoter	super_enhancer	RNU6-1244P
chr10	33005000	chr10	60475000	5.47E-03	super_enhancer	promoter	FAM133CP
chr10	33005000	chr10	115425000	1.44E-03	super_enhancer	promoter	NRAP
chr10	33295000	chr10	95245000	2.71E-03	promoter	super_enhancer	ITGB1
chr10	33325000	chr10	52355000	1.02E-02	promoter	super_enhancer	RN7SL847P
chr10	33625000	chr10	71995000	4.51E-03	promoter	super_enhancer	NRP1
chr10	34965000	chr10	119655000	1.53E-03	promoter	super_enhancer	RP11-8L18.2
chr10	35105000	chr10	52455000	7.31E-03	promoter	super_enhancer	PARD3
chr10	35215000	chr10	71245000	7.17E-03	promoter	super_enhancer	RNU6-847P
chr10	35215000	chr10	93425000	3.34E-03	promoter	super_enhancer	RNU6-847P
chr10	35535000	chr10	68125000	6.02E-03	promoter	super_enhancer	CCNY
chr10	35625000	chr10	73905000	4.25E-03	promoter	super_enhancer	RP11-324I22.4
chr10	35625000	chr10	75215000	3.76E-03	promoter	super_enhancer	RP11-324I22.4
chr10	35985000	chr10	99775000	2.45E-03	promoter	super_enhancer	RPL7P37
chr10	36725000	chr10	52485000	6.56E-03	promoter	super_enhancer	MTND4P18
chr10	36725000	chr10	73895000	5.30E-03	promoter	super_enhancer	MTND4P18

chr10	37235000	chr10	133265000	1.24E-03	promoter	super_enhancer	MKNK2P1
chr10	37415000	chr10	122105000	1.08E-03	promoter	super_enhancer	ANKRD30A
chr10	37585000	chr10	52485000	6.17E-03	promoter	super_enhancer	RN7SL314P
chr10	38265000	chr10	52335000	8.56E-03	promoter	super_enhancer	RP11-393J16.4
chr10	38265000	chr10	52425000	4.94E-03	promoter	super_enhancer	RP11-393J16.4
chr10	38265000	chr10	110345000	1.27E-03	promoter	super_enhancer	RP11-393J16.4
chr10	38425000	chr10	52365000	7.23E-03	promoter	super_enhancer	RP11-508N22.12
chr10	38425000	chr10	93405000	2.05E-03	promoter	super_enhancer	RP11-508N22.12
chr10	38475000	chr10	68125000	3.65E-03	promoter	super_enhancer	RP11-508N22.10
chr10	42645000	chr10	110355000	3.02E-03	promoter	super_enhancer	KSR1P1
chr10	42705000	chr10	119255000	1.31E-03	promoter	super_enhancer	AL031601.4
chr10	43075000	chr10	52485000	1.48E-02	promoter	super_enhancer	AL022345.7
chr10	43075000	chr10	119305000	3.93E-03	promoter	super_enhancer	AL022345.7
chr10	43135000	chr10	68115000	6.61E-03	promoter	super_enhancer	ZNF33B
chr10	43135000	chr10	68125000	7.89E-03	promoter	super_enhancer	ZNF33B
chr10	43135000	chr10	79165000	9.40E-03	promoter	super_enhancer	ZNF33B
chr10	43135000	chr10	119655000	2.08E-03	promoter	super_enhancer	ZNF33B
chr10	43245000	chr10	88885000	4.98E-03	promoter	super_enhancer	DUXAP3
chr10	43635000	chr10	52435000	1.85E-02	promoter	super_enhancer	RP11-351D16.3
chr10	43635000	chr10	71835000	1.57E-02	promoter	super_enhancer	RP11-351D16.3
chr10	43635000	chr10	72265000	1.28E-02	promoter	super_enhancer	RP11-351D16.3
chr10	43635000	chr10	74035000	1.48E-02	promoter	super_enhancer	RP11-351D16.3
chr10	43635000	chr10	78975000	1.18E-02	promoter	super_enhancer	RP11-351D16.3
chr10	43905000	chr10	102595000	7.62E-03	promoter	super_enhancer	HNRNPF

chr10	43905000	chr10	132525000	3.25E-03	promoter	super_enhancer	HNRNPF
chr10	43915000	chr10	52335000	2.82E-02	promoter	super_enhancer	RP11-517P14.2
chr10	43915000	chr10	71975000	1.18E-02	promoter	super_enhancer	RP11-517P14.2
chr10	44125000	chr10	52325000	2.12E-02	promoter	super_enhancer	RP11-402L1.12
chr10	44125000	chr10	52335000	2.94E-02	promoter	super_enhancer	RP11-402L1.12
chr10	44125000	chr10	52425000	1.70E-02	promoter	super_enhancer	RP11-402L1.12
chr10	44125000	chr10	68105000	1.02E-02	promoter	super_enhancer	RP11-402L1.12
chr10	44145000	chr10	80485000	9.72E-03	promoter	super_enhancer	ZNF32
chr10	44985000	chr10	80205000	6.69E-03	promoter	super_enhancer	RP11-733D4.2
chr10	45455000	chr10	49545000	4.70E-02	promoter	super_enhancer	TMEM72-AS1
chr10	45455000	chr10	61895000	1.06E-02	promoter	super_enhancer	TMEM72-AS1
chr10	45455000	chr10	71845000	1.29E-02	promoter	super_enhancer	TMEM72-AS1
chr10	45475000	chr10	71775000	7.34E-03	promoter	super_enhancer	C10orf10
chr10	45475000	chr10	71985000	9.29E-03	promoter	super_enhancer	C10orf10
chr10	45475000	chr10	79165000	1.16E-02	promoter	super_enhancer	C10orf10
chr10	45475000	chr10	80195000	9.88E-03	promoter	super_enhancer	C10orf10
chr10	45495000	chr10	52335000	2.79E-02	promoter	super_enhancer	ZNF22
chr10	45495000	chr10	52425000	1.61E-02	promoter	super_enhancer	ZNF22
chr10	45675000	chr10	52425000	1.63E-02	promoter	super_enhancer	RP11-432I13.5
chr10	45685000	chr10	52425000	1.18E-02	promoter	super_enhancer	RNU6-1207P
chr10	45695000	chr10	73915000	9.63E-03	promoter	super_enhancer	RP11-432I13.6
chr10	45695000	chr10	79105000	7.86E-03	promoter	super_enhancer	RP11-432I13.6
chr10	45695000	chr10	101065000	4.37E-03	promoter	super_enhancer	RP11-432I13.6
chr10	47645000	chr10	71975000	1.04E-02	promoter	super_enhancer	ANTXRLP1

chr10	47645000	chr10	73935000	1.07E-02	promoter	super_enhancer	ANTXRLP1
chr10	47645000	chr10	74045000	1.16E-02	promoter	super_enhancer	ANTXRLP1
chr10	48355000	chr10	52455000	4.06E-02	promoter	super_enhancer	ZNF488
chr10	48355000	chr10	68125000	1.40E-02	promoter	super_enhancer	ZNF488
chr10	48355000	chr10	72325000	1.96E-02	promoter	super_enhancer	ZNF488
chr10	49515000	chr10	61895000	7.36E-03	promoter	super_enhancer	MAPK8
chr10	49535000	chr10	96075000	4.92E-03	super_enhancer	promoter	RP11-76P2.4
chr10	49535000	chr10	102495000	3.95E-03	super_enhancer	promoter	PAX2
chr10	49535000	chr10	105155000	3.22E-03	super_enhancer	promoter	MIR1307
chr10	49545000	chr10	52385000	2.56E-03	super_enhancer	promoter	SGMS1
chr10	49545000	chr10	93965000	6.53E-03	super_enhancer	promoter	CPEB3_ribozyme
chr10	49545000	chr10	123375000	2.85E-03	super_enhancer	promoter	RP11-62L18.3
chr10	49655000	chr10	52495000	4.79E-02	promoter	super_enhancer	RP11-541M12.6
chr10	49655000	chr10	80145000	9.04E-03	promoter	super_enhancer	RP11-541M12.6
chr10	49655000	chr10	89295000	4.09E-03	promoter	super_enhancer	RP11-541M12.6
chr10	49755000	chr10	89285000	5.34E-03	promoter	super_enhancer	RP11-541M12.3
chr10	49865000	chr10	68105000	1.11E-02	promoter	super_enhancer	ARHGAP22
chr10	49865000	chr10	73925000	1.42E-02	promoter	super_enhancer	ARHGAP22
chr10	49865000	chr10	79085000	9.39E-03	promoter	super_enhancer	ARHGAP22
chr10	50325000	chr10	79085000	1.08E-02	promoter	super_enhancer	VSTM4
chr10	50605000	chr10	52355000	2.54E-02	promoter	super_enhancer	DRGX
chr10	50605000	chr10	52455000	1.03E-02	promoter	super_enhancer	DRGX
chr10	50605000	chr10	68105000	1.54E-02	promoter	super_enhancer	DRGX
chr10	50605000	chr10	69215000	1.69E-02	promoter	super_enhancer	DRGX

chr10	50605000	chr10	74005000	2.12E-02	promoter	super_enhancer	DRGX
chr10	50605000	chr10	79075000	1.23E-02	promoter	super_enhancer	DRGX
chr10	50605000	chr10	79175000	1.82E-02	promoter	super_enhancer	DRGX
chr10	51565000	chr10	71965000	1.21E-02	promoter	super_enhancer	NCOA4
chr10	51565000	chr10	73915000	1.23E-02	promoter	super_enhancer	NCOA4
chr10	52315000	chr10	88855000	6.23E-03	super_enhancer	promoter	GLUD1
chr10	52325000	chr10	62775000	1.44E-02	super_enhancer	promoter	LINC00845
chr10	52325000	chr10	72545000	1.11E-02	super_enhancer	promoter	TBATA
chr10	52335000	chr10	65395000	2.18E-02	super_enhancer	promoter	MRPL35P2
chr10	52335000	chr10	72575000	1.74E-02	super_enhancer	promoter	SGPL1
chr10	52335000	chr10	74885000	1.38E-02	super_enhancer	promoter	RP11-152N13.16
chr10	52345000	chr10	93225000	5.67E-03	super_enhancer	promoter	RP11-108M11.3
chr10	52355000	chr10	104005000	5.75E-03	super_enhancer	promoter	GBF1
chr10	52365000	chr10	56245000	3.27E-02	super_enhancer	promoter	RP11-257I14.1
chr10	52385000	chr10	73935000	1.27E-02	promoter	super_enhancer	SGMS1
chr10	52415000	chr10	71255000	1.66E-02	promoter	super_enhancer	BEND3P1
chr10	52415000	chr10	71875000	1.72E-02	promoter	super_enhancer	BEND3P1
chr10	52425000	chr10	64885000	1.62E-02	super_enhancer	promoter	RP11-144G16.1
chr10	52435000	chr10	71355000	1.02E-02	super_enhancer	promoter	MTND2P15
chr10	52435000	chr10	81965000	6.66E-03	super_enhancer	promoter	ANXA11
chr10	52445000	chr10	53285000	1.92E-02	super_enhancer	promoter	AL157399.1
chr10	52445000	chr10	60145000	1.74E-02	super_enhancer	promoter	TFAM
chr10	52445000	chr10	81345000	7.91E-03	super_enhancer	promoter	MBL3P
chr10	52495000	chr10	69585000	1.16E-02	super_enhancer	promoter	RN7SL394P

chr10	52495000	chr10	75755000	1.14E-02	super_enhancer	promoter	VCL
chr10	52495000	chr10	82105000	7.06E-03	super_enhancer	promoter	DYDC2
chr10	52585000	chr10	80465000	1.26E-02	promoter	super_enhancer	RP11-449O16.2
chr10	52585000	chr10	80485000	1.17E-02	promoter	super_enhancer	RP11-449O16.2
chr10	52645000	chr10	71985000	1.18E-02	promoter	super_enhancer	A1CF
chr10	52645000	chr10	78975000	1.13E-02	promoter	super_enhancer	A1CF
chr10	53025000	chr10	75225000	8.27E-03	promoter	super_enhancer	AC022537.1
chr10	53285000	chr10	68155000	7.92E-03	promoter	super_enhancer	AL157399.1
chr10	53285000	chr10	71855000	9.82E-03	promoter	super_enhancer	AL157399.1
chr10	53285000	chr10	79155000	7.84E-03	promoter	super_enhancer	AL157399.1
chr10	53285000	chr10	133285000	1.31E-03	promoter	super_enhancer	AL157399.1
chr10	54075000	chr10	68155000	1.36E-02	promoter	super_enhancer	PRKG1-AS1
chr10	54515000	chr10	74055000	1.94E-02	promoter	super_enhancer	RP11-556E13.1
chr10	55525000	chr10	79135000	8.23E-03	promoter	super_enhancer	RP11-449J3.3
chr10	55525000	chr10	99995000	4.48E-03	promoter	super_enhancer	RP11-449J3.3
chr10	56245000	chr10	73895000	1.21E-02	promoter	super_enhancer	RP11-257I14.1
chr10	57505000	chr10	71975000	1.27E-02	promoter	super_enhancer	RP11-478B11.2
chr10	58855000	chr10	79155000	8.41E-03	promoter	super_enhancer	AC026393.1
chr10	59065000	chr10	71255000	1.79E-02	promoter	super_enhancer	MIR3924
chr10	60095000	chr10	133305000	1.84E-03	promoter	super_enhancer	UBE2D1
chr10	60145000	chr10	68115000	1.74E-02	promoter	super_enhancer	TFAM
chr10	60145000	chr10	74055000	2.25E-02	promoter	super_enhancer	TFAM
chr10	60455000	chr10	68145000	1.75E-02	promoter	super_enhancer	AC012168.1
chr10	60455000	chr10	73925000	2.04E-02	promoter	super_enhancer	AC012168.1

chr10	60935000	chr10	68125000	2.10E-02	promoter	super_enhancer	PHYHIPL
chr10	60935000	chr10	68145000	1.82E-02	promoter	super_enhancer	PHYHIPL
chr10	60935000	chr10	69055000	1.60E-02	promoter	super_enhancer	PHYHIPL
chr10	60935000	chr10	80205000	1.41E-02	promoter	super_enhancer	PHYHIPL
chr10	61895000	chr10	65395000	4.74E-02	super_enhancer	promoter	MRPL35P2
chr10	62775000	chr10	69205000	2.15E-02	promoter	super_enhancer	LINC00845
chr10	62775000	chr10	73995000	2.20E-02	promoter	super_enhancer	LINC00845
chr10	63445000	chr10	72335000	2.19E-02	promoter	super_enhancer	RP11-63A2.2
chr10	63445000	chr10	80505000	1.20E-02	promoter	super_enhancer	RP11-63A2.2
chr10	63875000	chr10	71875000	2.81E-02	promoter	super_enhancer	AL671972.1
chr10	63875000	chr10	119255000	3.61E-03	promoter	super_enhancer	AL671972.1
chr10	64565000	chr10	68155000	4.29E-02	promoter	super_enhancer	ADO
chr10	64565000	chr10	74005000	2.81E-02	promoter	super_enhancer	ADO
chr10	64565000	chr10	110525000	3.80E-03	promoter	super_enhancer	ADO
chr10	64885000	chr10	71865000	4.75E-02	promoter	super_enhancer	RP11-144G16.1
chr10	64885000	chr10	75225000	3.22E-02	promoter	super_enhancer	RP11-144G16.1
chr10	64885000	chr10	80185000	1.81E-02	promoter	super_enhancer	RP11-144G16.1
chr10	64885000	chr10	100005000	1.26E-02	promoter	super_enhancer	RP11-144G16.1
chr10	65225000	chr10	80495000	1.69E-02	promoter	super_enhancer	JMJD1C-AS1
chr10	65225000	chr10	95235000	9.29E-03	promoter	super_enhancer	JMJD1C-AS1
chr10	65395000	chr10	69205000	4.74E-02	promoter	super_enhancer	MRPL35P2
chr10	65395000	chr10	71985000	3.02E-02	promoter	super_enhancer	MRPL35P2
chr10	66685000	chr10	119685000	3.50E-03	promoter	super_enhancer	RP11-179K3.2
chr10	68085000	chr10	74115000	3.53E-02	super_enhancer	promoter	DNAJB12

chr10	68085000	chr10	81635000	1.08E-02	super_enhancer	promoter	CTSLP6
chr10	68085000	chr10	125535000	3.89E-03	super_enhancer	promoter	RP11-391M7.3
chr10	68085000	chr10	126135000	4.40E-03	super_enhancer	promoter	RP13-238F13.3
chr10	68095000	chr10	68685000	1.02E-02	super_enhancer	promoter	LRRTM3
chr10	68095000	chr10	75635000	1.56E-02	super_enhancer	promoter	CAMK2G
chr10	68095000	chr10	107445000	3.59E-03	super_enhancer	promoter	YWHAZP5
chr10	68105000	chr10	86005000	1.23E-02	super_enhancer	promoter	RGR
chr10	68105000	chr10	102735000	9.32E-03	super_enhancer	promoter	MIR608
chr10	68115000	chr10	85495000	6.64E-03	super_enhancer	promoter	RP11-344L13.1
chr10	68125000	chr10	74885000	2.59E-02	super_enhancer	promoter	RP11-152N13.16
chr10	68125000	chr10	100155000	1.03E-02	super_enhancer	promoter	MIR1287
chr10	68135000	chr10	82105000	1.16E-02	super_enhancer	promoter	DYDC2
chr10	68145000	chr10	74715000	2.45E-02	super_enhancer	promoter	PLA2G12B
chr10	68145000	chr10	80765000	1.62E-02	super_enhancer	promoter	RP11-481G8.2
chr10	68155000	chr10	81785000	1.24E-02	super_enhancer	promoter	C1DP3
chr10	68685000	chr10	69205000	2.05E-02	promoter	super_enhancer	LRRTM3
chr10	69225000	chr10	84625000	1.30E-02	super_enhancer	promoter	AL354749.1
chr10	69235000	chr10	101415000	1.13E-02	super_enhancer	promoter	RP11-483F11.7
chr10	69585000	chr10	79095000	2.04E-02	promoter	super_enhancer	RN7SL394P
chr10	69585000	chr10	80185000	1.65E-02	promoter	super_enhancer	RN7SL394P
chr10	69835000	chr10	79095000	2.27E-02	promoter	super_enhancer	HERC4
chr10	70305000	chr10	71995000	2.68E-02	promoter	super_enhancer	TMEM14D
chr10	70305000	chr10	79305000	3.42E-02	promoter	super_enhancer	TMEM14D
chr10	70305000	chr10	80195000	3.37E-02	promoter	super_enhancer	TMEM14D

chr10	70305000	chr10	80505000	2.58E-02	promoter	super_enhancer	TMEM14D
chr10	70305000	chr10	93415000	1.44E-02	promoter	super_enhancer	TMEM14D
chr10	70305000	chr10	93445000	1.57E-02	promoter	super_enhancer	TMEM14D
chr10	70715000	chr10	93425000	1.46E-02	promoter	super_enhancer	DDX21
chr10	70715000	chr10	113075000	7.72E-03	promoter	super_enhancer	DDX21
chr10	71235000	chr10	75635000	4.24E-02	super_enhancer	promoter	CAMK2G
chr10	71235000	chr10	93965000	1.37E-02	super_enhancer	promoter	CPEB3_ribozyme
chr10	71255000	chr10	75635000	4.79E-02	super_enhancer	promoter	CAMK2G
chr10	71255000	chr10	76585000	3.84E-02	super_enhancer	promoter	KAT6B
chr10	71335000	chr10	71755000	3.73E-03	promoter	super_enhancer	RP11-343J3.2
chr10	71335000	chr10	72295000	3.97E-02	promoter	super_enhancer	RP11-343J3.2
chr10	71335000	chr10	80175000	3.75E-02	promoter	super_enhancer	RP11-343J3.2
chr10	71335000	chr10	80465000	3.53E-02	promoter	super_enhancer	RP11-343J3.2
chr10	71335000	chr10	125035000	7.03E-03	promoter	super_enhancer	RP11-343J3.2
chr10	71355000	chr10	71865000	3.36E-02	promoter	super_enhancer	MTND2P15
chr10	71355000	chr10	75225000	2.28E-03	promoter	super_enhancer	MTND2P15
chr10	71355000	chr10	89265000	1.67E-02	promoter	super_enhancer	MTND2P15
chr10	71445000	chr10	79175000	4.21E-02	promoter	super_enhancer	RP11-242G20.1
chr10	71445000	chr10	86955000	1.46E-02	promoter	super_enhancer	RP11-242G20.1
chr10	71445000	chr10	91605000	1.79E-02	promoter	super_enhancer	RP11-242G20.1
chr10	71445000	chr10	100375000	1.08E-02	promoter	super_enhancer	RP11-242G20.1
chr10	71755000	chr10	75635000	3.46E-02	super_enhancer	promoter	CAMK2G
chr10	71755000	chr10	81345000	1.88E-02	super_enhancer	promoter	MBL3P
chr10	71765000	chr10	81635000	1.52E-02	super_enhancer	promoter	CTSLP6

chr10	71765000	chr10	84625000	1.57E-02	super_enhancer	promoter	AL354749.1
chr10	71765000	chr10	110945000	4.36E-03	super_enhancer	promoter	RNU5B-6P
chr10	71775000	chr10	95255000	8.89E-03	super_enhancer	promoter	CEP55
chr10	71835000	chr10	76585000	4.37E-02	super_enhancer	promoter	KAT6B
chr10	71835000	chr10	102755000	1.26E-02	super_enhancer	promoter	LZTS2
chr10	71845000	chr10	115615000	6.20E-03	super_enhancer	promoter	DCLRE1A
chr10	71855000	chr10	72545000	4.11E-02	super_enhancer	promoter	TBATA
chr10	71855000	chr10	91925000	1.36E-02	super_enhancer	promoter	RN7SKP143
chr10	71855000	chr10	119185000	5.36E-03	super_enhancer	promoter	CTA-109P11.4
chr10	71855000	chr10	122215000	6.35E-03	super_enhancer	promoter	PPAPDC1A
chr10	71875000	chr10	88025000	2.76E-02	super_enhancer	promoter	MIR346
chr10	71975000	chr10	102495000	1.00E-02	super_enhancer	promoter	PAX2
chr10	71985000	chr10	99185000	1.13E-02	super_enhancer	promoter	PGAM1
chr10	71995000	chr10	93655000	1.34E-02	super_enhancer	promoter	RP11-402D21.3
chr10	72005000	chr10	76185000	4.35E-02	super_enhancer	promoter	RAB5CP1
chr10	72005000	chr10	94335000	1.33E-02	super_enhancer	promoter	IDE
chr10	72195000	chr10	72315000	6.81E-03	promoter	super_enhancer	RP11-104F15.9
chr10	72245000	chr10	127465000	4.75E-03	super_enhancer	promoter	MMP21
chr10	72245000	chr10	131285000	2.95E-03	super_enhancer	promoter	AL355531.1
chr10	72255000	chr10	72545000	7.05E-03	super_enhancer	promoter	TBATA
chr10	72255000	chr10	72575000	8.58E-03	super_enhancer	promoter	SGPL1
chr10	72295000	chr10	74825000	7.12E-03	super_enhancer	promoter	RP11-344N10.4
chr10	72295000	chr10	81965000	3.09E-02	super_enhancer	promoter	ANXA11
chr10	72295000	chr10	102755000	1.37E-02	super_enhancer	promoter	LZTS2

chr10	72295000	chr10	126555000	5.91E-03	super_enhancer	promoter	NPM1P31
chr10	72315000	chr10	72555000	1.26E-03	super_enhancer	promoter	RP11-399N22.3
chr10	72325000	chr10	72545000	1.20E-02	super_enhancer	promoter	TBATA
chr10	72325000	chr10	72555000	9.07E-03	super_enhancer	promoter	RP11-399N22.3
chr10	72325000	chr10	74825000	8.64E-03	super_enhancer	promoter	RP11-344N10.4
chr10	72325000	chr10	100175000	2.08E-02	super_enhancer	promoter	PYROXD2
chr10	72335000	chr10	72545000	2.21E-02	super_enhancer	promoter	TBATA
chr10	72335000	chr10	72575000	2.03E-03	super_enhancer	promoter	SGPL1
chr10	72335000	chr10	93975000	1.56E-02	super_enhancer	promoter	NHP2P1
chr10	72545000	chr10	80165000	3.76E-02	promoter	super_enhancer	TBATA
chr10	72555000	chr10	79305000	2.84E-02	promoter	super_enhancer	RP11-399N22.3
chr10	72555000	chr10	79325000	2.72E-02	promoter	super_enhancer	RP11-399N22.3
chr10	72555000	chr10	80485000	2.17E-02	promoter	super_enhancer	RP11-399N22.3
chr10	72575000	chr10	102635000	1.44E-02	promoter	super_enhancer	SGPL1
chr10	72575000	chr10	122115000	4.71E-03	promoter	super_enhancer	SGPL1
chr10	73655000	chr10	74035000	4.60E-03	promoter	super_enhancer	AC073370.1
chr10	73655000	chr10	119665000	6.00E-03	promoter	super_enhancer	AC073370.1
chr10	73815000	chr10	73995000	1.77E-02	promoter	super_enhancer	RP11-150D20.5
chr10	73815000	chr10	74005000	2.41E-02	promoter	super_enhancer	RP11-150D20.5
chr10	73895000	chr10	73975000	1.94E-02	super_enhancer	promoter	ANAPC16
chr10	73895000	chr10	90775000	1.54E-02	super_enhancer	promoter	RP11-399O19.9
chr10	73905000	chr10	75175000	4.99E-02	super_enhancer	promoter	ANXA7
chr10	73905000	chr10	81205000	3.50E-02	super_enhancer	promoter	ZCCHC24
chr10	73915000	chr10	73975000	4.24E-02	super_enhancer	promoter	ANAPC16

chr10	73915000	chr10	74715000	2.68E-02	super_enhancer	promoter	PLA2G12B
chr10	73915000	chr10	123725000	4.84E-03	super_enhancer	promoter	RP11-500G22.5
chr10	73915000	chr10	126555000	5.10E-03	super_enhancer	promoter	NPM1P31
chr10	73925000	chr10	99095000	1.69E-02	super_enhancer	promoter	FRAT2
chr10	73935000	chr10	97455000	1.21E-02	super_enhancer	promoter	TCTN3
chr10	73935000	chr10	102935000	1.15E-02	super_enhancer	promoter	LINC01514
chr10	73935000	chr10	115615000	5.93E-03	super_enhancer	promoter	DCLRE1A
chr10	73935000	chr10	123375000	5.49E-03	super_enhancer	promoter	RP11-62L18.3
chr10	73975000	chr10	93475000	1.63E-02	promoter	super_enhancer	ANAPC16
chr10	74005000	chr10	80765000	4.44E-02	super_enhancer	promoter	RP11-481G8.2
chr10	74005000	chr10	81345000	4.09E-02	super_enhancer	promoter	MBL3P
chr10	74005000	chr10	102735000	1.96E-02	super_enhancer	promoter	MIR608
chr10	74005000	chr10	135475000	2.75E-03	super_enhancer	promoter	CLUHP5
chr10	74015000	chr10	74825000	3.01E-02	super_enhancer	promoter	RP11-344N10.4
chr10	74015000	chr10	93975000	1.83E-02	super_enhancer	promoter	NHP2P1
chr10	74015000	chr10	105615000	1.10E-02	super_enhancer	promoter	SH3PXD2A
chr10	74035000	chr10	75425000	3.23E-02	super_enhancer	promoter	SYNPO2L
chr10	74045000	chr10	124715000	4.33E-03	super_enhancer	promoter	C10orf88
chr10	74055000	chr10	74825000	3.29E-02	super_enhancer	promoter	RP11-344N10.4
chr10	74055000	chr10	104195000	1.46E-02	super_enhancer	promoter	MIR146B
chr10	74115000	chr10	79095000	4.49E-02	promoter	super_enhancer	DNAJB12
chr10	74515000	chr10	80485000	3.70E-02	promoter	super_enhancer	RP11-354E23.4
chr10	74525000	chr10	133255000	3.92E-03	promoter	super_enhancer	RP11-354E23.5
chr10	74715000	chr10	79075000	4.68E-02	promoter	super_enhancer	PLA2G12B

chr10	74885000	chr10	80455000	4.15E-02	promoter	super_enhancer	RP11-152N13.16
chr10	74885000	chr10	93465000	1.43E-02	promoter	super_enhancer	RP11-152N13.16
chr10	75175000	chr10	91605000	1.98E-02	promoter	super_enhancer	ANXA7
chr10	75215000	chr10	95255000	1.36E-02	super_enhancer	promoter	CEP55
chr10	75215000	chr10	100175000	1.58E-02	super_enhancer	promoter	PYROXD2
chr10	75225000	chr10	104605000	8.94E-03	super_enhancer	promoter	PFN1P11
chr10	75225000	chr10	105155000	9.45E-03	super_enhancer	promoter	MIR1307
chr10	75255000	chr10	102635000	1.36E-02	promoter	super_enhancer	PPP3CB-AS1
chr10	75465000	chr10	79135000	3.55E-02	promoter	super_enhancer	RNA5SP320
chr10	75465000	chr10	80165000	4.59E-02	promoter	super_enhancer	RNA5SP320
chr10	75635000	chr10	79145000	4.66E-02	promoter	super_enhancer	CAMK2G
chr10	75635000	chr10	95245000	1.23E-02	promoter	super_enhancer	CAMK2G
chr10	75755000	chr10	79295000	8.19E-05	promoter	super_enhancer	VCL
chr10	75755000	chr10	93435000	1.82E-02	promoter	super_enhancer	VCL
chr10	75765000	chr10	93385000	2.01E-02	promoter	super_enhancer	RP11-417O11.5
chr10	76105000	chr10	79315000	4.43E-03	promoter	super_enhancer	TIMM9P1
chr10	76105000	chr10	93435000	1.56E-02	promoter	super_enhancer	TIMM9P1
chr10	76185000	chr10	80485000	4.79E-02	promoter	super_enhancer	RAB5CP1
chr10	76185000	chr10	101045000	1.11E-02	promoter	super_enhancer	RAB5CP1
chr10	76185000	chr10	113085000	4.92E-03	promoter	super_enhancer	RAB5CP1
chr10	76415000	chr10	79345000	5.43E-03	promoter	super_enhancer	POLR3DP1
chr10	76415000	chr10	119265000	8.05E-03	promoter	super_enhancer	POLR3DP1
chr10	76505000	chr10	95235000	1.81E-02	promoter	super_enhancer	RP11-383B24.3
chr10	76505000	chr10	99775000	1.71E-02	promoter	super_enhancer	RP11-383B24.3

chr10	76505000	chr10	99965000	1.34E-02	promoter	super_enhancer	RP11-383B24.3
chr10	76785000	chr10	102605000	1.46E-02	promoter	super_enhancer	RP11-77G23.2
chr10	76785000	chr10	113075000	9.06E-03	promoter	super_enhancer	RP11-77G23.2
chr10	76885000	chr10	79325000	3.57E-04	promoter	super_enhancer	RP11-487I5.4
chr10	76885000	chr10	93445000	1.78E-02	promoter	super_enhancer	RP11-487I5.4
chr10	76945000	chr10	113065000	6.42E-03	promoter	super_enhancer	AL392111.1
chr10	78965000	chr10	85435000	2.57E-02	super_enhancer	promoter	RP11-344L13.2
chr10	78965000	chr10	86005000	2.85E-02	super_enhancer	promoter	RGR
chr10	78965000	chr10	123735000	5.72E-03	super_enhancer	promoter	NSMCE4A
chr10	78975000	chr10	93965000	1.99E-02	super_enhancer	promoter	CPEB3_ribozyme
chr10	79065000	chr10	98275000	1.40E-02	super_enhancer	promoter	TLL2
chr10	79075000	chr10	86005000	2.80E-02	super_enhancer	promoter	RGR
chr10	79075000	chr10	90635000	1.55E-02	super_enhancer	promoter	PTCD2P2
chr10	79075000	chr10	93225000	1.43E-02	super_enhancer	promoter	RP11-108M11.3
chr10	79085000	chr10	81785000	2.93E-03	super_enhancer	promoter	C1DP3
chr10	79085000	chr10	85075000	1.88E-02	super_enhancer	promoter	MARK2P15
chr10	79105000	chr10	100175000	1.95E-02	super_enhancer	promoter	PYROXD2
chr10	79115000	chr10	101905000	1.74E-02	super_enhancer	promoter	RP11-316M21.9
chr10	79125000	chr10	119185000	6.79E-03	super_enhancer	promoter	CTA-109P11.4
chr10	79135000	chr10	90635000	1.29E-02	super_enhancer	promoter	PTCD2P2
chr10	79135000	chr10	110945000	4.01E-03	super_enhancer	promoter	RNU5B-6P
chr10	79155000	chr10	100175000	2.21E-02	super_enhancer	promoter	PYROXD2
chr10	79155000	chr10	102495000	1.44E-02	super_enhancer	promoter	PAX2
chr10	79165000	chr10	104195000	1.92E-02	super_enhancer	promoter	MIR146B

chr10	79175000	chr10	98275000	1.70E-02	super_enhancer	promoter	TLL2
chr10	79175000	chr10	99185000	2.17E-02	super_enhancer	promoter	PGAM1
chr10	79185000	chr10	93975000	1.52E-02	super_enhancer	promoter	NHP2P1
chr10	79195000	chr10	90635000	1.94E-02	super_enhancer	promoter	PTCD2P2
chr10	79195000	chr10	102495000	1.41E-02	super_enhancer	promoter	PAX2
chr10	79195000	chr10	129705000	5.80E-03	super_enhancer	promoter	PTPRE
chr10	79295000	chr10	84625000	3.07E-02	super_enhancer	promoter	AL354749.1
chr10	79295000	chr10	120515000	6.56E-03	super_enhancer	promoter	CACUL1
chr10	79305000	chr10	86005000	3.83E-02	super_enhancer	promoter	RGR
chr10	79305000	chr10	99205000	1.95E-02	super_enhancer	promoter	ZDHHC16
chr10	79315000	chr10	100175000	2.43E-02	super_enhancer	promoter	PYROXD2
chr10	79315000	chr10	102735000	2.26E-02	super_enhancer	promoter	MIR608
chr10	79325000	chr10	85985000	2.69E-02	super_enhancer	promoter	LRIT2
chr10	79325000	chr10	88025000	3.66E-02	super_enhancer	promoter	MIR346
chr10	79325000	chr10	88515000	3.20E-02	super_enhancer	promoter	BMPR1A
chr10	79325000	chr10	93655000	1.98E-02	super_enhancer	promoter	RP11-402D21.3
chr10	79335000	chr10	89185000	1.85E-02	super_enhancer	promoter	LINC00864
chr10	79345000	chr10	121485000	5.95E-03	super_enhancer	promoter	INPP5F
chr10	80145000	chr10	127465000	7.04E-03	super_enhancer	promoter	MMP21
chr10	80155000	chr10	88855000	2.51E-02	super_enhancer	promoter	GLUD1
chr10	80155000	chr10	89705000	2.42E-02	super_enhancer	promoter	RP11-380G5.3
chr10	80155000	chr10	95045000	1.36E-02	super_enhancer	promoter	RP11-624L12.1
chr10	80165000	chr10	80765000	2.86E-02	super_enhancer	promoter	RP11-481G8.2
chr10	80165000	chr10	93655000	2.37E-02	super_enhancer	promoter	RP11-402D21.3

chr10	80165000	chr10	112865000	1.07E-02	super_enhancer	promoter	RP11-479A21.1
chr10	80195000	chr10	80765000	2.74E-02	super_enhancer	promoter	RP11-481G8.2
chr10	80195000	chr10	81965000	2.03E-03	super_enhancer	promoter	ANXA11
chr10	80195000	chr10	88915000	2.36E-02	super_enhancer	promoter	RN7SL733P
chr10	80205000	chr10	86005000	4.03E-02	super_enhancer	promoter	RGR
chr10	80205000	chr10	88515000	3.25E-02	super_enhancer	promoter	BMPR1A
chr10	80205000	chr10	116185000	9.06E-03	super_enhancer	promoter	RN7SL384P
chr10	80455000	chr10	100155000	2.10E-02	super_enhancer	promoter	MIR1287
chr10	80465000	chr10	84625000	4.58E-02	super_enhancer	promoter	AL354749.1
chr10	80465000	chr10	88855000	2.75E-02	super_enhancer	promoter	GLUD1
chr10	80465000	chr10	95255000	2.04E-02	super_enhancer	promoter	CEP55
chr10	80465000	chr10	100775000	1.13E-02	super_enhancer	promoter	ARL5AP2
chr10	80475000	chr10	80765000	2.32E-02	super_enhancer	promoter	RP11-481G8.2
chr10	80475000	chr10	88515000	3.10E-02	super_enhancer	promoter	BMPR1A
chr10	80485000	chr10	95255000	1.90E-02	super_enhancer	promoter	CEP55
chr10	80485000	chr10	101415000	1.63E-02	super_enhancer	promoter	RP11-483F11.7
chr10	80485000	chr10	104925000	1.09E-02	super_enhancer	promoter	AL360001.1
chr10	80495000	chr10	93965000	2.10E-02	super_enhancer	promoter	CPEB3_ribozyme
chr10	80505000	chr10	88515000	2.97E-02	super_enhancer	promoter	BMPR1A
chr10	80505000	chr10	88855000	2.34E-02	super_enhancer	promoter	GLUD1
chr10	80505000	chr10	100155000	1.83E-02	super_enhancer	promoter	MIR1287
chr10	81205000	chr10	89275000	2.89E-02	promoter	super_enhancer	ZCCHC24
chr10	81345000	chr10	101065000	1.64E-02	promoter	super_enhancer	MBL3P
chr10	81355000	chr10	93395000	1.88E-02	promoter	super_enhancer	SFTPA3P

chr10	81445000	chr10	89285000	1.24E-02	promoter	super_enhancer	RP11-119F19.5
chr10	81445000	chr10	91595000	1.15E-02	promoter	super_enhancer	RP11-119F19.5
chr10	81635000	chr10	133285000	2.50E-03	promoter	super_enhancer	CTSLP6
chr10	81965000	chr10	125095000	6.14E-03	promoter	super_enhancer	ANXA11
chr10	82105000	chr10	88885000	1.93E-02	promoter	super_enhancer	DYDC2
chr10	82105000	chr10	102595000	1.73E-02	promoter	super_enhancer	DYDC2
chr10	83635000	chr10	93475000	2.36E-02	promoter	super_enhancer	NRG3
chr10	83635000	chr10	132515000	4.71E-03	promoter	super_enhancer	NRG3
chr10	85435000	chr10	122595000	5.33E-03	promoter	super_enhancer	RP11-344L13.2
chr10	85495000	chr10	89285000	2.48E-02	promoter	super_enhancer	RP11-344L13.1
chr10	86005000	chr10	110375000	8.54E-03	promoter	super_enhancer	RGR
chr10	86955000	chr10	120515000	5.79E-03	super_enhancer	promoter	CACUL1
chr10	88025000	chr10	99985000	1.83E-02	promoter	super_enhancer	MIR346
chr10	88025000	chr10	110425000	1.33E-02	promoter	super_enhancer	MIR346
chr10	88855000	chr10	93395000	4.28E-02	promoter	super_enhancer	GLUD1
chr10	88885000	chr10	96075000	2.05E-02	super_enhancer	promoter	RP11-76P2.4
chr10	88885000	chr10	119805000	4.11E-03	super_enhancer	promoter	CASC2
chr10	88895000	chr10	104005000	1.29E-02	super_enhancer	promoter	GBF1
chr10	89185000	chr10	89285000	7.72E-03	promoter	super_enhancer	LINC00864
chr10	89185000	chr10	102615000	1.45E-02	promoter	super_enhancer	LINC00864
chr10	89265000	chr10	93965000	4.29E-02	super_enhancer	promoter	CPEB3_ribozyme
chr10	89265000	chr10	116445000	7.00E-03	super_enhancer	promoter	ABLIM1
chr10	89275000	chr10	91025000	1.29E-02	super_enhancer	promoter	AL353751.1
chr10	89285000	chr10	95045000	1.84E-02	super_enhancer	promoter	RP11-624L12.1

chr10	89295000	chr10	91215000	5.85E-03	super_enhancer	promoter	SLC16A12-AS1
chr10	89295000	chr10	93305000	4.27E-02	super_enhancer	promoter	RPS27P1
chr10	89295000	chr10	95735000	2.28E-02	super_enhancer	promoter	AL365510.1
chr10	89295000	chr10	112865000	7.86E-03	super_enhancer	promoter	RP11-479A21.1
chr10	89705000	chr10	110395000	9.00E-03	promoter	super_enhancer	RP11-380G5.3
chr10	90345000	chr10	101045000	1.72E-02	promoter	super_enhancer	RNLS
chr10	90635000	chr10	104335000	1.39E-02	promoter	super_enhancer	PTCD2P2
chr10	90775000	chr10	95235000	4.32E-02	promoter	super_enhancer	RP11-399O19.9
chr10	90775000	chr10	102615000	2.18E-02	promoter	super_enhancer	RP11-399O19.9
chr10	90775000	chr10	119305000	1.08E-02	promoter	super_enhancer	RP11-399O19.9
chr10	91025000	chr10	102615000	2.44E-02	promoter	super_enhancer	AL353751.1
chr10	91595000	chr10	102495000	1.71E-02	super_enhancer	promoter	PAX2
chr10	91595000	chr10	103845000	1.51E-02	super_enhancer	promoter	RP11-302K17.3
chr10	91605000	chr10	99205000	3.28E-02	super_enhancer	promoter	ZDHHC16
chr10	91615000	chr10	104475000	1.74E-02	super_enhancer	promoter	ARL3
chr10	93225000	chr10	101035000	1.78E-02	promoter	super_enhancer	RP11-108M11.3
chr10	93305000	chr10	93435000	3.46E-02	promoter	super_enhancer	RPS27P1
chr10	93385000	chr10	93655000	7.19E-04	super_enhancer	promoter	RP11-402D21.3
chr10	93385000	chr10	97825000	4.42E-02	super_enhancer	promoter	MIR3157
chr10	93385000	chr10	126135000	1.03E-02	super_enhancer	promoter	RP13-238F13.3
chr10	93395000	chr10	105995000	1.51E-02	super_enhancer	promoter	GSTO1
chr10	93395000	chr10	112865000	1.24E-02	super_enhancer	promoter	RP11-479A21.1
chr10	93415000	chr10	102745000	2.89E-02	super_enhancer	promoter	RP11-108L7.4
chr10	93425000	chr10	101905000	2.72E-02	super_enhancer	promoter	RP11-316M21.9

chr10	93425000	chr10	105735000	1.70E-02	super_enhancer	promoter	RN7SL524P
chr10	93445000	chr10	99095000	4.46E-02	super_enhancer	promoter	FRAT2
chr10	93445000	chr10	100175000	4.38E-02	super_enhancer	promoter	PYROXD2
chr10	93445000	chr10	105615000	1.85E-02	super_enhancer	promoter	SH3PXD2A
chr10	93465000	chr10	102495000	2.19E-02	super_enhancer	promoter	PAX2
chr10	93475000	chr10	93655000	4.82E-02	super_enhancer	promoter	RP11-402D21.3
chr10	93475000	chr10	100155000	4.36E-02	super_enhancer	promoter	MIR1287
chr10	93475000	chr10	101905000	3.23E-02	super_enhancer	promoter	RP11-316M21.9
chr10	93475000	chr10	102495000	2.60E-02	super_enhancer	promoter	PAX2
chr10	93475000	chr10	104195000	2.72E-02	super_enhancer	promoter	MIR146B
chr10	93965000	chr10	99785000	3.38E-02	promoter	super_enhancer	CPEB3_ribozyme
chr10	93965000	chr10	102615000	2.99E-02	promoter	super_enhancer	CPEB3_ribozyme
chr10	93975000	chr10	122595000	7.82E-03	promoter	super_enhancer	NHP2P1
chr10	94335000	chr10	102645000	2.80E-02	promoter	super_enhancer	IDE
chr10	95045000	chr10	99775000	3.68E-02	promoter	super_enhancer	RP11-624L12.1
chr10	95235000	chr10	103455000	1.97E-02	super_enhancer	promoter	FBXW4
chr10	95235000	chr10	116165000	1.54E-02	super_enhancer	promoter	AFAP1L2
chr10	95245000	chr10	95255000	4.65E-02	super_enhancer	promoter	CEP55
chr10	95735000	chr10	100005000	1.47E-03	promoter	super_enhancer	AL365510.1
chr10	96535000	chr10	110505000	9.06E-03	promoter	super_enhancer	MTND4P19
chr10	96535000	chr10	133015000	7.44E-03	promoter	super_enhancer	MTND4P19
chr10	97715000	chr10	112865000	1.15E-02	super_enhancer	promoter	RP11-479A21.1
chr10	97715000	chr10	116005000	9.23E-03	super_enhancer	promoter	CTB-1144G6.5
chr10	97715000	chr10	126555000	7.00E-03	super_enhancer	promoter	NPM1P31

chr10	99095000	chr10	125095000	1.16E-02	promoter	super_enhancer	FRAT2
chr10	99205000	chr10	132445000	6.77E-03	promoter	super_enhancer	ZDHHC16
chr10	99775000	chr10	105505000	4.56E-02	super_enhancer	promoter	SH3PXD2A-AS1
chr10	99775000	chr10	119135000	1.53E-02	super_enhancer	promoter	PDZD8
chr10	99785000	chr10	105615000	2.94E-02	super_enhancer	promoter	SH3PXD2A
chr10	99955000	chr10	104615000	2.72E-02	super_enhancer	promoter	RP11-753C18.12
chr10	99955000	chr10	116185000	1.03E-02	super_enhancer	promoter	RN7SL384P
chr10	99965000	chr10	123375000	9.88E-03	super_enhancer	promoter	RP11-62L18.3
chr10	99975000	chr10	124715000	6.37E-03	super_enhancer	promoter	C10orf88
chr10	99985000	chr10	100775000	3.08E-02	super_enhancer	promoter	ARL5AP2
chr10	99985000	chr10	123735000	7.99E-03	super_enhancer	promoter	NSMCE4A
chr10	99995000	chr10	116445000	8.47E-03	super_enhancer	promoter	ABLIM1
chr10	100005000	chr10	100775000	1.08E-02	super_enhancer	promoter	ARL5AP2
chr10	100155000	chr10	110445000	1.83E-02	promoter	super_enhancer	MIR1287
chr10	100155000	chr10	119265000	1.91E-02	promoter	super_enhancer	MIR1287
chr10	100155000	chr10	132445000	8.04E-03	promoter	super_enhancer	MIR1287
chr10	100155000	chr10	132535000	1.20E-02	promoter	super_enhancer	MIR1287
chr10	100175000	chr10	101065000	7.58E-04	promoter	super_enhancer	PYROXD2
chr10	100175000	chr10	122105000	1.28E-02	promoter	super_enhancer	PYROXD2
chr10	100775000	chr10	119305000	1.24E-02	promoter	super_enhancer	ARL5AP2
chr10	101065000	chr10	133785000	7.71E-03	super_enhancer	promoter	RP11-45A17.2
chr10	101875000	chr10	102615000	4.50E-02	promoter	super_enhancer	TPM4P1
chr10	102495000	chr10	102615000	2.70E-02	promoter	super_enhancer	PAX2
chr10	102495000	chr10	102635000	1.66E-03	promoter	super_enhancer	PAX2

chr10	102595000	chr10	116005000	2.01E-02	super_enhancer	promoter	CTB-1144G6.5
chr10	102595000	chr10	126685000	1.84E-02	super_enhancer	promoter	RP11-59C5.3
chr10	102595000	chr10	128995000	1.34E-02	super_enhancer	promoter	FAM196A
chr10	102605000	chr10	126135000	1.41E-02	super_enhancer	promoter	RP13-238F13.3
chr10	102625000	chr10	124175000	9.98E-03	super_enhancer	promoter	MIR3941
chr10	102625000	chr10	125765000	1.53E-02	super_enhancer	promoter	RP11-47G11.2
chr10	102645000	chr10	134145000	8.92E-03	super_enhancer	promoter	STK32C
chr10	102735000	chr10	119275000	2.14E-02	promoter	super_enhancer	MIR608
chr10	102735000	chr10	122125000	1.75E-02	promoter	super_enhancer	MIR608
chr10	102735000	chr10	125055000	1.41E-02	promoter	super_enhancer	MIR608
chr10	102755000	chr10	113075000	2.19E-02	promoter	super_enhancer	LZTS2
chr10	102935000	chr10	125045000	1.00E-02	promoter	super_enhancer	LINC01514
chr10	103125000	chr10	110445000	1.48E-02	promoter	super_enhancer	RNU2-59P
chr10	103125000	chr10	122585000	1.09E-02	promoter	super_enhancer	RNU2-59P
chr10	103455000	chr10	133305000	4.91E-03	promoter	super_enhancer	FBXW4
chr10	103825000	chr10	119665000	1.35E-02	promoter	super_enhancer	HPS6
chr10	103845000	chr10	122575000	9.27E-03	promoter	super_enhancer	RP11-302K17.3
chr10	103985000	chr10	119665000	1.59E-02	promoter	super_enhancer	ELOVL3
chr10	104335000	chr10	114185000	1.86E-02	super_enhancer	promoter	RP11-324O2.3
chr10	104335000	chr10	134145000	8.32E-03	super_enhancer	promoter	STK32C
chr10	104605000	chr10	119655000	1.13E-02	promoter	super_enhancer	PFN1P11
chr10	104615000	chr10	133305000	6.82E-03	promoter	super_enhancer	RP11-753C18.12
chr10	104935000	chr10	125055000	8.46E-03	promoter	super_enhancer	MARCKSL1P1
chr10	104975000	chr10	122115000	9.79E-03	promoter	super_enhancer	ST13P13

chr10	105155000	chr10	113075000	2.05E-02	promoter	super_enhancer	MIR1307
chr10	105505000	chr10	110375000	3.11E-02	promoter	super_enhancer	SH3PXD2A-AS1
chr10	105505000	chr10	110385000	3.24E-02	promoter	super_enhancer	SH3PXD2A-AS1
chr10	105615000	chr10	110355000	2.38E-02	promoter	super_enhancer	SH3PXD2A
chr10	105735000	chr10	125085000	1.07E-02	promoter	super_enhancer	RN7SL524P
chr10	105995000	chr10	110365000	2.54E-02	promoter	super_enhancer	GSTO1
chr10	105995000	chr10	110415000	2.29E-02	promoter	super_enhancer	GSTO1
chr10	105995000	chr10	125085000	9.24E-03	promoter	super_enhancer	GSTO1
chr10	105995000	chr10	133305000	5.65E-03	promoter	super_enhancer	GSTO1
chr10	106375000	chr10	110375000	3.13E-02	promoter	super_enhancer	RP11-127O4.2
chr10	106375000	chr10	110405000	3.40E-02	promoter	super_enhancer	RP11-127O4.2
chr10	106375000	chr10	110445000	2.89E-02	promoter	super_enhancer	RP11-127O4.2
chr10	106375000	chr10	133015000	1.14E-02	promoter	super_enhancer	RP11-127O4.2
chr10	107445000	chr10	110365000	3.97E-02	promoter	super_enhancer	YWHAZP5
chr10	108925000	chr10	110515000	7.36E-03	promoter	super_enhancer	SORCS1
chr10	110055000	chr10	110405000	1.91E-02	promoter	super_enhancer	PTGES3P5
chr10	110055000	chr10	119295000	1.06E-04	promoter	super_enhancer	PTGES3P5
chr10	110055000	chr10	125115000	9.42E-03	promoter	super_enhancer	PTGES3P5
chr10	110365000	chr10	116445000	1.50E-02	super_enhancer	promoter	ABLIM1
chr10	110365000	chr10	128825000	7.01E-03	super_enhancer	promoter	RP11-223P11.3
chr10	110405000	chr10	113975000	4.34E-02	super_enhancer	promoter	GPAM
chr10	110415000	chr10	120765000	1.42E-02	super_enhancer	promoter	AL157788.1
chr10	110425000	chr10	123495000	1.29E-02	super_enhancer	promoter	RP11-78A18.2
chr10	110425000	chr10	124895000	1.54E-02	super_enhancer	promoter	HMX3

chr10	110425000	chr10	126555000	1.19E-02	super_enhancer	promoter	NPM1P31
chr10	110435000	chr10	110465000	8.95E-03	super_enhancer	promoter	AL356476.1
chr10	110455000	chr10	115935000	1.39E-02	super_enhancer	promoter	CCDC186
chr10	110465000	chr10	110495000	4.12E-02	promoter	super_enhancer	AL356476.1
chr10	110485000	chr10	110945000	2.53E-02	super_enhancer	promoter	RNU5B-6P
chr10	110495000	chr10	119495000	1.07E-02	super_enhancer	promoter	RP11-328K15.1
chr10	110505000	chr10	111755000	1.07E-02	super_enhancer	promoter	ADD3
chr10	110505000	chr10	113975000	3.85E-02	super_enhancer	promoter	GPAM
chr10	110505000	chr10	115425000	1.97E-02	super_enhancer	promoter	NRAP
chr10	110525000	chr10	116005000	1.95E-02	super_enhancer	promoter	CTB-1144G6.5
chr10	110535000	chr10	129535000	1.00E-02	super_enhancer	promoter	RP11-288A5.2
chr10	110945000	chr10	113085000	4.89E-02	promoter	super_enhancer	RNU5B-6P
chr10	111755000	chr10	113085000	9.82E-03	promoter	super_enhancer	ADD3
chr10	111755000	chr10	119315000	1.75E-02	promoter	super_enhancer	ADD3
chr10	111755000	chr10	133285000	7.36E-03	promoter	super_enhancer	ADD3
chr10	112865000	chr10	125135000	1.30E-02	promoter	super_enhancer	RP11-479A21.1
chr10	112865000	chr10	132545000	1.13E-02	promoter	super_enhancer	RP11-479A21.1
chr10	113065000	chr10	125425000	1.02E-02	super_enhancer	promoter	GPR26
chr10	113065000	chr10	135475000	4.09E-03	super_enhancer	promoter	CLUHP5
chr10	113075000	chr10	123495000	1.69E-02	super_enhancer	promoter	RP11-78A18.2
chr10	113975000	chr10	125035000	2.07E-02	promoter	super_enhancer	GPAM
chr10	113975000	chr10	125085000	1.61E-02	promoter	super_enhancer	GPAM
chr10	113975000	chr10	132545000	1.29E-02	promoter	super_enhancer	GPAM
chr10	114185000	chr10	122575000	1.50E-02	promoter	super_enhancer	RP11-324O2.3

chr10	115935000	chr10	119655000	2.91E-02	promoter	super_enhancer	CCDC186
chr10	116005000	chr10	122105000	1.87E-02	promoter	super_enhancer	CTB-1144G6.5
chr10	116165000	chr10	133255000	1.36E-02	promoter	super_enhancer	AFAP1L2
chr10	116185000	chr10	122595000	2.40E-02	promoter	super_enhancer	RN7SL384P
chr10	116445000	chr10	119655000	3.79E-02	promoter	super_enhancer	ABLIM1
chr10	118305000	chr10	122145000	3.78E-02	promoter	super_enhancer	PNLIP
chr10	119185000	chr10	119265000	3.38E-02	promoter	super_enhancer	CTA-109P11.4
chr10	119185000	chr10	133295000	1.48E-02	promoter	super_enhancer	CTA-109P11.4
chr10	119255000	chr10	125425000	2.14E-02	super_enhancer	promoter	GPR26
chr10	119255000	chr10	125765000	2.60E-02	super_enhancer	promoter	RP11-47G11.2
chr10	119265000	chr10	119805000	1.55E-02	super_enhancer	promoter	CASC2
chr10	119265000	chr10	124515000	2.71E-02	super_enhancer	promoter	DMBT1P1
chr10	119265000	chr10	126135000	3.16E-02	super_enhancer	promoter	RP13-238F13.3
chr10	119275000	chr10	120355000	4.97E-03	super_enhancer	promoter	PRLHR
chr10	119285000	chr10	129535000	1.61E-02	super_enhancer	promoter	RP11-288A5.2
chr10	119305000	chr10	119805000	4.44E-03	super_enhancer	promoter	CASC2
chr10	119315000	chr10	120355000	4.08E-02	super_enhancer	promoter	PRLHR
chr10	119315000	chr10	124895000	3.20E-02	super_enhancer	promoter	HMX3
chr10	119315000	chr10	126135000	2.61E-02	super_enhancer	promoter	RP13-238F13.3
chr10	119315000	chr10	132795000	1.08E-02	super_enhancer	promoter	AL607076.1
chr10	119325000	chr10	126135000	2.55E-02	super_enhancer	promoter	RP13-238F13.3
chr10	119495000	chr10	122575000	4.17E-02	promoter	super_enhancer	RP11-328K15.1
chr10	119655000	chr10	126855000	2.40E-02	super_enhancer	promoter	MRPS21P6
chr10	119665000	chr10	128995000	1.82E-02	super_enhancer	promoter	FAM196A

chr10	119805000	chr10	133305000	7.80E-03	promoter	super_enhancer	CASC2
chr10	122105000	chr10	126685000	3.60E-02	super_enhancer	promoter	RP11-59C5.3
chr10	122105000	chr10	131265000	1.17E-02	super_enhancer	promoter	MGMT
chr10	122135000	chr10	132795000	1.37E-02	super_enhancer	promoter	AL607076.1
chr10	122575000	chr10	132085000	1.18E-02	super_enhancer	promoter	RP11-339B9.1
chr10	122585000	chr10	131935000	1.76E-02	super_enhancer	promoter	GLRX3
chr10	122595000	chr10	126855000	3.97E-02	super_enhancer	promoter	MRPS21P6
chr10	123375000	chr10	133275000	1.63E-02	promoter	super_enhancer	RP11-62L18.3
chr10	123495000	chr10	133265000	1.47E-02	promoter	super_enhancer	RP11-78A18.2
chr10	123725000	chr10	132525000	1.57E-02	promoter	super_enhancer	RP11-500G22.5
chr10	124175000	chr10	125145000	2.10E-02	promoter	super_enhancer	MIR3941
chr10	124895000	chr10	125055000	6.89E-03	promoter	super_enhancer	HMX3
chr10	124895000	chr10	125145000	5.63E-04	promoter	super_enhancer	HMX3
chr10	125035000	chr10	126135000	8.52E-03	super_enhancer	promoter	RP13-238F13.3
chr10	125045000	chr10	127835000	4.70E-02	super_enhancer	promoter	RNA5SP328
chr10	125105000	chr10	126135000	3.12E-02	super_enhancer	promoter	RP13-238F13.3
chr10	125145000	chr10	131265000	1.98E-02	super_enhancer	promoter	MGMT
chr10	125425000	chr10	132535000	2.19E-02	promoter	super_enhancer	GPR26
chr10	125535000	chr10	132535000	2.71E-02	promoter	super_enhancer	RP11-391M7.3
chr10	125765000	chr10	133015000	3.26E-02	promoter	super_enhancer	RP11-47G11.2
chr10	125765000	chr10	133255000	2.16E-02	promoter	super_enhancer	RP11-47G11.2
chr10	126605000	chr10	133305000	1.66E-02	promoter	super_enhancer	RP11-298J20.4
chr10	126855000	chr10	132535000	3.75E-02	promoter	super_enhancer	MRPS21P6
chr10	127465000	chr10	132535000	3.80E-02	promoter	super_enhancer	MMP21

chr10	129535000	chr10	133275000	4.32E-02	promoter	super_enhancer	RP11-288A5.2
chr10	132085000	chr10	132515000	4.96E-03	promoter	super_enhancer	RP11-339B9.1
chr10	132515000	chr10	134255000	1.17E-02	super_enhancer	promoter	RP11-432J24.3
chr10	132535000	chr10	132795000	2.09E-02	super_enhancer	promoter	AL607076.1
chr11	305000	chr11	355000	2.93E-02	promoter	super_enhancer	RP11-326C3.4
chr11	345000	chr11	8985000	3.21E-02	super_enhancer	promoter	TMEM9B-AS1
chr11	345000	chr11	12285000	2.87E-02	super_enhancer	promoter	RP11-265D17.2
chr11	345000	chr11	75625000	3.15E-03	super_enhancer	promoter	RP11-263C24.1
chr11	345000	chr11	85635000	3.51E-03	super_enhancer	promoter	RP11-90K17.2
chr11	345000	chr11	123325000	3.04E-03	super_enhancer	promoter	GRAMD1B
chr11	355000	chr11	465000	1.34E-02	super_enhancer	promoter	RP13-317D12.3
chr11	355000	chr11	65565000	5.30E-03	super_enhancer	promoter	RP11-770G2.5
chr11	355000	chr11	70035000	3.40E-03	super_enhancer	promoter	ANO1-AS1
chr11	355000	chr11	70325000	2.80E-03	super_enhancer	promoter	AP001271.3
chr11	355000	chr11	76745000	3.19E-03	super_enhancer	promoter	B3GNT6
chr11	355000	chr11	130165000	2.92E-03	super_enhancer	promoter	DDX18P5
chr11	355000	chr11	133995000	2.50E-03	super_enhancer	promoter	PTP4A2P2
chr11	365000	chr11	5365000	4.14E-02	super_enhancer	promoter	OR51B5
chr11	365000	chr11	6635000	3.97E-02	super_enhancer	promoter	TAF10
chr11	365000	chr11	12365000	2.34E-02	super_enhancer	promoter	AC025300.1
chr11	365000	chr11	17755000	1.70E-02	super_enhancer	promoter	KCNC1
chr11	365000	chr11	43755000	5.69E-03	super_enhancer	promoter	PHBP2
chr11	365000	chr11	60635000	5.62E-03	super_enhancer	promoter	ZP1
chr11	365000	chr11	105805000	2.15E-03	super_enhancer	promoter	AP000673.1

chr11	365000	chr11	115405000	2.96E-03	super_enhancer	promoter	RP11-136I14.2
chr11	385000	chr11	17215000	1.91E-02	super_enhancer	promoter	RP11-452G18.1
chr11	385000	chr11	47235000	9.40E-03	super_enhancer	promoter	DDB2
chr11	385000	chr11	61355000	6.64E-03	super_enhancer	promoter	RP11-855O10.2
chr11	385000	chr11	62705000	6.25E-03	super_enhancer	promoter	RN7SL259P
chr11	395000	chr11	18815000	2.08E-02	super_enhancer	promoter	PTPN5
chr11	395000	chr11	47375000	9.61E-03	super_enhancer	promoter	MYBPC3
chr11	395000	chr11	74395000	4.82E-03	super_enhancer	promoter	RN7SKP297
chr11	405000	chr11	6025000	4.11E-02	super_enhancer	promoter	OR56A4
chr11	405000	chr11	8715000	3.06E-02	super_enhancer	promoter	RP11-152H18.3
chr11	405000	chr11	9455000	2.74E-02	super_enhancer	promoter	CTD-2371O3.2
chr11	405000	chr11	18345000	1.30E-02	super_enhancer	promoter	HPS5
chr11	405000	chr11	62195000	5.38E-03	super_enhancer	promoter	CTD-2531D15.4
chr11	405000	chr11	70245000	4.20E-03	super_enhancer	promoter	AP000487.5
chr11	465000	chr11	12075000	2.40E-02	promoter	super_enhancer	RP13-317D12.3
chr11	575000	chr11	69325000	5.22E-03	promoter	super_enhancer	PHRF1
chr11	665000	chr11	34225000	1.14E-02	promoter	super_enhancer	RP11-754B17.1
chr11	665000	chr11	47965000	7.67E-03	promoter	super_enhancer	RP11-754B17.1
chr11	665000	chr11	65425000	6.83E-03	promoter	super_enhancer	RP11-754B17.1
chr11	665000	chr11	67025000	4.57E-03	promoter	super_enhancer	RP11-754B17.1
chr11	665000	chr11	68125000	5.23E-03	promoter	super_enhancer	RP11-754B17.1
chr11	695000	chr11	66325000	7.41E-03	promoter	super_enhancer	TMEM80
chr11	695000	chr11	68125000	6.34E-03	promoter	super_enhancer	TMEM80
chr11	825000	chr11	1555000	3.72E-02	promoter	super_enhancer	CRACR2B

chr11	825000	chr11	46365000	1.15E-02	promoter	super_enhancer	CRACR2B
chr11	825000	chr11	65275000	6.69E-03	promoter	super_enhancer	CRACR2B
chr11	825000	chr11	65295000	8.52E-03	promoter	super_enhancer	CRACR2B
chr11	825000	chr11	66855000	6.49E-03	promoter	super_enhancer	CRACR2B
chr11	845000	chr11	57365000	5.28E-03	super_enhancer	promoter	SERPING1
chr11	845000	chr11	60635000	5.94E-03	super_enhancer	promoter	ZP1
chr11	845000	chr11	66105000	5.18E-03	super_enhancer	promoter	RIN1
chr11	915000	chr11	10905000	2.10E-02	promoter	super_enhancer	CHID1
chr11	915000	chr11	13215000	2.63E-02	promoter	super_enhancer	CHID1
chr11	915000	chr11	65515000	5.37E-03	promoter	super_enhancer	CHID1
chr11	915000	chr11	120135000	4.14E-03	promoter	super_enhancer	CHID1
chr11	925000	chr11	67285000	7.02E-03	promoter	super_enhancer	AP2A2
chr11	1075000	chr11	65485000	5.90E-03	promoter	super_enhancer	MUC2
chr11	1155000	chr11	12985000	3.48E-02	super_enhancer	promoter	AC084859.1
chr11	1155000	chr11	14295000	3.43E-02	super_enhancer	promoter	RP11-21L19.1
chr11	1155000	chr11	57715000	4.65E-03	super_enhancer	promoter	OR5BD1P
chr11	1155000	chr11	63605000	6.55E-03	super_enhancer	promoter	MARK2
chr11	1155000	chr11	86035000	5.64E-03	super_enhancer	promoter	RN7SL225P
chr11	1555000	chr11	86385000	3.75E-03	super_enhancer	promoter	ME3
chr11	1565000	chr11	1685000	1.02E-02	super_enhancer	promoter	AP006285.7
chr11	1565000	chr11	11205000	2.28E-02	super_enhancer	promoter	RP11-179A10.3
chr11	1565000	chr11	18415000	2.10E-02	super_enhancer	promoter	LDHA
chr11	1565000	chr11	19425000	1.84E-02	super_enhancer	promoter	RNA5SP335
chr11	1565000	chr11	67125000	6.15E-03	super_enhancer	promoter	POLD4

chr11	1685000	chr11	2175000	2.08E-02	promoter	super_enhancer	AP006285.7
chr11	1685000	chr11	2195000	1.72E-02	promoter	super_enhancer	AP006285.7
chr11	1685000	chr11	2205000	9.85E-03	promoter	super_enhancer	AP006285.7
chr11	1685000	chr11	28825000	4.87E-03	promoter	super_enhancer	AP006285.7
chr11	1765000	chr11	1885000	4.63E-02	super_enhancer	promoter	AC051649.12
chr11	1765000	chr11	13485000	2.33E-02	super_enhancer	promoter	BTBD10
chr11	1765000	chr11	33825000	1.08E-02	super_enhancer	promoter	RP11-646J21.3
chr11	1765000	chr11	63745000	5.43E-03	super_enhancer	promoter	AP000721.1
chr11	1775000	chr11	9405000	4.50E-02	super_enhancer	promoter	IPO7
chr11	1775000	chr11	27385000	1.05E-02	super_enhancer	promoter	CCDC34
chr11	1775000	chr11	62195000	7.15E-03	super_enhancer	promoter	CTD-2531D15.4
chr11	1785000	chr11	2175000	1.85E-04	promoter	super_enhancer	CTSD
chr11	1785000	chr11	18395000	2.32E-02	promoter	super_enhancer	CTSD
chr11	1785000	chr11	35175000	1.17E-02	promoter	super_enhancer	CTSD
chr11	1785000	chr11	61695000	7.66E-03	promoter	super_enhancer	CTSD
chr11	1785000	chr11	119685000	4.70E-03	promoter	super_enhancer	CTSD
chr11	1825000	chr11	2175000	3.82E-02	promoter	super_enhancer	AC139143.2
chr11	1825000	chr11	69315000	4.25E-03	promoter	super_enhancer	AC139143.2
chr11	1885000	chr11	2175000	1.53E-02	promoter	super_enhancer	AC051649.12
chr11	1885000	chr11	2185000	3.13E-02	promoter	super_enhancer	AC051649.12
chr11	1885000	chr11	63665000	5.35E-03	promoter	super_enhancer	AC051649.12
chr11	1885000	chr11	65915000	5.41E-03	promoter	super_enhancer	AC051649.12
chr11	1885000	chr11	86165000	4.70E-03	promoter	super_enhancer	AC051649.12
chr11	2175000	chr11	2425000	3.18E-02	super_enhancer	promoter	AC124057.5

chr11	2175000	chr11	2445000	4.15E-02	super_enhancer	promoter	TRPM5
chr11	2175000	chr11	2465000	2.97E-03	super_enhancer	promoter	KCNQ1
chr11	2175000	chr11	71165000	5.24E-03	super_enhancer	promoter	DHCR7
chr11	2175000	chr11	83355000	3.54E-03	super_enhancer	promoter	RP11-160H12.2
chr11	2175000	chr11	85955000	4.72E-03	super_enhancer	promoter	EED
chr11	2185000	chr11	2325000	3.93E-02	super_enhancer	promoter	C11orf21
chr11	2185000	chr11	2445000	3.00E-05	super_enhancer	promoter	TRPM5
chr11	2185000	chr11	9595000	4.21E-02	super_enhancer	promoter	WEE1
chr11	2185000	chr11	35965000	1.36E-02	super_enhancer	promoter	LDLRAD3
chr11	2185000	chr11	63705000	5.96E-03	super_enhancer	promoter	NAA40
chr11	2185000	chr11	85955000	4.26E-03	super_enhancer	promoter	EED
chr11	2195000	chr11	2445000	2.68E-06	super_enhancer	promoter	TRPM5
chr11	2195000	chr11	2925000	4.54E-03	super_enhancer	promoter	SLC22A18AS
chr11	2195000	chr11	6625000	3.42E-03	super_enhancer	promoter	RRP8
chr11	2195000	chr11	8985000	4.85E-02	super_enhancer	promoter	TMEM9B-AS1
chr11	2195000	chr11	12945000	2.90E-02	super_enhancer	promoter	RP11-47J17.2
chr11	2195000	chr11	13485000	3.00E-02	super_enhancer	promoter	BTBD10
chr11	2195000	chr11	16145000	1.56E-02	super_enhancer	promoter	AC103794.1
chr11	2195000	chr11	45825000	1.13E-02	super_enhancer	promoter	SLC35C1
chr11	2195000	chr11	57195000	6.94E-03	super_enhancer	promoter	SLC43A3
chr11	2195000	chr11	67275000	6.79E-03	super_enhancer	promoter	PITPNM1
chr11	2195000	chr11	113845000	4.67E-03	super_enhancer	promoter	HTR3A
chr11	2195000	chr11	118795000	4.04E-03	super_enhancer	promoter	UPK2
chr11	2205000	chr11	2425000	1.70E-02	super_enhancer	promoter	AC124057.5

chr11	2205000	chr11	2445000	1.54E-05	super_enhancer	promoter	TRPM5
chr11	2205000	chr11	2465000	5.68E-03	super_enhancer	promoter	KCNQ1
chr11	2205000	chr11	13375000	2.52E-02	super_enhancer	promoter	RN7SKP151
chr11	2205000	chr11	57405000	4.88E-03	super_enhancer	promoter	AP000662.4
chr11	2205000	chr11	61355000	6.23E-03	super_enhancer	promoter	RP11-855O10.2
chr11	2205000	chr11	74205000	3.91E-03	super_enhancer	promoter	LIPT2
chr11	2205000	chr11	100665000	4.35E-03	super_enhancer	promoter	AC015600.1
chr11	2325000	chr11	2895000	7.53E-04	promoter	super_enhancer	C11orf21
chr11	2325000	chr11	47865000	6.83E-03	promoter	super_enhancer	C11orf21
chr11	2325000	chr11	61965000	2.59E-03	promoter	super_enhancer	C11orf21
chr11	2325000	chr11	67375000	3.40E-03	promoter	super_enhancer	C11orf21
chr11	2425000	chr11	2895000	4.82E-02	promoter	super_enhancer	AC124057.5
chr11	2425000	chr11	12075000	3.65E-02	promoter	super_enhancer	AC124057.5
chr11	2425000	chr11	46365000	1.08E-02	promoter	super_enhancer	AC124057.5
chr11	2425000	chr11	47945000	1.06E-02	promoter	super_enhancer	AC124057.5
chr11	2425000	chr11	69285000	4.43E-03	promoter	super_enhancer	AC124057.5
chr11	2425000	chr11	120135000	4.43E-03	promoter	super_enhancer	AC124057.5
chr11	2445000	chr11	64865000	6.99E-03	promoter	super_enhancer	TRPM5
chr11	2445000	chr11	65915000	6.62E-03	promoter	super_enhancer	TRPM5
chr11	2465000	chr11	2895000	4.06E-02	promoter	super_enhancer	KCNQ1
chr11	2465000	chr11	12045000	2.57E-02	promoter	super_enhancer	KCNQ1
chr11	2465000	chr11	46245000	8.87E-03	promoter	super_enhancer	KCNQ1
chr11	2465000	chr11	47865000	9.73E-03	promoter	super_enhancer	KCNQ1
chr11	2695000	chr11	2855000	6.52E-03	promoter	super_enhancer	KCNQ1OT1_2

chr11	2825000	chr11	9115000	4.23E-02	super_enhancer	promoter	KRT8P41
chr11	2825000	chr11	33825000	1.17E-02	super_enhancer	promoter	RP11-646J21.3
chr11	2825000	chr11	71315000	3.03E-03	super_enhancer	promoter	KRTAP5-11
chr11	2825000	chr11	133715000	2.20E-03	super_enhancer	promoter	SPATA19
chr11	2835000	chr11	17215000	1.92E-02	super_enhancer	promoter	RP11-452G18.1
chr11	2835000	chr11	46785000	7.34E-03	super_enhancer	promoter	SNORD67
chr11	2835000	chr11	70125000	4.19E-03	super_enhancer	promoter	H2AFZP4
chr11	2845000	chr11	6915000	3.90E-02	super_enhancer	promoter	OR2D2
chr11	2845000	chr11	102165000	3.01E-03	super_enhancer	promoter	RP11-864G5.1
chr11	2855000	chr11	15665000	2.37E-02	super_enhancer	promoter	RP11-396O20.1
chr11	2855000	chr11	17095000	2.01E-02	super_enhancer	promoter	AC116533.1
chr11	2855000	chr11	125445000	4.11E-03	super_enhancer	promoter	RNU6-1156P
chr11	2855000	chr11	129765000	4.36E-03	super_enhancer	promoter	NFRKB
chr11	2895000	chr11	10515000	4.90E-02	super_enhancer	promoter	AC021914.1
chr11	2895000	chr11	49945000	4.21E-03	super_enhancer	promoter	OR4R3P
chr11	2895000	chr11	63995000	7.07E-03	super_enhancer	promoter	TRPT1
chr11	2895000	chr11	66085000	6.78E-03	super_enhancer	promoter	CD248
chr11	2895000	chr11	66105000	6.60E-03	super_enhancer	promoter	RIN1
chr11	2895000	chr11	71165000	4.95E-03	super_enhancer	promoter	DHCR7
chr11	2895000	chr11	75645000	3.68E-03	super_enhancer	promoter	RNA5SP344
chr11	2925000	chr11	34265000	1.62E-02	promoter	super_enhancer	SLC22A18AS
chr11	2925000	chr11	62475000	6.54E-03	promoter	super_enhancer	SLC22A18AS
chr11	2925000	chr11	65265000	7.81E-03	promoter	super_enhancer	SLC22A18AS
chr11	2925000	chr11	65285000	8.50E-03	promoter	super_enhancer	SLC22A18AS

chr11	2935000	chr11	12285000	4.19E-02	super_enhancer	promoter	RP11-265D17.2
chr11	2935000	chr11	14295000	3.74E-02	super_enhancer	promoter	RP11-21L19.1
chr11	2935000	chr11	19425000	2.27E-02	super_enhancer	promoter	RNA5SP335
chr11	2935000	chr11	45825000	1.19E-02	super_enhancer	promoter	SLC35C1
chr11	2935000	chr11	62705000	7.48E-03	super_enhancer	promoter	RN7SL259P
chr11	2935000	chr11	75645000	3.94E-03	super_enhancer	promoter	RNA5SP344
chr11	2935000	chr11	93455000	4.50E-03	super_enhancer	promoter	SCARNA9
chr11	2935000	chr11	94475000	5.57E-03	super_enhancer	promoter	RP11-867G2.8
chr11	2935000	chr11	101785000	7.57E-06	super_enhancer	promoter	CEP126
chr11	2935000	chr11	102165000	5.07E-03	super_enhancer	promoter	RP11-864G5.1
chr11	2935000	chr11	123925000	4.47E-03	super_enhancer	promoter	OR10D5P
chr11	2985000	chr11	47855000	1.20E-02	promoter	super_enhancer	SNORA54
chr11	2985000	chr11	47905000	1.49E-02	promoter	super_enhancer	SNORA54
chr11	2985000	chr11	48025000	1.27E-02	promoter	super_enhancer	SNORA54
chr11	2985000	chr11	62555000	8.99E-03	promoter	super_enhancer	SNORA54
chr11	2985000	chr11	65285000	1.02E-02	promoter	super_enhancer	SNORA54
chr11	2985000	chr11	65475000	2.69E-05	promoter	super_enhancer	SNORA54
chr11	3015000	chr11	34275000	1.12E-02	promoter	super_enhancer	NAP1L4
chr11	3015000	chr11	47935000	1.11E-02	promoter	super_enhancer	NAP1L4
chr11	3015000	chr11	65385000	7.77E-03	promoter	super_enhancer	NAP1L4
chr11	3045000	chr11	21305000	1.43E-02	super_enhancer	promoter	CTD-2302A16.2
chr11	3045000	chr11	33915000	1.90E-02	super_enhancer	promoter	LMO2
chr11	3045000	chr11	57365000	8.58E-03	super_enhancer	promoter	SERPING1
chr11	3045000	chr11	66085000	8.59E-03	super_enhancer	promoter	CD248

chr11	3045000	chr11	66105000	8.37E-03	super_enhancer	promoter	RIN1
chr11	3045000	chr11	101785000	4.59E-03	super_enhancer	promoter	CEP126
chr11	3045000	chr11	102165000	1.80E-05	super_enhancer	promoter	RP11-864G5.1
chr11	3055000	chr11	18045000	1.65E-02	super_enhancer	promoter	RP1-59M18.2
chr11	3055000	chr11	27745000	1.79E-02	super_enhancer	promoter	BDNF
chr11	3055000	chr11	43755000	8.75E-03	super_enhancer	promoter	PHBP2
chr11	3055000	chr11	61295000	7.95E-03	super_enhancer	promoter	RP11-286N22.16
chr11	3055000	chr11	66035000	6.29E-03	super_enhancer	promoter	RAB1B
chr11	3055000	chr11	66085000	7.61E-03	super_enhancer	promoter	CD248
chr11	3055000	chr11	67395000	7.86E-03	super_enhancer	promoter	RP11-655M14.13
chr11	3055000	chr11	70125000	5.53E-03	super_enhancer	promoter	H2AFZP4
chr11	3055000	chr11	71515000	4.68E-03	super_enhancer	promoter	AP002495.1
chr11	3055000	chr11	75515000	5.35E-03	super_enhancer	promoter	RP11-535A19.1
chr11	3055000	chr11	100665000	5.70E-03	super_enhancer	promoter	AC015600.1
chr11	3125000	chr11	6465000	7.40E-03	super_enhancer	promoter	HPX
chr11	3125000	chr11	18175000	2.41E-02	super_enhancer	promoter	RP11-113D6.3
chr11	3125000	chr11	18345000	2.21E-02	super_enhancer	promoter	HPS5
chr11	3125000	chr11	33915000	1.76E-02	super_enhancer	promoter	LMO2
chr11	3125000	chr11	57105000	6.31E-03	super_enhancer	promoter	P2RX3
chr11	3125000	chr11	78035000	3.87E-03	super_enhancer	promoter	RP11-452H21.1
chr11	3125000	chr11	86385000	5.84E-03	super_enhancer	promoter	ME3
chr11	3125000	chr11	89955000	4.74E-03	super_enhancer	promoter	RP11-121L10.2
chr11	3125000	chr11	91115000	4.24E-03	super_enhancer	promoter	RP11-843F11.1
chr11	3135000	chr11	11805000	4.66E-02	super_enhancer	promoter	AC104383.1

chr11	3135000	chr11	12285000	4.88E-02	super_enhancer	promoter	RP11-265D17.2
chr11	3135000	chr11	12365000	4.22E-02	super_enhancer	promoter	AC025300.1
chr11	3135000	chr11	19735000	2.50E-02	super_enhancer	promoter	RP11-359E10.1
chr11	3135000	chr11	26745000	1.54E-02	super_enhancer	promoter	SLC5A12
chr11	3135000	chr11	80475000	2.32E-03	super_enhancer	promoter	RP11-686G23.2
chr11	3135000	chr11	117015000	3.86E-03	super_enhancer	promoter	RP11-109L13.5
chr11	3135000	chr11	119275000	5.63E-03	super_enhancer	promoter	RP11-334E6.4
chr11	3135000	chr11	120895000	4.10E-03	super_enhancer	promoter	TBCEL
chr11	3135000	chr11	126225000	5.44E-03	super_enhancer	promoter	ST3GAL4-AS1
chr11	3145000	chr11	12365000	4.02E-02	super_enhancer	promoter	AC025300.1
chr11	3145000	chr11	32075000	1.54E-02	super_enhancer	promoter	RP1-17K7.3
chr11	3145000	chr11	47375000	1.35E-02	super_enhancer	promoter	MYBPC3
chr11	3145000	chr11	64685000	8.01E-03	super_enhancer	promoter	ATG2A
chr11	3145000	chr11	65135000	8.31E-03	super_enhancer	promoter	RP11-867O8.5
chr11	3145000	chr11	66045000	9.54E-03	super_enhancer	promoter	RP11-867G23.4
chr11	3145000	chr11	66115000	4.73E-05	super_enhancer	promoter	B4GAT1
chr11	3145000	chr11	66445000	7.19E-03	super_enhancer	promoter	RBM4B
chr11	3145000	chr11	70035000	5.62E-03	super_enhancer	promoter	ANO1-AS1
chr11	3145000	chr11	101455000	6.00E-03	super_enhancer	promoter	RP11-748H22.1
chr11	3145000	chr11	102165000	5.54E-03	super_enhancer	promoter	RP11-864G5.1
chr11	3155000	chr11	3445000	2.15E-02	super_enhancer	promoter	FAM86GP
chr11	3155000	chr11	14665000	3.43E-02	super_enhancer	promoter	PSMA1
chr11	3155000	chr11	27495000	1.43E-02	super_enhancer	promoter	LGR4
chr11	3155000	chr11	36345000	1.24E-02	super_enhancer	promoter	RP11-514F3.4

chr11	3155000	chr11	47235000	1.23E-02	super_enhancer	promoter	DDB2
chr11	3155000	chr11	63705000	7.27E-03	super_enhancer	promoter	NAA40
chr11	3155000	chr11	85165000	4.52E-03	super_enhancer	promoter	RP11-482L11.1
chr11	3155000	chr11	102165000	5.47E-03	super_enhancer	promoter	RP11-864G5.1
chr11	3155000	chr11	107485000	5.01E-03	super_enhancer	promoter	AP000889.3
chr11	3155000	chr11	118465000	5.32E-03	super_enhancer	promoter	RNU6-1157P
chr11	3255000	chr11	34245000	1.37E-02	promoter	super_enhancer	MRGPRE
chr11	3255000	chr11	73325000	6.13E-03	promoter	super_enhancer	MRGPRE
chr11	3395000	chr11	12675000	3.02E-02	promoter	super_enhancer	AC123788.1
chr11	3395000	chr11	47985000	1.10E-02	promoter	super_enhancer	AC123788.1
chr11	3395000	chr11	63655000	6.78E-03	promoter	super_enhancer	AC123788.1
chr11	3445000	chr11	35175000	1.29E-02	promoter	super_enhancer	FAM86GP
chr11	3445000	chr11	47765000	1.32E-02	promoter	super_enhancer	FAM86GP
chr11	3445000	chr11	65265000	8.13E-03	promoter	super_enhancer	FAM86GP
chr11	3915000	chr11	11975000	3.25E-02	promoter	super_enhancer	AC090804.1
chr11	3915000	chr11	12685000	3.51E-02	promoter	super_enhancer	AC090804.1
chr11	3915000	chr11	35145000	1.11E-02	promoter	super_enhancer	AC090804.1
chr11	3915000	chr11	47925000	1.09E-02	promoter	super_enhancer	AC090804.1
chr11	3915000	chr11	65305000	7.19E-03	promoter	super_enhancer	AC090804.1
chr11	3995000	chr11	6605000	1.19E-02	promoter	super_enhancer	HNRNPA1P76
chr11	3995000	chr11	11975000	4.23E-02	promoter	super_enhancer	HNRNPA1P76
chr11	3995000	chr11	47965000	1.17E-02	promoter	super_enhancer	HNRNPA1P76
chr11	3995000	chr11	62545000	9.18E-03	promoter	super_enhancer	HNRNPA1P76
chr11	3995000	chr11	86755000	4.19E-03	promoter	super_enhancer	HNRNPA1P76

chr11	4115000	chr11	12045000	9.49E-04	promoter	super_enhancer	RRM1
chr11	4115000	chr11	65555000	9.64E-03	promoter	super_enhancer	RRM1
chr11	4115000	chr11	65915000	7.94E-03	promoter	super_enhancer	RRM1
chr11	4265000	chr11	11975000	1.59E-02	promoter	super_enhancer	SSU72P2
chr11	4265000	chr11	66315000	3.60E-03	promoter	super_enhancer	SSU72P2
chr11	4415000	chr11	12185000	3.36E-02	promoter	super_enhancer	TRIM21
chr11	4495000	chr11	11995000	4.93E-02	promoter	super_enhancer	OR52K3P
chr11	4495000	chr11	47885000	1.05E-02	promoter	super_enhancer	OR52K3P
chr11	4565000	chr11	111725000	4.67E-03	promoter	super_enhancer	OR52M1
chr11	4615000	chr11	47965000	8.32E-03	promoter	super_enhancer	OR52I1
chr11	4665000	chr11	123465000	4.16E-03	promoter	super_enhancer	OR51E1
chr11	4725000	chr11	47765000	1.28E-02	promoter	super_enhancer	MMP26
chr11	4775000	chr11	66015000	4.94E-03	promoter	super_enhancer	OR51F4P
chr11	4995000	chr11	123455000	3.99E-03	promoter	super_enhancer	OR51A5P
chr11	5035000	chr11	34255000	1.15E-02	promoter	super_enhancer	OR51P1P
chr11	5125000	chr11	48025000	8.49E-03	promoter	super_enhancer	OR52J1P
chr11	5125000	chr11	57305000	7.27E-03	promoter	super_enhancer	OR52J1P
chr11	5225000	chr11	94745000	4.79E-03	promoter	super_enhancer	AC104389.16
chr11	5265000	chr11	35145000	9.20E-03	promoter	super_enhancer	HBBP1
chr11	5265000	chr11	86745000	3.41E-03	promoter	super_enhancer	HBBP1
chr11	5275000	chr11	10905000	3.17E-02	promoter	super_enhancer	AC104389.1
chr11	5275000	chr11	35175000	1.00E-02	promoter	super_enhancer	AC104389.1
chr11	5275000	chr11	123445000	3.51E-03	promoter	super_enhancer	AC104389.1
chr11	5275000	chr11	128215000	2.79E-03	promoter	super_enhancer	AC104389.1

chr11	5315000	chr11	12065000	2.25E-02	promoter	super_enhancer	OR51AB1P
chr11	5315000	chr11	12165000	3.72E-02	promoter	super_enhancer	OR51AB1P
chr11	5315000	chr11	34255000	9.05E-03	promoter	super_enhancer	OR51AB1P
chr11	5365000	chr11	12075000	4.41E-02	promoter	super_enhancer	OR51B5
chr11	5365000	chr11	46245000	9.98E-03	promoter	super_enhancer	OR51B5
chr11	5365000	chr11	62505000	6.36E-03	promoter	super_enhancer	OR51B5
chr11	5375000	chr11	61685000	5.57E-03	promoter	super_enhancer	AC104389.31
chr11	5375000	chr11	65905000	5.25E-03	promoter	super_enhancer	AC104389.31
chr11	5475000	chr11	48025000	7.80E-03	promoter	super_enhancer	OR51I2
chr11	5575000	chr11	28835000	6.94E-03	promoter	super_enhancer	OR52H2P
chr11	5775000	chr11	65285000	4.17E-03	promoter	super_enhancer	OR52N4
chr11	5785000	chr11	18405000	1.85E-02	promoter	super_enhancer	OR56B2P
chr11	5785000	chr11	57325000	5.38E-03	promoter	super_enhancer	OR56B2P
chr11	5905000	chr11	10905000	3.69E-02	promoter	super_enhancer	OR52E4
chr11	5905000	chr11	19675000	1.91E-02	promoter	super_enhancer	OR52E4
chr11	5905000	chr11	34275000	1.03E-02	promoter	super_enhancer	OR52E4
chr11	5905000	chr11	62005000	5.50E-03	promoter	super_enhancer	OR52E4
chr11	5925000	chr11	10905000	3.91E-02	promoter	super_enhancer	OR52Q1P
chr11	6025000	chr11	12045000	4.46E-02	promoter	super_enhancer	OR56A4
chr11	6025000	chr11	18385000	2.21E-02	promoter	super_enhancer	OR56A4
chr11	6025000	chr11	34275000	1.19E-02	promoter	super_enhancer	OR56A4
chr11	6175000	chr11	100745000	4.21E-03	promoter	super_enhancer	OR52B1P
chr11	6225000	chr11	28835000	1.37E-02	promoter	super_enhancer	RP11-290F24.6
chr11	6225000	chr11	62485000	5.96E-03	promoter	super_enhancer	RP11-290F24.6

chr11	6255000	chr11	47935000	1.21E-02	promoter	super_enhancer	CNGA4
chr11	6255000	chr11	65305000	8.68E-03	promoter	super_enhancer	CNGA4
chr11	6255000	chr11	86735000	5.16E-03	promoter	super_enhancer	CNGA4
chr11	6345000	chr11	86065000	5.34E-03	promoter	super_enhancer	RP11-304C12.5
chr11	6465000	chr11	65385000	8.58E-03	promoter	super_enhancer	HPX
chr11	6465000	chr11	65495000	8.18E-03	promoter	super_enhancer	HPX
chr11	6605000	chr11	12325000	2.75E-02	super_enhancer	promoter	RP11-573E11.2
chr11	6605000	chr11	13485000	3.85E-02	super_enhancer	promoter	BTBD10
chr11	6605000	chr11	18345000	2.10E-02	super_enhancer	promoter	HPS5
chr11	6605000	chr11	48375000	7.44E-03	super_enhancer	promoter	OR4C4P
chr11	6605000	chr11	66115000	8.43E-03	super_enhancer	promoter	B4GAT1
chr11	6605000	chr11	66445000	6.24E-03	super_enhancer	promoter	RBM4B
chr11	6605000	chr11	82795000	4.09E-03	super_enhancer	promoter	RP11-113K21.6
chr11	6635000	chr11	13215000	4.78E-02	promoter	super_enhancer	TAF10
chr11	6635000	chr11	35145000	1.43E-02	promoter	super_enhancer	TAF10
chr11	6635000	chr11	48015000	1.14E-02	promoter	super_enhancer	TAF10
chr11	6635000	chr11	65305000	9.00E-03	promoter	super_enhancer	TAF10
chr11	6635000	chr11	66315000	9.53E-03	promoter	super_enhancer	TAF10
chr11	6705000	chr11	12695000	4.01E-02	promoter	super_enhancer	MRPL17
chr11	6705000	chr11	65315000	7.86E-03	promoter	super_enhancer	MRPL17
chr11	6705000	chr11	65465000	7.77E-03	promoter	super_enhancer	MRPL17
chr11	6805000	chr11	65275000	5.05E-03	promoter	super_enhancer	OR2AG1
chr11	6915000	chr11	65615000	7.21E-03	promoter	super_enhancer	OR2D2
chr11	7535000	chr11	18405000	2.94E-02	promoter	super_enhancer	CTD-2516F10.2

chr11	7535000	chr11	64855000	8.77E-03	promoter	super_enhancer	CTD-2516F10.2
chr11	7535000	chr11	65295000	1.02E-02	promoter	super_enhancer	CTD-2516F10.2
chr11	7535000	chr11	65515000	7.60E-03	promoter	super_enhancer	CTD-2516F10.2
chr11	7535000	chr11	86795000	4.29E-03	promoter	super_enhancer	CTD-2516F10.2
chr11	7535000	chr11	126345000	5.52E-03	promoter	super_enhancer	CTD-2516F10.2
chr11	7795000	chr11	65425000	7.87E-03	promoter	super_enhancer	OR5P1P
chr11	7805000	chr11	65275000	8.53E-03	promoter	super_enhancer	RP11-494M8.1
chr11	7805000	chr11	65485000	1.11E-02	promoter	super_enhancer	RP11-494M8.1
chr11	7905000	chr11	18395000	3.02E-02	promoter	super_enhancer	RP11-35J10.5
chr11	7905000	chr11	47855000	1.09E-02	promoter	super_enhancer	RP11-35J10.5
chr11	7905000	chr11	65265000	8.36E-03	promoter	super_enhancer	RP11-35J10.5
chr11	7905000	chr11	65275000	7.86E-03	promoter	super_enhancer	RP11-35J10.5
chr11	7905000	chr11	65615000	9.91E-03	promoter	super_enhancer	RP11-35J10.5
chr11	7905000	chr11	86785000	6.01E-03	promoter	super_enhancer	RP11-35J10.5
chr11	8705000	chr11	47925000	1.46E-02	promoter	super_enhancer	AC091053.1
chr11	8705000	chr11	65275000	7.93E-03	promoter	super_enhancer	AC091053.1
chr11	8715000	chr11	35145000	1.53E-02	promoter	super_enhancer	RP11-152H18.3
chr11	8715000	chr11	61715000	8.13E-03	promoter	super_enhancer	RP11-152H18.3
chr11	8715000	chr11	65285000	9.20E-03	promoter	super_enhancer	RP11-152H18.3
chr11	8715000	chr11	86735000	5.35E-03	promoter	super_enhancer	RP11-152H18.3
chr11	8955000	chr11	48005000	1.43E-02	promoter	super_enhancer	C11orf16
chr11	8955000	chr11	114285000	3.79E-03	promoter	super_enhancer	C11orf16
chr11	9025000	chr11	19665000	2.85E-02	promoter	super_enhancer	RP11-467K18.2
chr11	9025000	chr11	47945000	1.42E-02	promoter	super_enhancer	RP11-467K18.2

chr11	9025000	chr11	129865000	4.17E-03	promoter	super_enhancer	RP11-467K18.2
chr11	9115000	chr11	65285000	9.11E-03	promoter	super_enhancer	KRT8P41
chr11	9365000	chr11	18995000	2.77E-02	super_enhancer	promoter	RP11-583F24.3
chr11	9365000	chr11	68385000	6.89E-03	super_enhancer	promoter	CTD-2007L18.5
chr11	9365000	chr11	72505000	6.93E-03	super_enhancer	promoter	ARAP1
chr11	9365000	chr11	82515000	3.97E-03	super_enhancer	promoter	RP11-718B12.1
chr11	9405000	chr11	65295000	1.10E-02	promoter	super_enhancer	IPO7
chr11	9405000	chr11	65515000	8.22E-03	promoter	super_enhancer	IPO7
chr11	9405000	chr11	86165000	6.14E-03	promoter	super_enhancer	IPO7
chr11	9455000	chr11	69325000	6.21E-03	promoter	super_enhancer	CTD-2371O3.2
chr11	9685000	chr11	12065000	9.56E-03	promoter	super_enhancer	SWAP70
chr11	9685000	chr11	47875000	1.65E-02	promoter	super_enhancer	SWAP70
chr11	9685000	chr11	65425000	1.25E-02	promoter	super_enhancer	SWAP70
chr11	9685000	chr11	107835000	4.47E-03	promoter	super_enhancer	SWAP70
chr11	10295000	chr11	18385000	2.55E-02	promoter	super_enhancer	RP11-351I24.3
chr11	10295000	chr11	28835000	1.20E-02	promoter	super_enhancer	RP11-351I24.3
chr11	10295000	chr11	46365000	1.25E-02	promoter	super_enhancer	RP11-351I24.3
chr11	10295000	chr11	47945000	1.22E-02	promoter	super_enhancer	RP11-351I24.3
chr11	10295000	chr11	126345000	4.49E-03	promoter	super_enhancer	RP11-351I24.3
chr11	10305000	chr11	57195000	7.82E-03	super_enhancer	promoter	SLC43A3
chr11	10305000	chr11	61295000	8.06E-03	super_enhancer	promoter	RP11-286N22.16
chr11	10305000	chr11	126225000	4.22E-03	super_enhancer	promoter	ST3GAL4-AS1
chr11	10325000	chr11	48005000	1.34E-02	promoter	super_enhancer	RP11-351I24.1
chr11	10325000	chr11	65495000	8.51E-03	promoter	super_enhancer	RP11-351I24.1

chr11	10325000	chr11	100745000	4.06E-03	promoter	super_enhancer	RP11-351I24.1
chr11	10515000	chr11	47925000	1.80E-02	promoter	super_enhancer	AC021914.1
chr11	10515000	chr11	47935000	1.34E-04	promoter	super_enhancer	AC021914.1
chr11	10515000	chr11	65305000	1.15E-02	promoter	super_enhancer	AC021914.1
chr11	10515000	chr11	107835000	4.46E-03	promoter	super_enhancer	AC021914.1
chr11	10805000	chr11	11995000	2.75E-02	promoter	super_enhancer	RP11-685M7.5
chr11	10805000	chr11	12065000	5.52E-03	promoter	super_enhancer	RP11-685M7.5
chr11	10805000	chr11	66965000	7.50E-03	promoter	super_enhancer	RP11-685M7.5
chr11	10885000	chr11	11645000	2.65E-02	super_enhancer	promoter	GALNT18
chr11	10885000	chr11	12365000	2.51E-02	super_enhancer	promoter	AC025300.1
chr11	10885000	chr11	17035000	2.74E-02	super_enhancer	promoter	OR7E14P
chr11	10885000	chr11	45535000	9.22E-03	super_enhancer	promoter	RP11-430H10.4
chr11	10885000	chr11	46135000	7.78E-03	super_enhancer	promoter	AC024475.1
chr11	10885000	chr11	74165000	4.41E-03	super_enhancer	promoter	RP11-702H23.6
chr11	10895000	chr11	12285000	4.94E-02	super_enhancer	promoter	RP11-265D17.2
chr11	10895000	chr11	22135000	1.58E-02	super_enhancer	promoter	CTD-2019O4.1
chr11	10895000	chr11	33185000	1.31E-02	super_enhancer	promoter	CSTF3
chr11	10895000	chr11	56085000	3.24E-03	super_enhancer	promoter	OR8K3
chr11	10895000	chr11	66035000	5.23E-03	super_enhancer	promoter	RAB1B
chr11	10905000	chr11	12115000	4.65E-02	super_enhancer	promoter	MICAL2
chr11	10905000	chr11	12845000	6.86E-03	super_enhancer	promoter	RP11-47J17.3
chr11	10905000	chr11	16045000	2.73E-02	super_enhancer	promoter	CTD-3096P4.1
chr11	10905000	chr11	17215000	2.82E-02	super_enhancer	promoter	RP11-452G18.1
chr11	10905000	chr11	19425000	2.24E-02	super_enhancer	promoter	RNA5SP335

chr11	10905000	chr11	20385000	1.40E-02	super_enhancer	promoter	HTATIP2
chr11	10905000	chr11	55035000	2.84E-03	super_enhancer	promoter	RP11-72M10.2
chr11	10905000	chr11	77525000	3.61E-03	super_enhancer	promoter	AP000580.1
chr11	11205000	chr11	46365000	1.06E-02	promoter	super_enhancer	RP11-179A10.3
chr11	11265000	chr11	12075000	9.54E-03	promoter	super_enhancer	CTD-3224I3.3
chr11	11265000	chr11	62475000	6.31E-03	promoter	super_enhancer	CTD-3224I3.3
chr11	11375000	chr11	47915000	8.86E-03	promoter	super_enhancer	CSNK2A3
chr11	11375000	chr11	57325000	6.49E-03	promoter	super_enhancer	CSNK2A3
chr11	11645000	chr11	62025000	8.45E-03	promoter	super_enhancer	GALNT18
chr11	11645000	chr11	66005000	9.56E-03	promoter	super_enhancer	GALNT18
chr11	11805000	chr11	13215000	8.23E-03	promoter	super_enhancer	AC104383.1
chr11	11805000	chr11	48005000	1.48E-02	promoter	super_enhancer	AC104383.1
chr11	11975000	chr11	14295000	1.62E-02	super_enhancer	promoter	RP11-21L19.1
chr11	11975000	chr11	18715000	4.17E-02	super_enhancer	promoter	TMEM86A
chr11	11975000	chr11	45825000	1.37E-02	super_enhancer	promoter	SLC35C1
chr11	11975000	chr11	66035000	6.55E-03	super_enhancer	promoter	RAB1B
chr11	11975000	chr11	71935000	6.41E-03	super_enhancer	promoter	INPPL1
chr11	11975000	chr11	76495000	6.52E-03	super_enhancer	promoter	RP11-21L23.3
chr11	11975000	chr11	93585000	3.93E-03	super_enhancer	promoter	VSTM5
chr11	11975000	chr11	119275000	4.34E-03	super_enhancer	promoter	RP11-334E6.4
chr11	11995000	chr11	27745000	2.64E-02	super_enhancer	promoter	BDNF
chr11	11995000	chr11	44995000	1.74E-02	super_enhancer	promoter	AC068858.1
chr11	11995000	chr11	57245000	1.04E-02	super_enhancer	promoter	RP11-624G17.3
chr11	11995000	chr11	64685000	9.74E-03	super_enhancer	promoter	ATG2A

chr11	11995000	chr11	71615000	5.22E-03	super_enhancer	promoter	AP000719.1
chr11	11995000	chr11	95525000	5.51E-03	super_enhancer	promoter	FAM76B
chr11	11995000	chr11	122295000	4.27E-03	super_enhancer	promoter	MIR100HG
chr11	11995000	chr11	129985000	4.08E-03	super_enhancer	promoter	U4
chr11	12045000	chr11	12945000	2.00E-02	super_enhancer	promoter	RP11-47J17.2
chr11	12045000	chr11	18175000	3.51E-02	super_enhancer	promoter	RP11-113D6.3
chr11	12045000	chr11	63605000	7.12E-03	super_enhancer	promoter	MARK2
chr11	12045000	chr11	77525000	4.73E-03	super_enhancer	promoter	AP000580.1
chr11	12045000	chr11	124275000	3.16E-03	super_enhancer	promoter	OR8X1P
chr11	12055000	chr11	12945000	3.63E-02	super_enhancer	promoter	RP11-47J17.2
chr11	12055000	chr11	18345000	3.93E-02	super_enhancer	promoter	HPS5
chr11	12055000	chr11	57635000	9.78E-03	super_enhancer	promoter	OR5BA1P
chr11	12065000	chr11	12325000	1.06E-02	super_enhancer	promoter	RP11-573E11.2
chr11	12065000	chr11	12845000	9.78E-04	super_enhancer	promoter	RP11-47J17.3
chr11	12065000	chr11	12985000	1.56E-02	super_enhancer	promoter	AC084859.1
chr11	12065000	chr11	61525000	5.02E-03	super_enhancer	promoter	DKFZP434K028
chr11	12065000	chr11	63705000	5.32E-03	super_enhancer	promoter	NAA40
chr11	12075000	chr11	12285000	4.03E-05	super_enhancer	promoter	RP11-265D17.2
chr11	12075000	chr11	16635000	4.87E-02	super_enhancer	promoter	C11orf58
chr11	12075000	chr11	59475000	7.66E-03	super_enhancer	promoter	RN7SKP192
chr11	12075000	chr11	71515000	5.72E-03	super_enhancer	promoter	AP002495.1
chr11	12075000	chr11	91115000	4.11E-03	super_enhancer	promoter	RP11-843F11.1
chr11	12075000	chr11	95695000	5.28E-03	super_enhancer	promoter	RP11-697H10.1
chr11	12085000	chr11	71135000	7.52E-03	super_enhancer	promoter	FLJ42102

chr11	12085000	chr11	73915000	4.71E-03	super_enhancer	promoter	AP000577.2
chr11	12085000	chr11	123625000	4.14E-03	super_enhancer	promoter	OR6X1
chr11	12115000	chr11	47965000	1.27E-02	promoter	super_enhancer	MICAL2
chr11	12115000	chr11	63685000	1.05E-02	promoter	super_enhancer	MICAL2
chr11	12115000	chr11	129845000	4.03E-03	promoter	super_enhancer	MICAL2
chr11	12165000	chr11	12285000	4.92E-02	super_enhancer	promoter	RP11-265D17.2
chr11	12165000	chr11	25945000	2.37E-02	super_enhancer	promoter	RP11-20J1.1
chr11	12165000	chr11	56215000	5.18E-03	super_enhancer	promoter	OR5M4P
chr11	12165000	chr11	62415000	9.43E-03	super_enhancer	promoter	GANAB
chr11	12165000	chr11	80835000	3.32E-03	super_enhancer	promoter	AP003464.1
chr11	12165000	chr11	86385000	6.18E-03	super_enhancer	promoter	ME3
chr11	12185000	chr11	18995000	2.72E-02	super_enhancer	promoter	RP11-583F24.3
chr11	12185000	chr11	57345000	8.19E-03	super_enhancer	promoter	RPS4XP13
chr11	12285000	chr11	47855000	1.33E-02	promoter	super_enhancer	RP11-265D17.2
chr11	12285000	chr11	72735000	6.38E-03	promoter	super_enhancer	RP11-265D17.2
chr11	12365000	chr11	65385000	9.32E-03	promoter	super_enhancer	AC025300.1
chr11	12675000	chr11	26595000	2.02E-02	super_enhancer	promoter	MUC15
chr11	12675000	chr11	26745000	1.80E-02	super_enhancer	promoter	SLC5A12
chr11	12675000	chr11	33915000	2.03E-02	super_enhancer	promoter	LMO2
chr11	12675000	chr11	47375000	1.47E-02	super_enhancer	promoter	MYBPC3
chr11	12675000	chr11	66085000	8.20E-03	super_enhancer	promoter	CD248
chr11	12675000	chr11	66445000	7.40E-03	super_enhancer	promoter	RBM4B
chr11	12685000	chr11	27745000	2.57E-02	super_enhancer	promoter	BDNF
chr11	12685000	chr11	33185000	1.96E-02	super_enhancer	promoter	CSTF3

chr11	12685000	chr11	72495000	9.15E-03	super_enhancer	promoter	MIR4692
chr11	12695000	chr11	33375000	1.15E-02	super_enhancer	promoter	AL122015.1
chr11	12695000	chr11	62585000	7.23E-03	super_enhancer	promoter	RNU6-118P
chr11	12695000	chr11	74545000	4.29E-03	super_enhancer	promoter	AP000560.3
chr11	12695000	chr11	125035000	3.85E-03	super_enhancer	promoter	PKNOX2-AS1
chr11	12845000	chr11	35165000	1.61E-02	promoter	super_enhancer	RP11-47J17.3
chr11	12845000	chr11	65515000	1.66E-05	promoter	super_enhancer	RP11-47J17.3
chr11	12845000	chr11	86755000	2.83E-03	promoter	super_enhancer	RP11-47J17.3
chr11	12945000	chr11	13215000	4.28E-02	promoter	super_enhancer	RP11-47J17.2
chr11	12945000	chr11	64865000	7.28E-03	promoter	super_enhancer	RP11-47J17.2
chr11	12945000	chr11	129855000	4.07E-03	promoter	super_enhancer	RP11-47J17.2
chr11	12985000	chr11	13215000	5.01E-03	promoter	super_enhancer	AC084859.1
chr11	12985000	chr11	100745000	4.39E-03	promoter	super_enhancer	AC084859.1
chr11	13215000	chr11	101455000	5.10E-03	super_enhancer	promoter	RP11-748H22.1
chr11	13375000	chr11	18385000	9.31E-04	promoter	super_enhancer	RN7SKP151
chr11	13375000	chr11	68125000	7.88E-03	promoter	super_enhancer	RN7SKP151
chr11	14295000	chr11	63665000	9.09E-03	promoter	super_enhancer	RP11-21L19.1
chr11	14295000	chr11	69425000	7.39E-03	promoter	super_enhancer	RP11-21L19.1
chr11	14665000	chr11	47765000	1.53E-02	promoter	super_enhancer	PSMA1
chr11	14665000	chr11	119695000	3.75E-03	promoter	super_enhancer	PSMA1
chr11	14915000	chr11	47855000	1.01E-02	promoter	super_enhancer	CYP2R1
chr11	14915000	chr11	47985000	1.35E-02	promoter	super_enhancer	CYP2R1
chr11	14995000	chr11	35165000	2.14E-02	promoter	super_enhancer	CALCA
chr11	15645000	chr11	62465000	8.55E-03	promoter	super_enhancer	RP11-531H8.2

chr11	16145000	chr11	62485000	3.50E-03	promoter	super_enhancer	AC103794.1
chr11	17035000	chr11	48015000	1.20E-02	promoter	super_enhancer	OR7E14P
chr11	17075000	chr11	47865000	8.62E-03	promoter	super_enhancer	AC116533.2
chr11	17095000	chr11	33695000	1.44E-02	promoter	super_enhancer	AC116533.1
chr11	17095000	chr11	62495000	7.22E-03	promoter	super_enhancer	AC116533.1
chr11	17115000	chr11	35155000	1.42E-02	promoter	super_enhancer	AC116533.4
chr11	17115000	chr11	47995000	1.24E-02	promoter	super_enhancer	AC116533.4
chr11	17215000	chr11	65265000	8.19E-03	promoter	super_enhancer	RP11-452G18.1
chr11	17215000	chr11	67355000	7.26E-03	promoter	super_enhancer	RP11-452G18.1
chr11	17215000	chr11	123465000	3.70E-03	promoter	super_enhancer	RP11-452G18.1
chr11	17755000	chr11	35165000	2.46E-02	promoter	super_enhancer	KCNC1
chr11	17755000	chr11	47915000	1.26E-02	promoter	super_enhancer	KCNC1
chr11	17755000	chr11	61695000	9.53E-03	promoter	super_enhancer	KCNC1
chr11	17755000	chr11	86795000	4.22E-03	promoter	super_enhancer	KCNC1
chr11	18035000	chr11	34225000	2.06E-02	promoter	super_enhancer	SERGEF
chr11	18065000	chr11	65385000	6.98E-03	promoter	super_enhancer	TPH1
chr11	18065000	chr11	69295000	4.90E-03	promoter	super_enhancer	TPH1
chr11	18175000	chr11	34645000	1.80E-02	promoter	super_enhancer	RP11-113D6.3
chr11	18175000	chr11	65555000	8.53E-03	promoter	super_enhancer	RP11-113D6.3
chr11	18345000	chr11	65505000	6.77E-03	promoter	super_enhancer	HPS5
chr11	18385000	chr11	22385000	3.36E-02	super_enhancer	promoter	CTD-2140G10.4
chr11	18385000	chr11	22515000	4.41E-02	super_enhancer	promoter	LINC01495
chr11	18385000	chr11	32915000	1.77E-02	super_enhancer	promoter	QSER1
chr11	18385000	chr11	41415000	9.72E-03	super_enhancer	promoter	RP11-63C8.1

chr11	18385000	chr11	47375000	1.36E-02	super_enhancer	promoter	MYBPC3
chr11	18385000	chr11	57635000	7.74E-03	super_enhancer	promoter	OR5BA1P
chr11	18395000	chr11	36345000	1.62E-02	super_enhancer	promoter	RP11-514F3.4
chr11	18395000	chr11	115405000	3.31E-03	super_enhancer	promoter	RP11-136I14.2
chr11	18405000	chr11	27015000	2.35E-04	super_enhancer	promoter	FIBIN
chr11	18405000	chr11	32075000	2.02E-02	super_enhancer	promoter	RP1-17K7.3
chr11	18405000	chr11	57245000	8.99E-03	super_enhancer	promoter	RP11-624G17.3
chr11	18405000	chr11	94375000	3.59E-03	super_enhancer	promoter	RP11-867G2.6
chr11	18415000	chr11	34275000	1.82E-02	promoter	super_enhancer	LDHA
chr11	18715000	chr11	34645000	2.29E-02	promoter	super_enhancer	TMEM86A
chr11	18715000	chr11	69285000	6.57E-03	promoter	super_enhancer	TMEM86A
chr11	18815000	chr11	35175000	2.05E-02	promoter	super_enhancer	PTPN5
chr11	18815000	chr11	47955000	1.88E-02	promoter	super_enhancer	PTPN5
chr11	18895000	chr11	126405000	2.58E-03	promoter	super_enhancer	RP11-1081L13.3
chr11	18995000	chr11	34225000	1.93E-02	promoter	super_enhancer	RP11-583F24.3
chr11	18995000	chr11	129645000	3.04E-03	promoter	super_enhancer	RP11-583F24.3
chr11	19425000	chr11	34225000	2.37E-02	promoter	super_enhancer	RNA5SP335
chr11	19425000	chr11	34245000	1.77E-02	promoter	super_enhancer	RNA5SP335
chr11	19425000	chr11	61965000	5.78E-03	promoter	super_enhancer	RNA5SP335
chr11	19425000	chr11	67285000	1.00E-02	promoter	super_enhancer	RNA5SP335
chr11	19665000	chr11	27745000	2.85E-02	super_enhancer	promoter	BDNF
chr11	19665000	chr11	36345000	1.61E-02	super_enhancer	promoter	RP11-514F3.4
chr11	19665000	chr11	60145000	5.98E-03	super_enhancer	promoter	MS4A7
chr11	19665000	chr11	61295000	8.85E-03	super_enhancer	promoter	RP11-286N22.16

chr11	19665000	chr11	62305000	7.34E-03	super_enhancer	promoter	RP11-864I4.3
chr11	19665000	chr11	62415000	8.15E-03	super_enhancer	promoter	GANAB
chr11	19675000	chr11	47295000	1.42E-02	super_enhancer	promoter	RP11-17G12.3
chr11	19735000	chr11	34235000	1.79E-02	promoter	super_enhancer	RP11-359E10.1
chr11	19735000	chr11	47875000	1.52E-02	promoter	super_enhancer	RP11-359E10.1
chr11	21305000	chr11	34225000	1.49E-02	promoter	super_enhancer	CTD-2302A16.2
chr11	21305000	chr11	47915000	7.64E-03	promoter	super_enhancer	CTD-2302A16.2
chr11	21405000	chr11	64095000	5.63E-03	promoter	super_enhancer	RNA5SP337
chr11	22135000	chr11	61685000	6.86E-03	promoter	super_enhancer	CTD-2019O4.1
chr11	22215000	chr11	28825000	2.33E-02	promoter	super_enhancer	ANO5
chr11	22215000	chr11	86185000	3.78E-03	promoter	super_enhancer	ANO5
chr11	22515000	chr11	35145000	1.96E-02	promoter	super_enhancer	LINC01495
chr11	22515000	chr11	35165000	2.56E-02	promoter	super_enhancer	LINC01495
chr11	22515000	chr11	47935000	1.49E-02	promoter	super_enhancer	LINC01495
chr11	22515000	chr11	72755000	4.61E-03	promoter	super_enhancer	LINC01495
chr11	22515000	chr11	86175000	3.99E-03	promoter	super_enhancer	LINC01495
chr11	23185000	chr11	46235000	1.27E-02	promoter	super_enhancer	RP11-266A24.1
chr11	24285000	chr11	46235000	1.38E-02	promoter	super_enhancer	RP11-2F20.1
chr11	25755000	chr11	46565000	1.31E-02	promoter	super_enhancer	RP11-405K6.1
chr11	26595000	chr11	34655000	2.22E-02	promoter	super_enhancer	MUC15
chr11	26595000	chr11	46565000	1.94E-02	promoter	super_enhancer	MUC15
chr11	26595000	chr11	47985000	1.90E-02	promoter	super_enhancer	MUC15
chr11	27015000	chr11	73325000	5.95E-03	promoter	super_enhancer	FIBIN
chr11	27385000	chr11	62475000	6.70E-03	promoter	super_enhancer	CCDC34

chr11	27495000	chr11	61715000	8.76E-03	promoter	super_enhancer	LGR4
chr11	27495000	chr11	66325000	9.78E-03	promoter	super_enhancer	LGR4
chr11	27505000	chr11	69325000	6.57E-03	promoter	super_enhancer	RP11-159H22.1
chr11	27745000	chr11	34275000	3.18E-02	promoter	super_enhancer	BDNF
chr11	27745000	chr11	46565000	2.42E-02	promoter	super_enhancer	BDNF
chr11	27745000	chr11	47965000	1.93E-02	promoter	super_enhancer	BDNF
chr11	28825000	chr11	33375000	3.35E-02	super_enhancer	promoter	AL122015.1
chr11	28825000	chr11	72495000	9.33E-03	super_enhancer	promoter	MIR4692
chr11	28825000	chr11	73545000	6.63E-03	super_enhancer	promoter	RN7SKP243
chr11	28825000	chr11	124275000	2.64E-03	super_enhancer	promoter	OR8X1P
chr11	28835000	chr11	33915000	3.76E-02	super_enhancer	promoter	LMO2
chr11	28835000	chr11	50255000	6.07E-03	super_enhancer	promoter	RP11-347H15.5
chr11	28835000	chr11	58395000	7.73E-03	super_enhancer	promoter	AP001350.4
chr11	28835000	chr11	82355000	2.80E-03	super_enhancer	promoter	RP11-179A16.2
chr11	28835000	chr11	86005000	3.85E-03	super_enhancer	promoter	SETP17
chr11	29615000	chr11	72745000	3.96E-03	promoter	super_enhancer	RP5-1027O15.1
chr11	29615000	chr11	118915000	2.51E-03	promoter	super_enhancer	RP5-1027O15.1
chr11	29735000	chr11	47865000	1.57E-02	promoter	super_enhancer	CTD-3138F19.1
chr11	30605000	chr11	114305000	2.85E-03	promoter	super_enhancer	RP5-1024C24.1
chr11	32075000	chr11	34265000	1.30E-02	promoter	super_enhancer	RP1-17K7.3
chr11	32075000	chr11	46245000	2.49E-02	promoter	super_enhancer	RP1-17K7.3
chr11	32075000	chr11	47905000	2.50E-02	promoter	super_enhancer	RP1-17K7.3
chr11	32075000	chr11	65295000	1.52E-02	promoter	super_enhancer	RP1-17K7.3
chr11	32085000	chr11	69435000	4.18E-05	promoter	super_enhancer	RP1-17K7.1

chr11	32365000	chr11	34255000	1.01E-02	promoter	super_enhancer	THEM7P
chr11	32365000	chr11	35165000	6.09E-03	promoter	super_enhancer	THEM7P
chr11	32365000	chr11	47875000	2.23E-02	promoter	super_enhancer	THEM7P
chr11	32605000	chr11	68125000	1.23E-02	promoter	super_enhancer	EIF3M
chr11	32615000	chr11	57305000	1.44E-02	promoter	super_enhancer	HNRNPA3P9
chr11	32615000	chr11	64095000	9.71E-03	promoter	super_enhancer	HNRNPA3P9
chr11	32615000	chr11	65905000	1.00E-02	promoter	super_enhancer	HNRNPA3P9
chr11	32915000	chr11	62025000	1.27E-02	promoter	super_enhancer	QSER1
chr11	32915000	chr11	126345000	4.44E-03	promoter	super_enhancer	QSER1
chr11	33185000	chr11	47875000	2.66E-02	promoter	super_enhancer	CSTF3
chr11	33185000	chr11	129855000	4.02E-03	promoter	super_enhancer	CSTF3
chr11	33375000	chr11	33695000	5.39E-04	promoter	super_enhancer	AL122015.1
chr11	33375000	chr11	47855000	1.64E-02	promoter	super_enhancer	AL122015.1
chr11	33375000	chr11	65915000	8.91E-03	promoter	super_enhancer	AL122015.1
chr11	33425000	chr11	47985000	2.90E-02	promoter	super_enhancer	RP1-130L23.1
chr11	33425000	chr11	128175000	2.83E-03	promoter	super_enhancer	RP1-130L23.1
chr11	33695000	chr11	41735000	1.92E-02	super_enhancer	promoter	LINC01499
chr11	33695000	chr11	66045000	1.40E-02	super_enhancer	promoter	RP11-867G23.4
chr11	33695000	chr11	71165000	8.13E-03	super_enhancer	promoter	DHCR7
chr11	33795000	chr11	46555000	2.19E-02	promoter	super_enhancer	FBXO3-AS1
chr11	33795000	chr11	62545000	1.26E-02	promoter	super_enhancer	FBXO3-AS1
chr11	33795000	chr11	68015000	9.64E-03	promoter	super_enhancer	FBXO3-AS1
chr11	33795000	chr11	69435000	8.53E-03	promoter	super_enhancer	FBXO3-AS1
chr11	33825000	chr11	35145000	4.91E-02	promoter	super_enhancer	RP11-646J21.3

chr11	33825000	chr11	47985000	3.23E-02	promoter	super_enhancer	RP11-646J21.3
chr11	33825000	chr11	57315000	1.52E-02	promoter	super_enhancer	RP11-646J21.3
chr11	33825000	chr11	61705000	9.58E-05	promoter	super_enhancer	RP11-646J21.3
chr11	33825000	chr11	65275000	1.40E-02	promoter	super_enhancer	RP11-646J21.3
chr11	33825000	chr11	67025000	1.21E-02	promoter	super_enhancer	RP11-646J21.3
chr11	33845000	chr11	111735000	3.83E-03	promoter	super_enhancer	RP11-646J21.2
chr11	33915000	chr11	65265000	1.64E-02	promoter	super_enhancer	LMO2
chr11	33915000	chr11	65555000	1.76E-02	promoter	super_enhancer	LMO2
chr11	34225000	chr11	45275000	3.58E-02	super_enhancer	promoter	CTD-2560E9.5
chr11	34225000	chr11	57245000	1.83E-02	super_enhancer	promoter	RP11-624G17.3
chr11	34225000	chr11	61735000	1.39E-02	super_enhancer	promoter	FTH1
chr11	34225000	chr11	71135000	1.10E-02	super_enhancer	promoter	FLJ42102
chr11	34225000	chr11	72525000	9.80E-03	super_enhancer	promoter	ATG16L2
chr11	34235000	chr11	46775000	2.41E-02	super_enhancer	promoter	MIR5582
chr11	34245000	chr11	67525000	6.76E-03	super_enhancer	promoter	RP11-655M14.4
chr11	34245000	chr11	73115000	7.98E-03	super_enhancer	promoter	RP11-809N8.4
chr11	34255000	chr11	45275000	2.80E-02	super_enhancer	promoter	CTD-2560E9.5
chr11	34255000	chr11	69185000	9.07E-03	super_enhancer	promoter	RP11-211G23.2
chr11	34255000	chr11	72975000	8.60E-03	super_enhancer	promoter	P2RY6
chr11	34255000	chr11	113845000	3.64E-03	super_enhancer	promoter	HTR3A
chr11	34265000	chr11	50005000	1.11E-02	super_enhancer	promoter	OR4C12
chr11	34265000	chr11	58135000	1.02E-02	super_enhancer	promoter	OR5B1P
chr11	34265000	chr11	60635000	1.84E-02	super_enhancer	promoter	ZP1
chr11	34265000	chr11	61295000	1.70E-02	super_enhancer	promoter	RP11-286N22.16

chr11	34265000	chr11	66085000	1.55E-02	super_enhancer	promoter	CD248
chr11	34265000	chr11	72295000	1.45E-02	super_enhancer	promoter	RP11-169D4.2
chr11	34275000	chr11	57195000	1.28E-02	super_enhancer	promoter	SLC43A3
chr11	34275000	chr11	60635000	1.37E-02	super_enhancer	promoter	ZP1
chr11	34535000	chr11	62035000	1.12E-02	promoter	super_enhancer	ELF5
chr11	34535000	chr11	68125000	1.41E-02	promoter	super_enhancer	ELF5
chr11	34535000	chr11	69305000	1.17E-02	promoter	super_enhancer	ELF5
chr11	34645000	chr11	44995000	3.35E-02	super_enhancer	promoter	AC068858.1
chr11	34645000	chr11	47235000	2.94E-02	super_enhancer	promoter	DDB2
chr11	34645000	chr11	48925000	1.39E-02	super_enhancer	promoter	RP11-56P9.10
chr11	34645000	chr11	50005000	1.03E-02	super_enhancer	promoter	OR4C12
chr11	34645000	chr11	59635000	1.18E-02	super_enhancer	promoter	TCN1
chr11	34645000	chr11	118215000	4.34E-03	super_enhancer	promoter	CD3G
chr11	34655000	chr11	61295000	1.29E-02	super_enhancer	promoter	RP11-286N22.16
chr11	34655000	chr11	66105000	1.15E-02	super_enhancer	promoter	RIN1
chr11	34655000	chr11	77525000	6.84E-03	super_enhancer	promoter	AP000580.1
chr11	35145000	chr11	40135000	3.31E-02	super_enhancer	promoter	RP11-454H19.2
chr11	35145000	chr11	66105000	1.32E-02	super_enhancer	promoter	RIN1
chr11	35145000	chr11	69635000	9.06E-03	super_enhancer	promoter	FGF3
chr11	35145000	chr11	74545000	7.61E-03	super_enhancer	promoter	AP000560.3
chr11	35145000	chr11	107515000	3.94E-03	super_enhancer	promoter	AP000889.1
chr11	35155000	chr11	43755000	2.44E-02	super_enhancer	promoter	PHBP2
chr11	35155000	chr11	47375000	3.23E-02	super_enhancer	promoter	MYBPC3
chr11	35155000	chr11	57195000	1.60E-02	super_enhancer	promoter	SLC43A3

chr11	35155000	chr11	57405000	1.37E-02	super_enhancer	promoter	AP000662.4
chr11	35155000	chr11	77025000	8.09E-03	super_enhancer	promoter	TOMM20P1
chr11	35155000	chr11	77775000	8.40E-03	super_enhancer	promoter	THRSP
chr11	35155000	chr11	85565000	6.45E-03	super_enhancer	promoter	CCDC83
chr11	35155000	chr11	104995000	3.42E-03	super_enhancer	promoter	CASP1P1
chr11	35155000	chr11	123635000	3.12E-03	super_enhancer	promoter	RNU1-21P
chr11	35165000	chr11	35395000	3.45E-02	super_enhancer	promoter	AC090625.1
chr11	35165000	chr11	59515000	1.75E-02	super_enhancer	promoter	OR10V2P
chr11	35165000	chr11	64785000	1.74E-02	super_enhancer	promoter	MIR6879
chr11	35165000	chr11	76155000	1.16E-02	super_enhancer	promoter	C11orf30
chr11	35165000	chr11	82445000	8.39E-03	super_enhancer	promoter	FAM181B
chr11	35165000	chr11	83355000	6.99E-03	super_enhancer	promoter	RP11-160H12.2
chr11	35165000	chr11	100665000	7.25E-03	super_enhancer	promoter	AC015600.1
chr11	35165000	chr11	123325000	4.15E-03	super_enhancer	promoter	GRAMD1B
chr11	35175000	chr11	45535000	2.76E-02	super_enhancer	promoter	RP11-430H10.4
chr11	35175000	chr11	71825000	9.55E-03	super_enhancer	promoter	ANAPC15
chr11	35395000	chr11	73385000	7.29E-03	promoter	super_enhancer	AC090625.1
chr11	35965000	chr11	48015000	3.25E-02	promoter	super_enhancer	LDLRAD3
chr11	35965000	chr11	63675000	1.25E-02	promoter	super_enhancer	LDLRAD3
chr11	35965000	chr11	69345000	1.37E-02	promoter	super_enhancer	LDLRAD3
chr11	36615000	chr11	47925000	3.22E-02	promoter	super_enhancer	C11orf74
chr11	39895000	chr11	47885000	2.74E-02	promoter	super_enhancer	RP11-810F22.1
chr11	39895000	chr11	47965000	2.80E-02	promoter	super_enhancer	RP11-810F22.1
chr11	39895000	chr11	63655000	1.48E-02	promoter	super_enhancer	RP11-810F22.1

chr11	41415000	chr11	66015000	1.48E-02	promoter	super_enhancer	RP11-63C8.1
chr11	41415000	chr11	67285000	1.51E-02	promoter	super_enhancer	RP11-63C8.1
chr11	41735000	chr11	119685000	2.76E-03	promoter	super_enhancer	LINC01499
chr11	43755000	chr11	86725000	6.89E-03	promoter	super_enhancer	PHBP2
chr11	44585000	chr11	57315000	2.63E-02	promoter	super_enhancer	CD82
chr11	44585000	chr11	62505000	2.45E-02	promoter	super_enhancer	CD82
chr11	44995000	chr11	62495000	2.60E-02	promoter	super_enhancer	AC068858.1
chr11	44995000	chr11	63675000	1.81E-02	promoter	super_enhancer	AC068858.1
chr11	45535000	chr11	65425000	3.04E-02	promoter	super_enhancer	RP11-430H10.4
chr11	45565000	chr11	65505000	2.61E-02	promoter	super_enhancer	RP11-958J22.1
chr11	45565000	chr11	94745000	1.17E-02	promoter	super_enhancer	RP11-958J22.1
chr11	45825000	chr11	46245000	3.81E-02	promoter	super_enhancer	SLC35C1
chr11	45825000	chr11	61685000	2.62E-02	promoter	super_enhancer	SLC35C1
chr11	45825000	chr11	65485000	3.17E-02	promoter	super_enhancer	SLC35C1
chr11	45825000	chr11	69335000	1.93E-02	promoter	super_enhancer	SLC35C1
chr11	45835000	chr11	47995000	3.98E-02	promoter	super_enhancer	CTD-2210P24.6
chr11	45835000	chr11	61685000	2.92E-02	promoter	super_enhancer	CTD-2210P24.6
chr11	45835000	chr11	64095000	2.51E-02	promoter	super_enhancer	CTD-2210P24.6
chr11	45835000	chr11	69355000	2.27E-02	promoter	super_enhancer	CTD-2210P24.6
chr11	45835000	chr11	123445000	5.21E-03	promoter	super_enhancer	CTD-2210P24.6
chr11	46135000	chr11	62495000	2.24E-02	promoter	super_enhancer	AC024475.1
chr11	46135000	chr11	64245000	2.26E-02	promoter	super_enhancer	AC024475.1
chr11	46135000	chr11	66005000	2.25E-02	promoter	super_enhancer	AC024475.1
chr11	46135000	chr11	86755000	7.44E-03	promoter	super_enhancer	AC024475.1

chr11	46235000	chr11	56785000	2.16E-02	super_enhancer	promoter	OR5AK1P
chr11	46235000	chr11	59845000	2.12E-02	super_enhancer	promoter	RP11-736I10.2
chr11	46235000	chr11	61975000	3.69E-02	super_enhancer	promoter	SCGB2A1
chr11	46235000	chr11	64135000	3.18E-02	super_enhancer	promoter	MIR1237
chr11	46235000	chr11	64685000	3.13E-02	super_enhancer	promoter	ATG2A
chr11	46235000	chr11	66045000	3.62E-02	super_enhancer	promoter	RP11-867G23.4
chr11	46235000	chr11	67055000	2.58E-02	super_enhancer	promoter	ANKRD13D
chr11	46235000	chr11	69185000	2.20E-02	super_enhancer	promoter	RP11-211G23.2
chr11	46235000	chr11	74975000	1.65E-02	super_enhancer	promoter	CTD-2562J17.6
chr11	46235000	chr11	77775000	1.66E-02	super_enhancer	promoter	THRSP
chr11	46235000	chr11	85395000	1.52E-02	super_enhancer	promoter	CREBZF
chr11	46235000	chr11	93455000	1.06E-02	super_enhancer	promoter	SCARNA9
chr11	46235000	chr11	120895000	4.66E-03	super_enhancer	promoter	TBCEL
chr11	46235000	chr11	126115000	5.56E-03	super_enhancer	promoter	RN7SL351P
chr11	46245000	chr11	59855000	1.97E-02	super_enhancer	promoter	MS4A2
chr11	46245000	chr11	61195000	2.50E-02	super_enhancer	promoter	RP11-286N22.6
chr11	46245000	chr11	67385000	1.98E-02	super_enhancer	promoter	DOC2GP
chr11	46245000	chr11	73915000	1.01E-02	super_enhancer	promoter	AP000577.2
chr11	46245000	chr11	89865000	7.36E-03	super_enhancer	promoter	NAALAD2
chr11	46245000	chr11	112835000	4.83E-03	super_enhancer	promoter	RP11-629G13.1
chr11	46365000	chr11	58745000	1.80E-02	super_enhancer	promoter	GLYATL1P1
chr11	46365000	chr11	67275000	2.53E-02	super_enhancer	promoter	PITPNM1
chr11	46365000	chr11	67805000	2.20E-02	super_enhancer	promoter	TCIRG1
chr11	46365000	chr11	74165000	1.89E-02	super_enhancer	promoter	RP11-702H23.6

chr11	46365000	chr11	92955000	6.58E-03	super_enhancer	promoter	RP11-573M3.4
chr11	46365000	chr11	94245000	1.02E-02	super_enhancer	promoter	C11orf97
chr11	46365000	chr11	130165000	4.47E-03	super_enhancer	promoter	DDX18P5
chr11	46555000	chr11	59855000	1.98E-02	super_enhancer	promoter	MS4A2
chr11	46555000	chr11	89865000	7.33E-03	super_enhancer	promoter	NAALAD2
chr11	46555000	chr11	125615000	4.02E-03	super_enhancer	promoter	PATE1
chr11	46565000	chr11	57415000	2.71E-02	super_enhancer	promoter	CLP1
chr11	46565000	chr11	59635000	3.18E-04	super_enhancer	promoter	TCN1
chr11	46565000	chr11	64915000	2.60E-02	super_enhancer	promoter	RNU2-23P
chr11	46565000	chr11	105945000	5.46E-03	super_enhancer	promoter	AASDHPPT
chr11	46565000	chr11	118465000	6.18E-03	super_enhancer	promoter	RNU6-1157P
chr11	46775000	chr11	61725000	2.38E-02	promoter	super_enhancer	MIR5582
chr11	46775000	chr11	64095000	2.20E-02	promoter	super_enhancer	MIR5582
chr11	46775000	chr11	65295000	3.03E-02	promoter	super_enhancer	MIR5582
chr11	46785000	chr11	128185000	2.60E-03	promoter	super_enhancer	SNORD67
chr11	47235000	chr11	61685000	2.76E-02	promoter	super_enhancer	DDB2
chr11	47235000	chr11	64245000	2.82E-02	promoter	super_enhancer	DDB2
chr11	47235000	chr11	65305000	3.04E-02	promoter	super_enhancer	DDB2
chr11	47235000	chr11	69305000	1.99E-02	promoter	super_enhancer	DDB2
chr11	47235000	chr11	72755000	1.32E-02	promoter	super_enhancer	DDB2
chr11	47235000	chr11	120215000	4.69E-03	promoter	super_enhancer	DDB2
chr11	47295000	chr11	72755000	1.35E-02	promoter	super_enhancer	RP11-17G12.3
chr11	47375000	chr11	94745000	1.19E-02	promoter	super_enhancer	MYBPC3
chr11	47375000	chr11	129645000	5.01E-03	promoter	super_enhancer	MYBPC3

chr11	47665000	chr11	57315000	3.26E-02	promoter	super_enhancer	MTCH2
chr11	47665000	chr11	57325000	3.31E-02	promoter	super_enhancer	MTCH2
chr11	47665000	chr11	59545000	2.91E-02	promoter	super_enhancer	MTCH2
chr11	47665000	chr11	61715000	2.96E-02	promoter	super_enhancer	MTCH2
chr11	47665000	chr11	66315000	3.30E-02	promoter	super_enhancer	MTCH2
chr11	47765000	chr11	60635000	3.63E-02	super_enhancer	promoter	ZP1
chr11	47765000	chr11	61355000	3.49E-02	super_enhancer	promoter	RP11-855O10.2
chr11	47765000	chr11	68275000	2.04E-02	super_enhancer	promoter	AP000807.1
chr11	47765000	chr11	71305000	1.76E-02	super_enhancer	promoter	OR7E87P
chr11	47765000	chr11	78035000	1.15E-02	super_enhancer	promoter	RP11-452H21.1
chr11	47765000	chr11	111895000	4.83E-03	super_enhancer	promoter	DLAT
chr11	47765000	chr11	124085000	3.21E-03	super_enhancer	promoter	OR8G3P
chr11	47855000	chr11	58135000	1.68E-02	super_enhancer	promoter	OR5B1P
chr11	47855000	chr11	58395000	2.57E-02	super_enhancer	promoter	AP001350.4
chr11	47855000	chr11	59335000	2.29E-02	super_enhancer	promoter	AP000442.4
chr11	47855000	chr11	67385000	1.84E-02	super_enhancer	promoter	DOC2GP
chr11	47855000	chr11	73115000	1.47E-02	super_enhancer	promoter	RP11-809N8.4
chr11	47855000	chr11	74545000	1.21E-02	super_enhancer	promoter	AP000560.3
chr11	47855000	chr11	86085000	1.07E-02	super_enhancer	promoter	CCDC81
chr11	47855000	chr11	86385000	1.13E-02	super_enhancer	promoter	ME3
chr11	47855000	chr11	101785000	5.50E-03	super_enhancer	promoter	CEP126
chr11	47865000	chr11	58395000	3.35E-02	super_enhancer	promoter	AP001350.4
chr11	47865000	chr11	119185000	6.28E-03	super_enhancer	promoter	MIR6756
chr11	47865000	chr11	119475000	6.06E-03	super_enhancer	promoter	KRT8P7

chr11	47875000	chr11	58135000	2.08E-02	super_enhancer	promoter	OR5B1P
chr11	47875000	chr11	86385000	1.40E-02	super_enhancer	promoter	ME3
chr11	47875000	chr11	93755000	1.15E-02	super_enhancer	promoter	HEPHL1
chr11	47875000	chr11	121235000	4.22E-03	super_enhancer	promoter	BMPR1APS2
chr11	47885000	chr11	56615000	2.28E-02	super_enhancer	promoter	AP000479.1
chr11	47885000	chr11	64135000	2.57E-02	super_enhancer	promoter	MIR1237
chr11	47885000	chr11	67085000	1.89E-02	super_enhancer	promoter	RAD9A
chr11	47885000	chr11	67805000	2.03E-02	super_enhancer	promoter	TCIRG1
chr11	47885000	chr11	83355000	8.62E-03	super_enhancer	promoter	RP11-160H12.2
chr11	47885000	chr11	85635000	1.03E-02	super_enhancer	promoter	RP11-90K17.2
chr11	47905000	chr11	56125000	2.26E-02	super_enhancer	promoter	RPL5P29
chr11	47905000	chr11	59385000	2.75E-02	super_enhancer	promoter	OSBP
chr11	47905000	chr11	61735000	2.66E-02	super_enhancer	promoter	FTH1
chr11	47905000	chr11	66115000	3.46E-02	super_enhancer	promoter	B4GAT1
chr11	47905000	chr11	89955000	1.03E-02	super_enhancer	promoter	RP11-121L10.2
chr11	47905000	chr11	122525000	5.34E-03	super_enhancer	promoter	UBASH3B
chr11	47915000	chr11	56615000	2.14E-02	super_enhancer	promoter	AP000479.1
chr11	47915000	chr11	59515000	2.44E-02	super_enhancer	promoter	OR10V2P
chr11	47915000	chr11	81555000	5.17E-03	super_enhancer	promoter	RP11-876F14.2
chr11	47915000	chr11	85635000	9.63E-03	super_enhancer	promoter	RP11-90K17.2
chr11	47925000	chr11	55705000	2.80E-02	super_enhancer	promoter	OR5I1
chr11	47925000	chr11	57405000	3.23E-02	super_enhancer	promoter	AP000662.4
chr11	47925000	chr11	59845000	2.15E-02	super_enhancer	promoter	RP11-736I10.2
chr11	47925000	chr11	82355000	9.53E-03	super_enhancer	promoter	RP11-179A16.2

chr11	47925000	chr11	85635000	1.28E-02	super_enhancer	promoter	RP11-90K17.2
chr11	47925000	chr11	118175000	5.90E-03	super_enhancer	promoter	CD3E
chr11	47935000	chr11	61295000	3.21E-02	super_enhancer	promoter	RP11-286N22.16
chr11	47935000	chr11	61355000	3.35E-02	super_enhancer	promoter	RP11-855O10.2
chr11	47935000	chr11	64765000	2.91E-02	super_enhancer	promoter	BATF2
chr11	47935000	chr11	94135000	9.57E-03	super_enhancer	promoter	GPR83
chr11	47935000	chr11	123985000	3.99E-03	super_enhancer	promoter	VWA5A
chr11	47935000	chr11	133995000	3.75E-03	super_enhancer	promoter	PTP4A2P2
chr11	47945000	chr11	55245000	2.59E-02	super_enhancer	promoter	OR4A14P
chr11	47945000	chr11	56125000	2.28E-02	super_enhancer	promoter	RPL5P29
chr11	47945000	chr11	57105000	2.85E-02	super_enhancer	promoter	P2RX3
chr11	47945000	chr11	58395000	3.22E-02	super_enhancer	promoter	AP001350.4
chr11	47945000	chr11	59335000	2.86E-02	super_enhancer	promoter	AP000442.4
chr11	47945000	chr11	61975000	3.52E-02	super_enhancer	promoter	SCGB2A1
chr11	47945000	chr11	74065000	1.39E-02	super_enhancer	promoter	HNRNPA1P40
chr11	47945000	chr11	77775000	1.55E-02	super_enhancer	promoter	THRSP
chr11	47945000	chr11	85395000	1.41E-02	super_enhancer	promoter	CREBZF
chr11	47945000	chr11	95725000	9.77E-03	super_enhancer	promoter	AP000870.1
chr11	47945000	chr11	111385000	5.71E-03	super_enhancer	promoter	C11orf88
chr11	47945000	chr11	118465000	6.11E-03	super_enhancer	promoter	RNU6-1157P
chr11	47955000	chr11	56395000	2.71E-02	super_enhancer	promoter	OR5M12P
chr11	47955000	chr11	57345000	4.06E-02	super_enhancer	promoter	RPS4XP13
chr11	47955000	chr11	76385000	1.70E-02	super_enhancer	promoter	RP11-672A2.3
chr11	47955000	chr11	109455000	5.49E-03	super_enhancer	promoter	RP11-708B6.2

chr11	47955000	chr11	115405000	5.41E-03	super_enhancer	promoter	RP11-136I14.2
chr11	47965000	chr11	69975000	1.57E-02	super_enhancer	promoter	RP11-805J14.3
chr11	47965000	chr11	76745000	1.38E-02	super_enhancer	promoter	B3GNT6
chr11	47965000	chr11	102165000	7.81E-03	super_enhancer	promoter	RP11-864G5.1
chr11	47985000	chr11	56065000	2.85E-02	super_enhancer	promoter	OR8I1P
chr11	47985000	chr11	56805000	2.37E-02	super_enhancer	promoter	OR5AK4P
chr11	47985000	chr11	59385000	2.98E-02	super_enhancer	promoter	OSBP
chr11	47985000	chr11	61355000	3.76E-02	super_enhancer	promoter	RP11-855O10.2
chr11	47985000	chr11	66405000	3.17E-02	super_enhancer	promoter	RBM4
chr11	47985000	chr11	86385000	1.52E-02	super_enhancer	promoter	ME3
chr11	47985000	chr11	95525000	1.13E-02	super_enhancer	promoter	FAM76B
chr11	47995000	chr11	66405000	3.23E-02	super_enhancer	promoter	RBM4
chr11	47995000	chr11	70245000	2.44E-02	super_enhancer	promoter	AP000487.5
chr11	47995000	chr11	72525000	1.86E-02	super_enhancer	promoter	ATG16L2
chr11	47995000	chr11	74975000	1.69E-02	super_enhancer	promoter	CTD-2562J17.6
chr11	47995000	chr11	85395000	1.54E-02	super_enhancer	promoter	CREBZF
chr11	47995000	chr11	88845000	5.60E-03	super_enhancer	promoter	AP001482.1
chr11	47995000	chr11	93585000	1.05E-02	super_enhancer	promoter	VSTM5
chr11	47995000	chr11	110585000	6.57E-03	super_enhancer	promoter	ARHGAP20
chr11	47995000	chr11	118135000	4.15E-03	super_enhancer	promoter	MPZL2
chr11	48005000	chr11	51515000	4.97E-02	super_enhancer	promoter	OR4C46
chr11	48005000	chr11	56755000	2.08E-02	super_enhancer	promoter	OR5AK2
chr11	48005000	chr11	58565000	2.21E-02	super_enhancer	promoter	TMA16P1
chr11	48005000	chr11	93465000	8.79E-03	super_enhancer	promoter	SNORD6

chr11	48015000	chr11	58135000	1.84E-02	super_enhancer	promoter	OR5B1P
chr11	48015000	chr11	59335000	2.51E-02	super_enhancer	promoter	AP000442.4
chr11	48015000	chr11	61525000	2.42E-02	super_enhancer	promoter	DKFZP434K028
chr11	48015000	chr11	67085000	1.95E-02	super_enhancer	promoter	RAD9A
chr11	48015000	chr11	67125000	2.49E-02	super_enhancer	promoter	POLD4
chr11	48015000	chr11	78035000	9.98E-03	super_enhancer	promoter	RP11-452H21.1
chr11	48025000	chr11	51515000	4.38E-02	super_enhancer	promoter	OR4C46
chr11	48025000	chr11	64885000	2.09E-02	super_enhancer	promoter	ZNHIT2
chr11	48025000	chr11	86085000	1.15E-02	super_enhancer	promoter	CCDC81
chr11	48285000	chr11	57325000	2.52E-02	promoter	super_enhancer	OR4X1
chr11	48345000	chr11	67025000	2.31E-02	promoter	super_enhancer	OR4C3
chr11	48345000	chr11	118925000	6.10E-03	promoter	super_enhancer	OR4C3
chr11	48375000	chr11	57325000	2.35E-02	promoter	super_enhancer	OR4C4P
chr11	48375000	chr11	126345000	3.84E-03	promoter	super_enhancer	OR4C4P
chr11	48455000	chr11	59545000	2.23E-02	promoter	super_enhancer	OR4C10P
chr11	48925000	chr11	120215000	2.47E-03	promoter	super_enhancer	RP11-56P9.10
chr11	49905000	chr11	86755000	3.62E-03	promoter	super_enhancer	RP11-163O19.3
chr11	49905000	chr11	118915000	2.25E-03	promoter	super_enhancer	RP11-163O19.3
chr11	49945000	chr11	118915000	2.62E-03	promoter	super_enhancer	OR4R3P
chr11	50005000	chr11	57325000	1.57E-02	promoter	super_enhancer	OR4C12
chr11	51395000	chr11	62465000	1.52E-02	promoter	super_enhancer	OR4A6P
chr11	51395000	chr11	69345000	9.91E-03	promoter	super_enhancer	OR4A6P
chr11	51395000	chr11	72745000	6.79E-03	promoter	super_enhancer	OR4A6P
chr11	55135000	chr11	66005000	2.27E-02	promoter	super_enhancer	OR4A15

chr11	55135000	chr11	122195000	2.64E-03	promoter	super_enhancer	OR4A15
chr11	55155000	chr11	67355000	1.77E-02	promoter	super_enhancer	OR4A9P
chr11	55155000	chr11	72755000	9.54E-03	promoter	super_enhancer	OR4A9P
chr11	55155000	chr11	126405000	2.72E-03	promoter	super_enhancer	OR4A9P
chr11	55605000	chr11	65915000	1.45E-02	promoter	super_enhancer	OR5D16
chr11	55705000	chr11	57305000	1.71E-02	promoter	super_enhancer	OR5I1
chr11	55705000	chr11	61695000	3.03E-02	promoter	super_enhancer	OR5I1
chr11	55705000	chr11	86155000	9.68E-03	promoter	super_enhancer	OR5I1
chr11	55865000	chr11	63655000	2.51E-02	promoter	super_enhancer	OR8I4P
chr11	55905000	chr11	66015000	2.18E-02	promoter	super_enhancer	OR8J3
chr11	56065000	chr11	61695000	3.33E-02	promoter	super_enhancer	OR8I1P
chr11	56065000	chr11	62015000	2.62E-02	promoter	super_enhancer	OR8I1P
chr11	56215000	chr11	72755000	7.87E-03	promoter	super_enhancer	OR5M4P
chr11	56345000	chr11	63685000	3.09E-02	promoter	super_enhancer	OR5M10
chr11	56395000	chr11	61725000	3.10E-02	promoter	super_enhancer	OR5M12P
chr11	56505000	chr11	86065000	6.96E-03	promoter	super_enhancer	RP11-100N3.2
chr11	56505000	chr11	122195000	2.28E-03	promoter	super_enhancer	RP11-100N3.2
chr11	56545000	chr11	62505000	3.63E-02	promoter	super_enhancer	OR5G1P
chr11	56545000	chr11	64865000	2.69E-02	promoter	super_enhancer	OR5G1P
chr11	56615000	chr11	62495000	3.28E-02	promoter	super_enhancer	AP000479.1
chr11	56615000	chr11	86785000	1.12E-02	promoter	super_enhancer	AP000479.1
chr11	56615000	chr11	123465000	3.43E-03	promoter	super_enhancer	AP000479.1
chr11	56755000	chr11	86735000	7.90E-03	promoter	super_enhancer	OR5AK2
chr11	56785000	chr11	62025000	2.75E-02	promoter	super_enhancer	OR5AK1P

chr11	56805000	chr11	86805000	5.98E-03	promoter	super_enhancer	OR5AK4P
chr11	57105000	chr11	57325000	1.94E-02	promoter	super_enhancer	P2RX3
chr11	57105000	chr11	64095000	2.75E-02	promoter	super_enhancer	P2RX3
chr11	57105000	chr11	118625000	3.60E-03	promoter	super_enhancer	P2RX3
chr11	57195000	chr11	67355000	3.01E-02	promoter	super_enhancer	SLC43A3
chr11	57195000	chr11	68125000	3.12E-02	promoter	super_enhancer	SLC43A3
chr11	57245000	chr11	65465000	4.19E-02	promoter	super_enhancer	RP11-624G17.3
chr11	57245000	chr11	65485000	4.73E-02	promoter	super_enhancer	RP11-624G17.3
chr11	57245000	chr11	118615000	4.92E-03	promoter	super_enhancer	RP11-624G17.3
chr11	57305000	chr11	59515000	1.40E-02	super_enhancer	promoter	OR10V2P
chr11	57305000	chr11	63035000	4.49E-02	super_enhancer	promoter	AP000438.4
chr11	57305000	chr11	66035000	3.46E-02	super_enhancer	promoter	RAB1B
chr11	57305000	chr11	72495000	3.46E-02	super_enhancer	promoter	MIR4692
chr11	57305000	chr11	77525000	1.98E-02	super_enhancer	promoter	AP000580.1
chr11	57305000	chr11	86385000	1.73E-02	super_enhancer	promoter	ME3
chr11	57315000	chr11	62575000	4.59E-02	super_enhancer	promoter	NXF1
chr11	57315000	chr11	107275000	3.76E-03	super_enhancer	promoter	SMARCE1P1
chr11	57315000	chr11	113645000	5.27E-03	super_enhancer	promoter	ZW10
chr11	57315000	chr11	130875000	3.18E-03	super_enhancer	promoter	RN7SL167P
chr11	57325000	chr11	57435000	1.58E-02	super_enhancer	promoter	ZDHHC5
chr11	57325000	chr11	64765000	3.87E-02	super_enhancer	promoter	BATF2
chr11	57325000	chr11	75525000	1.80E-02	super_enhancer	promoter	UVRAG
chr11	57325000	chr11	78035000	1.20E-02	super_enhancer	promoter	RP11-452H21.1
chr11	57365000	chr11	62035000	4.19E-02	promoter	super_enhancer	SERPING1

chr11	57365000	chr11	66855000	3.14E-02	promoter	super_enhancer	SERPING1
chr11	57365000	chr11	72745000	1.73E-02	promoter	super_enhancer	SERPING1
chr11	57365000	chr11	122165000	4.36E-03	promoter	super_enhancer	SERPING1
chr11	57405000	chr11	62005000	4.44E-02	promoter	super_enhancer	AP000662.4
chr11	57405000	chr11	128195000	3.37E-03	promoter	super_enhancer	AP000662.4
chr11	57415000	chr11	62465000	4.53E-02	promoter	super_enhancer	CLP1
chr11	57415000	chr11	65305000	3.36E-02	promoter	super_enhancer	CLP1
chr11	57415000	chr11	73335000	1.85E-02	promoter	super_enhancer	CLP1
chr11	57435000	chr11	65385000	3.60E-02	promoter	super_enhancer	ZDHHC5
chr11	57435000	chr11	86165000	1.35E-02	promoter	super_enhancer	ZDHHC5
chr11	57635000	chr11	73335000	2.44E-02	promoter	super_enhancer	OR5BA1P
chr11	57635000	chr11	111735000	6.31E-03	promoter	super_enhancer	OR5BA1P
chr11	58135000	chr11	63665000	2.80E-02	promoter	super_enhancer	OR5B1P
chr11	58395000	chr11	65275000	3.86E-02	promoter	super_enhancer	AP001350.4
chr11	58395000	chr11	65485000	4.89E-02	promoter	super_enhancer	AP001350.4
chr11	58565000	chr11	62555000	4.70E-02	promoter	super_enhancer	TMA16P1
chr11	58745000	chr11	62015000	4.81E-02	promoter	super_enhancer	GLYATL1P1
chr11	59245000	chr11	63665000	4.67E-02	promoter	super_enhancer	OR4D10
chr11	59245000	chr11	63685000	2.07E-03	promoter	super_enhancer	OR4D10
chr11	59245000	chr11	64095000	4.09E-02	promoter	super_enhancer	OR4D10
chr11	59335000	chr11	66315000	4.56E-02	promoter	super_enhancer	AP000442.4
chr11	59385000	chr11	69335000	2.44E-02	promoter	super_enhancer	OSBP
chr11	59475000	chr11	65275000	4.02E-02	promoter	super_enhancer	RN7SKP192
chr11	59475000	chr11	86175000	1.04E-02	promoter	super_enhancer	RN7SKP192

chr11	59475000	chr11	120215000	4.13E-03	promoter	super_enhancer	RN7SKP192
chr11	59515000	chr11	86735000	1.47E-02	promoter	super_enhancer	OR10V2P
chr11	59545000	chr11	61355000	2.51E-02	super_enhancer	promoter	RP11-855O10.2
chr11	59545000	chr11	69455000	2.61E-02	super_enhancer	promoter	CCND1
chr11	59555000	chr11	82925000	1.22E-02	super_enhancer	promoter	RP11-727A23.7
chr11	59555000	chr11	110225000	6.27E-03	super_enhancer	promoter	RP11-347E10.1
chr11	59635000	chr11	67285000	3.73E-02	promoter	super_enhancer	TCN1
chr11	59665000	chr11	72735000	2.01E-02	promoter	super_enhancer	SRD5A3P1
chr11	59705000	chr11	86805000	1.00E-02	promoter	super_enhancer	OOSP1
chr11	59845000	chr11	65315000	3.83E-02	promoter	super_enhancer	RP11-736I10.2
chr11	59845000	chr11	100745000	5.28E-03	promoter	super_enhancer	RP11-736I10.2
chr11	59855000	chr11	67375000	2.57E-02	promoter	super_enhancer	MS4A2
chr11	60635000	chr11	69275000	3.37E-02	promoter	super_enhancer	ZP1
chr11	60635000	chr11	86785000	2.15E-02	promoter	super_enhancer	ZP1
chr11	61195000	chr11	67375000	4.17E-02	promoter	super_enhancer	RP11-286N22.6
chr11	61195000	chr11	69445000	2.70E-02	promoter	super_enhancer	RP11-286N22.6
chr11	61195000	chr11	86785000	1.82E-02	promoter	super_enhancer	RP11-286N22.6
chr11	61195000	chr11	128205000	3.37E-03	promoter	super_enhancer	RP11-286N22.6
chr11	61295000	chr11	119685000	6.41E-03	promoter	super_enhancer	RP11-286N22.16
chr11	61355000	chr11	69295000	3.78E-02	promoter	super_enhancer	RP11-855O10.2
chr11	61355000	chr11	69345000	3.94E-02	promoter	super_enhancer	RP11-855O10.2
chr11	61525000	chr11	69415000	3.04E-02	promoter	super_enhancer	DKFZP434K028
chr11	61685000	chr11	73915000	1.67E-02	super_enhancer	promoter	AP000577.2
chr11	61685000	chr11	75645000	1.85E-02	super_enhancer	promoter	RNA5SP344

chr11	61685000	chr11	86035000	1.89E-02	super_enhancer	promoter	RN7SL225P
chr11	61685000	chr11	92085000	1.14E-02	super_enhancer	promoter	FAT3
chr11	61685000	chr11	93755000	1.39E-02	super_enhancer	promoter	HEPHL1
chr11	61685000	chr11	123325000	4.71E-03	super_enhancer	promoter	GRAMD1B
chr11	61695000	chr11	67805000	4.95E-02	super_enhancer	promoter	TCIRG1
chr11	61695000	chr11	71615000	2.32E-02	super_enhancer	promoter	AP000719.1
chr11	61695000	chr11	71935000	3.43E-02	super_enhancer	promoter	INPPL1
chr11	61695000	chr11	73915000	1.84E-02	super_enhancer	promoter	AP000577.2
chr11	61695000	chr11	113185000	7.70E-03	super_enhancer	promoter	RP11-839D17.3
chr11	61705000	chr11	67385000	4.43E-02	super_enhancer	promoter	DOC2GP
chr11	61705000	chr11	74545000	2.08E-02	super_enhancer	promoter	AP000560.3
chr11	61715000	chr11	63035000	4.40E-02	super_enhancer	promoter	AP000438.4
chr11	61715000	chr11	71185000	2.90E-02	super_enhancer	promoter	MIR6754
chr11	61715000	chr11	74395000	3.01E-02	super_enhancer	promoter	RN7SKP297
chr11	61715000	chr11	116645000	6.63E-03	super_enhancer	promoter	BUD13
chr11	61725000	chr11	69185000	3.18E-02	super_enhancer	promoter	RP11-211G23.2
chr11	61725000	chr11	76385000	2.15E-02	super_enhancer	promoter	RP11-672A2.3
chr11	61965000	chr11	61975000	2.65E-03	super_enhancer	promoter	SCGB2A1
chr11	61965000	chr11	62885000	4.32E-02	super_enhancer	promoter	RPL29P22
chr11	61965000	chr11	122485000	3.68E-03	super_enhancer	promoter	GLULP3
chr11	61975000	chr11	62025000	1.12E-02	promoter	super_enhancer	SCGB2A1
chr11	61975000	chr11	62555000	1.00E-02	promoter	super_enhancer	SCGB2A1
chr11	61975000	chr11	69345000	4.38E-02	promoter	super_enhancer	SCGB2A1
chr11	61975000	chr11	69435000	4.30E-02	promoter	super_enhancer	SCGB2A1

chr11	61975000	chr11	111735000	8.47E-03	promoter	super_enhancer	SCGB2A1
chr11	62005000	chr11	62195000	4.43E-02	super_enhancer	promoter	CTD-2531D15.4
chr11	62005000	chr11	63035000	8.94E-03	super_enhancer	promoter	AP000438.4
chr11	62005000	chr11	69455000	3.22E-02	super_enhancer	promoter	CCND1
chr11	62005000	chr11	73115000	2.49E-02	super_enhancer	promoter	RP11-809N8.4
chr11	62005000	chr11	91115000	1.02E-02	super_enhancer	promoter	RP11-843F11.1
chr11	62005000	chr11	124735000	6.09E-03	super_enhancer	promoter	ROBO3
chr11	62015000	chr11	67525000	3.10E-02	super_enhancer	promoter	RP11-655M14.4
chr11	62015000	chr11	73695000	2.27E-02	super_enhancer	promoter	UCP2
chr11	62015000	chr11	91115000	1.01E-02	super_enhancer	promoter	RP11-843F11.1
chr11	62015000	chr11	118465000	6.12E-03	super_enhancer	promoter	RNU6-1157P
chr11	62025000	chr11	71305000	2.65E-02	super_enhancer	promoter	OR7E87P
chr11	62035000	chr11	66445000	4.96E-02	super_enhancer	promoter	RBM4B
chr11	62035000	chr11	70125000	2.35E-02	super_enhancer	promoter	H2AFZP4
chr11	62035000	chr11	71165000	2.21E-02	super_enhancer	promoter	DHCR7
chr11	62045000	chr11	77525000	9.80E-03	super_enhancer	promoter	AP000580.1
chr11	62055000	chr11	70125000	1.79E-02	super_enhancer	promoter	H2AFZP4
chr11	62105000	chr11	67375000	4.80E-02	promoter	super_enhancer	ASRGL1
chr11	62105000	chr11	69445000	2.91E-02	promoter	super_enhancer	ASRGL1
chr11	62105000	chr11	122195000	4.41E-03	promoter	super_enhancer	ASRGL1
chr11	62195000	chr11	62475000	4.99E-02	promoter	super_enhancer	CTD-2531D15.4
chr11	62195000	chr11	62555000	4.32E-02	promoter	super_enhancer	CTD-2531D15.4
chr11	62195000	chr11	69425000	3.91E-02	promoter	super_enhancer	CTD-2531D15.4
chr11	62195000	chr11	73315000	3.36E-02	promoter	super_enhancer	CTD-2531D15.4

chr11	62195000	chr11	86725000	1.80E-02	promoter	super_enhancer	CTD-2531D15.4
chr11	62195000	chr11	124895000	3.67E-03	promoter	super_enhancer	CTD-2531D15.4
chr11	62305000	chr11	111725000	6.50E-03	promoter	super_enhancer	RP11-864I4.3
chr11	62325000	chr11	69325000	3.30E-02	promoter	super_enhancer	AHNAK
chr11	62325000	chr11	119685000	5.37E-03	promoter	super_enhancer	AHNAK
chr11	62335000	chr11	122185000	5.14E-03	promoter	super_enhancer	MIR6747
chr11	62415000	chr11	73385000	2.20E-02	promoter	super_enhancer	GANAB
chr11	62465000	chr11	63405000	2.86E-02	super_enhancer	promoter	RP11-697H9.2
chr11	62465000	chr11	70125000	3.66E-02	super_enhancer	promoter	H2AFZP4
chr11	62465000	chr11	85165000	1.68E-02	super_enhancer	promoter	RP11-482L11.1
chr11	62465000	chr11	87035000	1.79E-02	super_enhancer	promoter	RP11-777F6.3
chr11	62465000	chr11	117005000	4.60E-03	super_enhancer	promoter	RP11-109L13.1
chr11	62475000	chr11	62705000	2.28E-02	super_enhancer	promoter	RN7SL259P
chr11	62475000	chr11	72495000	3.56E-02	super_enhancer	promoter	MIR4692
chr11	62475000	chr11	82445000	1.49E-02	super_enhancer	promoter	FAM181B
chr11	62475000	chr11	113185000	6.50E-03	super_enhancer	promoter	RP11-839D17.3
chr11	62475000	chr11	116685000	5.57E-03	super_enhancer	promoter	AP006216.5
chr11	62485000	chr11	64085000	1.22E-02	super_enhancer	promoter	PRDX5
chr11	62485000	chr11	71185000	2.16E-02	super_enhancer	promoter	MIR6754
chr11	62485000	chr11	80475000	6.16E-03	super_enhancer	promoter	RP11-686G23.2
chr11	62485000	chr11	129685000	2.91E-03	super_enhancer	promoter	TMEM45B
chr11	62495000	chr11	63405000	2.65E-02	super_enhancer	promoter	RP11-697H9.2
chr11	62495000	chr11	63995000	7.04E-03	super_enhancer	promoter	TRPT1
chr11	62495000	chr11	70165000	3.48E-02	super_enhancer	promoter	CTA-797E19.1

chr11	62495000	chr11	92955000	9.17E-03	super_enhancer	promoter	RP11-573M3.4
chr11	62505000	chr11	72395000	3.42E-02	super_enhancer	promoter	ARAP1-AS1
chr11	62505000	chr11	76495000	3.14E-02	super_enhancer	promoter	RP11-21L23.3
chr11	62505000	chr11	118965000	6.91E-03	super_enhancer	promoter	AP003391.1
chr11	62555000	chr11	68815000	4.92E-02	super_enhancer	promoter	TPCN2
chr11	62555000	chr11	77525000	2.29E-02	super_enhancer	promoter	AP000580.1
chr11	62555000	chr11	118175000	6.49E-03	super_enhancer	promoter	CD3E
chr11	62555000	chr11	122485000	5.71E-03	super_enhancer	promoter	GLULP3
chr11	62575000	chr11	63685000	5.68E-04	promoter	super_enhancer	NXF1
chr11	62585000	chr11	64095000	4.07E-02	promoter	super_enhancer	RNU6-118P
chr11	62885000	chr11	64245000	4.21E-02	promoter	super_enhancer	RPL29P22
chr11	62885000	chr11	107835000	5.30E-03	promoter	super_enhancer	RPL29P22
chr11	62905000	chr11	86165000	1.90E-02	promoter	super_enhancer	SLC22A10
chr11	63035000	chr11	86745000	1.47E-02	promoter	super_enhancer	AP000438.4
chr11	63385000	chr11	69305000	3.88E-02	promoter	super_enhancer	PLA2G16
chr11	63385000	chr11	86155000	1.76E-02	promoter	super_enhancer	PLA2G16
chr11	63385000	chr11	119685000	5.35E-03	promoter	super_enhancer	PLA2G16
chr11	63405000	chr11	123475000	5.90E-03	promoter	super_enhancer	RP11-697H9.2
chr11	63605000	chr11	69325000	4.64E-02	promoter	super_enhancer	MARK2
chr11	63605000	chr11	69435000	4.75E-02	promoter	super_enhancer	MARK2
chr11	63655000	chr11	71165000	4.00E-02	super_enhancer	promoter	DHCR7
chr11	63655000	chr11	86005000	1.94E-02	super_enhancer	promoter	SETP17
chr11	63655000	chr11	118795000	6.94E-03	super_enhancer	promoter	UPK2
chr11	63665000	chr11	70245000	4.22E-02	super_enhancer	promoter	AP000487.5

chr11	63665000	chr11	77525000	2.26E-02	super_enhancer	promoter	AP000580.1
chr11	63665000	chr11	82905000	1.45E-02	super_enhancer	promoter	RP11-727A23.5
chr11	63665000	chr11	125035000	5.78E-03	super_enhancer	promoter	PKNOX2-AS1
chr11	63665000	chr11	130735000	3.83E-03	super_enhancer	promoter	RP11-890B15.2
chr11	63675000	chr11	63935000	1.53E-02	super_enhancer	promoter	MACROD1
chr11	63675000	chr11	74395000	2.50E-02	super_enhancer	promoter	RN7SKP297
chr11	63675000	chr11	77775000	1.78E-02	super_enhancer	promoter	THRSP
chr11	63675000	chr11	87035000	1.27E-02	super_enhancer	promoter	RP11-777F6.3
chr11	63675000	chr11	94245000	1.07E-02	super_enhancer	promoter	C11orf97
chr11	63675000	chr11	115405000	4.73E-03	super_enhancer	promoter	RP11-136I14.2
chr11	63685000	chr11	71515000	3.74E-02	super_enhancer	promoter	AP002495.1
chr11	63685000	chr11	72505000	4.44E-02	super_enhancer	promoter	ARAP1
chr11	63685000	chr11	73695000	3.64E-02	super_enhancer	promoter	UCP2
chr11	63685000	chr11	75645000	2.75E-02	super_enhancer	promoter	RNA5SP344
chr11	63685000	chr11	82795000	2.49E-02	super_enhancer	promoter	RP11-113K21.6
chr11	63685000	chr11	89955000	1.84E-02	super_enhancer	promoter	RP11-121L10.2
chr11	63685000	chr11	93475000	1.69E-02	super_enhancer	promoter	C11orf54
chr11	63685000	chr11	96075000	1.98E-02	super_enhancer	promoter	MIR1260B
chr11	63935000	chr11	68015000	2.48E-03	promoter	super_enhancer	MACROD1
chr11	63995000	chr11	86785000	2.43E-02	promoter	super_enhancer	TRPT1
chr11	64015000	chr11	64095000	2.44E-02	promoter	super_enhancer	PPP1R14B
chr11	64015000	chr11	69445000	4.51E-02	promoter	super_enhancer	PPP1R14B
chr11	64015000	chr11	73325000	3.20E-02	promoter	super_enhancer	PPP1R14B
chr11	64085000	chr11	64095000	1.44E-02	promoter	super_enhancer	PRDX5

chr11	64085000	chr11	69305000	4.14E-02	promoter	super_enhancer	PRDX5
chr11	64085000	chr11	129845000	3.81E-03	promoter	super_enhancer	PRDX5
chr11	64095000	chr11	70125000	3.87E-02	super_enhancer	promoter	H2AFZP4
chr11	64095000	chr11	93585000	1.19E-02	super_enhancer	promoter	VSTM5
chr11	64095000	chr11	118135000	4.16E-03	super_enhancer	promoter	MPZL2
chr11	64095000	chr11	121875000	4.10E-03	super_enhancer	promoter	RNU6-256P
chr11	64135000	chr11	66855000	1.05E-02	promoter	super_enhancer	MIR1237
chr11	64245000	chr11	124735000	8.00E-03	super_enhancer	promoter	ROBO3
chr11	64685000	chr11	64855000	2.40E-02	promoter	super_enhancer	ATG2A
chr11	64685000	chr11	114305000	6.74E-03	promoter	super_enhancer	ATG2A
chr11	64765000	chr11	64855000	3.46E-02	promoter	super_enhancer	BATF2
chr11	64765000	chr11	64865000	3.31E-02	promoter	super_enhancer	BATF2
chr11	64795000	chr11	64865000	1.55E-06	promoter	super_enhancer	AP000436.4
chr11	64855000	chr11	71135000	1.14E-03	super_enhancer	promoter	FLJ42102
chr11	64855000	chr11	71725000	2.91E-02	super_enhancer	promoter	RP11-849H4.4
chr11	64855000	chr11	107485000	1.05E-02	super_enhancer	promoter	AP000889.3
chr11	64855000	chr11	111885000	7.55E-03	super_enhancer	promoter	RP11-708L7.6
chr11	64865000	chr11	72065000	4.05E-02	super_enhancer	promoter	RP11-45F15.2
chr11	64865000	chr11	73695000	3.12E-02	super_enhancer	promoter	UCP2
chr11	64865000	chr11	118445000	6.84E-03	super_enhancer	promoter	IFT46
chr11	65135000	chr11	65275000	1.59E-03	promoter	super_enhancer	RP11-867O8.5
chr11	65135000	chr11	65285000	3.65E-04	promoter	super_enhancer	RP11-867O8.5
chr11	65265000	chr11	96075000	1.78E-02	super_enhancer	promoter	MIR1260B
chr11	65275000	chr11	67805000	1.10E-02	super_enhancer	promoter	TCIRG1

chr11	65275000	chr11	72525000	3.54E-02	super_enhancer	promoter	ATG16L2
chr11	65275000	chr11	76745000	2.78E-02	super_enhancer	promoter	B3GNT6
chr11	65275000	chr11	77525000	2.71E-02	super_enhancer	promoter	AP000580.1
chr11	65275000	chr11	118465000	7.88E-03	super_enhancer	promoter	RNU6-1157P
chr11	65285000	chr11	118465000	9.12E-03	super_enhancer	promoter	RNU6-1157P
chr11	65285000	chr11	126085000	5.36E-03	super_enhancer	promoter	RNU4-86P
chr11	65295000	chr11	67395000	4.08E-02	super_enhancer	promoter	RP11-655M14.13
chr11	65295000	chr11	85565000	2.29E-02	super_enhancer	promoter	CCDC83
chr11	65295000	chr11	94135000	1.86E-02	super_enhancer	promoter	GPR83
chr11	65295000	chr11	118965000	9.56E-03	super_enhancer	promoter	AP003391.1
chr11	65295000	chr11	125495000	7.81E-03	super_enhancer	promoter	CHEK1
chr11	65305000	chr11	93465000	1.57E-02	super_enhancer	promoter	SNORD6
chr11	65305000	chr11	112035000	9.18E-03	super_enhancer	promoter	IL18
chr11	65315000	chr11	66035000	3.71E-03	super_enhancer	promoter	RAB1B
chr11	65315000	chr11	66105000	2.35E-02	super_enhancer	promoter	RIN1
chr11	65315000	chr11	85905000	2.48E-02	super_enhancer	promoter	RP11-12D16.2
chr11	65315000	chr11	94245000	1.86E-02	super_enhancer	promoter	C11orf97
chr11	65315000	chr11	123815000	5.71E-03	super_enhancer	promoter	OR6T1
chr11	65385000	chr11	87035000	2.20E-02	super_enhancer	promoter	RP11-777F6.3
chr11	65385000	chr11	93585000	1.62E-02	super_enhancer	promoter	VSTM5
chr11	65385000	chr11	94475000	1.99E-02	super_enhancer	promoter	RP11-867G2.8
chr11	65385000	chr11	107275000	6.35E-03	super_enhancer	promoter	SMARCE1P1
chr11	65385000	chr11	117075000	7.64E-03	super_enhancer	promoter	AP000892.6
chr11	65425000	chr11	65565000	2.91E-03	super_enhancer	promoter	RP11-770G2.5

chr11	65425000	chr11	73545000	4.67E-02	super_enhancer	promoter	RN7SKP243
chr11	65425000	chr11	76495000	4.80E-02	super_enhancer	promoter	RP11-21L23.3
chr11	65425000	chr11	77025000	3.51E-02	super_enhancer	promoter	TOMM20P1
chr11	65425000	chr11	93475000	1.89E-02	super_enhancer	promoter	C11orf54
chr11	65425000	chr11	93755000	2.25E-02	super_enhancer	promoter	HEPHL1
chr11	65465000	chr11	73545000	4.12E-02	super_enhancer	promoter	RN7SKP243
chr11	65465000	chr11	90015000	1.87E-02	super_enhancer	promoter	TUBAP2
chr11	65475000	chr11	112045000	4.39E-03	super_enhancer	promoter	BCO2
chr11	65485000	chr11	65565000	3.11E-02	super_enhancer	promoter	RP11-770G2.5
chr11	65485000	chr11	66105000	2.15E-02	super_enhancer	promoter	RIN1
chr11	65485000	chr11	71375000	3.68E-02	super_enhancer	promoter	AP003498.1
chr11	65485000	chr11	86005000	2.50E-02	super_enhancer	promoter	SETP17
chr11	65485000	chr11	116695000	1.05E-02	super_enhancer	promoter	APOA4
chr11	65485000	chr11	119055000	1.15E-02	super_enhancer	promoter	PDZD3
chr11	65495000	chr11	65565000	3.40E-02	super_enhancer	promoter	RP11-770G2.5
chr11	65495000	chr11	74395000	4.28E-02	super_enhancer	promoter	RN7SKP297
chr11	65505000	chr11	85905000	2.11E-02	super_enhancer	promoter	RP11-12D16.2
chr11	65515000	chr11	67055000	4.16E-02	super_enhancer	promoter	ANKRD13D
chr11	65515000	chr11	72065000	4.28E-02	super_enhancer	promoter	RP11-45F15.2
chr11	65515000	chr11	94375000	1.32E-02	super_enhancer	promoter	RP11-867G2.6
chr11	65515000	chr11	117185000	8.38E-03	super_enhancer	promoter	CEP164
chr11	65515000	chr11	129765000	5.65E-03	super_enhancer	promoter	NFRKB
chr11	65555000	chr11	65795000	2.73E-02	super_enhancer	promoter	CATSPER1
chr11	65555000	chr11	66045000	2.06E-02	super_enhancer	promoter	RP11-867G23.4

chr11	65555000	chr11	72505000	4.99E-02	super_enhancer	promoter	ARAP1
chr11	65555000	chr11	95525000	1.77E-02	super_enhancer	promoter	FAM76B
chr11	65555000	chr11	118215000	9.31E-03	super_enhancer	promoter	CD3G
chr11	65555000	chr11	118705000	9.67E-03	super_enhancer	promoter	SETP16
chr11	65565000	chr11	65615000	3.24E-03	promoter	super_enhancer	RP11-770G2.5
chr11	65565000	chr11	128195000	6.15E-03	promoter	super_enhancer	RP11-770G2.5
chr11	65615000	chr11	75525000	3.93E-02	super_enhancer	promoter	UVRAG
chr11	65615000	chr11	85645000	2.29E-02	super_enhancer	promoter	SLC25A1P1
chr11	65615000	chr11	89865000	1.64E-02	super_enhancer	promoter	NAALAD2
chr11	65615000	chr11	118195000	9.97E-03	super_enhancer	promoter	RP11-215H18.5
chr11	65615000	chr11	122935000	7.52E-03	super_enhancer	promoter	HSPA8
chr11	65795000	chr11	114295000	7.14E-03	promoter	super_enhancer	CATSPER1
chr11	65905000	chr11	71165000	4.96E-02	super_enhancer	promoter	DHCR7
chr11	65905000	chr11	80365000	1.59E-02	super_enhancer	promoter	ARL6IP1P3
chr11	65905000	chr11	101785000	9.34E-03	super_enhancer	promoter	CEP126
chr11	65905000	chr11	119205000	8.29E-03	super_enhancer	promoter	RNF26
chr11	65915000	chr11	72395000	4.61E-02	super_enhancer	promoter	ARAP1-AS1
chr11	65915000	chr11	129245000	5.57E-03	super_enhancer	promoter	BARX2
chr11	66005000	chr11	96075000	1.94E-02	super_enhancer	promoter	MIR1260B
chr11	66005000	chr11	121875000	5.55E-03	super_enhancer	promoter	RNU6-256P
chr11	66015000	chr11	85565000	2.17E-02	super_enhancer	promoter	CCDC83
chr11	66015000	chr11	88845000	9.38E-03	super_enhancer	promoter	AP001482.1
chr11	66015000	chr11	105805000	7.73E-03	super_enhancer	promoter	AP000673.1
chr11	66015000	chr11	113185000	9.92E-03	super_enhancer	promoter	RP11-839D17.3

chr11	66085000	chr11	86795000	1.83E-02	promoter	super_enhancer	CD248
chr11	66085000	chr11	122185000	6.43E-03	promoter	super_enhancer	CD248
chr11	66105000	chr11	86155000	2.55E-02	promoter	super_enhancer	RIN1
chr11	66315000	chr11	66405000	2.74E-02	super_enhancer	promoter	RBM4
chr11	66315000	chr11	66445000	1.57E-02	super_enhancer	promoter	RBM4B
chr11	66315000	chr11	85635000	2.56E-02	super_enhancer	promoter	RP11-90K17.2
chr11	66315000	chr11	94245000	2.12E-02	super_enhancer	promoter	C11orf97
chr11	66315000	chr11	102165000	1.64E-02	super_enhancer	promoter	RP11-864G5.1
chr11	66315000	chr11	126165000	7.43E-03	super_enhancer	promoter	RP11-712L6.7
chr11	66325000	chr11	96075000	2.08E-02	super_enhancer	promoter	MIR1260B
chr11	66325000	chr11	110225000	1.05E-02	super_enhancer	promoter	RP11-347E10.1
chr11	66325000	chr11	126085000	5.69E-03	super_enhancer	promoter	RNU4-86P
chr11	66445000	chr11	118915000	8.04E-03	promoter	super_enhancer	RBM4B
chr11	66855000	chr11	67085000	2.38E-02	super_enhancer	promoter	RAD9A
chr11	66855000	chr11	74395000	4.43E-02	super_enhancer	promoter	RN7SKP297
chr11	66855000	chr11	82795000	2.30E-02	super_enhancer	promoter	RP11-113K21.6
chr11	66855000	chr11	86085000	2.24E-02	super_enhancer	promoter	CCDC81
chr11	66855000	chr11	93325000	1.33E-04	super_enhancer	promoter	RP11-178H8.2
chr11	66855000	chr11	96515000	1.47E-02	super_enhancer	promoter	RP11-360K13.2
chr11	66855000	chr11	124505000	5.69E-03	super_enhancer	promoter	RNA5SP352
chr11	66855000	chr11	130185000	4.30E-03	super_enhancer	promoter	ZBTB44
chr11	66965000	chr11	67085000	8.60E-03	super_enhancer	promoter	RAD9A
chr11	66965000	chr11	100665000	1.30E-02	super_enhancer	promoter	AC015600.1
chr11	67025000	chr11	72525000	4.23E-02	super_enhancer	promoter	ATG16L2

chr11	67025000	chr11	118025000	7.40E-03	super_enhancer	promoter	SCN4B
chr11	67025000	chr11	120195000	6.86E-03	super_enhancer	promoter	TMEM136
chr11	67275000	chr11	123475000	7.87E-03	promoter	super_enhancer	PITPNM1
chr11	67285000	chr11	74545000	4.23E-02	super_enhancer	promoter	AP000560.3
chr11	67285000	chr11	86005000	2.57E-02	super_enhancer	promoter	SETP17
chr11	67285000	chr11	92805000	1.59E-02	super_enhancer	promoter	RP11-183A22.3
chr11	67285000	chr11	100665000	1.85E-02	super_enhancer	promoter	AC015600.1
chr11	67285000	chr11	107275000	7.28E-03	super_enhancer	promoter	SMARCE1P1
chr11	67285000	chr11	107485000	1.29E-02	super_enhancer	promoter	AP000889.3
chr11	67285000	chr11	121235000	7.09E-03	super_enhancer	promoter	BMPR1APS2
chr11	67285000	chr11	128395000	6.44E-03	super_enhancer	promoter	RP11-1007G5.2
chr11	67355000	chr11	94245000	1.69E-02	super_enhancer	promoter	C11orf97
chr11	67375000	chr11	86005000	1.91E-02	super_enhancer	promoter	SETP17
chr11	67375000	chr11	116645000	7.48E-03	super_enhancer	promoter	BUD13
chr11	67385000	chr11	73325000	4.23E-02	promoter	super_enhancer	DOC2GP
chr11	67395000	chr11	69445000	1.76E-02	promoter	super_enhancer	RP11-655M14.13
chr11	67395000	chr11	123475000	8.31E-03	promoter	super_enhancer	RP11-655M14.13
chr11	67505000	chr11	74545000	1.78E-02	super_enhancer	promoter	AP000560.3
chr11	67505000	chr11	118515000	3.83E-03	super_enhancer	promoter	MIR6716
chr11	67525000	chr11	120125000	4.89E-03	promoter	super_enhancer	RP11-655M14.4
chr11	67525000	chr11	122185000	3.99E-03	promoter	super_enhancer	RP11-655M14.4
chr11	67735000	chr11	118925000	5.74E-03	promoter	super_enhancer	RP5-901A4.5
chr11	67775000	chr11	118625000	6.80E-03	promoter	super_enhancer	ALDH3B1
chr11	67805000	chr11	69345000	4.04E-02	promoter	super_enhancer	TCIRG1

chr11	67805000	chr11	69355000	4.30E-02	promoter	super_enhancer	TCIRG1
chr11	68015000	chr11	85565000	2.02E-02	super_enhancer	promoter	CCDC83
chr11	68115000	chr11	74065000	3.91E-02	super_enhancer	promoter	HNRNPA1P40
chr11	68115000	chr11	74205000	4.69E-02	super_enhancer	promoter	LIPT2
chr11	68125000	chr11	93455000	1.69E-02	super_enhancer	promoter	SCARNA9
chr11	68125000	chr11	118445000	7.99E-03	super_enhancer	promoter	IFT46
chr11	69185000	chr11	69415000	1.49E-02	promoter	super_enhancer	RP11-211G23.2
chr11	69185000	chr11	129855000	5.69E-03	promoter	super_enhancer	RP11-211G23.2
chr11	69275000	chr11	74975000	3.70E-02	super_enhancer	promoter	CTD-2562J17.6
chr11	69275000	chr11	76155000	3.62E-02	super_enhancer	promoter	C11orf30
chr11	69275000	chr11	82795000	2.14E-02	super_enhancer	promoter	RP11-113K21.6
chr11	69285000	chr11	69455000	1.99E-02	super_enhancer	promoter	CCND1
chr11	69285000	chr11	118195000	6.62E-03	super_enhancer	promoter	RP11-215H18.5
chr11	69295000	chr11	73915000	3.60E-02	super_enhancer	promoter	AP000577.2
chr11	69295000	chr11	75115000	4.14E-02	super_enhancer	promoter	SNORD15B
chr11	69295000	chr11	102715000	9.51E-03	super_enhancer	promoter	MMP3
chr11	69305000	chr11	71165000	1.91E-02	super_enhancer	promoter	DHCR7
chr11	69305000	chr11	118175000	7.24E-03	super_enhancer	promoter	CD3E
chr11	69305000	chr11	118515000	7.19E-03	super_enhancer	promoter	MIR6716
chr11	69315000	chr11	73915000	4.86E-04	super_enhancer	promoter	AP000577.2
chr11	69315000	chr11	100665000	1.20E-02	super_enhancer	promoter	AC015600.1
chr11	69315000	chr11	102775000	6.41E-03	super_enhancer	promoter	RNU7-159P
chr11	69315000	chr11	116645000	6.44E-03	super_enhancer	promoter	BUD13
chr11	69325000	chr11	74545000	4.16E-02	super_enhancer	promoter	AP000560.3

chr11	69325000	chr11	116685000	6.96E-03	super_enhancer	promoter	AP006216.5
chr11	69325000	chr11	133715000	3.03E-03	super_enhancer	promoter	SPATA19
chr11	69345000	chr11	82795000	2.40E-02	super_enhancer	promoter	RP11-113K21.6
chr11	69345000	chr11	102715000	9.98E-03	super_enhancer	promoter	MMP3
chr11	69355000	chr11	109695000	7.09E-03	super_enhancer	promoter	RP11-262A12.1
chr11	69415000	chr11	71135000	2.30E-02	super_enhancer	promoter	FLJ42102
chr11	69415000	chr11	102715000	9.57E-03	super_enhancer	promoter	MMP3
chr11	69425000	chr11	87035000	1.90E-02	super_enhancer	promoter	RP11-777F6.3
chr11	69425000	chr11	93455000	1.39E-02	super_enhancer	promoter	SCARNA9
chr11	69425000	chr11	102165000	1.18E-02	super_enhancer	promoter	RP11-864G5.1
chr11	69425000	chr11	118965000	6.96E-03	super_enhancer	promoter	AP003391.1
chr11	69435000	chr11	78035000	2.31E-02	super_enhancer	promoter	RP11-452H21.1
chr11	69435000	chr11	85395000	2.45E-02	super_enhancer	promoter	CREBZF
chr11	69435000	chr11	126085000	4.70E-03	super_enhancer	promoter	RNU4-86P
chr11	69445000	chr11	69975000	4.69E-02	super_enhancer	promoter	RP11-805J14.3
chr11	69445000	chr11	77025000	2.72E-02	super_enhancer	promoter	TOMM20P1
chr11	69445000	chr11	85905000	1.97E-02	super_enhancer	promoter	RP11-12D16.2
chr11	69445000	chr11	117185000	7.49E-03	super_enhancer	promoter	CEP164
chr11	69445000	chr11	129245000	4.88E-03	super_enhancer	promoter	BARX2
chr11	69445000	chr11	134605000	2.99E-03	super_enhancer	promoter	RP11-469N6.1
chr11	69455000	chr11	86755000	1.63E-02	promoter	super_enhancer	CCND1
chr11	69455000	chr11	126345000	6.92E-03	promoter	super_enhancer	CCND1
chr11	69635000	chr11	111735000	7.52E-03	promoter	super_enhancer	FGF3
chr11	69975000	chr11	118615000	5.56E-03	promoter	super_enhancer	RP11-805J14.3

chr11	69975000	chr11	119695000	5.45E-03	promoter	super_enhancer	RP11-805J14.3
chr11	70035000	chr11	107835000	7.39E-03	promoter	super_enhancer	ANO1-AS1
chr11	70125000	chr11	129845000	4.90E-03	promoter	super_enhancer	H2AFZP4
chr11	70165000	chr11	86185000	1.99E-02	promoter	super_enhancer	CTA-797E19.1
chr11	71135000	chr11	86185000	2.10E-02	promoter	super_enhancer	FLJ42102
chr11	71135000	chr11	119695000	6.31E-03	promoter	super_enhancer	FLJ42102
chr11	71165000	chr11	107835000	8.08E-03	promoter	super_enhancer	DHCR7
chr11	71315000	chr11	120135000	5.37E-03	promoter	super_enhancer	KRTAP5-11
chr11	71515000	chr11	72745000	2.97E-02	promoter	super_enhancer	AP002495.1
chr11	71515000	chr11	86065000	1.80E-02	promoter	super_enhancer	AP002495.1
chr11	71815000	chr11	86185000	2.22E-02	promoter	super_enhancer	LAMTOR1
chr11	71935000	chr11	111735000	9.93E-03	promoter	super_enhancer	INPPL1
chr11	71935000	chr11	123465000	7.63E-03	promoter	super_enhancer	INPPL1
chr11	72395000	chr11	86735000	2.86E-02	promoter	super_enhancer	ARAP1-AS1
chr11	72505000	chr11	73315000	4.55E-02	promoter	super_enhancer	ARAP1
chr11	72505000	chr11	86175000	2.26E-02	promoter	super_enhancer	ARAP1
chr11	72735000	chr11	74165000	4.43E-02	super_enhancer	promoter	RP11-702H23.6
chr11	72745000	chr11	93395000	1.35E-02	super_enhancer	promoter	CEP295
chr11	72745000	chr11	93455000	1.38E-02	super_enhancer	promoter	SCARNA9
chr11	72755000	chr11	77845000	3.25E-02	super_enhancer	promoter	RNU6-126P
chr11	72975000	chr11	122165000	6.58E-03	promoter	super_enhancer	P2RY6
chr11	73315000	chr11	122295000	6.72E-03	super_enhancer	promoter	MIR100HG
chr11	73325000	chr11	86545000	2.11E-02	super_enhancer	promoter	OR7E13P
chr11	73335000	chr11	102165000	1.61E-02	super_enhancer	promoter	RP11-864G5.1

chr11	73335000	chr11	129765000	7.14E-03	super_enhancer	promoter	NFRKB
chr11	73335000	chr11	130185000	5.03E-03	super_enhancer	promoter	ZBTB44
chr11	73385000	chr11	111795000	7.70E-03	super_enhancer	promoter	CRYAB
chr11	73915000	chr11	86805000	1.30E-02	promoter	super_enhancer	AP000577.2
chr11	74205000	chr11	86165000	2.91E-02	promoter	super_enhancer	LIPT2
chr11	74395000	chr11	86725000	2.95E-02	promoter	super_enhancer	RN7SKP297
chr11	74395000	chr11	118615000	3.57E-05	promoter	super_enhancer	RN7SKP297
chr11	74545000	chr11	129635000	5.52E-03	promoter	super_enhancer	AP000560.3
chr11	75515000	chr11	86175000	2.46E-02	promoter	super_enhancer	RP11-535A19.1
chr11	75515000	chr11	94745000	2.22E-02	promoter	super_enhancer	RP11-535A19.1
chr11	75625000	chr11	86155000	2.82E-02	promoter	super_enhancer	RP11-263C24.1
chr11	76155000	chr11	128195000	6.35E-03	promoter	super_enhancer	C11orf30
chr11	76385000	chr11	114305000	7.97E-03	promoter	super_enhancer	RP11-672A2.3
chr11	76385000	chr11	129855000	6.97E-03	promoter	super_enhancer	RP11-672A2.3
chr11	76495000	chr11	86725000	3.46E-02	promoter	super_enhancer	RP11-21L23.3
chr11	76495000	chr11	122165000	8.66E-03	promoter	super_enhancer	RP11-21L23.3
chr11	76745000	chr11	122185000	6.70E-03	promoter	super_enhancer	B3GNT6
chr11	77525000	chr11	111735000	1.02E-02	promoter	super_enhancer	AP000580.1
chr11	78035000	chr11	114305000	6.04E-03	promoter	super_enhancer	RP11-452H21.1
chr11	80365000	chr11	86155000	3.58E-02	promoter	super_enhancer	ARL6IP1P3
chr11	80475000	chr11	86065000	1.92E-02	promoter	super_enhancer	RP11-686G23.2
chr11	80835000	chr11	128215000	3.26E-03	promoter	super_enhancer	AP003464.1
chr11	82355000	chr11	86755000	3.20E-02	promoter	super_enhancer	RP11-179A16.2
chr11	82445000	chr11	118925000	1.02E-02	promoter	super_enhancer	FAM181B

chr11	82445000	chr11	129845000	6.25E-03	promoter	super_enhancer	FAM181B
chr11	82925000	chr11	86755000	4.70E-02	promoter	super_enhancer	RP11-727A23.7
chr11	82925000	chr11	100745000	1.47E-02	promoter	super_enhancer	RP11-727A23.7
chr11	85395000	chr11	128185000	6.53E-03	promoter	super_enhancer	CREBZF
chr11	85565000	chr11	86065000	1.91E-02	promoter	super_enhancer	CCDC83
chr11	85635000	chr11	86065000	3.54E-02	promoter	super_enhancer	RP11-90K17.2
chr11	85635000	chr11	118925000	1.22E-02	promoter	super_enhancer	RP11-90K17.2
chr11	85635000	chr11	120135000	1.11E-02	promoter	super_enhancer	RP11-90K17.2
chr11	85645000	chr11	86065000	1.26E-03	promoter	super_enhancer	SLC25A1P1
chr11	85905000	chr11	128195000	8.47E-03	promoter	super_enhancer	RP11-12D16.2
chr11	85955000	chr11	107845000	1.80E-02	promoter	super_enhancer	EED
chr11	86005000	chr11	107855000	1.30E-02	promoter	super_enhancer	SETP17
chr11	86035000	chr11	86795000	4.72E-02	promoter	super_enhancer	RN7SL225P
chr11	86035000	chr11	119695000	1.19E-02	promoter	super_enhancer	RN7SL225P
chr11	86065000	chr11	93455000	3.28E-02	super_enhancer	promoter	SCARNA9
chr11	86065000	chr11	101455000	2.50E-02	super_enhancer	promoter	RP11-748H22.1
chr11	86065000	chr11	125035000	9.74E-03	super_enhancer	promoter	PKNOX2-AS1
chr11	86085000	chr11	111725000	1.48E-02	promoter	super_enhancer	CCDC81
chr11	86165000	chr11	86545000	1.98E-02	super_enhancer	promoter	OR7E13P
chr11	86165000	chr11	87035000	4.39E-02	super_enhancer	promoter	RP11-777F6.3
chr11	86175000	chr11	86545000	3.12E-02	super_enhancer	promoter	OR7E13P
chr11	86175000	chr11	93475000	3.04E-02	super_enhancer	promoter	C11orf54
chr11	86175000	chr11	94245000	3.19E-02	super_enhancer	promoter	C11orf97
chr11	86175000	chr11	100665000	2.39E-02	super_enhancer	promoter	AC015600.1

chr11	86175000	chr11	113845000	1.27E-02	super_enhancer	promoter	HTR3A
chr11	86185000	chr11	93465000	3.07E-02	super_enhancer	promoter	SNORD6
chr11	86185000	chr11	120195000	1.10E-02	super_enhancer	promoter	TMEM136
chr11	86725000	chr11	94135000	3.51E-02	super_enhancer	promoter	GPR83
chr11	86725000	chr11	102165000	2.39E-02	super_enhancer	promoter	RP11-864G5.1
chr11	86725000	chr11	102715000	1.82E-02	super_enhancer	promoter	MMP3
chr11	86725000	chr11	108465000	1.66E-02	super_enhancer	promoter	EXPH5
chr11	86725000	chr11	125035000	1.03E-02	super_enhancer	promoter	PKNOX2-AS1
chr11	86735000	chr11	91725000	3.97E-02	super_enhancer	promoter	RP11-806N19.2
chr11	86735000	chr11	93325000	4.51E-02	super_enhancer	promoter	RP11-178H8.2
chr11	86735000	chr11	93395000	4.11E-02	super_enhancer	promoter	CEP295
chr11	86735000	chr11	129765000	9.98E-03	super_enhancer	promoter	NFRKB
chr11	86745000	chr11	93755000	4.56E-02	super_enhancer	promoter	HEPHL1
chr11	86745000	chr11	107515000	1.79E-02	super_enhancer	promoter	AP000889.1
chr11	86745000	chr11	115605000	1.41E-02	super_enhancer	promoter	CTC-774J1.1
chr11	86755000	chr11	116645000	1.02E-02	super_enhancer	promoter	BUD13
chr11	86755000	chr11	122485000	8.06E-03	super_enhancer	promoter	GLULP3
chr11	86785000	chr11	95215000	4.00E-02	super_enhancer	promoter	RP11-338H14.1
chr11	86785000	chr11	105695000	1.83E-02	super_enhancer	promoter	RNU4-55P
chr11	86795000	chr11	87035000	3.62E-02	super_enhancer	promoter	RP11-777F6.3
chr11	86795000	chr11	95215000	2.79E-02	super_enhancer	promoter	RP11-338H14.1
chr11	86795000	chr11	134305000	4.85E-03	super_enhancer	promoter	RP11-627G23.1
chr11	86805000	chr11	87035000	8.26E-03	super_enhancer	promoter	RP11-777F6.3
chr11	86805000	chr11	93755000	3.50E-02	super_enhancer	promoter	HEPHL1

chr11	86805000	chr11	117185000	1.16E-02	super_enhancer	promoter	CEP164
chr11	87035000	chr11	94745000	4.11E-02	promoter	super_enhancer	RP11-777F6.3
chr11	88845000	chr11	94735000	1.85E-02	promoter	super_enhancer	AP001482.1
chr11	89035000	chr11	129635000	6.36E-03	promoter	super_enhancer	RP11-796A5.4
chr11	89955000	chr11	128185000	6.19E-03	promoter	super_enhancer	RP11-121L10.2
chr11	91115000	chr11	107855000	1.38E-02	promoter	super_enhancer	RP11-843F11.1
chr11	91115000	chr11	126345000	1.02E-02	promoter	super_enhancer	RP11-843F11.1
chr11	91115000	chr11	129865000	7.12E-03	promoter	super_enhancer	RP11-843F11.1
chr11	92885000	chr11	111735000	1.28E-02	promoter	super_enhancer	RP11-573M3.3
chr11	92885000	chr11	123445000	8.28E-03	promoter	super_enhancer	RP11-573M3.3
chr11	92955000	chr11	107845000	1.64E-02	promoter	super_enhancer	RP11-573M3.4
chr11	92955000	chr11	129855000	7.31E-03	promoter	super_enhancer	RP11-573M3.4
chr11	93395000	chr11	122175000	1.14E-02	promoter	super_enhancer	CEP295
chr11	93465000	chr11	114315000	1.13E-02	promoter	super_enhancer	SNORD6
chr11	93465000	chr11	128485000	8.53E-03	promoter	super_enhancer	SNORD6
chr11	93465000	chr11	129635000	9.29E-03	promoter	super_enhancer	SNORD6
chr11	93475000	chr11	100745000	3.13E-02	promoter	super_enhancer	C11orf54
chr11	94375000	chr11	100745000	3.49E-02	promoter	super_enhancer	RP11-867G2.6
chr11	94375000	chr11	128205000	7.42E-03	promoter	super_enhancer	RP11-867G2.6
chr11	94475000	chr11	118915000	1.99E-02	promoter	super_enhancer	RP11-867G2.8
chr11	94645000	chr11	126345000	1.42E-02	promoter	super_enhancer	ST13P11
chr11	94645000	chr11	128485000	1.04E-02	promoter	super_enhancer	ST13P11
chr11	94735000	chr11	123885000	8.69E-03	super_enhancer	promoter	OR10G4
chr11	94745000	chr11	108875000	2.67E-02	super_enhancer	promoter	RNU6-654P

chr11	94745000	chr11	133995000	8.80E-03	super_enhancer	promoter	PTP4A2P2
chr11	95215000	chr11	107825000	2.49E-02	promoter	super_enhancer	RP11-338H14.1
chr11	95525000	chr11	122165000	1.34E-02	promoter	super_enhancer	FAM76B
chr11	95725000	chr11	111725000	2.04E-02	promoter	super_enhancer	AP000870.1
chr11	96075000	chr11	129865000	1.21E-02	promoter	super_enhancer	MIR1260B
chr11	96515000	chr11	100745000	1.77E-03	promoter	super_enhancer	RP11-360K13.2
chr11	96515000	chr11	129845000	1.07E-02	promoter	super_enhancer	RP11-360K13.2
chr11	97815000	chr11	122165000	1.09E-02	promoter	super_enhancer	RP11-379J13.2
chr11	97815000	chr11	123465000	1.19E-02	promoter	super_enhancer	RP11-379J13.2
chr11	100745000	chr11	111895000	1.81E-02	super_enhancer	promoter	DLAT
chr11	101455000	chr11	119695000	1.99E-02	promoter	super_enhancer	RP11-748H22.1
chr11	101455000	chr11	122175000	1.89E-02	promoter	super_enhancer	RP11-748H22.1
chr11	102715000	chr11	122175000	1.43E-02	promoter	super_enhancer	MMP3
chr11	102775000	chr11	118615000	1.25E-02	promoter	super_enhancer	RNU7-159P
chr11	102775000	chr11	123455000	1.11E-02	promoter	super_enhancer	RNU7-159P
chr11	102775000	chr11	129855000	9.74E-03	promoter	super_enhancer	RNU7-159P
chr11	103815000	chr11	107845000	4.95E-02	promoter	super_enhancer	RP11-617B3.2
chr11	103815000	chr11	107855000	3.70E-02	promoter	super_enhancer	RP11-617B3.2
chr11	105945000	chr11	119695000	1.50E-02	promoter	super_enhancer	AASDHPPT
chr11	107275000	chr11	118615000	1.29E-02	promoter	super_enhancer	SMARCE1P1
chr11	107485000	chr11	122175000	2.07E-02	promoter	super_enhancer	AP000889.3
chr11	107515000	chr11	128515000	1.28E-02	promoter	super_enhancer	AP000889.1
chr11	107835000	chr11	112035000	4.57E-02	super_enhancer	promoter	IL18
chr11	107835000	chr11	117005000	1.50E-02	super_enhancer	promoter	RP11-109L13.1

chr11	107845000	chr11	115605000	3.54E-02	super_enhancer	promoter	CTC-774J1.1
chr11	107845000	chr11	116695000	3.34E-02	super_enhancer	promoter	APOA4
chr11	107845000	chr11	125495000	2.05E-02	super_enhancer	promoter	CHEK1
chr11	107855000	chr11	115605000	2.66E-02	super_enhancer	promoter	CTC-774J1.1
chr11	107855000	chr11	118515000	2.10E-02	super_enhancer	promoter	MIR6716
chr11	107855000	chr11	123325000	1.46E-02	super_enhancer	promoter	GRAMD1B
chr11	107855000	chr11	123925000	1.63E-02	super_enhancer	promoter	OR10D5P
chr11	107855000	chr11	125035000	1.68E-02	super_enhancer	promoter	PKNOX2-AS1
chr11	108465000	chr11	114295000	2.99E-02	promoter	super_enhancer	EXPH5
chr11	108535000	chr11	126345000	1.54E-02	promoter	super_enhancer	DDX10
chr11	108535000	chr11	128215000	9.42E-03	promoter	super_enhancer	DDX10
chr11	109135000	chr11	118615000	2.12E-02	promoter	super_enhancer	SNORD39
chr11	109135000	chr11	120215000	2.02E-02	promoter	super_enhancer	SNORD39
chr11	109455000	chr11	114295000	2.90E-02	promoter	super_enhancer	RP11-708B6.2
chr11	110225000	chr11	129845000	1.55E-02	promoter	super_enhancer	RP11-347E10.1
chr11	111385000	chr11	129865000	1.45E-02	promoter	super_enhancer	C11orf88
chr11	111725000	chr11	117015000	3.28E-02	super_enhancer	promoter	RP11-109L13.5
chr11	111725000	chr11	118465000	3.77E-02	super_enhancer	promoter	RNU6-1157P
chr11	111725000	chr11	118705000	3.88E-02	super_enhancer	promoter	SETP16
chr11	111725000	chr11	119205000	3.78E-02	super_enhancer	promoter	RNF26
chr11	111725000	chr11	131075000	1.70E-02	super_enhancer	promoter	AP002856.6
chr11	111735000	chr11	111895000	3.57E-02	super_enhancer	promoter	DLAT
chr11	111735000	chr11	111945000	4.68E-02	super_enhancer	promoter	PIH1D2
chr11	111735000	chr11	119055000	4.03E-02	super_enhancer	promoter	PDZD3

chr11	111735000	chr11	121875000	1.90E-02	super_enhancer	promoter	RNU6-256P
chr11	111735000	chr11	124275000	1.94E-02	super_enhancer	promoter	OR8X1P
chr11	111735000	chr11	131075000	1.75E-02	super_enhancer	promoter	AP002856.6
chr11	111795000	chr11	128175000	1.58E-02	promoter	super_enhancer	CRYAB
chr11	111885000	chr11	122165000	1.96E-02	promoter	super_enhancer	RP11-708L7.6
chr11	111895000	chr11	118925000	2.97E-02	promoter	super_enhancer	DLAT
chr11	111895000	chr11	129865000	1.26E-02	promoter	super_enhancer	DLAT
chr11	112035000	chr11	118625000	2.94E-02	promoter	super_enhancer	IL18
chr11	112035000	chr11	123475000	2.47E-02	promoter	super_enhancer	IL18
chr11	112035000	chr11	126405000	1.93E-02	promoter	super_enhancer	IL18
chr11	112035000	chr11	129625000	1.36E-02	promoter	super_enhancer	IL18
chr11	112045000	chr11	119695000	1.79E-02	promoter	super_enhancer	BCO2
chr11	113185000	chr11	114295000	4.04E-02	promoter	super_enhancer	RP11-839D17.3
chr11	113185000	chr11	122165000	2.58E-02	promoter	super_enhancer	RP11-839D17.3
chr11	113185000	chr11	129635000	2.06E-02	promoter	super_enhancer	RP11-839D17.3
chr11	113275000	chr11	120125000	3.83E-02	promoter	super_enhancer	RP11-159N11.3
chr11	113775000	chr11	118925000	4.50E-02	promoter	super_enhancer	HTR3B
chr11	114295000	chr11	123325000	1.74E-02	super_enhancer	promoter	GRAMD1B
chr11	114305000	chr11	119185000	4.48E-02	super_enhancer	promoter	MIR6756
chr11	114305000	chr11	122525000	2.57E-02	super_enhancer	promoter	UBASH3B
chr11	114315000	chr11	123965000	1.17E-02	super_enhancer	promoter	OR10D4P
chr11	114315000	chr11	124275000	1.68E-02	super_enhancer	promoter	OR8X1P
chr11	115405000	chr11	123455000	2.68E-02	promoter	super_enhancer	RP11-136I14.2
chr11	115405000	chr11	123475000	2.99E-02	promoter	super_enhancer	RP11-136I14.2

chr11	115405000	chr11	126345000	2.67E-02	promoter	super_enhancer	RP11-136I14.2
chr11	115605000	chr11	122195000	3.12E-02	promoter	super_enhancer	CTC-774J1.1
chr11	116685000	chr11	128515000	1.85E-02	promoter	super_enhancer	AP006216.5
chr11	116695000	chr11	123475000	4.12E-02	promoter	super_enhancer	APOA4
chr11	117005000	chr11	128515000	1.26E-02	promoter	super_enhancer	RP11-109L13.1
chr11	117005000	chr11	129855000	1.59E-02	promoter	super_enhancer	RP11-109L13.1
chr11	117015000	chr11	128185000	1.30E-02	promoter	super_enhancer	RP11-109L13.5
chr11	117185000	chr11	122165000	1.34E-03	promoter	super_enhancer	CEP164
chr11	117185000	chr11	123475000	4.88E-02	promoter	super_enhancer	CEP164
chr11	118025000	chr11	128175000	2.14E-02	promoter	super_enhancer	SCN4B
chr11	118135000	chr11	128185000	1.25E-02	promoter	super_enhancer	MPZL2
chr11	118175000	chr11	124895000	2.14E-02	promoter	super_enhancer	CD3E
chr11	118195000	chr11	129845000	2.47E-02	promoter	super_enhancer	RP11-215H18.5
chr11	118215000	chr11	123455000	4.97E-02	promoter	super_enhancer	CD3G
chr11	118445000	chr11	118925000	8.37E-03	promoter	super_enhancer	IFT46
chr11	118445000	chr11	123455000	4.57E-02	promoter	super_enhancer	IFT46
chr11	118445000	chr11	129845000	2.26E-02	promoter	super_enhancer	IFT46
chr11	118515000	chr11	123445000	4.77E-02	promoter	super_enhancer	MIR6716
chr11	118515000	chr11	123455000	4.76E-02	promoter	super_enhancer	MIR6716
chr11	118515000	chr11	128215000	2.09E-02	promoter	super_enhancer	MIR6716
chr11	118575000	chr11	118625000	3.99E-02	promoter	super_enhancer	AP002954.4
chr11	118575000	chr11	118915000	3.61E-02	promoter	super_enhancer	AP002954.4
chr11	118615000	chr11	118795000	3.97E-02	super_enhancer	promoter	UPK2
chr11	118615000	chr11	123185000	4.51E-02	super_enhancer	promoter	RP11-109E10.1

chr11	118615000	chr11	124275000	3.17E-02	super_enhancer	promoter	OR8X1P
chr11	118615000	chr11	125745000	2.70E-02	super_enhancer	promoter	RP11-680F20.10
chr11	118615000	chr11	130165000	2.05E-02	super_enhancer	promoter	DDX18P5
chr11	118625000	chr11	122935000	4.57E-02	super_enhancer	promoter	HSPA8
chr11	118795000	chr11	118925000	2.62E-02	promoter	super_enhancer	UPK2
chr11	118875000	chr11	123445000	4.87E-02	promoter	super_enhancer	RPL23AP64
chr11	118915000	chr11	119185000	2.16E-02	super_enhancer	promoter	MIR6756
chr11	118915000	chr11	123325000	1.52E-03	super_enhancer	promoter	GRAMD1B
chr11	118915000	chr11	123635000	4.69E-02	super_enhancer	promoter	RNU1-21P
chr11	118915000	chr11	126175000	3.52E-02	super_enhancer	promoter	RP11-712L6.5
chr11	118915000	chr11	128395000	2.65E-02	super_enhancer	promoter	RP11-1007G5.2
chr11	118915000	chr11	131075000	2.75E-02	super_enhancer	promoter	AP002856.6
chr11	118915000	chr11	131855000	1.92E-02	super_enhancer	promoter	RP11-697E14.2
chr11	118925000	chr11	124295000	4.27E-02	super_enhancer	promoter	OR8B4
chr11	118925000	chr11	126225000	3.95E-02	super_enhancer	promoter	ST3GAL4-AS1
chr11	119185000	chr11	128205000	2.22E-02	promoter	super_enhancer	MIR6756
chr11	119205000	chr11	128505000	3.22E-02	promoter	super_enhancer	RNF26
chr11	119275000	chr11	128205000	2.23E-02	promoter	super_enhancer	RP11-334E6.4
chr11	119275000	chr11	129855000	3.01E-02	promoter	super_enhancer	RP11-334E6.4
chr11	119475000	chr11	128215000	2.37E-02	promoter	super_enhancer	KRT8P7
chr11	119475000	chr11	128485000	2.74E-02	promoter	super_enhancer	KRT8P7
chr11	119695000	chr11	125035000	4.37E-02	super_enhancer	promoter	PKNOX2-AS1
chr11	119695000	chr11	125615000	5.56E-04	super_enhancer	promoter	PATE1
chr11	120125000	chr11	126115000	4.17E-02	super_enhancer	promoter	RN7SL351P

chr11	120195000	chr11	128205000	2.32E-02	promoter	super_enhancer	TMEM136
chr11	120215000	chr11	126085000	2.92E-02	super_enhancer	promoter	RNU4-86P
chr11	120895000	chr11	128175000	2.01E-02	promoter	super_enhancer	TBCEL
chr11	121235000	chr11	129845000	2.19E-02	promoter	super_enhancer	BMPR1APS2
chr11	122175000	chr11	129245000	3.29E-02	super_enhancer	promoter	BARX2
chr11	122175000	chr11	133995000	1.83E-02	super_enhancer	promoter	PTP4A2P2
chr11	122185000	chr11	122295000	9.70E-03	super_enhancer	promoter	MIR100HG
chr11	122185000	chr11	129985000	2.35E-02	super_enhancer	promoter	U4
chr11	122195000	chr11	126085000	4.18E-02	super_enhancer	promoter	RNU4-86P
chr11	122295000	chr11	128485000	3.13E-02	promoter	super_enhancer	MIR100HG
chr11	122935000	chr11	129625000	2.60E-02	promoter	super_enhancer	HSPA8
chr11	123185000	chr11	123445000	6.46E-03	promoter	super_enhancer	RP11-109E10.1
chr11	123185000	chr11	128195000	4.13E-02	promoter	super_enhancer	RP11-109E10.1
chr11	123185000	chr11	128505000	4.10E-02	promoter	super_enhancer	RP11-109E10.1
chr11	123325000	chr11	129645000	3.26E-02	promoter	super_enhancer	GRAMD1B
chr11	123455000	chr11	124275000	1.59E-02	super_enhancer	promoter	OR8X1P
chr11	123475000	chr11	130185000	2.95E-02	super_enhancer	promoter	ZBTB44
chr11	123625000	chr11	129865000	2.77E-02	promoter	super_enhancer	OR6X1
chr11	123635000	chr11	128515000	3.22E-02	promoter	super_enhancer	RNU1-21P
chr11	123815000	chr11	129635000	3.31E-02	promoter	super_enhancer	OR6T1
chr11	123985000	chr11	128505000	4.29E-02	promoter	super_enhancer	VWA5A
chr11	124055000	chr11	128485000	4.57E-02	promoter	super_enhancer	OR10D3
chr11	124085000	chr11	129855000	2.85E-02	promoter	super_enhancer	OR8G3P
chr11	124095000	chr11	128515000	3.59E-02	promoter	super_enhancer	OR8G2P

chr11	124415000	chr11	128205000	3.97E-02	promoter	super_enhancer	OR8B12
chr11	125615000	chr11	128485000	3.46E-03	promoter	super_enhancer	PATE1
chr11	126165000	chr11	126345000	3.84E-02	promoter	super_enhancer	RP11-712L6.7
chr11	126175000	chr11	128215000	7.35E-03	promoter	super_enhancer	RP11-712L6.5
chr11	126225000	chr11	128485000	1.54E-05	promoter	super_enhancer	ST3GAL4-AS1
chr11	126345000	chr11	131255000	4.52E-02	super_enhancer	promoter	RNU6ATAC12P
chr11	126405000	chr11	132275000	2.76E-02	super_enhancer	promoter	RP11-684N3.1
chr11	126405000	chr11	134095000	2.51E-02	super_enhancer	promoter	NCAPD3
chr11	128205000	chr11	129685000	1.33E-02	super_enhancer	promoter	TMEM45B
chr11	128395000	chr11	129625000	2.79E-02	promoter	super_enhancer	RP11-1007G5.2
chr11	128395000	chr11	129845000	2.44E-02	promoter	super_enhancer	RP11-1007G5.2
chr11	129245000	chr11	129625000	9.20E-04	promoter	super_enhancer	BARX2
chr11	129645000	chr11	130735000	4.99E-02	super_enhancer	promoter	RP11-890B15.2
chr11	129645000	chr11	132275000	2.98E-03	super_enhancer	promoter	RP11-684N3.1
chr11	129865000	chr11	130185000	2.15E-02	super_enhancer	promoter	ZBTB44
chr11	129865000	chr11	134305000	3.73E-02	super_enhancer	promoter	RP11-627G23.1
chr12	325000	chr12	6555000	3.22E-02	promoter	super_enhancer	SLC6A12
chr12	325000	chr12	31755000	9.00E-03	promoter	super_enhancer	SLC6A12
chr12	325000	chr12	53355000	6.33E-03	promoter	super_enhancer	SLC6A12
chr12	575000	chr12	8185000	4.09E-02	super_enhancer	promoter	FOXJ2
chr12	575000	chr12	12925000	2.51E-02	super_enhancer	promoter	STX8P1
chr12	575000	chr12	101605000	4.17E-03	super_enhancer	promoter	SLC5A8
chr12	575000	chr12	122745000	4.65E-03	super_enhancer	promoter	RP11-512M8.11
chr12	575000	chr12	125045000	4.53E-03	super_enhancer	promoter	RP11-83B20.6

chr12	1495000	chr12	6555000	4.68E-02	promoter	super_enhancer	RP5-951N9.2
chr12	1495000	chr12	50195000	9.10E-03	promoter	super_enhancer	RP5-951N9.2
chr12	1495000	chr12	93805000	3.11E-03	promoter	super_enhancer	RP5-951N9.2
chr12	2115000	chr12	52935000	7.03E-03	promoter	super_enhancer	DCP1B
chr12	2115000	chr12	122425000	2.94E-03	promoter	super_enhancer	DCP1B
chr12	2975000	chr12	101605000	5.27E-03	super_enhancer	promoter	SLC5A8
chr12	2975000	chr12	118805000	4.85E-03	super_enhancer	promoter	AC026367.1
chr12	2975000	chr12	120555000	6.08E-03	super_enhancer	promoter	RAB35
chr12	2995000	chr12	12725000	3.34E-02	promoter	super_enhancer	RP11-372B4.3
chr12	2995000	chr12	95635000	4.69E-03	promoter	super_enhancer	RP11-372B4.3
chr12	3835000	chr12	53235000	1.14E-02	promoter	super_enhancer	RNU6-174P
chr12	3835000	chr12	131915000	3.58E-03	promoter	super_enhancer	RNU6-174P
chr12	4385000	chr12	55725000	6.08E-03	promoter	super_enhancer	CCND2-AS1
chr12	4555000	chr12	31755000	1.10E-02	promoter	super_enhancer	FGF6
chr12	4745000	chr12	120235000	2.94E-03	promoter	super_enhancer	RP11-500M8.6
chr12	4745000	chr12	123135000	2.69E-03	promoter	super_enhancer	RP11-500M8.6
chr12	5425000	chr12	6285000	8.26E-03	promoter	super_enhancer	RP11-319E16.2
chr12	5425000	chr12	12875000	2.08E-02	promoter	super_enhancer	RP11-319E16.2
chr12	6055000	chr12	6335000	5.16E-03	promoter	super_enhancer	ANO2
chr12	6055000	chr12	13225000	3.71E-02	promoter	super_enhancer	ANO2
chr12	6205000	chr12	13095000	3.68E-02	promoter	super_enhancer	RN7SL69P
chr12	6235000	chr12	6275000	1.44E-02	promoter	super_enhancer	VWF
chr12	6235000	chr12	52185000	8.37E-03	promoter	super_enhancer	VWF
chr12	6275000	chr12	13125000	3.60E-02	super_enhancer	promoter	SNORD88

chr12	6275000	chr12	31015000	1.61E-02	super_enhancer	promoter	RP11-77I22.4
chr12	6275000	chr12	42755000	8.84E-03	super_enhancer	promoter	RP11-351C21.2
chr12	6275000	chr12	46765000	7.71E-03	super_enhancer	promoter	RP11-474P2.2
chr12	6275000	chr12	54385000	1.15E-02	super_enhancer	promoter	MIR196A2
chr12	6275000	chr12	95945000	4.26E-03	super_enhancer	promoter	USP44
chr12	6285000	chr12	70965000	4.04E-03	super_enhancer	promoter	RP11-785D18.3
chr12	6335000	chr12	13595000	2.39E-02	super_enhancer	promoter	RNA5SP353
chr12	6335000	chr12	19455000	2.13E-02	super_enhancer	promoter	RN7SL67P
chr12	6335000	chr12	57335000	5.55E-03	super_enhancer	promoter	RP11-474N8.9
chr12	6335000	chr12	117195000	2.48E-03	super_enhancer	promoter	RP11-407P2.1
chr12	6345000	chr12	14345000	3.18E-02	super_enhancer	promoter	RP11-298E10.1
chr12	6345000	chr12	25405000	1.69E-02	super_enhancer	promoter	KRAS
chr12	6345000	chr12	79895000	5.12E-03	super_enhancer	promoter	RP11-359M6.2
chr12	6375000	chr12	31945000	1.90E-02	super_enhancer	promoter	H3F3C
chr12	6375000	chr12	48745000	1.08E-02	super_enhancer	promoter	ZNF641
chr12	6375000	chr12	57905000	7.42E-03	super_enhancer	promoter	MIR6758
chr12	6375000	chr12	121455000	4.95E-03	super_enhancer	promoter	C12orf43
chr12	6375000	chr12	125385000	5.04E-03	super_enhancer	promoter	RNU6-927P
chr12	6535000	chr12	12965000	4.05E-02	super_enhancer	promoter	DDX47
chr12	6535000	chr12	21815000	1.84E-02	super_enhancer	promoter	RP11-59N23.3
chr12	6535000	chr12	31465000	1.83E-02	super_enhancer	promoter	RP11-627K11.3
chr12	6535000	chr12	50505000	1.17E-02	super_enhancer	promoter	COX14
chr12	6535000	chr12	127545000	3.10E-03	super_enhancer	promoter	RP11-575F12.1
chr12	6545000	chr12	11325000	4.90E-02	super_enhancer	promoter	PRH1

chr12	6545000	chr12	13175000	3.66E-02	super_enhancer	promoter	RP11-377D9.1
chr12	6545000	chr12	14345000	3.30E-02	super_enhancer	promoter	RP11-298E10.1
chr12	6545000	chr12	25055000	2.16E-02	super_enhancer	promoter	RP11-662I13.3
chr12	6545000	chr12	57235000	7.73E-03	super_enhancer	promoter	RP11-74M13.6
chr12	6555000	chr12	6575000	1.06E-02	super_enhancer	promoter	RP1-102E24.10
chr12	6555000	chr12	13115000	3.84E-02	super_enhancer	promoter	RP11-392P7.7
chr12	6555000	chr12	49455000	1.18E-02	super_enhancer	promoter	KMT2D
chr12	6555000	chr12	55945000	7.90E-03	super_enhancer	promoter	OR6C4
chr12	6555000	chr12	56615000	8.23E-03	super_enhancer	promoter	NABP2
chr12	6555000	chr12	75885000	3.91E-03	super_enhancer	promoter	RP11-585P4.5
chr12	6555000	chr12	132905000	4.55E-03	super_enhancer	promoter	RP13-895J2.7
chr12	6555000	chr12	133065000	3.67E-03	super_enhancer	promoter	FBRSL1
chr12	6575000	chr12	12885000	3.85E-02	promoter	super_enhancer	RP1-102E24.10
chr12	6575000	chr12	13205000	2.88E-02	promoter	super_enhancer	RP1-102E24.10
chr12	6575000	chr12	55735000	5.93E-03	promoter	super_enhancer	RP1-102E24.10
chr12	6665000	chr12	54425000	9.17E-03	promoter	super_enhancer	IFFO1
chr12	6875000	chr12	6925000	2.64E-02	promoter	super_enhancer	PTMS
chr12	6875000	chr12	53355000	1.17E-02	promoter	super_enhancer	PTMS
chr12	6875000	chr12	124925000	5.74E-03	promoter	super_enhancer	PTMS
chr12	6895000	chr12	12835000	3.80E-02	promoter	super_enhancer	CD4
chr12	6895000	chr12	52955000	6.78E-03	promoter	super_enhancer	CD4
chr12	6925000	chr12	7055000	8.14E-03	super_enhancer	promoter	U47924.31
chr12	6925000	chr12	8045000	1.34E-02	super_enhancer	promoter	SLC2A14
chr12	6925000	chr12	12765000	3.87E-02	super_enhancer	promoter	CREBL2

chr12	6925000	chr12	15115000	2.67E-02	super_enhancer	promoter	ARHGDIB
chr12	6925000	chr12	27935000	2.26E-02	super_enhancer	promoter	RP11-860B13.1
chr12	6925000	chr12	27955000	2.07E-02	super_enhancer	promoter	RP11-860B13.3
chr12	6925000	chr12	50355000	1.21E-02	super_enhancer	promoter	AQP5
chr12	6925000	chr12	118815000	3.77E-03	super_enhancer	promoter	RP11-131L12.2
chr12	7055000	chr12	50225000	1.26E-02	promoter	super_enhancer	U47924.31
chr12	7095000	chr12	52285000	1.12E-02	promoter	super_enhancer	C1S
chr12	7245000	chr12	12725000	4.02E-02	promoter	super_enhancer	C1R
chr12	7245000	chr12	47825000	5.79E-03	promoter	super_enhancer	C1R
chr12	7245000	chr12	121785000	4.91E-03	promoter	super_enhancer	C1R
chr12	7285000	chr12	111405000	6.48E-03	promoter	super_enhancer	RP11-273B20.1
chr12	7905000	chr12	52235000	1.01E-02	promoter	super_enhancer	CLEC4C
chr12	8185000	chr12	47825000	6.26E-03	promoter	super_enhancer	FOXJ2
chr12	8185000	chr12	123125000	4.70E-03	promoter	super_enhancer	FOXJ2
chr12	8295000	chr12	52195000	6.82E-03	promoter	super_enhancer	GCSHP4
chr12	8295000	chr12	52995000	6.10E-03	promoter	super_enhancer	GCSHP4
chr12	8765000	chr12	50825000	7.94E-03	promoter	super_enhancer	AICDA
chr12	8765000	chr12	124385000	4.08E-03	promoter	super_enhancer	AICDA
chr12	8785000	chr12	10215000	1.86E-02	promoter	super_enhancer	RP11-438L7.1
chr12	8895000	chr12	31505000	2.03E-02	promoter	super_enhancer	RPSAP51
chr12	8895000	chr12	131905000	3.63E-03	promoter	super_enhancer	RPSAP51
chr12	9795000	chr12	50805000	8.91E-03	promoter	super_enhancer	RP11-705C15.5
chr12	10185000	chr12	53345000	9.58E-03	super_enhancer	promoter	KRT8
chr12	10185000	chr12	68395000	2.86E-03	super_enhancer	promoter	RPL39P28

chr12	10195000	chr12	11325000	3.32E-03	super_enhancer	promoter	PRH1
chr12	10195000	chr12	11655000	2.89E-02	super_enhancer	promoter	PRB2
chr12	10195000	chr12	15955000	2.57E-02	super_enhancer	promoter	RP11-6B19.3
chr12	10205000	chr12	48165000	7.28E-03	super_enhancer	promoter	RAPGEF3
chr12	10205000	chr12	105345000	2.06E-03	super_enhancer	promoter	AC090051.1
chr12	10215000	chr12	10265000	2.50E-02	super_enhancer	promoter	CLEC1A
chr12	10215000	chr12	24735000	1.28E-02	super_enhancer	promoter	KNOP1P1
chr12	10325000	chr12	123105000	3.01E-03	promoter	super_enhancer	OLR1
chr12	10365000	chr12	12725000	6.40E-03	promoter	super_enhancer	GABARAPL1
chr12	10515000	chr12	96425000	2.26E-03	promoter	super_enhancer	RP11-277P12.9
chr12	10955000	chr12	46535000	6.64E-03	promoter	super_enhancer	TAS2R7
chr12	10985000	chr12	12805000	1.06E-02	promoter	super_enhancer	RP11-144O23.8
chr12	11185000	chr12	55735000	5.20E-03	promoter	super_enhancer	TAS2R31
chr12	11215000	chr12	12835000	1.37E-02	promoter	super_enhancer	TAS2R46
chr12	11665000	chr12	12835000	6.00E-05	promoter	super_enhancer	DDX55P1
chr12	11665000	chr12	131875000	3.39E-03	promoter	super_enhancer	DDX55P1
chr12	12605000	chr12	12835000	4.28E-02	promoter	super_enhancer	RP11-253I19.4
chr12	12605000	chr12	53235000	1.23E-02	promoter	super_enhancer	RP11-253I19.4
chr12	12605000	chr12	53375000	1.06E-02	promoter	super_enhancer	RP11-253I19.4
chr12	12605000	chr12	53385000	1.00E-02	promoter	super_enhancer	RP11-253I19.4
chr12	12715000	chr12	12805000	3.74E-02	promoter	super_enhancer	DUSP16
chr12	12715000	chr12	31755000	1.23E-02	promoter	super_enhancer	DUSP16
chr12	12725000	chr12	12925000	2.75E-02	super_enhancer	promoter	STX8P1
chr12	12725000	chr12	70005000	4.84E-03	super_enhancer	promoter	LRRC10

chr12	12805000	chr12	21655000	2.21E-02	super_enhancer	promoter	RECQL
chr12	12805000	chr12	46985000	1.12E-02	super_enhancer	promoter	OR7A19P
chr12	12805000	chr12	50505000	1.29E-02	super_enhancer	promoter	COX14
chr12	12805000	chr12	54675000	9.67E-03	super_enhancer	promoter	CBX5
chr12	12805000	chr12	57125000	8.75E-03	super_enhancer	promoter	NACA
chr12	12805000	chr12	125015000	4.14E-03	super_enhancer	promoter	RP11-83B20.2
chr12	12835000	chr12	47475000	9.01E-03	super_enhancer	promoter	AMIGO2
chr12	12835000	chr12	50505000	1.27E-02	super_enhancer	promoter	COX14
chr12	12835000	chr12	58335000	7.48E-03	super_enhancer	promoter	XRCC6BP1
chr12	12875000	chr12	14135000	3.70E-02	super_enhancer	promoter	GRIN2B
chr12	12875000	chr12	93345000	3.40E-03	super_enhancer	promoter	RNU6-1329P
chr12	12875000	chr12	118575000	3.17E-03	super_enhancer	promoter	VSIG10
chr12	12925000	chr12	123135000	3.80E-03	promoter	super_enhancer	STX8P1
chr12	12965000	chr12	13205000	1.89E-02	promoter	super_enhancer	DDX47
chr12	12995000	chr12	52965000	1.15E-02	promoter	super_enhancer	RP11-59H1.1
chr12	12995000	chr12	55725000	8.14E-03	promoter	super_enhancer	RP11-59H1.1
chr12	12995000	chr12	116415000	2.85E-03	promoter	super_enhancer	RP11-59H1.1
chr12	13105000	chr12	31745000	2.46E-02	promoter	super_enhancer	GPRC5D
chr12	13115000	chr12	47815000	1.12E-02	promoter	super_enhancer	RP11-392P7.7
chr12	13115000	chr12	50825000	1.06E-02	promoter	super_enhancer	RP11-392P7.7
chr12	13205000	chr12	27925000	1.94E-02	super_enhancer	promoter	MANSC4
chr12	13205000	chr12	27955000	1.94E-02	super_enhancer	promoter	RP11-860B13.3
chr12	13205000	chr12	42305000	1.03E-02	super_enhancer	promoter	RP11-2A1.1
chr12	13205000	chr12	59315000	4.16E-03	super_enhancer	promoter	LRIG3

chr12	13205000	chr12	120685000	3.59E-03	super_enhancer	promoter	RPS20P31
chr12	13215000	chr12	14345000	7.36E-03	super_enhancer	promoter	RP11-298E10.1
chr12	13225000	chr12	14135000	2.16E-02	super_enhancer	promoter	GRIN2B
chr12	13225000	chr12	22875000	2.45E-02	super_enhancer	promoter	RPS27P22
chr12	13225000	chr12	26275000	2.51E-02	super_enhancer	promoter	SSPN
chr12	13225000	chr12	63395000	6.60E-03	super_enhancer	promoter	RP11-848D3.4
chr12	13225000	chr12	73185000	4.01E-03	super_enhancer	promoter	AC131213.1
chr12	14135000	chr12	56525000	9.27E-03	promoter	super_enhancer	GRIN2B
chr12	14925000	chr12	95205000	3.66E-03	promoter	super_enhancer	HIST4H4
chr12	15115000	chr12	52965000	8.80E-03	promoter	super_enhancer	ARHGDIB
chr12	15115000	chr12	53355000	8.77E-03	promoter	super_enhancer	ARHGDIB
chr12	15125000	chr12	31505000	2.15E-02	promoter	super_enhancer	PDE6H
chr12	15825000	chr12	124685000	2.53E-03	promoter	super_enhancer	RNU6-251P
chr12	16555000	chr12	55725000	6.96E-03	promoter	super_enhancer	RP11-473N11.2
chr12	16665000	chr12	52885000	1.14E-02	promoter	super_enhancer	RP11-424M22.1
chr12	17225000	chr12	31735000	1.07E-02	promoter	super_enhancer	RNU6-837P
chr12	17225000	chr12	46465000	5.73E-03	promoter	super_enhancer	RNU6-837P
chr12	19455000	chr12	31745000	3.54E-02	promoter	super_enhancer	RN7SL67P
chr12	19455000	chr12	50255000	1.42E-02	promoter	super_enhancer	RN7SL67P
chr12	19565000	chr12	46455000	1.24E-02	promoter	super_enhancer	PDCD5P1
chr12	19565000	chr12	116545000	2.40E-03	promoter	super_enhancer	PDCD5P1
chr12	20705000	chr12	52885000	1.05E-02	promoter	super_enhancer	RP11-424C20.2
chr12	21815000	chr12	70735000	4.19E-03	promoter	super_enhancer	RP11-59N23.3
chr12	22095000	chr12	31505000	3.24E-02	promoter	super_enhancer	ABCC9

chr12	22095000	chr12	31735000	2.39E-02	promoter	super_enhancer	ABCC9
chr12	22725000	chr12	52245000	1.61E-02	promoter	super_enhancer	LRRC34P1
chr12	24365000	chr12	47825000	7.76E-03	promoter	super_enhancer	MIR920
chr12	24375000	chr12	31505000	3.98E-02	promoter	super_enhancer	RP11-778H2.1
chr12	24375000	chr12	50805000	1.43E-02	promoter	super_enhancer	RP11-778H2.1
chr12	24375000	chr12	52965000	1.34E-02	promoter	super_enhancer	RP11-778H2.1
chr12	24735000	chr12	50775000	1.33E-02	promoter	super_enhancer	KNOP1P1
chr12	25055000	chr12	50235000	1.84E-02	promoter	super_enhancer	RP11-662I13.3
chr12	25255000	chr12	52945000	1.25E-02	promoter	super_enhancer	RP11-713N11.4
chr12	25255000	chr12	54425000	9.11E-03	promoter	super_enhancer	RP11-713N11.4
chr12	25955000	chr12	31735000	3.61E-02	promoter	super_enhancer	TDGP1
chr12	26275000	chr12	47755000	1.53E-02	promoter	super_enhancer	SSPN
chr12	27175000	chr12	46455000	2.07E-02	promoter	super_enhancer	MED21
chr12	27175000	chr12	76015000	6.35E-03	promoter	super_enhancer	MED21
chr12	27175000	chr12	123115000	4.98E-03	promoter	super_enhancer	MED21
chr12	27235000	chr12	52145000	1.81E-02	promoter	super_enhancer	C12orf71
chr12	27235000	chr12	54415000	1.64E-02	promoter	super_enhancer	C12orf71
chr12	27235000	chr12	124915000	5.11E-03	promoter	super_enhancer	C12orf71
chr12	27275000	chr12	31765000	4.09E-02	promoter	super_enhancer	RP11-582E3.4
chr12	27315000	chr12	50765000	1.46E-02	promoter	super_enhancer	RP11-582E3.2
chr12	27485000	chr12	52915000	1.68E-02	promoter	super_enhancer	ARNTL2
chr12	27485000	chr12	75985000	6.22E-03	promoter	super_enhancer	ARNTL2
chr12	27675000	chr12	50255000	2.12E-02	promoter	super_enhancer	PPFIBP1
chr12	27675000	chr12	52205000	2.10E-02	promoter	super_enhancer	PPFIBP1

chr12	27855000	chr12	53215000	1.73E-02	promoter	super_enhancer	RP11-1060J15.5
chr12	27855000	chr12	104685000	5.17E-03	promoter	super_enhancer	RP11-1060J15.5
chr12	27865000	chr12	52205000	2.33E-02	promoter	super_enhancer	RP11-1060J15.4
chr12	27865000	chr12	55735000	1.54E-02	promoter	super_enhancer	RP11-1060J15.4
chr12	27925000	chr12	50215000	2.09E-02	promoter	super_enhancer	MANSC4
chr12	27925000	chr12	52975000	2.12E-02	promoter	super_enhancer	MANSC4
chr12	27925000	chr12	120235000	4.75E-03	promoter	super_enhancer	MANSC4
chr12	27935000	chr12	46455000	1.97E-02	promoter	super_enhancer	RP11-860B13.1
chr12	27935000	chr12	50805000	2.22E-02	promoter	super_enhancer	RP11-860B13.1
chr12	27955000	chr12	52205000	1.92E-02	promoter	super_enhancer	RP11-860B13.3
chr12	28345000	chr12	50795000	1.17E-02	promoter	super_enhancer	RP11-967K21.1
chr12	29485000	chr12	104225000	2.38E-03	promoter	super_enhancer	RP11-996F15.5
chr12	29535000	chr12	30795000	3.78E-02	promoter	super_enhancer	ERGIC2
chr12	29535000	chr12	50795000	1.39E-02	promoter	super_enhancer	ERGIC2
chr12	29535000	chr12	122915000	3.70E-03	promoter	super_enhancer	ERGIC2
chr12	30795000	chr12	48965000	1.88E-02	super_enhancer	promoter	LALBA
chr12	30795000	chr12	57335000	1.21E-02	super_enhancer	promoter	RP11-474N8.9
chr12	30795000	chr12	58335000	1.18E-02	super_enhancer	promoter	XRCC6BP1
chr12	30795000	chr12	89745000	4.15E-03	super_enhancer	promoter	RP11-823E8.3
chr12	31015000	chr12	122925000	4.22E-03	promoter	super_enhancer	RP11-77I22.4
chr12	31165000	chr12	50215000	1.65E-02	promoter	super_enhancer	RP11-551L14.6
chr12	31465000	chr12	46475000	2.51E-02	promoter	super_enhancer	RP11-627K11.3
chr12	31465000	chr12	104285000	4.66E-03	promoter	super_enhancer	RP11-627K11.3
chr12	31465000	chr12	123065000	4.87E-03	promoter	super_enhancer	RP11-627K11.3

chr12	31485000	chr12	48555000	2.29E-02	super_enhancer	promoter	RP11-474C8.3
chr12	31485000	chr12	50505000	2.42E-02	super_enhancer	promoter	COX14
chr12	31485000	chr12	53075000	2.44E-02	super_enhancer	promoter	KRT1
chr12	31485000	chr12	68845000	9.75E-03	super_enhancer	promoter	RP11-81H14.2
chr12	31485000	chr12	76425000	7.93E-03	super_enhancer	promoter	RP11-290L1.2
chr12	31485000	chr12	116955000	4.57E-03	super_enhancer	promoter	RP11-148B3.1
chr12	31495000	chr12	53345000	2.63E-02	super_enhancer	promoter	KRT8
chr12	31495000	chr12	53645000	2.34E-02	super_enhancer	promoter	MFSD5
chr12	31495000	chr12	57335000	1.61E-02	super_enhancer	promoter	RP11-474N8.9
chr12	31495000	chr12	65815000	1.19E-02	super_enhancer	promoter	KRT18P60
chr12	31495000	chr12	66485000	1.15E-02	super_enhancer	promoter	AC078927.1
chr12	31495000	chr12	69795000	1.02E-02	super_enhancer	promoter	RN7SL804P
chr12	31495000	chr12	112365000	4.54E-03	super_enhancer	promoter	ADAM1B
chr12	31495000	chr12	113345000	5.09E-03	super_enhancer	promoter	OAS1
chr12	31505000	chr12	34315000	4.97E-03	super_enhancer	promoter	RP11-313F23.2
chr12	31505000	chr12	45465000	2.93E-02	super_enhancer	promoter	RP11-478B9.2
chr12	31505000	chr12	55655000	1.83E-02	super_enhancer	promoter	OR6C69P
chr12	31505000	chr12	57905000	1.82E-02	super_enhancer	promoter	MIR6758
chr12	31515000	chr12	32505000	2.12E-04	super_enhancer	promoter	RP11-817I4.2
chr12	31515000	chr12	53895000	2.38E-02	super_enhancer	promoter	MAP3K12
chr12	31515000	chr12	57845000	1.50E-02	super_enhancer	promoter	INHBE
chr12	31525000	chr12	65015000	1.07E-02	super_enhancer	promoter	MIR548C
chr12	31525000	chr12	101805000	4.25E-03	super_enhancer	promoter	RNU6-1068P
chr12	31735000	chr12	32505000	2.92E-02	super_enhancer	promoter	RP11-817I4.2

chr12	31735000	chr12	43955000	1.83E-02	super_enhancer	promoter	RP11-73B8.1
chr12	31735000	chr12	48575000	2.72E-02	super_enhancer	promoter	ASB8
chr12	31735000	chr12	52625000	1.92E-02	super_enhancer	promoter	KRT7
chr12	31735000	chr12	56495000	1.61E-02	super_enhancer	promoter	RP11-603J24.9
chr12	31735000	chr12	56615000	1.67E-02	super_enhancer	promoter	NABP2
chr12	31735000	chr12	64155000	8.75E-03	super_enhancer	promoter	RP11-415I12.1
chr12	31735000	chr12	104945000	4.81E-03	super_enhancer	promoter	RP11-43D4.3
chr12	31745000	chr12	39455000	2.97E-02	super_enhancer	promoter	RP11-554L12.2
chr12	31745000	chr12	55865000	1.71E-02	super_enhancer	promoter	OR6C70
chr12	31745000	chr12	56915000	1.90E-02	super_enhancer	promoter	RBMS2
chr12	31745000	chr12	57145000	2.01E-02	super_enhancer	promoter	HSD17B6
chr12	31745000	chr12	98985000	6.62E-03	super_enhancer	promoter	RP11-481K9.4
chr12	31755000	chr12	54395000	1.45E-02	super_enhancer	promoter	HOXC-AS1
chr12	31755000	chr12	117135000	4.17E-03	super_enhancer	promoter	RP11-497G19.2
chr12	31765000	chr12	38555000	2.08E-02	super_enhancer	promoter	RNA5SP358
chr12	31765000	chr12	57635000	1.35E-02	super_enhancer	promoter	NDUFA4L2
chr12	32045000	chr12	50255000	2.61E-02	promoter	super_enhancer	RP11-428G5.6
chr12	32045000	chr12	52905000	2.20E-02	promoter	super_enhancer	RP11-428G5.6
chr12	32045000	chr12	116425000	3.43E-03	promoter	super_enhancer	RP11-428G5.6
chr12	32225000	chr12	46485000	2.78E-02	promoter	super_enhancer	RP11-843B15.1
chr12	32255000	chr12	46265000	2.43E-02	promoter	super_enhancer	RP11-843B15.3
chr12	32255000	chr12	46475000	2.36E-02	promoter	super_enhancer	RP11-843B15.3
chr12	32255000	chr12	46525000	2.03E-02	promoter	super_enhancer	RP11-843B15.3
chr12	32255000	chr12	75985000	7.02E-03	promoter	super_enhancer	RP11-843B15.3

chr12	32255000	chr12	111405000	4.81E-03	promoter	super_enhancer	RP11-843B15.3
chr12	32505000	chr12	75955000	5.96E-03	promoter	super_enhancer	RP11-817I4.2
chr12	32505000	chr12	131855000	3.14E-03	promoter	super_enhancer	RP11-817I4.2
chr12	33015000	chr12	50245000	2.38E-02	promoter	super_enhancer	RP11-8P13.1
chr12	33015000	chr12	122425000	4.40E-03	promoter	super_enhancer	RP11-8P13.1
chr12	33195000	chr12	46475000	1.90E-02	promoter	super_enhancer	AC026357.1
chr12	34175000	chr12	63855000	5.65E-03	promoter	super_enhancer	ALG10
chr12	38555000	chr12	52925000	2.10E-02	promoter	super_enhancer	RNA5SP358
chr12	38555000	chr12	63925000	9.69E-03	promoter	super_enhancer	RNA5SP358
chr12	38595000	chr12	54415000	2.42E-02	promoter	super_enhancer	TUBB8P5
chr12	38985000	chr12	124385000	3.22E-03	promoter	super_enhancer	RP11-804F13.1
chr12	39455000	chr12	63895000	5.94E-03	promoter	super_enhancer	RP11-554L12.2
chr12	39455000	chr12	123065000	2.80E-03	promoter	super_enhancer	RP11-554L12.2
chr12	39935000	chr12	63925000	1.28E-02	promoter	super_enhancer	RP11-242C24.3
chr12	39935000	chr12	96435000	5.75E-03	promoter	super_enhancer	RP11-242C24.3
chr12	40215000	chr12	55735000	1.35E-02	promoter	super_enhancer	SNORA22
chr12	41085000	chr12	55735000	2.00E-02	promoter	super_enhancer	CNTN1
chr12	42095000	chr12	46465000	4.35E-02	promoter	super_enhancer	MTND1P24
chr12	42305000	chr12	47755000	8.43E-04	promoter	super_enhancer	RP11-2A1.1
chr12	42305000	chr12	55725000	2.15E-02	promoter	super_enhancer	RP11-2A1.1
chr12	42465000	chr12	75955000	7.52E-03	promoter	super_enhancer	RP11-440G2.2
chr12	42755000	chr12	52875000	1.87E-02	promoter	super_enhancer	RP11-351C21.2
chr12	43085000	chr12	47755000	3.95E-02	promoter	super_enhancer	RP11-25I15.3
chr12	43085000	chr12	50805000	3.21E-02	promoter	super_enhancer	RP11-25I15.3

chr12	43325000	chr12	52235000	2.39E-02	promoter	super_enhancer	RP11-510P12.1
chr12	43685000	chr12	50815000	3.94E-02	promoter	super_enhancer	AC079603.1
chr12	43685000	chr12	56525000	2.46E-02	promoter	super_enhancer	AC079603.1
chr12	43955000	chr12	131865000	3.10E-03	promoter	super_enhancer	RP11-73B8.1
chr12	44405000	chr12	47825000	3.56E-02	promoter	super_enhancer	ZNF75BP
chr12	45445000	chr12	46435000	9.93E-03	promoter	super_enhancer	DBX2
chr12	45445000	chr12	56525000	2.69E-02	promoter	super_enhancer	DBX2
chr12	45465000	chr12	46385000	2.68E-02	promoter	super_enhancer	RP11-478B9.2
chr12	45595000	chr12	120265000	3.56E-03	promoter	super_enhancer	RP11-139E19.2
chr12	46005000	chr12	95205000	7.75E-03	promoter	super_enhancer	RP11-352M15.1
chr12	46265000	chr12	48875000	5.62E-03	super_enhancer	promoter	C12orf54
chr12	46265000	chr12	54655000	2.94E-02	super_enhancer	promoter	RP11-968A15.2
chr12	46265000	chr12	56225000	3.60E-02	super_enhancer	promoter	DNAJC14
chr12	46265000	chr12	57915000	3.12E-02	super_enhancer	promoter	DDIT3
chr12	46265000	chr12	75785000	8.22E-03	super_enhancer	promoter	CAPS2
chr12	46265000	chr12	105465000	5.72E-03	super_enhancer	promoter	RP11-61E11.1
chr12	46385000	chr12	48895000	8.13E-03	super_enhancer	promoter	RP11-722P11.4
chr12	46385000	chr12	53405000	4.01E-02	super_enhancer	promoter	RP11-983P16.2
chr12	46385000	chr12	54785000	4.91E-02	super_enhancer	promoter	ZNF385A
chr12	46385000	chr12	56265000	4.00E-02	super_enhancer	promoter	RN7SL676P
chr12	46385000	chr12	57145000	3.09E-02	super_enhancer	promoter	HSD17B6
chr12	46385000	chr12	66565000	1.82E-02	super_enhancer	promoter	RP11-745O10.4
chr12	46385000	chr12	90675000	8.14E-03	super_enhancer	promoter	RP11-753N8.1
chr12	46385000	chr12	100595000	6.72E-03	super_enhancer	promoter	RP11-175P13.3

chr12	46385000	chr12	120705000	5.82E-03	super_enhancer	promoter	PXN
chr12	46435000	chr12	66485000	1.40E-02	super_enhancer	promoter	AC078927.1
chr12	46435000	chr12	113375000	4.63E-03	super_enhancer	promoter	OAS3
chr12	46435000	chr12	130555000	3.21E-03	super_enhancer	promoter	RP11-474D1.2
chr12	46445000	chr12	57065000	2.53E-02	super_enhancer	promoter	RN7SL809P
chr12	46445000	chr12	78745000	7.14E-03	super_enhancer	promoter	RP11-754N21.1
chr12	46445000	chr12	110435000	4.59E-03	super_enhancer	promoter	GIT2
chr12	46455000	chr12	52625000	4.14E-02	super_enhancer	promoter	KRT7
chr12	46455000	chr12	53575000	3.88E-02	super_enhancer	promoter	CSAD
chr12	46455000	chr12	53695000	5.46E-04	super_enhancer	promoter	RP11-680A11.5
chr12	46455000	chr12	56265000	3.19E-02	super_enhancer	promoter	RN7SL676P
chr12	46465000	chr12	48095000	6.93E-06	super_enhancer	promoter	RP1-197B17.5
chr12	46465000	chr12	48165000	3.63E-02	super_enhancer	promoter	RAPGEF3
chr12	46465000	chr12	57065000	2.46E-02	super_enhancer	promoter	RN7SL809P
chr12	46465000	chr12	111065000	4.93E-03	super_enhancer	promoter	RN7SL387P
chr12	46465000	chr12	116955000	4.77E-03	super_enhancer	promoter	RP11-148B3.1
chr12	46475000	chr12	48095000	2.81E-03	super_enhancer	promoter	RP1-197B17.5
chr12	46475000	chr12	48435000	1.59E-02	super_enhancer	promoter	RP1-228P16.8
chr12	46475000	chr12	48565000	1.94E-02	super_enhancer	promoter	RP11-474C8.5
chr12	46475000	chr12	48575000	2.46E-02	super_enhancer	promoter	ASB8
chr12	46475000	chr12	49195000	1.22E-02	super_enhancer	promoter	RP11-579D7.8
chr12	46475000	chr12	120495000	4.61E-03	super_enhancer	promoter	RP1-267D11.1
chr12	46485000	chr12	63755000	1.27E-02	super_enhancer	promoter	RP11-1022B3.1
chr12	46485000	chr12	78525000	7.83E-03	super_enhancer	promoter	RP11-136F16.2

chr12	46485000	chr12	117425000	3.34E-03	super_enhancer	promoter	RP11-231I16.1
chr12	46495000	chr12	53405000	2.27E-02	super_enhancer	promoter	RP11-983P16.2
chr12	46495000	chr12	69815000	9.97E-03	super_enhancer	promoter	RP11-159A18.1
chr12	46525000	chr12	46765000	4.52E-02	super_enhancer	promoter	RP11-474P2.2
chr12	46525000	chr12	53845000	2.69E-02	super_enhancer	promoter	PCBP2
chr12	46525000	chr12	54675000	3.01E-02	super_enhancer	promoter	CBX5
chr12	46535000	chr12	46765000	2.15E-02	super_enhancer	promoter	RP11-474P2.2
chr12	46535000	chr12	47475000	1.27E-02	super_enhancer	promoter	AMIGO2
chr12	46535000	chr12	48125000	5.35E-04	super_enhancer	promoter	RP1-197B17.7
chr12	46535000	chr12	52345000	4.86E-02	super_enhancer	promoter	ACVR1B
chr12	46535000	chr12	53345000	4.91E-02	super_enhancer	promoter	KRT8
chr12	46535000	chr12	71065000	1.17E-02	super_enhancer	promoter	FAHD2P1
chr12	46535000	chr12	90275000	6.34E-03	super_enhancer	promoter	RP11-654D12.2
chr12	46535000	chr12	100405000	4.49E-03	super_enhancer	promoter	RPS4XP1
chr12	46545000	chr12	53075000	4.92E-02	super_enhancer	promoter	KRT1
chr12	46545000	chr12	56905000	2.54E-02	super_enhancer	promoter	RP11-153M3.1
chr12	46545000	chr12	58175000	2.02E-02	super_enhancer	promoter	TSFM
chr12	46545000	chr12	66525000	1.09E-02	super_enhancer	promoter	LLPH
chr12	46555000	chr12	48095000	3.50E-02	super_enhancer	promoter	RP1-197B17.5
chr12	46555000	chr12	83545000	7.85E-03	super_enhancer	promoter	RPL6P25
chr12	46555000	chr12	121415000	4.41E-03	super_enhancer	promoter	HNF1A
chr12	46555000	chr12	122235000	5.33E-03	super_enhancer	promoter	RP11-347I19.8
chr12	46665000	chr12	52905000	4.75E-02	promoter	super_enhancer	SLC38A1
chr12	46665000	chr12	63855000	1.44E-02	promoter	super_enhancer	SLC38A1

chr12	46765000	chr12	52915000	3.61E-02	promoter	super_enhancer	RP11-474P2.2
chr12	46985000	chr12	52955000	4.13E-02	promoter	super_enhancer	OR7A19P
chr12	47275000	chr12	48095000	2.36E-02	super_enhancer	promoter	RP1-197B17.5
chr12	47275000	chr12	57915000	2.77E-02	super_enhancer	promoter	DDIT3
chr12	47755000	chr12	56755000	2.98E-02	super_enhancer	promoter	STAT2
chr12	47755000	chr12	57635000	3.35E-02	super_enhancer	promoter	NDUFA4L2
chr12	47755000	chr12	65815000	1.56E-02	super_enhancer	promoter	KRT18P60
chr12	47755000	chr12	120705000	4.69E-03	super_enhancer	promoter	PXN
chr12	47825000	chr12	48895000	2.71E-02	super_enhancer	promoter	RP11-722P11.4
chr12	47825000	chr12	55615000	1.11E-02	super_enhancer	promoter	OR10A7
chr12	47825000	chr12	57905000	1.62E-02	super_enhancer	promoter	MIR6758
chr12	47825000	chr12	85065000	5.27E-03	super_enhancer	promoter	RP11-701B6.1
chr12	47825000	chr12	91755000	3.02E-03	super_enhancer	promoter	RP11-121E16.1
chr12	48165000	chr12	95625000	7.71E-03	promoter	super_enhancer	RAPGEF3
chr12	48275000	chr12	52245000	5.75E-03	promoter	super_enhancer	RP11-89H19.1
chr12	48275000	chr12	95645000	8.65E-03	promoter	super_enhancer	RP11-89H19.1
chr12	48275000	chr12	124385000	6.54E-03	promoter	super_enhancer	RP11-89H19.1
chr12	48405000	chr12	63845000	1.74E-02	promoter	super_enhancer	RP1-228P16.3
chr12	48565000	chr12	122425000	4.83E-03	promoter	super_enhancer	RP11-474C8.5
chr12	48575000	chr12	63855000	1.89E-02	promoter	super_enhancer	ASB8
chr12	48575000	chr12	76015000	1.26E-02	promoter	super_enhancer	ASB8
chr12	48575000	chr12	124855000	4.42E-03	promoter	super_enhancer	ASB8
chr12	48575000	chr12	131895000	6.54E-03	promoter	super_enhancer	ASB8
chr12	48745000	chr12	63855000	1.57E-02	promoter	super_enhancer	ZNF641

chr12	48745000	chr12	75975000	1.19E-02	promoter	super_enhancer	ZNF641
chr12	48755000	chr12	63925000	1.94E-02	promoter	super_enhancer	RP11-370I10.11
chr12	48755000	chr12	96445000	5.83E-03	promoter	super_enhancer	RP11-370I10.11
chr12	48865000	chr12	56525000	3.41E-02	promoter	super_enhancer	ANP32D
chr12	48895000	chr12	95625000	5.35E-03	promoter	super_enhancer	RP11-722P11.4
chr12	48965000	chr12	120235000	5.29E-03	promoter	super_enhancer	LALBA
chr12	49455000	chr12	123145000	5.61E-03	promoter	super_enhancer	KMT2D
chr12	49465000	chr12	75985000	1.35E-02	promoter	super_enhancer	RHEBL1
chr12	49525000	chr12	56525000	4.03E-02	promoter	super_enhancer	TUBA1B
chr12	49525000	chr12	95645000	6.97E-03	promoter	super_enhancer	TUBA1B
chr12	50165000	chr12	50225000	1.47E-02	promoter	super_enhancer	LSM6P2
chr12	50165000	chr12	50235000	3.69E-03	promoter	super_enhancer	LSM6P2
chr12	50165000	chr12	52235000	3.10E-02	promoter	super_enhancer	LSM6P2
chr12	50165000	chr12	93805000	9.45E-03	promoter	super_enhancer	LSM6P2
chr12	50165000	chr12	131885000	4.39E-03	promoter	super_enhancer	LSM6P2
chr12	50195000	chr12	51665000	1.84E-02	super_enhancer	promoter	SMAGP
chr12	50195000	chr12	56515000	4.54E-02	super_enhancer	promoter	RP11-603J24.6
chr12	50195000	chr12	69605000	1.95E-02	super_enhancer	promoter	RP11-324P9.1
chr12	50195000	chr12	121535000	5.66E-03	super_enhancer	promoter	RP11-216P16.5
chr12	50205000	chr12	50355000	3.60E-02	super_enhancer	promoter	AQP5
chr12	50205000	chr12	56585000	4.50E-02	super_enhancer	promoter	RP11-977G19.5
chr12	50215000	chr12	56545000	4.72E-02	super_enhancer	promoter	MYL6B
chr12	50215000	chr12	57845000	4.04E-02	super_enhancer	promoter	INHBE
chr12	50215000	chr12	70005000	2.15E-02	super_enhancer	promoter	LRRC10

chr12	50215000	chr12	112205000	6.96E-03	super_enhancer	promoter	ALDH2
chr12	50225000	chr12	60205000	1.99E-02	super_enhancer	promoter	RP11-813P10.2
chr12	50225000	chr12	65015000	2.11E-02	super_enhancer	promoter	MIR548C
chr12	50225000	chr12	68845000	2.16E-02	super_enhancer	promoter	RP11-81H14.2
chr12	50225000	chr12	117425000	4.51E-03	super_enhancer	promoter	RP11-231I16.1
chr12	50225000	chr12	132415000	5.30E-03	super_enhancer	promoter	RP11-417L19.4
chr12	50235000	chr12	65015000	2.16E-02	super_enhancer	promoter	MIR548C
chr12	50235000	chr12	68395000	1.35E-02	super_enhancer	promoter	RPL39P28
chr12	50235000	chr12	133795000	5.63E-03	super_enhancer	promoter	AC226150.3
chr12	50245000	chr12	56755000	4.52E-02	super_enhancer	promoter	STAT2
chr12	50245000	chr12	63245000	2.13E-02	super_enhancer	promoter	RNU1-83P
chr12	50245000	chr12	79895000	1.41E-02	super_enhancer	promoter	RP11-359M6.2
chr12	50245000	chr12	133615000	4.07E-03	super_enhancer	promoter	RP11-386I8.6
chr12	50255000	chr12	50355000	4.71E-02	super_enhancer	promoter	AQP5
chr12	50255000	chr12	57125000	4.33E-02	super_enhancer	promoter	NACA
chr12	50255000	chr12	122345000	5.36E-03	super_enhancer	promoter	RNU7-170P
chr12	50305000	chr12	104685000	1.07E-02	promoter	super_enhancer	RP11-70F11.8
chr12	50305000	chr12	124825000	4.92E-03	promoter	super_enhancer	RP11-70F11.8
chr12	50345000	chr12	52155000	4.58E-03	promoter	super_enhancer	AQP2
chr12	50345000	chr12	52205000	4.14E-02	promoter	super_enhancer	AQP2
chr12	50355000	chr12	116525000	5.80E-03	promoter	super_enhancer	AQP5
chr12	50505000	chr12	50765000	1.75E-02	promoter	super_enhancer	COX14
chr12	50505000	chr12	52235000	4.82E-02	promoter	super_enhancer	COX14
chr12	50505000	chr12	63905000	2.57E-02	promoter	super_enhancer	COX14

chr12	50585000	chr12	50785000	2.36E-02	promoter	super_enhancer	RP3-405J10.3
chr12	50765000	chr12	56585000	4.74E-02	super_enhancer	promoter	RP11-977G19.5
chr12	50765000	chr12	65175000	2.39E-02	super_enhancer	promoter	TBC1D30
chr12	50765000	chr12	66645000	2.35E-02	super_enhancer	promoter	MIR6502
chr12	50765000	chr12	121415000	5.21E-03	super_enhancer	promoter	HNF1A
chr12	50775000	chr12	109885000	7.68E-03	super_enhancer	promoter	RP11-256L11.3
chr12	50785000	chr12	63395000	3.02E-02	super_enhancer	promoter	RP11-848D3.4
chr12	50785000	chr12	64615000	2.58E-02	super_enhancer	promoter	RPS11P6
chr12	50785000	chr12	72245000	1.90E-02	super_enhancer	promoter	RP11-2H8.4
chr12	50785000	chr12	81255000	1.10E-02	super_enhancer	promoter	RP11-299A16.1
chr12	50785000	chr12	110305000	7.82E-03	super_enhancer	promoter	RN7SL441P
chr12	50795000	chr12	56615000	4.75E-02	super_enhancer	promoter	NABP2
chr12	50795000	chr12	64515000	1.86E-02	super_enhancer	promoter	RP11-196H14.4
chr12	50805000	chr12	55615000	3.62E-02	super_enhancer	promoter	OR10A7
chr12	50805000	chr12	58175000	3.67E-02	super_enhancer	promoter	TSFM
chr12	50805000	chr12	94225000	8.88E-03	super_enhancer	promoter	RN7SL630P
chr12	50805000	chr12	119615000	5.95E-03	super_enhancer	promoter	RP11-64B16.3
chr12	50805000	chr12	122985000	5.58E-03	super_enhancer	promoter	RP11-15J22.8
chr12	50815000	chr12	79895000	1.68E-02	super_enhancer	promoter	RP11-359M6.2
chr12	50815000	chr12	121125000	6.15E-03	super_enhancer	promoter	MLEC
chr12	50825000	chr12	57145000	4.46E-02	super_enhancer	promoter	HSD17B6
chr12	50825000	chr12	57985000	4.23E-02	super_enhancer	promoter	PIP4K2C
chr12	51665000	chr12	52245000	2.57E-02	promoter	super_enhancer	SMAGP
chr12	51665000	chr12	104265000	7.00E-03	promoter	super_enhancer	SMAGP

chr12	51665000	chr12	123145000	5.67E-03	promoter	super_enhancer	SMAGP
chr12	51785000	chr12	116405000	4.24E-03	promoter	super_enhancer	SLC4A8
chr12	52145000	chr12	69815000	2.18E-02	super_enhancer	promoter	RP11-159A18.1
chr12	52145000	chr12	71315000	1.48E-02	super_enhancer	promoter	PTPRR
chr12	52145000	chr12	104945000	8.21E-03	super_enhancer	promoter	RP11-43D4.3
chr12	52155000	chr12	93345000	1.09E-02	super_enhancer	promoter	RNU6-1329P
chr12	52155000	chr12	110385000	5.79E-03	super_enhancer	promoter	RP11-837J7.4
chr12	52155000	chr12	120685000	6.06E-03	super_enhancer	promoter	RPS20P31
chr12	52155000	chr12	133615000	4.59E-03	super_enhancer	promoter	RP11-386I8.6
chr12	52185000	chr12	132195000	3.90E-03	super_enhancer	promoter	SFSWAP
chr12	52195000	chr12	116325000	4.42E-03	super_enhancer	promoter	RP11-110L15.2
chr12	52195000	chr12	117425000	3.63E-03	super_enhancer	promoter	RP11-231I16.1
chr12	52195000	chr12	122345000	4.59E-03	super_enhancer	promoter	RNU7-170P
chr12	52195000	chr12	133405000	4.29E-03	super_enhancer	promoter	GOLGA3
chr12	52205000	chr12	125385000	5.87E-03	super_enhancer	promoter	RNU6-927P
chr12	52235000	chr12	52735000	4.71E-02	super_enhancer	promoter	KRT89P
chr12	52235000	chr12	94855000	8.95E-03	super_enhancer	promoter	CEP83
chr12	52245000	chr12	53075000	3.40E-02	super_enhancer	promoter	KRT1
chr12	52245000	chr12	90275000	9.34E-03	super_enhancer	promoter	RP11-654D12.2
chr12	52245000	chr12	117195000	4.65E-03	super_enhancer	promoter	RP11-407P2.1
chr12	52285000	chr12	67985000	2.10E-02	super_enhancer	promoter	RP11-335O4.1
chr12	52285000	chr12	76425000	2.08E-02	super_enhancer	promoter	RP11-290L1.2
chr12	52285000	chr12	110935000	7.57E-03	super_enhancer	promoter	SNORD50
chr12	52345000	chr12	52945000	2.11E-02	promoter	super_enhancer	ACVR1B

chr12	52505000	chr12	52975000	3.56E-03	promoter	super_enhancer	AC078864.1
chr12	52505000	chr12	122415000	2.69E-03	promoter	super_enhancer	AC078864.1
chr12	52605000	chr12	53375000	2.38E-02	promoter	super_enhancer	C12orf80
chr12	52605000	chr12	53385000	5.90E-04	promoter	super_enhancer	C12orf80
chr12	52625000	chr12	53235000	9.73E-04	promoter	super_enhancer	KRT7
chr12	52625000	chr12	121795000	6.07E-03	promoter	super_enhancer	KRT7
chr12	52625000	chr12	124865000	5.96E-03	promoter	super_enhancer	KRT7
chr12	52635000	chr12	52975000	1.28E-02	promoter	super_enhancer	AC021066.1
chr12	52635000	chr12	75995000	1.74E-02	promoter	super_enhancer	AC021066.1
chr12	52685000	chr12	104265000	4.17E-03	promoter	super_enhancer	KRT81
chr12	52715000	chr12	52985000	2.01E-02	promoter	super_enhancer	KRT83
chr12	52715000	chr12	53045000	1.29E-02	promoter	super_enhancer	KRT83
chr12	52715000	chr12	53375000	3.67E-05	promoter	super_enhancer	KRT83
chr12	52715000	chr12	53385000	3.75E-03	promoter	super_enhancer	KRT83
chr12	52715000	chr12	76015000	1.55E-02	promoter	super_enhancer	KRT83
chr12	52715000	chr12	113195000	7.03E-03	promoter	super_enhancer	KRT83
chr12	52715000	chr12	121805000	7.71E-03	promoter	super_enhancer	KRT83
chr12	52715000	chr12	123115000	6.50E-03	promoter	super_enhancer	KRT83
chr12	52735000	chr12	52905000	1.94E-02	promoter	super_enhancer	KRT89P
chr12	52735000	chr12	52975000	2.43E-02	promoter	super_enhancer	KRT89P
chr12	52735000	chr12	53355000	3.18E-02	promoter	super_enhancer	KRT89P
chr12	52735000	chr12	53375000	6.83E-03	promoter	super_enhancer	KRT89P
chr12	52735000	chr12	53385000	2.44E-02	promoter	super_enhancer	KRT89P
chr12	52865000	chr12	53215000	2.25E-02	promoter	super_enhancer	AC055736.4

chr12	52875000	chr12	118575000	4.15E-03	super_enhancer	promoter	VSIG10
chr12	52885000	chr12	60115000	2.60E-02	super_enhancer	promoter	RP11-813P10.1
chr12	52885000	chr12	120685000	6.39E-03	super_enhancer	promoter	RPS20P31
chr12	52905000	chr12	53175000	3.83E-02	super_enhancer	promoter	RP11-153F5.2
chr12	52905000	chr12	95775000	8.19E-03	super_enhancer	promoter	RP11-167N24.6
chr12	52905000	chr12	104425000	5.27E-03	super_enhancer	promoter	RP11-490H24.5
chr12	52905000	chr12	132675000	4.75E-03	super_enhancer	promoter	RP13-977J11.2
chr12	52915000	chr12	53405000	3.99E-02	super_enhancer	promoter	RP11-983P16.2
chr12	52915000	chr12	104985000	6.74E-03	super_enhancer	promoter	MIR3922
chr12	52915000	chr12	123565000	4.35E-03	super_enhancer	promoter	RP11-197N18.7
chr12	52925000	chr12	64225000	2.49E-02	super_enhancer	promoter	AC084357.2
chr12	52925000	chr12	107305000	7.29E-03	super_enhancer	promoter	RPL30P12
chr12	52935000	chr12	53075000	2.65E-04	super_enhancer	promoter	KRT1
chr12	52935000	chr12	53175000	4.96E-02	super_enhancer	promoter	RP11-153F5.2
chr12	52935000	chr12	66695000	2.60E-02	super_enhancer	promoter	HELB
chr12	52935000	chr12	94855000	1.04E-02	super_enhancer	promoter	CEP83
chr12	52935000	chr12	95775000	1.02E-02	super_enhancer	promoter	RP11-167N24.6
chr12	52935000	chr12	104945000	9.88E-03	super_enhancer	promoter	RP11-43D4.3
chr12	52935000	chr12	108955000	8.70E-03	super_enhancer	promoter	SART3
chr12	52945000	chr12	60205000	3.04E-02	super_enhancer	promoter	RP11-813P10.2
chr12	52945000	chr12	66695000	2.93E-02	super_enhancer	promoter	HELB
chr12	52945000	chr12	112855000	9.21E-03	super_enhancer	promoter	PTPN11
chr12	52945000	chr12	115205000	8.01E-03	super_enhancer	promoter	RP4-601P9.1
chr12	52955000	chr12	70965000	1.72E-02	super_enhancer	promoter	RP11-785D18.3

chr12	52955000	chr12	120705000	5.56E-03	super_enhancer	promoter	PXN
chr12	52955000	chr12	123495000	5.89E-03	super_enhancer	promoter	MIR4304
chr12	52965000	chr12	64425000	2.95E-02	super_enhancer	promoter	RP11-1029F8.1
chr12	52965000	chr12	96455000	9.68E-03	super_enhancer	promoter	YPEL5P3
chr12	52975000	chr12	66645000	3.23E-02	super_enhancer	promoter	MIR6502
chr12	52975000	chr12	85065000	1.40E-02	super_enhancer	promoter	RP11-701B6.1
chr12	52975000	chr12	121125000	7.16E-03	super_enhancer	promoter	MLEC
chr12	52975000	chr12	133065000	4.91E-03	super_enhancer	promoter	FBRSL1
chr12	52985000	chr12	113375000	7.50E-03	super_enhancer	promoter	OAS3
chr12	52995000	chr12	53175000	4.50E-02	super_enhancer	promoter	RP11-153F5.2
chr12	52995000	chr12	96185000	5.80E-03	super_enhancer	promoter	NTN4
chr12	53045000	chr12	93925000	1.25E-02	super_enhancer	promoter	RP11-356O22.4
chr12	53045000	chr12	112365000	7.18E-03	super_enhancer	promoter	ADAM1B
chr12	53075000	chr12	95625000	9.68E-03	promoter	super_enhancer	KRT1
chr12	53075000	chr12	124385000	7.93E-03	promoter	super_enhancer	KRT1
chr12	53085000	chr12	124395000	6.65E-03	promoter	super_enhancer	RP11-641A6.3
chr12	53175000	chr12	63905000	2.91E-02	promoter	super_enhancer	RP11-153F5.2
chr12	53175000	chr12	124925000	6.68E-03	promoter	super_enhancer	RP11-153F5.2
chr12	53215000	chr12	56495000	7.01E-03	super_enhancer	promoter	RP11-603J24.9
chr12	53215000	chr12	109125000	6.03E-03	super_enhancer	promoter	CORO1C
chr12	53215000	chr12	110905000	7.51E-03	super_enhancer	promoter	FAM216A
chr12	53215000	chr12	112205000	7.76E-03	super_enhancer	promoter	ALDH2
chr12	53215000	chr12	120685000	6.69E-03	super_enhancer	promoter	RPS20P31
chr12	53215000	chr12	132675000	5.73E-03	super_enhancer	promoter	RP13-977J11.2

chr12	53235000	chr12	63395000	3.62E-02	super_enhancer	promoter	RP11-848D3.4
chr12	53235000	chr12	69795000	2.57E-02	super_enhancer	promoter	RN7SL804P
chr12	53235000	chr12	98075000	8.94E-03	super_enhancer	promoter	RP11-528M18.3
chr12	53235000	chr12	113795000	8.24E-03	super_enhancer	promoter	PLBD2
chr12	53235000	chr12	115025000	6.42E-03	super_enhancer	promoter	OSTF1P1
chr12	53235000	chr12	123495000	8.85E-03	super_enhancer	promoter	MIR4304
chr12	53355000	chr12	75785000	1.25E-02	super_enhancer	promoter	CAPS2
chr12	53355000	chr12	111285000	7.82E-03	super_enhancer	promoter	CCDC63
chr12	53365000	chr12	98075000	6.95E-03	super_enhancer	promoter	RP11-528M18.3
chr12	53365000	chr12	104945000	8.58E-03	super_enhancer	promoter	RP11-43D4.3
chr12	53375000	chr12	120315000	8.12E-03	super_enhancer	promoter	CIT
chr12	53385000	chr12	68065000	2.14E-02	super_enhancer	promoter	RP11-335O4.3
chr12	53385000	chr12	69815000	2.47E-02	super_enhancer	promoter	RP11-159A18.1
chr12	53385000	chr12	79895000	1.79E-02	super_enhancer	promoter	RP11-359M6.2
chr12	53385000	chr12	94955000	1.01E-02	super_enhancer	promoter	MIR5700
chr12	53535000	chr12	123145000	5.72E-03	promoter	super_enhancer	HIGD1AP1
chr12	53535000	chr12	124845000	7.31E-03	promoter	super_enhancer	HIGD1AP1
chr12	53695000	chr12	95635000	7.35E-03	promoter	super_enhancer	RP11-680A11.5
chr12	53695000	chr12	124865000	5.61E-03	promoter	super_enhancer	RP11-680A11.5
chr12	53895000	chr12	70745000	2.07E-02	promoter	super_enhancer	MAP3K12
chr12	54385000	chr12	75965000	2.07E-02	promoter	super_enhancer	MIR196A2
chr12	54385000	chr12	113205000	6.72E-03	promoter	super_enhancer	MIR196A2
chr12	54395000	chr12	122925000	4.69E-03	promoter	super_enhancer	HOXC-AS1
chr12	54395000	chr12	124825000	3.98E-03	promoter	super_enhancer	HOXC-AS1

chr12	54415000	chr12	63665000	1.67E-02	super_enhancer	promoter	HNRNPA1P69
chr12	54415000	chr12	65565000	2.17E-02	super_enhancer	promoter	RP11-305O6.4
chr12	54415000	chr12	69365000	1.66E-02	super_enhancer	promoter	CPM
chr12	54415000	chr12	98985000	8.84E-03	super_enhancer	promoter	RP11-481K9.4
chr12	54415000	chr12	109535000	7.69E-03	super_enhancer	promoter	UNG
chr12	54475000	chr12	76015000	1.35E-02	promoter	super_enhancer	RP11-834C11.6
chr12	54475000	chr12	123145000	5.30E-03	promoter	super_enhancer	RP11-834C11.6
chr12	54625000	chr12	123125000	6.00E-03	promoter	super_enhancer	MIR3198-2
chr12	54695000	chr12	93805000	9.19E-03	promoter	super_enhancer	NFE2
chr12	54785000	chr12	122935000	5.97E-03	promoter	super_enhancer	ZNF385A
chr12	54785000	chr12	124825000	6.00E-03	promoter	super_enhancer	ZNF385A
chr12	55005000	chr12	113205000	5.17E-03	promoter	super_enhancer	GLYCAM1
chr12	55655000	chr12	75935000	1.24E-02	promoter	super_enhancer	OR6C69P
chr12	55725000	chr12	75605000	1.57E-02	super_enhancer	promoter	KCNC2
chr12	55725000	chr12	75785000	1.03E-02	super_enhancer	promoter	CAPS2
chr12	55725000	chr12	79895000	1.16E-04	super_enhancer	promoter	RP11-359M6.2
chr12	55725000	chr12	83565000	7.25E-03	super_enhancer	promoter	RP11-87P13.2
chr12	55725000	chr12	94965000	8.40E-03	super_enhancer	promoter	MIR7844
chr12	55725000	chr12	108275000	6.92E-03	super_enhancer	promoter	RP11-554D14.4
chr12	55735000	chr12	55945000	1.75E-02	super_enhancer	promoter	OR6C4
chr12	55735000	chr12	59805000	3.93E-02	super_enhancer	promoter	RP11-272B17.1
chr12	55735000	chr12	63665000	1.53E-02	super_enhancer	promoter	HNRNPA1P69
chr12	55735000	chr12	69005000	1.65E-02	super_enhancer	promoter	RAP1B
chr12	55735000	chr12	72105000	1.35E-02	super_enhancer	promoter	RP11-498M15.1

chr12	55865000	chr12	63845000	1.92E-02	promoter	super_enhancer	OR6C70
chr12	55865000	chr12	63865000	2.20E-02	promoter	super_enhancer	OR6C70
chr12	55865000	chr12	63895000	1.62E-02	promoter	super_enhancer	OR6C70
chr12	55915000	chr12	63895000	2.02E-02	promoter	super_enhancer	OR6C64P
chr12	55915000	chr12	75995000	1.40E-02	promoter	super_enhancer	OR6C64P
chr12	55945000	chr12	63865000	2.88E-02	promoter	super_enhancer	OR6C4
chr12	55945000	chr12	63895000	2.12E-02	promoter	super_enhancer	OR6C4
chr12	55945000	chr12	121785000	6.13E-03	promoter	super_enhancer	OR6C4
chr12	56005000	chr12	63855000	1.89E-02	promoter	super_enhancer	OR6U2P
chr12	56055000	chr12	95625000	9.51E-03	promoter	super_enhancer	RP11-644F5.16
chr12	56055000	chr12	111405000	9.59E-03	promoter	super_enhancer	RP11-644F5.16
chr12	56055000	chr12	131895000	6.51E-03	promoter	super_enhancer	RP11-644F5.16
chr12	56075000	chr12	124915000	6.85E-03	promoter	super_enhancer	METTL7B
chr12	56225000	chr12	75995000	1.73E-02	promoter	super_enhancer	DNAJC14
chr12	56225000	chr12	104685000	1.25E-02	promoter	super_enhancer	DNAJC14
chr12	56265000	chr12	70745000	2.11E-02	promoter	super_enhancer	RN7SL676P
chr12	56525000	chr12	56695000	2.17E-02	super_enhancer	promoter	CS
chr12	56525000	chr12	106695000	6.24E-03	super_enhancer	promoter	TCP11L2
chr12	56525000	chr12	107665000	6.27E-03	super_enhancer	promoter	SETP7
chr12	56525000	chr12	122985000	5.71E-03	super_enhancer	promoter	RP11-15J22.8
chr12	56525000	chr12	123565000	4.57E-03	super_enhancer	promoter	RP11-197N18.7
chr12	56615000	chr12	95655000	1.04E-02	promoter	super_enhancer	NABP2
chr12	56615000	chr12	116385000	6.01E-03	promoter	super_enhancer	NABP2
chr12	56615000	chr12	124865000	6.48E-03	promoter	super_enhancer	NABP2

chr12	56665000	chr12	93805000	8.53E-03	promoter	super_enhancer	RP11-977G19.14
chr12	56665000	chr12	131895000	5.07E-03	promoter	super_enhancer	RP11-977G19.14
chr12	56695000	chr12	116535000	4.61E-03	promoter	super_enhancer	CS
chr12	56695000	chr12	122915000	5.41E-03	promoter	super_enhancer	CS
chr12	56755000	chr12	116535000	5.11E-03	promoter	super_enhancer	STAT2
chr12	56905000	chr12	122415000	4.96E-03	promoter	super_enhancer	RP11-153M3.1
chr12	57065000	chr12	111405000	7.32E-03	promoter	super_enhancer	RN7SL809P
chr12	57125000	chr12	63895000	2.39E-02	promoter	super_enhancer	NACA
chr12	57125000	chr12	120265000	4.61E-03	promoter	super_enhancer	NACA
chr12	57235000	chr12	75955000	1.43E-02	promoter	super_enhancer	RP11-74M13.6
chr12	57235000	chr12	75995000	1.42E-02	promoter	super_enhancer	RP11-74M13.6
chr12	57335000	chr12	70745000	1.65E-02	promoter	super_enhancer	RP11-474N8.9
chr12	57335000	chr12	96445000	6.55E-03	promoter	super_enhancer	RP11-474N8.9
chr12	57445000	chr12	123145000	4.46E-03	promoter	super_enhancer	MYO1A
chr12	57635000	chr12	63925000	4.56E-02	promoter	super_enhancer	NDUFA4L2
chr12	57635000	chr12	104275000	1.10E-02	promoter	super_enhancer	NDUFA4L2
chr12	57645000	chr12	122935000	4.93E-03	promoter	super_enhancer	STAC3
chr12	57825000	chr12	63865000	3.72E-02	promoter	super_enhancer	R3HDM2
chr12	57825000	chr12	95625000	8.30E-03	promoter	super_enhancer	R3HDM2
chr12	57845000	chr12	63895000	2.54E-02	promoter	super_enhancer	INHBE
chr12	57845000	chr12	117145000	5.62E-03	promoter	super_enhancer	INHBE
chr12	57845000	chr12	123095000	5.10E-03	promoter	super_enhancer	INHBE
chr12	57915000	chr12	63915000	3.13E-02	promoter	super_enhancer	DDIT3
chr12	57915000	chr12	123085000	5.05E-03	promoter	super_enhancer	DDIT3

chr12	58115000	chr12	70735000	1.89E-02	promoter	super_enhancer	RP11-571M6.7
chr12	58115000	chr12	75955000	1.56E-02	promoter	super_enhancer	RP11-571M6.7
chr12	58165000	chr12	76015000	1.39E-02	promoter	super_enhancer	METTL21B
chr12	58335000	chr12	63865000	3.54E-02	promoter	super_enhancer	XRCC6BP1
chr12	59315000	chr12	63855000	2.39E-02	promoter	super_enhancer	LRIG3
chr12	59805000	chr12	104305000	5.50E-03	promoter	super_enhancer	RP11-272B17.1
chr12	59805000	chr12	123115000	3.52E-03	promoter	super_enhancer	RP11-272B17.1
chr12	59845000	chr12	123095000	3.71E-03	promoter	super_enhancer	RNU6-871P
chr12	60115000	chr12	104305000	4.92E-03	promoter	super_enhancer	RP11-813P10.1
chr12	61995000	chr12	104305000	7.75E-03	promoter	super_enhancer	RP11-410B16.1
chr12	63245000	chr12	116535000	4.48E-03	promoter	super_enhancer	RNU1-83P
chr12	63395000	chr12	123095000	5.23E-03	promoter	super_enhancer	RP11-848D3.4
chr12	63845000	chr12	69005000	2.77E-02	super_enhancer	promoter	RAP1B
chr12	63845000	chr12	118475000	4.54E-03	super_enhancer	promoter	RP11-33N14.5
chr12	63845000	chr12	120495000	4.66E-03	super_enhancer	promoter	RP1-267D11.1
chr12	63855000	chr12	69005000	2.48E-02	super_enhancer	promoter	RAP1B
chr12	63865000	chr12	67985000	3.64E-02	super_enhancer	promoter	RP11-335O4.1
chr12	63865000	chr12	130325000	3.27E-03	super_enhancer	promoter	RP11-572C21.2
chr12	63895000	chr12	64045000	3.85E-03	super_enhancer	promoter	RP11-972L6.2
chr12	63895000	chr12	67985000	2.71E-02	super_enhancer	promoter	RP11-335O4.1
chr12	63905000	chr12	83415000	1.02E-02	super_enhancer	promoter	RNU6-977P
chr12	63905000	chr12	83545000	1.20E-02	super_enhancer	promoter	RPL6P25
chr12	63905000	chr12	114405000	6.15E-03	super_enhancer	promoter	RBM19
chr12	63915000	chr12	68845000	3.13E-02	super_enhancer	promoter	RP11-81H14.2

chr12	63915000	chr12	74685000	1.18E-02	super_enhancer	promoter	VENTXP3
chr12	63915000	chr12	97885000	6.08E-03	super_enhancer	promoter	MIR1251
chr12	63915000	chr12	128365000	2.45E-03	super_enhancer	promoter	FLJ37505
chr12	63925000	chr12	66565000	4.21E-03	super_enhancer	promoter	RP11-745O10.4
chr12	63925000	chr12	70005000	3.51E-02	super_enhancer	promoter	LRRC10
chr12	63925000	chr12	70055000	2.62E-02	super_enhancer	promoter	RP11-588G21.1
chr12	63925000	chr12	88375000	8.09E-03	super_enhancer	promoter	AC090060.1
chr12	63925000	chr12	118475000	5.39E-03	super_enhancer	promoter	RP11-33N14.5
chr12	64045000	chr12	75965000	8.97E-03	promoter	super_enhancer	RP11-972L6.2
chr12	64215000	chr12	116535000	5.31E-03	promoter	super_enhancer	PABPC1P4
chr12	64225000	chr12	93795000	6.97E-03	promoter	super_enhancer	AC084357.2
chr12	64425000	chr12	121805000	6.38E-03	promoter	super_enhancer	RP11-1029F8.1
chr12	64515000	chr12	117145000	5.44E-03	promoter	super_enhancer	RP11-196H14.4
chr12	64515000	chr12	124635000	3.67E-03	promoter	super_enhancer	RP11-196H14.4
chr12	64615000	chr12	75995000	1.66E-02	promoter	super_enhancer	RPS11P6
chr12	65175000	chr12	104295000	7.14E-03	promoter	super_enhancer	TBC1D30
chr12	65675000	chr12	70745000	3.51E-02	promoter	super_enhancer	RP11-305O6.3
chr12	65675000	chr12	104685000	1.20E-02	promoter	super_enhancer	RP11-305O6.3
chr12	65815000	chr12	95625000	8.92E-03	promoter	super_enhancer	KRT18P60
chr12	66425000	chr12	75945000	2.19E-02	promoter	super_enhancer	RP11-366L20.4
chr12	66425000	chr12	75995000	2.09E-02	promoter	super_enhancer	RP11-366L20.4
chr12	66485000	chr12	75965000	2.27E-02	promoter	super_enhancer	AC078927.1
chr12	66525000	chr12	121795000	4.99E-03	promoter	super_enhancer	LLPH
chr12	66565000	chr12	122935000	4.99E-03	promoter	super_enhancer	RP11-745O10.4

chr12	66645000	chr12	123095000	6.34E-03	promoter	super_enhancer	MIR6502
chr12	68065000	chr12	93795000	7.98E-03	promoter	super_enhancer	RP11-335O4.3
chr12	68065000	chr12	124925000	5.75E-03	promoter	super_enhancer	RP11-335O4.3
chr12	68845000	chr12	75935000	2.69E-02	promoter	super_enhancer	RP11-81H14.2
chr12	68845000	chr12	95635000	1.07E-02	promoter	super_enhancer	RP11-81H14.2
chr12	68845000	chr12	122945000	6.59E-03	promoter	super_enhancer	RP11-81H14.2
chr12	68845000	chr12	124865000	7.11E-03	promoter	super_enhancer	RP11-81H14.2
chr12	69005000	chr12	104295000	6.39E-03	promoter	super_enhancer	RAP1B
chr12	69235000	chr12	124685000	4.21E-03	promoter	super_enhancer	RP11-611O2.3
chr12	69365000	chr12	95635000	7.91E-03	promoter	super_enhancer	CPM
chr12	69605000	chr12	70735000	2.92E-02	promoter	super_enhancer	RP11-324P9.1
chr12	69605000	chr12	95645000	1.00E-02	promoter	super_enhancer	RP11-324P9.1
chr12	69605000	chr12	123095000	5.30E-03	promoter	super_enhancer	RP11-324P9.1
chr12	69605000	chr12	123125000	5.43E-03	promoter	super_enhancer	RP11-324P9.1
chr12	69795000	chr12	70745000	5.44E-04	promoter	super_enhancer	RN7SL804P
chr12	69815000	chr12	76025000	3.22E-02	promoter	super_enhancer	RP11-159A18.1
chr12	70005000	chr12	122945000	7.01E-03	promoter	super_enhancer	LRRC10
chr12	70055000	chr12	96415000	9.39E-03	promoter	super_enhancer	RP11-588G21.1
chr12	70195000	chr12	70755000	2.95E-02	promoter	super_enhancer	AC025263.3
chr12	70735000	chr12	85065000	1.49E-02	super_enhancer	promoter	RP11-701B6.1
chr12	70745000	chr12	75415000	3.55E-02	super_enhancer	promoter	RP11-81K13.1
chr12	70745000	chr12	78225000	1.27E-02	super_enhancer	promoter	NAV3
chr12	70745000	chr12	92815000	1.05E-02	super_enhancer	promoter	CLLU1
chr12	70745000	chr12	109125000	5.54E-03	super_enhancer	promoter	CORO1C

chr12	71065000	chr12	104305000	8.66E-03	promoter	super_enhancer	FAHD2P1
chr12	72105000	chr12	75965000	4.52E-02	promoter	super_enhancer	RP11-498M15.1
chr12	72105000	chr12	104305000	8.44E-03	promoter	super_enhancer	RP11-498M15.1
chr12	72245000	chr12	75935000	4.81E-02	promoter	super_enhancer	RP11-2H8.4
chr12	75415000	chr12	75945000	3.93E-02	promoter	super_enhancer	RP11-81K13.1
chr12	75605000	chr12	96415000	1.46E-02	promoter	super_enhancer	KCNC2
chr12	75785000	chr12	104305000	8.12E-03	promoter	super_enhancer	CAPS2
chr12	75785000	chr12	122925000	5.12E-03	promoter	super_enhancer	CAPS2
chr12	75785000	chr12	131895000	4.67E-03	promoter	super_enhancer	CAPS2
chr12	75905000	chr12	123145000	4.31E-03	promoter	super_enhancer	KRR1
chr12	75935000	chr12	77115000	3.70E-02	super_enhancer	promoter	RPL7P43
chr12	75935000	chr12	92815000	1.36E-02	super_enhancer	promoter	CLLU1
chr12	75935000	chr12	95045000	1.67E-02	super_enhancer	promoter	TMCC3
chr12	75945000	chr12	88975000	1.75E-02	super_enhancer	promoter	KITLG
chr12	75945000	chr12	94225000	1.34E-02	super_enhancer	promoter	RN7SL630P
chr12	75965000	chr12	76955000	6.62E-03	super_enhancer	promoter	OSBPL8
chr12	75965000	chr12	89765000	1.81E-02	super_enhancer	promoter	RP11-1109F11.5
chr12	75965000	chr12	128925000	3.64E-03	super_enhancer	promoter	RP11-428I12.1
chr12	75975000	chr12	77115000	4.09E-03	super_enhancer	promoter	RPL7P43
chr12	75975000	chr12	98985000	1.23E-02	super_enhancer	promoter	RP11-481K9.4
chr12	75975000	chr12	108275000	1.01E-02	super_enhancer	promoter	RP11-554D14.4
chr12	75985000	chr12	101865000	1.00E-02	super_enhancer	promoter	RNA5SP367
chr12	75985000	chr12	116325000	6.97E-03	super_enhancer	promoter	RP11-110L15.2
chr12	75985000	chr12	125635000	6.57E-03	super_enhancer	promoter	RP11-158L12.5

chr12	75995000	chr12	76955000	4.59E-02	super_enhancer	promoter	OSBPL8
chr12	75995000	chr12	104985000	8.71E-03	super_enhancer	promoter	MIR3922
chr12	75995000	chr12	106645000	9.49E-03	super_enhancer	promoter	RP11-651L5.3
chr12	75995000	chr12	132585000	4.83E-03	super_enhancer	promoter	RP13-977J11.6
chr12	76015000	chr12	76425000	1.22E-02	super_enhancer	promoter	RP11-290L1.2
chr12	76015000	chr12	76955000	3.89E-03	super_enhancer	promoter	OSBPL8
chr12	76015000	chr12	120885000	6.10E-03	super_enhancer	promoter	TRIAP1
chr12	76015000	chr12	122745000	5.69E-03	super_enhancer	promoter	RP11-512M8.11
chr12	76015000	chr12	123465000	5.39E-03	super_enhancer	promoter	ARL6IP4
chr12	76025000	chr12	100595000	1.07E-02	super_enhancer	promoter	RP11-175P13.3
chr12	76025000	chr12	106695000	7.54E-03	super_enhancer	promoter	TCP11L2
chr12	76025000	chr12	113575000	7.91E-03	super_enhancer	promoter	RASAL1
chr12	76025000	chr12	124455000	5.28E-03	super_enhancer	promoter	RP11-214K3.19
chr12	76955000	chr12	96445000	1.06E-02	promoter	super_enhancer	OSBPL8
chr12	77115000	chr12	104295000	9.67E-03	promoter	super_enhancer	RPL7P43
chr12	78225000	chr12	121785000	4.79E-03	promoter	super_enhancer	NAV3
chr12	78755000	chr12	116385000	6.34E-03	promoter	super_enhancer	RP11-38F22.1
chr12	80085000	chr12	104305000	1.32E-02	promoter	super_enhancer	PAWR
chr12	80085000	chr12	124385000	8.95E-03	promoter	super_enhancer	PAWR
chr12	80085000	chr12	124635000	5.71E-03	promoter	super_enhancer	PAWR
chr12	83545000	chr12	104685000	1.79E-02	promoter	super_enhancer	RPL6P25
chr12	84055000	chr12	113185000	7.59E-03	promoter	super_enhancer	RP11-384P14.1
chr12	84665000	chr12	96425000	1.13E-02	promoter	super_enhancer	RP11-788H18.1
chr12	85065000	chr12	95625000	1.89E-02	promoter	super_enhancer	RP11-701B6.1

chr12	85065000	chr12	104265000	1.28E-02	promoter	super_enhancer	RP11-701B6.1
chr12	86275000	chr12	96445000	1.12E-02	promoter	super_enhancer	AC016993.1
chr12	87435000	chr12	124385000	8.08E-03	promoter	super_enhancer	RP11-324H9.1
chr12	87565000	chr12	120235000	9.00E-03	promoter	super_enhancer	RPL23AP68
chr12	88215000	chr12	116385000	8.23E-03	promoter	super_enhancer	RP11-248E9.7
chr12	88375000	chr12	122415000	6.62E-03	promoter	super_enhancer	AC090060.1
chr12	88445000	chr12	96445000	1.41E-02	promoter	super_enhancer	RNA5SP364
chr12	88535000	chr12	93805000	3.44E-02	promoter	super_enhancer	TMTC3
chr12	88535000	chr12	95635000	2.44E-02	promoter	super_enhancer	TMTC3
chr12	88535000	chr12	96435000	2.62E-02	promoter	super_enhancer	TMTC3
chr12	88535000	chr12	104265000	1.41E-02	promoter	super_enhancer	TMTC3
chr12	89745000	chr12	116385000	1.01E-02	promoter	super_enhancer	RP11-823E8.3
chr12	89765000	chr12	96435000	3.20E-02	promoter	super_enhancer	RP11-1109F11.5
chr12	89765000	chr12	123065000	1.06E-02	promoter	super_enhancer	RP11-1109F11.5
chr12	89765000	chr12	123095000	1.00E-02	promoter	super_enhancer	RP11-1109F11.5
chr12	89955000	chr12	131865000	9.99E-03	promoter	super_enhancer	RP11-981P6.1
chr12	90275000	chr12	131855000	5.75E-03	promoter	super_enhancer	RP11-654D12.2
chr12	90315000	chr12	104225000	1.61E-02	promoter	super_enhancer	BRWD1P2
chr12	92265000	chr12	93785000	1.67E-02	promoter	super_enhancer	RP11-587P21.1
chr12	92265000	chr12	96415000	4.41E-02	promoter	super_enhancer	RP11-587P21.1
chr12	92265000	chr12	104225000	1.68E-02	promoter	super_enhancer	RP11-587P21.1
chr12	92265000	chr12	121805000	1.12E-02	promoter	super_enhancer	RP11-587P21.1
chr12	92815000	chr12	124845000	1.26E-02	promoter	super_enhancer	CLLU1
chr12	93115000	chr12	93785000	2.63E-03	promoter	super_enhancer	PLEKHG7

chr12	93115000	chr12	104295000	1.56E-02	promoter	super_enhancer	PLEKHG7
chr12	93345000	chr12	113195000	1.63E-02	promoter	super_enhancer	RNU6-1329P
chr12	93785000	chr12	101605000	2.76E-02	super_enhancer	promoter	SLC5A8
chr12	93785000	chr12	111285000	1.85E-02	super_enhancer	promoter	CCDC63
chr12	93785000	chr12	120705000	1.44E-02	super_enhancer	promoter	PXN
chr12	93785000	chr12	120885000	1.31E-02	super_enhancer	promoter	TRIAP1
chr12	93795000	chr12	97885000	3.88E-02	super_enhancer	promoter	MIR1251
chr12	93805000	chr12	101805000	2.12E-02	super_enhancer	promoter	RNU6-1068P
chr12	93805000	chr12	102515000	1.48E-02	super_enhancer	promoter	NUP37
chr12	93805000	chr12	118605000	9.75E-03	super_enhancer	promoter	AC026366.1
chr12	93925000	chr12	122425000	1.30E-02	promoter	super_enhancer	RP11-356O22.4
chr12	93925000	chr12	131865000	1.07E-02	promoter	super_enhancer	RP11-356O22.4
chr12	94035000	chr12	95635000	1.30E-02	promoter	super_enhancer	RP11-887P2.3
chr12	94035000	chr12	123125000	1.06E-02	promoter	super_enhancer	RP11-887P2.3
chr12	94035000	chr12	124835000	8.48E-03	promoter	super_enhancer	RP11-887P2.3
chr12	94755000	chr12	104275000	1.76E-02	promoter	super_enhancer	RN7SL330P
chr12	94855000	chr12	96435000	2.43E-02	promoter	super_enhancer	CEP83
chr12	94955000	chr12	95205000	3.54E-02	promoter	super_enhancer	MIR5700
chr12	94955000	chr12	104305000	2.75E-02	promoter	super_enhancer	MIR5700
chr12	94955000	chr12	121805000	1.52E-02	promoter	super_enhancer	MIR5700
chr12	94985000	chr12	95205000	1.36E-02	promoter	super_enhancer	LSM3P2
chr12	95045000	chr12	122425000	1.50E-02	promoter	super_enhancer	TMCC3
chr12	95045000	chr12	123105000	1.56E-02	promoter	super_enhancer	TMCC3
chr12	95045000	chr12	123125000	1.60E-02	promoter	super_enhancer	TMCC3

chr12	95205000	chr12	95945000	3.62E-02	super_enhancer	promoter	USP44
chr12	95205000	chr12	116695000	1.25E-02	super_enhancer	promoter	AC060226.1
chr12	95205000	chr12	132195000	1.02E-02	super_enhancer	promoter	SFSWAP
chr12	95625000	chr12	110435000	1.60E-02	super_enhancer	promoter	GIT2
chr12	95625000	chr12	111925000	1.48E-02	super_enhancer	promoter	AC002395.1
chr12	95625000	chr12	112855000	1.74E-02	super_enhancer	promoter	PTPN11
chr12	95625000	chr12	122345000	1.18E-02	super_enhancer	promoter	RNU7-170P
chr12	95625000	chr12	127805000	6.51E-03	super_enhancer	promoter	RP11-955H22.4
chr12	95625000	chr12	133065000	7.04E-03	super_enhancer	promoter	FBRSL1
chr12	95635000	chr12	100595000	3.82E-02	super_enhancer	promoter	RP11-175P13.3
chr12	95635000	chr12	106695000	1.70E-02	super_enhancer	promoter	TCP11L2
chr12	95635000	chr12	107305000	1.99E-02	super_enhancer	promoter	RPL30P12
chr12	95635000	chr12	110935000	1.60E-02	super_enhancer	promoter	SNORD50
chr12	95635000	chr12	112705000	1.54E-02	super_enhancer	promoter	RN7SKP71
chr12	95635000	chr12	120315000	1.60E-02	super_enhancer	promoter	CIT
chr12	95635000	chr12	120355000	1.36E-02	super_enhancer	promoter	AC004813.1
chr12	95635000	chr12	120935000	1.19E-02	super_enhancer	promoter	NRAV
chr12	95635000	chr12	121455000	1.29E-02	super_enhancer	promoter	C12orf43
chr12	95635000	chr12	122345000	1.21E-02	super_enhancer	promoter	RNU7-170P
chr12	95645000	chr12	104235000	2.71E-02	super_enhancer	promoter	NT5DC3
chr12	95655000	chr12	117425000	1.25E-02	super_enhancer	promoter	RP11-231I16.1
chr12	95655000	chr12	130555000	9.33E-03	super_enhancer	promoter	RP11-474D1.2
chr12	95945000	chr12	104255000	2.49E-02	promoter	super_enhancer	USP44
chr12	95945000	chr12	120265000	1.23E-02	promoter	super_enhancer	USP44

chr12	95945000	chr12	123095000	1.36E-02	promoter	super_enhancer	USP44
chr12	96185000	chr12	104295000	2.00E-02	promoter	super_enhancer	NTN4
chr12	96185000	chr12	111405000	2.07E-02	promoter	super_enhancer	NTN4
chr12	96185000	chr12	122425000	1.09E-02	promoter	super_enhancer	NTN4
chr12	96415000	chr12	102225000	3.29E-02	super_enhancer	promoter	GNPTAB
chr12	96415000	chr12	104235000	2.95E-02	super_enhancer	promoter	NT5DC3
chr12	96415000	chr12	112695000	1.62E-02	super_enhancer	promoter	RP3-521E19.2
chr12	96415000	chr12	120875000	1.32E-02	super_enhancer	promoter	COX6A1
chr12	96415000	chr12	121355000	1.37E-02	super_enhancer	promoter	RPL12P33
chr12	96415000	chr12	127175000	6.62E-03	super_enhancer	promoter	RP11-407A16.3
chr12	96425000	chr12	108955000	1.99E-02	super_enhancer	promoter	SART3
chr12	96425000	chr12	117345000	9.70E-03	super_enhancer	promoter	RP11-240G22.1
chr12	96425000	chr12	121455000	1.22E-02	super_enhancer	promoter	C12orf43
chr12	96425000	chr12	131135000	8.43E-03	super_enhancer	promoter	RP11-662M24.1
chr12	96435000	chr12	102055000	3.49E-02	super_enhancer	promoter	RP11-755O11.2
chr12	96435000	chr12	108955000	2.55E-02	super_enhancer	promoter	SART3
chr12	96435000	chr12	109125000	1.80E-02	super_enhancer	promoter	CORO1C
chr12	96435000	chr12	117345000	1.24E-02	super_enhancer	promoter	RP11-240G22.1
chr12	96435000	chr12	117685000	1.11E-02	super_enhancer	promoter	TCEB1P32
chr12	96435000	chr12	133815000	9.76E-03	super_enhancer	promoter	RNU6-717P
chr12	96445000	chr12	106645000	2.06E-02	super_enhancer	promoter	RP11-651L5.3
chr12	96455000	chr12	124865000	1.49E-02	promoter	super_enhancer	YPEL5P3
chr12	96795000	chr12	104305000	2.87E-02	promoter	super_enhancer	CDK17
chr12	97825000	chr12	116425000	9.76E-03	promoter	super_enhancer	RMST

chr12	97885000	chr12	113185000	1.93E-02	promoter	super_enhancer	MIR1251
chr12	97955000	chr12	124925000	9.96E-03	promoter	super_enhancer	RMST_9
chr12	98115000	chr12	120235000	1.06E-02	promoter	super_enhancer	RNU6-36P
chr12	99325000	chr12	104255000	2.68E-02	promoter	super_enhancer	RP11-473C19.1
chr12	100405000	chr12	113185000	1.71E-02	promoter	super_enhancer	RPS4XP1
chr12	100405000	chr12	124835000	9.17E-03	promoter	super_enhancer	RPS4XP1
chr12	100405000	chr12	131885000	6.78E-03	promoter	super_enhancer	RPS4XP1
chr12	101605000	chr12	113195000	2.17E-02	promoter	super_enhancer	SLC5A8
chr12	101605000	chr12	123115000	1.59E-02	promoter	super_enhancer	SLC5A8
chr12	101865000	chr12	104275000	7.46E-03	promoter	super_enhancer	RNA5SP367
chr12	102625000	chr12	104255000	1.38E-02	promoter	super_enhancer	RP11-18O15.1
chr12	102625000	chr12	117145000	1.84E-02	promoter	super_enhancer	RP11-18O15.1
chr12	102625000	chr12	120235000	1.82E-02	promoter	super_enhancer	RP11-18O15.1
chr12	102875000	chr12	131905000	9.57E-03	promoter	super_enhancer	IGF1
chr12	104225000	chr12	109425000	2.58E-02	super_enhancer	promoter	AC190387.2
chr12	104235000	chr12	131905000	8.23E-05	promoter	super_enhancer	NT5DC3
chr12	104255000	chr12	112125000	3.00E-02	super_enhancer	promoter	BRAP
chr12	104255000	chr12	117685000	1.33E-02	super_enhancer	promoter	TCEB1P32
chr12	104265000	chr12	106695000	3.52E-03	super_enhancer	promoter	TCP11L2
chr12	104265000	chr12	112205000	2.72E-02	super_enhancer	promoter	ALDH2
chr12	104275000	chr12	120355000	2.51E-02	super_enhancer	promoter	AC004813.1
chr12	104275000	chr12	120495000	2.36E-02	super_enhancer	promoter	RP1-267D11.1
chr12	104275000	chr12	122345000	2.21E-02	super_enhancer	promoter	RNU7-170P
chr12	104285000	chr12	105465000	7.07E-03	super_enhancer	promoter	RP11-61E11.1

chr12	104285000	chr12	109125000	3.84E-02	super_enhancer	promoter	CORO1C
chr12	104285000	chr12	131325000	1.03E-02	super_enhancer	promoter	STX2
chr12	104295000	chr12	123235000	1.59E-02	super_enhancer	promoter	RP11-324E6.4
chr12	104295000	chr12	126675000	1.19E-02	super_enhancer	promoter	RP4-765H13.1
chr12	104305000	chr12	110905000	3.77E-02	super_enhancer	promoter	FAM216A
chr12	104305000	chr12	120555000	2.28E-02	super_enhancer	promoter	RAB35
chr12	104305000	chr12	123495000	2.42E-02	super_enhancer	promoter	MIR4304
chr12	104325000	chr12	124395000	2.06E-02	promoter	super_enhancer	TTC41P
chr12	104425000	chr12	131915000	8.15E-03	promoter	super_enhancer	RP11-490H24.5
chr12	104685000	chr12	105465000	1.48E-02	super_enhancer	promoter	RP11-61E11.1
chr12	104685000	chr12	112365000	4.20E-02	super_enhancer	promoter	ADAM1B
chr12	104685000	chr12	112705000	4.10E-02	super_enhancer	promoter	RN7SKP71
chr12	104685000	chr12	113345000	4.28E-02	super_enhancer	promoter	OAS1
chr12	104685000	chr12	120315000	3.80E-02	super_enhancer	promoter	CIT
chr12	104685000	chr12	121545000	2.90E-02	super_enhancer	promoter	RP11-216P16.6
chr12	104945000	chr12	124915000	2.28E-02	promoter	super_enhancer	RP11-43D4.3
chr12	104985000	chr12	122945000	1.80E-02	promoter	super_enhancer	MIR3922
chr12	104985000	chr12	124915000	1.79E-02	promoter	super_enhancer	MIR3922
chr12	105345000	chr12	121785000	2.12E-02	promoter	super_enhancer	AC090051.1
chr12	105345000	chr12	122915000	1.91E-02	promoter	super_enhancer	AC090051.1
chr12	106535000	chr12	121805000	1.66E-02	promoter	super_enhancer	NUAK1
chr12	106645000	chr12	124835000	1.70E-02	promoter	super_enhancer	RP11-651L5.3
chr12	106695000	chr12	123115000	1.61E-02	promoter	super_enhancer	TCP11L2
chr12	107305000	chr12	120235000	2.59E-02	promoter	super_enhancer	RPL30P12

chr12	107305000	chr12	121795000	2.49E-02	promoter	super_enhancer	RPL30P12
chr12	107305000	chr12	122415000	1.99E-02	promoter	super_enhancer	RPL30P12
chr12	107665000	chr12	124825000	1.52E-02	promoter	super_enhancer	SETP7
chr12	107665000	chr12	131865000	1.30E-02	promoter	super_enhancer	SETP7
chr12	108695000	chr12	117145000	3.16E-02	promoter	super_enhancer	AC063957.1
chr12	108695000	chr12	131905000	1.56E-02	promoter	super_enhancer	AC063957.1
chr12	108955000	chr12	120265000	2.37E-02	promoter	super_enhancer	SART3
chr12	108955000	chr12	122405000	2.88E-02	promoter	super_enhancer	SART3
chr12	108955000	chr12	123125000	2.57E-02	promoter	super_enhancer	SART3
chr12	109125000	chr12	124855000	1.30E-02	promoter	super_enhancer	CORO1C
chr12	109425000	chr12	113185000	4.49E-02	promoter	super_enhancer	AC190387.2
chr12	109885000	chr12	110045000	1.81E-02	promoter	super_enhancer	RP11-256L11.3
chr12	109885000	chr12	116385000	3.82E-02	promoter	super_enhancer	RP11-256L11.3
chr12	109885000	chr12	117145000	3.62E-02	promoter	super_enhancer	RP11-256L11.3
chr12	109915000	chr12	121795000	2.63E-02	promoter	super_enhancer	KCTD10
chr12	109915000	chr12	124385000	2.48E-02	promoter	super_enhancer	KCTD10
chr12	110045000	chr12	120495000	2.90E-02	super_enhancer	promoter	RP1-267D11.1
chr12	110045000	chr12	124155000	2.43E-02	super_enhancer	promoter	TCTN2
chr12	110305000	chr12	122915000	2.68E-02	promoter	super_enhancer	RN7SL441P
chr12	110305000	chr12	123135000	2.50E-02	promoter	super_enhancer	RN7SL441P
chr12	110305000	chr12	124845000	2.85E-02	promoter	super_enhancer	RN7SL441P
chr12	110385000	chr12	116535000	2.80E-02	promoter	super_enhancer	RP11-837J7.4
chr12	110385000	chr12	122945000	2.22E-02	promoter	super_enhancer	RP11-837J7.4
chr12	110385000	chr12	124635000	1.53E-02	promoter	super_enhancer	RP11-837J7.4

chr12	110435000	chr12	123115000	2.22E-02	promoter	super_enhancer	GIT2
chr12	110495000	chr12	123085000	2.06E-02	promoter	super_enhancer	RP11-946P6.4
chr12	110495000	chr12	124865000	2.67E-02	promoter	super_enhancer	RP11-946P6.4
chr12	110905000	chr12	116545000	2.70E-02	promoter	super_enhancer	FAM216A
chr12	110905000	chr12	123085000	2.13E-02	promoter	super_enhancer	FAM216A
chr12	110935000	chr12	123125000	2.38E-02	promoter	super_enhancer	SNORD50
chr12	111065000	chr12	122415000	3.29E-04	promoter	super_enhancer	RN7SL387P
chr12	111065000	chr12	122935000	2.36E-02	promoter	super_enhancer	RN7SL387P
chr12	111065000	chr12	124865000	2.87E-02	promoter	super_enhancer	RN7SL387P
chr12	111285000	chr12	123115000	2.71E-02	promoter	super_enhancer	CCDC63
chr12	111285000	chr12	124925000	2.95E-02	promoter	super_enhancer	CCDC63
chr12	111405000	chr12	124455000	2.58E-02	super_enhancer	promoter	RP11-214K3.19
chr12	111405000	chr12	133615000	1.88E-02	super_enhancer	promoter	RP11-386I8.6
chr12	111925000	chr12	122425000	2.37E-02	promoter	super_enhancer	AC002395.1
chr12	111925000	chr12	123095000	2.44E-02	promoter	super_enhancer	AC002395.1
chr12	111925000	chr12	124925000	2.58E-02	promoter	super_enhancer	AC002395.1
chr12	112125000	chr12	122425000	4.37E-04	promoter	super_enhancer	BRAP
chr12	112125000	chr12	123135000	3.03E-02	promoter	super_enhancer	BRAP
chr12	112125000	chr12	124685000	2.46E-02	promoter	super_enhancer	BRAP
chr12	112205000	chr12	124855000	2.06E-02	promoter	super_enhancer	ALDH2
chr12	112365000	chr12	116405000	4.40E-02	promoter	super_enhancer	ADAM1B
chr12	112365000	chr12	116425000	4.31E-02	promoter	super_enhancer	ADAM1B
chr12	112365000	chr12	122425000	2.50E-02	promoter	super_enhancer	ADAM1B
chr12	112365000	chr12	123095000	2.57E-02	promoter	super_enhancer	ADAM1B

chr12	112365000	chr12	124685000	2.09E-02	promoter	super_enhancer	ADAM1B
chr12	112705000	chr12	120235000	3.60E-02	promoter	super_enhancer	RN7SKP71
chr12	112705000	chr12	124825000	2.24E-02	promoter	super_enhancer	RN7SKP71
chr12	112855000	chr12	123135000	3.11E-02	promoter	super_enhancer	PTPN11
chr12	113185000	chr12	124005000	2.86E-02	super_enhancer	promoter	RP11-972P1.11
chr12	113185000	chr12	125785000	3.11E-02	super_enhancer	promoter	RP11-604N13.1
chr12	113185000	chr12	132415000	2.05E-02	super_enhancer	promoter	RP11-417L19.4
chr12	113195000	chr12	117685000	3.46E-02	super_enhancer	promoter	TCEB1P32
chr12	113205000	chr12	117995000	6.47E-04	super_enhancer	promoter	AC079127.1
chr12	113205000	chr12	118575000	3.81E-02	super_enhancer	promoter	VSIG10
chr12	113205000	chr12	132905000	1.66E-02	super_enhancer	promoter	RP13-895J2.7
chr12	113375000	chr12	131895000	2.42E-02	promoter	super_enhancer	OAS3
chr12	113575000	chr12	131865000	2.06E-02	promoter	super_enhancer	RASAL1
chr12	113795000	chr12	123125000	3.09E-02	promoter	super_enhancer	PLBD2
chr12	113865000	chr12	116385000	8.21E-03	promoter	super_enhancer	SDS
chr12	113865000	chr12	123125000	3.30E-02	promoter	super_enhancer	SDS
chr12	114405000	chr12	116385000	1.52E-02	promoter	super_enhancer	RBM19
chr12	114405000	chr12	116415000	8.34E-03	promoter	super_enhancer	RBM19
chr12	114405000	chr12	116425000	8.32E-03	promoter	super_enhancer	RBM19
chr12	114405000	chr12	131865000	2.15E-02	promoter	super_enhancer	RBM19
chr12	114515000	chr12	124395000	3.18E-02	promoter	super_enhancer	RP11-100F15.1
chr12	114535000	chr12	124915000	2.87E-02	promoter	super_enhancer	HAUS8P1
chr12	114535000	chr12	131885000	1.12E-04	promoter	super_enhancer	HAUS8P1
chr12	114845000	chr12	123105000	2.59E-02	promoter	super_enhancer	TBX5-AS1

chr12	115025000	chr12	116385000	2.91E-02	promoter	super_enhancer	OSTF1P1
chr12	115205000	chr12	120265000	4.28E-02	promoter	super_enhancer	RP4-601P9.1
chr12	115205000	chr12	124835000	2.56E-02	promoter	super_enhancer	RP4-601P9.1
chr12	115515000	chr12	124925000	3.08E-02	promoter	super_enhancer	RP11-25E2.1
chr12	116385000	chr12	117135000	4.02E-02	super_enhancer	promoter	RP11-497G19.2
chr12	116385000	chr12	131475000	1.92E-02	super_enhancer	promoter	RP11-76C10.6
chr12	116395000	chr12	117135000	6.21E-05	super_enhancer	promoter	RP11-497G19.2
chr12	116395000	chr12	117345000	4.69E-02	super_enhancer	promoter	RP11-240G22.1
chr12	116395000	chr12	121545000	4.24E-02	super_enhancer	promoter	RP11-216P16.6
chr12	116395000	chr12	122235000	4.49E-02	super_enhancer	promoter	RP11-347I19.8
chr12	116395000	chr12	125045000	2.57E-02	super_enhancer	promoter	RP11-83B20.6
chr12	116405000	chr12	123465000	2.67E-02	super_enhancer	promoter	ARL6IP4
chr12	116405000	chr12	125635000	2.32E-02	super_enhancer	promoter	RP11-158L12.5
chr12	116415000	chr12	117175000	9.23E-04	super_enhancer	promoter	RNFT2
chr12	116415000	chr12	120875000	3.96E-02	super_enhancer	promoter	COX6A1
chr12	116415000	chr12	124155000	2.46E-02	super_enhancer	promoter	TCTN2
chr12	116415000	chr12	125045000	2.23E-02	super_enhancer	promoter	RP11-83B20.6
chr12	116425000	chr12	116985000	3.21E-02	super_enhancer	promoter	MAP1LC3B2
chr12	116425000	chr12	117345000	3.97E-03	super_enhancer	promoter	RP11-240G22.1
chr12	116425000	chr12	121125000	4.25E-02	super_enhancer	promoter	MLEC
chr12	116425000	chr12	128435000	1.64E-02	super_enhancer	promoter	LINC00508
chr12	116435000	chr12	116715000	9.43E-04	super_enhancer	promoter	MED13L
chr12	116435000	chr12	121455000	4.35E-02	super_enhancer	promoter	C12orf43
chr12	116435000	chr12	122345000	3.56E-02	super_enhancer	promoter	RNU7-170P

chr12	116525000	chr12	127545000	1.76E-02	super_enhancer	promoter	RP11-575F12.1
chr12	116525000	chr12	132415000	2.18E-02	super_enhancer	promoter	RP11-417L19.4
chr12	116525000	chr12	133655000	1.72E-02	super_enhancer	promoter	RP11-386I8.4
chr12	116535000	chr12	117425000	6.87E-03	super_enhancer	promoter	RP11-231I16.1
chr12	116535000	chr12	121535000	3.85E-02	super_enhancer	promoter	RP11-216P16.5
chr12	116535000	chr12	123215000	3.15E-02	super_enhancer	promoter	HCAR1
chr12	116535000	chr12	125045000	2.59E-02	super_enhancer	promoter	RP11-83B20.6
chr12	116535000	chr12	133445000	1.84E-02	super_enhancer	promoter	RP11-46H11.11
chr12	116545000	chr12	117135000	2.48E-02	super_enhancer	promoter	RP11-497G19.2
chr12	116555000	chr12	123465000	2.41E-02	super_enhancer	promoter	ARL6IP4
chr12	116555000	chr12	125385000	2.16E-02	super_enhancer	promoter	RNU6-927P
chr12	116555000	chr12	125785000	2.31E-02	super_enhancer	promoter	RP11-604N13.1
chr12	116695000	chr12	120265000	1.24E-03	promoter	super_enhancer	AC060226.1
chr12	116695000	chr12	124395000	2.89E-02	promoter	super_enhancer	AC060226.1
chr12	116715000	chr12	131905000	1.68E-02	promoter	super_enhancer	MED13L
chr12	116985000	chr12	124915000	3.61E-02	promoter	super_enhancer	MAP1LC3B2
chr12	117145000	chr12	132105000	2.26E-02	super_enhancer	promoter	RP11-495K9.10
chr12	117175000	chr12	122405000	4.49E-02	promoter	super_enhancer	RNFT2
chr12	117345000	chr12	122415000	3.63E-02	promoter	super_enhancer	RP11-240G22.1
chr12	117345000	chr12	123095000	3.40E-02	promoter	super_enhancer	RP11-240G22.1
chr12	117425000	chr12	131895000	2.11E-02	promoter	super_enhancer	RP11-231I16.1
chr12	117685000	chr12	124635000	2.00E-02	promoter	super_enhancer	TCEB1P32
chr12	117685000	chr12	131905000	1.49E-02	promoter	super_enhancer	TCEB1P32
chr12	118475000	chr12	124925000	4.54E-02	promoter	super_enhancer	RP11-33N14.5

chr12	118575000	chr12	124395000	4.78E-02	promoter	super_enhancer	VSIG10
chr12	118575000	chr12	124635000	3.24E-02	promoter	super_enhancer	VSIG10
chr12	118605000	chr12	131885000	1.53E-02	promoter	super_enhancer	AC026366.1
chr12	118805000	chr12	120235000	4.15E-03	promoter	super_enhancer	AC026367.1
chr12	118815000	chr12	131865000	2.10E-02	promoter	super_enhancer	RP11-131L12.2
chr12	118815000	chr12	131895000	2.39E-02	promoter	super_enhancer	RP11-131L12.2
chr12	119555000	chr12	124635000	4.28E-02	promoter	super_enhancer	RP11-364C11.2
chr12	120235000	chr12	131355000	2.98E-02	super_enhancer	promoter	RNU6-1077P
chr12	120235000	chr12	132685000	2.66E-02	super_enhancer	promoter	RP13-977J11.3
chr12	120265000	chr12	126625000	2.44E-02	super_enhancer	promoter	RP3-446N13.4
chr12	120555000	chr12	131855000	2.50E-02	promoter	super_enhancer	RAB35
chr12	120555000	chr12	131865000	3.07E-02	promoter	super_enhancer	RAB35
chr12	120685000	chr12	131905000	2.75E-02	promoter	super_enhancer	RPS20P31
chr12	120685000	chr12	131915000	2.37E-02	promoter	super_enhancer	RPS20P31
chr12	120705000	chr12	123105000	1.35E-02	promoter	super_enhancer	PXN
chr12	120705000	chr12	131895000	3.80E-02	promoter	super_enhancer	PXN
chr12	120935000	chr12	124395000	4.23E-03	promoter	super_enhancer	NRAV
chr12	120935000	chr12	131885000	2.23E-02	promoter	super_enhancer	NRAV
chr12	121125000	chr12	121805000	4.51E-03	promoter	super_enhancer	MLEC
chr12	121805000	chr12	126625000	4.42E-02	super_enhancer	promoter	RP3-446N13.4
chr12	121805000	chr12	133195000	3.13E-02	super_enhancer	promoter	P2RX2
chr12	121805000	chr12	133815000	2.96E-02	super_enhancer	promoter	RNU6-717P
chr12	122345000	chr12	131895000	3.72E-02	promoter	super_enhancer	RNU7-170P
chr12	122405000	chr12	131295000	3.28E-02	super_enhancer	promoter	RP11-989F5.1

chr12	122405000	chr12	133655000	2.71E-02	super_enhancer	promoter	RP11-386I8.4
chr12	122415000	chr12	123635000	9.43E-03	super_enhancer	promoter	PITPNM2
chr12	122415000	chr12	126615000	4.63E-02	super_enhancer	promoter	RP3-446N13.3
chr12	122415000	chr12	126625000	4.32E-02	super_enhancer	promoter	RP3-446N13.4
chr12	122415000	chr12	127825000	3.63E-02	super_enhancer	promoter	RP11-955H22.3
chr12	122425000	chr12	132685000	2.53E-02	super_enhancer	promoter	RP13-977J11.3
chr12	122925000	chr12	127545000	4.20E-02	super_enhancer	promoter	RP11-575F12.1
chr12	122935000	chr12	130555000	2.72E-02	super_enhancer	promoter	RP11-474D1.2
chr12	122935000	chr12	132415000	2.85E-02	super_enhancer	promoter	RP11-417L19.4
chr12	122945000	chr12	127825000	4.54E-02	super_enhancer	promoter	RP11-955H22.3
chr12	122945000	chr12	133615000	2.82E-02	super_enhancer	promoter	RP11-386I8.6
chr12	123065000	chr12	132675000	3.46E-02	super_enhancer	promoter	RP13-977J11.2
chr12	123075000	chr12	133615000	2.54E-02	super_enhancer	promoter	RP11-386I8.6
chr12	123095000	chr12	133615000	2.71E-02	super_enhancer	promoter	RP11-386I8.6
chr12	123105000	chr12	123235000	1.67E-02	super_enhancer	promoter	RP11-324E6.4
chr12	123105000	chr12	123465000	1.17E-03	super_enhancer	promoter	ARL6IP4
chr12	123105000	chr12	124455000	4.21E-02	super_enhancer	promoter	RP11-214K3.19
chr12	123105000	chr12	133405000	3.23E-02	super_enhancer	promoter	GOLGA3
chr12	123115000	chr12	123215000	7.49E-03	super_enhancer	promoter	HCAR1
chr12	123115000	chr12	123235000	4.73E-02	super_enhancer	promoter	RP11-324E6.4
chr12	123115000	chr12	132415000	3.16E-02	super_enhancer	promoter	RP11-417L19.4
chr12	123125000	chr12	123235000	3.66E-04	super_enhancer	promoter	RP11-324E6.4
chr12	123125000	chr12	123635000	4.13E-02	super_enhancer	promoter	PITPNM2
chr12	123125000	chr12	132685000	2.87E-02	super_enhancer	promoter	RP13-977J11.3

chr12	123135000	chr12	123215000	7.91E-04	super_enhancer	promoter	HCAR1
chr12	123135000	chr12	133795000	3.14E-02	super_enhancer	promoter	AC226150.3
chr12	123145000	chr12	133655000	2.39E-02	super_enhancer	promoter	RP11-386I8.4
chr12	123565000	chr12	124855000	2.61E-02	promoter	super_enhancer	RP11-197N18.7
chr12	123565000	chr12	131905000	2.53E-02	promoter	super_enhancer	RP11-197N18.7
chr12	123635000	chr12	131895000	4.22E-02	promoter	super_enhancer	PITPNM2
chr12	124155000	chr12	131905000	3.35E-02	promoter	super_enhancer	TCTN2
chr12	124385000	chr12	130555000	4.54E-02	super_enhancer	promoter	RP11-474D1.2
chr12	124385000	chr12	131325000	3.60E-02	super_enhancer	promoter	STX2
chr12	124385000	chr12	132675000	4.36E-02	super_enhancer	promoter	RP13-977J11.2
chr12	124395000	chr12	132565000	3.92E-02	super_enhancer	promoter	RP13-820C6.2
chr12	124455000	chr12	131895000	3.77E-02	promoter	super_enhancer	RP11-214K3.19
chr12	124635000	chr12	130325000	2.86E-02	super_enhancer	promoter	RP11-572C21.2
chr12	124635000	chr12	131325000	2.41E-02	super_enhancer	promoter	STX2
chr12	124685000	chr12	125015000	1.56E-02	super_enhancer	promoter	RP11-83B20.2
chr12	124685000	chr12	125635000	1.90E-02	super_enhancer	promoter	RP11-158L12.5
chr12	124685000	chr12	129155000	3.30E-02	super_enhancer	promoter	AC107020.1
chr12	124685000	chr12	133815000	2.69E-02	super_enhancer	promoter	RNU6-717P
chr12	124825000	chr12	133655000	2.58E-02	super_enhancer	promoter	RP11-386I8.4
chr12	124835000	chr12	133065000	2.52E-02	super_enhancer	promoter	FBRSL1
chr12	124835000	chr12	133445000	3.05E-02	super_enhancer	promoter	RP11-46H11.11
chr12	124865000	chr12	125015000	4.47E-02	super_enhancer	promoter	RP11-83B20.2
chr12	124865000	chr12	131355000	4.73E-02	super_enhancer	promoter	RNU6-1077P
chr12	124915000	chr12	132905000	4.15E-02	super_enhancer	promoter	RP13-895J2.7

chr12	124915000	chr12	133265000	2.85E-02	super_enhancer	promoter	POLE
chr12	124925000	chr12	133655000	3.25E-02	super_enhancer	promoter	RP11-386I8.4
chr12	125475000	chr12	131855000	3.86E-02	promoter	super_enhancer	DHX37
chr12	125635000	chr12	131875000	4.08E-02	promoter	super_enhancer	RP11-158L12.5
chr12	126615000	chr12	131895000	4.50E-02	promoter	super_enhancer	RP3-446N13.3
chr12	126625000	chr12	131915000	2.89E-02	promoter	super_enhancer	RP3-446N13.4
chr12	131355000	chr12	131895000	4.09E-02	promoter	super_enhancer	RNU6-1077P
chr12	131875000	chr12	132195000	4.71E-03	super_enhancer	promoter	SFSWAP
chr13	19745000	chr13	75775000	4.85E-03	promoter	super_enhancer	SMPD4P2
chr13	20505000	chr13	97265000	2.88E-03	promoter	super_enhancer	AL591925.1
chr13	22245000	chr13	97265000	1.61E-03	promoter	super_enhancer	FGF9
chr13	22365000	chr13	48775000	5.13E-03	promoter	super_enhancer	RN7SL766P
chr13	23425000	chr13	44265000	1.11E-02	promoter	super_enhancer	RP11-363G2.4
chr13	23425000	chr13	98485000	2.05E-03	promoter	super_enhancer	RP11-363G2.4
chr13	23425000	chr13	114035000	3.18E-03	promoter	super_enhancer	RP11-363G2.4
chr13	23755000	chr13	113435000	2.28E-03	promoter	super_enhancer	SGCG
chr13	24905000	chr13	44305000	8.71E-03	promoter	super_enhancer	LINC00566
chr13	24905000	chr13	73085000	2.76E-03	promoter	super_enhancer	LINC00566
chr13	25255000	chr13	48775000	1.17E-02	promoter	super_enhancer	ATP12A
chr13	25255000	chr13	98495000	3.39E-03	promoter	super_enhancer	ATP12A
chr13	25755000	chr13	44265000	1.54E-02	promoter	super_enhancer	LINC00463
chr13	25755000	chr13	44285000	1.04E-02	promoter	super_enhancer	LINC00463
chr13	25785000	chr13	110955000	3.40E-03	promoter	super_enhancer	LINC01076
chr13	25875000	chr13	75705000	3.60E-03	promoter	super_enhancer	NUPL1

chr13	27825000	chr13	44275000	1.07E-02	promoter	super_enhancer	RP11-428O18.6
chr13	28025000	chr13	45355000	1.24E-02	promoter	super_enhancer	MTIF3
chr13	28025000	chr13	45365000	1.40E-02	promoter	super_enhancer	MTIF3
chr13	28255000	chr13	110015000	2.98E-03	promoter	super_enhancer	RP11-87C7.3
chr13	28255000	chr13	114035000	3.31E-03	promoter	super_enhancer	RP11-87C7.3
chr13	29825000	chr13	48775000	9.99E-03	promoter	super_enhancer	MTUS2-AS2
chr13	29825000	chr13	75795000	4.08E-03	promoter	super_enhancer	MTUS2-AS2
chr13	29825000	chr13	109275000	2.38E-03	promoter	super_enhancer	MTUS2-AS2
chr13	29915000	chr13	110955000	3.56E-03	promoter	super_enhancer	RP11-351N4.4
chr13	30735000	chr13	44295000	1.59E-02	promoter	super_enhancer	LINC00384
chr13	30855000	chr13	44305000	1.45E-02	promoter	super_enhancer	RNU6-64P
chr13	32605000	chr13	112725000	2.88E-03	promoter	super_enhancer	FRY
chr13	33085000	chr13	44255000	1.55E-02	promoter	super_enhancer	N4BP2L2-IT2
chr13	33085000	chr13	75695000	4.54E-03	promoter	super_enhancer	N4BP2L2-IT2
chr13	33615000	chr13	75795000	4.36E-03	promoter	super_enhancer	AL161898.1
chr13	34405000	chr13	44305000	1.75E-02	promoter	super_enhancer	RNU5A-4P
chr13	35515000	chr13	44305000	1.64E-02	promoter	super_enhancer	NBEA
chr13	35825000	chr13	48795000	1.42E-02	promoter	super_enhancer	PHBP13
chr13	36945000	chr13	44255000	2.34E-02	promoter	super_enhancer	SPG20
chr13	37005000	chr13	44255000	2.16E-02	promoter	super_enhancer	CCNA1
chr13	37425000	chr13	45355000	2.33E-02	promoter	super_enhancer	SMAD9-IT1
chr13	37515000	chr13	44255000	2.08E-02	promoter	super_enhancer	EIF4A1P5
chr13	37595000	chr13	44305000	1.99E-02	promoter	super_enhancer	AL138706.2
chr13	37635000	chr13	48775000	1.94E-02	promoter	super_enhancer	SUPT20H

chr13	37635000	chr13	75725000	6.43E-03	promoter	super_enhancer	SUPT20H
chr13	38445000	chr13	44295000	1.68E-02	promoter	super_enhancer	TRPC4
chr13	39745000	chr13	44325000	3.29E-02	promoter	super_enhancer	NXT1P1
chr13	39745000	chr13	73335000	7.18E-03	promoter	super_enhancer	NXT1P1
chr13	40755000	chr13	44245000	4.57E-02	promoter	super_enhancer	LINC00332
chr13	40795000	chr13	45365000	4.88E-02	promoter	super_enhancer	LINC00548
chr13	41345000	chr13	45355000	4.84E-02	promoter	super_enhancer	MRPS31
chr13	41635000	chr13	48775000	2.30E-02	promoter	super_enhancer	WBP4
chr13	41635000	chr13	97235000	3.66E-03	promoter	super_enhancer	WBP4
chr13	41695000	chr13	113445000	2.58E-03	promoter	super_enhancer	RN7SL597P
chr13	42015000	chr13	44275000	3.24E-03	promoter	super_enhancer	OR7E155P
chr13	42645000	chr13	98495000	3.49E-03	promoter	super_enhancer	MAPK6PS3
chr13	42645000	chr13	113425000	2.71E-03	promoter	super_enhancer	MAPK6PS3
chr13	42845000	chr13	44235000	1.96E-02	promoter	super_enhancer	AKAP11
chr13	42945000	chr13	44265000	2.28E-02	promoter	super_enhancer	FABP3P2
chr13	42945000	chr13	44325000	1.49E-02	promoter	super_enhancer	FABP3P2
chr13	43755000	chr13	44275000	2.27E-03	promoter	super_enhancer	RP11-460G11.2
chr13	43755000	chr13	44305000	2.74E-02	promoter	super_enhancer	RP11-460G11.2
chr13	43755000	chr13	75725000	6.35E-03	promoter	super_enhancer	RP11-460G11.2
chr13	44235000	chr13	73035000	7.38E-03	super_enhancer	promoter	RPL18AP17
chr13	44235000	chr13	76445000	6.35E-03	super_enhancer	promoter	LMO7DN
chr13	44235000	chr13	81825000	4.49E-03	super_enhancer	promoter	RP11-452B18.2
chr13	44235000	chr13	91145000	3.77E-03	super_enhancer	promoter	LINC01049
chr13	44245000	chr13	45875000	1.54E-02	super_enhancer	promoter	AL138963.1

chr13	44245000	chr13	51795000	2.57E-02	super_enhancer	promoter	FAM124A
chr13	44245000	chr13	95955000	4.65E-03	super_enhancer	promoter	ABCC4
chr13	44255000	chr13	106115000	2.81E-03	super_enhancer	promoter	DAOA-AS1_2
chr13	44255000	chr13	107315000	3.07E-03	super_enhancer	promoter	ATP5G1P5
chr13	44265000	chr13	47035000	3.80E-02	super_enhancer	promoter	OR7E101P
chr13	44265000	chr13	52495000	2.55E-02	super_enhancer	promoter	RP11-327P2.6
chr13	44305000	chr13	47035000	3.88E-02	super_enhancer	promoter	OR7E101P
chr13	44305000	chr13	51485000	2.13E-02	super_enhancer	promoter	RNASEH2B-AS1
chr13	44305000	chr13	73425000	6.49E-03	super_enhancer	promoter	AL391384.1
chr13	44305000	chr13	73765000	8.06E-03	super_enhancer	promoter	RNU4-10P
chr13	44315000	chr13	47035000	3.61E-02	super_enhancer	promoter	OR7E101P
chr13	44315000	chr13	98885000	3.31E-03	super_enhancer	promoter	RP11-573N10.1
chr13	44315000	chr13	99825000	4.47E-03	super_enhancer	promoter	RPS6P23
chr13	44315000	chr13	114145000	4.44E-03	super_enhancer	promoter	DCUN1D2
chr13	44325000	chr13	96345000	3.20E-03	super_enhancer	promoter	MTND5P2
chr13	44685000	chr13	48785000	3.38E-02	promoter	super_enhancer	SMIM2-AS1
chr13	44735000	chr13	75695000	7.67E-03	promoter	super_enhancer	SMIM2
chr13	44735000	chr13	112725000	3.89E-03	promoter	super_enhancer	SMIM2
chr13	44945000	chr13	110955000	5.49E-03	promoter	super_enhancer	RP11-269C23.5
chr13	45355000	chr13	91785000	3.44E-03	super_enhancer	promoter	LINC00379
chr13	45365000	chr13	58205000	3.34E-04	super_enhancer	promoter	PCDH17
chr13	45365000	chr13	113465000	4.17E-03	super_enhancer	promoter	RP11-73M11.3
chr13	45385000	chr13	112735000	3.12E-03	promoter	super_enhancer	LINC00330
chr13	45575000	chr13	75705000	9.15E-03	promoter	super_enhancer	RN7SL49P

chr13	45575000	chr13	114005000	3.77E-03	promoter	super_enhancer	RN7SL49P
chr13	45635000	chr13	98485000	4.25E-03	promoter	super_enhancer	RN7SKP3
chr13	45965000	chr13	75705000	8.63E-03	promoter	super_enhancer	RCN1P2
chr13	45965000	chr13	75725000	7.76E-03	promoter	super_enhancer	RCN1P2
chr13	46275000	chr13	73095000	8.25E-03	promoter	super_enhancer	LINC01055
chr13	46275000	chr13	75695000	7.33E-03	promoter	super_enhancer	LINC01055
chr13	46275000	chr13	110945000	3.80E-03	promoter	super_enhancer	LINC01055
chr13	46745000	chr13	75705000	9.24E-03	promoter	super_enhancer	RN7SL288P
chr13	46745000	chr13	75785000	8.65E-03	promoter	super_enhancer	RN7SL288P
chr13	46745000	chr13	110015000	4.35E-03	promoter	super_enhancer	RN7SL288P
chr13	46745000	chr13	110955000	5.11E-03	promoter	super_enhancer	RN7SL288P
chr13	46985000	chr13	109245000	1.49E-03	promoter	super_enhancer	RNU6-68P
chr13	47035000	chr13	73095000	4.06E-03	promoter	super_enhancer	OR7E101P
chr13	47425000	chr13	48795000	1.50E-02	promoter	super_enhancer	HTR2A-AS1
chr13	48795000	chr13	98085000	3.55E-03	super_enhancer	promoter	RAP2A
chr13	48795000	chr13	111775000	3.85E-03	super_enhancer	promoter	ARHGEF7-IT1
chr13	48895000	chr13	75795000	7.52E-03	promoter	super_enhancer	PPP1R26P1
chr13	49345000	chr13	110965000	3.65E-03	promoter	super_enhancer	PSME2P2
chr13	49345000	chr13	113445000	2.78E-03	promoter	super_enhancer	PSME2P2
chr13	50655000	chr13	73095000	7.86E-03	promoter	super_enhancer	DLEU2_6
chr13	50745000	chr13	75695000	7.83E-03	promoter	super_enhancer	ST13P4
chr13	51485000	chr13	75785000	8.19E-03	promoter	super_enhancer	RNASEH2B-AS1
chr13	51485000	chr13	114065000	4.39E-03	promoter	super_enhancer	RNASEH2B-AS1
chr13	51655000	chr13	112725000	3.46E-03	promoter	super_enhancer	GUCY1B2

chr13	51795000	chr13	109275000	5.64E-03	promoter	super_enhancer	FAM124A
chr13	52735000	chr13	75695000	8.02E-03	promoter	super_enhancer	NEK3
chr13	52735000	chr13	114015000	3.46E-03	promoter	super_enhancer	NEK3
chr13	53215000	chr13	97225000	4.47E-03	promoter	super_enhancer	HNRNPA1L2
chr13	53315000	chr13	97275000	6.46E-03	promoter	super_enhancer	LECT1
chr13	53395000	chr13	75725000	9.75E-03	promoter	super_enhancer	PPIAP26
chr13	53985000	chr13	114015000	3.39E-03	promoter	super_enhancer	RP11-384G23.2
chr13	58565000	chr13	75705000	9.68E-03	promoter	super_enhancer	RNA5SP30
chr13	62895000	chr13	114015000	3.00E-03	promoter	super_enhancer	LINC01074
chr13	67565000	chr13	73095000	1.78E-02	promoter	super_enhancer	PCDH9-AS4
chr13	69795000	chr13	97265000	4.64E-03	promoter	super_enhancer	LINC00383
chr13	71475000	chr13	75775000	4.67E-04	promoter	super_enhancer	AL391123.1
chr13	73335000	chr13	88325000	1.53E-02	super_enhancer	promoter	MIR4500HG
chr13	73335000	chr13	110775000	7.00E-03	super_enhancer	promoter	RP11-472K17.3
chr13	73355000	chr13	75785000	3.14E-03	promoter	super_enhancer	PIBF1
chr13	73425000	chr13	112715000	5.87E-03	promoter	super_enhancer	AL391384.1
chr13	73675000	chr13	114035000	1.08E-02	promoter	super_enhancer	FABP5P1
chr13	73765000	chr13	98505000	9.87E-03	promoter	super_enhancer	RNU4-10P
chr13	74805000	chr13	97235000	7.46E-03	promoter	super_enhancer	LINC00402
chr13	75695000	chr13	108185000	4.75E-03	super_enhancer	promoter	MIR1267
chr13	75695000	chr13	112705000	5.61E-03	super_enhancer	promoter	SNORD44
chr13	75705000	chr13	88325000	1.75E-02	super_enhancer	promoter	MIR4500HG
chr13	75705000	chr13	107305000	7.16E-03	super_enhancer	promoter	LINC00443
chr13	75705000	chr13	111415000	7.87E-03	super_enhancer	promoter	RPL21P107

chr13	75715000	chr13	98795000	1.14E-02	super_enhancer	promoter	FARP1
chr13	75715000	chr13	108185000	6.26E-03	super_enhancer	promoter	MIR1267
chr13	75715000	chr13	109755000	7.64E-03	super_enhancer	promoter	MYO16-AS2
chr13	75715000	chr13	114875000	6.13E-03	super_enhancer	promoter	RASA3-IT1
chr13	75725000	chr13	103555000	6.77E-03	super_enhancer	promoter	RP11-255P5.3
chr13	75725000	chr13	114455000	5.36E-03	super_enhancer	promoter	LINC00552
chr13	75795000	chr13	110685000	5.27E-03	super_enhancer	promoter	RP11-403A3.2
chr13	75795000	chr13	113465000	6.56E-03	super_enhancer	promoter	RP11-73M11.3
chr13	75815000	chr13	109255000	5.37E-03	promoter	super_enhancer	CTAGE11P
chr13	76125000	chr13	110965000	1.83E-05	promoter	super_enhancer	COMMD6
chr13	76195000	chr13	109265000	6.20E-03	promoter	super_enhancer	LMO7
chr13	78495000	chr13	112725000	8.10E-03	promoter	super_enhancer	EDNRB
chr13	79335000	chr13	97235000	8.99E-03	promoter	super_enhancer	RPL21P111
chr13	79335000	chr13	114005000	5.93E-03	promoter	super_enhancer	RPL21P111
chr13	79965000	chr13	97275000	9.66E-03	promoter	super_enhancer	RNA5SP33
chr13	79965000	chr13	110005000	5.81E-03	promoter	super_enhancer	RNA5SP33
chr13	80585000	chr13	97235000	1.33E-02	promoter	super_enhancer	LINC01080
chr13	80915000	chr13	98505000	1.64E-02	promoter	super_enhancer	SPRY2
chr13	81195000	chr13	110965000	7.20E-03	promoter	super_enhancer	HNRNPA1P31
chr13	81825000	chr13	97265000	9.58E-03	promoter	super_enhancer	RP11-452B18.2
chr13	85135000	chr13	112735000	5.29E-03	promoter	super_enhancer	LINC00333
chr13	86635000	chr13	110955000	1.02E-02	promoter	super_enhancer	RP11-30L8.1
chr13	90015000	chr13	99985000	1.63E-02	promoter	super_enhancer	SP3P
chr13	91145000	chr13	98495000	1.91E-02	promoter	super_enhancer	LINC01049

chr13	91145000	chr13	99975000	1.32E-02	promoter	super_enhancer	LINC01049
chr13	91145000	chr13	110015000	1.14E-02	promoter	super_enhancer	LINC01049
chr13	91785000	chr13	97225000	1.77E-02	promoter	super_enhancer	LINC00379
chr13	91925000	chr13	113445000	6.89E-03	promoter	super_enhancer	PPIAP23
chr13	93375000	chr13	97225000	2.75E-02	promoter	super_enhancer	GPC5-AS1
chr13	95955000	chr13	110955000	2.34E-02	promoter	super_enhancer	ABCC4
chr13	95955000	chr13	113425000	1.67E-02	promoter	super_enhancer	ABCC4
chr13	96045000	chr13	110965000	1.46E-02	promoter	super_enhancer	MEMO1P5
chr13	96085000	chr13	97265000	1.75E-02	promoter	super_enhancer	CLDN10
chr13	97235000	chr13	113345000	1.29E-02	super_enhancer	promoter	RP11-88E10.5
chr13	97245000	chr13	98015000	4.78E-02	super_enhancer	promoter	RNA5SP37
chr13	97245000	chr13	111635000	1.55E-02	super_enhancer	promoter	LINC00431
chr13	97255000	chr13	111835000	1.22E-02	super_enhancer	promoter	RP11-17E4.1
chr13	97265000	chr13	109755000	1.40E-02	super_enhancer	promoter	MYO16-AS2
chr13	97265000	chr13	109855000	1.29E-02	super_enhancer	promoter	MYO16-AS1
chr13	98015000	chr13	110955000	1.89E-02	promoter	super_enhancer	RNA5SP37
chr13	98485000	chr13	111775000	1.46E-02	super_enhancer	promoter	ARHGEF7-IT1
chr13	98495000	chr13	99405000	1.80E-02	super_enhancer	promoter	SLC15A1
chr13	98505000	chr13	99825000	3.48E-02	super_enhancer	promoter	RPS6P23
chr13	98505000	chr13	102105000	4.90E-02	super_enhancer	promoter	ITGBL1
chr13	98605000	chr13	109245000	1.43E-02	promoter	super_enhancer	IPO5
chr13	98605000	chr13	113445000	1.51E-02	promoter	super_enhancer	IPO5
chr13	98795000	chr13	99985000	2.78E-03	promoter	super_enhancer	FARP1
chr13	98985000	chr13	109245000	1.23E-02	promoter	super_enhancer	RP11-261P24.2

chr13	98985000	chr13	110015000	2.10E-02	promoter	super_enhancer	RP11-261P24.2
chr13	98985000	chr13	114035000	1.93E-02	promoter	super_enhancer	RP11-261P24.2
chr13	99205000	chr13	112735000	1.61E-02	promoter	super_enhancer	RP11-295B17.6
chr13	99205000	chr13	113435000	1.98E-02	promoter	super_enhancer	RP11-295B17.6
chr13	99405000	chr13	112715000	2.70E-02	promoter	super_enhancer	SLC15A1
chr13	99405000	chr13	113395000	2.22E-02	promoter	super_enhancer	SLC15A1
chr13	99825000	chr13	114005000	1.85E-02	promoter	super_enhancer	RPS6P23
chr13	100625000	chr13	109275000	2.70E-02	promoter	super_enhancer	ZIC5
chr13	102105000	chr13	109255000	1.84E-02	promoter	super_enhancer	ITGBL1
chr13	102105000	chr13	112715000	2.06E-02	promoter	super_enhancer	ITGBL1
chr13	103395000	chr13	110005000	2.41E-02	promoter	super_enhancer	LINC00283
chr13	103555000	chr13	110955000	2.69E-02	promoter	super_enhancer	RP11-255P5.3
chr13	103555000	chr13	114035000	1.97E-02	promoter	super_enhancer	RP11-255P5.3
chr13	106115000	chr13	114015000	1.91E-02	promoter	super_enhancer	DAOA-AS1_2
chr13	109245000	chr13	110775000	8.26E-03	super_enhancer	promoter	RP11-472K17.3
chr13	109855000	chr13	114035000	4.69E-02	promoter	super_enhancer	MYO16-AS1
chr13	110005000	chr13	111635000	1.63E-02	super_enhancer	promoter	LINC00431
chr13	110015000	chr13	110685000	1.42E-02	super_enhancer	promoter	RP11-403A3.2
chr13	111605000	chr13	113435000	1.31E-02	promoter	super_enhancer	RP11-365P13.6
chr13	111635000	chr13	114025000	6.35E-03	promoter	super_enhancer	LINC00431
chr13	112705000	chr13	112715000	1.94E-02	promoter	super_enhancer	SNORD44
chr13	112705000	chr13	113425000	2.00E-02	promoter	super_enhancer	SNORD44
chr13	113345000	chr13	114025000	2.84E-03	promoter	super_enhancer	RP11-88E10.5
chr13	113405000	chr13	113465000	2.26E-02	super_enhancer	promoter	RP11-73M11.3

chr13	113405000	chr13	114455000	4.62E-02	super_enhancer	promoter	LINC00552
chr14	20585000	chr14	35355000	8.52E-03	promoter	super_enhancer	OR4K17
chr14	20665000	chr14	35395000	1.10E-02	promoter	super_enhancer	OR11G2
chr14	20685000	chr14	69945000	2.31E-03	promoter	super_enhancer	AL356019.1
chr14	20795000	chr14	35395000	1.28E-02	promoter	super_enhancer	SNORD126
chr14	20815000	chr14	61565000	3.36E-03	promoter	super_enhancer	RP11-203M5.2
chr14	20815000	chr14	73785000	2.91E-03	promoter	super_enhancer	RP11-203M5.2
chr14	20905000	chr14	35325000	1.64E-04	promoter	super_enhancer	KLHL33
chr14	20905000	chr14	69855000	5.45E-03	promoter	super_enhancer	KLHL33
chr14	21575000	chr14	49885000	9.25E-03	promoter	super_enhancer	RP11-998D10.4
chr14	21675000	chr14	68705000	3.24E-03	promoter	super_enhancer	LINC00641
chr14	21675000	chr14	93105000	2.37E-03	promoter	super_enhancer	LINC00641
chr14	21755000	chr14	35325000	1.77E-02	promoter	super_enhancer	RPGRIP1
chr14	21755000	chr14	49905000	7.70E-03	promoter	super_enhancer	RPGRIP1
chr14	21755000	chr14	69895000	4.89E-03	promoter	super_enhancer	RPGRIP1
chr14	21755000	chr14	100445000	2.41E-03	promoter	super_enhancer	RPGRIP1
chr14	21925000	chr14	35395000	1.63E-02	promoter	super_enhancer	CHD8
chr14	21925000	chr14	68975000	5.25E-03	promoter	super_enhancer	CHD8
chr14	21925000	chr14	76915000	4.21E-03	promoter	super_enhancer	CHD8
chr14	21945000	chr14	69985000	4.39E-03	promoter	super_enhancer	RAB2B
chr14	21945000	chr14	103075000	2.58E-03	promoter	super_enhancer	RAB2B
chr14	21945000	chr14	104675000	2.74E-03	promoter	super_enhancer	RAB2B
chr14	22055000	chr14	100395000	2.52E-03	promoter	super_enhancer	AE000658.25
chr14	22185000	chr14	49905000	6.84E-03	promoter	super_enhancer	AE000658.31

chr14	22185000	chr14	74575000	3.95E-03	promoter	super_enhancer	AE000658.31
chr14	22355000	chr14	49895000	5.31E-03	promoter	super_enhancer	TRAV12-2
chr14	22465000	chr14	68605000	2.37E-03	promoter	super_enhancer	TRAV17
chr14	22615000	chr14	55095000	3.96E-03	promoter	super_enhancer	TRAV27
chr14	22615000	chr14	69975000	2.62E-03	promoter	super_enhancer	TRAV27
chr14	22635000	chr14	76955000	4.51E-03	promoter	super_enhancer	TRAV30
chr14	22945000	chr14	22975000	2.48E-04	promoter	super_enhancer	TRAJ60
chr14	22945000	chr14	99565000	2.03E-03	promoter	super_enhancer	TRAJ60
chr14	22955000	chr14	68515000	3.42E-03	promoter	super_enhancer	TRAJ52
chr14	22955000	chr14	68935000	4.00E-03	promoter	super_enhancer	TRAJ52
chr14	22975000	chr14	59655000	6.20E-03	super_enhancer	promoter	DAAM1
chr14	22985000	chr14	68515000	4.24E-03	promoter	super_enhancer	TRAJ28
chr14	22995000	chr14	49975000	8.15E-03	promoter	super_enhancer	TRAJ18
chr14	22995000	chr14	102555000	2.96E-03	promoter	super_enhancer	TRAJ18
chr14	23025000	chr14	35345000	1.50E-02	promoter	super_enhancer	AE000662.92
chr14	23165000	chr14	35325000	1.58E-02	promoter	super_enhancer	CTD-2555K7.1
chr14	23235000	chr14	49905000	7.47E-03	promoter	super_enhancer	OXA1L
chr14	23235000	chr14	100425000	2.88E-03	promoter	super_enhancer	OXA1L
chr14	23305000	chr14	35385000	1.82E-02	promoter	super_enhancer	MMP14
chr14	23305000	chr14	55115000	8.53E-03	promoter	super_enhancer	MMP14
chr14	23505000	chr14	68975000	5.89E-03	promoter	super_enhancer	PSMB5
chr14	23565000	chr14	35345000	1.45E-02	promoter	super_enhancer	ACIN1
chr14	23565000	chr14	61565000	4.19E-03	promoter	super_enhancer	ACIN1
chr14	23655000	chr14	68705000	4.65E-03	promoter	super_enhancer	RNF212B

chr14	23655000	chr14	68815000	5.16E-03	promoter	super_enhancer	RNF212B
chr14	23655000	chr14	69875000	5.58E-03	promoter	super_enhancer	RNF212B
chr14	23655000	chr14	69985000	5.80E-03	promoter	super_enhancer	RNF212B
chr14	23775000	chr14	35355000	2.15E-02	promoter	super_enhancer	BCL2L2-PABPN1
chr14	23775000	chr14	35385000	1.91E-02	promoter	super_enhancer	BCL2L2-PABPN1
chr14	23775000	chr14	55085000	8.34E-03	promoter	super_enhancer	BCL2L2-PABPN1
chr14	23775000	chr14	93085000	2.89E-03	promoter	super_enhancer	BCL2L2-PABPN1
chr14	23825000	chr14	35395000	2.23E-02	promoter	super_enhancer	RP11-124D2.3
chr14	23825000	chr14	55085000	8.44E-03	promoter	super_enhancer	RP11-124D2.3
chr14	23825000	chr14	104975000	3.11E-03	promoter	super_enhancer	RP11-124D2.3
chr14	23835000	chr14	101725000	2.76E-03	promoter	super_enhancer	EFS
chr14	23905000	chr14	35335000	1.57E-02	promoter	super_enhancer	MYH7
chr14	24025000	chr14	61565000	5.37E-03	promoter	super_enhancer	ZFHX2
chr14	24025000	chr14	70025000	6.09E-03	promoter	super_enhancer	ZFHX2
chr14	24025000	chr14	76935000	4.67E-03	promoter	super_enhancer	ZFHX2
chr14	24615000	chr14	69935000	5.01E-03	promoter	super_enhancer	RNF31
chr14	24665000	chr14	35345000	1.68E-02	promoter	super_enhancer	TM9SF1
chr14	24665000	chr14	35395000	1.88E-02	promoter	super_enhancer	TM9SF1
chr14	24675000	chr14	35385000	1.68E-02	promoter	super_enhancer	TSSK4
chr14	24675000	chr14	50065000	1.03E-02	promoter	super_enhancer	TSSK4
chr14	24685000	chr14	49885000	1.03E-02	promoter	super_enhancer	AL136419.6
chr14	24735000	chr14	76935000	4.08E-03	promoter	super_enhancer	TGM1
chr14	24735000	chr14	93085000	2.37E-03	promoter	super_enhancer	TGM1
chr14	24775000	chr14	35355000	1.99E-02	promoter	super_enhancer	LTB4R2

chr14	24805000	chr14	35325000	2.60E-02	promoter	super_enhancer	ADCY4
chr14	24805000	chr14	49905000	1.08E-02	promoter	super_enhancer	ADCY4
chr14	25065000	chr14	93045000	3.15E-03	promoter	super_enhancer	RP11-104E19.1
chr14	27065000	chr14	50045000	6.81E-03	promoter	super_enhancer	RP11-483C6.1
chr14	27065000	chr14	104975000	1.63E-03	promoter	super_enhancer	RP11-483C6.1
chr14	27305000	chr14	35365000	1.15E-02	promoter	super_enhancer	RP11-384J4.2
chr14	28315000	chr14	35395000	1.57E-02	promoter	super_enhancer	CTD-3006G17.2
chr14	29235000	chr14	35395000	2.26E-02	promoter	super_enhancer	FOXG1-AS1
chr14	32545000	chr14	69885000	5.15E-03	promoter	super_enhancer	ARHGAP5
chr14	34955000	chr14	68525000	7.24E-03	promoter	super_enhancer	RPL23AP71
chr14	34955000	chr14	76925000	1.05E-02	promoter	super_enhancer	RPL23AP71
chr14	35145000	chr14	35325000	2.84E-02	promoter	super_enhancer	RNU6-1261P
chr14	35145000	chr14	35345000	1.56E-02	promoter	super_enhancer	RNU6-1261P
chr14	35145000	chr14	35385000	3.74E-02	promoter	super_enhancer	RNU6-1261P
chr14	35185000	chr14	69965000	7.01E-03	promoter	super_enhancer	CFL2
chr14	35185000	chr14	70015000	6.69E-03	promoter	super_enhancer	CFL2
chr14	35335000	chr14	55765000	9.95E-03	super_enhancer	promoter	CHMP4BP1
chr14	35355000	chr14	35875000	8.86E-04	super_enhancer	promoter	NFKBIA
chr14	35355000	chr14	50795000	1.50E-02	super_enhancer	promoter	RP11-247L20.4
chr14	35355000	chr14	74225000	6.17E-03	super_enhancer	promoter	MIR4505
chr14	35365000	chr14	45745000	1.43E-02	super_enhancer	promoter	RNU6-552P
chr14	35365000	chr14	61105000	7.87E-03	super_enhancer	promoter	SALRNA1
chr14	35365000	chr14	68125000	6.99E-03	super_enhancer	promoter	RNA5SP386
chr14	35375000	chr14	36295000	3.77E-02	super_enhancer	promoter	BRMS1L

chr14	35375000	chr14	39645000	4.62E-02	super_enhancer	promoter	RP11-407N17.4
chr14	35375000	chr14	42075000	1.99E-02	super_enhancer	promoter	CTD-2298J14.2
chr14	35375000	chr14	78015000	5.97E-03	super_enhancer	promoter	AF111168.3
chr14	35375000	chr14	97105000	3.64E-03	super_enhancer	promoter	RN7SKP108
chr14	35375000	chr14	106745000	2.68E-03	super_enhancer	promoter	HOMER2P2
chr14	35385000	chr14	45335000	1.12E-04	super_enhancer	promoter	DOCK11P1
chr14	35385000	chr14	90835000	3.22E-03	super_enhancer	promoter	RPL21P11
chr14	35395000	chr14	75745000	7.78E-03	super_enhancer	promoter	FOS
chr14	35395000	chr14	96485000	2.99E-03	super_enhancer	promoter	RP11-241N4.2
chr14	35395000	chr14	103655000	3.29E-03	super_enhancer	promoter	LINC00605
chr14	35705000	chr14	49895000	1.28E-02	promoter	super_enhancer	RPL7AP3
chr14	35705000	chr14	68795000	6.11E-03	promoter	super_enhancer	RPL7AP3
chr14	36235000	chr14	68575000	5.03E-03	promoter	super_enhancer	QRSL1P3
chr14	36295000	chr14	49895000	1.01E-02	promoter	super_enhancer	BRMS1L
chr14	36295000	chr14	76915000	4.85E-03	promoter	super_enhancer	BRMS1L
chr14	37005000	chr14	68685000	6.07E-03	promoter	super_enhancer	PHKBP2
chr14	37005000	chr14	102555000	3.35E-03	promoter	super_enhancer	PHKBP2
chr14	37005000	chr14	104695000	4.17E-03	promoter	super_enhancer	PHKBP2
chr14	37655000	chr14	93135000	3.27E-03	promoter	super_enhancer	RNU6-273P
chr14	38065000	chr14	68605000	5.26E-03	promoter	super_enhancer	FOXA1
chr14	38475000	chr14	70025000	7.10E-03	promoter	super_enhancer	CTD-2058B24.3
chr14	39195000	chr14	55085000	1.21E-02	promoter	super_enhancer	KRT8P1
chr14	39195000	chr14	76925000	8.36E-03	promoter	super_enhancer	KRT8P1
chr14	39625000	chr14	49975000	1.59E-02	promoter	super_enhancer	RPL7AP2

chr14	39625000	chr14	50055000	1.83E-02	promoter	super_enhancer	RPL7AP2
chr14	39625000	chr14	70005000	8.04E-03	promoter	super_enhancer	RPL7AP2
chr14	39645000	chr14	50005000	1.97E-02	promoter	super_enhancer	RP11-407N17.4
chr14	39645000	chr14	68515000	7.22E-03	promoter	super_enhancer	RP11-407N17.4
chr14	44855000	chr14	50065000	2.85E-02	promoter	super_enhancer	RP11-305B6.3
chr14	44855000	chr14	69895000	7.43E-03	promoter	super_enhancer	RP11-305B6.3
chr14	44855000	chr14	74555000	6.38E-03	promoter	super_enhancer	RP11-305B6.3
chr14	45335000	chr14	68595000	7.83E-03	promoter	super_enhancer	DOCK11P1
chr14	45365000	chr14	50005000	2.59E-02	promoter	super_enhancer	C14orf28
chr14	45745000	chr14	69845000	7.95E-03	promoter	super_enhancer	RNU6-552P
chr14	45745000	chr14	70025000	9.19E-03	promoter	super_enhancer	RNU6-552P
chr14	45845000	chr14	49975000	2.95E-02	promoter	super_enhancer	RP11-454K7.1
chr14	46185000	chr14	68695000	7.09E-03	promoter	super_enhancer	RP11-369C8.1
chr14	48145000	chr14	49975000	6.72E-03	promoter	super_enhancer	MDGA2
chr14	48265000	chr14	49895000	2.94E-03	promoter	super_enhancer	LINC00648
chr14	49885000	chr14	58305000	1.89E-02	super_enhancer	promoter	RN7SKP99
chr14	49885000	chr14	60625000	1.88E-02	super_enhancer	promoter	SCOCP1
chr14	49885000	chr14	60975000	2.02E-02	super_enhancer	promoter	SIX6
chr14	49885000	chr14	75125000	1.09E-02	super_enhancer	promoter	RP11-173A8.2
chr14	49895000	chr14	60045000	9.65E-03	super_enhancer	promoter	CCDC175
chr14	49895000	chr14	61345000	1.05E-02	super_enhancer	promoter	RP11-193F5.1
chr14	49895000	chr14	83485000	4.72E-03	super_enhancer	promoter	RP11-452D21.2
chr14	49895000	chr14	91295000	5.83E-03	super_enhancer	promoter	CTD-3035D6.2
chr14	49895000	chr14	103655000	3.51E-03	super_enhancer	promoter	LINC00605

chr14	49895000	chr14	104845000	4.33E-03	super_enhancer	promoter	RP11-260M19.1
chr14	49905000	chr14	56585000	1.53E-02	super_enhancer	promoter	CTD-2588E21.1
chr14	49905000	chr14	60045000	9.42E-03	super_enhancer	promoter	CCDC175
chr14	49905000	chr14	61125000	1.44E-02	super_enhancer	promoter	SIX1
chr14	49905000	chr14	101245000	4.62E-03	super_enhancer	promoter	MEG3
chr14	49905000	chr14	101435000	3.38E-03	super_enhancer	promoter	SNORD114-12
chr14	49975000	chr14	75595000	7.45E-03	super_enhancer	promoter	NEK9
chr14	49995000	chr14	54415000	4.10E-02	super_enhancer	promoter	MIR5580
chr14	49995000	chr14	67665000	1.42E-02	super_enhancer	promoter	RP11-125H8.1
chr14	49995000	chr14	69095000	1.03E-02	super_enhancer	promoter	CTD-2325P2.4
chr14	49995000	chr14	71685000	9.49E-03	super_enhancer	promoter	RP6-91H8.5
chr14	49995000	chr14	74225000	8.56E-03	super_enhancer	promoter	MIR4505
chr14	49995000	chr14	75125000	8.92E-03	super_enhancer	promoter	RP11-173A8.2
chr14	49995000	chr14	94405000	6.22E-03	super_enhancer	promoter	RP11-131H24.4
chr14	50005000	chr14	88965000	5.84E-03	super_enhancer	promoter	RP11-507K2.2
chr14	50005000	chr14	97105000	4.89E-03	super_enhancer	promoter	RN7SKP108
chr14	50045000	chr14	65935000	1.46E-02	super_enhancer	promoter	RPL21P8
chr14	50045000	chr14	71375000	9.67E-03	super_enhancer	promoter	RP1-292L20.3
chr14	50045000	chr14	77425000	1.04E-02	super_enhancer	promoter	RP11-7F17.7
chr14	50055000	chr14	54425000	4.08E-02	super_enhancer	promoter	BMP4
chr14	50055000	chr14	65935000	1.50E-02	super_enhancer	promoter	RPL21P8
chr14	50055000	chr14	66455000	1.42E-02	super_enhancer	promoter	CTD-2014B16.5
chr14	50065000	chr14	51555000	1.50E-02	super_enhancer	promoter	RP11-1140I5.1
chr14	50065000	chr14	54415000	4.83E-02	super_enhancer	promoter	MIR5580

chr14	50065000	chr14	58755000	2.00E-02	super_enhancer	promoter	LINC00216
chr14	50065000	chr14	69725000	1.48E-02	super_enhancer	promoter	GALNT16
chr14	50065000	chr14	71165000	1.22E-02	super_enhancer	promoter	LINC01269
chr14	50155000	chr14	68145000	1.43E-02	promoter	super_enhancer	POLE2
chr14	50155000	chr14	93075000	5.35E-03	promoter	super_enhancer	POLE2
chr14	50305000	chr14	104695000	6.27E-03	promoter	super_enhancer	RNU6-539P
chr14	50475000	chr14	55115000	3.80E-02	promoter	super_enhancer	LINC01588
chr14	50475000	chr14	104685000	5.22E-03	promoter	super_enhancer	LINC01588
chr14	50795000	chr14	55125000	3.86E-02	promoter	super_enhancer	RP11-247L20.4
chr14	50795000	chr14	93065000	6.13E-03	promoter	super_enhancer	RP11-247L20.4
chr14	50795000	chr14	100405000	4.72E-03	promoter	super_enhancer	RP11-247L20.4
chr14	50915000	chr14	69885000	9.84E-03	promoter	super_enhancer	RP11-406H23.2
chr14	50915000	chr14	93025000	5.64E-03	promoter	super_enhancer	RP11-406H23.2
chr14	51105000	chr14	55115000	3.42E-02	promoter	super_enhancer	RN7SL452P
chr14	51135000	chr14	68595000	8.53E-03	promoter	super_enhancer	SAV1
chr14	51135000	chr14	69945000	9.29E-03	promoter	super_enhancer	SAV1
chr14	51865000	chr14	71145000	1.11E-02	promoter	super_enhancer	RP11-255G12.3
chr14	51955000	chr14	76965000	1.03E-02	promoter	super_enhancer	FRMD6
chr14	52455000	chr14	71145000	1.06E-02	promoter	super_enhancer	RP11-463J10.4
chr14	52455000	chr14	104975000	4.13E-03	promoter	super_enhancer	RP11-463J10.4
chr14	54415000	chr14	69975000	1.40E-02	promoter	super_enhancer	MIR5580
chr14	54415000	chr14	70025000	1.69E-02	promoter	super_enhancer	MIR5580
chr14	54415000	chr14	73785000	1.19E-02	promoter	super_enhancer	MIR5580
chr14	54425000	chr14	68935000	1.30E-02	promoter	super_enhancer	BMP4

chr14	55085000	chr14	60975000	2.57E-02	super_enhancer	promoter	SIX6
chr14	55085000	chr14	97105000	5.09E-03	super_enhancer	promoter	RN7SKP108
chr14	55095000	chr14	58755000	3.96E-02	super_enhancer	promoter	LINC00216
chr14	55095000	chr14	58895000	3.30E-02	super_enhancer	promoter	TIMM9
chr14	55115000	chr14	76815000	1.06E-02	super_enhancer	promoter	AC016543.1
chr14	55125000	chr14	56215000	4.84E-02	super_enhancer	promoter	RP11-813I20.2
chr14	55125000	chr14	64085000	1.78E-02	super_enhancer	promoter	RNU6-597P
chr14	55325000	chr14	68565000	1.25E-02	promoter	super_enhancer	RNU6ATAC9P
chr14	55325000	chr14	68685000	1.44E-02	promoter	super_enhancer	RNU6ATAC9P
chr14	55325000	chr14	93045000	7.49E-03	promoter	super_enhancer	RNU6ATAC9P
chr14	55325000	chr14	93125000	7.19E-03	promoter	super_enhancer	RNU6ATAC9P
chr14	55345000	chr14	68155000	1.43E-02	promoter	super_enhancer	MIR4308
chr14	55345000	chr14	92995000	5.90E-03	promoter	super_enhancer	MIR4308
chr14	55345000	chr14	102585000	5.10E-03	promoter	super_enhancer	MIR4308
chr14	55495000	chr14	93105000	5.70E-03	promoter	super_enhancer	WDHD1
chr14	55695000	chr14	93085000	5.07E-03	promoter	super_enhancer	AL158801.1
chr14	55695000	chr14	99565000	5.24E-03	promoter	super_enhancer	AL158801.1
chr14	56025000	chr14	76945000	1.11E-02	promoter	super_enhancer	KTN1
chr14	56025000	chr14	93035000	4.32E-03	promoter	super_enhancer	KTN1
chr14	56215000	chr14	103075000	6.91E-03	promoter	super_enhancer	RP11-813I20.2
chr14	57115000	chr14	68965000	1.30E-02	promoter	super_enhancer	RP11-1085N6.2
chr14	57115000	chr14	69855000	1.28E-02	promoter	super_enhancer	RP11-1085N6.2
chr14	58305000	chr14	61565000	3.46E-02	promoter	super_enhancer	RN7SKP99
chr14	58645000	chr14	69895000	1.54E-02	promoter	super_enhancer	RN7SL598P

chr14	58645000	chr14	73785000	1.20E-02	promoter	super_enhancer	RN7SL598P
chr14	58645000	chr14	100425000	6.01E-03	promoter	super_enhancer	RN7SL598P
chr14	58755000	chr14	61565000	4.91E-02	promoter	super_enhancer	LINC00216
chr14	58755000	chr14	68695000	1.20E-02	promoter	super_enhancer	LINC00216
chr14	58765000	chr14	69895000	1.12E-02	promoter	super_enhancer	C14orf37
chr14	58765000	chr14	69975000	1.04E-02	promoter	super_enhancer	C14orf37
chr14	58765000	chr14	93075000	4.64E-03	promoter	super_enhancer	C14orf37
chr14	58865000	chr14	68805000	1.74E-02	promoter	super_enhancer	RP11-517O13.1
chr14	58895000	chr14	69895000	1.33E-02	promoter	super_enhancer	TIMM9
chr14	58895000	chr14	69975000	1.23E-02	promoter	super_enhancer	TIMM9
chr14	58895000	chr14	103125000	3.68E-03	promoter	super_enhancer	TIMM9
chr14	59295000	chr14	68955000	2.00E-02	promoter	super_enhancer	RP11-112J1.3
chr14	59655000	chr14	68555000	1.58E-02	promoter	super_enhancer	DAAM1
chr14	59655000	chr14	74575000	1.51E-02	promoter	super_enhancer	DAAM1
chr14	59655000	chr14	76935000	1.36E-02	promoter	super_enhancer	DAAM1
chr14	59655000	chr14	100435000	5.78E-03	promoter	super_enhancer	DAAM1
chr14	60045000	chr14	68705000	1.05E-02	promoter	super_enhancer	CCDC175
chr14	60045000	chr14	70035000	1.47E-02	promoter	super_enhancer	CCDC175
chr14	60115000	chr14	68555000	1.40E-02	promoter	super_enhancer	MIR5586
chr14	60625000	chr14	68155000	2.21E-02	promoter	super_enhancer	SCOCP1
chr14	60625000	chr14	70025000	1.86E-02	promoter	super_enhancer	SCOCP1
chr14	60625000	chr14	93145000	6.48E-03	promoter	super_enhancer	SCOCP1
chr14	60625000	chr14	99565000	5.73E-03	promoter	super_enhancer	SCOCP1
chr14	60625000	chr14	102565000	6.45E-03	promoter	super_enhancer	SCOCP1

chr14	60715000	chr14	93085000	5.01E-03	promoter	super_enhancer	CTD-2184C24.2
chr14	60975000	chr14	68815000	2.05E-02	promoter	super_enhancer	SIX6
chr14	60975000	chr14	104965000	5.82E-03	promoter	super_enhancer	SIX6
chr14	61105000	chr14	68535000	1.61E-02	promoter	super_enhancer	SALRNA1
chr14	61105000	chr14	74585000	1.24E-02	promoter	super_enhancer	SALRNA1
chr14	61105000	chr14	76945000	1.41E-02	promoter	super_enhancer	SALRNA1
chr14	61125000	chr14	93005000	6.15E-03	promoter	super_enhancer	SIX1
chr14	61345000	chr14	68705000	1.38E-02	promoter	super_enhancer	RP11-193F5.1
chr14	61345000	chr14	69935000	1.49E-02	promoter	super_enhancer	RP11-193F5.1
chr14	61345000	chr14	70035000	1.85E-02	promoter	super_enhancer	RP11-193F5.1
chr14	61345000	chr14	93095000	6.12E-03	promoter	super_enhancer	RP11-193F5.1
chr14	61345000	chr14	104675000	5.03E-03	promoter	super_enhancer	RP11-193F5.1
chr14	61565000	chr14	77495000	7.20E-03	super_enhancer	promoter	IRF2BPL
chr14	61565000	chr14	77925000	1.05E-02	super_enhancer	promoter	VIPAS39
chr14	61565000	chr14	106715000	3.36E-03	super_enhancer	promoter	IGHV3-22
chr14	61995000	chr14	69905000	1.92E-02	promoter	super_enhancer	RP11-47I22.4
chr14	61995000	chr14	70035000	2.43E-02	promoter	super_enhancer	RP11-47I22.4
chr14	62585000	chr14	68975000	2.10E-02	promoter	super_enhancer	RP11-355I22.2
chr14	62585000	chr14	93135000	5.47E-03	promoter	super_enhancer	RP11-355I22.2
chr14	62585000	chr14	104965000	4.39E-03	promoter	super_enhancer	RP11-355I22.2
chr14	63595000	chr14	68145000	3.33E-02	promoter	super_enhancer	RP11-1112J20.2
chr14	63595000	chr14	76955000	1.32E-02	promoter	super_enhancer	RP11-1112J20.2
chr14	63595000	chr14	100695000	5.93E-03	promoter	super_enhancer	RP11-1112J20.2
chr14	64035000	chr14	93045000	9.31E-03	promoter	super_enhancer	CTD-2302E22.1

chr14	64065000	chr14	69875000	2.91E-02	promoter	super_enhancer	CTD-2302E22.2
chr14	64065000	chr14	69935000	2.85E-02	promoter	super_enhancer	CTD-2302E22.2
chr14	64085000	chr14	68555000	2.61E-02	promoter	super_enhancer	RNU6-597P
chr14	64085000	chr14	68955000	3.31E-02	promoter	super_enhancer	RNU6-597P
chr14	64085000	chr14	69875000	2.76E-02	promoter	super_enhancer	RNU6-597P
chr14	64085000	chr14	71145000	1.99E-02	promoter	super_enhancer	RNU6-597P
chr14	64195000	chr14	68805000	3.68E-02	promoter	super_enhancer	SGPP1
chr14	64195000	chr14	68815000	3.49E-02	promoter	super_enhancer	SGPP1
chr14	64195000	chr14	73755000	1.93E-02	promoter	super_enhancer	SGPP1
chr14	64195000	chr14	93075000	8.15E-03	promoter	super_enhancer	SGPP1
chr14	64275000	chr14	68545000	3.30E-02	promoter	super_enhancer	RPS28P1
chr14	64275000	chr14	93095000	1.02E-02	promoter	super_enhancer	RPS28P1
chr14	64275000	chr14	93105000	9.76E-03	promoter	super_enhancer	RPS28P1
chr14	64275000	chr14	102545000	6.53E-03	promoter	super_enhancer	RPS28P1
chr14	64805000	chr14	93075000	8.67E-03	promoter	super_enhancer	ESR2
chr14	64915000	chr14	68965000	4.64E-02	promoter	super_enhancer	CTD-2555O16.2
chr14	65735000	chr14	68585000	4.15E-02	promoter	super_enhancer	RPL21P7
chr14	65735000	chr14	68695000	4.40E-02	promoter	super_enhancer	RPL21P7
chr14	65735000	chr14	68945000	4.74E-02	promoter	super_enhancer	RPL21P7
chr14	65735000	chr14	93095000	9.32E-03	promoter	super_enhancer	RPL21P7
chr14	65745000	chr14	73785000	1.94E-02	promoter	super_enhancer	PTBP1P
chr14	65935000	chr14	69975000	3.79E-02	promoter	super_enhancer	RPL21P8
chr14	65935000	chr14	93135000	8.37E-03	promoter	super_enhancer	RPL21P8
chr14	66455000	chr14	74585000	1.78E-02	promoter	super_enhancer	CTD-2014B16.5

chr14	67065000	chr14	93035000	5.85E-03	promoter	super_enhancer	AL139295.1
chr14	67065000	chr14	102555000	6.90E-03	promoter	super_enhancer	AL139295.1
chr14	67665000	chr14	102525000	9.47E-03	promoter	super_enhancer	RP11-125H8.1
chr14	68125000	chr14	100415000	9.52E-03	promoter	super_enhancer	RNA5SP386
chr14	68145000	chr14	77245000	2.57E-02	super_enhancer	promoter	RP11-488C13.7
chr14	68145000	chr14	80855000	1.54E-02	super_enhancer	promoter	DIO2
chr14	68145000	chr14	105445000	7.67E-03	super_enhancer	promoter	AHNAK2
chr14	68155000	chr14	85995000	1.39E-02	super_enhancer	promoter	RP11-497E19.2
chr14	68155000	chr14	100705000	7.88E-03	super_enhancer	promoter	RP11-638I2.4
chr14	68285000	chr14	69855000	1.72E-02	promoter	super_enhancer	RAD51B
chr14	68285000	chr14	69955000	1.14E-03	promoter	super_enhancer	RAD51B
chr14	68515000	chr14	103345000	5.05E-03	super_enhancer	promoter	AL117209.1
chr14	68525000	chr14	77245000	1.71E-02	super_enhancer	promoter	RP11-488C13.7
chr14	68525000	chr14	77965000	1.79E-02	super_enhancer	promoter	ISM2
chr14	68545000	chr14	71375000	4.46E-02	super_enhancer	promoter	RP1-292L20.3
chr14	68545000	chr14	77925000	1.49E-02	super_enhancer	promoter	VIPAS39
chr14	68545000	chr14	103745000	5.52E-03	super_enhancer	promoter	RAP2CP1
chr14	68545000	chr14	104265000	4.84E-03	super_enhancer	promoter	SNORD51
chr14	68565000	chr14	73495000	3.18E-02	super_enhancer	promoter	ZFYVE1
chr14	68565000	chr14	104605000	7.86E-03	super_enhancer	promoter	CTD-2223O18.1
chr14	68575000	chr14	75475000	1.73E-02	super_enhancer	promoter	RP11-950C14.3
chr14	68585000	chr14	107035000	3.21E-03	super_enhancer	promoter	IGHV5-51
chr14	68595000	chr14	89055000	7.10E-03	super_enhancer	promoter	RP11-507K2.6
chr14	68605000	chr14	93375000	6.30E-03	super_enhancer	promoter	RP11-862G15.2

chr14	68605000	chr14	101405000	3.71E-03	super_enhancer	promoter	SNORD113-5
chr14	68675000	chr14	106365000	7.11E-03	super_enhancer	promoter	IGHD2-15
chr14	68695000	chr14	76305000	1.41E-02	super_enhancer	promoter	RP11-270M14.1
chr14	68705000	chr14	72315000	2.20E-02	super_enhancer	promoter	RP6-114E22.1
chr14	68705000	chr14	76315000	1.60E-02	super_enhancer	promoter	RP11-270M14.4
chr14	68705000	chr14	99625000	5.41E-03	super_enhancer	promoter	AL162151.4
chr14	68705000	chr14	103605000	6.97E-03	super_enhancer	promoter	RP11-736N17.4
chr14	68705000	chr14	107185000	4.64E-03	super_enhancer	promoter	IGHV3-71
chr14	68795000	chr14	69355000	3.18E-02	super_enhancer	promoter	HMGN1P3
chr14	68795000	chr14	76045000	2.02E-02	super_enhancer	promoter	FLVCR2
chr14	68795000	chr14	91295000	1.02E-02	super_enhancer	promoter	CTD-3035D6.2
chr14	68795000	chr14	101525000	4.98E-03	super_enhancer	promoter	MIR154
chr14	68805000	chr14	75725000	2.30E-02	super_enhancer	promoter	RP11-293M10.2
chr14	68805000	chr14	102605000	7.10E-03	super_enhancer	promoter	WDR20
chr14	68805000	chr14	105635000	7.82E-03	super_enhancer	promoter	JAG2
chr14	68815000	chr14	95665000	8.98E-03	super_enhancer	promoter	RP11-725G5.3
chr14	68945000	chr14	73655000	2.81E-02	super_enhancer	promoter	RP4-687K1.2
chr14	68955000	chr14	69095000	7.56E-03	super_enhancer	promoter	CTD-2325P2.4
chr14	68955000	chr14	101465000	6.54E-03	super_enhancer	promoter	SNORD113
chr14	68955000	chr14	103565000	6.94E-03	super_enhancer	promoter	EXOC3L4
chr14	68965000	chr14	69355000	5.76E-03	super_enhancer	promoter	HMGN1P3
chr14	68975000	chr14	74705000	3.59E-02	super_enhancer	promoter	VSX2
chr14	68975000	chr14	77965000	2.79E-02	super_enhancer	promoter	ISM2
chr14	68975000	chr14	92415000	9.02E-03	super_enhancer	promoter	FBLN5

chr14	69095000	chr14	100405000	7.23E-03	promoter	super_enhancer	CTD-2325P2.4
chr14	69355000	chr14	69955000	1.17E-02	promoter	super_enhancer	HMGN1P3
chr14	69355000	chr14	100445000	8.53E-03	promoter	super_enhancer	HMGN1P3
chr14	69445000	chr14	74575000	2.97E-02	promoter	super_enhancer	ACTN1-AS1
chr14	69445000	chr14	102545000	6.65E-03	promoter	super_enhancer	ACTN1-AS1
chr14	69445000	chr14	104685000	7.43E-03	promoter	super_enhancer	ACTN1-AS1
chr14	69725000	chr14	69865000	1.59E-02	promoter	super_enhancer	GALNT16
chr14	69725000	chr14	69925000	9.24E-03	promoter	super_enhancer	GALNT16
chr14	69725000	chr14	69965000	2.16E-02	promoter	super_enhancer	GALNT16
chr14	69725000	chr14	70015000	2.36E-03	promoter	super_enhancer	GALNT16
chr14	69725000	chr14	70025000	4.95E-04	promoter	super_enhancer	GALNT16
chr14	69725000	chr14	93135000	1.09E-02	promoter	super_enhancer	GALNT16
chr14	69845000	chr14	79665000	1.29E-02	super_enhancer	promoter	RP11-588P7.1
chr14	69855000	chr14	73675000	2.69E-02	super_enhancer	promoter	AC004846.1
chr14	69855000	chr14	76315000	2.23E-02	super_enhancer	promoter	RP11-270M14.4
chr14	69855000	chr14	89865000	1.02E-02	super_enhancer	promoter	RP11-33N16.2
chr14	69865000	chr14	90835000	6.53E-03	super_enhancer	promoter	RPL21P11
chr14	69865000	chr14	106875000	3.70E-03	super_enhancer	promoter	IGHVIII-38-1
chr14	69875000	chr14	76315000	2.31E-02	super_enhancer	promoter	RP11-270M14.4
chr14	69875000	chr14	77965000	2.86E-02	super_enhancer	promoter	ISM2
chr14	69875000	chr14	106445000	6.16E-03	super_enhancer	promoter	AB019441.29
chr14	69885000	chr14	75895000	2.27E-02	super_enhancer	promoter	RP11-293M10.6
chr14	69885000	chr14	77425000	2.14E-02	super_enhancer	promoter	RP11-7F17.7
chr14	69885000	chr14	77915000	1.81E-02	super_enhancer	promoter	AF111168.1

chr14	69885000	chr14	101465000	5.58E-03	super_enhancer	promoter	SNORD113
chr14	69895000	chr14	71165000	2.36E-02	super_enhancer	promoter	LINC01269
chr14	69895000	chr14	92335000	1.01E-02	super_enhancer	promoter	TC2N
chr14	69895000	chr14	94415000	9.29E-03	super_enhancer	promoter	MIR4506
chr14	69905000	chr14	70265000	2.58E-02	super_enhancer	promoter	SLC10A1
chr14	69905000	chr14	73655000	4.40E-02	super_enhancer	promoter	RP4-687K1.2
chr14	69905000	chr14	77435000	2.54E-02	super_enhancer	promoter	RN7SL356P
chr14	69905000	chr14	100575000	8.20E-03	super_enhancer	promoter	MIR342
chr14	69905000	chr14	103345000	6.97E-03	super_enhancer	promoter	AL117209.1
chr14	69905000	chr14	104395000	9.11E-03	super_enhancer	promoter	C14orf2
chr14	69915000	chr14	77245000	2.47E-02	super_enhancer	promoter	RP11-488C13.7
chr14	69915000	chr14	99385000	8.36E-03	super_enhancer	promoter	RP11-1127D7.2
chr14	69915000	chr14	101245000	8.75E-03	super_enhancer	promoter	MEG3
chr14	69915000	chr14	106975000	4.99E-03	super_enhancer	promoter	IGHV3-47
chr14	69925000	chr14	101495000	6.04E-03	super_enhancer	promoter	MIR494
chr14	69925000	chr14	105445000	5.46E-03	super_enhancer	promoter	AHNAK2
chr14	69935000	chr14	77425000	2.57E-02	super_enhancer	promoter	RP11-7F17.7
chr14	69935000	chr14	105865000	5.88E-03	super_enhancer	promoter	TEX22
chr14	69945000	chr14	104395000	9.28E-03	super_enhancer	promoter	C14orf2
chr14	69965000	chr14	101055000	6.05E-03	super_enhancer	promoter	BEGAIN
chr14	69975000	chr14	75475000	2.61E-02	super_enhancer	promoter	RP11-950C14.3
chr14	69975000	chr14	77825000	1.83E-02	super_enhancer	promoter	RP11-493G17.4
chr14	69985000	chr14	75745000	3.69E-02	super_enhancer	promoter	FOS
chr14	69985000	chr14	77915000	2.30E-02	super_enhancer	promoter	AF111168.1

chr14	69995000	chr14	76305000	2.37E-02	super_enhancer	promoter	RP11-270M14.1
chr14	69995000	chr14	93215000	1.04E-02	super_enhancer	promoter	LGMN
chr14	69995000	chr14	100705000	7.76E-03	super_enhancer	promoter	RP11-638I2.4
chr14	69995000	chr14	103745000	7.60E-03	super_enhancer	promoter	RAP2CP1
chr14	70005000	chr14	73535000	4.94E-02	super_enhancer	promoter	RP11-109N23.5
chr14	70005000	chr14	74845000	3.85E-02	super_enhancer	promoter	RPS2P2
chr14	70005000	chr14	94985000	8.27E-03	super_enhancer	promoter	SERPINA12
chr14	70005000	chr14	105715000	8.17E-03	super_enhancer	promoter	BTBD6
chr14	70005000	chr14	106775000	8.39E-03	super_enhancer	promoter	IGHV7-27
chr14	70015000	chr14	105955000	8.56E-03	super_enhancer	promoter	C14orf80
chr14	70025000	chr14	75125000	3.44E-02	super_enhancer	promoter	RP11-173A8.2
chr14	70025000	chr14	75475000	3.18E-02	super_enhancer	promoter	RP11-950C14.3
chr14	70025000	chr14	79165000	1.68E-02	super_enhancer	promoter	RP11-232C2.3
chr14	70035000	chr14	70265000	4.59E-02	super_enhancer	promoter	SLC10A1
chr14	70035000	chr14	71375000	2.62E-02	super_enhancer	promoter	RP1-292L20.3
chr14	70035000	chr14	75745000	4.42E-02	super_enhancer	promoter	FOS
chr14	70035000	chr14	76815000	3.23E-02	super_enhancer	promoter	AC016543.1
chr14	70035000	chr14	103995000	9.59E-03	super_enhancer	promoter	RP11-600F24.7
chr14	70035000	chr14	104315000	9.79E-03	super_enhancer	promoter	PPP1R13B
chr14	70035000	chr14	104395000	1.17E-02	super_enhancer	promoter	C14orf2
chr14	70035000	chr14	106835000	8.89E-03	super_enhancer	promoter	IGHV7-34-1
chr14	70265000	chr14	100395000	8.92E-03	promoter	super_enhancer	SLC10A1
chr14	70265000	chr14	102565000	5.42E-05	promoter	super_enhancer	SLC10A1
chr14	70265000	chr14	102585000	1.04E-02	promoter	super_enhancer	SLC10A1

chr14	70715000	chr14	100385000	7.98E-03	promoter	super_enhancer	ADAM21P1
chr14	70885000	chr14	76955000	4.60E-04	promoter	super_enhancer	SYNJ2BP-COX16
chr14	71125000	chr14	74845000	3.82E-02	super_enhancer	promoter	RPS2P2
chr14	71135000	chr14	82245000	1.57E-02	super_enhancer	promoter	RP11-226P1.1
chr14	71145000	chr14	75745000	3.96E-02	super_enhancer	promoter	FOS
chr14	71145000	chr14	95515000	8.45E-03	super_enhancer	promoter	RP11-991C1.2
chr14	71145000	chr14	106735000	6.79E-03	super_enhancer	promoter	IGHV1-24
chr14	71165000	chr14	74585000	4.69E-02	promoter	super_enhancer	LINC01269
chr14	71165000	chr14	100445000	7.28E-03	promoter	super_enhancer	LINC01269
chr14	71375000	chr14	104675000	7.82E-03	promoter	super_enhancer	RP1-292L20.3
chr14	71685000	chr14	103075000	8.70E-03	promoter	super_enhancer	RP6-91H8.5
chr14	71865000	chr14	76955000	3.12E-02	promoter	super_enhancer	SNORD56B
chr14	71915000	chr14	100395000	6.64E-03	promoter	super_enhancer	RN7SL683P
chr14	72315000	chr14	100415000	5.49E-03	promoter	super_enhancer	RP6-114E22.1
chr14	73495000	chr14	102585000	1.25E-02	promoter	super_enhancer	ZFYVE1
chr14	73535000	chr14	100435000	7.54E-03	promoter	super_enhancer	RP11-109N23.5
chr14	73655000	chr14	103145000	7.85E-03	promoter	super_enhancer	RP4-687K1.2
chr14	73675000	chr14	93115000	7.37E-03	promoter	super_enhancer	AC004846.1
chr14	73785000	chr14	90835000	9.58E-03	super_enhancer	promoter	RPL21P11
chr14	74225000	chr14	100695000	9.61E-03	promoter	super_enhancer	MIR4505
chr14	74485000	chr14	93095000	1.75E-02	promoter	super_enhancer	CCDC176
chr14	74485000	chr14	100695000	1.35E-02	promoter	super_enhancer	CCDC176
chr14	74555000	chr14	78265000	4.76E-02	super_enhancer	promoter	ADCK1
chr14	74555000	chr14	93375000	1.19E-02	super_enhancer	promoter	RP11-862G15.2

chr14	74555000	chr14	104845000	9.34E-03	super_enhancer	promoter	RP11-260M19.1
chr14	74565000	chr14	101405000	7.24E-03	super_enhancer	promoter	SNORD113-5
chr14	74575000	chr14	85995000	1.68E-02	super_enhancer	promoter	RP11-497E19.2
chr14	74575000	chr14	91885000	1.04E-02	super_enhancer	promoter	CCDC88C
chr14	74585000	chr14	75895000	1.68E-02	super_enhancer	promoter	RP11-293M10.6
chr14	74585000	chr14	77825000	4.87E-02	super_enhancer	promoter	RP11-493G17.4
chr14	74585000	chr14	105045000	7.90E-03	super_enhancer	promoter	C14orf180
chr14	74845000	chr14	102585000	1.14E-02	promoter	super_enhancer	RPS2P2
chr14	75125000	chr14	93065000	1.37E-02	promoter	super_enhancer	RP11-173A8.2
chr14	75595000	chr14	99575000	6.83E-03	promoter	super_enhancer	NEK9
chr14	75725000	chr14	102545000	8.27E-03	promoter	super_enhancer	RP11-293M10.2
chr14	75745000	chr14	93055000	1.52E-02	promoter	super_enhancer	FOS
chr14	75745000	chr14	93095000	1.69E-02	promoter	super_enhancer	FOS
chr14	75895000	chr14	100385000	9.76E-03	promoter	super_enhancer	RP11-293M10.6
chr14	76305000	chr14	100405000	9.14E-03	promoter	super_enhancer	RP11-270M14.1
chr14	76315000	chr14	99565000	8.75E-03	promoter	super_enhancer	RP11-270M14.4
chr14	76745000	chr14	76905000	4.40E-02	promoter	super_enhancer	RP11-361H10.5
chr14	76815000	chr14	93085000	1.11E-02	promoter	super_enhancer	AC016543.1
chr14	76815000	chr14	103125000	7.91E-03	promoter	super_enhancer	AC016543.1
chr14	76905000	chr14	91725000	1.79E-02	super_enhancer	promoter	CTD-2547L24.4
chr14	76915000	chr14	101515000	1.13E-02	super_enhancer	promoter	MIR544A
chr14	76925000	chr14	92335000	1.90E-02	super_enhancer	promoter	TC2N
chr14	76925000	chr14	100805000	1.46E-02	super_enhancer	promoter	RP11-638I2.9
chr14	76925000	chr14	103345000	1.15E-02	super_enhancer	promoter	AL117209.1

chr14	76935000	chr14	100705000	9.50E-03	super_enhancer	promoter	RP11-638I2.4
chr14	76945000	chr14	105715000	1.07E-02	super_enhancer	promoter	BTBD6
chr14	76955000	chr14	91295000	1.86E-02	super_enhancer	promoter	CTD-3035D6.2
chr14	76955000	chr14	107075000	7.80E-03	super_enhancer	promoter	IGHV3-57
chr14	76965000	chr14	88965000	1.72E-02	super_enhancer	promoter	RP11-507K2.2
chr14	76965000	chr14	101515000	1.15E-02	super_enhancer	promoter	MIR544A
chr14	76965000	chr14	106735000	9.68E-03	super_enhancer	promoter	IGHV1-24
chr14	76975000	chr14	100575000	1.05E-02	super_enhancer	promoter	MIR342
chr14	76975000	chr14	101395000	9.92E-03	super_enhancer	promoter	SNORD113-3
chr14	76975000	chr14	103745000	9.78E-03	super_enhancer	promoter	RAP2CP1
chr14	76975000	chr14	106735000	9.13E-03	super_enhancer	promoter	IGHV1-24
chr14	77245000	chr14	93115000	1.77E-02	promoter	super_enhancer	RP11-488C13.7
chr14	77245000	chr14	100415000	1.49E-02	promoter	super_enhancer	RP11-488C13.7
chr14	77245000	chr14	101725000	1.03E-02	promoter	super_enhancer	RP11-488C13.7
chr14	77245000	chr14	104705000	1.20E-02	promoter	super_enhancer	RP11-488C13.7
chr14	77425000	chr14	100695000	1.29E-02	promoter	super_enhancer	RP11-7F17.7
chr14	77425000	chr14	103075000	1.25E-02	promoter	super_enhancer	RP11-7F17.7
chr14	77425000	chr14	104675000	1.26E-02	promoter	super_enhancer	RP11-7F17.7
chr14	77425000	chr14	104975000	9.77E-03	promoter	super_enhancer	RP11-7F17.7
chr14	77825000	chr14	100445000	8.80E-03	promoter	super_enhancer	RP11-493G17.4
chr14	77825000	chr14	103075000	1.11E-02	promoter	super_enhancer	RP11-493G17.4
chr14	77915000	chr14	101715000	1.00E-02	promoter	super_enhancer	AF111168.1
chr14	77965000	chr14	100405000	1.41E-02	promoter	super_enhancer	ISM2
chr14	77965000	chr14	101725000	1.17E-02	promoter	super_enhancer	ISM2

chr14	77965000	chr14	102545000	1.21E-02	promoter	super_enhancer	ISM2
chr14	77965000	chr14	104685000	1.34E-02	promoter	super_enhancer	ISM2
chr14	78265000	chr14	102575000	1.10E-02	promoter	super_enhancer	ADCK1
chr14	78265000	chr14	102585000	1.27E-02	promoter	super_enhancer	ADCK1
chr14	78865000	chr14	103075000	8.63E-03	promoter	super_enhancer	AC007056.1
chr14	79715000	chr14	100395000	7.83E-03	promoter	super_enhancer	RP11-588P7.2
chr14	82245000	chr14	93065000	1.79E-04	promoter	super_enhancer	RP11-226P1.1
chr14	82245000	chr14	93105000	1.83E-02	promoter	super_enhancer	RP11-226P1.1
chr14	82245000	chr14	93145000	1.60E-02	promoter	super_enhancer	RP11-226P1.1
chr14	85995000	chr14	93015000	2.34E-02	promoter	super_enhancer	RP11-497E19.2
chr14	88035000	chr14	93115000	3.21E-02	promoter	super_enhancer	RP11-325D8.1
chr14	89865000	chr14	102545000	1.32E-02	promoter	super_enhancer	RP11-33N16.2
chr14	91295000	chr14	102525000	2.30E-02	promoter	super_enhancer	CTD-3035D6.2
chr14	91295000	chr14	102585000	2.45E-02	promoter	super_enhancer	CTD-3035D6.2
chr14	91725000	chr14	100405000	2.24E-02	promoter	super_enhancer	CTD-2547L24.4
chr14	91725000	chr14	100415000	2.68E-02	promoter	super_enhancer	CTD-2547L24.4
chr14	92335000	chr14	93125000	1.42E-02	promoter	super_enhancer	TC2N
chr14	92335000	chr14	100415000	2.58E-02	promoter	super_enhancer	TC2N
chr14	92335000	chr14	104965000	1.72E-02	promoter	super_enhancer	TC2N
chr14	92415000	chr14	100425000	1.88E-02	promoter	super_enhancer	FBLN5
chr14	92415000	chr14	100695000	1.89E-02	promoter	super_enhancer	FBLN5
chr14	92995000	chr14	100705000	2.32E-02	super_enhancer	promoter	RP11-638I2.4
chr14	92995000	chr14	101515000	2.38E-02	super_enhancer	promoter	MIR544A
chr14	92995000	chr14	106995000	1.29E-02	super_enhancer	promoter	IGHG1

chr14	93005000	chr14	101545000	1.71E-02	super_enhancer	promoter	AL132709.1
chr14	93015000	chr14	103565000	1.61E-02	super_enhancer	promoter	EXOC3L4
chr14	93015000	chr14	105955000	1.91E-02	super_enhancer	promoter	C14orf80
chr14	93025000	chr14	101295000	2.12E-02	super_enhancer	promoter	MEG3_2
chr14	93025000	chr14	105045000	1.97E-02	super_enhancer	promoter	C14orf180
chr14	93025000	chr14	105235000	2.17E-02	super_enhancer	promoter	RP11-982M15.2
chr14	93025000	chr14	107035000	1.29E-02	super_enhancer	promoter	IGHV5-51
chr14	93035000	chr14	99395000	2.22E-02	super_enhancer	promoter	RP11-1127D7.1
chr14	93035000	chr14	104265000	1.18E-02	super_enhancer	promoter	SNORD51
chr14	93035000	chr14	106385000	1.18E-02	super_enhancer	promoter	IGHD1-1
chr14	93035000	chr14	106775000	1.35E-02	super_enhancer	promoter	IGHV7-27
chr14	93045000	chr14	101525000	1.82E-02	super_enhancer	promoter	MIR154
chr14	93045000	chr14	106735000	1.85E-02	super_enhancer	promoter	IGHV1-24
chr14	93055000	chr14	104845000	2.03E-02	super_enhancer	promoter	RP11-260M19.1
chr14	93055000	chr14	105235000	2.04E-02	super_enhancer	promoter	RP11-982M15.2
chr14	93055000	chr14	106355000	1.64E-02	super_enhancer	promoter	IGHD2-21
chr14	93065000	chr14	107275000	1.32E-02	super_enhancer	promoter	IGHVII-78-1
chr14	93075000	chr14	103615000	1.75E-02	super_enhancer	promoter	RPL21P12
chr14	93075000	chr14	107095000	1.05E-02	super_enhancer	promoter	IGHVII-60-1
chr14	93085000	chr14	98445000	2.23E-02	super_enhancer	promoter	LINC01550
chr14	93085000	chr14	105715000	1.48E-02	super_enhancer	promoter	BTBD6
chr14	93095000	chr14	95795000	4.22E-03	super_enhancer	promoter	RP11-725G5.2
chr14	93095000	chr14	99395000	3.39E-02	super_enhancer	promoter	RP11-1127D7.1
chr14	93095000	chr14	101245000	2.82E-02	super_enhancer	promoter	MEG3

chr14	93095000	chr14	104605000	2.84E-02	super_enhancer	promoter	CTD-2223O18.1
chr14	93105000	chr14	103995000	1.90E-02	super_enhancer	promoter	RP11-600F24.7
chr14	93105000	chr14	104605000	2.72E-02	super_enhancer	promoter	CTD-2223O18.1
chr14	93125000	chr14	101545000	2.31E-02	super_enhancer	promoter	AL132709.1
chr14	93125000	chr14	106935000	1.25E-02	super_enhancer	promoter	IGHVIII-44
chr14	93135000	chr14	99395000	2.88E-02	super_enhancer	promoter	RP11-1127D7.1
chr14	93135000	chr14	106385000	1.51E-02	super_enhancer	promoter	IGHD1-1
chr14	93135000	chr14	107275000	1.12E-02	super_enhancer	promoter	IGHVII-78-1
chr14	93145000	chr14	105445000	1.59E-02	super_enhancer	promoter	AHNAK2
chr14	93215000	chr14	100385000	2.47E-02	promoter	super_enhancer	LGMN
chr14	94385000	chr14	99585000	3.02E-02	promoter	super_enhancer	FAM181A
chr14	94385000	chr14	102545000	2.08E-02	promoter	super_enhancer	FAM181A
chr14	94405000	chr14	100425000	3.74E-02	promoter	super_enhancer	RP11-131H24.4
chr14	94405000	chr14	104695000	3.35E-02	promoter	super_enhancer	RP11-131H24.4
chr14	95385000	chr14	100395000	3.35E-02	promoter	super_enhancer	RP11-179A9.3
chr14	95605000	chr14	100435000	2.61E-02	promoter	super_enhancer	MIR3173
chr14	95605000	chr14	101715000	2.27E-02	promoter	super_enhancer	MIR3173
chr14	95605000	chr14	103125000	1.56E-02	promoter	super_enhancer	MIR3173
chr14	95625000	chr14	100395000	2.70E-02	promoter	super_enhancer	DICER1
chr14	95665000	chr14	102545000	2.42E-02	promoter	super_enhancer	RP11-725G5.3
chr14	96485000	chr14	99585000	8.82E-04	promoter	super_enhancer	RP11-241N4.2
chr14	96485000	chr14	100385000	3.57E-02	promoter	super_enhancer	RP11-241N4.2
chr14	96485000	chr14	102545000	1.97E-02	promoter	super_enhancer	RP11-241N4.2
chr14	96735000	chr14	102545000	3.26E-02	promoter	super_enhancer	RP11-404P21.3

chr14	96735000	chr14	102555000	3.98E-02	promoter	super_enhancer	RP11-404P21.3
chr14	97105000	chr14	100445000	4.75E-02	promoter	super_enhancer	RN7SKP108
chr14	97925000	chr14	102575000	3.49E-02	promoter	super_enhancer	CTD-2506J14.1
chr14	98445000	chr14	99585000	2.26E-02	promoter	super_enhancer	LINC01550
chr14	98445000	chr14	104705000	2.69E-02	promoter	super_enhancer	LINC01550
chr14	99565000	chr14	105885000	3.53E-02	super_enhancer	promoter	MTA1
chr14	99565000	chr14	106975000	1.88E-02	super_enhancer	promoter	IGHV3-47
chr14	99575000	chr14	106745000	1.66E-02	super_enhancer	promoter	HOMER2P2
chr14	99575000	chr14	106775000	2.25E-02	super_enhancer	promoter	IGHV7-27
chr14	99575000	chr14	107095000	1.32E-02	super_enhancer	promoter	IGHVII-60-1
chr14	99585000	chr14	106445000	1.92E-02	super_enhancer	promoter	AB019441.29
chr14	99585000	chr14	106975000	1.69E-02	super_enhancer	promoter	IGHV3-47
chr14	99595000	chr14	106005000	2.16E-02	super_enhancer	promoter	ATP5G1P1
chr14	99595000	chr14	106975000	1.85E-02	super_enhancer	promoter	IGHV3-47
chr14	99595000	chr14	106995000	1.74E-02	super_enhancer	promoter	IGHG1
chr14	99595000	chr14	107115000	1.63E-02	super_enhancer	promoter	IGHV3-64
chr14	100385000	chr14	101515000	4.44E-02	super_enhancer	promoter	MIR544A
chr14	100385000	chr14	105155000	3.17E-02	super_enhancer	promoter	INF2
chr14	100385000	chr14	105445000	3.62E-02	super_enhancer	promoter	AHNAK2
chr14	100385000	chr14	105865000	2.67E-02	super_enhancer	promoter	TEX22
chr14	100385000	chr14	106975000	2.21E-02	super_enhancer	promoter	IGHV3-47
chr14	100395000	chr14	105145000	3.71E-02	super_enhancer	promoter	MIR4710
chr14	100405000	chr14	100705000	2.51E-02	super_enhancer	promoter	RP11-638I2.4
chr14	100405000	chr14	101525000	2.49E-02	super_enhancer	promoter	MIR154

chr14	100405000	chr14	107185000	2.17E-02	super_enhancer	promoter	IGHV3-71
chr14	100415000	chr14	105325000	2.71E-02	super_enhancer	promoter	CTD-3051D23.4
chr14	100425000	chr14	101525000	3.01E-02	super_enhancer	promoter	MIR154
chr14	100425000	chr14	105145000	4.20E-02	super_enhancer	promoter	MIR4710
chr14	100425000	chr14	105155000	3.36E-02	super_enhancer	promoter	INF2
chr14	100425000	chr14	106005000	2.79E-02	super_enhancer	promoter	ATP5G1P1
chr14	100425000	chr14	106715000	2.49E-02	super_enhancer	promoter	IGHV3-22
chr14	100435000	chr14	101685000	3.66E-02	super_enhancer	promoter	RP11-8L8.1
chr14	100435000	chr14	105635000	3.45E-02	super_enhancer	promoter	JAG2
chr14	100445000	chr14	100575000	2.49E-02	super_enhancer	promoter	MIR342
chr14	100445000	chr14	101515000	3.59E-02	super_enhancer	promoter	MIR544A
chr14	100445000	chr14	104845000	4.10E-02	super_enhancer	promoter	RP11-260M19.1
chr14	100445000	chr14	105145000	3.34E-02	super_enhancer	promoter	MIR4710
chr14	100695000	chr14	105865000	3.07E-02	super_enhancer	promoter	TEX22
chr14	100695000	chr14	106385000	3.45E-02	super_enhancer	promoter	IGHD1-1
chr14	100695000	chr14	106935000	2.32E-02	super_enhancer	promoter	IGHVIII-44
chr14	101055000	chr14	102585000	1.24E-02	promoter	super_enhancer	BEGAIN
chr14	101245000	chr14	102555000	5.03E-03	promoter	super_enhancer	MEG3
chr14	101295000	chr14	102525000	3.41E-02	promoter	super_enhancer	MEG3_2
chr14	101365000	chr14	102555000	3.14E-02	promoter	super_enhancer	MEG8_2
chr14	101395000	chr14	101715000	2.15E-02	promoter	super_enhancer	SNORD113-3
chr14	101395000	chr14	102555000	4.20E-02	promoter	super_enhancer	SNORD113-3
chr14	101395000	chr14	102585000	4.29E-02	promoter	super_enhancer	SNORD113-3
chr14	101415000	chr14	101715000	2.09E-03	promoter	super_enhancer	SNORD114-1

chr14	101505000	chr14	101715000	4.51E-02	promoter	super_enhancer	AL132709.3
chr14	101505000	chr14	101725000	2.77E-02	promoter	super_enhancer	AL132709.3
chr14	101525000	chr14	101715000	3.34E-02	promoter	super_enhancer	MIR154
chr14	101525000	chr14	102555000	3.92E-03	promoter	super_enhancer	MIR154
chr14	101525000	chr14	102575000	3.15E-02	promoter	super_enhancer	MIR154
chr14	101545000	chr14	101715000	7.65E-03	promoter	super_enhancer	AL132709.1
chr14	101725000	chr14	101875000	2.80E-03	super_enhancer	promoter	LINC00524
chr14	102525000	chr14	107275000	3.25E-02	super_enhancer	promoter	IGHVII-78-1
chr14	102535000	chr14	104265000	9.98E-03	super_enhancer	promoter	SNORD51
chr14	102555000	chr14	106825000	3.51E-02	super_enhancer	promoter	IGHVII-33-1
chr14	103005000	chr14	103155000	2.83E-02	promoter	super_enhancer	MIR4309
chr14	103075000	chr14	103345000	1.18E-02	super_enhancer	promoter	AL117209.1
chr14	103155000	chr14	107115000	3.38E-02	super_enhancer	promoter	IGHV3-64
chr14	103995000	chr14	104695000	4.94E-02	promoter	super_enhancer	RP11-600F24.7
chr14	104685000	chr14	105955000	4.87E-02	super_enhancer	promoter	C14orf80
chr14	104845000	chr14	104975000	2.73E-02	promoter	super_enhancer	RP11-260M19.1
chr14	104965000	chr14	106575000	8.12E-03	super_enhancer	promoter	IGHV3-11
chr15	20175000	chr15	67155000	2.95E-03	promoter	super_enhancer	SLC20A1P3
chr15	20845000	chr15	75045000	4.52E-03	promoter	super_enhancer	IGHV1OR15-6
chr15	22545000	chr15	90855000	4.06E-03	promoter	super_enhancer	RP11-603B24.2
chr15	23115000	chr15	67085000	4.44E-03	promoter	super_enhancer	RP11-26F2.2
chr15	23115000	chr15	88625000	2.07E-03	promoter	super_enhancer	RP11-26F2.2
chr15	25335000	chr15	74195000	3.81E-03	promoter	super_enhancer	SNORD116-22
chr15	25475000	chr15	59305000	7.05E-03	promoter	super_enhancer	SNORD115-33

chr15	25495000	chr15	74395000	2.21E-03	promoter	super_enhancer	SNORD115-44
chr15	26095000	chr15	66725000	3.42E-03	promoter	super_enhancer	MIR4715
chr15	26175000	chr15	60385000	5.55E-03	promoter	super_enhancer	RP11-446P9.1
chr15	26905000	chr15	98885000	2.50E-03	promoter	super_enhancer	AC011196.3
chr15	27185000	chr15	74405000	3.14E-03	promoter	super_enhancer	GABRB3
chr15	28345000	chr15	67125000	6.39E-03	promoter	super_enhancer	OCA2
chr15	29905000	chr15	40345000	1.90E-02	promoter	super_enhancer	TUBBP8
chr15	30115000	chr15	67025000	5.64E-03	promoter	super_enhancer	RP11-680F8.3
chr15	31195000	chr15	59305000	6.67E-03	promoter	super_enhancer	FAN1
chr15	31285000	chr15	67125000	5.92E-03	promoter	super_enhancer	MTMR10
chr15	32965000	chr15	70475000	5.82E-03	promoter	super_enhancer	RP11-1000B6.2
chr15	34265000	chr15	98905000	2.63E-03	promoter	super_enhancer	RP11-74D7.3
chr15	34635000	chr15	60415000	9.49E-03	promoter	super_enhancer	NOP10
chr15	34635000	chr15	75045000	7.25E-03	promoter	super_enhancer	NOP10
chr15	35235000	chr15	67025000	5.74E-03	promoter	super_enhancer	RP11-83J16.1
chr15	35235000	chr15	67035000	5.58E-03	promoter	super_enhancer	RP11-83J16.1
chr15	35385000	chr15	72225000	3.82E-03	promoter	super_enhancer	RP11-323I15.2
chr15	35415000	chr15	40345000	3.31E-02	promoter	super_enhancer	PRELID1P4
chr15	35575000	chr15	88645000	2.90E-03	promoter	super_enhancer	NUTF2P6
chr15	36545000	chr15	41945000	2.29E-02	promoter	super_enhancer	RP11-184D12.1
chr15	36905000	chr15	67175000	7.10E-03	promoter	super_enhancer	RP11-618N24.1
chr15	36905000	chr15	74195000	5.57E-03	promoter	super_enhancer	RP11-618N24.1
chr15	37395000	chr15	98855000	2.32E-03	promoter	super_enhancer	MEIS2
chr15	38365000	chr15	67175000	8.44E-03	promoter	super_enhancer	RP11-1008C21.2

chr15	40075000	chr15	45455000	2.73E-02	promoter	super_enhancer	FSIP1
chr15	40075000	chr15	59295000	1.08E-02	promoter	super_enhancer	FSIP1
chr15	40095000	chr15	75675000	5.78E-03	promoter	super_enhancer	RP11-37C7.1
chr15	40225000	chr15	45455000	2.50E-02	promoter	super_enhancer	EIF2AK4
chr15	40245000	chr15	63495000	6.71E-03	promoter	super_enhancer	H3F3AP1
chr15	40245000	chr15	67035000	9.81E-03	promoter	super_enhancer	H3F3AP1
chr15	40245000	chr15	69055000	7.68E-03	promoter	super_enhancer	H3F3AP1
chr15	40335000	chr15	80635000	4.07E-03	super_enhancer	promoter	LINC00927
chr15	40345000	chr15	40545000	3.10E-02	super_enhancer	promoter	C15orf56
chr15	40345000	chr15	52485000	1.85E-02	super_enhancer	promoter	GNB5
chr15	40345000	chr15	56535000	1.12E-02	super_enhancer	promoter	RFX7
chr15	40345000	chr15	76005000	6.19E-03	super_enhancer	promoter	CSPG4
chr15	40345000	chr15	92875000	5.12E-03	super_enhancer	promoter	RP11-152L20.3
chr15	40545000	chr15	67125000	9.81E-03	promoter	super_enhancer	C15orf56
chr15	40545000	chr15	90875000	5.32E-03	promoter	super_enhancer	C15orf56
chr15	40605000	chr15	67025000	1.09E-02	promoter	super_enhancer	RP11-133K1.1
chr15	40605000	chr15	75655000	1.07E-02	promoter	super_enhancer	RP11-133K1.1
chr15	40675000	chr15	67075000	1.08E-02	promoter	super_enhancer	KNSTRN
chr15	40775000	chr15	60425000	1.12E-02	promoter	super_enhancer	RP11-64K12.1
chr15	40775000	chr15	70495000	7.97E-03	promoter	super_enhancer	RP11-64K12.1
chr15	40775000	chr15	90215000	6.16E-03	promoter	super_enhancer	RP11-64K12.1
chr15	41135000	chr15	74425000	1.04E-02	promoter	super_enhancer	SPINT1
chr15	41245000	chr15	66725000	1.17E-02	promoter	super_enhancer	CHAC1
chr15	41575000	chr15	41935000	3.75E-02	promoter	super_enhancer	OIP5-AS1

chr15	41635000	chr15	66725000	1.18E-02	promoter	super_enhancer	RP11-16O9.2
chr15	41635000	chr15	66735000	1.23E-02	promoter	super_enhancer	RP11-16O9.2
chr15	41635000	chr15	75655000	1.24E-02	promoter	super_enhancer	RP11-16O9.2
chr15	41695000	chr15	60415000	1.52E-02	promoter	super_enhancer	NDUFAF1
chr15	41695000	chr15	70485000	1.05E-02	promoter	super_enhancer	NDUFAF1
chr15	41905000	chr15	63495000	7.32E-03	promoter	super_enhancer	RP11-380D11.2
chr15	41905000	chr15	67105000	9.45E-03	promoter	super_enhancer	RP11-380D11.2
chr15	41925000	chr15	45725000	3.64E-02	super_enhancer	promoter	MIR147B
chr15	41925000	chr15	64925000	9.05E-03	super_enhancer	promoter	RP11-330L19.2
chr15	41925000	chr15	66995000	8.63E-03	super_enhancer	promoter	SMAD6
chr15	41925000	chr15	69315000	8.04E-03	super_enhancer	promoter	RP11-809H16.3
chr15	41925000	chr15	77285000	5.81E-03	super_enhancer	promoter	PSTPIP1
chr15	41925000	chr15	78775000	5.30E-03	super_enhancer	promoter	RP11-650L12.1
chr15	41925000	chr15	101325000	3.87E-03	super_enhancer	promoter	AC087762.1
chr15	41935000	chr15	42875000	4.74E-02	super_enhancer	promoter	EIF4EBP2P2
chr15	41935000	chr15	45725000	3.94E-02	super_enhancer	promoter	MIR147B
chr15	41935000	chr15	51035000	1.67E-02	super_enhancer	promoter	RP11-507J18.5
chr15	41935000	chr15	65245000	4.85E-03	super_enhancer	promoter	CTD-2017F17.2
chr15	41935000	chr15	86315000	5.81E-03	super_enhancer	promoter	MIR1276
chr15	41935000	chr15	93855000	3.54E-03	super_enhancer	promoter	RP11-266O8.1
chr15	41945000	chr15	63675000	1.45E-02	super_enhancer	promoter	CA12
chr15	41945000	chr15	63795000	1.08E-02	super_enhancer	promoter	AC007950.2
chr15	41945000	chr15	65055000	1.42E-02	super_enhancer	promoter	MIR1272
chr15	41945000	chr15	72335000	8.61E-03	super_enhancer	promoter	EIF5A2P1

chr15	41945000	chr15	75505000	8.45E-03	super_enhancer	promoter	RP11-69H7.3
chr15	42185000	chr15	67025000	1.21E-02	promoter	super_enhancer	RP11-23P13.6
chr15	42185000	chr15	92825000	4.59E-03	promoter	super_enhancer	RP11-23P13.6
chr15	42265000	chr15	70475000	9.38E-03	promoter	super_enhancer	EHD4
chr15	42265000	chr15	74195000	7.94E-03	promoter	super_enhancer	EHD4
chr15	42565000	chr15	75065000	8.08E-03	promoter	super_enhancer	GANC
chr15	42565000	chr15	91945000	3.67E-03	promoter	super_enhancer	GANC
chr15	42605000	chr15	90195000	3.96E-03	promoter	super_enhancer	BNIP3P5
chr15	42825000	chr15	59295000	1.38E-02	promoter	super_enhancer	RP11-265N6.1
chr15	42825000	chr15	75065000	9.32E-03	promoter	super_enhancer	RP11-265N6.1
chr15	42825000	chr15	89115000	5.90E-03	promoter	super_enhancer	RP11-265N6.1
chr15	42875000	chr15	75665000	8.03E-03	promoter	super_enhancer	EIF4EBP2P2
chr15	43415000	chr15	60415000	1.31E-02	promoter	super_enhancer	TMEM62
chr15	43415000	chr15	75035000	7.57E-03	promoter	super_enhancer	TMEM62
chr15	43595000	chr15	51845000	2.21E-02	promoter	super_enhancer	TGM7
chr15	43595000	chr15	75665000	9.50E-03	promoter	super_enhancer	TGM7
chr15	43595000	chr15	90165000	6.09E-03	promoter	super_enhancer	TGM7
chr15	43685000	chr15	60405000	1.01E-02	promoter	super_enhancer	RN7SL487P
chr15	43685000	chr15	66725000	9.47E-03	promoter	super_enhancer	RN7SL487P
chr15	43685000	chr15	88625000	4.09E-03	promoter	super_enhancer	RN7SL487P
chr15	43685000	chr15	90195000	4.81E-03	promoter	super_enhancer	RN7SL487P
chr15	43935000	chr15	66785000	4.86E-03	promoter	super_enhancer	AC011330.13
chr15	44075000	chr15	63475000	1.19E-02	promoter	super_enhancer	AC018512.1
chr15	44075000	chr15	67055000	1.15E-02	promoter	super_enhancer	AC018512.1

chr15	44075000	chr15	75055000	9.93E-03	promoter	super_enhancer	AC018512.1
chr15	44355000	chr15	60415000	1.39E-02	promoter	super_enhancer	GAPDHP55
chr15	45005000	chr15	88625000	4.96E-03	promoter	super_enhancer	PATL2
chr15	45265000	chr15	74205000	5.79E-03	promoter	super_enhancer	RNU6-966P
chr15	45265000	chr15	90225000	4.14E-03	promoter	super_enhancer	RNU6-966P
chr15	45295000	chr15	45455000	4.37E-02	promoter	super_enhancer	RNU1-78P
chr15	45295000	chr15	92815000	3.67E-03	promoter	super_enhancer	RNU1-78P
chr15	45455000	chr15	45695000	2.49E-02	super_enhancer	promoter	GATM
chr15	45455000	chr15	45735000	7.29E-03	super_enhancer	promoter	RP11-519G16.5
chr15	45455000	chr15	45815000	1.32E-02	super_enhancer	promoter	SLC30A4
chr15	45455000	chr15	52045000	2.68E-02	super_enhancer	promoter	LYSMD2
chr15	45455000	chr15	71145000	1.03E-02	super_enhancer	promoter	LRRC49
chr15	45455000	chr15	76605000	9.25E-03	super_enhancer	promoter	ETFA
chr15	45455000	chr15	88795000	5.92E-03	super_enhancer	promoter	NTRK3-AS1
chr15	45455000	chr15	89745000	6.05E-03	super_enhancer	promoter	RP11-326A19.5
chr15	45615000	chr15	67145000	9.05E-03	promoter	super_enhancer	CTD-2651B20.5
chr15	45695000	chr15	59265000	1.46E-02	promoter	super_enhancer	GATM
chr15	45695000	chr15	66735000	1.03E-02	promoter	super_enhancer	GATM
chr15	45695000	chr15	75665000	7.76E-03	promoter	super_enhancer	GATM
chr15	45725000	chr15	60415000	1.46E-02	promoter	super_enhancer	MIR147B
chr15	45725000	chr15	72235000	5.93E-03	promoter	super_enhancer	MIR147B
chr15	45735000	chr15	59275000	1.51E-02	promoter	super_enhancer	RP11-519G16.5
chr15	45735000	chr15	67095000	1.20E-02	promoter	super_enhancer	RP11-519G16.5
chr15	45815000	chr15	74425000	1.21E-02	promoter	super_enhancer	SLC30A4

chr15	45995000	chr15	59265000	1.54E-02	promoter	super_enhancer	CTD-2306A12.1
chr15	46985000	chr15	63505000	7.08E-03	promoter	super_enhancer	RP11-627D16.1
chr15	47565000	chr15	59275000	9.75E-03	promoter	super_enhancer	RN7SKP139
chr15	48235000	chr15	92825000	4.32E-03	promoter	super_enhancer	CTD-2007H18.1
chr15	48495000	chr15	67055000	1.14E-02	promoter	super_enhancer	RP11-605F22.1
chr15	48625000	chr15	66725000	9.42E-03	promoter	super_enhancer	RP11-154J22.1
chr15	48955000	chr15	74205000	7.71E-03	promoter	super_enhancer	RP11-227D13.5
chr15	49075000	chr15	60375000	1.27E-02	promoter	super_enhancer	RP11-485O10.2
chr15	49075000	chr15	75665000	7.36E-03	promoter	super_enhancer	RP11-485O10.2
chr15	49335000	chr15	75065000	8.44E-03	promoter	super_enhancer	RN7SL577P
chr15	49335000	chr15	90865000	4.40E-03	promoter	super_enhancer	RN7SL577P
chr15	49635000	chr15	67165000	8.07E-03	promoter	super_enhancer	RP11-295H24.4
chr15	49715000	chr15	75665000	8.66E-03	promoter	super_enhancer	FGF7
chr15	50755000	chr15	70485000	1.19E-02	promoter	super_enhancer	RNA5SP395
chr15	51035000	chr15	63495000	9.68E-03	promoter	super_enhancer	RP11-507J18.5
chr15	51035000	chr15	100705000	2.98E-03	promoter	super_enhancer	RP11-507J18.5
chr15	51605000	chr15	74425000	1.43E-02	promoter	super_enhancer	MIR7973-2
chr15	51605000	chr15	75655000	1.31E-02	promoter	super_enhancer	MIR7973-2
chr15	51705000	chr15	59305000	1.60E-02	promoter	super_enhancer	RP11-707P17.1
chr15	51835000	chr15	80955000	6.61E-03	super_enhancer	promoter	RNU6-380P
chr15	51835000	chr15	81935000	4.81E-03	super_enhancer	promoter	RP11-499F3.1
chr15	51845000	chr15	64995000	1.42E-02	super_enhancer	promoter	OAZ2
chr15	51915000	chr15	74185000	9.83E-03	promoter	super_enhancer	DMXL2
chr15	52325000	chr15	67175000	1.47E-02	promoter	super_enhancer	RP11-430B1.1

chr15	52325000	chr15	74405000	9.77E-03	promoter	super_enhancer	RP11-430B1.1
chr15	52325000	chr15	90165000	6.36E-03	promoter	super_enhancer	RP11-430B1.1
chr15	52405000	chr15	59295000	2.02E-02	promoter	super_enhancer	BCL2L10
chr15	52485000	chr15	59285000	2.46E-02	promoter	super_enhancer	GNB5
chr15	52485000	chr15	67085000	1.57E-02	promoter	super_enhancer	GNB5
chr15	54305000	chr15	67165000	1.08E-02	promoter	super_enhancer	UNC13C
chr15	54615000	chr15	98865000	3.10E-03	promoter	super_enhancer	HNRNPA1P74
chr15	55665000	chr15	59295000	4.70E-02	promoter	super_enhancer	MIR628
chr15	55665000	chr15	69045000	1.49E-02	promoter	super_enhancer	MIR628
chr15	56535000	chr15	90165000	5.39E-03	promoter	super_enhancer	RFX7
chr15	56535000	chr15	92825000	4.18E-03	promoter	super_enhancer	RFX7
chr15	56615000	chr15	74405000	8.84E-03	promoter	super_enhancer	RP11-844G16.1
chr15	58445000	chr15	63505000	2.64E-02	promoter	super_enhancer	RP11-925D8.5
chr15	59185000	chr15	66785000	1.64E-02	promoter	super_enhancer	AC025918.1
chr15	59185000	chr15	75665000	1.22E-02	promoter	super_enhancer	AC025918.1
chr15	59185000	chr15	75675000	1.11E-02	promoter	super_enhancer	AC025918.1
chr15	59255000	chr15	59845000	3.38E-03	super_enhancer	promoter	RP11-112N19.2
chr15	59255000	chr15	64925000	3.27E-02	super_enhancer	promoter	RP11-330L19.2
chr15	59255000	chr15	65715000	2.84E-02	super_enhancer	promoter	IGDCC4
chr15	59255000	chr15	85175000	1.01E-02	super_enhancer	promoter	SCAND2P
chr15	59255000	chr15	86685000	9.72E-03	super_enhancer	promoter	AGBL1
chr15	59255000	chr15	98065000	6.71E-03	super_enhancer	promoter	CTD-2147F2.2
chr15	59265000	chr15	63245000	4.26E-02	super_enhancer	promoter	RP11-244F12.1
chr15	59265000	chr15	65055000	3.93E-02	super_enhancer	promoter	MIR1272

chr15	59265000	chr15	72565000	1.82E-02	super_enhancer	promoter	PARP6
chr15	59265000	chr15	75505000	1.58E-02	super_enhancer	promoter	RP11-69H7.3
chr15	59275000	chr15	68125000	1.72E-02	super_enhancer	promoter	RP11-34F13.3
chr15	59275000	chr15	69365000	1.76E-02	super_enhancer	promoter	EWSAT1
chr15	59275000	chr15	78285000	1.02E-02	super_enhancer	promoter	RP11-114H24.6
chr15	59275000	chr15	82555000	7.70E-03	super_enhancer	promoter	EFTUD1
chr15	59275000	chr15	89745000	8.69E-03	super_enhancer	promoter	RP11-326A19.5
chr15	59275000	chr15	93015000	6.68E-03	super_enhancer	promoter	RP11-763K15.1
chr15	59275000	chr15	98065000	5.63E-03	super_enhancer	promoter	CTD-2147F2.2
chr15	59285000	chr15	65245000	1.47E-02	super_enhancer	promoter	CTD-2017F17.2
chr15	59285000	chr15	67835000	2.09E-02	super_enhancer	promoter	RP11-502I4.3
chr15	59285000	chr15	69755000	1.82E-02	super_enhancer	promoter	RP11-253M7.4
chr15	59285000	chr15	70115000	1.70E-02	super_enhancer	promoter	RP11-19J5.2
chr15	59285000	chr15	74165000	1.58E-02	super_enhancer	promoter	TBC1D21
chr15	59285000	chr15	74465000	1.75E-02	super_enhancer	promoter	ISLR
chr15	59295000	chr15	64885000	3.07E-02	super_enhancer	promoter	RP11-330L19.1
chr15	59295000	chr15	65055000	3.64E-02	super_enhancer	promoter	MIR1272
chr15	59295000	chr15	74705000	1.63E-02	super_enhancer	promoter	MIR6881
chr15	59295000	chr15	74755000	1.52E-02	super_enhancer	promoter	UBL7
chr15	59305000	chr15	63675000	4.58E-02	super_enhancer	promoter	CA12
chr15	59305000	chr15	65505000	2.39E-02	super_enhancer	promoter	CILP
chr15	59305000	chr15	65715000	2.45E-02	super_enhancer	promoter	IGDCC4
chr15	59305000	chr15	80305000	5.27E-05	super_enhancer	promoter	RP11-758M2.1
chr15	59305000	chr15	85295000	1.05E-02	super_enhancer	promoter	RP11-7M10.2

chr15	59505000	chr15	67025000	2.01E-02	promoter	super_enhancer	RNU4-80P
chr15	59505000	chr15	92825000	5.29E-03	promoter	super_enhancer	RNU4-80P
chr15	59635000	chr15	63505000	4.34E-02	promoter	super_enhancer	RNU6-212P
chr15	59635000	chr15	67135000	2.11E-02	promoter	super_enhancer	RNU6-212P
chr15	59695000	chr15	66755000	2.93E-02	promoter	super_enhancer	RP11-356M20.2
chr15	59695000	chr15	74185000	1.99E-02	promoter	super_enhancer	RP11-356M20.2
chr15	59695000	chr15	74395000	8.91E-03	promoter	super_enhancer	RP11-356M20.2
chr15	59695000	chr15	100725000	7.47E-03	promoter	super_enhancer	RP11-356M20.2
chr15	59845000	chr15	66785000	1.84E-02	promoter	super_enhancer	RP11-112N19.2
chr15	59845000	chr15	88635000	5.91E-03	promoter	super_enhancer	RP11-112N19.2
chr15	60295000	chr15	70495000	1.66E-02	promoter	super_enhancer	FOXB1
chr15	60295000	chr15	71285000	1.48E-02	promoter	super_enhancer	FOXB1
chr15	60295000	chr15	98855000	4.39E-03	promoter	super_enhancer	FOXB1
chr15	60365000	chr15	69315000	2.23E-02	super_enhancer	promoter	RP11-809H16.3
chr15	60365000	chr15	69395000	1.94E-02	super_enhancer	promoter	AC027088.1
chr15	60365000	chr15	73975000	1.91E-02	super_enhancer	promoter	CD276
chr15	60365000	chr15	76015000	1.85E-02	super_enhancer	promoter	ODF3L1
chr15	60365000	chr15	94425000	5.42E-03	super_enhancer	promoter	RP11-76E17.4
chr15	60375000	chr15	64925000	3.71E-02	super_enhancer	promoter	RP11-330L19.2
chr15	60375000	chr15	80215000	1.34E-02	super_enhancer	promoter	RP11-81A1.6
chr15	60375000	chr15	80545000	1.14E-02	super_enhancer	promoter	LINC01314
chr15	60375000	chr15	83595000	1.04E-02	super_enhancer	promoter	RP11-382A20.5
chr15	60375000	chr15	90295000	1.01E-02	super_enhancer	promoter	MESP1
chr15	60375000	chr15	98535000	5.83E-03	super_enhancer	promoter	RP11-753A21.1

chr15	60385000	chr15	64455000	3.60E-02	super_enhancer	promoter	PPIB
chr15	60385000	chr15	70115000	1.72E-02	super_enhancer	promoter	RP11-19J5.2
chr15	60385000	chr15	72335000	1.63E-02	super_enhancer	promoter	EIF5A2P1
chr15	60385000	chr15	74495000	1.55E-02	super_enhancer	promoter	RP11-60L3.1
chr15	60395000	chr15	63895000	2.39E-03	super_enhancer	promoter	USP3-AS1
chr15	60395000	chr15	84285000	1.03E-02	super_enhancer	promoter	AC087738.1
chr15	60395000	chr15	98675000	6.53E-03	super_enhancer	promoter	RP11-183E24.2
chr15	60405000	chr15	78915000	1.12E-02	super_enhancer	promoter	CHRNA3
chr15	60405000	chr15	90295000	8.49E-03	super_enhancer	promoter	MESP1
chr15	60415000	chr15	69125000	2.48E-02	super_enhancer	promoter	RP11-696L21.2
chr15	60415000	chr15	84495000	8.14E-03	super_enhancer	promoter	RNU6-1339P
chr15	60415000	chr15	92875000	8.45E-03	super_enhancer	promoter	RP11-152L20.3
chr15	60425000	chr15	85295000	1.10E-02	super_enhancer	promoter	RP11-7M10.2
chr15	60425000	chr15	96885000	6.39E-03	super_enhancer	promoter	RP11-262A16.1
chr15	62035000	chr15	71275000	1.02E-02	promoter	super_enhancer	AC018618.1
chr15	62525000	chr15	66715000	4.38E-02	promoter	super_enhancer	RP11-1O10.1
chr15	62565000	chr15	66735000	3.28E-02	promoter	super_enhancer	RP11-299H22.6
chr15	62635000	chr15	67155000	4.68E-02	promoter	super_enhancer	MIR6085
chr15	62635000	chr15	70495000	2.27E-02	promoter	super_enhancer	MIR6085
chr15	62635000	chr15	72225000	1.36E-02	promoter	super_enhancer	MIR6085
chr15	62655000	chr15	63475000	1.02E-02	promoter	super_enhancer	HMGN1P26
chr15	63245000	chr15	71295000	1.62E-02	promoter	super_enhancer	RP11-244F12.1
chr15	63335000	chr15	70485000	2.99E-02	promoter	super_enhancer	TPM1
chr15	63335000	chr15	90875000	1.14E-02	promoter	super_enhancer	TPM1

chr15	63475000	chr15	71095000	2.08E-02	super_enhancer	promoter	RPL29P30
chr15	63475000	chr15	76195000	1.31E-02	super_enhancer	promoter	FBXO22
chr15	63475000	chr15	89765000	1.03E-02	super_enhancer	promoter	RLBP1
chr15	63485000	chr15	68725000	3.48E-02	super_enhancer	promoter	ITGA11
chr15	63485000	chr15	73455000	1.27E-02	super_enhancer	promoter	NPM1P43
chr15	63485000	chr15	90625000	8.35E-03	super_enhancer	promoter	RP11-617F23.1
chr15	63485000	chr15	95975000	5.71E-03	super_enhancer	promoter	LINC00924
chr15	63495000	chr15	74165000	1.20E-02	super_enhancer	promoter	TBC1D21
chr15	63495000	chr15	75105000	1.32E-02	super_enhancer	promoter	LMAN1L
chr15	63495000	chr15	76555000	7.93E-03	super_enhancer	promoter	TYRO3P
chr15	63495000	chr15	80215000	4.96E-05	super_enhancer	promoter	RP11-81A1.6
chr15	63505000	chr15	83225000	1.03E-02	super_enhancer	promoter	RP11-152F13.10
chr15	63505000	chr15	90645000	1.00E-02	super_enhancer	promoter	CTD-2315E11.1
chr15	63515000	chr15	73075000	2.17E-02	super_enhancer	promoter	ADPGK-AS1
chr15	63515000	chr15	76635000	1.39E-02	super_enhancer	promoter	RP11-685G9.2
chr15	64365000	chr15	90215000	1.25E-02	promoter	super_enhancer	DAPK2
chr15	64885000	chr15	70475000	3.37E-02	promoter	super_enhancer	RP11-330L19.1
chr15	64885000	chr15	90195000	1.03E-02	promoter	super_enhancer	RP11-330L19.1
chr15	64925000	chr15	69045000	4.22E-02	promoter	super_enhancer	RP11-330L19.2
chr15	64925000	chr15	90855000	8.25E-03	promoter	super_enhancer	RP11-330L19.2
chr15	65715000	chr15	67075000	2.17E-03	promoter	super_enhancer	IGDCC4
chr15	65905000	chr15	66755000	1.05E-02	promoter	super_enhancer	VWA9
chr15	66065000	chr15	67025000	5.27E-03	promoter	super_enhancer	RP11-16E23.4
chr15	66085000	chr15	66735000	2.31E-02	promoter	super_enhancer	DENND4A

chr15	66085000	chr15	66745000	7.08E-03	promoter	super_enhancer	DENND4A
chr15	66585000	chr15	67075000	6.07E-03	promoter	super_enhancer	DIS3L
chr15	66585000	chr15	67105000	2.03E-02	promoter	super_enhancer	DIS3L
chr15	66585000	chr15	75045000	2.30E-02	promoter	super_enhancer	DIS3L
chr15	66585000	chr15	90225000	9.05E-03	promoter	super_enhancer	DIS3L
chr15	66585000	chr15	98885000	6.88E-03	promoter	super_enhancer	DIS3L
chr15	66715000	chr15	67815000	2.75E-02	super_enhancer	promoter	IQCH-AS1
chr15	66715000	chr15	74595000	2.39E-02	super_enhancer	promoter	RP11-60L3.2
chr15	66715000	chr15	78635000	2.10E-02	super_enhancer	promoter	RP11-5O23.2
chr15	66725000	chr15	72065000	3.24E-02	super_enhancer	promoter	AC104938.1
chr15	66725000	chr15	72335000	2.96E-02	super_enhancer	promoter	EIF5A2P1
chr15	66725000	chr15	80215000	1.80E-02	super_enhancer	promoter	RP11-81A1.6
chr15	66735000	chr15	68125000	2.00E-02	super_enhancer	promoter	RP11-34F13.3
chr15	66735000	chr15	72565000	3.24E-02	super_enhancer	promoter	PARP6
chr15	66735000	chr15	94405000	7.26E-03	super_enhancer	promoter	RP11-76E17.3
chr15	66745000	chr15	72065000	2.22E-02	super_enhancer	promoter	AC104938.1
chr15	66745000	chr15	72335000	2.03E-02	super_enhancer	promoter	EIF5A2P1
chr15	66745000	chr15	76055000	1.31E-02	super_enhancer	promoter	RP11-24M17.6
chr15	66745000	chr15	89495000	7.97E-03	super_enhancer	promoter	RP11-63A23.2
chr15	66755000	chr15	75105000	2.72E-02	super_enhancer	promoter	LMAN1L
chr15	66755000	chr15	78885000	1.87E-02	super_enhancer	promoter	RP11-650L12.2
chr15	66755000	chr15	90295000	1.40E-02	super_enhancer	promoter	MESP1
chr15	66765000	chr15	67835000	3.86E-02	super_enhancer	promoter	RP11-502I4.3
chr15	66765000	chr15	75595000	1.62E-02	super_enhancer	promoter	DNM1P34

chr15	66765000	chr15	75805000	2.08E-02	super_enhancer	promoter	CTD-2323K18.2
chr15	66765000	chr15	84495000	9.16E-03	super_enhancer	promoter	RNU6-1339P
chr15	66765000	chr15	89165000	1.17E-02	super_enhancer	promoter	AEN
chr15	66775000	chr15	72065000	3.67E-02	super_enhancer	promoter	AC104938.1
chr15	66775000	chr15	78385000	2.11E-02	super_enhancer	promoter	SNORA63
chr15	66775000	chr15	94775000	8.82E-03	super_enhancer	promoter	MCTP2
chr15	66785000	chr15	71635000	3.08E-02	super_enhancer	promoter	RP11-592N21.1
chr15	66785000	chr15	83595000	1.21E-02	super_enhancer	promoter	RP11-382A20.5
chr15	66875000	chr15	67075000	4.15E-02	promoter	super_enhancer	LINC01169
chr15	66875000	chr15	67115000	7.78E-04	promoter	super_enhancer	LINC01169
chr15	66875000	chr15	67155000	1.14E-03	promoter	super_enhancer	LINC01169
chr15	66875000	chr15	67175000	2.87E-03	promoter	super_enhancer	LINC01169
chr15	66875000	chr15	67185000	6.47E-03	promoter	super_enhancer	LINC01169
chr15	66995000	chr15	67025000	8.17E-04	promoter	super_enhancer	SMAD6
chr15	66995000	chr15	67045000	3.46E-02	promoter	super_enhancer	SMAD6
chr15	66995000	chr15	67055000	4.21E-02	promoter	super_enhancer	SMAD6
chr15	66995000	chr15	67155000	4.94E-02	promoter	super_enhancer	SMAD6
chr15	66995000	chr15	75045000	2.90E-02	promoter	super_enhancer	SMAD6
chr15	67025000	chr15	74505000	2.72E-02	super_enhancer	promoter	STRA6
chr15	67025000	chr15	76135000	2.12E-02	super_enhancer	promoter	UBE2Q2
chr15	67035000	chr15	72525000	3.56E-02	super_enhancer	promoter	PKM
chr15	67035000	chr15	76555000	1.56E-02	super_enhancer	promoter	TYRO3P
chr15	67035000	chr15	77335000	2.06E-02	super_enhancer	promoter	RP11-797A18.3
chr15	67035000	chr15	85295000	1.58E-02	super_enhancer	promoter	RP11-7M10.2

chr15	67035000	chr15	91565000	1.29E-02	super_enhancer	promoter	AC068831.10
chr15	67045000	chr15	74495000	2.56E-02	super_enhancer	promoter	RP11-60L3.1
chr15	67045000	chr15	83775000	1.74E-02	super_enhancer	promoter	TM6SF1
chr15	67055000	chr15	75315000	2.60E-02	super_enhancer	promoter	RP11-151H2.1
chr15	67055000	chr15	75505000	2.29E-02	super_enhancer	promoter	RP11-69H7.3
chr15	67055000	chr15	77285000	1.84E-02	super_enhancer	promoter	PSTPIP1
chr15	67055000	chr15	85115000	1.21E-02	super_enhancer	promoter	UBE2Q2P1
chr15	67055000	chr15	89745000	1.28E-02	super_enhancer	promoter	RP11-326A19.5
chr15	67065000	chr15	72265000	2.99E-02	super_enhancer	promoter	RP11-390D11.1
chr15	67065000	chr15	74705000	2.52E-02	super_enhancer	promoter	MIR6881
chr15	67065000	chr15	75615000	1.94E-02	super_enhancer	promoter	ANP32BP1
chr15	67065000	chr15	80215000	1.79E-02	super_enhancer	promoter	RP11-81A1.6
chr15	67065000	chr15	90645000	1.13E-02	super_enhancer	promoter	CTD-2315E11.1
chr15	67065000	chr15	93975000	7.29E-03	super_enhancer	promoter	RP11-164C12.1
chr15	67075000	chr15	68125000	4.62E-02	super_enhancer	promoter	RP11-34F13.3
chr15	67075000	chr15	78955000	2.26E-02	super_enhancer	promoter	RPL18P11
chr15	67075000	chr15	101835000	8.37E-03	super_enhancer	promoter	SNRPA1
chr15	67085000	chr15	74595000	2.65E-02	super_enhancer	promoter	RP11-60L3.2
chr15	67085000	chr15	74755000	2.61E-02	super_enhancer	promoter	UBL7
chr15	67085000	chr15	75505000	2.40E-02	super_enhancer	promoter	RP11-69H7.3
chr15	67085000	chr15	75545000	1.61E-02	super_enhancer	promoter	NIFKP4
chr15	67095000	chr15	75315000	2.58E-02	super_enhancer	promoter	RP11-151H2.1
chr15	67095000	chr15	78635000	2.15E-02	super_enhancer	promoter	RP11-5O23.2
chr15	67095000	chr15	90235000	1.34E-02	super_enhancer	promoter	PEX11A

chr15	67105000	chr15	74505000	2.38E-02	super_enhancer	promoter	STRA6
chr15	67105000	chr15	75305000	2.19E-02	super_enhancer	promoter	AC015720.1
chr15	67105000	chr15	78425000	1.79E-02	super_enhancer	promoter	CIB2
chr15	67115000	chr15	78285000	1.99E-02	super_enhancer	promoter	RP11-114H24.6
chr15	67115000	chr15	78635000	2.63E-02	super_enhancer	promoter	RP11-5O23.2
chr15	67115000	chr15	78915000	2.32E-02	super_enhancer	promoter	CHRNA3
chr15	67115000	chr15	80265000	1.64E-02	super_enhancer	promoter	BCL2A1
chr15	67115000	chr15	86195000	1.59E-02	super_enhancer	promoter	RNU6-1280P
chr15	67125000	chr15	73455000	2.05E-02	super_enhancer	promoter	NPM1P43
chr15	67125000	chr15	76605000	2.37E-02	super_enhancer	promoter	ETFA
chr15	67125000	chr15	83395000	1.37E-02	super_enhancer	promoter	AC105339.2
chr15	67135000	chr15	74355000	1.90E-02	super_enhancer	promoter	DNM1P33
chr15	67135000	chr15	75105000	2.50E-02	super_enhancer	promoter	LMAN1L
chr15	67135000	chr15	76135000	1.91E-02	super_enhancer	promoter	UBE2Q2
chr15	67145000	chr15	72335000	2.74E-02	super_enhancer	promoter	EIF5A2P1
chr15	67145000	chr15	73735000	2.03E-02	super_enhancer	promoter	REC114
chr15	67145000	chr15	74755000	2.06E-02	super_enhancer	promoter	UBL7
chr15	67155000	chr15	80265000	1.50E-02	super_enhancer	promoter	BCL2A1
chr15	67165000	chr15	84385000	8.68E-03	super_enhancer	promoter	RNU6-401P
chr15	67175000	chr15	80275000	1.73E-02	super_enhancer	promoter	RP11-81A1.8
chr15	67185000	chr15	67835000	2.09E-03	super_enhancer	promoter	RP11-502I4.3
chr15	67185000	chr15	68125000	4.19E-02	super_enhancer	promoter	RP11-34F13.3
chr15	67185000	chr15	71145000	4.56E-02	super_enhancer	promoter	LRRC49
chr15	67185000	chr15	71185000	3.37E-02	super_enhancer	promoter	THAP10

chr15	67185000	chr15	75305000	2.15E-02	super_enhancer	promoter	AC015720.1
chr15	67185000	chr15	76015000	2.20E-02	super_enhancer	promoter	ODF3L1
chr15	67185000	chr15	81935000	9.61E-03	super_enhancer	promoter	RP11-499F3.1
chr15	67185000	chr15	82555000	1.05E-02	super_enhancer	promoter	EFTUD1
chr15	67525000	chr15	74425000	2.69E-02	promoter	super_enhancer	RPS24P16
chr15	67525000	chr15	74435000	1.73E-02	promoter	super_enhancer	RPS24P16
chr15	67815000	chr15	74405000	1.98E-02	promoter	super_enhancer	IQCH-AS1
chr15	69035000	chr15	69125000	3.39E-02	super_enhancer	promoter	RP11-696L21.2
chr15	69035000	chr15	75315000	2.92E-02	super_enhancer	promoter	RP11-151H2.1
chr15	69035000	chr15	78675000	1.35E-02	super_enhancer	promoter	RP11-5O23.1
chr15	69035000	chr15	84495000	9.57E-03	super_enhancer	promoter	RNU6-1339P
chr15	69045000	chr15	75105000	3.30E-02	super_enhancer	promoter	LMAN1L
chr15	69055000	chr15	73735000	3.01E-02	super_enhancer	promoter	REC114
chr15	69055000	chr15	90235000	1.24E-02	super_enhancer	promoter	PEX11A
chr15	69125000	chr15	88635000	1.07E-02	promoter	super_enhancer	RP11-696L21.2
chr15	69315000	chr15	72225000	4.72E-02	promoter	super_enhancer	RP11-809H16.3
chr15	69315000	chr15	74405000	3.29E-02	promoter	super_enhancer	RP11-809H16.3
chr15	69365000	chr15	74435000	3.28E-02	promoter	super_enhancer	EWSAT1
chr15	69365000	chr15	90165000	1.22E-02	promoter	super_enhancer	EWSAT1
chr15	70475000	chr15	71635000	3.91E-02	super_enhancer	promoter	RP11-592N21.1
chr15	70475000	chr15	76005000	2.57E-02	super_enhancer	promoter	CSPG4
chr15	70475000	chr15	76555000	2.33E-02	super_enhancer	promoter	TYRO3P
chr15	70475000	chr15	80275000	2.08E-02	super_enhancer	promoter	RP11-81A1.8
chr15	70475000	chr15	89165000	1.53E-02	super_enhancer	promoter	AEN

chr15	70495000	chr15	74165000	4.99E-02	super_enhancer	promoter	TBC1D21
chr15	70495000	chr15	75615000	3.12E-02	super_enhancer	promoter	ANP32BP1
chr15	70495000	chr15	76635000	2.20E-02	super_enhancer	promoter	RP11-685G9.2
chr15	70495000	chr15	89905000	1.32E-02	super_enhancer	promoter	LINC00925
chr15	71095000	chr15	91945000	9.69E-03	promoter	super_enhancer	RPL29P30
chr15	71185000	chr15	75655000	4.49E-02	promoter	super_enhancer	THAP10
chr15	71275000	chr15	75505000	2.55E-02	super_enhancer	promoter	RP11-69H7.3
chr15	71295000	chr15	72065000	9.02E-04	super_enhancer	promoter	AC104938.1
chr15	71295000	chr15	75895000	3.71E-02	super_enhancer	promoter	CTD-2323K18.1
chr15	71295000	chr15	77335000	2.52E-02	super_enhancer	promoter	RP11-797A18.3
chr15	71295000	chr15	78385000	2.22E-02	super_enhancer	promoter	SNORA63
chr15	71295000	chr15	85295000	1.53E-02	super_enhancer	promoter	RP11-7M10.2
chr15	71295000	chr15	93015000	9.18E-03	super_enhancer	promoter	RP11-763K15.1
chr15	71635000	chr15	90855000	1.06E-02	promoter	super_enhancer	RP11-592N21.1
chr15	72225000	chr15	72975000	4.67E-02	super_enhancer	promoter	RP11-361M10.4
chr15	72225000	chr15	102155000	6.60E-03	super_enhancer	promoter	RP11-14C10.5
chr15	72235000	chr15	75615000	3.84E-02	super_enhancer	promoter	ANP32BP1
chr15	72235000	chr15	83395000	1.20E-02	super_enhancer	promoter	AC105339.2
chr15	72335000	chr15	74185000	1.24E-02	promoter	super_enhancer	EIF5A2P1
chr15	72975000	chr15	88645000	1.20E-02	promoter	super_enhancer	RP11-361M10.4
chr15	73455000	chr15	92815000	8.06E-03	promoter	super_enhancer	NPM1P43
chr15	73735000	chr15	74185000	2.37E-03	promoter	super_enhancer	REC114
chr15	73975000	chr15	90225000	1.63E-02	promoter	super_enhancer	CD276
chr15	74025000	chr15	74205000	1.38E-03	promoter	super_enhancer	RP11-8P11.4

chr15	74045000	chr15	74195000	4.61E-02	promoter	super_enhancer	C15orf59
chr15	74165000	chr15	74195000	4.78E-03	promoter	super_enhancer	TBC1D21
chr15	74185000	chr15	75505000	3.69E-03	super_enhancer	promoter	RP11-69H7.3
chr15	74185000	chr15	80265000	2.70E-02	super_enhancer	promoter	BCL2A1
chr15	74185000	chr15	98015000	7.53E-03	super_enhancer	promoter	RNA5SP401
chr15	74185000	chr15	98085000	9.04E-03	super_enhancer	promoter	RP11-461F11.2
chr15	74195000	chr15	75305000	4.81E-02	super_enhancer	promoter	AC015720.1
chr15	74195000	chr15	75505000	2.96E-02	super_enhancer	promoter	RP11-69H7.3
chr15	74195000	chr15	78425000	4.65E-02	super_enhancer	promoter	CIB2
chr15	74195000	chr15	87475000	1.23E-02	super_enhancer	promoter	AC016987.1
chr15	74205000	chr15	83425000	1.31E-02	super_enhancer	promoter	AC105339.3
chr15	74205000	chr15	83475000	1.16E-02	super_enhancer	promoter	FSD2
chr15	74205000	chr15	87175000	1.24E-02	super_enhancer	promoter	RP11-182L7.1
chr15	74395000	chr15	74595000	2.34E-04	super_enhancer	promoter	RP11-60L3.2
chr15	74395000	chr15	75595000	6.86E-03	super_enhancer	promoter	DNM1P34
chr15	74395000	chr15	75615000	8.78E-03	super_enhancer	promoter	ANP32BP1
chr15	74395000	chr15	76135000	3.28E-03	super_enhancer	promoter	UBE2Q2
chr15	74395000	chr15	78955000	2.45E-02	super_enhancer	promoter	RPL18P11
chr15	74395000	chr15	80215000	1.79E-02	super_enhancer	promoter	RP11-81A1.6
chr15	74395000	chr15	89765000	9.11E-03	super_enhancer	promoter	RLBP1
chr15	74405000	chr15	74505000	2.97E-02	super_enhancer	promoter	STRA6
chr15	74405000	chr15	90545000	1.60E-02	super_enhancer	promoter	ZNF710
chr15	74415000	chr15	74505000	1.88E-02	super_enhancer	promoter	STRA6
chr15	74415000	chr15	74595000	7.94E-05	super_enhancer	promoter	RP11-60L3.2

chr15	74415000	chr15	75595000	2.48E-02	super_enhancer	promoter	DNM1P34
chr15	74415000	chr15	75895000	2.03E-03	super_enhancer	promoter	CTD-2323K18.1
chr15	74415000	chr15	78425000	4.96E-02	super_enhancer	promoter	CIB2
chr15	74415000	chr15	84285000	1.88E-02	super_enhancer	promoter	AC087738.1
chr15	74425000	chr15	74595000	1.48E-05	super_enhancer	promoter	RP11-60L3.2
chr15	74425000	chr15	75615000	7.49E-04	super_enhancer	promoter	ANP32BP1
chr15	74425000	chr15	76015000	4.14E-02	super_enhancer	promoter	ODF3L1
chr15	74425000	chr15	79105000	4.32E-02	super_enhancer	promoter	ADAMTS7
chr15	74425000	chr15	83225000	2.59E-02	super_enhancer	promoter	RP11-152F13.10
chr15	74425000	chr15	93305000	1.56E-02	super_enhancer	promoter	CTD-2313J17.3
chr15	74435000	chr15	75595000	2.21E-02	super_enhancer	promoter	DNM1P34
chr15	74465000	chr15	75665000	4.71E-02	promoter	super_enhancer	ISLR
chr15	74495000	chr15	89105000	1.23E-02	promoter	super_enhancer	RP11-60L3.1
chr15	74505000	chr15	90195000	1.61E-02	promoter	super_enhancer	STRA6
chr15	74705000	chr15	90205000	1.68E-02	promoter	super_enhancer	MIR6881
chr15	74705000	chr15	90225000	1.72E-02	promoter	super_enhancer	MIR6881
chr15	75035000	chr15	89165000	1.86E-02	super_enhancer	promoter	AEN
chr15	75035000	chr15	98105000	7.58E-03	super_enhancer	promoter	RP11-461F11.3
chr15	75045000	chr15	86295000	2.58E-02	super_enhancer	promoter	RP11-158M2.3
chr15	75055000	chr15	75615000	7.86E-03	super_enhancer	promoter	ANP32BP1
chr15	75055000	chr15	90395000	1.48E-02	super_enhancer	promoter	MIR5094
chr15	75065000	chr15	80035000	2.90E-02	super_enhancer	promoter	RP11-591L14.2
chr15	75305000	chr15	89115000	1.90E-02	promoter	super_enhancer	AC015720.1
chr15	75305000	chr15	91945000	1.29E-02	promoter	super_enhancer	AC015720.1

chr15	75305000	chr15	100705000	7.26E-03	promoter	super_enhancer	AC015720.1
chr15	75545000	chr15	91945000	8.41E-03	promoter	super_enhancer	NIFKP4
chr15	75655000	chr15	76055000	3.93E-02	super_enhancer	promoter	RP11-24M17.6
chr15	75655000	chr15	100275000	1.11E-02	super_enhancer	promoter	LYSMD4
chr15	75665000	chr15	80855000	3.02E-02	super_enhancer	promoter	RP11-379K22.2
chr15	75665000	chr15	80955000	3.24E-02	super_enhancer	promoter	RNU6-380P
chr15	75665000	chr15	85485000	2.13E-02	super_enhancer	promoter	RP11-255M6.1
chr15	75665000	chr15	85725000	1.46E-02	super_enhancer	promoter	NIFKP8
chr15	75675000	chr15	93325000	1.18E-02	super_enhancer	promoter	CTD-2313J17.5
chr15	76005000	chr15	100715000	6.23E-03	promoter	super_enhancer	CSPG4
chr15	76055000	chr15	91945000	1.20E-02	promoter	super_enhancer	RP11-24M17.6
chr15	76135000	chr15	90205000	1.55E-02	promoter	super_enhancer	UBE2Q2
chr15	77285000	chr15	89115000	1.82E-02	promoter	super_enhancer	PSTPIP1
chr15	77285000	chr15	92815000	1.23E-02	promoter	super_enhancer	PSTPIP1
chr15	78385000	chr15	91965000	1.41E-02	promoter	super_enhancer	SNORA63
chr15	78635000	chr15	98885000	1.50E-02	promoter	super_enhancer	RP11-5O23.2
chr15	78675000	chr15	100715000	6.91E-03	promoter	super_enhancer	RP11-5O23.1
chr15	78955000	chr15	90855000	1.77E-02	promoter	super_enhancer	RPL18P11
chr15	79105000	chr15	89115000	1.66E-02	promoter	super_enhancer	ADAMTS7
chr15	79585000	chr15	88645000	1.31E-02	promoter	super_enhancer	ANKRD34C
chr15	79585000	chr15	98885000	1.06E-02	promoter	super_enhancer	ANKRD34C
chr15	79725000	chr15	88625000	1.72E-02	promoter	super_enhancer	KIAA1024
chr15	80035000	chr15	98905000	8.13E-03	promoter	super_enhancer	RP11-591L14.2
chr15	80045000	chr15	91945000	1.23E-02	promoter	super_enhancer	RP11-591L14.1

chr15	80045000	chr15	100725000	1.10E-02	promoter	super_enhancer	RP11-591L14.1
chr15	80215000	chr15	90215000	2.77E-02	promoter	super_enhancer	RP11-81A1.6
chr15	80275000	chr15	90165000	1.98E-02	promoter	super_enhancer	RP11-81A1.8
chr15	80445000	chr15	90205000	1.47E-02	promoter	super_enhancer	FAH
chr15	80855000	chr15	90855000	1.40E-02	promoter	super_enhancer	RP11-379K22.2
chr15	80955000	chr15	100715000	9.15E-03	promoter	super_enhancer	RNU6-380P
chr15	81135000	chr15	89115000	2.08E-02	promoter	super_enhancer	MIR549A
chr15	81135000	chr15	91945000	1.32E-02	promoter	super_enhancer	MIR549A
chr15	81685000	chr15	90165000	1.89E-02	promoter	super_enhancer	CTD-2240J17.2
chr15	81685000	chr15	92815000	1.39E-02	promoter	super_enhancer	CTD-2240J17.2
chr15	81685000	chr15	100705000	7.41E-03	promoter	super_enhancer	CTD-2240J17.2
chr15	81695000	chr15	98895000	1.17E-02	promoter	super_enhancer	CTD-2240J17.4
chr15	81845000	chr15	100725000	1.21E-02	promoter	super_enhancer	CTD-2034I4.2
chr15	81935000	chr15	91945000	1.13E-02	promoter	super_enhancer	RP11-499F3.1
chr15	82555000	chr15	90225000	1.86E-02	promoter	super_enhancer	EFTUD1
chr15	83475000	chr15	90215000	2.16E-02	promoter	super_enhancer	FSD2
chr15	83595000	chr15	90215000	3.39E-02	promoter	super_enhancer	RP11-382A20.5
chr15	83655000	chr15	98905000	1.04E-02	promoter	super_enhancer	HOMER2
chr15	83775000	chr15	91965000	2.05E-02	promoter	super_enhancer	TM6SF1
chr15	84385000	chr15	88635000	2.56E-02	promoter	super_enhancer	RNU6-401P
chr15	84925000	chr15	98865000	9.45E-03	promoter	super_enhancer	UBE2Q2P8
chr15	85115000	chr15	88635000	3.80E-02	promoter	super_enhancer	UBE2Q2P1
chr15	85115000	chr15	91965000	1.85E-02	promoter	super_enhancer	UBE2Q2P1
chr15	85485000	chr15	90165000	4.00E-02	promoter	super_enhancer	RP11-255M6.1

chr15	85485000	chr15	90225000	3.96E-02	promoter	super_enhancer	RP11-255M6.1
chr15	85875000	chr15	98895000	2.01E-02	promoter	super_enhancer	ADAMTS7P4
chr15	85875000	chr15	100715000	1.72E-02	promoter	super_enhancer	ADAMTS7P4
chr15	86295000	chr15	92815000	2.59E-02	promoter	super_enhancer	RP11-158M2.3
chr15	86365000	chr15	90195000	4.90E-02	promoter	super_enhancer	RNU6-231P
chr15	87175000	chr15	89115000	7.09E-03	promoter	super_enhancer	RP11-182L7.1
chr15	87585000	chr15	88645000	1.25E-02	promoter	super_enhancer	RP11-138H10.2
chr15	88625000	chr15	94615000	2.27E-02	super_enhancer	promoter	LINC01579
chr15	88645000	chr15	89165000	2.88E-02	super_enhancer	promoter	AEN
chr15	89105000	chr15	89745000	1.75E-02	super_enhancer	promoter	RP11-326A19.5
chr15	89115000	chr15	89495000	3.00E-02	super_enhancer	promoter	RP11-63A23.2
chr15	89115000	chr15	94405000	2.77E-02	super_enhancer	promoter	RP11-76E17.3
chr15	89115000	chr15	101835000	1.91E-02	super_enhancer	promoter	SNRPA1
chr15	90165000	chr15	95975000	2.45E-02	super_enhancer	promoter	LINC00924
chr15	90195000	chr15	90825000	3.03E-02	super_enhancer	promoter	RP11-697E2.10
chr15	90195000	chr15	102265000	1.14E-02	super_enhancer	promoter	TARSL2
chr15	90205000	chr15	101255000	1.93E-04	super_enhancer	promoter	RP11-66B24.5
chr15	90205000	chr15	101835000	1.84E-02	super_enhancer	promoter	SNRPA1
chr15	90215000	chr15	90625000	6.68E-03	super_enhancer	promoter	RP11-617F23.1
chr15	90215000	chr15	98545000	1.88E-02	super_enhancer	promoter	AC144835.1
chr15	90625000	chr15	98885000	2.22E-02	promoter	super_enhancer	RP11-617F23.1
chr15	90645000	chr15	98905000	2.16E-02	promoter	super_enhancer	CTD-2315E11.1
chr15	90855000	chr15	91465000	2.33E-02	super_enhancer	promoter	AC068831.15
chr15	90865000	chr15	96875000	2.61E-02	super_enhancer	promoter	MIR1469

chr15	90875000	chr15	91465000	4.56E-02	super_enhancer	promoter	AC068831.15
chr15	90875000	chr15	91565000	1.14E-04	super_enhancer	promoter	AC068831.10
chr15	91945000	chr15	92875000	4.59E-03	super_enhancer	promoter	RP11-152L20.3
chr15	92805000	chr15	98825000	2.16E-02	super_enhancer	promoter	RP11-167B3.1
chr15	92815000	chr15	93015000	2.03E-03	super_enhancer	promoter	RP11-763K15.1
chr15	92825000	chr15	101085000	1.71E-02	super_enhancer	promoter	CERS3
chr15	93115000	chr15	98905000	1.97E-02	promoter	super_enhancer	LINC00930
chr15	93145000	chr15	98875000	3.01E-02	promoter	super_enhancer	RP11-386M24.9
chr15	93145000	chr15	100715000	2.13E-02	promoter	super_enhancer	RP11-386M24.9
chr15	93325000	chr15	98855000	2.09E-02	promoter	super_enhancer	CTD-2313J17.5
chr15	93975000	chr15	100725000	2.29E-02	promoter	super_enhancer	RP11-164C12.1
chr15	94615000	chr15	98895000	3.39E-02	promoter	super_enhancer	LINC01579
chr15	94625000	chr15	100705000	1.30E-02	promoter	super_enhancer	AC080032.1
chr15	94775000	chr15	98865000	3.23E-02	promoter	super_enhancer	MCTP2
chr15	95975000	chr15	98895000	4.86E-02	promoter	super_enhancer	LINC00924
chr15	96885000	chr15	98865000	5.30E-03	promoter	super_enhancer	RP11-262A16.1
chr15	98085000	chr15	100715000	4.84E-02	promoter	super_enhancer	RP11-461F11.2
chr15	98535000	chr15	98855000	3.67E-02	promoter	super_enhancer	RP11-753A21.1
chr15	98675000	chr15	98865000	3.64E-02	promoter	super_enhancer	RP11-183E24.2
chr15	98885000	chr15	102345000	4.62E-02	super_enhancer	promoter	OR4F6
chr15	98895000	chr15	101875000	9.46E-04	super_enhancer	promoter	PCSK6-AS1
chr16	85000	chr16	90115000	3.17E-03	super_enhancer	promoter	URAHP
chr16	125000	chr16	315000	8.59E-03	promoter	super_enhancer	Z69720.3
chr16	125000	chr16	9115000	4.27E-02	promoter	super_enhancer	Z69720.3

chr16	125000	chr16	30775000	1.92E-02	promoter	super_enhancer	Z69720.3
chr16	125000	chr16	57835000	6.77E-03	promoter	super_enhancer	Z69720.3
chr16	125000	chr16	66395000	7.48E-03	promoter	super_enhancer	Z69720.3
chr16	175000	chr16	365000	3.81E-03	promoter	super_enhancer	Z69666.2
chr16	175000	chr16	15995000	2.89E-02	promoter	super_enhancer	Z69666.2
chr16	175000	chr16	16025000	2.66E-02	promoter	super_enhancer	Z69666.2
chr16	175000	chr16	29825000	1.92E-02	promoter	super_enhancer	Z69666.2
chr16	175000	chr16	30775000	2.02E-02	promoter	super_enhancer	Z69666.2
chr16	175000	chr16	67245000	4.46E-03	promoter	super_enhancer	Z69666.2
chr16	285000	chr16	315000	2.39E-02	promoter	super_enhancer	ITFG3
chr16	285000	chr16	11605000	4.43E-02	promoter	super_enhancer	ITFG3
chr16	285000	chr16	30895000	1.36E-02	promoter	super_enhancer	ITFG3
chr16	285000	chr16	85565000	4.34E-03	promoter	super_enhancer	ITFG3
chr16	315000	chr16	22105000	1.56E-02	super_enhancer	promoter	RP11-101E7.2
chr16	315000	chr16	30545000	1.37E-02	super_enhancer	promoter	AC002310.12
chr16	345000	chr16	28875000	1.96E-02	super_enhancer	promoter	RP11-22P6.2
chr16	345000	chr16	30135000	2.26E-02	super_enhancer	promoter	MAPK3
chr16	345000	chr16	31105000	1.99E-02	super_enhancer	promoter	RP11-196G11.3
chr16	345000	chr16	88135000	3.43E-03	super_enhancer	promoter	RP11-863P13.4
chr16	355000	chr16	27875000	1.65E-02	super_enhancer	promoter	RNU6-159P
chr16	355000	chr16	28505000	1.35E-02	super_enhancer	promoter	APOBR
chr16	355000	chr16	30035000	1.95E-02	super_enhancer	promoter	DOC2A
chr16	355000	chr16	66615000	5.43E-03	super_enhancer	promoter	RP11-403P17.2
chr16	355000	chr16	70805000	4.34E-03	super_enhancer	promoter	RP11-394B2.3

chr16	355000	chr16	85825000	4.22E-03	super_enhancer	promoter	RP11-568J23.6
chr16	365000	chr16	25105000	1.63E-02	super_enhancer	promoter	RP11-266L9.3
chr16	365000	chr16	57125000	7.97E-03	super_enhancer	promoter	CPNE2
chr16	365000	chr16	85475000	5.31E-03	super_enhancer	promoter	AC092377.1
chr16	365000	chr16	90115000	3.75E-03	super_enhancer	promoter	URAHP
chr16	475000	chr16	19115000	2.48E-02	promoter	super_enhancer	LA16c-359F1.1
chr16	475000	chr16	56985000	7.07E-03	promoter	super_enhancer	LA16c-359F1.1
chr16	585000	chr16	29825000	1.97E-02	promoter	super_enhancer	MIR5587
chr16	585000	chr16	57615000	4.78E-03	promoter	super_enhancer	MIR5587
chr16	585000	chr16	84605000	4.17E-03	promoter	super_enhancer	MIR5587
chr16	605000	chr16	16035000	1.92E-02	promoter	super_enhancer	LA16c-366D1.3
chr16	605000	chr16	19125000	1.50E-02	promoter	super_enhancer	LA16c-366D1.3
chr16	605000	chr16	30655000	1.08E-02	promoter	super_enhancer	LA16c-366D1.3
chr16	605000	chr16	70765000	2.13E-03	promoter	super_enhancer	LA16c-366D1.3
chr16	725000	chr16	765000	4.63E-02	promoter	super_enhancer	RHBDL1
chr16	725000	chr16	73035000	4.67E-03	promoter	super_enhancer	RHBDL1
chr16	735000	chr16	73095000	4.67E-03	promoter	super_enhancer	JMJD8
chr16	765000	chr16	855000	9.85E-04	super_enhancer	promoter	PRR25
chr16	855000	chr16	1085000	3.64E-03	promoter	super_enhancer	PRR25
chr16	855000	chr16	9055000	4.27E-02	promoter	super_enhancer	PRR25
chr16	855000	chr16	31055000	2.30E-02	promoter	super_enhancer	PRR25
chr16	855000	chr16	57655000	8.87E-03	promoter	super_enhancer	PRR25
chr16	855000	chr16	81505000	5.21E-03	promoter	super_enhancer	PRR25
chr16	855000	chr16	82645000	4.98E-03	promoter	super_enhancer	PRR25

chr16	855000	chr16	86375000	3.71E-03	promoter	super_enhancer	PRR25
chr16	855000	chr16	88785000	6.04E-03	promoter	super_enhancer	PRR25
chr16	955000	chr16	1305000	3.71E-03	promoter	super_enhancer	LA16c-306A4.2
chr16	1085000	chr16	1765000	1.25E-03	super_enhancer	promoter	LA16c-329F2.1
chr16	1085000	chr16	20835000	1.71E-02	super_enhancer	promoter	U4atac
chr16	1085000	chr16	30065000	2.11E-02	super_enhancer	promoter	FAM57B
chr16	1085000	chr16	31815000	1.25E-02	super_enhancer	promoter	VN1R67P
chr16	1085000	chr16	77285000	3.12E-03	super_enhancer	promoter	RP11-538I12.2
chr16	1085000	chr16	82155000	3.59E-03	super_enhancer	promoter	CTD-2588J6.1
chr16	1155000	chr16	1295000	4.74E-02	promoter	super_enhancer	LA16c-349E11.1
chr16	1155000	chr16	1305000	5.78E-03	promoter	super_enhancer	LA16c-349E11.1
chr16	1155000	chr16	86365000	3.31E-03	promoter	super_enhancer	LA16c-349E11.1
chr16	1295000	chr16	12705000	1.93E-02	super_enhancer	promoter	CTD-3037G24.3
chr16	1295000	chr16	68405000	4.35E-03	super_enhancer	promoter	RP11-71L14.3
chr16	1305000	chr16	30495000	1.45E-02	super_enhancer	promoter	RNU7-61P
chr16	1305000	chr16	31565000	1.10E-02	super_enhancer	promoter	CTD-2014E2.6
chr16	1305000	chr16	50185000	6.55E-03	super_enhancer	promoter	RPL10P14
chr16	1315000	chr16	84615000	4.33E-03	promoter	super_enhancer	PRSS29P
chr16	1315000	chr16	85505000	4.32E-03	promoter	super_enhancer	PRSS29P
chr16	1315000	chr16	85525000	3.40E-03	promoter	super_enhancer	PRSS29P
chr16	1465000	chr16	15995000	2.66E-02	promoter	super_enhancer	UNKL
chr16	1465000	chr16	84585000	4.36E-03	promoter	super_enhancer	UNKL
chr16	1495000	chr16	1685000	5.81E-03	promoter	super_enhancer	CCDC154
chr16	1495000	chr16	15585000	2.55E-02	promoter	super_enhancer	CCDC154

chr16	1495000	chr16	86395000	3.52E-03	promoter	super_enhancer	CCDC154
chr16	1605000	chr16	19135000	2.33E-02	promoter	super_enhancer	LA16c-380F5.3
chr16	1605000	chr16	57625000	4.91E-03	promoter	super_enhancer	LA16c-380F5.3
chr16	1605000	chr16	85525000	3.41E-03	promoter	super_enhancer	LA16c-380F5.3
chr16	1685000	chr16	22105000	1.83E-02	super_enhancer	promoter	RP11-101E7.2
chr16	1685000	chr16	85205000	4.33E-03	super_enhancer	promoter	CTC-786C10.1
chr16	1685000	chr16	89155000	4.21E-03	super_enhancer	promoter	ACSF3
chr16	1745000	chr16	1755000	9.95E-03	super_enhancer	promoter	MAPK8IP3
chr16	1745000	chr16	28635000	1.56E-02	super_enhancer	promoter	SULT1A1
chr16	1745000	chr16	29085000	1.06E-02	super_enhancer	promoter	RRN3P2
chr16	1745000	chr16	31885000	1.28E-02	super_enhancer	promoter	ZNF267
chr16	1745000	chr16	34405000	6.01E-03	super_enhancer	promoter	UBE2MP1
chr16	1755000	chr16	57265000	4.49E-03	promoter	super_enhancer	MAPK8IP3
chr16	1755000	chr16	69405000	5.30E-03	promoter	super_enhancer	MAPK8IP3
chr16	1765000	chr16	15995000	2.74E-02	promoter	super_enhancer	LA16c-329F2.1
chr16	1845000	chr16	9065000	2.85E-02	promoter	super_enhancer	SPSB3
chr16	1845000	chr16	15585000	1.80E-02	promoter	super_enhancer	SPSB3
chr16	1845000	chr16	29825000	1.12E-02	promoter	super_enhancer	SPSB3
chr16	1845000	chr16	30775000	1.18E-02	promoter	super_enhancer	SPSB3
chr16	1845000	chr16	89325000	2.27E-03	promoter	super_enhancer	SPSB3
chr16	1895000	chr16	11625000	3.04E-02	promoter	super_enhancer	LA16c-429E7.1
chr16	1895000	chr16	19135000	2.23E-02	promoter	super_enhancer	LA16c-429E7.1
chr16	1935000	chr16	69415000	4.68E-03	promoter	super_enhancer	MEIOB
chr16	1935000	chr16	70405000	5.30E-03	promoter	super_enhancer	MEIOB

chr16	1935000	chr16	81455000	3.93E-03	promoter	super_enhancer	MEIOB
chr16	2015000	chr16	15575000	3.13E-02	promoter	super_enhancer	RPS2
chr16	2015000	chr16	81525000	3.34E-03	promoter	super_enhancer	RPS2
chr16	2035000	chr16	4945000	1.14E-02	promoter	super_enhancer	NOXO1
chr16	2035000	chr16	11615000	3.53E-02	promoter	super_enhancer	NOXO1
chr16	2035000	chr16	15995000	3.03E-02	promoter	super_enhancer	NOXO1
chr16	2035000	chr16	16005000	3.07E-02	promoter	super_enhancer	NOXO1
chr16	2035000	chr16	56985000	7.37E-03	promoter	super_enhancer	NOXO1
chr16	2035000	chr16	66525000	6.00E-03	promoter	super_enhancer	NOXO1
chr16	2035000	chr16	66535000	3.67E-03	promoter	super_enhancer	NOXO1
chr16	2075000	chr16	11635000	4.23E-02	promoter	super_enhancer	SLC9A3R2
chr16	2075000	chr16	15595000	3.37E-02	promoter	super_enhancer	SLC9A3R2
chr16	2075000	chr16	70745000	6.02E-03	promoter	super_enhancer	SLC9A3R2
chr16	2205000	chr16	57275000	4.85E-03	promoter	super_enhancer	SNORD60
chr16	2205000	chr16	57805000	6.35E-03	promoter	super_enhancer	SNORD60
chr16	2255000	chr16	16015000	3.39E-02	promoter	super_enhancer	AC009065.2
chr16	2255000	chr16	81465000	4.77E-03	promoter	super_enhancer	AC009065.2
chr16	2265000	chr16	9125000	4.53E-02	promoter	super_enhancer	PGP
chr16	2265000	chr16	15655000	2.51E-02	promoter	super_enhancer	PGP
chr16	2285000	chr16	9075000	4.61E-02	promoter	super_enhancer	DNASE1L2
chr16	2285000	chr16	31065000	1.85E-02	promoter	super_enhancer	DNASE1L2
chr16	2285000	chr16	31465000	2.04E-02	promoter	super_enhancer	DNASE1L2
chr16	2285000	chr16	57295000	8.77E-03	promoter	super_enhancer	DNASE1L2
chr16	2285000	chr16	85525000	3.94E-03	promoter	super_enhancer	DNASE1L2

chr16	2285000	chr16	87805000	4.17E-03	promoter	super_enhancer	DNASE1L2
chr16	2325000	chr16	19095000	2.75E-02	promoter	super_enhancer	MIR4717
chr16	2325000	chr16	31055000	2.38E-02	promoter	super_enhancer	MIR4717
chr16	2325000	chr16	70765000	4.42E-03	promoter	super_enhancer	MIR4717
chr16	2325000	chr16	82665000	5.30E-03	promoter	super_enhancer	MIR4717
chr16	2325000	chr16	84615000	5.45E-03	promoter	super_enhancer	MIR4717
chr16	2525000	chr16	19125000	2.88E-02	promoter	super_enhancer	TBC1D24
chr16	2525000	chr16	57285000	4.75E-03	promoter	super_enhancer	TBC1D24
chr16	2545000	chr16	15675000	3.96E-02	promoter	super_enhancer	RP11-20I23.1
chr16	2545000	chr16	29825000	2.38E-02	promoter	super_enhancer	RP11-20I23.1
chr16	2545000	chr16	57315000	7.70E-03	promoter	super_enhancer	RP11-20I23.1
chr16	2545000	chr16	81525000	4.33E-03	promoter	super_enhancer	RP11-20I23.1
chr16	2895000	chr16	18995000	2.07E-02	super_enhancer	promoter	TMC7
chr16	2895000	chr16	29675000	1.57E-02	super_enhancer	promoter	AC009133.22
chr16	2895000	chr16	89725000	3.79E-03	super_enhancer	promoter	CHMP1A
chr16	2905000	chr16	14165000	4.17E-02	super_enhancer	promoter	MKL2
chr16	2905000	chr16	14395000	4.10E-02	super_enhancer	promoter	RP11-65J21.3
chr16	2905000	chr16	15705000	3.20E-02	super_enhancer	promoter	MIR6506
chr16	2905000	chr16	25085000	1.85E-02	super_enhancer	promoter	RP11-266L9.6
chr16	2905000	chr16	28635000	1.78E-02	super_enhancer	promoter	SULT1A1
chr16	2905000	chr16	31715000	1.70E-02	super_enhancer	promoter	CTD-2358C21.5
chr16	2915000	chr16	28635000	1.78E-02	super_enhancer	promoter	SULT1A1
chr16	2915000	chr16	48505000	6.07E-03	super_enhancer	promoter	RP11-44I10.5
chr16	2915000	chr16	56485000	6.72E-03	super_enhancer	promoter	OGFOD1

chr16	2925000	chr16	11165000	4.34E-02	super_enhancer	promoter	RP11-66H6.3
chr16	2925000	chr16	30585000	2.42E-02	super_enhancer	promoter	ZNF688
chr16	2925000	chr16	56665000	7.43E-03	super_enhancer	promoter	MT1M
chr16	2925000	chr16	87435000	3.95E-03	super_enhancer	promoter	RP11-178L8.3
chr16	3005000	chr16	19115000	3.15E-02	promoter	super_enhancer	LA16c-321D4.2
chr16	3055000	chr16	15595000	2.70E-02	promoter	super_enhancer	LA16c-380H5.2
chr16	3055000	chr16	30775000	1.89E-02	promoter	super_enhancer	LA16c-380H5.2
chr16	3055000	chr16	84595000	4.01E-03	promoter	super_enhancer	LA16c-380H5.2
chr16	3055000	chr16	86375000	2.84E-03	promoter	super_enhancer	LA16c-380H5.2
chr16	3075000	chr16	11615000	3.61E-02	promoter	super_enhancer	HCFC1R1
chr16	3075000	chr16	15995000	3.03E-02	promoter	super_enhancer	HCFC1R1
chr16	3075000	chr16	16005000	4.78E-04	promoter	super_enhancer	HCFC1R1
chr16	3075000	chr16	57805000	6.63E-03	promoter	super_enhancer	HCFC1R1
chr16	3075000	chr16	84595000	4.33E-03	promoter	super_enhancer	HCFC1R1
chr16	3115000	chr16	30665000	2.12E-02	promoter	super_enhancer	IL32
chr16	3115000	chr16	31455000	1.64E-02	promoter	super_enhancer	IL32
chr16	3115000	chr16	66535000	3.56E-03	promoter	super_enhancer	IL32
chr16	3235000	chr16	15495000	3.33E-02	promoter	super_enhancer	AJ003147.8
chr16	3235000	chr16	15595000	3.11E-02	promoter	super_enhancer	AJ003147.8
chr16	3235000	chr16	15675000	3.46E-02	promoter	super_enhancer	AJ003147.8
chr16	3235000	chr16	16005000	3.28E-02	promoter	super_enhancer	AJ003147.8
chr16	3235000	chr16	16035000	3.82E-02	promoter	super_enhancer	AJ003147.8
chr16	3235000	chr16	82665000	4.70E-03	promoter	super_enhancer	AJ003147.8
chr16	3235000	chr16	82675000	3.37E-03	promoter	super_enhancer	AJ003147.8

chr16	3235000	chr16	85565000	4.15E-03	promoter	super_enhancer	AJ003147.8
chr16	3265000	chr16	4945000	4.69E-02	promoter	super_enhancer	OR1F2P
chr16	3265000	chr16	11615000	3.56E-02	promoter	super_enhancer	OR1F2P
chr16	3265000	chr16	30895000	1.32E-02	promoter	super_enhancer	OR1F2P
chr16	3265000	chr16	31055000	2.00E-02	promoter	super_enhancer	OR1F2P
chr16	3355000	chr16	15675000	3.05E-02	promoter	super_enhancer	ZNF75A
chr16	3355000	chr16	19125000	2.60E-02	promoter	super_enhancer	ZNF75A
chr16	3375000	chr16	11615000	3.60E-02	promoter	super_enhancer	RP11-433P17.1
chr16	3375000	chr16	16015000	3.06E-02	promoter	super_enhancer	RP11-433P17.1
chr16	3375000	chr16	19125000	2.73E-02	promoter	super_enhancer	RP11-433P17.1
chr16	3375000	chr16	66395000	7.47E-03	promoter	super_enhancer	RP11-433P17.1
chr16	3405000	chr16	4305000	7.29E-03	promoter	super_enhancer	OR2C1
chr16	3405000	chr16	29825000	1.88E-04	promoter	super_enhancer	OR2C1
chr16	3405000	chr16	57815000	6.53E-03	promoter	super_enhancer	OR2C1
chr16	3405000	chr16	57835000	6.95E-03	promoter	super_enhancer	OR2C1
chr16	3415000	chr16	81515000	3.54E-03	promoter	super_enhancer	LA16c-306E5.2
chr16	3495000	chr16	15505000	2.91E-02	promoter	super_enhancer	ZNF597
chr16	3495000	chr16	31465000	1.81E-02	promoter	super_enhancer	ZNF597
chr16	3535000	chr16	9055000	4.29E-02	promoter	super_enhancer	MIR6126
chr16	3535000	chr16	15665000	2.61E-02	promoter	super_enhancer	MIR6126
chr16	3535000	chr16	30665000	1.82E-02	promoter	super_enhancer	MIR6126
chr16	3535000	chr16	70685000	4.35E-03	promoter	super_enhancer	MIR6126
chr16	3545000	chr16	19135000	2.95E-02	promoter	super_enhancer	C16orf90
chr16	3545000	chr16	27315000	1.28E-02	promoter	super_enhancer	C16orf90

chr16	3545000	chr16	31455000	1.87E-02	promoter	super_enhancer	C16orf90
chr16	3585000	chr16	11615000	4.26E-02	promoter	super_enhancer	LA16c-390H2.1
chr16	3585000	chr16	15605000	3.72E-02	promoter	super_enhancer	LA16c-390H2.1
chr16	3585000	chr16	30775000	2.33E-02	promoter	super_enhancer	LA16c-390H2.1
chr16	3955000	chr16	19325000	3.03E-02	super_enhancer	promoter	CTA-363E6.1
chr16	3955000	chr16	25035000	2.02E-02	super_enhancer	promoter	AC133552.1
chr16	3955000	chr16	28075000	2.09E-02	super_enhancer	promoter	GSG1L
chr16	3955000	chr16	30715000	1.69E-02	super_enhancer	promoter	RP11-2C24.9
chr16	3955000	chr16	30905000	2.08E-02	super_enhancer	promoter	MIR762
chr16	3955000	chr16	68235000	5.21E-03	super_enhancer	promoter	RP11-67A1.4
chr16	3965000	chr16	10785000	4.84E-02	super_enhancer	promoter	RP11-109M19.1
chr16	3965000	chr16	15225000	1.99E-02	super_enhancer	promoter	AC139256.1
chr16	3965000	chr16	29215000	1.58E-02	super_enhancer	promoter	RP11-426C22.1
chr16	3965000	chr16	31885000	1.36E-02	super_enhancer	promoter	ZNF267
chr16	3965000	chr16	90115000	3.51E-03	super_enhancer	promoter	URAHP
chr16	3975000	chr16	20795000	2.22E-02	super_enhancer	promoter	RNU6-944P
chr16	3975000	chr16	22825000	1.81E-02	super_enhancer	promoter	HS3ST2
chr16	3975000	chr16	27465000	1.85E-02	super_enhancer	promoter	IL21R-AS1
chr16	3975000	chr16	30515000	2.04E-02	super_enhancer	promoter	MIR4518
chr16	3975000	chr16	31365000	1.69E-02	super_enhancer	promoter	ITGAX
chr16	3975000	chr16	86545000	4.82E-03	super_enhancer	promoter	AC009108.2
chr16	3985000	chr16	11475000	4.53E-02	super_enhancer	promoter	CTD-3088G3.4
chr16	3985000	chr16	28995000	2.53E-02	super_enhancer	promoter	LAT
chr16	3985000	chr16	30035000	2.25E-02	super_enhancer	promoter	DOC2A

chr16	3985000	chr16	31215000	2.11E-02	super_enhancer	promoter	PYCARD
chr16	3985000	chr16	47495000	6.13E-03	super_enhancer	promoter	PHKB
chr16	3985000	chr16	69665000	4.75E-03	super_enhancer	promoter	RP11-140H17.2
chr16	3985000	chr16	89525000	2.79E-03	super_enhancer	promoter	RP1-168P16.3
chr16	3995000	chr16	11475000	4.08E-02	super_enhancer	promoter	CTD-3088G3.4
chr16	3995000	chr16	15225000	1.78E-04	super_enhancer	promoter	AC139256.1
chr16	3995000	chr16	29085000	1.07E-02	super_enhancer	promoter	RRN3P2
chr16	3995000	chr16	30135000	2.07E-02	super_enhancer	promoter	MAPK3
chr16	3995000	chr16	31185000	1.44E-02	super_enhancer	promoter	NDUFA3P6
chr16	3995000	chr16	31885000	1.28E-02	super_enhancer	promoter	ZNF267
chr16	3995000	chr16	57495000	6.46E-03	super_enhancer	promoter	AC009052.12
chr16	3995000	chr16	68625000	3.54E-03	super_enhancer	promoter	RP11-615I2.6
chr16	4005000	chr16	14115000	3.72E-02	super_enhancer	promoter	CTD-2135D7.4
chr16	4005000	chr16	15105000	3.08E-02	super_enhancer	promoter	MIR1972-1
chr16	4005000	chr16	20085000	2.18E-02	super_enhancer	promoter	GPR139
chr16	4005000	chr16	56665000	6.73E-03	super_enhancer	promoter	MT1M
chr16	4005000	chr16	68295000	6.67E-03	super_enhancer	promoter	RP11-96D1.7
chr16	4015000	chr16	11375000	4.90E-02	super_enhancer	promoter	PRM1
chr16	4015000	chr16	19535000	2.13E-02	super_enhancer	promoter	CCP110
chr16	4015000	chr16	25035000	1.78E-02	super_enhancer	promoter	AC133552.1
chr16	4015000	chr16	30125000	1.89E-02	super_enhancer	promoter	GDPD3
chr16	4015000	chr16	68775000	5.76E-03	super_enhancer	promoter	RNA5SP429
chr16	4015000	chr16	71495000	3.41E-03	super_enhancer	promoter	RP11-510M2.2
chr16	4235000	chr16	15995000	3.36E-02	promoter	super_enhancer	RP11-95P2.3

chr16	4235000	chr16	19095000	2.63E-02	promoter	super_enhancer	RP11-95P2.3
chr16	4235000	chr16	57295000	8.75E-03	promoter	super_enhancer	RP11-95P2.3
chr16	4305000	chr16	11475000	4.22E-02	super_enhancer	promoter	CTD-3088G3.4
chr16	4305000	chr16	11835000	2.95E-02	super_enhancer	promoter	RP11-490O6.2
chr16	4305000	chr16	30065000	2.05E-02	super_enhancer	promoter	FAM57B
chr16	4305000	chr16	85205000	4.09E-03	super_enhancer	promoter	CTC-786C10.1
chr16	4365000	chr16	11615000	4.44E-02	promoter	super_enhancer	GLIS2
chr16	4365000	chr16	11635000	4.58E-02	promoter	super_enhancer	GLIS2
chr16	4365000	chr16	15615000	3.38E-02	promoter	super_enhancer	GLIS2
chr16	4365000	chr16	29825000	2.18E-02	promoter	super_enhancer	GLIS2
chr16	4365000	chr16	30665000	2.39E-02	promoter	super_enhancer	GLIS2
chr16	4365000	chr16	70405000	5.59E-03	promoter	super_enhancer	GLIS2
chr16	4475000	chr16	19125000	3.25E-02	promoter	super_enhancer	DNAJA3
chr16	4475000	chr16	57305000	8.19E-03	promoter	super_enhancer	DNAJA3
chr16	4475000	chr16	70715000	5.87E-03	promoter	super_enhancer	DNAJA3
chr16	4525000	chr16	11625000	4.51E-02	promoter	super_enhancer	HMOX2
chr16	4525000	chr16	15575000	3.86E-02	promoter	super_enhancer	HMOX2
chr16	4525000	chr16	57825000	7.19E-03	promoter	super_enhancer	HMOX2
chr16	4525000	chr16	85515000	3.56E-03	promoter	super_enhancer	HMOX2
chr16	4525000	chr16	89335000	3.91E-03	promoter	super_enhancer	HMOX2
chr16	4585000	chr16	21515000	2.26E-02	promoter	super_enhancer	RP11-709D24.8
chr16	4585000	chr16	70755000	5.92E-03	promoter	super_enhancer	RP11-709D24.8
chr16	4585000	chr16	75245000	5.66E-03	promoter	super_enhancer	RP11-709D24.8
chr16	4585000	chr16	85555000	4.48E-03	promoter	super_enhancer	RP11-709D24.8

chr16	4605000	chr16	15495000	3.42E-02	promoter	super_enhancer	C16orf96
chr16	4605000	chr16	15675000	3.55E-02	promoter	super_enhancer	C16orf96
chr16	4605000	chr16	16015000	3.40E-02	promoter	super_enhancer	C16orf96
chr16	4605000	chr16	19105000	2.73E-02	promoter	super_enhancer	C16orf96
chr16	4605000	chr16	29825000	2.07E-02	promoter	super_enhancer	C16orf96
chr16	4605000	chr16	31465000	2.03E-02	promoter	super_enhancer	C16orf96
chr16	4685000	chr16	11615000	4.42E-02	promoter	super_enhancer	RN7SL850P
chr16	4685000	chr16	15595000	3.23E-02	promoter	super_enhancer	RN7SL850P
chr16	4685000	chr16	15655000	3.22E-02	promoter	super_enhancer	RN7SL850P
chr16	4685000	chr16	19095000	2.63E-02	promoter	super_enhancer	RN7SL850P
chr16	4685000	chr16	19115000	2.93E-02	promoter	super_enhancer	RN7SL850P
chr16	4685000	chr16	27325000	2.05E-02	promoter	super_enhancer	RN7SL850P
chr16	4685000	chr16	56975000	5.43E-03	promoter	super_enhancer	RN7SL850P
chr16	4685000	chr16	74725000	4.83E-03	promoter	super_enhancer	RN7SL850P
chr16	4685000	chr16	85565000	4.03E-03	promoter	super_enhancer	RN7SL850P
chr16	4785000	chr16	9055000	1.91E-03	promoter	super_enhancer	ANKS3
chr16	4785000	chr16	16005000	3.01E-02	promoter	super_enhancer	ANKS3
chr16	4785000	chr16	29825000	1.85E-02	promoter	super_enhancer	ANKS3
chr16	4785000	chr16	31055000	1.95E-02	promoter	super_enhancer	ANKS3
chr16	4785000	chr16	70715000	4.84E-03	promoter	super_enhancer	ANKS3
chr16	4785000	chr16	84515000	3.97E-03	promoter	super_enhancer	ANKS3
chr16	4905000	chr16	19535000	2.19E-02	super_enhancer	promoter	CCP110
chr16	4905000	chr16	28835000	1.72E-02	super_enhancer	promoter	RP11-1348G14.8
chr16	4905000	chr16	29675000	1.88E-02	super_enhancer	promoter	AC009133.22

chr16	4905000	chr16	65615000	4.56E-03	super_enhancer	promoter	RP11-351A20.1
chr16	4915000	chr16	12175000	3.93E-02	super_enhancer	promoter	RPS23P6
chr16	4915000	chr16	24345000	1.86E-02	super_enhancer	promoter	SNORA1
chr16	4915000	chr16	27215000	1.16E-02	super_enhancer	promoter	KDM8
chr16	4915000	chr16	30545000	1.65E-02	super_enhancer	promoter	AC002310.12
chr16	4915000	chr16	31225000	1.99E-02	super_enhancer	promoter	TRIM72
chr16	4915000	chr16	46885000	8.54E-03	super_enhancer	promoter	RP11-480G7.3
chr16	4925000	chr16	11835000	3.40E-02	super_enhancer	promoter	RP11-490O6.2
chr16	4925000	chr16	11945000	1.18E-03	super_enhancer	promoter	RSL1D1
chr16	4925000	chr16	14115000	3.88E-02	super_enhancer	promoter	CTD-2135D7.4
chr16	4925000	chr16	15795000	3.54E-02	super_enhancer	promoter	CTB-193M12.5
chr16	4925000	chr16	19325000	2.92E-02	super_enhancer	promoter	CTA-363E6.1
chr16	4925000	chr16	28565000	2.00E-02	super_enhancer	promoter	RP11-666O2.2
chr16	4925000	chr16	28995000	2.49E-02	super_enhancer	promoter	LAT
chr16	4925000	chr16	30035000	2.21E-02	super_enhancer	promoter	DOC2A
chr16	4925000	chr16	30495000	1.95E-02	super_enhancer	promoter	RNU7-61P
chr16	4925000	chr16	31405000	1.68E-02	super_enhancer	promoter	ITGAD
chr16	4925000	chr16	68035000	5.43E-03	super_enhancer	promoter	DPEP2
chr16	4925000	chr16	89725000	4.50E-03	super_enhancer	promoter	CHMP1A
chr16	4935000	chr16	15085000	4.25E-02	super_enhancer	promoter	RP11-680G24.4
chr16	4935000	chr16	19535000	2.48E-02	super_enhancer	promoter	CCP110
chr16	4935000	chr16	21205000	2.25E-02	super_enhancer	promoter	AF001550.7
chr16	4935000	chr16	28835000	1.94E-02	super_enhancer	promoter	RP11-1348G14.8
chr16	4935000	chr16	29435000	1.27E-02	super_enhancer	promoter	AC025279.1

chr16	4935000	chr16	30495000	2.08E-02	super_enhancer	promoter	RNU7-61P
chr16	4935000	chr16	31195000	2.12E-02	super_enhancer	promoter	RP11-388M20.6
chr16	4935000	chr16	50715000	8.73E-03	super_enhancer	promoter	SNX20
chr16	4935000	chr16	66615000	6.20E-03	super_enhancer	promoter	RP11-403P17.2
chr16	4935000	chr16	68405000	5.05E-03	super_enhancer	promoter	RP11-71L14.3
chr16	4935000	chr16	85645000	4.43E-03	super_enhancer	promoter	GSE1
chr16	4945000	chr16	12175000	4.49E-02	super_enhancer	promoter	RPS23P6
chr16	4945000	chr16	15835000	3.57E-02	super_enhancer	promoter	AF001548.6
chr16	4945000	chr16	19405000	2.86E-02	super_enhancer	promoter	CTA-363E6.3
chr16	4945000	chr16	31105000	2.10E-02	super_enhancer	promoter	RP11-196G11.3
chr16	4945000	chr16	31195000	2.11E-02	super_enhancer	promoter	RP11-388M20.6
chr16	4945000	chr16	56645000	6.63E-03	super_enhancer	promoter	RP11-249C24.10
chr16	4945000	chr16	58665000	5.75E-03	super_enhancer	promoter	CNOT1
chr16	4945000	chr16	74585000	3.89E-03	super_enhancer	promoter	RP11-572F4.1
chr16	4945000	chr16	85205000	4.61E-03	super_enhancer	promoter	CTC-786C10.1
chr16	4955000	chr16	14295000	2.78E-02	super_enhancer	promoter	CTA-276F8.2
chr16	4955000	chr16	14995000	1.32E-02	super_enhancer	promoter	MIR3179-1
chr16	4955000	chr16	18995000	2.82E-02	super_enhancer	promoter	TMC7
chr16	4955000	chr16	19535000	2.45E-02	super_enhancer	promoter	CCP110
chr16	4955000	chr16	34985000	1.12E-02	super_enhancer	promoter	RNA5SP416
chr16	4955000	chr16	58265000	6.85E-03	super_enhancer	promoter	CCDC113
chr16	4955000	chr16	83965000	3.80E-03	super_enhancer	promoter	RP11-483P21.6
chr16	5085000	chr16	16015000	3.87E-02	promoter	super_enhancer	NAGPA
chr16	5085000	chr16	70705000	5.85E-03	promoter	super_enhancer	NAGPA

chr16	7055000	chr16	30785000	1.72E-02	promoter	super_enhancer	RNU6-328P
chr16	7055000	chr16	89305000	3.13E-03	promoter	super_enhancer	RNU6-328P
chr16	7665000	chr16	31455000	1.21E-02	promoter	super_enhancer	RP11-26O3.1
chr16	8715000	chr16	11605000	1.46E-02	promoter	super_enhancer	METTL22
chr16	8715000	chr16	27325000	2.56E-02	promoter	super_enhancer	METTL22
chr16	8715000	chr16	31065000	2.29E-02	promoter	super_enhancer	METTL22
chr16	8715000	chr16	57295000	1.02E-02	promoter	super_enhancer	METTL22
chr16	8855000	chr16	18995000	3.39E-02	super_enhancer	promoter	TMC7
chr16	8855000	chr16	30585000	2.72E-02	super_enhancer	promoter	ZNF688
chr16	8855000	chr16	30905000	2.38E-02	super_enhancer	promoter	MIR762
chr16	8855000	chr16	47795000	6.17E-03	super_enhancer	promoter	CTB-55P19.1
chr16	8855000	chr16	48455000	9.25E-03	super_enhancer	promoter	AC023818.1
chr16	8855000	chr16	58265000	7.51E-03	super_enhancer	promoter	CCDC113
chr16	8855000	chr16	68295000	7.69E-03	super_enhancer	promoter	RP11-96D1.7
chr16	8855000	chr16	71265000	2.91E-03	super_enhancer	promoter	HYDIN
chr16	8855000	chr16	73545000	4.87E-03	super_enhancer	promoter	RP11-44L9.3
chr16	8865000	chr16	10785000	2.46E-02	super_enhancer	promoter	RP11-109M19.1
chr16	8865000	chr16	15705000	8.15E-04	super_enhancer	promoter	MIR6506
chr16	8865000	chr16	31185000	1.65E-02	super_enhancer	promoter	NDUFA3P6
chr16	8865000	chr16	31195000	2.09E-02	super_enhancer	promoter	RP11-388M20.6
chr16	8865000	chr16	31725000	1.57E-02	super_enhancer	promoter	ZNF720
chr16	8865000	chr16	89045000	3.55E-03	super_enhancer	promoter	CBFA2T3
chr16	8865000	chr16	90085000	3.83E-03	super_enhancer	promoter	GAS8
chr16	8875000	chr16	28075000	2.19E-02	super_enhancer	promoter	GSG1L

chr16	8875000	chr16	31565000	1.60E-02	super_enhancer	promoter	CTD-2014E2.6
chr16	8875000	chr16	54965000	7.13E-03	super_enhancer	promoter	IRX5
chr16	8875000	chr16	74585000	3.74E-03	super_enhancer	promoter	RP11-572F4.1
chr16	8885000	chr16	31885000	1.56E-02	super_enhancer	promoter	ZNF267
chr16	9055000	chr16	14245000	3.61E-02	super_enhancer	promoter	CTA-276F8.1
chr16	9055000	chr16	30125000	1.98E-02	super_enhancer	promoter	GDPD3
chr16	9055000	chr16	30905000	1.91E-02	super_enhancer	promoter	MIR762
chr16	9065000	chr16	10935000	4.70E-03	super_enhancer	promoter	RP11-876N24.1
chr16	9065000	chr16	11035000	1.71E-03	super_enhancer	promoter	RP11-876N24.5
chr16	9065000	chr16	14295000	4.42E-02	super_enhancer	promoter	CTA-276F8.2
chr16	9065000	chr16	30715000	1.86E-02	super_enhancer	promoter	RP11-2C24.9
chr16	9065000	chr16	31185000	1.82E-02	super_enhancer	promoter	NDUFA3P6
chr16	9065000	chr16	51185000	9.81E-03	super_enhancer	promoter	SALL1
chr16	9065000	chr16	66585000	7.58E-03	super_enhancer	promoter	CKLF
chr16	9075000	chr16	12705000	4.74E-02	super_enhancer	promoter	CTD-3037G24.3
chr16	9075000	chr16	30935000	1.85E-02	super_enhancer	promoter	FBXL19-AS1
chr16	9085000	chr16	10675000	4.70E-02	super_enhancer	promoter	EMP2
chr16	9085000	chr16	11035000	2.16E-02	super_enhancer	promoter	RP11-876N24.5
chr16	9085000	chr16	16465000	1.51E-02	super_enhancer	promoter	AC138969.1
chr16	9085000	chr16	19405000	3.14E-02	super_enhancer	promoter	CTA-363E6.3
chr16	9085000	chr16	21665000	2.05E-02	super_enhancer	promoter	IGSF6
chr16	9085000	chr16	28635000	1.93E-02	super_enhancer	promoter	SULT1A1
chr16	9085000	chr16	30035000	2.46E-02	super_enhancer	promoter	DOC2A
chr16	9085000	chr16	31615000	1.06E-02	super_enhancer	promoter	ZNF720P1

chr16	9085000	chr16	32375000	1.54E-02	super_enhancer	promoter	RP11-17M15.4
chr16	9095000	chr16	10675000	8.38E-04	super_enhancer	promoter	EMP2
chr16	9095000	chr16	10815000	1.99E-02	super_enhancer	promoter	RP11-109M19.4
chr16	9095000	chr16	15225000	3.51E-02	super_enhancer	promoter	AC139256.1
chr16	9095000	chr16	23585000	2.33E-02	super_enhancer	promoter	CTD-2196E14.7
chr16	9095000	chr16	29625000	1.53E-02	super_enhancer	promoter	SLC7A5P1
chr16	9095000	chr16	30935000	2.48E-02	super_enhancer	promoter	FBXL19-AS1
chr16	9095000	chr16	31365000	2.08E-02	super_enhancer	promoter	ITGAX
chr16	9095000	chr16	34255000	1.11E-02	super_enhancer	promoter	CTD-2144E22.5
chr16	9095000	chr16	75745000	4.49E-03	super_enhancer	promoter	ATP5F1P7
chr16	9105000	chr16	10815000	1.24E-03	super_enhancer	promoter	RP11-109M19.4
chr16	9105000	chr16	10935000	6.19E-03	super_enhancer	promoter	RP11-876N24.1
chr16	9105000	chr16	11165000	2.34E-02	super_enhancer	promoter	RP11-66H6.3
chr16	9105000	chr16	28075000	2.50E-02	super_enhancer	promoter	GSG1L
chr16	9105000	chr16	28635000	2.16E-02	super_enhancer	promoter	SULT1A1
chr16	9105000	chr16	30125000	2.55E-02	super_enhancer	promoter	GDPD3
chr16	9105000	chr16	30545000	2.21E-02	super_enhancer	promoter	AC002310.12
chr16	9105000	chr16	30585000	2.80E-02	super_enhancer	promoter	ZNF688
chr16	9105000	chr16	74585000	4.26E-03	super_enhancer	promoter	RP11-572F4.1
chr16	9115000	chr16	15705000	4.72E-02	super_enhancer	promoter	MIR6506
chr16	9115000	chr16	28565000	2.44E-02	super_enhancer	promoter	RP11-666O2.2
chr16	9115000	chr16	31105000	2.38E-02	super_enhancer	promoter	RP11-196G11.3
chr16	9115000	chr16	54325000	8.60E-03	super_enhancer	promoter	RP11-434E6.4
chr16	9125000	chr16	10675000	7.02E-03	super_enhancer	promoter	EMP2

chr16	9125000	chr16	11035000	2.60E-02	super_enhancer	promoter	RP11-876N24.5
chr16	9125000	chr16	28995000	2.98E-02	super_enhancer	promoter	LAT
chr16	9125000	chr16	34175000	6.86E-03	super_enhancer	promoter	CTD-2144E22.9
chr16	9185000	chr16	11605000	1.73E-02	promoter	super_enhancer	C16orf72
chr16	9185000	chr16	15675000	4.71E-02	promoter	super_enhancer	C16orf72
chr16	9185000	chr16	19125000	3.39E-02	promoter	super_enhancer	C16orf72
chr16	9185000	chr16	30785000	2.15E-02	promoter	super_enhancer	C16orf72
chr16	9185000	chr16	31065000	1.95E-02	promoter	super_enhancer	C16orf72
chr16	9185000	chr16	31075000	1.97E-02	promoter	super_enhancer	C16orf72
chr16	9185000	chr16	88755000	3.92E-03	promoter	super_enhancer	C16orf72
chr16	10205000	chr16	16025000	4.09E-02	promoter	super_enhancer	IMPDH1P11
chr16	10205000	chr16	30665000	2.24E-02	promoter	super_enhancer	IMPDH1P11
chr16	10205000	chr16	30785000	1.99E-02	promoter	super_enhancer	IMPDH1P11
chr16	10205000	chr16	57815000	6.34E-03	promoter	super_enhancer	IMPDH1P11
chr16	10205000	chr16	66395000	7.31E-03	promoter	super_enhancer	IMPDH1P11
chr16	10205000	chr16	81515000	3.30E-03	promoter	super_enhancer	IMPDH1P11
chr16	10545000	chr16	16025000	4.18E-02	promoter	super_enhancer	RP11-609N14.4
chr16	10545000	chr16	30655000	2.00E-02	promoter	super_enhancer	RP11-609N14.4
chr16	10545000	chr16	74745000	3.72E-03	promoter	super_enhancer	RP11-609N14.4
chr16	10675000	chr16	11635000	3.05E-02	promoter	super_enhancer	EMP2
chr16	10675000	chr16	19135000	3.78E-02	promoter	super_enhancer	EMP2
chr16	10675000	chr16	57815000	7.78E-03	promoter	super_enhancer	EMP2
chr16	10785000	chr16	15485000	4.93E-02	promoter	super_enhancer	RP11-109M19.1
chr16	10785000	chr16	56815000	4.66E-03	promoter	super_enhancer	RP11-109M19.1

chr16	10935000	chr16	66365000	7.32E-03	promoter	super_enhancer	RP11-876N24.1
chr16	11035000	chr16	29825000	2.30E-02	promoter	super_enhancer	RP11-876N24.5
chr16	11035000	chr16	30655000	2.30E-02	promoter	super_enhancer	RP11-876N24.5
chr16	11375000	chr16	57635000	8.14E-03	promoter	super_enhancer	PRM1
chr16	11375000	chr16	81455000	4.67E-03	promoter	super_enhancer	PRM1
chr16	11475000	chr16	31075000	2.22E-02	promoter	super_enhancer	CTD-3088G3.4
chr16	11605000	chr16	12185000	4.38E-02	super_enhancer	promoter	RP11-276H1.3
chr16	11605000	chr16	15835000	4.04E-03	super_enhancer	promoter	AF001548.6
chr16	11605000	chr16	23725000	3.13E-02	super_enhancer	promoter	ERN2
chr16	11605000	chr16	23765000	3.36E-02	super_enhancer	promoter	CHP2
chr16	11605000	chr16	24345000	3.10E-02	super_enhancer	promoter	SNORA1
chr16	11605000	chr16	29875000	2.98E-02	super_enhancer	promoter	CDIPT
chr16	11605000	chr16	30065000	3.29E-02	super_enhancer	promoter	FAM57B
chr16	11605000	chr16	31725000	2.20E-02	super_enhancer	promoter	ZNF720
chr16	11605000	chr16	34985000	1.54E-02	super_enhancer	promoter	RNA5SP416
chr16	11605000	chr16	56665000	9.24E-03	super_enhancer	promoter	MT1M
chr16	11605000	chr16	58915000	6.94E-03	super_enhancer	promoter	RP11-700H13.1
chr16	11605000	chr16	75415000	4.44E-03	super_enhancer	promoter	RP11-252K23.2
chr16	11615000	chr16	17565000	4.70E-02	super_enhancer	promoter	XYLT1
chr16	11615000	chr16	28195000	1.45E-02	super_enhancer	promoter	RNY1P10
chr16	11615000	chr16	31885000	1.57E-02	super_enhancer	promoter	ZNF267
chr16	11615000	chr16	46885000	9.79E-03	super_enhancer	promoter	RP11-480G7.3
chr16	11615000	chr16	48505000	6.10E-03	super_enhancer	promoter	RP11-44I10.5
chr16	11615000	chr16	66995000	5.83E-03	super_enhancer	promoter	CES3

chr16	11615000	chr16	67185000	5.27E-03	super_enhancer	promoter	B3GNT9
chr16	11625000	chr16	21205000	2.58E-02	super_enhancer	promoter	AF001550.7
chr16	11625000	chr16	21665000	2.15E-02	super_enhancer	promoter	IGSF6
chr16	11625000	chr16	23725000	2.43E-02	super_enhancer	promoter	ERN2
chr16	11625000	chr16	28875000	2.27E-02	super_enhancer	promoter	RP11-22P6.2
chr16	11625000	chr16	29435000	1.38E-02	super_enhancer	promoter	AC025279.1
chr16	11625000	chr16	30135000	2.58E-02	super_enhancer	promoter	MAPK3
chr16	11625000	chr16	71365000	3.76E-03	super_enhancer	promoter	AC106736.1
chr16	11635000	chr16	30135000	2.65E-02	super_enhancer	promoter	MAPK3
chr16	11635000	chr16	30495000	2.29E-02	super_enhancer	promoter	RNU7-61P
chr16	11835000	chr16	19105000	2.89E-02	promoter	super_enhancer	RP11-490O6.2
chr16	11835000	chr16	70715000	4.30E-03	promoter	super_enhancer	RP11-490O6.2
chr16	11945000	chr16	19115000	4.46E-02	promoter	super_enhancer	RSL1D1
chr16	11945000	chr16	19135000	4.37E-02	promoter	super_enhancer	RSL1D1
chr16	12175000	chr16	30665000	2.69E-02	promoter	super_enhancer	RPS23P6
chr16	12185000	chr16	31055000	2.47E-02	promoter	super_enhancer	RP11-276H1.3
chr16	12975000	chr16	14315000	2.83E-02	promoter	super_enhancer	RP11-14K2.1
chr16	12975000	chr16	27325000	2.08E-02	promoter	super_enhancer	RP11-14K2.1
chr16	12995000	chr16	14315000	3.18E-02	promoter	super_enhancer	SHISA9
chr16	14115000	chr16	27315000	1.77E-02	promoter	super_enhancer	CTD-2135D7.4
chr16	14165000	chr16	19135000	2.34E-03	promoter	super_enhancer	MKL2
chr16	14245000	chr16	19095000	3.95E-02	promoter	super_enhancer	CTA-276F8.1
chr16	14245000	chr16	27325000	1.98E-02	promoter	super_enhancer	CTA-276F8.1
chr16	14245000	chr16	56795000	4.83E-03	promoter	super_enhancer	CTA-276F8.1

chr16	14245000	chr16	57305000	6.26E-03	promoter	super_enhancer	CTA-276F8.1
chr16	14295000	chr16	30895000	1.41E-02	promoter	super_enhancer	CTA-276F8.2
chr16	14295000	chr16	84585000	3.57E-03	promoter	super_enhancer	CTA-276F8.2
chr16	14315000	chr16	24345000	2.31E-02	super_enhancer	promoter	SNORA1
chr16	14315000	chr16	29675000	2.18E-02	super_enhancer	promoter	AC009133.22
chr16	14315000	chr16	85475000	4.15E-03	super_enhancer	promoter	AC092377.1
chr16	14315000	chr16	87635000	3.38E-03	super_enhancer	promoter	JPH3
chr16	14465000	chr16	15615000	2.06E-02	promoter	super_enhancer	RP11-65J21.1
chr16	14465000	chr16	31455000	2.77E-02	promoter	super_enhancer	RP11-65J21.1
chr16	14465000	chr16	73075000	4.80E-03	promoter	super_enhancer	RP11-65J21.1
chr16	14995000	chr16	15655000	4.57E-02	promoter	super_enhancer	MIR3179-1
chr16	15085000	chr16	15605000	2.03E-02	promoter	super_enhancer	RP11-680G24.4
chr16	15085000	chr16	30785000	3.23E-02	promoter	super_enhancer	RP11-680G24.4
chr16	15085000	chr16	57615000	6.68E-03	promoter	super_enhancer	RP11-680G24.4
chr16	15085000	chr16	66525000	8.31E-03	promoter	super_enhancer	RP11-680G24.4
chr16	15485000	chr16	22625000	2.95E-02	super_enhancer	promoter	RP11-105C19.2
chr16	15485000	chr16	28635000	1.74E-04	super_enhancer	promoter	SULT1A1
chr16	15485000	chr16	31105000	2.08E-02	super_enhancer	promoter	RP11-196G11.3
chr16	15485000	chr16	31185000	1.65E-02	super_enhancer	promoter	NDUFA3P6
chr16	15495000	chr16	31365000	2.26E-02	super_enhancer	promoter	ITGAX
chr16	15495000	chr16	50775000	9.90E-03	super_enhancer	promoter	RP11-327F22.2
chr16	15505000	chr16	15705000	1.14E-02	super_enhancer	promoter	MIR6506
chr16	15505000	chr16	18295000	4.35E-02	super_enhancer	promoter	CTD-2036A2.1
chr16	15505000	chr16	30035000	3.09E-02	super_enhancer	promoter	DOC2A

chr16	15505000	chr16	34255000	1.15E-02	super_enhancer	promoter	CTD-2144E22.5
chr16	15505000	chr16	68035000	6.33E-03	super_enhancer	promoter	DPEP2
chr16	15575000	chr16	28565000	3.20E-02	super_enhancer	promoter	RP11-666O2.2
chr16	15575000	chr16	29675000	3.15E-02	super_enhancer	promoter	AC009133.22
chr16	15575000	chr16	31565000	2.29E-02	super_enhancer	promoter	CTD-2014E2.6
chr16	15575000	chr16	31715000	2.53E-02	super_enhancer	promoter	CTD-2358C21.5
chr16	15575000	chr16	85825000	4.82E-03	super_enhancer	promoter	RP11-568J23.6
chr16	15585000	chr16	15705000	2.19E-02	super_enhancer	promoter	MIR6506
chr16	15585000	chr16	28855000	2.87E-02	super_enhancer	promoter	MIR4721
chr16	15585000	chr16	31085000	3.01E-02	super_enhancer	promoter	ZNF646
chr16	15585000	chr16	89725000	4.57E-03	super_enhancer	promoter	CHMP1A
chr16	15585000	chr16	89885000	4.23E-03	super_enhancer	promoter	SPIRE2
chr16	15595000	chr16	25085000	2.59E-02	super_enhancer	promoter	RP11-266L9.6
chr16	15595000	chr16	30495000	2.63E-02	super_enhancer	promoter	RNU7-61P
chr16	15595000	chr16	31105000	2.65E-02	super_enhancer	promoter	RP11-196G11.3
chr16	15595000	chr16	31195000	2.66E-02	super_enhancer	promoter	RP11-388M20.6
chr16	15595000	chr16	31725000	1.99E-02	super_enhancer	promoter	ZNF720
chr16	15595000	chr16	57125000	9.53E-03	super_enhancer	promoter	CPNE2
chr16	15605000	chr16	23765000	3.62E-02	super_enhancer	promoter	CHP2
chr16	15605000	chr16	28855000	2.95E-02	super_enhancer	promoter	MIR4721
chr16	15605000	chr16	29625000	1.76E-02	super_enhancer	promoter	SLC7A5P1
chr16	15605000	chr16	31195000	2.95E-02	super_enhancer	promoter	RP11-388M20.6
chr16	15605000	chr16	31215000	3.06E-02	super_enhancer	promoter	PYCARD
chr16	15605000	chr16	51185000	1.16E-02	super_enhancer	promoter	SALL1

chr16	15615000	chr16	21205000	4.11E-02	super_enhancer	promoter	AF001550.7
chr16	15615000	chr16	28565000	2.79E-02	super_enhancer	promoter	RP11-666O2.2
chr16	15615000	chr16	30135000	3.09E-02	super_enhancer	promoter	MAPK3
chr16	15615000	chr16	30495000	2.67E-02	super_enhancer	promoter	RNU7-61P
chr16	15615000	chr16	30905000	2.69E-02	super_enhancer	promoter	MIR762
chr16	15615000	chr16	31185000	2.13E-02	super_enhancer	promoter	NDUFA3P6
chr16	15615000	chr16	31875000	1.74E-02	super_enhancer	promoter	RBM22P13
chr16	15615000	chr16	49745000	7.79E-03	super_enhancer	promoter	CTD-2595P9.2
chr16	15615000	chr16	72025000	3.88E-03	super_enhancer	promoter	RPL39P31
chr16	15655000	chr16	23075000	2.58E-02	super_enhancer	promoter	RP11-20G6.3
chr16	15655000	chr16	27875000	2.62E-02	super_enhancer	promoter	RNU6-159P
chr16	15655000	chr16	28995000	3.41E-02	super_enhancer	promoter	LAT
chr16	15655000	chr16	47485000	6.91E-03	super_enhancer	promoter	RNA5SP424
chr16	15655000	chr16	73265000	5.06E-03	super_enhancer	promoter	AC140912.1
chr16	15665000	chr16	22385000	2.89E-02	super_enhancer	promoter	RP11-21M24.2
chr16	15665000	chr16	23465000	3.15E-02	super_enhancer	promoter	COG7
chr16	15665000	chr16	29985000	2.63E-02	super_enhancer	promoter	TAOK2
chr16	15665000	chr16	30515000	2.72E-02	super_enhancer	promoter	MIR4518
chr16	15665000	chr16	30935000	2.68E-02	super_enhancer	promoter	FBXL19-AS1
chr16	15665000	chr16	34985000	1.45E-02	super_enhancer	promoter	RNA5SP416
chr16	15665000	chr16	53425000	7.01E-03	super_enhancer	promoter	RP11-44F14.10
chr16	15675000	chr16	18575000	4.61E-02	super_enhancer	promoter	NOMO2
chr16	15675000	chr16	30905000	2.97E-02	super_enhancer	promoter	MIR762
chr16	15675000	chr16	31195000	2.99E-02	super_enhancer	promoter	RP11-388M20.6

chr16	15675000	chr16	33605000	1.98E-02	super_enhancer	promoter	IGHV3OR16-12
chr16	15675000	chr16	57495000	9.04E-03	super_enhancer	promoter	AC009052.12
chr16	15675000	chr16	67515000	7.03E-03	super_enhancer	promoter	ATP6V0D1
chr16	15675000	chr16	67575000	6.75E-03	super_enhancer	promoter	CTD-2012K14.2
chr16	15675000	chr16	69665000	5.78E-03	super_enhancer	promoter	RP11-140H17.2
chr16	15675000	chr16	71335000	5.16E-03	super_enhancer	promoter	CTD-2520I13.1
chr16	15705000	chr16	30785000	2.74E-02	promoter	super_enhancer	MIR6506
chr16	15705000	chr16	81525000	3.80E-03	promoter	super_enhancer	MIR6506
chr16	15795000	chr16	56805000	7.41E-03	promoter	super_enhancer	CTB-193M12.5
chr16	15835000	chr16	27325000	3.05E-02	promoter	super_enhancer	AF001548.6
chr16	15835000	chr16	57265000	5.96E-03	promoter	super_enhancer	AF001548.6
chr16	15995000	chr16	28565000	2.98E-02	super_enhancer	promoter	RP11-666O2.2
chr16	15995000	chr16	29215000	2.40E-02	super_enhancer	promoter	RP11-426C22.1
chr16	15995000	chr16	68295000	8.10E-03	super_enhancer	promoter	RP11-96D1.7
chr16	15995000	chr16	68345000	6.81E-03	super_enhancer	promoter	SLC7A6OS
chr16	16005000	chr16	57495000	8.77E-03	super_enhancer	promoter	AC009052.12
chr16	16005000	chr16	64295000	3.86E-03	super_enhancer	promoter	RP11-744D14.1
chr16	16005000	chr16	85475000	5.43E-03	super_enhancer	promoter	AC092377.1
chr16	16015000	chr16	22625000	4.39E-02	super_enhancer	promoter	RP11-105C19.2
chr16	16015000	chr16	23725000	3.46E-02	super_enhancer	promoter	ERN2
chr16	16015000	chr16	29625000	1.76E-02	super_enhancer	promoter	SLC7A5P1
chr16	16015000	chr16	33945000	2.98E-02	super_enhancer	promoter	DUX4L46
chr16	16015000	chr16	56765000	7.81E-03	super_enhancer	promoter	RP11-343H19.1
chr16	16015000	chr16	67195000	6.76E-03	super_enhancer	promoter	TRADD

chr16	16015000	chr16	68015000	7.74E-03	super_enhancer	promoter	DPEP3
chr16	16015000	chr16	75735000	6.24E-03	super_enhancer	promoter	RP11-490B18.7
chr16	16025000	chr16	25105000	2.60E-02	super_enhancer	promoter	RP11-266L9.3
chr16	16025000	chr16	31085000	2.78E-02	super_enhancer	promoter	ZNF646
chr16	16025000	chr16	47485000	6.84E-03	super_enhancer	promoter	RNA5SP424
chr16	16025000	chr16	48685000	7.08E-03	super_enhancer	promoter	AC007611.1
chr16	16035000	chr16	22105000	4.86E-02	super_enhancer	promoter	RP11-101E7.2
chr16	16035000	chr16	28875000	3.46E-02	super_enhancer	promoter	RP11-22P6.2
chr16	16035000	chr16	30065000	3.86E-02	super_enhancer	promoter	FAM57B
chr16	16035000	chr16	30515000	3.32E-02	super_enhancer	promoter	MIR4518
chr16	16035000	chr16	30905000	5.83E-04	super_enhancer	promoter	MIR762
chr16	16035000	chr16	32925000	1.14E-02	super_enhancer	promoter	IGHV3OR16-6
chr16	16035000	chr16	48885000	1.30E-02	super_enhancer	promoter	RP11-42I10.3
chr16	16035000	chr16	70115000	4.31E-03	super_enhancer	promoter	RP11-106J23.1
chr16	16035000	chr16	84225000	4.70E-03	super_enhancer	promoter	ADAD2
chr16	16465000	chr16	31465000	1.08E-02	promoter	super_enhancer	AC138969.1
chr16	16465000	chr16	66535000	1.65E-03	promoter	super_enhancer	AC138969.1
chr16	16855000	chr16	67245000	3.54E-03	promoter	super_enhancer	RP11-14N9.1
chr16	17565000	chr16	66515000	7.78E-03	promoter	super_enhancer	XYLT1
chr16	17565000	chr16	81465000	5.13E-03	promoter	super_enhancer	XYLT1
chr16	17635000	chr16	31455000	3.53E-04	promoter	super_enhancer	RP11-916L7.1
chr16	18055000	chr16	84515000	4.93E-03	promoter	super_enhancer	CTA-481E9.2
chr16	18575000	chr16	29825000	1.23E-02	promoter	super_enhancer	NOMO2
chr16	19095000	chr16	19405000	1.68E-03	super_enhancer	promoter	CTA-363E6.3

chr16	19095000	chr16	19535000	1.52E-04	super_enhancer	promoter	CCP110
chr16	19095000	chr16	27655000	2.73E-02	super_enhancer	promoter	AC002551.1
chr16	19095000	chr16	28875000	3.07E-02	super_enhancer	promoter	RP11-22P6.2
chr16	19095000	chr16	28985000	6.12E-04	super_enhancer	promoter	SPNS1
chr16	19095000	chr16	46605000	8.48E-03	super_enhancer	promoter	RP11-46D6.5
chr16	19095000	chr16	49375000	1.02E-02	super_enhancer	promoter	RP11-491F9.3
chr16	19095000	chr16	69795000	4.96E-03	super_enhancer	promoter	WWP2
chr16	19105000	chr16	19405000	5.67E-04	super_enhancer	promoter	CTA-363E6.3
chr16	19105000	chr16	19515000	3.26E-02	super_enhancer	promoter	CTA-363E6.8
chr16	19105000	chr16	19535000	3.27E-02	super_enhancer	promoter	CCP110
chr16	19105000	chr16	27465000	3.02E-02	super_enhancer	promoter	IL21R-AS1
chr16	19105000	chr16	27875000	3.20E-02	super_enhancer	promoter	RNU6-159P
chr16	19105000	chr16	28565000	5.57E-04	super_enhancer	promoter	RP11-666O2.2
chr16	19105000	chr16	29435000	1.95E-02	super_enhancer	promoter	AC025279.1
chr16	19105000	chr16	30515000	3.09E-02	super_enhancer	promoter	MIR4518
chr16	19105000	chr16	31185000	2.50E-02	super_enhancer	promoter	NDUFA3P6
chr16	19105000	chr16	31195000	3.17E-02	super_enhancer	promoter	RP11-388M20.6
chr16	19105000	chr16	49745000	8.67E-03	super_enhancer	promoter	CTD-2595P9.2
chr16	19115000	chr16	19535000	2.09E-03	super_enhancer	promoter	CCP110
chr16	19115000	chr16	28195000	2.25E-02	super_enhancer	promoter	RNY1P10
chr16	19115000	chr16	28565000	3.51E-02	super_enhancer	promoter	RP11-666O2.2
chr16	19115000	chr16	28875000	3.42E-02	super_enhancer	promoter	RP11-22P6.2
chr16	19115000	chr16	30585000	3.83E-02	super_enhancer	promoter	ZNF688
chr16	19115000	chr16	30715000	2.72E-02	super_enhancer	promoter	RP11-2C24.9

chr16	19115000	chr16	31105000	3.34E-02	super_enhancer	promoter	RP11-196G11.3
chr16	19115000	chr16	46865000	1.15E-02	super_enhancer	promoter	C16orf87
chr16	19115000	chr16	56685000	1.00E-02	super_enhancer	promoter	MT1B
chr16	19125000	chr16	19405000	1.50E-03	super_enhancer	promoter	CTA-363E6.3
chr16	19125000	chr16	26075000	3.33E-02	super_enhancer	promoter	HSPE1P16
chr16	19125000	chr16	28565000	3.67E-02	super_enhancer	promoter	RP11-666O2.2
chr16	19125000	chr16	28875000	3.58E-02	super_enhancer	promoter	RP11-22P6.2
chr16	19125000	chr16	29415000	1.59E-02	super_enhancer	promoter	NPIPB11
chr16	19125000	chr16	29435000	2.16E-02	super_enhancer	promoter	AC025279.1
chr16	19125000	chr16	30495000	3.48E-02	super_enhancer	promoter	RNU7-61P
chr16	19125000	chr16	30715000	2.85E-02	super_enhancer	promoter	RP11-2C24.9
chr16	19125000	chr16	31215000	3.64E-02	super_enhancer	promoter	PYCARD
chr16	19125000	chr16	31405000	2.96E-02	super_enhancer	promoter	ITGAD
chr16	19125000	chr16	66585000	9.55E-03	super_enhancer	promoter	CKLF
chr16	19135000	chr16	19325000	2.12E-02	super_enhancer	promoter	CTA-363E6.1
chr16	19135000	chr16	24345000	4.97E-02	super_enhancer	promoter	SNORA1
chr16	19135000	chr16	25035000	4.33E-02	super_enhancer	promoter	AC133552.1
chr16	19135000	chr16	28565000	3.45E-02	super_enhancer	promoter	RP11-666O2.2
chr16	19135000	chr16	28835000	3.13E-02	super_enhancer	promoter	RP11-1348G14.8
chr16	19135000	chr16	28875000	3.36E-02	super_enhancer	promoter	RP11-22P6.2
chr16	19135000	chr16	28995000	4.24E-02	super_enhancer	promoter	LAT
chr16	19135000	chr16	29085000	2.01E-02	super_enhancer	promoter	RRN3P2
chr16	19135000	chr16	31875000	2.12E-02	super_enhancer	promoter	RBM22P13
chr16	19135000	chr16	50185000	1.19E-02	super_enhancer	promoter	RPL10P14

chr16	19135000	chr16	57575000	9.12E-03	super_enhancer	promoter	ADGRG5
chr16	19325000	chr16	29825000	3.65E-02	promoter	super_enhancer	CTA-363E6.1
chr16	19325000	chr16	30785000	3.52E-02	promoter	super_enhancer	CTA-363E6.1
chr16	19325000	chr16	57845000	1.12E-02	promoter	super_enhancer	CTA-363E6.1
chr16	19325000	chr16	82665000	5.60E-03	promoter	super_enhancer	CTA-363E6.1
chr16	19325000	chr16	89335000	4.06E-03	promoter	super_enhancer	CTA-363E6.1
chr16	19405000	chr16	30665000	3.69E-02	promoter	super_enhancer	CTA-363E6.3
chr16	19515000	chr16	56605000	7.38E-03	promoter	super_enhancer	CTA-363E6.8
chr16	19535000	chr16	70685000	5.76E-03	promoter	super_enhancer	CCP110
chr16	20085000	chr16	27315000	2.03E-02	promoter	super_enhancer	GPR139
chr16	20795000	chr16	66355000	6.65E-03	promoter	super_enhancer	RNU6-944P
chr16	20835000	chr16	81475000	3.67E-03	promoter	super_enhancer	U4atac
chr16	21205000	chr16	31055000	3.27E-02	promoter	super_enhancer	AF001550.7
chr16	21205000	chr16	56615000	8.45E-03	promoter	super_enhancer	AF001550.7
chr16	21205000	chr16	57665000	6.97E-03	promoter	super_enhancer	AF001550.7
chr16	21205000	chr16	66395000	8.90E-03	promoter	super_enhancer	AF001550.7
chr16	21315000	chr16	57795000	6.29E-03	promoter	super_enhancer	CRYM
chr16	21315000	chr16	81455000	2.70E-03	promoter	super_enhancer	CRYM
chr16	21515000	chr16	29215000	2.83E-02	super_enhancer	promoter	RP11-426C22.1
chr16	21515000	chr16	29675000	3.37E-02	super_enhancer	promoter	AC009133.22
chr16	21515000	chr16	30495000	3.17E-02	super_enhancer	promoter	RNU7-61P
chr16	21515000	chr16	31405000	2.67E-02	super_enhancer	promoter	ITGAD
chr16	21515000	chr16	31885000	2.20E-02	super_enhancer	promoter	ZNF267
chr16	21515000	chr16	57405000	8.85E-03	super_enhancer	promoter	CX3CL1

chr16	21665000	chr16	84595000	3.70E-03	promoter	super_enhancer	IGSF6
chr16	22375000	chr16	27325000	3.82E-02	promoter	super_enhancer	RP11-21M24.3
chr16	22375000	chr16	30895000	1.85E-02	promoter	super_enhancer	RP11-21M24.3
chr16	22375000	chr16	88785000	3.63E-03	promoter	super_enhancer	RP11-21M24.3
chr16	22625000	chr16	31465000	3.85E-02	promoter	super_enhancer	RP11-105C19.2
chr16	22625000	chr16	66395000	1.08E-02	promoter	super_enhancer	RP11-105C19.2
chr16	22625000	chr16	66505000	9.06E-03	promoter	super_enhancer	RP11-105C19.2
chr16	23225000	chr16	66375000	6.88E-03	promoter	super_enhancer	CTC-391G2.1
chr16	23465000	chr16	31455000	3.14E-02	promoter	super_enhancer	COG7
chr16	23585000	chr16	27315000	3.73E-02	promoter	super_enhancer	CTD-2196E14.7
chr16	23585000	chr16	30665000	7.64E-04	promoter	super_enhancer	CTD-2196E14.7
chr16	23585000	chr16	66385000	5.36E-03	promoter	super_enhancer	CTD-2196E14.7
chr16	23765000	chr16	30655000	4.61E-02	promoter	super_enhancer	CHP2
chr16	23765000	chr16	66535000	5.10E-03	promoter	super_enhancer	CHP2
chr16	25085000	chr16	82675000	3.60E-03	promoter	super_enhancer	RP11-266L9.6
chr16	25105000	chr16	31075000	4.20E-02	promoter	super_enhancer	RP11-266L9.3
chr16	25105000	chr16	56985000	9.92E-03	promoter	super_enhancer	RP11-266L9.3
chr16	25105000	chr16	75235000	5.52E-03	promoter	super_enhancer	RP11-266L9.3
chr16	26045000	chr16	27325000	4.69E-02	promoter	super_enhancer	HMGN2P3
chr16	26045000	chr16	31055000	1.65E-03	promoter	super_enhancer	HMGN2P3
chr16	27315000	chr16	51795000	4.73E-03	super_enhancer	promoter	LINC01571
chr16	27315000	chr16	55365000	7.01E-03	super_enhancer	promoter	RP11-26L20.4
chr16	27315000	chr16	56715000	7.20E-03	super_enhancer	promoter	MT1X
chr16	27325000	chr16	34255000	2.30E-02	super_enhancer	promoter	CTD-2144E22.5

chr16	27325000	chr16	48685000	1.12E-02	super_enhancer	promoter	AC007611.1
chr16	27325000	chr16	57405000	1.25E-02	super_enhancer	promoter	CX3CL1
chr16	27655000	chr16	70745000	7.02E-03	promoter	super_enhancer	AC002551.1
chr16	28075000	chr16	57625000	9.97E-03	promoter	super_enhancer	GSG1L
chr16	28075000	chr16	70745000	8.53E-03	promoter	super_enhancer	GSG1L
chr16	28635000	chr16	56815000	6.73E-03	promoter	super_enhancer	SULT1A1
chr16	28635000	chr16	66385000	7.50E-03	promoter	super_enhancer	SULT1A1
chr16	28835000	chr16	73065000	6.12E-03	promoter	super_enhancer	RP11-1348G14.8
chr16	28835000	chr16	88785000	6.24E-03	promoter	super_enhancer	RP11-1348G14.8
chr16	28855000	chr16	56605000	1.25E-02	promoter	super_enhancer	MIR4721
chr16	28855000	chr16	57625000	1.02E-02	promoter	super_enhancer	MIR4721
chr16	28855000	chr16	70435000	9.22E-03	promoter	super_enhancer	MIR4721
chr16	28855000	chr16	88785000	6.63E-03	promoter	super_enhancer	MIR4721
chr16	28875000	chr16	57845000	1.49E-02	promoter	super_enhancer	RP11-22P6.2
chr16	28995000	chr16	57655000	1.92E-02	promoter	super_enhancer	LAT
chr16	29085000	chr16	66395000	8.38E-03	promoter	super_enhancer	RRN3P2
chr16	29215000	chr16	30665000	7.74E-03	promoter	super_enhancer	RP11-426C22.1
chr16	29215000	chr16	57625000	8.79E-03	promoter	super_enhancer	RP11-426C22.1
chr16	29215000	chr16	81465000	5.69E-03	promoter	super_enhancer	RP11-426C22.1
chr16	29215000	chr16	85525000	4.40E-03	promoter	super_enhancer	RP11-426C22.1
chr16	29435000	chr16	57835000	8.55E-03	promoter	super_enhancer	AC025279.1
chr16	29625000	chr16	57825000	7.90E-03	promoter	super_enhancer	SLC7A5P1
chr16	29625000	chr16	89305000	3.26E-03	promoter	super_enhancer	SLC7A5P1
chr16	29635000	chr16	57655000	9.25E-03	promoter	super_enhancer	RP11-368N21.1

chr16	29825000	chr16	29985000	6.64E-03	super_enhancer	promoter	TAOK2
chr16	29825000	chr16	81215000	6.70E-03	super_enhancer	promoter	AC092718.1
chr16	29875000	chr16	57645000	1.34E-02	promoter	super_enhancer	CDIPT
chr16	29875000	chr16	85555000	6.71E-03	promoter	super_enhancer	CDIPT
chr16	29985000	chr16	84505000	3.26E-03	promoter	super_enhancer	TAOK2
chr16	30035000	chr16	30655000	4.31E-03	promoter	super_enhancer	DOC2A
chr16	30035000	chr16	69405000	1.15E-02	promoter	super_enhancer	DOC2A
chr16	30065000	chr16	70705000	1.06E-02	promoter	super_enhancer	FAM57B
chr16	30125000	chr16	30665000	8.67E-03	promoter	super_enhancer	GDPD3
chr16	30125000	chr16	85525000	5.86E-03	promoter	super_enhancer	GDPD3
chr16	30495000	chr16	57845000	1.65E-02	promoter	super_enhancer	RNU7-61P
chr16	30515000	chr16	56985000	1.54E-02	promoter	super_enhancer	MIR4518
chr16	30515000	chr16	57815000	1.39E-02	promoter	super_enhancer	MIR4518
chr16	30515000	chr16	84995000	6.59E-03	promoter	super_enhancer	MIR4518
chr16	30545000	chr16	57675000	1.29E-02	promoter	super_enhancer	AC002310.12
chr16	30545000	chr16	57805000	1.31E-02	promoter	super_enhancer	AC002310.12
chr16	30545000	chr16	57835000	1.37E-02	promoter	super_enhancer	AC002310.12
chr16	30585000	chr16	56975000	1.29E-02	promoter	super_enhancer	ZNF688
chr16	30585000	chr16	56995000	1.90E-02	promoter	super_enhancer	ZNF688
chr16	30585000	chr16	57825000	1.64E-02	promoter	super_enhancer	ZNF688
chr16	30655000	chr16	30715000	9.19E-03	super_enhancer	promoter	RP11-2C24.9
chr16	30655000	chr16	50775000	1.89E-02	super_enhancer	promoter	RP11-327F22.2
chr16	30655000	chr16	66585000	1.42E-02	super_enhancer	promoter	CKLF
chr16	30655000	chr16	71115000	1.06E-02	super_enhancer	promoter	RP11-23E19.1

chr16	30665000	chr16	66615000	1.31E-02	super_enhancer	promoter	RP11-403P17.2
chr16	30715000	chr16	30895000	4.95E-02	promoter	super_enhancer	RP11-2C24.9
chr16	30775000	chr16	48505000	1.52E-02	super_enhancer	promoter	RP11-44I10.5
chr16	30775000	chr16	75495000	9.63E-03	super_enhancer	promoter	RP11-77K12.3
chr16	30785000	chr16	31365000	3.08E-02	super_enhancer	promoter	ITGAX
chr16	30785000	chr16	57495000	1.52E-02	super_enhancer	promoter	AC009052.12
chr16	30895000	chr16	34255000	4.26E-02	super_enhancer	promoter	CTD-2144E22.5
chr16	30895000	chr16	67575000	7.66E-03	super_enhancer	promoter	CTD-2012K14.2
chr16	30895000	chr16	71365000	5.11E-03	super_enhancer	promoter	AC106736.1
chr16	30905000	chr16	57635000	1.34E-02	promoter	super_enhancer	MIR762
chr16	30935000	chr16	56975000	1.11E-02	promoter	super_enhancer	FBXL19-AS1
chr16	30935000	chr16	57815000	1.42E-02	promoter	super_enhancer	FBXL19-AS1
chr16	30935000	chr16	74725000	8.51E-03	promoter	super_enhancer	FBXL19-AS1
chr16	30935000	chr16	84515000	7.17E-03	promoter	super_enhancer	FBXL19-AS1
chr16	31055000	chr16	31085000	4.89E-02	super_enhancer	promoter	ZNF646
chr16	31055000	chr16	31185000	1.19E-02	super_enhancer	promoter	NDUFA3P6
chr16	31055000	chr16	31875000	6.37E-03	super_enhancer	promoter	RBM22P13
chr16	31055000	chr16	48885000	2.28E-02	super_enhancer	promoter	RP11-42I10.3
chr16	31055000	chr16	75285000	8.44E-03	super_enhancer	promoter	RP11-331F4.5
chr16	31065000	chr16	31875000	2.46E-02	super_enhancer	promoter	RBM22P13
chr16	31065000	chr16	35115000	3.96E-02	super_enhancer	promoter	RP11-43P5.1
chr16	31065000	chr16	46605000	1.53E-02	super_enhancer	promoter	RP11-46D6.5
chr16	31065000	chr16	80575000	7.37E-03	super_enhancer	promoter	DYNLRB2
chr16	31065000	chr16	81215000	6.39E-03	super_enhancer	promoter	AC092718.1

chr16	31065000	chr16	81645000	6.16E-03	super_enhancer	promoter	MIR6504
chr16	31075000	chr16	31885000	5.03E-03	super_enhancer	promoter	ZNF267
chr16	31075000	chr16	34765000	4.32E-02	super_enhancer	promoter	RP11-80F22.8
chr16	31075000	chr16	67425000	1.17E-02	super_enhancer	promoter	RNU1-123P
chr16	31075000	chr16	67595000	8.76E-03	super_enhancer	promoter	CTCF
chr16	31075000	chr16	75735000	9.06E-03	super_enhancer	promoter	RP11-490B18.7
chr16	31075000	chr16	88705000	4.87E-03	super_enhancer	promoter	IL17C
chr16	31085000	chr16	66395000	1.70E-02	promoter	super_enhancer	ZNF646
chr16	31105000	chr16	66395000	1.61E-02	promoter	super_enhancer	RP11-196G11.3
chr16	31185000	chr16	31455000	1.30E-02	promoter	super_enhancer	NDUFA3P6
chr16	31185000	chr16	70685000	8.33E-03	promoter	super_enhancer	NDUFA3P6
chr16	31185000	chr16	82675000	4.54E-03	promoter	super_enhancer	NDUFA3P6
chr16	31195000	chr16	57625000	1.23E-02	promoter	super_enhancer	RP11-388M20.6
chr16	31195000	chr16	66385000	1.02E-02	promoter	super_enhancer	RP11-388M20.6
chr16	31215000	chr16	31455000	2.06E-02	promoter	super_enhancer	PYCARD
chr16	31215000	chr16	31465000	1.25E-02	promoter	super_enhancer	PYCARD
chr16	31215000	chr16	56975000	1.23E-02	promoter	super_enhancer	PYCARD
chr16	31215000	chr16	81515000	7.02E-03	promoter	super_enhancer	PYCARD
chr16	31225000	chr16	31465000	6.11E-03	promoter	super_enhancer	TRIM72
chr16	31225000	chr16	57795000	1.86E-02	promoter	super_enhancer	TRIM72
chr16	31225000	chr16	70765000	8.33E-03	promoter	super_enhancer	TRIM72
chr16	31225000	chr16	74725000	9.79E-03	promoter	super_enhancer	TRIM72
chr16	31225000	chr16	85495000	7.52E-03	promoter	super_enhancer	TRIM72
chr16	31225000	chr16	85505000	8.31E-03	promoter	super_enhancer	TRIM72

chr16	31225000	chr16	85565000	7.16E-03	promoter	super_enhancer	TRIM72
chr16	31225000	chr16	86395000	7.24E-03	promoter	super_enhancer	TRIM72
chr16	31365000	chr16	70415000	7.23E-03	promoter	super_enhancer	ITGAX
chr16	31455000	chr16	31875000	2.91E-02	super_enhancer	promoter	RBM22P13
chr16	31455000	chr16	46885000	2.13E-02	super_enhancer	promoter	RP11-480G7.3
chr16	31465000	chr16	31715000	3.80E-02	super_enhancer	promoter	CTD-2358C21.5
chr16	31465000	chr16	58665000	1.23E-02	super_enhancer	promoter	CNOT1
chr16	31465000	chr16	69165000	8.60E-03	super_enhancer	promoter	CIRH1A
chr16	31465000	chr16	75035000	7.28E-03	super_enhancer	promoter	WDR59
chr16	31565000	chr16	57675000	1.15E-02	promoter	super_enhancer	CTD-2014E2.6
chr16	31565000	chr16	66505000	1.04E-02	promoter	super_enhancer	CTD-2014E2.6
chr16	31615000	chr16	82635000	2.64E-03	promoter	super_enhancer	ZNF720P1
chr16	31715000	chr16	56815000	7.89E-03	promoter	super_enhancer	CTD-2358C21.5
chr16	31715000	chr16	70705000	8.68E-03	promoter	super_enhancer	CTD-2358C21.5
chr16	31725000	chr16	81515000	5.24E-03	promoter	super_enhancer	ZNF720
chr16	31815000	chr16	70415000	6.11E-03	promoter	super_enhancer	VN1R67P
chr16	31875000	chr16	57675000	1.02E-02	promoter	super_enhancer	RBM22P13
chr16	31885000	chr16	56985000	1.23E-02	promoter	super_enhancer	ZNF267
chr16	32915000	chr16	57295000	8.84E-03	promoter	super_enhancer	IGHV3OR16-15
chr16	32915000	chr16	70415000	4.16E-03	promoter	super_enhancer	IGHV3OR16-15
chr16	32915000	chr16	70425000	4.82E-03	promoter	super_enhancer	IGHV3OR16-15
chr16	32915000	chr16	84505000	1.74E-03	promoter	super_enhancer	IGHV3OR16-15
chr16	34175000	chr16	57285000	3.76E-03	promoter	super_enhancer	CTD-2144E22.9
chr16	34175000	chr16	84615000	2.73E-03	promoter	super_enhancer	CTD-2144E22.9

chr16	34275000	chr16	81505000	3.81E-03	promoter	super_enhancer	CCNYL3
chr16	34325000	chr16	67245000	3.75E-03	promoter	super_enhancer	CTD-2144E22.10
chr16	34655000	chr16	81525000	2.26E-03	promoter	super_enhancer	AGGF1P5
chr16	34715000	chr16	66515000	6.21E-03	promoter	super_enhancer	FRG2DP
chr16	34765000	chr16	66355000	5.98E-03	promoter	super_enhancer	RP11-80F22.8
chr16	34985000	chr16	57275000	8.17E-03	promoter	super_enhancer	RNA5SP416
chr16	34985000	chr16	66515000	8.58E-03	promoter	super_enhancer	RNA5SP416
chr16	35115000	chr16	74725000	4.48E-03	promoter	super_enhancer	RP11-43P5.1
chr16	46605000	chr16	66505000	1.21E-02	promoter	super_enhancer	RP11-46D6.5
chr16	46605000	chr16	70425000	8.85E-03	promoter	super_enhancer	RP11-46D6.5
chr16	46605000	chr16	86395000	5.19E-03	promoter	super_enhancer	RP11-46D6.5
chr16	46825000	chr16	56605000	1.67E-02	promoter	super_enhancer	MYLK3
chr16	46825000	chr16	57295000	2.05E-02	promoter	super_enhancer	MYLK3
chr16	46825000	chr16	57795000	1.86E-02	promoter	super_enhancer	MYLK3
chr16	46825000	chr16	57835000	1.74E-02	promoter	super_enhancer	MYLK3
chr16	46825000	chr16	66395000	1.57E-02	promoter	super_enhancer	MYLK3
chr16	46825000	chr16	70695000	7.21E-03	promoter	super_enhancer	MYLK3
chr16	46825000	chr16	84585000	6.76E-03	promoter	super_enhancer	MYLK3
chr16	46865000	chr16	57795000	2.11E-02	promoter	super_enhancer	C16orf87
chr16	46865000	chr16	66365000	1.22E-02	promoter	super_enhancer	C16orf87
chr16	46865000	chr16	73035000	9.65E-03	promoter	super_enhancer	C16orf87
chr16	46865000	chr16	89335000	5.34E-03	promoter	super_enhancer	C16orf87
chr16	46885000	chr16	56965000	3.17E-02	promoter	super_enhancer	RP11-480G7.3
chr16	46885000	chr16	70695000	9.50E-03	promoter	super_enhancer	RP11-480G7.3

chr16	46885000	chr16	70705000	1.25E-02	promoter	super_enhancer	RP11-480G7.3
chr16	46885000	chr16	73055000	9.19E-03	promoter	super_enhancer	RP11-480G7.3
chr16	46885000	chr16	81455000	7.77E-03	promoter	super_enhancer	RP11-480G7.3
chr16	47485000	chr16	56815000	8.82E-03	promoter	super_enhancer	RNA5SP424
chr16	47485000	chr16	73055000	5.94E-03	promoter	super_enhancer	RNA5SP424
chr16	47485000	chr16	73085000	5.15E-03	promoter	super_enhancer	RNA5SP424
chr16	47495000	chr16	57635000	1.34E-02	promoter	super_enhancer	PHKB
chr16	47495000	chr16	82645000	5.64E-03	promoter	super_enhancer	PHKB
chr16	47495000	chr16	84595000	5.61E-03	promoter	super_enhancer	PHKB
chr16	47795000	chr16	70745000	7.69E-03	promoter	super_enhancer	CTB-55P19.1
chr16	47795000	chr16	75245000	7.40E-03	promoter	super_enhancer	CTB-55P19.1
chr16	48095000	chr16	70745000	9.92E-03	promoter	super_enhancer	RP11-3M1.3
chr16	48455000	chr16	57295000	2.66E-02	promoter	super_enhancer	AC023818.1
chr16	48455000	chr16	57675000	2.10E-02	promoter	super_enhancer	AC023818.1
chr16	48685000	chr16	57615000	1.15E-02	promoter	super_enhancer	AC007611.1
chr16	48885000	chr16	56795000	2.13E-02	promoter	super_enhancer	RP11-42I10.3
chr16	48885000	chr16	66375000	1.72E-02	promoter	super_enhancer	RP11-42I10.3
chr16	48885000	chr16	66515000	1.69E-02	promoter	super_enhancer	RP11-42I10.3
chr16	48885000	chr16	70765000	9.88E-03	promoter	super_enhancer	RP11-42I10.3
chr16	49745000	chr16	57675000	1.97E-02	promoter	super_enhancer	CTD-2595P9.2
chr16	49745000	chr16	81505000	7.51E-03	promoter	super_enhancer	CTD-2595P9.2
chr16	50185000	chr16	57625000	2.29E-02	promoter	super_enhancer	RPL10P14
chr16	50715000	chr16	66375000	1.88E-02	promoter	super_enhancer	SNX20
chr16	50715000	chr16	81485000	9.89E-03	promoter	super_enhancer	SNX20

chr16	50775000	chr16	57665000	2.45E-02	promoter	super_enhancer	RP11-327F22.2
chr16	50775000	chr16	57675000	2.78E-02	promoter	super_enhancer	RP11-327F22.2
chr16	51185000	chr16	66515000	2.05E-02	promoter	super_enhancer	SALL1
chr16	51705000	chr16	57265000	1.47E-02	promoter	super_enhancer	RN7SKP142
chr16	51795000	chr16	56615000	2.38E-02	promoter	super_enhancer	LINC01571
chr16	52975000	chr16	57655000	3.26E-02	promoter	super_enhancer	RP11-467J12.2
chr16	53045000	chr16	70705000	1.02E-02	promoter	super_enhancer	RP11-467J12.3
chr16	53045000	chr16	82635000	4.71E-03	promoter	super_enhancer	RP11-467J12.3
chr16	53345000	chr16	56805000	3.18E-02	promoter	super_enhancer	RP11-454F8.4
chr16	53345000	chr16	57675000	2.91E-02	promoter	super_enhancer	RP11-454F8.4
chr16	53345000	chr16	73095000	7.25E-03	promoter	super_enhancer	RP11-454F8.4
chr16	53345000	chr16	86395000	5.39E-03	promoter	super_enhancer	RP11-454F8.4
chr16	53425000	chr16	75255000	1.14E-02	promoter	super_enhancer	RP11-44F14.10
chr16	53515000	chr16	56615000	1.55E-03	promoter	super_enhancer	RP11-44F14.7
chr16	53515000	chr16	86385000	6.68E-03	promoter	super_enhancer	RP11-44F14.7
chr16	53525000	chr16	66515000	1.43E-02	promoter	super_enhancer	RP11-44F14.6
chr16	53525000	chr16	74745000	8.20E-03	promoter	super_enhancer	RP11-44F14.6
chr16	54025000	chr16	56815000	4.11E-02	promoter	super_enhancer	RP11-357N13.6
chr16	54025000	chr16	57265000	3.85E-02	promoter	super_enhancer	RP11-357N13.6
chr16	54025000	chr16	67245000	1.23E-02	promoter	super_enhancer	RP11-357N13.6
chr16	54025000	chr16	81505000	8.90E-03	promoter	super_enhancer	RP11-357N13.6
chr16	54035000	chr16	57805000	4.65E-02	promoter	super_enhancer	RP11-357N13.2
chr16	54035000	chr16	70405000	1.35E-02	promoter	super_enhancer	RP11-357N13.2
chr16	54305000	chr16	73075000	1.02E-02	promoter	super_enhancer	RP11-324D17.1

chr16	54325000	chr16	84595000	1.03E-02	promoter	super_enhancer	RP11-434E6.4
chr16	55365000	chr16	56805000	1.52E-02	promoter	super_enhancer	RP11-26L20.4
chr16	56225000	chr16	66515000	1.82E-02	promoter	super_enhancer	RP11-461O7.1
chr16	56335000	chr16	66375000	2.72E-02	promoter	super_enhancer	RP11-441F2.2
chr16	56485000	chr16	57845000	3.37E-02	promoter	super_enhancer	OGFOD1
chr16	56485000	chr16	85555000	9.42E-03	promoter	super_enhancer	OGFOD1
chr16	56555000	chr16	57625000	4.87E-03	promoter	super_enhancer	BBS2
chr16	56555000	chr16	66395000	3.42E-02	promoter	super_enhancer	BBS2
chr16	56555000	chr16	66525000	3.42E-04	promoter	super_enhancer	BBS2
chr16	56555000	chr16	82655000	1.07E-02	promoter	super_enhancer	BBS2
chr16	56555000	chr16	86375000	8.01E-03	promoter	super_enhancer	BBS2
chr16	56605000	chr16	69165000	1.47E-02	super_enhancer	promoter	CIRH1A
chr16	56605000	chr16	85045000	8.52E-03	super_enhancer	promoter	ZDHHC7
chr16	56605000	chr16	90035000	8.15E-03	super_enhancer	promoter	RP11-566K11.5
chr16	56615000	chr16	66785000	1.76E-02	super_enhancer	promoter	RP11-61A14.4
chr16	56615000	chr16	69345000	1.96E-02	super_enhancer	promoter	VPS4A
chr16	56615000	chr16	79705000	9.23E-03	super_enhancer	promoter	AC009159.1
chr16	56615000	chr16	80575000	1.28E-02	super_enhancer	promoter	DYNLRB2
chr16	56645000	chr16	57835000	3.93E-02	promoter	super_enhancer	RP11-249C24.10
chr16	56645000	chr16	66395000	2.81E-02	promoter	super_enhancer	RP11-249C24.10
chr16	56645000	chr16	70415000	1.43E-02	promoter	super_enhancer	RP11-249C24.10
chr16	56645000	chr16	88785000	9.89E-03	promoter	super_enhancer	RP11-249C24.10
chr16	56665000	chr16	56805000	2.78E-02	promoter	super_enhancer	MT1M
chr16	56665000	chr16	57275000	1.77E-02	promoter	super_enhancer	MT1M

chr16	56665000	chr16	70705000	1.77E-02	promoter	super_enhancer	MT1M
chr16	56685000	chr16	57275000	2.33E-02	promoter	super_enhancer	MT1B
chr16	56685000	chr16	66395000	3.30E-02	promoter	super_enhancer	MT1B
chr16	56685000	chr16	66535000	1.55E-02	promoter	super_enhancer	MT1B
chr16	56685000	chr16	82665000	1.26E-02	promoter	super_enhancer	MT1B
chr16	56715000	chr16	66355000	2.43E-02	promoter	super_enhancer	MT1X
chr16	56715000	chr16	81505000	1.21E-02	promoter	super_enhancer	MT1X
chr16	56765000	chr16	57305000	4.46E-02	promoter	super_enhancer	RP11-343H19.1
chr16	56765000	chr16	74725000	1.33E-02	promoter	super_enhancer	RP11-343H19.1
chr16	56805000	chr16	57125000	3.53E-02	super_enhancer	promoter	CPNE2
chr16	56805000	chr16	68345000	1.74E-02	super_enhancer	promoter	SLC7A6OS
chr16	56805000	chr16	90085000	7.34E-03	super_enhancer	promoter	GAS8
chr16	56815000	chr16	62375000	1.19E-02	super_enhancer	promoter	RP11-75D3.1
chr16	56815000	chr16	66335000	1.25E-02	super_enhancer	promoter	RNA5SP428
chr16	56815000	chr16	68035000	1.24E-02	super_enhancer	promoter	DPEP2
chr16	56815000	chr16	77925000	4.67E-03	super_enhancer	promoter	RP11-281J9.1
chr16	56965000	chr16	68035000	4.44E-04	super_enhancer	promoter	DPEP2
chr16	56965000	chr16	73265000	2.18E-02	super_enhancer	promoter	AC140912.1
chr16	56975000	chr16	81645000	7.93E-03	super_enhancer	promoter	MIR6504
chr16	56975000	chr16	83025000	7.44E-03	super_enhancer	promoter	CTD-3253I12.1
chr16	56985000	chr16	57125000	7.14E-03	super_enhancer	promoter	CPNE2
chr16	56985000	chr16	58665000	1.30E-02	super_enhancer	promoter	CNOT1
chr16	56985000	chr16	66755000	1.68E-02	super_enhancer	promoter	RP11-63M22.2
chr16	56985000	chr16	67225000	2.38E-02	super_enhancer	promoter	EXOC3L1

chr16	56985000	chr16	67425000	2.64E-02	super_enhancer	promoter	RNU1-123P
chr16	56985000	chr16	68295000	2.77E-02	super_enhancer	promoter	RP11-96D1.7
chr16	56995000	chr16	57125000	3.25E-02	super_enhancer	promoter	CPNE2
chr16	56995000	chr16	65615000	2.30E-02	super_enhancer	promoter	RP11-351A20.1
chr16	56995000	chr16	66615000	2.63E-02	super_enhancer	promoter	RP11-403P17.2
chr16	56995000	chr16	67185000	2.29E-02	super_enhancer	promoter	B3GNT9
chr16	56995000	chr16	67465000	2.55E-02	super_enhancer	promoter	HSD11B2
chr16	56995000	chr16	68775000	2.62E-02	super_enhancer	promoter	RNA5SP429
chr16	56995000	chr16	71325000	1.46E-02	super_enhancer	promoter	CMTR2
chr16	56995000	chr16	75495000	1.70E-02	super_enhancer	promoter	RP11-77K12.3
chr16	56995000	chr16	81645000	1.17E-02	super_enhancer	promoter	MIR6504
chr16	56995000	chr16	82205000	7.74E-03	super_enhancer	promoter	MPHOSPH6
chr16	56995000	chr16	89225000	5.45E-03	super_enhancer	promoter	LINC00304
chr16	57125000	chr16	67245000	2.13E-02	promoter	super_enhancer	CPNE2
chr16	57125000	chr16	84605000	1.17E-02	promoter	super_enhancer	CPNE2
chr16	57125000	chr16	85505000	1.36E-02	promoter	super_enhancer	CPNE2
chr16	57125000	chr16	86365000	1.35E-02	promoter	super_enhancer	CPNE2
chr16	57265000	chr16	67575000	1.46E-02	super_enhancer	promoter	CTD-2012K14.2
chr16	57265000	chr16	68855000	1.45E-02	super_enhancer	promoter	FTLP14
chr16	57265000	chr16	75285000	9.13E-03	super_enhancer	promoter	RP11-331F4.5
chr16	57275000	chr16	58235000	4.76E-02	super_enhancer	promoter	RN7SL645P
chr16	57275000	chr16	84075000	8.37E-03	super_enhancer	promoter	RNA5SP432
chr16	57285000	chr16	75145000	9.69E-03	super_enhancer	promoter	RP11-252E2.1
chr16	57285000	chr16	85645000	7.18E-03	super_enhancer	promoter	GSE1

chr16	57295000	chr16	57575000	2.93E-02	super_enhancer	promoter	ADGRG5
chr16	57295000	chr16	66585000	3.46E-02	super_enhancer	promoter	CKLF
chr16	57295000	chr16	66995000	2.82E-02	super_enhancer	promoter	CES3
chr16	57295000	chr16	74455000	1.05E-02	super_enhancer	promoter	RP11-252A24.5
chr16	57295000	chr16	87395000	1.18E-02	super_enhancer	promoter	RP11-178L8.9
chr16	57305000	chr16	58595000	2.47E-02	super_enhancer	promoter	SNORA76A
chr16	57305000	chr16	66585000	3.08E-02	super_enhancer	promoter	CKLF
chr16	57305000	chr16	67225000	2.44E-02	super_enhancer	promoter	EXOC3L1
chr16	57305000	chr16	81055000	1.32E-02	super_enhancer	promoter	CMC2
chr16	57305000	chr16	86545000	1.17E-02	super_enhancer	promoter	AC009108.2
chr16	57315000	chr16	67595000	1.75E-02	super_enhancer	promoter	CTCF
chr16	57315000	chr16	68035000	2.00E-02	super_enhancer	promoter	DPEP2
chr16	57315000	chr16	69365000	1.92E-02	super_enhancer	promoter	PDF
chr16	57315000	chr16	69665000	1.62E-02	super_enhancer	promoter	RP11-140H17.2
chr16	57355000	chr16	57665000	2.46E-02	promoter	super_enhancer	RPL23AP91
chr16	57355000	chr16	66375000	2.49E-02	promoter	super_enhancer	RPL23AP91
chr16	57355000	chr16	82675000	8.53E-03	promoter	super_enhancer	RPL23AP91
chr16	57355000	chr16	85565000	9.55E-03	promoter	super_enhancer	RPL23AP91
chr16	57355000	chr16	88755000	9.39E-03	promoter	super_enhancer	RPL23AP91
chr16	57355000	chr16	88765000	8.01E-06	promoter	super_enhancer	RPL23AP91
chr16	57405000	chr16	66365000	2.39E-02	promoter	super_enhancer	CX3CL1
chr16	57405000	chr16	88755000	1.03E-02	promoter	super_enhancer	CX3CL1
chr16	57495000	chr16	57635000	1.53E-02	promoter	super_enhancer	AC009052.12
chr16	57495000	chr16	57805000	4.59E-02	promoter	super_enhancer	AC009052.12

chr16	57495000	chr16	66375000	2.66E-02	promoter	super_enhancer	AC009052.12
chr16	57495000	chr16	70405000	1.97E-02	promoter	super_enhancer	AC009052.12
chr16	57495000	chr16	84515000	1.18E-02	promoter	super_enhancer	AC009052.12
chr16	57575000	chr16	84615000	1.19E-02	promoter	super_enhancer	ADGRG5
chr16	57575000	chr16	87805000	8.92E-03	promoter	super_enhancer	ADGRG5
chr16	57575000	chr16	88775000	8.76E-03	promoter	super_enhancer	ADGRG5
chr16	57615000	chr16	58005000	1.15E-02	super_enhancer	promoter	CNGB1
chr16	57615000	chr16	58035000	4.72E-04	super_enhancer	promoter	ZNF319
chr16	57615000	chr16	58455000	4.76E-02	super_enhancer	promoter	RP11-481J2.2
chr16	57615000	chr16	66995000	1.64E-02	super_enhancer	promoter	CES3
chr16	57615000	chr16	68855000	1.49E-02	super_enhancer	promoter	FTLP14
chr16	57615000	chr16	69365000	1.45E-02	super_enhancer	promoter	PDF
chr16	57615000	chr16	77625000	7.70E-03	super_enhancer	promoter	RP11-571O6.1
chr16	57615000	chr16	84955000	6.27E-03	super_enhancer	promoter	RP11-254F19.4
chr16	57625000	chr16	58005000	1.17E-05	super_enhancer	promoter	CNGB1
chr16	57625000	chr16	63215000	1.87E-02	super_enhancer	promoter	UBE2FP2
chr16	57635000	chr16	66335000	2.03E-02	super_enhancer	promoter	RNA5SP428
chr16	57635000	chr16	67185000	1.92E-02	super_enhancer	promoter	B3GNT9
chr16	57635000	chr16	69375000	1.69E-02	super_enhancer	promoter	COG8
chr16	57635000	chr16	81215000	1.01E-02	super_enhancer	promoter	AC092718.1
chr16	57635000	chr16	89725000	9.13E-03	super_enhancer	promoter	CHMP1A
chr16	57645000	chr16	67595000	1.67E-04	super_enhancer	promoter	CTCF
chr16	57645000	chr16	82045000	9.84E-03	super_enhancer	promoter	SDR42E1
chr16	57655000	chr16	58035000	6.36E-03	super_enhancer	promoter	ZNF319

chr16	57655000	chr16	67295000	3.06E-02	super_enhancer	promoter	CTC-277H1.7
chr16	57655000	chr16	67515000	2.64E-02	super_enhancer	promoter	ATP6V0D1
chr16	57655000	chr16	70835000	1.81E-02	super_enhancer	promoter	VAC14
chr16	57655000	chr16	75745000	1.41E-02	super_enhancer	promoter	ATP5F1P7
chr16	57655000	chr16	82875000	1.10E-02	super_enhancer	promoter	AC099506.1
chr16	57655000	chr16	89225000	5.81E-03	super_enhancer	promoter	LINC00304
chr16	57665000	chr16	58235000	5.91E-03	super_enhancer	promoter	RN7SL645P
chr16	57665000	chr16	66785000	1.59E-02	super_enhancer	promoter	RP11-61A14.4
chr16	57665000	chr16	66995000	2.06E-02	super_enhancer	promoter	CES3
chr16	57665000	chr16	67195000	1.91E-04	super_enhancer	promoter	TRADD
chr16	57665000	chr16	67515000	1.97E-02	super_enhancer	promoter	ATP6V0D1
chr16	57665000	chr16	75495000	1.36E-02	super_enhancer	promoter	RP11-77K12.3
chr16	57665000	chr16	84225000	8.35E-03	super_enhancer	promoter	ADAD2
chr16	57675000	chr16	83025000	9.93E-03	super_enhancer	promoter	CTD-3253I12.1
chr16	57675000	chr16	89225000	4.92E-03	super_enhancer	promoter	LINC00304
chr16	57795000	chr16	58035000	1.07E-03	super_enhancer	promoter	ZNF319
chr16	57795000	chr16	67225000	2.63E-02	super_enhancer	promoter	EXOC3L1
chr16	57805000	chr16	57865000	1.68E-02	super_enhancer	promoter	RNU6-20P
chr16	57805000	chr16	58005000	1.56E-02	super_enhancer	promoter	CNGB1
chr16	57805000	chr16	66615000	2.50E-02	super_enhancer	promoter	RP11-403P17.2
chr16	57805000	chr16	67515000	2.30E-02	super_enhancer	promoter	ATP6V0D1
chr16	57805000	chr16	68005000	2.22E-02	super_enhancer	promoter	SLC12A4
chr16	57805000	chr16	68235000	1.98E-02	super_enhancer	promoter	RP11-67A1.4
chr16	57805000	chr16	71325000	1.37E-02	super_enhancer	promoter	CMTR2

chr16	57805000	chr16	81255000	1.22E-02	super_enhancer	promoter	PKD1L2
chr16	57815000	chr16	57865000	4.39E-03	super_enhancer	promoter	RNU6-20P
chr16	57815000	chr16	58005000	4.70E-03	super_enhancer	promoter	CNGB1
chr16	57815000	chr16	66485000	2.51E-02	super_enhancer	promoter	CTD-2258A20.3
chr16	57815000	chr16	66755000	1.36E-04	super_enhancer	promoter	RP11-63M22.2
chr16	57815000	chr16	68295000	2.69E-02	super_enhancer	promoter	RP11-96D1.7
chr16	57815000	chr16	69165000	1.62E-02	super_enhancer	promoter	CIRH1A
chr16	57815000	chr16	77285000	9.76E-03	super_enhancer	promoter	RP11-538I12.2
chr16	57825000	chr16	67295000	2.63E-02	super_enhancer	promoter	CTC-277H1.7
chr16	57825000	chr16	67875000	2.27E-02	super_enhancer	promoter	THAP11
chr16	57835000	chr16	65615000	2.36E-02	super_enhancer	promoter	RP11-351A20.1
chr16	57835000	chr16	66615000	2.64E-02	super_enhancer	promoter	RP11-403P17.2
chr16	57835000	chr16	67465000	2.53E-02	super_enhancer	promoter	HSD11B2
chr16	57835000	chr16	75035000	1.31E-02	super_enhancer	promoter	WDR59
chr16	57835000	chr16	81215000	1.19E-02	super_enhancer	promoter	AC092718.1
chr16	57835000	chr16	81995000	1.12E-02	super_enhancer	promoter	RN7SKP176
chr16	57835000	chr16	85205000	1.21E-02	super_enhancer	promoter	CTC-786C10.1
chr16	57845000	chr16	58005000	1.50E-03	super_enhancer	promoter	CNGB1
chr16	57845000	chr16	63385000	2.38E-02	super_enhancer	promoter	RPS15AP34
chr16	57845000	chr16	66615000	2.90E-02	super_enhancer	promoter	RP11-403P17.2
chr16	57845000	chr16	68295000	3.14E-02	super_enhancer	promoter	RP11-96D1.7
chr16	57845000	chr16	68405000	2.21E-02	super_enhancer	promoter	RP11-71L14.3
chr16	57865000	chr16	66395000	3.71E-02	promoter	super_enhancer	RNU6-20P
chr16	57865000	chr16	74725000	1.72E-02	promoter	super_enhancer	RNU6-20P

chr16	57865000	chr16	86345000	1.11E-02	promoter	super_enhancer	RNU6-20P
chr16	57865000	chr16	88785000	1.25E-02	promoter	super_enhancer	RNU6-20P
chr16	58005000	chr16	66505000	3.25E-02	promoter	super_enhancer	CNGB1
chr16	58005000	chr16	66525000	2.96E-02	promoter	super_enhancer	CNGB1
chr16	58005000	chr16	69405000	2.45E-02	promoter	super_enhancer	CNGB1
chr16	58005000	chr16	81455000	1.23E-02	promoter	super_enhancer	CNGB1
chr16	58005000	chr16	81485000	1.41E-02	promoter	super_enhancer	CNGB1
chr16	58005000	chr16	89315000	1.18E-02	promoter	super_enhancer	CNGB1
chr16	58035000	chr16	75255000	1.60E-02	promoter	super_enhancer	ZNF319
chr16	58035000	chr16	84525000	7.05E-03	promoter	super_enhancer	ZNF319
chr16	58035000	chr16	84615000	1.06E-02	promoter	super_enhancer	ZNF319
chr16	58235000	chr16	66505000	3.06E-02	promoter	super_enhancer	RN7SL645P
chr16	58235000	chr16	73085000	1.27E-02	promoter	super_enhancer	RN7SL645P
chr16	58235000	chr16	88775000	9.35E-03	promoter	super_enhancer	RN7SL645P
chr16	58265000	chr16	66375000	2.69E-02	promoter	super_enhancer	CCDC113
chr16	58265000	chr16	70715000	1.98E-02	promoter	super_enhancer	CCDC113
chr16	58265000	chr16	70755000	2.07E-02	promoter	super_enhancer	CCDC113
chr16	58455000	chr16	66365000	2.41E-02	promoter	super_enhancer	RP11-481J2.2
chr16	58455000	chr16	66525000	2.65E-02	promoter	super_enhancer	RP11-481J2.2
chr16	58455000	chr16	74735000	1.59E-02	promoter	super_enhancer	RP11-481J2.2
chr16	58455000	chr16	81495000	1.19E-02	promoter	super_enhancer	RP11-481J2.2
chr16	58455000	chr16	84525000	7.91E-03	promoter	super_enhancer	RP11-481J2.2
chr16	58455000	chr16	85495000	1.04E-02	promoter	super_enhancer	RP11-481J2.2
chr16	58595000	chr16	70405000	1.56E-02	promoter	super_enhancer	SNORA76A

chr16	58665000	chr16	70765000	1.23E-02	promoter	super_enhancer	CNOT1
chr16	58665000	chr16	74735000	1.35E-02	promoter	super_enhancer	CNOT1
chr16	58915000	chr16	73095000	1.16E-02	promoter	super_enhancer	RP11-700H13.1
chr16	60465000	chr16	66395000	3.55E-02	promoter	super_enhancer	AC009081.1
chr16	60465000	chr16	81495000	9.87E-03	promoter	super_enhancer	AC009081.1
chr16	62375000	chr16	66365000	2.85E-02	promoter	super_enhancer	RP11-75D3.1
chr16	62375000	chr16	73095000	9.88E-03	promoter	super_enhancer	RP11-75D3.1
chr16	62755000	chr16	66535000	1.98E-02	promoter	super_enhancer	AC092116.1
chr16	63165000	chr16	66525000	4.19E-02	promoter	super_enhancer	RP11-96H17.1
chr16	63215000	chr16	66515000	4.79E-02	promoter	super_enhancer	UBE2FP2
chr16	63385000	chr16	66355000	4.85E-02	promoter	super_enhancer	RPS15AP34
chr16	63385000	chr16	66515000	4.52E-02	promoter	super_enhancer	RPS15AP34
chr16	64295000	chr16	66375000	3.24E-03	promoter	super_enhancer	RP11-744D14.1
chr16	65615000	chr16	81475000	1.38E-02	promoter	super_enhancer	RP11-351A20.1
chr16	66335000	chr16	66525000	5.74E-04	promoter	super_enhancer	RNA5SP428
chr16	66335000	chr16	85515000	1.10E-02	promoter	super_enhancer	RNA5SP428
chr16	66355000	chr16	75035000	2.05E-02	super_enhancer	promoter	WDR59
chr16	66365000	chr16	67225000	1.39E-02	super_enhancer	promoter	EXOC3L1
chr16	66365000	chr16	70235000	3.00E-02	super_enhancer	promoter	RP11-296I10.5
chr16	66365000	chr16	71115000	4.35E-02	super_enhancer	promoter	RP11-23E19.1
chr16	66365000	chr16	75495000	2.30E-02	super_enhancer	promoter	RP11-77K12.3
chr16	66365000	chr16	78275000	1.57E-02	super_enhancer	promoter	RP11-190D6.2
chr16	66375000	chr16	81055000	1.96E-02	super_enhancer	promoter	CMC2
chr16	66395000	chr16	71265000	2.98E-02	super_enhancer	promoter	HYDIN

chr16	66395000	chr16	71325000	4.21E-02	super_enhancer	promoter	CMTR2
chr16	66395000	chr16	72615000	2.15E-02	super_enhancer	promoter	AC092289.1
chr16	66395000	chr16	79705000	1.92E-02	super_enhancer	promoter	AC009159.1
chr16	66395000	chr16	90085000	1.76E-02	super_enhancer	promoter	GAS8
chr16	66485000	chr16	66525000	3.66E-02	promoter	super_enhancer	CTD-2258A20.3
chr16	66485000	chr16	75245000	2.86E-02	promoter	super_enhancer	CTD-2258A20.3
chr16	66505000	chr16	74325000	2.80E-02	super_enhancer	promoter	AC009120.11
chr16	66505000	chr16	88535000	1.43E-02	super_enhancer	promoter	MIR5189
chr16	66515000	chr16	67225000	3.36E-02	super_enhancer	promoter	EXOC3L1
chr16	66515000	chr16	68035000	2.68E-02	super_enhancer	promoter	DPEP2
chr16	66525000	chr16	74315000	2.44E-02	super_enhancer	promoter	AC009120.10
chr16	66525000	chr16	74875000	2.20E-02	super_enhancer	promoter	RP11-787D11.1
chr16	66525000	chr16	75285000	2.25E-02	super_enhancer	promoter	RP11-331F4.5
chr16	66525000	chr16	81215000	1.74E-02	super_enhancer	promoter	AC092718.1
chr16	66525000	chr16	83965000	1.49E-02	super_enhancer	promoter	RP11-483P21.6
chr16	66525000	chr16	85625000	1.70E-02	super_enhancer	promoter	CTD-2542L18.1
chr16	66525000	chr16	87525000	1.46E-02	super_enhancer	promoter	RP11-482M8.1
chr16	66535000	chr16	89045000	8.03E-03	super_enhancer	promoter	CBFA2T3
chr16	66585000	chr16	82665000	2.20E-02	promoter	super_enhancer	CKLF
chr16	66615000	chr16	74735000	2.65E-02	promoter	super_enhancer	RP11-403P17.2
chr16	66615000	chr16	82665000	1.86E-02	promoter	super_enhancer	RP11-403P17.2
chr16	66755000	chr16	70745000	3.76E-02	promoter	super_enhancer	RP11-63M22.2
chr16	66755000	chr16	73075000	1.62E-02	promoter	super_enhancer	RP11-63M22.2
chr16	66755000	chr16	75245000	1.96E-02	promoter	super_enhancer	RP11-63M22.2

chr16	66995000	chr16	67245000	9.70E-03	promoter	super_enhancer	CES3
chr16	66995000	chr16	73025000	3.31E-02	promoter	super_enhancer	CES3
chr16	67185000	chr16	74725000	2.50E-02	promoter	super_enhancer	B3GNT9
chr16	67185000	chr16	81485000	1.72E-02	promoter	super_enhancer	B3GNT9
chr16	67185000	chr16	81495000	1.65E-02	promoter	super_enhancer	B3GNT9
chr16	67185000	chr16	85505000	1.55E-02	promoter	super_enhancer	B3GNT9
chr16	67185000	chr16	88755000	1.28E-02	promoter	super_enhancer	B3GNT9
chr16	67185000	chr16	88785000	1.49E-02	promoter	super_enhancer	B3GNT9
chr16	67195000	chr16	73045000	2.90E-02	promoter	super_enhancer	TRADD
chr16	67245000	chr16	73265000	2.56E-02	super_enhancer	promoter	AC140912.1
chr16	67245000	chr16	81995000	1.26E-02	super_enhancer	promoter	RN7SKP176
chr16	67295000	chr16	73055000	3.25E-02	promoter	super_enhancer	CTC-277H1.7
chr16	67295000	chr16	81475000	2.04E-02	promoter	super_enhancer	CTC-277H1.7
chr16	67295000	chr16	85495000	1.75E-02	promoter	super_enhancer	CTC-277H1.7
chr16	67295000	chr16	89305000	1.45E-02	promoter	super_enhancer	CTC-277H1.7
chr16	67425000	chr16	81495000	2.04E-02	promoter	super_enhancer	RNU1-123P
chr16	67515000	chr16	74745000	2.54E-02	promoter	super_enhancer	ATP6V0D1
chr16	67515000	chr16	81465000	1.89E-02	promoter	super_enhancer	ATP6V0D1
chr16	67515000	chr16	86395000	1.46E-02	promoter	super_enhancer	ATP6V0D1
chr16	67595000	chr16	88785000	1.39E-02	promoter	super_enhancer	CTCF
chr16	67695000	chr16	74735000	2.74E-02	promoter	super_enhancer	ACD
chr16	67695000	chr16	89325000	1.15E-02	promoter	super_enhancer	ACD
chr16	67775000	chr16	75235000	2.80E-02	promoter	super_enhancer	RP11-167P11.2
chr16	67775000	chr16	84995000	1.65E-02	promoter	super_enhancer	RP11-167P11.2

chr16	67775000	chr16	85505000	1.77E-02	promoter	super_enhancer	RP11-167P11.2
chr16	67915000	chr16	88775000	1.10E-02	promoter	super_enhancer	CTC-479C5.10
chr16	68005000	chr16	74745000	2.69E-02	promoter	super_enhancer	SLC12A4
chr16	68015000	chr16	73095000	3.80E-02	promoter	super_enhancer	DPEP3
chr16	68035000	chr16	73075000	2.94E-02	promoter	super_enhancer	DPEP2
chr16	68295000	chr16	82675000	1.72E-02	promoter	super_enhancer	RP11-96D1.7
chr16	68295000	chr16	84595000	2.15E-02	promoter	super_enhancer	RP11-96D1.7
chr16	68295000	chr16	85565000	1.87E-02	promoter	super_enhancer	RP11-96D1.7
chr16	68345000	chr16	73055000	3.65E-02	promoter	super_enhancer	SLC7A6OS
chr16	68345000	chr16	75235000	3.03E-02	promoter	super_enhancer	SLC7A6OS
chr16	68345000	chr16	81525000	1.67E-02	promoter	super_enhancer	SLC7A6OS
chr16	68405000	chr16	74745000	2.50E-02	promoter	super_enhancer	RP11-71L14.3
chr16	68405000	chr16	81495000	1.65E-02	promoter	super_enhancer	RP11-71L14.3
chr16	68405000	chr16	82645000	1.58E-02	promoter	super_enhancer	RP11-71L14.3
chr16	68625000	chr16	85005000	1.32E-02	promoter	super_enhancer	RP11-615I2.6
chr16	69165000	chr16	85515000	1.05E-02	promoter	super_enhancer	CIRH1A
chr16	69345000	chr16	84515000	1.83E-02	promoter	super_enhancer	VPS4A
chr16	69365000	chr16	73055000	4.82E-02	promoter	super_enhancer	PDF
chr16	69375000	chr16	75235000	3.11E-02	promoter	super_enhancer	COG8
chr16	69375000	chr16	81485000	1.87E-02	promoter	super_enhancer	COG8
chr16	69375000	chr16	81515000	1.61E-02	promoter	super_enhancer	COG8
chr16	69405000	chr16	70235000	3.95E-03	super_enhancer	promoter	RP11-296I10.5
chr16	69405000	chr16	85205000	1.88E-02	super_enhancer	promoter	CTC-786C10.1
chr16	69405000	chr16	87395000	1.58E-02	super_enhancer	promoter	RP11-178L8.9

chr16	69415000	chr16	69665000	2.12E-04	super_enhancer	promoter	RP11-140H17.2
chr16	69415000	chr16	85825000	1.44E-02	super_enhancer	promoter	RP11-568J23.6
chr16	69665000	chr16	87805000	1.22E-02	promoter	super_enhancer	RP11-140H17.2
chr16	69795000	chr16	81485000	1.69E-02	promoter	super_enhancer	WWP2
chr16	70235000	chr16	84615000	1.11E-02	promoter	super_enhancer	RP11-296I10.5
chr16	70405000	chr16	81995000	1.81E-02	super_enhancer	promoter	RN7SKP176
chr16	70405000	chr16	84075000	1.88E-02	super_enhancer	promoter	RNA5SP432
chr16	70405000	chr16	85205000	1.89E-02	super_enhancer	promoter	CTC-786C10.1
chr16	70405000	chr16	87245000	1.42E-02	super_enhancer	promoter	RP11-899L11.1
chr16	70425000	chr16	75495000	4.08E-02	super_enhancer	promoter	RP11-77K12.3
chr16	70435000	chr16	75035000	3.72E-02	super_enhancer	promoter	WDR59
chr16	70435000	chr16	80575000	2.42E-02	super_enhancer	promoter	DYNLRB2
chr16	70435000	chr16	85475000	2.23E-02	super_enhancer	promoter	AC092377.1
chr16	70685000	chr16	70805000	1.96E-05	super_enhancer	promoter	RP11-394B2.3
chr16	70685000	chr16	85725000	1.71E-02	super_enhancer	promoter	GINS2
chr16	70695000	chr16	74325000	4.15E-02	super_enhancer	promoter	AC009120.11
chr16	70695000	chr16	89725000	1.22E-02	super_enhancer	promoter	CHMP1A
chr16	70705000	chr16	70805000	1.06E-02	super_enhancer	promoter	RP11-394B2.3
chr16	70705000	chr16	74455000	3.14E-02	super_enhancer	promoter	RP11-252A24.5
chr16	70705000	chr16	80575000	2.30E-02	super_enhancer	promoter	DYNLRB2
chr16	70705000	chr16	82205000	1.23E-02	super_enhancer	promoter	MPHOSPH6
chr16	70715000	chr16	70805000	5.51E-03	super_enhancer	promoter	RP11-394B2.3
chr16	70745000	chr16	81215000	2.00E-02	super_enhancer	promoter	AC092718.1
chr16	70745000	chr16	86015000	1.79E-02	super_enhancer	promoter	RP11-542M13.2

chr16	70745000	chr16	89295000	1.13E-02	super_enhancer	promoter	RP11-46C24.6
chr16	70765000	chr16	72035000	1.30E-03	super_enhancer	promoter	PKD1L3
chr16	70765000	chr16	75145000	3.31E-02	super_enhancer	promoter	RP11-252E2.1
chr16	70765000	chr16	83965000	1.27E-02	super_enhancer	promoter	RP11-483P21.6
chr16	70835000	chr16	75235000	3.77E-02	promoter	super_enhancer	VAC14
chr16	71325000	chr16	75255000	4.52E-02	promoter	super_enhancer	CMTR2
chr16	71325000	chr16	85525000	1.14E-02	promoter	super_enhancer	CMTR2
chr16	71335000	chr16	86355000	1.23E-02	promoter	super_enhancer	CTD-2520I13.1
chr16	71365000	chr16	75235000	3.94E-02	promoter	super_enhancer	AC106736.1
chr16	71365000	chr16	82665000	1.62E-02	promoter	super_enhancer	AC106736.1
chr16	72025000	chr16	88775000	1.04E-02	promoter	super_enhancer	RPL39P31
chr16	72035000	chr16	73065000	3.51E-03	promoter	super_enhancer	PKD1L3
chr16	73025000	chr16	73545000	6.71E-03	super_enhancer	promoter	RP11-44L9.3
chr16	73025000	chr16	75145000	6.82E-03	super_enhancer	promoter	RP11-252E2.1
chr16	73025000	chr16	81995000	1.99E-02	super_enhancer	promoter	RN7SKP176
chr16	73035000	chr16	78055000	2.65E-02	super_enhancer	promoter	CLEC3A
chr16	73035000	chr16	78085000	3.15E-02	super_enhancer	promoter	KRT8P22
chr16	73035000	chr16	89295000	1.20E-02	super_enhancer	promoter	RP11-46C24.6
chr16	73045000	chr16	84075000	1.80E-02	super_enhancer	promoter	RNA5SP432
chr16	73055000	chr16	74255000	1.59E-02	super_enhancer	promoter	AC009120.8
chr16	73055000	chr16	81055000	2.06E-02	super_enhancer	promoter	CMC2
chr16	73055000	chr16	84075000	1.69E-02	super_enhancer	promoter	RNA5SP432
chr16	73075000	chr16	73265000	3.60E-02	super_enhancer	promoter	AC140912.1
chr16	73075000	chr16	83965000	1.36E-02	super_enhancer	promoter	RP11-483P21.6

chr16	73085000	chr16	73265000	3.44E-02	super_enhancer	promoter	AC140912.1
chr16	73085000	chr16	81645000	1.50E-02	super_enhancer	promoter	MIR6504
chr16	73095000	chr16	82875000	1.52E-02	super_enhancer	promoter	AC099506.1
chr16	73125000	chr16	81495000	2.25E-02	promoter	super_enhancer	HCCAT5
chr16	74255000	chr16	74735000	3.09E-02	promoter	super_enhancer	AC009120.8
chr16	74455000	chr16	81505000	1.62E-02	promoter	super_enhancer	RP11-252A24.5
chr16	74455000	chr16	82675000	1.05E-02	promoter	super_enhancer	RP11-252A24.5
chr16	74725000	chr16	75145000	2.91E-03	super_enhancer	promoter	RP11-252E2.1
chr16	74725000	chr16	86015000	2.10E-02	super_enhancer	promoter	RP11-542M13.2
chr16	74725000	chr16	88535000	1.70E-02	super_enhancer	promoter	MIR5189
chr16	74725000	chr16	89005000	1.72E-02	super_enhancer	promoter	RP11-830F9.6
chr16	74745000	chr16	78085000	4.81E-02	super_enhancer	promoter	KRT8P22
chr16	74745000	chr16	90035000	1.48E-02	super_enhancer	promoter	RP11-566K11.5
chr16	74875000	chr16	81485000	2.55E-02	promoter	super_enhancer	RP11-787D11.1
chr16	74875000	chr16	84615000	2.03E-02	promoter	super_enhancer	RP11-787D11.1
chr16	75035000	chr16	86395000	1.59E-02	promoter	super_enhancer	WDR59
chr16	75235000	chr16	79805000	2.71E-02	super_enhancer	promoter	MAFTRR
chr16	75235000	chr16	80075000	2.42E-02	super_enhancer	promoter	RP11-148M9.1
chr16	75235000	chr16	81215000	3.01E-02	super_enhancer	promoter	AC092718.1
chr16	75235000	chr16	81645000	2.74E-02	super_enhancer	promoter	MIR6504
chr16	75245000	chr16	79805000	3.15E-02	super_enhancer	promoter	MAFTRR
chr16	75245000	chr16	85045000	2.20E-02	super_enhancer	promoter	ZDHHC7
chr16	75255000	chr16	81255000	3.78E-02	super_enhancer	promoter	PKD1L2
chr16	75285000	chr16	88785000	1.96E-02	promoter	super_enhancer	RP11-331F4.5

chr16	75495000	chr16	84605000	2.17E-02	promoter	super_enhancer	RP11-77K12.3
chr16	75495000	chr16	84615000	2.62E-02	promoter	super_enhancer	RP11-77K12.3
chr16	75735000	chr16	81515000	3.39E-02	promoter	super_enhancer	RP11-490B18.7
chr16	75745000	chr16	81455000	2.48E-02	promoter	super_enhancer	ATP5F1P7
chr16	75745000	chr16	81505000	2.88E-02	promoter	super_enhancer	ATP5F1P7
chr16	75745000	chr16	81525000	2.31E-02	promoter	super_enhancer	ATP5F1P7
chr16	76615000	chr16	81455000	1.91E-02	promoter	super_enhancer	RP11-58C22.2
chr16	77285000	chr16	81485000	3.44E-02	promoter	super_enhancer	RP11-538I12.2
chr16	77925000	chr16	81525000	2.99E-02	promoter	super_enhancer	RP11-281J9.1
chr16	77925000	chr16	82675000	1.95E-02	promoter	super_enhancer	RP11-281J9.1
chr16	78275000	chr16	84605000	2.34E-02	promoter	super_enhancer	RP11-190D6.2
chr16	79635000	chr16	85505000	3.40E-02	promoter	super_enhancer	MAF
chr16	79805000	chr16	87805000	1.43E-02	promoter	super_enhancer	MAFTRR
chr16	80575000	chr16	85525000	3.65E-02	promoter	super_enhancer	DYNLRB2
chr16	81055000	chr16	87805000	2.74E-02	promoter	super_enhancer	CMC2
chr16	81215000	chr16	84525000	4.50E-02	promoter	super_enhancer	AC092718.1
chr16	81455000	chr16	86545000	3.75E-02	super_enhancer	promoter	AC009108.2
chr16	81475000	chr16	81645000	2.61E-02	super_enhancer	promoter	MIR6504
chr16	81475000	chr16	90035000	2.27E-02	super_enhancer	promoter	RP11-566K11.5
chr16	81485000	chr16	90035000	2.38E-02	super_enhancer	promoter	RP11-566K11.5
chr16	81495000	chr16	83965000	4.06E-03	super_enhancer	promoter	RP11-483P21.6
chr16	81505000	chr16	89155000	2.84E-02	super_enhancer	promoter	ACSF3
chr16	81505000	chr16	90035000	2.44E-02	super_enhancer	promoter	RP11-566K11.5
chr16	81525000	chr16	84955000	4.52E-02	super_enhancer	promoter	RP11-254F19.4

chr16	81525000	chr16	86545000	3.59E-02	super_enhancer	promoter	AC009108.2
chr16	82045000	chr16	86375000	3.07E-02	promoter	super_enhancer	SDR42E1
chr16	82155000	chr16	86385000	4.05E-02	promoter	super_enhancer	CTD-2588J6.1
chr16	82545000	chr16	86355000	4.15E-02	promoter	super_enhancer	RP11-481E4.2
chr16	82645000	chr16	84225000	1.53E-02	super_enhancer	promoter	ADAD2
chr16	82645000	chr16	86755000	4.60E-02	super_enhancer	promoter	CTD-2015G9.2
chr16	82655000	chr16	83965000	2.12E-02	super_enhancer	promoter	RP11-483P21.6
chr16	83025000	chr16	86375000	4.32E-02	promoter	super_enhancer	CTD-3253I12.1
chr16	84075000	chr16	84525000	6.06E-03	promoter	super_enhancer	RNA5SP432
chr16	84225000	chr16	89335000	3.05E-02	promoter	super_enhancer	ADAD2
chr16	84295000	chr16	84515000	9.26E-03	promoter	super_enhancer	RP11-558A11.1
chr16	84295000	chr16	84525000	3.09E-02	promoter	super_enhancer	RP11-558A11.1
chr16	84525000	chr16	85645000	2.93E-02	super_enhancer	promoter	GSE1
chr16	84525000	chr16	90035000	2.56E-02	super_enhancer	promoter	RP11-566K11.5
chr16	84605000	chr16	88135000	4.07E-02	super_enhancer	promoter	RP11-863P13.4
chr16	84605000	chr16	88535000	4.58E-02	super_enhancer	promoter	MIR5189
chr16	84955000	chr16	84995000	1.60E-02	promoter	super_enhancer	RP11-254F19.4
chr16	84995000	chr16	85205000	3.57E-02	super_enhancer	promoter	CTC-786C10.1
chr16	85005000	chr16	85045000	1.42E-02	super_enhancer	promoter	ZDHHC7
chr16	85045000	chr16	88765000	2.21E-02	promoter	super_enhancer	ZDHHC7
chr16	85045000	chr16	88775000	4.59E-02	promoter	super_enhancer	ZDHHC7
chr16	85475000	chr16	85495000	4.55E-02	promoter	super_enhancer	AC092377.1
chr16	85475000	chr16	85525000	2.45E-02	promoter	super_enhancer	AC092377.1
chr16	85475000	chr16	85565000	5.31E-03	promoter	super_enhancer	AC092377.1

chr16	85525000	chr16	87525000	7.68E-03	super_enhancer	promoter	RP11-482M8.1
chr16	85785000	chr16	86355000	1.99E-02	promoter	super_enhancer	C16orf74
chr16	85785000	chr16	88775000	1.91E-03	promoter	super_enhancer	C16orf74
chr16	85785000	chr16	89335000	4.61E-02	promoter	super_enhancer	C16orf74
chr16	86015000	chr16	86365000	1.31E-02	promoter	super_enhancer	RP11-542M13.2
chr16	86345000	chr16	86545000	6.18E-04	super_enhancer	promoter	AC009108.2
chr16	86375000	chr16	86545000	1.92E-02	super_enhancer	promoter	AC009108.2
chr16	86375000	chr16	87395000	3.58E-02	super_enhancer	promoter	RP11-178L8.9
chr16	86385000	chr16	86545000	1.68E-05	super_enhancer	promoter	AC009108.2
chr16	86395000	chr16	86545000	2.28E-02	super_enhancer	promoter	AC009108.2
chr16	86395000	chr16	87395000	7.92E-03	super_enhancer	promoter	RP11-178L8.9
chr16	86755000	chr16	87805000	4.31E-02	promoter	super_enhancer	CTD-2015G9.2
chr16	88705000	chr16	89325000	1.76E-02	promoter	super_enhancer	IL17C
chr16	88775000	chr16	89155000	1.28E-04	super_enhancer	promoter	ACSF3
chr16	88775000	chr16	89295000	3.18E-03	super_enhancer	promoter	RP11-46C24.6
chr16	89295000	chr16	89305000	1.43E-02	promoter	super_enhancer	RP11-46C24.6
chr16	89315000	chr16	89725000	4.02E-02	super_enhancer	promoter	CHMP1A
chr16	89325000	chr16	89885000	3.87E-02	super_enhancer	promoter	SPIRE2
chr16	89335000	chr16	89885000	3.81E-02	super_enhancer	promoter	SPIRE2
chr17	5000	chr17	965000	1.01E-03	promoter	super_enhancer	AC108004.1
chr17	5000	chr17	16295000	1.98E-02	promoter	super_enhancer	AC108004.1
chr17	5000	chr17	17125000	2.61E-02	promoter	super_enhancer	AC108004.1
chr17	5000	chr17	17845000	1.61E-02	promoter	super_enhancer	AC108004.1
chr17	5000	chr17	79795000	4.47E-03	promoter	super_enhancer	AC108004.1

chr17	205000	chr17	915000	2.50E-02	promoter	super_enhancer	RP11-1260E13.3
chr17	205000	chr17	925000	1.69E-03	promoter	super_enhancer	RP11-1260E13.3
chr17	205000	chr17	27555000	1.36E-02	promoter	super_enhancer	RP11-1260E13.3
chr17	205000	chr17	56935000	5.33E-03	promoter	super_enhancer	RP11-1260E13.3
chr17	205000	chr17	77255000	4.48E-03	promoter	super_enhancer	RP11-1260E13.3
chr17	205000	chr17	80825000	4.99E-03	promoter	super_enhancer	RP11-1260E13.3
chr17	205000	chr17	81095000	3.36E-03	promoter	super_enhancer	RP11-1260E13.3
chr17	265000	chr17	915000	4.61E-03	promoter	super_enhancer	AC108004.3
chr17	265000	chr17	7845000	3.08E-02	promoter	super_enhancer	AC108004.3
chr17	265000	chr17	80255000	4.09E-03	promoter	super_enhancer	AC108004.3
chr17	265000	chr17	81065000	3.44E-03	promoter	super_enhancer	AC108004.3
chr17	405000	chr17	935000	2.00E-02	promoter	super_enhancer	RP5-1029F21.3
chr17	405000	chr17	17735000	1.88E-02	promoter	super_enhancer	RP5-1029F21.3
chr17	405000	chr17	17755000	1.45E-02	promoter	super_enhancer	RP5-1029F21.3
chr17	405000	chr17	38445000	9.86E-03	promoter	super_enhancer	RP5-1029F21.3
chr17	405000	chr17	40095000	9.21E-03	promoter	super_enhancer	RP5-1029F21.3
chr17	405000	chr17	80265000	4.73E-03	promoter	super_enhancer	RP5-1029F21.3
chr17	425000	chr17	905000	7.75E-03	promoter	super_enhancer	RP5-1029F21.4
chr17	425000	chr17	17105000	1.95E-02	promoter	super_enhancer	RP5-1029F21.4
chr17	425000	chr17	27555000	1.18E-02	promoter	super_enhancer	RP5-1029F21.4
chr17	425000	chr17	43355000	7.13E-03	promoter	super_enhancer	RP5-1029F21.4
chr17	425000	chr17	79845000	2.88E-03	promoter	super_enhancer	RP5-1029F21.4
chr17	455000	chr17	905000	3.06E-03	promoter	super_enhancer	RP5-1029F21.2
chr17	455000	chr17	955000	2.19E-02	promoter	super_enhancer	RP5-1029F21.2

chr17	455000	chr17	43455000	7.70E-03	promoter	super_enhancer	RP5-1029F21.2
chr17	455000	chr17	80895000	3.78E-03	promoter	super_enhancer	RP5-1029F21.2
chr17	625000	chr17	905000	4.89E-03	promoter	super_enhancer	VPS53
chr17	625000	chr17	43465000	1.04E-02	promoter	super_enhancer	VPS53
chr17	625000	chr17	75695000	5.88E-03	promoter	super_enhancer	VPS53
chr17	625000	chr17	80765000	3.89E-03	promoter	super_enhancer	VPS53
chr17	635000	chr17	17825000	2.00E-02	promoter	super_enhancer	FAM57A
chr17	635000	chr17	29175000	1.22E-02	promoter	super_enhancer	FAM57A
chr17	635000	chr17	36935000	9.62E-03	promoter	super_enhancer	FAM57A
chr17	635000	chr17	38905000	8.66E-03	promoter	super_enhancer	FAM57A
chr17	655000	chr17	21285000	1.56E-02	promoter	super_enhancer	DBIL5P
chr17	655000	chr17	81055000	3.82E-03	promoter	super_enhancer	DBIL5P
chr17	685000	chr17	905000	2.03E-03	promoter	super_enhancer	RNMTL1
chr17	685000	chr17	17765000	2.17E-02	promoter	super_enhancer	RNMTL1
chr17	685000	chr17	42025000	9.33E-03	promoter	super_enhancer	RNMTL1
chr17	685000	chr17	42425000	9.32E-03	promoter	super_enhancer	RNMTL1
chr17	685000	chr17	43455000	8.29E-03	promoter	super_enhancer	RNMTL1
chr17	685000	chr17	80775000	4.93E-03	promoter	super_enhancer	RNMTL1
chr17	695000	chr17	905000	3.43E-02	promoter	super_enhancer	RP11-676J12.8
chr17	695000	chr17	17075000	2.51E-02	promoter	super_enhancer	RP11-676J12.8
chr17	695000	chr17	48065000	7.61E-03	promoter	super_enhancer	RP11-676J12.8
chr17	695000	chr17	57925000	5.55E-03	promoter	super_enhancer	RP11-676J12.8
chr17	695000	chr17	73345000	3.95E-03	promoter	super_enhancer	RP11-676J12.8
chr17	885000	chr17	42015000	1.20E-02	promoter	super_enhancer	AC015884.1

chr17	885000	chr17	49525000	7.20E-03	promoter	super_enhancer	AC015884.1
chr17	885000	chr17	62775000	6.52E-03	promoter	super_enhancer	AC015884.1
chr17	885000	chr17	80265000	6.09E-03	promoter	super_enhancer	AC015884.1
chr17	885000	chr17	80775000	5.64E-03	promoter	super_enhancer	AC015884.1
chr17	885000	chr17	81095000	4.17E-03	promoter	super_enhancer	AC015884.1
chr17	905000	chr17	6915000	3.78E-02	super_enhancer	promoter	RNASEK
chr17	905000	chr17	7145000	3.60E-02	super_enhancer	promoter	CTD-2545G14.6
chr17	905000	chr17	7185000	3.73E-02	super_enhancer	promoter	SLC2A4
chr17	905000	chr17	26795000	1.19E-02	super_enhancer	promoter	RPS7P1
chr17	905000	chr17	30655000	1.32E-02	super_enhancer	promoter	RP11-227G15.2
chr17	905000	chr17	40665000	7.26E-03	super_enhancer	promoter	MIR5010
chr17	905000	chr17	40975000	6.94E-03	super_enhancer	promoter	PSME3
chr17	905000	chr17	75915000	3.51E-03	super_enhancer	promoter	RNU1-80P
chr17	905000	chr17	77705000	4.35E-03	super_enhancer	promoter	ENPP7
chr17	915000	chr17	5095000	4.65E-02	super_enhancer	promoter	ZNF594
chr17	915000	chr17	69295000	3.08E-03	super_enhancer	promoter	RNU7-155P
chr17	915000	chr17	77775000	3.48E-03	super_enhancer	promoter	CBX8
chr17	915000	chr17	80195000	2.50E-03	super_enhancer	promoter	MIR6787
chr17	925000	chr17	6505000	4.26E-02	super_enhancer	promoter	RNA5SP435
chr17	925000	chr17	7155000	4.26E-02	super_enhancer	promoter	ELP5
chr17	925000	chr17	17875000	2.29E-02	super_enhancer	promoter	LRRC48
chr17	925000	chr17	29845000	1.53E-02	super_enhancer	promoter	RN7SL45P
chr17	935000	chr17	1085000	9.23E-03	super_enhancer	promoter	AC016292.1
chr17	935000	chr17	6985000	3.99E-02	super_enhancer	promoter	CLEC10A

chr17	935000	chr17	7475000	4.01E-02	super_enhancer	promoter	EIF4A1
chr17	935000	chr17	13845000	1.61E-02	super_enhancer	promoter	AC005304.1
chr17	935000	chr17	26925000	1.72E-02	super_enhancer	promoter	SPAG5-AS1
chr17	935000	chr17	39095000	8.35E-03	super_enhancer	promoter	KRT23
chr17	935000	chr17	42145000	1.05E-02	super_enhancer	promoter	LSM12
chr17	935000	chr17	79885000	5.05E-03	super_enhancer	promoter	MAFG-AS1
chr17	945000	chr17	1085000	2.77E-02	super_enhancer	promoter	AC016292.1
chr17	945000	chr17	5675000	3.74E-02	super_enhancer	promoter	WSCD1
chr17	945000	chr17	16595000	1.14E-02	super_enhancer	promoter	RNU6-405P
chr17	945000	chr17	40835000	9.54E-03	super_enhancer	promoter	CNTNAP1
chr17	945000	chr17	43225000	5.37E-03	super_enhancer	promoter	HEXIM1
chr17	955000	chr17	17675000	2.08E-02	super_enhancer	promoter	RAI1-AS1
chr17	955000	chr17	40685000	8.54E-03	super_enhancer	promoter	PTP4A2P1
chr17	955000	chr17	41995000	8.62E-03	super_enhancer	promoter	FAM215A
chr17	955000	chr17	74565000	4.29E-03	super_enhancer	promoter	RP11-666A8.9
chr17	955000	chr17	79885000	4.68E-03	super_enhancer	promoter	MAFG-AS1
chr17	965000	chr17	4125000	1.95E-03	super_enhancer	promoter	RYKP1
chr17	965000	chr17	26035000	9.41E-03	super_enhancer	promoter	RP1-66C13.2
chr17	965000	chr17	79925000	4.14E-03	super_enhancer	promoter	RP11-498C9.17
chr17	965000	chr17	80675000	3.01E-03	super_enhancer	promoter	RP11-388C12.1
chr17	1085000	chr17	1485000	2.48E-02	promoter	super_enhancer	AC016292.1
chr17	1085000	chr17	36875000	1.26E-02	promoter	super_enhancer	AC016292.1
chr17	1085000	chr17	42335000	1.19E-02	promoter	super_enhancer	AC016292.1
chr17	1085000	chr17	48055000	8.24E-03	promoter	super_enhancer	AC016292.1

chr17	1085000	chr17	80255000	4.70E-03	promoter	super_enhancer	AC016292.1
chr17	1085000	chr17	80495000	1.13E-05	promoter	super_enhancer	AC016292.1
chr17	1085000	chr17	80785000	3.41E-03	promoter	super_enhancer	AC016292.1
chr17	1085000	chr17	80835000	4.49E-03	promoter	super_enhancer	AC016292.1
chr17	1115000	chr17	1945000	4.38E-02	super_enhancer	promoter	OVCA2
chr17	1115000	chr17	7255000	4.66E-02	super_enhancer	promoter	KCTD11
chr17	1115000	chr17	10195000	2.67E-02	super_enhancer	promoter	RP11-401O9.4
chr17	1115000	chr17	18245000	1.90E-02	super_enhancer	promoter	MIR6778
chr17	1115000	chr17	42875000	1.04E-02	super_enhancer	promoter	CTC-296K1.4
chr17	1115000	chr17	56555000	5.52E-03	super_enhancer	promoter	SETP3
chr17	1255000	chr17	17015000	2.12E-02	promoter	super_enhancer	RP11-818O24.2
chr17	1255000	chr17	77315000	3.86E-03	promoter	super_enhancer	RP11-818O24.2
chr17	1305000	chr17	77305000	2.98E-03	promoter	super_enhancer	YWHAE
chr17	1485000	chr17	7465000	4.36E-02	super_enhancer	promoter	SENP3
chr17	1485000	chr17	7905000	4.63E-02	super_enhancer	promoter	GUCY2D
chr17	1485000	chr17	31255000	1.18E-02	super_enhancer	promoter	TMEM98
chr17	1485000	chr17	38215000	1.22E-02	super_enhancer	promoter	THRA
chr17	1485000	chr17	47485000	1.09E-02	super_enhancer	promoter	AC091180.1
chr17	1485000	chr17	55175000	8.03E-03	super_enhancer	promoter	RP11-166P13.4
chr17	1555000	chr17	6555000	4.28E-02	super_enhancer	promoter	MED31
chr17	1555000	chr17	13505000	2.99E-02	super_enhancer	promoter	HS3ST3A1
chr17	1555000	chr17	73975000	5.47E-03	super_enhancer	promoter	ACOX1
chr17	1565000	chr17	55355000	6.65E-03	super_enhancer	promoter	RP11-426D19.1
chr17	1565000	chr17	80025000	2.61E-03	super_enhancer	promoter	DUS1L

chr17	1575000	chr17	1735000	1.45E-03	super_enhancer	promoter	SMYD4
chr17	1575000	chr17	10325000	1.52E-02	super_enhancer	promoter	MYH8
chr17	1575000	chr17	15695000	1.00E-02	super_enhancer	promoter	AC015922.7
chr17	1575000	chr17	28705000	1.32E-02	super_enhancer	promoter	CPD
chr17	1575000	chr17	46725000	9.26E-03	super_enhancer	promoter	RP11-357H14.17
chr17	1575000	chr17	58755000	6.89E-03	super_enhancer	promoter	BCAS3
chr17	1575000	chr17	73495000	5.78E-03	super_enhancer	promoter	MIR6785
chr17	1575000	chr17	79375000	3.67E-03	super_enhancer	promoter	MIR4740
chr17	1585000	chr17	1665000	1.14E-02	super_enhancer	promoter	SERPINF1
chr17	1585000	chr17	1735000	3.33E-02	super_enhancer	promoter	SMYD4
chr17	1585000	chr17	18085000	2.58E-02	super_enhancer	promoter	ALKBH5
chr17	1585000	chr17	28615000	1.05E-02	super_enhancer	promoter	RNU6-1267P
chr17	1595000	chr17	34145000	6.38E-03	super_enhancer	promoter	AC015849.13
chr17	1595000	chr17	40665000	7.75E-03	super_enhancer	promoter	MIR5010
chr17	1595000	chr17	48555000	6.98E-03	super_enhancer	promoter	RSAD1
chr17	1595000	chr17	79365000	3.65E-03	super_enhancer	promoter	RP11-1055B8.4
chr17	1615000	chr17	27345000	1.42E-02	promoter	super_enhancer	TLCD2
chr17	1615000	chr17	27535000	1.25E-02	promoter	super_enhancer	TLCD2
chr17	1615000	chr17	38415000	8.18E-03	promoter	super_enhancer	TLCD2
chr17	1615000	chr17	38445000	9.84E-03	promoter	super_enhancer	TLCD2
chr17	1615000	chr17	80825000	4.64E-03	promoter	super_enhancer	TLCD2
chr17	1645000	chr17	17105000	2.03E-02	promoter	super_enhancer	SERPINF2
chr17	1645000	chr17	17695000	1.58E-02	promoter	super_enhancer	SERPINF2
chr17	1645000	chr17	29155000	1.26E-02	promoter	super_enhancer	SERPINF2

chr17	1645000	chr17	40485000	6.71E-03	promoter	super_enhancer	SERPINF2
chr17	1645000	chr17	75715000	3.70E-03	promoter	super_enhancer	SERPINF2
chr17	1645000	chr17	77315000	3.21E-03	promoter	super_enhancer	SERPINF2
chr17	1645000	chr17	79845000	2.85E-03	promoter	super_enhancer	SERPINF2
chr17	1665000	chr17	80815000	4.54E-03	promoter	super_enhancer	SERPINF1
chr17	1735000	chr17	16275000	2.21E-02	promoter	super_enhancer	SMYD4
chr17	1735000	chr17	17015000	2.39E-02	promoter	super_enhancer	SMYD4
chr17	1735000	chr17	42415000	1.05E-02	promoter	super_enhancer	SMYD4
chr17	1945000	chr17	77315000	4.41E-03	promoter	super_enhancer	OVCA2
chr17	1955000	chr17	17745000	2.23E-02	promoter	super_enhancer	MIR212
chr17	1955000	chr17	17755000	1.80E-04	promoter	super_enhancer	MIR212
chr17	1955000	chr17	42395000	1.30E-02	promoter	super_enhancer	MIR212
chr17	1955000	chr17	59835000	5.89E-03	promoter	super_enhancer	MIR212
chr17	1955000	chr17	77295000	5.04E-03	promoter	super_enhancer	MIR212
chr17	1955000	chr17	80815000	4.47E-03	promoter	super_enhancer	MIR212
chr17	1955000	chr17	80825000	5.88E-03	promoter	super_enhancer	MIR212
chr17	1975000	chr17	2405000	4.95E-03	promoter	super_enhancer	RP11-667K14.14
chr17	1975000	chr17	36875000	1.19E-02	promoter	super_enhancer	RP11-667K14.14
chr17	1975000	chr17	38425000	8.20E-03	promoter	super_enhancer	RP11-667K14.14
chr17	1975000	chr17	78605000	2.98E-03	promoter	super_enhancer	RP11-667K14.14
chr17	1975000	chr17	79805000	4.27E-03	promoter	super_enhancer	RP11-667K14.14
chr17	2005000	chr17	17765000	2.56E-02	promoter	super_enhancer	RP11-667K14.13
chr17	2005000	chr17	27565000	1.59E-02	promoter	super_enhancer	RP11-667K14.13
chr17	2005000	chr17	80825000	6.05E-03	promoter	super_enhancer	RP11-667K14.13

chr17	2135000	chr17	17705000	1.62E-02	promoter	super_enhancer	AL450226.2
chr17	2135000	chr17	80755000	2.27E-03	promoter	super_enhancer	AL450226.2
chr17	2235000	chr17	42395000	9.84E-03	promoter	super_enhancer	SNORD91A
chr17	2305000	chr17	17115000	2.71E-02	promoter	super_enhancer	MNT
chr17	2305000	chr17	38885000	8.89E-03	promoter	super_enhancer	MNT
chr17	2305000	chr17	80265000	5.99E-03	promoter	super_enhancer	MNT
chr17	2365000	chr17	17015000	2.26E-02	promoter	super_enhancer	RP1-59D14.8
chr17	2365000	chr17	17095000	2.44E-02	promoter	super_enhancer	RP1-59D14.8
chr17	2365000	chr17	17745000	2.01E-02	promoter	super_enhancer	RP1-59D14.8
chr17	2365000	chr17	21295000	1.52E-02	promoter	super_enhancer	RP1-59D14.8
chr17	2365000	chr17	50655000	4.92E-03	promoter	super_enhancer	RP1-59D14.8
chr17	2365000	chr17	80775000	4.72E-03	promoter	super_enhancer	RP1-59D14.8
chr17	2385000	chr17	7835000	4.35E-02	super_enhancer	promoter	RP11-1099M24.7
chr17	2385000	chr17	40265000	9.87E-03	super_enhancer	promoter	DHX58
chr17	2395000	chr17	37355000	1.01E-02	super_enhancer	promoter	CACNB1
chr17	2405000	chr17	6505000	4.65E-02	super_enhancer	promoter	RNA5SP435
chr17	2405000	chr17	8095000	3.83E-02	super_enhancer	promoter	C17orf59
chr17	2405000	chr17	33905000	7.80E-03	super_enhancer	promoter	AP2B1
chr17	2405000	chr17	35295000	1.22E-02	super_enhancer	promoter	RP11-445F12.1
chr17	2405000	chr17	46035000	7.97E-03	super_enhancer	promoter	PRR15L
chr17	2405000	chr17	76275000	3.32E-03	super_enhancer	promoter	RP11-219G17.4
chr17	2415000	chr17	17495000	2.09E-02	super_enhancer	promoter	PEMT
chr17	2415000	chr17	18015000	2.71E-02	super_enhancer	promoter	RP11-258F1.2
chr17	2415000	chr17	43685000	6.42E-03	super_enhancer	promoter	RPS26P8

chr17	2615000	chr17	17105000	2.92E-02	promoter	super_enhancer	RP11-74E22.3
chr17	2615000	chr17	27335000	1.79E-02	promoter	super_enhancer	RP11-74E22.3
chr17	2615000	chr17	28745000	1.36E-02	promoter	super_enhancer	RP11-74E22.3
chr17	2615000	chr17	38415000	1.06E-02	promoter	super_enhancer	RP11-74E22.3
chr17	2615000	chr17	40095000	1.19E-02	promoter	super_enhancer	RP11-74E22.3
chr17	2615000	chr17	40105000	1.07E-02	promoter	super_enhancer	RP11-74E22.3
chr17	2615000	chr17	73315000	5.28E-03	promoter	super_enhancer	RP11-74E22.3
chr17	2615000	chr17	79805000	4.94E-03	promoter	super_enhancer	RP11-74E22.3
chr17	3345000	chr17	75085000	3.90E-03	promoter	super_enhancer	RP11-147K16.3
chr17	3375000	chr17	48055000	8.47E-03	promoter	super_enhancer	ASPA
chr17	3375000	chr17	67415000	3.62E-03	promoter	super_enhancer	ASPA
chr17	3375000	chr17	77255000	5.06E-03	promoter	super_enhancer	ASPA
chr17	3625000	chr17	75705000	3.99E-03	promoter	super_enhancer	CTD-3195I5.3
chr17	3625000	chr17	80485000	4.02E-03	promoter	super_enhancer	CTD-3195I5.3
chr17	4045000	chr17	56925000	3.13E-03	promoter	super_enhancer	CYB5D2
chr17	4125000	chr17	38475000	8.89E-03	promoter	super_enhancer	RYKP1
chr17	4125000	chr17	75125000	4.09E-03	promoter	super_enhancer	RYKP1
chr17	4205000	chr17	16295000	2.04E-02	promoter	super_enhancer	MFSD1P1
chr17	4205000	chr17	17075000	2.35E-02	promoter	super_enhancer	MFSD1P1
chr17	4205000	chr17	36845000	9.48E-03	promoter	super_enhancer	MFSD1P1
chr17	4205000	chr17	43415000	1.08E-02	promoter	super_enhancer	MFSD1P1
chr17	4205000	chr17	78345000	3.58E-03	promoter	super_enhancer	MFSD1P1
chr17	4205000	chr17	80475000	4.24E-03	promoter	super_enhancer	MFSD1P1
chr17	4205000	chr17	80855000	3.03E-03	promoter	super_enhancer	MFSD1P1

chr17	4465000	chr17	17035000	2.15E-02	promoter	super_enhancer	GGT6
chr17	4465000	chr17	68355000	3.70E-03	promoter	super_enhancer	GGT6
chr17	4475000	chr17	17855000	2.40E-02	promoter	super_enhancer	TXNP4
chr17	4475000	chr17	42395000	1.30E-02	promoter	super_enhancer	TXNP4
chr17	4635000	chr17	17095000	3.02E-02	promoter	super_enhancer	MED11
chr17	4635000	chr17	42395000	1.38E-02	promoter	super_enhancer	MED11
chr17	4635000	chr17	43355000	1.08E-02	promoter	super_enhancer	MED11
chr17	4675000	chr17	17125000	3.79E-02	promoter	super_enhancer	TM4SF5
chr17	4675000	chr17	29145000	1.78E-02	promoter	super_enhancer	TM4SF5
chr17	4785000	chr17	17075000	2.75E-02	promoter	super_enhancer	RN7SL784P
chr17	4785000	chr17	17095000	2.58E-02	promoter	super_enhancer	RN7SL784P
chr17	4785000	chr17	17735000	2.34E-02	promoter	super_enhancer	RN7SL784P
chr17	4785000	chr17	78535000	4.13E-03	promoter	super_enhancer	RN7SL784P
chr17	4785000	chr17	79845000	3.47E-03	promoter	super_enhancer	RN7SL784P
chr17	4785000	chr17	80895000	3.79E-03	promoter	super_enhancer	RN7SL784P
chr17	4835000	chr17	77305000	4.08E-03	promoter	super_enhancer	GP1BA
chr17	4835000	chr17	80255000	5.31E-03	promoter	super_enhancer	GP1BA
chr17	4845000	chr17	36875000	1.59E-02	promoter	super_enhancer	SLC25A11
chr17	4845000	chr17	57875000	5.77E-03	promoter	super_enhancer	SLC25A11
chr17	4845000	chr17	57955000	7.27E-03	promoter	super_enhancer	SLC25A11
chr17	4875000	chr17	17725000	4.16E-04	promoter	super_enhancer	RP5-1050D4.2
chr17	4875000	chr17	36845000	1.49E-02	promoter	super_enhancer	RP5-1050D4.2
chr17	4875000	chr17	45285000	1.08E-02	promoter	super_enhancer	RP5-1050D4.2
chr17	4875000	chr17	75845000	4.00E-03	promoter	super_enhancer	RP5-1050D4.2

chr17	4875000	chr17	78595000	5.65E-03	promoter	super_enhancer	RP5-1050D4.2
chr17	4875000	chr17	79835000	5.17E-03	promoter	super_enhancer	RP5-1050D4.2
chr17	4955000	chr17	9835000	4.35E-02	promoter	super_enhancer	SLC52A1
chr17	4955000	chr17	17125000	3.91E-02	promoter	super_enhancer	SLC52A1
chr17	4955000	chr17	27525000	1.56E-02	promoter	super_enhancer	SLC52A1
chr17	4955000	chr17	27545000	1.39E-02	promoter	super_enhancer	SLC52A1
chr17	4955000	chr17	73315000	5.76E-03	promoter	super_enhancer	SLC52A1
chr17	4955000	chr17	78345000	5.14E-03	promoter	super_enhancer	SLC52A1
chr17	5095000	chr17	17065000	2.01E-02	promoter	super_enhancer	ZNF594
chr17	5095000	chr17	17105000	2.66E-02	promoter	super_enhancer	ZNF594
chr17	5095000	chr17	17685000	2.29E-02	promoter	super_enhancer	ZNF594
chr17	5095000	chr17	36835000	1.17E-02	promoter	super_enhancer	ZNF594
chr17	5095000	chr17	36855000	1.18E-02	promoter	super_enhancer	ZNF594
chr17	5095000	chr17	42015000	1.03E-02	promoter	super_enhancer	ZNF594
chr17	5095000	chr17	73295000	4.50E-03	promoter	super_enhancer	ZNF594
chr17	5095000	chr17	75685000	3.92E-03	promoter	super_enhancer	ZNF594
chr17	5185000	chr17	15955000	2.41E-02	promoter	super_enhancer	RABEP1
chr17	5185000	chr17	17855000	2.29E-02	promoter	super_enhancer	RABEP1
chr17	5185000	chr17	29135000	1.33E-02	promoter	super_enhancer	RABEP1
chr17	5185000	chr17	38425000	9.00E-03	promoter	super_enhancer	RABEP1
chr17	5185000	chr17	75085000	4.91E-03	promoter	super_enhancer	RABEP1
chr17	5185000	chr17	78885000	4.17E-03	promoter	super_enhancer	RABEP1
chr17	5275000	chr17	16295000	2.81E-02	promoter	super_enhancer	RP11-420A6.2
chr17	5275000	chr17	17755000	2.11E-02	promoter	super_enhancer	RP11-420A6.2

chr17	5275000	chr17	29175000	1.52E-02	promoter	super_enhancer	RP11-420A6.2
chr17	5275000	chr17	64325000	5.97E-03	promoter	super_enhancer	RP11-420A6.2
chr17	5275000	chr17	78585000	4.31E-03	promoter	super_enhancer	RP11-420A6.2
chr17	5275000	chr17	80755000	3.60E-03	promoter	super_enhancer	RP11-420A6.2
chr17	5675000	chr17	17765000	2.30E-02	promoter	super_enhancer	WSCD1
chr17	5675000	chr17	27565000	1.37E-02	promoter	super_enhancer	WSCD1
chr17	5675000	chr17	57895000	5.02E-03	promoter	super_enhancer	WSCD1
chr17	6095000	chr17	29175000	1.44E-02	promoter	super_enhancer	RP11-960B9.2
chr17	6095000	chr17	80485000	4.24E-03	promoter	super_enhancer	RP11-960B9.2
chr17	6505000	chr17	17065000	2.00E-02	promoter	super_enhancer	RNA5SP435
chr17	6505000	chr17	27545000	1.06E-02	promoter	super_enhancer	RNA5SP435
chr17	6505000	chr17	57875000	4.21E-03	promoter	super_enhancer	RNA5SP435
chr17	6545000	chr17	15965000	2.03E-02	promoter	super_enhancer	KIAA0753
chr17	6545000	chr17	27355000	1.92E-02	promoter	super_enhancer	KIAA0753
chr17	6545000	chr17	42425000	9.72E-03	promoter	super_enhancer	KIAA0753
chr17	6545000	chr17	80475000	4.89E-03	promoter	super_enhancer	KIAA0753
chr17	6555000	chr17	36855000	9.88E-03	promoter	super_enhancer	MED31
chr17	6555000	chr17	42425000	7.68E-03	promoter	super_enhancer	MED31
chr17	6555000	chr17	62775000	4.52E-03	promoter	super_enhancer	MED31
chr17	6735000	chr17	17055000	2.71E-02	promoter	super_enhancer	TEKT1
chr17	6735000	chr17	17085000	2.47E-02	promoter	super_enhancer	TEKT1
chr17	6735000	chr17	36885000	1.28E-02	promoter	super_enhancer	TEKT1
chr17	6735000	chr17	40315000	1.23E-02	promoter	super_enhancer	TEKT1
chr17	6735000	chr17	42005000	1.03E-02	promoter	super_enhancer	TEKT1

chr17	6735000	chr17	42405000	1.20E-02	promoter	super_enhancer	TEKT1
chr17	6735000	chr17	75125000	5.50E-03	promoter	super_enhancer	TEKT1
chr17	6915000	chr17	16295000	3.17E-02	promoter	super_enhancer	RNASEK
chr17	6915000	chr17	36825000	1.49E-02	promoter	super_enhancer	RNASEK
chr17	6915000	chr17	36865000	1.39E-02	promoter	super_enhancer	RNASEK
chr17	6915000	chr17	40515000	1.26E-02	promoter	super_enhancer	RNASEK
chr17	6915000	chr17	56915000	7.46E-03	promoter	super_enhancer	RNASEK
chr17	6925000	chr17	17015000	3.02E-02	promoter	super_enhancer	BCL6B
chr17	6925000	chr17	27345000	2.06E-02	promoter	super_enhancer	BCL6B
chr17	6985000	chr17	15985000	2.18E-02	promoter	super_enhancer	CLEC10A
chr17	6985000	chr17	17015000	2.62E-02	promoter	super_enhancer	CLEC10A
chr17	6985000	chr17	48065000	7.98E-03	promoter	super_enhancer	CLEC10A
chr17	6985000	chr17	57885000	3.59E-03	promoter	super_enhancer	CLEC10A
chr17	6985000	chr17	64355000	5.02E-03	promoter	super_enhancer	CLEC10A
chr17	7145000	chr17	17705000	3.17E-02	promoter	super_enhancer	CTD-2545G14.6
chr17	7145000	chr17	17725000	2.79E-02	promoter	super_enhancer	CTD-2545G14.6
chr17	7155000	chr17	48065000	8.18E-03	promoter	super_enhancer	ELP5
chr17	7165000	chr17	16285000	3.19E-02	promoter	super_enhancer	RP1-4G17.5
chr17	7165000	chr17	17055000	2.66E-02	promoter	super_enhancer	RP1-4G17.5
chr17	7165000	chr17	42015000	1.26E-02	promoter	super_enhancer	RP1-4G17.5
chr17	7165000	chr17	72395000	5.32E-03	promoter	super_enhancer	RP1-4G17.5
chr17	7165000	chr17	78345000	4.78E-03	promoter	super_enhancer	RP1-4G17.5
chr17	7185000	chr17	17725000	2.92E-02	promoter	super_enhancer	SLC2A4
chr17	7185000	chr17	27525000	1.69E-02	promoter	super_enhancer	SLC2A4

chr17	7185000	chr17	57895000	6.88E-03	promoter	super_enhancer	SLC2A4
chr17	7185000	chr17	75085000	6.07E-03	promoter	super_enhancer	SLC2A4
chr17	7185000	chr17	79785000	5.97E-03	promoter	super_enhancer	SLC2A4
chr17	7255000	chr17	17685000	3.01E-02	promoter	super_enhancer	KCTD11
chr17	7255000	chr17	36845000	1.36E-02	promoter	super_enhancer	KCTD11
chr17	7255000	chr17	36885000	1.29E-02	promoter	super_enhancer	KCTD11
chr17	7255000	chr17	59825000	5.33E-03	promoter	super_enhancer	KCTD11
chr17	7255000	chr17	79795000	5.41E-03	promoter	super_enhancer	KCTD11
chr17	7385000	chr17	17085000	2.09E-02	promoter	super_enhancer	SLC35G6
chr17	7385000	chr17	17735000	2.51E-02	promoter	super_enhancer	SLC35G6
chr17	7385000	chr17	36865000	1.13E-02	promoter	super_enhancer	SLC35G6
chr17	7465000	chr17	15955000	2.62E-02	promoter	super_enhancer	SENP3
chr17	7465000	chr17	15965000	2.17E-02	promoter	super_enhancer	SENP3
chr17	7475000	chr17	17065000	2.50E-02	promoter	super_enhancer	EIF4A1
chr17	7475000	chr17	29125000	1.90E-02	promoter	super_enhancer	EIF4A1
chr17	7475000	chr17	56925000	4.95E-03	promoter	super_enhancer	EIF4A1
chr17	7475000	chr17	78885000	4.48E-03	promoter	super_enhancer	EIF4A1
chr17	7835000	chr17	17035000	3.36E-02	promoter	super_enhancer	RP11-1099M24.7
chr17	7835000	chr17	17125000	4.31E-02	promoter	super_enhancer	RP11-1099M24.7
chr17	7835000	chr17	17755000	2.47E-02	promoter	super_enhancer	RP11-1099M24.7
chr17	7835000	chr17	27535000	1.94E-04	promoter	super_enhancer	RP11-1099M24.7
chr17	7835000	chr17	29135000	1.68E-02	promoter	super_enhancer	RP11-1099M24.7
chr17	7835000	chr17	36825000	1.52E-02	promoter	super_enhancer	RP11-1099M24.7
chr17	7835000	chr17	38465000	1.25E-02	promoter	super_enhancer	RP11-1099M24.7

chr17	7835000	chr17	42335000	1.51E-02	promoter	super_enhancer	RP11-1099M24.7
chr17	7845000	chr17	7905000	4.60E-02	super_enhancer	promoter	GUCY2D
chr17	7845000	chr17	11925000	4.53E-02	super_enhancer	promoter	RPL21P122
chr17	7845000	chr17	12695000	3.66E-02	super_enhancer	promoter	RP11-1090M7.1
chr17	7845000	chr17	12895000	4.05E-02	super_enhancer	promoter	RP11-597M12.1
chr17	7845000	chr17	15155000	3.91E-02	super_enhancer	promoter	MIR4731
chr17	7845000	chr17	15215000	3.34E-02	super_enhancer	promoter	RNU6-799P
chr17	7845000	chr17	16715000	1.66E-02	super_enhancer	promoter	NOS2P4
chr17	7845000	chr17	18085000	4.07E-02	super_enhancer	promoter	ALKBH5
chr17	7845000	chr17	18565000	1.03E-02	super_enhancer	promoter	TBC1D28
chr17	7845000	chr17	43225000	8.23E-03	super_enhancer	promoter	HEXIM1
chr17	7845000	chr17	58755000	8.27E-03	super_enhancer	promoter	BCAS3
chr17	7845000	chr17	77775000	4.72E-03	super_enhancer	promoter	CBX8
chr17	7905000	chr17	21285000	2.32E-02	promoter	super_enhancer	GUCY2D
chr17	7905000	chr17	79785000	5.49E-03	promoter	super_enhancer	GUCY2D
chr17	8095000	chr17	17065000	2.60E-02	promoter	super_enhancer	C17orf59
chr17	8095000	chr17	17105000	3.44E-02	promoter	super_enhancer	C17orf59
chr17	8095000	chr17	17125000	3.96E-02	promoter	super_enhancer	C17orf59
chr17	8095000	chr17	21295000	1.98E-02	promoter	super_enhancer	C17orf59
chr17	8095000	chr17	27565000	1.50E-04	promoter	super_enhancer	C17orf59
chr17	8095000	chr17	29135000	1.53E-02	promoter	super_enhancer	C17orf59
chr17	8095000	chr17	45375000	1.08E-02	promoter	super_enhancer	C17orf59
chr17	8115000	chr17	17125000	4.58E-02	promoter	super_enhancer	AURKB
chr17	8115000	chr17	27335000	2.28E-02	promoter	super_enhancer	AURKB

chr17	8315000	chr17	15985000	2.80E-02	promoter	super_enhancer	NDEL1
chr17	8315000	chr17	17125000	4.22E-02	promoter	super_enhancer	NDEL1
chr17	8315000	chr17	29155000	2.07E-02	promoter	super_enhancer	NDEL1
chr17	8315000	chr17	38445000	1.43E-02	promoter	super_enhancer	NDEL1
chr17	8465000	chr17	17035000	2.63E-02	promoter	super_enhancer	RP11-713H12.1
chr17	8465000	chr17	17685000	2.49E-02	promoter	super_enhancer	RP11-713H12.1
chr17	8465000	chr17	17825000	2.39E-02	promoter	super_enhancer	RP11-713H12.1
chr17	8465000	chr17	17845000	2.05E-02	promoter	super_enhancer	RP11-713H12.1
chr17	8465000	chr17	75715000	4.29E-03	promoter	super_enhancer	RP11-713H12.1
chr17	8465000	chr17	80895000	3.54E-03	promoter	super_enhancer	RP11-713H12.1
chr17	8535000	chr17	29145000	2.01E-02	promoter	super_enhancer	MYH10
chr17	8535000	chr17	57895000	6.92E-03	promoter	super_enhancer	MYH10
chr17	8535000	chr17	77295000	5.64E-03	promoter	super_enhancer	MYH10
chr17	8535000	chr17	78505000	6.33E-03	promoter	super_enhancer	MYH10
chr17	8925000	chr17	17115000	2.99E-02	promoter	super_enhancer	NTN1
chr17	8925000	chr17	17845000	2.28E-02	promoter	super_enhancer	NTN1
chr17	9185000	chr17	15955000	3.13E-02	promoter	super_enhancer	RP11-85B7.4
chr17	9355000	chr17	17025000	2.44E-02	promoter	super_enhancer	AC087501.1
chr17	9355000	chr17	17035000	3.05E-02	promoter	super_enhancer	AC087501.1
chr17	9355000	chr17	17065000	2.57E-02	promoter	super_enhancer	AC087501.1
chr17	9355000	chr17	17715000	2.78E-02	promoter	super_enhancer	AC087501.1
chr17	9355000	chr17	64355000	5.08E-03	promoter	super_enhancer	AC087501.1
chr17	9695000	chr17	78585000	4.70E-03	promoter	super_enhancer	DHRS7C
chr17	9725000	chr17	17685000	2.64E-02	promoter	super_enhancer	RP11-477N12.3

chr17	9725000	chr17	36875000	1.23E-02	promoter	super_enhancer	RP11-477N12.3
chr17	9835000	chr17	15245000	2.74E-02	super_enhancer	promoter	TEKT3
chr17	9835000	chr17	30655000	1.60E-02	super_enhancer	promoter	RP11-227G15.2
chr17	9835000	chr17	40195000	9.37E-03	super_enhancer	promoter	CTD-2132N18.2
chr17	10195000	chr17	15955000	3.48E-02	promoter	super_enhancer	RP11-401O9.4
chr17	10195000	chr17	17025000	2.64E-02	promoter	super_enhancer	RP11-401O9.4
chr17	10195000	chr17	17825000	2.87E-02	promoter	super_enhancer	RP11-401O9.4
chr17	10195000	chr17	43355000	9.89E-03	promoter	super_enhancer	RP11-401O9.4
chr17	10285000	chr17	29155000	2.18E-02	promoter	super_enhancer	RP11-799N11.1
chr17	10285000	chr17	80855000	4.16E-03	promoter	super_enhancer	RP11-799N11.1
chr17	10325000	chr17	15975000	1.77E-02	promoter	super_enhancer	MYH8
chr17	10325000	chr17	17035000	2.07E-02	promoter	super_enhancer	MYH8
chr17	10635000	chr17	17095000	3.26E-02	promoter	super_enhancer	TMEM220
chr17	10635000	chr17	17765000	2.81E-02	promoter	super_enhancer	TMEM220
chr17	10635000	chr17	72395000	4.13E-03	promoter	super_enhancer	TMEM220
chr17	10695000	chr17	16275000	4.01E-02	promoter	super_enhancer	RP11-963H4.5
chr17	10695000	chr17	17705000	3.72E-02	promoter	super_enhancer	RP11-963H4.5
chr17	10695000	chr17	29115000	1.79E-02	promoter	super_enhancer	RP11-963H4.5
chr17	10695000	chr17	80265000	5.75E-03	promoter	super_enhancer	RP11-963H4.5
chr17	10765000	chr17	16295000	3.68E-02	promoter	super_enhancer	RPL15P21
chr17	10765000	chr17	17725000	2.79E-02	promoter	super_enhancer	RPL15P21
chr17	10765000	chr17	27525000	1.39E-02	promoter	super_enhancer	RPL15P21
chr17	11145000	chr17	41785000	1.17E-02	promoter	super_enhancer	SHISA6
chr17	11145000	chr17	50635000	5.70E-03	promoter	super_enhancer	SHISA6

chr17	11925000	chr17	15975000	3.47E-02	promoter	super_enhancer	RPL21P122
chr17	11925000	chr17	28745000	1.24E-02	promoter	super_enhancer	RPL21P122
chr17	11985000	chr17	17045000	4.01E-02	promoter	super_enhancer	MIR744
chr17	11985000	chr17	17745000	3.20E-02	promoter	super_enhancer	MIR744
chr17	11985000	chr17	21295000	1.87E-02	promoter	super_enhancer	MIR744
chr17	11985000	chr17	43455000	8.64E-03	promoter	super_enhancer	MIR744
chr17	12105000	chr17	27565000	1.85E-02	promoter	super_enhancer	RP11-471L13.2
chr17	12105000	chr17	57935000	7.55E-03	promoter	super_enhancer	RP11-471L13.2
chr17	12695000	chr17	17055000	3.80E-02	promoter	super_enhancer	RP11-1090M7.1
chr17	12695000	chr17	17825000	6.07E-04	promoter	super_enhancer	RP11-1090M7.1
chr17	12895000	chr17	17685000	4.46E-02	promoter	super_enhancer	RP11-597M12.1
chr17	12895000	chr17	17725000	4.02E-02	promoter	super_enhancer	RP11-597M12.1
chr17	12895000	chr17	42335000	1.30E-02	promoter	super_enhancer	RP11-597M12.1
chr17	12895000	chr17	50945000	5.27E-03	promoter	super_enhancer	RP11-597M12.1
chr17	13505000	chr17	80795000	5.10E-03	promoter	super_enhancer	HS3ST3A1
chr17	14685000	chr17	21295000	2.99E-02	promoter	super_enhancer	AC005863.1
chr17	15215000	chr17	17035000	1.54E-03	promoter	super_enhancer	RNU6-799P
chr17	15555000	chr17	21285000	4.19E-02	promoter	super_enhancer	RP11-385D13.1
chr17	15635000	chr17	27335000	1.69E-02	promoter	super_enhancer	TBC1D26
chr17	15635000	chr17	43395000	7.28E-03	promoter	super_enhancer	TBC1D26
chr17	15685000	chr17	17705000	1.67E-02	promoter	super_enhancer	RNA5SP436
chr17	15685000	chr17	40515000	1.48E-02	promoter	super_enhancer	RNA5SP436
chr17	15695000	chr17	62775000	3.57E-03	promoter	super_enhancer	AC015922.7
chr17	15945000	chr17	26585000	3.57E-02	super_enhancer	promoter	CTD-2008P7.7

chr17	15945000	chr17	30735000	3.00E-02	super_enhancer	promoter	RP11-227G15.10
chr17	15945000	chr17	43225000	1.18E-02	super_enhancer	promoter	HEXIM1
chr17	15945000	chr17	51185000	8.06E-03	super_enhancer	promoter	AC091154.1
chr17	15945000	chr17	58605000	1.03E-02	super_enhancer	promoter	APPBP2
chr17	15945000	chr17	76395000	6.61E-03	super_enhancer	promoter	SNORA30
chr17	15955000	chr17	20495000	3.64E-02	super_enhancer	promoter	ZSWIM5P2
chr17	15955000	chr17	55055000	7.78E-03	super_enhancer	promoter	SCPEP1
chr17	15955000	chr17	55695000	7.28E-03	super_enhancer	promoter	RN7SL449P
chr17	15965000	chr17	20495000	3.03E-02	super_enhancer	promoter	ZSWIM5P2
chr17	15965000	chr17	34345000	1.18E-02	super_enhancer	promoter	CCL23
chr17	15975000	chr17	29335000	1.59E-02	super_enhancer	promoter	RP11-848P1.7
chr17	15975000	chr17	33345000	1.41E-02	super_enhancer	promoter	RP5-837J1.6
chr17	15975000	chr17	34085000	1.48E-02	super_enhancer	promoter	RP1-161P9.5
chr17	15975000	chr17	34245000	1.28E-02	super_enhancer	promoter	LRRC37A9P
chr17	15975000	chr17	46115000	1.09E-02	super_enhancer	promoter	COPZ2
chr17	15985000	chr17	17325000	4.55E-02	super_enhancer	promoter	AC073621.2
chr17	15985000	chr17	29065000	1.38E-02	super_enhancer	promoter	CTD-2349P21.3
chr17	15985000	chr17	32015000	1.30E-02	super_enhancer	promoter	RP11-118G23.1
chr17	15995000	chr17	16875000	4.02E-02	super_enhancer	promoter	TNFRSF13B
chr17	15995000	chr17	40685000	7.14E-03	super_enhancer	promoter	PTP4A2P1
chr17	16095000	chr17	40095000	1.53E-02	promoter	super_enhancer	RN7SL442P
chr17	16185000	chr17	29125000	2.58E-02	promoter	super_enhancer	MIR1288
chr17	16185000	chr17	50925000	6.80E-03	promoter	super_enhancer	MIR1288
chr17	16275000	chr17	47925000	1.20E-02	super_enhancer	promoter	TAC4

chr17	16275000	chr17	66455000	5.89E-03	super_enhancer	promoter	WIPI1
chr17	16275000	chr17	80675000	4.41E-03	super_enhancer	promoter	RP11-388C12.1
chr17	16285000	chr17	26695000	3.46E-02	super_enhancer	promoter	CTB-96E2.2
chr17	16285000	chr17	29845000	2.59E-02	super_enhancer	promoter	RN7SL45P
chr17	16285000	chr17	30845000	1.57E-02	super_enhancer	promoter	RP11-466A19.3
chr17	16285000	chr17	49265000	1.03E-02	super_enhancer	promoter	RP11-481C4.2
chr17	16285000	chr17	66245000	5.65E-03	super_enhancer	promoter	RP11-147L13.2
chr17	16295000	chr17	27895000	2.42E-02	super_enhancer	promoter	ABHD15
chr17	16295000	chr17	29335000	2.11E-02	super_enhancer	promoter	RP11-848P1.7
chr17	16295000	chr17	33925000	1.75E-02	super_enhancer	promoter	CTC-507E2.2
chr17	16295000	chr17	55355000	9.77E-03	super_enhancer	promoter	RP11-426D19.1
chr17	16345000	chr17	17015000	1.72E-02	promoter	super_enhancer	SNORD65
chr17	16345000	chr17	17085000	3.48E-02	promoter	super_enhancer	SNORD65
chr17	16345000	chr17	27355000	3.64E-02	promoter	super_enhancer	SNORD65
chr17	16345000	chr17	73325000	7.12E-03	promoter	super_enhancer	SNORD65
chr17	16395000	chr17	17115000	2.30E-02	promoter	super_enhancer	LRRC75A
chr17	16395000	chr17	40115000	2.30E-02	promoter	super_enhancer	LRRC75A
chr17	16395000	chr17	42005000	1.67E-02	promoter	super_enhancer	LRRC75A
chr17	16395000	chr17	68335000	6.95E-03	promoter	super_enhancer	LRRC75A
chr17	16595000	chr17	21285000	3.49E-02	promoter	super_enhancer	RNU6-405P
chr17	16715000	chr17	27345000	1.69E-02	promoter	super_enhancer	NOS2P4
chr17	16715000	chr17	41785000	1.02E-02	promoter	super_enhancer	NOS2P4
chr17	16875000	chr17	17045000	4.33E-02	promoter	super_enhancer	TNFRSF13B
chr17	16875000	chr17	29105000	2.17E-02	promoter	super_enhancer	TNFRSF13B

chr17	16875000	chr17	38415000	1.54E-02	promoter	super_enhancer	TNFRSF13B
chr17	16875000	chr17	77435000	6.37E-03	promoter	super_enhancer	TNFRSF13B
chr17	16885000	chr17	17035000	1.67E-03	promoter	super_enhancer	AC104024.1
chr17	16885000	chr17	17845000	1.90E-02	promoter	super_enhancer	AC104024.1
chr17	16885000	chr17	42345000	1.85E-02	promoter	super_enhancer	AC104024.1
chr17	16885000	chr17	42415000	1.82E-02	promoter	super_enhancer	AC104024.1
chr17	16885000	chr17	75095000	6.91E-03	promoter	super_enhancer	AC104024.1
chr17	16945000	chr17	17035000	2.17E-02	promoter	super_enhancer	MPRIP
chr17	16945000	chr17	27545000	1.52E-02	promoter	super_enhancer	MPRIP
chr17	16945000	chr17	29165000	1.46E-02	promoter	super_enhancer	MPRIP
chr17	16945000	chr17	57915000	5.84E-03	promoter	super_enhancer	MPRIP
chr17	17015000	chr17	27175000	3.02E-02	super_enhancer	promoter	RP11-20B24.3
chr17	17015000	chr17	28615000	2.40E-02	super_enhancer	promoter	RNU6-1267P
chr17	17015000	chr17	28905000	1.98E-02	super_enhancer	promoter	AC006050.2
chr17	17015000	chr17	36905000	2.12E-02	super_enhancer	promoter	CTB-58E17.5
chr17	17015000	chr17	74305000	6.20E-03	super_enhancer	promoter	QRICH2
chr17	17015000	chr17	78995000	5.82E-03	super_enhancer	promoter	CTD-2561B21.11
chr17	17025000	chr17	21365000	4.61E-02	super_enhancer	promoter	RP11-728E14.3
chr17	17025000	chr17	28445000	1.86E-02	super_enhancer	promoter	RP11-1148O4.2
chr17	17025000	chr17	37455000	1.26E-02	super_enhancer	promoter	AC005288.1
chr17	17025000	chr17	79935000	4.88E-03	super_enhancer	promoter	ASPSCR1
chr17	17035000	chr17	26795000	3.04E-02	super_enhancer	promoter	RPS7P1
chr17	17035000	chr17	26925000	3.39E-02	super_enhancer	promoter	SPAG5-AS1
chr17	17035000	chr17	27075000	3.21E-02	super_enhancer	promoter	AC010761.9

chr17	17035000	chr17	28615000	2.44E-02	super_enhancer	promoter	RNU6-1267P
chr17	17035000	chr17	37775000	2.22E-02	super_enhancer	promoter	AC087491.2
chr17	17035000	chr17	79375000	5.28E-03	super_enhancer	promoter	MIR4740
chr17	17045000	chr17	17495000	4.32E-02	super_enhancer	promoter	PEMT
chr17	17045000	chr17	26645000	2.64E-02	super_enhancer	promoter	KRT18P55
chr17	17045000	chr17	32805000	1.73E-02	super_enhancer	promoter	AC011193.1
chr17	17045000	chr17	37025000	1.97E-02	super_enhancer	promoter	LASP1
chr17	17045000	chr17	38805000	1.75E-02	super_enhancer	promoter	SMARCE1
chr17	17045000	chr17	56495000	9.18E-03	super_enhancer	promoter	RNF43
chr17	17045000	chr17	76395000	6.34E-03	super_enhancer	promoter	SNORA30
chr17	17055000	chr17	27045000	2.89E-02	super_enhancer	promoter	RAB34
chr17	17055000	chr17	29625000	1.50E-02	super_enhancer	promoter	OMG
chr17	17055000	chr17	35975000	1.57E-02	super_enhancer	promoter	MIR378J
chr17	17055000	chr17	38805000	1.55E-02	super_enhancer	promoter	SMARCE1
chr17	17055000	chr17	74095000	5.38E-03	super_enhancer	promoter	MIR6868
chr17	17055000	chr17	74845000	6.19E-03	super_enhancer	promoter	LINC00868
chr17	17055000	chr17	77825000	5.66E-03	super_enhancer	promoter	RP11-353N14.3
chr17	17065000	chr17	20495000	4.93E-02	super_enhancer	promoter	ZSWIM5P2
chr17	17065000	chr17	30555000	1.84E-02	super_enhancer	promoter	UBL5P2
chr17	17065000	chr17	46145000	1.10E-02	super_enhancer	promoter	RP5-890E16.5
chr17	17065000	chr17	49265000	8.86E-03	super_enhancer	promoter	RP11-481C4.2
chr17	17075000	chr17	29295000	2.82E-02	super_enhancer	promoter	RNF135
chr17	17075000	chr17	33895000	2.12E-02	super_enhancer	promoter	RP11-1094M14.11
chr17	17075000	chr17	40265000	2.19E-02	super_enhancer	promoter	DHX58

chr17	17075000	chr17	40925000	1.90E-02	super_enhancer	promoter	VPS25
chr17	17075000	chr17	41925000	1.59E-02	super_enhancer	promoter	CD300LG
chr17	17075000	chr17	48585000	1.82E-02	super_enhancer	promoter	MYCBPAP
chr17	17075000	chr17	80415000	6.04E-03	super_enhancer	promoter	NARF
chr17	17085000	chr17	28105000	2.28E-02	super_enhancer	promoter	RP11-82O19.2
chr17	17085000	chr17	35295000	1.97E-02	super_enhancer	promoter	RP11-445F12.1
chr17	17085000	chr17	40975000	1.19E-02	super_enhancer	promoter	PSME3
chr17	17095000	chr17	48785000	1.28E-02	super_enhancer	promoter	ANKRD40
chr17	17105000	chr17	27225000	3.53E-02	super_enhancer	promoter	FLOT2
chr17	17105000	chr17	35295000	2.77E-02	super_enhancer	promoter	RP11-445F12.1
chr17	17105000	chr17	35355000	1.68E-02	super_enhancer	promoter	RP11-333J10.3
chr17	17105000	chr17	40665000	1.75E-02	super_enhancer	promoter	MIR5010
chr17	17105000	chr17	41465000	1.81E-02	super_enhancer	promoter	RNU2-4P
chr17	17105000	chr17	47075000	1.24E-02	super_enhancer	promoter	RP11-501C14.5
chr17	17115000	chr17	20395000	4.46E-02	super_enhancer	promoter	YWHAEP3
chr17	17115000	chr17	20885000	4.87E-02	super_enhancer	promoter	RP11-746M1.2
chr17	17115000	chr17	28255000	2.37E-02	super_enhancer	promoter	EFCAB5
chr17	17115000	chr17	33345000	2.09E-02	super_enhancer	promoter	RP5-837J1.6
chr17	17115000	chr17	39345000	1.61E-02	super_enhancer	promoter	KRTAP9-2
chr17	17115000	chr17	47575000	1.66E-02	super_enhancer	promoter	RP11-81K2.2
chr17	17115000	chr17	48555000	1.36E-02	super_enhancer	promoter	RSAD1
chr17	17115000	chr17	79995000	6.49E-03	super_enhancer	promoter	RP11-1182P23.5
chr17	17125000	chr17	17185000	1.23E-02	super_enhancer	promoter	COPS3
chr17	17125000	chr17	17885000	6.03E-03	super_enhancer	promoter	AC087163.3

chr17	17125000	chr17	21935000	4.51E-02	super_enhancer	promoter	RP11-744K17.8
chr17	17125000	chr17	30335000	3.27E-02	super_enhancer	promoter	RP11-640N20.1
chr17	17125000	chr17	34145000	1.78E-02	super_enhancer	promoter	AC015849.13
chr17	17125000	chr17	35975000	2.32E-02	super_enhancer	promoter	MIR378J
chr17	17125000	chr17	38215000	2.44E-02	super_enhancer	promoter	THRA
chr17	17125000	chr17	74025000	6.14E-03	super_enhancer	promoter	EVPL
chr17	17185000	chr17	38915000	1.90E-02	promoter	super_enhancer	COPS3
chr17	17185000	chr17	41795000	2.01E-02	promoter	super_enhancer	COPS3
chr17	17185000	chr17	43365000	1.97E-02	promoter	super_enhancer	COPS3
chr17	17185000	chr17	78885000	6.32E-03	promoter	super_enhancer	COPS3
chr17	17325000	chr17	45275000	1.64E-02	promoter	super_enhancer	AC073621.2
chr17	17325000	chr17	77285000	6.19E-03	promoter	super_enhancer	AC073621.2
chr17	17495000	chr17	17765000	1.49E-02	promoter	super_enhancer	PEMT
chr17	17495000	chr17	27345000	3.22E-02	promoter	super_enhancer	PEMT
chr17	17495000	chr17	27575000	2.81E-02	promoter	super_enhancer	PEMT
chr17	17495000	chr17	29145000	2.74E-02	promoter	super_enhancer	PEMT
chr17	17495000	chr17	29155000	3.04E-02	promoter	super_enhancer	PEMT
chr17	17495000	chr17	41785000	1.93E-02	promoter	super_enhancer	PEMT
chr17	17495000	chr17	75085000	6.76E-03	promoter	super_enhancer	PEMT
chr17	17585000	chr17	17705000	1.44E-04	promoter	super_enhancer	RAI1
chr17	17585000	chr17	17725000	3.38E-02	promoter	super_enhancer	RAI1
chr17	17585000	chr17	17745000	7.96E-03	promoter	super_enhancer	RAI1
chr17	17585000	chr17	17825000	3.20E-05	promoter	super_enhancer	RAI1
chr17	17585000	chr17	27575000	3.25E-02	promoter	super_enhancer	RAI1

chr17	17585000	chr17	36915000	2.22E-02	promoter	super_enhancer	RAI1
chr17	17585000	chr17	40515000	1.90E-02	promoter	super_enhancer	RAI1
chr17	17585000	chr17	42395000	2.22E-02	promoter	super_enhancer	RAI1
chr17	17585000	chr17	50665000	6.88E-03	promoter	super_enhancer	RAI1
chr17	17685000	chr17	26905000	2.87E-02	super_enhancer	promoter	ALDOC
chr17	17685000	chr17	39845000	1.64E-02	super_enhancer	promoter	EIF1
chr17	17695000	chr17	26685000	3.33E-02	super_enhancer	promoter	POLDIP2
chr17	17695000	chr17	26695000	3.36E-02	super_enhancer	promoter	CTB-96E2.2
chr17	17695000	chr17	26795000	2.84E-02	super_enhancer	promoter	RPS7P1
chr17	17695000	chr17	33515000	1.53E-02	super_enhancer	promoter	RP11-799D4.2
chr17	17695000	chr17	40685000	1.41E-02	super_enhancer	promoter	PTP4A2P1
chr17	17695000	chr17	45055000	1.31E-02	super_enhancer	promoter	LRRC37A17P
chr17	17695000	chr17	47865000	1.19E-02	super_enhancer	promoter	KAT7
chr17	17695000	chr17	51185000	7.23E-03	super_enhancer	promoter	AC091154.1
chr17	17695000	chr17	56065000	7.57E-03	super_enhancer	promoter	RP11-159D12.3
chr17	17705000	chr17	17885000	2.59E-02	super_enhancer	promoter	AC087163.3
chr17	17705000	chr17	25375000	2.12E-02	super_enhancer	promoter	GTF2IP6
chr17	17705000	chr17	25745000	3.24E-02	super_enhancer	promoter	TBC1D3P5
chr17	17705000	chr17	46985000	1.30E-02	super_enhancer	promoter	UBE2Z
chr17	17705000	chr17	80935000	2.84E-03	super_enhancer	promoter	RP11-1197K16.2
chr17	17715000	chr17	20455000	4.36E-02	super_enhancer	promoter	TBC1D3P3
chr17	17715000	chr17	26695000	3.64E-02	super_enhancer	promoter	CTB-96E2.2
chr17	17715000	chr17	27045000	3.33E-02	super_enhancer	promoter	RAB34
chr17	17715000	chr17	27225000	3.17E-02	super_enhancer	promoter	FLOT2

chr17	17715000	chr17	29235000	2.75E-02	super_enhancer	promoter	TEFM
chr17	17715000	chr17	30485000	2.44E-02	super_enhancer	promoter	CTD-2095E4.3
chr17	17715000	chr17	36245000	1.94E-02	super_enhancer	promoter	YWHAEP7
chr17	17715000	chr17	79905000	6.26E-03	super_enhancer	promoter	MYADML2
chr17	17725000	chr17	27175000	2.87E-02	super_enhancer	promoter	RP11-20B24.3
chr17	17725000	chr17	33825000	1.74E-02	super_enhancer	promoter	RP11-1094M14.8
chr17	17725000	chr17	38305000	1.76E-02	super_enhancer	promoter	AC068669.1
chr17	17725000	chr17	78235000	4.64E-03	super_enhancer	promoter	RNF213
chr17	17735000	chr17	17875000	2.59E-02	super_enhancer	promoter	LRRC48
chr17	17735000	chr17	26695000	3.72E-02	super_enhancer	promoter	CTB-96E2.2
chr17	17735000	chr17	27175000	3.17E-02	super_enhancer	promoter	RP11-20B24.3
chr17	17735000	chr17	27895000	2.72E-02	super_enhancer	promoter	ABHD15
chr17	17735000	chr17	28445000	2.39E-02	super_enhancer	promoter	RP11-1148O4.2
chr17	17735000	chr17	40195000	1.77E-02	super_enhancer	promoter	CTD-2132N18.2
chr17	17735000	chr17	40835000	2.12E-02	super_enhancer	promoter	CNTNAP1
chr17	17735000	chr17	40985000	1.76E-02	super_enhancer	promoter	BECN1
chr17	17735000	chr17	62745000	7.82E-03	super_enhancer	promoter	MINOS1P2
chr17	17735000	chr17	74965000	6.91E-03	super_enhancer	promoter	RP11-87G24.3
chr17	17745000	chr17	29295000	2.32E-02	super_enhancer	promoter	RNF135
chr17	17745000	chr17	34345000	1.56E-02	super_enhancer	promoter	CCL23
chr17	17745000	chr17	40975000	1.38E-02	super_enhancer	promoter	PSME3
chr17	17745000	chr17	79905000	5.82E-03	super_enhancer	promoter	MYADML2
chr17	17755000	chr17	61525000	7.17E-03	super_enhancer	promoter	CYB561
chr17	17755000	chr17	74565000	4.97E-03	super_enhancer	promoter	RP11-666A8.9

chr17	17755000	chr17	79935000	4.75E-03	super_enhancer	promoter	ASPSCR1
chr17	17765000	chr17	27045000	6.04E-04	super_enhancer	promoter	RAB34
chr17	17765000	chr17	34145000	1.42E-02	super_enhancer	promoter	AC015849.13
chr17	17765000	chr17	35295000	2.55E-02	super_enhancer	promoter	RP11-445F12.1
chr17	17765000	chr17	39625000	1.80E-02	super_enhancer	promoter	KRT32
chr17	17765000	chr17	42285000	1.41E-02	super_enhancer	promoter	MIR6782
chr17	17765000	chr17	48015000	1.24E-02	super_enhancer	promoter	RP11-304F15.5
chr17	17825000	chr17	17875000	3.31E-02	super_enhancer	promoter	LRRC48
chr17	17825000	chr17	26695000	3.63E-02	super_enhancer	promoter	CTB-96E2.2
chr17	17825000	chr17	27045000	3.32E-02	super_enhancer	promoter	RAB34
chr17	17825000	chr17	28705000	2.67E-02	super_enhancer	promoter	CPD
chr17	17825000	chr17	30955000	1.73E-02	super_enhancer	promoter	RP11-220C2.1
chr17	17825000	chr17	77925000	6.27E-03	super_enhancer	promoter	RP11-353N14.7
chr17	17835000	chr17	25855000	2.14E-02	super_enhancer	promoter	RP11-720N19.1
chr17	17835000	chr17	33495000	1.84E-02	super_enhancer	promoter	RP11-799D4.3
chr17	17835000	chr17	55525000	7.59E-03	super_enhancer	promoter	RP11-226M10.3
chr17	17835000	chr17	67945000	3.75E-03	super_enhancer	promoter	AC002539.2
chr17	17845000	chr17	33925000	1.63E-02	super_enhancer	promoter	CTC-507E2.2
chr17	17845000	chr17	46045000	1.28E-02	super_enhancer	promoter	CDK5RAP3
chr17	17855000	chr17	27045000	3.22E-02	super_enhancer	promoter	RAB34
chr17	17855000	chr17	27055000	2.73E-02	super_enhancer	promoter	TLCD1
chr17	17855000	chr17	29055000	2.13E-02	super_enhancer	promoter	CTD-2349P21.6
chr17	17855000	chr17	42995000	1.62E-02	super_enhancer	promoter	GFAP
chr17	17855000	chr17	79305000	6.97E-03	super_enhancer	promoter	TMEM105

chr17	17875000	chr17	27535000	2.84E-02	promoter	super_enhancer	LRRC48
chr17	17875000	chr17	27565000	2.74E-02	promoter	super_enhancer	LRRC48
chr17	17875000	chr17	73355000	5.25E-03	promoter	super_enhancer	LRRC48
chr17	17885000	chr17	29105000	2.56E-02	promoter	super_enhancer	AC087163.3
chr17	17885000	chr17	80835000	6.40E-03	promoter	super_enhancer	AC087163.3
chr17	18015000	chr17	27525000	3.28E-02	promoter	super_enhancer	RP11-258F1.2
chr17	18015000	chr17	27545000	2.91E-02	promoter	super_enhancer	RP11-258F1.2
chr17	18015000	chr17	36935000	2.36E-02	promoter	super_enhancer	RP11-258F1.2
chr17	18015000	chr17	38895000	2.11E-02	promoter	super_enhancer	RP11-258F1.2
chr17	18015000	chr17	40315000	2.25E-02	promoter	super_enhancer	RP11-258F1.2
chr17	18015000	chr17	43455000	1.86E-02	promoter	super_enhancer	RP11-258F1.2
chr17	18015000	chr17	72415000	1.02E-02	promoter	super_enhancer	RP11-258F1.2
chr17	18085000	chr17	27565000	4.08E-02	promoter	super_enhancer	ALKBH5
chr17	18085000	chr17	29135000	3.55E-02	promoter	super_enhancer	ALKBH5
chr17	18085000	chr17	38425000	2.16E-02	promoter	super_enhancer	ALKBH5
chr17	18085000	chr17	40485000	2.10E-02	promoter	super_enhancer	ALKBH5
chr17	18215000	chr17	29105000	2.42E-02	promoter	super_enhancer	RPL7AP65
chr17	18215000	chr17	36925000	2.17E-02	promoter	super_enhancer	RPL7AP65
chr17	18215000	chr17	44905000	1.31E-02	promoter	super_enhancer	RPL7AP65
chr17	18215000	chr17	50665000	1.97E-05	promoter	super_enhancer	RPL7AP65
chr17	18215000	chr17	75725000	6.77E-03	promoter	super_enhancer	RPL7AP65
chr17	18245000	chr17	50915000	6.14E-03	promoter	super_enhancer	MIR6778
chr17	18355000	chr17	38465000	1.07E-02	promoter	super_enhancer	AL353997.11
chr17	18355000	chr17	75095000	4.00E-03	promoter	super_enhancer	AL353997.11

chr17	18355000	chr17	80895000	3.42E-03	promoter	super_enhancer	AL353997.11
chr17	18765000	chr17	27525000	2.30E-02	promoter	super_enhancer	CTD-2145A24.4
chr17	18765000	chr17	29175000	2.31E-02	promoter	super_enhancer	CTD-2145A24.4
chr17	18765000	chr17	36875000	1.99E-02	promoter	super_enhancer	CTD-2145A24.4
chr17	18765000	chr17	42005000	1.28E-02	promoter	super_enhancer	CTD-2145A24.4
chr17	18765000	chr17	64685000	6.82E-03	promoter	super_enhancer	CTD-2145A24.4
chr17	19505000	chr17	27345000	3.43E-02	promoter	super_enhancer	RP11-1113L8.7
chr17	19505000	chr17	72415000	7.27E-03	promoter	super_enhancer	RP11-1113L8.7
chr17	19505000	chr17	78575000	5.70E-03	promoter	super_enhancer	RP11-1113L8.7
chr17	20225000	chr17	27355000	3.21E-02	promoter	super_enhancer	RNU6-467P
chr17	20225000	chr17	48045000	1.44E-02	promoter	super_enhancer	RNU6-467P
chr17	20225000	chr17	73315000	5.10E-03	promoter	super_enhancer	RNU6-467P
chr17	20395000	chr17	48055000	6.62E-03	promoter	super_enhancer	YWHAEP3
chr17	20455000	chr17	27565000	1.31E-02	promoter	super_enhancer	TBC1D3P3
chr17	20455000	chr17	29175000	1.07E-02	promoter	super_enhancer	TBC1D3P3
chr17	20455000	chr17	50045000	3.26E-03	promoter	super_enhancer	TBC1D3P3
chr17	20495000	chr17	68335000	4.11E-03	promoter	super_enhancer	ZSWIM5P2
chr17	20515000	chr17	27345000	3.37E-02	promoter	super_enhancer	AC087499.5
chr17	20515000	chr17	29155000	2.86E-02	promoter	super_enhancer	AC087499.5
chr17	20515000	chr17	42405000	1.41E-02	promoter	super_enhancer	AC087499.5
chr17	20515000	chr17	78585000	4.44E-03	promoter	super_enhancer	AC087499.5
chr17	20515000	chr17	80475000	5.74E-03	promoter	super_enhancer	AC087499.5
chr17	20615000	chr17	57865000	5.88E-03	promoter	super_enhancer	AC087499.8
chr17	20615000	chr17	59835000	7.02E-03	promoter	super_enhancer	AC087499.8

chr17	20675000	chr17	27535000	3.45E-02	promoter	super_enhancer	HNRNPA1P19
chr17	20675000	chr17	45325000	1.08E-02	promoter	super_enhancer	HNRNPA1P19
chr17	21165000	chr17	57925000	9.24E-03	promoter	super_enhancer	EIF1P5
chr17	21165000	chr17	79805000	6.44E-03	promoter	super_enhancer	EIF1P5
chr17	21285000	chr17	25855000	4.67E-02	super_enhancer	promoter	RP11-720N19.1
chr17	21285000	chr17	27715000	3.51E-02	super_enhancer	promoter	RNU4-34P
chr17	21285000	chr17	31205000	2.77E-02	super_enhancer	promoter	MYO1D
chr17	21285000	chr17	33345000	2.25E-02	super_enhancer	promoter	RP5-837J1.6
chr17	21285000	chr17	33805000	2.04E-02	super_enhancer	promoter	RP11-686D22.9
chr17	21285000	chr17	36575000	2.08E-02	super_enhancer	promoter	ARHGAP23
chr17	21285000	chr17	60595000	8.01E-03	super_enhancer	promoter	RP11-464D20.2
chr17	21285000	chr17	79935000	6.06E-03	super_enhancer	promoter	ASPSCR1
chr17	21295000	chr17	26605000	4.41E-02	super_enhancer	promoter	AC061975.1
chr17	21295000	chr17	30735000	2.94E-02	super_enhancer	promoter	RP11-227G15.10
chr17	21365000	chr17	27575000	3.72E-02	promoter	super_enhancer	RP11-728E14.3
chr17	21435000	chr17	27565000	3.83E-02	promoter	super_enhancer	RP11-822E23.2
chr17	21435000	chr17	81045000	4.84E-03	promoter	super_enhancer	RP11-822E23.2
chr17	21455000	chr17	42335000	1.60E-02	promoter	super_enhancer	RP11-822E23.3
chr17	21455000	chr17	48065000	9.99E-03	promoter	super_enhancer	RP11-822E23.3
chr17	21455000	chr17	50925000	6.36E-03	promoter	super_enhancer	RP11-822E23.3
chr17	21495000	chr17	78595000	4.20E-03	promoter	super_enhancer	RP11-822E23.5
chr17	21825000	chr17	57955000	5.72E-03	promoter	super_enhancer	FAM27L
chr17	21935000	chr17	73325000	4.55E-03	promoter	super_enhancer	RP11-744K17.8
chr17	22195000	chr17	49525000	5.21E-03	promoter	super_enhancer	RP11-285M22.2

chr17	22195000	chr17	50675000	4.70E-03	promoter	super_enhancer	RP11-285M22.2
chr17	25375000	chr17	36875000	1.76E-02	promoter	super_enhancer	GTF2IP6
chr17	25745000	chr17	40105000	2.08E-02	promoter	super_enhancer	TBC1D3P5
chr17	25745000	chr17	49515000	1.36E-02	promoter	super_enhancer	TBC1D3P5
chr17	26005000	chr17	57895000	9.92E-03	promoter	super_enhancer	RP11-19P22.6
chr17	26035000	chr17	42015000	1.90E-02	promoter	super_enhancer	RP1-66C13.2
chr17	26035000	chr17	57875000	6.87E-03	promoter	super_enhancer	RP1-66C13.2
chr17	26035000	chr17	73295000	6.41E-03	promoter	super_enhancer	RP1-66C13.2
chr17	26545000	chr17	40115000	3.15E-02	promoter	super_enhancer	CTD-2008P7.10
chr17	26575000	chr17	36925000	4.12E-02	promoter	super_enhancer	CTD-2008P7.8
chr17	26575000	chr17	41785000	3.70E-02	promoter	super_enhancer	CTD-2008P7.8
chr17	26575000	chr17	45385000	2.64E-02	promoter	super_enhancer	CTD-2008P7.8
chr17	26575000	chr17	62775000	1.44E-02	promoter	super_enhancer	CTD-2008P7.8
chr17	26575000	chr17	75695000	1.31E-02	promoter	super_enhancer	CTD-2008P7.8
chr17	26585000	chr17	42325000	3.24E-02	promoter	super_enhancer	CTD-2008P7.7
chr17	26585000	chr17	57875000	1.05E-02	promoter	super_enhancer	CTD-2008P7.7
chr17	26585000	chr17	80815000	7.74E-03	promoter	super_enhancer	CTD-2008P7.7
chr17	26605000	chr17	50675000	1.32E-02	promoter	super_enhancer	AC061975.1
chr17	26605000	chr17	68395000	5.90E-03	promoter	super_enhancer	AC061975.1
chr17	26605000	chr17	79795000	8.50E-03	promoter	super_enhancer	AC061975.1
chr17	26645000	chr17	36935000	2.31E-02	promoter	super_enhancer	KRT18P55
chr17	26645000	chr17	41795000	2.29E-02	promoter	super_enhancer	KRT18P55
chr17	26645000	chr17	48075000	1.53E-02	promoter	super_enhancer	KRT18P55
chr17	26685000	chr17	40315000	2.86E-02	promoter	super_enhancer	POLDIP2

chr17	26685000	chr17	43445000	2.22E-02	promoter	super_enhancer	POLDIP2
chr17	26695000	chr17	38475000	2.99E-02	promoter	super_enhancer	CTB-96E2.2
chr17	26695000	chr17	45315000	2.23E-02	promoter	super_enhancer	CTB-96E2.2
chr17	26695000	chr17	45365000	2.32E-02	promoter	super_enhancer	CTB-96E2.2
chr17	26695000	chr17	64665000	9.93E-03	promoter	super_enhancer	CTB-96E2.2
chr17	26695000	chr17	73315000	9.87E-03	promoter	super_enhancer	CTB-96E2.2
chr17	26735000	chr17	38915000	2.39E-02	promoter	super_enhancer	SLC46A1
chr17	26735000	chr17	40115000	2.66E-02	promoter	super_enhancer	SLC46A1
chr17	26735000	chr17	50065000	1.09E-02	promoter	super_enhancer	SLC46A1
chr17	26795000	chr17	73305000	8.17E-03	promoter	super_enhancer	RPS7P1
chr17	26905000	chr17	38415000	2.26E-02	promoter	super_enhancer	ALDOC
chr17	26905000	chr17	40485000	1.95E-02	promoter	super_enhancer	ALDOC
chr17	26905000	chr17	64675000	9.53E-03	promoter	super_enhancer	ALDOC
chr17	26905000	chr17	75725000	7.52E-03	promoter	super_enhancer	ALDOC
chr17	26905000	chr17	81065000	5.76E-03	promoter	super_enhancer	ALDOC
chr17	26925000	chr17	40115000	3.23E-02	promoter	super_enhancer	SPAG5-AS1
chr17	26925000	chr17	56915000	1.36E-02	promoter	super_enhancer	SPAG5-AS1
chr17	26925000	chr17	80265000	9.85E-03	promoter	super_enhancer	SPAG5-AS1
chr17	26935000	chr17	38415000	2.28E-02	promoter	super_enhancer	RP11-192H23.6
chr17	26935000	chr17	64655000	8.86E-03	promoter	super_enhancer	RP11-192H23.6
chr17	26935000	chr17	64685000	9.00E-03	promoter	super_enhancer	RP11-192H23.6
chr17	27045000	chr17	36855000	3.54E-02	promoter	super_enhancer	RAB34
chr17	27045000	chr17	43395000	2.10E-02	promoter	super_enhancer	RAB34
chr17	27045000	chr17	45275000	2.22E-02	promoter	super_enhancer	RAB34

chr17	27045000	chr17	78595000	8.13E-03	promoter	super_enhancer	RAB34
chr17	27055000	chr17	42415000	2.24E-02	promoter	super_enhancer	TLCD1
chr17	27075000	chr17	79805000	8.09E-03	promoter	super_enhancer	AC010761.9
chr17	27175000	chr17	38875000	2.18E-02	promoter	super_enhancer	RP11-20B24.3
chr17	27175000	chr17	40075000	2.60E-02	promoter	super_enhancer	RP11-20B24.3
chr17	27175000	chr17	48045000	2.67E-02	promoter	super_enhancer	RP11-20B24.3
chr17	27175000	chr17	57885000	7.31E-03	promoter	super_enhancer	RP11-20B24.3
chr17	27175000	chr17	78355000	8.61E-03	promoter	super_enhancer	RP11-20B24.3
chr17	27225000	chr17	36825000	3.32E-02	promoter	super_enhancer	FLOT2
chr17	27225000	chr17	36885000	2.96E-02	promoter	super_enhancer	FLOT2
chr17	27225000	chr17	43365000	2.74E-02	promoter	super_enhancer	FLOT2
chr17	27325000	chr17	32335000	3.22E-02	super_enhancer	promoter	RP11-1086I4.2
chr17	27325000	chr17	33885000	3.41E-02	super_enhancer	promoter	SLFN14
chr17	27325000	chr17	34145000	2.46E-02	super_enhancer	promoter	AC015849.13
chr17	27325000	chr17	36105000	2.75E-02	super_enhancer	promoter	HNF1B
chr17	27325000	chr17	41475000	2.22E-02	super_enhancer	promoter	ARL4D
chr17	27335000	chr17	77825000	8.55E-03	super_enhancer	promoter	RP11-353N14.3
chr17	27345000	chr17	42995000	2.72E-02	super_enhancer	promoter	GFAP
chr17	27345000	chr17	57185000	1.39E-02	super_enhancer	promoter	TRIM37
chr17	27355000	chr17	41925000	2.50E-02	super_enhancer	promoter	CD300LG
chr17	27355000	chr17	56555000	1.31E-02	super_enhancer	promoter	SETP3
chr17	27525000	chr17	29055000	1.77E-02	super_enhancer	promoter	CTD-2349P21.6
chr17	27525000	chr17	35295000	3.43E-02	super_enhancer	promoter	RP11-445F12.1
chr17	27525000	chr17	38305000	2.43E-02	super_enhancer	promoter	AC068669.1

chr17	27525000	chr17	40865000	1.91E-02	super_enhancer	promoter	CTD-3193K9.1
chr17	27535000	chr17	39405000	1.69E-02	super_enhancer	promoter	KRTAP9-4
chr17	27535000	chr17	40385000	2.32E-02	super_enhancer	promoter	RP11-358B23.6
chr17	27545000	chr17	33425000	3.22E-02	super_enhancer	promoter	Vault
chr17	27545000	chr17	41465000	1.77E-02	super_enhancer	promoter	RNU2-4P
chr17	27545000	chr17	46895000	1.41E-02	super_enhancer	promoter	TTLL6
chr17	27545000	chr17	55175000	1.09E-02	super_enhancer	promoter	RP11-166P13.4
chr17	27545000	chr17	72595000	5.90E-03	super_enhancer	promoter	CTD-2006K23.1
chr17	27555000	chr17	28255000	3.63E-02	super_enhancer	promoter	EFCAB5
chr17	27555000	chr17	34245000	3.58E-02	super_enhancer	promoter	LRRC37A9P
chr17	27555000	chr17	40265000	3.05E-02	super_enhancer	promoter	DHX58
chr17	27555000	chr17	46705000	1.96E-02	super_enhancer	promoter	HOXB9
chr17	27565000	chr17	39685000	2.99E-02	super_enhancer	promoter	KRT19
chr17	27565000	chr17	42635000	2.38E-02	super_enhancer	promoter	FZD2
chr17	27575000	chr17	29035000	4.61E-03	super_enhancer	promoter	SUZ12P1
chr17	27575000	chr17	32015000	4.44E-02	super_enhancer	promoter	RP11-118G23.1
chr17	27715000	chr17	36925000	3.05E-02	promoter	super_enhancer	RNU4-34P
chr17	27715000	chr17	73285000	7.79E-03	promoter	super_enhancer	RNU4-34P
chr17	27895000	chr17	42345000	2.35E-02	promoter	super_enhancer	ABHD15
chr17	27895000	chr17	72415000	9.10E-03	promoter	super_enhancer	ABHD15
chr17	28045000	chr17	45315000	1.75E-02	promoter	super_enhancer	RPL21P123
chr17	28045000	chr17	59805000	8.55E-03	promoter	super_enhancer	RPL21P123
chr17	28045000	chr17	80895000	5.85E-03	promoter	super_enhancer	RPL21P123
chr17	28105000	chr17	38425000	2.35E-02	promoter	super_enhancer	RP11-82O19.2

chr17	28255000	chr17	78595000	6.24E-03	promoter	super_enhancer	EFCAB5
chr17	28395000	chr17	29105000	2.25E-02	promoter	super_enhancer	RP11-1148O4.1
chr17	28395000	chr17	36835000	2.99E-02	promoter	super_enhancer	RP11-1148O4.1
chr17	28395000	chr17	36925000	2.96E-02	promoter	super_enhancer	RP11-1148O4.1
chr17	28395000	chr17	75095000	6.81E-03	promoter	super_enhancer	RP11-1148O4.1
chr17	28395000	chr17	78585000	5.77E-03	promoter	super_enhancer	RP11-1148O4.1
chr17	28565000	chr17	29105000	1.76E-02	promoter	super_enhancer	RP11-354P11.2
chr17	28565000	chr17	36865000	3.42E-02	promoter	super_enhancer	RP11-354P11.2
chr17	28565000	chr17	36885000	3.25E-02	promoter	super_enhancer	RP11-354P11.2
chr17	28575000	chr17	41785000	3.00E-02	promoter	super_enhancer	SNORD63
chr17	28575000	chr17	81115000	5.47E-03	promoter	super_enhancer	SNORD63
chr17	28615000	chr17	42345000	2.32E-02	promoter	super_enhancer	RNU6-1267P
chr17	28615000	chr17	43465000	2.28E-02	promoter	super_enhancer	RNU6-1267P
chr17	28615000	chr17	78565000	5.86E-03	promoter	super_enhancer	RNU6-1267P
chr17	28705000	chr17	41785000	2.99E-02	promoter	super_enhancer	CPD
chr17	28705000	chr17	45315000	2.00E-02	promoter	super_enhancer	CPD
chr17	28705000	chr17	73365000	6.35E-03	promoter	super_enhancer	CPD
chr17	28745000	chr17	37125000	2.91E-02	super_enhancer	promoter	FBXO47
chr17	28745000	chr17	46895000	1.72E-02	super_enhancer	promoter	TTLL6
chr17	28745000	chr17	48585000	2.01E-02	super_enhancer	promoter	MYCBPAP
chr17	28745000	chr17	61045000	6.67E-03	super_enhancer	promoter	RP11-180P8.1
chr17	28745000	chr17	61525000	1.07E-02	super_enhancer	promoter	CYB561
chr17	28905000	chr17	38475000	2.10E-02	promoter	super_enhancer	AC006050.2
chr17	28905000	chr17	50675000	9.76E-03	promoter	super_enhancer	AC006050.2

chr17	28905000	chr17	77415000	4.23E-03	promoter	super_enhancer	AC006050.2
chr17	28905000	chr17	78585000	5.07E-03	promoter	super_enhancer	AC006050.2
chr17	29035000	chr17	40115000	5.56E-04	promoter	super_enhancer	SUZ12P1
chr17	29035000	chr17	40315000	2.86E-02	promoter	super_enhancer	SUZ12P1
chr17	29035000	chr17	77415000	6.02E-03	promoter	super_enhancer	SUZ12P1
chr17	29055000	chr17	36875000	3.14E-02	promoter	super_enhancer	CTD-2349P21.6
chr17	29055000	chr17	45375000	1.88E-02	promoter	super_enhancer	CTD-2349P21.6
chr17	29055000	chr17	72375000	8.00E-03	promoter	super_enhancer	CTD-2349P21.6
chr17	29055000	chr17	78345000	6.21E-03	promoter	super_enhancer	CTD-2349P21.6
chr17	29065000	chr17	36935000	2.19E-02	promoter	super_enhancer	CTD-2349P21.3
chr17	29065000	chr17	42015000	1.96E-02	promoter	super_enhancer	CTD-2349P21.3
chr17	29105000	chr17	35295000	4.34E-02	super_enhancer	promoter	RP11-445F12.1
chr17	29105000	chr17	37355000	2.87E-02	super_enhancer	promoter	CACNB1
chr17	29105000	chr17	48475000	1.60E-02	super_enhancer	promoter	LRRC59
chr17	29115000	chr17	38215000	3.07E-02	super_enhancer	promoter	THRA
chr17	29115000	chr17	42385000	2.76E-02	super_enhancer	promoter	RUNDC3A
chr17	29115000	chr17	48015000	1.75E-02	super_enhancer	promoter	RP11-304F15.5
chr17	29115000	chr17	48285000	2.22E-02	super_enhancer	promoter	RP11-893F2.5
chr17	29115000	chr17	56975000	1.20E-02	super_enhancer	promoter	RP11-579O24.1
chr17	29125000	chr17	34345000	4.48E-02	super_enhancer	promoter	CCL23
chr17	29125000	chr17	37325000	4.34E-02	super_enhancer	promoter	ARL5C
chr17	29125000	chr17	45195000	2.11E-02	super_enhancer	promoter	AC002558.1
chr17	29125000	chr17	47325000	2.30E-02	super_enhancer	promoter	FLJ40194
chr17	29125000	chr17	47925000	2.36E-02	super_enhancer	promoter	TAC4

chr17	29125000	chr17	67705000	7.44E-03	super_enhancer	promoter	RP1-200G19.1
chr17	29125000	chr17	74085000	1.08E-02	super_enhancer	promoter	RP11-449J21.3
chr17	29125000	chr17	74965000	1.01E-02	super_enhancer	promoter	RP11-87G24.3
chr17	29125000	chr17	76275000	7.84E-03	super_enhancer	promoter	RP11-219G17.4
chr17	29135000	chr17	40975000	2.14E-02	super_enhancer	promoter	PSME3
chr17	29135000	chr17	48925000	1.77E-02	super_enhancer	promoter	RPL5P33
chr17	29135000	chr17	62075000	9.45E-03	super_enhancer	promoter	PRR29
chr17	29135000	chr17	73495000	1.00E-02	super_enhancer	promoter	MIR6785
chr17	29145000	chr17	33905000	4.23E-02	super_enhancer	promoter	AP2B1
chr17	29145000	chr17	39205000	1.97E-02	super_enhancer	promoter	KRTAP2-1
chr17	29145000	chr17	46985000	1.92E-02	super_enhancer	promoter	UBE2Z
chr17	29145000	chr17	60705000	1.56E-02	super_enhancer	promoter	CTD-3035K23.7
chr17	29155000	chr17	36245000	4.19E-02	super_enhancer	promoter	YWHAEP7
chr17	29155000	chr17	38185000	4.21E-02	super_enhancer	promoter	SNORD124
chr17	29155000	chr17	39205000	2.19E-02	super_enhancer	promoter	KRTAP2-1
chr17	29155000	chr17	40835000	3.84E-02	super_enhancer	promoter	CNTNAP1
chr17	29155000	chr17	42265000	2.77E-02	super_enhancer	promoter	ASB16-AS1
chr17	29155000	chr17	60085000	1.36E-02	super_enhancer	promoter	RP11-342K2.1
chr17	29155000	chr17	61905000	1.56E-02	super_enhancer	promoter	PSMC5
chr17	29155000	chr17	76165000	8.58E-03	super_enhancer	promoter	SYNGR2
chr17	29165000	chr17	33515000	3.58E-02	super_enhancer	promoter	RP11-799D4.2
chr17	29165000	chr17	34085000	4.12E-02	super_enhancer	promoter	RP1-161P9.5
chr17	29165000	chr17	34315000	3.69E-02	super_enhancer	promoter	CCL14
chr17	29165000	chr17	36245000	2.83E-02	super_enhancer	promoter	YWHAEP7

chr17	29165000	chr17	36905000	2.99E-02	super_enhancer	promoter	CTB-58E17.5
chr17	29165000	chr17	56065000	8.94E-03	super_enhancer	promoter	RP11-159D12.3
chr17	29165000	chr17	56745000	1.03E-02	super_enhancer	promoter	RNU1-108P
chr17	29175000	chr17	40195000	2.61E-02	super_enhancer	promoter	CTD-2132N18.2
chr17	29175000	chr17	47785000	2.01E-02	super_enhancer	promoter	RP11-613C6.2
chr17	29175000	chr17	48555000	1.82E-02	super_enhancer	promoter	RSAD1
chr17	29175000	chr17	74085000	8.73E-03	super_enhancer	promoter	RP11-449J21.3
chr17	29225000	chr17	42005000	2.11E-02	promoter	super_enhancer	RP13-753N3.3
chr17	29225000	chr17	57935000	1.32E-02	promoter	super_enhancer	RP13-753N3.3
chr17	29225000	chr17	64725000	9.58E-03	promoter	super_enhancer	RP13-753N3.3
chr17	29225000	chr17	68335000	7.25E-03	promoter	super_enhancer	RP13-753N3.3
chr17	29235000	chr17	41795000	3.00E-02	promoter	super_enhancer	TEFM
chr17	29235000	chr17	57955000	1.26E-02	promoter	super_enhancer	TEFM
chr17	29285000	chr17	36875000	3.98E-02	promoter	super_enhancer	AC091177.1
chr17	29285000	chr17	38875000	2.36E-02	promoter	super_enhancer	AC091177.1
chr17	29285000	chr17	45225000	1.70E-02	promoter	super_enhancer	AC091177.1
chr17	29285000	chr17	50065000	1.29E-02	promoter	super_enhancer	AC091177.1
chr17	29285000	chr17	64715000	9.94E-03	promoter	super_enhancer	AC091177.1
chr17	29285000	chr17	73285000	8.86E-03	promoter	super_enhancer	AC091177.1
chr17	29295000	chr17	36865000	3.24E-02	promoter	super_enhancer	RNF135
chr17	29295000	chr17	42025000	2.37E-02	promoter	super_enhancer	RNF135
chr17	29295000	chr17	45295000	1.66E-02	promoter	super_enhancer	RNF135
chr17	29335000	chr17	62795000	4.08E-03	promoter	super_enhancer	RP11-848P1.7
chr17	29625000	chr17	40085000	1.83E-02	promoter	super_enhancer	OMG

chr17	29625000	chr17	57935000	9.34E-03	promoter	super_enhancer	OMG
chr17	29625000	chr17	78575000	5.17E-03	promoter	super_enhancer	OMG
chr17	29625000	chr17	80475000	5.86E-03	promoter	super_enhancer	OMG
chr17	29845000	chr17	42325000	3.30E-02	promoter	super_enhancer	RN7SL45P
chr17	29845000	chr17	56915000	1.36E-02	promoter	super_enhancer	RN7SL45P
chr17	29845000	chr17	68385000	6.79E-03	promoter	super_enhancer	RN7SL45P
chr17	29845000	chr17	80255000	8.30E-03	promoter	super_enhancer	RN7SL45P
chr17	30335000	chr17	38455000	2.95E-02	promoter	super_enhancer	RP11-640N20.1
chr17	30335000	chr17	42025000	2.65E-02	promoter	super_enhancer	RP11-640N20.1
chr17	30335000	chr17	48045000	2.90E-02	promoter	super_enhancer	RP11-640N20.1
chr17	30335000	chr17	72395000	9.02E-03	promoter	super_enhancer	RP11-640N20.1
chr17	30335000	chr17	77425000	7.21E-03	promoter	super_enhancer	RP11-640N20.1
chr17	30455000	chr17	36835000	4.53E-02	promoter	super_enhancer	CTD-2095E4.5
chr17	30455000	chr17	36855000	4.54E-02	promoter	super_enhancer	CTD-2095E4.5
chr17	30455000	chr17	36935000	3.78E-02	promoter	super_enhancer	CTD-2095E4.5
chr17	30455000	chr17	48045000	2.97E-02	promoter	super_enhancer	CTD-2095E4.5
chr17	30485000	chr17	40115000	3.28E-02	promoter	super_enhancer	CTD-2095E4.3
chr17	30485000	chr17	43395000	2.14E-02	promoter	super_enhancer	CTD-2095E4.3
chr17	30555000	chr17	59785000	8.16E-03	promoter	super_enhancer	UBL5P2
chr17	30555000	chr17	68335000	6.96E-03	promoter	super_enhancer	UBL5P2
chr17	30555000	chr17	73305000	7.74E-03	promoter	super_enhancer	UBL5P2
chr17	30555000	chr17	80765000	5.72E-03	promoter	super_enhancer	UBL5P2
chr17	30655000	chr17	40315000	3.76E-02	promoter	super_enhancer	RP11-227G15.2
chr17	30655000	chr17	42335000	4.21E-02	promoter	super_enhancer	RP11-227G15.2

chr17	30655000	chr17	42345000	3.65E-02	promoter	super_enhancer	RP11-227G15.2
chr17	30655000	chr17	50065000	1.72E-02	promoter	super_enhancer	RP11-227G15.2
chr17	30735000	chr17	45325000	2.11E-02	promoter	super_enhancer	RP11-227G15.10
chr17	30735000	chr17	50135000	1.56E-02	promoter	super_enhancer	RP11-227G15.10
chr17	30845000	chr17	73325000	6.05E-03	promoter	super_enhancer	RP11-466A19.3
chr17	30955000	chr17	49505000	1.10E-02	promoter	super_enhancer	RP11-220C2.1
chr17	31205000	chr17	38455000	3.36E-02	promoter	super_enhancer	MYO1D
chr17	31205000	chr17	42025000	2.86E-02	promoter	super_enhancer	MYO1D
chr17	31255000	chr17	40485000	2.12E-02	promoter	super_enhancer	TMEM98
chr17	31255000	chr17	45375000	2.07E-02	promoter	super_enhancer	TMEM98
chr17	31255000	chr17	78555000	6.42E-03	promoter	super_enhancer	TMEM98
chr17	32015000	chr17	42005000	1.95E-02	promoter	super_enhancer	RP11-118G23.1
chr17	32015000	chr17	43365000	2.42E-02	promoter	super_enhancer	RP11-118G23.1
chr17	32015000	chr17	49505000	1.23E-02	promoter	super_enhancer	RP11-118G23.1
chr17	32335000	chr17	38915000	2.40E-02	promoter	super_enhancer	RP11-1086I4.2
chr17	32645000	chr17	64665000	7.66E-03	promoter	super_enhancer	CCL8
chr17	32645000	chr17	72365000	8.08E-03	promoter	super_enhancer	CCL8
chr17	32805000	chr17	62795000	4.05E-03	promoter	super_enhancer	AC011193.1
chr17	32805000	chr17	64685000	8.87E-03	promoter	super_enhancer	AC011193.1
chr17	33345000	chr17	43415000	3.66E-02	promoter	super_enhancer	RP5-837J1.6
chr17	33345000	chr17	78535000	7.84E-03	promoter	super_enhancer	RP5-837J1.6
chr17	33495000	chr17	40515000	4.21E-02	promoter	super_enhancer	RP11-799D4.3
chr17	33495000	chr17	42025000	3.58E-02	promoter	super_enhancer	RP11-799D4.3
chr17	33495000	chr17	42425000	3.50E-02	promoter	super_enhancer	RP11-799D4.3

chr17	33495000	chr17	57915000	1.50E-02	promoter	super_enhancer	RP11-799D4.3
chr17	33495000	chr17	78535000	9.54E-03	promoter	super_enhancer	RP11-799D4.3
chr17	33495000	chr17	79855000	8.43E-03	promoter	super_enhancer	RP11-799D4.3
chr17	33515000	chr17	38445000	4.38E-02	promoter	super_enhancer	RP11-799D4.2
chr17	33515000	chr17	45365000	2.01E-02	promoter	super_enhancer	RP11-799D4.2
chr17	33515000	chr17	72405000	8.85E-03	promoter	super_enhancer	RP11-799D4.2
chr17	33515000	chr17	73315000	7.50E-03	promoter	super_enhancer	RP11-799D4.2
chr17	33735000	chr17	50045000	1.09E-02	promoter	super_enhancer	RP11-686D22.3
chr17	33865000	chr17	40095000	4.23E-02	promoter	super_enhancer	SLFN12L
chr17	33865000	chr17	41785000	3.92E-02	promoter	super_enhancer	SLFN12L
chr17	33865000	chr17	81055000	7.37E-03	promoter	super_enhancer	SLFN12L
chr17	33885000	chr17	49525000	1.56E-02	promoter	super_enhancer	SLFN14
chr17	33895000	chr17	45375000	2.50E-02	promoter	super_enhancer	RP11-1094M14.11
chr17	33895000	chr17	68335000	7.68E-03	promoter	super_enhancer	RP11-1094M14.11
chr17	33905000	chr17	45175000	1.91E-02	promoter	super_enhancer	AP2B1
chr17	33905000	chr17	50065000	1.15E-02	promoter	super_enhancer	AP2B1
chr17	33905000	chr17	50155000	1.17E-02	promoter	super_enhancer	AP2B1
chr17	33925000	chr17	48045000	3.04E-02	promoter	super_enhancer	CTC-507E2.2
chr17	33925000	chr17	50045000	1.29E-02	promoter	super_enhancer	CTC-507E2.2
chr17	33925000	chr17	79835000	6.98E-03	promoter	super_enhancer	CTC-507E2.2
chr17	34085000	chr17	80795000	8.26E-03	promoter	super_enhancer	RP1-161P9.5
chr17	34145000	chr17	78525000	4.64E-03	promoter	super_enhancer	AC015849.13
chr17	34195000	chr17	40095000	4.15E-02	promoter	super_enhancer	AC015849.2
chr17	34195000	chr17	42005000	2.67E-02	promoter	super_enhancer	AC015849.2

chr17	34195000	chr17	56935000	1.33E-02	promoter	super_enhancer	AC015849.2
chr17	34245000	chr17	42335000	3.62E-02	promoter	super_enhancer	LRRC37A9P
chr17	34245000	chr17	68325000	6.48E-03	promoter	super_enhancer	LRRC37A9P
chr17	34315000	chr17	42025000	3.26E-02	promoter	super_enhancer	CCL14
chr17	34315000	chr17	43365000	3.42E-02	promoter	super_enhancer	CCL14
chr17	34345000	chr17	38455000	4.70E-02	promoter	super_enhancer	CCL23
chr17	34345000	chr17	43455000	2.23E-02	promoter	super_enhancer	CCL23
chr17	34345000	chr17	64325000	9.73E-03	promoter	super_enhancer	CCL23
chr17	34345000	chr17	64345000	7.83E-03	promoter	super_enhancer	CCL23
chr17	34775000	chr17	48075000	1.13E-02	promoter	super_enhancer	RNU6-1192P
chr17	35085000	chr17	56755000	1.46E-02	super_enhancer	promoter	RNU1-52P
chr17	35085000	chr17	57785000	1.26E-02	super_enhancer	promoter	PTRH2
chr17	35095000	chr17	35295000	1.62E-03	super_enhancer	promoter	RP11-445F12.1
chr17	35095000	chr17	35305000	1.90E-02	super_enhancer	promoter	AATF
chr17	35095000	chr17	45195000	1.92E-02	super_enhancer	promoter	AC002558.1
chr17	35095000	chr17	49265000	1.55E-02	super_enhancer	promoter	RP11-481C4.2
chr17	35095000	chr17	55175000	1.51E-02	super_enhancer	promoter	RP11-166P13.4
chr17	35295000	chr17	44905000	3.16E-02	promoter	super_enhancer	RP11-445F12.1
chr17	35295000	chr17	49525000	2.34E-02	promoter	super_enhancer	RP11-445F12.1
chr17	35305000	chr17	42425000	3.32E-02	promoter	super_enhancer	AATF
chr17	35305000	chr17	43415000	4.01E-02	promoter	super_enhancer	AATF
chr17	36105000	chr17	64705000	8.36E-03	promoter	super_enhancer	HNF1B
chr17	36245000	chr17	42345000	4.60E-02	promoter	super_enhancer	YWHAEP7
chr17	36575000	chr17	36825000	1.40E-03	promoter	super_enhancer	ARHGAP23

chr17	36575000	chr17	79785000	1.02E-02	promoter	super_enhancer	ARHGAP23
chr17	36575000	chr17	80775000	1.01E-02	promoter	super_enhancer	ARHGAP23
chr17	36825000	chr17	48475000	2.81E-02	super_enhancer	promoter	LRRC59
chr17	36825000	chr17	58095000	9.63E-03	super_enhancer	promoter	TBC1D3P1
chr17	36825000	chr17	79995000	1.05E-02	super_enhancer	promoter	RP11-1182P23.5
chr17	36835000	chr17	37125000	1.91E-02	super_enhancer	promoter	FBXO47
chr17	36835000	chr17	37355000	4.01E-02	super_enhancer	promoter	CACNB1
chr17	36835000	chr17	80375000	1.08E-02	super_enhancer	promoter	HEXDC
chr17	36845000	chr17	43025000	3.81E-02	super_enhancer	promoter	KIF18B
chr17	36845000	chr17	60705000	1.95E-02	super_enhancer	promoter	CTD-3035K23.7
chr17	36855000	chr17	47325000	3.17E-02	super_enhancer	promoter	FLJ40194
chr17	36855000	chr17	49265000	2.38E-02	super_enhancer	promoter	RP11-481C4.2
chr17	36855000	chr17	75465000	8.31E-03	super_enhancer	promoter	RP11-75C10.7
chr17	36855000	chr17	76275000	9.33E-03	super_enhancer	promoter	RP11-219G17.4
chr17	36865000	chr17	47865000	2.74E-02	super_enhancer	promoter	KAT7
chr17	36865000	chr17	62505000	1.17E-02	super_enhancer	promoter	DDX5
chr17	36865000	chr17	79225000	9.31E-03	super_enhancer	promoter	RP11-455O6.8
chr17	36875000	chr17	46075000	3.61E-02	super_enhancer	promoter	RP11-6N17.10
chr17	36875000	chr17	47575000	3.91E-02	super_enhancer	promoter	RP11-81K2.2
chr17	36875000	chr17	79375000	9.34E-03	super_enhancer	promoter	MIR4740
chr17	36885000	chr17	37355000	7.73E-03	super_enhancer	promoter	CACNB1
chr17	36885000	chr17	47865000	2.61E-02	super_enhancer	promoter	KAT7
chr17	36885000	chr17	73495000	1.32E-02	super_enhancer	promoter	MIR6785
chr17	36905000	chr17	38435000	4.31E-02	promoter	super_enhancer	CTB-58E17.5

chr17	36905000	chr17	45305000	3.64E-02	promoter	super_enhancer	CTB-58E17.5
chr17	36905000	chr17	48045000	4.46E-02	promoter	super_enhancer	CTB-58E17.5
chr17	36915000	chr17	48015000	2.62E-02	super_enhancer	promoter	RP11-304F15.5
chr17	36915000	chr17	48845000	2.70E-02	super_enhancer	promoter	LINC00483
chr17	36915000	chr17	60535000	1.59E-02	super_enhancer	promoter	TLK2
chr17	36915000	chr17	74555000	9.70E-03	super_enhancer	promoter	RP11-666A8.8
chr17	36925000	chr17	37395000	1.77E-02	super_enhancer	promoter	RP11-690G19.4
chr17	36925000	chr17	43495000	4.11E-02	super_enhancer	promoter	AC091132.2
chr17	36925000	chr17	46235000	3.10E-02	super_enhancer	promoter	MIR1203
chr17	36925000	chr17	47965000	3.49E-02	super_enhancer	promoter	RP11-304F15.4
chr17	36925000	chr17	58755000	2.17E-02	super_enhancer	promoter	BCAS3
chr17	36925000	chr17	77825000	1.11E-02	super_enhancer	promoter	RP11-353N14.3
chr17	36925000	chr17	79935000	1.04E-02	super_enhancer	promoter	ASPSCR1
chr17	36935000	chr17	37125000	2.93E-02	super_enhancer	promoter	FBXO47
chr17	36935000	chr17	42275000	4.36E-02	super_enhancer	promoter	CTB-175E5.7
chr17	36935000	chr17	42755000	3.58E-02	super_enhancer	promoter	RP11-1072C15.4
chr17	36935000	chr17	60475000	1.00E-02	super_enhancer	promoter	RPS23P7
chr17	36935000	chr17	62075000	1.26E-02	super_enhancer	promoter	PRR29
chr17	36935000	chr17	74705000	8.29E-03	super_enhancer	promoter	RP11-318A15.8
chr17	37025000	chr17	38435000	4.71E-02	promoter	super_enhancer	LASP1
chr17	37025000	chr17	38475000	4.27E-02	promoter	super_enhancer	LASP1
chr17	37125000	chr17	59805000	1.39E-02	promoter	super_enhancer	FBXO47
chr17	37125000	chr17	78595000	9.98E-03	promoter	super_enhancer	FBXO47
chr17	37125000	chr17	81045000	7.88E-03	promoter	super_enhancer	FBXO47

chr17	37325000	chr17	43455000	4.64E-02	promoter	super_enhancer	ARL5C
chr17	37325000	chr17	50655000	1.92E-02	promoter	super_enhancer	ARL5C
chr17	37325000	chr17	57865000	1.48E-02	promoter	super_enhancer	ARL5C
chr17	37325000	chr17	77305000	9.09E-03	promoter	super_enhancer	ARL5C
chr17	37355000	chr17	45365000	3.49E-02	promoter	super_enhancer	CACNB1
chr17	37395000	chr17	76875000	1.31E-02	promoter	super_enhancer	RP11-690G19.4
chr17	37455000	chr17	48045000	3.43E-02	promoter	super_enhancer	AC005288.1
chr17	37455000	chr17	75705000	7.83E-03	promoter	super_enhancer	AC005288.1
chr17	37455000	chr17	78545000	8.00E-03	promoter	super_enhancer	AC005288.1
chr17	37455000	chr17	79835000	7.30E-03	promoter	super_enhancer	AC005288.1
chr17	37775000	chr17	50915000	1.78E-02	promoter	super_enhancer	AC087491.2
chr17	38185000	chr17	57945000	2.27E-02	promoter	super_enhancer	SNORD124
chr17	38215000	chr17	40075000	2.23E-02	promoter	super_enhancer	THRA
chr17	38215000	chr17	45365000	3.86E-02	promoter	super_enhancer	THRA
chr17	38215000	chr17	73305000	1.15E-02	promoter	super_enhancer	THRA
chr17	38305000	chr17	38455000	1.34E-03	promoter	super_enhancer	AC068669.1
chr17	38305000	chr17	81085000	7.20E-03	promoter	super_enhancer	AC068669.1
chr17	38375000	chr17	38435000	4.02E-02	promoter	super_enhancer	WIPF2
chr17	38375000	chr17	38455000	2.75E-02	promoter	super_enhancer	WIPF2
chr17	38415000	chr17	46035000	3.37E-02	super_enhancer	promoter	PRR15L
chr17	38415000	chr17	48285000	3.16E-02	super_enhancer	promoter	RP11-893F2.5
chr17	38425000	chr17	38805000	3.38E-02	super_enhancer	promoter	SMARCE1
chr17	38425000	chr17	43325000	4.53E-02	super_enhancer	promoter	MAP3K14-AS1
chr17	38425000	chr17	80375000	8.44E-03	super_enhancer	promoter	HEXDC

chr17	38435000	chr17	39845000	3.91E-02	super_enhancer	promoter	EIF1
chr17	38435000	chr17	48845000	2.75E-02	super_enhancer	promoter	LINC00483
chr17	38435000	chr17	51835000	1.16E-02	super_enhancer	promoter	RPS2P48
chr17	38435000	chr17	73495000	1.41E-02	super_enhancer	promoter	MIR6785
chr17	38435000	chr17	73875000	1.12E-02	super_enhancer	promoter	TRIM47
chr17	38435000	chr17	74305000	1.03E-02	super_enhancer	promoter	QRICH2
chr17	38435000	chr17	75565000	1.32E-02	super_enhancer	promoter	RP11-13K12.5
chr17	38445000	chr17	56555000	1.75E-02	super_enhancer	promoter	SETP3
chr17	38445000	chr17	79935000	1.09E-02	super_enhancer	promoter	ASPSCR1
chr17	38455000	chr17	80185000	6.38E-03	super_enhancer	promoter	SLC16A3
chr17	38465000	chr17	47785000	3.08E-02	super_enhancer	promoter	RP11-613C6.2
chr17	38465000	chr17	51615000	1.44E-02	super_enhancer	promoter	AC090079.1
chr17	38465000	chr17	53405000	1.63E-02	super_enhancer	promoter	RP11-515O17.3
chr17	38465000	chr17	79365000	8.88E-03	super_enhancer	promoter	RP11-1055B8.4
chr17	38465000	chr17	80415000	7.88E-03	super_enhancer	promoter	NARF
chr17	38475000	chr17	38975000	1.28E-02	super_enhancer	promoter	TMEM99
chr17	38475000	chr17	74865000	1.01E-02	super_enhancer	promoter	MGAT5B
chr17	38475000	chr17	76275000	8.50E-03	super_enhancer	promoter	RP11-219G17.4
chr17	38575000	chr17	64325000	1.38E-02	promoter	super_enhancer	TOP2A
chr17	38575000	chr17	73285000	1.11E-02	promoter	super_enhancer	TOP2A
chr17	38575000	chr17	79835000	8.66E-03	promoter	super_enhancer	TOP2A
chr17	38755000	chr17	38905000	2.11E-03	promoter	super_enhancer	RP5-1028K7.1
chr17	38755000	chr17	40105000	3.45E-04	promoter	super_enhancer	RP5-1028K7.1
chr17	38755000	chr17	45325000	2.83E-02	promoter	super_enhancer	RP5-1028K7.1

chr17	38755000	chr17	48055000	2.83E-02	promoter	super_enhancer	RP5-1028K7.1
chr17	38805000	chr17	56905000	1.83E-02	promoter	super_enhancer	SMARCE1
chr17	38875000	chr17	39625000	2.48E-02	super_enhancer	promoter	KRT32
chr17	38875000	chr17	60885000	1.34E-02	super_enhancer	promoter	RP11-453A12.1
chr17	38895000	chr17	73935000	1.10E-02	super_enhancer	promoter	FBF1
chr17	38895000	chr17	79375000	7.77E-03	super_enhancer	promoter	MIR4740
chr17	38905000	chr17	39625000	3.67E-02	super_enhancer	promoter	KRT32
chr17	38905000	chr17	47575000	3.45E-02	super_enhancer	promoter	RP11-81K2.2
chr17	38915000	chr17	39625000	4.94E-02	super_enhancer	promoter	KRT32
chr17	38915000	chr17	67315000	9.12E-03	super_enhancer	promoter	SNRPGP4
chr17	38975000	chr17	40105000	4.97E-02	promoter	super_enhancer	TMEM99
chr17	38975000	chr17	48075000	2.45E-02	promoter	super_enhancer	TMEM99
chr17	39095000	chr17	45275000	3.46E-02	promoter	super_enhancer	KRT23
chr17	39205000	chr17	49505000	1.52E-02	promoter	super_enhancer	KRTAP2-1
chr17	39205000	chr17	77295000	7.12E-03	promoter	super_enhancer	KRTAP2-1
chr17	39205000	chr17	77435000	7.48E-03	promoter	super_enhancer	KRTAP2-1
chr17	39205000	chr17	78345000	6.59E-03	promoter	super_enhancer	KRTAP2-1
chr17	39255000	chr17	45305000	3.00E-02	promoter	super_enhancer	KRTAP4-8
chr17	39275000	chr17	45325000	2.67E-02	promoter	super_enhancer	KRTAP4-11
chr17	39335000	chr17	45295000	3.59E-02	promoter	super_enhancer	KRTAP4-2
chr17	39335000	chr17	45315000	4.22E-02	promoter	super_enhancer	KRTAP4-2
chr17	39335000	chr17	45365000	4.38E-02	promoter	super_enhancer	KRTAP4-2
chr17	39335000	chr17	50935000	1.40E-02	promoter	super_enhancer	KRTAP4-2
chr17	39335000	chr17	64725000	1.41E-02	promoter	super_enhancer	KRTAP4-2

chr17	39335000	chr17	75705000	9.77E-03	promoter	super_enhancer	KRTAP4-2
chr17	39335000	chr17	78535000	9.94E-03	promoter	super_enhancer	KRTAP4-2
chr17	39335000	chr17	80775000	1.09E-02	promoter	super_enhancer	KRTAP4-2
chr17	39335000	chr17	81065000	8.45E-03	promoter	super_enhancer	KRTAP4-2
chr17	39345000	chr17	57915000	1.52E-02	promoter	super_enhancer	KRTAP9-2
chr17	39345000	chr17	57955000	1.62E-02	promoter	super_enhancer	KRTAP9-2
chr17	39345000	chr17	64325000	1.33E-02	promoter	super_enhancer	KRTAP9-2
chr17	39345000	chr17	73305000	1.05E-02	promoter	super_enhancer	KRTAP9-2
chr17	39345000	chr17	78545000	9.09E-03	promoter	super_enhancer	KRTAP9-2
chr17	39345000	chr17	81105000	8.34E-03	promoter	super_enhancer	KRTAP9-2
chr17	39405000	chr17	45285000	2.71E-02	promoter	super_enhancer	KRTAP9-4
chr17	39405000	chr17	68345000	5.53E-03	promoter	super_enhancer	KRTAP9-4
chr17	39405000	chr17	75125000	7.53E-03	promoter	super_enhancer	KRTAP9-4
chr17	39405000	chr17	78575000	6.70E-03	promoter	super_enhancer	KRTAP9-4
chr17	39555000	chr17	57935000	2.34E-02	promoter	super_enhancer	KRT33B
chr17	39555000	chr17	72425000	1.45E-02	promoter	super_enhancer	KRT33B
chr17	39625000	chr17	41795000	1.77E-02	promoter	super_enhancer	KRT32
chr17	39625000	chr17	45385000	4.71E-02	promoter	super_enhancer	KRT32
chr17	39685000	chr17	72395000	1.45E-02	promoter	super_enhancer	KRT19
chr17	39845000	chr17	45375000	4.82E-02	promoter	super_enhancer	EIF1
chr17	39845000	chr17	57925000	1.68E-02	promoter	super_enhancer	EIF1
chr17	39845000	chr17	59785000	1.24E-02	promoter	super_enhancer	EIF1
chr17	39845000	chr17	78885000	9.27E-03	promoter	super_enhancer	EIF1
chr17	39845000	chr17	80495000	9.96E-03	promoter	super_enhancer	EIF1

chr17	40075000	chr17	46725000	1.07E-03	super_enhancer	promoter	RP11-357H14.17
chr17	40075000	chr17	62505000	1.31E-02	super_enhancer	promoter	DDX5
chr17	40075000	chr17	78995000	1.01E-02	super_enhancer	promoter	CTD-2561B21.11
chr17	40075000	chr17	79335000	1.12E-02	super_enhancer	promoter	RP11-1055B8.2
chr17	40085000	chr17	40265000	2.30E-03	super_enhancer	promoter	DHX58
chr17	40085000	chr17	48945000	2.83E-02	super_enhancer	promoter	TOB1
chr17	40085000	chr17	61855000	1.61E-02	super_enhancer	promoter	CCDC47
chr17	40085000	chr17	73935000	1.27E-02	super_enhancer	promoter	FBF1
chr17	40095000	chr17	40665000	2.10E-02	super_enhancer	promoter	MIR5010
chr17	40095000	chr17	73165000	1.25E-02	super_enhancer	promoter	HN1
chr17	40105000	chr17	46705000	3.75E-02	super_enhancer	promoter	HOXB9
chr17	40105000	chr17	77925000	1.06E-02	super_enhancer	promoter	RP11-353N14.7
chr17	40105000	chr17	79825000	1.13E-02	super_enhancer	promoter	RP11-498C9.3
chr17	40115000	chr17	40905000	4.68E-02	super_enhancer	promoter	CTD-3193K9.11
chr17	40115000	chr17	40975000	3.32E-02	super_enhancer	promoter	PSME3
chr17	40115000	chr17	79225000	1.18E-02	super_enhancer	promoter	RP11-455O6.8
chr17	40195000	chr17	68315000	1.09E-02	promoter	super_enhancer	CTD-2132N18.2
chr17	40195000	chr17	75125000	1.22E-02	promoter	super_enhancer	CTD-2132N18.2
chr17	40195000	chr17	80495000	1.10E-02	promoter	super_enhancer	CTD-2132N18.2
chr17	40195000	chr17	80835000	1.03E-02	promoter	super_enhancer	CTD-2132N18.2
chr17	40195000	chr17	81045000	8.40E-03	promoter	super_enhancer	CTD-2132N18.2
chr17	40265000	chr17	64715000	1.62E-02	promoter	super_enhancer	DHX58
chr17	40265000	chr17	73295000	1.42E-02	promoter	super_enhancer	DHX58
chr17	40265000	chr17	76875000	1.48E-02	promoter	super_enhancer	DHX58

chr17	40315000	chr17	47575000	4.53E-02	super_enhancer	promoter	RP11-81K2.2
chr17	40315000	chr17	54965000	2.09E-02	super_enhancer	promoter	RP11-670E13.5
chr17	40315000	chr17	77025000	9.61E-03	super_enhancer	promoter	C1QTNF1-AS1
chr17	40385000	chr17	75125000	1.12E-02	promoter	super_enhancer	RP11-358B23.6
chr17	40485000	chr17	40665000	4.25E-03	super_enhancer	promoter	MIR5010
chr17	40485000	chr17	56555000	1.52E-02	super_enhancer	promoter	SETP3
chr17	40485000	chr17	56755000	1.81E-02	super_enhancer	promoter	RNU1-52P
chr17	40485000	chr17	62075000	1.35E-02	super_enhancer	promoter	PRR29
chr17	40485000	chr17	76275000	8.16E-03	super_enhancer	promoter	RP11-219G17.4
chr17	40485000	chr17	79635000	1.13E-02	super_enhancer	promoter	OXLD1
chr17	40515000	chr17	48555000	3.51E-02	super_enhancer	promoter	RSAD1
chr17	40515000	chr17	79375000	9.19E-03	super_enhancer	promoter	MIR4740
chr17	40665000	chr17	80755000	7.17E-03	promoter	super_enhancer	MIR5010
chr17	40685000	chr17	48075000	3.32E-02	promoter	super_enhancer	PTP4A2P1
chr17	40685000	chr17	72405000	1.35E-02	promoter	super_enhancer	PTP4A2P1
chr17	40835000	chr17	50035000	1.99E-02	promoter	super_enhancer	CNTNAP1
chr17	40835000	chr17	78525000	1.11E-02	promoter	super_enhancer	CNTNAP1
chr17	40835000	chr17	79805000	1.35E-02	promoter	super_enhancer	CNTNAP1
chr17	40905000	chr17	45295000	4.74E-02	promoter	super_enhancer	CTD-3193K9.11
chr17	40905000	chr17	49515000	2.76E-02	promoter	super_enhancer	CTD-3193K9.11
chr17	40905000	chr17	50645000	1.81E-02	promoter	super_enhancer	CTD-3193K9.11
chr17	40905000	chr17	76875000	1.24E-02	promoter	super_enhancer	CTD-3193K9.11
chr17	40905000	chr17	77285000	9.09E-03	promoter	super_enhancer	CTD-3193K9.11
chr17	40905000	chr17	80265000	1.17E-02	promoter	super_enhancer	CTD-3193K9.11

chr17	40975000	chr17	57875000	1.38E-02	promoter	super_enhancer	PSME3
chr17	40975000	chr17	57935000	1.99E-02	promoter	super_enhancer	PSME3
chr17	40985000	chr17	80865000	9.27E-03	promoter	super_enhancer	BECN1
chr17	41465000	chr17	68375000	1.04E-02	promoter	super_enhancer	RNU2-4P
chr17	41465000	chr17	72375000	1.41E-02	promoter	super_enhancer	RNU2-4P
chr17	41465000	chr17	81115000	7.75E-03	promoter	super_enhancer	RNU2-4P
chr17	41475000	chr17	56905000	1.95E-02	promoter	super_enhancer	ARL4D
chr17	41475000	chr17	75845000	7.43E-03	promoter	super_enhancer	ARL4D
chr17	41475000	chr17	80265000	1.19E-02	promoter	super_enhancer	ARL4D
chr17	41785000	chr17	56745000	2.71E-02	super_enhancer	promoter	RNU1-108P
chr17	41785000	chr17	77925000	1.43E-02	super_enhancer	promoter	RP11-353N14.7
chr17	41785000	chr17	80275000	1.02E-02	super_enhancer	promoter	CD7
chr17	41795000	chr17	66195000	1.56E-02	super_enhancer	promoter	LOC440461
chr17	41925000	chr17	80265000	1.14E-02	promoter	super_enhancer	CD300LG
chr17	41995000	chr17	42425000	1.08E-02	promoter	super_enhancer	FAM215A
chr17	41995000	chr17	59825000	1.44E-02	promoter	super_enhancer	FAM215A
chr17	42005000	chr17	42145000	2.29E-02	super_enhancer	promoter	LSM12
chr17	42005000	chr17	42265000	2.83E-02	super_enhancer	promoter	ASB16-AS1
chr17	42005000	chr17	46975000	4.35E-02	super_enhancer	promoter	RP11-501C14.9
chr17	42005000	chr17	47925000	4.27E-02	super_enhancer	promoter	TAC4
chr17	42005000	chr17	56745000	1.98E-02	super_enhancer	promoter	RNU1-108P
chr17	42005000	chr17	78195000	1.04E-02	super_enhancer	promoter	SGSH
chr17	42015000	chr17	57185000	2.44E-02	super_enhancer	promoter	TRIM37
chr17	42025000	chr17	48845000	3.88E-02	super_enhancer	promoter	LINC00483

chr17	42145000	chr17	59855000	1.56E-02	promoter	super_enhancer	LSM12
chr17	42145000	chr17	75125000	1.38E-02	promoter	super_enhancer	LSM12
chr17	42145000	chr17	79845000	9.84E-03	promoter	super_enhancer	LSM12
chr17	42265000	chr17	42415000	2.47E-02	promoter	super_enhancer	ASB16-AS1
chr17	42265000	chr17	42425000	1.64E-02	promoter	super_enhancer	ASB16-AS1
chr17	42265000	chr17	57915000	1.85E-02	promoter	super_enhancer	ASB16-AS1
chr17	42265000	chr17	77325000	9.17E-03	promoter	super_enhancer	ASB16-AS1
chr17	42265000	chr17	78505000	1.27E-02	promoter	super_enhancer	ASB16-AS1
chr17	42265000	chr17	80265000	1.26E-02	promoter	super_enhancer	ASB16-AS1
chr17	42275000	chr17	42415000	2.86E-03	promoter	super_enhancer	CTB-175E5.7
chr17	42275000	chr17	42425000	1.81E-02	promoter	super_enhancer	CTB-175E5.7
chr17	42275000	chr17	57925000	1.89E-02	promoter	super_enhancer	CTB-175E5.7
chr17	42285000	chr17	42345000	3.46E-02	promoter	super_enhancer	MIR6782
chr17	42285000	chr17	42405000	8.42E-03	promoter	super_enhancer	MIR6782
chr17	42285000	chr17	81065000	8.06E-03	promoter	super_enhancer	MIR6782
chr17	42325000	chr17	61975000	1.71E-02	super_enhancer	promoter	CSH1
chr17	42335000	chr17	42385000	2.08E-02	super_enhancer	promoter	RUNDC3A
chr17	42335000	chr17	43225000	2.34E-02	super_enhancer	promoter	HEXIM1
chr17	42335000	chr17	58755000	3.10E-02	super_enhancer	promoter	BCAS3
chr17	42335000	chr17	61905000	2.70E-02	super_enhancer	promoter	PSMC5
chr17	42345000	chr17	42385000	4.00E-02	super_enhancer	promoter	RUNDC3A
chr17	42345000	chr17	48015000	4.67E-02	super_enhancer	promoter	RP11-304F15.5
chr17	42385000	chr17	42405000	3.33E-02	promoter	super_enhancer	RUNDC3A
chr17	42385000	chr17	42425000	2.58E-02	promoter	super_enhancer	RUNDC3A

chr17	42385000	chr17	73355000	1.19E-02	promoter	super_enhancer	RUNDC3A
chr17	42385000	chr17	75095000	1.37E-02	promoter	super_enhancer	RUNDC3A
chr17	42385000	chr17	78515000	1.31E-02	promoter	super_enhancer	RUNDC3A
chr17	42395000	chr17	57275000	2.57E-02	super_enhancer	promoter	CTD-2510F5.6
chr17	42395000	chr17	62075000	2.11E-02	super_enhancer	promoter	PRR29
chr17	42395000	chr17	79925000	1.62E-02	super_enhancer	promoter	RP11-498C9.17
chr17	42405000	chr17	73105000	1.27E-02	super_enhancer	promoter	ARMC7
chr17	42405000	chr17	76165000	1.13E-02	super_enhancer	promoter	SYNGR2
chr17	42405000	chr17	76275000	1.05E-02	super_enhancer	promoter	RP11-219G17.4
chr17	42415000	chr17	43225000	2.07E-02	super_enhancer	promoter	HEXIM1
chr17	42415000	chr17	56595000	2.85E-02	super_enhancer	promoter	MTMR4
chr17	42635000	chr17	50665000	1.89E-02	promoter	super_enhancer	FZD2
chr17	42635000	chr17	75695000	1.68E-02	promoter	super_enhancer	FZD2
chr17	42635000	chr17	79845000	1.01E-02	promoter	super_enhancer	FZD2
chr17	42635000	chr17	81105000	1.11E-02	promoter	super_enhancer	FZD2
chr17	42755000	chr17	45175000	6.52E-03	promoter	super_enhancer	RP11-1072C15.4
chr17	42755000	chr17	72425000	1.21E-02	promoter	super_enhancer	RP11-1072C15.4
chr17	42785000	chr17	48055000	4.33E-02	promoter	super_enhancer	DBF4B
chr17	42785000	chr17	79845000	7.86E-03	promoter	super_enhancer	DBF4B
chr17	42835000	chr17	79795000	1.13E-02	promoter	super_enhancer	ADAM11
chr17	42835000	chr17	80485000	9.70E-03	promoter	super_enhancer	ADAM11
chr17	42875000	chr17	77315000	1.16E-02	promoter	super_enhancer	CTC-296K1.4
chr17	42875000	chr17	77415000	9.11E-03	promoter	super_enhancer	CTC-296K1.4
chr17	42875000	chr17	78355000	1.37E-02	promoter	super_enhancer	CTC-296K1.4

chr17	42995000	chr17	57935000	2.67E-02	promoter	super_enhancer	GFAP
chr17	42995000	chr17	64345000	1.50E-02	promoter	super_enhancer	GFAP
chr17	43025000	chr17	75095000	1.09E-02	promoter	super_enhancer	KIF18B
chr17	43045000	chr17	57935000	2.85E-02	promoter	super_enhancer	C1QL1
chr17	43045000	chr17	80825000	1.58E-02	promoter	super_enhancer	C1QL1
chr17	43225000	chr17	72405000	1.21E-02	promoter	super_enhancer	HEXIM1
chr17	43325000	chr17	43355000	3.97E-02	promoter	super_enhancer	MAP3K14-AS1
chr17	43325000	chr17	43455000	2.40E-03	promoter	super_enhancer	MAP3K14-AS1
chr17	43325000	chr17	43465000	3.48E-05	promoter	super_enhancer	MAP3K14-AS1
chr17	43325000	chr17	68325000	1.02E-02	promoter	super_enhancer	MAP3K14-AS1
chr17	43355000	chr17	79615000	1.04E-02	super_enhancer	promoter	NPLOC4
chr17	43365000	chr17	70815000	1.58E-02	super_enhancer	promoter	SCARNA24
chr17	43365000	chr17	77025000	1.24E-02	super_enhancer	promoter	C1QTNF1-AS1
chr17	43395000	chr17	43495000	2.67E-02	super_enhancer	promoter	AC091132.2
chr17	43395000	chr17	48475000	4.57E-02	super_enhancer	promoter	LRRC59
chr17	43395000	chr17	56085000	2.45E-02	super_enhancer	promoter	SRSF1
chr17	43395000	chr17	56745000	2.10E-02	super_enhancer	promoter	RNU1-108P
chr17	43395000	chr17	57215000	1.88E-02	super_enhancer	promoter	MIR454
chr17	43395000	chr17	75565000	1.40E-02	super_enhancer	promoter	RP11-13K12.5
chr17	43405000	chr17	43495000	1.86E-02	super_enhancer	promoter	AC091132.2
chr17	43405000	chr17	46975000	2.44E-03	super_enhancer	promoter	RP11-501C14.9
chr17	43405000	chr17	48845000	4.59E-02	super_enhancer	promoter	LINC00483
chr17	43405000	chr17	75565000	1.50E-02	super_enhancer	promoter	RP11-13K12.5
chr17	43405000	chr17	79305000	1.32E-02	super_enhancer	promoter	TMEM105

chr17	43405000	chr17	80875000	8.74E-03	super_enhancer	promoter	RP11-497H17.1
chr17	43415000	chr17	74955000	1.91E-02	super_enhancer	promoter	RP11-87G24.6
chr17	43415000	chr17	77025000	1.44E-02	super_enhancer	promoter	C1QTNF1-AS1
chr17	43445000	chr17	62075000	1.58E-02	super_enhancer	promoter	PRR29
chr17	43445000	chr17	79995000	1.07E-02	super_enhancer	promoter	RP11-1182P23.5
chr17	43455000	chr17	73405000	1.06E-02	super_enhancer	promoter	RNU6-938P
chr17	43455000	chr17	74555000	1.07E-02	super_enhancer	promoter	RP11-666A8.8
chr17	43455000	chr17	76375000	9.09E-03	super_enhancer	promoter	PGS1
chr17	43455000	chr17	77775000	1.06E-02	super_enhancer	promoter	CBX8
chr17	43465000	chr17	60595000	2.05E-02	super_enhancer	promoter	RP11-464D20.2
chr17	43495000	chr17	75085000	1.27E-02	promoter	super_enhancer	AC091132.2
chr17	43585000	chr17	56495000	1.24E-02	super_enhancer	promoter	RNF43
chr17	43585000	chr17	73975000	7.09E-03	super_enhancer	promoter	ACOX1
chr17	43585000	chr17	74305000	6.63E-03	super_enhancer	promoter	QRICH2
chr17	43685000	chr17	57935000	1.87E-02	promoter	super_enhancer	RPS26P8
chr17	44905000	chr17	55695000	2.03E-02	super_enhancer	promoter	RN7SL449P
chr17	44905000	chr17	57215000	2.02E-02	super_enhancer	promoter	MIR454
chr17	44905000	chr17	70025000	1.05E-02	super_enhancer	promoter	LINC01152
chr17	45175000	chr17	74555000	1.17E-02	super_enhancer	promoter	RP11-666A8.8
chr17	45195000	chr17	81085000	7.12E-03	promoter	super_enhancer	AC002558.1
chr17	45275000	chr17	46075000	4.32E-02	super_enhancer	promoter	RP11-6N17.10
chr17	45275000	chr17	55175000	3.13E-02	super_enhancer	promoter	RP11-166P13.4
chr17	45285000	chr17	68625000	9.38E-03	super_enhancer	promoter	RP11-1058G23.1
chr17	45285000	chr17	74095000	1.19E-02	super_enhancer	promoter	MIR6868

chr17	45285000	chr17	75915000	1.18E-02	super_enhancer	promoter	RNU1-80P
chr17	45295000	chr17	60535000	1.79E-02	super_enhancer	promoter	TLK2
chr17	45295000	chr17	80935000	4.40E-03	super_enhancer	promoter	RP11-1197K16.2
chr17	45305000	chr17	47575000	1.74E-02	super_enhancer	promoter	RP11-81K2.2
chr17	45305000	chr17	60595000	1.98E-02	super_enhancer	promoter	RP11-464D20.2
chr17	45305000	chr17	74865000	1.31E-02	super_enhancer	promoter	MGAT5B
chr17	45315000	chr17	60595000	1.93E-02	super_enhancer	promoter	RP11-464D20.2
chr17	45315000	chr17	74085000	1.47E-02	super_enhancer	promoter	RP11-449J21.3
chr17	45325000	chr17	47575000	1.06E-02	super_enhancer	promoter	RP11-81K2.2
chr17	45325000	chr17	61855000	1.53E-02	super_enhancer	promoter	CCDC47
chr17	45325000	chr17	75915000	9.69E-03	super_enhancer	promoter	RNU1-80P
chr17	45365000	chr17	46975000	2.75E-02	super_enhancer	promoter	RP11-501C14.9
chr17	45365000	chr17	73165000	1.40E-02	super_enhancer	promoter	HN1
chr17	45365000	chr17	80415000	1.07E-02	super_enhancer	promoter	NARF
chr17	45375000	chr17	47965000	1.22E-02	super_enhancer	promoter	RP11-304F15.4
chr17	45375000	chr17	55175000	3.33E-02	super_enhancer	promoter	RP11-166P13.4
chr17	45375000	chr17	73495000	1.95E-02	super_enhancer	promoter	MIR6785
chr17	45375000	chr17	74085000	1.64E-02	super_enhancer	promoter	RP11-449J21.3
chr17	45375000	chr17	78965000	1.09E-02	super_enhancer	promoter	CHMP6
chr17	45385000	chr17	57275000	2.42E-02	super_enhancer	promoter	CTD-2510F5.6
chr17	45385000	chr17	60705000	2.84E-02	super_enhancer	promoter	CTD-3035K23.7
chr17	45385000	chr17	62075000	1.95E-02	super_enhancer	promoter	PRR29
chr17	46035000	chr17	57875000	2.05E-02	promoter	super_enhancer	PRR15L
chr17	46035000	chr17	57925000	2.51E-02	promoter	super_enhancer	PRR15L

chr17	46035000	chr17	81105000	1.19E-02	promoter	super_enhancer	PRR15L
chr17	46045000	chr17	50645000	4.16E-02	promoter	super_enhancer	CDK5RAP3
chr17	46045000	chr17	50945000	3.94E-02	promoter	super_enhancer	CDK5RAP3
chr17	46045000	chr17	64715000	1.93E-02	promoter	super_enhancer	CDK5RAP3
chr17	46045000	chr17	77255000	1.57E-02	promoter	super_enhancer	CDK5RAP3
chr17	46075000	chr17	57875000	2.02E-02	promoter	super_enhancer	RP11-6N17.10
chr17	46075000	chr17	72365000	1.69E-02	promoter	super_enhancer	RP11-6N17.10
chr17	46075000	chr17	79835000	1.17E-02	promoter	super_enhancer	RP11-6N17.10
chr17	46115000	chr17	64735000	1.53E-04	promoter	super_enhancer	COPZ2
chr17	46115000	chr17	80895000	1.23E-02	promoter	super_enhancer	COPZ2
chr17	46125000	chr17	81055000	7.61E-03	promoter	super_enhancer	RP5-890E16.2
chr17	46145000	chr17	57945000	2.55E-02	promoter	super_enhancer	RP5-890E16.5
chr17	46145000	chr17	81105000	1.06E-02	promoter	super_enhancer	RP5-890E16.5
chr17	46235000	chr17	80255000	1.19E-02	promoter	super_enhancer	MIR1203
chr17	46275000	chr17	50945000	3.17E-02	promoter	super_enhancer	RNU6-1152P
chr17	46275000	chr17	57935000	2.34E-02	promoter	super_enhancer	RNU6-1152P
chr17	46275000	chr17	64735000	1.29E-02	promoter	super_enhancer	RNU6-1152P
chr17	46275000	chr17	75705000	1.02E-02	promoter	super_enhancer	RNU6-1152P
chr17	46275000	chr17	80825000	1.22E-02	promoter	super_enhancer	RNU6-1152P
chr17	46635000	chr17	80825000	1.32E-02	promoter	super_enhancer	HOXB-AS2
chr17	46705000	chr17	72375000	1.65E-02	promoter	super_enhancer	HOXB9
chr17	46705000	chr17	80755000	8.95E-03	promoter	super_enhancer	HOXB9
chr17	46725000	chr17	57915000	2.73E-02	promoter	super_enhancer	RP11-357H14.17
chr17	46725000	chr17	75725000	1.66E-02	promoter	super_enhancer	RP11-357H14.17

chr17	46895000	chr17	56935000	2.68E-02	promoter	super_enhancer	TTLL6
chr17	46895000	chr17	57955000	2.54E-02	promoter	super_enhancer	TTLL6
chr17	46895000	chr17	78605000	9.20E-03	promoter	super_enhancer	TTLL6
chr17	46975000	chr17	64355000	1.71E-02	promoter	super_enhancer	RP11-501C14.9
chr17	46975000	chr17	78585000	1.04E-02	promoter	super_enhancer	RP11-501C14.9
chr17	46975000	chr17	80495000	1.19E-02	promoter	super_enhancer	RP11-501C14.9
chr17	47075000	chr17	57915000	1.97E-02	promoter	super_enhancer	RP11-501C14.5
chr17	47075000	chr17	72365000	1.37E-02	promoter	super_enhancer	RP11-501C14.5
chr17	47485000	chr17	48055000	3.67E-02	promoter	super_enhancer	AC091180.1
chr17	47485000	chr17	48075000	7.47E-03	promoter	super_enhancer	AC091180.1
chr17	47485000	chr17	68395000	1.26E-02	promoter	super_enhancer	AC091180.1
chr17	47485000	chr17	73325000	1.84E-02	promoter	super_enhancer	AC091180.1
chr17	47575000	chr17	59795000	2.24E-02	promoter	super_enhancer	RP11-81K2.2
chr17	47575000	chr17	64675000	2.60E-02	promoter	super_enhancer	RP11-81K2.2
chr17	47575000	chr17	78515000	1.66E-02	promoter	super_enhancer	RP11-81K2.2
chr17	47575000	chr17	79785000	1.76E-02	promoter	super_enhancer	RP11-81K2.2
chr17	47575000	chr17	80255000	1.63E-02	promoter	super_enhancer	RP11-81K2.2
chr17	47575000	chr17	80835000	1.53E-02	promoter	super_enhancer	RP11-81K2.2
chr17	47655000	chr17	48055000	1.04E-02	promoter	super_enhancer	RP5-1029K10.4
chr17	47655000	chr17	48075000	4.21E-04	promoter	super_enhancer	RP5-1029K10.4
chr17	47655000	chr17	56935000	3.49E-02	promoter	super_enhancer	RP5-1029K10.4
chr17	47655000	chr17	72395000	1.96E-02	promoter	super_enhancer	RP5-1029K10.4
chr17	47655000	chr17	72405000	2.33E-02	promoter	super_enhancer	RP5-1029K10.4
chr17	47655000	chr17	78355000	1.85E-02	promoter	super_enhancer	RP5-1029K10.4

chr17	47785000	chr17	48045000	1.55E-02	promoter	super_enhancer	RP11-613C6.2
chr17	47785000	chr17	48075000	1.73E-02	promoter	super_enhancer	RP11-613C6.2
chr17	47785000	chr17	73315000	1.75E-02	promoter	super_enhancer	RP11-613C6.2
chr17	47785000	chr17	80265000	1.71E-02	promoter	super_enhancer	RP11-613C6.2
chr17	47785000	chr17	80505000	1.53E-02	promoter	super_enhancer	RP11-613C6.2
chr17	47865000	chr17	57955000	2.64E-02	promoter	super_enhancer	KAT7
chr17	47865000	chr17	64725000	1.98E-02	promoter	super_enhancer	KAT7
chr17	47865000	chr17	73305000	1.52E-02	promoter	super_enhancer	KAT7
chr17	47865000	chr17	73355000	1.24E-02	promoter	super_enhancer	KAT7
chr17	47865000	chr17	78885000	1.25E-02	promoter	super_enhancer	KAT7
chr17	47925000	chr17	48065000	2.97E-02	promoter	super_enhancer	TAC4
chr17	47925000	chr17	48075000	3.01E-02	promoter	super_enhancer	TAC4
chr17	47925000	chr17	64675000	2.29E-02	promoter	super_enhancer	TAC4
chr17	47925000	chr17	77415000	1.06E-02	promoter	super_enhancer	TAC4
chr17	47925000	chr17	78555000	1.37E-02	promoter	super_enhancer	TAC4
chr17	47965000	chr17	48045000	1.33E-03	promoter	super_enhancer	RP11-304F15.4
chr17	47965000	chr17	48065000	1.85E-04	promoter	super_enhancer	RP11-304F15.4
chr17	47965000	chr17	48075000	1.88E-03	promoter	super_enhancer	RP11-304F15.4
chr17	47965000	chr17	59825000	2.28E-02	promoter	super_enhancer	RP11-304F15.4
chr17	47965000	chr17	81105000	1.38E-02	promoter	super_enhancer	RP11-304F15.4
chr17	48015000	chr17	57895000	2.31E-02	promoter	super_enhancer	RP11-304F15.5
chr17	48045000	chr17	60005000	3.95E-02	super_enhancer	promoter	INTS2
chr17	48045000	chr17	62755000	2.50E-02	super_enhancer	promoter	RP13-104F24.1
chr17	48045000	chr17	74555000	1.95E-02	super_enhancer	promoter	RP11-666A8.8

chr17	48045000	chr17	80535000	1.16E-02	super_enhancer	promoter	RP13-638C3.6
chr17	48055000	chr17	57605000	2.73E-02	super_enhancer	promoter	LINC01476
chr17	48065000	chr17	74955000	1.55E-02	super_enhancer	promoter	RP11-87G24.6
chr17	48075000	chr17	56755000	3.07E-02	super_enhancer	promoter	RNU1-52P
chr17	48075000	chr17	61855000	2.29E-02	super_enhancer	promoter	CCDC47
chr17	48075000	chr17	69295000	1.36E-02	super_enhancer	promoter	RNU7-155P
chr17	48075000	chr17	79635000	1.64E-02	super_enhancer	promoter	OXLD1
chr17	48075000	chr17	80375000	1.37E-02	super_enhancer	promoter	HEXDC
chr17	48285000	chr17	57875000	2.58E-02	promoter	super_enhancer	RP11-893F2.5
chr17	48365000	chr17	57925000	2.95E-02	promoter	super_enhancer	TMEM92-AS1
chr17	48365000	chr17	57945000	3.35E-02	promoter	super_enhancer	TMEM92-AS1
chr17	48475000	chr17	75685000	1.34E-02	promoter	super_enhancer	LRRC59
chr17	48475000	chr17	77255000	1.53E-02	promoter	super_enhancer	LRRC59
chr17	48475000	chr17	79835000	1.19E-02	promoter	super_enhancer	LRRC59
chr17	48555000	chr17	57935000	3.29E-02	promoter	super_enhancer	RSAD1
chr17	48555000	chr17	73365000	1.24E-02	promoter	super_enhancer	RSAD1
chr17	48555000	chr17	77255000	1.62E-02	promoter	super_enhancer	RSAD1
chr17	48555000	chr17	81065000	1.16E-02	promoter	super_enhancer	RSAD1
chr17	48585000	chr17	57945000	3.84E-02	promoter	super_enhancer	MYCBPAP
chr17	48585000	chr17	73295000	2.03E-02	promoter	super_enhancer	MYCBPAP
chr17	48785000	chr17	57875000	2.11E-02	promoter	super_enhancer	ANKRD40
chr17	48785000	chr17	67395000	1.55E-02	promoter	super_enhancer	ANKRD40
chr17	48785000	chr17	68345000	1.13E-02	promoter	super_enhancer	ANKRD40
chr17	48785000	chr17	78585000	1.14E-02	promoter	super_enhancer	ANKRD40

chr17	48875000	chr17	62795000	1.10E-02	promoter	super_enhancer	RP11-294J22.5
chr17	48875000	chr17	64725000	2.32E-02	promoter	super_enhancer	RP11-294J22.5
chr17	48925000	chr17	57865000	2.31E-02	promoter	super_enhancer	RPL5P33
chr17	48925000	chr17	67415000	1.42E-02	promoter	super_enhancer	RPL5P33
chr17	48925000	chr17	73365000	1.29E-02	promoter	super_enhancer	RPL5P33
chr17	48945000	chr17	57865000	1.99E-02	promoter	super_enhancer	TOB1
chr17	48945000	chr17	64725000	1.90E-02	promoter	super_enhancer	TOB1
chr17	49265000	chr17	72405000	1.64E-02	promoter	super_enhancer	RP11-481C4.2
chr17	49515000	chr17	54445000	3.13E-02	super_enhancer	promoter	RP11-502F1.2
chr17	49515000	chr17	56065000	3.02E-02	super_enhancer	promoter	RP11-159D12.3
chr17	49515000	chr17	60375000	1.67E-02	super_enhancer	promoter	RP11-51L5.5
chr17	49515000	chr17	68625000	1.10E-02	super_enhancer	promoter	RP11-1058G23.1
chr17	49515000	chr17	70815000	1.53E-02	super_enhancer	promoter	SCARNA24
chr17	49525000	chr17	51185000	9.44E-03	super_enhancer	promoter	AC091154.1
chr17	49525000	chr17	57615000	2.13E-02	super_enhancer	promoter	RNU4-13P
chr17	49525000	chr17	60705000	2.76E-02	super_enhancer	promoter	CTD-3035K23.7
chr17	50045000	chr17	61525000	1.91E-02	super_enhancer	promoter	CYB561
chr17	50055000	chr17	54965000	6.27E-04	super_enhancer	promoter	RP11-670E13.5
chr17	50065000	chr17	55055000	3.29E-02	super_enhancer	promoter	SCPEP1
chr17	50075000	chr17	64635000	1.59E-02	super_enhancer	promoter	RNA5SP445
chr17	50075000	chr17	79365000	9.71E-03	super_enhancer	promoter	RP11-1055B8.4
chr17	50135000	chr17	66455000	1.31E-02	super_enhancer	promoter	WIPI1
chr17	50145000	chr17	53405000	4.92E-02	super_enhancer	promoter	RP11-515O17.3
chr17	50145000	chr17	79825000	1.11E-02	super_enhancer	promoter	RP11-498C9.3

chr17	50155000	chr17	56065000	2.68E-02	super_enhancer	promoter	RP11-159D12.3
chr17	50155000	chr17	78945000	1.11E-02	super_enhancer	promoter	CTD-2561B21.5
chr17	50635000	chr17	60705000	2.78E-02	super_enhancer	promoter	CTD-3035K23.7
chr17	50655000	chr17	74085000	1.21E-02	super_enhancer	promoter	RP11-449J21.3
chr17	50665000	chr17	51615000	1.55E-02	super_enhancer	promoter	AC090079.1
chr17	50665000	chr17	56085000	2.70E-02	super_enhancer	promoter	SRSF1
chr17	50665000	chr17	57785000	1.66E-02	super_enhancer	promoter	PTRH2
chr17	50665000	chr17	58095000	9.48E-03	super_enhancer	promoter	TBC1D3P1
chr17	50685000	chr17	56595000	2.91E-02	super_enhancer	promoter	MTMR4
chr17	50935000	chr17	55355000	3.25E-02	super_enhancer	promoter	RP11-426D19.1
chr17	50935000	chr17	57185000	2.13E-02	super_enhancer	promoter	TRIM37
chr17	50935000	chr17	57785000	1.76E-02	super_enhancer	promoter	PTRH2
chr17	50945000	chr17	73875000	1.15E-02	super_enhancer	promoter	TRIM47
chr17	51835000	chr17	62795000	5.48E-03	promoter	super_enhancer	RPS2P48
chr17	51835000	chr17	64695000	1.02E-02	promoter	super_enhancer	RPS2P48
chr17	53045000	chr17	68335000	1.32E-02	promoter	super_enhancer	STXBP4
chr17	53405000	chr17	68395000	1.07E-02	promoter	super_enhancer	RP11-515O17.3
chr17	53405000	chr17	72425000	1.62E-02	promoter	super_enhancer	RP11-515O17.3
chr17	54965000	chr17	64355000	2.44E-02	promoter	super_enhancer	RP11-670E13.5
chr17	54965000	chr17	68345000	1.40E-02	promoter	super_enhancer	RP11-670E13.5
chr17	55055000	chr17	72365000	1.95E-02	promoter	super_enhancer	SCPEP1
chr17	55175000	chr17	64735000	2.47E-02	promoter	super_enhancer	RP11-166P13.4
chr17	55355000	chr17	72405000	2.52E-02	promoter	super_enhancer	RP11-426D19.1
chr17	55525000	chr17	72385000	2.07E-02	promoter	super_enhancer	RP11-226M10.3

chr17	55695000	chr17	73355000	1.37E-02	promoter	super_enhancer	RN7SL449P
chr17	56065000	chr17	59835000	1.44E-03	promoter	super_enhancer	RP11-159D12.3
chr17	56295000	chr17	73325000	2.02E-02	promoter	super_enhancer	LPO
chr17	56495000	chr17	67435000	1.91E-02	promoter	super_enhancer	RNF43
chr17	56535000	chr17	68315000	1.61E-02	promoter	super_enhancer	AC023992.1
chr17	56555000	chr17	75725000	1.64E-02	promoter	super_enhancer	SETP3
chr17	56755000	chr17	57925000	7.01E-03	promoter	super_enhancer	RNU1-52P
chr17	56755000	chr17	67405000	2.05E-02	promoter	super_enhancer	RNU1-52P
chr17	56755000	chr17	73295000	2.16E-02	promoter	super_enhancer	RNU1-52P
chr17	56755000	chr17	77255000	2.01E-02	promoter	super_enhancer	RNU1-52P
chr17	56905000	chr17	57605000	3.43E-02	super_enhancer	promoter	LINC01476
chr17	56905000	chr17	58755000	2.41E-02	super_enhancer	promoter	BCAS3
chr17	56905000	chr17	64635000	3.07E-02	super_enhancer	promoter	RNA5SP445
chr17	56915000	chr17	66195000	2.44E-02	super_enhancer	promoter	LOC440461
chr17	56925000	chr17	67705000	1.25E-02	super_enhancer	promoter	RP1-200G19.1
chr17	56925000	chr17	68165000	1.29E-02	super_enhancer	promoter	KCNJ2
chr17	56935000	chr17	60375000	5.00E-02	super_enhancer	promoter	RP11-51L5.5
chr17	56935000	chr17	62505000	3.39E-02	super_enhancer	promoter	DDX5
chr17	56975000	chr17	62775000	4.09E-02	promoter	super_enhancer	RP11-579O24.1
chr17	56975000	chr17	64655000	2.87E-02	promoter	super_enhancer	RP11-579O24.1
chr17	57185000	chr17	68375000	1.91E-02	promoter	super_enhancer	TRIM37
chr17	57215000	chr17	77295000	1.72E-02	promoter	super_enhancer	MIR454
chr17	57275000	chr17	64725000	2.98E-02	promoter	super_enhancer	CTD-2510F5.6
chr17	57275000	chr17	72375000	2.27E-02	promoter	super_enhancer	CTD-2510F5.6

chr17	57275000	chr17	73335000	1.47E-02	promoter	super_enhancer	CTD-2510F5.6
chr17	57275000	chr17	78315000	1.86E-02	promoter	super_enhancer	CTD-2510F5.6
chr17	57605000	chr17	57935000	5.48E-03	promoter	super_enhancer	LINC01476
chr17	57605000	chr17	73365000	1.56E-02	promoter	super_enhancer	LINC01476
chr17	57605000	chr17	77305000	1.38E-02	promoter	super_enhancer	LINC01476
chr17	57615000	chr17	57945000	4.24E-02	promoter	super_enhancer	RNU4-13P
chr17	57615000	chr17	62785000	3.61E-02	promoter	super_enhancer	RNU4-13P
chr17	57615000	chr17	80495000	1.44E-02	promoter	super_enhancer	RNU4-13P
chr17	57785000	chr17	64695000	2.77E-02	promoter	super_enhancer	PTRH2
chr17	57785000	chr17	68325000	1.61E-02	promoter	super_enhancer	PTRH2
chr17	57785000	chr17	78565000	1.40E-02	promoter	super_enhancer	PTRH2
chr17	57875000	chr17	62755000	2.95E-02	super_enhancer	promoter	RP13-104F24.1
chr17	57915000	chr17	62755000	3.52E-02	super_enhancer	promoter	RP13-104F24.1
chr17	57915000	chr17	73875000	1.98E-02	super_enhancer	promoter	TRIM47
chr17	57915000	chr17	76375000	1.40E-02	super_enhancer	promoter	PGS1
chr17	57915000	chr17	80415000	1.37E-02	super_enhancer	promoter	NARF
chr17	57925000	chr17	58095000	3.00E-02	super_enhancer	promoter	TBC1D3P1
chr17	57925000	chr17	74795000	2.02E-02	super_enhancer	promoter	CTD-2246P4.1
chr17	57935000	chr17	78235000	1.65E-02	super_enhancer	promoter	RNF213
chr17	57945000	chr17	77705000	2.41E-02	super_enhancer	promoter	ENPP7
chr17	57945000	chr17	79885000	2.04E-02	super_enhancer	promoter	MAFG-AS1
chr17	57955000	chr17	78965000	1.42E-02	super_enhancer	promoter	CHMP6
chr17	58095000	chr17	73305000	1.18E-02	promoter	super_enhancer	TBC1D3P1
chr17	58605000	chr17	64325000	4.64E-02	promoter	super_enhancer	APPBP2

chr17	58605000	chr17	77295000	2.19E-02	promoter	super_enhancer	APPBP2
chr17	58755000	chr17	78555000	2.16E-02	promoter	super_enhancer	BCAS3
chr17	58755000	chr17	80485000	2.00E-02	promoter	super_enhancer	BCAS3
chr17	58755000	chr17	80835000	2.08E-02	promoter	super_enhancer	BCAS3
chr17	58805000	chr17	77295000	2.20E-02	promoter	super_enhancer	AC111155.1
chr17	59785000	chr17	61905000	9.77E-03	super_enhancer	promoter	PSMC5
chr17	59785000	chr17	71825000	1.74E-02	super_enhancer	promoter	LINC00469
chr17	59785000	chr17	76395000	1.58E-02	super_enhancer	promoter	SNORA30
chr17	59805000	chr17	71065000	2.13E-02	super_enhancer	promoter	RP11-766H1.1
chr17	59805000	chr17	78975000	1.70E-02	super_enhancer	promoter	CTD-2561B21.7
chr17	59825000	chr17	60595000	1.35E-02	super_enhancer	promoter	RP11-464D20.2
chr17	59835000	chr17	64635000	1.13E-03	super_enhancer	promoter	RNA5SP445
chr17	59835000	chr17	66195000	3.16E-02	super_enhancer	promoter	LOC440461
chr17	59845000	chr17	60595000	8.85E-03	super_enhancer	promoter	RP11-464D20.2
chr17	59855000	chr17	78195000	1.51E-02	super_enhancer	promoter	SGSH
chr17	60005000	chr17	75695000	2.97E-02	promoter	super_enhancer	INTS2
chr17	60085000	chr17	80255000	1.91E-02	promoter	super_enhancer	RP11-342K2.1
chr17	60325000	chr17	80815000	1.51E-02	promoter	super_enhancer	RP11-51L5.3
chr17	60375000	chr17	72385000	1.69E-02	promoter	super_enhancer	RP11-51L5.5
chr17	60375000	chr17	73305000	1.65E-02	promoter	super_enhancer	RP11-51L5.5
chr17	60475000	chr17	77415000	1.05E-02	promoter	super_enhancer	RPS23P7
chr17	60475000	chr17	78355000	1.56E-02	promoter	super_enhancer	RPS23P7
chr17	60595000	chr17	64735000	4.57E-02	promoter	super_enhancer	RP11-464D20.2
chr17	60595000	chr17	78315000	2.10E-02	promoter	super_enhancer	RP11-464D20.2

chr17	60705000	chr17	73285000	3.20E-02	promoter	super_enhancer	CTD-3035K23.7
chr17	60705000	chr17	78595000	2.55E-02	promoter	super_enhancer	CTD-3035K23.7
chr17	60885000	chr17	72385000	2.64E-02	promoter	super_enhancer	RP11-453A12.1
chr17	60885000	chr17	73315000	2.65E-02	promoter	super_enhancer	RP11-453A12.1
chr17	61525000	chr17	79785000	2.44E-02	promoter	super_enhancer	CYB561
chr17	61855000	chr17	72365000	2.93E-02	promoter	super_enhancer	CCDC47
chr17	61855000	chr17	72375000	2.99E-02	promoter	super_enhancer	CCDC47
chr17	61855000	chr17	72415000	4.78E-04	promoter	super_enhancer	CCDC47
chr17	61855000	chr17	75715000	2.42E-02	promoter	super_enhancer	CCDC47
chr17	61855000	chr17	80495000	2.06E-02	promoter	super_enhancer	CCDC47
chr17	61905000	chr17	72395000	3.27E-02	promoter	super_enhancer	PSMC5
chr17	61905000	chr17	72405000	3.87E-02	promoter	super_enhancer	PSMC5
chr17	61905000	chr17	73355000	2.56E-02	promoter	super_enhancer	PSMC5
chr17	62505000	chr17	73295000	2.28E-02	promoter	super_enhancer	DDX5
chr17	62505000	chr17	79855000	1.50E-02	promoter	super_enhancer	DDX5
chr17	62745000	chr17	73325000	3.59E-04	promoter	super_enhancer	MINOS1P2
chr17	62745000	chr17	73335000	1.94E-02	promoter	super_enhancer	MINOS1P2
chr17	62775000	chr17	74955000	2.96E-02	super_enhancer	promoter	RP11-87G24.6
chr17	62775000	chr17	74965000	2.85E-02	super_enhancer	promoter	RP11-87G24.3
chr17	64325000	chr17	68165000	4.83E-02	super_enhancer	promoter	KCNJ2
chr17	64325000	chr17	72745000	3.57E-02	super_enhancer	promoter	RP11-452I5.2
chr17	64325000	chr17	73105000	2.88E-02	super_enhancer	promoter	ARMC7
chr17	64325000	chr17	74795000	2.93E-02	super_enhancer	promoter	CTD-2246P4.1
chr17	64325000	chr17	79925000	2.66E-02	super_enhancer	promoter	RP11-498C9.17

chr17	64335000	chr17	67705000	4.35E-02	super_enhancer	promoter	RP1-200G19.1
chr17	64335000	chr17	73105000	2.29E-02	super_enhancer	promoter	ARMC7
chr17	64335000	chr17	73875000	2.39E-02	super_enhancer	promoter	TRIM47
chr17	64335000	chr17	74305000	2.17E-02	super_enhancer	promoter	QRICH2
chr17	64355000	chr17	80185000	1.55E-02	super_enhancer	promoter	SLC16A3
chr17	64665000	chr17	68775000	3.32E-02	super_enhancer	promoter	RP11-238F2.1
chr17	64675000	chr17	71065000	4.04E-02	super_enhancer	promoter	RP11-766H1.1
chr17	64675000	chr17	71515000	3.09E-02	super_enhancer	promoter	RP11-449L23.3
chr17	64675000	chr17	74545000	2.98E-02	super_enhancer	promoter	RP11-666A8.7
chr17	64685000	chr17	79925000	2.67E-02	super_enhancer	promoter	RP11-498C9.17
chr17	64695000	chr17	76005000	1.72E-02	super_enhancer	promoter	RP11-637C24.5
chr17	64695000	chr17	79935000	2.03E-02	super_enhancer	promoter	ASPSCR1
chr17	64705000	chr17	73165000	2.22E-02	super_enhancer	promoter	HN1
chr17	64715000	chr17	70025000	3.49E-02	super_enhancer	promoter	LINC01152
chr17	64715000	chr17	71825000	3.11E-02	super_enhancer	promoter	LINC00469
chr17	64715000	chr17	73165000	2.87E-02	super_enhancer	promoter	HN1
chr17	64735000	chr17	71065000	3.19E-02	super_enhancer	promoter	RP11-766H1.1
chr17	64735000	chr17	71825000	2.72E-02	super_enhancer	promoter	LINC00469
chr17	64735000	chr17	72675000	2.90E-02	super_enhancer	promoter	CTD-2006K23.2
chr17	66155000	chr17	72385000	3.56E-02	promoter	super_enhancer	RP11-147L13.7
chr17	66155000	chr17	73355000	2.58E-02	promoter	super_enhancer	RP11-147L13.7
chr17	66185000	chr17	67405000	1.91E-02	promoter	super_enhancer	RP11-147L13.14
chr17	66185000	chr17	72395000	2.88E-02	promoter	super_enhancer	RP11-147L13.14
chr17	66195000	chr17	67435000	2.90E-02	promoter	super_enhancer	LOC440461

chr17	66195000	chr17	72405000	4.37E-02	promoter	super_enhancer	LOC440461
chr17	66195000	chr17	73295000	3.36E-02	promoter	super_enhancer	LOC440461
chr17	66195000	chr17	75685000	2.46E-02	promoter	super_enhancer	LOC440461
chr17	66195000	chr17	79765000	1.84E-02	promoter	super_enhancer	LOC440461
chr17	66245000	chr17	73315000	2.86E-02	promoter	super_enhancer	RP11-147L13.2
chr17	66455000	chr17	67415000	5.26E-03	promoter	super_enhancer	WIPI1
chr17	66455000	chr17	72385000	3.85E-02	promoter	super_enhancer	WIPI1
chr17	66795000	chr17	72425000	3.89E-02	promoter	super_enhancer	RP11-118B18.2
chr17	66795000	chr17	75705000	2.21E-02	promoter	super_enhancer	RP11-118B18.2
chr17	66795000	chr17	80825000	2.39E-02	promoter	super_enhancer	RP11-118B18.2
chr17	67095000	chr17	77315000	1.98E-02	promoter	super_enhancer	MIR4524B
chr17	67095000	chr17	78545000	2.00E-02	promoter	super_enhancer	MIR4524B
chr17	67095000	chr17	80785000	1.41E-02	promoter	super_enhancer	MIR4524B
chr17	67095000	chr17	80815000	1.74E-02	promoter	super_enhancer	MIR4524B
chr17	67315000	chr17	80865000	1.53E-02	promoter	super_enhancer	SNRPGP4
chr17	67395000	chr17	68625000	1.99E-02	super_enhancer	promoter	RP11-1058G23.1
chr17	67395000	chr17	73875000	3.58E-02	super_enhancer	promoter	TRIM47
chr17	67425000	chr17	68775000	1.65E-02	super_enhancer	promoter	RP11-238F2.1
chr17	67425000	chr17	71515000	4.79E-02	super_enhancer	promoter	RP11-449L23.3
chr17	67425000	chr17	74085000	4.01E-02	super_enhancer	promoter	RP11-449J21.3
chr17	67435000	chr17	68625000	1.75E-02	super_enhancer	promoter	RP11-1058G23.1
chr17	67435000	chr17	74845000	2.97E-02	super_enhancer	promoter	LINC00868
chr17	67705000	chr17	72425000	4.01E-02	promoter	super_enhancer	RP1-200G19.1
chr17	67945000	chr17	72415000	4.75E-02	promoter	super_enhancer	AC002539.2

chr17	67945000	chr17	75695000	3.04E-02	promoter	super_enhancer	AC002539.2
chr17	67945000	chr17	79835000	1.74E-02	promoter	super_enhancer	AC002539.2
chr17	68325000	chr17	71825000	4.98E-02	super_enhancer	promoter	LINC00469
chr17	68325000	chr17	73405000	3.00E-02	super_enhancer	promoter	RNU6-938P
chr17	68345000	chr17	72745000	4.50E-02	super_enhancer	promoter	RP11-452I5.2
chr17	68345000	chr17	74085000	3.28E-02	super_enhancer	promoter	RP11-449J21.3
chr17	68345000	chr17	77025000	1.81E-02	super_enhancer	promoter	C1QTNF1-AS1
chr17	68345000	chr17	79905000	1.90E-02	super_enhancer	promoter	MYADML2
chr17	68355000	chr17	78995000	2.42E-02	super_enhancer	promoter	CTD-2561B21.11
chr17	68365000	chr17	77825000	1.65E-02	super_enhancer	promoter	RP11-353N14.3
chr17	68375000	chr17	72675000	4.98E-02	super_enhancer	promoter	CTD-2006K23.2
chr17	68375000	chr17	76375000	2.02E-02	super_enhancer	promoter	PGS1
chr17	68385000	chr17	79935000	1.93E-02	super_enhancer	promoter	ASPSCR1
chr17	68385000	chr17	80375000	1.98E-02	super_enhancer	promoter	HEXDC
chr17	68395000	chr17	70835000	2.74E-03	super_enhancer	promoter	RP11-171G2.1
chr17	68395000	chr17	72745000	4.30E-02	super_enhancer	promoter	RP11-452I5.2
chr17	68395000	chr17	77925000	1.93E-02	super_enhancer	promoter	RP11-353N14.7
chr17	68395000	chr17	79995000	1.80E-02	super_enhancer	promoter	RP11-1182P23.5
chr17	68625000	chr17	73355000	2.90E-02	promoter	super_enhancer	RP11-1058G23.1
chr17	70835000	chr17	72375000	3.58E-02	promoter	super_enhancer	RP11-171G2.1
chr17	71065000	chr17	77325000	3.49E-02	promoter	super_enhancer	RP11-766H1.1
chr17	71065000	chr17	79785000	3.60E-02	promoter	super_enhancer	RP11-766H1.1
chr17	71065000	chr17	80265000	3.77E-02	promoter	super_enhancer	RP11-766H1.1
chr17	71515000	chr17	80505000	2.82E-02	promoter	super_enhancer	RP11-449L23.3

chr17	72365000	chr17	79995000	4.28E-02	super_enhancer	promoter	RP11-1182P23.5
chr17	72375000	chr17	72595000	3.05E-02	super_enhancer	promoter	CTD-2006K23.1
chr17	72375000	chr17	78075000	4.74E-02	super_enhancer	promoter	GAA
chr17	72395000	chr17	72595000	1.92E-05	super_enhancer	promoter	CTD-2006K23.1
chr17	72425000	chr17	72675000	3.25E-02	super_enhancer	promoter	CTD-2006K23.2
chr17	72425000	chr17	80535000	2.39E-02	super_enhancer	promoter	RP13-638C3.6
chr17	72595000	chr17	80475000	4.01E-02	promoter	super_enhancer	CTD-2006K23.1
chr17	72745000	chr17	79775000	4.35E-02	promoter	super_enhancer	RP11-452I5.2
chr17	73165000	chr17	80495000	3.99E-02	promoter	super_enhancer	HN1
chr17	73285000	chr17	76275000	4.16E-03	super_enhancer	promoter	RP11-219G17.4
chr17	73285000	chr17	79935000	4.35E-02	super_enhancer	promoter	ASPSCR1
chr17	73295000	chr17	73405000	3.23E-02	super_enhancer	promoter	RNU6-938P
chr17	73295000	chr17	73495000	4.13E-02	super_enhancer	promoter	MIR6785
chr17	73305000	chr17	73405000	3.54E-02	super_enhancer	promoter	RNU6-938P
chr17	73305000	chr17	79335000	4.98E-02	super_enhancer	promoter	RP11-1055B8.2
chr17	73305000	chr17	79905000	4.47E-02	super_enhancer	promoter	MYADML2
chr17	73315000	chr17	73405000	2.09E-02	super_enhancer	promoter	RNU6-938P
chr17	73315000	chr17	79495000	4.13E-02	super_enhancer	promoter	FSCN2
chr17	73315000	chr17	79995000	4.58E-02	super_enhancer	promoter	RP11-1182P23.5
chr17	73315000	chr17	80535000	2.54E-02	super_enhancer	promoter	RP13-638C3.6
chr17	73325000	chr17	73405000	2.94E-02	super_enhancer	promoter	RNU6-938P
chr17	73325000	chr17	79615000	4.14E-02	super_enhancer	promoter	NPLOC4
chr17	73335000	chr17	77925000	4.75E-02	super_enhancer	promoter	RP11-353N14.7
chr17	73345000	chr17	74795000	3.81E-02	super_enhancer	promoter	CTD-2246P4.1

chr17	73345000	chr17	74845000	3.65E-02	super_enhancer	promoter	LINC00868
chr17	73355000	chr17	79365000	3.74E-02	super_enhancer	promoter	RP11-1055B8.4
chr17	73355000	chr17	79495000	3.30E-02	super_enhancer	promoter	FSCN2
chr17	73365000	chr17	79375000	3.12E-02	super_enhancer	promoter	MIR4740
chr17	73365000	chr17	79615000	3.10E-02	super_enhancer	promoter	NPLOC4
chr17	73405000	chr17	80265000	4.31E-02	promoter	super_enhancer	RNU6-938P
chr17	73495000	chr17	81055000	4.55E-02	promoter	super_enhancer	MIR6785
chr17	73495000	chr17	81075000	3.88E-02	promoter	super_enhancer	MIR6785
chr17	73875000	chr17	78605000	4.40E-02	promoter	super_enhancer	TRIM47
chr17	73875000	chr17	79835000	4.47E-02	promoter	super_enhancer	TRIM47
chr17	73935000	chr17	79855000	4.58E-02	promoter	super_enhancer	FBF1
chr17	73975000	chr17	79845000	4.06E-02	promoter	super_enhancer	ACOX1
chr17	73975000	chr17	80795000	4.12E-02	promoter	super_enhancer	ACOX1
chr17	73975000	chr17	81095000	3.37E-02	promoter	super_enhancer	ACOX1
chr17	74025000	chr17	81085000	2.11E-02	promoter	super_enhancer	EVPL
chr17	74305000	chr17	77255000	8.20E-06	promoter	super_enhancer	QRICH2
chr17	74305000	chr17	81075000	3.21E-02	promoter	super_enhancer	QRICH2
chr17	74545000	chr17	77415000	3.42E-03	promoter	super_enhancer	RP11-666A8.7
chr17	74545000	chr17	80495000	4.97E-02	promoter	super_enhancer	RP11-666A8.7
chr17	74565000	chr17	75125000	2.72E-02	promoter	super_enhancer	RP11-666A8.9
chr17	74705000	chr17	80815000	3.72E-02	promoter	super_enhancer	RP11-318A15.8
chr17	74705000	chr17	80825000	4.87E-02	promoter	super_enhancer	RP11-318A15.8
chr17	74795000	chr17	81115000	3.61E-02	promoter	super_enhancer	CTD-2246P4.1
chr17	74845000	chr17	81115000	3.74E-02	promoter	super_enhancer	LINC00868

chr17	74955000	chr17	80855000	4.46E-02	promoter	super_enhancer	RP11-87G24.6
chr17	75095000	chr17	76375000	4.18E-02	super_enhancer	promoter	PGS1
chr17	75465000	chr17	80485000	4.01E-02	promoter	super_enhancer	RP11-75C10.7
chr17	75465000	chr17	80825000	4.82E-02	promoter	super_enhancer	RP11-75C10.7
chr17	75565000	chr17	75705000	8.10E-04	promoter	super_enhancer	RP11-13K12.5
chr17	75845000	chr17	80675000	3.51E-02	super_enhancer	promoter	RP11-388C12.1
chr17	76375000	chr17	78575000	9.72E-03	promoter	super_enhancer	PGS1
chr17	76375000	chr17	81075000	3.96E-02	promoter	super_enhancer	PGS1
chr17	76875000	chr17	77705000	4.98E-03	super_enhancer	promoter	ENPP7
chr17	77025000	chr17	77325000	1.22E-02	promoter	super_enhancer	C1QTNF1-AS1
chr17	77295000	chr17	79375000	1.46E-02	super_enhancer	promoter	MIR4740
chr17	77315000	chr17	78945000	3.37E-02	super_enhancer	promoter	CTD-2561B21.5
chr17	77415000	chr17	78945000	2.09E-03	super_enhancer	promoter	CTD-2561B21.5
chr17	77425000	chr17	78945000	3.84E-02	super_enhancer	promoter	CTD-2561B21.5
chr17	77425000	chr17	80935000	3.73E-02	super_enhancer	promoter	RP11-1197K16.2
chr17	77705000	chr17	81065000	5.29E-03	promoter	super_enhancer	ENPP7
chr17	77775000	chr17	78885000	1.27E-02	promoter	super_enhancer	CBX8
chr17	77925000	chr17	79805000	2.25E-03	promoter	super_enhancer	RP11-353N14.7
chr17	78195000	chr17	78315000	2.51E-04	promoter	super_enhancer	SGSH
chr17	78195000	chr17	78885000	1.45E-02	promoter	super_enhancer	SGSH
chr17	78345000	chr17	78965000	1.77E-03	super_enhancer	promoter	CHMP6
chr17	78515000	chr17	78965000	3.31E-02	super_enhancer	promoter	CHMP6
chr17	78525000	chr17	79305000	4.89E-02	super_enhancer	promoter	TMEM105
chr17	78555000	chr17	78945000	6.11E-03	super_enhancer	promoter	CTD-2561B21.5

chr17	78595000	chr17	78945000	3.64E-02	super_enhancer	promoter	CTD-2561B21.5
chr17	78595000	chr17	78995000	8.89E-04	super_enhancer	promoter	CTD-2561B21.11
chr17	79225000	chr17	79775000	2.68E-02	promoter	super_enhancer	RP11-455O6.8
chr17	79225000	chr17	79795000	8.44E-03	promoter	super_enhancer	RP11-455O6.8
chr17	79495000	chr17	80475000	2.88E-02	promoter	super_enhancer	FSCN2
chr17	79495000	chr17	80835000	5.99E-03	promoter	super_enhancer	FSCN2
chr17	79615000	chr17	79775000	1.41E-02	promoter	super_enhancer	NPLOC4
chr17	79635000	chr17	79785000	2.93E-02	promoter	super_enhancer	OXLD1
chr17	79635000	chr17	79835000	1.04E-02	promoter	super_enhancer	OXLD1
chr17	79665000	chr17	79765000	1.54E-02	promoter	super_enhancer	RP13-1032I1.11
chr17	79665000	chr17	79805000	4.99E-02	promoter	super_enhancer	RP13-1032I1.11
chr17	79775000	chr17	79825000	2.96E-02	super_enhancer	promoter	RP11-498C9.3
chr17	79775000	chr17	80025000	1.04E-02	super_enhancer	promoter	DUS1L
chr17	79805000	chr17	79925000	1.08E-02	super_enhancer	promoter	RP11-498C9.17
chr17	79825000	chr17	79845000	2.35E-02	promoter	super_enhancer	RP11-498C9.3
chr17	79825000	chr17	79855000	3.80E-02	promoter	super_enhancer	RP11-498C9.3
chr17	79935000	chr17	81085000	6.79E-03	promoter	super_enhancer	ASPSCR1
chr17	79995000	chr17	80785000	4.62E-03	promoter	super_enhancer	RP11-1182P23.5
chr17	80185000	chr17	80485000	5.22E-04	promoter	super_enhancer	SLC16A3
chr17	80185000	chr17	80855000	2.35E-02	promoter	super_enhancer	SLC16A3
chr17	80185000	chr17	81095000	8.85E-03	promoter	super_enhancer	SLC16A3
chr17	80255000	chr17	80275000	4.45E-02	super_enhancer	promoter	CD7
chr17	80255000	chr17	80535000	1.24E-02	super_enhancer	promoter	RP13-638C3.6
chr17	80265000	chr17	80375000	2.18E-02	super_enhancer	promoter	HEXDC

chr17	80265000	chr17	80535000	1.03E-06	super_enhancer	promoter	RP13-638C3.6
chr17	80275000	chr17	80495000	1.02E-03	promoter	super_enhancer	CD7
chr17	80275000	chr17	80765000	1.23E-02	promoter	super_enhancer	CD7
chr17	80375000	chr17	80475000	2.58E-02	promoter	super_enhancer	HEXDC
chr17	80375000	chr17	80505000	1.69E-02	promoter	super_enhancer	HEXDC
chr17	80375000	chr17	80855000	8.32E-03	promoter	super_enhancer	HEXDC
chr17	80415000	chr17	80795000	1.60E-02	promoter	super_enhancer	NARF
chr17	80415000	chr17	81095000	3.61E-02	promoter	super_enhancer	NARF
chr17	80475000	chr17	80535000	4.09E-02	super_enhancer	promoter	RP13-638C3.6
chr17	80475000	chr17	80935000	4.31E-02	super_enhancer	promoter	RP11-1197K16.2
chr17	80495000	chr17	80535000	3.66E-02	super_enhancer	promoter	RP13-638C3.6
chr17	80505000	chr17	80675000	5.87E-03	super_enhancer	promoter	RP11-388C12.1
chr17	80535000	chr17	80835000	3.62E-02	promoter	super_enhancer	RP13-638C3.6
chr17	80535000	chr17	80865000	1.86E-02	promoter	super_enhancer	RP13-638C3.6
chr17	80535000	chr17	81055000	6.87E-03	promoter	super_enhancer	RP13-638C3.6
chr17	80535000	chr17	81085000	1.81E-02	promoter	super_enhancer	RP13-638C3.6
chr17	80675000	chr17	80815000	1.54E-02	promoter	super_enhancer	RP11-388C12.1
chr17	80675000	chr17	81105000	3.56E-02	promoter	super_enhancer	RP11-388C12.1
chr17	80765000	chr17	80875000	1.69E-02	super_enhancer	promoter	RP11-497H17.1
chr17	80765000	chr17	80935000	2.69E-02	super_enhancer	promoter	RP11-1197K16.2
chr17	80825000	chr17	80875000	3.61E-03	super_enhancer	promoter	RP11-497H17.1
chr17	80825000	chr17	80935000	1.93E-02	super_enhancer	promoter	RP11-1197K16.2
chr17	80875000	chr17	81105000	4.22E-02	promoter	super_enhancer	RP11-497H17.1
chr17	80875000	chr17	81115000	1.65E-02	promoter	super_enhancer	RP11-497H17.1

chr18	425000	chr18	9785000	1.38E-02	promoter	super_enhancer	RP11-720L2.2
chr18	515000	chr18	3795000	4.90E-02	promoter	super_enhancer	RP11-14P20.1
chr18	905000	chr18	8805000	2.04E-02	promoter	super_enhancer	ADCYAP1
chr18	905000	chr18	9925000	1.91E-02	promoter	super_enhancer	ADCYAP1
chr18	1365000	chr18	46315000	2.46E-03	promoter	super_enhancer	RP11-288C17.1
chr18	1655000	chr18	9805000	6.92E-05	promoter	super_enhancer	CTD-2015H3.2
chr18	1655000	chr18	9915000	1.37E-02	promoter	super_enhancer	CTD-2015H3.2
chr18	2555000	chr18	9755000	2.20E-02	promoter	super_enhancer	snoU109
chr18	2655000	chr18	9805000	2.30E-02	promoter	super_enhancer	CBX3P2
chr18	2655000	chr18	9905000	2.92E-02	promoter	super_enhancer	CBX3P2
chr18	3025000	chr18	3655000	1.22E-02	promoter	super_enhancer	RP11-193E15.4
chr18	3025000	chr18	8785000	3.68E-02	promoter	super_enhancer	RP11-193E15.4
chr18	3025000	chr18	9745000	2.05E-02	promoter	super_enhancer	RP11-193E15.4
chr18	3415000	chr18	3655000	2.90E-02	promoter	super_enhancer	BOD1P1
chr18	3465000	chr18	3595000	1.35E-03	promoter	super_enhancer	GAPLINC
chr18	3595000	chr18	9885000	4.12E-02	super_enhancer	promoter	TXNDC2
chr18	3605000	chr18	10625000	1.39E-02	super_enhancer	promoter	CCDC58P1
chr18	3625000	chr18	8155000	2.40E-02	super_enhancer	promoter	RP11-376P6.3
chr18	3625000	chr18	9135000	2.36E-02	super_enhancer	promoter	ANKRD12
chr18	3625000	chr18	19285000	1.55E-02	super_enhancer	promoter	ABHD3
chr18	3625000	chr18	20715000	1.19E-02	super_enhancer	promoter	CABLES1
chr18	3625000	chr18	26405000	4.65E-03	super_enhancer	promoter	RP11-510D21.2
chr18	3655000	chr18	9885000	3.05E-02	super_enhancer	promoter	TXNDC2
chr18	3765000	chr18	6955000	4.11E-02	super_enhancer	promoter	RP11-781P6.1

chr18	3765000	chr18	8705000	3.82E-02	super_enhancer	promoter	MTCL1
chr18	3775000	chr18	8705000	3.60E-02	super_enhancer	promoter	MTCL1
chr18	3785000	chr18	6955000	4.21E-02	super_enhancer	promoter	RP11-781P6.1
chr18	3785000	chr18	9135000	2.25E-02	super_enhancer	promoter	ANKRD12
chr18	3785000	chr18	37645000	4.26E-03	super_enhancer	promoter	LINC01477
chr18	3795000	chr18	23805000	8.11E-03	super_enhancer	promoter	TAF4B
chr18	3795000	chr18	24805000	6.54E-03	super_enhancer	promoter	RP11-57J16.1
chr18	3885000	chr18	9735000	2.58E-02	promoter	super_enhancer	MIR6718
chr18	3885000	chr18	9905000	2.73E-02	promoter	super_enhancer	MIR6718
chr18	4265000	chr18	9845000	3.07E-02	promoter	super_enhancer	DLGAP1-AS5
chr18	4455000	chr18	8785000	3.48E-02	promoter	super_enhancer	DLGAP1
chr18	5955000	chr18	9835000	4.71E-02	promoter	super_enhancer	RP11-793A3.1
chr18	5955000	chr18	46305000	4.02E-03	promoter	super_enhancer	RP11-793A3.1
chr18	6025000	chr18	9825000	3.60E-02	promoter	super_enhancer	RP11-793A3.2
chr18	6415000	chr18	9915000	4.73E-02	promoter	super_enhancer	L3MBTL4
chr18	6455000	chr18	9815000	3.03E-02	promoter	super_enhancer	RNU6-349P
chr18	6455000	chr18	9825000	4.21E-02	promoter	super_enhancer	RNU6-349P
chr18	8775000	chr18	9615000	4.67E-02	super_enhancer	promoter	PPP4R1
chr18	8785000	chr18	20165000	1.42E-02	super_enhancer	promoter	RPS4XP18
chr18	8795000	chr18	19495000	1.30E-02	super_enhancer	promoter	RP11-268I9.4
chr18	8805000	chr18	23805000	8.49E-03	super_enhancer	promoter	TAF4B
chr18	8805000	chr18	32965000	7.05E-03	super_enhancer	promoter	RP11-322E11.1
chr18	9615000	chr18	9725000	1.45E-03	promoter	super_enhancer	PPP4R1
chr18	9615000	chr18	9735000	4.32E-02	promoter	super_enhancer	PPP4R1

chr18	9715000	chr18	10415000	1.76E-02	super_enhancer	promoter	LINC01254
chr18	9725000	chr18	13715000	2.75E-02	super_enhancer	promoter	RN7SL362P
chr18	9735000	chr18	10525000	8.13E-04	super_enhancer	promoter	NAPG
chr18	9745000	chr18	37725000	4.75E-03	super_enhancer	promoter	RPL17P45
chr18	9755000	chr18	19785000	1.57E-02	super_enhancer	promoter	RP11-627G18.1
chr18	9755000	chr18	61085000	5.68E-03	super_enhancer	promoter	RP11-635N19.2
chr18	9765000	chr18	10625000	2.25E-02	super_enhancer	promoter	CCDC58P1
chr18	9765000	chr18	19435000	8.97E-03	super_enhancer	promoter	RP11-268I9.1
chr18	9775000	chr18	29765000	6.27E-03	super_enhancer	promoter	MEP1B
chr18	9785000	chr18	19495000	1.33E-02	super_enhancer	promoter	RP11-268I9.4
chr18	9785000	chr18	22305000	1.01E-02	super_enhancer	promoter	RP11-449D8.5
chr18	9815000	chr18	11995000	3.92E-03	super_enhancer	promoter	RP11-820I16.3
chr18	9825000	chr18	19475000	1.39E-02	super_enhancer	promoter	RP11-268I9.3
chr18	9825000	chr18	32925000	9.31E-03	super_enhancer	promoter	ZNF24
chr18	9825000	chr18	48745000	6.11E-03	super_enhancer	promoter	MEX3C
chr18	9825000	chr18	77835000	2.57E-03	super_enhancer	promoter	SLC25A6P4
chr18	9835000	chr18	19285000	3.08E-02	super_enhancer	promoter	ABHD3
chr18	9835000	chr18	43965000	9.44E-03	super_enhancer	promoter	RP11-63I8.1
chr18	9845000	chr18	9885000	3.63E-02	super_enhancer	promoter	TXNDC2
chr18	9845000	chr18	20715000	1.90E-02	super_enhancer	promoter	CABLES1
chr18	9845000	chr18	21475000	1.12E-02	super_enhancer	promoter	AC010754.1
chr18	9905000	chr18	19285000	2.60E-02	super_enhancer	promoter	ABHD3
chr18	9915000	chr18	77155000	2.37E-03	super_enhancer	promoter	NFATC1
chr18	9925000	chr18	33175000	5.84E-03	super_enhancer	promoter	RP11-712P20.2

chr18	13525000	chr18	46305000	5.22E-03	promoter	super_enhancer	RP11-53B2.6
chr18	13715000	chr18	48025000	3.54E-03	promoter	super_enhancer	RN7SL362P
chr18	23845000	chr18	36155000	7.18E-03	promoter	super_enhancer	RP11-25D3.1
chr18	23845000	chr18	48005000	5.25E-03	promoter	super_enhancer	RP11-25D3.1
chr18	28925000	chr18	36185000	7.03E-03	promoter	super_enhancer	RNU6-167P
chr18	29265000	chr18	36185000	6.87E-03	promoter	super_enhancer	RP11-549B18.1
chr18	33295000	chr18	48025000	6.53E-03	promoter	super_enhancer	RP11-873E20.1
chr18	34505000	chr18	48015000	7.04E-03	promoter	super_enhancer	RP11-58G13.1
chr18	36155000	chr18	39535000	2.27E-02	super_enhancer	promoter	PIK3C3
chr18	36155000	chr18	54815000	5.83E-03	super_enhancer	promoter	CTD-2526M8.3
chr18	36185000	chr18	48745000	8.67E-03	super_enhancer	promoter	MEX3C
chr18	37645000	chr18	48015000	7.55E-03	promoter	super_enhancer	LINC01477
chr18	39535000	chr18	48005000	1.01E-02	promoter	super_enhancer	PIK3C3
chr18	43225000	chr18	47995000	1.79E-02	promoter	super_enhancer	RP11-116O18.1
chr18	44335000	chr18	47995000	2.70E-02	promoter	super_enhancer	RP11-742D12.2
chr18	45405000	chr18	46285000	9.55E-03	promoter	super_enhancer	RP11-380M21.4
chr18	46065000	chr18	48015000	1.86E-03	promoter	super_enhancer	CTIF
chr18	46295000	chr18	46975000	2.52E-02	super_enhancer	promoter	RP11-110H1.9
chr18	46295000	chr18	48555000	4.75E-02	super_enhancer	promoter	SMAD4
chr18	46305000	chr18	46975000	2.66E-03	super_enhancer	promoter	RP11-110H1.9
chr18	46305000	chr18	46995000	3.93E-03	super_enhancer	promoter	PRR13P4
chr18	46305000	chr18	47365000	1.98E-02	super_enhancer	promoter	RP11-886H22.1
chr18	46315000	chr18	46975000	2.23E-02	super_enhancer	promoter	RP11-110H1.9
chr18	46315000	chr18	47025000	5.31E-03	super_enhancer	promoter	RP11-110H1.8

chr18	46315000	chr18	48555000	4.38E-02	super_enhancer	promoter	SMAD4
chr18	46315000	chr18	67955000	6.99E-03	super_enhancer	promoter	SOCS6
chr18	46315000	chr18	74535000	3.70E-03	super_enhancer	promoter	RP11-162A12.2
chr18	46325000	chr18	46975000	4.18E-02	super_enhancer	promoter	RP11-110H1.9
chr18	46325000	chr18	47815000	1.40E-02	super_enhancer	promoter	CXXC1
chr18	46325000	chr18	57365000	1.09E-02	super_enhancer	promoter	CCBE1
chr18	46325000	chr18	77155000	5.77E-03	super_enhancer	promoter	NFATC1
chr18	47735000	chr18	47995000	7.17E-03	promoter	super_enhancer	RN7SL310P
chr18	47985000	chr18	74825000	6.44E-03	super_enhancer	promoter	RP11-4B16.4
chr18	47985000	chr18	77255000	4.49E-03	super_enhancer	promoter	RP11-196B3.2
chr18	47995000	chr18	57125000	9.74E-03	super_enhancer	promoter	RP11-27G24.3
chr18	48005000	chr18	48195000	1.68E-02	super_enhancer	promoter	RP11-10I6.2
chr18	48005000	chr18	48555000	3.49E-02	super_enhancer	promoter	SMAD4
chr18	48005000	chr18	53805000	1.41E-02	super_enhancer	promoter	LINC01539
chr18	48005000	chr18	61315000	5.54E-03	super_enhancer	promoter	SERPINB11
chr18	48005000	chr18	64805000	6.48E-03	super_enhancer	promoter	RPL31P9
chr18	48015000	chr18	57125000	8.61E-03	super_enhancer	promoter	RP11-27G24.3
chr18	48025000	chr18	77015000	3.55E-03	super_enhancer	promoter	RP11-1136J12.1
chr18	48025000	chr18	77335000	3.29E-03	super_enhancer	promoter	RP11-567M16.1
chr19	305000	chr19	885000	1.09E-02	promoter	super_enhancer	CTD-3113P16.5
chr19	305000	chr19	11145000	2.83E-02	promoter	super_enhancer	CTD-3113P16.5
chr19	305000	chr19	11155000	2.75E-02	promoter	super_enhancer	CTD-3113P16.5
chr19	305000	chr19	13795000	2.45E-02	promoter	super_enhancer	CTD-3113P16.5
chr19	305000	chr19	45725000	9.14E-03	promoter	super_enhancer	CTD-3113P16.5

chr19	305000	chr19	56095000	6.54E-03	promoter	super_enhancer	CTD-3113P16.5
chr19	345000	chr19	625000	2.95E-02	promoter	super_enhancer	MIER2
chr19	345000	chr19	1875000	4.88E-02	promoter	super_enhancer	MIER2
chr19	345000	chr19	18675000	2.67E-02	promoter	super_enhancer	MIER2
chr19	345000	chr19	47595000	9.19E-03	promoter	super_enhancer	MIER2
chr19	475000	chr19	565000	2.90E-02	promoter	super_enhancer	ODF3L2
chr19	475000	chr19	55535000	6.68E-03	promoter	super_enhancer	ODF3L2
chr19	555000	chr19	8455000	2.79E-02	super_enhancer	promoter	MIR4999
chr19	555000	chr19	8645000	3.71E-02	super_enhancer	promoter	AC130469.2
chr19	555000	chr19	17395000	3.09E-02	super_enhancer	promoter	USHBP1
chr19	555000	chr19	19145000	2.37E-02	super_enhancer	promoter	SUGP2
chr19	555000	chr19	41815000	1.03E-02	super_enhancer	promoter	CCDC97
chr19	555000	chr19	46235000	1.17E-02	super_enhancer	promoter	FBXO46
chr19	565000	chr19	7985000	3.44E-02	super_enhancer	promoter	CTD-3193O13.1
chr19	565000	chr19	13075000	2.21E-02	super_enhancer	promoter	DAND5
chr19	565000	chr19	16285000	2.07E-02	super_enhancer	promoter	CIB3
chr19	565000	chr19	36605000	9.87E-03	super_enhancer	promoter	TBCB
chr19	565000	chr19	52645000	3.75E-03	super_enhancer	promoter	CTC-471J1.9
chr19	565000	chr19	58995000	4.10E-03	super_enhancer	promoter	RNU6-1337P
chr19	575000	chr19	635000	4.91E-03	super_enhancer	promoter	POLRMT
chr19	575000	chr19	8215000	3.27E-02	super_enhancer	promoter	FBN3
chr19	575000	chr19	9195000	2.95E-02	super_enhancer	promoter	OR1M4P
chr19	575000	chr19	13055000	2.42E-02	super_enhancer	promoter	CTC-425F1.4
chr19	575000	chr19	38865000	9.85E-03	super_enhancer	promoter	PSMD8

chr19	575000	chr19	40025000	1.06E-02	super_enhancer	promoter	TDGF1P7
chr19	575000	chr19	58515000	6.86E-03	super_enhancer	promoter	ZNF606
chr19	615000	chr19	12255000	3.06E-02	super_enhancer	promoter	CTC-359D24.3
chr19	615000	chr19	12835000	4.06E-02	super_enhancer	promoter	TNPO2
chr19	615000	chr19	14965000	2.63E-02	super_enhancer	promoter	AC005255.5
chr19	615000	chr19	16015000	1.75E-02	super_enhancer	promoter	AC005336.5
chr19	615000	chr19	54695000	7.89E-03	super_enhancer	promoter	MBOAT7
chr19	625000	chr19	635000	3.48E-02	super_enhancer	promoter	POLRMT
chr19	625000	chr19	6435000	4.55E-02	super_enhancer	promoter	SLC25A41
chr19	625000	chr19	11545000	2.79E-02	super_enhancer	promoter	PRKCSH
chr19	625000	chr19	15495000	2.35E-02	super_enhancer	promoter	AC005785.5
chr19	625000	chr19	17395000	3.01E-02	super_enhancer	promoter	USHBP1
chr19	625000	chr19	36545000	1.07E-02	super_enhancer	promoter	WDR62
chr19	625000	chr19	36705000	8.91E-03	super_enhancer	promoter	ZNF146
chr19	625000	chr19	41905000	1.12E-02	super_enhancer	promoter	CTC-435M10.10
chr19	625000	chr19	49845000	6.88E-03	super_enhancer	promoter	CTC-301O7.4
chr19	635000	chr19	885000	3.16E-02	promoter	super_enhancer	POLRMT
chr19	635000	chr19	15315000	1.95E-02	promoter	super_enhancer	POLRMT
chr19	635000	chr19	19605000	1.77E-02	promoter	super_enhancer	POLRMT
chr19	665000	chr19	695000	4.49E-02	super_enhancer	promoter	PRSS57
chr19	665000	chr19	12395000	2.50E-02	super_enhancer	promoter	CTD-2666L21.2
chr19	665000	chr19	13045000	2.98E-02	super_enhancer	promoter	FARSA
chr19	665000	chr19	31045000	1.21E-02	super_enhancer	promoter	CTD-2001J20.1
chr19	665000	chr19	39305000	1.39E-02	super_enhancer	promoter	LGALS4

chr19	665000	chr19	42575000	9.61E-03	super_enhancer	promoter	GRIK5
chr19	665000	chr19	55995000	7.58E-03	super_enhancer	promoter	NAT14
chr19	675000	chr19	5675000	4.75E-02	super_enhancer	promoter	RPL36
chr19	675000	chr19	12075000	2.89E-02	super_enhancer	promoter	ZNF763
chr19	675000	chr19	15135000	2.71E-02	super_enhancer	promoter	SLC1A6
chr19	675000	chr19	29125000	1.06E-02	super_enhancer	promoter	AC005616.1
chr19	675000	chr19	46735000	1.01E-02	super_enhancer	promoter	AC006262.10
chr19	685000	chr19	9695000	2.31E-02	super_enhancer	promoter	ZNF121
chr19	685000	chr19	9795000	2.11E-02	super_enhancer	promoter	RPS4XP22
chr19	685000	chr19	42855000	7.42E-03	super_enhancer	promoter	MIR8077
chr19	685000	chr19	47355000	6.16E-03	super_enhancer	promoter	AP2S1
chr19	695000	chr19	11065000	3.74E-02	promoter	super_enhancer	PRSS57
chr19	695000	chr19	17095000	3.14E-02	promoter	super_enhancer	PRSS57
chr19	695000	chr19	17115000	3.63E-02	promoter	super_enhancer	PRSS57
chr19	695000	chr19	18005000	3.13E-02	promoter	super_enhancer	PRSS57
chr19	695000	chr19	19615000	2.11E-02	promoter	super_enhancer	PRSS57
chr19	695000	chr19	40825000	1.38E-02	promoter	super_enhancer	PRSS57
chr19	695000	chr19	43435000	7.08E-03	promoter	super_enhancer	PRSS57
chr19	785000	chr19	825000	3.26E-02	super_enhancer	promoter	AZU1
chr19	785000	chr19	925000	4.20E-03	super_enhancer	promoter	ARID3A
chr19	785000	chr19	7585000	4.80E-02	super_enhancer	promoter	CTD-2207O23.11
chr19	785000	chr19	12075000	3.24E-02	super_enhancer	promoter	ZNF763
chr19	785000	chr19	12755000	4.08E-02	super_enhancer	promoter	CTD-2192J16.15
chr19	785000	chr19	49375000	7.58E-03	super_enhancer	promoter	PPP1R15A

chr19	805000	chr19	11165000	3.45E-02	promoter	super_enhancer	MIR4745
chr19	805000	chr19	17795000	2.96E-02	promoter	super_enhancer	MIR4745
chr19	805000	chr19	41235000	1.48E-02	promoter	super_enhancer	MIR4745
chr19	805000	chr19	58555000	1.00E-02	promoter	super_enhancer	MIR4745
chr19	825000	chr19	11145000	1.02E-03	promoter	super_enhancer	AZU1
chr19	825000	chr19	43765000	9.57E-03	promoter	super_enhancer	AZU1
chr19	825000	chr19	45785000	1.53E-02	promoter	super_enhancer	AZU1
chr19	825000	chr19	48325000	9.05E-03	promoter	super_enhancer	AZU1
chr19	875000	chr19	1275000	1.94E-02	super_enhancer	promoter	C19orf24
chr19	875000	chr19	6335000	3.61E-02	super_enhancer	promoter	ACER1
chr19	875000	chr19	7585000	5.48E-04	super_enhancer	promoter	CTD-2207O23.11
chr19	875000	chr19	11545000	2.14E-02	super_enhancer	promoter	PRKCSH
chr19	875000	chr19	12985000	1.93E-02	super_enhancer	promoter	HOOK2
chr19	875000	chr19	13735000	1.91E-02	super_enhancer	promoter	CACNA1A
chr19	875000	chr19	15135000	2.06E-02	super_enhancer	promoter	SLC1A6
chr19	875000	chr19	18385000	1.80E-02	super_enhancer	promoter	KIAA1683
chr19	875000	chr19	52615000	2.78E-03	super_enhancer	promoter	CTC-471J1.10
chr19	885000	chr19	1095000	4.90E-02	super_enhancer	promoter	POLR2E
chr19	885000	chr19	1815000	8.03E-03	super_enhancer	promoter	CTB-31O20.3
chr19	885000	chr19	7815000	3.48E-02	super_enhancer	promoter	RPL21P129
chr19	885000	chr19	12035000	2.31E-02	super_enhancer	promoter	ZNF700
chr19	885000	chr19	13735000	2.52E-02	super_enhancer	promoter	CACNA1A
chr19	885000	chr19	39615000	1.05E-02	super_enhancer	promoter	PAK4
chr19	885000	chr19	45445000	1.01E-02	super_enhancer	promoter	APOC4

chr19	885000	chr19	58735000	5.95E-03	super_enhancer	promoter	AC020915.2
chr19	895000	chr19	1065000	4.89E-02	super_enhancer	promoter	HMHA1
chr19	895000	chr19	7445000	3.29E-02	super_enhancer	promoter	CTD-2207O23.3
chr19	895000	chr19	11545000	2.06E-02	super_enhancer	promoter	PRKCSH
chr19	895000	chr19	15495000	1.51E-04	super_enhancer	promoter	AC005785.5
chr19	895000	chr19	16125000	2.35E-02	super_enhancer	promoter	LINC00661
chr19	895000	chr19	36525000	9.59E-03	super_enhancer	promoter	CLIP3
chr19	895000	chr19	40075000	7.20E-03	super_enhancer	promoter	CTB-60E11.12
chr19	895000	chr19	54205000	5.05E-03	super_enhancer	promoter	MIR518B
chr19	895000	chr19	58175000	4.26E-03	super_enhancer	promoter	AC003682.17
chr19	905000	chr19	925000	4.83E-02	super_enhancer	promoter	ARID3A
chr19	905000	chr19	10655000	4.23E-02	super_enhancer	promoter	ATG4D
chr19	905000	chr19	10765000	3.47E-02	super_enhancer	promoter	ILF3-AS1
chr19	905000	chr19	12985000	3.09E-02	super_enhancer	promoter	HOOK2
chr19	905000	chr19	16215000	2.87E-02	super_enhancer	promoter	CTD-2231E14.5
chr19	905000	chr19	17445000	3.63E-02	super_enhancer	promoter	GTPBP3
chr19	905000	chr19	19175000	2.86E-02	super_enhancer	promoter	SLC25A42
chr19	905000	chr19	39305000	1.52E-02	super_enhancer	promoter	LGALS4
chr19	925000	chr19	1175000	3.43E-02	promoter	super_enhancer	ARID3A
chr19	925000	chr19	1185000	5.70E-04	promoter	super_enhancer	ARID3A
chr19	925000	chr19	14395000	3.06E-02	promoter	super_enhancer	ARID3A
chr19	925000	chr19	18005000	3.39E-02	promoter	super_enhancer	ARID3A
chr19	925000	chr19	45555000	1.35E-02	promoter	super_enhancer	ARID3A
chr19	925000	chr19	46865000	9.95E-03	promoter	super_enhancer	ARID3A

chr19	925000	chr19	56095000	1.06E-02	promoter	super_enhancer	ARID3A
chr19	925000	chr19	56105000	1.05E-02	promoter	super_enhancer	ARID3A
chr19	955000	chr19	19615000	1.90E-02	promoter	super_enhancer	LLNLR-260G6.1
chr19	955000	chr19	43545000	5.10E-03	promoter	super_enhancer	LLNLR-260G6.1
chr19	955000	chr19	45425000	1.08E-02	promoter	super_enhancer	LLNLR-260G6.1
chr19	955000	chr19	47605000	7.33E-03	promoter	super_enhancer	LLNLR-260G6.1
chr19	1065000	chr19	5165000	4.48E-02	promoter	super_enhancer	HMHA1
chr19	1065000	chr19	18005000	1.91E-02	promoter	super_enhancer	HMHA1
chr19	1065000	chr19	40575000	6.94E-03	promoter	super_enhancer	HMHA1
chr19	1065000	chr19	41205000	8.83E-03	promoter	super_enhancer	HMHA1
chr19	1065000	chr19	41245000	8.72E-03	promoter	super_enhancer	HMHA1
chr19	1065000	chr19	43195000	7.97E-03	promoter	super_enhancer	HMHA1
chr19	1065000	chr19	45555000	7.57E-03	promoter	super_enhancer	HMHA1
chr19	1095000	chr19	1185000	9.10E-04	promoter	super_enhancer	POLR2E
chr19	1095000	chr19	1255000	2.65E-02	promoter	super_enhancer	POLR2E
chr19	1095000	chr19	36135000	1.42E-02	promoter	super_enhancer	POLR2E
chr19	1095000	chr19	40785000	1.19E-02	promoter	super_enhancer	POLR2E
chr19	1095000	chr19	44755000	8.66E-03	promoter	super_enhancer	POLR2E
chr19	1095000	chr19	48575000	6.34E-03	promoter	super_enhancer	POLR2E
chr19	1095000	chr19	56105000	9.26E-03	promoter	super_enhancer	POLR2E
chr19	1175000	chr19	3585000	1.14E-02	super_enhancer	promoter	GIPC3
chr19	1175000	chr19	11435000	2.79E-02	super_enhancer	promoter	CTC-510F12.4
chr19	1175000	chr19	11495000	2.79E-02	super_enhancer	promoter	EPOR
chr19	1175000	chr19	11695000	2.79E-02	super_enhancer	promoter	GAPDHP76

chr19	1175000	chr19	13105000	1.95E-02	super_enhancer	promoter	NFIX
chr19	1175000	chr19	16245000	2.47E-02	super_enhancer	promoter	HSH2D
chr19	1175000	chr19	19315000	1.91E-02	super_enhancer	promoter	NR2C2AP
chr19	1175000	chr19	42805000	9.28E-03	super_enhancer	promoter	PRR19
chr19	1185000	chr19	2015000	4.68E-02	super_enhancer	promoter	LLNLR-304A6.2
chr19	1185000	chr19	9435000	3.34E-02	super_enhancer	promoter	ZNF559-ZNF177
chr19	1185000	chr19	11485000	3.31E-02	super_enhancer	promoter	SWSAP1
chr19	1185000	chr19	35645000	1.24E-02	super_enhancer	promoter	FXYD5
chr19	1185000	chr19	40725000	1.24E-02	super_enhancer	promoter	TTC9B
chr19	1255000	chr19	7745000	4.04E-02	super_enhancer	promoter	TRAPPC5
chr19	1255000	chr19	13075000	2.74E-02	super_enhancer	promoter	DAND5
chr19	1255000	chr19	32835000	9.48E-03	super_enhancer	promoter	ZNF507
chr19	1255000	chr19	39615000	9.85E-03	super_enhancer	promoter	PAK4
chr19	1255000	chr19	42105000	9.88E-03	super_enhancer	promoter	CEACAMP3
chr19	1255000	chr19	44575000	8.93E-03	super_enhancer	promoter	ZNF284
chr19	1255000	chr19	45005000	8.75E-03	super_enhancer	promoter	ZNF180
chr19	1255000	chr19	47975000	6.80E-03	super_enhancer	promoter	SLC8A2
chr19	1255000	chr19	49055000	5.53E-03	super_enhancer	promoter	SULT2B1
chr19	1255000	chr19	50365000	6.27E-03	super_enhancer	promoter	AC018766.4
chr19	1265000	chr19	8645000	4.43E-02	super_enhancer	promoter	AC130469.2
chr19	1265000	chr19	11455000	3.19E-02	super_enhancer	promoter	CCDC159
chr19	1265000	chr19	12625000	3.65E-02	super_enhancer	promoter	ZNF709
chr19	1265000	chr19	15255000	2.72E-02	super_enhancer	promoter	RNU6-782P
chr19	1265000	chr19	41905000	1.29E-02	super_enhancer	promoter	CTC-435M10.10

chr19	1265000	chr19	50095000	5.71E-03	super_enhancer	promoter	NOSIP
chr19	1265000	chr19	50295000	6.94E-03	super_enhancer	promoter	MIR6799
chr19	1265000	chr19	54665000	9.02E-03	super_enhancer	promoter	LENG1
chr19	1265000	chr19	55825000	7.15E-03	super_enhancer	promoter	CTD-2105E13.14
chr19	1275000	chr19	6535000	4.82E-02	promoter	super_enhancer	C19orf24
chr19	1275000	chr19	11115000	2.77E-02	promoter	super_enhancer	C19orf24
chr19	1275000	chr19	19375000	2.26E-02	promoter	super_enhancer	C19orf24
chr19	1275000	chr19	45575000	9.83E-03	promoter	super_enhancer	C19orf24
chr19	1285000	chr19	2035000	4.10E-02	promoter	super_enhancer	MUM1
chr19	1285000	chr19	3135000	3.77E-02	promoter	super_enhancer	MUM1
chr19	1285000	chr19	3435000	1.75E-02	promoter	super_enhancer	MUM1
chr19	1285000	chr19	17775000	2.61E-02	promoter	super_enhancer	MUM1
chr19	1285000	chr19	17785000	3.79E-04	promoter	super_enhancer	MUM1
chr19	1285000	chr19	18465000	2.69E-02	promoter	super_enhancer	MUM1
chr19	1285000	chr19	38705000	9.88E-03	promoter	super_enhancer	MUM1
chr19	1375000	chr19	7395000	2.08E-02	promoter	super_enhancer	AC004623.3
chr19	1375000	chr19	14385000	2.86E-02	promoter	super_enhancer	AC004623.3
chr19	1375000	chr19	16355000	3.13E-02	promoter	super_enhancer	AC004623.3
chr19	1375000	chr19	18455000	3.12E-02	promoter	super_enhancer	AC004623.3
chr19	1375000	chr19	18485000	2.94E-02	promoter	super_enhancer	AC004623.3
chr19	1375000	chr19	18495000	3.00E-02	promoter	super_enhancer	AC004623.3
chr19	1375000	chr19	46865000	8.52E-03	promoter	super_enhancer	AC004623.3
chr19	1375000	chr19	46895000	8.78E-03	promoter	super_enhancer	AC004623.3
chr19	1375000	chr19	48585000	8.29E-03	promoter	super_enhancer	AC004623.3

chr19	1445000	chr19	6535000	4.62E-02	promoter	super_enhancer	APC2
chr19	1445000	chr19	47605000	5.86E-03	promoter	super_enhancer	APC2
chr19	1515000	chr19	15315000	2.73E-02	promoter	super_enhancer	ADAMTSL5
chr19	1515000	chr19	40425000	1.43E-02	promoter	super_enhancer	ADAMTSL5
chr19	1515000	chr19	44745000	9.32E-03	promoter	super_enhancer	ADAMTSL5
chr19	1515000	chr19	46205000	1.37E-02	promoter	super_enhancer	ADAMTSL5
chr19	1555000	chr19	1845000	3.58E-02	promoter	super_enhancer	AC027307.2
chr19	1555000	chr19	2035000	2.09E-03	promoter	super_enhancer	AC027307.2
chr19	1555000	chr19	13805000	3.58E-02	promoter	super_enhancer	AC027307.2
chr19	1555000	chr19	38655000	1.36E-02	promoter	super_enhancer	AC027307.2
chr19	1555000	chr19	41215000	1.47E-02	promoter	super_enhancer	AC027307.2
chr19	1555000	chr19	47655000	7.77E-03	promoter	super_enhancer	AC027307.2
chr19	1605000	chr19	1745000	4.02E-02	promoter	super_enhancer	AC005943.2
chr19	1605000	chr19	13835000	3.35E-02	promoter	super_enhancer	AC005943.2
chr19	1605000	chr19	16605000	3.32E-02	promoter	super_enhancer	AC005943.2
chr19	1605000	chr19	18415000	3.48E-02	promoter	super_enhancer	AC005943.2
chr19	1605000	chr19	40805000	1.38E-02	promoter	super_enhancer	AC005943.2
chr19	1745000	chr19	11435000	4.11E-02	super_enhancer	promoter	CTC-510F12.4
chr19	1745000	chr19	11495000	4.11E-02	super_enhancer	promoter	EPOR
chr19	1745000	chr19	16955000	3.59E-02	super_enhancer	promoter	CTD-2538G9.5
chr19	1745000	chr19	39755000	1.59E-02	super_enhancer	promoter	IFNL4P1
chr19	1745000	chr19	46235000	1.51E-02	super_enhancer	promoter	FBXO46
chr19	1745000	chr19	59025000	9.95E-03	super_enhancer	promoter	SLC27A5
chr19	1815000	chr19	3365000	3.35E-02	promoter	super_enhancer	CTB-31O20.3

chr19	1815000	chr19	15275000	3.34E-02	promoter	super_enhancer	CTB-31O20.3
chr19	1815000	chr19	17095000	3.42E-02	promoter	super_enhancer	CTB-31O20.3
chr19	1815000	chr19	18685000	3.22E-02	promoter	super_enhancer	CTB-31O20.3
chr19	1815000	chr19	39055000	1.47E-02	promoter	super_enhancer	CTB-31O20.3
chr19	1845000	chr19	13055000	3.24E-02	super_enhancer	promoter	CTC-425F1.4
chr19	1845000	chr19	16465000	3.07E-02	super_enhancer	promoter	CTD-2013N17.7
chr19	1845000	chr19	16955000	3.24E-02	super_enhancer	promoter	CTD-2538G9.5
chr19	1845000	chr19	18385000	2.87E-02	super_enhancer	promoter	KIAA1683
chr19	1845000	chr19	56825000	7.53E-03	super_enhancer	promoter	AC006116.19
chr19	1845000	chr19	57155000	7.19E-03	super_enhancer	promoter	SMIM17
chr19	1855000	chr19	8645000	4.25E-02	super_enhancer	promoter	AC130469.2
chr19	1855000	chr19	17665000	2.31E-02	super_enhancer	promoter	COLGALT1
chr19	1855000	chr19	39755000	1.25E-02	super_enhancer	promoter	IFNL4P1
chr19	1855000	chr19	40855000	1.06E-02	super_enhancer	promoter	C19orf47
chr19	1855000	chr19	49055000	6.10E-03	super_enhancer	promoter	SULT2B1
chr19	1855000	chr19	49315000	6.75E-03	super_enhancer	promoter	BCAT2
chr19	1855000	chr19	49865000	6.95E-03	super_enhancer	promoter	DKKL1
chr19	1855000	chr19	49955000	6.65E-03	super_enhancer	promoter	ALDH16A1
chr19	1855000	chr19	54975000	5.73E-03	super_enhancer	promoter	LENG9
chr19	1865000	chr19	2435000	2.48E-02	super_enhancer	promoter	MIR7108
chr19	1865000	chr19	11695000	5.94E-04	super_enhancer	promoter	GAPDHP76
chr19	1865000	chr19	19845000	1.86E-02	super_enhancer	promoter	ZNF14
chr19	1865000	chr19	35935000	1.01E-02	super_enhancer	promoter	FFAR2
chr19	1865000	chr19	50975000	6.99E-03	super_enhancer	promoter	Clostridiales-1

chr19	1875000	chr19	13055000	2.65E-02	super_enhancer	promoter	CTC-425F1.4
chr19	1875000	chr19	17565000	2.68E-02	super_enhancer	promoter	CTD-2521M24.11
chr19	1875000	chr19	45505000	1.05E-02	super_enhancer	promoter	RELB
chr19	1875000	chr19	54135000	7.14E-03	super_enhancer	promoter	DPRX
chr19	1905000	chr19	2575000	3.25E-02	promoter	super_enhancer	SCAMP4
chr19	1955000	chr19	2085000	1.29E-03	promoter	super_enhancer	CSNK1G2-AS1
chr19	1955000	chr19	13165000	3.05E-02	promoter	super_enhancer	CSNK1G2-AS1
chr19	1955000	chr19	17115000	3.25E-02	promoter	super_enhancer	CSNK1G2-AS1
chr19	2005000	chr19	2075000	5.52E-05	promoter	super_enhancer	LLNLR-304A6.1
chr19	2005000	chr19	11095000	3.81E-02	promoter	super_enhancer	LLNLR-304A6.1
chr19	2005000	chr19	18365000	3.04E-02	promoter	super_enhancer	LLNLR-304A6.1
chr19	2005000	chr19	40425000	1.36E-02	promoter	super_enhancer	LLNLR-304A6.1
chr19	2015000	chr19	2065000	5.76E-04	promoter	super_enhancer	LLNLR-304A6.2
chr19	2015000	chr19	2075000	1.06E-03	promoter	super_enhancer	LLNLR-304A6.2
chr19	2015000	chr19	2085000	2.46E-02	promoter	super_enhancer	LLNLR-304A6.2
chr19	2015000	chr19	2175000	9.19E-04	promoter	super_enhancer	LLNLR-304A6.2
chr19	2025000	chr19	2235000	4.96E-02	super_enhancer	promoter	MIR1227
chr19	2025000	chr19	2435000	2.52E-02	super_enhancer	promoter	MIR7108
chr19	2025000	chr19	9945000	3.89E-02	super_enhancer	promoter	PIN1
chr19	2025000	chr19	17445000	3.74E-02	super_enhancer	promoter	GTPBP3
chr19	2025000	chr19	19145000	2.91E-02	super_enhancer	promoter	SUGP2
chr19	2035000	chr19	2435000	1.02E-02	super_enhancer	promoter	MIR7108
chr19	2035000	chr19	6425000	4.96E-02	super_enhancer	promoter	KHSRP
chr19	2035000	chr19	16435000	2.75E-02	super_enhancer	promoter	CTD-2562J15.6

chr19	2035000	chr19	17395000	2.95E-02	super_enhancer	promoter	USHBP1
chr19	2035000	chr19	55565000	6.38E-03	super_enhancer	promoter	CTC-550B14.6
chr19	2045000	chr19	36175000	9.12E-03	super_enhancer	promoter	AC002314.4
chr19	2045000	chr19	42055000	7.90E-03	super_enhancer	promoter	CEACAM21
chr19	2045000	chr19	42415000	8.52E-03	super_enhancer	promoter	CTD-2575K13.6
chr19	2055000	chr19	12175000	3.03E-02	super_enhancer	promoter	ZNF844
chr19	2055000	chr19	37995000	9.39E-03	super_enhancer	promoter	CTD-3064H18.2
chr19	2055000	chr19	39305000	1.23E-02	super_enhancer	promoter	LGALS4
chr19	2055000	chr19	40725000	1.13E-02	super_enhancer	promoter	TTC9B
chr19	2055000	chr19	46145000	7.86E-03	super_enhancer	promoter	EML2-AS1
chr19	2055000	chr19	48775000	6.96E-03	super_enhancer	promoter	CTC-241F20.4
chr19	2065000	chr19	10515000	3.06E-02	super_enhancer	promoter	MIR1181
chr19	2065000	chr19	10765000	3.67E-02	super_enhancer	promoter	ILF3-AS1
chr19	2065000	chr19	14695000	3.47E-02	super_enhancer	promoter	RN7SL337P
chr19	2065000	chr19	15345000	2.54E-02	super_enhancer	promoter	EPHX3
chr19	2065000	chr19	19145000	2.92E-02	super_enhancer	promoter	SUGP2
chr19	2065000	chr19	40725000	1.42E-02	super_enhancer	promoter	TTC9B
chr19	2065000	chr19	50335000	7.90E-03	super_enhancer	promoter	MIR6800
chr19	2075000	chr19	11435000	4.17E-02	super_enhancer	promoter	CTC-510F12.4
chr19	2075000	chr19	13075000	3.92E-02	super_enhancer	promoter	DAND5
chr19	2075000	chr19	13125000	3.52E-02	super_enhancer	promoter	CTC-239J10.1
chr19	2075000	chr19	21565000	9.33E-03	super_enhancer	promoter	CTD-2561J22.1
chr19	2075000	chr19	39185000	1.27E-02	super_enhancer	promoter	CTB-186G2.4
chr19	2075000	chr19	42465000	1.49E-02	super_enhancer	promoter	RABAC1

chr19	2075000	chr19	46235000	1.51E-02	super_enhancer	promoter	FBXO46
chr19	2075000	chr19	50005000	1.01E-02	super_enhancer	promoter	MIR150
chr19	2085000	chr19	2785000	4.69E-02	super_enhancer	promoter	THOP1
chr19	2085000	chr19	11495000	4.38E-02	super_enhancer	promoter	EPOR
chr19	2085000	chr19	42055000	1.31E-02	super_enhancer	promoter	CEACAM21
chr19	2085000	chr19	55215000	1.13E-02	super_enhancer	promoter	AC098784.1
chr19	2095000	chr19	2165000	2.00E-02	promoter	super_enhancer	IZUMO4
chr19	2095000	chr19	15315000	2.46E-02	promoter	super_enhancer	IZUMO4
chr19	2095000	chr19	17075000	2.23E-02	promoter	super_enhancer	IZUMO4
chr19	2095000	chr19	17765000	2.78E-02	promoter	super_enhancer	IZUMO4
chr19	2095000	chr19	41255000	1.27E-02	promoter	super_enhancer	IZUMO4
chr19	2095000	chr19	48875000	7.31E-03	promoter	super_enhancer	IZUMO4
chr19	2155000	chr19	8455000	3.73E-02	super_enhancer	promoter	MIR4999
chr19	2155000	chr19	14965000	2.70E-02	super_enhancer	promoter	AC005255.5
chr19	2155000	chr19	17845000	3.44E-02	super_enhancer	promoter	CTD-3149D2.4
chr19	2155000	chr19	19545000	2.07E-02	super_enhancer	promoter	MIR640
chr19	2155000	chr19	59085000	7.62E-03	super_enhancer	promoter	MZF1
chr19	2165000	chr19	2435000	1.87E-03	super_enhancer	promoter	MIR7108
chr19	2165000	chr19	13905000	2.40E-02	super_enhancer	promoter	ZSWIM4
chr19	2165000	chr19	17905000	2.71E-02	super_enhancer	promoter	B3GNT3
chr19	2165000	chr19	18655000	2.29E-02	super_enhancer	promoter	FKBP8
chr19	2165000	chr19	42575000	8.17E-03	super_enhancer	promoter	GRIK5
chr19	2165000	chr19	46105000	9.42E-03	super_enhancer	promoter	OPA3
chr19	2165000	chr19	54485000	7.42E-03	super_enhancer	promoter	MIR935

chr19	2165000	chr19	55825000	5.84E-03	super_enhancer	promoter	CTD-2105E13.14
chr19	2175000	chr19	12985000	3.30E-02	super_enhancer	promoter	HOOK2
chr19	2175000	chr19	38875000	1.24E-02	super_enhancer	promoter	AC005789.9
chr19	2175000	chr19	52345000	6.76E-03	super_enhancer	promoter	AC006272.2
chr19	2175000	chr19	54485000	9.95E-03	super_enhancer	promoter	MIR935
chr19	2235000	chr19	2505000	1.51E-02	promoter	super_enhancer	MIR1227
chr19	2235000	chr19	11055000	3.05E-02	promoter	super_enhancer	MIR1227
chr19	2235000	chr19	16575000	2.84E-02	promoter	super_enhancer	MIR1227
chr19	2235000	chr19	18665000	2.73E-02	promoter	super_enhancer	MIR1227
chr19	2235000	chr19	41215000	1.29E-02	promoter	super_enhancer	MIR1227
chr19	2435000	chr19	17105000	3.05E-02	promoter	super_enhancer	MIR7108
chr19	2435000	chr19	17795000	2.57E-02	promoter	super_enhancer	MIR7108
chr19	2475000	chr19	2525000	2.56E-02	promoter	super_enhancer	GADD45B
chr19	2475000	chr19	2545000	3.25E-03	promoter	super_enhancer	GADD45B
chr19	2475000	chr19	2555000	1.43E-03	promoter	super_enhancer	GADD45B
chr19	2475000	chr19	2565000	1.52E-04	promoter	super_enhancer	GADD45B
chr19	2475000	chr19	2575000	2.23E-04	promoter	super_enhancer	GADD45B
chr19	2475000	chr19	3145000	2.27E-02	promoter	super_enhancer	GADD45B
chr19	2475000	chr19	11035000	3.75E-02	promoter	super_enhancer	GADD45B
chr19	2475000	chr19	11055000	3.47E-02	promoter	super_enhancer	GADD45B
chr19	2475000	chr19	11075000	3.39E-02	promoter	super_enhancer	GADD45B
chr19	2475000	chr19	13805000	3.60E-02	promoter	super_enhancer	GADD45B
chr19	2475000	chr19	17115000	3.52E-02	promoter	super_enhancer	GADD45B
chr19	2475000	chr19	18415000	3.25E-02	promoter	super_enhancer	GADD45B

chr19	2475000	chr19	18475000	3.14E-02	promoter	super_enhancer	GADD45B
chr19	2475000	chr19	19605000	2.42E-02	promoter	super_enhancer	GADD45B
chr19	2505000	chr19	13985000	4.59E-02	super_enhancer	promoter	MIR181C
chr19	2505000	chr19	19545000	2.55E-02	super_enhancer	promoter	MIR640
chr19	2505000	chr19	28285000	1.20E-02	super_enhancer	promoter	LINC00662
chr19	2505000	chr19	35225000	1.34E-02	super_enhancer	promoter	CTD-2553L13.10
chr19	2505000	chr19	44145000	1.65E-02	super_enhancer	promoter	CADM4
chr19	2505000	chr19	46105000	1.41E-02	super_enhancer	promoter	OPA3
chr19	2505000	chr19	46155000	1.44E-02	super_enhancer	promoter	RN7SL836P
chr19	2505000	chr19	48775000	1.01E-02	super_enhancer	promoter	CTC-241F20.4
chr19	2505000	chr19	55875000	1.02E-02	super_enhancer	promoter	FAM71E2
chr19	2515000	chr19	5915000	6.66E-03	super_enhancer	promoter	AC104532.4
chr19	2515000	chr19	11465000	4.30E-02	super_enhancer	promoter	LPPR2
chr19	2515000	chr19	12035000	3.39E-02	super_enhancer	promoter	ZNF700
chr19	2515000	chr19	13905000	3.60E-02	super_enhancer	promoter	ZSWIM4
chr19	2515000	chr19	15905000	3.87E-02	super_enhancer	promoter	OR10H5
chr19	2515000	chr19	15935000	3.04E-02	super_enhancer	promoter	ZNF861P
chr19	2515000	chr19	34745000	1.64E-02	super_enhancer	promoter	KIAA0355
chr19	2515000	chr19	35455000	1.38E-02	super_enhancer	promoter	ZNF792
chr19	2515000	chr19	42465000	1.58E-02	super_enhancer	promoter	RABAC1
chr19	2515000	chr19	45035000	1.52E-02	super_enhancer	promoter	CEACAM20
chr19	2515000	chr19	46145000	1.13E-02	super_enhancer	promoter	EML2-AS1
chr19	2515000	chr19	52645000	6.87E-03	super_enhancer	promoter	CTC-471J1.9
chr19	2525000	chr19	9195000	4.81E-02	super_enhancer	promoter	OR1M4P

chr19	2525000	chr19	13885000	3.46E-02	super_enhancer	promoter	CTB-5E10.3
chr19	2525000	chr19	17325000	3.93E-02	super_enhancer	promoter	USE1
chr19	2525000	chr19	17905000	3.65E-02	super_enhancer	promoter	B3GNT3
chr19	2525000	chr19	34895000	1.53E-02	super_enhancer	promoter	PDCD2L
chr19	2525000	chr19	39305000	1.59E-02	super_enhancer	promoter	LGALS4
chr19	2525000	chr19	43905000	1.01E-02	super_enhancer	promoter	TEX101
chr19	2535000	chr19	16295000	2.90E-02	super_enhancer	promoter	FAM32A
chr19	2535000	chr19	37095000	8.75E-03	super_enhancer	promoter	ZNF382
chr19	2535000	chr19	50335000	6.75E-03	super_enhancer	promoter	MIR6800
chr19	2535000	chr19	59025000	3.12E-05	super_enhancer	promoter	SLC27A5
chr19	2545000	chr19	10615000	2.23E-02	super_enhancer	promoter	KEAP1
chr19	2545000	chr19	14695000	3.38E-02	super_enhancer	promoter	RN7SL337P
chr19	2545000	chr19	16125000	3.93E-02	super_enhancer	promoter	LINC00661
chr19	2545000	chr19	43775000	7.69E-03	super_enhancer	promoter	PSG9
chr19	2545000	chr19	45075000	1.18E-02	super_enhancer	promoter	AC138472.4
chr19	2545000	chr19	45875000	1.30E-02	super_enhancer	promoter	ERCC2
chr19	2545000	chr19	51415000	7.34E-03	super_enhancer	promoter	KLK4
chr19	2545000	chr19	55995000	8.04E-03	super_enhancer	promoter	NAT14
chr19	2555000	chr19	2945000	2.41E-02	super_enhancer	promoter	ZNF77
chr19	2555000	chr19	9885000	3.92E-02	super_enhancer	promoter	UBE2L4
chr19	2555000	chr19	12755000	4.42E-02	super_enhancer	promoter	CTD-2192J16.15
chr19	2555000	chr19	17845000	3.50E-02	super_enhancer	promoter	CTD-3149D2.4
chr19	2555000	chr19	41725000	1.43E-02	super_enhancer	promoter	AXL
chr19	2555000	chr19	50555000	6.07E-03	super_enhancer	promoter	CTD-2126E3.3

chr19	2565000	chr19	9215000	4.99E-02	super_enhancer	promoter	OR7G2
chr19	2565000	chr19	12165000	3.44E-02	super_enhancer	promoter	CTD-2006C1.10
chr19	2565000	chr19	13055000	3.45E-02	super_enhancer	promoter	CTC-425F1.4
chr19	2565000	chr19	35135000	1.36E-02	super_enhancer	promoter	CTD-2553L13.5
chr19	2565000	chr19	49375000	8.43E-03	super_enhancer	promoter	PPP1R15A
chr19	2575000	chr19	13045000	3.43E-02	super_enhancer	promoter	FARSA
chr19	2575000	chr19	13265000	2.45E-02	super_enhancer	promoter	CTC-250I14.6
chr19	2575000	chr19	15005000	2.80E-02	super_enhancer	promoter	OR7A1P
chr19	2575000	chr19	57125000	6.38E-03	super_enhancer	promoter	AC007228.8
chr19	2595000	chr19	41215000	1.15E-02	promoter	super_enhancer	RN7SL121P
chr19	2645000	chr19	4705000	2.79E-02	promoter	super_enhancer	CTC-265F19.1
chr19	2645000	chr19	45555000	1.40E-02	promoter	super_enhancer	CTC-265F19.1
chr19	2645000	chr19	45565000	1.66E-02	promoter	super_enhancer	CTC-265F19.1
chr19	2785000	chr19	11135000	3.87E-02	promoter	super_enhancer	THOP1
chr19	2785000	chr19	16585000	3.87E-02	promoter	super_enhancer	THOP1
chr19	2785000	chr19	17095000	3.29E-02	promoter	super_enhancer	THOP1
chr19	2945000	chr19	3135000	3.55E-02	promoter	super_enhancer	ZNF77
chr19	2945000	chr19	3445000	4.54E-02	promoter	super_enhancer	ZNF77
chr19	3135000	chr19	11545000	4.18E-02	super_enhancer	promoter	PRKCSH
chr19	3135000	chr19	13055000	3.84E-02	super_enhancer	promoter	CTC-425F1.4
chr19	3135000	chr19	13875000	3.88E-02	super_enhancer	promoter	MRI1
chr19	3135000	chr19	43065000	1.77E-02	super_enhancer	promoter	CEACAM1
chr19	3135000	chr19	47835000	9.53E-03	super_enhancer	promoter	C5AR2
chr19	3135000	chr19	58175000	7.98E-03	super_enhancer	promoter	AC003682.17

chr19	3145000	chr19	12645000	3.36E-02	super_enhancer	promoter	CTD-2192J16.21
chr19	3145000	chr19	36045000	1.16E-02	super_enhancer	promoter	AC002389.1
chr19	3155000	chr19	12945000	3.90E-02	super_enhancer	promoter	MAST1
chr19	3155000	chr19	13005000	3.30E-02	super_enhancer	promoter	AD000092.3
chr19	3155000	chr19	19045000	3.19E-02	super_enhancer	promoter	AC002985.3
chr19	3155000	chr19	56125000	8.41E-03	super_enhancer	promoter	CTD-2537I9.18
chr19	3165000	chr19	3185000	4.84E-02	super_enhancer	promoter	NCLN
chr19	3165000	chr19	11455000	3.56E-02	super_enhancer	promoter	CCDC159
chr19	3165000	chr19	15775000	2.17E-02	super_enhancer	promoter	CYP4F10P
chr19	3165000	chr19	50365000	7.91E-03	super_enhancer	promoter	AC018766.4
chr19	3175000	chr19	4305000	4.96E-02	super_enhancer	promoter	FSD1
chr19	3175000	chr19	4445000	3.70E-02	super_enhancer	promoter	MIR4746
chr19	3175000	chr19	7895000	4.37E-02	super_enhancer	promoter	EVI5L
chr19	3175000	chr19	42725000	6.97E-03	super_enhancer	promoter	DEDD2
chr19	3185000	chr19	11025000	3.86E-02	promoter	super_enhancer	NCLN
chr19	3185000	chr19	43195000	1.11E-02	promoter	super_enhancer	NCLN
chr19	3185000	chr19	45785000	1.22E-02	promoter	super_enhancer	NCLN
chr19	3185000	chr19	46195000	1.01E-02	promoter	super_enhancer	NCLN
chr19	3225000	chr19	9075000	3.55E-02	promoter	super_enhancer	CELF5
chr19	3225000	chr19	13155000	2.67E-02	promoter	super_enhancer	CELF5
chr19	3365000	chr19	6395000	3.64E-03	super_enhancer	promoter	GTF2F1
chr19	3365000	chr19	11545000	2.51E-02	super_enhancer	promoter	PRKCSH
chr19	3375000	chr19	9885000	4.25E-02	super_enhancer	promoter	UBE2L4
chr19	3375000	chr19	10655000	4.66E-02	super_enhancer	promoter	ATG4D

chr19	3375000	chr19	11495000	3.88E-02	super_enhancer	promoter	EPOR
chr19	3375000	chr19	54205000	7.97E-03	super_enhancer	promoter	MIR518B
chr19	3375000	chr19	54495000	9.70E-03	super_enhancer	promoter	CACNG6
chr19	3375000	chr19	59025000	8.64E-03	super_enhancer	promoter	SLC27A5
chr19	3385000	chr19	14405000	2.58E-02	super_enhancer	promoter	CTD-2189E23.1
chr19	3385000	chr19	36425000	1.34E-02	super_enhancer	promoter	LRFN3
chr19	3385000	chr19	49865000	7.27E-03	super_enhancer	promoter	DKKL1
chr19	3385000	chr19	50005000	8.29E-03	super_enhancer	promoter	MIR150
chr19	3395000	chr19	11695000	4.13E-02	super_enhancer	promoter	GAPDHP76
chr19	3395000	chr19	16285000	3.51E-02	super_enhancer	promoter	CIB3
chr19	3395000	chr19	42055000	1.16E-02	super_enhancer	promoter	CEACAM21
chr19	3395000	chr19	54975000	6.82E-03	super_enhancer	promoter	LENG9
chr19	3395000	chr19	55385000	7.84E-03	super_enhancer	promoter	FCAR
chr19	3395000	chr19	56995000	7.20E-03	super_enhancer	promoter	AC098795.1
chr19	3435000	chr19	4535000	2.80E-02	super_enhancer	promoter	PLIN5
chr19	3435000	chr19	11465000	3.73E-02	super_enhancer	promoter	LPPR2
chr19	3435000	chr19	12035000	2.90E-02	super_enhancer	promoter	ZNF700
chr19	3435000	chr19	13105000	2.62E-02	super_enhancer	promoter	NFIX
chr19	3435000	chr19	44085000	1.20E-02	super_enhancer	promoter	XRCC1
chr19	3435000	chr19	52065000	6.33E-03	super_enhancer	promoter	SIGLEC28P
chr19	3445000	chr19	6555000	4.65E-03	super_enhancer	promoter	CTD-2396E7.7
chr19	3445000	chr19	19175000	3.00E-02	super_enhancer	promoter	SLC25A42
chr19	3445000	chr19	35265000	1.12E-02	super_enhancer	promoter	ZNF599
chr19	3455000	chr19	9945000	3.64E-02	super_enhancer	promoter	PIN1

chr19	3455000	chr19	21105000	1.00E-02	super_enhancer	promoter	ZNF85
chr19	3455000	chr19	36005000	1.08E-02	super_enhancer	promoter	DMKN
chr19	3455000	chr19	40025000	1.16E-02	super_enhancer	promoter	TDGF1P7
chr19	3455000	chr19	45035000	1.07E-02	super_enhancer	promoter	CEACAM20
chr19	3455000	chr19	57875000	6.74E-03	super_enhancer	promoter	ZNF547
chr19	3465000	chr19	9945000	3.06E-02	super_enhancer	promoter	PIN1
chr19	3465000	chr19	13105000	1.89E-02	super_enhancer	promoter	NFIX
chr19	3465000	chr19	17665000	1.91E-02	super_enhancer	promoter	COLGALT1
chr19	3465000	chr19	18655000	2.06E-02	super_enhancer	promoter	FKBP8
chr19	3465000	chr19	54245000	5.74E-03	super_enhancer	promoter	MIR517C
chr19	3475000	chr19	13735000	3.16E-02	super_enhancer	promoter	CACNA1A
chr19	3475000	chr19	41345000	9.94E-03	super_enhancer	promoter	CTC-490E21.11
chr19	3475000	chr19	52075000	5.40E-03	super_enhancer	promoter	ZNF175
chr19	3505000	chr19	4055000	4.40E-02	promoter	super_enhancer	FZR1
chr19	3505000	chr19	13835000	3.14E-02	promoter	super_enhancer	FZR1
chr19	3505000	chr19	18415000	3.24E-02	promoter	super_enhancer	FZR1
chr19	3505000	chr19	41225000	1.41E-02	promoter	super_enhancer	FZR1
chr19	3555000	chr19	13265000	2.27E-02	super_enhancer	promoter	CTC-250I14.6
chr19	3555000	chr19	36055000	1.16E-02	super_enhancer	promoter	ATP4A
chr19	3555000	chr19	45655000	1.29E-02	super_enhancer	promoter	AC005757.7
chr19	3565000	chr19	10445000	4.49E-02	super_enhancer	promoter	RAVER1
chr19	3565000	chr19	10765000	4.09E-02	super_enhancer	promoter	ILF3-AS1
chr19	3565000	chr19	11455000	3.76E-02	super_enhancer	promoter	CCDC159
chr19	3565000	chr19	12075000	3.86E-02	super_enhancer	promoter	ZNF763

chr19	3565000	chr19	22035000	9.82E-03	super_enhancer	promoter	ZNF43
chr19	3565000	chr19	36105000	1.53E-02	super_enhancer	promoter	AC002115.9
chr19	3575000	chr19	3585000	6.12E-03	super_enhancer	promoter	GIPC3
chr19	3575000	chr19	7985000	3.50E-03	super_enhancer	promoter	CTD-3193O13.1
chr19	3575000	chr19	12035000	2.99E-02	super_enhancer	promoter	ZNF700
chr19	3575000	chr19	50295000	7.11E-03	super_enhancer	promoter	MIR6799
chr19	3585000	chr19	4135000	1.72E-02	promoter	super_enhancer	GIPC3
chr19	3585000	chr19	13165000	3.82E-02	promoter	super_enhancer	GIPC3
chr19	3585000	chr19	16605000	3.57E-02	promoter	super_enhancer	GIPC3
chr19	3585000	chr19	17105000	3.93E-02	promoter	super_enhancer	GIPC3
chr19	3585000	chr19	38715000	1.40E-02	promoter	super_enhancer	GIPC3
chr19	3585000	chr19	43195000	1.39E-02	promoter	super_enhancer	GIPC3
chr19	3585000	chr19	45725000	1.44E-02	promoter	super_enhancer	GIPC3
chr19	3585000	chr19	48975000	1.16E-02	promoter	super_enhancer	GIPC3
chr19	3985000	chr19	4045000	5.82E-03	promoter	super_enhancer	EEF2
chr19	3985000	chr19	4135000	4.11E-04	promoter	super_enhancer	EEF2
chr19	3985000	chr19	4155000	3.47E-03	promoter	super_enhancer	EEF2
chr19	3985000	chr19	11055000	4.34E-02	promoter	super_enhancer	EEF2
chr19	3985000	chr19	11115000	4.37E-02	promoter	super_enhancer	EEF2
chr19	3985000	chr19	17105000	3.90E-02	promoter	super_enhancer	EEF2
chr19	3985000	chr19	17795000	6.05E-06	promoter	super_enhancer	EEF2
chr19	4045000	chr19	13075000	3.95E-02	super_enhancer	promoter	DAND5
chr19	4045000	chr19	40175000	1.28E-02	super_enhancer	promoter	AC093063.3
chr19	4045000	chr19	42895000	1.59E-02	super_enhancer	promoter	CNFN

chr19	4045000	chr19	46155000	1.29E-02	super_enhancer	promoter	RN7SL836P
chr19	4045000	chr19	50355000	8.11E-03	super_enhancer	promoter	PTOV1-AS1
chr19	4045000	chr19	56165000	9.00E-03	super_enhancer	promoter	U2AF2
chr19	4045000	chr19	58995000	6.68E-03	super_enhancer	promoter	RNU6-1337P
chr19	4055000	chr19	12615000	2.25E-02	super_enhancer	promoter	CTD-3105H18.10
chr19	4055000	chr19	13005000	3.82E-02	super_enhancer	promoter	AD000092.3
chr19	4055000	chr19	36105000	1.59E-02	super_enhancer	promoter	AC002115.9
chr19	4055000	chr19	38545000	1.71E-02	super_enhancer	promoter	AC011465.1
chr19	4055000	chr19	39755000	1.53E-02	super_enhancer	promoter	IFNL4P1
chr19	4055000	chr19	56905000	7.39E-03	super_enhancer	promoter	ZNF582
chr19	4105000	chr19	9945000	4.70E-02	super_enhancer	promoter	PIN1
chr19	4105000	chr19	10445000	4.61E-02	super_enhancer	promoter	RAVER1
chr19	4105000	chr19	12305000	2.75E-02	super_enhancer	promoter	CTD-2666L21.1
chr19	4105000	chr19	13075000	3.69E-02	super_enhancer	promoter	DAND5
chr19	4105000	chr19	36235000	1.22E-02	super_enhancer	promoter	PSENEN
chr19	4115000	chr19	5905000	2.43E-03	super_enhancer	promoter	AC024592.12
chr19	4115000	chr19	16435000	3.52E-02	super_enhancer	promoter	CTD-2562J15.6
chr19	4115000	chr19	18385000	2.94E-02	super_enhancer	promoter	KIAA1683
chr19	4115000	chr19	35305000	1.09E-02	super_enhancer	promoter	CTC-523E23.1
chr19	4125000	chr19	11435000	4.43E-02	super_enhancer	promoter	CTC-510F12.4
chr19	4125000	chr19	11495000	4.40E-02	super_enhancer	promoter	EPOR
chr19	4125000	chr19	14445000	3.34E-02	super_enhancer	promoter	CTC-548K16.1
chr19	4125000	chr19	16955000	3.51E-02	super_enhancer	promoter	CTD-2538G9.5
chr19	4125000	chr19	19045000	3.55E-02	super_enhancer	promoter	AC002985.3

chr19	4125000	chr19	51685000	6.78E-03	super_enhancer	promoter	CTD-3187F8.14
chr19	4135000	chr19	13125000	3.50E-02	super_enhancer	promoter	CTC-239J10.1
chr19	4135000	chr19	16215000	3.13E-02	super_enhancer	promoter	CTD-2231E14.5
chr19	4135000	chr19	33315000	1.71E-02	super_enhancer	promoter	CTD-2085J24.5
chr19	4135000	chr19	42465000	1.40E-02	super_enhancer	promoter	RABAC1
chr19	4135000	chr19	46145000	9.96E-03	super_enhancer	promoter	EML2-AS1
chr19	4135000	chr19	49375000	8.37E-03	super_enhancer	promoter	PPP1R15A
chr19	4145000	chr19	12255000	3.39E-02	super_enhancer	promoter	CTC-359D24.3
chr19	4145000	chr19	46105000	1.13E-02	super_enhancer	promoter	OPA3
chr19	4155000	chr19	14445000	3.50E-02	super_enhancer	promoter	CTC-548K16.1
chr19	4155000	chr19	36035000	1.52E-02	super_enhancer	promoter	TMEM147
chr19	4155000	chr19	41725000	1.59E-02	super_enhancer	promoter	AXL
chr19	4305000	chr19	15295000	3.27E-02	promoter	super_enhancer	FSD1
chr19	4305000	chr19	40625000	9.67E-03	promoter	super_enhancer	FSD1
chr19	4355000	chr19	16285000	3.78E-02	super_enhancer	promoter	CIB3
chr19	4355000	chr19	43775000	8.52E-03	super_enhancer	promoter	PSG9
chr19	4365000	chr19	11495000	4.78E-02	super_enhancer	promoter	EPOR
chr19	4365000	chr19	15005000	3.10E-02	super_enhancer	promoter	OR7A1P
chr19	4365000	chr19	17445000	4.18E-02	super_enhancer	promoter	GTPBP3
chr19	4375000	chr19	12835000	4.15E-02	super_enhancer	promoter	TNPO2
chr19	4375000	chr19	28285000	9.13E-03	super_enhancer	promoter	LINC00662
chr19	4375000	chr19	36505000	1.25E-02	super_enhancer	promoter	AC002116.8
chr19	4375000	chr19	38545000	1.43E-02	super_enhancer	promoter	AC011465.1
chr19	4375000	chr19	44555000	8.01E-03	super_enhancer	promoter	ZNF223

chr19	4385000	chr19	11485000	3.76E-02	super_enhancer	promoter	SWSAP1
chr19	4385000	chr19	17905000	3.06E-02	super_enhancer	promoter	B3GNT3
chr19	4385000	chr19	18435000	2.90E-02	super_enhancer	promoter	LSM4
chr19	4385000	chr19	42055000	9.59E-03	super_enhancer	promoter	CEACAM21
chr19	4385000	chr19	49315000	6.61E-03	super_enhancer	promoter	BCAT2
chr19	4385000	chr19	58515000	7.57E-03	super_enhancer	promoter	ZNF606
chr19	4395000	chr19	12175000	3.69E-02	super_enhancer	promoter	ZNF844
chr19	4395000	chr19	38825000	1.14E-02	super_enhancer	promoter	CATSPERG
chr19	4395000	chr19	45395000	9.35E-03	super_enhancer	promoter	CTB-129P6.4
chr19	4395000	chr19	45445000	1.08E-02	super_enhancer	promoter	APOC4
chr19	4395000	chr19	59085000	6.99E-03	super_enhancer	promoter	MZF1
chr19	4445000	chr19	13845000	3.32E-02	promoter	super_enhancer	MIR4746
chr19	4445000	chr19	16585000	3.83E-02	promoter	super_enhancer	MIR4746
chr19	4445000	chr19	41205000	1.41E-02	promoter	super_enhancer	MIR4746
chr19	4535000	chr19	4575000	2.61E-02	promoter	super_enhancer	PLIN5
chr19	4535000	chr19	17115000	4.33E-02	promoter	super_enhancer	PLIN5
chr19	4535000	chr19	18465000	3.64E-02	promoter	super_enhancer	PLIN5
chr19	4535000	chr19	18675000	3.21E-02	promoter	super_enhancer	PLIN5
chr19	4535000	chr19	38265000	1.22E-02	promoter	super_enhancer	PLIN5
chr19	4535000	chr19	38725000	1.55E-02	promoter	super_enhancer	PLIN5
chr19	4535000	chr19	43525000	7.49E-03	promoter	super_enhancer	PLIN5
chr19	4545000	chr19	5625000	1.68E-02	super_enhancer	promoter	SAFB2
chr19	4545000	chr19	9795000	4.28E-02	super_enhancer	promoter	RPS4XP22
chr19	4545000	chr19	13005000	3.14E-02	super_enhancer	promoter	AD000092.3

chr19	4545000	chr19	53935000	7.06E-03	super_enhancer	promoter	TPM3P9
chr19	4555000	chr19	9315000	4.98E-02	super_enhancer	promoter	OR7E25P
chr19	4555000	chr19	14445000	3.14E-02	super_enhancer	promoter	CTC-548K16.1
chr19	4555000	chr19	14965000	2.83E-02	super_enhancer	promoter	AC005255.5
chr19	4555000	chr19	17845000	3.58E-02	super_enhancer	promoter	CTD-3149D2.4
chr19	4555000	chr19	40725000	1.33E-02	super_enhancer	promoter	TTC9B
chr19	4555000	chr19	42585000	1.17E-02	super_enhancer	promoter	CTB-59C6.3
chr19	4555000	chr19	46105000	1.14E-02	super_enhancer	promoter	OPA3
chr19	4555000	chr19	54665000	8.91E-03	super_enhancer	promoter	LENG1
chr19	4565000	chr19	11925000	2.83E-02	super_enhancer	promoter	ZNF440
chr19	4565000	chr19	40175000	9.46E-03	super_enhancer	promoter	AC093063.3
chr19	4575000	chr19	4725000	3.59E-02	super_enhancer	promoter	DPP9
chr19	4575000	chr19	11455000	4.63E-02	super_enhancer	promoter	CCDC159
chr19	4575000	chr19	12985000	3.89E-02	super_enhancer	promoter	HOOK2
chr19	4575000	chr19	13215000	4.11E-02	super_enhancer	promoter	LYL1
chr19	4575000	chr19	17665000	3.21E-02	super_enhancer	promoter	COLGALT1
chr19	4575000	chr19	44645000	1.15E-02	super_enhancer	promoter	ZNF234
chr19	4575000	chr19	50185000	7.97E-03	super_enhancer	promoter	MIR5088
chr19	4575000	chr19	51895000	7.60E-03	super_enhancer	promoter	C19orf84
chr19	4695000	chr19	11455000	3.88E-02	super_enhancer	promoter	CCDC159
chr19	4695000	chr19	35325000	1.30E-02	super_enhancer	promoter	CTC-523E23.4
chr19	4695000	chr19	44165000	1.18E-02	super_enhancer	promoter	RN7SL368P
chr19	4695000	chr19	45005000	1.05E-02	super_enhancer	promoter	ZNF180
chr19	4695000	chr19	56445000	6.69E-03	super_enhancer	promoter	NLRP13

chr19	4705000	chr19	11465000	4.18E-02	super_enhancer	promoter	LPPR2
chr19	4705000	chr19	42725000	1.07E-02	super_enhancer	promoter	DEDD2
chr19	4705000	chr19	44615000	9.71E-03	super_enhancer	promoter	ZNF225
chr19	4705000	chr19	48895000	7.66E-03	super_enhancer	promoter	KDELR1
chr19	4715000	chr19	6555000	2.33E-02	super_enhancer	promoter	CTD-2396E7.7
chr19	4715000	chr19	13985000	4.53E-02	super_enhancer	promoter	MIR181C
chr19	4715000	chr19	45875000	1.44E-02	super_enhancer	promoter	ERCC2
chr19	4725000	chr19	16355000	3.58E-02	promoter	super_enhancer	DPP9
chr19	4725000	chr19	16585000	3.96E-02	promoter	super_enhancer	DPP9
chr19	4725000	chr19	16595000	3.78E-02	promoter	super_enhancer	DPP9
chr19	4725000	chr19	18365000	3.43E-02	promoter	super_enhancer	DPP9
chr19	4725000	chr19	18455000	3.54E-02	promoter	super_enhancer	DPP9
chr19	4865000	chr19	5675000	3.04E-02	super_enhancer	promoter	RPL36
chr19	4865000	chr19	13045000	3.58E-02	super_enhancer	promoter	FARSA
chr19	4865000	chr19	13125000	3.43E-02	super_enhancer	promoter	CTC-239J10.1
chr19	4865000	chr19	15675000	2.87E-02	super_enhancer	promoter	CYP4F23P
chr19	4865000	chr19	41905000	1.32E-02	super_enhancer	promoter	CTC-435M10.10
chr19	4865000	chr19	48615000	7.50E-03	super_enhancer	promoter	PLA2G4C
chr19	4865000	chr19	48825000	7.49E-03	super_enhancer	promoter	CCDC114
chr19	5135000	chr19	5455000	1.20E-02	super_enhancer	promoter	ZNRF4
chr19	5135000	chr19	13045000	4.64E-02	super_enhancer	promoter	FARSA
chr19	5135000	chr19	17905000	4.53E-02	super_enhancer	promoter	B3GNT3
chr19	5135000	chr19	40135000	1.67E-02	super_enhancer	promoter	AC005176.3
chr19	5135000	chr19	51815000	7.38E-03	super_enhancer	promoter	IGLON5

chr19	5135000	chr19	55805000	1.12E-02	super_enhancer	promoter	AC020922.1
chr19	5145000	chr19	5455000	7.43E-03	super_enhancer	promoter	ZNRF4
chr19	5145000	chr19	13215000	4.13E-02	super_enhancer	promoter	LYL1
chr19	5145000	chr19	44575000	1.27E-02	super_enhancer	promoter	ZNF284
chr19	5145000	chr19	54665000	1.03E-02	super_enhancer	promoter	LENG1
chr19	5155000	chr19	12615000	1.90E-02	super_enhancer	promoter	CTD-3105H18.10
chr19	5155000	chr19	13055000	3.05E-02	super_enhancer	promoter	CTC-425F1.4
chr19	5155000	chr19	13905000	2.73E-02	super_enhancer	promoter	ZSWIM4
chr19	5155000	chr19	19845000	1.77E-02	super_enhancer	promoter	ZNF14
chr19	5155000	chr19	50095000	4.86E-03	super_enhancer	promoter	NOSIP
chr19	5155000	chr19	56125000	7.30E-03	super_enhancer	promoter	CTD-2537I9.18
chr19	5165000	chr19	6555000	3.11E-02	super_enhancer	promoter	CTD-2396E7.7
chr19	5165000	chr19	13875000	3.07E-02	super_enhancer	promoter	MRI1
chr19	5165000	chr19	18435000	2.92E-02	super_enhancer	promoter	LSM4
chr19	5165000	chr19	23255000	8.16E-03	super_enhancer	promoter	CTD-2291D10.3
chr19	5165000	chr19	41935000	1.10E-02	super_enhancer	promoter	B3GNT8
chr19	5165000	chr19	44165000	1.06E-02	super_enhancer	promoter	RN7SL368P
chr19	5165000	chr19	51895000	5.60E-03	super_enhancer	promoter	C19orf84
chr19	5175000	chr19	12615000	2.19E-02	super_enhancer	promoter	CTD-3105H18.10
chr19	5175000	chr19	13905000	3.15E-02	super_enhancer	promoter	ZSWIM4
chr19	5175000	chr19	40135000	1.27E-02	super_enhancer	promoter	AC005176.3
chr19	5175000	chr19	41725000	1.40E-02	super_enhancer	promoter	AXL
chr19	5175000	chr19	42855000	1.15E-02	super_enhancer	promoter	MIR8077
chr19	5175000	chr19	57875000	7.70E-03	super_enhancer	promoter	ZNF547

chr19	5175000	chr19	58985000	5.91E-03	super_enhancer	promoter	ZNF446
chr19	5185000	chr19	5625000	4.76E-02	super_enhancer	promoter	SAFB2
chr19	5185000	chr19	12165000	4.66E-02	super_enhancer	promoter	CTD-2006C1.10
chr19	5185000	chr19	42415000	1.45E-02	super_enhancer	promoter	CTD-2575K13.6
chr19	5185000	chr19	50295000	8.56E-03	super_enhancer	promoter	MIR6799
chr19	5455000	chr19	17085000	3.61E-02	promoter	super_enhancer	ZNRF4
chr19	5455000	chr19	17105000	3.84E-02	promoter	super_enhancer	ZNRF4
chr19	5455000	chr19	19395000	2.84E-02	promoter	super_enhancer	ZNRF4
chr19	5455000	chr19	45425000	1.20E-02	promoter	super_enhancer	ZNRF4
chr19	5625000	chr19	15305000	3.96E-02	promoter	super_enhancer	SAFB2
chr19	5625000	chr19	16325000	3.36E-02	promoter	super_enhancer	SAFB2
chr19	5625000	chr19	16595000	4.18E-02	promoter	super_enhancer	SAFB2
chr19	5625000	chr19	38735000	1.53E-02	promoter	super_enhancer	SAFB2
chr19	5625000	chr19	41235000	1.62E-02	promoter	super_enhancer	SAFB2
chr19	5625000	chr19	45565000	1.60E-02	promoter	super_enhancer	SAFB2
chr19	5625000	chr19	46195000	1.29E-02	promoter	super_enhancer	SAFB2
chr19	5625000	chr19	47635000	8.77E-03	promoter	super_enhancer	SAFB2
chr19	5675000	chr19	11105000	4.59E-02	promoter	super_enhancer	RPL36
chr19	5675000	chr19	19375000	2.60E-02	promoter	super_enhancer	RPL36
chr19	5675000	chr19	47635000	6.73E-03	promoter	super_enhancer	RPL36
chr19	5785000	chr19	16345000	4.34E-02	promoter	super_enhancer	PRR22
chr19	5785000	chr19	18465000	4.26E-02	promoter	super_enhancer	PRR22
chr19	5905000	chr19	15305000	3.59E-02	promoter	super_enhancer	AC024592.12
chr19	5905000	chr19	17775000	3.18E-02	promoter	super_enhancer	AC024592.12

chr19	5905000	chr19	18455000	3.54E-02	promoter	super_enhancer	AC024592.12
chr19	5905000	chr19	18485000	3.33E-02	promoter	super_enhancer	AC024592.12
chr19	5905000	chr19	41265000	1.30E-02	promoter	super_enhancer	AC024592.12
chr19	5915000	chr19	15305000	3.49E-02	promoter	super_enhancer	AC104532.4
chr19	6065000	chr19	6465000	1.99E-02	super_enhancer	promoter	SLC25A23
chr19	6065000	chr19	12645000	4.56E-02	super_enhancer	promoter	CTD-2192J16.21
chr19	6065000	chr19	12985000	4.09E-02	super_enhancer	promoter	HOOK2
chr19	6065000	chr19	13265000	3.02E-02	super_enhancer	promoter	CTC-250I14.6
chr19	6065000	chr19	13985000	4.70E-02	super_enhancer	promoter	MIR181C
chr19	6065000	chr19	17395000	4.26E-02	super_enhancer	promoter	USHBP1
chr19	6065000	chr19	36055000	1.34E-02	super_enhancer	promoter	ATP4A
chr19	6065000	chr19	42575000	1.11E-02	super_enhancer	promoter	GRIK5
chr19	6065000	chr19	46235000	1.45E-02	super_enhancer	promoter	FBXO46
chr19	6075000	chr19	11545000	4.06E-02	super_enhancer	promoter	PRKCSH
chr19	6075000	chr19	13875000	3.11E-02	super_enhancer	promoter	MRI1
chr19	6075000	chr19	58735000	5.66E-03	super_enhancer	promoter	AC020915.2
chr19	6085000	chr19	12625000	4.98E-02	super_enhancer	promoter	ZNF709
chr19	6085000	chr19	12645000	4.35E-02	super_enhancer	promoter	CTD-2192J16.21
chr19	6085000	chr19	13075000	4.39E-02	super_enhancer	promoter	DAND5
chr19	6085000	chr19	15005000	3.02E-02	super_enhancer	promoter	OR7A1P
chr19	6085000	chr19	15575000	3.47E-02	super_enhancer	promoter	RASAL3
chr19	6085000	chr19	36105000	1.53E-02	super_enhancer	promoter	AC002115.9
chr19	6085000	chr19	49375000	8.13E-03	super_enhancer	promoter	PPP1R15A
chr19	6085000	chr19	55155000	8.14E-03	super_enhancer	promoter	LILRB4

chr19	6135000	chr19	6595000	6.69E-03	promoter	super_enhancer	ACSBG2
chr19	6135000	chr19	13835000	3.37E-02	promoter	super_enhancer	ACSBG2
chr19	6135000	chr19	15275000	3.01E-02	promoter	super_enhancer	ACSBG2
chr19	6135000	chr19	18415000	3.24E-02	promoter	super_enhancer	ACSBG2
chr19	6335000	chr19	15305000	3.52E-02	promoter	super_enhancer	ACER1
chr19	6335000	chr19	16355000	3.49E-02	promoter	super_enhancer	ACER1
chr19	6395000	chr19	13835000	4.17E-02	promoter	super_enhancer	GTF2F1
chr19	6395000	chr19	17775000	3.50E-02	promoter	super_enhancer	GTF2F1
chr19	6395000	chr19	18005000	3.67E-02	promoter	super_enhancer	GTF2F1
chr19	6395000	chr19	19585000	2.32E-02	promoter	super_enhancer	GTF2F1
chr19	6425000	chr19	9075000	4.30E-03	promoter	super_enhancer	KHSRP
chr19	6425000	chr19	16575000	3.08E-02	promoter	super_enhancer	KHSRP
chr19	6435000	chr19	47605000	7.72E-03	promoter	super_enhancer	SLC25A41
chr19	6465000	chr19	45565000	1.37E-02	promoter	super_enhancer	SLC25A23
chr19	6525000	chr19	14445000	3.59E-02	super_enhancer	promoter	CTC-548K16.1
chr19	6535000	chr19	13215000	4.20E-02	super_enhancer	promoter	LYL1
chr19	6535000	chr19	13265000	2.98E-02	super_enhancer	promoter	CTC-250I14.6
chr19	6535000	chr19	15675000	3.04E-02	super_enhancer	promoter	CYP4F23P
chr19	6535000	chr19	17325000	4.00E-02	super_enhancer	promoter	USE1
chr19	6535000	chr19	17905000	3.72E-02	super_enhancer	promoter	B3GNT3
chr19	6535000	chr19	41725000	1.47E-02	super_enhancer	promoter	AXL
chr19	6535000	chr19	46685000	1.05E-02	super_enhancer	promoter	AC006262.4
chr19	6535000	chr19	50815000	7.56E-03	super_enhancer	promoter	CTB-191K22.5
chr19	6545000	chr19	16285000	3.57E-02	super_enhancer	promoter	CIB3

chr19	6545000	chr19	16675000	3.29E-02	super_enhancer	promoter	CTD-3222D19.10
chr19	6545000	chr19	18385000	3.11E-02	super_enhancer	promoter	KIAA1683
chr19	6545000	chr19	42805000	1.21E-02	super_enhancer	promoter	PRR19
chr19	6545000	chr19	46765000	1.02E-02	super_enhancer	promoter	IGFL1P2
chr19	6555000	chr19	14385000	3.03E-02	promoter	super_enhancer	CTD-2396E7.7
chr19	6555000	chr19	48965000	8.77E-03	promoter	super_enhancer	CTD-2396E7.7
chr19	6565000	chr19	16355000	3.57E-02	promoter	super_enhancer	CLIC4P2
chr19	6565000	chr19	46895000	8.95E-03	promoter	super_enhancer	CLIC4P2
chr19	6575000	chr19	11485000	2.90E-02	super_enhancer	promoter	SWSAP1
chr19	6575000	chr19	12645000	2.21E-02	super_enhancer	promoter	CTD-2192J16.21
chr19	6575000	chr19	36045000	1.68E-05	super_enhancer	promoter	AC002389.1
chr19	6585000	chr19	7695000	1.15E-03	super_enhancer	promoter	XAB2
chr19	6585000	chr19	11545000	4.64E-02	super_enhancer	promoter	PRKCSH
chr19	6585000	chr19	12075000	4.39E-02	super_enhancer	promoter	ZNF763
chr19	6585000	chr19	12305000	3.09E-02	super_enhancer	promoter	CTD-2666L21.1
chr19	6585000	chr19	15945000	2.17E-02	super_enhancer	promoter	UCA1
chr19	6595000	chr19	7745000	1.32E-02	super_enhancer	promoter	TRAPPC5
chr19	6595000	chr19	10615000	4.02E-02	super_enhancer	promoter	KEAP1
chr19	6595000	chr19	35225000	1.12E-02	super_enhancer	promoter	CTD-2553L13.10
chr19	6605000	chr19	16215000	3.03E-02	super_enhancer	promoter	CTD-2231E14.5
chr19	6605000	chr19	19775000	2.24E-02	super_enhancer	promoter	ATP13A1
chr19	6605000	chr19	40185000	1.27E-02	super_enhancer	promoter	AC093063.2
chr19	7295000	chr19	13155000	4.80E-02	promoter	super_enhancer	INSR
chr19	7295000	chr19	15305000	4.14E-02	promoter	super_enhancer	INSR

chr19	7295000	chr19	17135000	4.27E-02	promoter	super_enhancer	INSR
chr19	7395000	chr19	47355000	4.16E-03	super_enhancer	promoter	AP2S1
chr19	7445000	chr19	16345000	3.92E-02	promoter	super_enhancer	CTD-2207O23.3
chr19	7445000	chr19	18475000	4.01E-02	promoter	super_enhancer	CTD-2207O23.3
chr19	7445000	chr19	40825000	1.52E-02	promoter	super_enhancer	CTD-2207O23.3
chr19	7585000	chr19	19375000	3.52E-02	promoter	super_enhancer	CTD-2207O23.11
chr19	7585000	chr19	41245000	1.58E-02	promoter	super_enhancer	CTD-2207O23.11
chr19	7585000	chr19	46785000	1.10E-02	promoter	super_enhancer	CTD-2207O23.11
chr19	7585000	chr19	47645000	8.77E-03	promoter	super_enhancer	CTD-2207O23.11
chr19	7585000	chr19	48985000	1.06E-02	promoter	super_enhancer	CTD-2207O23.11
chr19	7695000	chr19	15325000	4.35E-02	promoter	super_enhancer	XAB2
chr19	7695000	chr19	56115000	7.10E-03	promoter	super_enhancer	XAB2
chr19	7745000	chr19	18005000	3.76E-02	promoter	super_enhancer	TRAPPC5
chr19	7895000	chr19	19375000	3.53E-02	promoter	super_enhancer	EVI5L
chr19	7895000	chr19	47615000	9.03E-03	promoter	super_enhancer	EVI5L
chr19	7895000	chr19	55545000	9.16E-03	promoter	super_enhancer	EVI5L
chr19	7935000	chr19	14385000	4.07E-02	promoter	super_enhancer	CTD-3193O13.11
chr19	7935000	chr19	16585000	4.23E-02	promoter	super_enhancer	CTD-3193O13.11
chr19	7935000	chr19	18365000	3.58E-02	promoter	super_enhancer	CTD-3193O13.11
chr19	7935000	chr19	19395000	2.91E-02	promoter	super_enhancer	CTD-3193O13.11
chr19	7945000	chr19	15275000	4.22E-02	promoter	super_enhancer	LYPLA2P2
chr19	7945000	chr19	16585000	4.74E-02	promoter	super_enhancer	LYPLA2P2
chr19	7945000	chr19	45565000	1.62E-02	promoter	super_enhancer	LYPLA2P2
chr19	7945000	chr19	46205000	1.53E-02	promoter	super_enhancer	LYPLA2P2

chr19	7985000	chr19	16575000	4.80E-02	promoter	super_enhancer	CTD-3193O13.1
chr19	7985000	chr19	46885000	1.23E-02	promoter	super_enhancer	CTD-3193O13.1
chr19	8305000	chr19	41225000	2.15E-02	promoter	super_enhancer	CTD-3020H12.4
chr19	8305000	chr19	43525000	9.36E-03	promoter	super_enhancer	CTD-3020H12.4
chr19	8385000	chr19	15295000	4.33E-02	promoter	super_enhancer	RPS28
chr19	8385000	chr19	41205000	1.53E-02	promoter	super_enhancer	RPS28
chr19	8455000	chr19	15295000	3.39E-02	promoter	super_enhancer	MIR4999
chr19	8455000	chr19	18495000	2.98E-02	promoter	super_enhancer	MIR4999
chr19	8455000	chr19	43785000	6.57E-03	promoter	super_enhancer	MIR4999
chr19	8455000	chr19	47605000	6.48E-03	promoter	super_enhancer	MIR4999
chr19	8455000	chr19	55545000	6.78E-03	promoter	super_enhancer	MIR4999
chr19	8645000	chr19	11155000	1.40E-02	promoter	super_enhancer	AC130469.2
chr19	8645000	chr19	18665000	4.04E-02	promoter	super_enhancer	AC130469.2
chr19	8645000	chr19	38705000	1.27E-02	promoter	super_enhancer	AC130469.2
chr19	9045000	chr19	13265000	4.23E-02	super_enhancer	promoter	CTC-250I14.6
chr19	9045000	chr19	21665000	1.01E-02	super_enhancer	promoter	LINC00664
chr19	9045000	chr19	54135000	7.94E-03	super_enhancer	promoter	DPRX
chr19	9055000	chr19	39615000	7.26E-03	super_enhancer	promoter	PAK4
chr19	9055000	chr19	39755000	8.31E-03	super_enhancer	promoter	IFNL4P1
chr19	9055000	chr19	42105000	7.22E-03	super_enhancer	promoter	CEACAMP3
chr19	9055000	chr19	52065000	3.66E-03	super_enhancer	promoter	SIGLEC28P
chr19	9055000	chr19	54205000	4.59E-03	super_enhancer	promoter	MIR518B
chr19	9065000	chr19	16285000	4.32E-02	super_enhancer	promoter	CIB3
chr19	9065000	chr19	41935000	1.34E-02	super_enhancer	promoter	B3GNT8

chr19	9065000	chr19	45875000	1.35E-02	super_enhancer	promoter	ERCC2
chr19	9065000	chr19	52645000	5.89E-03	super_enhancer	promoter	CTC-471J1.9
chr19	9075000	chr19	10765000	2.01E-02	super_enhancer	promoter	ILF3-AS1
chr19	9075000	chr19	31855000	1.34E-02	super_enhancer	promoter	CTC-439O9.1
chr19	9075000	chr19	41905000	1.23E-02	super_enhancer	promoter	CTC-435M10.10
chr19	9075000	chr19	50465000	5.59E-03	super_enhancer	promoter	SIGLEC11
chr19	9195000	chr19	38295000	1.07E-02	promoter	super_enhancer	OR1M4P
chr19	9195000	chr19	41235000	1.56E-02	promoter	super_enhancer	OR1M4P
chr19	9215000	chr19	15285000	4.76E-02	promoter	super_enhancer	OR7G2
chr19	9215000	chr19	18375000	3.98E-02	promoter	super_enhancer	OR7G2
chr19	9215000	chr19	19615000	2.67E-02	promoter	super_enhancer	OR7G2
chr19	9295000	chr19	11025000	2.93E-02	promoter	super_enhancer	TCEB1P29
chr19	9295000	chr19	17105000	4.17E-02	promoter	super_enhancer	TCEB1P29
chr19	9295000	chr19	56095000	8.93E-03	promoter	super_enhancer	TCEB1P29
chr19	9345000	chr19	18505000	3.88E-02	promoter	super_enhancer	OR7D1P
chr19	9345000	chr19	56095000	9.84E-03	promoter	super_enhancer	OR7D1P
chr19	9435000	chr19	18495000	3.77E-02	promoter	super_enhancer	ZNF559-ZNF177
chr19	9695000	chr19	16345000	4.48E-02	promoter	super_enhancer	ZNF121
chr19	9695000	chr19	56115000	6.95E-03	promoter	super_enhancer	ZNF121
chr19	9795000	chr19	17125000	4.11E-02	promoter	super_enhancer	RPS4XP22
chr19	9885000	chr19	17105000	4.74E-02	promoter	super_enhancer	UBE2L4
chr19	9885000	chr19	19605000	3.02E-02	promoter	super_enhancer	UBE2L4
chr19	9905000	chr19	17795000	3.94E-02	promoter	super_enhancer	ZNF846
chr19	9905000	chr19	43435000	7.50E-03	promoter	super_enhancer	ZNF846

chr19	9945000	chr19	16325000	4.11E-02	promoter	super_enhancer	PIN1
chr19	9945000	chr19	16345000	4.48E-02	promoter	super_enhancer	PIN1
chr19	9945000	chr19	18465000	3.80E-02	promoter	super_enhancer	PIN1
chr19	9945000	chr19	18505000	3.90E-02	promoter	super_enhancer	PIN1
chr19	9945000	chr19	19585000	2.40E-02	promoter	super_enhancer	PIN1
chr19	9945000	chr19	43195000	1.35E-02	promoter	super_enhancer	PIN1
chr19	9945000	chr19	44745000	9.77E-03	promoter	super_enhancer	PIN1
chr19	10445000	chr19	17795000	4.29E-02	promoter	super_enhancer	RAVER1
chr19	10445000	chr19	18375000	4.28E-02	promoter	super_enhancer	RAVER1
chr19	10445000	chr19	18505000	4.34E-02	promoter	super_enhancer	RAVER1
chr19	10445000	chr19	19395000	3.50E-02	promoter	super_enhancer	RAVER1
chr19	10445000	chr19	41325000	1.49E-02	promoter	super_enhancer	RAVER1
chr19	10515000	chr19	33045000	1.16E-02	promoter	super_enhancer	MIR1181
chr19	10515000	chr19	41205000	1.26E-02	promoter	super_enhancer	MIR1181
chr19	10515000	chr19	43325000	5.50E-03	promoter	super_enhancer	MIR1181
chr19	10515000	chr19	43765000	7.78E-03	promoter	super_enhancer	MIR1181
chr19	10515000	chr19	45725000	1.16E-02	promoter	super_enhancer	MIR1181
chr19	10615000	chr19	17095000	2.84E-02	promoter	super_enhancer	KEAP1
chr19	10615000	chr19	18415000	2.72E-02	promoter	super_enhancer	KEAP1
chr19	10615000	chr19	19585000	1.53E-02	promoter	super_enhancer	KEAP1
chr19	10615000	chr19	41205000	9.34E-03	promoter	super_enhancer	KEAP1
chr19	10655000	chr19	13165000	1.38E-02	promoter	super_enhancer	ATG4D
chr19	10655000	chr19	39065000	1.95E-02	promoter	super_enhancer	ATG4D
chr19	10655000	chr19	45565000	1.89E-02	promoter	super_enhancer	ATG4D

chr19	10765000	chr19	18415000	4.57E-02	promoter	super_enhancer	ILF3-AS1
chr19	10765000	chr19	18685000	3.95E-02	promoter	super_enhancer	ILF3-AS1
chr19	11025000	chr19	18385000	4.86E-02	super_enhancer	promoter	KIAA1683
chr19	11025000	chr19	20845000	2.14E-02	super_enhancer	promoter	CTC-513N18.7
chr19	11025000	chr19	59025000	1.12E-02	super_enhancer	promoter	SLC27A5
chr19	11025000	chr19	59055000	5.25E-03	super_enhancer	promoter	RN7SL525P
chr19	11035000	chr19	11925000	3.97E-03	super_enhancer	promoter	ZNF440
chr19	11035000	chr19	12835000	4.46E-02	super_enhancer	promoter	TNPO2
chr19	11035000	chr19	50145000	8.68E-03	super_enhancer	promoter	SCAF1
chr19	11045000	chr19	14065000	6.51E-03	super_enhancer	promoter	PODNL1
chr19	11055000	chr19	12445000	3.53E-02	super_enhancer	promoter	ZNF563
chr19	11055000	chr19	39615000	1.41E-02	super_enhancer	promoter	PAK4
chr19	11055000	chr19	41815000	1.35E-02	super_enhancer	promoter	CCDC97
chr19	11065000	chr19	15775000	4.58E-02	super_enhancer	promoter	CYP4F10P
chr19	11065000	chr19	40725000	1.67E-02	super_enhancer	promoter	TTC9B
chr19	11065000	chr19	40855000	1.48E-02	super_enhancer	promoter	C19orf47
chr19	11065000	chr19	45075000	1.42E-02	super_enhancer	promoter	AC138472.4
chr19	11065000	chr19	51845000	7.69E-03	super_enhancer	promoter	VSIG10L
chr19	11065000	chr19	55215000	1.11E-02	super_enhancer	promoter	AC098784.1
chr19	11075000	chr19	21415000	1.19E-02	super_enhancer	promoter	RPL36AP51
chr19	11075000	chr19	35395000	1.32E-02	super_enhancer	promoter	CTC-523E23.14
chr19	11075000	chr19	40855000	1.34E-02	super_enhancer	promoter	C19orf47
chr19	11075000	chr19	47165000	1.05E-02	super_enhancer	promoter	DACT3
chr19	11075000	chr19	49955000	8.11E-03	super_enhancer	promoter	ALDH16A1

chr19	11075000	chr19	50935000	7.63E-03	super_enhancer	promoter	MYBPC2
chr19	11085000	chr19	11435000	4.22E-02	super_enhancer	promoter	CTC-510F12.4
chr19	11085000	chr19	36605000	1.96E-02	super_enhancer	promoter	TBCB
chr19	11085000	chr19	53195000	8.03E-03	super_enhancer	promoter	ZNF83
chr19	11085000	chr19	55215000	1.14E-02	super_enhancer	promoter	AC098784.1
chr19	11085000	chr19	57875000	9.64E-03	super_enhancer	promoter	ZNF547
chr19	11095000	chr19	19145000	4.43E-02	super_enhancer	promoter	SUGP2
chr19	11095000	chr19	36485000	1.82E-02	super_enhancer	promoter	SDHAF1
chr19	11095000	chr19	39575000	1.77E-02	super_enhancer	promoter	PAPL
chr19	11095000	chr19	44615000	1.28E-02	super_enhancer	promoter	ZNF225
chr19	11095000	chr19	45165000	1.76E-02	super_enhancer	promoter	CEACAM19
chr19	11095000	chr19	46765000	1.30E-02	super_enhancer	promoter	IGFL1P2
chr19	11095000	chr19	54665000	1.14E-02	super_enhancer	promoter	LENG1
chr19	11105000	chr19	44455000	1.14E-02	super_enhancer	promoter	ZNF221
chr19	11105000	chr19	50435000	8.15E-03	super_enhancer	promoter	MIR4751
chr19	11105000	chr19	51685000	7.65E-03	super_enhancer	promoter	CTD-3187F8.14
chr19	11115000	chr19	18655000	4.14E-02	super_enhancer	promoter	FKBP8
chr19	11115000	chr19	19045000	4.63E-02	super_enhancer	promoter	AC002985.3
chr19	11115000	chr19	51815000	6.49E-03	super_enhancer	promoter	IGLON5
chr19	11135000	chr19	11485000	3.19E-02	super_enhancer	promoter	SWSAP1
chr19	11145000	chr19	13905000	9.40E-03	super_enhancer	promoter	ZSWIM4
chr19	11145000	chr19	17325000	2.66E-03	super_enhancer	promoter	USE1
chr19	11145000	chr19	19045000	1.60E-03	super_enhancer	promoter	AC002985.3
chr19	11145000	chr19	33795000	2.41E-02	super_enhancer	promoter	CTD-2540B15.9

chr19	11145000	chr19	36005000	1.83E-02	super_enhancer	promoter	DMKN
chr19	11145000	chr19	41675000	1.96E-02	super_enhancer	promoter	RPL36P16
chr19	11145000	chr19	54215000	1.27E-02	super_enhancer	promoter	MIR517A
chr19	11155000	chr19	16435000	2.81E-03	super_enhancer	promoter	CTD-2562J15.6
chr19	11155000	chr19	33355000	2.34E-02	super_enhancer	promoter	RN7SKP22
chr19	11155000	chr19	48775000	1.11E-02	super_enhancer	promoter	CTC-241F20.4
chr19	11155000	chr19	58995000	8.08E-03	super_enhancer	promoter	RNU6-1337P
chr19	11165000	chr19	19045000	4.60E-02	super_enhancer	promoter	AC002985.3
chr19	11165000	chr19	33795000	1.97E-02	super_enhancer	promoter	CTD-2540B15.9
chr19	11165000	chr19	41725000	1.65E-02	super_enhancer	promoter	AXL
chr19	11165000	chr19	42805000	9.48E-05	super_enhancer	promoter	PRR19
chr19	11165000	chr19	52005000	7.99E-03	super_enhancer	promoter	SIGLEC12
chr19	11165000	chr19	58875000	8.18E-03	super_enhancer	promoter	CTD-2619J13.8
chr19	11435000	chr19	15315000	2.81E-03	promoter	super_enhancer	CTC-510F12.4
chr19	11435000	chr19	43545000	6.79E-03	promoter	super_enhancer	CTC-510F12.4
chr19	11435000	chr19	47665000	7.88E-03	promoter	super_enhancer	CTC-510F12.4
chr19	11455000	chr19	19365000	4.53E-02	promoter	super_enhancer	CCDC159
chr19	11455000	chr19	38255000	1.25E-02	promoter	super_enhancer	CCDC159
chr19	11455000	chr19	38735000	1.54E-02	promoter	super_enhancer	CCDC159
chr19	11465000	chr19	46895000	1.07E-02	promoter	super_enhancer	LPPR2
chr19	11465000	chr19	47585000	9.72E-03	promoter	super_enhancer	LPPR2
chr19	11485000	chr19	33075000	2.17E-02	promoter	super_enhancer	SWSAP1
chr19	11485000	chr19	40815000	1.77E-02	promoter	super_enhancer	SWSAP1
chr19	11495000	chr19	45425000	1.43E-02	promoter	super_enhancer	EPOR

chr19	11545000	chr19	19385000	3.68E-02	promoter	super_enhancer	PRKCSH
chr19	11545000	chr19	41215000	1.74E-02	promoter	super_enhancer	PRKCSH
chr19	11545000	chr19	48325000	9.35E-03	promoter	super_enhancer	PRKCSH
chr19	11925000	chr19	18665000	4.66E-02	promoter	super_enhancer	ZNF440
chr19	12035000	chr19	40615000	6.52E-03	promoter	super_enhancer	ZNF700
chr19	12165000	chr19	18015000	4.86E-02	promoter	super_enhancer	CTD-2006C1.10
chr19	12165000	chr19	45405000	1.36E-02	promoter	super_enhancer	CTD-2006C1.10
chr19	12255000	chr19	19375000	4.35E-02	promoter	super_enhancer	CTC-359D24.3
chr19	12255000	chr19	41325000	1.50E-02	promoter	super_enhancer	CTC-359D24.3
chr19	12255000	chr19	43765000	1.01E-02	promoter	super_enhancer	CTC-359D24.3
chr19	12305000	chr19	46205000	1.22E-02	promoter	super_enhancer	CTD-2666L21.1
chr19	12395000	chr19	38645000	1.36E-02	promoter	super_enhancer	CTD-2666L21.2
chr19	12395000	chr19	38695000	1.38E-02	promoter	super_enhancer	CTD-2666L21.2
chr19	12395000	chr19	40805000	1.31E-02	promoter	super_enhancer	CTD-2666L21.2
chr19	12445000	chr19	48575000	6.29E-03	promoter	super_enhancer	ZNF563
chr19	12495000	chr19	55535000	6.44E-03	promoter	super_enhancer	CTD-3105H18.4
chr19	12625000	chr19	45405000	1.68E-02	promoter	super_enhancer	ZNF709
chr19	12645000	chr19	18675000	4.90E-02	promoter	super_enhancer	CTD-2192J16.21
chr19	12645000	chr19	47595000	1.08E-02	promoter	super_enhancer	CTD-2192J16.21
chr19	12755000	chr19	14505000	4.97E-02	promoter	super_enhancer	CTD-2192J16.15
chr19	12755000	chr19	38725000	2.26E-02	promoter	super_enhancer	CTD-2192J16.15
chr19	12945000	chr19	33075000	2.65E-02	promoter	super_enhancer	MAST1
chr19	12945000	chr19	38655000	2.13E-02	promoter	super_enhancer	MAST1
chr19	12945000	chr19	40585000	1.49E-02	promoter	super_enhancer	MAST1

chr19	12945000	chr19	40785000	1.82E-02	promoter	super_enhancer	MAST1
chr19	12945000	chr19	41225000	2.26E-02	promoter	super_enhancer	MAST1
chr19	12945000	chr19	41245000	2.11E-02	promoter	super_enhancer	MAST1
chr19	13005000	chr19	48975000	1.32E-02	promoter	super_enhancer	AD000092.3
chr19	13005000	chr19	55545000	1.01E-02	promoter	super_enhancer	AD000092.3
chr19	13045000	chr19	45405000	1.48E-02	promoter	super_enhancer	FARSA
chr19	13055000	chr19	48965000	1.24E-02	promoter	super_enhancer	CTC-425F1.4
chr19	13075000	chr19	13165000	1.74E-03	promoter	super_enhancer	DAND5
chr19	13075000	chr19	13175000	3.26E-03	promoter	super_enhancer	DAND5
chr19	13075000	chr19	40795000	1.98E-02	promoter	super_enhancer	DAND5
chr19	13075000	chr19	40805000	1.76E-02	promoter	super_enhancer	DAND5
chr19	13105000	chr19	13165000	8.92E-03	promoter	super_enhancer	NFIX
chr19	13125000	chr19	43465000	5.29E-03	promoter	super_enhancer	CTC-239J10.1
chr19	13155000	chr19	31045000	1.70E-02	super_enhancer	promoter	CTD-2001J20.1
chr19	13155000	chr19	54665000	1.07E-02	super_enhancer	promoter	LENG1
chr19	13165000	chr19	34515000	1.45E-02	super_enhancer	promoter	RPS4XP20
chr19	13165000	chr19	38795000	1.53E-02	super_enhancer	promoter	C19orf33
chr19	13165000	chr19	42725000	1.43E-02	super_enhancer	promoter	DEDD2
chr19	13165000	chr19	50195000	1.05E-02	super_enhancer	promoter	CTB-33G10.6
chr19	13175000	chr19	19145000	4.76E-02	super_enhancer	promoter	SUGP2
chr19	13175000	chr19	19175000	4.79E-02	super_enhancer	promoter	SLC25A42
chr19	13175000	chr19	39575000	1.54E-02	super_enhancer	promoter	PAPL
chr19	13175000	chr19	41305000	1.06E-02	super_enhancer	promoter	EGLN2
chr19	13175000	chr19	41905000	1.49E-02	super_enhancer	promoter	CTC-435M10.10

chr19	13215000	chr19	40745000	1.80E-02	promoter	super_enhancer	LYL1
chr19	13215000	chr19	40785000	1.54E-02	promoter	super_enhancer	LYL1
chr19	13265000	chr19	18505000	4.73E-02	promoter	super_enhancer	CTC-250I14.6
chr19	13265000	chr19	38725000	1.23E-02	promoter	super_enhancer	CTC-250I14.6
chr19	13265000	chr19	40575000	1.01E-02	promoter	super_enhancer	CTC-250I14.6
chr19	13265000	chr19	41215000	1.36E-02	promoter	super_enhancer	CTC-250I14.6
chr19	13735000	chr19	13805000	3.45E-02	promoter	super_enhancer	CACNA1A
chr19	13735000	chr19	13835000	3.06E-02	promoter	super_enhancer	CACNA1A
chr19	13735000	chr19	13845000	2.06E-02	promoter	super_enhancer	CACNA1A
chr19	13735000	chr19	14385000	1.44E-02	promoter	super_enhancer	CACNA1A
chr19	13735000	chr19	19585000	3.76E-02	promoter	super_enhancer	CACNA1A
chr19	13785000	chr19	35835000	2.09E-02	super_enhancer	promoter	MIR5196
chr19	13785000	chr19	39575000	1.85E-02	super_enhancer	promoter	PAPL
chr19	13785000	chr19	58865000	9.36E-03	super_enhancer	promoter	A1BG
chr19	13795000	chr19	39185000	1.71E-02	super_enhancer	promoter	CTB-186G2.4
chr19	13795000	chr19	40875000	2.30E-02	super_enhancer	promoter	MIR6796
chr19	13795000	chr19	42895000	2.19E-02	super_enhancer	promoter	CNFN
chr19	13795000	chr19	49055000	1.00E-02	super_enhancer	promoter	SULT2B1
chr19	13795000	chr19	49585000	1.15E-02	super_enhancer	promoter	RN7SL708P
chr19	13795000	chr19	51645000	1.15E-02	super_enhancer	promoter	SIGLEC7
chr19	13795000	chr19	54975000	9.16E-03	super_enhancer	promoter	LENG9
chr19	13805000	chr19	15775000	1.50E-02	super_enhancer	promoter	CYP4F10P
chr19	13805000	chr19	30415000	2.17E-02	super_enhancer	promoter	URI1
chr19	13805000	chr19	38865000	1.95E-02	super_enhancer	promoter	PSMD8

chr19	13805000	chr19	39185000	1.72E-02	super_enhancer	promoter	CTB-186G2.4
chr19	13805000	chr19	39695000	1.95E-02	super_enhancer	promoter	SYCN
chr19	13805000	chr19	41535000	2.00E-02	super_enhancer	promoter	CYP2A7P1
chr19	13805000	chr19	42725000	1.64E-02	super_enhancer	promoter	DEDD2
chr19	13805000	chr19	44555000	1.32E-02	super_enhancer	promoter	ZNF223
chr19	13805000	chr19	45075000	1.74E-02	super_enhancer	promoter	AC138472.4
chr19	13805000	chr19	50975000	1.08E-02	super_enhancer	promoter	Clostridiales-1
chr19	13805000	chr19	52195000	9.01E-03	super_enhancer	promoter	MIR99B
chr19	13835000	chr19	44395000	1.13E-02	super_enhancer	promoter	RP11-15A1.4
chr19	13835000	chr19	45165000	1.78E-02	super_enhancer	promoter	CEACAM19
chr19	13835000	chr19	46765000	1.31E-02	super_enhancer	promoter	IGFL1P2
chr19	13845000	chr19	38865000	1.67E-02	super_enhancer	promoter	PSMD8
chr19	13845000	chr19	39755000	1.84E-02	super_enhancer	promoter	IFNL4P1
chr19	13845000	chr19	44085000	1.55E-02	super_enhancer	promoter	XRCC1
chr19	13845000	chr19	57875000	9.67E-03	super_enhancer	promoter	ZNF547
chr19	13875000	chr19	46795000	1.30E-02	promoter	super_enhancer	MRI1
chr19	13885000	chr19	48555000	1.08E-02	promoter	super_enhancer	CTB-5E10.3
chr19	13905000	chr19	38725000	1.67E-02	promoter	super_enhancer	ZSWIM4
chr19	13905000	chr19	40575000	1.37E-02	promoter	super_enhancer	ZSWIM4
chr19	13985000	chr19	38695000	2.19E-02	promoter	super_enhancer	MIR181C
chr19	14065000	chr19	43195000	1.79E-02	promoter	super_enhancer	PODNL1
chr19	14065000	chr19	56095000	1.25E-02	promoter	super_enhancer	PODNL1
chr19	14385000	chr19	44645000	1.26E-02	super_enhancer	promoter	ZNF234
chr19	14395000	chr19	20845000	2.41E-02	super_enhancer	promoter	CTC-513N18.7

chr19	14395000	chr19	38865000	1.56E-02	super_enhancer	promoter	PSMD8
chr19	14395000	chr19	42855000	1.42E-02	super_enhancer	promoter	MIR8077
chr19	14395000	chr19	56755000	7.07E-03	super_enhancer	promoter	ZSCAN5D
chr19	14405000	chr19	38725000	1.58E-02	promoter	super_enhancer	CTD-2189E23.1
chr19	14405000	chr19	48575000	7.16E-03	promoter	super_enhancer	CTD-2189E23.1
chr19	14445000	chr19	42115000	1.67E-02	promoter	super_enhancer	CTC-548K16.1
chr19	14445000	chr19	43765000	1.14E-02	promoter	super_enhancer	CTC-548K16.1
chr19	14505000	chr19	39305000	2.02E-02	super_enhancer	promoter	LGALS4
chr19	14505000	chr19	49245000	1.02E-02	super_enhancer	promoter	RASIP1
chr19	14505000	chr19	57125000	7.69E-03	super_enhancer	promoter	AC007228.8
chr19	14695000	chr19	35315000	1.57E-02	promoter	super_enhancer	RN7SL337P
chr19	14695000	chr19	41265000	1.89E-02	promoter	super_enhancer	RN7SL337P
chr19	14965000	chr19	38685000	1.66E-02	promoter	super_enhancer	AC005255.5
chr19	14965000	chr19	38725000	1.69E-02	promoter	super_enhancer	AC005255.5
chr19	14965000	chr19	47645000	9.42E-03	promoter	super_enhancer	AC005255.5
chr19	15005000	chr19	45405000	1.41E-02	promoter	super_enhancer	OR7A1P
chr19	15005000	chr19	46195000	1.35E-02	promoter	super_enhancer	OR7A1P
chr19	15235000	chr19	15285000	1.38E-02	promoter	super_enhancer	AC003956.1
chr19	15235000	chr19	16575000	1.67E-02	promoter	super_enhancer	AC003956.1
chr19	15235000	chr19	38685000	2.07E-02	promoter	super_enhancer	AC003956.1
chr19	15255000	chr19	38665000	1.81E-02	promoter	super_enhancer	RNU6-782P
chr19	15255000	chr19	39075000	1.78E-02	promoter	super_enhancer	RNU6-782P
chr19	15275000	chr19	15495000	3.20E-02	super_enhancer	promoter	AC005785.5
chr19	15275000	chr19	15675000	1.71E-02	super_enhancer	promoter	CYP4F23P

chr19	15275000	chr19	15905000	6.83E-03	super_enhancer	promoter	OR10H5
chr19	15275000	chr19	39575000	1.91E-02	super_enhancer	promoter	PAPL
chr19	15275000	chr19	39695000	1.81E-02	super_enhancer	promoter	SYCN
chr19	15275000	chr19	42255000	1.52E-02	super_enhancer	promoter	CEACAM6
chr19	15275000	chr19	48775000	1.13E-02	super_enhancer	promoter	CTC-241F20.4
chr19	15275000	chr19	53775000	6.26E-03	super_enhancer	promoter	CTD-2245F17.9
chr19	15275000	chr19	53785000	9.50E-03	super_enhancer	promoter	FAM90A27P
chr19	15285000	chr19	17325000	3.27E-02	super_enhancer	promoter	USE1
chr19	15285000	chr19	21415000	1.93E-02	super_enhancer	promoter	RPL36AP51
chr19	15285000	chr19	38825000	1.74E-02	super_enhancer	promoter	CATSPERG
chr19	15285000	chr19	38875000	1.61E-02	super_enhancer	promoter	AC005789.9
chr19	15285000	chr19	41815000	1.62E-02	super_enhancer	promoter	CCDC97
chr19	15285000	chr19	46735000	1.59E-02	super_enhancer	promoter	AC006262.10
chr19	15285000	chr19	52725000	8.97E-03	super_enhancer	promoter	MIR6801
chr19	15285000	chr19	54135000	1.11E-02	super_enhancer	promoter	DPRX
chr19	15285000	chr19	57155000	8.99E-03	super_enhancer	promoter	SMIM17
chr19	15295000	chr19	35485000	2.12E-02	super_enhancer	promoter	GRAMD1A
chr19	15295000	chr19	39575000	1.98E-02	super_enhancer	promoter	PAPL
chr19	15295000	chr19	40135000	1.91E-02	super_enhancer	promoter	AC005176.3
chr19	15295000	chr19	57875000	1.08E-02	super_enhancer	promoter	ZNF547
chr19	15305000	chr19	15345000	1.86E-02	super_enhancer	promoter	EPHX3
chr19	15305000	chr19	15495000	2.32E-03	super_enhancer	promoter	AC005785.5
chr19	15305000	chr19	15575000	1.21E-02	super_enhancer	promoter	RASAL3
chr19	15305000	chr19	15675000	1.21E-05	super_enhancer	promoter	CYP4F23P

chr19	15305000	chr19	21105000	2.79E-02	super_enhancer	promoter	ZNF85
chr19	15305000	chr19	28475000	2.00E-02	super_enhancer	promoter	AC005357.1
chr19	15305000	chr19	39575000	2.08E-02	super_enhancer	promoter	PAPL
chr19	15305000	chr19	44615000	1.49E-02	super_enhancer	promoter	ZNF225
chr19	15305000	chr19	48825000	1.11E-02	super_enhancer	promoter	CCDC114
chr19	15315000	chr19	36265000	1.58E-02	super_enhancer	promoter	ARHGAP33
chr19	15315000	chr19	38795000	1.49E-02	super_enhancer	promoter	C19orf33
chr19	15315000	chr19	39615000	1.59E-02	super_enhancer	promoter	PAK4
chr19	15315000	chr19	39695000	1.65E-02	super_enhancer	promoter	SYCN
chr19	15315000	chr19	41905000	1.68E-02	super_enhancer	promoter	CTC-435M10.10
chr19	15315000	chr19	49055000	8.44E-03	super_enhancer	promoter	SULT2B1
chr19	15325000	chr19	16295000	4.60E-02	super_enhancer	promoter	FAM32A
chr19	15325000	chr19	38875000	1.80E-02	super_enhancer	promoter	AC005789.9
chr19	15325000	chr19	39755000	2.19E-02	super_enhancer	promoter	IFNL4P1
chr19	15325000	chr19	42055000	1.68E-02	super_enhancer	promoter	CEACAM21
chr19	15345000	chr19	40805000	1.48E-02	promoter	super_enhancer	EPHX3
chr19	15495000	chr19	38725000	1.83E-02	promoter	super_enhancer	AC005785.5
chr19	15495000	chr19	43545000	7.29E-03	promoter	super_enhancer	AC005785.5
chr19	15495000	chr19	45425000	1.53E-02	promoter	super_enhancer	AC005785.5
chr19	15675000	chr19	17075000	4.07E-02	promoter	super_enhancer	CYP4F23P
chr19	15725000	chr19	16075000	3.96E-02	promoter	super_enhancer	AD000091.2
chr19	15725000	chr19	16595000	4.15E-02	promoter	super_enhancer	AD000091.2
chr19	15725000	chr19	18685000	6.09E-03	promoter	super_enhancer	AD000091.2
chr19	15725000	chr19	38675000	1.62E-02	promoter	super_enhancer	AD000091.2

chr19	15725000	chr19	38685000	1.58E-02	promoter	super_enhancer	AD000091.2
chr19	15725000	chr19	38715000	1.53E-02	promoter	super_enhancer	AD000091.2
chr19	15725000	chr19	38735000	1.63E-02	promoter	super_enhancer	AD000091.2
chr19	15775000	chr19	38655000	1.43E-02	promoter	super_enhancer	CYP4F10P
chr19	15775000	chr19	38665000	1.41E-02	promoter	super_enhancer	CYP4F10P
chr19	15775000	chr19	38695000	1.41E-02	promoter	super_enhancer	CYP4F10P
chr19	15775000	chr19	38725000	1.38E-02	promoter	super_enhancer	CYP4F10P
chr19	15775000	chr19	38735000	1.39E-02	promoter	super_enhancer	CYP4F10P
chr19	15775000	chr19	45785000	1.38E-02	promoter	super_enhancer	CYP4F10P
chr19	15775000	chr19	48575000	6.17E-03	promoter	super_enhancer	CYP4F10P
chr19	15905000	chr19	33065000	2.65E-02	promoter	super_enhancer	OR10H5
chr19	15935000	chr19	17795000	2.35E-02	promoter	super_enhancer	ZNF861P
chr19	16015000	chr19	39055000	1.21E-02	promoter	super_enhancer	AC005336.5
chr19	16075000	chr19	45445000	1.42E-02	super_enhancer	promoter	APOC4
chr19	16075000	chr19	55845000	9.52E-03	super_enhancer	promoter	TMEM150B
chr19	16075000	chr19	57155000	8.00E-03	super_enhancer	promoter	SMIM17
chr19	16075000	chr19	58135000	6.14E-03	super_enhancer	promoter	AC003682.16
chr19	16075000	chr19	58175000	7.70E-03	super_enhancer	promoter	AC003682.17
chr19	16085000	chr19	34515000	1.24E-02	super_enhancer	promoter	RPS4XP20
chr19	16085000	chr19	36605000	1.75E-02	super_enhancer	promoter	TBCB
chr19	16085000	chr19	49865000	8.30E-03	super_enhancer	promoter	DKKL1
chr19	16085000	chr19	53935000	8.51E-03	super_enhancer	promoter	TPM3P9
chr19	16085000	chr19	54695000	8.21E-03	super_enhancer	promoter	MBOAT7
chr19	16125000	chr19	40595000	2.35E-02	promoter	super_enhancer	LINC00661

chr19	16125000	chr19	40625000	1.87E-02	promoter	super_enhancer	LINC00661
chr19	16125000	chr19	40715000	2.42E-02	promoter	super_enhancer	LINC00661
chr19	16125000	chr19	40785000	2.31E-02	promoter	super_enhancer	LINC00661
chr19	16125000	chr19	40795000	2.81E-02	promoter	super_enhancer	LINC00661
chr19	16125000	chr19	43445000	8.89E-03	promoter	super_enhancer	LINC00661
chr19	16125000	chr19	45565000	2.68E-02	promoter	super_enhancer	LINC00661
chr19	16125000	chr19	45585000	2.45E-02	promoter	super_enhancer	LINC00661
chr19	16125000	chr19	45785000	2.61E-02	promoter	super_enhancer	LINC00661
chr19	16215000	chr19	38295000	1.42E-02	promoter	super_enhancer	CTD-2231E14.5
chr19	16215000	chr19	40605000	1.62E-02	promoter	super_enhancer	CTD-2231E14.5
chr19	16215000	chr19	40625000	1.38E-02	promoter	super_enhancer	CTD-2231E14.5
chr19	16215000	chr19	43765000	1.23E-02	promoter	super_enhancer	CTD-2231E14.5
chr19	16245000	chr19	40615000	1.08E-02	promoter	super_enhancer	HSH2D
chr19	16245000	chr19	40745000	2.30E-02	promoter	super_enhancer	HSH2D
chr19	16245000	chr19	40815000	2.30E-02	promoter	super_enhancer	HSH2D
chr19	16245000	chr19	41225000	2.42E-02	promoter	super_enhancer	HSH2D
chr19	16245000	chr19	42115000	2.06E-02	promoter	super_enhancer	HSH2D
chr19	16245000	chr19	45585000	2.09E-02	promoter	super_enhancer	HSH2D
chr19	16245000	chr19	46215000	1.83E-02	promoter	super_enhancer	HSH2D
chr19	16285000	chr19	40425000	2.34E-02	promoter	super_enhancer	CIB3
chr19	16285000	chr19	40585000	1.60E-02	promoter	super_enhancer	CIB3
chr19	16285000	chr19	40595000	1.99E-02	promoter	super_enhancer	CIB3
chr19	16285000	chr19	40715000	2.13E-04	promoter	super_enhancer	CIB3
chr19	16285000	chr19	40745000	2.30E-02	promoter	super_enhancer	CIB3

chr19	16285000	chr19	40815000	2.30E-02	promoter	super_enhancer	CIB3
chr19	16285000	chr19	41245000	2.27E-02	promoter	super_enhancer	CIB3
chr19	16285000	chr19	43785000	1.27E-02	promoter	super_enhancer	CIB3
chr19	16285000	chr19	46215000	1.83E-02	promoter	super_enhancer	CIB3
chr19	16295000	chr19	18465000	2.73E-02	promoter	super_enhancer	FAM32A
chr19	16295000	chr19	40425000	2.32E-02	promoter	super_enhancer	FAM32A
chr19	16295000	chr19	40815000	2.28E-02	promoter	super_enhancer	FAM32A
chr19	16295000	chr19	41215000	2.41E-02	promoter	super_enhancer	FAM32A
chr19	16295000	chr19	41325000	2.09E-02	promoter	super_enhancer	FAM32A
chr19	16295000	chr19	47615000	1.27E-02	promoter	super_enhancer	FAM32A
chr19	16325000	chr19	38795000	1.65E-02	super_enhancer	promoter	C19orf33
chr19	16325000	chr19	40025000	1.27E-06	super_enhancer	promoter	TDGF1P7
chr19	16325000	chr19	40135000	1.85E-02	super_enhancer	promoter	AC005176.3
chr19	16325000	chr19	40185000	1.86E-02	super_enhancer	promoter	AC093063.2
chr19	16325000	chr19	40195000	2.03E-02	super_enhancer	promoter	LGALS14
chr19	16325000	chr19	40305000	1.98E-02	super_enhancer	promoter	LLNLR-268E12.1
chr19	16325000	chr19	41905000	1.86E-02	super_enhancer	promoter	CTC-435M10.10
chr19	16325000	chr19	42105000	1.74E-02	super_enhancer	promoter	CEACAMP3
chr19	16325000	chr19	42575000	1.42E-02	super_enhancer	promoter	GRIK5
chr19	16325000	chr19	44615000	1.37E-02	super_enhancer	promoter	ZNF225
chr19	16325000	chr19	45395000	1.42E-02	super_enhancer	promoter	CTB-129P6.4
chr19	16345000	chr19	16465000	4.98E-02	super_enhancer	promoter	CTD-2013N17.7
chr19	16345000	chr19	36265000	1.93E-02	super_enhancer	promoter	ARHGAP33
chr19	16345000	chr19	39265000	1.35E-02	super_enhancer	promoter	LGALS7

chr19	16345000	chr19	40305000	2.17E-02	super_enhancer	promoter	LLNLR-268E12.1
chr19	16345000	chr19	40725000	2.11E-02	super_enhancer	promoter	TTC9B
chr19	16345000	chr19	41905000	2.03E-02	super_enhancer	promoter	CTC-435M10.10
chr19	16345000	chr19	42855000	1.81E-02	super_enhancer	promoter	MIR8077
chr19	16345000	chr19	43065000	2.31E-02	super_enhancer	promoter	CEACAM1
chr19	16345000	chr19	58125000	1.14E-02	super_enhancer	promoter	ZNF134
chr19	16345000	chr19	58995000	8.79E-03	super_enhancer	promoter	RNU6-1337P
chr19	16355000	chr19	40025000	2.32E-02	super_enhancer	promoter	TDGF1P7
chr19	16355000	chr19	40195000	2.92E-04	super_enhancer	promoter	LGALS14
chr19	16355000	chr19	41305000	1.56E-02	super_enhancer	promoter	EGLN2
chr19	16355000	chr19	41345000	1.65E-02	super_enhancer	promoter	CTC-490E21.11
chr19	16355000	chr19	41905000	2.20E-02	super_enhancer	promoter	CTC-435M10.10
chr19	16355000	chr19	42415000	1.97E-02	super_enhancer	promoter	CTD-2575K13.6
chr19	16355000	chr19	45595000	2.27E-02	super_enhancer	promoter	CTB-179K24.3
chr19	16355000	chr19	45655000	2.23E-02	super_enhancer	promoter	AC005757.7
chr19	16355000	chr19	46735000	1.93E-02	super_enhancer	promoter	AC006262.10
chr19	16355000	chr19	55385000	1.15E-02	super_enhancer	promoter	FCAR
chr19	16435000	chr19	40605000	1.94E-04	promoter	super_enhancer	CTD-2562J15.6
chr19	16435000	chr19	40745000	2.43E-02	promoter	super_enhancer	CTD-2562J15.6
chr19	16435000	chr19	40825000	2.31E-02	promoter	super_enhancer	CTD-2562J15.6
chr19	16435000	chr19	43195000	2.17E-02	promoter	super_enhancer	CTD-2562J15.6
chr19	16435000	chr19	45565000	2.41E-02	promoter	super_enhancer	CTD-2562J15.6
chr19	16465000	chr19	38645000	2.13E-02	promoter	super_enhancer	CTD-2013N17.7
chr19	16465000	chr19	39055000	2.10E-02	promoter	super_enhancer	CTD-2013N17.7

chr19	16465000	chr19	40595000	1.90E-02	promoter	super_enhancer	CTD-2013N17.7
chr19	16465000	chr19	40825000	2.08E-02	promoter	super_enhancer	CTD-2013N17.7
chr19	16465000	chr19	46195000	1.75E-02	promoter	super_enhancer	CTD-2013N17.7
chr19	16465000	chr19	48875000	1.21E-02	promoter	super_enhancer	CTD-2013N17.7
chr19	16575000	chr19	18385000	4.31E-02	super_enhancer	promoter	KIAA1683
chr19	16575000	chr19	40025000	2.18E-06	super_enhancer	promoter	TDGF1P7
chr19	16575000	chr19	40075000	1.98E-02	super_enhancer	promoter	CTB-60E11.12
chr19	16575000	chr19	40135000	2.22E-02	super_enhancer	promoter	AC005176.3
chr19	16575000	chr19	40175000	2.02E-02	super_enhancer	promoter	AC093063.3
chr19	16575000	chr19	40305000	2.84E-04	super_enhancer	promoter	LLNLR-268E12.1
chr19	16575000	chr19	44555000	1.47E-02	super_enhancer	promoter	ZNF223
chr19	16575000	chr19	45075000	1.94E-02	super_enhancer	promoter	AC138472.4
chr19	16575000	chr19	46155000	1.96E-02	super_enhancer	promoter	RN7SL836P
chr19	16585000	chr19	34585000	2.48E-02	super_enhancer	promoter	RPS4XP21
chr19	16585000	chr19	40025000	3.45E-04	super_enhancer	promoter	TDGF1P7
chr19	16585000	chr19	40175000	2.26E-02	super_enhancer	promoter	AC093063.3
chr19	16585000	chr19	40195000	2.71E-02	super_enhancer	promoter	LGALS14
chr19	16585000	chr19	40855000	2.27E-02	super_enhancer	promoter	C19orf47
chr19	16585000	chr19	41305000	1.76E-02	super_enhancer	promoter	EGLN2
chr19	16585000	chr19	41535000	2.49E-02	super_enhancer	promoter	CYP2A7P1
chr19	16585000	chr19	44605000	1.91E-02	super_enhancer	promoter	AC084219.3
chr19	16585000	chr19	50355000	1.35E-02	super_enhancer	promoter	PTOV1-AS1
chr19	16585000	chr19	55485000	1.27E-02	super_enhancer	promoter	CTC-550B14.1
chr19	16585000	chr19	56165000	1.46E-02	super_enhancer	promoter	U2AF2

chr19	16595000	chr19	34895000	2.70E-02	super_enhancer	promoter	PDCD2L
chr19	16595000	chr19	40075000	2.12E-02	super_enhancer	promoter	CTB-60E11.12
chr19	16595000	chr19	40135000	2.37E-02	super_enhancer	promoter	AC005176.3
chr19	16595000	chr19	40195000	2.60E-02	super_enhancer	promoter	LGALS14
chr19	16595000	chr19	41535000	2.39E-02	super_enhancer	promoter	CYP2A7P1
chr19	16595000	chr19	42415000	2.13E-02	super_enhancer	promoter	CTD-2575K13.6
chr19	16595000	chr19	44575000	1.97E-02	super_enhancer	promoter	ZNF284
chr19	16595000	chr19	47355000	1.70E-02	super_enhancer	promoter	AP2S1
chr19	16595000	chr19	50365000	1.34E-02	super_enhancer	promoter	AC018766.4
chr19	16595000	chr19	55085000	1.38E-02	super_enhancer	promoter	LILRA1
chr19	16605000	chr19	34515000	1.84E-02	super_enhancer	promoter	RPS4XP20
chr19	16605000	chr19	38865000	2.12E-02	super_enhancer	promoter	PSMD8
chr19	16605000	chr19	40135000	2.15E-02	super_enhancer	promoter	AC005176.3
chr19	16605000	chr19	40185000	2.16E-02	super_enhancer	promoter	AC093063.2
chr19	16605000	chr19	40875000	2.51E-02	super_enhancer	promoter	MIR6796
chr19	16605000	chr19	41305000	1.53E-02	super_enhancer	promoter	EGLN2
chr19	16605000	chr19	44615000	1.59E-02	super_enhancer	promoter	ZNF225
chr19	16675000	chr19	40585000	1.52E-02	promoter	super_enhancer	CTD-3222D19.10
chr19	16675000	chr19	40605000	1.77E-02	promoter	super_enhancer	CTD-3222D19.10
chr19	16675000	chr19	43515000	7.71E-03	promoter	super_enhancer	CTD-3222D19.10
chr19	16675000	chr19	46795000	1.49E-02	promoter	super_enhancer	CTD-3222D19.10
chr19	16955000	chr19	17075000	3.07E-02	promoter	super_enhancer	CTD-2538G9.5
chr19	16955000	chr19	17115000	1.55E-02	promoter	super_enhancer	CTD-2538G9.5
chr19	16955000	chr19	17315000	1.16E-03	promoter	super_enhancer	CTD-2538G9.5

chr19	16955000	chr19	40815000	2.40E-02	promoter	super_enhancer	CTD-2538G9.5
chr19	17075000	chr19	35135000	1.81E-02	super_enhancer	promoter	CTD-2553L13.5
chr19	17075000	chr19	36485000	1.90E-02	super_enhancer	promoter	SDHAF1
chr19	17075000	chr19	36605000	2.11E-02	super_enhancer	promoter	TBCB
chr19	17075000	chr19	40725000	1.82E-02	super_enhancer	promoter	TTC9B
chr19	17085000	chr19	21105000	4.55E-02	super_enhancer	promoter	ZNF85
chr19	17085000	chr19	41305000	1.64E-02	super_enhancer	promoter	EGLN2
chr19	17085000	chr19	42805000	2.10E-02	super_enhancer	promoter	PRR19
chr19	17085000	chr19	45165000	2.35E-02	super_enhancer	promoter	CEACAM19
chr19	17085000	chr19	45505000	2.18E-02	super_enhancer	promoter	RELB
chr19	17085000	chr19	46105000	2.02E-04	super_enhancer	promoter	OPA3
chr19	17085000	chr19	54535000	1.21E-02	super_enhancer	promoter	CTD-3093M3.1
chr19	17085000	chr19	55085000	1.34E-02	super_enhancer	promoter	LILRA1
chr19	17085000	chr19	59025000	1.41E-02	super_enhancer	promoter	SLC27A5
chr19	17095000	chr19	17395000	3.20E-02	super_enhancer	promoter	USHBP1
chr19	17095000	chr19	35935000	1.89E-02	super_enhancer	promoter	FFAR2
chr19	17095000	chr19	39755000	2.38E-02	super_enhancer	promoter	IFNL4P1
chr19	17095000	chr19	40195000	2.40E-02	super_enhancer	promoter	LGALS14
chr19	17095000	chr19	41935000	2.11E-02	super_enhancer	promoter	B3GNT8
chr19	17105000	chr19	36055000	2.39E-02	super_enhancer	promoter	ATP4A
chr19	17105000	chr19	39695000	2.43E-02	super_enhancer	promoter	SYCN
chr19	17105000	chr19	40195000	2.71E-02	super_enhancer	promoter	LGALS14
chr19	17105000	chr19	42145000	2.15E-02	super_enhancer	promoter	DNAJC19P3
chr19	17105000	chr19	42585000	2.23E-02	super_enhancer	promoter	CTB-59C6.3

chr19	17105000	chr19	46235000	2.44E-02	super_enhancer	promoter	FBXO46
chr19	17105000	chr19	54225000	1.64E-02	super_enhancer	promoter	MIR520G
chr19	17115000	chr19	36705000	2.13E-02	super_enhancer	promoter	ZNF146
chr19	17115000	chr19	40075000	2.27E-02	super_enhancer	promoter	CTB-60E11.12
chr19	17115000	chr19	40135000	2.54E-02	super_enhancer	promoter	AC005176.3
chr19	17115000	chr19	40185000	2.55E-02	super_enhancer	promoter	AC093063.2
chr19	17115000	chr19	40305000	3.42E-06	super_enhancer	promoter	LLNLR-268E12.1
chr19	17115000	chr19	44175000	2.34E-02	super_enhancer	promoter	PLAUR
chr19	17115000	chr19	45035000	2.39E-02	super_enhancer	promoter	CEACAM20
chr19	17115000	chr19	45875000	2.41E-02	super_enhancer	promoter	ERCC2
chr19	17125000	chr19	38825000	2.21E-02	super_enhancer	promoter	CATSPERG
chr19	17125000	chr19	40075000	2.03E-02	super_enhancer	promoter	CTB-60E11.12
chr19	17125000	chr19	42415000	2.04E-02	super_enhancer	promoter	CTD-2575K13.6
chr19	17125000	chr19	47975000	1.41E-02	super_enhancer	promoter	SLC8A2
chr19	17125000	chr19	58845000	9.22E-03	super_enhancer	promoter	MIR6806
chr19	17135000	chr19	40075000	2.26E-02	super_enhancer	promoter	CTB-60E11.12
chr19	17135000	chr19	40875000	2.94E-02	super_enhancer	promoter	MIR6796
chr19	17135000	chr19	41725000	2.75E-02	super_enhancer	promoter	AXL
chr19	17135000	chr19	54705000	1.35E-02	super_enhancer	promoter	AC012314.20
chr19	17135000	chr19	59055000	7.19E-03	super_enhancer	promoter	RN7SL525P
chr19	17315000	chr19	17325000	2.49E-02	super_enhancer	promoter	USE1
chr19	17315000	chr19	41725000	2.16E-02	super_enhancer	promoter	AXL
chr19	17315000	chr19	45875000	1.88E-02	super_enhancer	promoter	ERCC2
chr19	17325000	chr19	38675000	2.72E-02	promoter	super_enhancer	USE1

chr19	17325000	chr19	38725000	2.71E-02	promoter	super_enhancer	USE1
chr19	17325000	chr19	39405000	2.90E-02	promoter	super_enhancer	USE1
chr19	17325000	chr19	40585000	1.95E-02	promoter	super_enhancer	USE1
chr19	17325000	chr19	41225000	2.94E-02	promoter	super_enhancer	USE1
chr19	17325000	chr19	45555000	2.32E-02	promoter	super_enhancer	USE1
chr19	17395000	chr19	19385000	3.79E-03	promoter	super_enhancer	USHBP1
chr19	17395000	chr19	41225000	2.98E-02	promoter	super_enhancer	USHBP1
chr19	17395000	chr19	45405000	2.35E-02	promoter	super_enhancer	USHBP1
chr19	17445000	chr19	18465000	1.49E-02	promoter	super_enhancer	GTPBP3
chr19	17445000	chr19	40595000	2.43E-02	promoter	super_enhancer	GTPBP3
chr19	17445000	chr19	45575000	2.36E-02	promoter	super_enhancer	GTPBP3
chr19	17565000	chr19	38645000	2.49E-02	promoter	super_enhancer	CTD-2521M24.11
chr19	17565000	chr19	38695000	2.53E-02	promoter	super_enhancer	CTD-2521M24.11
chr19	17565000	chr19	38715000	2.80E-04	promoter	super_enhancer	CTD-2521M24.11
chr19	17565000	chr19	40425000	2.60E-02	promoter	super_enhancer	CTD-2521M24.11
chr19	17565000	chr19	45555000	2.12E-02	promoter	super_enhancer	CTD-2521M24.11
chr19	17565000	chr19	45565000	2.52E-02	promoter	super_enhancer	CTD-2521M24.11
chr19	17665000	chr19	43765000	1.27E-02	promoter	super_enhancer	COLGALT1
chr19	17665000	chr19	45575000	1.75E-02	promoter	super_enhancer	COLGALT1
chr19	17665000	chr19	45715000	1.83E-02	promoter	super_enhancer	COLGALT1
chr19	17765000	chr19	41815000	2.07E-02	super_enhancer	promoter	CCDC97
chr19	17765000	chr19	45875000	2.19E-02	super_enhancer	promoter	ERCC2
chr19	17765000	chr19	47975000	1.43E-02	super_enhancer	promoter	SLC8A2
chr19	17765000	chr19	48615000	1.26E-02	super_enhancer	promoter	PLA2G4C

chr19	17765000	chr19	53485000	9.36E-03	super_enhancer	promoter	CTD-2620I22.1
chr19	17765000	chr19	55955000	1.25E-02	super_enhancer	promoter	SHISA7
chr19	17775000	chr19	40135000	2.20E-02	super_enhancer	promoter	AC005176.3
chr19	17775000	chr19	40195000	2.41E-02	super_enhancer	promoter	LGALS14
chr19	17775000	chr19	41305000	1.56E-02	super_enhancer	promoter	EGLN2
chr19	17775000	chr19	42465000	2.19E-02	super_enhancer	promoter	RABAC1
chr19	17775000	chr19	42855000	1.95E-02	super_enhancer	promoter	MIR8077
chr19	17775000	chr19	44125000	2.00E-02	super_enhancer	promoter	ZNF428
chr19	17775000	chr19	44395000	1.41E-02	super_enhancer	promoter	RP11-15A1.4
chr19	17775000	chr19	46145000	1.52E-02	super_enhancer	promoter	EML2-AS1
chr19	17775000	chr19	51685000	1.01E-02	super_enhancer	promoter	CTD-3187F8.14
chr19	17785000	chr19	22785000	3.09E-02	super_enhancer	promoter	AC011467.1
chr19	17785000	chr19	41725000	2.52E-02	super_enhancer	promoter	AXL
chr19	17785000	chr19	44085000	2.09E-02	super_enhancer	promoter	XRCC1
chr19	17785000	chr19	44575000	1.91E-02	super_enhancer	promoter	ZNF284
chr19	17785000	chr19	51645000	1.32E-02	super_enhancer	promoter	SIGLEC7
chr19	17795000	chr19	29125000	2.43E-02	super_enhancer	promoter	AC005616.1
chr19	17795000	chr19	30095000	2.73E-02	super_enhancer	promoter	POP4
chr19	17795000	chr19	36485000	2.39E-02	super_enhancer	promoter	SDHAF1
chr19	17795000	chr19	39575000	2.29E-02	super_enhancer	promoter	PAPL
chr19	17795000	chr19	40855000	2.01E-02	super_enhancer	promoter	C19orf47
chr19	17795000	chr19	44455000	1.53E-02	super_enhancer	promoter	ZNF221
chr19	17795000	chr19	45395000	1.67E-02	super_enhancer	promoter	CTB-129P6.4
chr19	17845000	chr19	38655000	2.83E-02	promoter	super_enhancer	CTD-3149D2.4

chr19	17845000	chr19	43525000	1.28E-02	promoter	super_enhancer	CTD-3149D2.4
chr19	17905000	chr19	18005000	3.10E-02	promoter	super_enhancer	B3GNT3
chr19	17905000	chr19	40745000	2.72E-02	promoter	super_enhancer	B3GNT3
chr19	17905000	chr19	45585000	2.46E-02	promoter	super_enhancer	B3GNT3
chr19	18005000	chr19	38825000	2.28E-02	super_enhancer	promoter	CATSPERG
chr19	18005000	chr19	40135000	2.35E-02	super_enhancer	promoter	AC005176.3
chr19	18005000	chr19	44555000	1.55E-02	super_enhancer	promoter	ZNF223
chr19	18005000	chr19	49245000	1.33E-02	super_enhancer	promoter	RASIP1
chr19	18005000	chr19	50975000	1.25E-02	super_enhancer	promoter	Clostridiales-1
chr19	18005000	chr19	52395000	9.70E-03	super_enhancer	promoter	ZNF577
chr19	18015000	chr19	41815000	1.91E-02	super_enhancer	promoter	CCDC97
chr19	18015000	chr19	42055000	1.78E-02	super_enhancer	promoter	CEACAM21
chr19	18015000	chr19	44615000	1.57E-02	super_enhancer	promoter	ZNF225
chr19	18365000	chr19	38545000	3.05E-02	super_enhancer	promoter	AC011465.1
chr19	18365000	chr19	38865000	2.47E-02	super_enhancer	promoter	PSMD8
chr19	18365000	chr19	40185000	2.50E-02	super_enhancer	promoter	AC093063.2
chr19	18365000	chr19	44125000	2.26E-02	super_enhancer	promoter	ZNF428
chr19	18365000	chr19	45395000	1.89E-02	super_enhancer	promoter	CTB-129P6.4
chr19	18365000	chr19	45595000	2.55E-02	super_enhancer	promoter	CTB-179K24.3
chr19	18365000	chr19	47355000	1.77E-02	super_enhancer	promoter	AP2S1
chr19	18365000	chr19	55875000	1.46E-02	super_enhancer	promoter	FAM71E2
chr19	18365000	chr19	58875000	1.28E-02	super_enhancer	promoter	CTD-2619J13.8
chr19	18375000	chr19	18435000	1.01E-02	super_enhancer	promoter	LSM4
chr19	18375000	chr19	40175000	2.18E-02	super_enhancer	promoter	AC093063.3

chr19	18375000	chr19	54205000	1.36E-02	super_enhancer	promoter	MIR518B
chr19	18375000	chr19	55265000	7.53E-03	super_enhancer	promoter	KIR2DP1
chr19	18385000	chr19	18415000	1.09E-02	promoter	super_enhancer	KIAA1683
chr19	18385000	chr19	18455000	3.56E-02	promoter	super_enhancer	KIAA1683
chr19	18385000	chr19	18505000	2.63E-03	promoter	super_enhancer	KIAA1683
chr19	18385000	chr19	40605000	1.93E-02	promoter	super_enhancer	KIAA1683
chr19	18385000	chr19	41215000	2.53E-02	promoter	super_enhancer	KIAA1683
chr19	18385000	chr19	45425000	1.91E-02	promoter	super_enhancer	KIAA1683
chr19	18415000	chr19	36055000	2.55E-02	super_enhancer	promoter	ATP4A
chr19	18415000	chr19	36645000	2.28E-02	super_enhancer	promoter	COX7A1
chr19	18415000	chr19	38865000	2.59E-02	super_enhancer	promoter	PSMD8
chr19	18415000	chr19	40185000	2.62E-02	super_enhancer	promoter	AC093063.2
chr19	18415000	chr19	41815000	2.34E-02	super_enhancer	promoter	CCDC97
chr19	18415000	chr19	41935000	2.51E-02	super_enhancer	promoter	B3GNT8
chr19	18415000	chr19	42895000	2.88E-02	super_enhancer	promoter	CNFN
chr19	18415000	chr19	44125000	2.37E-02	super_enhancer	promoter	ZNF428
chr19	18415000	chr19	45395000	1.98E-02	super_enhancer	promoter	CTB-129P6.4
chr19	18435000	chr19	36135000	2.89E-02	promoter	super_enhancer	LSM4
chr19	18435000	chr19	38655000	2.71E-02	promoter	super_enhancer	LSM4
chr19	18435000	chr19	45715000	2.37E-02	promoter	super_enhancer	LSM4
chr19	18435000	chr19	48335000	1.40E-02	promoter	super_enhancer	LSM4
chr19	18435000	chr19	55545000	1.45E-02	promoter	super_enhancer	LSM4
chr19	18455000	chr19	34045000	3.61E-02	super_enhancer	promoter	CTD-2329C7.2
chr19	18455000	chr19	43245000	1.67E-02	super_enhancer	promoter	CEACAM8

chr19	18455000	chr19	44145000	2.64E-02	super_enhancer	promoter	CADM4
chr19	18455000	chr19	48615000	1.41E-02	super_enhancer	promoter	PLA2G4C
chr19	18455000	chr19	49055000	1.29E-02	super_enhancer	promoter	SULT2B1
chr19	18455000	chr19	50145000	1.35E-02	super_enhancer	promoter	SCAF1
chr19	18455000	chr19	52645000	1.05E-02	super_enhancer	promoter	CTC-471J1.9
chr19	18465000	chr19	30415000	2.74E-02	super_enhancer	promoter	URI1
chr19	18465000	chr19	38545000	2.93E-02	super_enhancer	promoter	AC011465.1
chr19	18465000	chr19	39575000	2.49E-02	super_enhancer	promoter	PAPL
chr19	18465000	chr19	42465000	2.37E-02	super_enhancer	promoter	RABAC1
chr19	18465000	chr19	45505000	2.24E-02	super_enhancer	promoter	RELB
chr19	18475000	chr19	29705000	2.72E-02	super_enhancer	promoter	UQCRFS1
chr19	18475000	chr19	36665000	2.43E-02	super_enhancer	promoter	AC002984.2
chr19	18475000	chr19	38545000	3.11E-02	super_enhancer	promoter	AC011465.1
chr19	18475000	chr19	38795000	2.27E-02	super_enhancer	promoter	C19orf33
chr19	18475000	chr19	44605000	1.94E-02	super_enhancer	promoter	AC084219.3
chr19	18475000	chr19	46235000	2.49E-02	super_enhancer	promoter	FBXO46
chr19	18485000	chr19	33795000	3.36E-02	super_enhancer	promoter	CTD-2540B15.9
chr19	18485000	chr19	42255000	2.01E-02	super_enhancer	promoter	CEACAM6
chr19	18485000	chr19	42805000	2.21E-02	super_enhancer	promoter	PRR19
chr19	18485000	chr19	44645000	1.78E-02	super_enhancer	promoter	ZNF234
chr19	18485000	chr19	45875000	2.31E-02	super_enhancer	promoter	ERCC2
chr19	18485000	chr19	46145000	1.69E-02	super_enhancer	promoter	EML2-AS1
chr19	18485000	chr19	47105000	1.76E-02	super_enhancer	promoter	PPP5D1
chr19	18485000	chr19	50335000	1.32E-02	super_enhancer	promoter	MIR6800

chr19	18485000	chr19	55995000	1.35E-02	super_enhancer	promoter	NAT14
chr19	18495000	chr19	36055000	2.44E-02	super_enhancer	promoter	ATP4A
chr19	18495000	chr19	36105000	2.94E-02	super_enhancer	promoter	AC002115.9
chr19	18495000	chr19	38545000	3.07E-02	super_enhancer	promoter	AC011465.1
chr19	18495000	chr19	39305000	2.85E-02	super_enhancer	promoter	LGALS4
chr19	18495000	chr19	42725000	2.05E-02	super_enhancer	promoter	DEDD2
chr19	18495000	chr19	45125000	2.19E-02	super_enhancer	promoter	CEACAM22P
chr19	18495000	chr19	50555000	1.09E-02	super_enhancer	promoter	CTD-2126E3.3
chr19	18505000	chr19	39185000	2.16E-02	super_enhancer	promoter	CTB-186G2.4
chr19	18505000	chr19	40175000	2.25E-02	super_enhancer	promoter	AC093063.3
chr19	18505000	chr19	40195000	2.70E-02	super_enhancer	promoter	LGALS14
chr19	18505000	chr19	50935000	1.25E-02	super_enhancer	promoter	MYBPC2
chr19	18515000	chr19	38795000	2.34E-02	super_enhancer	promoter	C19orf33
chr19	18515000	chr19	38865000	2.59E-02	super_enhancer	promoter	PSMD8
chr19	18515000	chr19	38875000	2.78E-04	super_enhancer	promoter	AC005789.9
chr19	18515000	chr19	40875000	3.05E-02	super_enhancer	promoter	MIR6796
chr19	18515000	chr19	44145000	2.66E-02	super_enhancer	promoter	CADM4
chr19	18515000	chr19	50335000	1.41E-02	super_enhancer	promoter	MIR6800
chr19	18655000	chr19	40425000	2.48E-02	promoter	super_enhancer	FKBP8
chr19	18655000	chr19	41325000	2.51E-04	promoter	super_enhancer	FKBP8
chr19	18655000	chr19	44755000	1.47E-02	promoter	super_enhancer	FKBP8
chr19	18665000	chr19	36525000	3.13E-02	super_enhancer	promoter	CLIP3
chr19	18665000	chr19	38875000	2.27E-02	super_enhancer	promoter	AC005789.9
chr19	18665000	chr19	40025000	2.67E-02	super_enhancer	promoter	TDGF1P7

chr19	18665000	chr19	43775000	1.44E-02	super_enhancer	promoter	PSG9
chr19	18665000	chr19	44175000	2.31E-02	super_enhancer	promoter	PLAUR
chr19	18665000	chr19	45125000	2.21E-02	super_enhancer	promoter	CEACAM22P
chr19	18665000	chr19	45445000	2.20E-02	super_enhancer	promoter	APOC4
chr19	18665000	chr19	45655000	2.53E-02	super_enhancer	promoter	AC005757.7
chr19	18665000	chr19	45875000	2.38E-02	super_enhancer	promoter	ERCC2
chr19	18665000	chr19	55875000	1.48E-02	super_enhancer	promoter	FAM71E2
chr19	18675000	chr19	21705000	4.86E-02	super_enhancer	promoter	BNIP3P26
chr19	18675000	chr19	23785000	2.39E-02	super_enhancer	promoter	VN1R93P
chr19	18675000	chr19	34745000	2.38E-02	super_enhancer	promoter	KIAA0355
chr19	18675000	chr19	42895000	2.36E-02	super_enhancer	promoter	CNFN
chr19	18675000	chr19	44165000	1.94E-02	super_enhancer	promoter	RN7SL368P
chr19	18675000	chr19	44405000	1.76E-02	super_enhancer	promoter	RP11-15A1.2
chr19	18675000	chr19	45505000	2.01E-02	super_enhancer	promoter	RELB
chr19	18685000	chr19	34045000	3.27E-02	super_enhancer	promoter	CTD-2329C7.2
chr19	18685000	chr19	35265000	2.04E-02	super_enhancer	promoter	ZNF599
chr19	18685000	chr19	36055000	2.29E-02	super_enhancer	promoter	ATP4A
chr19	18685000	chr19	39185000	2.05E-02	super_enhancer	promoter	CTB-186G2.4
chr19	18685000	chr19	40855000	2.15E-02	super_enhancer	promoter	C19orf47
chr19	18685000	chr19	42145000	2.03E-02	super_enhancer	promoter	DNAJC19P3
chr19	18685000	chr19	46145000	1.62E-02	super_enhancer	promoter	EML2-AS1
chr19	19045000	chr19	38695000	4.17E-04	promoter	super_enhancer	AC002985.3
chr19	19045000	chr19	40795000	3.01E-02	promoter	super_enhancer	AC002985.3
chr19	19045000	chr19	41205000	2.89E-02	promoter	super_enhancer	AC002985.3

chr19	19045000	chr19	46205000	3.61E-04	promoter	super_enhancer	AC002985.3
chr19	19145000	chr19	38685000	2.39E-02	promoter	super_enhancer	SUGP2
chr19	19145000	chr19	38695000	2.48E-02	promoter	super_enhancer	SUGP2
chr19	19145000	chr19	38725000	2.99E-04	promoter	super_enhancer	SUGP2
chr19	19145000	chr19	38735000	2.46E-02	promoter	super_enhancer	SUGP2
chr19	19145000	chr19	48325000	1.39E-02	promoter	super_enhancer	SUGP2
chr19	19175000	chr19	38665000	2.52E-02	promoter	super_enhancer	SLC25A42
chr19	19175000	chr19	38695000	2.52E-02	promoter	super_enhancer	SLC25A42
chr19	19175000	chr19	39055000	2.44E-02	promoter	super_enhancer	SLC25A42
chr19	19175000	chr19	45425000	2.01E-02	promoter	super_enhancer	SLC25A42
chr19	19175000	chr19	45785000	2.41E-02	promoter	super_enhancer	SLC25A42
chr19	19175000	chr19	46215000	1.99E-02	promoter	super_enhancer	SLC25A42
chr19	19315000	chr19	19395000	1.87E-02	promoter	super_enhancer	NR2C2AP
chr19	19315000	chr19	19585000	7.64E-03	promoter	super_enhancer	NR2C2AP
chr19	19315000	chr19	19595000	3.16E-02	promoter	super_enhancer	NR2C2AP
chr19	19315000	chr19	19605000	1.74E-02	promoter	super_enhancer	NR2C2AP
chr19	19315000	chr19	33055000	3.17E-02	promoter	super_enhancer	NR2C2AP
chr19	19315000	chr19	38255000	1.83E-02	promoter	super_enhancer	NR2C2AP
chr19	19315000	chr19	38275000	1.87E-02	promoter	super_enhancer	NR2C2AP
chr19	19315000	chr19	41205000	2.27E-02	promoter	super_enhancer	NR2C2AP
chr19	19365000	chr19	32735000	3.29E-02	super_enhancer	promoter	RNA5SP472
chr19	19365000	chr19	34045000	3.88E-02	super_enhancer	promoter	CTD-2329C7.2
chr19	19365000	chr19	36235000	2.70E-02	super_enhancer	promoter	PSENEN
chr19	19365000	chr19	36305000	3.01E-02	super_enhancer	promoter	PRODH2

chr19	19365000	chr19	36395000	3.43E-02	super_enhancer	promoter	AD000864.6
chr19	19365000	chr19	36485000	3.04E-02	super_enhancer	promoter	SDHAF1
chr19	19365000	chr19	36665000	2.67E-02	super_enhancer	promoter	AC002984.2
chr19	19365000	chr19	36705000	2.35E-02	super_enhancer	promoter	ZNF146
chr19	19365000	chr19	38545000	3.41E-02	super_enhancer	promoter	AC011465.1
chr19	19365000	chr19	41535000	2.78E-02	super_enhancer	promoter	CYP2A7P1
chr19	19365000	chr19	42895000	3.04E-02	super_enhancer	promoter	CNFN
chr19	19375000	chr19	32835000	2.43E-02	super_enhancer	promoter	ZNF507
chr19	19375000	chr19	34045000	6.63E-06	super_enhancer	promoter	CTD-2329C7.2
chr19	19375000	chr19	36165000	2.61E-02	super_enhancer	promoter	UPK1A-AS1
chr19	19375000	chr19	36305000	2.62E-02	super_enhancer	promoter	PRODH2
chr19	19375000	chr19	38545000	2.97E-02	super_enhancer	promoter	AC011465.1
chr19	19375000	chr19	42895000	2.65E-02	super_enhancer	promoter	CNFN
chr19	19375000	chr19	46765000	1.78E-02	super_enhancer	promoter	IGFL1P2
chr19	19385000	chr19	30095000	2.74E-02	super_enhancer	promoter	POP4
chr19	19385000	chr19	36305000	2.36E-02	super_enhancer	promoter	PRODH2
chr19	19385000	chr19	36425000	2.50E-02	super_enhancer	promoter	LRFN3
chr19	19385000	chr19	36485000	2.85E-04	super_enhancer	promoter	SDHAF1
chr19	19385000	chr19	58175000	1.00E-02	super_enhancer	promoter	AC003682.17
chr19	19395000	chr19	33795000	3.07E-02	super_enhancer	promoter	CTD-2540B15.9
chr19	19395000	chr19	36235000	2.16E-02	super_enhancer	promoter	PSENEN
chr19	19395000	chr19	38545000	2.72E-02	super_enhancer	promoter	AC011465.1
chr19	19395000	chr19	54265000	1.40E-02	super_enhancer	promoter	CTC-339O9.1
chr19	19395000	chr19	54485000	1.41E-02	super_enhancer	promoter	MIR935

chr19	19395000	chr19	55155000	1.22E-02	super_enhancer	promoter	LILRB4
chr19	19395000	chr19	56145000	1.27E-02	super_enhancer	promoter	ZNF580
chr19	19585000	chr19	36105000	2.01E-02	super_enhancer	promoter	AC002115.9
chr19	19585000	chr19	36175000	1.74E-02	super_enhancer	promoter	AC002314.4
chr19	19595000	chr19	36645000	1.57E-02	super_enhancer	promoter	COX7A1
chr19	19595000	chr19	47115000	1.44E-02	super_enhancer	promoter	CTB-12A17.3
chr19	19595000	chr19	53795000	8.27E-03	super_enhancer	promoter	BIRC8
chr19	19615000	chr19	23255000	3.14E-02	super_enhancer	promoter	CTD-2291D10.3
chr19	19615000	chr19	44085000	1.62E-02	super_enhancer	promoter	XRCC1
chr19	19615000	chr19	45125000	1.57E-02	super_enhancer	promoter	CEACAM22P
chr19	19755000	chr19	58555000	1.18E-02	promoter	super_enhancer	GMIP
chr19	19775000	chr19	45425000	1.60E-02	promoter	super_enhancer	ATP13A1
chr19	19845000	chr19	56095000	1.13E-02	promoter	super_enhancer	ZNF14
chr19	20235000	chr19	45425000	1.24E-02	promoter	super_enhancer	CTC-260E6.2
chr19	21105000	chr19	55555000	6.03E-03	promoter	super_enhancer	ZNF85
chr19	21325000	chr19	38675000	9.54E-03	promoter	super_enhancer	ZNF431
chr19	21415000	chr19	45565000	9.71E-03	promoter	super_enhancer	RPL36AP51
chr19	22545000	chr19	35315000	8.04E-03	promoter	super_enhancer	CTB-31C7.3
chr19	22685000	chr19	46895000	9.05E-03	promoter	super_enhancer	AC004004.2
chr19	22685000	chr19	58555000	8.75E-03	promoter	super_enhancer	AC004004.2
chr19	23025000	chr19	38245000	9.74E-03	promoter	super_enhancer	BNIP3P35
chr19	23785000	chr19	46795000	7.05E-03	promoter	super_enhancer	VN1R93P
chr19	28285000	chr19	38715000	1.77E-02	promoter	super_enhancer	LINC00662
chr19	28285000	chr19	44745000	1.15E-02	promoter	super_enhancer	LINC00662

chr19	28285000	chr19	47605000	9.30E-03	promoter	super_enhancer	LINC00662
chr19	28285000	chr19	56105000	9.93E-03	promoter	super_enhancer	LINC00662
chr19	28285000	chr19	56115000	7.05E-03	promoter	super_enhancer	LINC00662
chr19	28475000	chr19	33055000	4.98E-02	promoter	super_enhancer	AC005357.1
chr19	28475000	chr19	39075000	2.19E-02	promoter	super_enhancer	AC005357.1
chr19	28555000	chr19	38655000	2.37E-02	promoter	super_enhancer	CTD-2043I16.1
chr19	28805000	chr19	33045000	4.80E-02	promoter	super_enhancer	CTB-70G10.1
chr19	29125000	chr19	55525000	1.12E-02	promoter	super_enhancer	AC005616.1
chr19	29285000	chr19	40785000	1.77E-02	promoter	super_enhancer	MAN1A2P1
chr19	29285000	chr19	48545000	9.25E-03	promoter	super_enhancer	MAN1A2P1
chr19	29455000	chr19	45585000	1.63E-02	promoter	super_enhancer	LINC00906
chr19	29705000	chr19	38725000	2.60E-02	promoter	super_enhancer	UQCRFS1
chr19	29705000	chr19	43765000	1.52E-02	promoter	super_enhancer	UQCRFS1
chr19	29705000	chr19	45575000	2.05E-02	promoter	super_enhancer	UQCRFS1
chr19	29725000	chr19	45725000	1.62E-02	promoter	super_enhancer	RN7SL340P
chr19	29725000	chr19	46205000	1.66E-02	promoter	super_enhancer	RN7SL340P
chr19	30095000	chr19	46885000	1.92E-02	promoter	super_enhancer	POP4
chr19	30155000	chr19	36135000	4.29E-02	promoter	super_enhancer	PLEKHF1
chr19	30155000	chr19	43525000	1.36E-02	promoter	super_enhancer	PLEKHF1
chr19	30155000	chr19	56105000	1.60E-02	promoter	super_enhancer	PLEKHF1
chr19	30415000	chr19	38265000	2.46E-02	promoter	super_enhancer	URI1
chr19	30415000	chr19	38665000	3.11E-02	promoter	super_enhancer	URI1
chr19	30415000	chr19	41255000	2.96E-02	promoter	super_enhancer	URI1
chr19	30415000	chr19	46865000	1.66E-02	promoter	super_enhancer	URI1

chr19	31045000	chr19	38725000	3.21E-02	promoter	super_enhancer	CTD-2001J20.1
chr19	31855000	chr19	38245000	3.14E-02	promoter	super_enhancer	CTC-439O9.1
chr19	31855000	chr19	56105000	1.78E-02	promoter	super_enhancer	CTC-439O9.1
chr19	32735000	chr19	38655000	4.89E-02	promoter	super_enhancer	RNA5SP472
chr19	32835000	chr19	33065000	1.28E-02	promoter	super_enhancer	ZNF507
chr19	32835000	chr19	38295000	3.16E-02	promoter	super_enhancer	ZNF507
chr19	32835000	chr19	45575000	2.55E-02	promoter	super_enhancer	ZNF507
chr19	32835000	chr19	45715000	2.66E-02	promoter	super_enhancer	ZNF507
chr19	32895000	chr19	38695000	4.50E-02	promoter	super_enhancer	DPY19L3
chr19	32895000	chr19	45575000	2.75E-02	promoter	super_enhancer	DPY19L3
chr19	33045000	chr19	37895000	3.47E-02	super_enhancer	promoter	CTD-2086O20.1
chr19	33045000	chr19	38185000	3.88E-02	super_enhancer	promoter	CTD-2528L19.3
chr19	33045000	chr19	39305000	4.50E-02	super_enhancer	promoter	LGALS4
chr19	33045000	chr19	54225000	1.79E-02	super_enhancer	promoter	MIR520G
chr19	33045000	chr19	56825000	1.36E-02	super_enhancer	promoter	AC006116.19
chr19	33055000	chr19	53795000	1.82E-02	super_enhancer	promoter	BIRC8
chr19	33055000	chr19	54665000	2.46E-02	super_enhancer	promoter	LENG1
chr19	33055000	chr19	55975000	1.85E-04	super_enhancer	promoter	ISOC2
chr19	33055000	chr19	56905000	1.79E-02	super_enhancer	promoter	ZNF582
chr19	33055000	chr19	56995000	1.78E-02	super_enhancer	promoter	AC098795.1
chr19	33065000	chr19	40025000	4.88E-02	super_enhancer	promoter	TDGF1P7
chr19	33065000	chr19	40725000	4.51E-02	super_enhancer	promoter	TTC9B
chr19	33065000	chr19	42575000	3.07E-02	super_enhancer	promoter	GRIK5
chr19	33065000	chr19	45445000	3.34E-02	super_enhancer	promoter	APOC4

chr19	33065000	chr19	46685000	2.86E-02	super_enhancer	promoter	AC006262.4
chr19	33065000	chr19	54205000	1.95E-02	super_enhancer	promoter	MIR518B
chr19	33075000	chr19	42105000	3.99E-02	super_enhancer	promoter	CEACAMP3
chr19	33075000	chr19	46735000	3.44E-02	super_enhancer	promoter	AC006262.10
chr19	33075000	chr19	51775000	1.35E-02	super_enhancer	promoter	CTD-3187F8.2
chr19	33075000	chr19	52255000	1.59E-02	super_enhancer	promoter	FPR2
chr19	33165000	chr19	40615000	2.70E-02	promoter	super_enhancer	RGS9BP
chr19	33165000	chr19	44745000	3.21E-02	promoter	super_enhancer	RGS9BP
chr19	33165000	chr19	46205000	4.54E-02	promoter	super_enhancer	RGS9BP
chr19	33165000	chr19	46215000	3.87E-02	promoter	super_enhancer	RGS9BP
chr19	33165000	chr19	46865000	2.94E-02	promoter	super_enhancer	RGS9BP
chr19	33165000	chr19	55525000	2.23E-02	promoter	super_enhancer	RGS9BP
chr19	33355000	chr19	40715000	4.58E-02	promoter	super_enhancer	RN7SKP22
chr19	33355000	chr19	42115000	4.19E-02	promoter	super_enhancer	RN7SKP22
chr19	33795000	chr19	43195000	4.22E-02	promoter	super_enhancer	CTD-2540B15.9
chr19	33795000	chr19	43535000	1.80E-02	promoter	super_enhancer	CTD-2540B15.9
chr19	33795000	chr19	47645000	2.37E-02	promoter	super_enhancer	CTD-2540B15.9
chr19	33795000	chr19	58555000	2.43E-02	promoter	super_enhancer	CTD-2540B15.9
chr19	34045000	chr19	43195000	4.39E-02	promoter	super_enhancer	CTD-2329C7.2
chr19	34045000	chr19	45405000	3.93E-02	promoter	super_enhancer	CTD-2329C7.2
chr19	34515000	chr19	36135000	2.27E-02	promoter	super_enhancer	RPS4XP20
chr19	34515000	chr19	38265000	4.93E-02	promoter	super_enhancer	RPS4XP20
chr19	34515000	chr19	56095000	1.63E-02	promoter	super_enhancer	RPS4XP20
chr19	34585000	chr19	46205000	5.64E-04	promoter	super_enhancer	RPS4XP21

chr19	34585000	chr19	46865000	2.16E-02	promoter	super_enhancer	RPS4XP21
chr19	34585000	chr19	55545000	1.72E-02	promoter	super_enhancer	RPS4XP21
chr19	34895000	chr19	40625000	4.04E-02	promoter	super_enhancer	PDCD2L
chr19	35025000	chr19	46895000	2.42E-02	promoter	super_enhancer	RPS26P55
chr19	35125000	chr19	38715000	1.69E-03	promoter	super_enhancer	CTD-2553L13.7
chr19	35265000	chr19	41225000	4.63E-02	promoter	super_enhancer	ZNF599
chr19	35305000	chr19	41225000	4.72E-02	promoter	super_enhancer	CTC-523E23.1
chr19	35305000	chr19	42115000	3.61E-02	promoter	super_enhancer	CTC-523E23.1
chr19	35315000	chr19	35325000	4.29E-04	super_enhancer	promoter	CTC-523E23.4
chr19	35315000	chr19	44605000	2.33E-02	super_enhancer	promoter	AC084219.3
chr19	35315000	chr19	50815000	1.47E-02	super_enhancer	promoter	CTB-191K22.5
chr19	35315000	chr19	55955000	1.38E-02	super_enhancer	promoter	SHISA7
chr19	35315000	chr19	56825000	1.30E-02	super_enhancer	promoter	AC006116.19
chr19	35395000	chr19	41265000	4.42E-02	promoter	super_enhancer	CTC-523E23.14
chr19	35395000	chr19	44755000	2.23E-02	promoter	super_enhancer	CTC-523E23.14
chr19	35455000	chr19	55525000	1.58E-02	promoter	super_enhancer	ZNF792
chr19	35485000	chr19	55525000	1.93E-02	promoter	super_enhancer	GRAMD1A
chr19	35645000	chr19	43535000	1.80E-02	promoter	super_enhancer	FXYD5
chr19	35725000	chr19	45565000	3.22E-02	promoter	super_enhancer	FAM187B2P
chr19	35725000	chr19	46875000	2.10E-02	promoter	super_enhancer	FAM187B2P
chr19	35835000	chr19	45565000	4.73E-02	promoter	super_enhancer	MIR5196
chr19	35835000	chr19	47735000	2.45E-02	promoter	super_enhancer	MIR5196
chr19	35935000	chr19	40595000	4.98E-02	promoter	super_enhancer	FFAR2
chr19	35935000	chr19	43195000	3.56E-02	promoter	super_enhancer	FFAR2

chr19	36005000	chr19	45585000	3.78E-02	promoter	super_enhancer	DMKN
chr19	36035000	chr19	43755000	1.80E-02	promoter	super_enhancer	TMEM147
chr19	36035000	chr19	48585000	3.13E-04	promoter	super_enhancer	TMEM147
chr19	36055000	chr19	44745000	2.65E-02	promoter	super_enhancer	ATP4A
chr19	36055000	chr19	56115000	1.46E-02	promoter	super_enhancer	ATP4A
chr19	36105000	chr19	46885000	3.12E-02	promoter	super_enhancer	AC002115.9
chr19	36105000	chr19	55545000	2.26E-02	promoter	super_enhancer	AC002115.9
chr19	36135000	chr19	44175000	4.33E-02	super_enhancer	promoter	PLAUR
chr19	36135000	chr19	44575000	7.39E-04	super_enhancer	promoter	ZNF284
chr19	36135000	chr19	45035000	4.23E-02	super_enhancer	promoter	CEACAM20
chr19	36135000	chr19	47355000	3.06E-02	super_enhancer	promoter	AP2S1
chr19	36135000	chr19	48675000	1.78E-02	super_enhancer	promoter	LIG1
chr19	36135000	chr19	48775000	2.53E-02	super_enhancer	promoter	CTC-241F20.4
chr19	36135000	chr19	55565000	2.09E-02	super_enhancer	promoter	CTC-550B14.6
chr19	36135000	chr19	56165000	2.20E-02	super_enhancer	promoter	U2AF2
chr19	36135000	chr19	56755000	1.64E-02	super_enhancer	promoter	ZSCAN5D
chr19	36135000	chr19	58175000	1.74E-02	super_enhancer	promoter	AC003682.17
chr19	36165000	chr19	45565000	4.40E-02	promoter	super_enhancer	UPK1A-AS1
chr19	36175000	chr19	48875000	2.20E-02	promoter	super_enhancer	AC002314.4
chr19	36175000	chr19	48965000	2.76E-02	promoter	super_enhancer	AC002314.4
chr19	36195000	chr19	38705000	7.11E-03	promoter	super_enhancer	ZBTB32
chr19	36195000	chr19	45725000	3.89E-02	promoter	super_enhancer	ZBTB32
chr19	36195000	chr19	47605000	2.22E-02	promoter	super_enhancer	ZBTB32
chr19	36245000	chr19	46195000	4.28E-02	promoter	super_enhancer	AC002398.11

chr19	36245000	chr19	47725000	2.72E-02	promoter	super_enhancer	AC002398.11
chr19	36265000	chr19	40585000	4.89E-02	promoter	super_enhancer	ARHGAP33
chr19	36265000	chr19	43785000	2.44E-02	promoter	super_enhancer	ARHGAP33
chr19	36265000	chr19	44755000	2.52E-02	promoter	super_enhancer	ARHGAP33
chr19	36265000	chr19	56105000	2.06E-02	promoter	super_enhancer	ARHGAP33
chr19	36265000	chr19	56115000	1.46E-02	promoter	super_enhancer	ARHGAP33
chr19	36305000	chr19	55525000	1.98E-02	promoter	super_enhancer	PRODH2
chr19	36395000	chr19	38655000	2.10E-02	promoter	super_enhancer	AD000864.6
chr19	36395000	chr19	43445000	1.98E-02	promoter	super_enhancer	AD000864.6
chr19	36395000	chr19	46795000	6.03E-04	promoter	super_enhancer	AD000864.6
chr19	36395000	chr19	55525000	2.27E-02	promoter	super_enhancer	AD000864.6
chr19	36395000	chr19	56095000	2.70E-02	promoter	super_enhancer	AD000864.6
chr19	36425000	chr19	38675000	1.76E-02	promoter	super_enhancer	LRFN3
chr19	36425000	chr19	39405000	4.49E-04	promoter	super_enhancer	LRFN3
chr19	36485000	chr19	39065000	7.07E-04	promoter	super_enhancer	SDHAF1
chr19	36485000	chr19	40805000	2.87E-03	promoter	super_enhancer	SDHAF1
chr19	36485000	chr19	42115000	1.63E-03	promoter	super_enhancer	SDHAF1
chr19	36485000	chr19	45565000	4.61E-02	promoter	super_enhancer	SDHAF1
chr19	36505000	chr19	45555000	3.92E-02	promoter	super_enhancer	AC002116.8
chr19	36505000	chr19	58555000	2.41E-02	promoter	super_enhancer	AC002116.8
chr19	36525000	chr19	38725000	2.18E-02	promoter	super_enhancer	CLIP3
chr19	36525000	chr19	43445000	2.03E-02	promoter	super_enhancer	CLIP3
chr19	36525000	chr19	44755000	3.36E-02	promoter	super_enhancer	CLIP3
chr19	36525000	chr19	46795000	3.51E-02	promoter	super_enhancer	CLIP3

chr19	36525000	chr19	46875000	5.81E-04	promoter	super_enhancer	CLIP3
chr19	36525000	chr19	55555000	2.33E-02	promoter	super_enhancer	CLIP3
chr19	36525000	chr19	56095000	2.74E-02	promoter	super_enhancer	CLIP3
chr19	36545000	chr19	44755000	2.76E-02	promoter	super_enhancer	WDR62
chr19	36605000	chr19	38675000	2.53E-02	promoter	super_enhancer	TBCB
chr19	36605000	chr19	47725000	2.59E-02	promoter	super_enhancer	TBCB
chr19	36605000	chr19	48965000	3.41E-02	promoter	super_enhancer	TBCB
chr19	36645000	chr19	55555000	1.66E-02	promoter	super_enhancer	COX7A1
chr19	36665000	chr19	38275000	1.82E-03	promoter	super_enhancer	AC002984.2
chr19	36665000	chr19	40615000	3.76E-02	promoter	super_enhancer	AC002984.2
chr19	36665000	chr19	46795000	2.76E-02	promoter	super_enhancer	AC002984.2
chr19	36705000	chr19	40625000	4.91E-02	promoter	super_enhancer	ZNF146
chr19	36705000	chr19	48565000	1.77E-02	promoter	super_enhancer	ZNF146
chr19	36705000	chr19	55555000	1.30E-04	promoter	super_enhancer	ZNF146
chr19	37665000	chr19	45785000	4.09E-02	promoter	super_enhancer	ZNF585A
chr19	37665000	chr19	48875000	2.14E-02	promoter	super_enhancer	ZNF585A
chr19	37995000	chr19	38305000	3.70E-03	promoter	super_enhancer	CTD-3064H18.2
chr19	38185000	chr19	56105000	2.10E-02	promoter	super_enhancer	CTD-2528L19.3
chr19	38245000	chr19	52675000	1.59E-02	super_enhancer	promoter	CTC-471J1.8
chr19	38245000	chr19	54225000	2.08E-02	super_enhancer	promoter	MIR520G
chr19	38245000	chr19	58515000	1.79E-02	super_enhancer	promoter	ZNF606
chr19	38255000	chr19	47165000	2.79E-02	super_enhancer	promoter	DACT3
chr19	38255000	chr19	49585000	2.14E-02	super_enhancer	promoter	RN7SL708P
chr19	38255000	chr19	52255000	1.54E-02	super_enhancer	promoter	FPR2

chr19	38255000	chr19	54185000	2.05E-02	super_enhancer	promoter	MIR520F
chr19	38255000	chr19	54245000	1.98E-02	super_enhancer	promoter	MIR517C
chr19	38255000	chr19	54975000	1.55E-02	super_enhancer	promoter	LENG9
chr19	38255000	chr19	55975000	1.71E-02	super_enhancer	promoter	ISOC2
chr19	38255000	chr19	56905000	1.59E-02	super_enhancer	promoter	ZNF582
chr19	38255000	chr19	58515000	1.97E-02	super_enhancer	promoter	ZNF606
chr19	38255000	chr19	58985000	1.37E-02	super_enhancer	promoter	ZNF446
chr19	38265000	chr19	54535000	1.71E-02	super_enhancer	promoter	CTD-3093M3.1
chr19	38265000	chr19	56165000	1.88E-02	super_enhancer	promoter	U2AF2
chr19	38265000	chr19	56825000	1.64E-02	super_enhancer	promoter	AC006116.19
chr19	38275000	chr19	44165000	4.78E-02	super_enhancer	promoter	RN7SL368P
chr19	38275000	chr19	45075000	4.08E-02	super_enhancer	promoter	AC138472.4
chr19	38275000	chr19	51895000	1.71E-02	super_enhancer	promoter	C19orf84
chr19	38275000	chr19	55565000	1.90E-02	super_enhancer	promoter	CTC-550B14.6
chr19	38275000	chr19	56835000	1.43E-02	super_enhancer	promoter	AC006116.22
chr19	38275000	chr19	58545000	1.91E-02	super_enhancer	promoter	ZSCAN1
chr19	38275000	chr19	58875000	1.72E-02	super_enhancer	promoter	CTD-2619J13.8
chr19	38295000	chr19	44285000	4.54E-02	super_enhancer	promoter	KCNN4
chr19	38295000	chr19	51685000	1.49E-02	super_enhancer	promoter	CTD-3187F8.14
chr19	38295000	chr19	55995000	1.67E-02	super_enhancer	promoter	NAT14
chr19	38295000	chr19	56195000	1.60E-02	super_enhancer	promoter	AC010525.1
chr19	38295000	chr19	56905000	1.42E-02	super_enhancer	promoter	ZNF582
chr19	38305000	chr19	44405000	4.30E-02	super_enhancer	promoter	RP11-15A1.2
chr19	38305000	chr19	53195000	1.71E-02	super_enhancer	promoter	ZNF83

chr19	38305000	chr19	55215000	2.34E-02	super_enhancer	promoter	AC098784.1
chr19	38645000	chr19	38875000	2.64E-02	super_enhancer	promoter	AC005789.9
chr19	38645000	chr19	46735000	4.50E-02	super_enhancer	promoter	AC006262.10
chr19	38645000	chr19	51895000	2.11E-02	super_enhancer	promoter	C19orf84
chr19	38645000	chr19	58875000	2.12E-02	super_enhancer	promoter	CTD-2619J13.8
chr19	38655000	chr19	39755000	3.01E-02	super_enhancer	promoter	IFNL4P1
chr19	38655000	chr19	47105000	3.76E-02	super_enhancer	promoter	PPP5D1
chr19	38655000	chr19	54255000	3.02E-02	super_enhancer	promoter	MIR519A1
chr19	38655000	chr19	54695000	2.49E-02	super_enhancer	promoter	MBOAT7
chr19	38665000	chr19	46145000	3.82E-02	super_enhancer	promoter	EML2-AS1
chr19	38665000	chr19	46155000	4.83E-02	super_enhancer	promoter	RN7SL836P
chr19	38665000	chr19	50365000	2.66E-02	super_enhancer	promoter	AC018766.4
chr19	38665000	chr19	50815000	2.54E-02	super_enhancer	promoter	CTB-191K22.5
chr19	38665000	chr19	54985000	2.30E-02	super_enhancer	promoter	CDC42EP5
chr19	38675000	chr19	38875000	3.74E-02	super_enhancer	promoter	AC005789.9
chr19	38675000	chr19	43065000	4.60E-03	super_enhancer	promoter	CEACAM1
chr19	38675000	chr19	51775000	1.66E-02	super_enhancer	promoter	CTD-3187F8.2
chr19	38675000	chr19	58865000	2.18E-04	super_enhancer	promoter	A1BG
chr19	38685000	chr19	39755000	5.89E-04	super_enhancer	promoter	IFNL4P1
chr19	38685000	chr19	49315000	2.57E-02	super_enhancer	promoter	BCAT2
chr19	38685000	chr19	51815000	1.85E-02	super_enhancer	promoter	IGLON5
chr19	38685000	chr19	52895000	1.86E-02	super_enhancer	promoter	CTD-3018O17.5
chr19	38685000	chr19	54175000	2.36E-02	super_enhancer	promoter	MIR1323
chr19	38695000	chr19	51895000	2.15E-02	super_enhancer	promoter	C19orf84

chr19	38695000	chr19	53935000	2.59E-02	super_enhancer	promoter	TPM3P9
chr19	38695000	chr19	54785000	2.75E-02	super_enhancer	promoter	LILRB2
chr19	38695000	chr19	55975000	2.20E-02	super_enhancer	promoter	ISOC2
chr19	38705000	chr19	38825000	2.92E-02	super_enhancer	promoter	CATSPERG
chr19	38705000	chr19	43775000	3.46E-02	super_enhancer	promoter	PSG9
chr19	38705000	chr19	54225000	2.38E-02	super_enhancer	promoter	MIR520G
chr19	38705000	chr19	54465000	2.76E-02	super_enhancer	promoter	CACNG8
chr19	38715000	chr19	39755000	2.95E-02	super_enhancer	promoter	IFNL4P1
chr19	38715000	chr19	54225000	2.74E-02	super_enhancer	promoter	MIR520G
chr19	38715000	chr19	57875000	2.18E-02	super_enhancer	promoter	ZNF547
chr19	38725000	chr19	38795000	3.78E-02	super_enhancer	promoter	C19orf33
chr19	38725000	chr19	38825000	1.92E-02	super_enhancer	promoter	CATSPERG
chr19	38725000	chr19	46145000	3.78E-02	super_enhancer	promoter	EML2-AS1
chr19	38725000	chr19	55635000	2.44E-02	super_enhancer	promoter	MIR7975
chr19	38725000	chr19	58985000	1.73E-02	super_enhancer	promoter	ZNF446
chr19	38735000	chr19	38795000	2.96E-02	super_enhancer	promoter	C19orf33
chr19	38735000	chr19	38825000	1.16E-02	super_enhancer	promoter	CATSPERG
chr19	38735000	chr19	46735000	4.58E-02	super_enhancer	promoter	AC006262.10
chr19	38735000	chr19	50145000	2.49E-02	super_enhancer	promoter	SCAF1
chr19	38735000	chr19	54825000	2.42E-02	super_enhancer	promoter	LILRA5
chr19	38795000	chr19	45405000	4.53E-02	promoter	super_enhancer	C19orf33
chr19	38865000	chr19	56105000	2.63E-02	promoter	super_enhancer	PSMD8
chr19	38865000	chr19	56115000	1.87E-02	promoter	super_enhancer	PSMD8
chr19	38875000	chr19	39405000	4.25E-02	promoter	super_enhancer	AC005789.9

chr19	38875000	chr19	46865000	2.96E-02	promoter	super_enhancer	AC005789.9
chr19	38875000	chr19	47615000	2.58E-02	promoter	super_enhancer	AC005789.9
chr19	38875000	chr19	56105000	2.39E-02	promoter	super_enhancer	AC005789.9
chr19	39055000	chr19	41815000	9.19E-03	super_enhancer	promoter	CCDC97
chr19	39055000	chr19	49545000	1.86E-02	super_enhancer	promoter	CTB-60B18.17
chr19	39055000	chr19	51645000	2.67E-02	super_enhancer	promoter	SIGLEC7
chr19	39055000	chr19	52075000	1.77E-02	super_enhancer	promoter	ZNF175
chr19	39055000	chr19	54335000	1.81E-02	super_enhancer	promoter	AC008753.1
chr19	39055000	chr19	56165000	2.52E-02	super_enhancer	promoter	U2AF2
chr19	39065000	chr19	39265000	1.63E-02	super_enhancer	promoter	LGALS7
chr19	39065000	chr19	47115000	4.64E-02	super_enhancer	promoter	CTB-12A17.3
chr19	39065000	chr19	48675000	2.26E-02	super_enhancer	promoter	LIG1
chr19	39075000	chr19	47115000	4.33E-02	super_enhancer	promoter	CTB-12A17.3
chr19	39075000	chr19	47355000	3.75E-02	super_enhancer	promoter	AP2S1
chr19	39075000	chr19	47835000	3.05E-02	super_enhancer	promoter	C5AR2
chr19	39075000	chr19	49965000	2.34E-02	super_enhancer	promoter	CTD-3148I10.9
chr19	39075000	chr19	51845000	2.18E-02	super_enhancer	promoter	VSIG10L
chr19	39265000	chr19	46895000	2.40E-02	promoter	super_enhancer	LGALS7
chr19	39265000	chr19	48875000	1.91E-02	promoter	super_enhancer	LGALS7
chr19	39265000	chr19	55535000	1.27E-02	promoter	super_enhancer	LGALS7
chr19	39305000	chr19	43185000	4.91E-02	promoter	super_enhancer	LGALS4
chr19	39305000	chr19	47605000	3.34E-02	promoter	super_enhancer	LGALS4
chr19	39305000	chr19	55545000	2.86E-02	promoter	super_enhancer	LGALS4
chr19	39405000	chr19	39755000	3.07E-02	super_enhancer	promoter	IFNL4P1

chr19	39405000	chr19	50815000	2.84E-02	super_enhancer	promoter	CTB-191K22.5
chr19	39405000	chr19	58865000	2.36E-02	super_enhancer	promoter	A1BG
chr19	39575000	chr19	43325000	4.99E-02	promoter	super_enhancer	PAPL
chr19	39575000	chr19	56105000	2.93E-02	promoter	super_enhancer	PAPL
chr19	39615000	chr19	58555000	2.68E-02	promoter	super_enhancer	PAK4
chr19	39695000	chr19	46785000	4.10E-02	promoter	super_enhancer	SYCN
chr19	39695000	chr19	46795000	4.12E-02	promoter	super_enhancer	SYCN
chr19	39695000	chr19	48335000	2.84E-02	promoter	super_enhancer	SYCN
chr19	39695000	chr19	55525000	2.39E-02	promoter	super_enhancer	SYCN
chr19	39695000	chr19	56095000	2.84E-02	promoter	super_enhancer	SYCN
chr19	39755000	chr19	43465000	3.82E-02	promoter	super_enhancer	IFNL4P1
chr19	39755000	chr19	43545000	4.62E-02	promoter	super_enhancer	IFNL4P1
chr19	39755000	chr19	48875000	3.27E-02	promoter	super_enhancer	IFNL4P1
chr19	39755000	chr19	56095000	3.15E-02	promoter	super_enhancer	IFNL4P1
chr19	40075000	chr19	40575000	2.76E-02	promoter	super_enhancer	CTB-60E11.12
chr19	40075000	chr19	40585000	3.26E-03	promoter	super_enhancer	CTB-60E11.12
chr19	40075000	chr19	40615000	2.51E-02	promoter	super_enhancer	CTB-60E11.12
chr19	40075000	chr19	40715000	1.97E-02	promoter	super_enhancer	CTB-60E11.12
chr19	40135000	chr19	48985000	3.63E-02	promoter	super_enhancer	AC005176.3
chr19	40185000	chr19	43445000	4.54E-02	promoter	super_enhancer	AC093063.2
chr19	40195000	chr19	46895000	4.57E-02	promoter	super_enhancer	LGALS14
chr19	40195000	chr19	48985000	4.00E-02	promoter	super_enhancer	LGALS14
chr19	40195000	chr19	56115000	2.31E-02	promoter	super_enhancer	LGALS14
chr19	40305000	chr19	40715000	2.91E-03	promoter	super_enhancer	LLNLR-268E12.1

chr19	40425000	chr19	55975000	2.60E-02	super_enhancer	promoter	ISOC2
chr19	40575000	chr19	50465000	1.98E-02	super_enhancer	promoter	SIGLEC11
chr19	40575000	chr19	53195000	1.98E-02	super_enhancer	promoter	ZNF83
chr19	40585000	chr19	50355000	2.14E-02	super_enhancer	promoter	PTOV1-AS1
chr19	40585000	chr19	51845000	1.77E-02	super_enhancer	promoter	VSIG10L
chr19	40585000	chr19	56445000	1.72E-02	super_enhancer	promoter	NLRP13
chr19	40595000	chr19	40725000	3.61E-02	super_enhancer	promoter	TTC9B
chr19	40595000	chr19	50185000	2.43E-02	super_enhancer	promoter	MIR5088
chr19	40595000	chr19	54465000	3.50E-02	super_enhancer	promoter	CACNG8
chr19	40595000	chr19	57875000	2.38E-02	super_enhancer	promoter	ZNF547
chr19	40605000	chr19	42465000	2.93E-02	super_enhancer	promoter	RABAC1
chr19	40615000	chr19	41815000	2.86E-02	super_enhancer	promoter	CCDC97
chr19	40615000	chr19	45125000	4.00E-02	super_enhancer	promoter	CEACAM22P
chr19	40615000	chr19	46685000	2.67E-02	super_enhancer	promoter	AC006262.4
chr19	40615000	chr19	54265000	1.58E-02	super_enhancer	promoter	CTC-339O9.1
chr19	40625000	chr19	46685000	3.93E-02	super_enhancer	promoter	AC006262.4
chr19	40715000	chr19	52195000	2.20E-02	super_enhancer	promoter	MIR99B
chr19	40715000	chr19	58845000	1.79E-02	super_enhancer	promoter	MIR6806
chr19	40715000	chr19	58985000	1.87E-02	super_enhancer	promoter	ZNF446
chr19	40725000	chr19	55535000	2.22E-02	promoter	super_enhancer	TTC9B
chr19	40725000	chr19	55555000	2.77E-02	promoter	super_enhancer	TTC9B
chr19	40745000	chr19	49585000	3.37E-02	super_enhancer	promoter	RN7SL708P
chr19	40745000	chr19	50185000	4.06E-04	super_enhancer	promoter	MIR5088
chr19	40745000	chr19	50975000	3.02E-02	super_enhancer	promoter	Clostridiales-1

chr19	40745000	chr19	54185000	3.14E-02	super_enhancer	promoter	MIR520F
chr19	40785000	chr19	46765000	4.82E-02	super_enhancer	promoter	IGFL1P2
chr19	40785000	chr19	49585000	2.89E-02	super_enhancer	promoter	RN7SL708P
chr19	40785000	chr19	50815000	2.62E-02	super_enhancer	promoter	CTB-191K22.5
chr19	40785000	chr19	54225000	3.01E-02	super_enhancer	promoter	MIR520G
chr19	40785000	chr19	54665000	2.91E-02	super_enhancer	promoter	LENG1
chr19	40785000	chr19	57815000	1.94E-02	super_enhancer	promoter	CTC-444N24.11
chr19	40795000	chr19	52195000	2.58E-02	super_enhancer	promoter	MIR99B
chr19	40795000	chr19	55995000	3.00E-02	super_enhancer	promoter	NAT14
chr19	40795000	chr19	56125000	3.28E-02	super_enhancer	promoter	CTD-2537I9.18
chr19	40805000	chr19	42575000	2.76E-02	super_enhancer	promoter	GRIK5
chr19	40805000	chr19	42895000	3.43E-02	super_enhancer	promoter	CNFN
chr19	40805000	chr19	50365000	3.01E-02	super_enhancer	promoter	AC018766.4
chr19	40815000	chr19	41725000	1.83E-02	super_enhancer	promoter	AXL
chr19	40815000	chr19	49555000	2.33E-02	super_enhancer	promoter	CTB-60B18.15
chr19	40825000	chr19	41535000	2.91E-02	super_enhancer	promoter	CYP2A7P1
chr19	40825000	chr19	48495000	2.93E-02	super_enhancer	promoter	BSPH1
chr19	40825000	chr19	53785000	2.61E-02	super_enhancer	promoter	FAM90A27P
chr19	40825000	chr19	54665000	3.24E-02	super_enhancer	promoter	LENG1
chr19	40825000	chr19	56825000	2.50E-02	super_enhancer	promoter	AC006116.19
chr19	40855000	chr19	55545000	2.61E-02	promoter	super_enhancer	C19orf47
chr19	40875000	chr19	47605000	4.35E-02	promoter	super_enhancer	MIR6796
chr19	40875000	chr19	48575000	3.21E-02	promoter	super_enhancer	MIR6796
chr19	40875000	chr19	55555000	3.15E-02	promoter	super_enhancer	MIR6796

chr19	41205000	chr19	41815000	4.25E-02	super_enhancer	promoter	CCDC97
chr19	41205000	chr19	42145000	4.41E-02	super_enhancer	promoter	DNAJC19P3
chr19	41205000	chr19	45165000	6.88E-03	super_enhancer	promoter	CEACAM19
chr19	41205000	chr19	48895000	3.70E-02	super_enhancer	promoter	KDELR1
chr19	41205000	chr19	54265000	3.54E-02	super_enhancer	promoter	CTC-339O9.1
chr19	41205000	chr19	54785000	3.41E-02	super_enhancer	promoter	LILRB2
chr19	41205000	chr19	59025000	3.09E-02	super_enhancer	promoter	SLC27A5
chr19	41215000	chr19	54985000	3.03E-02	super_enhancer	promoter	CDC42EP5
chr19	41215000	chr19	55805000	3.51E-02	super_enhancer	promoter	AC020922.1
chr19	41215000	chr19	58175000	2.59E-02	super_enhancer	promoter	AC003682.17
chr19	41225000	chr19	44605000	6.48E-03	super_enhancer	promoter	AC084219.3
chr19	41225000	chr19	49865000	3.66E-02	super_enhancer	promoter	DKKL1
chr19	41225000	chr19	49955000	3.48E-02	super_enhancer	promoter	ALDH16A1
chr19	41225000	chr19	53935000	3.39E-02	super_enhancer	promoter	TPM3P9
chr19	41225000	chr19	55995000	3.14E-02	super_enhancer	promoter	NAT14
chr19	41225000	chr19	56125000	3.44E-02	super_enhancer	promoter	CTD-2537I9.18
chr19	41225000	chr19	58735000	2.60E-02	super_enhancer	promoter	AC020915.2
chr19	41235000	chr19	50365000	3.45E-02	super_enhancer	promoter	AC018766.4
chr19	41235000	chr19	56145000	3.18E-02	super_enhancer	promoter	ZNF580
chr19	41235000	chr19	58125000	2.95E-02	super_enhancer	promoter	ZNF134
chr19	41245000	chr19	49055000	3.19E-02	super_enhancer	promoter	SULT2B1
chr19	41245000	chr19	51365000	2.94E-02	super_enhancer	promoter	KLK2
chr19	41245000	chr19	56905000	2.50E-02	super_enhancer	promoter	ZNF582
chr19	41255000	chr19	41905000	1.31E-02	super_enhancer	promoter	CTC-435M10.10

chr19	41255000	chr19	50355000	5.47E-04	super_enhancer	promoter	PTOV1-AS1
chr19	41255000	chr19	50935000	3.03E-02	super_enhancer	promoter	MYBPC2
chr19	41255000	chr19	53915000	2.44E-02	super_enhancer	promoter	RPL39P36
chr19	41255000	chr19	55265000	1.75E-02	super_enhancer	promoter	KIR2DP1
chr19	41255000	chr19	56125000	3.30E-02	super_enhancer	promoter	CTD-2537I9.18
chr19	41265000	chr19	50365000	3.09E-02	super_enhancer	promoter	AC018766.4
chr19	41265000	chr19	51895000	2.47E-02	super_enhancer	promoter	C19orf84
chr19	41265000	chr19	56995000	2.29E-02	super_enhancer	promoter	AC098795.1
chr19	41265000	chr19	58865000	2.39E-02	super_enhancer	promoter	A1BG
chr19	41305000	chr19	46785000	4.05E-02	promoter	super_enhancer	EGLN2
chr19	41305000	chr19	47665000	2.36E-02	promoter	super_enhancer	EGLN2
chr19	41325000	chr19	41535000	1.26E-02	super_enhancer	promoter	CYP2A7P1
chr19	41325000	chr19	50815000	2.96E-02	super_enhancer	promoter	CTB-191K22.5
chr19	41325000	chr19	51645000	3.08E-02	super_enhancer	promoter	SIGLEC7
chr19	41325000	chr19	52735000	1.77E-02	super_enhancer	promoter	CTD-2525I3.3
chr19	41325000	chr19	53195000	2.44E-02	super_enhancer	promoter	ZNF83
chr19	41345000	chr19	48975000	3.54E-02	promoter	super_enhancer	CTC-490E21.11
chr19	41905000	chr19	47655000	4.53E-02	promoter	super_enhancer	CTC-435M10.10
chr19	41905000	chr19	48965000	4.99E-02	promoter	super_enhancer	CTC-435M10.10
chr19	41905000	chr19	48985000	4.63E-02	promoter	super_enhancer	CTC-435M10.10
chr19	41935000	chr19	43185000	2.77E-02	promoter	super_enhancer	B3GNT8
chr19	42055000	chr19	55545000	2.62E-02	promoter	super_enhancer	CEACAM21
chr19	42105000	chr19	44755000	6.87E-03	promoter	super_enhancer	CEACAMP3
chr19	42115000	chr19	49545000	2.44E-02	super_enhancer	promoter	CTB-60B18.17

chr19	42255000	chr19	43525000	2.46E-02	promoter	super_enhancer	CEACAM6
chr19	42255000	chr19	47605000	4.05E-02	promoter	super_enhancer	CEACAM6
chr19	42255000	chr19	48555000	3.79E-02	promoter	super_enhancer	CEACAM6
chr19	42255000	chr19	48985000	4.04E-02	promoter	super_enhancer	CEACAM6
chr19	42465000	chr19	48875000	4.42E-02	promoter	super_enhancer	RABAC1
chr19	42575000	chr19	47655000	4.06E-02	promoter	super_enhancer	GRIK5
chr19	42585000	chr19	43535000	4.41E-02	promoter	super_enhancer	CTB-59C6.3
chr19	42895000	chr19	45585000	2.16E-02	promoter	super_enhancer	CNFN
chr19	43195000	chr19	43245000	6.74E-04	super_enhancer	promoter	CEACAM8
chr19	43195000	chr19	50365000	3.88E-02	super_enhancer	promoter	AC018766.4
chr19	43195000	chr19	54825000	3.16E-02	super_enhancer	promoter	LILRA5
chr19	43195000	chr19	56145000	3.25E-02	super_enhancer	promoter	ZNF580
chr19	43245000	chr19	48335000	3.47E-02	promoter	super_enhancer	CEACAM8
chr19	43245000	chr19	56095000	2.49E-02	promoter	super_enhancer	CEACAM8
chr19	43385000	chr19	43435000	2.42E-02	promoter	super_enhancer	PSG1
chr19	43425000	chr19	43535000	2.96E-02	promoter	super_enhancer	PSG6
chr19	43425000	chr19	55555000	1.06E-02	promoter	super_enhancer	PSG6
chr19	43435000	chr19	55875000	1.83E-02	super_enhancer	promoter	FAM71E2
chr19	43445000	chr19	44645000	1.45E-02	super_enhancer	promoter	ZNF234
chr19	43445000	chr19	54945000	1.18E-02	super_enhancer	promoter	CTD-2587H19.2
chr19	43465000	chr19	46685000	4.15E-02	super_enhancer	promoter	AC006262.4
chr19	43465000	chr19	46765000	3.91E-02	super_enhancer	promoter	IGFL1P2
chr19	43465000	chr19	55875000	1.18E-02	super_enhancer	promoter	FAM71E2
chr19	43475000	chr19	54185000	1.55E-02	super_enhancer	promoter	MIR520F

chr19	43515000	chr19	47355000	3.56E-02	super_enhancer	promoter	AP2S1
chr19	43515000	chr19	49965000	1.46E-02	super_enhancer	promoter	CTD-3148I10.9
chr19	43525000	chr19	49845000	2.32E-02	super_enhancer	promoter	CTC-301O7.4
chr19	43525000	chr19	50555000	1.61E-02	super_enhancer	promoter	CTD-2126E3.3
chr19	43535000	chr19	47105000	4.25E-02	super_enhancer	promoter	PPP5D1
chr19	43545000	chr19	44175000	2.08E-02	super_enhancer	promoter	PLAUR
chr19	43545000	chr19	47975000	2.88E-02	super_enhancer	promoter	SLC8A2
chr19	43545000	chr19	49965000	1.60E-02	super_enhancer	promoter	CTD-3148I10.9
chr19	43545000	chr19	51945000	1.32E-02	super_enhancer	promoter	CTD-3073N11.5
chr19	43765000	chr19	44615000	1.76E-02	super_enhancer	promoter	ZNF225
chr19	43765000	chr19	48675000	2.86E-02	super_enhancer	promoter	LIG1
chr19	43765000	chr19	48825000	3.58E-02	super_enhancer	promoter	CCDC114
chr19	43765000	chr19	56165000	2.36E-02	super_enhancer	promoter	U2AF2
chr19	43785000	chr19	45445000	2.34E-02	super_enhancer	promoter	APOC4
chr19	43785000	chr19	50815000	2.44E-02	super_enhancer	promoter	CTB-191K22.5
chr19	43785000	chr19	51685000	1.93E-02	super_enhancer	promoter	CTD-3187F8.14
chr19	43905000	chr19	47665000	4.58E-02	promoter	super_enhancer	TEX101
chr19	44125000	chr19	56095000	3.76E-02	promoter	super_enhancer	ZNF428
chr19	44175000	chr19	55535000	2.61E-02	promoter	super_enhancer	PLAUR
chr19	44395000	chr19	56095000	2.72E-02	promoter	super_enhancer	RP11-15A1.4
chr19	44455000	chr19	44755000	1.27E-02	promoter	super_enhancer	ZNF221
chr19	44645000	chr19	44745000	3.63E-03	promoter	super_enhancer	ZNF234
chr19	44745000	chr19	54705000	2.53E-02	super_enhancer	promoter	AC012314.20
chr19	44745000	chr19	56195000	2.44E-02	super_enhancer	promoter	AC010525.1

chr19	44745000	chr19	58875000	2.25E-02	super_enhancer	promoter	CTD-2619J13.8
chr19	44755000	chr19	57905000	1.76E-02	super_enhancer	promoter	AC004076.7
chr19	45075000	chr19	45785000	4.74E-02	promoter	super_enhancer	AC138472.4
chr19	45165000	chr19	45425000	1.30E-02	promoter	super_enhancer	CEACAM19
chr19	45395000	chr19	45405000	2.13E-03	promoter	super_enhancer	CTB-129P6.4
chr19	45405000	chr19	54585000	3.69E-02	super_enhancer	promoter	TARM1
chr19	45405000	chr19	54705000	3.46E-02	super_enhancer	promoter	AC012314.20
chr19	45405000	chr19	55845000	3.67E-02	super_enhancer	promoter	TMEM150B
chr19	45405000	chr19	55875000	3.72E-02	super_enhancer	promoter	FAM71E2
chr19	45405000	chr19	57185000	1.93E-02	super_enhancer	promoter	AC007228.9
chr19	45505000	chr19	45575000	3.65E-02	promoter	super_enhancer	RELB
chr19	45555000	chr19	46145000	2.97E-02	super_enhancer	promoter	EML2-AS1
chr19	45555000	chr19	51015000	4.95E-02	super_enhancer	promoter	JOSD2
chr19	45555000	chr19	58125000	3.38E-02	super_enhancer	promoter	ZNF134
chr19	45565000	chr19	54135000	4.85E-02	super_enhancer	promoter	DPRX
chr19	45565000	chr19	55875000	4.47E-02	super_enhancer	promoter	FAM71E2
chr19	45565000	chr19	55955000	4.08E-02	super_enhancer	promoter	SHISA7
chr19	45565000	chr19	56145000	4.37E-02	super_enhancer	promoter	ZNF580
chr19	45575000	chr19	54665000	4.30E-02	super_enhancer	promoter	LENG1
chr19	45575000	chr19	56195000	3.42E-02	super_enhancer	promoter	AC010525.1
chr19	45575000	chr19	59085000	3.23E-02	super_enhancer	promoter	MZF1
chr19	45585000	chr19	45595000	3.45E-02	super_enhancer	promoter	CTB-179K24.3
chr19	45585000	chr19	52195000	3.88E-02	super_enhancer	promoter	MIR99B
chr19	45585000	chr19	54485000	4.68E-02	super_enhancer	promoter	MIR935

chr19	45585000	chr19	54985000	7.02E-04	super_enhancer	promoter	CDC42EP5
chr19	45585000	chr19	55875000	4.10E-02	super_enhancer	promoter	FAM71E2
chr19	45655000	chr19	55535000	3.24E-02	promoter	super_enhancer	AC005757.7
chr19	45715000	chr19	52345000	3.80E-02	super_enhancer	promoter	AC006272.2
chr19	45715000	chr19	53935000	4.27E-02	super_enhancer	promoter	TPM3P9
chr19	45715000	chr19	54975000	3.19E-02	super_enhancer	promoter	LENG9
chr19	45725000	chr19	56195000	3.72E-02	super_enhancer	promoter	AC010525.1
chr19	45785000	chr19	53195000	4.02E-02	super_enhancer	promoter	ZNF83
chr19	46195000	chr19	59025000	3.63E-02	super_enhancer	promoter	SLC27A5
chr19	46205000	chr19	56165000	4.37E-02	super_enhancer	promoter	U2AF2
chr19	46205000	chr19	57185000	2.32E-02	super_enhancer	promoter	AC007228.9
chr19	46215000	chr19	52785000	2.85E-02	super_enhancer	promoter	MIR643
chr19	46215000	chr19	55845000	3.76E-02	super_enhancer	promoter	TMEM150B
chr19	46215000	chr19	58985000	2.55E-02	super_enhancer	promoter	ZNF446
chr19	46215000	chr19	59055000	1.71E-02	super_enhancer	promoter	RN7SL525P
chr19	46645000	chr19	46795000	1.18E-02	promoter	super_enhancer	IGFL2
chr19	46785000	chr19	46975000	1.16E-02	super_enhancer	promoter	PNMAL1
chr19	46785000	chr19	47115000	4.66E-02	super_enhancer	promoter	CTB-12A17.3
chr19	46785000	chr19	55875000	3.41E-02	super_enhancer	promoter	FAM71E2
chr19	46795000	chr19	52345000	3.62E-02	super_enhancer	promoter	AC006272.2
chr19	46795000	chr19	58175000	2.59E-02	super_enhancer	promoter	AC003682.17
chr19	46795000	chr19	58465000	2.70E-02	super_enhancer	promoter	CTD-2583A14.1
chr19	46865000	chr19	55635000	3.03E-02	super_enhancer	promoter	MIR7975
chr19	46875000	chr19	46975000	2.31E-02	super_enhancer	promoter	PNMAL1

chr19	46875000	chr19	53195000	3.31E-02	super_enhancer	promoter	ZNF83
chr19	46875000	chr19	54135000	3.86E-02	super_enhancer	promoter	DPRX
chr19	46885000	chr19	54225000	4.22E-02	super_enhancer	promoter	MIR520G
chr19	46885000	chr19	54695000	3.43E-02	super_enhancer	promoter	MBOAT7
chr19	46895000	chr19	51415000	4.75E-02	super_enhancer	promoter	KLK4
chr19	46895000	chr19	55825000	2.75E-02	super_enhancer	promoter	CTD-2105E13.14
chr19	47115000	chr19	48325000	9.93E-03	promoter	super_enhancer	CTB-12A17.3
chr19	47165000	chr19	47635000	2.09E-02	promoter	super_enhancer	DACT3
chr19	47355000	chr19	47615000	2.94E-02	promoter	super_enhancer	AP2S1
chr19	47585000	chr19	52035000	3.24E-02	super_enhancer	promoter	SIGLEC6
chr19	47585000	chr19	54465000	4.61E-02	super_enhancer	promoter	CACNG8
chr19	47585000	chr19	55635000	3.09E-02	super_enhancer	promoter	MIR7975
chr19	47585000	chr19	58985000	2.04E-02	super_enhancer	promoter	ZNF446
chr19	47595000	chr19	48825000	4.09E-02	super_enhancer	promoter	CCDC114
chr19	47595000	chr19	54205000	3.77E-02	super_enhancer	promoter	MIR518B
chr19	47595000	chr19	58875000	2.67E-02	super_enhancer	promoter	CTD-2619J13.8
chr19	47605000	chr19	51845000	4.06E-02	super_enhancer	promoter	VSIG10L
chr19	47605000	chr19	56855000	1.43E-02	super_enhancer	promoter	AC006116.13
chr19	47615000	chr19	55085000	3.20E-02	super_enhancer	promoter	LILRA1
chr19	47635000	chr19	53935000	3.53E-02	super_enhancer	promoter	TPM3P9
chr19	47655000	chr19	54695000	3.12E-02	super_enhancer	promoter	MBOAT7
chr19	47655000	chr19	55385000	2.73E-02	super_enhancer	promoter	FCAR
chr19	47665000	chr19	47975000	2.24E-02	super_enhancer	promoter	SLC8A2
chr19	47665000	chr19	55155000	2.46E-02	super_enhancer	promoter	LILRB4

chr19	47725000	chr19	55805000	3.04E-02	super_enhancer	promoter	AC020922.1
chr19	47735000	chr19	52195000	3.69E-02	super_enhancer	promoter	MIR99B
chr19	47735000	chr19	58735000	2.19E-02	super_enhancer	promoter	AC020915.2
chr19	47735000	chr19	59085000	2.41E-02	super_enhancer	promoter	MZF1
chr19	47835000	chr19	48335000	1.67E-03	promoter	super_enhancer	C5AR2
chr19	47975000	chr19	48325000	1.93E-03	promoter	super_enhancer	SLC8A2
chr19	48325000	chr19	56755000	2.38E-02	super_enhancer	promoter	ZSCAN5D
chr19	48335000	chr19	52075000	3.95E-02	super_enhancer	promoter	ZNF175
chr19	48335000	chr19	59025000	2.84E-02	super_enhancer	promoter	SLC27A5
chr19	48495000	chr19	48565000	3.93E-02	promoter	super_enhancer	BSPH1
chr19	48495000	chr19	48575000	1.40E-02	promoter	super_enhancer	BSPH1
chr19	48545000	chr19	52075000	4.04E-02	super_enhancer	promoter	ZNF175
chr19	48545000	chr19	54245000	3.65E-02	super_enhancer	promoter	MIR517C
chr19	48545000	chr19	54945000	3.18E-02	super_enhancer	promoter	CTD-2587H19.2
chr19	48545000	chr19	56835000	2.05E-02	super_enhancer	promoter	AC006116.22
chr19	48555000	chr19	48615000	2.45E-02	super_enhancer	promoter	PLA2G4C
chr19	48555000	chr19	54785000	4.31E-02	super_enhancer	promoter	LILRB2
chr19	48555000	chr19	55265000	2.08E-02	super_enhancer	promoter	KIR2DP1
chr19	48565000	chr19	49375000	1.46E-02	super_enhancer	promoter	PPP1R15A
chr19	48565000	chr19	58985000	1.98E-02	super_enhancer	promoter	ZNF446
chr19	48585000	chr19	48775000	1.63E-02	super_enhancer	promoter	CTC-241F20.4
chr19	48675000	chr19	55525000	2.44E-02	promoter	super_enhancer	LIG1
chr19	48775000	chr19	48985000	2.02E-02	promoter	super_enhancer	CTC-241F20.4
chr19	48825000	chr19	48975000	4.72E-02	promoter	super_enhancer	CCDC114

chr19	48895000	chr19	48975000	1.74E-02	promoter	super_enhancer	KDELR1
chr19	48975000	chr19	55635000	4.80E-02	super_enhancer	promoter	MIR7975
chr19	48985000	chr19	55875000	4.36E-02	super_enhancer	promoter	FAM71E2
chr19	49055000	chr19	58555000	3.00E-02	promoter	super_enhancer	SULT2B1
chr19	49315000	chr19	55525000	3.57E-02	promoter	super_enhancer	BCAT2
chr19	49315000	chr19	56105000	3.93E-02	promoter	super_enhancer	BCAT2
chr19	50095000	chr19	55555000	3.20E-02	promoter	super_enhancer	NOSIP
chr19	50295000	chr19	55545000	4.28E-02	promoter	super_enhancer	MIR6799
chr19	51015000	chr19	55535000	4.13E-02	promoter	super_enhancer	JOSD2
chr19	51845000	chr19	58555000	3.98E-02	promoter	super_enhancer	VSIG10L
chr19	52615000	chr19	55535000	4.47E-02	promoter	super_enhancer	CTC-471J1.10
chr19	52895000	chr19	58555000	4.45E-02	promoter	super_enhancer	CTD-3018O17.5
chr19	53775000	chr19	58555000	4.34E-02	promoter	super_enhancer	CTD-2245F17.9
chr19	53795000	chr19	55555000	1.75E-02	promoter	super_enhancer	BIRC8
chr19	53935000	chr19	58555000	3.06E-03	promoter	super_enhancer	TPM3P9
chr19	54215000	chr19	58555000	4.68E-03	promoter	super_enhancer	MIR517A
chr19	54255000	chr19	58555000	4.94E-03	promoter	super_enhancer	MIR519A1
chr19	54485000	chr19	55555000	2.18E-02	promoter	super_enhancer	MIR935
chr19	54705000	chr19	56115000	3.21E-02	promoter	super_enhancer	AC012314.20
chr19	54945000	chr19	55535000	4.72E-02	promoter	super_enhancer	CTD-2587H19.2
chr19	55215000	chr19	58555000	9.60E-03	promoter	super_enhancer	AC098784.1
chr19	55525000	chr19	55955000	1.20E-02	super_enhancer	promoter	SHISA7
chr19	55525000	chr19	56125000	2.73E-02	super_enhancer	promoter	CTD-2537I9.18
chr19	55545000	chr19	55975000	4.76E-02	super_enhancer	promoter	ISOC2

chr19	55875000	chr19	56115000	4.57E-02	promoter	super_enhancer	FAM71E2
chr19	56105000	chr19	58175000	1.93E-02	super_enhancer	promoter	AC003682.17
chr19	56105000	chr19	58545000	1.75E-02	super_enhancer	promoter	ZSCAN1
chr19	56115000	chr19	56145000	3.88E-02	super_enhancer	promoter	ZNF580
chr20	155000	chr20	4135000	2.26E-02	promoter	super_enhancer	AL360078.1
chr20	155000	chr20	31595000	6.19E-03	promoter	super_enhancer	AL360078.1
chr20	155000	chr20	36105000	6.45E-03	promoter	super_enhancer	AL360078.1
chr20	365000	chr20	3155000	4.74E-02	super_enhancer	promoter	LZTS3
chr20	365000	chr20	4005000	3.58E-02	super_enhancer	promoter	FTLP3
chr20	375000	chr20	5485000	1.68E-02	super_enhancer	promoter	LINC00654
chr20	385000	chr20	35505000	8.34E-03	super_enhancer	promoter	TLDC2
chr20	395000	chr20	61925000	3.14E-03	super_enhancer	promoter	COL20A1
chr20	405000	chr20	21285000	4.49E-03	super_enhancer	promoter	RP1-198K11.5
chr20	405000	chr20	62665000	4.18E-03	super_enhancer	promoter	LINC00176
chr20	415000	chr20	55575000	4.61E-03	super_enhancer	promoter	AL117380.2
chr20	425000	chr20	35275000	7.79E-03	super_enhancer	promoter	SLA2
chr20	425000	chr20	43025000	6.33E-03	super_enhancer	promoter	HNF4A-AS1
chr20	525000	chr20	35315000	7.05E-03	promoter	super_enhancer	CSNK2A1
chr20	525000	chr20	62645000	3.51E-03	promoter	super_enhancer	CSNK2A1
chr20	1305000	chr20	45325000	6.33E-03	promoter	super_enhancer	SDCBP2-AS1
chr20	1305000	chr20	50135000	5.13E-03	promoter	super_enhancer	SDCBP2-AS1
chr20	1305000	chr20	53585000	3.27E-03	promoter	super_enhancer	SDCBP2-AS1
chr20	1305000	chr20	62645000	4.04E-03	promoter	super_enhancer	SDCBP2-AS1
chr20	1875000	chr20	25235000	4.90E-03	promoter	super_enhancer	RP5-858L17.1

chr20	1875000	chr20	45355000	6.14E-03	promoter	super_enhancer	RP5-858L17.1
chr20	1975000	chr20	33795000	7.78E-03	promoter	super_enhancer	PDYN
chr20	1975000	chr20	35855000	8.32E-03	promoter	super_enhancer	PDYN
chr20	2635000	chr20	35895000	8.47E-03	promoter	super_enhancer	SNORD110
chr20	2635000	chr20	50505000	6.04E-03	promoter	super_enhancer	SNORD110
chr20	2795000	chr20	36465000	8.00E-03	promoter	super_enhancer	C20orf141
chr20	2795000	chr20	56765000	4.52E-03	promoter	super_enhancer	C20orf141
chr20	2845000	chr20	3815000	1.65E-02	promoter	super_enhancer	PTPRA
chr20	2845000	chr20	44685000	5.95E-03	promoter	super_enhancer	PTPRA
chr20	2845000	chr20	50505000	5.52E-03	promoter	super_enhancer	PTPRA
chr20	3065000	chr20	40995000	6.25E-03	promoter	super_enhancer	AVP
chr20	3065000	chr20	53925000	3.34E-03	promoter	super_enhancer	AVP
chr20	3075000	chr20	47455000	7.98E-03	promoter	super_enhancer	RN7SL555P
chr20	3155000	chr20	36835000	6.63E-03	promoter	super_enhancer	LZTS3
chr20	3185000	chr20	34375000	1.33E-02	promoter	super_enhancer	DDRGK1
chr20	3185000	chr20	35865000	1.15E-02	promoter	super_enhancer	DDRGK1
chr20	3185000	chr20	36475000	9.70E-03	promoter	super_enhancer	DDRGK1
chr20	3385000	chr20	36085000	7.01E-03	promoter	super_enhancer	AL117334.1
chr20	3645000	chr20	50495000	6.62E-03	promoter	super_enhancer	GFRA4
chr20	3805000	chr20	21285000	4.94E-03	super_enhancer	promoter	RP1-198K11.5
chr20	3805000	chr20	30595000	1.00E-02	super_enhancer	promoter	RNA5SP481
chr20	3805000	chr20	36155000	8.42E-03	super_enhancer	promoter	RP11-425M5.7
chr20	3905000	chr20	4135000	2.77E-02	promoter	super_enhancer	RP4-681N20.5
chr20	3905000	chr20	34375000	1.33E-02	promoter	super_enhancer	RP4-681N20.5

chr20	3905000	chr20	50545000	6.25E-03	promoter	super_enhancer	RP4-681N20.5
chr20	4005000	chr20	35885000	1.11E-02	promoter	super_enhancer	FTLP3
chr20	4115000	chr20	25935000	4.93E-03	super_enhancer	promoter	RP4-760C5.5
chr20	4115000	chr20	61885000	3.36E-03	super_enhancer	promoter	FLJ16779
chr20	4135000	chr20	30795000	1.35E-02	super_enhancer	promoter	POFUT1
chr20	4135000	chr20	35375000	9.97E-03	super_enhancer	promoter	NDRG3
chr20	4135000	chr20	35795000	8.85E-03	super_enhancer	promoter	RP3-343K2.4
chr20	4135000	chr20	44605000	7.48E-03	super_enhancer	promoter	FTLP1
chr20	4135000	chr20	61735000	5.90E-03	super_enhancer	promoter	HAR1B
chr20	4805000	chr20	38145000	7.52E-03	promoter	super_enhancer	RASSF2
chr20	5095000	chr20	50545000	5.01E-03	promoter	super_enhancer	TMEM230
chr20	5295000	chr20	25235000	6.00E-03	promoter	super_enhancer	PROKR2
chr20	6105000	chr20	51345000	4.07E-03	promoter	super_enhancer	FERMT1
chr20	7235000	chr20	36075000	6.27E-03	promoter	super_enhancer	LINC01428
chr20	9555000	chr20	50125000	4.79E-03	promoter	super_enhancer	RP5-986I17.2
chr20	9555000	chr20	50285000	4.99E-03	promoter	super_enhancer	RP5-986I17.2
chr20	9555000	chr20	56775000	5.05E-03	promoter	super_enhancer	RP5-986I17.2
chr20	10595000	chr20	44735000	7.39E-03	promoter	super_enhancer	RP5-1099D15.1
chr20	10595000	chr20	47455000	7.46E-03	promoter	super_enhancer	RP5-1099D15.1
chr20	12225000	chr20	43955000	4.80E-03	promoter	super_enhancer	RP4-796I8.1
chr20	13955000	chr20	49685000	6.11E-03	promoter	super_enhancer	RNU6-278P
chr20	17945000	chr20	50305000	5.96E-03	promoter	super_enhancer	SNORD17
chr20	18625000	chr20	47335000	8.54E-03	promoter	super_enhancer	DUXAP7
chr20	18765000	chr20	43945000	7.07E-03	promoter	super_enhancer	EEF1A1P34

chr20	18765000	chr20	43955000	5.12E-03	promoter	super_enhancer	EEF1A1P34
chr20	18765000	chr20	49755000	5.50E-03	promoter	super_enhancer	EEF1A1P34
chr20	19195000	chr20	38185000	1.11E-02	promoter	super_enhancer	RP11-97N19.2
chr20	19195000	chr20	50495000	8.90E-03	promoter	super_enhancer	RP11-97N19.2
chr20	19195000	chr20	51315000	6.30E-03	promoter	super_enhancer	RP11-97N19.2
chr20	19265000	chr20	50195000	9.21E-03	promoter	super_enhancer	RP5-1027G4.3
chr20	19265000	chr20	50565000	1.02E-02	promoter	super_enhancer	RP5-1027G4.3
chr20	19805000	chr20	49765000	6.77E-03	promoter	super_enhancer	RPL12L3
chr20	20235000	chr20	36075000	1.19E-02	promoter	super_enhancer	RPL17P1
chr20	21095000	chr20	31595000	1.47E-02	promoter	super_enhancer	RPL24P2
chr20	21145000	chr20	38205000	6.79E-03	promoter	super_enhancer	RP5-872K7.7
chr20	21285000	chr20	32355000	1.42E-02	promoter	super_enhancer	RP1-198K11.5
chr20	21485000	chr20	35855000	1.90E-02	promoter	super_enhancer	RP5-984P4.6
chr20	21485000	chr20	38145000	1.32E-02	promoter	super_enhancer	RP5-984P4.6
chr20	21495000	chr20	38135000	1.07E-02	promoter	super_enhancer	NKX2-2
chr20	21595000	chr20	51635000	5.50E-03	promoter	super_enhancer	Nkx2-2as
chr20	21725000	chr20	45355000	1.04E-02	promoter	super_enhancer	SLC25A6P1
chr20	21725000	chr20	51755000	5.56E-03	promoter	super_enhancer	SLC25A6P1
chr20	22255000	chr20	38125000	1.08E-02	promoter	super_enhancer	RP5-828K20.1
chr20	22255000	chr20	57705000	4.51E-03	promoter	super_enhancer	RP5-828K20.1
chr20	22325000	chr20	43975000	9.34E-03	promoter	super_enhancer	RP5-1004I9.2
chr20	22715000	chr20	50525000	7.45E-03	promoter	super_enhancer	KRT18P3
chr20	23015000	chr20	44685000	1.17E-02	promoter	super_enhancer	SSTR4
chr20	24975000	chr20	25225000	2.19E-03	promoter	super_enhancer	APMAP

chr20	24975000	chr20	36845000	1.70E-02	promoter	super_enhancer	APMAP
chr20	25175000	chr20	38165000	1.57E-02	promoter	super_enhancer	ENTPD6
chr20	25175000	chr20	50125000	4.29E-05	promoter	super_enhancer	ENTPD6
chr20	25175000	chr20	50515000	8.84E-03	promoter	super_enhancer	ENTPD6
chr20	25225000	chr20	29945000	2.61E-02	super_enhancer	promoter	RNA5SP480
chr20	25225000	chr20	37065000	1.56E-02	super_enhancer	promoter	SNHG17
chr20	25225000	chr20	37075000	1.68E-02	super_enhancer	promoter	SNHG11
chr20	25225000	chr20	37145000	1.08E-02	super_enhancer	promoter	AL049868.1
chr20	25225000	chr20	44335000	1.02E-02	super_enhancer	promoter	WFDC10B
chr20	25235000	chr20	29845000	3.06E-02	super_enhancer	promoter	DEFB115
chr20	25235000	chr20	35725000	1.92E-02	super_enhancer	promoter	RBL1
chr20	25235000	chr20	36305000	1.42E-02	super_enhancer	promoter	RP4-640H8.2
chr20	25235000	chr20	56535000	8.78E-03	super_enhancer	promoter	RP13-379L11.2
chr20	25605000	chr20	49695000	1.11E-02	promoter	super_enhancer	NANP
chr20	26095000	chr20	49715000	1.03E-02	promoter	super_enhancer	NCOR1P1
chr20	26115000	chr20	38205000	9.63E-03	promoter	super_enhancer	RP13-329D4.3
chr20	29845000	chr20	38115000	1.54E-02	promoter	super_enhancer	DEFB115
chr20	29845000	chr20	38205000	2.58E-02	promoter	super_enhancer	DEFB115
chr20	29845000	chr20	47465000	3.10E-04	promoter	super_enhancer	DEFB115
chr20	29845000	chr20	53585000	1.06E-02	promoter	super_enhancer	DEFB115
chr20	29845000	chr20	53785000	1.03E-02	promoter	super_enhancer	DEFB115
chr20	29875000	chr20	35305000	4.79E-02	promoter	super_enhancer	RP5-854E16.2
chr20	29945000	chr20	35855000	4.74E-02	promoter	super_enhancer	RNA5SP480
chr20	29945000	chr20	36495000	4.43E-02	promoter	super_enhancer	RNA5SP480

chr20	29945000	chr20	41025000	1.85E-02	promoter	super_enhancer	RNA5SP480
chr20	29945000	chr20	45335000	2.63E-02	promoter	super_enhancer	RNA5SP480
chr20	29945000	chr20	50435000	1.46E-02	promoter	super_enhancer	RNA5SP480
chr20	29945000	chr20	50545000	1.82E-02	promoter	super_enhancer	RNA5SP480
chr20	29945000	chr20	57695000	1.28E-02	promoter	super_enhancer	RNA5SP480
chr20	29955000	chr20	41015000	2.42E-04	promoter	super_enhancer	DEFB118
chr20	29955000	chr20	45345000	2.60E-02	promoter	super_enhancer	DEFB118
chr20	29955000	chr20	57705000	1.26E-02	promoter	super_enhancer	DEFB118
chr20	30065000	chr20	51335000	1.63E-02	promoter	super_enhancer	DEFB124
chr20	30065000	chr20	51365000	1.66E-02	promoter	super_enhancer	DEFB124
chr20	30065000	chr20	57705000	1.30E-02	promoter	super_enhancer	DEFB124
chr20	30135000	chr20	43985000	3.42E-02	promoter	super_enhancer	MCTS2P
chr20	30135000	chr20	44685000	5.66E-04	promoter	super_enhancer	MCTS2P
chr20	30135000	chr20	49685000	2.69E-02	promoter	super_enhancer	MCTS2P
chr20	30135000	chr20	57695000	1.65E-02	promoter	super_enhancer	MCTS2P
chr20	30195000	chr20	43975000	2.80E-02	promoter	super_enhancer	MIR3193
chr20	30195000	chr20	49785000	1.92E-02	promoter	super_enhancer	MIR3193
chr20	30195000	chr20	50165000	3.15E-04	promoter	super_enhancer	MIR3193
chr20	30195000	chr20	51335000	1.71E-02	promoter	super_enhancer	MIR3193
chr20	30195000	chr20	62365000	1.52E-02	promoter	super_enhancer	MIR3193
chr20	30225000	chr20	36455000	4.94E-02	promoter	super_enhancer	COX4I2
chr20	30225000	chr20	43985000	3.61E-02	promoter	super_enhancer	COX4I2
chr20	30225000	chr20	49675000	3.30E-02	promoter	super_enhancer	COX4I2
chr20	30225000	chr20	50135000	2.47E-02	promoter	super_enhancer	COX4I2

chr20	30225000	chr20	50505000	2.97E-02	promoter	super_enhancer	COX4I2
chr20	30225000	chr20	51035000	2.48E-02	promoter	super_enhancer	COX4I2
chr20	30555000	chr20	38155000	4.99E-02	promoter	super_enhancer	XKR7
chr20	30555000	chr20	43985000	3.87E-02	promoter	super_enhancer	XKR7
chr20	30555000	chr20	45345000	3.57E-02	promoter	super_enhancer	XKR7
chr20	30555000	chr20	47435000	3.29E-02	promoter	super_enhancer	XKR7
chr20	30555000	chr20	50555000	2.41E-02	promoter	super_enhancer	XKR7
chr20	30555000	chr20	53785000	1.55E-02	promoter	super_enhancer	XKR7
chr20	30595000	chr20	38155000	4.82E-02	promoter	super_enhancer	RNA5SP481
chr20	30595000	chr20	49695000	3.41E-02	promoter	super_enhancer	RNA5SP481
chr20	30595000	chr20	50555000	2.32E-02	promoter	super_enhancer	RNA5SP481
chr20	30795000	chr20	38185000	4.00E-02	promoter	super_enhancer	POFUT1
chr20	30795000	chr20	44705000	4.10E-04	promoter	super_enhancer	POFUT1
chr20	30795000	chr20	49785000	2.27E-02	promoter	super_enhancer	POFUT1
chr20	30795000	chr20	56775000	2.40E-02	promoter	super_enhancer	POFUT1
chr20	30795000	chr20	56785000	2.01E-02	promoter	super_enhancer	POFUT1
chr20	30825000	chr20	36835000	4.94E-02	promoter	super_enhancer	MIR1825
chr20	30825000	chr20	38215000	3.38E-02	promoter	super_enhancer	MIR1825
chr20	30825000	chr20	43975000	3.17E-02	promoter	super_enhancer	MIR1825
chr20	30825000	chr20	50305000	2.17E-02	promoter	super_enhancer	MIR1825
chr20	30825000	chr20	51335000	1.93E-02	promoter	super_enhancer	MIR1825
chr20	30865000	chr20	36085000	4.94E-02	promoter	super_enhancer	KIF3B
chr20	30865000	chr20	50195000	1.81E-02	promoter	super_enhancer	KIF3B
chr20	30865000	chr20	50435000	1.42E-02	promoter	super_enhancer	KIF3B

chr20	30865000	chr20	56795000	1.49E-02	promoter	super_enhancer	KIF3B
chr20	30945000	chr20	36855000	4.57E-02	promoter	super_enhancer	ASXL1
chr20	30945000	chr20	38195000	2.69E-02	promoter	super_enhancer	ASXL1
chr20	30945000	chr20	44725000	2.74E-02	promoter	super_enhancer	ASXL1
chr20	30945000	chr20	50125000	1.84E-02	promoter	super_enhancer	ASXL1
chr20	30945000	chr20	50155000	1.85E-02	promoter	super_enhancer	ASXL1
chr20	30945000	chr20	62645000	1.27E-02	promoter	super_enhancer	ASXL1
chr20	31595000	chr20	36305000	1.72E-03	super_enhancer	promoter	RP4-640H8.2
chr20	31595000	chr20	42425000	3.54E-02	super_enhancer	promoter	RNU6-639P
chr20	31595000	chr20	47105000	3.55E-02	super_enhancer	promoter	RP5-991B18.2
chr20	31645000	chr20	50125000	2.68E-04	promoter	super_enhancer	AL121756.1
chr20	31645000	chr20	62365000	1.67E-02	promoter	super_enhancer	AL121756.1
chr20	31695000	chr20	41235000	3.54E-02	super_enhancer	promoter	RP4-730D4.1
chr20	31695000	chr20	43025000	3.46E-02	super_enhancer	promoter	HNF4A-AS1
chr20	31695000	chr20	44115000	2.93E-02	super_enhancer	promoter	RP3-461P17.9
chr20	31695000	chr20	47805000	2.35E-02	super_enhancer	promoter	STAU1
chr20	31695000	chr20	49555000	2.42E-02	super_enhancer	promoter	AL034553.1
chr20	31715000	chr20	49095000	2.18E-02	super_enhancer	promoter	COX6CP2
chr20	31715000	chr20	56535000	2.10E-02	super_enhancer	promoter	RP13-379L11.2
chr20	31775000	chr20	38185000	3.34E-02	promoter	super_enhancer	SOCS2P1
chr20	31775000	chr20	43985000	2.79E-02	promoter	super_enhancer	SOCS2P1
chr20	31775000	chr20	47455000	2.53E-02	promoter	super_enhancer	SOCS2P1
chr20	31775000	chr20	49675000	2.53E-02	promoter	super_enhancer	SOCS2P1
chr20	31775000	chr20	49725000	1.65E-02	promoter	super_enhancer	SOCS2P1

chr20	31775000	chr20	49765000	1.79E-02	promoter	super_enhancer	SOCS2P1
chr20	31775000	chr20	51345000	1.58E-02	promoter	super_enhancer	SOCS2P1
chr20	31805000	chr20	44735000	3.51E-02	promoter	super_enhancer	RP11-49G10.3
chr20	31905000	chr20	43945000	3.77E-02	promoter	super_enhancer	BPIFB5P
chr20	31905000	chr20	44695000	6.24E-04	promoter	super_enhancer	BPIFB5P
chr20	31905000	chr20	50525000	2.75E-02	promoter	super_enhancer	BPIFB5P
chr20	32245000	chr20	43965000	4.17E-02	promoter	super_enhancer	RP1-63M2.7
chr20	32245000	chr20	47335000	3.87E-02	promoter	super_enhancer	RP1-63M2.7
chr20	32245000	chr20	49695000	4.08E-02	promoter	super_enhancer	RP1-63M2.7
chr20	32275000	chr20	44725000	4.17E-02	promoter	super_enhancer	E2F1
chr20	32275000	chr20	50305000	2.59E-02	promoter	super_enhancer	E2F1
chr20	32355000	chr20	39995000	4.73E-02	super_enhancer	promoter	EMILIN3
chr20	32355000	chr20	42425000	3.68E-02	super_enhancer	promoter	RNU6-639P
chr20	32355000	chr20	42985000	3.61E-02	super_enhancer	promoter	RP5-881L22.5
chr20	32355000	chr20	57875000	1.91E-02	super_enhancer	promoter	EDN3
chr20	32355000	chr20	62475000	1.61E-02	super_enhancer	promoter	C20orf181
chr20	32805000	chr20	44685000	5.35E-04	promoter	super_enhancer	XPOTP1
chr20	32805000	chr20	49675000	3.00E-02	promoter	super_enhancer	XPOTP1
chr20	32805000	chr20	50155000	2.21E-02	promoter	super_enhancer	XPOTP1
chr20	32805000	chr20	53905000	9.84E-03	promoter	super_enhancer	XPOTP1
chr20	32805000	chr20	62355000	1.71E-02	promoter	super_enhancer	XPOTP1
chr20	32805000	chr20	62625000	1.44E-02	promoter	super_enhancer	XPOTP1
chr20	32835000	chr20	47475000	3.25E-02	promoter	super_enhancer	RP4-785G19.2
chr20	32875000	chr20	50125000	2.79E-02	promoter	super_enhancer	CTD-3216D2.5

chr20	33065000	chr20	41015000	2.69E-02	promoter	super_enhancer	FDX1P1
chr20	33065000	chr20	43985000	3.27E-02	promoter	super_enhancer	FDX1P1
chr20	33065000	chr20	49765000	2.08E-02	promoter	super_enhancer	FDX1P1
chr20	33135000	chr20	62355000	1.94E-02	promoter	super_enhancer	MAP1LC3A
chr20	33275000	chr20	43935000	3.74E-02	promoter	super_enhancer	RP11-346K17.3
chr20	33275000	chr20	50505000	2.87E-02	promoter	super_enhancer	RP11-346K17.3
chr20	33545000	chr20	43945000	4.33E-02	promoter	super_enhancer	GSS
chr20	33545000	chr20	43965000	4.09E-02	promoter	super_enhancer	GSS
chr20	33545000	chr20	50545000	2.96E-02	promoter	super_enhancer	GSS
chr20	33545000	chr20	62645000	1.96E-02	promoter	super_enhancer	GSS
chr20	33735000	chr20	47475000	3.84E-02	promoter	super_enhancer	EDEM2
chr20	33785000	chr20	49995000	2.87E-02	super_enhancer	promoter	AL079339.1
chr20	33785000	chr20	61275000	1.35E-02	super_enhancer	promoter	RP11-93B14.6
chr20	33795000	chr20	40315000	4.07E-02	super_enhancer	promoter	RNU6-1018P
chr20	33795000	chr20	45795000	3.07E-02	super_enhancer	promoter	MIR3616
chr20	33795000	chr20	54875000	1.57E-02	super_enhancer	promoter	RP11-380D15.3
chr20	33805000	chr20	62645000	1.97E-02	promoter	super_enhancer	MT1P3
chr20	34175000	chr20	53785000	1.53E-02	promoter	super_enhancer	RPL37P1
chr20	34195000	chr20	44685000	4.63E-02	promoter	super_enhancer	FER1L4
chr20	34235000	chr20	38115000	3.42E-02	promoter	super_enhancer	RNU6-759P
chr20	34235000	chr20	49705000	2.53E-02	promoter	super_enhancer	RNU6-759P
chr20	34365000	chr20	44335000	4.14E-02	super_enhancer	promoter	WFDC10B
chr20	34365000	chr20	47015000	4.06E-02	super_enhancer	promoter	RP1-66N13.1
chr20	34365000	chr20	62585000	2.40E-02	super_enhancer	promoter	UCKL1-AS1

chr20	34375000	chr20	48445000	3.48E-02	super_enhancer	promoter	RP5-1041C10.3
chr20	34375000	chr20	61285000	2.24E-02	super_enhancer	promoter	RP11-93B14.10
chr20	34375000	chr20	61925000	2.10E-02	super_enhancer	promoter	COL20A1
chr20	34385000	chr20	44335000	4.07E-02	super_enhancer	promoter	WFDC10B
chr20	34385000	chr20	45795000	4.06E-02	super_enhancer	promoter	MIR3616
chr20	34385000	chr20	51825000	2.85E-02	super_enhancer	promoter	RP11-15M15.2
chr20	34395000	chr20	34465000	4.07E-02	super_enhancer	promoter	HIGD1AP16
chr20	34395000	chr20	48185000	3.22E-02	super_enhancer	promoter	PTGIS
chr20	34395000	chr20	54045000	1.46E-02	super_enhancer	promoter	LINC01441
chr20	34475000	chr20	35875000	1.44E-02	promoter	super_enhancer	RNU4-40P
chr20	34475000	chr20	43965000	3.69E-02	promoter	super_enhancer	RNU4-40P
chr20	34475000	chr20	49675000	3.54E-02	promoter	super_enhancer	RNU4-40P
chr20	34475000	chr20	49775000	3.41E-02	promoter	super_enhancer	RNU4-40P
chr20	34475000	chr20	56785000	2.09E-02	promoter	super_enhancer	RNU4-40P
chr20	34665000	chr20	43985000	4.32E-02	promoter	super_enhancer	HMGB3P2
chr20	34665000	chr20	45325000	3.92E-02	promoter	super_enhancer	HMGB3P2
chr20	34665000	chr20	50305000	2.62E-02	promoter	super_enhancer	HMGB3P2
chr20	34745000	chr20	47315000	3.17E-02	promoter	super_enhancer	RP11-234K24.3
chr20	35275000	chr20	49675000	3.84E-02	promoter	super_enhancer	SLA2
chr20	35305000	chr20	45795000	3.97E-02	super_enhancer	promoter	MIR3616
chr20	35305000	chr20	55935000	1.49E-02	super_enhancer	promoter	MTRNR2L3
chr20	35305000	chr20	57765000	2.98E-04	super_enhancer	promoter	ZNF831
chr20	35305000	chr20	61735000	2.24E-02	super_enhancer	promoter	HAR1B
chr20	35315000	chr20	44115000	3.72E-02	super_enhancer	promoter	RP3-461P17.9

chr20	35315000	chr20	44375000	3.12E-02	super_enhancer	promoter	HNRNPA1P3
chr20	35315000	chr20	44485000	4.24E-02	super_enhancer	promoter	ZSWIM3
chr20	35315000	chr20	49555000	2.98E-02	super_enhancer	promoter	AL034553.1
chr20	35315000	chr20	57415000	2.11E-02	super_enhancer	promoter	GNAS
chr20	35315000	chr20	61265000	7.85E-03	super_enhancer	promoter	RP13-30A9.2
chr20	35375000	chr20	43935000	4.22E-02	promoter	super_enhancer	NDRG3
chr20	35375000	chr20	53615000	1.82E-02	promoter	super_enhancer	NDRG3
chr20	35375000	chr20	62625000	1.63E-02	promoter	super_enhancer	NDRG3
chr20	35505000	chr20	44705000	3.84E-02	promoter	super_enhancer	TLDC2
chr20	35505000	chr20	44735000	4.61E-02	promoter	super_enhancer	TLDC2
chr20	35505000	chr20	50505000	3.90E-02	promoter	super_enhancer	TLDC2
chr20	35725000	chr20	43965000	4.18E-02	promoter	super_enhancer	RBL1
chr20	35725000	chr20	47455000	3.91E-02	promoter	super_enhancer	RBL1
chr20	35725000	chr20	49685000	3.35E-02	promoter	super_enhancer	RBL1
chr20	35725000	chr20	50565000	3.27E-02	promoter	super_enhancer	RBL1
chr20	35725000	chr20	56815000	1.71E-02	promoter	super_enhancer	RBL1
chr20	35795000	chr20	44705000	2.85E-02	promoter	super_enhancer	RP3-343K2.4
chr20	35795000	chr20	49715000	2.95E-02	promoter	super_enhancer	RP3-343K2.4
chr20	35795000	chr20	50525000	3.23E-04	promoter	super_enhancer	RP3-343K2.4
chr20	35795000	chr20	50555000	2.18E-02	promoter	super_enhancer	RP3-343K2.4
chr20	35795000	chr20	56795000	1.95E-02	promoter	super_enhancer	RP3-343K2.4
chr20	35855000	chr20	37055000	3.03E-02	super_enhancer	promoter	SNORA71B
chr20	35855000	chr20	42295000	4.90E-02	super_enhancer	promoter	MYBL2
chr20	35855000	chr20	43595000	4.48E-02	super_enhancer	promoter	STK4-AS1

chr20	35865000	chr20	36155000	1.25E-02	super_enhancer	promoter	RP11-425M5.7
chr20	35865000	chr20	48185000	3.64E-02	super_enhancer	promoter	PTGIS
chr20	35865000	chr20	49195000	3.65E-02	super_enhancer	promoter	RP4-530I15.9
chr20	35865000	chr20	55925000	1.73E-02	super_enhancer	promoter	RAE1
chr20	35865000	chr20	61925000	2.01E-02	super_enhancer	promoter	COL20A1
chr20	35875000	chr20	36155000	1.35E-02	super_enhancer	promoter	RP11-425M5.7
chr20	35875000	chr20	36605000	1.79E-02	super_enhancer	promoter	RN7SKP185
chr20	35875000	chr20	37055000	4.40E-02	super_enhancer	promoter	SNORA71B
chr20	35875000	chr20	44145000	5.00E-02	super_enhancer	promoter	SPINT3
chr20	35875000	chr20	49995000	4.23E-02	super_enhancer	promoter	AL079339.1
chr20	35885000	chr20	37165000	1.59E-02	super_enhancer	promoter	RPS3P2
chr20	35885000	chr20	52685000	3.17E-02	super_enhancer	promoter	MIR4756
chr20	35895000	chr20	36155000	3.87E-03	super_enhancer	promoter	RP11-425M5.7
chr20	35895000	chr20	37075000	7.08E-04	super_enhancer	promoter	SNHG11
chr20	35895000	chr20	44115000	4.20E-02	super_enhancer	promoter	RP3-461P17.9
chr20	35895000	chr20	61505000	1.87E-02	super_enhancer	promoter	ARF4P2
chr20	35895000	chr20	61925000	1.86E-02	super_enhancer	promoter	COL20A1
chr20	36075000	chr20	43095000	4.74E-02	super_enhancer	promoter	C20orf62
chr20	36075000	chr20	44335000	3.96E-02	super_enhancer	promoter	WFDC10B
chr20	36075000	chr20	46415000	3.89E-02	super_enhancer	promoter	SULF2
chr20	36075000	chr20	47655000	3.34E-02	super_enhancer	promoter	SNAP23P
chr20	36075000	chr20	47835000	4.01E-02	super_enhancer	promoter	DDX27
chr20	36075000	chr20	57875000	2.10E-02	super_enhancer	promoter	EDN3
chr20	36075000	chr20	59945000	2.04E-02	super_enhancer	promoter	WI2-8325B5.1

chr20	36075000	chr20	62665000	2.14E-02	super_enhancer	promoter	LINC00176
chr20	36085000	chr20	47105000	4.20E-02	super_enhancer	promoter	RP5-991B18.2
chr20	36095000	chr20	49555000	3.40E-02	super_enhancer	promoter	AL034553.1
chr20	36095000	chr20	57555000	2.53E-02	super_enhancer	promoter	NELFCD
chr20	36105000	chr20	36605000	2.05E-02	super_enhancer	promoter	RN7SKP185
chr20	36105000	chr20	45625000	4.85E-02	super_enhancer	promoter	GAPDHP54
chr20	36105000	chr20	46415000	4.82E-02	super_enhancer	promoter	SULF2
chr20	36105000	chr20	49615000	4.02E-02	super_enhancer	promoter	RP5-955M13.4
chr20	36105000	chr20	61285000	2.29E-02	super_enhancer	promoter	RP11-93B14.10
chr20	36155000	chr20	43975000	4.70E-02	promoter	super_enhancer	RP11-425M5.7
chr20	36155000	chr20	47435000	4.09E-02	promoter	super_enhancer	RP11-425M5.7
chr20	36155000	chr20	50125000	3.19E-02	promoter	super_enhancer	RP11-425M5.7
chr20	36305000	chr20	43975000	3.32E-02	promoter	super_enhancer	RP4-640H8.2
chr20	36305000	chr20	44725000	3.50E-02	promoter	super_enhancer	RP4-640H8.2
chr20	36305000	chr20	45335000	3.41E-02	promoter	super_enhancer	RP4-640H8.2
chr20	36305000	chr20	47465000	3.27E-02	promoter	super_enhancer	RP4-640H8.2
chr20	36305000	chr20	49675000	3.04E-02	promoter	super_enhancer	RP4-640H8.2
chr20	36305000	chr20	51625000	2.07E-02	promoter	super_enhancer	RP4-640H8.2
chr20	36305000	chr20	62655000	1.60E-02	promoter	super_enhancer	RP4-640H8.2
chr20	36455000	chr20	44035000	3.72E-02	super_enhancer	promoter	DBNDD2
chr20	36455000	chr20	57465000	1.01E-02	super_enhancer	promoter	RP1-309F20.3
chr20	36455000	chr20	57555000	1.94E-02	super_enhancer	promoter	NELFCD
chr20	36455000	chr20	61275000	1.44E-02	super_enhancer	promoter	RP11-93B14.6
chr20	36465000	chr20	42875000	4.56E-02	super_enhancer	promoter	GDAP1L1

chr20	36475000	chr20	47015000	3.85E-02	super_enhancer	promoter	RP1-66N13.1
chr20	36475000	chr20	47035000	3.05E-02	super_enhancer	promoter	RP1-66N13.4
chr20	36475000	chr20	47655000	3.43E-02	super_enhancer	promoter	SNAP23P
chr20	36475000	chr20	47805000	3.08E-02	super_enhancer	promoter	STAU1
chr20	36475000	chr20	48185000	3.23E-02	super_enhancer	promoter	PTGIS
chr20	36475000	chr20	49175000	2.34E-02	super_enhancer	promoter	RN7SL672P
chr20	36475000	chr20	56285000	1.57E-02	super_enhancer	promoter	NKILA
chr20	36475000	chr20	56885000	2.11E-02	super_enhancer	promoter	PPP4R1L
chr20	36475000	chr20	61505000	1.77E-02	super_enhancer	promoter	ARF4P2
chr20	36485000	chr20	42965000	4.25E-02	super_enhancer	promoter	R3HDML
chr20	36485000	chr20	43025000	4.86E-02	super_enhancer	promoter	HNF4A-AS1
chr20	36485000	chr20	43595000	4.30E-02	super_enhancer	promoter	STK4-AS1
chr20	36485000	chr20	43745000	4.39E-02	super_enhancer	promoter	WFDC5
chr20	36485000	chr20	47015000	3.48E-02	super_enhancer	promoter	RP1-66N13.1
chr20	36485000	chr20	47105000	3.91E-02	super_enhancer	promoter	RP5-991B18.2
chr20	36485000	chr20	49095000	2.45E-02	super_enhancer	promoter	COX6CP2
chr20	36485000	chr20	56135000	2.43E-02	super_enhancer	promoter	PCK1
chr20	36485000	chr20	61905000	1.63E-02	super_enhancer	promoter	NKAIN4
chr20	36495000	chr20	44115000	9.68E-04	super_enhancer	promoter	RP3-461P17.9
chr20	36495000	chr20	45625000	9.13E-04	super_enhancer	promoter	GAPDHP54
chr20	36495000	chr20	47035000	3.20E-02	super_enhancer	promoter	RP1-66N13.4
chr20	36605000	chr20	49755000	2.59E-02	promoter	super_enhancer	RN7SKP185
chr20	36795000	chr20	43925000	3.81E-02	promoter	super_enhancer	TGM2
chr20	36795000	chr20	47315000	3.15E-02	promoter	super_enhancer	TGM2

chr20	36795000	chr20	49755000	2.92E-02	promoter	super_enhancer	TGM2
chr20	36825000	chr20	42635000	2.65E-03	super_enhancer	promoter	RN7SL443P
chr20	36825000	chr20	62175000	2.02E-02	super_enhancer	promoter	RP4-697K14.15
chr20	36845000	chr20	52225000	2.74E-02	super_enhancer	promoter	AC005808.3
chr20	36845000	chr20	62375000	1.42E-02	super_enhancer	promoter	RP4-583P15.10
chr20	36855000	chr20	47105000	4.36E-02	super_enhancer	promoter	RP5-991B18.2
chr20	36855000	chr20	56725000	2.38E-02	super_enhancer	promoter	C20orf85
chr20	36855000	chr20	57555000	2.34E-02	super_enhancer	promoter	NELFCD
chr20	36855000	chr20	61925000	1.77E-02	super_enhancer	promoter	COL20A1
chr20	36955000	chr20	49675000	3.87E-02	promoter	super_enhancer	CTD-2308N23.2
chr20	36955000	chr20	50555000	2.62E-02	promoter	super_enhancer	CTD-2308N23.2
chr20	36955000	chr20	51335000	2.33E-02	promoter	super_enhancer	CTD-2308N23.2
chr20	36975000	chr20	47355000	3.77E-02	promoter	super_enhancer	LBP
chr20	36975000	chr20	50305000	3.23E-02	promoter	super_enhancer	LBP
chr20	36975000	chr20	50525000	3.73E-02	promoter	super_enhancer	LBP
chr20	36975000	chr20	51035000	3.53E-02	promoter	super_enhancer	LBP
chr20	37045000	chr20	43955000	4.05E-02	promoter	super_enhancer	RP4-564F22.5
chr20	37045000	chr20	50435000	2.68E-02	promoter	super_enhancer	RP4-564F22.5
chr20	37055000	chr20	47435000	1.05E-03	promoter	super_enhancer	SNORA71B
chr20	37055000	chr20	49785000	3.30E-02	promoter	super_enhancer	SNORA71B
chr20	37055000	chr20	50135000	3.59E-02	promoter	super_enhancer	SNORA71B
chr20	37055000	chr20	50155000	3.55E-02	promoter	super_enhancer	SNORA71B
chr20	37065000	chr20	50285000	3.23E-02	promoter	super_enhancer	SNHG17
chr20	37065000	chr20	50535000	3.23E-02	promoter	super_enhancer	SNHG17

chr20	37065000	chr20	56815000	1.85E-02	promoter	super_enhancer	SNHG17
chr20	37075000	chr20	50125000	3.35E-02	promoter	super_enhancer	SNHG11
chr20	37075000	chr20	50515000	3.19E-02	promoter	super_enhancer	SNHG11
chr20	37075000	chr20	62625000	2.08E-02	promoter	super_enhancer	SNHG11
chr20	37165000	chr20	50485000	2.61E-02	promoter	super_enhancer	RPS3P2
chr20	37245000	chr20	44695000	4.85E-02	promoter	super_enhancer	RP5-1100H13.3
chr20	37245000	chr20	44705000	4.14E-02	promoter	super_enhancer	RP5-1100H13.3
chr20	37245000	chr20	50145000	9.11E-04	promoter	super_enhancer	RP5-1100H13.3
chr20	37555000	chr20	41015000	2.88E-03	promoter	super_enhancer	FAM83D
chr20	37585000	chr20	50305000	2.82E-02	promoter	super_enhancer	RP4-616B8.5
chr20	37585000	chr20	51335000	2.49E-02	promoter	super_enhancer	RP4-616B8.5
chr20	38095000	chr20	52865000	1.67E-02	super_enhancer	promoter	AL133335.1
chr20	38095000	chr20	61905000	1.39E-02	super_enhancer	promoter	NKAIN4
chr20	38105000	chr20	44335000	4.36E-02	super_enhancer	promoter	WFDC10B
chr20	38105000	chr20	49995000	3.47E-02	super_enhancer	promoter	AL079339.1
chr20	38105000	chr20	52285000	2.40E-02	super_enhancer	promoter	RNU7-14P
chr20	38105000	chr20	56135000	2.43E-02	super_enhancer	promoter	PCK1
chr20	38125000	chr20	39825000	2.37E-02	super_enhancer	promoter	RPL23AP81
chr20	38135000	chr20	54875000	1.69E-02	super_enhancer	promoter	RP11-380D15.3
chr20	38135000	chr20	55905000	1.89E-02	super_enhancer	promoter	SPO11
chr20	38135000	chr20	61285000	1.60E-02	super_enhancer	promoter	RP11-93B14.10
chr20	38145000	chr20	55905000	1.85E-02	super_enhancer	promoter	SPO11
chr20	38155000	chr20	57765000	2.42E-02	super_enhancer	promoter	ZNF831
chr20	38165000	chr20	52105000	2.22E-02	super_enhancer	promoter	RP4-678D15.1

chr20	38165000	chr20	57285000	2.08E-02	super_enhancer	promoter	RP11-261P9.4
chr20	38175000	chr20	54875000	1.36E-02	super_enhancer	promoter	RP11-380D15.3
chr20	38175000	chr20	56535000	1.74E-02	super_enhancer	promoter	RP13-379L11.2
chr20	38185000	chr20	44045000	4.35E-02	super_enhancer	promoter	PIGT
chr20	38185000	chr20	56885000	1.81E-02	super_enhancer	promoter	PPP4R1L
chr20	38205000	chr20	57465000	1.02E-02	super_enhancer	promoter	RP1-309F20.3
chr20	38205000	chr20	58515000	1.51E-02	super_enhancer	promoter	PPP1R3D
chr20	38215000	chr20	40645000	3.17E-03	super_enhancer	promoter	RP5-1121H13.3
chr20	38215000	chr20	55905000	1.67E-02	super_enhancer	promoter	SPO11
chr20	39765000	chr20	51335000	2.65E-02	promoter	super_enhancer	PLCG1
chr20	39765000	chr20	53595000	2.47E-02	promoter	super_enhancer	PLCG1
chr20	39765000	chr20	56815000	1.90E-02	promoter	super_enhancer	PLCG1
chr20	39765000	chr20	57715000	2.41E-02	promoter	super_enhancer	PLCG1
chr20	39825000	chr20	49705000	3.11E-02	promoter	super_enhancer	RPL23AP81
chr20	39825000	chr20	56775000	2.35E-02	promoter	super_enhancer	RPL23AP81
chr20	39995000	chr20	51625000	3.24E-02	promoter	super_enhancer	EMILIN3
chr20	40115000	chr20	49775000	3.70E-02	promoter	super_enhancer	RP4-620E11.8
chr20	40115000	chr20	51745000	2.78E-02	promoter	super_enhancer	RP4-620E11.8
chr20	40175000	chr20	50565000	3.17E-02	promoter	super_enhancer	RPL12P11
chr20	40175000	chr20	51335000	2.26E-02	promoter	super_enhancer	RPL12P11
chr20	40175000	chr20	51635000	2.19E-02	promoter	super_enhancer	RPL12P11
chr20	40175000	chr20	62645000	1.71E-02	promoter	super_enhancer	RPL12P11
chr20	40995000	chr20	54025000	1.86E-02	super_enhancer	promoter	LINC01440
chr20	40995000	chr20	56135000	2.64E-02	super_enhancer	promoter	PCK1

chr20	40995000	chr20	57425000	1.53E-02	super_enhancer	promoter	GNAS-AS1_4
chr20	40995000	chr20	62585000	2.10E-02	super_enhancer	promoter	UCKL1-AS1
chr20	41005000	chr20	52685000	3.18E-02	super_enhancer	promoter	MIR4756
chr20	41015000	chr20	52105000	1.89E-02	super_enhancer	promoter	RP4-678D15.1
chr20	41015000	chr20	58515000	1.55E-02	super_enhancer	promoter	PPP1R3D
chr20	41025000	chr20	47835000	3.76E-02	super_enhancer	promoter	DDX27
chr20	41025000	chr20	57225000	1.15E-02	super_enhancer	promoter	STX16
chr20	41025000	chr20	57425000	1.21E-02	super_enhancer	promoter	GNAS-AS1_4
chr20	41235000	chr20	56775000	3.24E-02	promoter	super_enhancer	RP4-730D4.1
chr20	42135000	chr20	43965000	3.86E-02	promoter	super_enhancer	L3MBTL1
chr20	42135000	chr20	50495000	4.45E-02	promoter	super_enhancer	L3MBTL1
chr20	42135000	chr20	50535000	4.39E-02	promoter	super_enhancer	L3MBTL1
chr20	42135000	chr20	50545000	4.28E-02	promoter	super_enhancer	L3MBTL1
chr20	42295000	chr20	49755000	4.11E-02	promoter	super_enhancer	MYBL2
chr20	42295000	chr20	50305000	3.45E-02	promoter	super_enhancer	MYBL2
chr20	42295000	chr20	53595000	2.69E-02	promoter	super_enhancer	MYBL2
chr20	42295000	chr20	53915000	2.37E-02	promoter	super_enhancer	MYBL2
chr20	42425000	chr20	45335000	1.04E-02	promoter	super_enhancer	RNU6-639P
chr20	42425000	chr20	53595000	2.82E-02	promoter	super_enhancer	RNU6-639P
chr20	42425000	chr20	53925000	2.03E-02	promoter	super_enhancer	RNU6-639P
chr20	42635000	chr20	50285000	4.86E-02	promoter	super_enhancer	RN7SL443P
chr20	42875000	chr20	53595000	2.92E-02	promoter	super_enhancer	GDAP1L1
chr20	42875000	chr20	53635000	2.25E-02	promoter	super_enhancer	GDAP1L1
chr20	42875000	chr20	56785000	2.93E-02	promoter	super_enhancer	GDAP1L1

chr20	42965000	chr20	50195000	4.09E-02	promoter	super_enhancer	R3HDML
chr20	42965000	chr20	50535000	3.99E-02	promoter	super_enhancer	R3HDML
chr20	42985000	chr20	50305000	3.89E-02	promoter	super_enhancer	RP5-881L22.5
chr20	42985000	chr20	50495000	9.64E-04	promoter	super_enhancer	RP5-881L22.5
chr20	43025000	chr20	50495000	4.72E-02	promoter	super_enhancer	HNF4A-AS1
chr20	43025000	chr20	50515000	4.26E-02	promoter	super_enhancer	HNF4A-AS1
chr20	43055000	chr20	45325000	2.24E-02	promoter	super_enhancer	AL132772.1
chr20	43095000	chr20	47335000	3.72E-03	promoter	super_enhancer	C20orf62
chr20	43095000	chr20	50135000	4.48E-02	promoter	super_enhancer	C20orf62
chr20	43095000	chr20	56775000	3.58E-02	promoter	super_enhancer	C20orf62
chr20	43105000	chr20	49725000	3.92E-02	promoter	super_enhancer	TTPAL
chr20	43105000	chr20	50155000	4.23E-02	promoter	super_enhancer	TTPAL
chr20	43105000	chr20	50195000	4.37E-02	promoter	super_enhancer	TTPAL
chr20	43325000	chr20	56795000	2.20E-02	promoter	super_enhancer	LINC01260
chr20	43375000	chr20	50155000	4.63E-02	promoter	super_enhancer	KCNK15-AS1
chr20	43375000	chr20	56765000	2.73E-02	promoter	super_enhancer	KCNK15-AS1
chr20	43375000	chr20	57705000	2.43E-02	promoter	super_enhancer	KCNK15-AS1
chr20	43595000	chr20	50285000	4.88E-02	promoter	super_enhancer	STK4-AS1
chr20	43595000	chr20	50435000	3.80E-02	promoter	super_enhancer	STK4-AS1
chr20	43595000	chr20	50515000	4.38E-02	promoter	super_enhancer	STK4-AS1
chr20	43595000	chr20	50525000	4.94E-02	promoter	super_enhancer	STK4-AS1
chr20	43595000	chr20	56805000	3.26E-02	promoter	super_enhancer	STK4-AS1
chr20	43745000	chr20	51315000	3.39E-02	promoter	super_enhancer	WFDC5
chr20	43745000	chr20	57715000	3.13E-02	promoter	super_enhancer	WFDC5

chr20	43835000	chr20	44715000	3.95E-02	promoter	super_enhancer	SEMG1
chr20	43835000	chr20	50535000	4.40E-02	promoter	super_enhancer	SEMG1
chr20	43835000	chr20	50545000	4.29E-02	promoter	super_enhancer	SEMG1
chr20	43835000	chr20	51345000	3.35E-02	promoter	super_enhancer	SEMG1
chr20	43925000	chr20	55575000	2.70E-02	super_enhancer	promoter	AL117380.2
chr20	43935000	chr20	61435000	3.06E-02	super_enhancer	promoter	OGFR
chr20	43935000	chr20	62585000	3.22E-02	super_enhancer	promoter	UCKL1-AS1
chr20	43945000	chr20	44045000	3.19E-02	super_enhancer	promoter	PIGT
chr20	43945000	chr20	61505000	2.44E-02	super_enhancer	promoter	ARF4P2
chr20	43955000	chr20	61435000	2.08E-02	super_enhancer	promoter	OGFR
chr20	43965000	chr20	44045000	1.02E-03	super_enhancer	promoter	PIGT
chr20	43965000	chr20	44375000	2.98E-03	super_enhancer	promoter	HNRNPA1P3
chr20	43965000	chr20	54875000	2.69E-02	super_enhancer	promoter	RP11-380D15.3
chr20	43965000	chr20	57415000	2.98E-02	super_enhancer	promoter	GNAS
chr20	43965000	chr20	60295000	2.61E-02	super_enhancer	promoter	RP11-429E11.3
chr20	43975000	chr20	51825000	3.95E-02	super_enhancer	promoter	RP11-15M15.2
chr20	43985000	chr20	44375000	2.12E-02	super_enhancer	promoter	HNRNPA1P3
chr20	43985000	chr20	55305000	3.71E-02	super_enhancer	promoter	RP5-897D18.1
chr20	43985000	chr20	56285000	2.31E-02	super_enhancer	promoter	NKILA
chr20	43985000	chr20	58515000	2.61E-02	super_enhancer	promoter	PPP1R3D
chr20	43985000	chr20	60295000	2.82E-02	super_enhancer	promoter	RP11-429E11.3
chr20	43985000	chr20	62475000	2.49E-02	super_enhancer	promoter	C20orf181
chr20	44045000	chr20	50535000	4.82E-02	promoter	super_enhancer	PIGT
chr20	44045000	chr20	53905000	1.72E-02	promoter	super_enhancer	PIGT

chr20	44145000	chr20	53905000	1.90E-02	promoter	super_enhancer	SPINT3
chr20	44335000	chr20	50515000	4.65E-02	promoter	super_enhancer	WFDC10B
chr20	44335000	chr20	51355000	3.57E-02	promoter	super_enhancer	WFDC10B
chr20	44335000	chr20	57695000	2.63E-02	promoter	super_enhancer	WFDC10B
chr20	44485000	chr20	53775000	2.65E-02	promoter	super_enhancer	ZSWIM3
chr20	44485000	chr20	57695000	3.03E-02	promoter	super_enhancer	ZSWIM3
chr20	44605000	chr20	45325000	3.47E-03	promoter	super_enhancer	FTLP1
chr20	44605000	chr20	47465000	1.17E-02	promoter	super_enhancer	FTLP1
chr20	44685000	chr20	45095000	2.26E-03	super_enhancer	promoter	RP5-981L23.2
chr20	44685000	chr20	57415000	3.37E-02	super_enhancer	promoter	GNAS
chr20	44685000	chr20	58515000	2.73E-02	super_enhancer	promoter	PPP1R3D
chr20	44695000	chr20	57415000	3.15E-02	super_enhancer	promoter	GNAS
chr20	44695000	chr20	60295000	2.76E-02	super_enhancer	promoter	RP11-429E11.3
chr20	44695000	chr20	61285000	2.59E-02	super_enhancer	promoter	RP11-93B14.10
chr20	44715000	chr20	52285000	4.22E-02	super_enhancer	promoter	RNU7-14P
chr20	44715000	chr20	55935000	2.13E-02	super_enhancer	promoter	MTRNR2L3
chr20	44715000	chr20	62585000	3.04E-02	super_enhancer	promoter	UCKL1-AS1
chr20	44725000	chr20	45095000	4.82E-02	super_enhancer	promoter	RP5-981L23.2
chr20	44725000	chr20	55925000	2.40E-02	super_enhancer	promoter	RAE1
chr20	45095000	chr20	49775000	3.29E-03	promoter	super_enhancer	RP5-981L23.2
chr20	45325000	chr20	54875000	3.04E-02	super_enhancer	promoter	RP11-380D15.3
chr20	45335000	chr20	57415000	3.62E-02	super_enhancer	promoter	GNAS
chr20	45345000	chr20	57555000	3.55E-02	super_enhancer	promoter	NELFCD
chr20	45355000	chr20	62175000	3.04E-02	super_enhancer	promoter	RP4-697K14.15

chr20	45355000	chr20	62475000	3.00E-02	super_enhancer	promoter	C20orf181
chr20	45625000	chr20	51335000	4.89E-02	promoter	super_enhancer	GAPDHP54
chr20	45625000	chr20	56795000	3.69E-02	promoter	super_enhancer	GAPDHP54
chr20	46415000	chr20	47455000	9.39E-03	promoter	super_enhancer	SULF2
chr20	46415000	chr20	47465000	1.85E-03	promoter	super_enhancer	SULF2
chr20	46415000	chr20	47475000	4.85E-02	promoter	super_enhancer	SULF2
chr20	46415000	chr20	56805000	3.99E-02	promoter	super_enhancer	SULF2
chr20	47105000	chr20	56805000	4.64E-02	promoter	super_enhancer	RP5-991B18.2
chr20	47105000	chr20	62365000	3.62E-02	promoter	super_enhancer	RP5-991B18.2
chr20	47315000	chr20	54875000	3.27E-02	super_enhancer	promoter	RP11-380D15.3
chr20	47315000	chr20	60795000	3.44E-02	super_enhancer	promoter	HRH3
chr20	47335000	chr20	61285000	3.09E-02	super_enhancer	promoter	RP11-93B14.10
chr20	47355000	chr20	61285000	2.58E-02	super_enhancer	promoter	RP11-93B14.10
chr20	47435000	chr20	60795000	3.87E-02	super_enhancer	promoter	HRH3
chr20	47445000	chr20	56065000	3.51E-02	super_enhancer	promoter	HMGB1P1
chr20	47445000	chr20	56885000	3.87E-02	super_enhancer	promoter	PPP4R1L
chr20	47445000	chr20	57765000	4.27E-02	super_enhancer	promoter	ZNF831
chr20	47465000	chr20	56725000	4.69E-02	super_enhancer	promoter	C20orf85
chr20	47465000	chr20	61885000	2.75E-02	super_enhancer	promoter	FLJ16779
chr20	47475000	chr20	62585000	3.90E-02	super_enhancer	promoter	UCKL1-AS1
chr20	47475000	chr20	62665000	3.85E-02	super_enhancer	promoter	LINC00176
chr20	47835000	chr20	49775000	6.83E-03	promoter	super_enhancer	DDX27
chr20	47835000	chr20	50515000	1.31E-02	promoter	super_enhancer	DDX27
chr20	48185000	chr20	56785000	3.75E-02	promoter	super_enhancer	PTGIS

chr20	48445000	chr20	62355000	3.09E-02	promoter	super_enhancer	RP5-1041C10.3
chr20	49095000	chr20	53785000	3.85E-02	promoter	super_enhancer	COX6CP2
chr20	49175000	chr20	56785000	3.13E-02	promoter	super_enhancer	RN7SL672P
chr20	49555000	chr20	50505000	1.11E-02	promoter	super_enhancer	AL034553.1
chr20	49615000	chr20	50165000	2.37E-02	promoter	super_enhancer	RP5-955M13.4
chr20	49615000	chr20	50195000	2.31E-03	promoter	super_enhancer	RP5-955M13.4
chr20	49615000	chr20	50555000	3.30E-02	promoter	super_enhancer	RP5-955M13.4
chr20	49615000	chr20	50565000	1.84E-03	promoter	super_enhancer	RP5-955M13.4
chr20	49615000	chr20	51345000	3.12E-02	promoter	super_enhancer	RP5-955M13.4
chr20	49615000	chr20	51745000	2.39E-02	promoter	super_enhancer	RP5-955M13.4
chr20	49615000	chr20	56785000	4.78E-02	promoter	super_enhancer	RP5-955M13.4
chr20	49675000	chr20	55925000	4.20E-02	super_enhancer	promoter	RAE1
chr20	49685000	chr20	57875000	4.31E-02	super_enhancer	promoter	EDN3
chr20	49685000	chr20	61925000	3.23E-02	super_enhancer	promoter	COL20A1
chr20	49705000	chr20	62175000	3.40E-02	super_enhancer	promoter	RP4-697K14.15
chr20	49715000	chr20	57875000	4.61E-02	super_enhancer	promoter	EDN3
chr20	49715000	chr20	61285000	3.72E-02	super_enhancer	promoter	RP11-93B14.10
chr20	49725000	chr20	61265000	1.16E-02	super_enhancer	promoter	RP13-30A9.2
chr20	49755000	chr20	57415000	4.21E-02	super_enhancer	promoter	GNAS
chr20	49765000	chr20	55575000	4.65E-02	super_enhancer	promoter	AL117380.2
chr20	49765000	chr20	57555000	3.96E-02	super_enhancer	promoter	NELFCD
chr20	49765000	chr20	61435000	3.17E-02	super_enhancer	promoter	OGFR
chr20	49765000	chr20	62575000	2.55E-02	super_enhancer	promoter	MIR647
chr20	49765000	chr20	62585000	3.32E-02	super_enhancer	promoter	UCKL1-AS1

chr20	49785000	chr20	55905000	4.23E-02	super_enhancer	promoter	SPO11
chr20	49785000	chr20	57765000	3.84E-02	super_enhancer	promoter	ZNF831
chr20	49785000	chr20	62175000	2.64E-02	super_enhancer	promoter	RP4-697K14.15
chr20	49995000	chr20	50195000	2.46E-04	promoter	super_enhancer	AL079339.1
chr20	50125000	chr20	55035000	2.31E-03	super_enhancer	promoter	RPL39P
chr20	50125000	chr20	62585000	3.55E-02	super_enhancer	promoter	UCKL1-AS1
chr20	50135000	chr20	57285000	3.94E-02	super_enhancer	promoter	RP11-261P9.4
chr20	50135000	chr20	61265000	1.37E-02	super_enhancer	promoter	RP13-30A9.2
chr20	50145000	chr20	61435000	4.51E-02	super_enhancer	promoter	OGFR
chr20	50155000	chr20	55905000	4.93E-02	super_enhancer	promoter	SPO11
chr20	50155000	chr20	61505000	2.93E-02	super_enhancer	promoter	ARF4P2
chr20	50165000	chr20	55035000	3.42E-03	super_enhancer	promoter	RPL39P
chr20	50195000	chr20	60795000	4.08E-02	super_enhancer	promoter	HRH3
chr20	50285000	chr20	61735000	3.74E-02	super_enhancer	promoter	HAR1B
chr20	50305000	chr20	52105000	1.64E-02	super_enhancer	promoter	RP4-678D15.1
chr20	50305000	chr20	56535000	4.97E-02	super_enhancer	promoter	RP13-379L11.2
chr20	50305000	chr20	61285000	2.92E-02	super_enhancer	promoter	RP11-93B14.10
chr20	50385000	chr20	56775000	4.75E-02	promoter	super_enhancer	ATP9A
chr20	50435000	chr20	55035000	1.85E-03	super_enhancer	promoter	RPL39P
chr20	50435000	chr20	61285000	2.60E-02	super_enhancer	promoter	RP11-93B14.10
chr20	50435000	chr20	61735000	2.99E-02	super_enhancer	promoter	HAR1B
chr20	50485000	chr20	56885000	4.63E-02	super_enhancer	promoter	PPP4R1L
chr20	50485000	chr20	60795000	4.16E-02	super_enhancer	promoter	HRH3
chr20	50495000	chr20	52225000	3.43E-02	super_enhancer	promoter	AC005808.3

chr20	50495000	chr20	57225000	3.23E-02	super_enhancer	promoter	STX16
chr20	50495000	chr20	61905000	3.21E-02	super_enhancer	promoter	NKAIN4
chr20	50505000	chr20	57555000	1.62E-03	super_enhancer	promoter	NELFCD
chr20	50515000	chr20	57285000	3.95E-02	super_enhancer	promoter	RP11-261P9.4
chr20	50515000	chr20	57415000	4.26E-02	super_enhancer	promoter	GNAS
chr20	50525000	chr20	56285000	3.90E-02	super_enhancer	promoter	NKILA
chr20	50525000	chr20	61885000	2.68E-02	super_enhancer	promoter	FLJ16779
chr20	50535000	chr20	57555000	4.85E-02	super_enhancer	promoter	NELFCD
chr20	50535000	chr20	62175000	3.16E-02	super_enhancer	promoter	RP4-697K14.15
chr20	50545000	chr20	56285000	3.70E-02	super_enhancer	promoter	NKILA
chr20	50545000	chr20	60795000	4.12E-02	super_enhancer	promoter	HRH3
chr20	50555000	chr20	56885000	4.21E-02	super_enhancer	promoter	PPP4R1L
chr20	50565000	chr20	55925000	4.31E-02	super_enhancer	promoter	RAE1
chr20	51325000	chr20	57225000	2.32E-02	super_enhancer	promoter	STX16
chr20	51335000	chr20	51825000	3.00E-02	super_enhancer	promoter	RP11-15M15.2
chr20	51335000	chr20	57415000	4.31E-02	super_enhancer	promoter	GNAS
chr20	51345000	chr20	51825000	8.16E-03	super_enhancer	promoter	RP11-15M15.2
chr20	51365000	chr20	51825000	9.09E-03	super_enhancer	promoter	RP11-15M15.2
chr20	51365000	chr20	55925000	3.90E-02	super_enhancer	promoter	RAE1
chr20	51365000	chr20	59945000	3.36E-02	super_enhancer	promoter	WI2-8325B5.1
chr20	51365000	chr20	61285000	2.92E-02	super_enhancer	promoter	RP11-93B14.10
chr20	51375000	chr20	52105000	1.06E-02	super_enhancer	promoter	RP4-678D15.1
chr20	51375000	chr20	56135000	4.66E-02	super_enhancer	promoter	PCK1
chr20	51625000	chr20	60795000	4.21E-02	super_enhancer	promoter	HRH3

chr20	51625000	chr20	62585000	3.79E-02	super_enhancer	promoter	UCKL1-AS1
chr20	51635000	chr20	57875000	4.07E-02	super_enhancer	promoter	EDN3
chr20	51745000	chr20	56285000	4.93E-02	super_enhancer	promoter	NKILA
chr20	51745000	chr20	61435000	3.94E-02	super_enhancer	promoter	OGFR
chr20	51755000	chr20	57415000	4.59E-02	super_enhancer	promoter	GNAS
chr20	51755000	chr20	61905000	2.74E-02	super_enhancer	promoter	NKAIN4
chr20	53585000	chr20	61275000	2.55E-02	super_enhancer	promoter	RP11-93B14.6
chr20	53595000	chr20	58515000	4.76E-02	super_enhancer	promoter	PPP1R3D
chr20	53605000	chr20	60295000	3.37E-02	super_enhancer	promoter	RP11-429E11.3
chr20	53615000	chr20	55925000	4.35E-03	super_enhancer	promoter	RAE1
chr20	53625000	chr20	55905000	5.19E-04	super_enhancer	promoter	SPO11
chr20	53625000	chr20	56725000	5.31E-03	super_enhancer	promoter	C20orf85
chr20	53635000	chr20	60295000	3.17E-02	super_enhancer	promoter	RP11-429E11.3
chr20	53785000	chr20	54875000	3.88E-02	super_enhancer	promoter	RP11-380D15.3
chr20	53915000	chr20	59945000	4.19E-02	super_enhancer	promoter	WI2-8325B5.1
chr20	53915000	chr20	60795000	4.29E-02	super_enhancer	promoter	HRH3
chr20	56065000	chr20	56795000	9.41E-03	promoter	super_enhancer	HMGB1P1
chr20	56135000	chr20	56785000	9.79E-03	promoter	super_enhancer	PCK1
chr20	56285000	chr20	62355000	4.11E-02	promoter	super_enhancer	NKILA
chr20	56785000	chr20	61275000	2.16E-03	super_enhancer	promoter	RP11-93B14.6
chr20	56795000	chr20	57415000	7.92E-03	super_enhancer	promoter	GNAS
chr20	56795000	chr20	62475000	1.61E-03	super_enhancer	promoter	C20orf181
chr20	56805000	chr20	57415000	8.68E-03	super_enhancer	promoter	GNAS
chr20	56815000	chr20	57465000	1.14E-02	super_enhancer	promoter	RP1-309F20.3

chr20	56815000	chr20	57555000	4.28E-02	super_enhancer	promoter	NELFCD
chr20	56815000	chr20	61925000	4.46E-02	super_enhancer	promoter	COL20A1
chr20	61885000	chr20	62655000	9.21E-03	promoter	super_enhancer	FLJ16779
chr20	61905000	chr20	62655000	2.00E-02	promoter	super_enhancer	NKAIN4
chr20	62355000	chr20	62475000	2.77E-02	super_enhancer	promoter	C20orf181
chr20	62365000	chr20	62585000	2.18E-02	super_enhancer	promoter	UCKL1-AS1
chr20	62645000	chr20	62665000	3.59E-02	super_enhancer	promoter	LINC00176
chr20	62655000	chr20	62665000	1.42E-03	super_enhancer	promoter	LINC00176
chr21	10205000	chr21	32475000	7.47E-03	promoter	super_enhancer	SNX18P12
chr21	15095000	chr21	33395000	6.99E-03	promoter	super_enhancer	CH507-210P18.4
chr21	15135000	chr21	39005000	7.67E-03	promoter	super_enhancer	CH507-210P18.3
chr21	15135000	chr21	42685000	7.42E-03	promoter	super_enhancer	CH507-210P18.3
chr21	15165000	chr21	33385000	1.32E-02	promoter	super_enhancer	FAM207CP
chr21	15165000	chr21	38995000	8.96E-03	promoter	super_enhancer	FAM207CP
chr21	15515000	chr21	29205000	9.39E-03	promoter	super_enhancer	ERLEC1P1
chr21	15955000	chr21	34885000	1.30E-02	promoter	super_enhancer	SAMSN1-AS1
chr21	16775000	chr21	29185000	1.59E-02	promoter	super_enhancer	AJ006998.2
chr21	17495000	chr21	42205000	9.30E-03	promoter	super_enhancer	RPS26P5
chr21	18015000	chr21	41775000	6.77E-03	promoter	super_enhancer	AP000473.8
chr21	18585000	chr21	45355000	9.21E-03	promoter	super_enhancer	NEK4P1
chr21	18885000	chr21	29165000	1.73E-02	promoter	super_enhancer	CXADR
chr21	19165000	chr21	45355000	8.70E-03	promoter	super_enhancer	C21orf91-OT1
chr21	20965000	chr21	29225000	1.12E-02	promoter	super_enhancer	RPL37P4
chr21	20965000	chr21	43305000	8.81E-03	promoter	super_enhancer	RPL37P4

chr21	24255000	chr21	42185000	1.16E-02	promoter	super_enhancer	AP000949.1
chr21	24255000	chr21	43305000	1.11E-02	promoter	super_enhancer	AP000949.1
chr21	24465000	chr21	32465000	1.15E-02	promoter	super_enhancer	ZNF299P
chr21	24465000	chr21	34855000	1.29E-02	promoter	super_enhancer	ZNF299P
chr21	25215000	chr21	29175000	3.02E-02	promoter	super_enhancer	AP000960.1
chr21	25215000	chr21	42665000	9.06E-03	promoter	super_enhancer	AP000960.1
chr21	25415000	chr21	29195000	3.46E-02	promoter	super_enhancer	AP000477.3
chr21	25415000	chr21	42205000	1.17E-02	promoter	super_enhancer	AP000477.3
chr21	25695000	chr21	33395000	1.76E-02	promoter	super_enhancer	AP000469.2
chr21	26275000	chr21	33405000	1.94E-02	promoter	super_enhancer	AP000402.3
chr21	26405000	chr21	29215000	2.76E-02	promoter	super_enhancer	AP001342.1
chr21	26805000	chr21	32475000	2.96E-02	promoter	super_enhancer	LINC00158
chr21	26805000	chr21	34885000	2.10E-02	promoter	super_enhancer	LINC00158
chr21	26945000	chr21	29195000	4.56E-02	promoter	super_enhancer	MIR155
chr21	26945000	chr21	43345000	9.09E-03	promoter	super_enhancer	MIR155
chr21	27015000	chr21	34825000	2.06E-02	promoter	super_enhancer	RNGTTP1
chr21	27315000	chr21	34865000	2.84E-02	promoter	super_enhancer	RNU6-123P
chr21	27315000	chr21	38905000	1.94E-02	promoter	super_enhancer	RNU6-123P
chr21	27315000	chr21	39005000	1.96E-02	promoter	super_enhancer	RNU6-123P
chr21	27315000	chr21	41765000	1.13E-02	promoter	super_enhancer	RNU6-123P
chr21	27315000	chr21	42215000	1.09E-02	promoter	super_enhancer	RNU6-123P
chr21	27765000	chr21	29175000	1.00E-02	promoter	super_enhancer	CYYR1-AS1
chr21	27765000	chr21	42225000	1.61E-02	promoter	super_enhancer	CYYR1-AS1
chr21	27765000	chr21	43355000	1.47E-02	promoter	super_enhancer	CYYR1-AS1

chr21	28325000	chr21	32465000	2.80E-02	promoter	super_enhancer	MIR4759
chr21	28675000	chr21	32455000	4.51E-02	promoter	super_enhancer	AP001604.1
chr21	29095000	chr21	43575000	2.31E-02	promoter	super_enhancer	LINC00113
chr21	29165000	chr21	32435000	4.62E-02	super_enhancer	promoter	UBE3AP2
chr21	29175000	chr21	31915000	4.78E-02	super_enhancer	promoter	KRTAP19-6
chr21	29175000	chr21	31935000	4.03E-02	super_enhancer	promoter	KRTAP19-7
chr21	29175000	chr21	32015000	4.89E-02	super_enhancer	promoter	KRTAP20-3
chr21	29185000	chr21	33635000	4.63E-02	super_enhancer	promoter	AP000265.1
chr21	29185000	chr21	35445000	3.15E-02	super_enhancer	promoter	MRPS6
chr21	29195000	chr21	32435000	4.67E-02	super_enhancer	promoter	UBE3AP2
chr21	29195000	chr21	40985000	1.69E-02	super_enhancer	promoter	B3GALT5-AS1
chr21	29215000	chr21	32175000	2.94E-02	super_enhancer	promoter	KRTAP8-2P
chr21	29215000	chr21	33935000	1.89E-02	super_enhancer	promoter	RNA5SP490
chr21	29215000	chr21	47185000	8.85E-03	super_enhancer	promoter	RP1-101D8.1
chr21	30365000	chr21	43315000	1.94E-02	promoter	super_enhancer	LTN1
chr21	30365000	chr21	43325000	2.66E-02	promoter	super_enhancer	LTN1
chr21	30365000	chr21	43595000	2.45E-02	promoter	super_enhancer	LTN1
chr21	30565000	chr21	43595000	2.77E-02	promoter	super_enhancer	LINC00189
chr21	30565000	chr21	44865000	2.04E-02	promoter	super_enhancer	LINC00189
chr21	30595000	chr21	38925000	2.81E-02	promoter	super_enhancer	GAPDHP14
chr21	30595000	chr21	42235000	1.84E-02	promoter	super_enhancer	GAPDHP14
chr21	30595000	chr21	43295000	1.92E-02	promoter	super_enhancer	GAPDHP14
chr21	30595000	chr21	43355000	2.09E-02	promoter	super_enhancer	GAPDHP14
chr21	30595000	chr21	43725000	1.77E-02	promoter	super_enhancer	GAPDHP14

chr21	31655000	chr21	41785000	6.31E-03	promoter	super_enhancer	KRTAP24-1
chr21	31865000	chr21	42205000	1.62E-02	promoter	super_enhancer	KRTAP19-3
chr21	31915000	chr21	42205000	1.62E-02	promoter	super_enhancer	KRTAP19-6
chr21	31915000	chr21	44875000	1.83E-02	promoter	super_enhancer	KRTAP19-6
chr21	31935000	chr21	32465000	1.19E-02	promoter	super_enhancer	KRTAP19-7
chr21	31965000	chr21	33405000	1.46E-02	promoter	super_enhancer	KRTAP6-3
chr21	32015000	chr21	41785000	1.05E-02	promoter	super_enhancer	KRTAP20-3
chr21	32015000	chr21	42175000	1.43E-02	promoter	super_enhancer	KRTAP20-3
chr21	32015000	chr21	43715000	1.53E-02	promoter	super_enhancer	KRTAP20-3
chr21	32015000	chr21	45325000	2.08E-02	promoter	super_enhancer	KRTAP20-3
chr21	32135000	chr21	45335000	1.61E-02	promoter	super_enhancer	AP000244.11
chr21	32255000	chr21	32445000	1.96E-02	promoter	super_enhancer	KRTAP11-1
chr21	32255000	chr21	43335000	1.69E-02	promoter	super_enhancer	KRTAP11-1
chr21	32435000	chr21	32445000	2.85E-02	promoter	super_enhancer	UBE3AP2
chr21	32435000	chr21	43305000	1.72E-02	promoter	super_enhancer	UBE3AP2
chr21	32435000	chr21	43595000	2.08E-02	promoter	super_enhancer	UBE3AP2
chr21	32445000	chr21	33635000	3.84E-02	super_enhancer	promoter	AP000265.1
chr21	32445000	chr21	41885000	1.68E-02	super_enhancer	promoter	SNORA51
chr21	32475000	chr21	47945000	1.97E-02	super_enhancer	promoter	RNU6-396P
chr21	32825000	chr21	41775000	1.71E-02	promoter	super_enhancer	AP000563.2
chr21	32825000	chr21	42195000	2.39E-02	promoter	super_enhancer	AP000563.2
chr21	32825000	chr21	43295000	2.11E-02	promoter	super_enhancer	AP000563.2
chr21	33105000	chr21	42665000	1.82E-02	promoter	super_enhancer	SCAF4
chr21	33395000	chr21	40865000	1.88E-02	super_enhancer	promoter	RPS26P4

chr21	33405000	chr21	44035000	2.20E-02	super_enhancer	promoter	AP001626.1
chr21	33635000	chr21	43335000	1.99E-02	promoter	super_enhancer	AP000265.1
chr21	33765000	chr21	43275000	2.03E-02	promoter	super_enhancer	URB1
chr21	33935000	chr21	43295000	1.81E-02	promoter	super_enhancer	RNA5SP490
chr21	34145000	chr21	38925000	4.48E-02	promoter	super_enhancer	PAXBP1
chr21	34145000	chr21	45365000	2.30E-02	promoter	super_enhancer	PAXBP1
chr21	34165000	chr21	34815000	6.13E-03	promoter	super_enhancer	AP000280.66
chr21	34165000	chr21	34835000	2.40E-03	promoter	super_enhancer	AP000280.66
chr21	34165000	chr21	34845000	2.02E-02	promoter	super_enhancer	AP000280.66
chr21	34165000	chr21	43315000	2.03E-02	promoter	super_enhancer	AP000280.66
chr21	34215000	chr21	34845000	4.13E-02	promoter	super_enhancer	AP000281.1
chr21	34215000	chr21	34855000	1.30E-02	promoter	super_enhancer	AP000281.1
chr21	34215000	chr21	43335000	2.39E-02	promoter	super_enhancer	AP000281.1
chr21	34815000	chr21	44395000	2.95E-02	super_enhancer	promoter	PKNOX1
chr21	34815000	chr21	45595000	2.93E-02	super_enhancer	promoter	AP001056.1
chr21	34815000	chr21	46825000	1.89E-02	super_enhancer	promoter	LL21NC02-21A1.1
chr21	34825000	chr21	35885000	4.90E-02	super_enhancer	promoter	KCNE1
chr21	34825000	chr21	40685000	5.84E-04	super_enhancer	promoter	BRWD1-AS2
chr21	34825000	chr21	40985000	3.44E-02	super_enhancer	promoter	B3GALT5-AS1
chr21	34845000	chr21	40985000	3.15E-02	super_enhancer	promoter	B3GALT5-AS1
chr21	34845000	chr21	45595000	2.47E-02	super_enhancer	promoter	AP001056.1
chr21	34845000	chr21	48075000	1.07E-02	super_enhancer	promoter	DSTNP1
chr21	34855000	chr21	46295000	2.04E-02	super_enhancer	promoter	PTTG1IP
chr21	34875000	chr21	45905000	2.01E-02	super_enhancer	promoter	AP001065.15

chr21	34885000	chr21	40685000	3.63E-02	super_enhancer	promoter	BRWD1-AS2
chr21	34885000	chr21	40985000	3.68E-02	super_enhancer	promoter	B3GALT5-AS1
chr21	35015000	chr21	43375000	2.54E-04	promoter	super_enhancer	ITSN1
chr21	35305000	chr21	41775000	1.73E-02	promoter	super_enhancer	AP000304.12
chr21	35305000	chr21	43285000	2.38E-02	promoter	super_enhancer	AP000304.12
chr21	35735000	chr21	42225000	3.81E-02	promoter	super_enhancer	KCNE2
chr21	35885000	chr21	42205000	2.72E-02	promoter	super_enhancer	KCNE1
chr21	35885000	chr21	43375000	2.66E-02	promoter	super_enhancer	KCNE1
chr21	35885000	chr21	43725000	2.06E-02	promoter	super_enhancer	KCNE1
chr21	37505000	chr21	43375000	4.24E-02	promoter	super_enhancer	RPS9P1
chr21	37505000	chr21	45375000	2.69E-02	promoter	super_enhancer	RPS9P1
chr21	38075000	chr21	43315000	4.63E-02	promoter	super_enhancer	AP000697.6
chr21	38075000	chr21	44875000	4.68E-02	promoter	super_enhancer	AP000697.6
chr21	38315000	chr21	41775000	4.26E-02	promoter	super_enhancer	DPRXP5
chr21	38445000	chr21	38995000	9.15E-03	promoter	super_enhancer	PIGP
chr21	38445000	chr21	43715000	3.66E-02	promoter	super_enhancer	PIGP
chr21	38445000	chr21	45055000	3.07E-02	promoter	super_enhancer	PIGP
chr21	38905000	chr21	47745000	2.51E-02	super_enhancer	promoter	C21orf58
chr21	38915000	chr21	42935000	4.16E-02	super_enhancer	promoter	AP001610.9
chr21	38925000	chr21	40035000	4.56E-02	super_enhancer	promoter	ERG
chr21	38925000	chr21	40555000	2.35E-02	super_enhancer	promoter	AF129408.17
chr21	39005000	chr21	39705000	3.90E-03	super_enhancer	promoter	LINC01423
chr21	39005000	chr21	40545000	1.59E-02	super_enhancer	promoter	PCBP2P1
chr21	39005000	chr21	40685000	1.61E-02	super_enhancer	promoter	BRWD1-AS2

chr21	39005000	chr21	47475000	2.60E-02	super_enhancer	promoter	AP001476.3
chr21	39015000	chr21	45905000	3.10E-02	super_enhancer	promoter	AP001065.15
chr21	39015000	chr21	47675000	3.19E-02	super_enhancer	promoter	AP001469.9
chr21	39705000	chr21	45355000	3.78E-02	promoter	super_enhancer	LINC01423
chr21	40035000	chr21	45355000	3.54E-02	promoter	super_enhancer	ERG
chr21	40545000	chr21	43305000	2.65E-03	promoter	super_enhancer	PCBP2P1
chr21	40545000	chr21	44885000	3.56E-02	promoter	super_enhancer	PCBP2P1
chr21	40865000	chr21	42225000	1.60E-03	promoter	super_enhancer	RPS26P4
chr21	41585000	chr21	41775000	8.41E-03	promoter	super_enhancer	MIR4760
chr21	42665000	chr21	47745000	3.01E-02	super_enhancer	promoter	C21orf58
chr21	42675000	chr21	47575000	3.59E-02	super_enhancer	promoter	FTCD
chr21	43295000	chr21	46825000	4.96E-02	super_enhancer	promoter	LL21NC02-21A1.1
chr21	43315000	chr21	47575000	4.31E-02	super_enhancer	promoter	FTCD
chr21	43325000	chr21	44385000	1.83E-02	super_enhancer	promoter	AP001630.5
chr21	43335000	chr21	48025000	3.10E-02	super_enhancer	promoter	S100B
chr21	43345000	chr21	47745000	1.76E-03	super_enhancer	promoter	C21orf58
chr21	43585000	chr21	47575000	4.37E-02	super_enhancer	promoter	FTCD
chr21	43585000	chr21	47705000	4.85E-02	super_enhancer	promoter	YBEY
chr21	43605000	chr21	48075000	1.97E-02	super_enhancer	promoter	DSTNP1
chr21	43725000	chr21	46495000	3.68E-03	super_enhancer	promoter	SSR4P1
chr21	43725000	chr21	47945000	4.61E-02	super_enhancer	promoter	RNU6-396P
chr21	44385000	chr21	44865000	1.61E-02	promoter	super_enhancer	AP001630.5
chr21	44925000	chr21	45055000	5.01E-03	promoter	super_enhancer	CH507-42P11.3
chr21	45055000	chr21	47475000	5.69E-03	super_enhancer	promoter	AP001476.3

chr21	45285000	chr21	45365000	1.22E-03	promoter	super_enhancer	AGPAT3
chr21	45325000	chr21	46825000	2.47E-03	super_enhancer	promoter	LL21NC02-21A1.1
chr21	45335000	chr21	46105000	3.44E-02	super_enhancer	promoter	RP1-225L15.1
chr22	16905000	chr22	42935000	8.65E-03	promoter	super_enhancer	PABPC1P9
chr22	17065000	chr22	46085000	7.82E-03	promoter	super_enhancer	KCNMB3P1
chr22	17075000	chr22	49945000	1.06E-02	promoter	super_enhancer	CCT8L2
chr22	17185000	chr22	38715000	9.65E-03	promoter	super_enhancer	VWFP1
chr22	17415000	chr22	50295000	9.33E-03	promoter	super_enhancer	IGKV1OR22-1
chr22	17515000	chr22	37165000	2.24E-02	promoter	super_enhancer	AC006548.28
chr22	17515000	chr22	42925000	1.65E-02	promoter	super_enhancer	AC006548.28
chr22	17515000	chr22	46055000	1.47E-02	promoter	super_enhancer	AC006548.28
chr22	17565000	chr22	31635000	1.36E-02	promoter	super_enhancer	IL17RA
chr22	17565000	chr22	35585000	1.24E-02	promoter	super_enhancer	IL17RA
chr22	17565000	chr22	37315000	1.06E-02	promoter	super_enhancer	IL17RA
chr22	17575000	chr22	23235000	3.61E-02	promoter	super_enhancer	AC006946.12
chr22	17575000	chr22	37685000	1.62E-02	promoter	super_enhancer	AC006946.12
chr22	17835000	chr22	31635000	1.49E-02	promoter	super_enhancer	RN7SL843P
chr22	18295000	chr22	42745000	1.84E-02	promoter	super_enhancer	XXbac-B461K10.4
chr22	18295000	chr22	46515000	1.59E-02	promoter	super_enhancer	XXbac-B461K10.4
chr22	18565000	chr22	23235000	4.49E-02	promoter	super_enhancer	XXbac-B476C20.11
chr22	18565000	chr22	36365000	9.09E-03	promoter	super_enhancer	XXbac-B476C20.11
chr22	18565000	chr22	37325000	1.61E-02	promoter	super_enhancer	XXbac-B476C20.11
chr22	18565000	chr22	46525000	1.13E-02	promoter	super_enhancer	XXbac-B476C20.11
chr22	18615000	chr22	30255000	1.95E-02	promoter	super_enhancer	AC008079.10

chr22	18925000	chr22	37185000	1.14E-02	promoter	super_enhancer	PRODH
chr22	18985000	chr22	19895000	1.73E-02	promoter	super_enhancer	AC007326.9
chr22	18985000	chr22	24965000	3.91E-02	promoter	super_enhancer	AC007326.9
chr22	18985000	chr22	36355000	7.94E-03	promoter	super_enhancer	AC007326.9
chr22	18985000	chr22	37195000	1.59E-02	promoter	super_enhancer	AC007326.9
chr22	18985000	chr22	49955000	1.36E-02	promoter	super_enhancer	AC007326.9
chr22	19005000	chr22	29615000	1.89E-02	promoter	super_enhancer	DGCR9
chr22	19175000	chr22	19245000	1.41E-02	super_enhancer	promoter	KRT18P62
chr22	19175000	chr22	19555000	3.07E-02	super_enhancer	promoter	LINC00895
chr22	19175000	chr22	26825000	2.68E-02	super_enhancer	promoter	ASPHD2
chr22	19175000	chr22	30835000	3.90E-02	super_enhancer	promoter	RP4-539M6.18
chr22	19185000	chr22	24645000	3.19E-02	super_enhancer	promoter	AP000354.4
chr22	19185000	chr22	27015000	3.16E-02	super_enhancer	promoter	CRYBB1
chr22	19185000	chr22	39775000	1.81E-02	super_enhancer	promoter	RP3-333H23.9
chr22	19185000	chr22	46155000	9.75E-03	super_enhancer	promoter	MIR4762
chr22	19185000	chr22	50645000	1.77E-02	super_enhancer	promoter	RP3-402G11.27
chr22	19185000	chr22	50765000	1.89E-02	super_enhancer	promoter	DENND6B
chr22	19245000	chr22	30255000	2.10E-02	promoter	super_enhancer	KRT18P62
chr22	19245000	chr22	31635000	1.90E-02	promoter	super_enhancer	KRT18P62
chr22	19245000	chr22	38225000	1.68E-02	promoter	super_enhancer	KRT18P62
chr22	19435000	chr22	31645000	2.03E-02	promoter	super_enhancer	HIRA
chr22	19435000	chr22	36355000	7.43E-03	promoter	super_enhancer	HIRA
chr22	19435000	chr22	46775000	1.30E-02	promoter	super_enhancer	HIRA
chr22	19515000	chr22	42935000	1.78E-02	promoter	super_enhancer	CLDN5

chr22	19515000	chr22	49935000	1.43E-02	promoter	super_enhancer	CLDN5
chr22	19655000	chr22	19895000	4.29E-02	promoter	super_enhancer	AC000067.1
chr22	19655000	chr22	43115000	2.16E-02	promoter	super_enhancer	AC000067.1
chr22	19655000	chr22	45915000	1.77E-02	promoter	super_enhancer	AC000067.1
chr22	19875000	chr22	26825000	2.68E-02	super_enhancer	promoter	ASPHD2
chr22	19875000	chr22	31795000	2.97E-02	super_enhancer	promoter	DRG1
chr22	19875000	chr22	42075000	2.28E-02	super_enhancer	promoter	RNU6-476P
chr22	19875000	chr22	45575000	1.99E-02	super_enhancer	promoter	CTA-268H5.12
chr22	19875000	chr22	49175000	1.47E-02	super_enhancer	promoter	MIR4535
chr22	19885000	chr22	24335000	3.67E-02	super_enhancer	promoter	AP000351.4
chr22	19885000	chr22	25835000	3.60E-02	super_enhancer	promoter	IGLVIVOR22-1
chr22	19885000	chr22	42085000	2.03E-02	super_enhancer	promoter	C22orf46
chr22	19885000	chr22	43795000	2.06E-02	super_enhancer	promoter	RP4-754E20__A.5
chr22	19895000	chr22	24235000	4.57E-02	super_enhancer	promoter	MIF
chr22	19895000	chr22	43435000	1.63E-02	super_enhancer	promoter	AL022476.2
chr22	20005000	chr22	24995000	2.04E-03	promoter	super_enhancer	ARVCF
chr22	20005000	chr22	37345000	2.05E-02	promoter	super_enhancer	ARVCF
chr22	20005000	chr22	37765000	1.96E-02	promoter	super_enhancer	ARVCF
chr22	20005000	chr22	42925000	2.04E-02	promoter	super_enhancer	ARVCF
chr22	20005000	chr22	43105000	1.82E-02	promoter	super_enhancer	ARVCF
chr22	20105000	chr22	37685000	2.26E-02	promoter	super_enhancer	TRMT2A
chr22	20105000	chr22	41445000	1.58E-02	promoter	super_enhancer	TRMT2A
chr22	20105000	chr22	45945000	1.58E-02	promoter	super_enhancer	TRMT2A
chr22	20205000	chr22	23605000	4.98E-02	super_enhancer	promoter	RN7SL263P

chr22	20205000	chr22	29875000	2.14E-02	super_enhancer	promoter	RFPL1S
chr22	20205000	chr22	31955000	1.66E-02	super_enhancer	promoter	RP11-247I13.3
chr22	20205000	chr22	42395000	1.32E-02	super_enhancer	promoter	WBP2NL
chr22	20205000	chr22	44965000	1.13E-02	super_enhancer	promoter	LINC00207
chr22	20745000	chr22	37675000	1.38E-02	promoter	super_enhancer	USP41
chr22	20745000	chr22	43095000	1.61E-02	promoter	super_enhancer	USP41
chr22	21025000	chr22	24995000	4.70E-02	promoter	super_enhancer	ABHD17AP4
chr22	21025000	chr22	38695000	1.43E-02	promoter	super_enhancer	ABHD17AP4
chr22	21055000	chr22	42955000	5.07E-03	promoter	super_enhancer	TMEM191A
chr22	21355000	chr22	46775000	1.39E-02	promoter	super_enhancer	THAP7-AS1
chr22	21385000	chr22	31635000	1.65E-02	promoter	super_enhancer	AC002472.11
chr22	21385000	chr22	46495000	1.34E-02	promoter	super_enhancer	AC002472.11
chr22	21985000	chr22	37785000	1.67E-02	promoter	super_enhancer	YDJC
chr22	21985000	chr22	46505000	1.45E-02	promoter	super_enhancer	YDJC
chr22	21995000	chr22	42765000	1.19E-02	promoter	super_enhancer	KB-1440D3.14
chr22	22015000	chr22	36355000	8.20E-03	promoter	super_enhancer	KB-1440D3.13
chr22	22015000	chr22	42955000	7.91E-03	promoter	super_enhancer	KB-1440D3.13
chr22	22015000	chr22	49935000	1.25E-02	promoter	super_enhancer	KB-1440D3.13
chr22	22385000	chr22	42925000	1.48E-02	promoter	super_enhancer	IGLV4-69
chr22	22415000	chr22	37785000	1.69E-02	promoter	super_enhancer	IGLVV-66
chr22	22425000	chr22	42785000	1.63E-02	promoter	super_enhancer	IGLVIV-65
chr22	22425000	chr22	42945000	1.49E-02	promoter	super_enhancer	IGLVIV-65
chr22	22585000	chr22	23255000	3.15E-02	promoter	super_enhancer	LL22NC03-123E1.5
chr22	22585000	chr22	37205000	2.09E-02	promoter	super_enhancer	LL22NC03-123E1.5

chr22	22585000	chr22	42945000	1.52E-02	promoter	super_enhancer	LL22NC03-123E1.5
chr22	22735000	chr22	23215000	1.79E-03	promoter	super_enhancer	IGLV1-44
chr22	22735000	chr22	23245000	3.25E-02	promoter	super_enhancer	IGLV1-44
chr22	22735000	chr22	37255000	1.67E-02	promoter	super_enhancer	IGLV1-44
chr22	22735000	chr22	37305000	1.80E-02	promoter	super_enhancer	IGLV1-44
chr22	22735000	chr22	38225000	1.69E-02	promoter	super_enhancer	IGLV1-44
chr22	22775000	chr22	23255000	8.42E-03	promoter	super_enhancer	ASH2LP1
chr22	22775000	chr22	29625000	2.19E-02	promoter	super_enhancer	ASH2LP1
chr22	22775000	chr22	35585000	1.47E-02	promoter	super_enhancer	ASH2LP1
chr22	22785000	chr22	23225000	3.48E-03	promoter	super_enhancer	IGLV1-36
chr22	22785000	chr22	30825000	3.26E-02	promoter	super_enhancer	IGLV1-36
chr22	22805000	chr22	23215000	6.50E-03	promoter	super_enhancer	IGLV7-35
chr22	23075000	chr22	23245000	4.64E-02	promoter	super_enhancer	LL22NC03-48A11.14
chr22	23075000	chr22	37245000	1.58E-02	promoter	super_enhancer	LL22NC03-48A11.14
chr22	23075000	chr22	42915000	9.98E-03	promoter	super_enhancer	LL22NC03-48A11.14
chr22	23105000	chr22	23235000	4.88E-02	promoter	super_enhancer	IGLV3-13
chr22	23135000	chr22	23215000	1.30E-02	promoter	super_enhancer	AC244157.1
chr22	23135000	chr22	36435000	1.23E-02	promoter	super_enhancer	AC244157.1
chr22	23135000	chr22	37195000	1.52E-02	promoter	super_enhancer	AC244157.1
chr22	23135000	chr22	37685000	1.84E-02	promoter	super_enhancer	AC244157.1
chr22	23165000	chr22	23215000	4.13E-02	promoter	super_enhancer	IGLV2-8
chr22	23165000	chr22	30255000	2.14E-02	promoter	super_enhancer	IGLV2-8
chr22	23215000	chr22	47655000	1.16E-02	super_enhancer	promoter	RP1-111J24.1
chr22	23225000	chr22	23745000	8.13E-03	super_enhancer	promoter	ZDHHC8P1

chr22	23225000	chr22	30115000	3.77E-02	super_enhancer	promoter	CABP7
chr22	23225000	chr22	42475000	1.75E-02	super_enhancer	promoter	SMDT1
chr22	23235000	chr22	31065000	2.48E-02	super_enhancer	promoter	DUSP18
chr22	23235000	chr22	33455000	2.02E-02	super_enhancer	promoter	SYN3
chr22	23245000	chr22	27015000	3.95E-02	super_enhancer	promoter	CRYBB1
chr22	23245000	chr22	47655000	9.98E-03	super_enhancer	promoter	RP1-111J24.1
chr22	23255000	chr22	23735000	1.96E-02	super_enhancer	promoter	KB-1269D1.8
chr22	23255000	chr22	26955000	4.94E-02	super_enhancer	promoter	HMGB1P10
chr22	23255000	chr22	31955000	2.33E-02	super_enhancer	promoter	RP11-247I13.3
chr22	23255000	chr22	42105000	2.06E-02	super_enhancer	promoter	HMGN2P10
chr22	23265000	chr22	42725000	1.80E-02	promoter	super_enhancer	IGLC7
chr22	23605000	chr22	24965000	2.64E-02	promoter	super_enhancer	RN7SL263P
chr22	23605000	chr22	36365000	9.95E-03	promoter	super_enhancer	RN7SL263P
chr22	23605000	chr22	43115000	1.87E-02	promoter	super_enhancer	RN7SL263P
chr22	23605000	chr22	49965000	1.10E-02	promoter	super_enhancer	RN7SL263P
chr22	23605000	chr22	49975000	9.68E-03	promoter	super_enhancer	RN7SL263P
chr22	23665000	chr22	36395000	9.25E-03	promoter	super_enhancer	AP000343.1
chr22	23735000	chr22	30225000	3.57E-02	promoter	super_enhancer	KB-1269D1.8
chr22	23735000	chr22	31645000	2.76E-02	promoter	super_enhancer	KB-1269D1.8
chr22	23735000	chr22	37305000	2.05E-02	promoter	super_enhancer	KB-1269D1.8
chr22	23735000	chr22	37665000	2.06E-02	promoter	super_enhancer	KB-1269D1.8
chr22	23745000	chr22	36365000	9.85E-03	promoter	super_enhancer	ZDHHC8P1
chr22	23745000	chr22	36455000	1.24E-02	promoter	super_enhancer	ZDHHC8P1
chr22	23775000	chr22	42915000	1.03E-02	promoter	super_enhancer	AP000344.3

chr22	23975000	chr22	37685000	1.81E-02	promoter	super_enhancer	DRICH1
chr22	24215000	chr22	31655000	2.73E-02	promoter	super_enhancer	KB-1125A3.12
chr22	24215000	chr22	38315000	1.94E-02	promoter	super_enhancer	KB-1125A3.12
chr22	24235000	chr22	24975000	1.09E-02	promoter	super_enhancer	MIF
chr22	24235000	chr22	36415000	1.01E-02	promoter	super_enhancer	MIF
chr22	24235000	chr22	46505000	1.59E-02	promoter	super_enhancer	MIF
chr22	24235000	chr22	49945000	1.22E-02	promoter	super_enhancer	MIF
chr22	24245000	chr22	30225000	3.38E-02	promoter	super_enhancer	AP000350.1
chr22	24245000	chr22	41305000	1.32E-02	promoter	super_enhancer	AP000350.1
chr22	24335000	chr22	37185000	1.26E-02	promoter	super_enhancer	AP000351.4
chr22	24335000	chr22	42765000	1.02E-02	promoter	super_enhancer	AP000351.4
chr22	24405000	chr22	30225000	3.30E-02	promoter	super_enhancer	AP000351.13
chr22	24405000	chr22	43115000	1.94E-02	promoter	super_enhancer	AP000351.13
chr22	24405000	chr22	49955000	1.29E-02	promoter	super_enhancer	AP000351.13
chr22	24645000	chr22	29625000	2.25E-02	promoter	super_enhancer	AP000354.4
chr22	24645000	chr22	41295000	9.55E-03	promoter	super_enhancer	AP000354.4
chr22	24965000	chr22	30475000	4.55E-02	super_enhancer	promoter	HORMAD2
chr22	24975000	chr22	31875000	2.15E-02	super_enhancer	promoter	RNU6-28P
chr22	24975000	chr22	36935000	1.54E-02	super_enhancer	promoter	RP5-1119A7.11
chr22	24975000	chr22	38245000	1.69E-02	super_enhancer	promoter	ANKRD54
chr22	24975000	chr22	41615000	1.51E-02	super_enhancer	promoter	RP4-756G23.5
chr22	24985000	chr22	50985000	1.05E-02	super_enhancer	promoter	CTA-384D8.31
chr22	24995000	chr22	30235000	3.55E-02	super_enhancer	promoter	ASCC2
chr22	24995000	chr22	31365000	2.76E-02	super_enhancer	promoter	MORC2

chr22	24995000	chr22	32595000	2.69E-02	super_enhancer	promoter	IGLCOR22-1
chr22	24995000	chr22	35775000	2.03E-02	super_enhancer	promoter	HMOX1
chr22	24995000	chr22	40785000	1.79E-02	super_enhancer	promoter	RP5-1042K10.10
chr22	25055000	chr22	46755000	1.62E-02	promoter	super_enhancer	POM121L10P
chr22	25595000	chr22	50295000	1.22E-02	promoter	super_enhancer	CRYBB3
chr22	25615000	chr22	31645000	2.66E-02	promoter	super_enhancer	CRYBB2
chr22	25835000	chr22	46525000	1.19E-02	promoter	super_enhancer	IGLVIVOR22-1
chr22	26565000	chr22	45965000	1.55E-02	promoter	super_enhancer	SEZ6L
chr22	26825000	chr22	36435000	1.08E-02	promoter	super_enhancer	ASPHD2
chr22	26825000	chr22	37675000	1.07E-02	promoter	super_enhancer	ASPHD2
chr22	26825000	chr22	46005000	1.01E-02	promoter	super_enhancer	ASPHD2
chr22	26955000	chr22	36465000	1.46E-02	promoter	super_enhancer	HMGB1P10
chr22	26955000	chr22	37745000	1.88E-02	promoter	super_enhancer	HMGB1P10
chr22	27015000	chr22	31655000	3.26E-02	promoter	super_enhancer	CRYBB1
chr22	27285000	chr22	31655000	4.48E-02	promoter	super_enhancer	RP1-40G4P.1
chr22	27415000	chr22	35595000	1.52E-02	promoter	super_enhancer	CTA-992D9.11
chr22	27415000	chr22	42945000	1.54E-02	promoter	super_enhancer	CTA-992D9.11
chr22	27715000	chr22	37205000	2.41E-02	promoter	super_enhancer	CTA-929C8.7
chr22	27715000	chr22	37255000	1.93E-02	promoter	super_enhancer	CTA-929C8.7
chr22	28315000	chr22	46745000	1.24E-02	promoter	super_enhancer	TTC28-AS1
chr22	29195000	chr22	35585000	2.48E-02	promoter	super_enhancer	Z93930.1
chr22	29195000	chr22	36395000	1.61E-02	promoter	super_enhancer	Z93930.1
chr22	29195000	chr22	37305000	2.24E-02	promoter	super_enhancer	Z93930.1
chr22	29195000	chr22	37755000	1.80E-02	promoter	super_enhancer	Z93930.1

chr22	29195000	chr22	38315000	1.82E-02	promoter	super_enhancer	Z93930.1
chr22	29195000	chr22	49975000	1.15E-02	promoter	super_enhancer	Z93930.1
chr22	29615000	chr22	31795000	4.58E-03	super_enhancer	promoter	DRG1
chr22	29615000	chr22	42665000	1.68E-02	super_enhancer	promoter	OGFRP1
chr22	29615000	chr22	50495000	1.23E-02	super_enhancer	promoter	TTLL8
chr22	29625000	chr22	36575000	1.68E-02	super_enhancer	promoter	MTND1P10
chr22	29665000	chr22	39185000	1.77E-02	promoter	super_enhancer	RHBDD3
chr22	29835000	chr22	37315000	2.08E-02	promoter	super_enhancer	RFPL1
chr22	29875000	chr22	37765000	2.62E-02	promoter	super_enhancer	RFPL1S
chr22	29875000	chr22	42955000	1.21E-02	promoter	super_enhancer	RFPL1S
chr22	29875000	chr22	45935000	2.58E-02	promoter	super_enhancer	RFPL1S
chr22	30005000	chr22	37245000	2.90E-02	promoter	super_enhancer	RPEP4
chr22	30005000	chr22	37335000	2.67E-02	promoter	super_enhancer	RPEP4
chr22	30005000	chr22	38225000	2.62E-02	promoter	super_enhancer	RPEP4
chr22	30005000	chr22	39185000	2.46E-02	promoter	super_enhancer	RPEP4
chr22	30115000	chr22	31645000	3.28E-02	promoter	super_enhancer	CABP7
chr22	30115000	chr22	38165000	2.96E-02	promoter	super_enhancer	CABP7
chr22	30115000	chr22	38715000	3.16E-02	promoter	super_enhancer	CABP7
chr22	30205000	chr22	35795000	3.15E-02	super_enhancer	promoter	MCM5
chr22	30205000	chr22	40145000	1.42E-02	super_enhancer	promoter	RP1-172B20.6
chr22	30225000	chr22	31875000	1.76E-02	super_enhancer	promoter	RNU6-28P
chr22	30225000	chr22	33505000	2.25E-03	super_enhancer	promoter	RP1-302D9.2
chr22	30225000	chr22	44535000	2.05E-02	super_enhancer	promoter	RP4-671O14.6
chr22	30235000	chr22	37785000	2.30E-02	promoter	super_enhancer	ASCC2

chr22	30255000	chr22	34605000	2.84E-02	super_enhancer	promoter	LL22NC03-13G6.2
chr22	30255000	chr22	36035000	2.37E-02	super_enhancer	promoter	MB
chr22	30255000	chr22	42475000	2.01E-02	super_enhancer	promoter	SMDT1
chr22	30255000	chr22	50245000	1.61E-02	super_enhancer	promoter	RP11-494O16.4
chr22	30255000	chr22	50765000	1.73E-02	super_enhancer	promoter	DENND6B
chr22	30425000	chr22	37675000	2.41E-02	promoter	super_enhancer	RP3-394A18.1
chr22	30425000	chr22	38695000	3.29E-02	promoter	super_enhancer	RP3-394A18.1
chr22	30425000	chr22	42765000	2.43E-02	promoter	super_enhancer	RP3-394A18.1
chr22	30425000	chr22	42785000	2.61E-02	promoter	super_enhancer	RP3-394A18.1
chr22	30425000	chr22	42965000	2.61E-02	promoter	super_enhancer	RP3-394A18.1
chr22	30425000	chr22	45935000	2.67E-02	promoter	super_enhancer	RP3-394A18.1
chr22	30425000	chr22	49965000	1.77E-02	promoter	super_enhancer	RP3-394A18.1
chr22	30475000	chr22	38715000	3.16E-02	promoter	super_enhancer	HORMAD2
chr22	30635000	chr22	46495000	3.35E-02	promoter	super_enhancer	RP1-102K2.6
chr22	30695000	chr22	37665000	3.31E-02	promoter	super_enhancer	RP1-130H16.18
chr22	30695000	chr22	37765000	2.88E-02	promoter	super_enhancer	RP1-130H16.18
chr22	30695000	chr22	42905000	1.55E-02	promoter	super_enhancer	RP1-130H16.18
chr22	30805000	chr22	35595000	3.85E-02	promoter	super_enhancer	RP4-539M6.19
chr22	30805000	chr22	38315000	3.01E-02	promoter	super_enhancer	RP4-539M6.19
chr22	30805000	chr22	43115000	3.57E-02	promoter	super_enhancer	RP4-539M6.19
chr22	30805000	chr22	44365000	2.74E-02	promoter	super_enhancer	RP4-539M6.19
chr22	30805000	chr22	45925000	2.68E-02	promoter	super_enhancer	RP4-539M6.19
chr22	30825000	chr22	30835000	3.20E-02	super_enhancer	promoter	RP4-539M6.18
chr22	30825000	chr22	40785000	2.75E-02	super_enhancer	promoter	RP5-1042K10.10

chr22	30825000	chr22	48845000	2.01E-02	super_enhancer	promoter	RP11-536P6.3
chr22	30825000	chr22	50965000	2.88E-02	super_enhancer	promoter	SCO2
chr22	30835000	chr22	36455000	3.25E-02	promoter	super_enhancer	RP4-539M6.18
chr22	30835000	chr22	45935000	3.03E-02	promoter	super_enhancer	RP4-539M6.18
chr22	31065000	chr22	36375000	2.08E-02	promoter	super_enhancer	DUSP18
chr22	31065000	chr22	46745000	1.95E-02	promoter	super_enhancer	DUSP18
chr22	31365000	chr22	31635000	2.63E-03	promoter	super_enhancer	MORC2
chr22	31365000	chr22	36385000	1.58E-02	promoter	super_enhancer	MORC2
chr22	31365000	chr22	37205000	3.11E-02	promoter	super_enhancer	MORC2
chr22	31375000	chr22	42945000	1.84E-02	promoter	super_enhancer	RP3-430N8.11
chr22	31375000	chr22	45905000	1.57E-02	promoter	super_enhancer	RP3-430N8.11
chr22	31455000	chr22	37765000	2.50E-02	promoter	super_enhancer	RN7SL633P
chr22	31455000	chr22	38165000	2.39E-02	promoter	super_enhancer	RN7SL633P
chr22	31555000	chr22	31655000	1.07E-02	promoter	super_enhancer	RNF185
chr22	31555000	chr22	36425000	2.22E-02	promoter	super_enhancer	RNF185
chr22	31635000	chr22	36925000	2.76E-02	super_enhancer	promoter	EIF3D
chr22	31645000	chr22	50495000	1.80E-02	super_enhancer	promoter	TTLL8
chr22	31655000	chr22	39715000	2.12E-02	super_enhancer	promoter	AL022326.1
chr22	31655000	chr22	39745000	1.97E-02	super_enhancer	promoter	SYNGR1
chr22	31655000	chr22	39775000	2.03E-02	super_enhancer	promoter	RP3-333H23.9
chr22	31655000	chr22	41185000	1.79E-02	super_enhancer	promoter	RP3-408N23.4
chr22	31655000	chr22	42105000	2.53E-02	super_enhancer	promoter	HMGN2P10
chr22	31655000	chr22	45705000	1.80E-02	super_enhancer	promoter	FAM118A
chr22	31785000	chr22	37205000	4.18E-02	promoter	super_enhancer	RNU6-338P

chr22	31785000	chr22	37675000	2.54E-02	promoter	super_enhancer	RNU6-338P
chr22	31785000	chr22	45915000	2.30E-02	promoter	super_enhancer	RNU6-338P
chr22	31795000	chr22	37335000	3.48E-02	promoter	super_enhancer	DRG1
chr22	31795000	chr22	37685000	4.07E-02	promoter	super_enhancer	DRG1
chr22	31795000	chr22	46515000	2.20E-02	promoter	super_enhancer	DRG1
chr22	31875000	chr22	37235000	2.96E-02	promoter	super_enhancer	RNU6-28P
chr22	31875000	chr22	37345000	3.22E-02	promoter	super_enhancer	RNU6-28P
chr22	31885000	chr22	37235000	3.07E-02	promoter	super_enhancer	SFI1
chr22	31885000	chr22	37745000	6.08E-04	promoter	super_enhancer	SFI1
chr22	31935000	chr22	37175000	2.87E-02	promoter	super_enhancer	RP11-247I13.6
chr22	31955000	chr22	36365000	2.58E-02	promoter	super_enhancer	RP11-247I13.3
chr22	31955000	chr22	36465000	3.32E-02	promoter	super_enhancer	RP11-247I13.3
chr22	31955000	chr22	42955000	1.15E-02	promoter	super_enhancer	RP11-247I13.3
chr22	31955000	chr22	45935000	2.45E-02	promoter	super_enhancer	RP11-247I13.3
chr22	32435000	chr22	37665000	3.88E-02	promoter	super_enhancer	SC22CB-1E7.1
chr22	32525000	chr22	38715000	3.19E-02	promoter	super_enhancer	RP1-127L4.10
chr22	32525000	chr22	46525000	1.81E-02	promoter	super_enhancer	RP1-127L4.10
chr22	32555000	chr22	36375000	1.91E-02	promoter	super_enhancer	C22orf42
chr22	32555000	chr22	43075000	1.70E-02	promoter	super_enhancer	C22orf42
chr22	32555000	chr22	49965000	1.01E-02	promoter	super_enhancer	C22orf42
chr22	32595000	chr22	45915000	2.20E-02	promoter	super_enhancer	IGLCOR22-1
chr22	32595000	chr22	45975000	2.47E-02	promoter	super_enhancer	IGLCOR22-1
chr22	32665000	chr22	37255000	4.24E-02	promoter	super_enhancer	CPSF1P1
chr22	32665000	chr22	42785000	2.63E-02	promoter	super_enhancer	CPSF1P1

chr22	33455000	chr22	46515000	2.09E-02	promoter	super_enhancer	SYN3
chr22	33505000	chr22	37765000	4.06E-02	promoter	super_enhancer	RP1-302D9.2
chr22	33505000	chr22	42935000	2.37E-02	promoter	super_enhancer	RP1-302D9.2
chr22	33505000	chr22	46065000	1.88E-02	promoter	super_enhancer	RP1-302D9.2
chr22	35605000	chr22	48885000	2.14E-02	super_enhancer	promoter	FAM19A5
chr22	35645000	chr22	37305000	1.51E-02	promoter	super_enhancer	RNU7-167P
chr22	35645000	chr22	46745000	2.33E-02	promoter	super_enhancer	RNU7-167P
chr22	35645000	chr22	49945000	1.84E-02	promoter	super_enhancer	RNU7-167P
chr22	35775000	chr22	36375000	6.08E-03	promoter	super_enhancer	HMOX1
chr22	35795000	chr22	37245000	3.07E-02	promoter	super_enhancer	MCM5
chr22	35795000	chr22	41295000	3.81E-02	promoter	super_enhancer	MCM5
chr22	35795000	chr22	42945000	3.40E-02	promoter	super_enhancer	MCM5
chr22	35795000	chr22	46085000	2.15E-02	promoter	super_enhancer	MCM5
chr22	35795000	chr22	50305000	2.32E-02	promoter	super_enhancer	MCM5
chr22	36035000	chr22	42745000	2.86E-02	promoter	super_enhancer	MB
chr22	36035000	chr22	42755000	3.60E-02	promoter	super_enhancer	MB
chr22	36035000	chr22	45905000	1.88E-02	promoter	super_enhancer	MB
chr22	36035000	chr22	49955000	1.80E-02	promoter	super_enhancer	MB
chr22	36295000	chr22	36465000	3.19E-04	promoter	super_enhancer	NDUFA9P1
chr22	36295000	chr22	49945000	1.27E-02	promoter	super_enhancer	NDUFA9P1
chr22	36365000	chr22	37405000	1.96E-03	super_enhancer	promoter	TEX33
chr22	36365000	chr22	45575000	1.65E-02	super_enhancer	promoter	CTA-268H5.12
chr22	36365000	chr22	46545000	1.60E-02	super_enhancer	promoter	PPARA
chr22	36375000	chr22	36575000	2.88E-02	super_enhancer	promoter	MTND1P10

chr22	36375000	chr22	50765000	1.51E-02	super_enhancer	promoter	DENND6B
chr22	36385000	chr22	38245000	4.31E-03	super_enhancer	promoter	ANKRD54
chr22	36385000	chr22	50495000	1.14E-02	super_enhancer	promoter	TTLL8
chr22	36395000	chr22	36535000	2.84E-02	super_enhancer	promoter	CTA-212A2.1
chr22	36395000	chr22	42095000	3.48E-02	super_enhancer	promoter	MEI1
chr22	36435000	chr22	39915000	4.14E-02	super_enhancer	promoter	ATF4
chr22	36435000	chr22	43795000	2.63E-02	super_enhancer	promoter	RP4-754E20__A.5
chr22	36435000	chr22	45705000	1.96E-02	super_enhancer	promoter	FAM118A
chr22	36465000	chr22	41865000	3.15E-02	super_enhancer	promoter	PHF5A
chr22	36465000	chr22	42475000	2.98E-02	super_enhancer	promoter	SMDT1
chr22	36575000	chr22	42915000	1.66E-02	promoter	super_enhancer	MTND1P10
chr22	36575000	chr22	46495000	1.86E-02	promoter	super_enhancer	MTND1P10
chr22	36575000	chr22	46505000	1.88E-02	promoter	super_enhancer	MTND1P10
chr22	36835000	chr22	42765000	2.49E-02	promoter	super_enhancer	BX470187.1
chr22	36925000	chr22	37335000	2.31E-04	promoter	super_enhancer	EIF3D
chr22	36935000	chr22	37165000	1.63E-02	promoter	super_enhancer	RP5-1119A7.11
chr22	36935000	chr22	37245000	3.86E-02	promoter	super_enhancer	RP5-1119A7.11
chr22	36935000	chr22	37255000	3.10E-02	promoter	super_enhancer	RP5-1119A7.11
chr22	36935000	chr22	37305000	6.16E-03	promoter	super_enhancer	RP5-1119A7.11
chr22	36935000	chr22	37685000	2.92E-02	promoter	super_enhancer	RP5-1119A7.11
chr22	36935000	chr22	50295000	2.02E-02	promoter	super_enhancer	RP5-1119A7.11
chr22	37165000	chr22	50765000	3.44E-02	super_enhancer	promoter	DENND6B
chr22	37185000	chr22	37475000	4.89E-03	super_enhancer	promoter	RP5-1170K4.7
chr22	37185000	chr22	37825000	2.51E-02	super_enhancer	promoter	ELFN2

chr22	37185000	chr22	42665000	4.70E-02	super_enhancer	promoter	OGFRP1
chr22	37185000	chr22	44625000	2.73E-02	super_enhancer	promoter	RP4-671O14.7
chr22	37185000	chr22	50245000	2.68E-02	super_enhancer	promoter	RP11-494O16.4
chr22	37195000	chr22	37475000	1.27E-02	super_enhancer	promoter	RP5-1170K4.7
chr22	37205000	chr22	44625000	3.01E-02	super_enhancer	promoter	RP4-671O14.7
chr22	37235000	chr22	37405000	8.44E-03	super_enhancer	promoter	TEX33
chr22	37245000	chr22	37405000	5.69E-03	super_enhancer	promoter	TEX33
chr22	37245000	chr22	40985000	1.22E-03	super_enhancer	promoter	RPL4P6
chr22	37245000	chr22	42475000	4.44E-02	super_enhancer	promoter	SMDT1
chr22	37245000	chr22	46285000	1.84E-02	super_enhancer	promoter	WI2-85898F10.1
chr22	37255000	chr22	43255000	3.45E-02	super_enhancer	promoter	ARFGAP3
chr22	37255000	chr22	51195000	1.17E-02	super_enhancer	promoter	AC002056.3
chr22	37305000	chr22	37415000	1.74E-02	super_enhancer	promoter	MPST
chr22	37305000	chr22	43255000	3.74E-02	super_enhancer	promoter	ARFGAP3
chr22	37315000	chr22	37405000	3.92E-02	super_enhancer	promoter	TEX33
chr22	37315000	chr22	37475000	3.21E-02	super_enhancer	promoter	RP5-1170K4.7
chr22	37325000	chr22	37595000	4.80E-02	super_enhancer	promoter	C1QTNF6
chr22	37325000	chr22	38385000	3.50E-02	super_enhancer	promoter	MIR4534
chr22	37325000	chr22	50985000	2.57E-02	super_enhancer	promoter	CTA-384D8.31
chr22	37335000	chr22	37595000	3.31E-02	super_enhancer	promoter	C1QTNF6
chr22	37345000	chr22	41185000	4.85E-02	super_enhancer	promoter	RP3-408N23.4
chr22	37405000	chr22	37685000	3.01E-02	promoter	super_enhancer	TEX33
chr22	37475000	chr22	37775000	2.32E-02	promoter	super_enhancer	RP5-1170K4.7
chr22	37475000	chr22	46005000	2.70E-02	promoter	super_enhancer	RP5-1170K4.7

chr22	37595000	chr22	45935000	3.06E-02	promoter	super_enhancer	C1QTNF6
chr22	37665000	chr22	37825000	3.01E-02	super_enhancer	promoter	ELFN2
chr22	37665000	chr22	44535000	3.24E-02	super_enhancer	promoter	RP4-671O14.6
chr22	37685000	chr22	37825000	5.05E-03	super_enhancer	promoter	ELFN2
chr22	37685000	chr22	37945000	3.24E-02	super_enhancer	promoter	RP5-1177I5.3
chr22	37685000	chr22	45845000	4.37E-02	super_enhancer	promoter	RP1-102D24.5
chr22	37745000	chr22	37825000	1.67E-02	super_enhancer	promoter	ELFN2
chr22	37745000	chr22	43255000	4.18E-02	super_enhancer	promoter	ARFGAP3
chr22	37755000	chr22	37825000	2.83E-02	super_enhancer	promoter	ELFN2
chr22	37755000	chr22	44535000	2.71E-02	super_enhancer	promoter	RP4-671O14.6
chr22	37755000	chr22	48255000	1.80E-02	super_enhancer	promoter	RP13-455A7.1
chr22	37755000	chr22	50965000	2.51E-02	super_enhancer	promoter	SCO2
chr22	37765000	chr22	50765000	2.53E-02	super_enhancer	promoter	DENND6B
chr22	37785000	chr22	51195000	1.34E-02	super_enhancer	promoter	AC002056.3
chr22	37825000	chr22	46005000	2.21E-02	promoter	super_enhancer	ELFN2
chr22	37825000	chr22	46775000	2.28E-02	promoter	super_enhancer	ELFN2
chr22	37825000	chr22	49965000	1.83E-02	promoter	super_enhancer	ELFN2
chr22	37945000	chr22	44335000	3.74E-02	promoter	super_enhancer	RP5-1177I5.3
chr22	38055000	chr22	38175000	4.94E-02	promoter	super_enhancer	Z83844.1
chr22	38165000	chr22	50495000	2.28E-02	super_enhancer	promoter	TTLL8
chr22	38165000	chr22	50925000	2.15E-02	super_enhancer	promoter	MIOX
chr22	38245000	chr22	42905000	3.50E-02	promoter	super_enhancer	ANKRD54
chr22	38245000	chr22	45975000	4.40E-02	promoter	super_enhancer	ANKRD54
chr22	38245000	chr22	49945000	2.59E-02	promoter	super_enhancer	ANKRD54

chr22	38385000	chr22	44345000	2.33E-02	promoter	super_enhancer	MIR4534
chr22	38485000	chr22	38695000	1.61E-02	promoter	super_enhancer	CTA-228A9.3
chr22	38695000	chr22	45575000	4.25E-02	super_enhancer	promoter	CTA-268H5.12
chr22	38695000	chr22	47135000	3.74E-02	super_enhancer	promoter	CERK
chr22	38695000	chr22	50525000	3.55E-02	super_enhancer	promoter	MLC1
chr22	38705000	chr22	39135000	2.86E-02	super_enhancer	promoter	RP3-508I15.14
chr22	38705000	chr22	39775000	4.35E-02	super_enhancer	promoter	RP3-333H23.9
chr22	38705000	chr22	51035000	2.36E-02	super_enhancer	promoter	CTA-384D8.33
chr22	38715000	chr22	50645000	2.82E-02	super_enhancer	promoter	RP3-402G11.27
chr22	38905000	chr22	46755000	3.74E-02	promoter	super_enhancer	DDX17
chr22	39065000	chr22	46525000	2.90E-02	promoter	super_enhancer	RP3-508I15.10
chr22	39135000	chr22	43125000	2.97E-03	promoter	super_enhancer	RP3-508I15.14
chr22	39135000	chr22	46505000	4.37E-02	promoter	super_enhancer	RP3-508I15.14
chr22	39175000	chr22	50965000	2.99E-02	super_enhancer	promoter	SCO2
chr22	39395000	chr22	44345000	3.98E-02	promoter	super_enhancer	APOBEC3B-AS1
chr22	39745000	chr22	46745000	3.24E-02	promoter	super_enhancer	SYNGR1
chr22	39915000	chr22	44335000	4.82E-02	promoter	super_enhancer	ATF4
chr22	39915000	chr22	46005000	3.13E-02	promoter	super_enhancer	ATF4
chr22	40145000	chr22	45915000	3.68E-02	promoter	super_enhancer	RP1-172B20.6
chr22	40785000	chr22	50295000	2.35E-02	promoter	super_enhancer	RP5-1042K10.10
chr22	40925000	chr22	45945000	3.84E-02	promoter	super_enhancer	AL031594.1
chr22	41075000	chr22	46765000	4.06E-02	promoter	super_enhancer	MCHR1
chr22	41185000	chr22	49935000	2.58E-02	promoter	super_enhancer	RP3-408N23.4
chr22	41215000	chr22	46495000	3.20E-02	promoter	super_enhancer	SLC25A17

chr22	42085000	chr22	42785000	1.45E-02	promoter	super_enhancer	C22orf46
chr22	42095000	chr22	42785000	6.63E-03	promoter	super_enhancer	MEI1
chr22	42105000	chr22	42915000	2.32E-02	promoter	super_enhancer	HMGN2P10
chr22	42395000	chr22	42785000	2.14E-02	promoter	super_enhancer	WBP2NL
chr22	42395000	chr22	49945000	3.59E-02	promoter	super_enhancer	WBP2NL
chr22	42505000	chr22	42905000	7.21E-03	promoter	super_enhancer	OLA1P1
chr22	42665000	chr22	42765000	2.58E-02	promoter	super_enhancer	OGFRP1
chr22	42735000	chr22	48845000	3.65E-02	super_enhancer	promoter	RP11-536P6.3
chr22	42745000	chr22	50985000	3.95E-02	super_enhancer	promoter	CTA-384D8.31
chr22	42755000	chr22	50985000	4.96E-02	super_enhancer	promoter	CTA-384D8.31
chr22	42775000	chr22	47655000	4.57E-02	super_enhancer	promoter	RP1-111J24.1
chr22	42785000	chr22	50525000	4.63E-02	super_enhancer	promoter	MLC1
chr22	42925000	chr22	50985000	3.87E-02	super_enhancer	promoter	CTA-384D8.31
chr22	42925000	chr22	51195000	1.98E-02	super_enhancer	promoter	AC002056.3
chr22	42945000	chr22	50965000	9.45E-04	super_enhancer	promoter	SCO2
chr22	42955000	chr22	50495000	1.92E-02	super_enhancer	promoter	TTLL8
chr22	42965000	chr22	50765000	4.50E-02	super_enhancer	promoter	DENND6B
chr22	42965000	chr22	50965000	4.73E-02	super_enhancer	promoter	SCO2
chr22	43075000	chr22	50645000	4.82E-02	super_enhancer	promoter	RP3-402G11.27
chr22	43085000	chr22	50965000	4.81E-02	super_enhancer	promoter	SCO2
chr22	43095000	chr22	50765000	4.30E-02	super_enhancer	promoter	DENND6B
chr22	43095000	chr22	50925000	3.50E-02	super_enhancer	promoter	MIOX
chr22	43095000	chr22	50965000	4.51E-02	super_enhancer	promoter	SCO2
chr22	43115000	chr22	43435000	3.89E-02	super_enhancer	promoter	AL022476.2

chr22	43125000	chr22	50245000	4.40E-02	super_enhancer	promoter	RP11-494O16.4
chr22	44335000	chr22	51035000	4.54E-02	super_enhancer	promoter	CTA-384D8.33
chr22	44335000	chr22	51195000	2.29E-02	super_enhancer	promoter	AC002056.3
chr22	44355000	chr22	50245000	4.87E-02	super_enhancer	promoter	RP11-494O16.4
chr22	44535000	chr22	45925000	4.87E-02	promoter	super_enhancer	RP4-671O14.6
chr22	44535000	chr22	49945000	4.44E-02	promoter	super_enhancer	RP4-671O14.6
chr22	45845000	chr22	45925000	2.86E-02	promoter	super_enhancer	RP1-102D24.5
chr22	45845000	chr22	46065000	1.85E-02	promoter	super_enhancer	RP1-102D24.5
chr22	45965000	chr22	50525000	4.36E-03	super_enhancer	promoter	MLC1
chr22	46155000	chr22	49965000	3.98E-02	promoter	super_enhancer	MIR4762
chr22	46285000	chr22	46525000	4.15E-02	promoter	super_enhancer	WI2-85898F10.1
chr22	46455000	chr22	46495000	2.57E-02	promoter	super_enhancer	RP6-109B7.2
chr22	46495000	chr22	47135000	3.10E-02	super_enhancer	promoter	CERK
chr22	46495000	chr22	50525000	4.92E-03	super_enhancer	promoter	MLC1
chr22	46545000	chr22	46775000	3.23E-02	promoter	super_enhancer	PPARA
chr22	46755000	chr22	50965000	5.79E-03	super_enhancer	promoter	SCO2
chr22	48445000	chr22	49965000	2.76E-04	promoter	super_enhancer	CTA-280A3__B.2
chr22	50305000	chr22	50645000	1.48E-02	super_enhancer	promoter	RP3-402G11.27

Supplementary Data 1 Hi-ChIP analysis in S18 and S26-DNP cells

Hi-ChIP analysis in S26-DNP cells

loopChr1	loopMidSite1	loopChr2	loopMidSite2	p-value	loopAnnotType1	loopAnnotType2	targetGene
chr1	565000	chr1	183155000	2.77E-03	promoter	super_enhancer	RP5-857K21.5
chr1	895000	chr1	21635000	1.15E-02	promoter	super_enhancer	NOC2L

chr1	895000	chr1	43865000	5.11E-03	promoter	super_enhancer	NOC2L
chr1	895000	chr1	46185000	3.57E-03	promoter	super_enhancer	NOC2L
chr1	895000	chr1	233675000	2.58E-03	promoter	super_enhancer	NOC2L
chr1	945000	chr1	1005000	1.25E-02	promoter	super_enhancer	RP11-54O7.10
chr1	945000	chr1	151195000	3.15E-03	promoter	super_enhancer	RP11-54O7.10
chr1	945000	chr1	205295000	3.51E-03	promoter	super_enhancer	RP11-54O7.10
chr1	945000	chr1	235045000	2.71E-03	promoter	super_enhancer	RP11-54O7.10
chr1	955000	chr1	6275000	3.97E-02	promoter	super_enhancer	AGRN
chr1	955000	chr1	16125000	1.46E-02	promoter	super_enhancer	AGRN
chr1	955000	chr1	41865000	7.57E-03	promoter	super_enhancer	AGRN
chr1	955000	chr1	42935000	6.22E-03	promoter	super_enhancer	AGRN
chr1	955000	chr1	183185000	2.60E-03	promoter	super_enhancer	AGRN
chr1	995000	chr1	6255000	2.87E-02	promoter	super_enhancer	RP11-465B22.3
chr1	995000	chr1	235015000	2.94E-03	promoter	super_enhancer	RP11-465B22.3
chr1	1005000	chr1	1215000	5.92E-03	super_enhancer	promoter	SCNN1D
chr1	1005000	chr1	9715000	1.57E-02	super_enhancer	promoter	PIK3CD-AS1
chr1	1005000	chr1	18435000	1.17E-02	super_enhancer	promoter	RP11-174G17.3
chr1	1005000	chr1	20915000	9.27E-03	super_enhancer	promoter	CDA
chr1	1005000	chr1	241295000	1.44E-03	super_enhancer	promoter	MIR3123
chr1	1025000	chr1	23045000	6.21E-03	super_enhancer	promoter	MIR4684
chr1	1025000	chr1	28145000	4.10E-03	super_enhancer	promoter	RP3-426I6.6
chr1	1025000	chr1	40915000	3.58E-03	super_enhancer	promoter	ZFP69B
chr1	1105000	chr1	6305000	3.29E-02	promoter	super_enhancer	RP11-465B22.8
chr1	1105000	chr1	12555000	1.47E-02	promoter	super_enhancer	RP11-465B22.8

chr1	1105000	chr1	16195000	1.02E-02	promoter	super_enhancer	RP11-465B22.8
chr1	1105000	chr1	155315000	2.20E-03	promoter	super_enhancer	RP11-465B22.8
chr1	1115000	chr1	6255000	3.67E-02	promoter	super_enhancer	TTLL10-AS1
chr1	1115000	chr1	6325000	2.87E-02	promoter	super_enhancer	TTLL10-AS1
chr1	1115000	chr1	32725000	8.23E-03	promoter	super_enhancer	TTLL10-AS1
chr1	1115000	chr1	40875000	4.62E-03	promoter	super_enhancer	TTLL10-AS1
chr1	1115000	chr1	43855000	7.51E-03	promoter	super_enhancer	TTLL10-AS1
chr1	1115000	chr1	181095000	2.74E-03	promoter	super_enhancer	TTLL10-AS1
chr1	1215000	chr1	224275000	2.53E-03	promoter	super_enhancer	SCNN1D
chr1	1245000	chr1	32995000	7.48E-03	promoter	super_enhancer	ACAP3
chr1	1245000	chr1	200325000	3.03E-03	promoter	super_enhancer	ACAP3
chr1	1245000	chr1	228165000	1.57E-03	promoter	super_enhancer	ACAP3
chr1	1255000	chr1	7535000	1.77E-02	promoter	super_enhancer	RP5-890O3.9
chr1	1275000	chr1	203515000	2.91E-03	promoter	super_enhancer	MIR6808
chr1	1275000	chr1	235075000	3.39E-03	promoter	super_enhancer	MIR6808
chr1	1285000	chr1	16125000	6.88E-03	promoter	super_enhancer	DVL1
chr1	1285000	chr1	41855000	2.83E-03	promoter	super_enhancer	DVL1
chr1	1285000	chr1	94695000	7.19E-04	promoter	super_enhancer	DVL1
chr1	1335000	chr1	6275000	3.92E-02	promoter	super_enhancer	CCNL2
chr1	1355000	chr1	6295000	3.13E-02	promoter	super_enhancer	RP4-758J18.7
chr1	1355000	chr1	43915000	5.59E-03	promoter	super_enhancer	RP4-758J18.7
chr1	1355000	chr1	43925000	4.61E-03	promoter	super_enhancer	RP4-758J18.7
chr1	1355000	chr1	94795000	1.55E-03	promoter	super_enhancer	RP4-758J18.7
chr1	1385000	chr1	11835000	1.90E-02	promoter	super_enhancer	ATAD3C

chr1	1385000	chr1	15415000	1.37E-02	promoter	super_enhancer	ATAD3C
chr1	1385000	chr1	27495000	9.14E-03	promoter	super_enhancer	ATAD3C
chr1	1385000	chr1	200375000	1.99E-03	promoter	super_enhancer	ATAD3C
chr1	1515000	chr1	15355000	1.78E-02	promoter	super_enhancer	AL645728.1
chr1	1515000	chr1	16195000	1.41E-02	promoter	super_enhancer	AL645728.1
chr1	1515000	chr1	26355000	1.07E-02	promoter	super_enhancer	AL645728.1
chr1	1515000	chr1	43475000	6.26E-03	promoter	super_enhancer	AL645728.1
chr1	1515000	chr1	234615000	3.24E-03	promoter	super_enhancer	AL645728.1
chr1	1535000	chr1	6295000	3.13E-02	promoter	super_enhancer	C1orf233
chr1	1535000	chr1	8885000	2.23E-02	promoter	super_enhancer	C1orf233
chr1	1535000	chr1	9315000	2.05E-02	promoter	super_enhancer	C1orf233
chr1	1535000	chr1	46185000	3.12E-03	promoter	super_enhancer	C1orf233
chr1	1535000	chr1	164745000	1.66E-03	promoter	super_enhancer	C1orf233
chr1	1605000	chr1	6265000	2.56E-02	promoter	super_enhancer	RP11-345P4.6
chr1	1605000	chr1	6345000	2.14E-02	promoter	super_enhancer	RP11-345P4.6
chr1	1605000	chr1	12605000	1.30E-02	promoter	super_enhancer	RP11-345P4.6
chr1	1655000	chr1	26855000	5.80E-03	promoter	super_enhancer	CDK11B
chr1	1655000	chr1	203555000	2.06E-03	promoter	super_enhancer	CDK11B
chr1	1845000	chr1	8875000	2.08E-02	promoter	super_enhancer	CALML6
chr1	1845000	chr1	12545000	1.52E-02	promoter	super_enhancer	CALML6
chr1	1845000	chr1	12585000	1.99E-02	promoter	super_enhancer	CALML6
chr1	1845000	chr1	152015000	2.71E-03	promoter	super_enhancer	CALML6
chr1	1845000	chr1	214475000	2.20E-03	promoter	super_enhancer	CALML6
chr1	1935000	chr1	43905000	7.28E-03	promoter	super_enhancer	CFAP74

chr1	2145000	chr1	27515000	7.42E-03	promoter	super_enhancer	FAAP20
chr1	2145000	chr1	32985000	7.82E-03	promoter	super_enhancer	FAAP20
chr1	2145000	chr1	43865000	5.24E-03	promoter	super_enhancer	FAAP20
chr1	2145000	chr1	221965000	2.61E-03	promoter	super_enhancer	FAAP20
chr1	2145000	chr1	226835000	2.74E-03	promoter	super_enhancer	FAAP20
chr1	2155000	chr1	6275000	4.75E-02	promoter	super_enhancer	AL590822.1
chr1	2155000	chr1	6325000	3.31E-02	promoter	super_enhancer	AL590822.1
chr1	2155000	chr1	27515000	7.64E-03	promoter	super_enhancer	AL590822.1
chr1	2155000	chr1	27535000	7.61E-03	promoter	super_enhancer	AL590822.1
chr1	2155000	chr1	32735000	7.27E-03	promoter	super_enhancer	AL590822.1
chr1	2155000	chr1	42925000	5.95E-03	promoter	super_enhancer	AL590822.1
chr1	2285000	chr1	6255000	3.98E-02	promoter	super_enhancer	RP4-740C4.5
chr1	2285000	chr1	20595000	1.05E-02	promoter	super_enhancer	RP4-740C4.5
chr1	2285000	chr1	41895000	5.91E-03	promoter	super_enhancer	RP4-740C4.5
chr1	2285000	chr1	42965000	3.22E-03	promoter	super_enhancer	RP4-740C4.5
chr1	2285000	chr1	94645000	1.88E-03	promoter	super_enhancer	RP4-740C4.5
chr1	2295000	chr1	3455000	2.74E-02	promoter	super_enhancer	RP4-740C4.7
chr1	2295000	chr1	26865000	8.70E-03	promoter	super_enhancer	RP4-740C4.7
chr1	2295000	chr1	32735000	6.57E-03	promoter	super_enhancer	RP4-740C4.7
chr1	2345000	chr1	6265000	3.98E-02	promoter	super_enhancer	PEX10
chr1	2345000	chr1	6335000	3.41E-02	promoter	super_enhancer	PEX10
chr1	2345000	chr1	6345000	3.30E-02	promoter	super_enhancer	PEX10
chr1	2345000	chr1	42955000	4.26E-03	promoter	super_enhancer	PEX10
chr1	2345000	chr1	152045000	2.39E-03	promoter	super_enhancer	PEX10

chr1	2345000	chr1	204125000	2.69E-03	promoter	super_enhancer	PEX10
chr1	2345000	chr1	207305000	2.11E-03	promoter	super_enhancer	PEX10
chr1	2425000	chr1	9285000	2.19E-02	promoter	super_enhancer	RP3-395M20.2
chr1	2425000	chr1	9315000	2.54E-02	promoter	super_enhancer	RP3-395M20.2
chr1	2425000	chr1	11795000	2.13E-02	promoter	super_enhancer	RP3-395M20.2
chr1	2565000	chr1	6255000	4.43E-02	promoter	super_enhancer	MMEL1
chr1	2565000	chr1	7535000	2.06E-02	promoter	super_enhancer	MMEL1
chr1	2565000	chr1	16155000	1.39E-02	promoter	super_enhancer	MMEL1
chr1	2565000	chr1	114435000	1.71E-03	promoter	super_enhancer	MMEL1
chr1	2565000	chr1	233125000	2.16E-03	promoter	super_enhancer	MMEL1
chr1	2985000	chr1	6325000	4.61E-02	promoter	super_enhancer	LINC00982
chr1	2985000	chr1	27485000	1.04E-02	promoter	super_enhancer	LINC00982
chr1	2985000	chr1	27515000	8.19E-03	promoter	super_enhancer	LINC00982
chr1	3455000	chr1	7755000	4.49E-02	super_enhancer	promoter	RP11-338N10.1
chr1	3455000	chr1	8485000	2.06E-02	super_enhancer	promoter	RP5-1115A15.1
chr1	3455000	chr1	9565000	2.97E-02	super_enhancer	promoter	RP13-392I16.1
chr1	3455000	chr1	16345000	1.85E-02	super_enhancer	promoter	CLCNKA
chr1	3455000	chr1	17305000	1.21E-02	super_enhancer	promoter	RP1-37C10.3
chr1	3455000	chr1	35525000	5.04E-03	super_enhancer	promoter	ZMYM1
chr1	3455000	chr1	151345000	2.75E-03	super_enhancer	promoter	SELENBP1
chr1	3455000	chr1	204655000	2.78E-03	super_enhancer	promoter	LRRN2
chr1	3465000	chr1	6685000	4.80E-02	super_enhancer	promoter	THAP3
chr1	3465000	chr1	16485000	1.79E-02	super_enhancer	promoter	RP11-276H7.3
chr1	3465000	chr1	20505000	7.67E-03	super_enhancer	promoter	PLA2G2C

chr1	3465000	chr1	21115000	9.46E-03	super_enhancer	promoter	HP1BP3
chr1	3465000	chr1	22235000	6.88E-03	super_enhancer	promoter	RP11-26H16.1
chr1	3465000	chr1	36025000	6.59E-03	super_enhancer	promoter	KIAA0319L
chr1	3465000	chr1	53835000	4.10E-03	super_enhancer	promoter	RP11-117D22.2
chr1	3465000	chr1	172115000	1.48E-03	super_enhancer	promoter	DNM3OS
chr1	3465000	chr1	210405000	1.61E-03	super_enhancer	promoter	SERTAD4
chr1	3465000	chr1	224035000	1.75E-03	super_enhancer	promoter	TP53BP2
chr1	3585000	chr1	8095000	4.28E-02	promoter	super_enhancer	RP5-1092A11.5
chr1	3585000	chr1	11805000	2.00E-02	promoter	super_enhancer	RP5-1092A11.5
chr1	3585000	chr1	27495000	1.03E-02	promoter	super_enhancer	RP5-1092A11.5
chr1	3585000	chr1	230095000	1.56E-03	promoter	super_enhancer	RP5-1092A11.5
chr1	3775000	chr1	12565000	1.75E-02	promoter	super_enhancer	CEP104
chr1	3775000	chr1	16145000	1.57E-02	promoter	super_enhancer	CEP104
chr1	3775000	chr1	230125000	1.73E-03	promoter	super_enhancer	CEP104
chr1	4715000	chr1	6275000	2.07E-02	promoter	super_enhancer	AJAP1
chr1	4715000	chr1	155305000	2.54E-03	promoter	super_enhancer	AJAP1
chr1	4715000	chr1	234995000	3.29E-03	promoter	super_enhancer	AJAP1
chr1	5955000	chr1	12605000	2.52E-02	promoter	super_enhancer	AL356261.1
chr1	5955000	chr1	40875000	5.06E-03	promoter	super_enhancer	AL356261.1
chr1	5955000	chr1	183145000	2.65E-03	promoter	super_enhancer	AL356261.1
chr1	5955000	chr1	200435000	2.44E-03	promoter	super_enhancer	AL356261.1
chr1	6255000	chr1	26645000	1.22E-02	super_enhancer	promoter	UBXN11
chr1	6255000	chr1	160255000	1.97E-03	super_enhancer	promoter	RP11-574F21.3
chr1	6255000	chr1	224485000	2.58E-03	super_enhancer	promoter	RP11-365O16.5

chr1	6255000	chr1	226625000	2.77E-03	super_enhancer	promoter	RP11-118H4.1
chr1	6265000	chr1	36185000	6.71E-03	super_enhancer	promoter	C1orf216
chr1	6265000	chr1	39425000	6.31E-03	super_enhancer	promoter	EIF1P2
chr1	6265000	chr1	54305000	4.46E-03	super_enhancer	promoter	NDC1
chr1	6275000	chr1	6685000	1.58E-02	super_enhancer	promoter	THAP3
chr1	6275000	chr1	16765000	2.20E-02	super_enhancer	promoter	SPATA21
chr1	6275000	chr1	45965000	8.44E-03	super_enhancer	promoter	MMACHC
chr1	6275000	chr1	90095000	2.86E-03	super_enhancer	promoter	RP11-413E1.4
chr1	6275000	chr1	92695000	1.99E-03	super_enhancer	promoter	ACTBP12
chr1	6275000	chr1	149285000	2.59E-03	super_enhancer	promoter	RP11-403I13.7
chr1	6275000	chr1	224625000	2.95E-03	super_enhancer	promoter	WDR26
chr1	6285000	chr1	6485000	3.97E-02	super_enhancer	promoter	HES2
chr1	6285000	chr1	15985000	1.39E-02	super_enhancer	promoter	RSC1A1
chr1	6285000	chr1	32255000	7.69E-03	super_enhancer	promoter	RP11-84A19.3
chr1	6285000	chr1	35225000	6.07E-03	super_enhancer	promoter	GJB4
chr1	6285000	chr1	45965000	7.32E-03	super_enhancer	promoter	MMACHC
chr1	6285000	chr1	58515000	3.47E-03	super_enhancer	promoter	HNRNPA1P6
chr1	6295000	chr1	11865000	2.92E-02	super_enhancer	promoter	CLCN6
chr1	6295000	chr1	11905000	3.58E-02	super_enhancer	promoter	NPPA-AS1_2
chr1	6295000	chr1	16295000	1.84E-02	super_enhancer	promoter	RP11-5P18.1
chr1	6295000	chr1	22385000	1.39E-02	super_enhancer	promoter	CDC42-IT1
chr1	6295000	chr1	46015000	7.87E-03	super_enhancer	promoter	AKR1A1
chr1	6295000	chr1	153645000	2.37E-03	super_enhancer	promoter	ILF2
chr1	6305000	chr1	6455000	2.13E-03	super_enhancer	promoter	ACOT7

chr1	6305000	chr1	6485000	1.02E-02	super_enhancer	promoter	HES2
chr1	6305000	chr1	28835000	1.12E-02	super_enhancer	promoter	SNORA73B
chr1	6305000	chr1	160865000	2.32E-03	super_enhancer	promoter	RP11-312J18.5
chr1	6305000	chr1	182925000	3.44E-03	super_enhancer	promoter	RP11-505O17.1
chr1	6305000	chr1	225965000	3.05E-03	super_enhancer	promoter	SRP9
chr1	6315000	chr1	6485000	4.33E-02	super_enhancer	promoter	HES2
chr1	6315000	chr1	15735000	1.97E-02	super_enhancer	promoter	EFHD2
chr1	6315000	chr1	16245000	1.51E-02	super_enhancer	promoter	RP1-134O19.3
chr1	6315000	chr1	16315000	1.87E-02	super_enhancer	promoter	RP11-5P18.3
chr1	6315000	chr1	28995000	1.02E-02	super_enhancer	promoter	GMEB1
chr1	6315000	chr1	35325000	6.84E-03	super_enhancer	promoter	SMIM12
chr1	6315000	chr1	45265000	6.60E-03	super_enhancer	promoter	PLK3
chr1	6315000	chr1	151585000	2.12E-03	super_enhancer	promoter	SNX27
chr1	6315000	chr1	180515000	2.41E-03	super_enhancer	promoter	AL358354.1
chr1	6325000	chr1	17515000	1.29E-02	super_enhancer	promoter	RP11-380J14.1
chr1	6335000	chr1	46155000	4.91E-03	super_enhancer	promoter	GPBP1L1
chr1	6335000	chr1	161185000	1.88E-03	super_enhancer	promoter	FCER1G
chr1	6335000	chr1	203275000	2.35E-03	super_enhancer	promoter	LINC01136
chr1	6335000	chr1	204655000	2.09E-03	super_enhancer	promoter	LRRN2
chr1	6335000	chr1	224955000	2.34E-03	super_enhancer	promoter	RP11-449J1.1
chr1	6345000	chr1	6685000	3.78E-02	super_enhancer	promoter	THAP3
chr1	6345000	chr1	8485000	4.73E-02	super_enhancer	promoter	RP5-1115A15.1
chr1	6345000	chr1	32645000	6.41E-03	super_enhancer	promoter	TXLNA
chr1	6345000	chr1	33815000	5.62E-03	super_enhancer	promoter	RP11-415J8.5

chr1	6345000	chr1	92545000	1.57E-03	super_enhancer	promoter	BTBD8
chr1	6345000	chr1	173605000	1.80E-03	super_enhancer	promoter	RP11-360D2.1
chr1	6345000	chr1	219935000	1.37E-03	super_enhancer	promoter	RNA5SP76
chr1	6345000	chr1	241695000	1.28E-03	super_enhancer	promoter	KMO
chr1	6355000	chr1	11865000	2.79E-02	super_enhancer	promoter	CLCN6
chr1	6355000	chr1	11905000	3.43E-02	super_enhancer	promoter	NPPA-AS1_2
chr1	6355000	chr1	17865000	1.74E-02	super_enhancer	promoter	ARHGEF10L
chr1	6355000	chr1	26485000	1.35E-02	super_enhancer	promoter	FAM110D
chr1	6355000	chr1	153365000	2.75E-03	super_enhancer	promoter	S100A8
chr1	6355000	chr1	161655000	1.58E-03	super_enhancer	promoter	RPL31P11
chr1	6455000	chr1	16165000	1.80E-02	promoter	super_enhancer	ACOT7
chr1	6455000	chr1	32755000	9.90E-03	promoter	super_enhancer	ACOT7
chr1	6455000	chr1	233675000	2.71E-03	promoter	super_enhancer	ACOT7
chr1	6485000	chr1	11795000	4.45E-02	promoter	super_enhancer	HES2
chr1	6485000	chr1	16195000	1.91E-02	promoter	super_enhancer	HES2
chr1	6485000	chr1	63685000	2.80E-03	promoter	super_enhancer	HES2
chr1	6485000	chr1	109265000	2.58E-03	promoter	super_enhancer	HES2
chr1	6615000	chr1	12565000	2.80E-02	promoter	super_enhancer	NOL9
chr1	6615000	chr1	16135000	1.96E-02	promoter	super_enhancer	NOL9
chr1	6615000	chr1	42935000	6.65E-03	promoter	super_enhancer	NOL9
chr1	6615000	chr1	94575000	3.06E-03	promoter	super_enhancer	NOL9
chr1	6675000	chr1	16165000	1.87E-02	promoter	super_enhancer	KLHL21
chr1	6675000	chr1	43935000	6.32E-03	promoter	super_enhancer	KLHL21
chr1	6675000	chr1	214455000	2.53E-03	promoter	super_enhancer	KLHL21

chr1	6685000	chr1	7535000	2.81E-02	promoter	super_enhancer	THAP3
chr1	6685000	chr1	11795000	3.54E-02	promoter	super_enhancer	THAP3
chr1	6685000	chr1	11805000	2.93E-02	promoter	super_enhancer	THAP3
chr1	6685000	chr1	204105000	2.79E-03	promoter	super_enhancer	THAP3
chr1	6685000	chr1	212585000	2.49E-03	promoter	super_enhancer	THAP3
chr1	6785000	chr1	7535000	6.75E-04	promoter	super_enhancer	RP11-242F24.1
chr1	6785000	chr1	7545000	2.52E-02	promoter	super_enhancer	RP11-242F24.1
chr1	6785000	chr1	7555000	1.21E-02	promoter	super_enhancer	RP11-242F24.1
chr1	6785000	chr1	11805000	3.81E-02	promoter	super_enhancer	RP11-242F24.1
chr1	6785000	chr1	15355000	2.46E-02	promoter	super_enhancer	RP11-242F24.1
chr1	6785000	chr1	15405000	2.22E-02	promoter	super_enhancer	RP11-242F24.1
chr1	6845000	chr1	11805000	2.34E-02	promoter	super_enhancer	RP11-312B8.1
chr1	6895000	chr1	12575000	2.52E-02	promoter	super_enhancer	RP11-312B8.2
chr1	7075000	chr1	15405000	2.08E-02	promoter	super_enhancer	RP11-334N17.1
chr1	7075000	chr1	26875000	1.43E-02	promoter	super_enhancer	RP11-334N17.1
chr1	7075000	chr1	27555000	1.24E-02	promoter	super_enhancer	RP11-334N17.1
chr1	7075000	chr1	55445000	5.10E-03	promoter	super_enhancer	RP11-334N17.1
chr1	7075000	chr1	230135000	2.77E-03	promoter	super_enhancer	RP11-334N17.1
chr1	7535000	chr1	15765000	1.59E-02	super_enhancer	promoter	CTRC
chr1	7535000	chr1	32665000	6.07E-03	super_enhancer	promoter	CCDC28B
chr1	7545000	chr1	17635000	1.85E-02	super_enhancer	promoter	PADI4
chr1	7545000	chr1	52175000	4.29E-03	super_enhancer	promoter	GAPDHP51
chr1	7545000	chr1	119255000	1.87E-03	super_enhancer	promoter	RP4-630J13.1
chr1	7555000	chr1	10115000	1.87E-03	super_enhancer	promoter	RP11-496H15.2

chr1	7555000	chr1	22095000	1.39E-02	super_enhancer	promoter	RP11-132G19.3
chr1	7555000	chr1	25725000	5.34E-03	super_enhancer	promoter	RP3-469D22.1
chr1	7555000	chr1	35325000	6.62E-03	super_enhancer	promoter	SMIM12
chr1	7555000	chr1	40505000	6.48E-03	super_enhancer	promoter	CAP1
chr1	7555000	chr1	70995000	2.74E-03	super_enhancer	promoter	RP11-42O15.2
chr1	7755000	chr1	11805000	4.56E-02	promoter	super_enhancer	RP11-338N10.1
chr1	7755000	chr1	203535000	3.21E-03	promoter	super_enhancer	RP11-338N10.1
chr1	7835000	chr1	11795000	5.00E-02	promoter	super_enhancer	RP3-467L1.6
chr1	7835000	chr1	15325000	2.04E-02	promoter	super_enhancer	RP3-467L1.6
chr1	7835000	chr1	43885000	6.86E-03	promoter	super_enhancer	RP3-467L1.6
chr1	7835000	chr1	221965000	2.62E-03	promoter	super_enhancer	RP3-467L1.6
chr1	7915000	chr1	12545000	2.94E-02	promoter	super_enhancer	UTS2
chr1	7915000	chr1	58585000	3.26E-03	promoter	super_enhancer	UTS2
chr1	7915000	chr1	204415000	3.16E-03	promoter	super_enhancer	UTS2
chr1	8095000	chr1	15985000	1.92E-02	super_enhancer	promoter	RSC1A1
chr1	8095000	chr1	17305000	1.82E-02	super_enhancer	promoter	RP1-37C10.3
chr1	8095000	chr1	21835000	1.53E-02	super_enhancer	promoter	ALPL
chr1	8095000	chr1	24645000	1.97E-02	super_enhancer	promoter	GRHL3
chr1	8095000	chr1	43735000	9.10E-03	super_enhancer	promoter	TMEM125
chr1	8095000	chr1	44175000	8.22E-03	super_enhancer	promoter	KDM4A-AS1
chr1	8095000	chr1	44655000	6.96E-03	super_enhancer	promoter	RP5-891H21.5
chr1	8095000	chr1	231255000	2.38E-03	super_enhancer	promoter	RP5-1097F14.1
chr1	8485000	chr1	8905000	2.07E-03	promoter	super_enhancer	RP5-1115A15.1
chr1	8485000	chr1	9285000	2.65E-02	promoter	super_enhancer	RP5-1115A15.1

chr1	8485000	chr1	12585000	2.72E-02	promoter	super_enhancer	RP5-1115A15.1
chr1	8485000	chr1	181105000	1.71E-03	promoter	super_enhancer	RP5-1115A15.1
chr1	8875000	chr1	16245000	1.69E-02	super_enhancer	promoter	RP1-134O19.3
chr1	8875000	chr1	25535000	1.18E-02	super_enhancer	promoter	RP4-706G24.1
chr1	8875000	chr1	32675000	9.54E-03	super_enhancer	promoter	DCDC2B
chr1	8875000	chr1	179335000	2.31E-03	super_enhancer	promoter	AXDND1
chr1	8875000	chr1	227935000	2.80E-03	super_enhancer	promoter	SNAP47-AS1
chr1	8875000	chr1	247365000	1.84E-03	super_enhancer	promoter	MIR3916
chr1	8885000	chr1	16345000	2.99E-02	super_enhancer	promoter	CLCNKA
chr1	8885000	chr1	16865000	1.99E-02	super_enhancer	promoter	FAM231C
chr1	8885000	chr1	17575000	2.23E-02	super_enhancer	promoter	PADI3
chr1	8885000	chr1	28415000	1.34E-02	super_enhancer	promoter	EYA3
chr1	8885000	chr1	48555000	6.05E-03	super_enhancer	promoter	CYP46A4P
chr1	8895000	chr1	22095000	1.83E-02	super_enhancer	promoter	RP11-132G19.3
chr1	8895000	chr1	34335000	8.61E-03	super_enhancer	promoter	CSMD2-AS1
chr1	8895000	chr1	149285000	2.36E-03	super_enhancer	promoter	RP11-403I13.7
chr1	8895000	chr1	182055000	3.26E-03	super_enhancer	promoter	RP11-538D16.3
chr1	8905000	chr1	15735000	2.76E-02	super_enhancer	promoter	EFHD2
chr1	8905000	chr1	110025000	2.21E-03	super_enhancer	promoter	ATXN7L2
chr1	8905000	chr1	149225000	2.37E-03	super_enhancer	promoter	RNVU1-18
chr1	8905000	chr1	228595000	2.29E-03	super_enhancer	promoter	TRIM11
chr1	8945000	chr1	15405000	2.70E-02	promoter	super_enhancer	RNU6-304P
chr1	8945000	chr1	16175000	2.30E-02	promoter	super_enhancer	RNU6-304P
chr1	8945000	chr1	21635000	1.89E-02	promoter	super_enhancer	RNU6-304P

chr1	8945000	chr1	43485000	9.82E-03	promoter	super_enhancer	RNU6-304P
chr1	8945000	chr1	94785000	2.64E-03	promoter	super_enhancer	RNU6-304P
chr1	8945000	chr1	155315000	2.84E-03	promoter	super_enhancer	RNU6-304P
chr1	8945000	chr1	156105000	3.06E-03	promoter	super_enhancer	RNU6-304P
chr1	8945000	chr1	203535000	3.27E-03	promoter	super_enhancer	RNU6-304P
chr1	9005000	chr1	94735000	1.76E-03	promoter	super_enhancer	CA6
chr1	9005000	chr1	221425000	1.29E-03	promoter	super_enhancer	CA6
chr1	9275000	chr1	12705000	4.70E-02	super_enhancer	promoter	AADACL4
chr1	9275000	chr1	15935000	2.50E-02	super_enhancer	promoter	RNU7-179P
chr1	9275000	chr1	16315000	2.58E-02	super_enhancer	promoter	RP11-5P18.3
chr1	9275000	chr1	28695000	1.12E-02	super_enhancer	promoter	PHACTR4
chr1	9275000	chr1	224485000	2.46E-03	super_enhancer	promoter	RP11-365O16.5
chr1	9275000	chr1	233425000	2.19E-03	super_enhancer	promoter	RPS7P3
chr1	9285000	chr1	15985000	1.76E-02	super_enhancer	promoter	RSC1A1
chr1	9285000	chr1	116915000	1.92E-03	super_enhancer	promoter	ATP1A1
chr1	9285000	chr1	150265000	2.32E-03	super_enhancer	promoter	MRPS21
chr1	9285000	chr1	155815000	2.24E-03	super_enhancer	promoter	RP11-101O6.2
chr1	9285000	chr1	222185000	1.85E-03	super_enhancer	promoter	AC092765.1
chr1	9295000	chr1	16565000	2.60E-02	super_enhancer	promoter	RSG1
chr1	9295000	chr1	28905000	1.25E-02	super_enhancer	promoter	SNORD99
chr1	9295000	chr1	32635000	8.62E-03	super_enhancer	promoter	RP4-622L5.2
chr1	9295000	chr1	44555000	8.06E-03	super_enhancer	promoter	RP5-1198O20.5
chr1	9295000	chr1	165185000	1.85E-03	super_enhancer	promoter	RP11-38C18.3
chr1	9305000	chr1	9635000	6.83E-06	super_enhancer	promoter	RP13-15M17.1

chr1	9305000	chr1	15935000	2.95E-02	super_enhancer	promoter	RNU7-179P
chr1	9305000	chr1	26495000	1.79E-02	super_enhancer	promoter	ZNF593
chr1	9305000	chr1	52005000	6.71E-03	super_enhancer	promoter	RNU6-1281P
chr1	9305000	chr1	226375000	3.66E-03	super_enhancer	promoter	ACBD3
chr1	9305000	chr1	233585000	2.53E-03	super_enhancer	promoter	RNU4-77P
chr1	9315000	chr1	9565000	4.74E-03	super_enhancer	promoter	RP13-392I16.1
chr1	9315000	chr1	9635000	3.70E-02	super_enhancer	promoter	RP13-15M17.1
chr1	9565000	chr1	235005000	2.94E-03	promoter	super_enhancer	RP13-392I16.1
chr1	9635000	chr1	41875000	1.08E-02	promoter	super_enhancer	RP13-15M17.1
chr1	9635000	chr1	229225000	2.48E-03	promoter	super_enhancer	RP13-15M17.1
chr1	9635000	chr1	235065000	2.43E-03	promoter	super_enhancer	RP13-15M17.1
chr1	9715000	chr1	150565000	2.56E-03	promoter	super_enhancer	PIK3CD-AS1
chr1	9715000	chr1	212595000	2.63E-03	promoter	super_enhancer	PIK3CD-AS1
chr1	9885000	chr1	16205000	2.01E-02	promoter	super_enhancer	CLSTN1
chr1	9885000	chr1	26875000	1.39E-02	promoter	super_enhancer	CLSTN1
chr1	9885000	chr1	41865000	8.35E-03	promoter	super_enhancer	CLSTN1
chr1	10115000	chr1	204425000	2.76E-03	promoter	super_enhancer	RP11-496H15.2
chr1	10955000	chr1	114455000	2.25E-03	promoter	super_enhancer	HSPE1P24
chr1	10955000	chr1	203535000	2.80E-03	promoter	super_enhancer	HSPE1P24
chr1	11785000	chr1	16555000	4.13E-02	super_enhancer	promoter	ANO7P1
chr1	11785000	chr1	53705000	5.39E-03	super_enhancer	promoter	MAGOH
chr1	11785000	chr1	59675000	3.71E-03	super_enhancer	promoter	HSD52
chr1	11785000	chr1	79215000	2.56E-03	super_enhancer	promoter	RP4-641G12.4
chr1	11795000	chr1	16315000	4.80E-02	super_enhancer	promoter	RP11-5P18.3

chr1	11795000	chr1	42845000	1.04E-02	super_enhancer	promoter	RIMKLA
chr1	11795000	chr1	53925000	7.58E-03	super_enhancer	promoter	DMRTB1
chr1	11795000	chr1	197725000	2.50E-03	super_enhancer	promoter	RP11-448G4.4
chr1	11805000	chr1	15735000	4.79E-02	super_enhancer	promoter	EFHD2
chr1	11805000	chr1	16485000	4.47E-02	super_enhancer	promoter	RP11-276H7.3
chr1	11805000	chr1	28695000	1.25E-02	super_enhancer	promoter	PHACTR4
chr1	11805000	chr1	35395000	9.00E-03	super_enhancer	promoter	DLGAP3
chr1	11805000	chr1	36025000	9.66E-03	super_enhancer	promoter	KIAA0319L
chr1	11805000	chr1	160175000	2.64E-03	super_enhancer	promoter	PEA15
chr1	11815000	chr1	19935000	1.45E-02	super_enhancer	promoter	RP5-1056L3.3
chr1	11815000	chr1	93545000	1.47E-03	super_enhancer	promoter	MTF2
chr1	11825000	chr1	47645000	9.04E-03	super_enhancer	promoter	CYP4A22-AS1
chr1	11825000	chr1	63605000	4.08E-03	super_enhancer	promoter	RP11-335E6.2
chr1	11825000	chr1	203275000	3.34E-03	super_enhancer	promoter	LINC01136
chr1	11825000	chr1	203445000	2.70E-03	super_enhancer	promoter	PRELP
chr1	11835000	chr1	16735000	4.85E-02	super_enhancer	promoter	AL358794.1
chr1	11835000	chr1	23415000	2.06E-02	super_enhancer	promoter	RP1-184J9.2
chr1	11835000	chr1	36185000	1.04E-02	super_enhancer	promoter	C1orf216
chr1	11835000	chr1	48555000	6.53E-03	super_enhancer	promoter	CYP46A4P
chr1	11835000	chr1	59465000	4.78E-03	super_enhancer	promoter	PHBP3
chr1	11835000	chr1	155215000	2.64E-03	super_enhancer	promoter	GBA
chr1	11835000	chr1	203765000	3.15E-03	super_enhancer	promoter	ZC3H11A
chr1	11835000	chr1	229175000	2.43E-03	super_enhancer	promoter	RP5-1065P14.3
chr1	11835000	chr1	229685000	3.10E-03	super_enhancer	promoter	RNA5SP78

chr1	11865000	chr1	16165000	3.38E-02	promoter	super_enhancer	CLCN6
chr1	11865000	chr1	32785000	1.03E-02	promoter	super_enhancer	CLCN6
chr1	11905000	chr1	16135000	4.68E-02	promoter	super_enhancer	NPPA-AS1_2
chr1	11905000	chr1	94785000	2.68E-03	promoter	super_enhancer	NPPA-AS1_2
chr1	11905000	chr1	164775000	2.14E-03	promoter	super_enhancer	NPPA-AS1_2
chr1	12545000	chr1	28995000	1.18E-02	super_enhancer	promoter	GMEB1
chr1	12545000	chr1	34335000	7.80E-03	super_enhancer	promoter	CSMD2-AS1
chr1	12545000	chr1	62095000	4.51E-03	super_enhancer	promoter	AC099791.1
chr1	12545000	chr1	69715000	2.51E-03	super_enhancer	promoter	RP11-74K19.1
chr1	12555000	chr1	52615000	5.67E-03	super_enhancer	promoter	RN7SL788P
chr1	12555000	chr1	62995000	3.74E-03	super_enhancer	promoter	RP11-293K19.1
chr1	12555000	chr1	114545000	2.34E-03	super_enhancer	promoter	RP4-590F24.1
chr1	12555000	chr1	180515000	2.63E-03	super_enhancer	promoter	AL358354.1
chr1	12555000	chr1	219935000	1.81E-03	super_enhancer	promoter	RNA5SP76
chr1	12565000	chr1	16565000	4.86E-02	super_enhancer	promoter	RSG1
chr1	12565000	chr1	110935000	1.98E-03	super_enhancer	promoter	SLC16A4
chr1	12565000	chr1	211665000	3.08E-03	super_enhancer	promoter	RP11-359E8.3
chr1	12565000	chr1	229455000	2.39E-03	super_enhancer	promoter	RP4-803J11.2
chr1	12575000	chr1	17865000	3.35E-02	super_enhancer	promoter	ARHGEF10L
chr1	12575000	chr1	23075000	1.76E-02	super_enhancer	promoter	AL611946.1
chr1	12575000	chr1	28415000	1.41E-02	super_enhancer	promoter	EYA3
chr1	12575000	chr1	50865000	5.72E-03	super_enhancer	promoter	HMGB1P45
chr1	12575000	chr1	95975000	2.34E-03	super_enhancer	promoter	RP11-286B14.1
chr1	12585000	chr1	15985000	4.31E-02	super_enhancer	promoter	RSC1A1

chr1	12585000	chr1	16485000	1.95E-03	super_enhancer	promoter	RP11-276H7.3
chr1	12585000	chr1	43035000	6.96E-03	super_enhancer	promoter	RNU6-536P
chr1	12585000	chr1	59545000	5.37E-03	super_enhancer	promoter	RP11-145M4.2
chr1	12585000	chr1	235815000	3.03E-03	super_enhancer	promoter	GNG4
chr1	12595000	chr1	15985000	3.70E-02	super_enhancer	promoter	RSC1A1
chr1	12595000	chr1	16245000	4.00E-02	super_enhancer	promoter	RP1-134O19.3
chr1	12595000	chr1	20355000	1.42E-02	super_enhancer	promoter	PLA2G5
chr1	12595000	chr1	62655000	5.56E-03	super_enhancer	promoter	PIGPP2
chr1	12595000	chr1	77525000	2.44E-03	super_enhancer	promoter	MIR7156
chr1	12595000	chr1	161135000	2.03E-03	super_enhancer	promoter	PPOX
chr1	12595000	chr1	233585000	2.04E-03	super_enhancer	promoter	RNU4-77P
chr1	12605000	chr1	16555000	4.57E-02	super_enhancer	promoter	ANO7P1
chr1	12605000	chr1	29475000	1.35E-02	super_enhancer	promoter	RP11-242O24.3
chr1	12605000	chr1	59755000	3.78E-03	super_enhancer	promoter	RP11-467C18.1
chr1	12605000	chr1	247615000	2.50E-03	super_enhancer	promoter	OR2B11
chr1	12705000	chr1	16165000	4.44E-02	promoter	super_enhancer	AADACL4
chr1	12805000	chr1	15335000	4.59E-02	promoter	super_enhancer	C1orf158
chr1	12805000	chr1	32715000	7.40E-03	promoter	super_enhancer	C1orf158
chr1	12805000	chr1	32775000	8.26E-03	promoter	super_enhancer	C1orf158
chr1	12805000	chr1	172935000	1.13E-03	promoter	super_enhancer	C1orf158
chr1	12835000	chr1	34785000	7.13E-03	promoter	super_enhancer	PRAMEF12
chr1	12985000	chr1	153565000	1.11E-03	promoter	super_enhancer	RP11-450I1.2
chr1	14925000	chr1	15425000	6.54E-05	promoter	super_enhancer	KAZN
chr1	14935000	chr1	15355000	1.14E-02	promoter	super_enhancer	AL034395.1

chr1	14935000	chr1	15405000	5.72E-03	promoter	super_enhancer	AL034395.1
chr1	14935000	chr1	32725000	1.21E-02	promoter	super_enhancer	AL034395.1
chr1	14935000	chr1	164745000	1.89E-03	promoter	super_enhancer	AL034395.1
chr1	15325000	chr1	26795000	8.48E-03	super_enhancer	promoter	RP3-476K8.3
chr1	15325000	chr1	41405000	1.08E-02	super_enhancer	promoter	RP11-348A7.1
chr1	15325000	chr1	46835000	8.07E-03	super_enhancer	promoter	RP4-603I14.3
chr1	15335000	chr1	229385000	2.24E-03	super_enhancer	promoter	TMEM78
chr1	15345000	chr1	47965000	7.38E-03	super_enhancer	promoter	RPL21P24
chr1	15345000	chr1	95285000	2.20E-03	super_enhancer	promoter	SLC44A3
chr1	15345000	chr1	156615000	2.70E-03	super_enhancer	promoter	RP11-284F21.10
chr1	15345000	chr1	215115000	2.08E-03	super_enhancer	promoter	RP11-323K10.1
chr1	15355000	chr1	35255000	8.43E-03	super_enhancer	promoter	RP1-34M23.5
chr1	15355000	chr1	62995000	4.09E-03	super_enhancer	promoter	RP11-293K19.1
chr1	15355000	chr1	65525000	3.85E-03	super_enhancer	promoter	MIR3671
chr1	15355000	chr1	92545000	2.27E-03	super_enhancer	promoter	BTBD8
chr1	15355000	chr1	101185000	1.80E-03	super_enhancer	promoter	VCAM1
chr1	15405000	chr1	34325000	1.03E-02	super_enhancer	promoter	HMGB4
chr1	15405000	chr1	47645000	8.44E-03	super_enhancer	promoter	CYP4A22-AS1
chr1	15405000	chr1	65525000	3.49E-03	super_enhancer	promoter	MIR3671
chr1	15405000	chr1	203145000	3.23E-03	super_enhancer	promoter	MYBPH
chr1	15415000	chr1	35315000	1.19E-02	super_enhancer	promoter	RP5-997D16.2
chr1	15415000	chr1	46915000	1.15E-02	super_enhancer	promoter	LINC01398
chr1	15415000	chr1	245135000	2.31E-03	super_enhancer	promoter	RP11-156E8.1
chr1	15425000	chr1	47875000	6.87E-03	super_enhancer	promoter	LINC01389

chr1	15425000	chr1	161195000	2.56E-03	super_enhancer	promoter	TOMM40L
chr1	15425000	chr1	192605000	1.74E-03	super_enhancer	promoter	RGS13
chr1	15765000	chr1	20585000	2.40E-02	promoter	super_enhancer	CTRC
chr1	15765000	chr1	32715000	1.39E-02	promoter	super_enhancer	CTRC
chr1	15765000	chr1	151945000	1.75E-03	promoter	super_enhancer	CTRC
chr1	15765000	chr1	233655000	2.18E-03	promoter	super_enhancer	CTRC
chr1	15935000	chr1	36385000	1.19E-02	promoter	super_enhancer	RNU7-179P
chr1	15985000	chr1	155315000	1.87E-03	promoter	super_enhancer	RSC1A1
chr1	16125000	chr1	26395000	1.63E-02	super_enhancer	promoter	TRIM63
chr1	16125000	chr1	79115000	2.48E-03	super_enhancer	promoter	IFI44
chr1	16125000	chr1	160085000	2.34E-03	super_enhancer	promoter	ATP1A2
chr1	16125000	chr1	168745000	1.72E-03	super_enhancer	promoter	RP1-10C16.1
chr1	16125000	chr1	199985000	2.92E-03	super_enhancer	promoter	RNU6-609P
chr1	16135000	chr1	16295000	8.22E-03	super_enhancer	promoter	RP11-5P18.1
chr1	16135000	chr1	16315000	1.18E-02	super_enhancer	promoter	RP11-5P18.3
chr1	16135000	chr1	22385000	2.71E-02	super_enhancer	promoter	CDC42-IT1
chr1	16135000	chr1	110135000	2.19E-03	super_enhancer	promoter	RNU6V
chr1	16145000	chr1	16245000	3.25E-02	super_enhancer	promoter	RP1-134O19.3
chr1	16145000	chr1	16295000	1.49E-02	super_enhancer	promoter	RP11-5P18.1
chr1	16145000	chr1	16315000	3.39E-04	super_enhancer	promoter	RP11-5P18.3
chr1	16145000	chr1	16345000	1.57E-02	super_enhancer	promoter	CLCNKA
chr1	16145000	chr1	52175000	5.54E-03	super_enhancer	promoter	GAPDHP51
chr1	16145000	chr1	57085000	5.96E-03	super_enhancer	promoter	RP11-377K22.2
chr1	16145000	chr1	63605000	4.60E-03	super_enhancer	promoter	RP11-335E6.2

chr1	16145000	chr1	92415000	3.34E-03	super_enhancer	promoter	BRDT
chr1	16145000	chr1	94395000	2.64E-03	super_enhancer	promoter	RP5-837O21.1
chr1	16145000	chr1	109235000	2.53E-03	super_enhancer	promoter	PRPF38B
chr1	16145000	chr1	157695000	1.97E-03	super_enhancer	promoter	VDAC1P9
chr1	16145000	chr1	175015000	3.17E-03	super_enhancer	promoter	SDCCAG3P2
chr1	16145000	chr1	231005000	3.38E-03	super_enhancer	promoter	C1orf198
chr1	16155000	chr1	16245000	2.52E-03	super_enhancer	promoter	RP1-134O19.3
chr1	16155000	chr1	16315000	1.68E-02	super_enhancer	promoter	RP11-5P18.3
chr1	16155000	chr1	93435000	2.21E-03	super_enhancer	promoter	RNU6-970P
chr1	16155000	chr1	112085000	1.88E-03	super_enhancer	promoter	RAP1A
chr1	16155000	chr1	116015000	2.49E-03	super_enhancer	promoter	RP4-663N10.2
chr1	16155000	chr1	165645000	2.21E-03	super_enhancer	promoter	RP11-466F5.4
chr1	16155000	chr1	215115000	2.24E-03	super_enhancer	promoter	RP11-323K10.1
chr1	16155000	chr1	224445000	2.58E-03	super_enhancer	promoter	MIR320B2
chr1	16155000	chr1	245135000	2.39E-03	super_enhancer	promoter	RP11-156E8.1
chr1	16165000	chr1	16295000	9.44E-03	super_enhancer	promoter	RP11-5P18.1
chr1	16165000	chr1	16345000	2.22E-03	super_enhancer	promoter	CLCNKA
chr1	16165000	chr1	113435000	2.05E-03	super_enhancer	promoter	RP3-522D1.2
chr1	16165000	chr1	185125000	2.10E-03	super_enhancer	promoter	SWT1
chr1	16175000	chr1	16245000	2.44E-03	super_enhancer	promoter	RP1-134O19.3
chr1	16175000	chr1	16315000	2.95E-03	super_enhancer	promoter	RP11-5P18.3
chr1	16175000	chr1	16485000	3.96E-02	super_enhancer	promoter	RP11-276H7.3
chr1	16175000	chr1	32665000	1.11E-02	super_enhancer	promoter	CCDC28B
chr1	16175000	chr1	62995000	3.58E-03	super_enhancer	promoter	RP11-293K19.1

chr1	16175000	chr1	63155000	3.80E-03	super_enhancer	promoter	DOCK7
chr1	16175000	chr1	102195000	1.66E-03	super_enhancer	promoter	RNU6-965P
chr1	16175000	chr1	238025000	1.91E-03	super_enhancer	promoter	RP11-193H5.1
chr1	16185000	chr1	20465000	3.94E-02	super_enhancer	promoter	PLA2G2F
chr1	16185000	chr1	35225000	8.34E-03	super_enhancer	promoter	GJB4
chr1	16185000	chr1	51795000	6.90E-03	super_enhancer	promoter	TTC39A-AS1
chr1	16185000	chr1	104095000	1.20E-03	super_enhancer	promoter	AMY2B
chr1	16185000	chr1	224485000	2.34E-03	super_enhancer	promoter	RP11-365O16.5
chr1	16195000	chr1	44445000	8.19E-03	super_enhancer	promoter	B4GALT2
chr1	16195000	chr1	84545000	1.61E-03	super_enhancer	promoter	RP11-486G15.2
chr1	16195000	chr1	93645000	1.93E-03	super_enhancer	promoter	CCDC18
chr1	16195000	chr1	171725000	2.16E-03	super_enhancer	promoter	RP4-560B9.6
chr1	16195000	chr1	233035000	2.04E-03	super_enhancer	promoter	AL122003.1
chr1	16205000	chr1	26185000	1.57E-02	super_enhancer	promoter	RP1-125I3.2
chr1	16205000	chr1	28585000	1.47E-02	super_enhancer	promoter	SESN2
chr1	16205000	chr1	32255000	9.43E-03	super_enhancer	promoter	RP11-84A19.3
chr1	16205000	chr1	46835000	7.34E-03	super_enhancer	promoter	RP4-603I14.3
chr1	16205000	chr1	51425000	5.91E-03	super_enhancer	promoter	CDKN2C
chr1	16205000	chr1	203275000	2.44E-03	super_enhancer	promoter	LINC01136
chr1	16245000	chr1	43895000	9.69E-03	promoter	super_enhancer	RP1-134O19.3
chr1	16245000	chr1	164745000	1.75E-03	promoter	super_enhancer	RP1-134O19.3
chr1	16295000	chr1	32775000	1.35E-02	promoter	super_enhancer	RP11-5P18.1
chr1	16295000	chr1	153615000	2.10E-03	promoter	super_enhancer	RP11-5P18.1
chr1	16295000	chr1	164645000	1.84E-03	promoter	super_enhancer	RP11-5P18.1

chr1	16295000	chr1	204095000	3.22E-03	promoter	super_enhancer	RP11-5P18.1
chr1	16315000	chr1	36385000	1.32E-02	promoter	super_enhancer	RP11-5P18.3
chr1	16315000	chr1	43485000	1.24E-02	promoter	super_enhancer	RP11-5P18.3
chr1	16345000	chr1	94575000	3.71E-03	promoter	super_enhancer	CLCNKA
chr1	16485000	chr1	32735000	1.65E-02	promoter	super_enhancer	RP11-276H7.3
chr1	16485000	chr1	32995000	1.68E-02	promoter	super_enhancer	RP11-276H7.3
chr1	16555000	chr1	85295000	2.47E-03	promoter	super_enhancer	ANO7P1
chr1	16555000	chr1	181085000	3.50E-03	promoter	super_enhancer	ANO7P1
chr1	16555000	chr1	181095000	2.71E-03	promoter	super_enhancer	ANO7P1
chr1	16565000	chr1	43405000	8.27E-03	promoter	super_enhancer	RSG1
chr1	16735000	chr1	32995000	1.68E-02	promoter	super_enhancer	AL358794.1
chr1	16765000	chr1	150605000	1.96E-03	promoter	super_enhancer	SPATA21
chr1	16865000	chr1	26875000	1.73E-02	promoter	super_enhancer	FAM231C
chr1	16865000	chr1	226815000	1.95E-03	promoter	super_enhancer	FAM231C
chr1	17215000	chr1	27505000	2.51E-02	promoter	super_enhancer	RP11-108M9.5
chr1	17215000	chr1	27525000	2.48E-02	promoter	super_enhancer	RP11-108M9.5
chr1	17215000	chr1	42955000	1.02E-02	promoter	super_enhancer	RP11-108M9.5
chr1	17215000	chr1	43375000	1.28E-02	promoter	super_enhancer	RP11-108M9.5
chr1	17215000	chr1	94625000	3.38E-03	promoter	super_enhancer	RP11-108M9.5
chr1	17515000	chr1	26875000	2.10E-02	promoter	super_enhancer	RP11-380J14.1
chr1	17515000	chr1	203515000	2.70E-03	promoter	super_enhancer	RP11-380J14.1
chr1	17575000	chr1	43865000	8.57E-03	promoter	super_enhancer	PADI3
chr1	17575000	chr1	46185000	5.84E-03	promoter	super_enhancer	PADI3
chr1	17575000	chr1	204125000	2.58E-03	promoter	super_enhancer	PADI3

chr1	17635000	chr1	20585000	3.71E-02	promoter	super_enhancer	PADI4
chr1	17635000	chr1	151945000	1.44E-03	promoter	super_enhancer	PADI4
chr1	17635000	chr1	205325000	2.88E-03	promoter	super_enhancer	PADI4
chr1	17865000	chr1	32765000	1.62E-02	promoter	super_enhancer	ARHGEF10L
chr1	17865000	chr1	55445000	6.59E-03	promoter	super_enhancer	ARHGEF10L
chr1	17865000	chr1	85315000	3.47E-03	promoter	super_enhancer	ARHGEF10L
chr1	17865000	chr1	94575000	3.42E-03	promoter	super_enhancer	ARHGEF10L
chr1	18435000	chr1	40865000	7.68E-03	promoter	super_enhancer	RP11-174G17.3
chr1	18435000	chr1	46185000	7.39E-03	promoter	super_enhancer	RP11-174G17.3
chr1	19225000	chr1	26875000	2.96E-02	promoter	super_enhancer	MIR1290
chr1	19225000	chr1	42945000	9.67E-03	promoter	super_enhancer	MIR1290
chr1	19225000	chr1	43925000	1.07E-02	promoter	super_enhancer	MIR1290
chr1	19225000	chr1	93765000	2.20E-03	promoter	super_enhancer	MIR1290
chr1	19225000	chr1	112635000	1.80E-03	promoter	super_enhancer	MIR1290
chr1	19935000	chr1	32985000	1.43E-02	promoter	super_enhancer	RP5-1056L3.3
chr1	19935000	chr1	41855000	9.01E-03	promoter	super_enhancer	RP5-1056L3.3
chr1	19935000	chr1	200455000	2.33E-03	promoter	super_enhancer	RP5-1056L3.3
chr1	19935000	chr1	221965000	2.32E-03	promoter	super_enhancer	RP5-1056L3.3
chr1	20355000	chr1	207025000	1.65E-03	promoter	super_enhancer	PLA2G5
chr1	20465000	chr1	32995000	1.69E-02	promoter	super_enhancer	PLA2G2F
chr1	20585000	chr1	28695000	1.33E-02	super_enhancer	promoter	PHACTR4
chr1	20585000	chr1	32395000	7.57E-03	super_enhancer	promoter	AL136115.1
chr1	20585000	chr1	35325000	8.27E-03	super_enhancer	promoter	SMIM12
chr1	20585000	chr1	44445000	7.21E-03	super_enhancer	promoter	B4GALT2

chr1	20595000	chr1	28585000	2.36E-02	super_enhancer	promoter	SESN2
chr1	20595000	chr1	36395000	8.49E-03	super_enhancer	promoter	AGO3
chr1	20595000	chr1	41405000	1.40E-02	super_enhancer	promoter	RP11-348A7.1
chr1	20595000	chr1	44655000	8.79E-03	super_enhancer	promoter	RP5-891H21.5
chr1	20595000	chr1	46645000	1.16E-02	super_enhancer	promoter	AL672043.1
chr1	20595000	chr1	53925000	8.12E-03	super_enhancer	promoter	DMRTB1
chr1	20595000	chr1	62655000	7.19E-03	super_enhancer	promoter	PIGPP2
chr1	20595000	chr1	87595000	3.06E-03	super_enhancer	promoter	LINC01140
chr1	20595000	chr1	92655000	2.85E-03	super_enhancer	promoter	RP4-621B10.8
chr1	20595000	chr1	169455000	2.58E-03	super_enhancer	promoter	SLC19A2
chr1	20595000	chr1	178695000	2.36E-03	super_enhancer	promoter	RP11-428K3.1
chr1	20595000	chr1	182405000	2.56E-03	super_enhancer	promoter	RP1-223H12.5
chr1	20595000	chr1	186265000	1.82E-03	super_enhancer	promoter	PRG4
chr1	20605000	chr1	145525000	2.05E-03	super_enhancer	promoter	ITGA10
chr1	20605000	chr1	159335000	1.45E-03	super_enhancer	promoter	OR10J8P
chr1	20605000	chr1	159825000	2.50E-03	super_enhancer	promoter	C1orf204
chr1	20605000	chr1	185265000	1.61E-03	super_enhancer	promoter	RP4-635A23.6
chr1	20615000	chr1	24255000	4.59E-02	super_enhancer	promoter	MIR378F
chr1	20615000	chr1	26645000	2.91E-02	super_enhancer	promoter	UBXN11
chr1	20615000	chr1	28855000	2.04E-02	super_enhancer	promoter	PRDX3P2
chr1	20615000	chr1	29205000	2.35E-02	super_enhancer	promoter	RP1-212P9.3
chr1	20615000	chr1	35315000	1.46E-02	super_enhancer	promoter	RP5-997D16.2
chr1	20615000	chr1	37375000	1.04E-02	super_enhancer	promoter	AC117945.1
chr1	20615000	chr1	113615000	1.58E-03	super_enhancer	promoter	LRIG2

chr1	20615000	chr1	175175000	2.25E-03	super_enhancer	promoter	RP5-1114G22.2
chr1	20615000	chr1	180925000	2.65E-03	super_enhancer	promoter	KIAA1614-AS1
chr1	20615000	chr1	238025000	2.13E-03	super_enhancer	promoter	RP11-193H5.1
chr1	20835000	chr1	41845000	9.79E-03	promoter	super_enhancer	MUL1
chr1	20835000	chr1	42965000	5.61E-03	promoter	super_enhancer	MUL1
chr1	20835000	chr1	223705000	1.55E-03	promoter	super_enhancer	MUL1
chr1	20835000	chr1	230105000	1.81E-03	promoter	super_enhancer	MUL1
chr1	20915000	chr1	230125000	2.05E-03	promoter	super_enhancer	CDA
chr1	21045000	chr1	34765000	9.45E-03	promoter	super_enhancer	KIF17
chr1	21045000	chr1	94705000	1.62E-03	promoter	super_enhancer	KIF17
chr1	21045000	chr1	94785000	2.39E-03	promoter	super_enhancer	KIF17
chr1	21045000	chr1	151975000	1.74E-03	promoter	super_enhancer	KIF17
chr1	21045000	chr1	204415000	2.85E-03	promoter	super_enhancer	KIF17
chr1	21115000	chr1	27495000	2.43E-02	promoter	super_enhancer	HP1BP3
chr1	21115000	chr1	234995000	2.56E-03	promoter	super_enhancer	HP1BP3
chr1	21625000	chr1	109585000	2.05E-03	super_enhancer	promoter	WDR47
chr1	21625000	chr1	203595000	2.35E-03	super_enhancer	promoter	ATP2B4
chr1	21625000	chr1	222885000	2.42E-03	super_enhancer	promoter	BROX
chr1	21625000	chr1	228395000	2.03E-03	super_enhancer	promoter	OBSCN
chr1	21635000	chr1	203775000	3.13E-03	super_enhancer	promoter	RP11-397P13.6
chr1	21755000	chr1	32995000	7.59E-03	promoter	super_enhancer	NBPF2P
chr1	21755000	chr1	164755000	1.11E-03	promoter	super_enhancer	NBPF2P
chr1	21835000	chr1	94635000	2.33E-03	promoter	super_enhancer	ALPL
chr1	22095000	chr1	27535000	2.94E-02	promoter	super_enhancer	RP11-132G19.3

chr1	22095000	chr1	42925000	1.36E-02	promoter	super_enhancer	RP11-132G19.3
chr1	22095000	chr1	43855000	1.57E-02	promoter	super_enhancer	RP11-132G19.3
chr1	22095000	chr1	55445000	8.31E-03	promoter	super_enhancer	RP11-132G19.3
chr1	22095000	chr1	159915000	2.70E-03	promoter	super_enhancer	RP11-132G19.3
chr1	22235000	chr1	26345000	2.94E-02	promoter	super_enhancer	RP11-26H16.1
chr1	22235000	chr1	27565000	1.97E-02	promoter	super_enhancer	RP11-26H16.1
chr1	22385000	chr1	155305000	2.31E-03	promoter	super_enhancer	CDC42-IT1
chr1	22395000	chr1	204115000	3.34E-03	promoter	super_enhancer	RP1-224A6.8
chr1	22395000	chr1	214465000	2.16E-03	promoter	super_enhancer	RP1-224A6.8
chr1	23045000	chr1	23185000	1.81E-02	promoter	super_enhancer	MIR4684
chr1	23045000	chr1	23195000	2.59E-05	promoter	super_enhancer	MIR4684
chr1	23045000	chr1	26865000	4.37E-02	promoter	super_enhancer	MIR4684
chr1	23075000	chr1	26865000	4.59E-02	promoter	super_enhancer	AL611946.1
chr1	23185000	chr1	44585000	9.72E-03	super_enhancer	promoter	KLF17
chr1	23185000	chr1	59755000	4.49E-03	super_enhancer	promoter	RP11-467C18.1
chr1	23195000	chr1	26535000	4.38E-02	super_enhancer	promoter	RP11-231P20.2
chr1	23195000	chr1	32225000	1.99E-02	super_enhancer	promoter	MIR4254
chr1	23195000	chr1	62765000	4.59E-03	super_enhancer	promoter	RNU6-371P
chr1	23195000	chr1	84945000	2.69E-03	super_enhancer	promoter	RPF1
chr1	23195000	chr1	90455000	2.66E-03	super_enhancer	promoter	RN7SKP272
chr1	23195000	chr1	155555000	2.10E-03	super_enhancer	promoter	DAP3P1
chr1	23195000	chr1	161185000	2.10E-03	super_enhancer	promoter	FCER1G
chr1	23195000	chr1	173145000	2.02E-03	super_enhancer	promoter	RP3-395P12.2
chr1	23345000	chr1	41905000	9.08E-03	promoter	super_enhancer	KDM1A

chr1	23345000	chr1	63625000	2.66E-03	promoter	super_enhancer	KDM1A
chr1	23345000	chr1	63695000	2.60E-03	promoter	super_enhancer	KDM1A
chr1	23345000	chr1	150605000	9.68E-04	promoter	super_enhancer	KDM1A
chr1	23385000	chr1	43855000	1.10E-02	promoter	super_enhancer	MIR4419A
chr1	23385000	chr1	43935000	8.36E-03	promoter	super_enhancer	MIR4419A
chr1	23385000	chr1	230105000	1.69E-03	promoter	super_enhancer	MIR4419A
chr1	23415000	chr1	32775000	2.30E-02	promoter	super_enhancer	RP1-184J9.2
chr1	23505000	chr1	27535000	3.42E-02	promoter	super_enhancer	LUZP1
chr1	23505000	chr1	31935000	1.44E-02	promoter	super_enhancer	LUZP1
chr1	24105000	chr1	43425000	1.26E-02	promoter	super_enhancer	TCEB3-AS1
chr1	24165000	chr1	112635000	1.27E-03	promoter	super_enhancer	HMGCL
chr1	24195000	chr1	234985000	1.93E-03	promoter	super_enhancer	FUCA1
chr1	24255000	chr1	34765000	1.23E-02	promoter	super_enhancer	MIR378F
chr1	24285000	chr1	32725000	1.61E-02	promoter	super_enhancer	PNRC2
chr1	24285000	chr1	183195000	1.61E-03	promoter	super_enhancer	PNRC2
chr1	24335000	chr1	43435000	1.41E-02	promoter	super_enhancer	RP11-4M23.7
chr1	24335000	chr1	43485000	1.52E-02	promoter	super_enhancer	RP11-4M23.7
chr1	24335000	chr1	91605000	3.05E-03	promoter	super_enhancer	RP11-4M23.7
chr1	24515000	chr1	32745000	2.64E-02	promoter	super_enhancer	IFNLR1
chr1	24645000	chr1	200415000	3.45E-03	promoter	super_enhancer	GRHL3
chr1	25105000	chr1	41845000	1.50E-02	promoter	super_enhancer	RNU6-1208P
chr1	25105000	chr1	150605000	1.91E-03	promoter	super_enhancer	RNU6-1208P
chr1	25105000	chr1	233725000	2.16E-03	promoter	super_enhancer	RNU6-1208P
chr1	25535000	chr1	26865000	2.72E-02	promoter	super_enhancer	RP4-706G24.1

chr1	25595000	chr1	26335000	3.05E-02	promoter	super_enhancer	RP3-465N24.5
chr1	25595000	chr1	32775000	1.61E-02	promoter	super_enhancer	RP3-465N24.5
chr1	25665000	chr1	26335000	4.84E-02	promoter	super_enhancer	RSRP1
chr1	25665000	chr1	41915000	1.33E-02	promoter	super_enhancer	RSRP1
chr1	25665000	chr1	94645000	1.90E-03	promoter	super_enhancer	RSRP1
chr1	26145000	chr1	32765000	2.99E-02	promoter	super_enhancer	MTFR1L
chr1	26185000	chr1	26335000	2.98E-02	promoter	super_enhancer	RP1-125I3.2
chr1	26185000	chr1	26345000	1.69E-02	promoter	super_enhancer	RP1-125I3.2
chr1	26185000	chr1	32765000	2.96E-02	promoter	super_enhancer	RP1-125I3.2
chr1	26185000	chr1	32785000	2.73E-02	promoter	super_enhancer	RP1-125I3.2
chr1	26185000	chr1	43475000	1.31E-02	promoter	super_enhancer	RP1-125I3.2
chr1	26185000	chr1	212565000	2.35E-03	promoter	super_enhancer	RP1-125I3.2
chr1	26215000	chr1	26345000	1.82E-02	promoter	super_enhancer	RP1-125I3.4
chr1	26215000	chr1	43375000	1.17E-02	promoter	super_enhancer	RP1-125I3.4
chr1	26215000	chr1	58595000	4.19E-03	promoter	super_enhancer	RP1-125I3.4
chr1	26215000	chr1	94715000	1.95E-03	promoter	super_enhancer	RP1-125I3.4
chr1	26215000	chr1	150565000	2.43E-03	promoter	super_enhancer	RP1-125I3.4
chr1	26215000	chr1	172905000	2.35E-03	promoter	super_enhancer	RP1-125I3.4
chr1	26325000	chr1	32725000	2.36E-02	promoter	super_enhancer	PAFAH2
chr1	26325000	chr1	43435000	1.38E-02	promoter	super_enhancer	PAFAH2
chr1	26325000	chr1	43895000	1.44E-02	promoter	super_enhancer	PAFAH2
chr1	26325000	chr1	204455000	2.70E-03	promoter	super_enhancer	PAFAH2
chr1	26325000	chr1	234985000	2.30E-03	promoter	super_enhancer	PAFAH2
chr1	26335000	chr1	33285000	2.09E-02	super_enhancer	promoter	YARS

chr1	26335000	chr1	94345000	2.85E-03	super_enhancer	promoter	DNTTIP2
chr1	26335000	chr1	175275000	1.79E-03	super_enhancer	promoter	RP3-518E13.2
chr1	26335000	chr1	182655000	1.95E-03	super_enhancer	promoter	RGS8
chr1	26335000	chr1	203275000	2.67E-03	super_enhancer	promoter	LINC01136
chr1	26345000	chr1	26485000	4.38E-02	super_enhancer	promoter	FAM110D
chr1	26345000	chr1	26605000	1.93E-02	super_enhancer	promoter	SH3BGRL3
chr1	26345000	chr1	116185000	2.44E-03	super_enhancer	promoter	VANGL1
chr1	26345000	chr1	117665000	2.64E-03	super_enhancer	promoter	TRIM45
chr1	26345000	chr1	229695000	2.60E-03	super_enhancer	promoter	ABCB10
chr1	26355000	chr1	52325000	9.03E-03	super_enhancer	promoter	TSEN15P2
chr1	26355000	chr1	109585000	2.35E-03	super_enhancer	promoter	WDR47
chr1	26355000	chr1	149285000	2.18E-03	super_enhancer	promoter	RP11-403I13.7
chr1	26355000	chr1	229645000	2.83E-03	super_enhancer	promoter	NUP133
chr1	26485000	chr1	43405000	1.30E-02	promoter	super_enhancer	FAM110D
chr1	26485000	chr1	43475000	1.54E-02	promoter	super_enhancer	FAM110D
chr1	26485000	chr1	230085000	2.33E-03	promoter	super_enhancer	FAM110D
chr1	26535000	chr1	156115000	2.76E-03	promoter	super_enhancer	RP11-231P20.2
chr1	26535000	chr1	204085000	2.78E-03	promoter	super_enhancer	RP11-231P20.2
chr1	26605000	chr1	41865000	1.87E-02	promoter	super_enhancer	SH3BGRL3
chr1	26645000	chr1	32745000	3.41E-02	promoter	super_enhancer	UBXN11
chr1	26645000	chr1	226845000	3.32E-03	promoter	super_enhancer	UBXN11
chr1	26795000	chr1	207325000	1.12E-03	promoter	super_enhancer	RP3-476K8.3
chr1	26855000	chr1	222885000	2.73E-03	super_enhancer	promoter	BROX
chr1	26865000	chr1	93475000	3.00E-03	super_enhancer	promoter	RNU6-210P

chr1	26865000	chr1	155905000	2.65E-03	super_enhancer	promoter	KIAA0907
chr1	26865000	chr1	201435000	2.92E-03	super_enhancer	promoter	RP11-134G8.10
chr1	26875000	chr1	40915000	1.69E-02	super_enhancer	promoter	ZFP69B
chr1	26875000	chr1	203275000	3.51E-03	super_enhancer	promoter	LINC01136
chr1	27485000	chr1	35975000	1.70E-02	super_enhancer	promoter	RP4-728D4.3
chr1	27485000	chr1	46015000	1.68E-02	super_enhancer	promoter	AKR1A1
chr1	27485000	chr1	46835000	1.48E-02	super_enhancer	promoter	RP4-603I14.3
chr1	27485000	chr1	47135000	1.35E-02	super_enhancer	promoter	TEX38
chr1	27485000	chr1	47185000	1.32E-02	super_enhancer	promoter	EFCAB14
chr1	27485000	chr1	110285000	2.21E-03	super_enhancer	promoter	GSTM3
chr1	27485000	chr1	151105000	2.41E-03	super_enhancer	promoter	RP11-68I18.10
chr1	27485000	chr1	226035000	2.97E-03	super_enhancer	promoter	RP11-285F7.2
chr1	27485000	chr1	229695000	2.53E-03	super_enhancer	promoter	ABCB10
chr1	27495000	chr1	32665000	3.43E-02	super_enhancer	promoter	CCDC28B
chr1	27495000	chr1	44495000	1.91E-02	super_enhancer	promoter	RP5-1198O20.4
chr1	27495000	chr1	203445000	2.66E-03	super_enhancer	promoter	PRELP
chr1	27505000	chr1	27975000	1.43E-02	super_enhancer	promoter	RP1-159A19.3
chr1	27505000	chr1	53815000	8.03E-03	super_enhancer	promoter	RP11-117D22.1
chr1	27515000	chr1	92655000	2.85E-03	super_enhancer	promoter	RP4-621B10.8
chr1	27515000	chr1	225925000	1.81E-03	super_enhancer	promoter	RP11-145A3.2
chr1	27525000	chr1	32635000	2.73E-02	super_enhancer	promoter	RP4-622L5.2
chr1	27525000	chr1	173795000	2.33E-03	super_enhancer	promoter	CENPL
chr1	27535000	chr1	28285000	1.19E-02	super_enhancer	promoter	XKR8
chr1	27535000	chr1	32225000	3.43E-02	super_enhancer	promoter	MIR4254

chr1	27535000	chr1	234795000	2.52E-03	super_enhancer	promoter	RP4-781K5.5
chr1	27535000	chr1	238025000	1.83E-03	super_enhancer	promoter	RP11-193H5.1
chr1	27545000	chr1	28015000	4.50E-02	super_enhancer	promoter	CHMP1AP1
chr1	27545000	chr1	42805000	1.92E-02	super_enhancer	promoter	RP11-223A3.1
chr1	27545000	chr1	65045000	4.67E-03	super_enhancer	promoter	MIR4794
chr1	27545000	chr1	119255000	1.74E-03	super_enhancer	promoter	RP4-630J13.1
chr1	27545000	chr1	160925000	2.53E-03	super_enhancer	promoter	ITLN2
chr1	27555000	chr1	27715000	3.44E-02	super_enhancer	promoter	RP1-144C9.2
chr1	27555000	chr1	33815000	2.47E-02	super_enhancer	promoter	RP11-415J8.5
chr1	27555000	chr1	59485000	8.56E-03	super_enhancer	promoter	LINC01358
chr1	27555000	chr1	110625000	2.60E-03	super_enhancer	promoter	LINC01397
chr1	27555000	chr1	226335000	2.64E-03	super_enhancer	promoter	RP11-275I14.4
chr1	27565000	chr1	28435000	7.99E-03	super_enhancer	promoter	RP5-1053E7.2
chr1	27565000	chr1	32645000	3.08E-02	super_enhancer	promoter	TXLNA
chr1	27565000	chr1	33815000	2.31E-02	super_enhancer	promoter	RP11-415J8.5
chr1	27565000	chr1	145095000	2.53E-03	super_enhancer	promoter	SEC22B
chr1	27715000	chr1	43865000	1.73E-02	promoter	super_enhancer	RP1-144C9.2
chr1	27715000	chr1	94665000	3.82E-03	promoter	super_enhancer	RP1-144C9.2
chr1	27715000	chr1	233685000	2.45E-03	promoter	super_enhancer	RP1-144C9.2
chr1	27975000	chr1	224255000	1.66E-03	promoter	super_enhancer	RP1-159A19.3
chr1	27995000	chr1	66265000	6.39E-03	promoter	super_enhancer	RP11-288L9.4
chr1	27995000	chr1	233665000	2.88E-03	promoter	super_enhancer	RP11-288L9.4
chr1	28015000	chr1	32765000	3.77E-02	promoter	super_enhancer	CHMP1AP1
chr1	28015000	chr1	183125000	2.30E-03	promoter	super_enhancer	CHMP1AP1

chr1	28015000	chr1	233635000	2.30E-03	promoter	super_enhancer	CHMP1AP1
chr1	28065000	chr1	36385000	2.03E-02	promoter	super_enhancer	RP3-426I6.2
chr1	28065000	chr1	43875000	1.47E-02	promoter	super_enhancer	RP3-426I6.2
chr1	28065000	chr1	226815000	2.22E-03	promoter	super_enhancer	RP3-426I6.2
chr1	28135000	chr1	43865000	9.67E-03	promoter	super_enhancer	AL020997.1
chr1	28135000	chr1	154975000	1.67E-03	promoter	super_enhancer	AL020997.1
chr1	28135000	chr1	235045000	1.61E-03	promoter	super_enhancer	AL020997.1
chr1	28145000	chr1	55445000	6.68E-03	promoter	super_enhancer	RP3-426I6.6
chr1	28285000	chr1	32995000	4.09E-02	promoter	super_enhancer	XKR8
chr1	28285000	chr1	235015000	3.85E-03	promoter	super_enhancer	XKR8
chr1	28415000	chr1	32755000	4.35E-02	promoter	super_enhancer	EYA3
chr1	28435000	chr1	32715000	3.97E-02	promoter	super_enhancer	RP5-1053E7.2
chr1	28435000	chr1	63695000	5.81E-03	promoter	super_enhancer	RP5-1053E7.2
chr1	28435000	chr1	93705000	3.21E-03	promoter	super_enhancer	RP5-1053E7.2
chr1	28585000	chr1	218595000	2.19E-03	promoter	super_enhancer	SESN2
chr1	28585000	chr1	233705000	1.86E-03	promoter	super_enhancer	SESN2
chr1	28695000	chr1	43415000	1.22E-02	promoter	super_enhancer	PHACTR4
chr1	28695000	chr1	152015000	2.56E-03	promoter	super_enhancer	PHACTR4
chr1	28695000	chr1	233675000	2.62E-03	promoter	super_enhancer	PHACTR4
chr1	28835000	chr1	33005000	3.82E-02	promoter	super_enhancer	SNORA73B
chr1	28835000	chr1	218575000	1.76E-03	promoter	super_enhancer	SNORA73B
chr1	28855000	chr1	204455000	3.04E-03	promoter	super_enhancer	PRDX3P2
chr1	28855000	chr1	234615000	2.71E-03	promoter	super_enhancer	PRDX3P2
chr1	28905000	chr1	204425000	3.40E-03	promoter	super_enhancer	SNORD99

chr1	28975000	chr1	150585000	2.47E-03	promoter	super_enhancer	RNU11
chr1	28995000	chr1	200315000	3.14E-03	promoter	super_enhancer	GMEB1
chr1	29205000	chr1	31945000	4.98E-02	promoter	super_enhancer	RP1-212P9.3
chr1	29205000	chr1	36385000	2.94E-02	promoter	super_enhancer	RP1-212P9.3
chr1	29205000	chr1	112705000	2.94E-03	promoter	super_enhancer	RP1-212P9.3
chr1	29205000	chr1	235015000	3.92E-03	promoter	super_enhancer	RP1-212P9.3
chr1	29475000	chr1	204455000	3.39E-03	promoter	super_enhancer	RP11-242O24.3
chr1	30885000	chr1	43405000	1.38E-02	promoter	super_enhancer	RP4-591L5.1
chr1	30885000	chr1	43915000	1.95E-02	promoter	super_enhancer	RP4-591L5.1
chr1	30885000	chr1	164635000	1.84E-03	promoter	super_enhancer	RP4-591L5.1
chr1	31925000	chr1	109655000	2.11E-03	super_enhancer	promoter	KIAA1324
chr1	31925000	chr1	113435000	1.64E-03	super_enhancer	promoter	RP3-522D1.2
chr1	31925000	chr1	167905000	1.57E-03	super_enhancer	promoter	DCAF6
chr1	31925000	chr1	247365000	1.65E-03	super_enhancer	promoter	MIR3916
chr1	31925000	chr1	248855000	1.52E-03	super_enhancer	promoter	XX-CR54.1
chr1	31935000	chr1	37095000	1.80E-02	super_enhancer	promoter	FTLP18
chr1	31935000	chr1	44115000	1.05E-02	super_enhancer	promoter	KDM4A
chr1	31935000	chr1	45205000	1.31E-02	super_enhancer	promoter	KIF2C
chr1	31935000	chr1	60255000	5.32E-03	super_enhancer	promoter	RP4-782L23.2
chr1	31935000	chr1	62785000	5.78E-03	super_enhancer	promoter	KANK4
chr1	31945000	chr1	40255000	1.11E-02	super_enhancer	promoter	BMP8B
chr1	31945000	chr1	50865000	6.61E-03	super_enhancer	promoter	HMGB1P45
chr1	31955000	chr1	45805000	1.03E-02	super_enhancer	promoter	TOE1
chr1	31955000	chr1	155165000	1.81E-03	super_enhancer	promoter	RP11-263K19.4

chr1	32225000	chr1	85295000	3.74E-03	promoter	super_enhancer	MIR4254
chr1	32225000	chr1	153535000	2.71E-03	promoter	super_enhancer	MIR4254
chr1	32225000	chr1	200425000	2.40E-03	promoter	super_enhancer	MIR4254
chr1	32255000	chr1	32735000	2.82E-02	promoter	super_enhancer	RP11-84A19.3
chr1	32255000	chr1	32745000	4.22E-02	promoter	super_enhancer	RP11-84A19.3
chr1	32255000	chr1	43925000	1.45E-02	promoter	super_enhancer	RP11-84A19.3
chr1	32255000	chr1	93705000	2.97E-03	promoter	super_enhancer	RP11-84A19.3
chr1	32395000	chr1	94585000	2.58E-03	promoter	super_enhancer	AL136115.1
chr1	32635000	chr1	43915000	1.86E-02	promoter	super_enhancer	RP4-622L5.2
chr1	32645000	chr1	32755000	2.87E-03	promoter	super_enhancer	TXLNA
chr1	32645000	chr1	41875000	2.76E-02	promoter	super_enhancer	TXLNA
chr1	32645000	chr1	214445000	2.90E-03	promoter	super_enhancer	TXLNA
chr1	32665000	chr1	32755000	6.29E-03	promoter	super_enhancer	CCDC28B
chr1	32665000	chr1	41855000	1.90E-02	promoter	super_enhancer	CCDC28B
chr1	32665000	chr1	226825000	3.03E-03	promoter	super_enhancer	CCDC28B
chr1	32675000	chr1	41855000	2.33E-02	promoter	super_enhancer	DCDC2B
chr1	32675000	chr1	41905000	3.28E-02	promoter	super_enhancer	DCDC2B
chr1	32715000	chr1	44655000	1.51E-02	super_enhancer	promoter	RP5-891H21.5
chr1	32715000	chr1	45205000	1.83E-02	super_enhancer	promoter	KIF2C
chr1	32715000	chr1	47875000	1.43E-02	super_enhancer	promoter	LINC01389
chr1	32715000	chr1	51525000	9.41E-03	super_enhancer	promoter	MIR4421
chr1	32715000	chr1	59675000	6.40E-03	super_enhancer	promoter	HSD52
chr1	32715000	chr1	101655000	2.62E-03	super_enhancer	promoter	RP5-1033H2.1
chr1	32715000	chr1	144875000	3.08E-03	super_enhancer	promoter	RP4-791M13.5

chr1	32725000	chr1	40255000	2.08E-02	super_enhancer	promoter		BMP8B
chr1	32725000	chr1	53165000	1.28E-02	super_enhancer	promoter		COA7
chr1	32735000	chr1	36335000	4.63E-02	super_enhancer	promoter		AGO1
chr1	32735000	chr1	54205000	1.16E-02	super_enhancer	promoter		GLIS1
chr1	32735000	chr1	95585000	3.11E-03	super_enhancer	promoter		RP11-57H12.2
chr1	32735000	chr1	228595000	2.21E-03	super_enhancer	promoter		TRIM11
chr1	32745000	chr1	37095000	3.44E-02	super_enhancer	promoter		FTLP18
chr1	32745000	chr1	40505000	2.80E-02	super_enhancer	promoter		CAP1
chr1	32745000	chr1	46915000	2.67E-02	super_enhancer	promoter		LINC01398
chr1	32755000	chr1	44655000	1.70E-02	super_enhancer	promoter		RP5-891H21.5
chr1	32755000	chr1	52615000	1.26E-02	super_enhancer	promoter		RN7SL788P
chr1	32755000	chr1	53705000	1.13E-02	super_enhancer	promoter		MAGOH
chr1	32755000	chr1	59485000	1.08E-02	super_enhancer	promoter		LINC01358
chr1	32755000	chr1	86175000	3.69E-03	super_enhancer	promoter		ZNHIT6
chr1	32755000	chr1	241815000	2.39E-03	super_enhancer	promoter		WDR64
chr1	32775000	chr1	56405000	9.55E-03	super_enhancer	promoter		PIGQP1
chr1	32775000	chr1	62505000	5.80E-05	super_enhancer	promoter		LAMTOR5P1
chr1	32775000	chr1	85465000	4.21E-03	super_enhancer	promoter		WDR63
chr1	32775000	chr1	211835000	2.96E-03	super_enhancer	promoter		FDPSP8
chr1	32785000	chr1	98675000	2.38E-03	super_enhancer	promoter		RP5-1070A16.1
chr1	32985000	chr1	38275000	3.27E-02	super_enhancer	promoter		YRDC
chr1	32985000	chr1	145525000	2.35E-03	super_enhancer	promoter		ITGA10
chr1	32995000	chr1	40505000	2.49E-02	super_enhancer	promoter		CAP1
chr1	32995000	chr1	44655000	1.57E-02	super_enhancer	promoter		RP5-891H21.5

chr1	32995000	chr1	52175000	9.16E-03	super_enhancer	promoter	GAPDHP51
chr1	32995000	chr1	110135000	2.30E-03	super_enhancer	promoter	RNU6V
chr1	32995000	chr1	153515000	3.50E-03	super_enhancer	promoter	S100A5
chr1	32995000	chr1	160765000	2.58E-03	super_enhancer	promoter	LY9
chr1	33005000	chr1	38325000	3.09E-02	super_enhancer	promoter	MTF1
chr1	33005000	chr1	244015000	2.39E-03	super_enhancer	promoter	AKT3
chr1	33285000	chr1	42925000	1.99E-02	promoter	super_enhancer	YARS
chr1	33285000	chr1	164605000	1.84E-03	promoter	super_enhancer	YARS
chr1	33285000	chr1	225455000	1.89E-03	promoter	super_enhancer	YARS
chr1	33445000	chr1	93775000	3.39E-03	promoter	super_enhancer	RP4-803A2.1
chr1	33445000	chr1	109285000	2.25E-03	promoter	super_enhancer	RP4-803A2.1
chr1	33445000	chr1	234655000	2.55E-03	promoter	super_enhancer	RP4-803A2.1
chr1	34755000	chr1	38275000	3.80E-02	super_enhancer	promoter	YRDC
chr1	34755000	chr1	39425000	2.83E-02	super_enhancer	promoter	EIF1P2
chr1	34755000	chr1	44445000	1.73E-02	super_enhancer	promoter	B4GALT2
chr1	34765000	chr1	38555000	3.05E-02	super_enhancer	promoter	MIR3659
chr1	34765000	chr1	111935000	1.77E-03	super_enhancer	promoter	PGCP1
chr1	34765000	chr1	155715000	1.96E-03	super_enhancer	promoter	MSTO2P
chr1	34765000	chr1	157695000	1.25E-03	super_enhancer	promoter	VDAC1P9
chr1	34765000	chr1	160175000	1.93E-03	super_enhancer	promoter	PEA15
chr1	34765000	chr1	205055000	1.93E-03	super_enhancer	promoter	TMEM81
chr1	34785000	chr1	35395000	4.94E-02	super_enhancer	promoter	DLGAP3
chr1	34785000	chr1	53685000	7.77E-03	super_enhancer	promoter	RP5-1024G6.7
chr1	34785000	chr1	53925000	8.84E-03	super_enhancer	promoter	DMRTB1

chr1	35135000	chr1	183145000	2.02E-03	promoter	super_enhancer	MIR552
chr1	35135000	chr1	183625000	1.63E-03	promoter	super_enhancer	MIR552
chr1	35225000	chr1	58585000	5.89E-03	promoter	super_enhancer	GJB4
chr1	35245000	chr1	200375000	1.71E-03	promoter	super_enhancer	AL121988.1
chr1	35245000	chr1	200415000	2.19E-03	promoter	super_enhancer	AL121988.1
chr1	35255000	chr1	233705000	1.20E-03	promoter	super_enhancer	RP1-34M23.5
chr1	35315000	chr1	172915000	1.93E-03	promoter	super_enhancer	RP5-997D16.2
chr1	35315000	chr1	230075000	2.37E-03	promoter	super_enhancer	RP5-997D16.2
chr1	35325000	chr1	55445000	1.11E-02	promoter	super_enhancer	SMIM12
chr1	35325000	chr1	94615000	2.67E-03	promoter	super_enhancer	SMIM12
chr1	35395000	chr1	42955000	1.65E-02	promoter	super_enhancer	DLGAP3
chr1	35525000	chr1	207005000	1.68E-03	promoter	super_enhancer	ZMYM1
chr1	35735000	chr1	43865000	1.73E-02	promoter	super_enhancer	ZMYM4
chr1	35735000	chr1	43935000	1.68E-02	promoter	super_enhancer	ZMYM4
chr1	35735000	chr1	55425000	8.22E-03	promoter	super_enhancer	ZMYM4
chr1	35735000	chr1	204085000	2.45E-03	promoter	super_enhancer	ZMYM4
chr1	35815000	chr1	41885000	2.35E-02	promoter	super_enhancer	RPL5P4
chr1	35815000	chr1	41905000	3.28E-02	promoter	super_enhancer	RPL5P4
chr1	35815000	chr1	44805000	1.79E-02	promoter	super_enhancer	RPL5P4
chr1	36025000	chr1	91605000	4.23E-03	promoter	super_enhancer	KIAA0319L
chr1	36025000	chr1	93775000	3.57E-03	promoter	super_enhancer	KIAA0319L
[truncated: 379,069 more chars]
